# Supplementary material for: Cellular reprogramming for successful CNS axon regeneration is driven by a temporally changing cast of transcription factors
Source: Sci Rep. 2019 Oct 2;9:14198. doi: 10.1038/s41598-019-50485-6 (PMC6775158; doi:10.1038/s41598-019-50485-6)
Supplement: Supplementary file 1 — Supplementary Information [file 41598_2019_50485_MOESM1_ESM.pdf]

Supplementary Information (SI)

Cellular reprogramming for successful CNS axon regeneration is driven by a temporally changing cast of transcription factors

Sumona P. Dhara[1], Andrea Rau[2,3], Michael J. Flister[4], Nicole M. Recka[1], Michael D. Laiosa[3], Paul L. Auer[3], Ava J. Udvadia[1*]

[1]Department of Biological Sciences, University of Wisconsin-Milwaukee, Milwaukee, WI 53201, USA

[2]GABI, INRA, AgroParisTech, Universite Paris-Saclay, 78350, Jouy-en-Josas, France

[3]Joseph J Zilber School of Public Health, University of Wisconsin-Milwaukee, Milwaukee, WI 53201, USA

[4]Department of Physiology, Medical College of Wisconsin, Milwaukee, WI 53226, USA

[*]To whom correspondence may be addressed: audvadia@uwm.edu

1. Detailed Materials and Methods, SI references
2. Figure S1. Accessible chromatin is mainly located distal to annotated genes.
3. Figure S2. Differentially expressed transcripts that encode transcription factors display regeneration stage-specific temporal patterning
4. Figure S3. Temporal patterning of differentially expressed leucine zipper (bZIP) family transcription factors.
5. Figure S4. Temporal patterning of differentially expressed basic helix-loop-helix (bHLH) family transcription factors.
6. Figure S5. Temporal patterning of differentially expressed 2 cys-2 his zinc finger (C2H2ZF) family transcription factors.
7. Figure S6. Temporal patterning of differentially expressed homeodomain family transcription factors.
8. Figure S7. Injury-induced eGFP expression in retinal ganglion cells can be visualized through the lens of intact animals.
9. Figure S8. FASTA sequence of transgene.
10. Table S1. Transcripts differentially expressed compared to controls (0 dpi) in at least one time point (5% FDR).
11. Table S2. Differentially expressed transcripts (1% FDR) clustered based on temporal expression pattern.
12. Table S3. Ingenuity Pathway Analysis of temporally clustered, differentially expressed genes.
13. Table S4. Differentially accessible sequences with distance to nearest annotated genes and nearest differentially-expressed genes.
14. Table S5. Differentially expressed transcripts that encode transcription factors with and without known motifs.
15. Table S6. Differentially expressed transcripts that encode transcription factors with known motifs.
16. Table S7. Transcription factors that are differentially expressed during regeneration.
17. Table S8. Gene ontology (GO) analysis of putative Jun transcriptional targets.
18. Table S9. Gene ontology analysis of regeneration-associated genes with peak expression during initial axon growth toward the midline.
19. Table S10. RNA sample quality control.
20. Table S11. ATAC-seq library sample quality control
21. Table S12. PCR primers for ATAC-seq libraries based on Nextera indices

Detailed Materials and Methods

Zebrafish husbandry and maintenance

Zebrafish husbandry and all experimental procedures were approved by the *Institutional Animal Care and Use Committee* (IACUC). Zebrafish colonies were maintained as previously described [1]. Adult fish were housed in recirculating rack systems (Aquatic Habitats, Apopka, FL) at 28.5°C on a 14-hour light, 10-hour dark cycle, and fed twice daily with Adult Zebrafish Complete Diet (VWR, West Chester, PA) and once daily with brine shrimp (*Artemia*). A wild type strain (Ekkwill, EK) was used as a negative control for FACS. A transgenic reporter strain constructed on the EK background, Tg (*Tru.gap43:egfp*) mil1, or *fgap43:egfp* [2] was used for all other experiments.

Zebrafish optic nerve injury

Optic nerve crush (ONC) lesions were performed on adult zebrafish, 7-9 months of age, as previously described [3]. Briefly, fish were anesthetized in 0.46 mg/mL tricaine (Argent Chemical Labs, Redmond, WA) in 30% Danieau [4]. The left optic nerve of anesthetized fish was exposed and crushed for 10 seconds using Dumont #5 forceps. The intact retina of an uninjured fish served as the unoperated control (0dpi). Fish were sacrificed 0, 2, 4, 7, or 12 days post injury (dpi) and retinas were dissected. Prior to dissection, left and right eyes of the fish were examined under Nikon eclipse TE2000-U fluorescence microscope for GFP fluorescence as an indicator of regeneration-induced transcriptional activity. GFP was easily detected through the lens in eyes which had previously undergone optic nerve crush (Fig. S7).

RNA-seq data generation

RNA Isolation. RNA was extracted and purified from retinas dissected from naïve (0 dpi) and regenerating adult fish at 2, 4, 7, and 12 dpi. Three biological replicates of RNA were obtained for each time point. To prevent RNA degradation, dissected retinas were immediately immersed in an RNA stabilization reagent (RLT buffer of RNeasy Micro Kit, Cat No./ID 74004, Qiagen; Valencia, CA). The retinas were homogenized (sterile Fisherbrand™ RNase-Free Disposable Pellet Pestles Cat No.12-141-368) and filtered (Cat No./ID: 79654, Qiagen). Total RNA was extracted from the homogenized mixture according to manufacturer instructions (RNeasy Micro Kit, Cat No./ID 74004, Qiagen). Total RNA concentration and purity were quantified with NanoDrop ND2000 spectrophotometer (Thermo Scientific) and QuBit fluorometer (Invitrogen), respectively. RNA integrity was quantified using 2100 Bioanalyzer (Agilent High Sensitivity RNA 6000 Pico Reagents Cat No. 5067-1514). For each biological replicate, 3-6 retinas were pooled, cleaned and concentrated (RNA Clean & Concentrator kit, SKU R1013, Zymo Research) to obtain 1 ug total RNA. Total RNA concentration, purity, and integrity of pooled samples was determined as described above. Only samples with RNA integrity numbers (RIN) ranging from 7.7 to 8.2 were used for sequencing (Table S10).

Library preparation and sequencing. cDNA libraries (n=3 for 0, 2, 4, 7, and 12 dpi) were generated using Tru-Seq Stranded Total & mRNA Sample Prep Kits, (Illumina 20020595) at University of Wisconsin-Madison Biotechnology Center (UWBC). Each cDNA library was indexed for multiplexing and subsequently sequenced on four lanes of the Illumina Hiseq2000 device, UWBC. Libraries were sequenced at 50 bp, 30–40 million paired-end reads/sample, on Illumina HiSeq 2500 at UWBC, Madison.

Bioinformatic analysis. After merging technical replicates of RNA-seq samples across lanes, TrimGalore (v0.4.4, --stringency 3 -q 20) was used in paired-end mode to trim adaptor sequences,

FastQC (v0.11.5) was used to validate sequence quality, and Kallisto [5] (v0.42.4) was used to build an index on a FASTA file consisting of the zebrafish transcriptome (GRCz10 Danio rerio genome assembly, Ensembl release 84 annotation) and the transgene sequence (Fig. S8). Kallisto was subsequently used to quantify transcript abundances using 500 bootstrap samples.

ATAC-seq data generation

Cell sorting. RGCs were collected from dissociated regenerating and control retinas, at each time point using fluorescent activated cell sorting (FACS). Zebrafish were sacrificed, and retinas were dissected and immersed in ice cold PBS with no calcium and magnesium. To one well of a 24-well plate, a single retina (divided into 8 uniform pieces) was added to 500 µL Accumax (STEMCELL Technologies). Each retina was chemically digested for 70 mins with agitation on a nutator. Reactions were quenched in heat inactivated fetal calf serum (HI-FCS; Gemini Bio-Products) in DMEM/F12 (Gibco) and lysates were mechanically dissociated by gentle pipetting. Undigested fragments were removed, and the cell suspension was pelleted at 200 g for 3 min. Supernatant was aspirated and the pellet re-suspended in 100 µl fresh quenching buffer (DMEM/F12 + 20% FCS). Cell suspensions from multiple retinas at each time point were pooled (0 dpi, 8-10 retina; 2, 4, 7, and 12 dpi, 4-6 retinas). Pooled cell suspension were filtered (Falcon, Cat No. 352235), and sorted for RGCs expressing GFP using a Becton Dickinson FACSAria™ III sorter fitted with the 100 µM nozzle. Negative control cells were used to set gates to separate GFP positive (GFP+) from GFP negative (GFP−) fractions. We collected 50,000 FACS-sorted GFP+ cells per sample that were immediately used for chromatin isolation as described below. Three biologically distinct replicates of pooled cells were collected for each time point.

Library preparation. ATAC-seq libraries were prepared using the Tn5 transposase system (Nextera DNA library kit, Illumina, FC-121–1030) as previously described [6], and purified using DNA Clean & Concentrator kit (SKU: ZD5205, Zymo).The purified samples were assessed for quality as described above (NanoDrop ND2000 spectrophotometer and QuBit fluorometer) and appropriate nucleosomal laddering was determined by 2100 Bioanalyzer (Agilent High Sensitivity DNA Kit, Cat No. 5067-4626) (Table S11). For PCR amplification and qPCR side reactions, PerfeCTa SYBR Green FastMix Quanta (VWR Cat No. 101414-150) was used (Table S12 for PCR Primers).

Sequencing. Prior to running the full sequence, Mi-seq was used to estimate sequencing depth. One sample was below the cut-off criteria and therefore omitted. The remaining fourteen samples were indexed for multiplexing and subsequently sequenced on four lanes of the Illumina Hiseq2000, UWBC. Data were sequenced at 50 bp to obtain approximately 25 million paired-end reads/samples.

Bioinformatic analysis. After merging technical replicates of ATAC-seq samples across lanes, TrimGalore (v0.4.4, --stringency 3 -q 20) was used in paired-end mode to trim adaptor sequences and FastQC (v0.11.5) was used to validate sequence quality. BWA-MEM [7] (v0.7.9a-r786) was used to align reads to the zebrafish genome (GRCz10) and transgene sequence. Duplicate and multiple mapped reads were removed using samtools (v1.6) [8]. After concatenating aligned reads across all replicates and time points, MACS2 [9] (v2.1.1.20160309, --no-model -g 1.37e+09 --keep-dup all --call-summits) was used in paired-end mode without shifting model to call peaks from aligned reads, and summits of deconvoluted subpeaks were identified. Only peaks with a p-value $< 10^{-10}$ were retained for subsequent analyses. For each remaining subpeak summit, a 500bp "peaklet" interval was defined using [summit - 250bp, summit + 249bp] using GenomicRanges (v1.30.3) [10]. We refer to these peaklets as consensus regions of accessible chromatin. Open

chromatin in each replicate of each time point was then quantified using DiffBind (v2.6.6, default parameters) by counting the number of overlapping reads for each retained peaklet.

Statistical analysis of RNA-seq and ATAC-seq data

Following pseudoalignment and quantification of transcripts, differentially expressed transcripts were identified using Sleuth (v0.29.0) [11]. Specifically, a full model, including a factor for each time point after injury (2, 4, 7, 12 dpi), was estimated for each transcript, and a Wald test was calculated for each coefficient to identify significant differences with the initial time point (0dpi). After controlling the false discovery rate (FDR) at 5% within each comparison using the Benjamini-Hochberg [12] approach, differentially expressed transcripts with respect to the baseline were identified for each post-injury time point. Beta values from the model were used as a biased estimator of log-fold change. Expression heatmaps (based on Z-scores calculated using either log fold-changes or log transcripts per million [TPM] estimates) were produced using ComplexHeatmap [13] (v1.17.1), where transcript clusters were identified using the K-means algorithm, and hierarchical clustering (Euclidean distance, complete linkage) was used to cluster rows. The Ingenuity Pathway Analysis tool (Qiagen, Redwood City, CA, USA) was used to analyze enrichment of molecular and functional gene networks within the differentially expressed gene sets (FDR<0.05) at each time point after injury (2, 4, 7, 12 dpi) compared with the initial time point (0dpi).

After quantifying peaklet accessibility, DESeq2 (v1.18.1) [14] was used to identify differentially accessible peaklets in an analogous manner to the RNA-seq analysis described above. As before, a full generalized linear model including a factor for each post-injury time point was estimated for each peaklet, and a Wald test was calculated for each coefficient to determine significant differences in accessibility compared to the baseline. Peaklets with FDR-controlled p-values < 0.05 in one of the four comparisons were considered to be differentially accessible. ChIPpeakAnno (v3.12.7) [14,15], the TxDb.Drerio.UCSC.danRer10.refGene UCSC annotation package (v3.4.2), and AnnotationHub (v2.10.1) were used to annotate peaklets with genes. Specifically, non-exonic (i.e., not overlapping exons by more than 50bp) peaklets overlapping a transcription start site (TSS) or within 1kb of a TSS were considered to represent proximal peaks, whereas those greater than 1kb but less than 100kb of a TSS were considered to represent distal peaks. All statistical analyses were performed in R (v3.4.3). Integrative Genome Viewer (IGV) was used to visualize RNA-seq and ATAC-seq alignments [16].

We used motif analysis to determine potential binding sites of our differentially expressed transcription factors within regions of accessible chromatin identified by ATAC-seq. Motif enrichment and discovery was carried out using various applications within the MEME Suite of motif-based sequence analysis tools (version 5.0.4) [17]. We compiled a user-supplied file for motifs corresponding to the transcription factors we identified as differentially expressed (Fig. 2), for which there were existing motifs in JASPAR or CIS-BP databases (Table S7). Motifs were formatted to MEME Motif format as specified with the MEME suite applications (meme-suite.org). The Analysis of Motif Enrichment (AME) tool [18] was used to determine motif enrichment in accessible chromatin surrounding genes in each temporal cluster (Fig. 1C). FASTA files of 500 bp peaklet sequences located proximal (≤1 kb from transcription start site) or distal (within > 1 kb, but ≤100 kb, from transcription start site) from differentially expressed genes were used in conjunction with our user-supplied motif file. Analysis was run using average odds score sequence scoring and Fisher's exact test for motif enrichment. The AME tool was also used to identify motifs within the differentially accessible chromatin regions surrounding the *jun* gene (Fig. 4), using both our user supplied motif file and the built in motif file for Eukaryotic DNA, Vertebrates (*in vivo* and *in silico*). The Find Individual Motif Occurrences (FIMO) tool [19] was used to scan for

additional binding sites with the putative *jun* promoter and enhancers sequences using our user-supplied motif list. AME and FIMO analysis were both run using default parameter settings.

References cited in Detailed Materials and Methods

1 Westerfield, M. *The Zebrafish Book: A Guide for the Laboratory Use of Zebrafish (Danio rerio)*. (1997).

2 Udvadia, A. J. 3.6 kb Genomic sequence from Takifugu capable of promoting axon growth-associated gene expression in developing and regenerating zebrafish neurons. *Gene Expr Patterns* **8**, 382-388, doi:10.1016/j.gep.2008.05.002 (2008).

3 Bormann, P., Zumsteg, V. M., Roth, L. W. A. & Reinhard, E. Target contact regulates GAP-43 and alpha-tubulin mRNA levels in regenerating retinal ganglion cells. *J Neurosci Res* **52**, 405-419, doi:Doi 10.1002/(Sici)1097-4547(19980515)52:4<405::Aid-Jnr4>3.0.Co;2-D (1998).

4 Manoli, M. & Driever, W. Fluorescence-activated cell sorting (FACS) of fluorescently tagged cells from zebrafish larvae for RNA isolation. *Cold Spring Harb Protoc* **2012**, doi:10.1101/pdb.prot069633 (2012).

5 Bray, N. L., Pimentel, H., Melsted, P. & Pachter, L. Near-optimal probabilistic RNA-seq quantification. *Nature Biotechnology* **34**, 525-527, doi:10.1038/nbt.3519 (2016).

6 Buenrostro, J. D., Wu, B., Chang, H. Y. & Greenleaf, W. J. ATAC-seq: A Method for Assaying Chromatin Accessibility Genome-Wide. *Curr Protoc Mol Biol* **109**, 21 29 21-29, doi:10.1002/0471142727.mb2129s109 (2015).

7 Li, H. & Durbin, R. Fast and accurate short read alignment with Burrows-Wheeler transform. *Bioinformatics* **25**, 1754-1760, doi:10.1093/bioinformatics/btp324 (2009).

8 Li, H., Handsaker, B., Wysoker, A., Fennell, T., Ruan, J., Homer, N., Marth, G., Abecasis, G., Durbin, R. & Proc, G. P. D. The Sequence Alignment/Map format and SAMtools. *Bioinformatics* **25**, 2078-2079, doi:10.1093/bioinformatics/btp352 (2009).

9 Zhang, Y., Liu, T., Meyer, C. A., Eeckhoute, J., Johnson, D. S., Bernstein, B. E., Nussbaum, C., Myers, R. M., Brown, M., Li, W. & Liu, X. S. Model-based Analysis of ChIP-Seq (MACS). *Genome Biology* **9**, doi:ARTN R137; 10.1186/gb-2008-9-9-r137 (2008).

10 Lawrence, M., Huber, W., Pages, H., Aboyoun, P., Carlson, M., Gentleman, R., Morgan, M. T. & Carey, V. J. Software for Computing and Annotating Genomic Ranges. *Plos Comput Biol* **9**, doi:ARTN e1003118; 10.1371/journal.pcbi.1003118 (2013).

11 Pimentel, H., Bray, N. L., Puente, S., Melsted, P. & Pachter, L. Differential analysis of RNA-seq incorporating quantification uncertainty. *Nat Methods* **14**, 687-+, doi:10.1038/nmeth.4324 (2017).

12 Benjamini, Y. & Hochberg, Y. Controlling the False Discovery Rate - a Practical and Powerful Approach to Multiple Testing. *J R Stat Soc B* **57**, 289-300 (1995).

13 Gu, Z., Eils, R. & Schlesner, M. Complex heatmaps reveal patterns and correlations in multidimensional genomic data. *Bioinformatics* **32**, 2847-2849, doi:10.1093/bioinformatics/btw313 (2016).

14 Love, M. I., Huber, W. & Anders, S. Moderated estimation of fold change and dispersion for RNA-seq data with DESeq2. *Genome Biol* **15**, 550, doi:10.1186/s13059-014-0550-8 (2014).

15 Zhu, L. J., Gazin, C., Lawson, N. D., Pages, H., Lin, S. M., Lapointe, D. S. & Green, M. R. ChIPpeakAnno: a Bioconductor package to annotate ChIP-seq and ChIP-chip data. *BMC Bioinformatics* **11**, 237, doi:10.1186/1471-2105-11-237 (2010).

16 Robinson, J. T., Thorvaldsdottir, H., Winckler, W., Guttman, M., Lander, E. S., Getz, G. & Mesirov, J. P. Integrative genomics viewer. *Nat Biotechnol* **29**, 24-26, doi:10.1038/nbt.1754 (2011).

17 Bailey, T. L., Johnson, J., Grant, C. E. & Noble, W. S. The MEME Suite. *Nucleic Acids Res* **43**, W39-49, doi:10.1093/nar/gkv416 (2015).

18 McLeay, R. C. & Bailey, T. L. Motif Enrichment Analysis: a unified framework and an evaluation on ChIP data. *BMC Bioinformatics* **11**, 165, doi:10.1186/1471-2105-11-165 (2010).

19 Grant, C. E., Bailey, T. L. & Noble, W. S. FIMO: scanning for occurrences of a given motif. *Bioinformatics* **27**, 1017-1018, doi:10.1093/bioinformatics/btr064 (2011).

Supplementary Figures

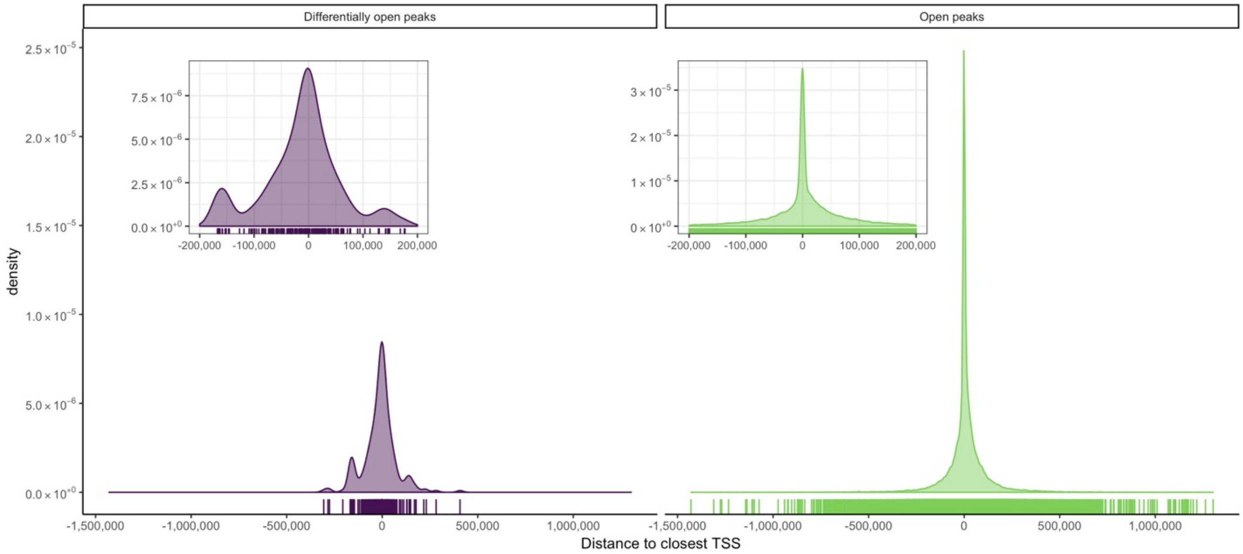

Figure S1. Accessible chromatin is mainly located distal to annotated genes. Distribution of accessible chromatin peaklets was plotted to determine the distance of the peaklet center to the nearest transcriptional start site (TSS). There were 42,198 high confidence open peaklets (green, right) identified ($p < 10^{-10}$). Accessibility of 233 peaklets changed in at least one time point compared to controls were considered differentially open (purple, left). Insets show magnified view of distribution of sequences within 200,000 kb of the TSS. There was a mostly equal distribution of sequences located upstream and downstream of the TSS, 20% found within 1 kb of the TSS.

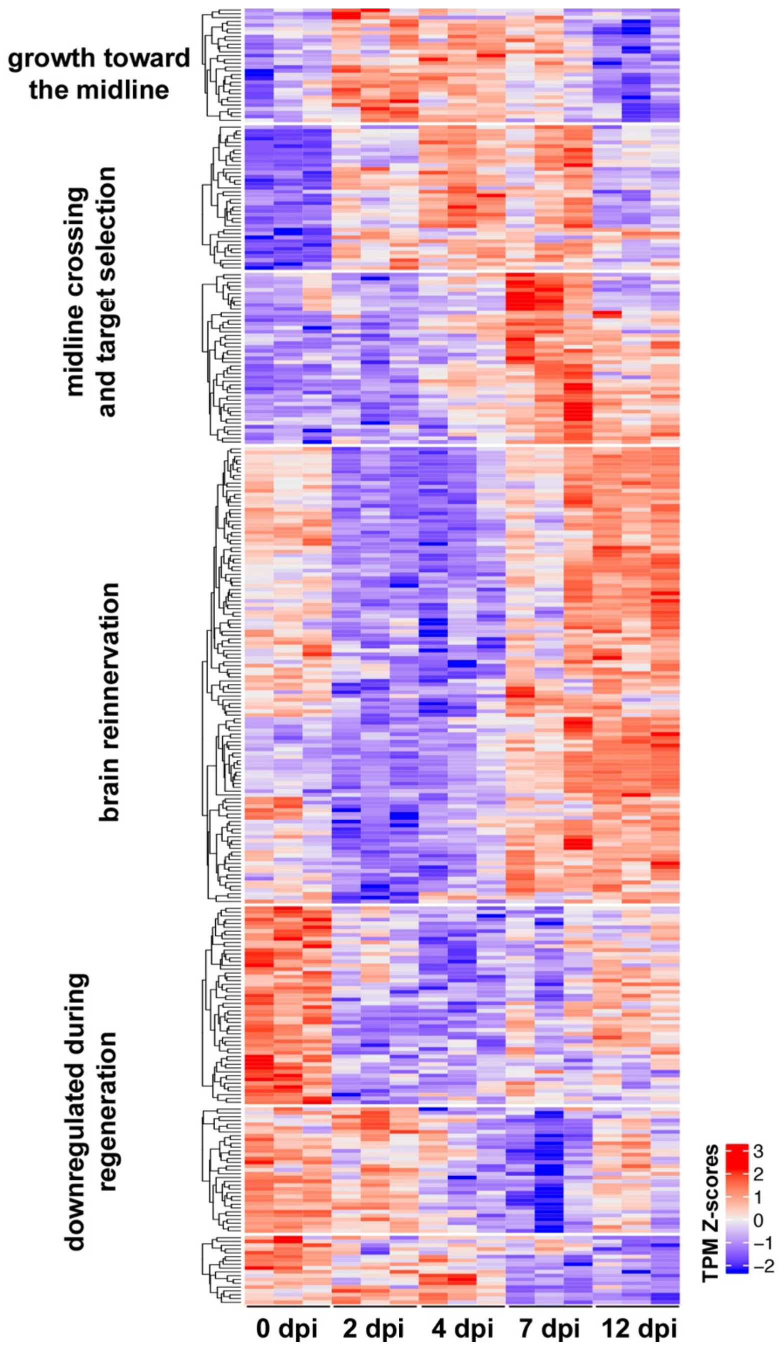

Figure S2. Differentially expressed transcripts that encode transcription factors display regeneration stage-specific temporal patterning. The expression heatmap of 339 differentially expressed transcripts represents 265 unique transcription factor (TF) genes. Unlike in Fig. 2C, this heatmap includes TFs with and without known DNA binding motifs.

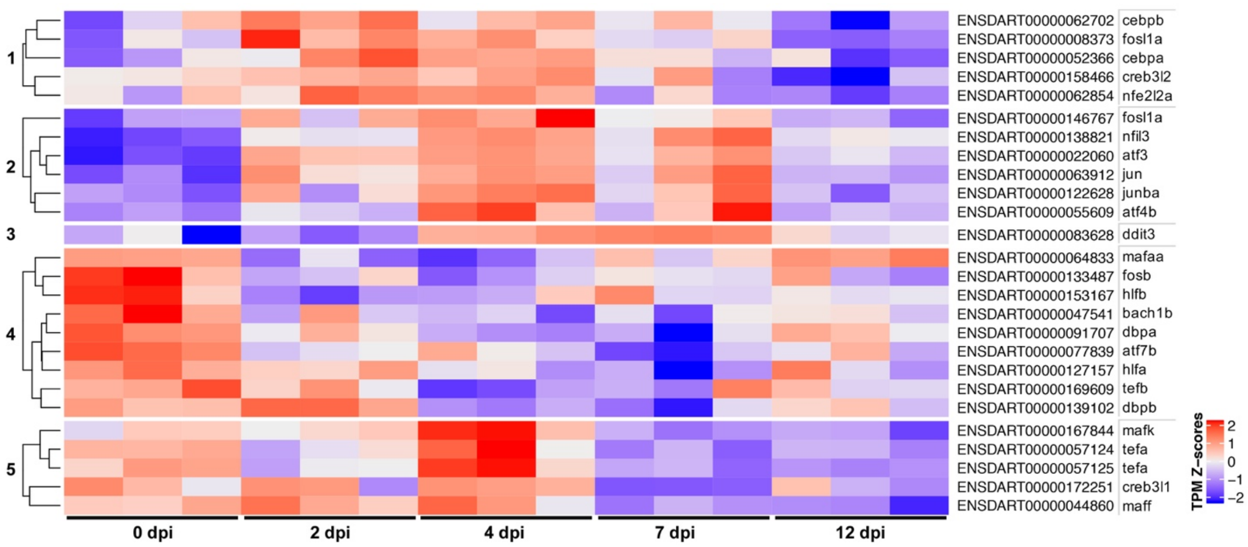

Figure S3. Temporal patterning of differentially expressed leucine zipper (bZIP) family transcription factors.

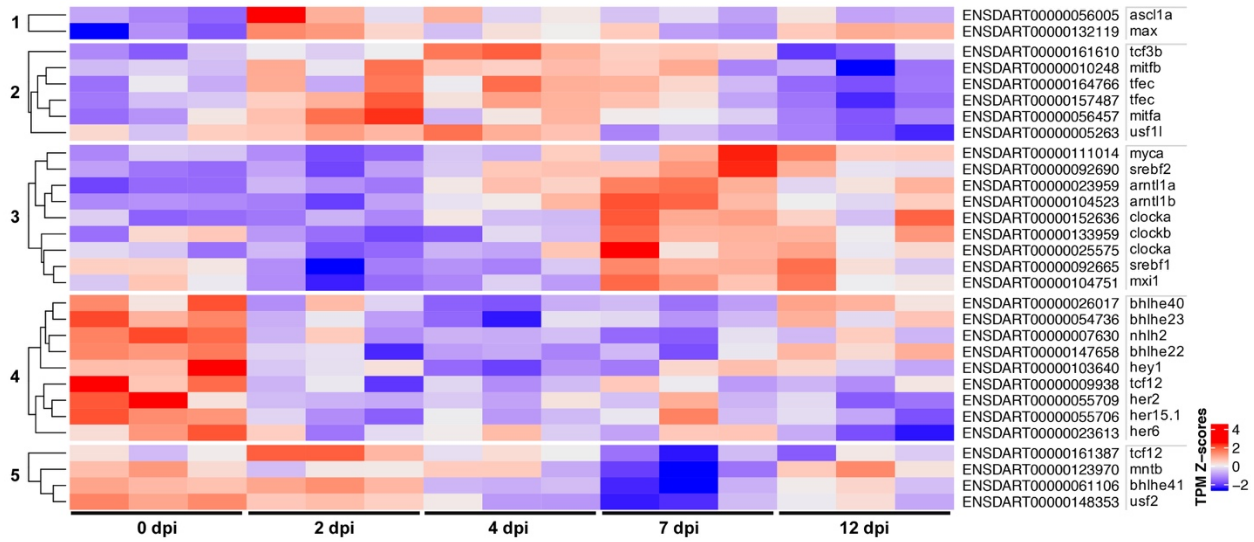

Figure S4. Temporal patterning of differentially expressed basic helix-loop-helix (bHLH) family transcription factors.

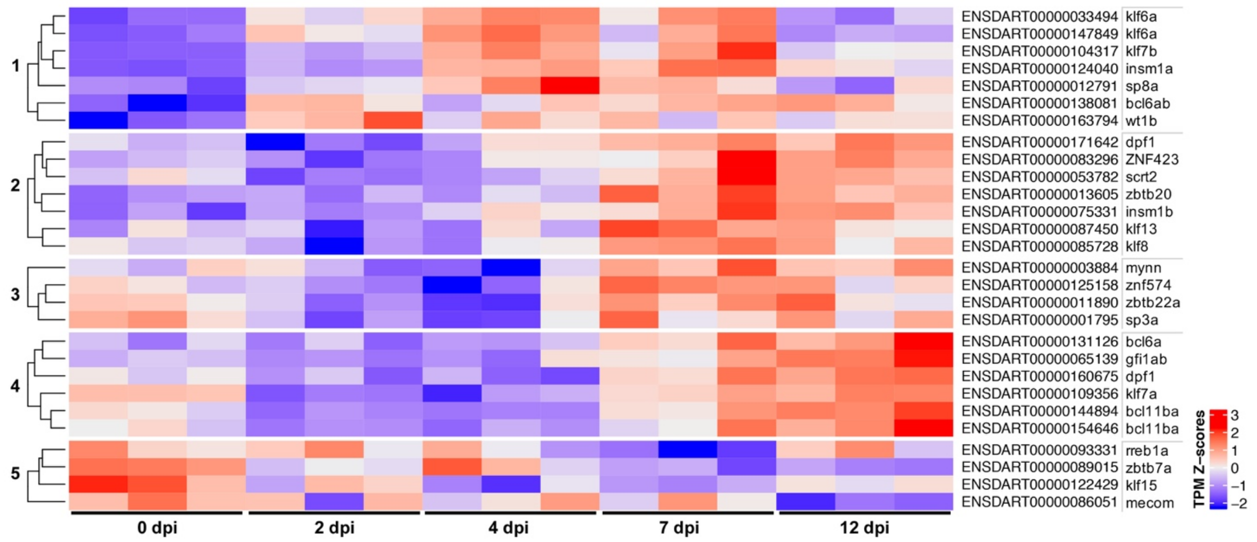

Figure S5. Temporal patterning of differentially expressed 2 cys-2 his zinc finger (C2H2ZF) family transcription factors.

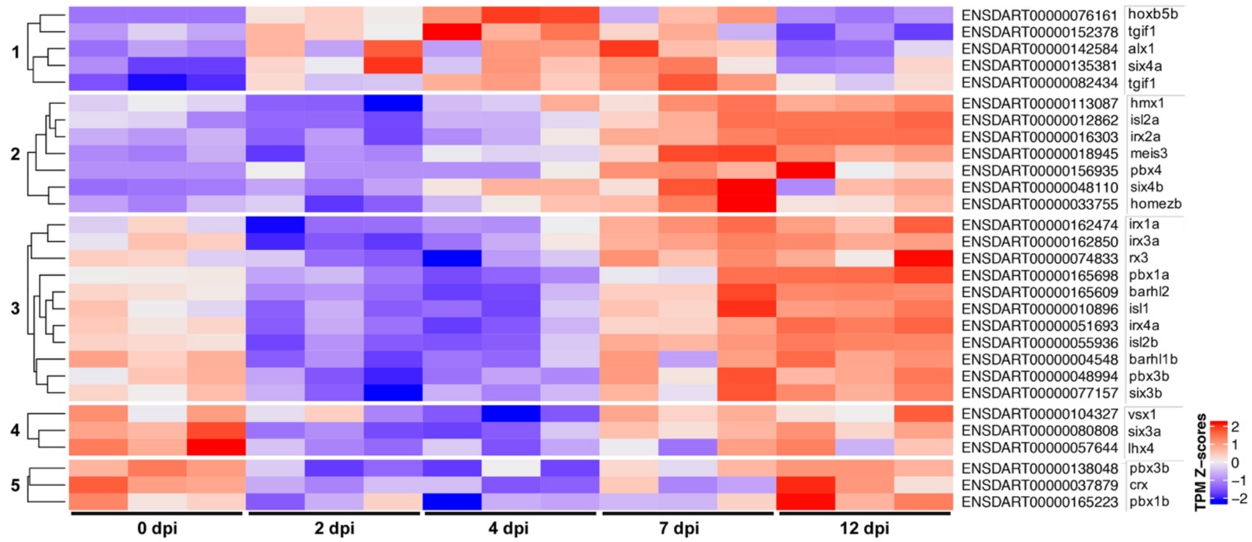

Figure S6. Temporal patterning of differentially expressed homeodomain family transcription factors.

>99999 TRANSGENE_contig
tcgactgtgtggcttccgtttgatccacaattgataattaaagggctggggtgaaacacggatcctcgtcttgtctcgtgtcttaactg
cttcttttccattcatacttgtaggttttaatatttcactctgagctggagcaattatcattcctgaatcatctgaactgccatggcaaca
gcttgctgatgtcagccaggagaataaataaatgaaacccttgaaacatctgccttttgctgagaattgtggttacaacttatcaata
caactaaccactgatggaatgtaactaaatccctgtgaatgatttccacatctgattagtttcattttatattttacttccactctgcgat
gtttttaaactgaccagtgttactttgcttgcaaaggaagaacatattggagttttattcacatttgcaaacacttaaacacaaaattt
agttcccagacttttgcagcaagttatactagtttaacaggttggccccttaaagtacttttgtcttctgctacttcctgatcgaaatacg
tttaatccgttttatttcacaatcggttgatgtttgtatccgagatgatacagaagtaaaactttttaaaagatttttttaaaagattttttt
tacaagcgtctaatccggccccagaatcacaatgctccagcaaaagctgcagtcaagtctccaaatcttgctgaatatgtatcatcg
tccatattcatcaggtcaaatttgtctattgttgaatatttactagactaaagagtaaatgcattcaaaattctgagttggtagaggag
caaaacttctctgcttcagatttcaacaaaaggaatcactcatttactcgattactccacaccaacgttgctaggatacctgagaaat
tgcccccaaaaacctacattcaagtagaattttcatctcagacgcattcggaatattggtgaagctgctcataaactctggaaaaac
ctctgaaatgcgttcggcacttgctcacctccacttgccgggtgaggaggaccgtgcacggctgttccaggtctcctcgcaggagat
gatgcatcagtgttctgcactgcagacaaacgtggcgggactaacagccattcagacatcagactctaagctcttaatcctacagtc
agatttgatcgtggtgagacgaaaaccttcaaattgcacttgatacatatacccaagcatgtggcagcgctttgttgccatagcaac
aacaactagagttgtcttctgcgttgactcctgcctcagctgctgtggttcacccggtgtcacctgcagactctaagttgatgtgtgcg
gtgactctaagaatgctctcattactgacaatcctggaaatttaattaaaaaatactatcactttgtttcctggtgtacgaggacacg
gacgtgcgcaggttagcttagcttggtagtttactgttagtgtcaaactgatggagtaaaagactgtaacagttaattaatgagggtg
ttaaaaggtcacagggttgatgtgcaacatttctggtgcgctcggatggttctttctcaaaagtatcctattttcgagacagagtgcca
atgaagaccttacacagggtgttgtcaggttgtacctgacagcttctcaatacagcgcgacactatcgatccgtgcaatcactacag
cggcagattctaccccttactggaaagtccatgtccagatctggacatggacttagcatatgctaatgtgttccagagcagttgatg
gagtttgcatacacagttctgtgtatctgggcaaacaaggcgtccaatcatctcttcatttagagtgttgaagattgcaaaaatctgt
aattggtgaatagatcagcacgtgttaatgaaacggataatttaatttatggctggttgccattactcactatattgtagagcaatga
tttggtatctgtcaggatttgcagtgtagtgaaaatgaagcgtaacagttggagaaatactacttaagtttttttttttccaagattta
gctgtgattgatgttcccctatcttccgtaatcaactaatagaagaaatttaattcaatctggcagcctctgaaaaacttacatcccc
tatttaagggcttcaaaaactgctcggtgaaaaactacccccagaaacaatgacttcagaacttggatgatctgaaacaatgaaa
cgcgaacagtggatttcatctgcatgattgtattaaattaaattaaacccctgagaagaaggtgtgtaaaggacaagcagggcagc
ggcagcggcagtggcaccggcagcctgcatatttccacgatgtgtgtcatccaggccagttcagaccatgatgtcacagtggcgctg
acgtattctgcattcaagtaacgatgaccaccaggaaggctgtggctcagcaggctgcttaatatcgcccatccatccatccatccat
ccatccatccatccatccatccatccatccatccatccatccatccatccatccatctgtagcatctgctgctgttaatgcgggggtgg
ggacactcagacaccagtttgtgttcctgtgtgtgtgcgtgtgtattatggaaaacaaatacggagtgtgggtgttttgcatggacat
gcacctgccatgctctgtaaatactgatggtgagacagtgccgggggggtgagggcaggggtgtgctcattaaaccacactgtgtg
tcaatgtgggcaggaaaaaatgggggagggggagagaaacagagtcagttcttcttgtctgccacacccggccctcttgcctcttttc
ttctctctccctattcctctctctctctctctcttcctattcctctttctctttccctcgctctctcttactcctcctcctctgctcttctggtc
tatctgctggtgtttccatgagaacagagtgagctgcgtttcggggggagtagtggtagtgagggtgatgtggcttaaatgcgcgcg
cgcgtgtgtgagtgtgcacgcacgtgtgtgtgtgtggcatcatcatctttctcacattgctcctggcagtgagatgctgctggctgagc
caaaaaccatctttgtgacatcgccgtggcaatttggaaacactcattaaagacttggaaagtgaaaagcgagggcaacatatgta
tatggctgcacgcttgatgaaaggatacgatgatacagatgagtggataaagcaaattggaggtacccttgcagctattaagggag
aacctttttttagggtggggccgcatcttgaagtcaggcagagagacgaggcggggaggagggagggagggagggagggaggga
gggaggggtttaaagggtgggcttacagtcattgtcagtgagagcgagagaaaaagagagagaggagcaactgaagaaggagt
gcaagcagactgggatgagtaaaagcactgggcagcttagctgttgatacagcctgaaagagagaaagagagagagagagagt
gtgtgtgtgtgcgcgagagcgtgtgagagagaagtgaaaaattgaggagcaaactcaagggaagaaagaagcagagcatcaaa
agggaggatagaaccaacttgaaccatgctgtgctgcataagaagaaccaaaccggtcgccaccatggtgagcaagggcgagga
gctgttcaccggggtggtgcccatcctggtcgagctggacggcgacgtaaacggccacaagttcagcgtgtccggcgagggcgag

ggcgatgccacctacggcaagctgaccctgaagttcatctgcaccaccggcaagctgcccgtgccctggcccaccctcgtgaccac
cctgacctacggcgtgcagtgcttcagccgctaccccgaccacatgaagcagcacgacttcttcaagtccgccatgcccgaaggcta
cgtccaggagcgcaccatcttcttcaaggacgacggcaactacaagacccgcgccgaggtgaagttcgagggcgacaccctggtg
aaccgcatcgagctgaagggcatcgacttcaaggaggacggcaacatcctggggcacaagctggagtacaactacaacagccac
aacgtctatatcatggccgacaagcagaagaacggcatcaaggtgaacttcaagatccgccacaacatcgaggacggcagcgtg
cagctcgccgaccactaccagcagaacacccccatcggcgacggccccgtgctgctgcccgacaaccactacctgagcacccagt
ccgccctgagcaaagaccccaacgagaagcgcgatcacatggtcctgctggagttcgtgaccgccgccgggatcactctcggcatg
gacgagctgtacaagtaaggatccactagtgatgcagatccccggatctttgtgaaggaaccttacttctgtggtgtgacataattg
gacaaactacctacagagatttaaagctctaaggtaaatataaaatttttaagtgtataatgtgttaaactactgattctaattgtttg
tgtattttagattccaacctatggaactgatgaatgggagcagtggtggaatgcctttaatgaggaaaacctgttttgctcagaaga
aatgccatctagtgatgatgaggctactgctgactctcaacattctactcctccaaaaaagaagagaaaggtagaagaccccaagg
actttccttcagaattgctaagttttttgagtcatgctgtgtttagtaatagaactcttgcttgctttgctatttacaccacaaaggaaa
aagctgcactgctatacaagaaaattatggaaaaatattctgtaacctttataagtaggcataacagttataatcataacatactgtt
ttttcttactccacacaggcatagagtgtctgctattaataactatgctcaaaaattgtgtacctttagctttttaatttgtaaaggggtt
aataaggaatatttgatgtatagtgccttgactagagatcataatcagccataccacatttgtagaggttttacttgctttaaaaaac
ctcccacacctccccctgaacctgaaacataaaatgaatgcaattgttgttgttaacttgtttattgcagcttataatggttacaaata
aagcaatagcatcacaaatttcacaaataaagcatttttttcactgcattctagttgtggtttgtccaaactcatcaatgtatcttatca
tgtctggatctgcatattctatagtgtcacctaaatctgc

Fig. S7. Sequence of GAP43-GFP transgene. Transgene sequence in FATSA format. GAP43-GFP fusion protein coding sequences highlighted in yellow.

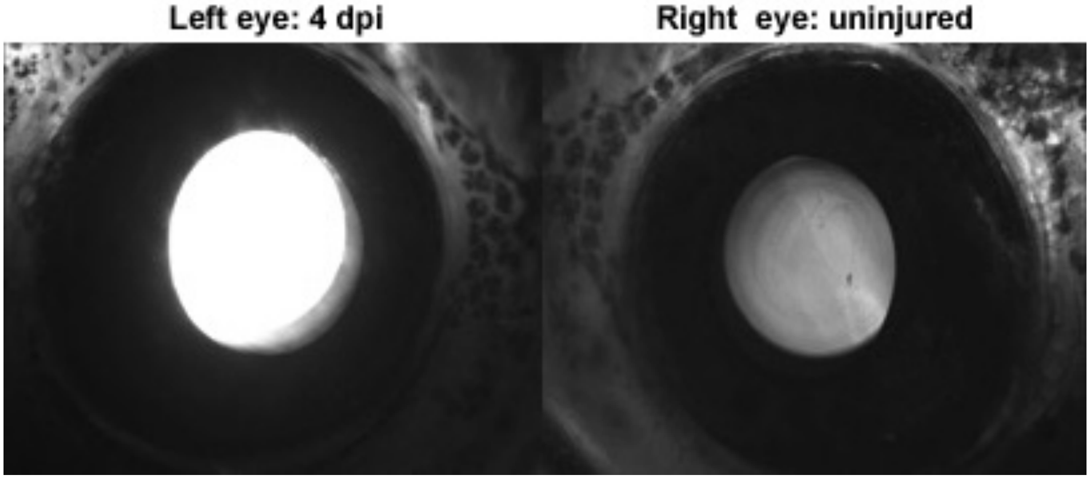

Figure S8. Injury-induced eGFP expression in retinal ganglion cells can be visualized through the lens of intact animals. Optic nerve injury can be validated based GFP expression, which is easily visualized through the lens. Example of injury and naïve eyes from the same fish 4 dpi.

Table S1. Transcripts differentially expressed compared to controls (0 dpi) in at least one time point (5% FDR).

Ensembl transcript ID	zebrafish gene symbol	LFC: 2dpi-0dpi	LFC: 4dpi-0dpi	LFC: 7dpi-0dpi	LFC: 12dpi-0dpi
ENSDART00000000069	slc9a3r1a	-0.019828621	-0.165096913	-0.579587833	-0.318451026
ENSDART00000000192	ptpn4b	-0.253123991	-0.355280061	-0.416236309	-0.192007
ENSDART00000000280	stat1b	-0.005066017	0.035352481	0.819259146	0.412054224
ENSDART00000000486	cntn2	0.015280519	0.733067848	1.819345027	1.713832915
ENSDART00000000678	opn1mw4	-0.668549106	-0.384195773	-0.152811605	-0.428104315
ENSDART00000000744	sypl2b	0.279822836	0.157132955	0.007001172	-0.094022715
ENSDART00000000804	slc8a1b	-0.216070414	-0.730017061	-0.564883211	-0.309532287
ENSDART00000000876	nr4a1	-2.262372442	-2.399082592	-2.145415905	-1.543723254
ENSDART00000001313	rimbp2	-0.29201819	-0.480722019	-0.187717263	0.029819479
ENSDART00000001444	g2e3	0.958887749	1.066626105	0.575974767	0.288448068
ENSDART00000001678	adam8a	1.934717518	1.802449426	1.368408045	-0.21633234
ENSDART00000001795	sp3a	-0.269877105	-0.305437708	-0.047306155	-0.033496274
ENSDART00000001805	csmd2	-0.646636229	-0.876947398	-0.300952212	-0.072675683
ENSDART00000001861	slc6a22.1	0.506403045	0.610928969	0.211334169	-0.287297819
ENSDART00000001907	slc16a3	-0.228617593	-0.852511558	-0.452127784	-0.215137636
ENSDART00000002027	gulp1a	-0.229497305	-0.154479555	-0.273130036	-0.186901702
ENSDART00000002029	fkbp8	-0.081020923	-0.107047698	-0.329759128	-0.094897987
ENSDART00000002186	uck2a	0.69693934	0.733529046	0.658757388	0.439621207
ENSDART00000002250	hs6st2	0.156001692	0.361544481	0.514379395	0.208727247
ENSDART00000002309	mafba	0.316562739	0.132730939	0.096554959	0.286187946
ENSDART00000002393	rundc3aa	-0.355283185	0.971542583	1.209230568	0.944629469
ENSDART00000002453	acsl4b	0.682559347	0.484852434	0.056581739	-0.183902113
ENSDART00000002469	hspa4b	0.156626095	0.21868197	0.285696466	0.103348348
ENSDART00000002501	ip6k2a	-0.189409699	-0.526120261	-0.315517036	-0.114843314
ENSDART00000002556	mrto4	0.589078781	0.447556222	0.419365915	0.058126759
ENSDART00000002595	rpl21	0.36985839	0.3662658	0.201825483	-0.004056179
ENSDART00000002641	kif26aa	0.255726279	0.47696992	0.58781469	0.342072456
ENSDART00000002684	ddx26b	-0.289572133	-0.160183273	-0.164172394	-0.052122897
ENSDART00000002691	tspan7b	-0.507607245	-0.383440036	-0.192864148	0.037733122
ENSDART00000002741	itprip	0.724094373	0.694272578	0.397322653	-0.062298334
ENSDART00000002908	olfm1a	-0.612571688	-0.410284408	-0.115653646	0.220963821
ENSDART00000002932	marcksb	1.016829958	1.677411962	1.758104803	1.229873931
ENSDART00000002945	NPC2 (1 of many)	3.182180208	2.136779184	1.408480393	0.09501531
ENSDART00000002961	rcor2	-0.353333584	0.726682254	0.762799057	0.664353552
ENSDART00000003001	rpl23a	0.412203534	0.396855245	0.244635942	-0.052563059
ENSDART00000003008	gad1b	-0.375298425	-0.380219055	-0.456528647	-0.347996362
ENSDART00000003042	mdkb	-0.457982371	-0.488604373	-0.629508021	-0.485178616
ENSDART00000003066	cyth1a	0.591135613	0.827117646	0.654961234	0.137500638
ENSDART00000003076	usp28	1.676441425	4.659547988	4.384020448	3.839785573
ENSDART00000003170	mid1ip1l	-0.359873821	-0.329776238	-0.564188118	-0.321447922
ENSDART00000003193	gpr183a	0.785395164	0.62770375	0.383370825	-0.244463733
ENSDART00000003248	nek2	0.657099139	0.725246979	0.481556646	0.191253132
ENSDART00000003278	tacr3l	-0.098648497	-0.084970679	-0.345458842	-0.125397678
ENSDART00000003293	mid2	-0.366666589	-0.252968542	-0.080242025	-0.101833487
ENSDART00000003296	sars	0.182171112	0.390636242	0.505424837	0.283972917
ENSDART00000003303	rnf13	4.71068286	4.417862481	3.703911232	4.352869871
ENSDART00000003314	nusap1	0.825744448	0.908244509	0.647266522	0.3629471
ENSDART00000003335	snx12	-0.102084433	-0.181980514	-0.47779818	-0.16493026

ENSDART00000003346	pdcd2l	-0.007609279	-0.153705238	-0.388070334	-0.362770652
ENSDART00000003465	gipc2	-0.142551263	-0.216969239	-0.344609382	-0.269869981
ENSDART00000003475	ppef1	-3.70073529	-0.21405279	-0.165262449	-0.400509129
ENSDART00000003517	trmt61a	0.394655787	0.457245144	0.184403345	0.19719299
ENSDART00000003548	znf385a	-0.544762633	-0.635515273	-0.316063497	-0.128464507
ENSDART00000003550	nmnat2	-0.104759046	0.216048796	0.282752276	0.230034653
ENSDART00000003612		-0.145765054	-0.499207076	-0.35686816	-0.607333409
ENSDART00000003648	wdr3	0.482670527	0.422648714	0.349517488	0.089241222
ENSDART00000003690	acana	0.770985542	0.283939017	0.058793221	0.105884754
ENSDART00000003736	anos1b	-0.470963792	-0.442368787	-0.444507654	-0.318716779
ENSDART00000003745	vim	0.110317427	0.81438402	0.975636783	0.646819018
ENSDART00000003752	cct3	0.348496547	0.407130663	0.410390239	0.168454536
ENSDART00000003790	pwp1	0.32756693	0.411455433	0.339342319	0.085530698
ENSDART00000003825	cplx2l	-1.121107993	-0.523528158	0.430342309	0.533514858
ENSDART00000003891	jupa	0.359241944	1.297843964	1.552619484	0.724987275
ENSDART00000003913	ifih1	0.480118819	0.196152277	1.199192931	0.158891974
ENSDART00000003939	syngr1a	0.053450312	-0.149997984	-0.371497084	-0.066149387
ENSDART00000003947	flot2a	0.262220916	0.241361404	0.622111227	0.24129723
ENSDART00000003998	ewsr1b	0.136063726	0.084077199	0.439371598	0.241694854
ENSDART00000004034	hpca	-0.538662149	-0.391929378	-0.242660916	-0.195867715
ENSDART00000004043	enpp4	-0.15333394	-0.216742775	-0.388554121	-0.343087399
ENSDART00000004065	zgc:91909	0.594004329	0.47164071	0.350756766	0.105236702
ENSDART00000004075	uqcc2	-0.167833305	-0.222493804	-0.340331706	-0.224913678
ENSDART00000004109	gng3	-0.129761127	0.224238645	0.384018688	0.329285885
ENSDART00000004200	sarm1	0.137263218	0.386089559	0.382312262	0.28972731
ENSDART00000004238	rpl7a	0.295188189	0.336446742	0.303958011	0.013338352
ENSDART00000004241	inhbaa	0.208890493	-0.32024378	-0.370155553	-0.220482209
ENSDART00000004392	fkbp9	0.758370209	0.583896419	0.427971915	-0.289954899
ENSDART00000004416	lrp1ba	-0.680071532	-0.933155051	-0.315048731	0.19015716
ENSDART00000004420	rab4a	-0.104588348	-0.223085036	-0.330744686	-0.154422823
ENSDART00000004423	iars	0.363891152	0.394414274	0.599518689	0.155539422
ENSDART00000004474	mapre1b	0.531914947	0.849936108	0.970422656	0.515027849
ENSDART00000004521	arih2	-0.124662771	-0.251962223	-0.001375347	0.051084605
ENSDART00000004548	barhl1b	-2.472825095	-1.41656094	-0.268019579	0.230183047
ENSDART00000004550	rnf145a	-0.045034762	-0.327456151	-0.349649694	0.141940758
ENSDART00000004588	asic1a	0.159371955	0.158599541	0.248620425	0.288433907
ENSDART00000004601	laptm4a	-0.20461666	-0.134693111	-0.288435619	-0.251769605
ENSDART00000004622	sf3b4	0.222493415	0.286225027	0.527062646	0.243398323
ENSDART00000004626	sec62	-0.335700502	-0.25907023	-0.14783183	-0.155829549
ENSDART00000004656	GCA	0.318444393	0.283040087	0.060810751	-0.123164086
ENSDART00000004664	tram1	0.410276471	0.318086482	0.103470746	-0.163408872
ENSDART00000004717	igf1	-0.688444818	-0.421253017	-0.11318285	0.107184312
ENSDART00000004740	rab34b	1.81284078	2.340901968	1.779523446	1.639598632
ENSDART00000004780	man2b1	0.352578138	0.195228445	0.062298599	-0.189062834
ENSDART00000004903	rdh10b	-0.385872948	-0.19159484	-0.565090954	-0.486336464
ENSDART00000005053	slc12a4	-0.135764977	-0.039802706	-0.399029267	-0.999098872
ENSDART00000005086	atp1a1b	-0.434454878	-0.290341007	-0.096388542	-0.096973501
ENSDART00000005105	psme1	0.846890997	0.915850539	1.159835522	0.124970843
ENSDART00000005119	eif3i	0.208433038	0.349717288	0.244101269	0.053458955
ENSDART00000005143	mycb	0.44444506	0.5301099	0.532628488	0.161240961
ENSDART00000005191	uqcrb	-0.157513362	-0.342943294	-0.33904791	-0.120275765

ENSDART00000005299	hsd17b12a	-0.345584522	-0.261321345	-0.169519073	-0.263954396
ENSDART00000005337	rimkla	-0.133054455	0.30447461	0.568720788	0.413212633
ENSDART00000005366	tpd52l2b	0.128420316	0.410964244	0.59220548	0.186138167
ENSDART00000005381	zgc:110269	0.510417857	0.424502267	0.418900744	0.314541955
ENSDART00000005382	gadd45bb	0.573666184	0.621043614	0.188615995	0.24843391
ENSDART00000005453	chd4a	0.299643374	0.423708585	0.655765013	0.391160008
ENSDART00000005479	chm	-0.006745339	0.178224285	0.286548011	0.253225199
ENSDART00000005496	kctd9b	0.161429839	0.406047159	0.541753399	0.319048316
ENSDART00000005547	gnb3b	-0.185553204	-0.343974986	-0.508555347	-0.13419791
ENSDART00000005568	pdlim3b	0.923424522	1.668936507	1.372862264	1.014758632
ENSDART00000005573	tmem237b	0.093826402	-0.187405072	-0.456194249	-0.210768209
ENSDART00000005590	churc1	-0.030881068	-0.24423848	-0.319974325	-0.284115834
ENSDART00000005593	casp3a	0.498469533	1.198942327	1.332280554	0.912417093
ENSDART00000005609	kifap3a	0.203573738	0.628570592	0.743430197	0.605809471
ENSDART00000005616	rnpep	0.523525834	0.750055071	0.77634383	0.462027268
ENSDART00000005638	stxbp1b	-0.193317072	-0.160131943	-0.312017598	-0.116900269
ENSDART00000005720	stat1a	0.035880276	0.045195542	0.615137639	0.020671748
ENSDART00000005724	ncanb	-0.308688969	-0.586976696	-0.410319756	-0.132925594
ENSDART00000005733	tma16	-0.013223994	-0.144098168	-0.425909208	-0.168461973
ENSDART00000005738	slitrk2	-0.40615183	-0.499760308	-0.343598039	-0.061449053
ENSDART00000005740	mef2aa	-3.741432455	-2.723175321	-1.742061709	-1.012535024
ENSDART00000005784	itgb1bp1	0.440140968	0.384181368	0.282182476	-0.012450945
ENSDART00000005842	fgf1a	-0.526483511	-0.462706743	-0.482028176	-0.1224057
ENSDART00000005847	nav3	0.773382381	1.009593057	0.901548272	0.783565613
ENSDART00000005869	rpp14	-0.113270836	-0.030182103	-0.321668148	-0.171027578
ENSDART00000005929	ppp3ca	-0.536938929	-0.189476468	0.016656951	0.377870736
ENSDART00000005944	rpl5a	0.381988038	0.550810639	0.338076702	0.013320357
ENSDART00000005957	lrit1a	-0.250152343	-0.282879742	-0.392873609	-0.351289311
ENSDART00000005989	dffb	0.378338656	0.455339874	0.443055793	0.165200782
ENSDART00000006058	eif2s1a	0.414016542	0.357076684	0.28859634	0.112415797
ENSDART00000006061	tcea3	-0.180343963	-0.073708986	-0.351054612	-0.319778395
ENSDART00000006085	cbl	0.165119386	0.1775588	0.379174787	0.206196339
ENSDART00000006132	cfl1	0.199336875	0.296057395	0.303504194	0.166132927
ENSDART00000006211	prkcba	-0.217161161	-0.38326462	-0.338691874	-0.182528175
ENSDART00000006290	plekhb2	-0.293609185	-0.084638835	-0.118110885	-0.124049463
ENSDART00000006380	tbx3a	-0.244591708	-0.352646993	-0.144447405	0.06961498
ENSDART00000006381	psen2	0.338086739	0.204848015	0.067000942	-0.024014416
ENSDART00000006417	pgm1	-0.367653769	-0.345273975	-0.348210636	-0.086795004
ENSDART00000006435	gpr27	-0.291018325	-0.294873264	-0.134468137	0.025589784
ENSDART00000006474	glra4b	-0.56482245	-0.497893783	0.107334542	0.502166045
ENSDART00000006489	acsl4a	0.116872844	1.158097412	1.477099844	0.59458295
ENSDART00000006513	pdhb	-0.272240562	-0.336402892	-0.245891496	-0.069634702
ENSDART00000006602	pde4a	-0.284660623	-0.353015908	-0.053068214	0.108985293
ENSDART00000006604	clpp	0.129850837	0.170330862	0.350507109	0.146117928
ENSDART00000006612	tbr1b	-1.166029945	-1.008207372	0.617040877	1.169470893
ENSDART00000006619	rbpms2b	-0.996572205	-0.617915271	-0.145310289	0.143289699
ENSDART00000006724	smarcd3b	-0.214336649	0.06424201	0.499562006	0.504466248
ENSDART00000006778	acat2	-0.122422954	0.489045334	0.688039864	0.280940334
ENSDART00000006802	cct7	0.251283975	0.283261629	0.291096583	0.139424973
ENSDART00000006843	cacng1a	0.923524414	3.482209925	3.70002605	2.994326832
ENSDART00000006908	itgb3b	1.52546911	1.915133846	1.613887964	0.668451164

ENSDART00000006927	use1	-0.314698173	-0.230168127	-0.342013543	-0.20782604
ENSDART00000006990	elovl5	-0.296553838	-0.148251934	0.039955151	-0.062523987
ENSDART00000007021	atp6v1ba	-0.168038972	-0.268033099	-0.367112615	-0.152327119
ENSDART00000007103	nuak1a	-0.384666887	-0.409555272	0.121768678	0.275010156
ENSDART00000007122	guca1b	-0.373174817	-0.243090423	-0.518795688	-0.641476933
ENSDART00000007204	ddx49	0.428211201	0.38977455	0.371001972	0.147828916
ENSDART00000007208	lrrc4bb	-0.502121898	-0.528156819	0.019633372	0.067706598
ENSDART00000007231	psmb1	0.336843399	0.41477024	0.441532829	0.103495117
ENSDART00000007271	mtfr1l	-0.183189215	-0.389013192	-0.122809179	-0.106769923
ENSDART00000007293	tcap	1.354758323	0.76915174	1.088235317	0.716372996
ENSDART00000007308	wnt10a	-1.246823676	-1.58888825	-0.322358389	0.209755977
ENSDART00000007401	MAP3K13	-0.231843501	-0.363439198	-0.331818795	-0.269324908
ENSDART00000007512	pole3	0.11728021	0.33533824	0.217028028	0.042569097
ENSDART00000007522	anos1a	-0.500742525	-0.407287946	-0.526812004	-0.211300468
ENSDART00000007531	slit2	-0.281096395	-0.311205466	-0.174560483	0.038385548
ENSDART00000007584	snap25a	-0.520469397	-0.457109198	-0.208714288	-0.117586032
ENSDART00000007587	gtf2h2	0.253100515	0.350059727	0.329257684	0.204207466
ENSDART00000007624	plch2a	-0.294880531	-0.004127951	0.226839247	0.19499931
ENSDART00000007630	nhlh2	-0.28794611	-0.324673306	-0.385652145	-0.261092419
ENSDART00000007642	zgc:110239	0.831334701	0.383173307	0.0742642	-0.168536089
ENSDART00000007778	grik1a	-0.423674769	-0.190804556	-0.271114709	-0.0631136
ENSDART00000007789	idh1	0.209391072	0.491026617	0.59658334	0.190896441
ENSDART00000007797	slc30a4	0.328612859	0.21837447	0.122260678	0.141372312
ENSDART00000007806	zbtb16a	-0.634450415	-0.731920804	0.190781828	-0.364381887
ENSDART00000007827	spra	0.429147243	0.5653912	0.349944557	-0.12347627
ENSDART00000007856	fkbp16	-0.168302487	-0.129290801	-0.313457803	-0.012446152
ENSDART00000007857	mettl2a	0.337109376	0.448868897	0.378403392	0.127678255
ENSDART00000007961	nt5c2l1	0.559923749	0.945665914	1.614610333	0.555210778
ENSDART00000007972	dlgap4b	-0.346497047	-0.558852799	-0.093125576	-0.08715572
ENSDART00000008010	pdk2a	-0.070635847	0.148907691	-0.31523766	-0.171233724
ENSDART00000008038	sulf2a	0.420520728	1.274767381	2.360438199	2.491815243
ENSDART00000008058	aak1a	-0.071484403	-0.294378944	-0.458052722	-0.070668535
ENSDART00000008152	sgk2b	1.195404929	0.789404089	0.784574894	0.645661759
ENSDART00000008287	pgam1a	-0.270559913	-0.416576315	-0.324017463	-0.168254728
ENSDART00000008302	insra	-0.066772744	-0.178139649	-0.319689592	-0.098012672
ENSDART00000008326	pon2	0.005358778	-0.010211923	-0.591626082	-0.490299784
ENSDART00000008373	fosl1a	0.78271954	0.570182098	0.226810387	-0.332448721
ENSDART00000008402	sart3	-0.246563675	-0.213758299	-0.263959387	-0.22518745
ENSDART00000008594	tmem178	-0.626672748	-0.469011917	-0.272377327	0.052520712
ENSDART00000008607	ttyh2l	0.127438148	-0.071060271	-0.346686037	-0.327410338
ENSDART00000008638	rgma	-0.505076193	-0.28541733	-0.181405309	0.127564695
ENSDART00000008663	adam10b	0.007023444	0.111335196	0.430661155	0.328928973
ENSDART00000008711	gys1	-0.668450567	-0.607630136	-0.28295897	-0.009086585
ENSDART00000008785	anp32a	-0.194673883	-0.086461888	-0.275568041	-0.184432261
ENSDART00000008807	rpl12	0.258765274	0.397825613	0.190712058	-0.053203791
ENSDART00000008840	otofa	-0.788283647	-0.975123326	-0.33101193	-0.149384457
ENSDART00000008854	wsb1	0.135165037	0.298497884	0.131347407	-0.114776528
ENSDART00000008906	znf503	-0.367880648	-0.389528031	0.161538025	0.184463
ENSDART00000008986	atp6v1e1a	0.082323928	-0.133398426	-0.212735096	-0.280773093
ENSDART00000009164	esco2	1.573729566	0.878542321	0.358323204	0.160150267
ENSDART00000009178	impdh2	0.589155818	0.398351191	0.511972012	0.456829015

ENSDART00000009194	aimp2	0.035867833	0.204745928	0.516008081	0.307277143
ENSDART00000009241	rpl35	0.440738233	0.45418276	0.310964715	0.055658665
ENSDART00000009277	tuba1a	0.466164103	1.394077049	1.773814961	1.31379942
ENSDART00000009337	eno1a	-0.396364992	-0.428345122	-0.288646494	-0.130833414
ENSDART00000009343	pyroxd2	0.778556587	0.730253969	0.295998209	0.188824159
ENSDART00000009393	col1a1a	0.640444957	1.804362836	2.839654111	1.436438571
ENSDART00000009477	cct8	0.2739469	0.4225965	0.299361505	0.113502813
ENSDART00000009484	cct6a	0.227309436	0.27374384	0.264898076	0.101996724
ENSDART00000009549	rhag	0.97898574	1.27206332	1.678759018	0.617102069
ENSDART00000009569	slc12a5b	-0.543274129	-0.639831062	-0.420973005	-0.072437192
ENSDART00000009609	eif5a	0.382861639	0.513300938	0.466153608	0.171038659
ENSDART00000009653	kcna1b	-0.51649998	-0.917921415	-0.602149268	-0.420615578
ENSDART00000009689	mhc1uba	0.3733109	0.635354786	0.756757815	0.245538965
ENSDART00000009691	scml4	-0.348233268	-0.325522885	-0.113421585	0.115178638
ENSDART00000009740	smad7	-0.082405078	-0.352637537	-0.492507075	-0.066549718
ENSDART00000009777	glra3	-0.385455571	-0.554614528	-0.516083394	-0.22262256
ENSDART00000009888	casq1b	2.284244029	1.995698495	4.521796612	3.078320888
ENSDART00000009892	gabbr1a	-0.618262887	-0.683782394	0.013008531	0.484024166
ENSDART00000009938	tcf12	-0.385864267	-0.351608927	-0.283149928	-0.323464659
ENSDART00000009952	zfand5a	-0.376361025	-0.47427013	-1.180526792	-1.063292908
ENSDART00000010041	dhfr	0.095253657	-0.024940068	-0.334824981	-0.34089932
ENSDART00000010046	rhpn2	-0.531285649	-0.280012714	-0.367908087	-0.481376216
ENSDART00000010104	crtap	0.225199403	0.115819527	-0.25953471	-0.588755148
ENSDART00000010119	eef1a2	-1.586399577	-1.404358062	-0.455450934	0.174130308
ENSDART00000010140	igf2bp3	0.357904839	0.436869624	1.38352682	1.118120985
ENSDART00000010144	pvalb2	0.379417352	4.690875982	5.447468716	3.629116223
ENSDART00000010199	fam219ab	-0.329176512	-0.152550548	-0.14317011	0.01066054
ENSDART00000010246	ugt1ab	1.578212697	1.169819697	0.509084599	-0.046131609
ENSDART00000010256	eif3m	0.301540845	0.415293475	0.295465222	0.073592881
ENSDART00000010257	fam73a	-0.203941778	-0.403469011	-0.199714259	-0.045216291
ENSDART00000010261	pno1	0.396735226	0.379415162	0.20506239	-0.042887698
ENSDART00000010271	aida	0.207262438	0.531449642	0.462716258	0.148174666
ENSDART00000010274	dpysl5a	0.119382655	1.052869734	1.414498731	1.230769727
ENSDART00000010282	cbr1l	0.612622623	0.498855932	0.466697421	-0.088158588
ENSDART00000010378	myo3b	0.121341422	-0.396947151	-0.677290933	-0.232688387
ENSDART00000010420	actr1	0.079833108	0.201277948	0.258101559	0.148775132
ENSDART00000010452	zgc:91860	-0.346186809	-0.437676548	-0.463741509	-0.203120402
ENSDART00000010495	znrf1	0.045008698	0.17288769	0.379569247	0.252907322
ENSDART00000010512	zgc:92907	-0.132831847	-0.132364384	-0.338242819	-0.200606823
ENSDART00000010647	rcc2	0.312248055	0.54892666	0.398800486	0.1537421
ENSDART00000010683	impa1	0.486043053	0.448191747	0.382482237	0.291162031
ENSDART00000010757	rgmb	-0.151394227	0.132378035	0.469172545	0.35352068
ENSDART00000010824	acy1	-0.16534468	-0.281016974	-0.38333817	-0.31990568
ENSDART00000010982	fgf13a	-0.282182655	-0.227308636	-0.321295112	-0.222652121
ENSDART00000010997	tpm3	0.446911874	0.508209148	0.705423057	0.171806011
ENSDART00000011004	mfsd5	-0.162600193	-0.289194121	-0.512584457	-0.472173717
ENSDART00000011052	eif3d	0.194265429	0.254685579	0.234360887	-0.025541379
ENSDART00000011135	kita	-0.1368269	-0.266279657	-0.502022945	-0.592221276
ENSDART00000011143	myd88	0.15184495	0.21846972	-0.215204616	-0.645828486
ENSDART00000011149	fam185a	-0.225870361	-0.305876809	-0.396323932	-0.356813487
ENSDART00000011224	itga10	2.439207816	3.888845597	4.302533523	3.055176776

ENSDART00000011229	sub1b	-0.082257398	-0.179031151	-0.405374566	-0.271111897
ENSDART00000011251	rpl3	0.32045382	0.346921833	0.271797845	0.014406427
ENSDART00000011258	npl	0.373272645	0.54061543	0.468726439	0.170404917
ENSDART00000011283	cnr1	0.295670673	0.19447276	0.12958679	0.047335468
ENSDART00000011287	aqp4	-0.584037248	-0.688442177	-0.670108654	-0.488547149
ENSDART00000011305	dpp3	0.260304454	0.228408409	0.405249148	0.162562787
ENSDART00000011317	ntm	-0.235410973	-0.452806913	-0.580240928	-0.252707536
ENSDART00000011362	arrdc2	0.681126697	0.66886629	0.476399009	0.092022718
ENSDART00000011398	si:ch73-335l21.1	-0.01193014	-0.263037651	-0.302583762	-0.048051484
ENSDART00000011447	sae1	0.282597281	0.674186287	0.734155697	0.424524708
ENSDART00000011453	sypb	-0.342744234	-0.453708067	-0.33513289	-0.074863584
ENSDART00000011456	tsg101b	0.363139745	0.1150057	0.061390783	-0.157671493
ENSDART00000011519	slc6a1l	0.105782424	-0.162202998	-0.466558128	-0.662799909
ENSDART00000011568	syngr3a	-0.064295047	0.234968681	0.63147157	0.633880469
ENSDART00000011570	zgc:101716	0.810996291	0.904458728	0.54949556	0.44631829
ENSDART00000011627	irx7	-0.27830639	-0.509115666	-0.233145912	-0.150983114
ENSDART00000011691	baxa	0.500319714	0.752407609	0.690306826	0.30333895
ENSDART00000011699	nono	0.031722902	0.329157035	0.240624596	0.057167441
ENSDART00000011863	hdlbpa	0.44689202	0.322223522	0.244841165	-0.050194137
ENSDART00000011865	sec23b	-0.409405473	0.102005828	0.534469651	0.138588714
ENSDART00000011878	eif4a1b	0.134279506	0.344064551	0.309281202	0.284267369
ENSDART00000011936	ccdc106a	-0.452156269	-0.26003137	-0.330808493	-0.261299438
ENSDART00000012023	faimb	-0.164145301	-0.164693039	-0.391621682	-0.153828799
ENSDART00000012119	zgc:110366	-0.07598566	0.059679618	-0.31484475	-0.244737795
ENSDART00000012164	tmod2	0.222306993	0.411676025	0.560901639	0.297325519
ENSDART00000012229	fkbp1b	-0.199888531	-0.218085861	-0.490853212	-0.25173597
ENSDART00000012247	dhcr24	2.392110544	2.697063662	3.119814675	2.653468904
ENSDART00000012256	tnni2a.3	0.628164776	3.762159614	4.672246791	3.241031276
ENSDART00000012357	sav1	0.272575283	0.33526037	0.280505115	-0.039530435
ENSDART00000012376	gabrr1	-0.444264456	-0.314539223	-0.677749333	-0.339326443
ENSDART00000012391	cabp1a	-0.440359676	-0.517676368	-0.117661538	0.040314239
ENSDART00000012450	dvl2	0.778001668	0.759654351	0.507462592	-0.16358587
ENSDART00000012478	mmadhc	-0.097599671	-0.214733382	-0.311326383	-0.186284094
ENSDART00000012546	ctbp2a	-0.410954451	-0.209595307	-0.078015176	-0.146761341
ENSDART00000012580		-0.21861763	0.154213857	-0.169850923	-1.246152902
ENSDART00000012673	gnb3a	-0.611271729	-0.360057677	-0.265096652	-0.439594606
ENSDART00000012677	OTUD7A	-0.320444813	-0.33972382	-0.130240255	-0.027710623
ENSDART00000012686	dnase1l4.1	0.654985436	1.279054377	1.240763186	0.531962605
ENSDART00000012718	fabp11b	1.386159817	1.224465105	0.518278623	-0.419802831
ENSDART00000012791	sp8a	1.57251014	2.331511764	2.00847442	1.039704847
ENSDART00000012822	CR354540.1	-0.197702519	0.015464017	-0.410213127	-0.063874026
ENSDART00000012859	psma6b	0.220061705	0.322650996	0.197619136	0.013143764
ENSDART00000012862	isl2a	-0.663813396	0.011664777	0.746201858	1.074694305
ENSDART00000012938	phgdh	-0.315221747	-0.241109028	-0.448879252	-0.249449553
ENSDART00000012940	grm2b	-0.52717467	-0.642099924	-0.570224924	-0.520457538
ENSDART00000013003	tfap2b	-0.299930992	-0.578681033	-0.25971747	-0.279510711
ENSDART00000013066	ercc3	0.273698243	0.1312947	0.187090251	0.076904615
ENSDART00000013117	syt5b	-0.473395784	-0.439637374	-0.372750952	-0.393185839
ENSDART00000013228	cacna1aa	-0.425294008	-0.48842278	0.10428355	0.42170802
ENSDART00000013229	gnaq	0.258587746	0.466151888	0.597563156	0.37656513
ENSDART00000013263	ugdh	0.441443931	0.390023192	0.086037142	-0.108373731

ENSDART00000013311	grm6a	-0.718441456	-0.89272341	-0.019297194	0.441642652
ENSDART00000013360	ppp1r3cb	-0.357586146	-0.346668382	-0.405594995	-0.235559662
ENSDART00000013409	prmt3	0.583250552	0.486757337	0.420985526	-0.028812313
ENSDART00000013411	cahz	-0.282657507	-0.146895201	-0.382063619	-0.133810291
ENSDART00000013449	CHST13	0.129876823	0.161538207	-0.188391866	-0.557482268
ENSDART00000013575	bzw1a	0.288880361	0.296312188	0.255308329	0.039925909
ENSDART00000013588	klhl41b	0.067761781	2.964864958	3.322998807	2.346652378
ENSDART00000013605	zbtb20	0.021191929	0.070670156	0.462529396	0.345025042
ENSDART00000013690	rplp2l	0.526551673	0.441805947	0.587852857	0.20929518
ENSDART00000013781	mcm6	1.053827708	1.005302691	0.681665045	0.184026433
ENSDART00000013785	insig1	-0.148803263	0.843708665	1.082868772	0.366540247
ENSDART00000013797	asb8	-0.250585187	-0.305587852	-0.166685061	-0.071337431
ENSDART00000013835	bloc1s1	0.447187715	0.435712256	0.356793883	0.203995374
ENSDART00000013839	tmbim4	-0.093382601	-0.138429676	-0.303111951	-0.191824583
ENSDART00000013961	mycla	0.091841991	0.503579515	0.855574629	0.294011787
ENSDART00000014021	slc25a39	-0.175830036	-0.233948732	-0.630252381	-0.299226836
ENSDART00000014031	dpf2	0.062076591	-0.192206303	-0.356876024	-0.045146421
ENSDART00000014036	optn	-0.037971093	-0.094029875	-0.163689177	-0.30174806
ENSDART00000014049	wdr36	0.429321608	0.423447996	0.351992847	0.181701695
ENSDART00000014058	zgc:100829	0.836226257	1.366715788	1.414785917	0.344237354
ENSDART00000014095	rap2c	0.031678102	0.116919511	0.30035611	0.141485249
ENSDART00000014098	ggctb	-0.310700478	-0.20595478	-0.456981273	-1.259991115
ENSDART00000014168	zfp36l1b	-0.068733676	-0.015026144	-0.139788147	-0.387873136
ENSDART00000014183	colgalt2	-0.33250641	-0.46357994	-0.396923695	-0.603253205
ENSDART00000014207	myl1	0.550709909	1.927654149	2.148710994	1.508189135
ENSDART00000014274	glcea	-0.30114601	-0.147144768	-0.347048688	-0.226291229
ENSDART00000014306	mpp5a	-0.106349761	-0.16109327	-0.308554572	-0.109175977
ENSDART00000014568	urod	0.384025692	0.230369566	0.194017408	-0.024966445
ENSDART00000014632	katnb1	-0.1660519	-0.366507385	-0.386560357	-0.02476169
ENSDART00000014668	pcsk1	-0.444651661	-0.49391188	-0.327964124	-0.406864512
ENSDART00000014726	tp53i11b	-0.378677803	-0.267454179	-0.193372925	-0.148965425
ENSDART00000014729	arpc1a	0.133812581	0.244815919	0.290121908	0.113999438
ENSDART00000014806	npas2	-0.341343772	0.009000027	0.555800516	0.156695025
ENSDART00000014843	bdnf	-0.572929745	-0.434797241	-0.517838864	-0.969054184
ENSDART00000014871	akr7a3	0.445881137	0.133128629	0.063605049	0.058812872
ENSDART00000014877	robo2	-0.381239115	-0.949448522	0.586346925	0.554614257
ENSDART00000014897	srgap1b	-0.562468132	-0.53652608	-0.21984937	0.24230424
ENSDART00000014922	arhgap22	0.337853511	0.496203573	0.771443869	0.664153347
ENSDART00000014983	zgc:153867	0.36977967	0.600215172	0.463408621	0.079627153
ENSDART00000015034	blvrb	0.700108065	0.61517331	0.539502173	-0.031641171
ENSDART00000015040	hrasb	-0.035400047	0.468215746	0.71708775	0.509094476
ENSDART00000015081	COX5B (1 of many)	-0.161517675	-0.197504434	-0.340992957	-0.152202035
ENSDART00000015092	col1a1b	0.334762626	1.456833944	2.512390696	1.131086568
ENSDART00000015095	uts1	-0.304915972	-0.432455801	-0.651436618	-0.693973757
ENSDART00000015103	hps3	0.398719216	0.34180849	0.31802597	0.20234989
ENSDART00000015193	chmp4bb	0.349511966	0.500219693	0.425249872	0.076172611
ENSDART00000015279	rtn4rl1a	-0.620130019	-0.765846328	-0.205912996	0.07424375
ENSDART00000015286	ankrd13b	-0.294042347	0.149598935	0.484140903	0.605167909
ENSDART00000015333	gbx2	-0.202064704	-0.378779754	-0.261598975	-0.176372699
ENSDART00000015374	cyb5r1	0.481284363	0.621601719	0.549959374	0.29311095
ENSDART00000015401	ercc6l	1.183315334	0.605525038	0.299117061	-0.215859916

ENSDART00000015418	irf2bpl	-0.517127602	-0.432851187	-0.112506589	-0.180160421
ENSDART00000015628	klhl24b	0.257620397	0.582600724	0.358856753	0.009507758
ENSDART00000015629	stxbp1a	-0.407999477	-0.254916105	-0.074938098	0.048338374
ENSDART00000015632	nkain1	0.221220236	0.815235139	1.176280245	0.965286214
ENSDART00000015710	snrkb	-0.234771599	-0.444259361	-0.383002363	-0.24868656
ENSDART00000015732	mylz3	0.011883193	1.89110665	2.420669252	1.523105093
ENSDART00000015755	rasl11b	-0.21929532	-0.630137444	-0.720127948	-0.404206999
ENSDART00000015777	abce1	0.253644034	0.246716072	0.263123796	0.067670414
ENSDART00000015827	tnr	-0.348895518	-0.471355706	-0.275641147	-0.100175719
ENSDART00000015841	gstt1b	0.844147349	0.559916631	0.300223231	-0.404935077
ENSDART00000015951	bsg	-0.039046362	-0.213267965	-0.489263219	-0.308938953
ENSDART00000015956	efna1b	-0.412330789	-0.211680079	-0.214205067	-0.214663513
ENSDART00000015979	farsb	0.087018819	0.265294461	0.405710239	0.211888538
ENSDART00000016053	rnf144aa	-0.119412737	-0.323472384	-0.040822296	0.138732642
ENSDART00000016057	ctnnal1	0.110311248	0.013228232	-0.224558056	-0.622388694
ENSDART00000016099	CASKIN2	-0.196661177	-0.318487444	-0.498724654	-0.242993173
ENSDART00000016112	capns1b	0.258538261	0.719472725	0.61845775	0.248329876
ENSDART00000016135	nfe2l3	0.030439041	0.342226134	0.430712572	0.086440986
ENSDART00000016143	SPIN4 (1 of many)	-0.27419945	-0.33038212	-0.171096922	-0.003336972
ENSDART00000016181	ndrg3a	-0.359622862	-0.274997666	-0.226177976	-0.143111599
ENSDART00000016283	psmd11b	0.064194443	0.317482468	0.345168865	0.113362384
ENSDART00000016303	irx2a	-0.381386746	0.064410059	0.752093904	0.989751717
ENSDART00000016350	pgam1b	-0.297181524	-0.257905953	0.060817132	0.142368471
ENSDART00000016360	si:ch73-199e17.1	2.690029092	3.363503415	3.69470671	3.529091333
ENSDART00000016370	dirc2	0.134905484	0.442850685	0.565287267	0.464631841
ENSDART00000016464	dcps	0.045608002	0.377363095	-0.076988765	0.017970252
ENSDART00000016535	kcns3a	-1.299949965	-1.227726419	-0.464780559	0.150117362
ENSDART00000016591	fgf6a	0.502410209	1.375431955	1.297903392	1.026892284
ENSDART00000016597	nfkbiab	0.207474067	0.316720279	0.269395099	0.411969915
ENSDART00000016602	cdh23	-0.199390412	-0.313772685	-0.367682037	-0.223939401
ENSDART00000016628	fam129bb	0.861865647	1.020541987	0.89470832	0.182764632
ENSDART00000016710	scrn3	-0.247629441	-0.171036354	0.44401532	-0.052873668
ENSDART00000016791	eif3c	-0.057805139	-0.126818292	-0.360850099	0.005995136
ENSDART00000016803	grpel1	-0.106418203	-0.12256904	-0.467582595	-0.298130125
ENSDART00000016814	fmnl2a	0.496433192	0.213976057	-0.143298216	-0.634969289
ENSDART00000016860	ppp2r1bb	0.018499492	0.284521267	0.321334616	0.193996267
ENSDART00000016864	slc35f6	0.392001078	0.280281029	0.187768856	0.16995993
ENSDART00000016890	eif6	0.391815405	0.223439355	0.155230229	0.056706473
ENSDART00000016916	gria4b	-0.27171917	-0.41554348	-0.145968175	0.021630803
ENSDART00000016946	glud1a	0.489439701	0.519599049	0.659106358	0.391317016
ENSDART00000016983	spon1a	-0.566333419	-0.54864414	-0.425205045	-0.214533594
ENSDART00000017148	gclc	0.213009245	0.302558434	0.032586299	-0.083839473
ENSDART00000017153	hps4	0.574215829	0.524013089	0.377950963	0.050651634
ENSDART00000017176	dkc1	0.45463167	0.261033913	0.276931433	0.02149911
ENSDART00000017202	kcnk1b	-0.464281499	-0.11026216	0.124659295	0.114555904
ENSDART00000017229	ncam1a	-0.359842421	-0.143522661	0.045009487	0.122759016
ENSDART00000017230	snrpc	0.173923367	0.335809723	0.464722714	0.188235196
ENSDART00000017259	fgf13a	-0.403933655	-0.684393894	-0.638256176	-0.336496098
ENSDART00000017292	stxbp5l	-0.151460711	-0.273207638	-0.026036945	0.086181343
ENSDART00000017299	tdg.1	0.102853523	0.194463285	0.348171907	0.196594799
ENSDART00000017309	ca16b	-0.395585068	-0.492018229	-0.260071918	-0.046674115

ENSDART00000017359	sfpq	0.205055054	0.247501694	0.177806588	0.262507487
ENSDART00000017413	zmynd10	1.112930172	1.692414467	1.985637202	1.515304432
ENSDART00000017422	tbc1d17	0.129347814	0.49736446	0.343125168	-0.01149421
ENSDART00000017424	ptmaa	-0.012891551	-0.178423729	-0.469624362	-0.475147609
ENSDART00000017485	sf3b6	-0.19155437	-0.273140548	-0.376775786	-0.432460535
ENSDART00000017551	slc6a1b	-0.387372781	-0.441576322	-0.34448646	-0.23699282
ENSDART00000017593	tmem237a	0.016738535	-0.334661164	-0.543525535	-0.214177149
ENSDART00000017599	rem1	-0.151492945	-0.383680013	-0.100323724	-0.098680467
ENSDART00000017619	impdh1a	-0.178621661	-0.394468991	-0.26888728	-0.032885741
ENSDART00000017646	atp6v0a1a	1.074304865	0.491570366	0.543801777	0.26961956
ENSDART00000017679	ppp2r2ca	-0.437371106	-0.275733569	0.22042039	0.361372826
ENSDART00000017695	foxd3	1.678381818	3.49818723	3.736655174	1.826274371
ENSDART00000017763	CABZ01071180.1	-0.429470778	-0.866508186	-0.658991937	-0.375481088
ENSDART00000017774	cacng5a	-0.685385238	-0.470575642	-0.348446742	-0.159039188
ENSDART00000017829	HELZ2 (1 of many)	0.30258536	0.160661865	1.299699291	-0.234524229
ENSDART00000018047	zgc:112294	-0.129085452	-0.359907528	-0.863079854	-0.32905376
ENSDART00000018054	trh	-0.369432982	-0.400024601	-0.784012545	-0.865751572
ENSDART00000018117	ppp1r14aa	-0.10674343	-0.179038105	-0.425069279	-0.232436913
ENSDART00000018150	neurod6b	0.484226112	1.695956927	1.935318885	1.555476315
ENSDART00000018155	adss	-0.479311511	-0.372005861	-0.294412527	-0.197085254
ENSDART00000018159	FOXK2 (1 of many)	0.025048904	0.121059845	0.321917677	0.256498575
ENSDART00000018163	irf2bp1	-0.194489303	-0.25009473	-0.19907539	-0.124082211
ENSDART00000018228	gsk3b	-0.075471253	0.063596815	0.240256058	0.249724511
ENSDART00000018261	akr1b1	1.00051996	0.934286328	0.670184322	0.161867768
ENSDART00000018304	mcm3	0.755786584	0.39595981	0.332438259	0.197655199
ENSDART00000018347	cab39l1	-0.259625217	-0.162823278	-0.312896931	-0.294075261
ENSDART00000018351	zgc:65851	-1.669295979	-1.346342078	-0.126748353	0.319422369
ENSDART00000018408	anxa13l	0.767695981	1.876785961	1.969202275	1.362090979
ENSDART00000018461	vmp1	0.430900752	0.323245721	0.184562524	0.049764543
ENSDART00000018475	snrpd3	0.421865611	0.381193487	0.380446926	0.20019783
ENSDART00000018498	helz2	-0.063748361	-0.192163226	1.151139617	0.013207133
ENSDART00000018501	opn4.1	-0.494086134	-0.180456491	-0.369290026	-0.100666897
ENSDART00000018523	ahcy	0.45208938	0.606696476	0.316876579	-0.072835379
ENSDART00000018528		-0.854763044	-1.028413156	-0.142975543	-0.344918955
ENSDART00000018603	tbx4	-0.302508569	-0.188111077	-0.131947864	0.000377602
ENSDART00000018625	napab	0.495528529	0.395594582	0.072464634	-0.31666363
ENSDART00000018654	rnd1b	-0.711104866	-0.668463449	-0.311306501	-0.458881777
ENSDART00000018676	cyp3c1	0.69067592	0.432985427	-0.038151099	-0.472952311
ENSDART00000018685	syt9a	0.383050894	0.997731574	0.678563906	0.37205351
ENSDART00000018686	rrp15	0.358996202	0.261032346	0.197604486	-0.018179208
ENSDART00000018735	dnaja2l	-0.20745831	0.012628814	0.350147509	0.311991137
ENSDART00000018743	phf20a	0.312029532	0.419745206	0.325238046	0.318832686
ENSDART00000018792	spag7	-0.393898515	-0.535366384	-0.519915196	-0.305218132
ENSDART00000018886	ghra	-0.12746236	-0.080311259	-0.403757478	-0.551820215
ENSDART00000018945	meis3	-0.263611366	0.389783427	1.01804147	0.898863275
ENSDART00000018972	zgc:92818	0.012004445	-0.040419062	-0.784852086	-0.38349701
ENSDART00000019003	psmd10	0.510870261	0.455922769	0.143258659	0.040691699
ENSDART00000019029	atp6v1h	-0.249435289	-0.255917009	-0.243059404	-0.047539915
ENSDART00000019045	ebp	-0.186359255	0.947675675	1.363611199	0.55228711
ENSDART00000019053	faima	-0.147906168	-0.161120397	-0.359996788	-0.164587924
ENSDART00000019140	rorab	-0.499015892	-0.249117065	-0.271786747	0.004902877

ENSDART00000019149	rpl7	0.429749031	0.41708455	0.235093103	-0.015027688
ENSDART00000019165	apaf1	0.51528421	0.965000853	0.888709667	0.68935409
ENSDART00000019199	rab39ba	-0.125963686	0.3041991	0.44382958	0.206562916
ENSDART00000019294	si:dkeyp-75b4.9	-0.789027106	-0.841731007	-0.221050872	0.359721563
ENSDART00000019330	ech1	0.344696401	0.425292318	-0.031478205	-0.016576624
ENSDART00000019521	dip2ba	0.352814519	0.501689217	0.539560646	0.272762208
ENSDART00000019573	zgc:65894	-0.648921378	-0.373996254	0.357740851	0.502243837
ENSDART00000019595		-2.247733873	-0.556417246	-3.574262962	-0.309310232
ENSDART00000019617	rsad2	-0.056061565	-0.489318095	2.739834278	-0.378754222
ENSDART00000019647	psmc2	0.088369453	0.252149794	0.191509771	0.022895546
ENSDART00000019658	nacad	-0.254606168	0.44010123	0.744298293	0.29398429
ENSDART00000019698	anxa5b	1.162500386	1.67744918	1.46667303	0.432101083
ENSDART00000019706	phc2b	-0.309131033	0.04672236	0.268644424	0.076203283
ENSDART00000019748	lin7a	-0.334365216	-0.254636287	-0.400398823	-0.216397551
ENSDART00000019750	wdr5	0.151689533	0.300718903	0.396766724	0.199844693
ENSDART00000019766	tgfb3	-0.14127581	-0.182290633	-0.363811528	-0.271524635
ENSDART00000019770	gpm6ba	-0.26452505	-0.240754685	-0.112562472	0.004364713
ENSDART00000019818	ric8b	0.219425238	0.304273846	0.411142498	0.146708414
ENSDART00000019905	fndc4b	-0.870561937	-0.746602431	-0.973680622	-0.402474596
ENSDART00000019907	unc119.1	-0.258282731	-0.126927399	-0.208777989	-0.147908262
ENSDART00000019925	GNB4	0.24039241	0.43881347	0.966289057	1.074168682
ENSDART00000019936	prkacab	-0.083728046	-0.043118374	0.244894467	0.164419526
ENSDART00000019937	gadd45ga	0.98351774	1.304995817	0.165178221	0.462881948
ENSDART00000019949	ndrg2	-0.366275522	-0.294111898	-0.252300979	-0.169375948
ENSDART00000020017	aldh3b1	0.019655473	0.632122683	0.647018435	0.316141972
ENSDART00000020048	gsna	0.451913636	0.79824491	0.782008443	0.357949724
ENSDART00000020054	opcml	-0.412711216	-0.540582472	-0.347997034	-0.057254512
ENSDART00000020084	hsp90ab1	0.266636397	0.434039186	0.537534154	0.384889667
ENSDART00000020096	fgf13b	-3.880462854	-2.396384616	-1.469971299	0.217849124
ENSDART00000020122	ywhah	-0.243048068	-0.041853394	0.44002204	0.422069749
ENSDART00000020153	adck3	-0.178069281	-0.150101974	-0.272732357	-0.02457601
ENSDART00000020167	slc16a9a	0.895506163	0.735954298	0.591907512	-0.466816888
ENSDART00000020168	kctd5a	0.075119561	0.1276866	0.301730152	0.250326391
ENSDART00000020174	dynll2b	-0.376289244	-0.003912141	-0.039237308	0.00732876
ENSDART00000020183	fam102bb	-0.149766416	-0.311804442	-0.169646826	-0.06993891
ENSDART00000020249	dusp5	-0.842223428	-0.88819591	-1.001072274	-0.721604289
ENSDART00000020252	pdia6	0.374033372	0.009098395	0.091775115	0.023604955
ENSDART00000020256	lgsn	1.361702081	2.686172063	1.394569806	0.349584846
ENSDART00000020296	nadl1.2	-0.369828112	-0.124790776	0.28925914	0.362042383
ENSDART00000020311	rpl27	0.430086554	0.381494789	0.182914397	-0.034120883
ENSDART00000020342	sgsm3	-0.132410481	-0.068536613	0.176019735	0.283913195
ENSDART00000020497	snx13	-0.143691651	-0.273195754	-0.089112423	0.016117988
ENSDART00000020541	lipf	0.528006329	0.723139512	0.390796686	-0.03933789
ENSDART00000020569	creld1b	-0.396401062	-0.06913614	0.015512001	0.011512871
ENSDART00000020621	mapk4	-0.137443479	0.345820304	0.800085717	0.474998646
ENSDART00000020638	rcan1a	0.548382891	0.378475469	0.383543152	0.544764217
ENSDART00000020646	chrm1b	0.963286522	0.623965705	0.291783612	0.576118226
ENSDART00000020655	psma5	0.424960543	0.53028483	0.342202642	0.090736505
ENSDART00000020665	sgtb	-0.247959729	-0.080915193	-0.275270124	-0.210502305
ENSDART00000020741	aldoaa	-0.416493866	-0.444493146	-0.351916406	-0.154405211
ENSDART00000020810	sdcbp2	0.429533724	0.242026939	0.181097661	-0.126536592

ENSDART00000020824	pank1b	0.041844369	0.140368278	0.263424196	0.005383312
ENSDART00000020908	zc4h2	-0.133072632	0.229085423	0.337553441	0.264734015
ENSDART00000020970	pgm2	0.182379442	0.224260514	0.427220163	0.369397058
ENSDART00000020999	angptl1a	-0.447558553	-0.389827093	-0.516507166	-1.101603289
ENSDART00000021037	hspa4a	0.42074258	0.381012047	0.243421438	0.132816774
ENSDART00000021062	slc9a8	0.308335488	-0.627436336	-3.684031993	-1.491743311
ENSDART00000021069	rpl38	0.561397297	0.414839383	0.25425723	0.049186317
ENSDART00000021083	calm2a	-0.300457039	-0.171937069	0.044961812	0.17764614
ENSDART00000021092	snx27b	-0.196827884	-0.228935762	-0.254873921	-0.135355059
ENSDART00000021121	stx5al	-0.079551416	-0.239231712	-0.554538789	-0.564757113
ENSDART00000021168	rxrga	0.040179684	-0.194377683	-0.381512289	-0.377392107
ENSDART00000021213	cpne2	-0.275625296	-0.394159316	-0.352439432	-0.230978713
ENSDART00000021231	slmapb	-0.557559725	-0.496297491	-0.195575587	0.049672527
ENSDART00000021260	sept8b	-0.246039495	-0.120094514	-0.518439508	-0.250786208
ENSDART00000021299	nmd3	0.353895501	0.176055483	0.10015969	0.031379837
ENSDART00000021341	kif3ca	0.127548246	0.488322423	0.730937873	0.492487971
ENSDART00000021346	arl3l2	-0.339022452	-0.304754125	-0.490852638	-0.247921956
ENSDART00000021417	p2rx3a	0.048222852	-0.166951272	-1.130394212	-0.103958246
ENSDART00000021491	csnk1db	-0.098564588	-0.355768317	-0.198945325	-0.067100917
ENSDART00000021559	coro1b	0.006396016	-0.122229179	-0.278308819	-0.121710131
ENSDART00000021596	rxrbb	-0.183103481	-0.256930789	-0.18551154	-0.16195495
ENSDART00000021604	gins4	0.598069286	0.116671159	-0.218567942	-0.173827642
ENSDART00000021605	LRRC4C	-0.456880748	-0.587108705	-0.299621227	-0.131466817
ENSDART00000021609	gad2	-0.376538118	-0.375877531	-0.287892544	-0.231277899
ENSDART00000021666	rtca	0.450183742	0.82121323	0.721903733	0.20677388
ENSDART00000021693	ank2a	0.088061869	0.283890991	0.488712819	0.41975176
ENSDART00000021788	pbk	1.438031033	0.973567544	0.667760742	0.195406357
ENSDART00000021798	fabp11a	3.578286828	1.692122399	1.073895347	-0.266988673
ENSDART00000021950	mthfd1b	1.089156306	0.599589593	0.18588711	-0.096895618
ENSDART00000021976	nsa2	0.087749249	0.255476165	0.234966715	0.01893274
ENSDART00000022010	hivep2b	-0.404683257	-0.428225913	-0.085969125	0.061919909
ENSDART00000022042	scn8aa	-0.64882134	-0.631929626	0.169338069	0.591046475
ENSDART00000022044	dct	0.864997441	0.322109214	-0.10561397	-0.63566776
ENSDART00000022051	gins1	-0.049060202	-0.07395231	-0.409999594	-0.285702931
ENSDART00000022060	atf3	2.958540036	3.348918067	2.852908333	1.811501419
ENSDART00000022270	arhgap33	0.060975731	0.144058893	0.360327899	0.271763038
ENSDART00000022290	mdh1aa	-0.386096556	-0.387245145	-0.361974171	-0.162628891
ENSDART00000022307	atic	1.012801143	0.518350872	0.289570342	-0.11925608
ENSDART00000022356	ppp1r7	-0.289189989	-0.228515822	-0.279744824	-0.100278848
ENSDART00000022393	si:dkeyp-57f11.2	0.163453067	-0.122007101	-0.459561011	0.032532197
ENSDART00000022499	psmb3	0.223119041	0.273754026	0.150624654	-0.009410181
ENSDART00000022533	kcnj2a	-0.207812042	-0.209993339	-0.395014972	-1.14439113
ENSDART00000022549	atp1b3a	-0.438514495	-0.341784188	-0.389598734	-0.186751495
ENSDART00000022562	rhogb	0.97771224	0.454579503	0.287206454	-0.277565993
ENSDART00000022579	GABRG3	-0.524427233	-0.895243712	-0.602147004	-0.152848624
ENSDART00000022581	rab22a	-0.072513798	-0.08434934	-0.299709625	-0.230388715
ENSDART00000022586	lrrc40	0.466457292	0.391920358	0.288821604	-0.015697706
ENSDART00000022625	nrarpb	-0.295040721	-0.303522559	-0.284578601	-0.228026779
ENSDART00000022634	acp2	0.356430058	0.295051752	0.030540215	-0.026401831
ENSDART00000022646	cnot4b	0.11528648	0.170971297	0.271245163	0.121803983
ENSDART00000022660	si:ch211-195b15.7	-0.190059614	-0.249688476	-0.324813294	-0.157998073

ENSDART00000022663		-0.241007108	-3.679203505	-0.288618865	-1.771039097
ENSDART00000022688	tob1b	-0.393522131	-0.302261171	-0.612204875	-0.372656833
ENSDART00000022694	ehd3	-0.278899876	-0.241188601	-0.097281852	-0.017987416
ENSDART00000022729	unm_sa808	-0.546569433	-0.564171517	-0.267551607	0.046437921
ENSDART00000022765	riok1	0.429965951	0.477495547	0.301061387	0.021181676
ENSDART00000022768	ak5	-0.529999471	-0.599207091	-0.317508372	0.131326788
ENSDART00000022866	pisd	-0.555411298	-0.214837183	-0.086260422	-0.186182517
ENSDART00000022909	klhl18	-0.153412936	0.073357396	0.397749012	0.249762723
ENSDART00000022963	cdc14aa	-0.366168568	-0.36782201	-0.106140735	0.082726559
ENSDART00000022976	kctd16b	-0.982447595	-1.281976718	-0.566394426	0.110166911
ENSDART00000022998	ANO2 (1 of many)	0.005158406	-0.146342409	-0.548931715	-0.196350546
ENSDART00000023038	dacha	-0.511894205	-0.418991667	-0.004522839	0.112876213
ENSDART00000023054	atl3	0.317687991	0.347306029	0.121176286	-0.186993768
ENSDART00000023089	acadvl	0.315023187	0.311610409	0.17259027	-0.0260318
ENSDART00000023123	nup88	0.221684741	0.406336851	0.406160624	0.250704697
ENSDART00000023156	eef1a1l2	0.512554224	0.741741761	0.709552788	0.28897731
ENSDART00000023206	plk2b	-0.591714767	-0.133232909	-0.517936071	-0.57159861
ENSDART00000023210	trim13	-0.37704188	-0.363410115	-0.204332616	0.075535016
ENSDART00000023278	fads2	-0.280275959	0.609167416	1.126645574	0.470269511
ENSDART00000023463	uap1l1	0.706648736	0.512468414	0.225773419	-0.015907861
ENSDART00000023509	ska2	-0.036357965	-0.092147723	-0.294463498	-0.176013793
ENSDART00000023547	anxa11b	0.050301812	-0.087626764	-0.004152721	-0.398004466
ENSDART00000023550	hsp90aa1.2	-0.115909172	-0.134363979	-0.322775345	-0.097362577
ENSDART00000023562	CABZ01041610.1	-1.012987922	-1.132303657	-0.526387248	-0.210938966
ENSDART00000023588	guca1a	0.527717677	-0.52699324	-1.299472888	-0.244316084
ENSDART00000023613	her6	-0.335042933	-0.234567638	-0.250120727	-0.59712361
ENSDART00000023686	ankrd33ab	-0.23872945	-0.400254403	-1.062972805	-0.551724897
ENSDART00000023709	ptp4a2b	0.271575512	0.562448581	0.634906429	0.086969331
ENSDART00000023763	wdcp	-0.038191062	-0.371104976	-0.391285873	-0.034783387
ENSDART00000023779	vcp	0.230561184	0.246570266	0.325389872	0.137878127
ENSDART00000023806	zgc:110319	-0.195130618	-0.122813174	-0.337219496	-0.098833156
ENSDART00000023831	cry5	-0.290776024	0.507969364	-0.405324234	-0.228415974
ENSDART00000023833	eif2s3	0.172990624	0.349255612	0.311895175	0.045890851
ENSDART00000023926	eif3eb	-0.055777547	-0.145564801	-0.286794414	-0.128155341
ENSDART00000023944	lmnl3	-0.134674249	0.538751787	0.920098245	0.528719758
ENSDART00000023959	arntl1a	0.340904931	1.030627189	1.45255612	0.975945139
ENSDART00000024034	gpsm2	0.06632826	-0.389217225	-0.522197566	-0.062079955
ENSDART00000024082	psmb6	0.195043333	0.259353241	0.359595812	0.192042468
ENSDART00000024135	tubb2	-0.097026879	1.085064176	1.27895467	1.027950073
ENSDART00000024136	gngt2a	0.175894019	0.078473268	-0.788482627	-0.299650763
ENSDART00000024194	kif11	1.409119378	1.269759488	0.810392576	0.401186663
ENSDART00000024208	nutf2l	0.951686549	0.855710394	0.537014601	-0.220899011
ENSDART00000024287	zgc:165604	-0.009089907	-0.228105246	-0.581619742	-0.405549266
ENSDART00000024296	egln1b	-0.145407238	-0.296649941	-0.383343901	-0.196291651
ENSDART00000024304	per3	-0.478794008	-0.23341732	-0.352073188	-0.024889246
ENSDART00000024309	rb1	0.736743325	0.968558289	0.720111001	0.238176467
ENSDART00000024313	rnf150b	-0.152451949	-0.254343985	-0.250476516	-0.120482055
ENSDART00000024316	mcm5	1.292009744	0.913737369	0.774968175	0.275487022
ENSDART00000024320	ybx1	0.257630768	0.252218726	0.414084055	0.18414263
ENSDART00000024328	slc34a2a	-1.294382652	-0.588426598	-0.664087867	-1.114341851
ENSDART00000024331	glsb	0.029280722	-0.254060441	-0.66092512	-0.487127171

ENSDART00000024354	csad	0.826762252	0.889896753	0.709970739	0.637298928
ENSDART00000024415	epas1a	0.419060345	0.571698023	0.827681076	-0.059626191
ENSDART00000024528	emc7	-0.102957427	-0.142334415	-0.248955976	-0.189343286
ENSDART00000024615	rnpepl1	0.274953884	0.219791217	0.571525784	0.173823915
ENSDART00000024619	gorasp1a	-0.145027203	-0.174354089	-0.499227113	-0.186036756
ENSDART00000024662	plppr3a	1.050789931	1.738581828	1.609411361	1.082826483
ENSDART00000024720	si:ch211-282j22.3	0.265772408	0.220333711	0.187153701	0.074808124
ENSDART00000024778	robo3	-0.327155095	-0.251732012	-0.124129181	0.058627004
ENSDART00000024832	stat5a	-0.212236095	-0.408877789	-0.375060924	-0.185569558
ENSDART00000024858	chchd10	-0.331794878	-0.431239071	-0.4916218	-0.229989739
ENSDART00000024872	creb3l3l	0.316205692	0.628018553	0.525420097	0.254183225
ENSDART00000025031	pou4f1	-0.37458842	-0.169318799	0.774588614	1.160187758
ENSDART00000025046	ppp1caa	0.04938914	-0.175273742	-0.396680839	-0.049267892
ENSDART00000025096	larp1b	0.593217694	0.576647108	0.472990072	0.196836578
ENSDART00000025198	mettl21a	-0.380455489	-0.566803653	-0.570464602	-0.564292758
ENSDART00000025229	adi1	0.582007509	0.274560712	-0.200617197	-0.21352575
ENSDART00000025256	igfbp2b	-0.742463234	-0.107964006	0.616733017	0.74050304
ENSDART00000025326	csnk1da	-0.048665312	-0.227600133	-0.273972646	-0.14458728
ENSDART00000025385	cers2a	0.59368354	0.657003513	0.583941604	0.192392368
ENSDART00000025414	slc2a1a	0.059681786	-0.155207235	-0.469818878	-0.137194664
ENSDART00000025466	slc18a2	-0.576881609	-0.281211576	-0.258781682	-0.379077041
ENSDART00000025487	icmt	-0.205683018	-0.185657781	-0.262148423	-0.151909752
ENSDART00000025494	hprt1l	-0.364550689	-0.385937863	-0.083973479	-0.129534044
ENSDART00000025496	rras	0.483455604	0.817924325	0.502791397	-0.193021858
ENSDART00000025501	snap23.1	0.353906863	0.265231797	-0.028292671	-0.317654401
ENSDART00000025535	sept5a	0.521581645	1.371610673	0.945407012	0.566204111
ENSDART00000025550	top1mt	0.816108961	1.133963099	0.883225094	0.407533529
ENSDART00000025573	CABZ01071723.1	-0.48632791	-0.607491421	-0.117657015	0.102596694
ENSDART00000025583	fgf8a	-0.058331217	-0.819626176	0.05604093	0.237151994
ENSDART00000025620	ppiaa	0.369681961	0.563046067	0.555154111	0.174901331
ENSDART00000025698	zgc:153311	-0.134647599	-0.156342149	-0.388760407	-1.773822225
ENSDART00000025782	nup93	0.17632244	0.241668195	0.293793434	0.152368608
ENSDART00000025852	tnni2b.1	-0.577234038	4.833234821	5.215670785	3.899605393
ENSDART00000025860	si:dkey-247m21.3	0.550902269	0.531374836	-0.221903085	-0.681264404
ENSDART00000025877	cldn12	-0.148592994	-0.140436414	-0.371702065	-0.263635262
ENSDART00000025912	si:dkey-32n7.4	0.676729111	0.251567535	0.290364244	0.056388056
ENSDART00000025962	gyg1a	-0.132586486	-0.231554909	-0.320101499	-0.261622046
ENSDART00000025997	dip2cb	-0.352423277	-0.192868324	0.083423296	0.161945092
ENSDART00000026085	ptges	-0.133597623	-0.186036004	-0.449634161	-0.455296325
ENSDART00000026145	AMOTL1	1.0586494	0.570711762	0.741896115	0.335943589
ENSDART00000026152	asap2a	-0.416411014	-0.084267369	-0.025927141	0.001189582
ENSDART00000026174	dgkh	-0.6307495	-1.007774241	-0.588261808	-0.107017931
ENSDART00000026178	kif4	0.630775369	0.400037801	0.195646982	0.069714171
ENSDART00000026180	fabp7a	0.745585804	2.003621538	1.857780253	1.020257385
ENSDART00000026303	rasd1	-0.723056439	-0.693453912	-0.473472236	-0.755311511
ENSDART00000026316	sema3gb	-0.148485986	-0.183297493	-0.3621349	-0.465943338
ENSDART00000026339	gtpbp4	0.113641949	0.327640711	0.222548232	0.071359586
ENSDART00000026378	slc25a6	-0.475274891	-0.471356749	-0.229323143	0.038491163
ENSDART00000026401	TMEM178B (1 of many)	-0.387053719	-0.214500403	-0.049460199	0.011667684
ENSDART00000026409	cct4	0.459151422	0.538338734	0.621039126	0.313903974
ENSDART00000026492	flncb	1.767414623	1.156524725	0.931280558	0.481368808

ENSDART00000026692	ubtd1a	-0.10869502	-0.342281946	-0.985605367	-0.704345293
ENSDART00000026765	slc18a3a	-0.342520426	-0.454539851	-0.359489571	-0.310297658
ENSDART00000026766	aldocb	-0.483629021	-0.540701781	-0.406696036	-0.135568984
ENSDART00000026800	kifap3b	0.203180282	0.588115089	0.640508934	0.450843691
ENSDART00000026814	ptp4a1	0.419787699	0.591512581	0.636748721	0.300535035
ENSDART00000026865	l3mbtl1a	-0.339631378	-0.338161449	-0.153879513	-0.055902641
ENSDART00000026924	dnah7	-0.347974455	-0.178245432	-0.682598609	-0.174885691
ENSDART00000026992	sox4a	0.168735758	0.553498244	0.871364123	0.630648218
ENSDART00000027050	cnga3b	-0.139313288	-0.277577971	-1.175814494	-0.697938349
ENSDART00000027115	nob1	-0.149963351	-0.185404709	-0.224057031	-0.30485959
ENSDART00000027158	psmd3	0.079773681	0.202742016	0.46801517	0.212038089
ENSDART00000027274	efna3a	-0.422282495	-0.289576322	-0.118198025	0.092530837
ENSDART00000027345	tmem59l	-0.559867163	-0.384910477	-0.005343137	0.202377241
ENSDART00000027379	bicral	0.180958951	0.201052129	0.429766407	0.252075745
ENSDART00000027393	ckmt1	0.722688207	0.598583347	0.435615456	0.204934783
ENSDART00000027398	kcna2a	-1.558634612	-1.413069345	-0.244650937	0.296687437
ENSDART00000027417	zgc:171704	0.227240571	-0.665719796	-1.161293822	-1.438167938
ENSDART00000027454	si:ch211-207i1.2	-0.470697042	-0.923472632	-1.21436549	-0.514660002
ENSDART00000027463	hmx4	-0.340009377	-0.236809718	-0.106768059	0.00156649
ENSDART00000027465	cacna2d4b	-0.370016882	-0.141565087	-0.247577188	0.068079685
ENSDART00000027466	fam63b	-0.160774327	0.127626681	0.377768257	0.305765252
ENSDART00000027532	mapkapk2a	-0.086338753	-0.192582349	-0.252851985	-0.027631364
ENSDART00000027598	tpm3	0.692886998	2.316316343	2.715886041	1.570432549
ENSDART00000027616	eif4g2a	0.333990776	0.435258045	0.644700731	0.350291836
ENSDART00000027689	amph	-0.260662658	-0.30969295	-0.237665092	0.01178982
ENSDART00000027718	fxr2	0.070260526	0.251047986	0.370057736	0.114198992
ENSDART00000027758	rtn1b	-0.626628578	0.207141964	0.617559397	0.433726227
ENSDART00000027957	hmgcl	-0.03513095	-0.160364915	-0.261992968	-0.251204537
ENSDART00000028003	ankrd22	0.462762131	0.494960479	0.738379791	0.088098985
ENSDART00000028033	emc8	-0.03555204	-0.206564635	-0.268269465	-0.211006035
ENSDART00000028048	necap1	-0.117195999	-0.341344035	-0.478671947	-0.252467934
ENSDART00000028090	eif2ak1	0.218229206	0.400243646	0.398265404	0.179590234
ENSDART00000028108	ddc	-0.192852254	-0.317464474	-0.303298772	0.040886808
ENSDART00000028219	pvalb4	-1.549055704	2.87360786	4.516864647	3.395003162
ENSDART00000028225	mao	-0.257909806	-0.164169194	-0.255680521	-0.167259868
ENSDART00000028285	pgbd5	-0.471148265	-0.169774685	0.263561856	0.22277577
ENSDART00000028338	scamp5a	-0.358452184	-0.354788931	-0.092970627	0.058203665
ENSDART00000028390	fgf12a	-0.792701948	-0.713373555	-0.153423419	0.185286633
ENSDART00000028417	lrit2	-0.281592969	-0.383251603	-0.165960328	0.039848719
ENSDART00000028500	nxn	-0.356492001	-0.304319321	-0.337794036	-0.002781587
ENSDART00000028607	chd6	0.00488027	-0.079639497	0.533277243	0.378096362
ENSDART00000028787	ahr1b	-0.27962376	-0.18218298	-0.129040126	0.07690852
ENSDART00000028883	gna13b	0.442397382	0.38652983	0.274274635	-0.034947864
ENSDART00000028895	negr1	-0.362135851	-0.256191375	-0.15121136	-0.084875523
ENSDART00000028946	tpd52l2a	0.038391394	-0.134825728	-0.275007322	-0.131471646
ENSDART00000028960	ndufa2	-0.147139307	-0.190704061	-0.363806149	-0.139491855
ENSDART00000028997	myo9ab	0.179745305	0.101321956	0.280102899	0.331620625
ENSDART00000029121	usp5	0.186533139	0.264124698	0.696851044	0.548402162
ENSDART00000029133	snu13b	0.760760317	0.56984098	0.429270049	-0.029914173
ENSDART00000029380	bnip4	0.50246171	0.517948631	-0.062532971	-0.337429982
ENSDART00000029387	ppan	0.365110957	0.289310564	0.234072786	0.008675203

ENSDART00000029457	sh2d3ca	-0.605491696	-0.301886794	0.051143961	0.055429229
ENSDART00000029459	gipr	-0.120368109	-0.334116608	-0.559985722	-0.423861096
ENSDART00000029492	cmtm7	0.705299486	0.845221281	0.768474344	-0.28587683
ENSDART00000029528	mospd2	0.309594516	0.158328804	0.007644114	-0.042795417
ENSDART00000029646	rplp1	0.363694976	0.319088557	0.167660064	0.013197371
ENSDART00000029703	kcnh1a	-0.531746722	-1.366001544	-0.565478925	0.09193536
ENSDART00000029774	tmem55bb	-0.03621571	-0.196356812	-0.337053328	-0.094153869
ENSDART00000029843	vezf1a	0.660507751	0.776194217	0.884683465	0.428803142
ENSDART00000029946	ube2b	-0.262228145	-0.117966704	-0.377845713	-0.176574464
ENSDART00000029981	ppp3cb	-0.453839737	-0.543662557	-0.385030051	0.007551117
ENSDART00000030125	znhit3	0.760210037	0.691966223	0.487369205	0.388320697
ENSDART00000030205	bnip3lb	0.087436849	0.295010863	0.335325724	0.120618456
ENSDART00000030211	gmfb	-0.040702268	0.286017295	0.157496122	0.031502906
ENSDART00000030213	mapk1	0.367982033	0.335025068	0.254642258	-0.112923758
ENSDART00000030409	asap1b	-0.387126717	-0.32480613	-0.179905202	-0.016544261
ENSDART00000030509	glra4a	-0.54562871	-0.440353116	-0.570533638	-0.182903567
ENSDART00000030579	crhbp	0.109353477	-0.053663997	-0.382440888	-0.742296052
ENSDART00000030691	clic4	0.290171682	0.490608957	0.279069846	0.046035564
ENSDART00000030773	foxo3a	-0.522674186	-0.346284342	-0.025883071	0.086783781
ENSDART00000030794	tmem169a	0.073168182	0.288108809	0.520379557	0.751344779
ENSDART00000030811	cables2b	-0.198257533	-0.480718843	-0.449759807	-0.060172476
ENSDART00000030885	uckl1a	0.183203205	-0.212616494	-0.705475026	-0.013040484
ENSDART00000030887	slc45a2	0.835000405	0.559834035	-0.13177777	-0.758141187
ENSDART00000030890	hmox1a	2.623035746	1.043671484	1.096486903	0.061370069
ENSDART00000030920	gid8a	-0.116648875	-0.277182294	-0.355798783	-0.233597587
ENSDART00000030995	umps	0.416416432	0.256033809	0.30736318	-0.006312473
ENSDART00000031047	cd63	1.025048213	0.521327717	0.067995805	-0.605090068
ENSDART00000031091	vsnl1a	-0.845008079	-0.77079299	-0.390375819	0.072718281
ENSDART00000031121	VDAC3 (1 of many)	-0.281245453	-0.237325849	-0.158924417	0.113945733
ENSDART00000031139	slc24a4b	-0.756345109	-1.188291659	-0.474432561	-0.06484409
ENSDART00000031165	enoph1	0.127295774	0.248943534	0.342353563	0.2531466
ENSDART00000031167	tfap2d	-0.967562253	-0.629795841	0.638492839	1.047009841
ENSDART00000031234	stxbp2	0.406820773	0.260992395	0.143612276	-0.234256086
ENSDART00000031265	rtn4r	-0.753039937	-0.836146377	-0.430012914	-0.160533624
ENSDART00000031390	caskin1	-0.735732711	-0.907721451	-0.315045095	-0.088633322
ENSDART00000031425	zgc:55582	-0.13184915	-0.205452993	-0.25748443	-0.094044364
ENSDART00000031426	skilb	-0.234186907	-0.553407151	-0.565936684	-0.420869627
ENSDART00000031470	pafah1b1b	-0.06625793	0.19164974	0.422888398	0.388967525
ENSDART00000031498	ccna2	1.569664461	1.378683751	0.895010724	0.581055828
ENSDART00000031546	chrna6	-0.487671995	-0.420962215	0.210734418	0.46609085
ENSDART00000031638	slc48a1a	-0.121591299	-0.156663294	-0.340136476	-0.182759405
ENSDART00000031650	hsp70l	-0.846526969	-1.057845455	-1.357523269	-1.215078404
ENSDART00000031727	vamp8	0.086516161	-0.020853386	-0.34297459	-0.531441391
ENSDART00000031937	diras1a	-0.474540918	-0.418703997	-0.179232074	-0.012563291
ENSDART00000032161	galnt14	-0.529482639	-0.362873656	-0.264119496	-0.01842007
ENSDART00000032212	fynrk	-0.162448847	-0.18539887	-0.276020606	-0.108651002
ENSDART00000032275	atp6v1c1a	-0.172326217	-0.24874129	-0.293288363	-0.174129603
ENSDART00000032290	esyt1a	0.414133861	0.186544426	0.071532791	-0.03493067
ENSDART00000032322	abcg2c	0.455571157	0.699504596	0.641510999	0.335341634
ENSDART00000032324	hddc3	0.491004501	0.803369277	0.623276564	0.474326204
ENSDART00000032331	gmppab	0.298836739	0.452114759	0.362408461	-0.028707454

ENSDART00000032392	dhdhl	0.557297889	0.381488116	0.091728361	-0.116661732
ENSDART00000032393	ginm1	-0.054454012	-0.092963972	-0.327748186	-0.224389181
ENSDART00000032459	aqp1a.1	-0.275584647	-0.402261221	-0.531838868	-0.410567004
ENSDART00000032498	tspan36	1.455763612	0.805559086	0.192569945	-0.715366149
ENSDART00000032502	nebl	-0.405575453	-0.348273521	-0.638746976	-0.69843843
ENSDART00000032540	usp14	0.236991779	0.380670741	0.291281165	0.061189525
ENSDART00000032547	lect2l	1.708944579	1.070031122	0.348802096	-0.787093126
ENSDART00000032603	tspo	0.47255795	0.736846252	0.52533392	0.11686825
ENSDART00000032695	asic4a	-0.405928595	-0.689262684	-0.527033258	-0.378352566
ENSDART00000032821	cyth1b	0.161176597	0.057433607	-0.079026086	-0.299717291
ENSDART00000032844	plekha6	-0.171611787	-0.149520127	-0.254818235	0.001528605
ENSDART00000032857	mapk11	0.353170314	0.603154564	0.6388701	0.458511735
ENSDART00000032899	clpxa	-0.125001004	-0.138480195	-0.243789891	-0.138582094
ENSDART00000032963	apoob	-0.330795	-0.271281795	-0.230754946	0.035824011
ENSDART00000033053	dennd5b	-0.288493162	-0.362789161	-0.132763243	0.070545146
ENSDART00000033248	fam107b	-0.588148397	-0.439287754	-0.362004691	-0.311865235
ENSDART00000033316	vangl2	0.207091244	0.447705982	0.579933447	0.26125294
ENSDART00000033325	slc25a24	-0.429859875	-0.309149544	-0.52291133	-0.286046915
ENSDART00000033361	ttyh3b	-0.3186879	-0.037617104	0.269211477	0.394612295
ENSDART00000033362	gatad2b	-0.40244942	-0.286045422	-0.214483951	-0.084688348
ENSDART00000033386	ocstamp	2.954317372	2.548446344	2.458244526	1.328553982
ENSDART00000033479	si:ch211-129c21.1	1.539581327	2.000726167	1.978342783	1.084446492
ENSDART00000033494	klf6a	1.084550888	1.797692613	1.625562666	0.460444617
ENSDART00000033545		0.299034458	0.290865093	0.474990251	0.248453432
ENSDART00000033566	smad1	0.486585209	1.249927606	1.264727704	0.536891999
ENSDART00000033574	slc24a5	0.460212086	0.054768672	-0.306746704	-1.141417252
ENSDART00000033657	grm6b	-0.379593806	-0.511743403	-0.159835887	0.100793439
ENSDART00000033663	rps21	0.311890725	0.366197651	0.105076559	-0.038309008
ENSDART00000033713	arpc1b	0.908662023	0.412162066	0.131006833	-0.30826968
ENSDART00000033724	fabp3	0.043357927	0.829727862	0.980692721	0.446444515
ENSDART00000033746	gins2	1.156804675	0.565959042	-0.013280867	0.106301442
ENSDART00000033761	glb1	0.347409105	0.187375641	0.031649312	0.073918812
ENSDART00000033980	lims1	0.497849916	0.369061975	0.247283344	-0.174205881
ENSDART00000034004	faf1	0.052082526	0.093691656	0.281166911	0.24525998
ENSDART00000034216	dync1h1	0.15631552	0.099035755	0.755817679	0.554910929
ENSDART00000034248	rab32a	0.364170748	0.270180665	-0.161567701	-0.659765413
ENSDART00000034313	gas2a	-0.351038034	-0.291680692	-0.523203366	-0.413177021
ENSDART00000034377	cpa5	2.043914839	0.12107472	0.19928898	0.161423339
ENSDART00000034421	cdk14	-0.37810724	-0.738234108	-0.33600573	-0.064327168
ENSDART00000034432	susd4	-0.32348116	0.086685706	0.47906988	0.317031782
ENSDART00000034441	tcp11l2	0.020775808	-0.208761169	-0.796222831	-0.248557177
ENSDART00000034523	tars	0.184292328	0.408838041	0.43347383	0.219690833
ENSDART00000034638	ccdc28a	-0.059055282	-0.067802136	-0.368600818	-0.274995246
ENSDART00000034705	ntmt1	-0.020729663	-0.187405157	-0.44452011	-0.329716724
ENSDART00000034737	cpne8	-0.307817154	-0.810818352	-0.660194536	0.005050706
ENSDART00000034784	adcyap1b	1.680397515	2.015053329	1.852069794	1.09666998
ENSDART00000034790	pcp4l1	-0.408039344	-0.390896541	-0.213514281	-0.035791454
ENSDART00000034829	rrp12	0.494057186	0.423927766	0.372142811	0.099318986
ENSDART00000034834	ppfia2	-0.326502158	-0.151128851	-0.013552631	0.061359529
ENSDART00000034849	grin1b	-0.325252553	-0.609188282	-0.185270574	0.122354295
ENSDART00000034850	dbi	1.011739121	0.830016329	0.708552366	0.209278197

ENSDART00000034883	mcf2a	-0.149015362	-0.337699782	-0.173487291	0.057006752
ENSDART00000034914	pvalb3	-0.375270417	3.922610155	4.415489459	3.238850832
ENSDART00000034935	desi2	0.073850834	0.377607475	0.427284588	0.193399467
ENSDART00000035031	sgk1	0.036456511	-0.228295468	-0.323414575	-0.393803665
ENSDART00000035067	abhd2a	-0.280282686	-0.122469223	-0.369410687	-0.218167862
ENSDART00000035093	col9a2	0.360349924	0.265960604	-0.052354611	-0.707154609
ENSDART00000035150	spast	0.060265858	0.143278561	0.351254395	0.195874089
ENSDART00000035152	kif26ab	0.198116066	0.127768449	0.42622306	0.434736458
ENSDART00000035239	nek1	-0.350009624	-0.188792207	-0.078432875	0.138948692
ENSDART00000035245	spire2	0.223570717	0.667119992	0.874236758	0.626613708
ENSDART00000035409	zc2hc1a	0.100393521	0.424633369	0.532716266	0.492034687
ENSDART00000035447	mtmr9	-0.272610717	-0.235611081	0.139540941	0.187819723
ENSDART00000035538	ptp4a3	-0.088675269	0.257809374	0.053091443	-0.336323707
ENSDART00000035628	srsf10a	-0.231452965	-0.136268467	-0.25871265	-0.135010634
ENSDART00000035670	polr2eb	0.35113115	0.294169174	0.063963778	-0.045691283
ENSDART00000035676	bnip3la	-0.292353372	-0.099807874	-0.059866968	-0.067415369
ENSDART00000035710	lin7c	-0.082526521	-0.252501014	-0.262742844	-0.186356266
ENSDART00000035737	slc11a2	0.286882779	0.187123756	0.119307344	-0.030269778
ENSDART00000035739	tmem134	0.246242014	0.369929781	0.082611565	-0.161334554
ENSDART00000035899	pkp2	0.428534184	1.016588803	1.016153499	0.312913104
ENSDART00000035907	sec24d	0.279449898	0.329558363	0.384805326	-0.077598974
ENSDART00000035944	clic5a	2.554130845	1.311062136	1.150965478	0.646568675
ENSDART00000036015	ryr1b	-0.116788998	-0.165312146	0.372637833	0.693396813
ENSDART00000036050	rs1a	-0.391328986	-0.392177441	-0.469736828	-0.271292581
ENSDART00000036153	ccdc3a	0.286306507	0.061698184	-0.1803174	-0.442994145
ENSDART00000036240	cers4b	-0.360584189	-0.184519104	-0.081616234	-0.015625515
ENSDART00000036373	cfap206	0.026049802	0.014543106	-0.421468514	-0.063922477
ENSDART00000036421	chek2	0.623199787	0.358982062	0.455251589	0.098448549
ENSDART00000036472	zgc:110852	-0.482143709	-0.509026678	-0.613205563	-0.094495965
ENSDART00000036513	trib3	0.704680301	0.933969628	0.780405513	0.230790744
ENSDART00000036531	gnai1	0.25941591	0.265271449	0.201009078	-0.026939111
ENSDART00000036581	cdk2	1.227545183	1.116666759	0.903071292	0.284538183
ENSDART00000036649	sfxn2	0.32661292	0.494950572	0.442482013	0.063629138
ENSDART00000036668	psmc1a	0.311771761	0.423109618	0.307731242	0.107467578
ENSDART00000036680	ptgr1	0.476212613	0.619935312	0.420689871	0.024158985
ENSDART00000036703	pfdn2	0.369409429	0.460943577	0.431067405	0.197162016
ENSDART00000036718	eif4e1c	0.366528838	0.391471958	0.37708707	0.13997088
ENSDART00000036729	spi1b	0.910663058	0.717004711	0.095052493	-0.117318513
ENSDART00000036760	tppp2	-0.225112914	0.608288105	0.560408419	0.667346525
ENSDART00000036797	uchl1	1.004812771	1.916965558	1.938952257	1.446591845
ENSDART00000036854	glcci1	-0.216167779	-0.276312685	-0.306037916	-0.0692944
ENSDART00000036891	rabac1	0.184715811	0.277575159	0.08241236	-0.087331685
ENSDART00000036926	vangl1	-0.329590873	-0.033233324	0.383553313	0.3451937
ENSDART00000036939	gadd45ba	1.15110753	1.253886794	1.06146692	0.82843487
ENSDART00000036997	camk2n1a	-0.377223565	-0.40737699	-0.242898936	-0.23919335
ENSDART00000037007	tpi1a	-0.595344218	-0.412573356	-0.319655345	-0.136192131
ENSDART00000037036	cnppd1	-0.251371332	-0.185078308	-0.27690601	-0.219995028
ENSDART00000037065	sccpdhb	-0.135500672	-0.263394229	-0.35527789	-0.147169147
ENSDART00000037109	srpk1a	-0.158297297	-0.128401953	-0.285156041	-0.13534624
ENSDART00000037126	eno2	-0.315372643	-0.196767149	0.300750061	0.433471501
ENSDART00000037195	kif26bb	1.206892082	1.55094827	1.874551064	1.519778039

ENSDART00000037224	cst14a.2	0.549538616	0.553010386	0.34149596	-0.057482802
ENSDART00000037265	olfm1b	-0.595000689	-1.108211527	-0.18099665	0.18250463
ENSDART00000037371	ppp1r13ba	-0.171240808	-0.158646598	-0.283609683	-0.120899195
ENSDART00000037698	uck2b	0.641974401	1.119646558	1.077673009	0.598617402
ENSDART00000037709	nol11	0.254424392	0.434300513	0.299474854	0.102573384
ENSDART00000037846	focad	0.435964232	0.512370823	0.456134064	0.112317494
ENSDART00000037848	dpp6b	-0.469010511	-0.191315433	-0.026922326	-0.081540125
ENSDART00000037850	dync1li2	0.268558729	0.496891146	0.585345447	0.309833954
ENSDART00000037879	crx	-0.254014844	-0.310425293	-0.22793154	-0.020194538
ENSDART00000037922	slc6a8	-0.298142259	-0.526146924	-0.491663812	-0.167137822
ENSDART00000038202	cndp2	1.63370988	0.725493655	0.197899383	-0.10985912
ENSDART00000038290	crhb	-0.533237668	-0.584543834	-0.601107267	-0.51443145
ENSDART00000038294	tp53inp1	-0.610161281	0.029061275	-0.033185195	-0.028504863
ENSDART00000038301	gnpda2	0.314221876	0.240267665	0.128522583	-0.033807169
ENSDART00000038310	ormdl3	-0.300841314	-0.124106092	-0.535837175	-0.413488994
ENSDART00000038330	khsrp	-0.050330793	0.071549867	0.273984704	0.161173774
ENSDART00000038391	pkz	0.411244814	0.129537507	1.595328762	0.021626224
ENSDART00000038495	ctnnb1	0.20653709	0.248644759	0.214149858	0.090949105
ENSDART00000038505	rprmb	-0.046727426	-0.153123084	-0.26580673	0.05838964
ENSDART00000038648	ptbp2b	0.207623817	0.150446749	0.253639385	0.163738179
ENSDART00000038674	tmem230a	-0.312877262	-0.360088684	-0.444507782	-0.485519275
ENSDART00000038696	flvcr2b	1.475160998	0.41510575	0.393658072	-0.063620422
ENSDART00000038740	galnt9	-0.705450847	-0.542663541	-0.098391024	0.177029625
ENSDART00000038888	hsdl2	0.331360296	0.293802861	0.248668235	0.085382052
ENSDART00000038924	sult1st1	0.0423301	-0.129133641	-0.497646461	-0.249352869
ENSDART00000038990	jak1	0.414591598	0.220201928	0.070657217	-0.102548961
ENSDART00000039043	rgs7bpb	-0.383267221	-0.448718374	-0.120132554	0.14756534
ENSDART00000039080	wasb	0.537774473	0.660070537	0.394408091	-0.045801603
ENSDART00000039161	hdhd2	0.366788589	0.057115496	-0.197391924	-0.146678217
ENSDART00000039206	rps23	0.388349294	0.322594165	0.070172062	-0.095059659
ENSDART00000039277	lhfpl3	-0.493635642	-0.41740998	-0.318633213	-0.189800628
ENSDART00000039295	lrrfip1a	0.201172846	-0.052744603	-0.60255637	-0.234396047
ENSDART00000039312	ipo4	0.472349209	0.340323729	0.32654819	0.07839591
ENSDART00000039399	cavin2a	-0.454722967	-0.463350309	-0.727916614	-0.482344794
ENSDART00000039443	tuba8l4	0.45016266	0.547239574	0.701695743	0.194541839
ENSDART00000039466	rnf25	0.190959954	0.415197213	0.378723734	0.235880676
ENSDART00000039485	gabarapl2	-0.111002032	-0.162035427	-0.331707696	-0.213225245
ENSDART00000039551	mef2ca	2.857314883	3.343180737	3.275011062	3.48649726
ENSDART00000039571	camk2a	-0.838498146	-0.905374301	-0.593078838	-0.446882891
ENSDART00000039585	klhl36	0.23105636	0.314614252	0.405624735	0.264885345
ENSDART00000039693	pgp	-0.344619279	-0.551044992	-0.227681437	-0.38433532
ENSDART00000039746	epb41b	0.556721113	0.962382373	1.378686301	0.45048608
ENSDART00000039788	uqcrq	-0.201371674	-0.242426303	-0.394323978	-0.159681057
ENSDART00000039865	sdhdb	-0.123077363	-0.199973196	-0.304203762	-0.144165352
ENSDART00000039868	usp4	-0.25602575	-0.139457748	-0.15513491	-0.170681969
ENSDART00000039987	pgm3	0.351341795	0.131342573	-0.075397144	-0.106723338
ENSDART00000040035	ccdc80l1	-0.195911171	-0.175911639	-0.396207214	-0.331422513
ENSDART00000040049	camk2d2	-0.441100303	-0.359209869	-0.184359733	0.062709499
ENSDART00000040066	adam9	0.358589887	0.583904956	0.549640677	0.256288209
ENSDART00000040086	pacsin1a	-0.311462824	-0.286213879	-0.425986821	-0.031983095
ENSDART00000040116	tnrc5	-0.132560784	-0.181210054	-0.368693498	-0.22290886

ENSDART00000040184	tenm1	-0.25843006	-0.314319075	-0.175496746	-0.005818614
ENSDART00000040275	kcnj11l	-0.790164361	-0.507671216	-0.85342057	-0.51870362
ENSDART00000040278	efna2a	-0.820985512	-0.748851824	-0.238813858	0.346525856
ENSDART00000040334	pik3r3b	-0.289525561	-0.103810065	0.221174364	0.296766414
ENSDART00000040346	efr3ba	-0.15754636	-0.295932904	-0.334562517	-0.212090743
ENSDART00000040434	asah1b	0.563509259	0.420868789	-0.056225927	-0.32971968
ENSDART00000040456	cdc42bpab	-0.484798472	-0.258449967	-0.177779388	0.139329121
ENSDART00000040500	tspan9a	-0.374305977	-0.544164723	-0.123124308	-0.153915406
ENSDART00000040502	trpc5a	-0.568729425	-1.014519921	-0.522696272	-0.18638518
ENSDART00000040537	gjd1a	-0.421902855	-0.214222628	0.04546907	0.177131603
ENSDART00000040542	arhgef12a	-0.286992716	-0.169562154	-0.197347098	-0.111099085
ENSDART00000040557	CRIP2 (1 of many)	-0.132882678	0.562285777	0.768710088	0.441120913
ENSDART00000040669	sphkap	-0.388395859	-0.454597188	0.021563144	0.051812339
ENSDART00000040672	mecp2	-0.242382035	-0.413395534	-0.149398689	-0.148226048
ENSDART00000040701	ing5a	0.004576245	-0.184827807	-0.360745549	-0.284929321
ENSDART00000040708	caprin2	-0.196766553	0.78279717	0.834337788	0.695156181
ENSDART00000040771	rpl34	0.291435523	0.406434571	0.222752448	-0.006604457
ENSDART00000040804	praf2	0.166225557	0.427661756	0.402173801	0.274020381
ENSDART00000040827	ncaph2	0.654906877	0.777407912	0.63027477	0.226543553
ENSDART00000040900	baxb	0.66837016	0.786823818	0.515591205	0.094501002
ENSDART00000041007	stmn1b	-0.180266883	0.501280237	0.34630752	0.107820579
ENSDART00000041114	psmb2	0.343581501	0.272779677	0.256301247	-0.006269993
ENSDART00000041191	gyg2	0.909324979	1.617533024	1.086480874	0.519765295
ENSDART00000041257	gsto2	0.760127953	0.383922047	-0.10799465	-0.396095115
ENSDART00000041279	tubb4b	0.433747505	0.468816243	0.626770783	0.445102188
ENSDART00000041388	cacng2a	-0.088843708	-0.391878721	-0.181623249	-0.021159404
ENSDART00000041417	camk1b	-0.259356662	-0.222277099	-0.408590245	-0.120593834
ENSDART00000041443	igsf21a	-0.224595347	-0.278632007	-0.419145006	-0.220026937
ENSDART00000041468	ap1ar	0.253231094	0.535778421	0.657902256	0.098067269
ENSDART00000041503	slc4a4a	-0.308542931	-0.316588825	-0.297229141	-0.158105481
ENSDART00000041504	tescb	0.44458368	-0.282095189	0.067591772	-0.116025945
ENSDART00000041707	unc119a	-0.21008147	-0.190962771	-0.5453967	-0.153347487
ENSDART00000041714	atp6v0a1b	-0.327889206	-0.302694525	-0.070700098	0.131627227
ENSDART00000041728	cyp26a1	-0.110572523	-0.30661175	-0.074827314	0.003208979
ENSDART00000041740	ubl7a	-0.21096997	-0.119372658	0.298633763	0.574535573
ENSDART00000041751	ercc1	0.342208194	0.265119266	0.084902107	0.028919147
ENSDART00000041800	epha8	-0.01929649	-0.346405332	0.164805877	0.748357168
ENSDART00000041805	metrn	-0.185458155	-0.302026239	-0.151541546	-0.276934548
ENSDART00000041820	lingo1a	-0.3685918	-0.267056385	-0.206204269	-0.004686852
ENSDART00000041861	syt1a	-0.326937152	-0.373717465	-0.346529457	-0.191055943
ENSDART00000041869	grin1a	-0.314741638	-0.554584424	-0.238417502	0.088185899
ENSDART00000041877	csrnp1a	1.299872398	1.369159722	1.350463811	0.717287101
ENSDART00000041992	dhrs12	0.21282475	0.60945999	-0.222288782	-0.248821854
ENSDART00000042083	gria4a	-0.246573777	-0.413439839	-0.038291419	0.265641743
ENSDART00000042123	cx52.6	-0.622226461	-0.079417665	-0.692157173	-0.611068819
ENSDART00000042134	dock7	0.063646619	0.209946461	0.48316367	0.274325565
ENSDART00000042162	tm7sf2	0.1050645	0.758038931	0.952561873	0.4137868
ENSDART00000042189	pdk2b	0.734399082	1.569570791	1.007918384	0.211070696
ENSDART00000042194	cers4a	-0.343157696	-0.266390398	-0.236875341	-0.142108033
ENSDART00000042200	aldoab	0.726673418	1.529219824	2.318485768	1.809025522
ENSDART00000042218	pafah1b1a	0.049322983	0.127134272	0.313096774	0.220746894

ENSDART00000042250	rap1b	0.339690926	0.041784403	0.019413921	-0.132436477
ENSDART00000042255	rab6bb	0.249992007	1.135776594	1.498200114	1.307756366
ENSDART00000042276	nxph1	-0.320960824	-0.220089718	-0.50268354	-0.230788559
ENSDART00000042297	kdelc1	0.328425656	0.736886183	0.437422887	0.039597286
ENSDART00000042307	fam60a	-0.454119179	-0.522927402	-0.624149133	-1.020018948
ENSDART00000042386	unm_sa1261	-0.368338169	-0.259611498	-0.383565927	-0.183992374
ENSDART00000042481	phf23a	-0.371442433	-0.230558793	-0.302265692	-0.28776115
ENSDART00000042572	ablim1b	0.023130185	-0.084172657	-0.818233794	-0.360713717
ENSDART00000042599	dennd6aa	-0.0532753	-0.029202145	-0.371955611	-0.136277272
ENSDART00000042624	arap3	-0.123633155	-0.147189694	-0.365577073	-0.154745368
ENSDART00000042683	cadpsb	-0.417914059	-0.394489719	0.149869704	0.381243736
ENSDART00000042963	chst11	0.296547318	0.476599287	0.638913095	0.263644905
ENSDART00000042972	srpk1b	-0.590887937	-0.595753709	-0.259675811	-0.090086557
ENSDART00000042984	epha6	-4.335346398	-2.736601299	-1.023821359	0.479123476
ENSDART00000043058	TENM2	-0.559723778	-0.553182426	-0.524601572	-0.11140967
ENSDART00000043076	ppdpfb	-0.038298907	0.045116804	-0.329828604	-0.321738012
ENSDART00000043091	iqsec1b	-0.102282724	-0.271543671	-0.319516795	-0.119256647
ENSDART00000043173	rpl18	0.389153851	0.347510342	0.235541148	0.00423952
ENSDART00000043180	gria3b	-0.628974157	-0.919920549	-0.567970281	0.039022237
ENSDART00000043226	guca1c	-0.531402053	-0.370893475	-0.804093243	-0.754648782
ENSDART00000043312	srsf5a	-0.406457659	-0.349179029	0.276259838	-0.003669678
ENSDART00000043429	jph2	-0.203422393	1.998676475	2.481431759	1.87668434
ENSDART00000043455	smad3b	-0.137393915	-0.114661975	-0.243963828	-0.226543078
ENSDART00000043492	trappc6bl	-0.12751818	-0.21743347	-0.476970052	-0.302538631
ENSDART00000043507	ciarta	-0.034891069	-0.156171585	-0.423543489	-0.111653268
ENSDART00000043651	dnal1	-0.182764591	-0.191705301	-0.306394992	-0.190228735
ENSDART00000043666	hs1bp3	1.060777617	0.463082325	0.168627927	-0.515305865
ENSDART00000043678	apobec2b	1.815363093	0.931239114	0.794076282	0.812995775
ENSDART00000043801	cabp5b	-0.44296443	-0.237302409	-0.884658288	-0.372660272
ENSDART00000043823	osbpl10b	-0.615302858	-0.415836047	-0.293972471	0.154234814
ENSDART00000043855	dclk2a	-0.104441255	-0.395008642	-0.62766162	-0.184810585
ENSDART00000043857	irx5a	-0.492824084	-0.561776224	0.072886283	0.234832625
ENSDART00000043924	mpp6b	-0.45376092	-0.273823891	-0.178237916	-0.157850955
ENSDART00000043932	atp2a1	1.138744452	4.285725335	5.558096993	3.994336477
ENSDART00000043933	ndufb7	-0.183005381	-0.27475981	-0.256491733	-0.145691426
ENSDART00000043945		-0.527606114	-0.345502165	-0.38556129	-0.085040536
ENSDART00000043953	mfsd2b	-0.226277331	-0.20295362	-0.451440771	0.020277702
ENSDART00000044000	plxna3	0.522678231	0.518528602	0.862237135	0.748340859
ENSDART00000044009	scdb	-0.293214367	-0.066392815	0.084209837	0.174747579
ENSDART00000044057	sept3	-0.17670354	0.196164293	0.478730018	0.43476862
ENSDART00000044150	dnajc9	-0.130894557	-0.15046904	-0.343680246	-0.373143883
ENSDART00000044154	tnnt2c	0.256126919	0.781011759	0.616305237	0.377618007
ENSDART00000044157	scn4ab	1.170816112	2.050777967	2.7725392	2.001159499
ENSDART00000044208	lmo1	-0.34602538	-0.891618988	-0.018405226	0.102622885
ENSDART00000044238	zgc:92066	1.060411872	1.124461317	0.621339815	0.051820002
ENSDART00000044241	KCNQ2 (1 of many)	-0.793205862	-0.43808803	0.589879296	0.84351704
ENSDART00000044264	mmp14b	0.997152397	1.115840894	0.915921565	0.094130452
ENSDART00000044276	dip2bb	-0.182549034	-0.085935994	0.236910154	0.379645319
ENSDART00000044294	fryb	-0.196859444	-0.540115494	-0.186847147	-0.085192364
ENSDART00000044314	itgav	0.188708464	-0.009103257	-0.190172369	-0.475418475
ENSDART00000044328	acss1	0.948368639	0.660526303	0.176495325	-0.460639914

ENSDART00000044371	tox	-0.382184105	-0.377592563	-0.085511133	0.05287602
ENSDART00000044423	magi1b	-0.521004582	-0.785194432	0.042021364	0.52355612
ENSDART00000044426	si:dkey-240h12.4	0.077516947	-0.039107616	-0.22879164	-0.507759534
ENSDART00000044453	ano5a	-0.027551778	0.223592536	0.548330121	0.427829524
ENSDART00000044647	ppil3	0.391623501	0.578322707	0.226885679	0.10080052
ENSDART00000044658	letmd1	0.311235989	0.320526266	0.39411459	0.349216193
ENSDART00000044678	GABRA2 (1 of many)	-0.18505593	-0.452041699	-0.058625322	0.015850219
ENSDART00000044733	NPBWR2	-0.533926323	-0.49881296	-0.385102988	-0.276547602
ENSDART00000044735	gria1b	-0.281408167	-0.37749512	-0.41239512	-0.179683873
ENSDART00000044860	maff	0.057535561	0.039208376	-0.366680053	-0.441007369
ENSDART00000044896	camk2d2	-0.471410347	-0.378784897	-0.323522916	-0.120871605
ENSDART00000044949	syt16	-0.001457693	-0.053066568	0.72824965	0.634879457
ENSDART00000044963	loxl4	1.867896709	1.134004715	1.5146614	0.310989698
ENSDART00000044986	rnd1a	-0.894483212	-0.275620607	-0.461458	-0.242681728
ENSDART00000045071	foxk2	0.01041623	0.131070865	0.347129546	0.265521213
ENSDART00000045086	prkceb	-0.397671937	-0.107653926	0.08012307	0.031299867
ENSDART00000045126	lama5	-0.044158589	0.007727115	-0.324426294	-0.496259172
ENSDART00000045232	mtss1la	-0.698215115	-0.447289487	0.119673501	-0.022641935
ENSDART00000045284	rpl37	0.494352175	0.447787745	0.229606145	-0.010646089
ENSDART00000045299	ola1	0.514191862	0.666266592	0.533008533	0.271611648
ENSDART00000045303	tmprss9	-0.116725282	-0.194770382	-0.365140883	-0.064254559
ENSDART00000045374	smad3a	-0.063329624	-0.157712113	-0.303211349	-0.108940629
ENSDART00000045391	srgap2	0.042169336	0.118632088	0.377794988	0.307955564
ENSDART00000045397	stx11b.1	1.953998203	1.413835145	1.924246083	1.02039349
ENSDART00000045410	thy1	0.906274294	2.922050483	2.917431913	2.440516966
ENSDART00000045479	syt4	0.199773705	0.561264928	0.42766733	-0.021116223
ENSDART00000045555	rab41	-0.452536385	-0.052299617	0.3824053	0.576200707
ENSDART00000045616	gabbr1b	-0.290531763	-0.52893984	-0.416723119	-0.192345482
ENSDART00000045628	irx6a	-0.424070635	-0.239579413	0.17038084	0.276181325
ENSDART00000045659	tcp11l1	0.225446633	0.445852163	0.278888616	0.19328127
ENSDART00000045675	slc52a2	0.16071928	0.405611618	0.292146022	0.046169521
ENSDART00000045682	rrp36	0.38981283	0.603671455	0.338055221	0.296271067
ENSDART00000045684	porcn	-0.472251654	-0.321238385	-0.136837542	0.064329115
ENSDART00000045697	zgc:56493	0.323838871	0.24043595	-0.000448434	-0.189381569
ENSDART00000045757	march5l	3.527750979	3.782483659	2.945706162	1.186881806
ENSDART00000045842	rcan3	-0.531033223	-0.615136042	-0.34287969	-0.068102336
ENSDART00000045861	slc43a2a	-0.304475968	-0.378976343	0.111239312	0.188992557
ENSDART00000045888	tkta	-0.540178562	-0.404491163	-0.145350137	0.055411831
ENSDART00000045933	sh3glb1b	0.424660866	0.274153114	0.044458674	-0.153303197
ENSDART00000046004	wnt2bb	-0.582117328	-0.289636407	-0.865141829	-0.874797573
ENSDART00000046050	pcbd1	0.519297333	0.526198373	-0.092439376	-0.055017497
ENSDART00000046066	capn1a	-0.174433704	-0.141559786	-0.304523077	-0.134358035
ENSDART00000046115	mfsd2aa	0.413173515	1.356876257	1.539393726	0.598141045
ENSDART00000046209	acbd7	0.172970527	0.805855312	0.630012733	0.123911744
ENSDART00000046211	lnx2a	-0.592157981	-0.322543056	-0.148137638	-0.128059395
ENSDART00000046218	flnca	6.980277858	6.302871335	6.08375751	5.531037957
ENSDART00000046253	prkcq	0.856641094	1.199706901	0.196829623	-0.396081861
ENSDART00000046268	pmelb	0.894230709	0.185058494	-0.384535315	-0.811085128
ENSDART00000046360	rhoua	-0.234114921	-0.11992892	-0.459682602	-0.396381281
ENSDART00000046438	kcnk2b	-1.164610191	-1.614380279	-0.460863171	0.258871322
ENSDART00000046498	sema3fa	-0.424113863	-0.318396861	-0.293026202	-0.101692261

ENSDART00000046530	rab42a	-0.542354056	-0.349553972	-0.104901238	0.132258211
ENSDART00000046542	igf1rb	-0.265329623	-0.432225627	-0.135218314	-0.147354602
ENSDART00000046587	ap2m1a	0.393397352	0.521859633	0.500225377	0.048547949
ENSDART00000046626	PRKAR2B	-0.181202107	-0.272867538	-0.191341626	-0.012099353
ENSDART00000046663	camta1b	-0.175908238	-0.543108898	-0.289499765	-0.001152787
ENSDART00000046678	pak2b	0.067537468	0.265866932	0.367938524	0.197303091
ENSDART00000046689	tmed3	0.310928608	0.094278442	-0.151364778	-0.47701209
ENSDART00000046712	zgc:86609	0.278113439	0.188615214	-0.290177028	-0.454070036
ENSDART00000046716	cited2	-0.252125812	-0.225219779	-0.427941506	-0.423861644
ENSDART00000046764	gmds	0.16546148	0.334907039	0.47465655	0.2924672
ENSDART00000046922	rab13	0.426602975	0.215892939	0.022612864	-0.308555932
ENSDART00000046933	sult1st5	0.218426293	0.869949792	0.854237199	0.702293309
ENSDART00000046934	coq9	-0.280335275	-0.284319265	-0.255484353	-0.03877329
ENSDART00000046951	ptpn11b	-0.12533711	-0.212829251	-0.391615008	-0.186327808
ENSDART00000046973	capza1a	0.117659515	0.282132124	0.303069026	0.119362421
ENSDART00000046995	txn2	-1.624875893	-1.011052145	-0.305336636	-0.0755861
ENSDART00000047020	casp9	0.406413734	0.845063479	0.618979742	0.291728295
ENSDART00000047069	tyms	1.498823911	1.293125646	0.841397173	0.487713483
ENSDART00000047073	oxsr1a	0.179223329	0.268680956	0.376160209	0.212719894
ENSDART00000047082	gdap1l1	-0.237605119	0.285676986	0.669438597	0.59731396
ENSDART00000047126	clcn4	-0.325495856	-0.290597126	0.018460992	0.082569002
ENSDART00000047143	specc1	0.250947915	0.397558549	0.627196335	0.309975843
ENSDART00000047175	ssr4	0.274556908	0.120119155	0.036407988	-0.174453213
ENSDART00000047191	glb1l	1.329711044	0.552831622	0.213363411	-0.364178392
ENSDART00000047362	msra	-0.031803809	-0.196438308	-0.422002476	-0.295991402
ENSDART00000047378	sst3	-0.489863502	-0.562162931	-0.649979789	-0.586308909
ENSDART00000047399	mmp24	-0.337136574	-0.281370104	0.467116638	0.594580786
ENSDART00000047409	myh14	0.151853201	0.372826224	0.643058289	0.555183408
ENSDART00000047416	slc4a8	-0.393460236	-0.539636776	-0.542467324	-0.330988764
ENSDART00000047541	bach1b	-0.343772395	-0.510091534	-0.468889885	-0.333515696
ENSDART00000047569	igf2b	-0.538912754	-0.46572679	-0.194582206	0.102619195
ENSDART00000047662	ppp1r13bb	-0.080629431	-0.11550057	-0.271468608	0.046859797
ENSDART00000047728	melk	0.808255278	0.366287521	0.23278244	0.524042022
ENSDART00000047857	orc3	3.881796907	3.620009487	3.715059064	3.478890874
ENSDART00000047954	ugcg	-0.094752728	0.184472082	0.264381156	0.087288534
ENSDART00000048036	gem	-0.746612275	-0.695957849	-0.479539177	-0.330372498
ENSDART00000048050	ITGB1BP2	-0.056534701	0.311580562	0.724894589	0.983194822
ENSDART00000048073	zgc:171775	0.625715462	0.657465213	0.200858631	-0.066721925
ENSDART00000048107	FP102018.1	0.402516182	0.222629184	-0.14999501	-0.581967671
ENSDART00000048110	six4b	0.757934328	2.322346312	2.776043581	1.88012445
ENSDART00000048365	syt6b	-0.461087231	-0.40037241	-0.458496339	-0.153902769
ENSDART00000048383	creld2	0.499343204	0.520824125	0.482480278	0.212076819
ENSDART00000048432	dlg4a	-0.380839229	-0.483618741	-0.31591813	-0.034480778
ENSDART00000048599	rps19	0.313599405	0.28541891	0.281484325	-0.024061243
ENSDART00000048707	srgap1b	-0.90586475	-0.695392849	-0.011664453	0.331175331
ENSDART00000048775	mbd3b	-0.291847963	-0.361950456	-0.358913558	-0.222238531
ENSDART00000048819	rassf2a	-0.204366055	-0.399787028	-0.377471637	-0.118487631
ENSDART00000048853	ube2d1a	0.133955448	0.280395319	0.282059322	0.156573319
ENSDART00000048855	mtus1b	-0.449558058	-0.427741832	0.166302333	0.000701193
ENSDART00000048866	ipmkb	-0.299703051	-0.158343907	0.084121168	0.000422995
ENSDART00000048871	desi1a	-0.247171878	-0.259133346	0.047182486	0.101740307

ENSDART00000048890	slc22a2	0.30008182	0.486824781	0.442015061	0.075671897
ENSDART00000048893	pcbp3	-0.37198127	-0.476664366	-0.345261768	-0.190932807
ENSDART00000048940	vill	-0.011902914	-0.216832622	-0.570278713	-0.337359605
ENSDART00000048977	abcf1	0.295994115	0.413143633	0.435605861	0.158915281
ENSDART00000048994	pbx3b	-0.545498331	-0.428509616	0.129533791	0.149586816
ENSDART00000049036	zgc:92275	-0.447578375	-0.84153571	-0.52710535	-0.386325614
ENSDART00000049075	add3a	-0.25124279	-0.167771329	-0.330721354	-0.242087591
ENSDART00000049099	trip13	1.738467479	1.594316711	0.735690402	0.585616546
ENSDART00000049135	si:dkey-261m9.12	0.781337004	0.750536245	0.608174972	0.211334904
ENSDART00000049154	pthlha	-0.57822331	-0.570371268	-0.522569114	-0.481173816
ENSDART00000049177	rab6ba	-0.532131764	-0.115244957	0.417564602	0.557714845
ENSDART00000049194	gpr37b	-0.562015213	0.012994785	0.671207906	0.757619967
ENSDART00000049240	tob1a	-0.553621268	-0.528794995	-0.629368997	-0.384085742
ENSDART00000049264	sdr16c5b	0.496225849	0.925512272	0.840394736	0.492626723
ENSDART00000049291	gria3a	0.136141556	-3.339713475	0.061156993	0.119617746
ENSDART00000049368	atat1	-0.456100083	-0.161841688	-0.10743096	0.240512647
ENSDART00000049373	cmtr1	3.529734054	4.85424569	5.483410402	3.408663479
ENSDART00000049425	sec61a1l	0.040274537	0.042437444	0.248643876	0.074434633
ENSDART00000049434	scamp4	0.767657025	0.801831806	0.544244726	0.399234701
ENSDART00000049437	cdc42bpb	0.021859432	-0.018472292	0.242347511	0.210611193
ENSDART00000049462	rab15	-0.425089968	-0.185335259	0.21479615	0.289096284
ENSDART00000049464	fermt2	0.28468341	0.302053037	0.328009634	0.040193822
ENSDART00000049465	slc19a1	0.32288456	0.126425631	-0.165912503	-0.040050165
ENSDART00000049572	ncapd3	0.694925235	0.6794902	0.488650623	0.149625384
ENSDART00000049589	col11a1b	0.748320952	1.159222727	1.621624084	0.283450272
ENSDART00000049633	zgc:110006	-0.212812767	-0.199160747	-0.43210422	-0.304919838
ENSDART00000049676	depdc1a	1.209441584	0.891467797	0.780861798	0.190374501
ENSDART00000049684	bag2	0.817498026	0.419391857	0.164101637	0.068852338
ENSDART00000049722	pfdn5	0.271437948	0.37616378	0.12572137	-0.064637497
ENSDART00000049793	gstm.1	-0.113008537	-0.081297777	-0.307261435	-0.386339749
ENSDART00000049836	bgnb	-0.474953019	-0.067188313	-0.175252125	-0.757542027
ENSDART00000049885	si:dkey-172j4.3	-0.146868316	-0.089398841	-0.317501099	-0.03734398
ENSDART00000049900	tagln2	0.588048217	1.077774727	1.105142914	0.190917953
ENSDART00000049992	syt9b	-0.672988403	-0.530192885	0.095421952	0.328394957
ENSDART00000050018	cnksr1	0.043033394	-0.349786212	-0.053406139	-0.094004348
ENSDART00000050037	chrnb3b	-0.414911692	-0.004263798	0.444800143	0.950014126
ENSDART00000050077	sdcbp	-0.585127298	-0.412438306	-0.175403243	-0.033038502
ENSDART00000050140	CABZ01088365.1	-0.08566459	-0.329955925	-0.093026456	-0.10850944
ENSDART00000050202	rcan3	-0.240590006	-0.307380747	-0.423427653	-0.130410568
ENSDART00000050217	efna1a	-0.276270159	-0.087399972	-0.269604188	-0.23920109
ENSDART00000050230	tspan3a	-0.260999172	-0.146975695	-0.364155673	-0.280149491
ENSDART00000050271	hexb	0.237342774	-0.182676311	-0.489143261	-0.233593029
ENSDART00000050303	b3gat2	-0.439065182	-0.488507748	-0.270928467	0.095807034
ENSDART00000050308	calm1b	-0.359535363	-0.241046962	-0.074464366	0.015414816
ENSDART00000050311	rltpr	-0.433814029	-0.24205503	0.339832334	0.433192877
ENSDART00000050332	gna12a	0.472300359	0.391620605	0.3757936	0.086884817
ENSDART00000050352	si:ch211-87m7.2	0.324289445	0.10825652	0.093171262	-0.186818955
ENSDART00000050399	npc2	0.729567353	0.287798394	-0.063690935	-0.310097689
ENSDART00000050445	trim2a	-0.427473932	-0.288810529	-0.127175492	0.041370255
ENSDART00000050559	sh3rf1	-0.402839399	-0.313691549	-0.2479685	-0.17125224
ENSDART00000050750	rrm2b	-0.324758311	-0.221575036	-0.394470152	-0.266012642

ENSDART00000050753	cd36	-0.281884107	0.047747268	0.431169618	0.458008288
ENSDART00000050762	phactr3b	-0.629592387	-0.363049766	-0.266715209	-0.083768273
ENSDART00000050847	GLDC	0.384969982	0.265449522	0.198357794	0.108750757
ENSDART00000050863	zgc:101858	0.531270185	0.546733132	0.428408309	0.278322374
ENSDART00000050898	ncf1	0.6701936	0.170526908	-0.157127601	-0.640736559
ENSDART00000050910	orai2	-0.105799627	0.023857148	0.452924674	0.120793773
ENSDART00000051182	arhgap4b	-0.41225839	-0.320516515	-0.225939723	-0.055826953
ENSDART00000051197	c10h21orf59	-0.175790498	-0.1331203	-0.516409592	-0.442586367
ENSDART00000051231	gnb2	-0.235263329	0.124417594	0.575032632	0.495502612
ENSDART00000051234	tnika	0.106489865	0.189584243	0.309030435	0.511149463
ENSDART00000051357	zmat5	-0.126081267	-0.164432805	-0.376616148	-0.245219161
ENSDART00000051392	spns3	1.320734766	0.750958136	0.798555508	-0.096372125
ENSDART00000051491	sfrp1a	0.324084954	0.211296795	-0.122014524	-1.0775933
ENSDART00000051515	zgc:110329	0.175997647	0.392691176	0.340182733	0.206652443
ENSDART00000051516	tacr1a	-0.234436772	-0.252170036	-0.371966451	-0.377528767
ENSDART00000051518	rasa1a	-0.264162997	-0.276154428	0.166590332	0.366548962
ENSDART00000051546	rps6ka3a	-0.063638799	0.031915539	-0.376231824	-0.261540289
ENSDART00000051552	mpdu1a	-0.142610001	-0.050886814	-0.417815071	-0.558529014
ENSDART00000051556	abca1b	0.962705135	0.587027009	0.239926413	-0.279230387
ENSDART00000051566	zgc:101016	0.072400267	-0.278957969	-0.822407258	-0.192676389
ENSDART00000051614	tchp	0.132551453	0.24050278	0.418776194	0.215450614
ENSDART00000051621	pgam5	0.243607349	0.33552261	0.455304621	0.149179919
ENSDART00000051644	coq5	-0.310043454	-0.139390844	-0.270196538	-0.205912752
ENSDART00000051655	snrnp27	-0.149284802	-0.196914421	-0.340354698	-0.296345743
ENSDART00000051664	ypel1	-0.285276636	-0.074600808	-0.32874479	-0.098702346
ENSDART00000051666	ppm1f	0.089738986	0.277096017	0.445138137	0.206148798
ENSDART00000051693	irx4a	-1.596922819	-2.014349322	0.164610846	0.924604512
ENSDART00000051697	evla	-0.055749697	0.354222037	0.437976697	0.322467243
ENSDART00000051723	si:ch211-193k19.1	-0.524075663	-0.333228258	-0.259244771	-0.088981638
ENSDART00000051763	rps3a	0.36528781	0.402890251	0.287932185	0.011460462
ENSDART00000051792	sema3aa	0.043541661	-0.100781063	-0.302534685	-0.247434431
ENSDART00000051807	lasp1	0.121443442	0.384838028	0.393366972	0.231292626
ENSDART00000051906	ube2c	1.239012745	1.032037835	0.553625781	0.164264884
ENSDART00000051919	n6amt1	0.421272913	0.445198021	0.366479977	0.202998784
ENSDART00000051948	si:dkey-17m8.2	-2.249269734	-0.678959768	-1.034434829	-1.318620097
ENSDART00000051974	drd4b	-0.419693377	0.024027774	-0.191636302	-0.159413451
ENSDART00000052029	cart3	-0.527262177	-0.518524777	-0.63363087	-0.567396556
ENSDART00000052061	cnn2	0.810381891	0.55886321	0.442877218	-0.431652883
ENSDART00000052065	si:rp71-39b20.4	-0.313076871	-0.443642109	-0.50831344	-0.2481846
ENSDART00000052067	insl3	-0.34446258	-0.603612124	-0.679111871	-0.291897986
ENSDART00000052082	rpl30	0.377785904	0.342931306	0.098750304	-0.086094728
ENSDART00000052083	fjx1	-0.061315226	-0.667932816	-0.149604151	0.07387804
ENSDART00000052090	fuca1.2	0.607737239	0.489644017	0.400376939	0.091450373
ENSDART00000052104	fuca1.1	0.706031916	0.625980807	0.554044121	0.174374129
ENSDART00000052113	lingo1b	-0.337763041	-0.148396867	-0.080123582	-0.033848116
ENSDART00000052124	fam49al	-0.409057809	-0.177161374	0.563238983	0.47605027
ENSDART00000052126	yars	0.350203533	0.365550787	0.323606131	0.105596802
ENSDART00000052168	hrh3	0.375251408	0.91270689	1.213693931	0.380712944
ENSDART00000052256	sumo3b	-0.129514175	-0.087060285	-0.253552673	-0.198560199
ENSDART00000052307	arrdc3b	-0.385234832	-0.285495364	-0.380608917	-0.283766566
ENSDART00000052318	mdka	-0.324471926	-0.37363799	-0.54050807	-0.370260207

ENSDART00000052322	zgc:110699	-0.266905411	-0.248249923	-0.482205646	-0.223841905
ENSDART00000052331	rps20	0.422414342	0.403711723	0.215205833	-0.059084102
ENSDART00000052346	gnao1b	-0.534691372	-0.39418636	-0.143300564	0.218777626
ENSDART00000052351	cnep1r1	-0.133092087	-0.135980199	-0.291785579	-0.071751039
ENSDART00000052385	tph1b	-0.370644209	-0.494882963	-0.236758451	-0.433606661
ENSDART00000052397	pias1a	-0.248631388	-0.263511268	-0.331035933	-0.095610748
ENSDART00000052421	txnipa	1.054359781	1.031010785	1.048664605	0.430054853
ENSDART00000052423	spry2	-0.373633249	-0.274093568	-0.317163264	-0.534996547
ENSDART00000052503	nudcd1	0.220268862	0.211574095	0.29643349	0.134588484
ENSDART00000052511	hnrnpa0l	0.098563881	0.214926285	0.391405743	0.211573162
ENSDART00000052537	zgc:163107	0.254281338	0.227112479	0.142188905	0.117476144
ENSDART00000052539	myo1ea	0.574953247	0.619444947	0.654028338	0.173056841
ENSDART00000052541	ccnb2	1.168966788	1.017487756	0.69127691	0.504285143
ENSDART00000052620	npy	-0.456788991	-0.449905237	-0.529362885	-0.310086201
ENSDART00000052638	slc27a2a	0.420886529	0.920976528	0.833136576	0.001602661
ENSDART00000052656	rras2	0.556656504	0.801505793	0.534247735	0.118931735
ENSDART00000052703	nucb2b	-0.072514611	-0.317049949	-0.490530665	-0.459714357
ENSDART00000052730	rps13	0.392793291	0.407259594	0.231403052	0.009148185
ENSDART00000052749	nod1	0.321629747	0.111770638	-0.064002417	-0.693750338
ENSDART00000052761	rpl39	0.335691979	0.34926929	0.1141905	-0.000371665
ENSDART00000052802	calb2b	-1.334486374	-1.407708939	-0.475837952	0.218368357
ENSDART00000052838	acta1a	0.35046601	2.371192607	2.304510866	0.91139897
ENSDART00000052871	pop7	-0.057197275	0.295354721	0.463638671	0.27819074
ENSDART00000052912	pcdh20	-0.38541457	-0.110151808	-0.462368809	-0.238274084
ENSDART00000052915	ash1l	0.173309311	0.167145004	0.317504355	0.12008578
ENSDART00000052917	slc3a2a	0.509465627	0.333845604	-0.024881296	-0.285712584
ENSDART00000052989	ache	-0.369275035	-0.306496087	-0.342496448	-0.099646921
ENSDART00000053001	tcn2	0.058701765	-0.020005828	-0.457202856	-0.602127411
ENSDART00000053003	hexim1	-0.295110864	-0.174423589	0.055276892	0.067518179
ENSDART00000053095	rhbdf1a	0.708583914	0.695350284	0.352388623	0.004181603
ENSDART00000053120	gpr185b	-0.57955621	-1.320827874	-0.56412655	-0.654076001
ENSDART00000053126	aanat1	-0.126925707	-0.471091345	-0.287328219	-0.040926683
ENSDART00000053139	atp6v0cb	-0.239525606	-0.317576827	-0.287566582	-0.021198281
ENSDART00000053187	stk35l	-0.090868048	-0.135202146	-0.364753171	-0.24725243
ENSDART00000053240	cab39l	0.313790093	0.462769475	0.441611468	0.290026864
ENSDART00000053267	hnrnpa1b	0.306954561	0.087684612	0.037367395	-0.002841599
ENSDART00000053284	bcl9	-0.057531558	0.216352129	0.432379429	0.260365529
ENSDART00000053285	ndufa7	-0.051624088	-0.210448889	-0.329202666	-0.117246353
ENSDART00000053304	si:ch211-114n24.6	0.627425485	0.596987541	0.770158215	0.21603311
ENSDART00000053310	tmem18	-0.091641733	-0.057752607	-0.353980434	-0.264448619
ENSDART00000053325	tomm40l	0.351677862	0.400415779	0.406067875	0.134025152
ENSDART00000053367	hmgn3	-0.174648563	-0.165335262	-0.310027237	-0.181225286
ENSDART00000053380	hax1	-0.143446597	-0.149458858	-0.363330115	-0.294300868
ENSDART00000053405	scamp2	0.377844511	0.106568039	0.060099408	-0.324550719
ENSDART00000053463	mgll	-0.18197879	-0.150318561	-0.320930933	-0.180307684
ENSDART00000053494	anks4b	-0.562445249	-0.270911364	-0.580434775	-0.289114333
ENSDART00000053750	acsl2	-0.784570064	-0.626588121	-0.673780261	-0.449722468
ENSDART00000053761	bms1	0.415838377	0.296751152	0.241962107	0.100266391
ENSDART00000053773	lsm6	-0.104076864	-0.094751595	-0.350900229	-0.413521857
ENSDART00000053806	gab1	2.484308926	2.134716604	2.429792903	1.630078037
ENSDART00000053834	psmc6	0.096829198	0.261162402	0.176391614	0.055545263

ENSDART00000053841	ddhd1b	-0.251051379	-0.26914625	-0.031733869	0.07838534
ENSDART00000053860	saxo2	-0.675755032	-0.618170257	-0.767927477	-1.172976969
ENSDART00000053869	slc44a2	0.03477078	0.96666703	1.3453814	0.899279007
ENSDART00000053916	mtnr1ab	-0.333808774	-0.363646472	-0.291230509	-0.411800208
ENSDART00000053925	mtmr7a	0.252266702	0.492232945	0.532543286	0.441567176
ENSDART00000053932	cbsa	1.196219296	0.806157212	0.553945426	0.279319174
ENSDART00000054007	slc8a4b	-0.011734485	-0.299949751	-0.451416872	-0.315264472
ENSDART00000054020	hivep3b	0.041000622	-0.364922266	-0.147208921	-0.149490959
ENSDART00000054026	rcc1	0.376288807	0.340916908	0.360562186	0.188805345
ENSDART00000054062	nek12	0.979847079	2.121821413	1.72566276	1.104924836
ENSDART00000054070	surf2	0.311577894	0.338934517	0.34052692	0.125736963
ENSDART00000054071		0.085813894	-0.09956216	0.306019728	0.602458638
ENSDART00000054078	rpa2	0.380370186	0.444560906	0.363788059	0.096462711
ENSDART00000054137	igfbp5b	-0.49960111	-0.321727345	0.242454388	0.365417817
ENSDART00000054175	smad5	0.268560343	0.329305064	0.367006369	-0.092635665
ENSDART00000054202	si:ch211-145b13.5	-0.57317034	0.068003489	-0.485735579	-0.183705508
ENSDART00000054209	cdkn2a/b	-0.651631236	-0.494220791	-0.672480074	-0.361655459
ENSDART00000054243	dpf2l	0.196658591	0.403359284	0.517168503	0.26603871
ENSDART00000054322	cnrip1b	-0.129671726	-0.251461369	-0.057872634	-0.024935203
ENSDART00000054386	qdprb1	-0.130677564	-0.146959168	-0.522581238	-0.268035824
ENSDART00000054408	gsg1l	-0.338795387	-0.334881942	-0.642215658	-0.428166696
ENSDART00000054452	dlgap1b	-0.55212867	-0.830902632	-0.186012386	0.281181047
ENSDART00000054462	smim19	-0.090105146	-0.174385509	-0.37090077	-0.289350438
ENSDART00000054472	tll1	-0.241128673	-0.208136	-0.494254448	-0.202017577
ENSDART00000054552	cdh8	-0.482196874	-0.526834841	-0.273874965	0.0505098
ENSDART00000054574	polr1e	0.39788071	0.231113321	0.09078837	-0.014285707
ENSDART00000054581	march1	-0.100945889	-0.377180996	-0.379666436	-0.107576973
ENSDART00000054664	tnnc1b	-0.083935925	3.176389417	3.430200861	1.884280525
ENSDART00000054674	mtnr1aa	-0.46530953	-0.508091587	-0.367618326	-0.477932737
ENSDART00000054687	il1rapl2	-0.293064258	-0.530563685	-0.194225592	0.06661817
ENSDART00000054689	atoh8	-0.386924835	-0.23478989	-0.297672036	-0.53086166
ENSDART00000054691	uba1	0.10035699	0.287144805	0.420196482	0.237625478
ENSDART00000054735	SYNPR (1 of many)	-0.204530212	-0.189982465	-0.242945057	-0.369440481
ENSDART00000054736	bhlhe23	-0.369485488	-0.514619457	-0.390416516	-0.190127988
ENSDART00000054760	zgc:162144	-0.154581168	-0.586579914	-0.580706302	-0.278802593
ENSDART00000054790	zmp:0000001069	-0.970537582	-1.254323928	-0.841864806	-0.072822598
ENSDART00000054833	rgs11	-0.582556718	-0.789821251	-0.427532995	0.127688375
ENSDART00000054837	ap1s2	-0.004289143	0.194115514	0.293597748	0.151563344
ENSDART00000054849	pls3	0.365005671	0.852391802	1.00799598	0.660436059
ENSDART00000054867	aup1	-0.021467306	-0.053295505	-0.309019838	-0.217937602
ENSDART00000054876	npm1b	0.13266464	0.298387409	0.130756001	0.083140409
ENSDART00000054877	fgf24	-0.371366592	-0.422467709	-0.342651042	-0.342396761
ENSDART00000054987	actb1	0.719668079	0.845173501	0.884967639	0.398548729
ENSDART00000054989	fscn1b	-0.529299226	-0.417661269	0.066759573	0.494675778
ENSDART00000055019	ndufa4	-0.243803126	-0.403243102	-0.444723985	-0.066106721
ENSDART00000055038	rybpa	-0.189184893	-0.138456944	0.268105644	0.373370341
ENSDART00000055071	nptx2a	-3.565846773	-2.700342636	-0.646259822	-0.789148036
ENSDART00000055134	ogfr	-0.383562939	-0.286066309	-0.21229445	-0.172842524
ENSDART00000055139	col9a3	0.340683061	0.20859259	-0.192921312	-1.195377277
ENSDART00000055152	taf11	-0.482193927	-0.885413263	-0.244432703	-0.649425725
ENSDART00000055160	il11a	1.677180571	0.610178512	-0.274837581	-0.887218575

ENSDART00000055171	grapa	0.991444674	1.026501701	0.907734856	0.689609359
ENSDART00000055186	atp5j2	-0.168904246	-0.309514947	-0.449394402	-0.201772036
ENSDART00000055253	filip1l	3.442073683	3.796340861	4.199635374	3.015929109
ENSDART00000055262	cdk5r1a	-0.345552765	-0.416237328	-0.217352163	-0.195826689
ENSDART00000055264	CA10 (1 of many)	-0.387267589	-0.307777286	-0.264643025	-0.127280824
ENSDART00000055269	gng13b	-0.70656822	-0.547882014	0.352208471	0.531882213
ENSDART00000055287	zgc:109934	0.762332695	0.487859158	-0.045189444	-0.423101752
ENSDART00000055325	psmb7	3.620821341	3.668842367	3.694371073	3.321198364
ENSDART00000055328	nek6	0.491226657	0.428884947	0.11516767	-0.052210357
ENSDART00000055336	dennd1a	0.280502184	0.250804932	0.331143912	0.195537503
ENSDART00000055340	fus	-0.011490396	0.383733399	0.662780299	0.416441518
ENSDART00000055380	tubb5	2.169554793	3.899556169	4.316772272	3.57490014
ENSDART00000055395	osr2	0.396980684	1.020974716	1.320896999	1.066319399
ENSDART00000055428	cbx7a	0.780269243	0.615351279	0.524687311	-0.270346219
ENSDART00000055465	si:ch211-149k23.9	-0.23663044	-0.394558494	-0.331258304	-0.323817823
ENSDART00000055473	grb2b	-0.073731957	0.038177184	-0.265388265	-0.174944785
ENSDART00000055487	chmp3	-0.028426397	-0.094862832	-0.316592568	-0.291442829
ENSDART00000055492	ddx5	0.105976659	0.207326629	0.32877191	0.140872666
ENSDART00000055567	gnrhr4	-0.356419162	-0.425772903	-0.482362822	-0.623317801
ENSDART00000055607	pdgfbb	-0.474199721	-0.401424866	-0.415940684	-0.433592391
ENSDART00000055609	atf4b	0.138344185	0.51758353	0.353617358	0.087935611
ENSDART00000055611	isca2	-0.127743819	-0.187288303	-0.524298231	-0.307747827
ENSDART00000055694	cdab	-0.082751258	-0.099425929	-0.28235988	-0.295865587
ENSDART00000055706	her15.1	-1.483608056	-1.154441068	-0.698110478	-1.436427969
ENSDART00000055709	her2	-0.984524994	-0.833253569	-0.717745512	-1.233522642
ENSDART00000055710	aldh4a1	-0.225772345	-0.209769264	-0.373180163	-0.178717825
ENSDART00000055756	tbc1d12a	-0.033893992	0.095679716	0.342628218	0.232107662
ENSDART00000055779	ggact.2	-0.390632373	-0.197002813	-0.565987089	-0.546417063
ENSDART00000055780	jpt2	0.208785852	0.245567055	0.12691728	-0.082734531
ENSDART00000055817	PIGG	-0.041747749	-0.262998575	-0.390921965	-0.198616638
ENSDART00000055890	znf385c	-0.397413591	-0.444425008	-0.419820845	-0.297765515
ENSDART00000055913	hist2h2l	-0.328500488	-0.315748168	-0.27355808	-0.150594371
ENSDART00000055932	pigh	-0.149859895	-0.076712594	-0.279647992	-0.164515657
ENSDART00000055936	isl2b	-1.527471977	-1.312125436	0.470803449	0.853726063
ENSDART00000055995	sagb	-0.512319213	-0.180320177	-0.444781708	-0.186196646
ENSDART00000056005	ascl1a	1.787445127	1.283325532	0.749862779	0.792492648
ENSDART00000056035	PMM1	-0.318342084	-0.457172695	-0.421149458	-0.159828821
ENSDART00000056081	sulf1	1.721344224	3.792219559	4.448503543	2.727690528
ENSDART00000056138	igsf8	-0.41438699	-0.276734989	-0.097715498	0.052342087
ENSDART00000056213	pik3r1	-0.34639661	-0.306212461	-0.436349985	-0.247937167
ENSDART00000056254	stap2a	0.934389037	1.208596726	1.287672321	0.743017194
ENSDART00000056278	SLC25A22 (1 of many)	-0.288459944	-0.379459616	0.002234165	0.21084669
ENSDART00000056286	h1f0	-0.326358805	0.273543898	0.626230484	0.593578182
ENSDART00000056294	pitrm1	0.368247543	0.389541704	0.328836098	0.178268037
ENSDART00000056295	psap	0.394315497	0.070717853	-0.202183441	-0.455675198
ENSDART00000056305	fzd8b	-0.95322142	-0.49703101	0.067617805	0.048955399
ENSDART00000056328	elovl4b	-0.472600721	-0.194626997	-0.10087467	0.110456628
ENSDART00000056333	CU929150.1	0.302438567	0.370614966	0.439965996	0.209083279
ENSDART00000056369	cadm2a	-0.177754586	-0.327047473	-0.068598077	-0.03429359
ENSDART00000056376	tmem55ba	0.03327073	0.191352043	0.318689436	0.072412325
ENSDART00000056420	alas2	0.719442022	0.937854985	1.477738462	0.179843085

ENSDART00000056457	mitfa	0.929754118	0.38849893	0.30836176	-0.311729197
ENSDART00000056460	gbp1	3.785035011	3.151116027	3.74676295	2.092352698
ENSDART00000056466	camk2d1	-0.163681889	-0.265767151	-0.291660383	0.000303403
ENSDART00000056514	gng7	-0.676363728	-0.720108736	-0.535048991	-0.288646977
ENSDART00000056522	skila	-0.013727131	-0.304210156	-0.124205506	-0.010199259
ENSDART00000056540	casq1a	0.340240931	1.131481384	1.513535686	1.324229104
ENSDART00000056544	tox4a	0.131870382	0.296259606	0.350040714	0.249645538
ENSDART00000056577	RBP1	0.71451345	0.476305531	0.296937751	0.089894332
ENSDART00000056639	faim2a	-0.345615546	-0.285647372	-0.169343793	0.000937504
ENSDART00000056671	brinp2	-0.564206507	-0.55260615	-0.353392227	0.031594069
ENSDART00000056686	mrc1b	0.993000172	0.810204425	0.489266441	-0.068136928
ENSDART00000056712	etfdh	0.23563688	0.425716128	0.377386118	0.136575345
ENSDART00000056721	ldhd	-0.445248015	-0.387879357	-0.435006793	-0.179899207
ENSDART00000056734	setd7	-0.044426299	-0.050437779	-0.332150365	-0.191170985
ENSDART00000056735	rgs20	-0.263112929	-0.317514258	-0.646869905	-0.425880318
ENSDART00000056795	hectd3	0.187585997	0.34309988	0.435708832	0.360997812
ENSDART00000056810	drd1b	-0.453660686	-0.522513213	-0.435699921	-0.247093083
ENSDART00000056865	ctnnbip1	0.431885199	0.971175617	1.010655462	0.599546466
ENSDART00000056885	CU929046.1	-0.462145697	-0.331087748	0.285634599	0.389113052
ENSDART00000056893	pdcd7	0.040703622	0.122107923	0.35136997	0.096989832
ENSDART00000056927	egln1a	-0.373304639	-0.331810821	-0.548972472	-0.202283187
ENSDART00000056939	zgc:85858	-0.254903494	-0.357469282	-0.615277126	-0.499477333
ENSDART00000056963	stk25b	-0.084642109	-0.27353983	-0.240916991	-0.092979316
ENSDART00000056987	marcksl1a	0.199644536	0.480185443	0.561780108	0.295794028
ENSDART00000056996	sfrp5	0.077859934	0.01622227	-0.244583159	-1.042810978
ENSDART00000057095	si:dkey-24p1.1	-0.474380833	-0.459390323	-0.572876221	-0.481612744
ENSDART00000057124	tefa	-0.17959692	0.074135039	-0.330628318	-0.262330789
ENSDART00000057125	tefa	-0.190705988	0.094171259	-0.306461854	-0.301060608
ENSDART00000057159	cacnb1	1.304600132	2.011299334	2.307200837	1.559835487
ENSDART00000057174	arpc5a	0.695733745	0.614813351	0.637681436	0.535387536
ENSDART00000057258	slc12a5a	-0.776929597	-2.750292921	-0.618148385	-0.058229169
ENSDART00000057299	st6galnac5a	-0.846236683	-0.849009548	-0.253708637	-0.007082793
ENSDART00000057318	dusp8b	0.80659762	0.962100249	0.680537568	0.386784732
ENSDART00000057320	zgc:171579	-1.053157551	-0.622493525	-1.271148863	-1.344481218
ENSDART00000057325	cacng4a	-0.326351391	-0.28139538	-0.566189708	-0.15243738
ENSDART00000057369	igfbp5a	-0.503493701	-0.12557648	0.011432007	-0.035083178
ENSDART00000057377	arg2	-0.266719608	-0.373190344	-0.501724428	-0.206121282
ENSDART00000057422	pacsin1a	-0.590114235	-0.673330755	-0.518895915	-0.206589128
ENSDART00000057439	parla	-0.763945698	-0.13237506	-0.4980372	-0.373141189
ENSDART00000057458	CABZ01088149.1	0.207455616	0.356974308	0.107522223	0.159078208
ENSDART00000057519	zgc:194209	0.014343488	-0.12072846	-0.449908342	-0.453116382
ENSDART00000057553	ch25hl1.1	0.477648419	0.50568744	-0.255154291	-1.200576847
ENSDART00000057565	sdhaf4	-0.101594286	-0.172555564	-0.390218832	-0.338776854
ENSDART00000057584	slc1a4	0.291595567	1.233259249	1.405286425	1.089173123
ENSDART00000057638	hk1	-0.255062585	-0.314354089	-0.268977492	-0.110279892
ENSDART00000057644	lhx4	-0.267668007	-0.268543602	-0.190125613	-0.119751289
ENSDART00000057645	qsox1	0.191462539	0.115464338	-0.177011398	-0.490873756
ENSDART00000057689	bag3	-0.537315577	-0.216588912	-0.658528557	-0.795247472
ENSDART00000057710	ccdc85a	-0.175789149	-0.226891363	-0.323862394	-0.128238988
ENSDART00000057865	ier3ip1	-0.032004312	-0.136273649	-0.345681209	-0.364367077
ENSDART00000057910	nrgna	-0.338737739	-0.336045781	-0.223139332	0.191467698

ENSDART00000057918	si:ch211-147h1.4	0.058919084	-0.340722361	-0.680330981	-0.211102009
ENSDART00000057957	itm2cb	-0.410626815	-0.396999775	-0.657592832	-0.426965619
ENSDART00000058093	ldlrap1b	-0.019793679	-0.383158495	-0.468203159	-0.036502488
ENSDART00000058147	dync2li1	-0.035887703	-0.047873047	-0.361961236	-0.288662687
ENSDART00000058255	bbs5	-0.153890395	-0.351139568	-0.380364475	-0.12338798
ENSDART00000058258	gng5	0.433032497	0.476321934	0.436326472	0.078774872
ENSDART00000058277	znf800b	0.363600272	0.378301813	0.156623376	-0.047842936
ENSDART00000058324	rpz4	0.754354311	0.61115296	0.469864295	-0.17226809
ENSDART00000058339	ap3s2	-0.205243617	-0.191498501	-0.306412801	-0.176311273
ENSDART00000058346	c1qbp	0.293247509	0.073830319	0.050211764	0.059638874
ENSDART00000058370	arhgap32b	-0.032405011	0.136784851	0.285715572	0.090147098
ENSDART00000058384	gapdhs	-0.42821082	-0.374069856	-0.238011978	-0.087569823
ENSDART00000058415	zmp:0000001075	-0.428240629	-0.582010832	-0.289943093	-0.116590577
ENSDART00000058424	fam46ba	-0.297360056	-0.251077248	-0.176640513	-0.109784644
ENSDART00000058466	fgfbp2a	0.160228453	0.394614467	0.900155936	0.480238405
ENSDART00000058470	pik3r1	-0.272621258	-0.391826992	-0.565271638	-0.177820971
ENSDART00000058484	cnn3b	-0.281927631	-0.203420372	-0.230407516	-0.341119299
ENSDART00000058485	rai14	0.377402754	0.592516996	0.371359301	0.056635681
ENSDART00000058574		3.231975259	2.942133944	3.360676782	3.717330017
ENSDART00000058605	scpep1	0.659557613	0.259300703	0.157418507	-0.140654612
ENSDART00000058628	ccsapb	-0.109779777	-0.249497929	-0.321372328	-0.06107705
ENSDART00000058665	kif20bb	0.867797834	0.730592963	0.371647572	0.126469391
ENSDART00000058667	RDH13 (1 of many)	-0.138024992	-0.086121321	-0.690305733	-0.540517763
ENSDART00000058685	zfpm2a	-0.080421638	-0.032548615	0.512175645	0.757319343
ENSDART00000058706	fosaa	-1.574082598	-1.225695241	-1.240461281	-1.154909276
ENSDART00000058736	grm4	-0.475368296	-0.757934708	-0.299132614	0.099413724
ENSDART00000058737	cdc42l	0.49085446	0.397672444	0.316872224	-0.053147981
ENSDART00000058773	rgs16	-0.222100312	-0.210502322	-0.307458084	-0.014937374
ENSDART00000058774	havcr1	1.378230425	0.803624411	0.332422006	0.11017803
ENSDART00000058785	fam210ab	-0.074078069	-0.284264661	-0.891770658	-0.393378695
ENSDART00000058789	qdpra	0.319429593	0.277303758	-0.201013706	-0.346583237
ENSDART00000058829	scrt1b	-0.466509454	-0.564612343	-0.054278048	0.250358448
ENSDART00000058843	krcp	1.066051129	1.030166402	0.671597771	0.484235506
ENSDART00000058876	kpnb3	0.233004274	0.111806623	0.257484325	0.090678098
ENSDART00000058877	rap2ab	-0.786170126	-0.835369537	-0.021239814	0.42494962
ENSDART00000058936	scamp5b	-0.278094691	-0.317619737	-0.403892079	-0.134676622
ENSDART00000058955	arl6ip1	-0.023708036	-0.021671154	-0.307639798	-0.304733223
ENSDART00000058965	apoeb	-0.08755429	-0.343001804	-0.600108968	-0.53348823
ENSDART00000059001	C5AR1	0.862718576	0.211750471	0.205995335	0.011542683
ENSDART00000059003	rx2	-0.299942042	-0.32670005	-0.113463003	0.138106865
ENSDART00000059013	sec61b	0.564454151	0.292288284	0.352954607	0.073391744
ENSDART00000059179	nptxra	-0.572157626	-0.495039684	-0.356374901	-0.015079326
ENSDART00000059228	vil1	-0.324461409	-0.136026611	-0.08718702	0.103993243
ENSDART00000059369	phykpl	0.561796588	0.786946988	0.37392556	0.380622564
ENSDART00000059402	n6amt2	0.433448701	0.441036405	0.122223886	-0.017461279
ENSDART00000059425	CU570684.1	3.082478322	1.756945445	2.993403253	2.236315825
ENSDART00000059446	znf385b	-0.260906147	-0.315001382	-0.161772411	-0.110285684
ENSDART00000059476	psmg1	0.316645081	0.323743917	0.365248072	-0.009289235
ENSDART00000059478	lrrc32	-0.083635057	-0.196945688	-0.286292563	-0.79945825
ENSDART00000059489	prmt8b	-0.385132635	-0.375733065	-0.525036565	0.065426538
ENSDART00000059550	lrrc51	-1.204791639	-0.640574252	-1.049736423	-1.0433072

ENSDART00000059586	spegb	0.60633626	1.046592342	1.773590673	1.534849845
ENSDART00000059619	fkbp14	0.418122006	0.409098495	-0.000966617	0.084647379
ENSDART00000059631	BX936415.1	-0.988751505	-1.218737364	-0.343900834	0.242173034
ENSDART00000059667	wdr75	0.34735847	0.29669502	0.189255306	0.039773826
ENSDART00000059732	id1	-0.232839225	-0.281205165	-0.284745791	-0.436615034
ENSDART00000059756	ralba	0.050810042	0.323679093	0.529596851	0.259207353
ENSDART00000059841	si:ch211-257p13.3	-0.713606094	-0.675183925	0.07538087	0.401418787
ENSDART00000059869	adra2a	-1.042718178	-0.710015995	-0.232479367	0.220270649
ENSDART00000059955	ildr1b	0.550873694	0.893755801	0.988897575	0.559320969
ENSDART00000059984	deptor	-0.356805464	-0.334367455	-0.324987791	-0.320690594
ENSDART00000060001	pnp6	-0.139557872	-0.321502493	-0.386186078	-0.030509602
ENSDART00000060005	rpl32	0.348697332	0.358910178	0.175532175	0.008609589
ENSDART00000060015	chka	-0.144459192	-0.274568124	-0.456317122	-0.171918553
ENSDART00000060049	hspa13	-0.254728034	-0.327282661	-0.28629746	-0.272283731
ENSDART00000060051	fgf14	-0.493332703	-0.385375543	-0.154886482	-0.098910242
ENSDART00000060056	tpi1b	-0.330637537	-0.371028005	-0.31545087	-0.122811105
ENSDART00000060160	calb2a	-1.240040436	-1.237007367	-0.232768326	0.397108945
ENSDART00000060162	hspb1	0.08558143	-0.277493669	-0.503626412	-0.55244605
ENSDART00000060174	jagn1a	-0.416430339	-0.038483688	-0.018273384	-0.314381472
ENSDART00000060181	zgc:114174	0.760260394	1.239485919	1.043412191	0.428827471
ENSDART00000060184	chka	-0.101617548	-0.321325171	-0.420229119	-0.136541498
ENSDART00000060193	thap3	0.219009088	0.189913345	0.546438828	0.599142566
ENSDART00000060251	wdr18	0.292919064	0.371056299	0.311893069	0.087148131
ENSDART00000060255	blmh	0.318055181	0.435968503	0.441694486	0.152219233
ENSDART00000060259	wnt2	-0.011888956	0.307798324	-0.019010839	-1.701804028
ENSDART00000060302	ddb2	-0.150084227	0.300264829	-0.452176446	-0.393222799
ENSDART00000060304	dhrs13a.3	-0.00917183	0.036729289	-0.280144118	-0.407439325
ENSDART00000060321	RAMP1	-0.401526014	-0.370225897	-0.385056936	-0.044799362
ENSDART00000060356	dgkh	-0.649006381	-0.810232451	-0.629585196	-0.212393687
ENSDART00000060363	rpl4	0.373964359	0.396853604	0.212646351	-0.016195336
ENSDART00000060425	ftr53	0.363344395	0.103507635	1.747305289	-0.30385669
ENSDART00000060444	rps29	0.369322836	0.397792199	0.13443893	-0.022749507
ENSDART00000060532	zgc:110796	-0.139659209	-0.284517331	-0.33969202	-0.16468656
ENSDART00000060561	csdc2a	-0.084744858	0.609682067	0.574951056	0.285883083
ENSDART00000060576	myoz1a	-0.659756236	2.254391903	2.759775555	1.48758278
ENSDART00000060577	tmem33	0.319754096	0.307056024	0.247854771	0.133996431
ENSDART00000060625	lgi3	-0.698992604	-0.869112792	-0.10986795	0.409134057
ENSDART00000060702	rmdn3	-0.094193374	-0.184889649	-0.247613016	-0.093111552
ENSDART00000060710	adgrg11	2.568700304	0.61302127	2.440739962	2.102266876
ENSDART00000060714	atp6ap1a	-0.204397719	-0.266136404	-0.201574338	-0.008870212
ENSDART00000060718	taz	0.896308653	4.991849973	1.010024269	2.916260354
ENSDART00000060745	uba52	0.32327466	0.351581625	0.155851402	-0.01111286
ENSDART00000060765	nppb	4.501656565	4.501350371	1.812509246	-0.191856821
ENSDART00000060766	rab11a	0.179968247	0.479437298	0.674000894	0.468733557
ENSDART00000060773	taar12b	2.538886261	1.95415769	1.735717491	1.72259581
ENSDART00000060812	adcyap1b	1.704872196	2.272116909	1.908485885	0.966573225
ENSDART00000060865	rasal1b	1.120636523	0.769242819	0.458217171	0.392656742
ENSDART00000060898	mrps28	-0.371192118	-0.496136281	-0.011483012	-0.046106248
ENSDART00000060910	pimr138	0.646921071	1.324373168	1.239639246	0.98486272
ENSDART00000060919	qars	0.348349072	0.542908344	0.574420452	0.306598142
ENSDART00000060938	snx9b	0.471862308	0.584797813	0.101490869	-0.223444858

ENSDART00000060946	sgsm1b	-0.173061984	-0.355540157	-0.215401885	-0.063998625
ENSDART00000060949	zfpm1	0.286528567	0.561608339	0.375954127	0.217874539
ENSDART00000061000	bbs2	-0.085680202	-0.169473458	-0.253361227	-0.025608691
ENSDART00000061001	gnb2l1	0.316385721	0.345297586	0.431111492	0.12899342
ENSDART00000061007	mt2	-0.024087588	-0.437804286	-0.890429121	-0.686083783
ENSDART00000061106	bhlhe41	0.043056173	-0.423617996	-0.754959095	-0.246095493
ENSDART00000061117	rrbp1b	-0.094086695	0.04423759	-0.324207933	-0.60750175
ENSDART00000061141	cep85l	-0.248281327	-0.136664261	0.313311334	0.132594592
ENSDART00000061149	TUBB4A (1 of many)	0.971780187	0.499035917	0.545475118	0.962590028
ENSDART00000061156	raph1a	0.024271858	0.223289034	0.290402674	0.224778316
ENSDART00000061196		0.755433358	1.498415514	1.365853078	0.846252469
ENSDART00000061261	cx43	-0.064274202	-0.132171143	-0.392515504	-0.502400185
ENSDART00000061265	rnf141	-0.12379903	-0.215585747	-0.342006011	-0.212187704
ENSDART00000061417	si:ch211-245h14.1	0.537591116	0.459433616	1.387404638	-0.014689392
ENSDART00000061435	hsbp1b	-0.02310974	-0.083751559	-0.317854229	-0.256774332
ENSDART00000061470	mtss1la	-0.342228031	0.053961203	0.113929451	0.018495568
ENSDART00000061497	OLFM4 (1 of many)	-0.269705239	-0.443597761	-0.460075072	-0.13609298
ENSDART00000061499	cxcr4b	1.134597457	1.176774484	0.975229504	0.34365956
ENSDART00000061523	il17a/f3	0.228114616	0.421937628	0.621201213	0.769032572
ENSDART00000061555	si:ch211-63o20.7	1.506216579	0.813490393	0.865397342	0.102228691
ENSDART00000061633	zgc:171971	-0.243215545	-0.195809688	-0.289637739	-0.171793375
ENSDART00000061653	pebp1	-0.450366142	-0.339729586	-0.205596712	0.038256249
ENSDART00000061736	si:dkey-4e7.3	0.80946555	0.660586871	0.14634027	0.03058403
ENSDART00000061745	inpp4ab	-0.337306716	-0.94773917	-0.831022148	-0.394471236
ENSDART00000061886	sema3ab	-0.067887191	-0.37217703	-0.138233187	0.034639157
ENSDART00000061926	stx11b.2	3.447906519	2.671571656	2.785952697	1.680276674
ENSDART00000061955	myl13	0.400602979	3.991209963	4.290518336	2.830575983
ENSDART00000062003	efnb3b	-0.314139927	-0.178367777	0.04557076	0.250001858
ENSDART00000062066	si:dkey-177p2.6	-0.250914133	-0.251912097	-0.422547135	-0.291399654
ENSDART00000062073	BCR (1 of many)	0.11359461	-0.080987241	-0.284063713	-0.288348423
ENSDART00000062143	zgc:77650	-0.109887255	-0.133378465	-0.376117658	-0.331935654
ENSDART00000062150	zgc:77752	-0.401367403	-0.150322453	-0.162634546	-0.096457711
ENSDART00000062181	RALGDS	-0.386705371	-0.180287867	-0.473841885	-0.479084169
ENSDART00000062185	rab40b	-0.04766047	0.014828604	-0.304246935	-0.082657522
ENSDART00000062220	gstt1a	1.233095877	1.492566267	1.464760691	0.555034442
ENSDART00000062229	p2rx7	0.280693894	0.946609864	0.955265868	0.573409065
ENSDART00000062257	slc39a1	0.740781667	0.522946282	0.350404642	0.245154327
ENSDART00000062360	nup205	0.380838378	0.174863772	0.543980624	0.366347355
ENSDART00000062383	ywhaqa	-0.013489826	0.266644821	0.309888274	0.200982907
ENSDART00000062402	tpd52l1	-0.180007689	-0.341056198	-0.772074089	-0.368132956
ENSDART00000062403	tmem9	-0.368514764	-0.231056659	-0.311288279	-0.066743785
ENSDART00000062518	gstr	-0.173225499	-0.241367808	-0.232576695	-0.339957813
ENSDART00000062551	cyp51	0.018772193	0.850105191	1.080881624	0.554137375
ENSDART00000062552	wtap	-0.338047264	-0.227200913	-0.06961558	-0.130824995
ENSDART00000062556	sod2	-0.183508685	-0.287491323	-0.424543766	-0.237833299
ENSDART00000062560	zgc:77784	0.130172136	0.171187774	0.614861949	0.368542156
ENSDART00000062576	thyn1	0.902996379	0.741810201	0.792519989	0.219218083
ENSDART00000062587	klf2a	0.563534905	1.202186524	1.977026531	0.968508443
ENSDART00000062603	cadm1b	-0.182938767	-0.538377364	-0.267052947	-0.164493851
ENSDART00000062633	s1pr1	-0.300458433	-0.0458986	-0.017104561	-0.199608488
ENSDART00000062671	tuba8l	1.253108099	0.545680562	0.42270127	-0.336104805

ENSDART00000062697	gfra2a	-0.450482933	-0.554254218	-0.069449306	-0.130685043
ENSDART00000062704	plaa	0.222653649	0.333324552	0.491597491	0.251933193
ENSDART00000062727	stx6	-0.088840172	-0.113781331	-0.247667952	-0.166950515
ENSDART00000062736	coasy	-0.31852882	-0.407402551	-0.404610538	-0.370618301
ENSDART00000062761	cnstb	-0.361465575	-0.117860324	0.271149791	0.383194937
ENSDART00000062845	mmp9	2.08287012	1.917473556	1.298502519	-0.06470518
ENSDART00000062850	agps	0.085764498	0.319290218	0.455652831	0.366743535
ENSDART00000062874	atp1b3b	-0.415562805	-0.319287363	-0.078895256	0.137689897
ENSDART00000062887	disp2	-0.441631902	-0.296469901	0.444242177	0.781646932
ENSDART00000062908	rpl7l1	0.414752526	0.336818546	0.137726222	-0.109457063
ENSDART00000062931	abracl	0.878200021	0.73529721	0.710676136	0.289919909
ENSDART00000062935	heca	-0.371152131	-0.569989768	-0.691744059	-0.51577268
ENSDART00000062983	rpl10a	0.388498934	0.402298329	0.17374506	-0.059011317
ENSDART00000063008	mfng	0.785568878	0.314558937	0.02546364	-0.36422945
ENSDART00000063071	dgcr2	-0.097084868	-0.122785849	-0.277264164	-0.131322908
ENSDART00000063081	cyp2ad3	0.42839208	0.705423845	0.252750259	0.171232166
ENSDART00000063107	cyp2p7	0.910849098	1.165275545	0.616791402	-0.023701278
ENSDART00000063151	napga	0.319825484	0.237345685	0.317095517	0.164414652
ENSDART00000063251	ctsz	1.117383701	0.530773826	0.448185866	0.142841944
ENSDART00000063337	cdca8	2.552618125	1.809029687	2.257850367	1.156797598
ENSDART00000063357	ccnb1	1.564145903	1.350801576	0.91228963	0.49209964
ENSDART00000063359	ucp3	0.449516929	0.816201403	1.070983817	0.155240874
ENSDART00000063418	nsun5	0.551107756	0.480901258	0.442024494	0.196344317
ENSDART00000063478	nfil3-2	-0.375816946	0.376110137	0.458578211	0.007201456
ENSDART00000063551	ppm1e	-0.4375512	-0.67599947	-0.28655858	-0.160579724
ENSDART00000063564	nmu	-0.490206998	-0.305049777	-0.445855788	-0.16147485
ENSDART00000063625	gpx3	0.093413749	0.050792899	-0.431389541	-0.288399492
ENSDART00000063648	nck2b	-0.186136113	-0.294499599	0.095653682	0.163593769
ENSDART00000063703	si:dkey-71h2.2	-0.547517928	-0.696070326	-0.446988451	-0.222080286
ENSDART00000063704	crip3	-0.295432006	-0.264072284	-0.393391872	-0.087537117
ENSDART00000063706	fndc4a	-0.898991439	-1.194204845	-0.829294946	-0.104966589
ENSDART00000063714	rapgef6	-0.363328977	-0.303671651	0.266966568	0.457046047
ENSDART00000063725	xkr6b	-0.377201403	-0.64365216	0.065079126	-0.045638513
ENSDART00000063764	si:dkey-5n18.1	1.420288455	0.932190706	0.716992196	-0.355341421
ENSDART00000063779	efhd1	-0.527832136	-0.620416333	-0.244685479	-0.230757866
ENSDART00000063781	gpr55a	3.100202316	2.329994693	2.002857636	2.112952219
ENSDART00000063783	itm2ca	-0.362486731	-0.282712517	-0.07258841	0.153576522
ENSDART00000063786	cab39	-0.24368294	-0.258313334	-0.267466597	-0.001027932
ENSDART00000063804	wu:fj39g12	-1.205265642	-1.354971991	-1.517691105	-1.318916155
ENSDART00000063816	kcnk3a	-0.460164643	-0.659361375	-0.48206205	-0.175491486
ENSDART00000063817	ndufb11	-0.172664849	-0.275426505	-0.329160032	-0.102461652
ENSDART00000063825	sprn	-0.451586162	-0.272184034	-0.06406637	0.092302795
ENSDART00000063832	rbbp8	-0.238022634	-0.227071096	0.03579094	-0.764989117
ENSDART00000063835	otx5	-0.207695123	-0.226833273	-0.417236259	-0.126675877
ENSDART00000063870	rpl11	0.4298733	0.509956948	0.251365855	0.019450433
ENSDART00000063874	vamp4	0.706049434	0.688342777	0.412447235	0.06985511
ENSDART00000063912	jun	1.166146477	1.429328618	1.29995447	0.480560375
ENSDART00000063938	mast1a	0.148358744	0.555318149	1.032634027	1.008672267
ENSDART00000063944	tmem30ab	-0.052321591	-0.186777496	-0.351757869	-0.167369044
ENSDART00000063950	psmc1b	0.214901771	0.338782243	0.27453656	0.103637979
ENSDART00000063953	zgc:65997	0.11562047	0.484476273	-0.11592626	-0.024456549

ENSDART00000064012	ca4a	-0.265180291	-0.250252223	-0.144355238	0.018302985
ENSDART00000064017	rapgef1a	0.181570205	0.344922552	0.212193731	0.084768062
ENSDART00000064032	eif4ebp1	0.426193835	0.395196044	0.352513449	0.114800609
ENSDART00000064067	ehbp1	0.439358276	0.535882532	0.636938582	0.35957066
ENSDART00000064111	faub	0.024926044	-0.061766338	-0.402186419	-0.29870419
ENSDART00000064112	glrx5	-0.15774683	-0.2561987	-0.313570229	-0.138985623
ENSDART00000064113	abt1	0.598506286	0.454995795	0.386684419	0.116646594
ENSDART00000064130	BEGAIN	-0.586123435	-0.609324776	-0.400772811	-0.09647247
ENSDART00000064241	nrxn3a	-0.593329637	-0.86602502	-0.45366796	-0.193651725
ENSDART00000064311	arhgdia	0.49745023	0.520698759	0.529544756	0.127344369
ENSDART00000064375	tmem244	-0.163876969	-0.255273585	-0.331911996	-0.04221405
ENSDART00000064376	sod1	-0.248477917	-0.212122084	-0.427881094	-0.390078569
ENSDART00000064403	nptnb	-0.282191976	-0.43563203	-0.351497687	-0.147485108
ENSDART00000064462	psma6l	0.604857346	0.425092318	0.840213456	-0.035624292
ENSDART00000064468	crmp1	-0.244499065	-0.053767261	0.158838362	0.344619203
ENSDART00000064509	stmn4l	2.57043941	3.576220537	3.295095816	2.31833518
ENSDART00000064511	il17a/f1	1.319538931	1.072672417	0.769667562	1.083526716
ENSDART00000064581	kcnip3b	-1.323762559	-2.171156238	-0.883181569	0.0425137
ENSDART00000064657	stx11a	1.187636483	0.704469765	0.33181163	-0.135694961
ENSDART00000064662	rassf2b	-0.5330742	-0.904631137	-0.740838414	-0.317057253
ENSDART00000064666	prnpb	1.397742128	1.699664466	1.642053833	0.642245778
ENSDART00000064672		-1.010331454	-0.756315191	-0.126164495	0.278123254
ENSDART00000064700	fuca2	0.436466876	0.137195427	-0.005101419	-0.171105064
ENSDART00000064738	atpif1b	-0.12884804	-0.286001583	-0.530012586	-0.266598201
ENSDART00000064739	rpl13a	0.411158528	0.410041852	0.123606951	-0.078504495
ENSDART00000064789	txn	1.421742139	1.973125	1.39538042	0.274434823
ENSDART00000064798	aspn	0.856747155	1.499777714	1.699781764	0.582997672
ENSDART00000064805	cenpp	0.292664259	0.48497191	0.107661064	-0.056951786
ENSDART00000064826	mov10a	0.329642907	0.141820564	0.774895549	-0.030140661
ENSDART00000064833	mafaa	-0.957052225	-1.143383392	-0.379735148	0.067354966
ENSDART00000064842	padi2	-0.110475283	-0.200123181	-0.394420172	-0.195593685
ENSDART00000064860	rbms1a	-0.125610759	0.088275426	0.5443086	0.313591986
ENSDART00000064866	prkab1a	-0.370484412	-0.353103565	-0.349388814	-0.328698623
ENSDART00000064878	gxylt2	-0.088947943	-0.180342387	-0.380560197	-0.388684923
ENSDART00000064902	ssbp4	-0.349245142	-0.241492171	0.147485085	0.214650497
ENSDART00000064913	fto	-0.098234146	-0.428383355	-0.257568512	-0.239748235
ENSDART00000064968	rasgef1bb	0.10103678	0.334291276	0.155368522	-0.094945106
ENSDART00000065057	itgb7	1.200094562	0.885337917	0.497721229	0.006866552
ENSDART00000065097	dpysl3	-0.247117182	0.32883927	0.696704704	0.442557487
ENSDART00000065132	zgc:171740	-0.488981736	-0.378281723	0.139236952	0.2855891
ENSDART00000065143	unc119b	-0.086346577	-0.282642438	-0.651558775	-0.265879719
ENSDART00000065159	zgc:158291	-1.165646756	-0.784542684	0.443310678	0.751149417
ENSDART00000065183	cldn2	0.624880574	0.284479914	-0.382379938	-0.379228488
ENSDART00000065208	nop16	0.539941841	0.465464987	0.330311237	0.01578578
ENSDART00000065228	csmd1a	-0.138553555	-0.429039185	-0.109478646	0.118637902
ENSDART00000065264	cdca5	0.52809349	0.795495163	0.547641137	0.341339334
ENSDART00000065337	kif20a	1.518648505	1.334799108	0.992824147	0.572892998
ENSDART00000065356	desmb	0.665915236	0.890084527	0.800282347	0.503228927
ENSDART00000065361	etv5b	-0.645686026	-0.347502384	-0.293939032	-0.317696675
ENSDART00000065366	st6gal1	-0.520593433	-0.711298232	-0.60540202	-0.072634403
ENSDART00000065372	kcnj3b	-0.605628976	-0.778191304	-0.104344748	0.360074992

ENSDART00000065373	eef1b2	0.250630086	0.293877839	0.677481758	0.365569434
ENSDART00000065380	camk1ga	-0.057459289	-0.334433278	-0.359469575	-0.058443268
ENSDART00000065397	fkbp2	0.018660881	-0.129009813	-0.324870267	-0.155859325
ENSDART00000065420	pacs1a	-0.146340142	-0.310676632	-0.118017371	-0.142794888
ENSDART00000065467	dedd1	-0.00326722	-0.100306912	-0.35251663	-0.041277003
ENSDART00000065495	emp2	0.342641107	0.530182619	0.397727804	0.205643264
ENSDART00000065500	abcc4	0.484659612	0.306343643	-0.287697763	-0.373355455
ENSDART00000065507	plppr2b	-0.22717931	-0.353326082	-0.20008884	0.028481334
ENSDART00000065551	zak	1.359635484	2.260289477	1.621487921	0.736191985
ENSDART00000065563	ccdc90b	0.430098539	0.770932327	0.278586215	0.111905478
ENSDART00000065567	guca1d	-0.53307102	-0.365098729	-0.535522544	-0.275955769
ENSDART00000065599	cadm1a	-0.54681119	-0.778847768	-0.187580903	0.116530415
ENSDART00000065600	sc5d	-0.12560715	0.619273704	0.802648489	0.437253012
ENSDART00000065664	dusp4	-0.466715295	-0.312689089	-0.30035434	-0.446313481
ENSDART00000065674	fybb	0.68375018	0.334314613	0.079534024	-0.242430054
ENSDART00000065728	nrsn1	0.207141455	0.540557286	0.398847193	0.186515655
ENSDART00000065755	gpn3	-0.172157475	-0.155442383	-0.381057864	-0.19097226
ENSDART00000065805	tspan10	0.90782785	0.406843364	-0.022479662	-0.576522773
ENSDART00000065807	kctd13	0.261042411	0.352189581	0.50608049	0.336398415
ENSDART00000065817	pou5f3	1.443769101	1.494882152	0.928756649	0.660724256
ENSDART00000065818	fut7	0.972435403	0.738351	0.320621995	-0.576532021
ENSDART00000065853	dhrs3b	-0.214859587	-0.196724114	-0.346466706	-0.463603398
ENSDART00000065929	hs6st3b	-0.878348455	-0.942691234	-0.255367675	0.253670478
ENSDART00000066177	tuba2	-0.341980556	0.290211936	0.695639518	0.704284087
ENSDART00000066192	glra2	-0.5075286	-0.700662657	-0.376893165	0.421840665
ENSDART00000066198	rab9a	0.076150809	0.228460008	0.322859875	0.206383991
ENSDART00000066230	ARL3 (1 of many)	-0.707746447	-0.419158458	-0.489675877	-0.206340441
ENSDART00000066256	vti1a	-0.147124368	-0.216073091	-0.331785573	-0.195609352
ENSDART00000066259	kcnk1a	-0.289802216	-0.227947241	-0.67177	-0.392395516
ENSDART00000066269	arl4d	-0.431926082	-0.429953919	-0.294676093	-0.224713874
ENSDART00000066288	spata20	-0.009504348	-0.162892766	-0.35319246	-0.173028944
ENSDART00000066290	UTS2R	-0.720663714	-0.982454784	-0.512077429	-0.545080013
ENSDART00000066294	cdk5r1b	-0.511562763	-0.495240798	0.085364893	0.188592346
ENSDART00000066372	id4	-0.719733282	-0.518590484	0.441847928	0.655214602
ENSDART00000066373	vdac1	-0.316380085	-0.269111014	-0.266508034	-0.031245506
ENSDART00000066380	ca7	-0.236035328	-0.017625204	-0.765902599	-0.026455411
ENSDART00000066382	aqp8a.1	-0.164652843	-0.325293072	-0.493634593	-0.109118528
ENSDART00000066385	hbz	0.819612283	1.064127771	1.5494854	0.440895582
ENSDART00000066386	shisa9a	-0.462438855	-0.347473048	-0.310604934	-0.078066291
ENSDART00000066389	tmem184ba	-0.204152722	-0.333415893	-0.147371729	-0.097737372
ENSDART00000066391	csnk1e	0.01172114	0.595315231	0.792117229	0.433182971
ENSDART00000066411	dlgap5	1.041688171	0.895823709	0.540002804	0.33349173
ENSDART00000066471	adam8b	1.018895271	1.190253553	1.122506289	0.517972783
ENSDART00000066477	dkk1b	-0.674554346	-1.187574562	-0.676297256	-1.09065776
ENSDART00000066506	cox6b1	0.122510297	0.115648463	0.536625345	0.628910558
ENSDART00000066590	rdh12l	0.806755691	0.842269039	0.848807771	0.120108035
ENSDART00000066623	st8sia3	-0.182339116	-0.349623634	-0.081530336	-0.065322773
ENSDART00000066625	smpx	0.108625243	0.797810146	1.548262684	1.15135764
ENSDART00000066655	mybl1	-0.255810306	-0.487313899	-0.72749049	-0.668391364
ENSDART00000066703	rdh10a	-0.287660525	-0.158056129	-0.356819377	-0.262167994
ENSDART00000066733	gpr22b	-0.9723222	-1.512791135	-0.598895258	-0.577277225

ENSDART00000066760	cct5	0.325276039	0.436851681	0.379245954	0.163513221
ENSDART00000066765	bmi1a	3.316489868	2.947911885	2.359801458	2.244842944
ENSDART00000066778	acad11	0.796145744	0.566674022	0.216041695	-0.190350168
ENSDART00000066784	fam49bb	0.049871707	-0.095277519	-0.294897796	-0.151467176
ENSDART00000066794	otulina	0.468513579	0.440716075	0.351949179	0.239625569
ENSDART00000066839	slc35g2b	-0.671846523	-0.750415444	-0.309110396	0.04424778
ENSDART00000066895	rassf8b	-0.275713104	-0.148423362	-0.148246743	-0.166873018
ENSDART00000066896	syt1a	-0.255336633	-0.349305824	-0.330024338	-0.178637525
ENSDART00000066963	atp6v1f	-0.089791813	-0.169571399	-0.304106913	-0.234245815
ENSDART00000066975	impdh1b	0.195914466	0.339716801	0.56808711	0.477613078
ENSDART00000066997	dram1	0.801491354	0.536766251	0.146394385	-0.606440034
ENSDART00000066999	ccdc53	0.300412133	0.290286375	0.140411091	0.00256916
ENSDART00000067005	bcat1	-0.064708558	-0.22197643	-0.301840187	-0.097404482
ENSDART00000067053	vta1	0.2430424	0.457630564	0.367763165	0.149685321
ENSDART00000067059	fam19a5b	-0.282093441	-0.382553277	-0.381033174	-0.505286252
ENSDART00000067066	parp6b	-0.443166413	0.101161009	0.402103535	0.516387314
ENSDART00000067078	plekhg5a	-0.147306721	-0.274518319	-0.555343847	-0.255866929
ENSDART00000067082	clta	-0.242881724	-0.298079508	-0.220247727	-0.021275929
ENSDART00000067147	ANKRD50	0.122102324	0.471943767	0.546358337	0.270036712
ENSDART00000067168	pdzrn4	0.330865082	1.464493171	1.433744497	0.793719225
ENSDART00000067190	tspan9b	-0.605960388	-0.476716761	-0.251844268	-0.266924971
ENSDART00000067193	adm2a	0.4356689	0.184728608	-0.002686414	-1.106691874
ENSDART00000067211	gpr37l1b	-0.390917116	-0.36533905	-0.206436073	-0.113142613
ENSDART00000067239	guca1g	-0.979413995	-1.042352228	-1.597708164	-1.275542792
ENSDART00000067258	syt10	-0.290010761	-3.233857576	-0.084108756	-0.40009033
ENSDART00000067312	si:dkey-106n21.1	0.584969753	0.102146183	-0.031661382	0.069325035
ENSDART00000067324	mfge8b	-0.041376155	-0.13672935	-0.391535511	-0.353629136
ENSDART00000067327	abhd2b	0.159640353	0.698443956	0.611964225	0.750739323
ENSDART00000067362	cart2	-0.749355761	-0.384853037	-0.719287733	-0.625188951
ENSDART00000067427	yeats4	0.136322543	0.363843408	0.318943581	0.037728114
ENSDART00000067434	nudt4b	-0.510387585	-0.468486348	-0.329729048	-0.412952232
ENSDART00000067446	slc38a4	-0.251589327	-0.331078479	-0.606665517	-0.538910759
ENSDART00000067448	acat1	-0.217922513	-0.251881532	-0.396031362	-0.209577052
ENSDART00000067461	si:ch211-152c2.3	-0.474479692	-0.343258906	-0.010850784	0.158497108
ENSDART00000067478	pkp3a	0.648319477	0.895152082	2.33162898	0.940793267
ENSDART00000067500	si:dkey-280e21.3	0.421024272	0.822626751	0.979586691	0.632877181
ENSDART00000067510	crabp1a	-0.186487239	-0.41656224	-0.606281006	-0.420918895
ENSDART00000067512	psma4	0.210284594	0.22504456	0.324917099	0.119970251
ENSDART00000067514	rbpms2a	-1.175610868	-1.156235961	0.045281157	0.593799851
ENSDART00000067531	syn2a	-0.50370548	-0.414636803	-0.227904446	0.132370635
ENSDART00000067537	elovl6l	0.404046488	1.053095893	1.924314262	1.109723769
ENSDART00000067542	kcnk10b	2.685681832	3.478119742	3.471523284	2.49042133
ENSDART00000067594	cst14b.1	1.003408293	1.132156972	0.601894798	0.102871452
ENSDART00000067599	angptl2a	1.671461404	1.166310219	0.762115684	0.407368061
ENSDART00000067637	dstyk	-0.298992266	-0.18939574	-0.18546883	-0.220617575
ENSDART00000067678	zgc:110339	0.949255799	0.562708107	0.125395868	-0.585210787
ENSDART00000067733	zgc:77838	-0.192172111	-0.379046905	-0.170230593	-0.131538824
ENSDART00000067741	cacng6b	-2.498204907	-1.377375045	-1.107680653	-1.191335449
ENSDART00000067762	MYO1D	-0.207118869	-0.403579734	-0.286614596	-0.314212256
ENSDART00000067764	stk17a	-0.185636748	-0.198053553	-0.515379898	-0.253038095
ENSDART00000067776	rab10	0.291742266	0.421774457	0.459760968	0.189497347

ENSDART00000073405	zgc:173552	0.695539535	0.568218274	1.113411469	0.383673074
ENSDART00000073452	si:ch211-113a14.12	0.801955974	0.847093718	0.855214481	0.503313711
ENSDART00000073462	rplp0	0.408607572	0.593255771	0.47280039	0.165347026
ENSDART00000073500	ptprz1a	-0.320567664	-0.32912405	-0.255947625	-0.47902768
ENSDART00000073511	hyal6	-0.14022603	-0.303368807	-0.413603406	-0.552708783
ENSDART00000073564	tes	0.818084026	1.011033966	0.902633694	0.089277909
ENSDART00000073583	islr2	0.313523936	1.12325114	1.332162091	1.179316934
ENSDART00000073588	kcnj11	-0.465815764	-0.671487298	-0.425150531	-0.293255153
ENSDART00000073617	opn4xa	-0.377358091	-0.474333233	-0.655013246	-0.269116892
ENSDART00000073634	slc30a5	-0.005479497	-0.126675199	-0.24346742	-0.137800332
ENSDART00000073694	smu1b	-0.023572641	-0.696487436	-0.934479138	-0.393819626
ENSDART00000073705	abcf1	0.284347738	0.522430424	0.46970431	0.327069748
ENSDART00000073726	cav2	-0.135895339	0.080426407	-0.366803726	-0.546607126
ENSDART00000073735	rrad	0.475668516	0.908053908	0.772751604	0.279273357
ENSDART00000073846	si:ch211-122f10.4	0.620706192	0.26696454	0.219379439	-0.011733355
ENSDART00000073861	gabarapb	-0.042475311	-0.021227012	-0.297506188	-0.271766658
ENSDART00000073903	crtc3	-0.232649231	-0.295606215	-0.176321155	-0.096709861
ENSDART00000073919	kcnc1b	-0.758440974	-0.784989596	-0.33092052	0.322314215
ENSDART00000073932		0.121194935	-0.376988949	-0.788016576	-0.265683315
ENSDART00000073936	acvr1bb	-0.291123202	-0.390021531	-0.349875902	-0.253586872
ENSDART00000073950	olfm1a	-0.852827208	-0.587563633	0.013637392	0.435090193
ENSDART00000073970	uap1	0.430311379	0.939111864	0.868311508	0.206630788
ENSDART00000073981	eif2s1b	0.229006291	0.299741922	0.510349465	0.185458194
ENSDART00000073985	rbfox2	-0.063885036	0.156691707	0.429046907	0.332565675
ENSDART00000074010	ubald1b	0.486188172	0.523878559	0.246068485	0.014830353
ENSDART00000074036	rcvrna	-0.553376367	-0.06531313	-0.11758335	-0.07626787
ENSDART00000074070	aff2	-0.074830038	-0.283904996	-0.159789643	-0.147467133
ENSDART00000074099	cabp2b	-0.664167129	-0.641383476	-0.676380934	-0.590070868
ENSDART00000074100	osgn1	0.255332714	0.013078065	-0.613186841	-0.487873824
ENSDART00000074117	aspa	-0.032383232	-0.304124913	-0.397629458	-0.358898864
ENSDART00000074161	slc4a2b	0.481685785	0.057728735	-0.200942778	-0.247347753
ENSDART00000074212	scrib	0.155467238	0.226272134	0.329350211	0.126740521
ENSDART00000074317	GSK3B (1 of many)	0.32408852	0.570538304	0.615748631	0.310967098
ENSDART00000074362	pcdh18b	-0.440881831	-0.447646282	-0.227758863	0.176890552
ENSDART00000074380	tsga10	-0.358572407	-0.32335115	-0.582555062	-0.41305108
ENSDART00000074384	stx4	0.365157378	0.335603422	0.150714004	-0.050227712
ENSDART00000074400	tia1	0.181056891	0.310121029	0.46787956	0.221267203
ENSDART00000074438	cenpi	0.547415003	0.708397465	0.401862169	0.054393549
ENSDART00000074458	ptpmt1	-0.138560892	-0.279060294	-0.535667209	-0.518833125
ENSDART00000074543	hs3st4	-0.453389419	-0.572006435	-0.310659668	0.002760951
ENSDART00000074609	suclg1	-0.204210404	-0.293159601	-0.171956898	0.002164837
ENSDART00000074678	chrnb3a	-0.683036397	-0.585209438	-0.069659384	0.207417172
ENSDART00000074685	glrbb	-0.387255204	-0.421576892	-0.370807969	-0.094075713
ENSDART00000074689	eif5b	0.118742051	0.204941132	0.292461428	0.069777195
ENSDART00000074698	opn3	-0.589287895	-0.235981184	0.224725485	-0.032745726
ENSDART00000074718	spire1b	-0.446651583	-0.478750814	-0.155393912	-0.07838085
ENSDART00000074786	ctso	0.33157854	0.154941794	-0.070268612	-0.012066877
ENSDART00000074838	kcnk3b	-0.808453975	-0.995140212	-0.30053552	-0.689829672
ENSDART00000074924	mbnl1	0.32889023	0.277718787	0.642145523	0.211541009
ENSDART00000074936	gabrr2a	-0.375623148	-0.534637844	-0.431940406	-0.359950744
ENSDART00000074950	slc25a28	-0.022495638	-0.038026223	-0.243484898	-0.120098609

ENSDART00000074959	slc35f1	-0.294905966	-0.396956867	-0.318533983	-0.137876179
ENSDART00000074960	cd22	0.815723271	0.646940316	0.539427268	-0.232061943
ENSDART00000074979	rnft2	0.06664962	0.449659345	0.588779493	0.279626887
ENSDART00000074997	CU302436.3	-0.857583363	-0.990723788	-0.187854306	0.199234775
ENSDART00000075009	eif2s2	0.324008789	0.419532633	0.338652657	0.07614456
ENSDART00000075028	rps11	0.42100484	0.441088802	0.128015907	-0.080449898
ENSDART00000075039	gosr2	0.009731507	0.24829488	0.212269525	-0.090945666
ENSDART00000075070	hsf2	-0.066635095	-0.276496416	-0.474097609	-0.186498896
ENSDART00000075092	pkib	-0.08506216	-0.264513554	-0.401500984	-0.212860417
ENSDART00000075112	clvs2	0.165980729	0.346379383	0.75563827	0.535875063
ENSDART00000075116	si:dkey-148a17.6	0.841894804	0.387365318	0.191323415	-0.035438886
ENSDART00000075123	pcp4a	-0.598752351	-0.632528063	-0.40265776	-0.022235336
ENSDART00000075129	lrrc47	0.095574868	0.246973338	0.327055852	0.135309168
ENSDART00000075150	bmp4	-0.321654174	-0.192558055	-0.354985644	-0.520317573
ENSDART00000075172	cttnbp2nla	-0.399030632	-0.078691774	-0.193018514	-0.275655386
ENSDART00000075184	snx1a	0.267188596	0.126701463	0.120486891	-0.033463537
ENSDART00000075187	pdzd11	1.031277997	1.70516213	1.574957324	1.135180778
ENSDART00000075223	cox7a2a	-0.206130471	-0.297851957	-0.422850723	-0.097231315
ENSDART00000075260	inab	-0.558694355	0.275313434	1.165237079	1.111612471
ENSDART00000075262	cad	0.41294438	0.156970425	0.074105835	0.04769287
ENSDART00000075278	atp1b4	-0.758712842	-0.43098592	-0.240547748	-0.077197984
ENSDART00000075286	slc2a15b	1.023992215	0.671159354	0.170031714	-0.73573625
ENSDART00000075299	zgc:153911	1.082638325	2.093301655	1.816780233	1.137984438
ENSDART00000075320	nampta	-0.083138724	-0.057980927	0.268248216	-0.062095237
ENSDART00000075331	insm1b	0.180818368	0.558805202	0.852549514	0.856981121
ENSDART00000075340	eef1a1b	-0.66677551	-0.473919347	-0.344351157	0.106841151
ENSDART00000075351	zgc:112285	0.930923442	1.110746674	0.609701543	0.79082813
ENSDART00000075398	cilp	1.006018993	0.43809517	0.394851989	0.084259634
ENSDART00000075400	eif3jb	0.284407943	0.11040548	-0.086691777	-0.1231685
ENSDART00000075421	sord	-0.008165085	-0.251228216	-0.437538687	-0.226988953
ENSDART00000075465	mylpfa	-0.627780558	3.258441537	4.096966032	2.542734101
ENSDART00000075491	pop5	0.444559136	0.7451635	0.571282976	0.537107281
ENSDART00000075495	rpl23	0.390673252	0.341184618	0.264108815	-0.005521244
ENSDART00000075499	si:dkey-283b1.7	-0.753135255	-0.256638623	-0.866666345	-0.691414461
ENSDART00000075510	ngb	-0.534714702	-0.310443771	-0.511019459	-0.235764625
ENSDART00000075513	aqp9b	-0.181865878	-0.358464178	-0.54013493	-0.322928595
ENSDART00000075519	aldh1a2	-0.136704985	-0.161016939	-0.528203419	-0.420522124
ENSDART00000075551	adhfe1	0.155892684	-0.065430004	-0.440766413	-0.267456669
ENSDART00000075663	cracr2b	0.080855847	0.057965052	-0.927246946	-0.842015409
ENSDART00000075743	eprs	0.181617497	0.208060281	0.357036503	0.248273691
ENSDART00000075749	ppp2r2ca	-0.492014929	-0.268923214	0.328067214	0.425314748
ENSDART00000075808	apbb3	-0.025817957	0.161567226	0.465828121	0.45985711
ENSDART00000075889	trub1	0.429771719	0.538562347	0.550227428	0.249034481
ENSDART00000075902	klhl43	1.602362948	3.319039146	3.627754288	3.174379945
ENSDART00000075903	crlf3	-0.351458679	-0.119437595	0.002747455	-0.299343819
ENSDART00000075918	pcmtd2	-0.427264647	-0.257111837	-0.149394946	0.062644737
ENSDART00000075927	rad50	0.535614901	0.654138999	0.420227838	0.362493118
ENSDART00000075935	vtnb	-0.377853158	-0.359520721	-0.399371834	-0.233041975
ENSDART00000075940	mtnr1ba	-0.423445838	-1.139676296	-0.754844062	-0.657118397
ENSDART00000075974	ism2b	-0.752954546	-0.582959852	-0.548810475	-0.377147389
ENSDART00000075993	crtc1b	-0.280354395	-0.577500657	-0.194615785	-0.207111517

ENSDART00000076004	tmem62	-0.08960985	0.130455024	0.308433272	0.432472028
ENSDART00000076009	hadhab	0.274869752	0.225585159	0.168828199	0.114580965
ENSDART00000076030	fbl	0.33280558	0.192834191	0.116054515	0.005015438
ENSDART00000076066	lin37	-0.271630989	-0.17922011	-0.496068109	-0.578879729
ENSDART00000076082	fetub	1.519329398	0.709563768	0.228067904	0.727615025
ENSDART00000076083	cdc42ep2	-0.005463107	0.666894523	0.327900404	0.083241845
ENSDART00000076157	rab24	0.15781613	0.389364959	0.281581252	0.130911228
ENSDART00000076160	mustn1a	1.455614739	1.360374319	1.112954668	0.977466313
ENSDART00000076161	hoxb5b	2.652791903	3.351280664	2.618897772	0.864721852
ENSDART00000076215	CR735102.1	-0.360410997	-0.514821634	-0.137026978	-0.257442941
ENSDART00000076238	rbm41	-0.118558432	-0.076666908	-0.261877568	-0.209896281
ENSDART00000076333	pgk1	-0.277163813	-0.302384935	-0.297237462	-0.087607565
ENSDART00000076399	nat10	0.304377518	0.292344426	0.160797024	-0.000572845
ENSDART00000076417	cdkn1bb	0.050164178	0.266815944	0.205910633	-0.014851012
ENSDART00000076423	sobpa	-0.461445043	-0.18351314	0.23019679	0.473568961
ENSDART00000076483	zgc:77151	0.249827469	0.539125177	0.736975565	0.447972658
ENSDART00000076496	stk33	-1.544458095	-0.103215934	-2.939032674	-0.830432799
ENSDART00000076502	rerglb	0.481268892	0.304392452	-0.423368722	-1.032950672
ENSDART00000076506	wisp1a	0.075916017	0.908100257	1.435223818	0.542654062
ENSDART00000076518	sla1	0.79177335	0.326196979	0.029071124	-0.326507352
ENSDART00000076554	si:zfos-464b6.2	0.5351721	0.505133501	0.952773319	0.026200547
ENSDART00000076571	rtn1a	0.31833938	1.022959786	0.981624341	0.706878795
ENSDART00000076574	rtn1a	-0.132166015	0.374408359	0.606191338	0.476970779
ENSDART00000076600	rpe65c	1.983711129	2.589375601	1.902404689	1.435704768
ENSDART00000076636	fzd2	0.563199333	0.424474998	0.171148118	-0.301884624
ENSDART00000076648	clip3	-0.386141796	-0.253171682	0.072987847	0.103065331
ENSDART00000076786	clec19a	0.036182435	0.047143104	-0.366633197	-0.838257146
ENSDART00000076815		2.456176486	3.15180058	2.959048559	2.414794476
ENSDART00000076925	ITPRIPL2	0.939030216	0.593569322	0.667833004	-0.090802943
ENSDART00000076929	prkg2	-0.322610383	-0.221985796	-0.523406711	-0.304941411
ENSDART00000076938	pogza	0.098614093	0.259761887	0.429750084	0.224276825
ENSDART00000076946	PDE4DIP	-0.575491599	-0.478988093	-0.210265098	-0.157443712
ENSDART00000076997	lmo4b	-0.24164689	-0.249534589	-0.123100185	-0.051398466
ENSDART00000077008	alox5ap	0.122223757	-0.082901221	-0.550940123	-0.70030614
ENSDART00000077047	btr09	0.55744156	0.619797073	1.272153942	-0.048548492
ENSDART00000077080	PTP4A3 (1 of many)	0.077833133	0.004217905	-0.463336754	-0.666773375
ENSDART00000077087	id3	0.047452456	0.423524517	0.285867988	-0.296627095
ENSDART00000077157	six3b	-0.273810969	-0.17356253	0.033591817	0.098241195
ENSDART00000077185	dgat1b	0.79138422	0.60487817	0.419420549	-0.346047138
ENSDART00000077197	tmsb	1.972985822	3.598018383	3.544381625	2.894174576
ENSDART00000077215	ppp2r5b	0.185911454	0.229029828	0.615811771	0.611674376
ENSDART00000077216	astn1	-0.280184115	-0.378831195	-0.109389357	0.054556021
ENSDART00000077222	ldlrad4b	-0.327819788	-0.475734059	-0.025614815	0.028486052
ENSDART00000077259	ebna1bp2	0.420936188	0.344892308	0.188849483	0.008127131
ENSDART00000077386	prss16	0.714972584	0.32483027	0.071612523	-0.240875958
ENSDART00000077406	cdh27	-0.746226456	-0.635554214	-0.974185768	-0.424771371
ENSDART00000077411	cxcl12b	-0.236947042	-0.2488008	-0.593903488	-0.559818867
ENSDART00000077418	ctsba	1.550723066	1.033464808	0.97579905	0.475659932
ENSDART00000077420	dtnbp1a	0.285624415	0.193705819	0.051855359	-0.043275565
ENSDART00000077445	pim3	-0.017745548	0.425023548	-0.018053873	0.016773558
ENSDART00000077459	smyd2a	0.109333535	0.326579335	0.423001533	0.365696401

ENSDART00000077462	slc6a9	-0.157728483	-0.388200876	-0.444419631	-0.487528264
ENSDART00000077476	prox1a	-0.192871011	-0.360465718	-0.389825586	-0.065307919
ENSDART00000077484	zhx2a	0.117201305	0.356185793	0.299966576	0.024327323
ENSDART00000077511	ccr9a	1.513300322	1.48326526	0.981085195	0.082724583
ENSDART00000077538	kpna2	3.376600286	3.247024938	2.263140647	0.702563762
ENSDART00000077539	tuba1c	-0.211130957	0.180769664	0.840172721	0.834422327
ENSDART00000077545	slc7a7	1.304066715	0.45830859	0.136139102	0.056436424
ENSDART00000077582	pitpnm3	-0.256916567	-0.419244886	-0.147473339	-0.136698838
ENSDART00000077619	b3gat1b	-0.266663197	-0.223974588	-0.393683459	-0.125347427
ENSDART00000077635	si:ch211-103n10.5	0.712451842	0.944946244	1.075959446	-0.046690849
ENSDART00000077662	myl6	0.570434868	0.203215664	-0.213132641	-0.482895856
ENSDART00000077664	atp2b1a	-0.283300071	-0.323367416	-0.218529441	-0.07251725
ENSDART00000077707	llph	0.612018484	0.617804437	0.382050706	0.042759152
ENSDART00000077715	si:dkey-25e12.3	1.07190149	0.953044464	0.701373655	0.117439727
ENSDART00000077724	gnb5b	-0.216593155	-0.279473354	-0.485943487	-0.092161444
ENSDART00000077783	hmox2a	0.068185569	0.120478633	-0.326668325	-0.242069945
ENSDART00000077805	gria2a	-0.63140941	-0.839709321	-0.362303249	-0.053629448
ENSDART00000077809	cyp26c1	-0.796873586	-0.763455549	-0.70044496	-0.582141171
ENSDART00000077823	lrit3a	-0.481897882	-0.48516269	-0.481656788	-0.455604814
ENSDART00000077834	rps27.2	0.365621497	0.39961171	0.113670294	-0.081169937
ENSDART00000077836	mmp20b	-0.526034453	-0.625086024	-0.345110188	-0.027134291
ENSDART00000077839	atf7b	-0.232485852	-0.196508033	-0.391624214	-0.206346003
ENSDART00000077868	si:ch211-212k18.7	1.166604646	0.658609076	0.758814708	-0.422210365
ENSDART00000077895	rnf7	-0.220302586	-0.252047578	-0.316298741	-0.185486087
ENSDART00000077898	grk7b	-0.582244813	-0.358943116	-0.402813205	0.000611673
ENSDART00000077951	pcolce2b	-0.119540012	-0.232226227	-0.447813206	-1.286575617
ENSDART00000077998	cgna	0.243171731	0.392675733	0.184983165	0.011488923
ENSDART00000078014	poldip2	0.012504595	-0.191194187	-0.45353852	-0.221096745
ENSDART00000078018	ruvbl2	0.355681647	0.304270351	0.334910735	0.092604809
ENSDART00000078024	crk	-0.263270816	-0.200000869	-0.157496558	-0.11349953
ENSDART00000078033	GPT (1 of many)	-0.491126295	-0.482727977	-0.247455346	-0.055451846
ENSDART00000078037	aclyb	-0.084913872	-0.379038696	-0.658479637	-0.284307838
ENSDART00000078053	tbcb	0.21865146	0.428683048	0.432538452	0.172774297
ENSDART00000078072	akap12b	2.975823835	3.328543122	3.653370868	2.600734247
ENSDART00000078079	pcnxl2	-0.518593139	-0.325199094	0.020646332	0.403616211
ENSDART00000078115	sdhda	-0.602799581	-0.148637611	0.027660422	0.175615096
ENSDART00000078137	ankrd54	0.051281727	0.23783078	0.382868701	0.32135773
ENSDART00000078148	smc1a	-3.399991944	-0.069804776	-1.248223526	-0.569589215
ENSDART00000078156	srm	0.127220715	0.22303682	0.345313656	0.085476496
ENSDART00000078181	SLC3A2 (1 of many)	-0.23816401	-0.122270906	-0.56307324	-1.283472558
ENSDART00000078187	foxo4	-0.056772502	-0.136599728	-0.36455832	-0.08188156
ENSDART00000078192	cnpy4	0.065580495	0.06657667	-0.1806698	-0.737660818
ENSDART00000078202	phka2	-0.128194416	-0.258622872	-0.071035307	0.033146819
ENSDART00000078226	mtnr1bb	-0.285812097	-0.289316068	-0.599481549	-0.276891324
ENSDART00000078232	cdh10a	-0.495858439	-0.491434243	-0.331011431	0.033804718
ENSDART00000078249	kcnc3a	-0.908848026	-1.288714191	-0.644595688	0.010477706
ENSDART00000078256	dopey2	-0.056439943	-0.30342326	-0.284521914	-0.1564256
ENSDART00000078266	rsl1d1	0.351365949	0.392552672	0.175928217	-0.070353732
ENSDART00000078277	msmo1	0.087631699	1.109674669	1.47165822	0.852850391
ENSDART00000078283	tex10	0.186689066	0.248875437	0.343572259	0.154977674
ENSDART00000078304	lzic	0.119250866	0.44378754	0.377529349	0.128075007

ENSDART00000078305	zswim5	-0.126655043	0.076098312	0.245252624	0.223511201
ENSDART00000078306	arhgef2	0.429610468	0.807274797	0.854804837	0.533632211
ENSDART00000078311	zgc:154093	-0.170397489	-0.310689773	-0.421136702	-0.191616401
ENSDART00000078316	nipa2	0.247262018	0.436255727	0.4433152	0.003440759
ENSDART00000078325	slc16a8	2.36549078	0.51323664	-0.815797835	-1.896302174
ENSDART00000078334	celsr3	0.469496099	0.512562938	0.74195357	0.599858827
ENSDART00000078336	klc3	-0.15725421	-0.267140148	-0.672772286	-0.331888789
ENSDART00000078352	tspan14	0.358298965	0.408510067	0.238594355	-0.012844708
ENSDART00000078412	rps8a	0.416303691	0.456350868	0.419741526	0.122907452
ENSDART00000078438		0.22834743	0.583517385	0.218267131	0.08549295
ENSDART00000078449	itih6	-0.23325998	-0.04381884	-0.494745518	-1.555031348
ENSDART00000078491	mov10b.2	0.18890489	0.151487185	1.352539948	-0.244846261
ENSDART00000078494	l3mbtl2	1.071567754	1.041266281	0.642956308	0.316295602
ENSDART00000078522	eef1g	0.462831941	0.651007925	0.567355432	0.209607784
ENSDART00000078529	kin	-1.437736057	-0.295663012	-0.493580296	-4.734617185
ENSDART00000078533	kcnd3	-0.299800885	-0.32147822	-0.292734142	-0.136341479
ENSDART00000078535	gtf2h5	0.788173845	1.2490535	0.921738211	0.911998292
ENSDART00000078543	syt11b	0.975892204	1.6420197	1.925407845	1.338618886
ENSDART00000078561	SPTBN4	-0.239897718	-0.33102434	0.293671093	0.445086753
ENSDART00000078594	tyrp1b	0.662132615	0.257868212	-0.309681889	-0.980797447
ENSDART00000078596	hspd1	0.512169965	0.358816938	0.201006014	0.20720259
ENSDART00000078611	jac2	3.404844888	3.429750736	2.921387124	1.113891823
ENSDART00000078630	nme7	0.176767548	0.421131107	0.35919241	0.2453347
ENSDART00000078634	eif5a2	0.493280465	0.485481598	0.418411017	0.051860815
ENSDART00000078642	vps37b	0.195137227	0.217160121	0.327187369	0.074828156
ENSDART00000078647	si:ch211-201h21.5	1.148616031	0.689283012	0.40532037	0.049879214
ENSDART00000078652	camk2g2	-0.278366483	-0.285453125	-0.22783561	-0.037372706
ENSDART00000078694	TMEM179B (1 of many)	0.551851512	0.299347782	0.026749154	-0.302916803
ENSDART00000078708	zgc:162952	-0.249018938	-0.407117986	-0.404487357	-0.328471526
ENSDART00000078723	dixdc1a	0.324551864	0.395166532	0.459267183	0.274985584
ENSDART00000078771	SBK1	-0.390774004	0.13684565	0.923517231	0.784697628
ENSDART00000078792	ahcyl1	0.058902937	0.000957907	-0.341276653	-0.275160426
ENSDART00000078795	ahcyl1	-0.360916144	-0.460734164	-0.414619806	-0.201451999
ENSDART00000078838	rab3aa	-0.225658115	-0.303506805	-0.374028659	-0.138563861
ENSDART00000078843	SLC22A7 (1 of many)	-0.18908726	-0.089234859	-0.388754878	-1.485871042
ENSDART00000078856	dlg3	-0.31041276	-0.361138693	-0.191257864	0.041004354
ENSDART00000078858	si:ch73-86n18.1	0.106229924	-0.155735751	-0.58770094	-0.854457693
ENSDART00000078866	ifi30	1.574791131	0.896478525	0.373397245	-0.020268978
ENSDART00000078877	sncga	-0.059233705	0.307631997	0.27775793	0.434885626
ENSDART00000078908	usp1	-0.163356677	-0.229514103	-0.329514373	-0.251997637
ENSDART00000078916	smim7	-0.217464923	-0.109941617	-0.350272436	-0.25934653
ENSDART00000078949	afap1l1b	1.061182692	1.814819842	1.643265844	0.877712611
ENSDART00000078953	afap1l1b	0.880715307	1.833091326	1.686561879	0.852421188
ENSDART00000078982	vat1	0.139786944	0.289776545	0.306149934	-0.025026234
ENSDART00000079019	spsb1	0.176345745	0.177736786	0.29021019	0.163667209
ENSDART00000079035	rap1gap	-0.57187807	-0.701331328	-0.299987324	-0.289210087
ENSDART00000079046	cpamd8	-0.122112474	-0.085213564	-0.191819718	-0.872911162
ENSDART00000079050	nutf2	0.337010875	0.380281227	0.325036311	0.156803484
ENSDART00000079092	si:dkey-261i16.5	-0.526813533	-0.563690076	-0.369398724	0.061616577
ENSDART00000079104	ndufs6	-0.146008815	-0.377028896	-0.443194118	-0.327081615
ENSDART00000079112	calca	-0.439449486	-0.32190484	-0.601830596	-0.728154291

ENSDART00000079138	ptenb	-0.337433521	-0.277401842	-0.145004066	-0.034401548
ENSDART00000079144	ptenb	-0.303152595	-0.238217531	-0.001915727	-0.031063744
ENSDART00000079173	lepr	1.173965688	1.129708824	0.920018098	0.6672678
ENSDART00000079202	abcb8	-0.111089791	-0.249333097	-0.256171214	-0.098593711
ENSDART00000079222	chaf1b	1.242408116	0.820706616	0.642546621	0.111426229
ENSDART00000079235	cd99l2	-0.167082345	0.448795434	0.91020128	0.864265064
ENSDART00000079283	tmeff1b	0.060962109	0.679659218	0.971148806	0.712213835
ENSDART00000079310	manba	0.495609624	0.109254813	0.005925635	-0.12409296
ENSDART00000079341	plch1	-0.457888719	-0.43639001	-0.345543016	-0.155469708
ENSDART00000079364	snapc2	-0.030032776	-0.185097862	-0.560264081	-0.400163167
ENSDART00000079397	ryk	-0.107777448	0.087264815	-0.11559458	-0.531439469
ENSDART00000079431	rtn2b	0.025228511	-0.123165337	-0.319794883	-0.05842346
ENSDART00000079443	GABRA2 (1 of many)	-0.623319426	-0.542833879	-0.157704672	0.138036393
ENSDART00000079454	vamp2	-0.197978353	-0.366012835	-0.326357547	-0.1060827
ENSDART00000079497	emg1	0.201493898	0.380905959	0.206415684	0.036355374
ENSDART00000079518	dnajc1	0.043021447	-0.028027956	-0.123370167	-0.287218054
ENSDART00000079528	ilk	0.338564211	0.350049719	0.346485507	0.032055843
ENSDART00000079536	nek7	0.300813238	0.263862785	0.126120434	-0.061245789
ENSDART00000079549	tpte	0.809167451	0.638911968	0.260848622	-0.250321254
ENSDART00000079559	ifit16	0.716638095	0.325468397	2.311988055	-0.283531995
ENSDART00000079563	fas	0.786193523	0.954741877	1.62170637	0.201092535
ENSDART00000079591	dpm1	-0.022510061	-0.080956932	-0.275574355	-0.162526678
ENSDART00000079597	vps36	-0.205754956	-0.373190829	-0.317459289	-0.134792851
ENSDART00000079629	ppm1nb	-0.279926594	-0.345062767	-0.462173038	-0.171806172
ENSDART00000079656	tvp23b	-0.013268416	-0.155098046	-0.309054243	-0.268412353
ENSDART00000079686	zmp:0000001103	0.143973249	-0.889323937	-0.759747318	-0.487193938
ENSDART00000079695	zwilch	1.159509442	0.995361025	0.861360163	0.105564769
ENSDART00000079711	slc25a1a	0.025053389	0.916416559	1.283110285	0.925187833
ENSDART00000079716	hpf1	-0.113759988	-0.292000236	-0.336217157	-0.156517495
ENSDART00000079778	ifit8	1.432186723	0.097505141	4.166007884	0.96250098
ENSDART00000079803	nmt1b	0.899927422	1.156945789	1.408075075	0.459974747
ENSDART00000079810	si:dkey-222b8.4	-0.076664011	-0.231174428	-0.530241167	-0.088965105
ENSDART00000079840	rorca	-0.65562057	-0.064052995	0.161319682	0.103804992
ENSDART00000079843	nceh1b.2	0.690825571	0.217907848	-0.110601617	-0.426325911
ENSDART00000079866	slc30a9	-0.061810697	-0.211371943	-0.307919743	-0.172076689
ENSDART00000079879	si:dkey-91i10.3	-0.580141681	-0.553279281	-0.678150475	-0.52463511
ENSDART00000079884	alox5a	0.143901368	-0.192427785	-0.334678776	-0.585419844
ENSDART00000079984	rpl22l1	0.678692893	0.584241724	0.331994973	-0.011632328
ENSDART00000080014	rps8b	-0.261943359	-0.309301007	-0.481979191	-0.409711249
ENSDART00000080016	ppp3ccb	-0.214631103	-0.298468254	0.05278016	0.038022829
ENSDART00000080033	SSBP2 (1 of many)	0.123939988	0.461173497	0.600467157	0.3572706
ENSDART00000080042	rab33a	-0.045588719	0.341497657	0.630733663	0.503197845
ENSDART00000080064	CR847953.1	0.050009503	-0.040451977	0.259802571	0.438904414
ENSDART00000080079	slc44a5b	0.101790496	-0.11939972	-0.347901034	0.093736244
ENSDART00000080100	slc24a2	-0.273129774	-0.420453101	-0.415034704	-0.05535122
ENSDART00000080129	ston2	-0.17811412	-0.063813163	-0.270400144	-0.092351554
ENSDART00000080135	gfpt1	0.380664344	0.49511154	0.544262456	0.337010461
ENSDART00000080256	nefma	0.572074143	2.009260502	2.281701505	1.529767044
ENSDART00000080289	prrc2c	0.060093623	0.047472332	0.306363857	0.189281148
ENSDART00000080313	arl4ca	-0.176267156	-0.177242747	-0.285935967	-0.135129968
ENSDART00000080328	nf1a	-0.041622168	-0.338478743	-0.243156878	-0.085905935

ENSDART00000080339	galm	0.868912331	0.484945087	-0.111697277	-0.429352779
ENSDART00000080342	josd2	0.375878923	0.743043487	0.777511515	0.447917768
ENSDART00000080351	dhx57	0.296631931	0.310315824	0.343123119	0.20162921
ENSDART00000080377	aldoca	2.758856293	1.548001605	2.09362575	1.794239524
ENSDART00000080385	SLC9A3R2 (1 of many)	-0.157518802	-0.380020166	-0.549234439	-0.123708468
ENSDART00000080389	fam13a	0.342651645	-0.065971229	-0.119440295	0.116639155
ENSDART00000080414	sbk3	-0.999751639	-0.617799332	-0.160630934	-0.723965523
ENSDART00000080423	ctsd	0.448869492	0.173368574	-0.412435712	-0.637183839
ENSDART00000080430	gfra2b	-0.62088032	-0.576945607	-0.392707521	-0.144958503
ENSDART00000080465	hells	0.812911201	0.936234984	0.557478835	0.182957003
ENSDART00000080486	ywhag1	-1.02392497	-0.686234935	-0.010129168	0.2177797
ENSDART00000080523	itgae.2	1.067269567	0.812178684	0.361968863	0.003662922
ENSDART00000080549	lyz	1.762095695	1.25446765	1.569214818	0.577231805
ENSDART00000080602	map7d2b	0.243598476	0.831190471	1.239754477	0.898189276
ENSDART00000080628	arpc3	0.270913011	0.099552933	0.079917753	-0.036372289
ENSDART00000080664	zgc:86709	1.428698865	3.355720442	2.498933956	1.732893579
ENSDART00000080673	syt11a	-0.340996032	-0.110491382	0.046613391	0.116186716
ENSDART00000080679	ccdc136b	-1.000484865	-0.898166187	-0.625061619	-0.323992777
ENSDART00000080712	slc43a3b	2.109023631	1.075213126	0.724428131	-0.154263604
ENSDART00000080771	slc6a16a	-0.167322319	-0.266319768	-0.350765511	-0.319541774
ENSDART00000080789	trpc4a	-0.390087102	-0.398102567	-0.462339329	-0.409517574
ENSDART00000080808	six3a	-0.437705937	-0.430575558	-0.140593988	-0.07114268
ENSDART00000080829	hspa14	0.672662829	0.694717457	0.8195912	0.418167344
ENSDART00000080854	stat3	0.856010749	0.444560766	0.379796349	-0.326021696
ENSDART00000080864	magt1	0.441285986	0.302582629	0.047526992	-0.172355248
ENSDART00000080875	b3gnt3	0.44585097	0.568958183	0.901392395	0.139618312
ENSDART00000080900	cfap57	2.52486974	2.757508228	2.941219811	1.994632033
ENSDART00000080904	sardh	0.759429129	0.915608159	0.345777903	-0.12435587
ENSDART00000080919	rpl36a	0.405736348	0.485454355	0.225930563	-0.002722727
ENSDART00000080927	snap25b	-0.418114286	-0.385475041	-0.102955296	0.076752829
ENSDART00000081039		2.046984335	2.965673996	2.312859948	-1.109370855
ENSDART00000081059	rps6kb1b	0.156747941	0.167680861	0.344534054	0.174989656
ENSDART00000081092	si:dkeyp-77h1.4	-0.383994825	0.24121529	0.967657356	0.94823494
ENSDART00000081129	cdk15	-0.731126626	-0.682923022	-0.544281357	-0.253398129
ENSDART00000081140	CT990561.1	-0.348057632	-0.380510005	-0.109063338	0.180807023
ENSDART00000081154	prpf18	-0.15179636	-0.149318949	-0.443970585	-0.330213785
ENSDART00000081170	cux1a	-0.092953946	-0.2283391	0.138990685	0.296890549
ENSDART00000081183	enc3	0.409273368	0.447706974	0.259987961	0.244016846
ENSDART00000081204	acot9.1	0.172924803	0.462969941	0.48161826	0.340925129
ENSDART00000081214	selt1a	-0.31898529	-0.284714031	-0.513594635	-0.412445291
ENSDART00000081223	krt5	2.400508858	4.729984226	4.862312842	5.813921541
ENSDART00000081228	abhd14a	-0.111769944	-0.123001167	-0.255304369	-0.088239573
ENSDART00000081272	gcn1	0.168820856	0.100284898	0.363305756	0.2232516
ENSDART00000081290	rnaset2l	0.733135995	0.630785596	0.575081946	0.019045787
ENSDART00000081323	abhd17c	-0.03998334	0.119899277	0.261541408	0.19734068
ENSDART00000081325	dynll1	-0.057380796	-0.1118203	-0.516198015	-0.112248637
ENSDART00000081326	prpf40a	0.136343368	0.189733856	0.260275336	0.074340716
ENSDART00000081338	slc9a5	0.890559011	1.045395859	1.329125407	1.036960607
ENSDART00000081343	plk1	1.547304713	1.292689538	1.02080622	0.322979307
ENSDART00000081359	zgc:110425	0.523386438	0.743109297	0.819385742	0.340090119
ENSDART00000081411	pole	0.480999213	0.458730193	0.174065324	-0.07089485

ENSDART00000081432	sprb	-0.390584563	-0.260880914	-0.194039821	-0.19414991
ENSDART00000081447	fbxo30a	0.072897711	0.325740781	0.333966964	0.186898423
ENSDART00000081468	ccdc79	0.331555315	0.505423262	0.318137438	-0.103372006
ENSDART00000081510	celf4	-0.384169548	-0.376019008	-0.02531238	0.147771891
ENSDART00000081546	trim46b	0.101540064	0.068781295	0.327282891	0.456457337
ENSDART00000081568	tcf19l	0.712871286	0.397385898	0.351815803	0.227291981
ENSDART00000081601	cept1a	0.150762086	0.296977372	0.445683893	0.275050142
ENSDART00000081611	cgnb	-0.200219306	-0.438466186	-1.067503957	-0.17391972
ENSDART00000081620	vax2	-0.290535146	-0.478626837	-0.10258237	-0.009557703
ENSDART00000081646	glrx	0.838457918	1.280146822	1.066830025	0.395822945
ENSDART00000081761	bin1b	-0.023096846	-0.153451242	-0.459181763	-0.132136814
ENSDART00000081781	PLEKHG3	-0.076813883	-0.173391418	-0.382548311	-0.034437681
ENSDART00000081794	rasgrf2a	-0.063040785	-0.093088949	-0.266637358	0.015786604
ENSDART00000081797	sash1b	-0.368433045	-0.365106826	-0.357216383	-0.178826864
ENSDART00000081811	zgc:112052	0.311036284	0.397766463	0.340201557	0.120599155
ENSDART00000081832	ptpdc1b	0.49240902	0.580136174	0.651643292	0.340422756
ENSDART00000081870	pcdh2ab6	0.090888519	0.246515279	0.356189629	0.303363778
ENSDART00000081926	CU856539.1	-0.250902601	-0.402416425	-0.31567263	-0.091643419
ENSDART00000081946	zgc:112332	-0.051059621	0.027246103	-0.289131429	-0.652053172
ENSDART00000081951	stx1b	-0.290947433	-0.255213869	0.04983789	0.170348415
ENSDART00000081966	rtn4a	0.42137623	0.811004738	0.55995797	0.082545275
ENSDART00000081978	KCNJ6	-0.364130454	-0.389723131	-0.355694518	-0.126078935
ENSDART00000081985	pim2	-0.011194591	-0.040743933	-0.388705404	-0.753111264
ENSDART00000081990	strip2	-0.530994895	-0.582233551	-0.263527962	-0.22210065
ENSDART00000082011	lim2.2	1.304610538	0.978295786	0.86763989	0.856876261
ENSDART00000082012	gsk3aa	-0.41333003	-0.370003942	-0.288947953	-0.203102626
ENSDART00000082050	zgc:174904	1.977093547	1.430607855	1.439069833	0.228460651
ENSDART00000082063	fam114a1	0.586474654	0.396933414	0.041198489	-0.237340023
ENSDART00000082066	atpv0e2	-0.268294846	-0.377237748	-0.604078331	-0.39998077
ENSDART00000082080	jupb	-0.499893077	-0.526312414	-0.258393629	-0.100168242
ENSDART00000082082	gars	0.395042441	0.664200633	0.717176297	0.392291214
ENSDART00000082097	PRSS35 (1 of many)	0.904416504	0.725234092	1.067628772	0.791677307
ENSDART00000082142	EFEMP1 (1 of many)	-1.76159347	-0.849874708	-0.584181957	-1.080912576
ENSDART00000082151	uqcrh	-0.212671975	-0.358890471	-0.337939592	-0.281374001
ENSDART00000082223	tax1bp3	0.85130321	1.256170012	1.075265217	0.22088107
ENSDART00000082264	pxdc1b	0.801924492	1.033443039	0.634881962	0.139425975
ENSDART00000082301	myrip	-0.137038516	-0.238384017	0.084718693	0.41131665
ENSDART00000082346	tfap2a	-0.238141907	-0.307957291	-0.242159487	-0.230339237
ENSDART00000082368	marco	0.980427549	0.782180823	1.00834904	0.091028138
ENSDART00000082434	tgif1	0.822910382	1.229522702	1.441745222	0.912580325
ENSDART00000082438	dlgap2a	-0.366533513	-0.540400426	-0.257659814	-0.037294062
ENSDART00000082458	sarnp	0.25573821	0.275816383	0.16902753	0.007612146
ENSDART00000082471	mfap2	0.61383585	0.779035446	0.354750417	0.055843134
ENSDART00000082517	rab43	0.357940531	0.212689136	-0.148032038	-0.068856369
ENSDART00000082523	impa2	-0.208830066	-0.398914852	-0.498863304	-0.267214166
ENSDART00000082604	galnt18b	-0.214631898	-0.351577935	-0.246678211	-0.218706176
ENSDART00000082620	dysf	1.140285476	2.000991026	2.040057287	1.067021482
ENSDART00000082622	fsd1l	-0.046506283	-0.340407639	-0.41095417	-0.201162325
ENSDART00000082698	kank4	-0.273638259	-0.263464734	-0.200789629	-0.103573896
ENSDART00000082715	camsap3	0.302702799	0.347512275	0.333666179	-0.022433363
ENSDART00000082745	EMB	-0.651102521	-0.703010006	-0.074268357	0.192993872

ENSDART00000082821	rims4	-0.331865117	-0.050822604	0.125345094	0.041526523
ENSDART00000082830	KIAA0895L	0.216380627	0.852873359	0.835551488	0.401334264
ENSDART00000082842	JPH3 (1 of many)	-0.540363483	-0.227235849	0.34555956	0.43863208
ENSDART00000082937	fscn2a	-0.244556215	-0.436229092	-0.571250568	-0.199107259
ENSDART00000082944	dock6	0.129469289	0.283374744	0.379764071	0.290753034
ENSDART00000082983	clip2	0.69105479	0.899482735	1.166942455	0.710683744
ENSDART00000083002	map1aa	0.023824463	0.057494175	0.8302085	0.470304447
ENSDART00000083010	acad9	0.301381925	0.15641426	-0.079322034	-0.023998781
ENSDART00000083033	sik1	1.146093805	0.631897288	0.512350737	1.123188354
ENSDART00000083040	hs3st2	-1.059711916	-1.469328404	-0.190665179	0.30791737
ENSDART00000083063	tal1	0.195096671	0.315620662	0.697094431	0.015122988
ENSDART00000083066	asphd2	-0.487940941	-0.316881347	-0.126036033	0.003992278
ENSDART00000083085	mtmr14	0.202842573	0.147255325	0.298709035	0.120380705
ENSDART00000083100		-0.514905306	-0.64333228	-0.306814422	-0.178642671
ENSDART00000083126	cidec	3.092638419	2.402632883	1.147285193	-0.231828568
ENSDART00000083212	fscn1a	-0.172521555	0.403697769	0.865067524	0.635846942
ENSDART00000083294	nol6	0.354535252	0.232360208	0.196448331	0.109158776
ENSDART00000083359	sec14l8	-0.359432343	-0.324487355	-0.138175947	-0.2576341
ENSDART00000083367	prcp	-0.058674879	-0.090308681	-0.37460188	-0.414832363
ENSDART00000083394	si:dkey-106c17.3	-0.374020521	-0.387399838	-0.436274502	-0.115892618
ENSDART00000083407	b4galnt4a	-0.141610106	-0.312267637	-0.129787335	-0.08625756
ENSDART00000083416	gabrd	-0.516993342	-0.41812722	-0.415766575	-0.292897654
ENSDART00000083427	slc25a29	-0.338331576	-0.636979798	-0.361185623	-0.191915619
ENSDART00000083449	dub	0.532370465	0.813595698	0.783551631	-0.001039848
ENSDART00000083453	slc32a1	-0.393731273	-0.378330473	-0.304655077	-0.16825712
ENSDART00000083467	parp8	0.336619688	0.399001121	0.399118225	0.307424215
ENSDART00000083569	oaz2b	-0.447559352	-0.669337328	-0.007273423	0.348858251
ENSDART00000083572	zgc:136864	0.242428375	0.324173418	-0.028258929	-0.082397484
ENSDART00000083605	tbc1d25	0.094218097	0.226970926	0.332123969	0.140604089
ENSDART00000083628	ddit3	-0.053032847	0.505000026	0.598711637	0.25299651
ENSDART00000083670	CABZ01041604.1	-0.549505735	-0.427354636	-0.238335089	-0.140824262
ENSDART00000083684	pappab	-0.696202319	-0.797866787	-0.223755037	-0.122490212
ENSDART00000083731	trpv1	1.343483137	1.058081357	0.730442906	-0.443240884
ENSDART00000083788	CU633832.1	-0.745373175	-0.699527481	-0.49975457	-0.170614876
ENSDART00000083797	TBC1D9B	0.273448063	0.469124554	0.581828184	0.40676136
ENSDART00000083830	sdc4	0.356242427	0.466156404	0.190102023	-0.370187936
ENSDART00000083890	usp24	0.219267617	0.201082864	0.325420731	0.216599954
ENSDART00000084007	TULP2	-0.184892554	-0.234742374	-0.492430755	-0.167640019
ENSDART00000084011	cplx4a	-0.220118176	-0.343953082	-0.589002173	-0.338126511
ENSDART00000084014	cplx1	-0.741807214	-0.862001833	-0.211893904	0.449532765
ENSDART00000084024	sv2c	-0.354928985	-0.65582646	-0.477522685	-0.220826118
ENSDART00000084035	znf532	-0.199573234	-0.524026601	-0.06012509	0.154210784
ENSDART00000084055	fzd7a	-0.350921417	-0.318886271	-0.299513467	-0.86880486
ENSDART00000084069	iqgap2	1.059547107	0.55852141	0.220571876	-0.354231411
ENSDART00000084119	si:ch1073-44g3.1	-0.384776638	-0.202587046	0.01646479	0.134112994
ENSDART00000084131	fam160b2	0.036570039	-0.082247362	-0.429951116	-0.30533058
ENSDART00000084135	ubtd1a	-0.22864625	-0.383233305	-0.947657938	-0.688823144
ENSDART00000084184	aimp1	0.209084138	0.262090446	0.396284966	0.096096937
ENSDART00000084238	CABZ01057411.1	-0.328182832	-0.451030622	-0.21027367	0.014791503
ENSDART00000084264	adcy2a	-0.574108823	-0.494423944	0.049230433	0.113035845
ENSDART00000084353	tbc1d10ab	-0.174770874	-0.349891521	-0.619429045	-0.244339697

ENSDART00000084354	cpeb3	-0.1650884	-0.603027317	0.172363697	0.2398868
ENSDART00000084355	zgc:165481	-0.665978496	-0.883055142	-0.199024252	-0.082238068
ENSDART00000084373	frmd4bb	0.582631667	0.828834738	0.835563839	0.412330382
ENSDART00000084378	crb2a	-0.010590407	-0.348182237	-0.479892445	-0.295243873
ENSDART00000084381	sybu	-0.763177411	-0.608942869	-0.49552138	-0.188366829
ENSDART00000084416	ablim1a	-0.251488969	-0.08542034	0.289459971	0.335228062
ENSDART00000084417	timm17b	-0.134748392	-0.15578708	-0.404232794	-0.18557959
ENSDART00000084448	psmd11a	0.167797415	0.349500372	0.318678727	0.030124317
ENSDART00000084512	pkn1a	0.199755386	0.283744748	0.437490279	0.320601704
ENSDART00000084517	vcpip1	0.020173722	0.114070565	0.246341011	0.321118019
ENSDART00000084530	coro2ba	-0.558160234	-0.686474796	-0.264666518	-0.204619183
ENSDART00000084598	vimp	-0.003252442	-0.076004385	-0.324006724	-0.304272734
ENSDART00000084714	zranb1a	-0.285770869	-0.28077285	-0.104830495	0.014952891
ENSDART00000084729	pecam1	0.670854861	0.415048291	0.182847943	0.022819591
ENSDART00000084730	zgc:162160	-0.758204424	-0.82447566	-0.164736838	0.207922006
ENSDART00000084771	pde9a	0.836076081	1.443202889	1.33955445	1.070879094
ENSDART00000084792	prosc	-0.109541047	-0.121094609	-0.31852643	-0.202300262
ENSDART00000084803	asic2	-0.588195088	-0.477145292	-0.317477641	-0.106481463
ENSDART00000084806	slc4a10b	-0.257067272	-0.281842585	-0.215225145	-0.174870087
ENSDART00000084819	arhgap35b	0.101645005	0.291379647	0.535032879	0.462050203
ENSDART00000084861	cish	-0.446878588	-0.18032932	-1.508647694	-1.506136587
ENSDART00000084890	si:ch211-284e13.4	-0.124688162	-0.262617592	0.237393976	0.369141192
ENSDART00000084965	cep104	-0.196545786	-0.225873142	-0.456327943	-0.208454995
ENSDART00000085121	sdk2b	-0.222566721	-0.365719278	-0.147657392	-0.012041014
ENSDART00000085135	tbl1x	0.180884774	0.566948084	0.5040067	0.049402086
ENSDART00000085142	map1b	-0.213270056	0.210763047	0.526451874	0.312155026
ENSDART00000085210	cacna1ha	-0.413195046	-0.405391107	-0.236177845	0.006546496
ENSDART00000085230	atl1	-0.283512177	0.138626411	0.619353197	0.442930265
ENSDART00000085252	pqlc3	-0.117366098	-0.27675705	-0.43476147	-0.253093536
ENSDART00000085253	mid1	-0.357661223	-0.330080838	-0.002902011	0.078651096
ENSDART00000085263	SELENOI	0.036305982	0.149651758	0.460720877	0.164646013
ENSDART00000085277	pfkmb	0.010653815	-0.414332737	-0.69351419	-0.144457554
ENSDART00000085284	pfkla	-0.114418267	-0.338020067	-0.177396742	0.008723864
ENSDART00000085294	tnfrsf9a	-0.468498688	-0.352709139	-0.665957795	-1.403982945
ENSDART00000085309	dpcd	0.675929667	0.665840567	0.458693669	0.16198721
ENSDART00000085319	sos2	0.062155314	0.173737983	0.464042198	0.208637802
ENSDART00000085370	kcnq5b	-0.41441932	-0.216130879	-0.15503054	-0.008181026
ENSDART00000085388	bmp3	-0.146774305	1.240822845	1.494378971	0.416428855
ENSDART00000085438	rps6ka5	-0.245466951	-0.387017016	-0.309352273	-0.119154274
ENSDART00000085442	mut	-0.094284216	-0.181855717	-0.336054518	-0.342951233
ENSDART00000085453	cluhb	-0.484145834	-0.430773206	0.678836708	-0.017745757
ENSDART00000085472	grm2a	-0.506007863	-0.654883229	-0.268518199	0.087304206
ENSDART00000085522	hspb6	-0.63697673	-0.382982575	-0.524822831	-0.392317705
ENSDART00000085528	zgc:158659	0.601601674	0.738184978	0.82446023	0.805817257
ENSDART00000085565	capn15	-0.382220369	-0.426351921	-0.021041552	0.033591442
ENSDART00000085573	rgs7bpa	-0.750985181	-0.701592527	-0.219110071	-0.146877341
ENSDART00000085612	pcdh7b	-0.228836495	-0.682608646	-0.209101131	0.321031694
ENSDART00000085675	clstn2	-0.244638643	-0.37840099	-0.228974377	0.094704371
ENSDART00000085684	ttll11	-0.16810055	0.020727161	0.348458395	0.408691778
ENSDART00000085693	gpm6bb	-0.236628216	-0.325741908	-0.400571921	-0.218025638
ENSDART00000085716	mtmr10	0.168573884	-0.120117023	-0.772748173	-0.357648365

ENSDART00000085719	si:ch211-10a23.2	0.141906082	0.927202925	1.15606105	0.59243708
ENSDART00000085743	AL935194.1	-0.774745358	-0.68914542	-0.32626184	0.039334462
ENSDART00000085764	PLOD3	0.414167142	0.202506542	0.107388816	-0.10168282
ENSDART00000085894	pgm5	1.697021259	1.783911903	2.232651815	1.757015772
ENSDART00000085993	pxnb	0.457322476	0.299419771	0.2051328	-0.178681091
ENSDART00000086051	mecom	-0.550304416	-0.298852875	-0.293768303	-1.334615988
ENSDART00000086117	kcnab2b	-0.362026581	-0.574025944	-0.45188395	-0.145196155
ENSDART00000086131	pik3c2a	-0.071329777	-0.019579026	-0.128057314	-0.294437789
ENSDART00000086176	nckap1	-0.438983658	-0.533344195	-0.439721489	-0.241138066
ENSDART00000086181	cabp7b	-0.513778995	-0.579329622	-0.46209057	-0.104843884
ENSDART00000086263	mettl7a	-0.420009286	-0.158826062	-0.791334734	-0.334080113
ENSDART00000086281	mavs	3.81695815	3.176539113	3.535647274	1.650162791
ENSDART00000086301	irge4	0.875091617	0.681146857	1.737500297	0.803175592
ENSDART00000086333	jarid2a	-0.109318405	0.11715985	0.373609719	0.35216542
ENSDART00000086409	dync1i1	-0.021724393	0.427961112	0.551451734	0.438467635
ENSDART00000086434	tmcc2	0.022092982	0.338134833	0.850580716	0.840264518
ENSDART00000086495	zgc:154077	-0.133678102	-0.278559633	-0.319223928	-0.190822097
ENSDART00000086537		0.422133332	0.379254853	0.651471983	0.472158305
ENSDART00000086617	gabbr2	-0.577324776	-0.926125922	-0.433004274	0.052999603
ENSDART00000086619	prkca	-0.184045571	-0.418285198	-0.5357326	-0.310736719
ENSDART00000086664	trpm1a	2.715802028	4.516032094	3.238851669	3.187690164
ENSDART00000086720	nfasca	-0.417931887	-0.576283691	-0.313335716	-0.150054847
ENSDART00000086753	dapk2a	0.129419785	-0.339935803	-1.219284243	-0.369104907
ENSDART00000086797	adgrl3.1	-0.114053477	-0.068166121	0.465121947	0.57391191
ENSDART00000086867	tapt1b	-0.153549174	-0.259614839	-0.218992302	-0.161727037
ENSDART00000086905	nrn1lb	-0.455174799	-0.417502528	-0.690990621	-0.656036806
ENSDART00000086946	mov10b.1	0.41644893	0.305142124	1.89370555	0.04709269
ENSDART00000086952	st14a	-0.283184933	0.988901668	1.786647129	1.271482733
ENSDART00000086994	nat15	-0.144704703	-0.149678764	-0.337458722	-0.152039739
ENSDART00000087070	abcc5	0.063506972	-0.209331316	-0.331966374	-0.070006315
ENSDART00000087097	ogfrl1	-0.189021726	-0.350288897	-0.29146473	0.000580163
ENSDART00000087105	myom2a	2.177324505	3.608086766	3.587707417	2.779461648
ENSDART00000087107	eif4g1a	0.252655247	-0.051267066	0.413833734	0.330964133
ENSDART00000087112	pfdn4	0.448340647	0.452924674	0.218263471	-0.022743355
ENSDART00000087114	alg5	0.317519293	0.464842808	0.220188706	0.059168575
ENSDART00000087115	rims1b	-0.155072254	0.041830315	0.378907446	0.287621284
ENSDART00000087118	xylt1	-0.272537601	-0.39007956	-0.067871098	0.053209242
ENSDART00000087148	cbln4	-0.802680954	-0.769511703	-0.146403482	0.304907793
ENSDART00000087191	mark4a	-0.415170271	-0.372344968	0.042679549	0.108705644
ENSDART00000087196	zgc:153240	-0.381380177	-0.392146036	-0.277166885	-0.130863015
ENSDART00000087204	dusp3a	-0.327203373	-0.053264752	0.592884224	0.622404395
ENSDART00000087280	cacnb3a	-0.323388935	-0.465191463	0.268224123	0.533679905
ENSDART00000087295	ppp1r9a	-0.056007418	-0.599550993	-0.15522086	-0.017719681
ENSDART00000087300	gabrb3	-0.106002759	-0.319333142	-0.574890474	-0.11105969
ENSDART00000087311	oca2	0.449332393	0.338475194	-0.09076694	-0.174077968
ENSDART00000087329	znf438	-0.159611183	-0.256202272	-0.311134609	-0.110177372
ENSDART00000087339	cdon	-0.358409006	-0.160970657	-0.495687672	-0.865355598
ENSDART00000087426	bcl11aa	-0.367520296	-0.334252656	0.232372171	0.511638417
ENSDART00000087441	GFOD1	-0.421601041	-0.219799435	-0.160457067	0.058971995
ENSDART00000087449	CR847968.1	-0.323687626	-0.307881439	0.124728655	0.148402357
ENSDART00000087450	klf13	-0.22166629	-0.059571459	0.622443971	0.288396102

ENSDART00000087565	eva1a	-0.378976356	-0.384921525	-0.250389955	-0.168669364
ENSDART00000087570	BRSK2 (1 of many)	-0.035673495	-0.289130617	-0.495870531	-0.115059951
ENSDART00000087586	c2cd4a	-0.882018106	-1.482044104	-0.368555759	0.13043866
ENSDART00000087624	arhgef1b	4.493077515	3.634214641	2.657484566	1.969740568
ENSDART00000087643	tesk2	-0.250604524	-0.077411225	-0.349635246	-0.097357509
ENSDART00000087654	adcy6a	-0.242190723	-0.49624198	-0.145481294	-0.053244608
ENSDART00000087726	igf2bp1	0.052821083	0.330444102	0.755149975	0.389599697
ENSDART00000087857	unc5db	-0.464548804	-0.771046574	-0.267052184	0.0901494
ENSDART00000087884	ccdc85b	-0.309651273	-0.084106622	-0.027008739	-0.094025951
ENSDART00000087991	fndc3bb	0.509196457	0.583942244	0.486760061	0.158477497
ENSDART00000088026	prmt5	0.184159659	0.212388642	0.353449866	0.122961111
ENSDART00000088027	ssx2ipb	-0.254273665	-0.300537366	-0.659689526	-0.365610708
ENSDART00000088033	trpm3	-1.238410284	-1.468940768	-0.88192323	-0.200606191
ENSDART00000088042	myo10l3	0.440398704	0.46516816	0.325219038	-0.023584336
ENSDART00000088093	sipa1l2	-0.401627573	-0.319202181	0.366701976	0.056822769
ENSDART00000088141	ankrd34bb	1.379198636	2.420328938	2.155206475	1.17838067
ENSDART00000088146	ensab	-0.270112525	-0.281607362	-0.344659286	-0.084313447
ENSDART00000088159	nrxn1a	-0.55423697	-0.307440551	0.007663974	-0.036725507
ENSDART00000088178	nrxn1a	-0.424942944	-0.357890595	-0.01594532	0.150075885
ENSDART00000088179	nrxn3a	-0.554627027	-0.919560714	0.023875656	0.130979956
ENSDART00000088199	zgc:162707	-0.481388546	-0.51007365	-0.179950315	-0.039369231
ENSDART00000088240	sypb	-0.232689126	-0.445901517	-0.375392134	-0.149058325
ENSDART00000088249	hcn4l	-0.487910369	-0.340719397	0.02727434	0.039415999
ENSDART00000088270	yjefn3	-0.490178998	-0.350279664	-0.399921095	-0.213170988
ENSDART00000088290	raph1b	0.812305175	0.733830465	0.671238907	0.330964135
ENSDART00000088336	setdb1a	-0.249989255	-0.274821833	-0.108228373	-0.04389578
ENSDART00000088342	cytip	0.001918005	-0.070156162	-0.471206178	-0.369058853
ENSDART00000088364	kif1aa	-0.06544832	0.34113245	0.707853532	0.648494399
ENSDART00000088488	opa3	-0.282490417	-0.240544978	-0.173330249	-0.094645025
ENSDART00000088513	gnl1	0.102638572	0.086645512	-0.243843993	-0.223952657
ENSDART00000088569	nyx	-0.258999398	-0.197132955	-0.26271962	-0.211182996
ENSDART00000088603	UNC13A	-0.361581411	-0.491389738	-0.199237568	0.353821
ENSDART00000088639	wscd2	-0.261498814	-0.268790529	-0.287619442	-0.123420729
ENSDART00000088643	col15a1b	-0.287399924	-0.285830353	-0.360508672	-0.35081124
ENSDART00000088653	prss12	-0.641371677	0.068962263	0.215098805	0.069158882
ENSDART00000088687	rxfp3.2b	-0.990302103	-0.900399551	-0.191860941	0.37665355
ENSDART00000088690	lman2	-0.00791489	-0.046317548	-0.243638306	-0.186515184
ENSDART00000088818	fhod3b	-0.341939339	-0.263444238	-0.179945034	0.005787251
ENSDART00000088833	si:ch73-233f7.1	-1.214678988	-0.552820604	-0.229157577	0.532052231
ENSDART00000088881	git2a	-0.368814104	-0.568589271	-0.278482365	-0.109167728
ENSDART00000088908	srgap1a	-0.173831935	-0.499543132	-0.183148374	0.021193005
ENSDART00000088973	sytl2a	0.735131572	0.479031428	-0.049007315	-0.597552798
ENSDART00000089012	kif1ab	-0.037675888	0.080069758	0.360995087	0.379282318
ENSDART00000089015	zbtb7a	-0.478666207	-0.203919761	-0.738823143	-0.713706626
ENSDART00000089033	lingo3a	-0.421566075	-0.428623374	-0.267183911	-0.077777224
ENSDART00000089042	kcnh4b	-0.293363138	-0.305961812	-0.46205676	-0.207459851
ENSDART00000089076	dot1l	0.094529933	0.165735937	0.448240264	0.463978672
ENSDART00000089079	mpnd	0.049906779	0.784892334	0.951903848	0.691834891
ENSDART00000089126	trhde.2	-0.409075032	-0.405058445	-0.383617798	-0.218104015
ENSDART00000089133	rufy2	-0.198896291	0.087437671	0.267347782	0.283743397
ENSDART00000089141	fsd1	0.140911421	0.537063785	0.66216846	0.489473142

ENSDART00000089158	hmha1a	1.15366088	0.773436896	0.705540127	-0.035689576
ENSDART00000089161	CCDC181	-0.238708752	-0.276570098	-0.364394143	-0.212879757
ENSDART00000089246	elmod1	-0.367956253	-0.511095234	0.063835683	0.117723407
ENSDART00000089325	mief1	-0.272432924	-0.240984001	-0.06454421	0.031387691
ENSDART00000089339	dph7	-0.317220664	-0.267955931	-0.402220665	-0.245139947
ENSDART00000089342	cfap126	-0.196227391	-0.233968621	-0.449699376	-0.526724945
ENSDART00000089408	shdb	-0.379456562	-0.060183647	0.137084622	0.164920585
ENSDART00000089442	klhl5	-0.428861339	-0.43123859	-0.099854083	-0.052484669
ENSDART00000089445	agap1	0.111728293	0.12652166	0.321279752	0.223152778
ENSDART00000089488	sytl5	-0.2422586	-0.383469303	0.0006109	0.143303123
ENSDART00000089526	otc	-0.228950714	-0.50691522	-0.941930968	-0.587900491
ENSDART00000089540	sacm1la	-0.121729942	-0.218997931	-0.253159012	-0.069593401
ENSDART00000089549	fam65a	0.138661993	0.299682143	0.16216271	0.248418418
ENSDART00000089574	tub	-0.338880988	-0.401453479	-0.101730157	-0.038037712
ENSDART00000089577	cacnb4b	-0.344473142	0.166216688	0.479684005	0.375262058
ENSDART00000089699	prrt1	-0.603201784	-0.702306626	-0.280611263	-0.047540659
ENSDART00000089748	rorb	-0.330969943	-0.539847879	-0.425468683	-0.138099942
ENSDART00000089867	ppp2r2cb	-0.452836704	-0.44451904	-0.189867818	-0.082146078
ENSDART00000089923	znf652	-0.30560984	-0.322535846	-0.127141181	-0.092457617
ENSDART00000089961	sik2a	-0.349078539	-0.249421195	-0.402456418	0.05469833
ENSDART00000089967	cacna1bb	-0.202836628	-0.60611593	-0.329318476	-0.282649478
ENSDART00000089968	rasl10a	-0.525764509	-0.884552715	-0.504672449	-0.419223822
ENSDART00000089992	hmgn7	-0.150988875	-0.15001014	-0.251989002	-0.139708395
ENSDART00000089999	b4galt3	-0.174643035	-0.177020263	-0.279641129	-0.298606277
ENSDART00000090010	phex	-0.386748819	-0.231869791	-0.214849122	-0.613717457
ENSDART00000090019	zeb2b	-0.694941899	-0.428168304	-0.296660745	0.024845807
ENSDART00000090079	synm	-0.113622285	-0.399027729	-0.70355851	-0.251171145
ENSDART00000090174	dock9b	0.356010204	0.810416379	0.943578275	0.58912554
ENSDART00000090191	flcn	0.595641364	0.372738928	0.082710169	0.079174241
ENSDART00000090221	cdc42ep5	0.276662145	0.611781241	0.632201471	-0.020036411
ENSDART00000090235	nfixb	-0.570962215	-0.312719979	-0.289246101	-0.209164384
ENSDART00000090252	atp2b3a	-0.293678426	-0.382019678	-0.276234519	-0.034568861
ENSDART00000090266	gpd2	0.305577979	0.440771229	0.397599751	0.146739419
ENSDART00000090292	ctnnd2b	-0.150466015	-0.062584536	0.302491723	0.338163791
ENSDART00000090306	xpr1a	-0.158299732	-0.301120383	0.179396195	0.424625304
ENSDART00000090335	hipk2	-0.356266924	-0.261702878	-0.185362868	-0.057591831
ENSDART00000090397	kiaa1549la	-0.446832614	-0.575803117	-0.232865535	0.10265337
ENSDART00000090406	dock11	0.704689621	0.535408523	0.615472635	0.363574103
ENSDART00000090483	cplx3a	-0.167017721	-0.181539522	-0.397723314	-0.182035461
ENSDART00000090484	tecpr1a	-0.079583722	-0.244227266	-0.30265493	-0.135390116
ENSDART00000090521	ankle2	0.59590492	0.614380049	0.713135872	0.291410446
ENSDART00000090528	rhoca	0.570199757	0.738551724	0.41277501	-0.074138712
ENSDART00000090534	ulk1a	0.04466428	0.367530579	0.126656159	0.034855767
ENSDART00000090548	ASTE1	0.186068328	0.308540808	0.373512724	0.303370928
ENSDART00000090580	si:dkey-215k6.1	-0.353341621	-0.406611115	-0.330167398	-0.116132596
ENSDART00000090596	fgf12b	-0.346267349	-0.034488788	0.704892235	0.660670447
ENSDART00000090611	sh3gl2a	-0.3620314	-0.426828792	-0.627861098	-0.256916064
ENSDART00000090669	pleca	-0.254130273	-0.426440212	-0.236918819	-0.12154508
ENSDART00000090689	brat1	0.263072231	0.449900152	0.263104838	0.157050832
ENSDART00000090709	coq7	-0.258539203	-0.273311663	-0.220704286	-0.134757577
ENSDART00000090711	ltc4s	0.402288552	0.209272555	0.13658761	0.029040107

ENSDART00000090748	pcdh1g9	0.151679401	0.129768355	0.681696549	0.559518569
ENSDART00000090757	kat2b	-0.251752686	-0.26045996	-0.262277009	-0.210249532
ENSDART00000090771	cyth1a	1.102348748	1.012723243	0.839881021	0.442796833
ENSDART00000090844	zgc:153018	-0.414733046	-0.080390611	-0.214556251	-0.032786964
ENSDART00000090864	lmod3	0.210035398	1.148748445	1.650370875	1.365208896
ENSDART00000090874	kcnh7	0.089634054	0.066677874	0.800119794	0.739010611
ENSDART00000090883	gpnmb	1.78024327	1.792109343	1.484400673	-0.264951498
ENSDART00000091004	pcdh1a	-0.630879138	-0.627674521	-0.227167517	0.17418403
ENSDART00000091017	pkn1b	0.119108789	0.041947386	0.390816496	0.137505167
ENSDART00000091021	col10a1a	0.454586287	3.186498396	3.689025905	2.153861861
ENSDART00000091124	aifm3	-0.238811768	-0.279253276	-0.598306352	-0.861256088
ENSDART00000091140	snx21	0.278697916	0.637246869	0.128859029	0.534229621
ENSDART00000091151	nell2b	-0.408104365	-0.533629775	-0.525750199	-0.236120482
ENSDART00000091156	tacc1	-0.166984032	-0.162668339	-0.396514006	-0.330157066
ENSDART00000091158	irg1l	1.781534333	2.6026377	1.369996198	-0.413577442
ENSDART00000091183	erfl3	-0.064022984	-0.222171088	-0.380622737	-0.084018803
ENSDART00000091205	sdk1b	-0.11409829	-0.274173881	-0.261923413	-0.147107702
ENSDART00000091241	si:ch73-22o12.1	-0.053659471	0.086463787	0.414689493	0.463272551
ENSDART00000091252	spata13	0.108055641	-0.023595371	-0.309799561	-0.91906317
ENSDART00000091271	prkg2l	-0.404320096	-0.412762377	-0.544649422	-0.030408858
ENSDART00000091331	prodha	-0.295055194	-0.294720047	-0.215860993	-0.495616796
ENSDART00000091351	gk5	0.51312557	0.289440127	0.273716274	-0.08764101
ENSDART00000091409	smarcad1a	0.370815487	0.374702329	0.447117461	0.290143851
ENSDART00000091416	cntn3a.1	-0.478191534	-0.24621663	-0.434975173	-0.073764559
ENSDART00000091452	TULP2	-0.183522253	-0.233522919	-0.588056807	-0.14814607
ENSDART00000091472	kcnv2b	-0.549604515	-0.416623158	-0.289225353	-0.099467789
ENSDART00000091489	ppp1r9bb	-0.563706295	-0.346664158	-0.080794638	0.037246954
ENSDART00000091508	FAM83G	0.624622597	0.848983587	0.795841019	0.137374674
ENSDART00000091532	ndnf	-0.379105533	-0.357674977	-0.332342823	-0.25490154
ENSDART00000091584	zgc:158785	-0.030208367	-0.597188759	-0.577152486	-0.343586348
ENSDART00000091599	sbf1	-0.602535014	-0.60076227	0.11831949	0.138965635
ENSDART00000091612	dab2ipa	-0.32378487	-0.312766229	-0.160534626	-0.215968275
ENSDART00000091615	iffo1a	-0.255558776	-0.319129649	-0.402938235	-0.1114809
ENSDART00000091620	atp8a1	-0.02813322	-0.262306637	-0.342910163	-0.163047911
ENSDART00000091644	abi1b	0.181595456	0.359192672	0.565695572	0.32038751
ENSDART00000091662	noc2l	0.778877814	0.364993344	0.393512667	-0.105586454
ENSDART00000091664	apc2	-0.58155429	-0.22615315	0.136652181	0.006999355
ENSDART00000091683	alkbh5	-0.400075016	-0.448742364	-0.161106699	-0.178285375
ENSDART00000091707	dbpa	-0.158262702	-0.327851995	-0.357551076	-0.129469939
ENSDART00000091726	fam78ba	-0.542782218	-0.34207093	0.322203074	0.384590985
ENSDART00000091727	ntrk3a	-0.313031117	-0.882724333	-0.168396888	0.114845125
ENSDART00000091729	mlc1	-0.413445193	-0.441446959	-0.597560289	-0.400243567
ENSDART00000091780	rc3h2	-0.185144786	-0.339077629	-0.231755111	-0.132461257
ENSDART00000091818	tulp4b	-0.531343855	-0.277019809	0.128892269	0.551121944
ENSDART00000091899	ccm2l	0.082170149	-0.344528731	-0.637200318	-0.089681617
ENSDART00000091901	psmd14	0.227724961	0.262600592	0.258422041	0.051950672
ENSDART00000091923	slc4a10a	0.0431458	-0.481021076	-0.208884142	0.00832231
ENSDART00000091932	gusb	-0.012758115	-0.102829729	-0.25264355	-0.30752198
ENSDART00000091955	nrxn2b	-0.363238969	-0.38605701	-0.167716958	0.046918181
ENSDART00000092013	tmtc1	-0.379958165	-0.299353758	-0.293525457	-0.123093715
ENSDART00000092050	stab1	0.830009942	0.662655195	0.610953846	0.245694157

ENSDART00000092051	CABZ01081780.1	-0.497076796	-0.683547714	-0.447049069	-0.155316145
ENSDART00000092114	ERBB4 (1 of many)	-0.124937285	-0.553537291	-0.521885412	-0.338309306
ENSDART00000092164	prmt2	0.865577917	0.607769301	0.137454327	0.149744108
ENSDART00000092182	ppm1la	-0.411282562	-0.137536548	0.156346356	0.193635075
ENSDART00000092183	lrrc3b	-0.469734111	-0.850621377	-0.496353665	-0.22903801
ENSDART00000092239	lix1l	-0.01751945	0.310063812	0.327664672	0.131969727
ENSDART00000092250	btbd11a	-0.250819977	-0.42148278	-0.144893414	0.093892491
ENSDART00000092257	PLD5	-0.97635635	-0.828911686	0.040708181	0.36058098
ENSDART00000092270	r3hdm4	0.109330042	0.341009284	0.430965659	0.322117566
ENSDART00000092290	pcdh9	-0.617638559	-1.059395061	-0.632995564	0.015160294
ENSDART00000092356	neto1	-0.404606374	-0.302098716	-0.383296194	-0.272667962
ENSDART00000092357	sgsm2	-0.379055867	-0.375730796	-0.097265456	-0.053118339
ENSDART00000092381	pcloa	-0.033919449	-0.560249847	-0.672386944	-0.219295292
ENSDART00000092389	nup210	0.743287563	0.315006844	0.505674911	0.088834448
ENSDART00000092406	apba2a	0.032423751	0.148342908	0.468800105	0.354794985
ENSDART00000092416	rabl2	-0.237808365	-0.250658742	-0.360133928	-0.144079351
ENSDART00000092435	mgat4a	0.252074722	0.11851857	0.178053587	0.019110648
ENSDART00000092493	ptprt	-0.175495642	-0.444255461	-0.308001112	-0.058909837
ENSDART00000092524	rasa3	-0.348887401	-0.411266738	-0.465367898	-0.079002088
ENSDART00000092646	lrrc73	-0.475821333	-0.175234523	0.140632325	0.202367883
ENSDART00000092647	cers1	-0.35221666	-0.292832737	-0.390981877	-0.135220693
ENSDART00000092665	srebf1	-0.417658309	-0.274360919	0.114636817	0.022214824
ENSDART00000092690	srebf2	-0.007030288	0.441941306	0.773565631	0.436893785
ENSDART00000092691	pea15	-0.578361557	-0.742039167	-0.342285902	-0.180997926
ENSDART00000092884	lrrc58b	0.353547699	0.596933578	0.36096561	0.06120599
ENSDART00000092948	peli1b	0.088983174	0.130189996	0.342697853	0.254903056
ENSDART00000093000	PLEKHB1	-0.366311468	-0.143931789	-0.403311135	-0.393296015
ENSDART00000093003	syt7a	-0.241216791	-0.606859779	-0.288317918	-0.007740028
ENSDART00000093005	DGKI	-0.31222183	-0.268945726	0.173367169	0.253167333
ENSDART00000093093	coro2bb	-0.315281988	-0.251087256	-0.471013192	-0.151819548
ENSDART00000093149	ddx21	0.146331643	0.244276574	0.410170406	0.14971067
ENSDART00000093155	hpse	0.712086441	0.364284216	0.211412521	-0.486650265
ENSDART00000093163	galnt11	-0.148415291	-0.19498093	-0.27912109	-0.157806623
ENSDART00000093166	nrxn1b	0.047890691	-0.072856775	-0.26075298	-0.08007638
ENSDART00000093193	CABZ01090021.1	0.615976841	0.876382917	0.705233449	0.369811976
ENSDART00000093199	tead3b	1.081516431	0.86043279	0.478204597	-0.215799128
ENSDART00000093236	TULP3	-0.257622072	-0.196815312	-0.318097587	-0.153320475
ENSDART00000093279	spi1b	0.891535687	0.563855895	0.100587929	-0.531177734
ENSDART00000093304	nrm	0.448051883	0.19826204	0.059665608	0.061320945
ENSDART00000093310	celf5a	-0.268154664	-0.455004312	0.014815598	0.070405312
ENSDART00000093331	rreb1a	-0.024901133	-0.155149005	-0.517234878	-0.050769944
ENSDART00000097176	CABZ01090749.1	1.277443607	2.06888261	1.727457254	0.676889483
ENSDART00000097194	serinc5	0.184853776	0.289193819	0.374387724	0.244648582
ENSDART00000097198	sc:d217	2.007573379	2.429847079	2.207258628	1.807569646
ENSDART00000097248	aldh2.2	0.244379894	-0.20748537	-0.365189715	-0.463727898
ENSDART00000097249	aldh2.2	-1.049924711	-1.051244298	-1.854940324	-3.57887137
ENSDART00000097330	dnm1b	-0.51283412	-0.480365631	0.101619237	0.531582232
ENSDART00000097338	napaa	-0.21867082	-0.198889828	-0.441263528	-0.268359175
ENSDART00000097359	dnajc25	0.258588311	0.413120409	0.370076595	0.197964188
ENSDART00000097460	hmgcra	-0.174557714	0.058201434	0.38988663	0.431312576
ENSDART00000097466	fam169aa	-0.25560307	-0.389662586	-0.539552997	-0.303655

ENSDART00000097670	ggcx	-0.008080389	-0.071755289	-0.320769539	-0.236029667
ENSDART00000097685	LINGO3 (1 of many)	-0.608919806	-0.847171007	-0.317394062	-0.126526617
ENSDART00000097695	cntnap3	-0.160493697	-0.529313782	-0.088115665	0.248028839
ENSDART00000097731	valopa	-0.109885721	-0.121152137	-0.343824948	-0.490387529
ENSDART00000097738	panx1b	0.147981258	0.33056409	0.233249696	0.121977096
ENSDART00000097770	gc3	-0.462816548	-0.433977737	-0.284956472	-0.124147363
ENSDART00000097792	tnikb	0.209649579	0.123927361	0.351444411	0.3418539
ENSDART00000097822	atp1b2b	-0.302706184	-0.327970639	-0.363261939	-0.05554983
ENSDART00000097934	cdh4	-0.709680135	-0.297434282	-0.207521755	0.118311374
ENSDART00000097935	si:dkey-226m8.10	-0.692305988	-0.817395111	-0.44855289	0.050175879
ENSDART00000097939	zcchc2	-0.095224914	-0.152148503	0.35105086	0.119260586
ENSDART00000098038	dscamb	-0.314235441	-0.377418781	-0.108405688	-0.031209686
ENSDART00000098045	gas1b	-0.225161666	-0.17230943	-0.331649886	-0.52065088
ENSDART00000098057	ftr19	0.135898929	0.299355243	0.876453981	-0.013001669
ENSDART00000098058	ftr22	0.119426338	-0.017860927	0.367700006	-0.042033613
ENSDART00000098072	myhz1.1	-1.045546706	2.305130821	3.388084443	2.053099835
ENSDART00000098082	GJD2 (1 of many)	-0.795483498	-0.746707283	0.031300804	0.456345915
ENSDART00000098173	vapal	-0.239328654	-0.269836839	-0.058452226	-0.037670486
ENSDART00000098209	sirt1	0.078955516	0.10137907	0.304759128	0.182124247
ENSDART00000098263	kctd9a	-0.029411295	-0.237424393	-0.359070767	-0.190010537
ENSDART00000098284	ftr14	0.428599203	0.304151818	1.741077228	-0.09285898
ENSDART00000098285	atf5a	0.558593719	0.754458044	0.445954377	0.160619888
ENSDART00000098311	KCNJ4	-0.486217707	-0.434414335	-0.027097558	0.056787682
ENSDART00000098361	nmba	-1.096273595	-1.192632984	-0.904325972	-1.052265102
ENSDART00000098424	trib2	-0.755876906	-0.434835466	-0.414057266	-0.366344874
ENSDART00000098545	tmem150aa	0.458575321	0.166363456	0.137813682	-0.033806431
ENSDART00000098567	CRAT (1 of many)	-0.211452678	-0.154808037	-0.361957852	-0.341818431
ENSDART00000098571	CD53	0.954510613	0.498572956	0.147686328	-0.376083033
ENSDART00000098575	trim110	-0.538539785	-0.074004136	-0.613819609	-0.257802554
ENSDART00000098590	cyb561a3a	0.500971443	0.776880119	0.690384128	0.607288952
ENSDART00000098616	rwdd	0.47601582	0.122947158	0.180975352	0.051928757
ENSDART00000098627	pros1	-0.136816155	-0.05658809	-0.154307635	-0.425593287
ENSDART00000098639	cntn5	-0.259764888	-0.493723855	-0.530432667	-0.172558087
ENSDART00000098643	tomm5	0.01929525	-0.165199909	-0.247037767	-0.265103531
ENSDART00000098648	gc2	-0.195652835	-0.378422178	-0.402398473	-0.153861921
ENSDART00000098667	camk2b1	-0.575252799	-0.701444666	-0.414382214	0.077165391
ENSDART00000098668	abcc8b	-0.217525237	-0.529877646	-0.409188595	-0.288785069
ENSDART00000098673	ptx3a	-0.211528365	-0.387085724	-0.417540192	-0.397382448
ENSDART00000098727	svopa	-0.499297278	-0.394465259	0.04106692	0.031976449
ENSDART00000098750	pdlim5b	0.340621907	0.643165703	0.410131138	0.075261902
ENSDART00000098840	ralgps1	-0.525377749	-0.528705894	-0.140236147	0.156796103
ENSDART00000098859	neurod6a	-0.523659161	-0.308368621	0.583701544	0.979244768
ENSDART00000098970	lin28a	3.476899763	1.864207832	0.92280153	0.893269961
ENSDART00000098982	h3f3b.1	-0.123835393	-0.18991532	-0.279701511	-0.152040805
ENSDART00000099003	plscr3b	0.864146968	0.903022775	0.644348755	-0.059087289
ENSDART00000099019	tmem91	-0.661722152	-0.699132453	-0.1920109	-0.057277853
ENSDART00000099049		-0.472159773	-0.420532241	-0.250637325	-0.02437969
ENSDART00000099056	gpx4a	-0.526913186	-0.311025548	-0.820323342	-0.895800055
ENSDART00000099089	ggcx	-0.059935848	-0.149142859	-0.333945915	-0.254577798
ENSDART00000099102	sept5a	0.488119217	0.804836681	0.729175054	0.142689277
ENSDART00000099138	ncf2	0.590402106	0.352177279	0.071326562	-0.071022128

ENSDART00000099180	elovl8a	-0.351208319	-0.254455279	-0.702580214	-0.196165774
ENSDART00000099192	htr5ab	-0.290360215	-0.577686019	-0.280786496	-0.015762839
ENSDART00000099202	igsf11	-0.183727476	-0.31314564	-0.202768681	-0.036422441
ENSDART00000099208	asph	-0.288641006	-0.271409423	-0.460024196	-0.387402543
ENSDART00000099235	rnf44	-0.234703995	-0.37643632	-0.256248531	0.012942846
ENSDART00000099244	CDHR2	-0.127835174	-0.277679177	-0.519160965	-0.069385603
ENSDART00000099248	rabggtb	0.075526469	0.438084725	0.330428907	0.205346366
ENSDART00000099283	dalrd3	-0.272196688	-0.367831508	-0.108158836	0.07067938
ENSDART00000099325	si:dkey-27p18.5	-0.821017867	-0.681399645	-0.140207831	0.113491694
ENSDART00000099389	dnlz	0.363796601	0.422259676	0.108203262	0.027839921
ENSDART00000099392	irgq2	-0.080759981	-0.21229475	-0.382096269	-0.042741954
ENSDART00000099476	fam174b	-0.249603276	-0.126773683	-0.376574062	-0.35637543
ENSDART00000099501	masp1	0.656652704	-0.018219479	-0.317919643	-0.733675787
ENSDART00000099528	spry4	-1.005535524	-0.789062042	-0.760146662	-1.480463024
ENSDART00000099532	vmhc	-0.018141777	3.134710049	3.519069106	1.786644694
ENSDART00000099566	si:ch211-244o22.2	0.576141582	0.625409237	0.697152634	0.270183175
ENSDART00000099568	gpr137bb	0.063339268	0.018950684	0.393403815	0.411006
ENSDART00000099607	slc6a17	-0.493487825	-0.358976735	-0.156955324	0.010739948
ENSDART00000099690	fam129ab	1.024572276	1.489725431	1.470862577	0.737761428
ENSDART00000099764	zgc:153031	0.216426658	0.538240257	0.500334626	0.476962119
ENSDART00000099769	ccdc22	0.325453994	0.346355629	0.367424682	0.130188245
ENSDART00000099839	map2k2b	4.411409189	4.089988152	4.160217635	3.732217772
ENSDART00000099849	arntl2	0.982355145	1.78511579	1.609893892	1.255639962
ENSDART00000099869	slc17a7b	-0.359089552	-0.531552161	-0.360552754	0.022409885
ENSDART00000099872	slc17a6b	-0.645728405	-0.242319052	0.108083805	0.273480994
ENSDART00000099891	atp5ib	-0.202173954	-0.194749139	-0.288932188	-0.060278853
ENSDART00000099934	kcnc1a	-0.987934197	-1.510396524	-0.387995788	0.170469595
ENSDART00000099947	samsn1a	0.989297054	0.26224771	0.078735734	-0.489522484
ENSDART00000099977	mex3c	0.161233198	0.169039948	0.509657606	0.094152947
ENSDART00000099978	pdlim4	0.618309518	0.542997573	0.115667653	-0.045450127
ENSDART00000099994	hspa8	0.258700063	0.242999424	0.401354403	0.332565903
ENSDART00000100000	gabra1	-0.401815194	-0.497271272	-0.174154846	0.00256896
ENSDART00000100022	H2AFX (1 of many)	-0.512580891	-0.39296931	-0.220510282	-0.111022185
ENSDART00000100074	pbx3a	-0.314908702	-0.751877287	-0.359257757	-0.077459645
ENSDART00000100103	acss2l	-0.342716857	-0.308956489	-0.115247207	0.030743328
ENSDART00000100110	pak6b	-0.707774149	-0.488636508	0.157100943	-0.07651745
ENSDART00000100117	znf143b	-4.095841085	-0.108331644	-1.7298033	-2.907687665
ENSDART00000100131	si:ch211-242e8.1	0.043404066	-0.450710823	-0.466087638	0.073034701
ENSDART00000100145	lgals9l1	1.730493466	0.73620688	0.17444521	-0.331961456
ENSDART00000100156	agpat4	1.221504112	1.6039826	1.49974957	0.799345848
ENSDART00000100181	sall3b	-0.170454734	-0.209016072	-0.285639823	-0.232809645
ENSDART00000100194	msi2b	-0.174290948	-0.286012891	-0.448717518	-0.171818835
ENSDART00000100223	zgc:91860	-0.412727201	-0.311203152	-0.280203316	-0.243367869
ENSDART00000100234	col2a1a	-0.262380123	-0.070947069	-0.203936285	-0.943179815
ENSDART00000100241	haao	-0.290824489	-0.384992406	-0.679902583	-0.875375738
ENSDART00000100286	fgfr4	-0.349487842	-0.279142199	-0.557520873	-0.83628708
ENSDART00000100287	grk7a	-0.29429259	-0.34179807	-0.766271921	-0.26689268
ENSDART00000100290	napbb	-0.403034353	-0.312472409	-0.341096083	-0.127811116
ENSDART00000100310	dbn1	0.014767176	0.221674025	0.550020524	0.324902581
ENSDART00000100320	dnmt3aa	-0.203167185	-0.31036097	-0.115665251	-0.0614106
ENSDART00000100322	kcnh5b	-0.532935362	-0.444322241	-0.174846578	0.219995697

ENSDART00000100327	nptx1l	-0.348659958	-0.433920996	-0.393251042	-0.286427391
ENSDART00000100332	fgf12b	-0.220479512	0.029676005	0.518232207	0.657400596
ENSDART00000100386	mstnb	-0.232479836	-0.19741782	-0.546065599	-0.487217319
ENSDART00000100401	aars	0.036948074	0.225050642	0.3787132	0.301138312
ENSDART00000100415	map3k7cl	1.690116609	2.517738065	2.168584787	1.392466275
ENSDART00000100438	rab38b	0.552254991	0.331427199	0.26198981	-0.020729573
ENSDART00000100444	fam19a5a	-0.63242802	-0.536601669	-0.265992198	0.12320759
ENSDART00000100453	cerk	-0.133196976	-0.125062952	-0.291644205	-0.1718331
ENSDART00000100458	si:dkey-73n10.1	1.648347098	0.766263068	0.204715566	-0.361349683
ENSDART00000100473	PLIN3	1.275027857	0.887739595	0.535269477	-0.111777778
ENSDART00000100596	pcdh1a6	-0.483816366	-0.257584823	-0.06041051	0.00168094
ENSDART00000100605	ttc32	-0.362267617	-0.196641209	-0.477779827	-0.443477008
ENSDART00000100619	zgc:158803	0.343521186	0.388812734	0.518620429	0.45576524
ENSDART00000100622	slc15a2	1.330001469	0.871350782	0.119367987	-0.187130808
ENSDART00000100639	chrm4a	-0.541979173	-0.464947812	-0.239039654	-0.216630506
ENSDART00000100658	esrra	-0.571475474	-0.395296451	0.169003635	0.212368184
ENSDART00000100667	skia	-0.180939747	-0.108825609	-0.328725059	-0.076853639
ENSDART00000100681	ncam2	-0.42007768	-0.748629002	-0.327699832	-0.08364547
ENSDART00000100743	cttnbp2	-0.40355339	-0.391763552	0.009167842	0.081098
ENSDART00000100762	inpp4ab	-0.155677368	-0.41981961	-0.539565797	-0.358079101
ENSDART00000100798	trip6	-0.01587524	-0.010041711	0.309937262	0.254192292
ENSDART00000100813	rps24	0.369574152	0.327820695	0.143165091	-0.049601165
ENSDART00000100869	ppp3r1b	-0.102347685	-0.214447853	-0.31663745	-0.211814655
ENSDART00000100877	zgc:153142	-0.183846915	-0.253996232	-0.437661908	-0.081274008
ENSDART00000100885	nrn1la	-0.520024314	-0.30927413	-0.690569972	-0.461401545
ENSDART00000100898	tnfsf12	0.387181383	0.213965186	0.122654268	0.108692776
ENSDART00000101014	cx32.2	1.233652332	0.399790197	-0.050586016	-0.568712262
ENSDART00000101037	nhp2	0.465910387	0.391566586	0.240162003	-0.008355829
ENSDART00000101038	tmie	0.304653896	1.158765103	1.299122704	1.103412502
ENSDART00000101044	hsbp1a	0.110662102	0.30138061	0.346862926	0.268898796
ENSDART00000101070	dachd	-0.296270747	-0.351582482	-0.130791564	-0.009393292
ENSDART00000101097	acp6	0.210112925	0.333975464	0.275264967	0.086639765
ENSDART00000101124	rnaseka	1.077486687	0.59165486	0.038549521	-0.354475184
ENSDART00000101134	khdrbs2	-0.661640499	-0.469301828	-0.265343985	-0.029039929
ENSDART00000101142	chsy3	0.236575675	0.054760791	-0.253204551	-0.378519013
ENSDART00000101143	mhc1zea	0.57323499	0.526487775	0.424884066	0.07882824
ENSDART00000101204	alcamb	1.334306533	2.490758317	2.513428399	1.826617796
ENSDART00000101208	abhd11	-0.157962098	-0.321289806	-0.312791151	-0.149912405
ENSDART00000101219	mettl27	0.439044633	0.662527528	0.547984428	0.178832583
ENSDART00000101231	syt7b	-0.85705254	-2.266705376	-0.529986528	0.010282974
ENSDART00000101265	pik3c3	0.317460591	0.079259824	0.186108571	0.079398024
ENSDART00000101282	bcr	0.172989029	0.500540591	0.708792261	0.385177654
ENSDART00000101292	si:dkey-238c7.16	0.35472996	0.602144814	0.450575535	-0.163119012
ENSDART00000101319	zgc:162396	0.344606585	0.405244483	0.227818995	0.060602938
ENSDART00000101394		-0.473756662	-0.114672377	-0.629243212	-2.05184474
ENSDART00000101477	emp1	-0.241905322	-0.037268798	0.203715295	0.377786296
ENSDART00000101513	ppp6r2b	-0.18278033	-0.275899098	-0.242929069	-0.103103109
ENSDART00000101530		-0.360327211	-0.388582556	-0.376333338	-0.16940017
ENSDART00000101537	mex3b	-0.20648165	0.480244705	0.713185315	0.514833911
ENSDART00000101576	tmem230b	0.001436175	-0.176978383	-0.445323711	-0.253116022
ENSDART00000101577	lrrfip1a	0.656945169	0.402220137	-0.125928686	-0.548022342

ENSDART00000101603	kidins220b	-0.01985848	-0.087358266	0.173253678	0.374974923
ENSDART00000101627	IGLON5	-0.423175903	-0.742308046	-0.313681293	0.102626627
ENSDART00000101631	satb1b	-0.360112936	-0.384116906	0.134197196	0.481946288
ENSDART00000101653	CU639469.1	-0.529716959	-0.505372118	-0.050085906	0.195515782
ENSDART00000101658	ppp1r1b	-0.639757199	-0.267805539	-0.098160681	-0.228099263
ENSDART00000101698	rpz3	0.288382306	0.347423497	0.479161923	0.327971533
ENSDART00000101707	dhx40	1.733776583	1.072360854	0.976040329	1.006521884
ENSDART00000101789	flot2b	0.047673595	0.464453756	0.590584487	0.461504068
ENSDART00000101943	rragca	0.302260739	0.264945177	0.40523597	0.261316897
ENSDART00000101948	GJA9 (1 of many)	-0.638779519	-0.432316335	-0.423080448	-0.46097286
ENSDART00000101974	erh	0.03890153	-0.044950443	-0.279975387	-0.23272597
ENSDART00000101982	irg1	0.318182673	0.173060394	-0.011525449	-0.063563032
ENSDART00000101985	zgc:162944	0.341402057	0.426793325	0.183161821	-0.332319729
ENSDART00000102011	G3BP2 (1 of many)	0.183053703	0.292940696	0.414353657	0.391197687
ENSDART00000102062	timp2b	0.538603085	0.084002119	-0.039686845	-0.313946909
ENSDART00000102075	rxrba	-5.032133145	-2.347844506	-3.410287183	-0.630106553
ENSDART00000102111	tpt1	0.26125953	0.357793849	0.090393072	-0.094022362
ENSDART00000102125	schip1	-0.065634739	-0.008431238	-0.356995513	-0.237294266
ENSDART00000102148	ddx3b	-0.056845376	-0.031755759	0.246518412	0.169198996
ENSDART00000102212	tdp2a	1.019634105	0.831370615	2.830207945	0.310574015
ENSDART00000102214	ndufv3	-0.094484605	-0.143659913	-0.265916135	-0.00411612
ENSDART00000102260	si:dkey-222f8.3	0.869989363	1.191058069	0.864781519	0.175715859
ENSDART00000102279	lingo2b	-0.319370755	0.274609796	0.545692974	0.078594547
ENSDART00000102305	cspg5a	-0.238742988	0.181941504	0.751580581	0.677972326
ENSDART00000102368	grin1a	-0.57807298	-0.694484674	-0.374619925	0.025284051
ENSDART00000102384	sesn2	0.221656214	0.707312613	0.831938385	0.650662976
ENSDART00000102411	dctn1b	0.519457248	0.529061234	0.697567556	0.548863629
ENSDART00000102419	igf2bp2a	0.598022036	0.736011973	0.87621145	0.419702221
ENSDART00000102431	CU571081.1	0.38462754	0.22357069	0.170924196	-0.045496543
ENSDART00000102434	ehhadh	0.70922182	0.453174195	0.063745289	-0.562849237
ENSDART00000102445	clasp1a	-0.444419251	-0.511465397	-0.218813787	-0.372606163
ENSDART00000102455	gucy1a3	-0.52073984	-0.506169014	-0.27571082	0.101070807
ENSDART00000102459	rbp2a	3.492314469	2.902874566	1.90821276	1.340524369
ENSDART00000102461	rgs8	-0.536939066	-0.575929959	-0.463779418	-0.057720257
ENSDART00000102520	palm1a	0.447081887	0.96607967	1.214937327	0.924772733
ENSDART00000102539	st8sia5	0.153973405	0.592846857	1.061043515	0.777183018
ENSDART00000102559	zgc:122979	-0.659472503	-0.379774871	-0.282725672	-0.191125017
ENSDART00000102562	ankrd10b	-0.585231519	-0.124121182	0.139197554	0.179030577
ENSDART00000102567	si:ch211-1o7.3	0.865836952	0.255817573	0.19284358	-0.410198985
ENSDART00000102665	aste1a	0.478587949	0.328068557	2.324198666	0.218780766
ENSDART00000102672	nck2a	0.205356684	0.423877669	0.371996615	0.147611944
ENSDART00000102681	pnp5a	0.202802193	0.499095293	0.029150263	-0.463436134
ENSDART00000102712	tgm2a	0.064390317	0.421175856	0.197637738	0.730671181
ENSDART00000102715	tuba8l3	1.032352614	1.505768874	1.918452626	1.697934809
ENSDART00000102767	fbxo9	-0.10612083	-0.188227567	-0.252741487	-0.179227033
ENSDART00000102782	gria2a	-0.472482927	-0.595061919	-0.203521695	0.050111807
ENSDART00000102788	epha7	-0.240787156	-0.359871475	-0.404301639	-0.038479801
ENSDART00000102790	glrba	-0.477875363	-0.33299196	-0.36632817	-0.172063586
ENSDART00000102791	klhl31	1.919909064	3.64974722	3.354681727	2.995697572
ENSDART00000102843	src	0.333102086	0.263030754	0.362117443	0.2047457
ENSDART00000102846	si:dkey-23a23.2	0.194667046	0.692432605	0.589344711	0.460081053

ENSDART00000102868	etnk2	-0.119773861	0.158574082	0.41890552	0.249052751
ENSDART00000102881	fam43b	-0.413239873	-0.283572592	-0.358392622	-0.273570929
ENSDART00000102898	zgc:158258	-0.205000336	-0.263168834	-0.54350319	-0.257007642
ENSDART00000102903	dmd	-0.080183076	-0.276170831	-0.483186786	-0.073725636
ENSDART00000102913	cyp2v1	0.603838095	0.398439801	-0.002186897	-0.384510352
ENSDART00000102952	suz12a	0.614499433	0.610664915	0.72051821	0.430761521
ENSDART00000102969	spock3	-0.0552616	-0.214726057	-0.592493725	-0.285702337
ENSDART00000102981	col8a1a	1.700212586	0.444548046	0.772515926	0.395334934
ENSDART00000103016	zgc:173552	1.605722523	1.309975449	1.476680982	1.096088035
ENSDART00000103043	nsfa	-0.472055845	-0.418381067	-0.313792962	-0.037512478
ENSDART00000103070	cdk17	0.018864851	-0.23502559	-0.366641807	-0.137520544
ENSDART00000103076	arl8bb	0.705280948	0.466975375	0.584557674	0.096505859
ENSDART00000103151	dlgap3	-0.304185948	-0.430889474	-0.103996234	0.188728565
ENSDART00000103267	fam212ab	-0.276107898	-0.473876533	-0.614056911	-0.421775209
ENSDART00000103293	ndufa5	-0.136898371	-0.38263765	-0.527827076	-0.351287298
ENSDART00000103352	vps18	0.165861852	0.202619769	0.265495404	0.159870348
ENSDART00000103365	ociad1	-0.01475249	-0.099734574	-0.308153486	-0.083780451
ENSDART00000103368	rpl22	0.33350855	0.471741899	0.142215466	-0.053336675
ENSDART00000103385	slc25a22	-0.5158616	-0.319809073	-0.784039688	-0.881615638
ENSDART00000103405	gch1	-0.102362632	-0.124839141	-0.313487955	0.056848423
ENSDART00000103407	tmem245	-0.353429601	-0.331655767	-0.089201446	0.066435984
ENSDART00000103448	tbx18	1.78643571	2.579858492	2.64743129	1.939376924
ENSDART00000103450	lactbl1b	-0.310148031	-0.346227281	-0.611014657	-0.245438949
ENSDART00000103463	dnajc2	0.193851448	0.30912042	0.441518195	0.223806707
ENSDART00000103467	zgc:77650	0.216349316	0.315934081	0.173077104	-0.055613825
ENSDART00000103471	khdrbs1b	-0.296913345	-0.271356827	-0.067247312	0.118087564
ENSDART00000103474	tspan13b	-0.348967086	-0.119531637	0.084177739	0.198302304
ENSDART00000103487	zgc:195001	-0.335765581	-0.344851478	-0.707965666	-0.31393888
ENSDART00000103491	rbp7b	2.051254968	1.285649693	0.558375641	-0.074104197
ENSDART00000103526	bicc1b	0.362058268	0.640014631	0.968410254	0.365236005
ENSDART00000103532	kcnh5a	-0.481841968	-0.667651405	-0.403869416	-0.116032422
ENSDART00000103549	skib	-0.170611371	-0.408422527	-0.193614247	-0.134954723
ENSDART00000103586	hdac9b	-0.63802499	-0.480501706	-0.6152094	-0.590506452
ENSDART00000103588	mxa	0.174383297	-0.012993595	1.447594397	0.019311963
ENSDART00000103602	lgals2a	1.886236295	1.628346862	1.325259297	0.487532368
ENSDART00000103622	irf7	-0.049332142	-0.2878598	1.533925917	-0.275107455
ENSDART00000103626	mief2	-0.089955279	-0.188542146	-0.285241779	-0.158709862
ENSDART00000103628	btbd6a	-0.278201553	-0.439382236	-0.547351526	-0.157769223
ENSDART00000103639	arf3a	0.291954045	0.68170241	0.689772164	0.341898272
ENSDART00000103640	hey1	-0.385659584	-0.747117397	-0.392306381	-0.416692063
ENSDART00000103646	kcng2	-0.538883844	-0.646084515	-0.39646557	0.008271529
ENSDART00000103660	clcn7	0.412276024	0.162279088	-0.074638152	-0.273644307
ENSDART00000103704	nap1l4a	0.303924114	0.290352176	0.138900988	0.027879829
ENSDART00000103750	fam131bb	-0.555314971	-0.519589863	-0.143114917	0.156539518
ENSDART00000103753	fn1a	2.705855593	3.095500526	2.8206804	2.414837328
ENSDART00000103754	fn1b	2.49600075	3.130597747	1.885882871	-0.148711913
ENSDART00000103755	fn1b	4.126396153	4.640072685	4.590004259	2.904538972
ENSDART00000103785	ggact.3	-1.209900002	-0.367470534	-1.050388105	-0.531293993
ENSDART00000103795	ggact.1	0.007439004	-0.050660849	-0.406911538	-0.573615848
ENSDART00000103815	stmn2a	0.140137036	1.176009379	1.538144395	1.355356024
ENSDART00000103831	ano10b	0.06580331	0.43549934	0.640553067	0.460128123

ENSDART00000103878	vhl	-0.19558438	-0.426476861	-0.33011461	-0.126927905
ENSDART00000103894	cacng8b	-0.589374624	-0.680390231	-0.379441318	-0.105621743
ENSDART00000103911	CABZ01077217.1	0.385992878	0.554652123	0.464993777	0.2375762
ENSDART00000103922	atat1	0.145026536	0.665460259	0.937418253	0.683690675
ENSDART00000103940	mgat1b	-0.294073166	-0.13896492	-0.278393093	-0.224242354
ENSDART00000103957	exoc3l1	0.634582138	1.052789014	1.334761372	0.478611156
ENSDART00000103969	magi1b	-0.261202469	-0.221356443	0.045512769	0.18273538
ENSDART00000103980	crip2	-0.517107368	-0.364195263	-0.546778441	-0.267901424
ENSDART00000103982	nfe2l1a	0.652859432	0.675562602	0.38648833	-0.18398816
ENSDART00000103992	ttll3	-0.156492299	-0.149773154	-0.299786209	-0.201934967
ENSDART00000104008	dtnbp1b	-0.424284075	-0.457257716	-0.424822836	-0.199214695
ENSDART00000104009	slc30a1b	0.469731317	0.847749615	0.68298124	0.247669117
ENSDART00000104027	rims3	-0.345163808	-0.70584123	-0.314103014	-0.01029762
ENSDART00000104043	dkk3b	0.124905707	-0.440538945	-0.566305744	-1.06504734
ENSDART00000104058	ncalda	0.174416215	0.325917683	0.093263441	-0.133323566
ENSDART00000104135	gabra5	-0.398056951	-0.510180941	0.262181183	0.751061332
ENSDART00000104140	dyrk4	0.302696779	-0.345665012	-0.79115152	0.018427723
ENSDART00000104188	igsf21b	-0.476667576	-0.274511883	-0.073843861	0.013583331
ENSDART00000104234	gjd2b	-0.226334837	-0.451484904	-0.506629051	-0.204050045
ENSDART00000104257	lpin1	-0.263973453	-0.162876624	-0.357103753	-0.3626544
ENSDART00000104279	znf516	-0.151066862	-0.30868422	-0.587761132	-0.246031248
ENSDART00000104289	rab3ab	-0.744433679	-0.710299004	-0.400971594	-0.021261155
ENSDART00000104293	pex5la	-0.678333589	-0.710207201	-0.577955484	-0.480904778
ENSDART00000104298	ndufb5	-0.141095269	-0.212773777	-0.353488244	-0.122828248
ENSDART00000104299	cnp	0.814960421	1.713687269	2.20652149	1.77606709
ENSDART00000104307	eml1	0.215532654	-0.343903678	-0.604837308	-0.271121591
ENSDART00000104317	klf7b	0.389436838	1.156291561	1.140404424	0.652703479
ENSDART00000104322	uchl3	0.256651569	0.374538377	0.322263704	0.147277389
ENSDART00000104327	vsx1	-0.248525466	-0.619641572	-0.034766195	-0.028538746
ENSDART00000104336	tnnc2	0.703924131	2.455705453	2.232729877	1.561721699
ENSDART00000104338	pitpnbl	0.001600172	0.291216057	0.176136036	0.011592783
ENSDART00000104353	atp2b1b	-0.132002912	-0.283527357	-0.37922998	-0.020226682
ENSDART00000104361	si:dkey-56d12.4	0.671595911	0.775149092	0.509958528	0.270735073
ENSDART00000104364	rps15	0.338617661	0.309197703	0.255669723	-0.012990535
ENSDART00000104423	rbbp9	0.243640055	0.525148359	0.333645697	0.149282905
ENSDART00000104475	tmsb4x	0.344214142	0.313013147	0.275125575	0.128255357
ENSDART00000104478	fxr1	0.018053797	0.098113697	0.377704225	0.105513892
ENSDART00000104481	slc17a7a	-0.380361502	-0.377656856	-0.259723773	-0.186490981
ENSDART00000104487	cox4i2	0.027176247	-0.218042175	-0.688187338	-0.440124172
ENSDART00000104496	dusp6	0.185513151	-0.01084107	0.164485744	0.322445555
ENSDART00000104519	stat3	0.6458865	0.459098575	0.438501049	0.201812376
ENSDART00000104520	cnbpb	-0.203757271	-0.252097684	-0.248932139	-0.261165322
ENSDART00000104523	arntl1b	-0.158144123	0.59291526	1.113237486	0.52944604
ENSDART00000104525	kmt2a	0.050001667	0.172870774	0.277996699	0.044684436
ENSDART00000104536	chsy1	0.021777196	-0.175630111	-0.644756219	-0.079001152
ENSDART00000104545	avpr2ab	-0.127228111	-0.325246207	-0.456539735	-1.288721053
ENSDART00000104576	psmd13	0.182320821	0.32294371	0.367344771	0.118433419
ENSDART00000104592	pm20d1.2	-0.022497234	-0.114097556	-0.391118153	-0.365853437
ENSDART00000104612	hmx3a	-0.260599707	-0.468562174	-0.112652566	-0.078307838
ENSDART00000104616	lepr	0.762949533	0.720319043	0.625239528	0.526452567
ENSDART00000104637	si:ch211-81a5.8	0.707959832	0.064083353	-0.292232881	-0.006669302

ENSDART00000104657	mrps21	-0.148781558	-0.231390216	-0.424136936	-0.351598574
ENSDART00000104673	camk1gb	-0.378218392	-0.417447419	0.009394779	-0.088307876
ENSDART00000104674	camk1db	-0.318172003	-0.339895825	-0.49572727	-0.229789777
ENSDART00000104687	pfn2	0.330558178	0.38737784	0.240819292	-0.058450663
ENSDART00000104708	abhd12	-0.134054463	-0.304895231	-0.501902497	-0.262631127
ENSDART00000104712	hmg20a	0.003064442	0.120221197	0.366113134	0.275609671
ENSDART00000104720	si:ch1073-385f13.3	0.258133799	0.237616235	1.745458762	-0.119672944
ENSDART00000104722	cdk5r2a	-0.371112934	-0.317321269	-0.137896092	-0.229143186
ENSDART00000104728	ssuh2.2	0.252481495	0.103488209	1.66757315	-0.204612589
ENSDART00000104730	ifit14	0.457651023	0.403391968	2.518119653	0.537556431
ENSDART00000104750	mllt11	-0.266364682	0.400357279	0.342752209	0.193353613
ENSDART00000104751	mxi1	-0.384587031	-0.210909301	0.22179917	0.104111801
ENSDART00000104791	zgc:153722	0.356828804	0.479757966	0.536710875	0.133740974
ENSDART00000104828	gtpbp2b	-0.421398738	-0.385176207	-0.273058228	-0.083654585
ENSDART00000104835	hps5	0.972059444	0.583168765	0.57443346	-0.161192354
ENSDART00000104845	tapbp.1	3.172722647	3.246630579	3.542395564	2.955685304
ENSDART00000104866	ldhbb	-0.207883318	-0.435615938	-0.519651289	-0.183614515
ENSDART00000104895	rgs7a	-0.76773786	-0.54211529	-0.053731925	0.298916153
ENSDART00000104933	eepd1	-0.181346781	-0.362247495	-0.01782264	0.011720383
ENSDART00000104950	atp1a3a	-0.469887861	-0.514207298	-0.064097938	0.181102676
ENSDART00000104999	ccdc85ca	-0.136040811	-0.297438948	-0.211938794	-0.169428565
ENSDART00000105120	tmem170b	-0.08312029	0.138452609	0.437118287	0.263377377
ENSDART00000105174	stm	3.414215545	0.041843024	-0.026881567	-0.531574221
ENSDART00000105179	zgc:158254	-0.229665852	-0.301892648	-0.223905123	-0.049497491
ENSDART00000105215	emilin1b	1.074845253	1.078203948	0.684747395	-0.035555204
ENSDART00000105323	zgc:162255	-0.250927131	-0.470845634	-0.44137647	-0.557741796
ENSDART00000105399	cbarpb	0.427437854	0.525694666	0.646908744	0.015511077
ENSDART00000105404	cirbpb	0.178847577	0.103859165	0.267594995	0.007629823
ENSDART00000105405	cirbpb	0.001082658	0.185977938	0.46521688	-0.018730312
ENSDART00000105477	lrrtm2	-0.540265042	-0.543880814	-0.470126158	-0.070951006
ENSDART00000105484	si:ch211-216b21.2	-0.960990997	-0.619204359	-0.135410073	0.236742995
ENSDART00000105485	si:dkey-78l4.14	-0.103336903	-0.24562657	-0.381336593	-0.194197896
ENSDART00000105503	rab3gap2	2.422276289	3.562211475	1.182834682	2.012188356
ENSDART00000105545	arl3	-0.028403306	-0.158533169	-0.38830015	-0.193573844
ENSDART00000105561	si:dkey-42p8.3	-0.126414242	-0.359710173	-0.388006213	-0.105772793
ENSDART00000105588	pcdh1b	-0.246217756	-0.446101289	-0.039720059	0.09005245
ENSDART00000105597	si:ch211-129c21.1	3.291282195	4.994402871	3.669154828	0.685912998
ENSDART00000105602	elovl6	-0.283618771	-0.171237628	-0.388002017	-0.174233768
ENSDART00000105608	prkcea	-0.17407648	-0.282581987	-0.238186172	-0.104981942
ENSDART00000105659	mhc1zja	0.668521736	0.771454181	0.462558446	0.066622239
ENSDART00000105667	si:dkey-121a11.3	-0.369671493	-0.58967553	-0.842073302	-0.52980672
ENSDART00000105681	cdc14ab	-0.104501589	-0.239167071	-0.39502699	-0.259112662
ENSDART00000105694	adprm	0.302167589	0.521514455	0.908630272	0.271324554
ENSDART00000105743	ntng1a	-0.262343031	-0.343435025	-0.06227485	0.248931143
ENSDART00000105749	nrros	0.925801893	0.55337814	0.265308462	-0.351361566
ENSDART00000105753	olfm3a	-0.365975807	-0.465135675	-0.139223772	0.043379292
ENSDART00000105767	fhl1a	0.562168934	1.496722819	1.339494009	0.93265064
ENSDART00000105774	ek1	-0.422572147	-0.621215995	-0.219935537	-0.023014864
ENSDART00000105813	krt222	-0.522679169	-0.470823148	-0.650543043	-0.842913393
ENSDART00000105818	guk1a	0.053300944	0.162526517	0.245626369	0.043825221
ENSDART00000105848	si:dkey-42i9.7	0.176745483	0.033035183	1.603344014	-0.243326761

ENSDART00000105854	josd1	-0.238139004	0.044983845	0.263174427	0.210087455
ENSDART00000105866	si:ch73-213k20.5	-0.515754761	-0.874847543	-0.892891327	-0.392973916
ENSDART00000105873	cry4	-0.404336974	0.122755184	0.671823255	0.515326773
ENSDART00000105878	fblim1	1.656130522	1.752691305	1.013125802	0.616877976
ENSDART00000105896	atp2a1l	-1.028985284	2.5381413	3.515981153	2.247616466
ENSDART00000105898	TSTD1	0.614724746	0.98570488	0.724640457	0.337367855
ENSDART00000105903	lsm5	0.135971292	-0.078983757	-0.333257253	-0.331997667
ENSDART00000105932	si:dkeyp-110e4.11	-0.775118699	-0.662012678	-0.111191342	0.348802036
ENSDART00000105942	si:dkey-253d23.3	0.99314399	1.287434296	0.804068321	0.742353386
ENSDART00000105967	cacna2d1a	-0.039509635	-0.246396203	-0.304011671	-0.176720638
ENSDART00000105974	pvalb9	-0.398960737	-0.428084025	-0.618672138	-0.278532192
ENSDART00000105988	si:ch211-156p11.1	0.766312966	0.897687307	0.740122106	0.441957966
ENSDART00000106048	ctnnd1	0.632970644	0.745690204	0.779606908	0.274224061
ENSDART00000106081	BX511034.2	0.124750693	0.347355362	0.433795514	0.117013159
ENSDART00000106096	drd1a	-0.279465728	-0.508847304	-0.2349577	-0.295766526
ENSDART00000106120	ywhag2	0.097311782	0.334968358	0.584303472	0.612363287
ENSDART00000106141	tma7	0.110821999	-0.104393908	-0.337001227	-0.221432499
ENSDART00000106152	top2a	1.478970046	0.796399019	1.005046229	0.716311641
ENSDART00000106166	rx1	-1.001905061	-0.358282402	0.191592029	-0.031411117
ENSDART00000106172	rac1a	0.326396929	0.503439113	0.328932342	0.053159748
ENSDART00000106186	prex2	-0.375401587	-0.276106515	-0.274390577	-0.295066887
ENSDART00000106198	zgc:66433	0.123524201	-0.419463467	-0.600279473	-0.27606853
ENSDART00000106229	zgc:113886	-1.108782489	-3.592108749	-0.501312332	-0.527928714
ENSDART00000106260	ankha	0.083956215	-0.292178887	-0.396040739	-0.042168676
ENSDART00000106566	CABZ01030107.1	2.449267078	1.581540517	1.3239843	0.803203173
ENSDART00000106619	nrn1a	-1.225032386	-1.272422646	-0.361032349	0.032061464
ENSDART00000106680	rai1	-0.196842736	-0.463425257	-0.402954237	-0.12043583
ENSDART00000106690	pcdh2g7	0.533636914	0.291763532	0.532651571	0.481339584
ENSDART00000106704	pcdh2ab1	-0.111727697	0.203588847	0.448801307	0.505664817
ENSDART00000108471	ipo7	0.142918554	0.150506384	0.285667017	0.157707046
ENSDART00000108493	gareml	2.608537205	3.376838547	3.284344596	2.814340641
ENSDART00000108496	clec11a	-1.064574084	-0.301046329	-0.192981843	-0.515534742
ENSDART00000108507	CSMD3	-0.201722796	-0.640032982	-0.257154173	0.075603806
ENSDART00000108535	gabrb1	-1.10767094	-1.249599593	-0.238929019	0.417524871
ENSDART00000108574	gramd1bb	-0.232214076	-0.405839915	-0.354417408	-0.144413075
ENSDART00000108581	si:dkey-17m8.1	0.789679514	0.574820222	0.300394764	-0.045841382
ENSDART00000108596	zgc:162150	0.993292495	1.420859127	1.257973066	0.026357222
ENSDART00000108629	lrch1	-0.183622444	-0.188824595	-0.180380725	-0.322184411
ENSDART00000108655	RHOBTB2 (1 of many)	-0.407905552	-0.427681874	-0.180479384	-0.131684888
ENSDART00000108729	adamtsl2	-0.842317462	-0.70962753	0.040474933	0.841307736
ENSDART00000108736	adam12	-0.319460786	-0.316431452	0.445307645	0.87790832
ENSDART00000108796	stox2b	0.143615187	0.758742406	1.120462456	0.794663921
ENSDART00000108804	brinp1	-0.191216427	-0.292751478	-0.180680558	0.046514423
ENSDART00000108808	frem2a	-0.381822233	-0.539670066	0.662155423	0.974116851
ENSDART00000108814	nrip2	-0.213998541	-0.302896779	-0.276048404	-0.173620142
ENSDART00000108818	arhgap32a	-0.576365369	-0.434660146	-0.258610983	0.095307278
ENSDART00000108831	espnla	0.888146475	0.74973908	0.792131149	0.719500985
ENSDART00000108916	SIPA1	-0.25031723	-0.283192607	0.116871701	0.232178471
ENSDART00000108928	pnpla8	-0.24833123	-0.178721384	-0.052341018	-0.031698962
ENSDART00000108943	shroom2a	0.03299057	-0.127271939	-0.499446332	-0.229050052
ENSDART00000108959	RNF208	-0.615021133	-0.757500788	-0.372077015	-0.309696014

ENSDART00000108963	CABZ01102109.1	0.394501993	0.771748701	0.296144389	0.075524582
ENSDART00000108989	adamts14	-0.185318243	-0.40325002	-0.417840329	-0.182643804
ENSDART00000108990	pex5lb	-0.323313621	-0.539021494	-0.34249137	-0.198103063
ENSDART00000108995	tex2	0.515345319	0.756490823	0.434164856	0.106684566
ENSDART00000109014	GJC1	-0.38880893	-0.552238053	-0.31502822	-0.300328618
ENSDART00000109017	usp31	-0.203091269	-0.25315355	-0.034862909	0.058179546
ENSDART00000109023	fmnl3	-0.186687955	-0.312847961	-0.533074314	-0.280720309
ENSDART00000109029	map6a	-0.330392405	-0.354970167	-0.034172445	0.05340091
ENSDART00000109031	gpr26	-0.593199232	-0.45461936	-0.805315901	-0.585023693
ENSDART00000109037	cdk5r2b	-0.48847507	-0.44246865	-0.592226601	-0.418268347
ENSDART00000109040	gpr158a	-0.331785981	-0.632662732	-0.236983197	0.105308258
ENSDART00000109044	grm8a	-0.470031444	-0.746629562	-0.459544685	-0.031467357
ENSDART00000109062	nckap5l	-0.29308537	-0.083948409	0.248864543	0.105880197
ENSDART00000109065	ccng1	0.498527324	0.814436551	0.266438219	-0.054038693
ENSDART00000109070	gigyf1b	-0.072846918	-0.184795854	-0.274652224	-0.080632892
ENSDART00000109091	mettl1	1.65076712	2.831574782	2.963448936	2.721211005
ENSDART00000109099	ttn.2	-4.072510837	1.236801213	2.302382204	1.171450021
ENSDART00000109124	rspo2	0.077521406	0.271943255	-0.228972258	-0.854369599
ENSDART00000109138	hbegfa	0.956645779	1.991576979	1.27153829	0.330229075
ENSDART00000109147	brsk2b	-0.075048445	-0.279504807	-0.192446814	0.052331491
ENSDART00000109171	rce1a	-0.218282831	-0.358569107	-0.271597779	-0.119151709
ENSDART00000109224	CCKBR (1 of many)	-0.56783642	-0.51463677	-0.498556224	-0.286398879
ENSDART00000109235	bicd2	0.048329883	-0.336297195	-0.602745642	-0.296802706
ENSDART00000109243	sema4bb	-0.591195487	-0.421350422	-0.25044186	-0.118505649
ENSDART00000109249	cenpo	3.029141821	1.947037559	2.256164745	0.98054484
ENSDART00000109252	nudt5	0.602324776	0.61383313	0.580277921	0.203918347
ENSDART00000109257	RASGRF1	-0.570969407	-0.754386918	-0.426488142	-0.098297531
ENSDART00000109259	smarcc1a	0.060519576	0.104503692	0.246223049	0.143325093
ENSDART00000109288	myo16	0.115485168	0.089369719	0.360073499	0.534996054
ENSDART00000109308	si:ch73-60h1.1	-0.222101504	-0.315217201	-0.340339223	-0.206722759
ENSDART00000109314	arhgap10	-0.214061464	-0.171864796	-0.398074347	-0.037252057
ENSDART00000109319	arf1	0.284053831	0.377774948	0.307132687	0.034662092
ENSDART00000109356	klf7a	-0.7063233	-0.659659661	-0.044315321	0.157900055
ENSDART00000109415	blzf1	-4.190621432	-2.580757916	0.161069597	-0.394790047
ENSDART00000109416	smc2	1.321753383	1.108242465	0.857262832	0.308883411
ENSDART00000109420	kcnip1b	-0.712749849	-0.701262469	-0.816924162	-0.561177799
ENSDART00000109425	nkd3	3.023582344	2.734865433	2.70294964	2.317338999
ENSDART00000109432	cercam	0.726258015	0.540687546	0.072936326	-0.232601995
ENSDART00000109440	adamts9	-0.162680626	-0.21784206	-0.326391297	-0.145144869
ENSDART00000109452	sbf1	-0.62469158	-0.583723087	0.106179087	0.127879066
ENSDART00000109464	g0s2	2.993222235	1.499538045	0.588261277	0.527162957
ENSDART00000109485	gal3st3	-0.139388785	-0.282505485	-0.388230748	-0.163229068
ENSDART00000109486	gprc5ba	-0.628238673	-0.569388482	-0.508289237	-0.226450363
ENSDART00000109497	tbxas1	0.141369479	-0.217979555	-0.576549291	-0.268338462
ENSDART00000109507	RAP1GDS1	-0.399835735	-0.351308804	0.076766568	0.119897865
ENSDART00000109511	si:ch211-186j3.6	-0.164289524	-0.357536961	-0.081167433	0.02827477
ENSDART00000109528	adgre5b.3	0.525818313	0.538353042	0.246499359	-0.175742704
ENSDART00000109535	gmps	0.181071124	0.257853381	0.376587367	0.112990813
ENSDART00000109537	snphb	-0.466057723	-0.636087511	-0.318006337	-0.040021342
ENSDART00000109546	unc5b	-0.076752071	-0.101810869	-0.423560106	-0.292578205
ENSDART00000109552	baz2ba	0.627489244	0.525669422	0.712410774	0.393101067

ENSDART00000109567	nhsa	-0.05015222	-0.331046598	-0.348641258	-0.190382203
ENSDART00000109568	pip4k2ab	-0.31785183	-0.37156543	-0.209120418	0.064881056
ENSDART00000109570		-0.315520739	-0.355339215	-1.137398742	-0.355379016
ENSDART00000109573	akap6	0.927127599	1.241947706	1.04133516	0.789730231
ENSDART00000109581	abtb1	-0.353930406	-0.557673054	-0.197302182	-0.044180273
ENSDART00000109604	palmdb	-0.228743892	-0.477529612	-0.779896236	-0.307691368
ENSDART00000109615	tmem255a	0.198392818	0.721353886	0.92551731	0.71998369
ENSDART00000109698	nacc1b	-0.541808458	-0.507646097	-0.425509734	-0.201090141
ENSDART00000109714	usp53b	-0.259276756	-0.08547014	-0.255057866	-0.127997302
ENSDART00000109732	amigo1	-0.336374535	-0.536025942	-0.442802906	-0.174075192
ENSDART00000109750	rap2aa	0.293487605	0.482823125	0.662590656	0.396980886
ENSDART00000109752	serpinh1a	0.578805227	0.380412306	0.080680913	-0.569755212
ENSDART00000109759	tmx2a	2.647169883	3.21425121	2.659532867	1.569734674
ENSDART00000109807	EMILIN3	0.322021142	0.194240185	0.000409902	-0.0946206
ENSDART00000109822	hps1	0.431913269	0.483030578	0.349219151	-0.038818149
ENSDART00000109831	BX088653.1	0.96716269	0.47589947	0.338658984	-0.013815104
ENSDART00000109833	zmat3	-0.462380241	-0.737159777	-0.151865097	0.090633241
ENSDART00000109853	zmp:0000000801	-0.4485216	-0.462342097	-0.503236916	-0.960013508
ENSDART00000109872	cln6a	0.07921086	-0.018544414	-0.27320282	-0.083540486
ENSDART00000109876	sun1	-0.255190881	-0.339472925	-0.466317308	-0.169389668
ENSDART00000109973	tp53bp1	0.061139928	0.539512942	0.657824217	0.222950524
ENSDART00000109990	abhd15a	-0.466192052	-0.318128979	-0.316342009	-0.301397533
ENSDART00000110004	frmpd3	-0.530320903	-0.511652794	-0.387982186	0.071931622
ENSDART00000110005	cdhr1a	-0.099288941	-0.157780229	-0.305559135	-0.101115232
ENSDART00000110016	ube2ql1	-0.274241146	-0.513839036	-0.485900432	-0.131799131
ENSDART00000110033	abhd8b	-0.069420963	-0.144372686	-0.35601389	-0.098305508
ENSDART00000110040	sox11a	1.573346229	1.723237215	1.23195588	0.781584101
ENSDART00000110041	lrrc38a	-0.167375954	-0.314913504	-0.608219505	-0.293673506
ENSDART00000110061	spock2	-0.746309396	-0.624301954	-0.158480844	0.116789254
ENSDART00000110064	plaua	1.293433748	1.730730518	1.0162544	0.507140212
ENSDART00000110069	CABZ01075131.1	-0.117445692	-0.062527677	0.307574487	0.429328576
ENSDART00000110076	gas1a	-0.414278752	-0.328067296	-0.317423655	-0.582681301
ENSDART00000110080	aatka	-0.15444172	-0.531359629	-0.399426039	-0.066680489
ENSDART00000110092	mdga1	-0.307586742	-0.496736277	-0.237358341	-0.048338544
ENSDART00000110100	hint3	0.280351117	0.473623985	0.484346588	0.216760955
ENSDART00000110126	cacng3b	-0.394325343	-0.354117689	-0.012321564	0.185829622
ENSDART00000110136	cntnap5b	-0.584561492	-0.553235513	-0.330365654	0.070638148
ENSDART00000110204	pikfyve	0.281293639	0.125670374	0.334758542	0.135267095
ENSDART00000110224	mon1bb	-0.275995109	-0.242724773	-0.120595351	-0.023350049
ENSDART00000110241	cnksr2a	-0.309766586	-0.445156957	-0.12266438	-0.140501824
ENSDART00000110270	pwp2h	0.562435944	0.598132459	0.373677258	0.062993981
ENSDART00000110278	rassf9	-0.213149801	-0.230795795	-0.114749715	-0.801660509
ENSDART00000110279	si:dkey-183c6.8	-0.455316373	-0.174866477	0.555526708	0.790514442
ENSDART00000110331	PTGFRN (1 of many)	-0.468065048	-0.642664771	-0.293257997	-0.171150885
ENSDART00000110370	nrcama	0.101341326	0.125318979	0.288644016	0.298095665
ENSDART00000110377	zgc:194398	-0.335442291	-0.298995581	-0.514027337	-0.216616028
ENSDART00000110383	map7a	-0.540958501	-0.669475763	-0.490700501	-0.102714716
ENSDART00000110409	frmd5	-0.554751162	-0.59840736	-0.384150671	0.071106799
ENSDART00000110411	cerkl	-0.014858902	-0.169171069	-0.284528511	-0.188701157
ENSDART00000110416	eif2b5	0.075177684	0.238615258	0.255578096	0.114861074
ENSDART00000110424	si:ch211-153b23.4	0.636187514	1.795807227	1.178477605	-0.329338933

ENSDART00000110431	si:ch73-280o22.2	-0.128270202	-0.267395445	-0.366965125	-0.13375727
ENSDART00000110432	kazna	-0.433352738	-0.292283864	-0.217890679	-0.318143053
ENSDART00000110447	slitrk3b	-0.707318961	-0.905108657	-0.279729387	-0.217159944
ENSDART00000110458	fam117ab	0.691322185	0.501468791	0.171633838	-0.275806067
ENSDART00000110478	zgc:174906	0.44828184	1.063594358	0.857934554	0.434989632
ENSDART00000110497	tmem63a	0.205919412	0.504153398	0.414725435	0.171827856
ENSDART00000110503	adam11	-0.835498408	-0.874358127	0.055154913	0.615136144
ENSDART00000110512	ybx1	0.187678935	0.306636132	0.543417204	0.157462635
ENSDART00000110522	tulp1a	-0.068953976	-0.228376734	-0.263123945	-0.07738281
ENSDART00000110529	bub1bb	1.321570387	1.337769105	1.007493385	0.59867765
ENSDART00000110544	znf219	-0.234403747	-0.210413301	-0.211690001	-0.32688667
ENSDART00000110547	ANKRD34A	-0.459666228	-0.579840169	-0.409587984	-0.250229823
ENSDART00000110571	fam150a	-0.089437558	-0.463371816	-0.643350488	-0.328581938
ENSDART00000110588	cdh26.1	1.937401648	2.777818936	3.097440102	2.219591598
ENSDART00000110590	VSTM2B	-0.57346103	-0.627600936	-0.34222959	-0.040178972
ENSDART00000110595	si:dkey-188g12.1	-0.689058181	-0.354561593	-0.987489008	-0.761086303
ENSDART00000110606	abhd16a	0.034068276	-0.290276411	-0.385494504	-0.249441823
ENSDART00000110622	si:ch211-222l21.1	0.830701135	0.950120833	0.646058632	0.069324807
ENSDART00000110627	EPB41L1	0.536765101	1.012243023	0.739789984	0.259906496
ENSDART00000110633	cdon	-0.443468077	-0.07594048	-0.652877368	-0.650457598
ENSDART00000110679	lrfn5b	-0.958299909	-0.447121499	0.131848444	0.317540452
ENSDART00000110691	wnt6b	2.452564896	1.637817496	0.779324842	0.681102996
ENSDART00000110696	mxb	-0.714840475	-2.62990329	3.629462021	-0.04990443
ENSDART00000110734	FAM163A	-0.371556039	-0.423320211	-0.272260914	0.021575603
ENSDART00000110751	tiam1a	-0.473694473	-0.373415926	0.157725394	0.265421677
ENSDART00000110777	eef1db	0.19406492	0.18930318	0.407362874	-0.014164442
ENSDART00000110789	esyt2b	-0.389945342	-0.213760019	-0.33183342	-0.035634516
ENSDART00000110804	hspb15	0.646957051	1.106828293	1.50955981	0.885055615
ENSDART00000110814	fam83b	-0.195583845	-0.26819738	-0.2682294	-0.101487813
ENSDART00000110821	ttc19	-0.356526006	-0.209564592	-0.274076967	-0.065932254
ENSDART00000110824	wdr17	-0.247986884	-0.387190174	-0.468364816	-0.181828903
ENSDART00000110835	myo18aa	-0.29501199	-0.246090679	-0.193185897	-0.027559028
ENSDART00000110842	rps2	0.387589328	0.42480999	0.452974598	0.136625115
ENSDART00000110848	HBZ (1 of many)	0.826283934	0.913969919	1.466570031	0.062329776
ENSDART00000110854	elk1	-0.317422178	-0.365267467	-0.144808984	-0.160403171
ENSDART00000110866	cln5	0.673394988	0.327334357	0.152385216	-0.174612372
ENSDART00000110879	magi2b	-0.318025437	-0.557603079	-0.245924365	-0.15150652
ENSDART00000110883	akap12b	0.700660963	1.064613646	0.897645503	0.160893575
ENSDART00000110884	zmiz1a	-0.245374026	-0.039847801	0.243895684	0.299607928
ENSDART00000110923	cnksr2b	-0.347259219	-0.644524762	-0.33094872	0.06260613
ENSDART00000110935	si:ch1073-59l16.1	1.356882908	0.676384004	0.249167454	0.069788375
ENSDART00000110964	bag6	0.132774646	0.150367292	0.354477336	0.199439987
ENSDART00000110974	zdhhc12b	-0.321463982	-0.336778233	-0.359206693	-0.209775575
ENSDART00000110976	col19a1	-0.113310455	-0.554338462	-0.336015886	-0.135833904
ENSDART00000110994	sqlea	0.510664466	1.875874264	2.415595993	1.866281513
ENSDART00000111002	si:ch211-74f19.2	0.463826256	0.294740419	-0.053551592	-0.452436201
ENSDART00000111021	si:dkeyp-14d3.1	-0.147889699	-0.589075251	-0.655456899	-0.257575305
ENSDART00000111025	boc	-0.443385595	-0.283925154	-0.350248278	-0.500405723
ENSDART00000111055	kcna4	-0.237309303	-0.448087799	-0.436530246	-0.275701844
ENSDART00000111058	mpx	0.612551173	0.415979564	0.081520104	-3.505775593
ENSDART00000111080	adgrb3	-0.692483265	-0.604853517	-0.34586076	-0.181234915

ENSDART00000111088	depdc5	0.225009609	0.230516632	0.305363773	0.172290661
ENSDART00000111108	CABZ01071972.1	-0.309847536	-0.540379608	-0.256707009	-0.057294184
ENSDART00000111111	nlgn4b	-0.558022607	-0.771924705	-0.300759795	-0.167214796
ENSDART00000111131	elfn1b	-1.012125284	-0.755320746	-0.301892543	0.27076153
ENSDART00000111140	rpl29	0.336664553	0.356246951	0.105865493	-0.061220703
ENSDART00000111146	si:ch211-26b3.4	-0.414256231	-0.420417531	-0.178854692	0.040407002
ENSDART00000111156	pdp1	-0.641430349	-0.338633085	-0.014499951	0.034332362
ENSDART00000111165	best2	-0.15773889	-0.249582303	-0.643700083	-1.151697377
ENSDART00000111189	gar1	0.542751892	0.313383695	0.267097115	0.089542944
ENSDART00000111203	tagln3a	-0.530904757	-0.489741378	-0.221881281	-0.037658487
ENSDART00000111213	ubash3bb	-0.454832578	-0.295198237	0.117674424	0.358244958
ENSDART00000111234	gdpd5a	-0.267734676	-0.323349922	-0.30731905	-0.184332435
ENSDART00000111246	cadm3	-0.068241621	-0.322804819	-0.138950978	-0.087560594
ENSDART00000111261	cdh24b	-0.501530291	-0.712397312	-0.362295258	-0.168930493
ENSDART00000111271	CABZ01080074.2	0.091182245	-0.411344825	-0.551440399	-0.297376381
ENSDART00000111278	sorcs2	-0.061568527	0.240045859	0.726875042	0.744539967
ENSDART00000111301	luzp2	-0.629816567	-0.548346899	0.040106065	0.088332731
ENSDART00000111303	rfx7	0.075232989	0.085195342	0.459783838	0.727409902
ENSDART00000111308	bbs9	-0.095835492	-0.30952763	-0.39815573	-0.067617558
ENSDART00000111321	zgc:152830	0.702895787	0.346388089	0.27465484	0.003141495
ENSDART00000111323	AMIGO3 (1 of many)	-0.274114526	-0.487248865	-0.174614423	-0.21591611
ENSDART00000111324	zgc:193807	2.638791453	3.489955154	3.039693941	3.396234279
ENSDART00000111343	lmf2a	-0.264313993	-0.256081583	-0.425329386	-0.228346273
ENSDART00000111374	ndufs7	-0.171726296	-0.309298972	-0.568241927	-0.290550046
ENSDART00000111400	kel	0.291873396	0.332474665	0.393429393	0.121747313
ENSDART00000111435	hdac5	-0.290855518	-0.361511948	-0.278953131	-0.082259238
ENSDART00000111438	mgea5	-0.307275123	-0.024811963	0.160874404	0.159343333
ENSDART00000111444	ppip5k1a	-0.365205244	-0.30690644	-0.053146194	0.037826946
ENSDART00000111453	myo19	-0.517639897	-0.807225171	-0.660718539	-0.325322766
ENSDART00000111454	UBA6	-0.095626975	0.120665542	0.496662318	0.328619297
ENSDART00000111456	rbm10	-0.2435311	-0.244375112	-0.319750026	-0.111931812
ENSDART00000111475	pcdh7a	0.109274008	0.268985487	0.465377244	0.354118271
ENSDART00000111480	bcorl1	0.013917394	0.173170994	0.344992278	0.232054818
ENSDART00000111506	lrrc75ba	-0.649545281	-0.408918781	-0.064365089	0.060080923
ENSDART00000111509	CABZ01072096.1	-0.19271783	-0.37213696	-0.269302922	-0.183488656
ENSDART00000111531	epdl1	1.357192284	1.365774088	0.753975689	0.235060778
ENSDART00000111535	elfn1a	-0.273088167	-0.336173663	-0.097567542	0.139029668
ENSDART00000111536	rmdn2	-0.153020266	-0.402861584	-0.346146607	-0.222410217
ENSDART00000111539	efcc1	-0.101491995	0.211154978	-0.621378561	-0.272291709
ENSDART00000111555	fbxo10	-0.524035405	0.01323878	-0.15955536	0.008552029
ENSDART00000111561	zmp:0000000735	0.220285812	0.110420777	1.549961519	0.06406158
ENSDART00000111571	rap1gap2b	-0.543266541	-0.656699315	-0.515603594	-0.222139298
ENSDART00000111625	ninl	-0.236310585	-0.326810382	-0.437556672	-0.154099243
ENSDART00000111636	DTX4 (1 of many)	-1.011696111	-1.148570348	-0.809918586	-0.692193186
ENSDART00000111639	rereb	-0.168137061	-0.390988508	-0.080335114	0.086629083
ENSDART00000111641	morn4	0.104472779	0.38320597	0.615725038	0.556227667
ENSDART00000111642	brinp3a.1	-0.710663483	-0.69660402	-0.30207639	-0.147819903
ENSDART00000111656	gpr78a	-0.727925057	-0.963113505	-0.708360358	-0.204121228
ENSDART00000111666	npdc1a	-0.090316422	-0.266587539	-0.30135563	-0.100212297
ENSDART00000111671	mri1	1.003264298	0.870246845	1.443034807	1.04475049
ENSDART00000111680	iqsec2b	-0.255745447	-0.305278608	-0.149665745	-0.145530664

ENSDART00000111688	zgc:109934	0.61674072	0.212807687	-0.164219088	-0.482167701
ENSDART00000111706	si:dkey-108k21.14	0.724790653	1.05338345	1.058888091	0.439899526
ENSDART00000111707	cacnb1	1.693120335	2.349827744	2.415610432	1.652036859
ENSDART00000111717	fscn2b	-0.120313044	-0.274854028	-0.483029563	-0.127989104
ENSDART00000111724	hist1h4l	0.596018889	0.814003935	0.407366731	0.206389719
ENSDART00000111748	pcdhb	0.251557806	0.39162054	0.85552916	0.748922663
ENSDART00000111753	vmp1	3.174699109	3.199538621	2.234258919	1.662616904
ENSDART00000111758	si:ch211-165d12.4	0.498072983	0.272355872	-0.037512583	-0.064496932
ENSDART00000111759	zmp:0000000794	-0.428447438	-0.466968786	-0.39807432	-0.23111401
ENSDART00000111767	si:ch211-39k3.2	-0.094085509	-0.095643794	-0.600534706	-0.396103136
ENSDART00000111799	HEPACAM (1 of many)	-0.300776869	-0.301240305	-0.377807505	-0.329578876
ENSDART00000111806	cenpe	1.006157891	0.692740621	0.695639634	0.175054418
ENSDART00000111823	GRIK3	-0.298812703	-0.633195231	-0.313411843	-0.167732562
ENSDART00000111841	BX248501.1	-0.587056195	-1.220156186	-0.870300177	-0.776464497
ENSDART00000111842	sall1a	-0.3702182	-0.245271728	-0.043742586	0.007554245
ENSDART00000111880	PDE2A	-0.027201032	-0.255047431	-0.265146028	-0.145249945
ENSDART00000111905	BX649498.1	-0.175767299	-0.206924623	-0.494153858	-0.425985895
ENSDART00000111923	ajuba	0.639088613	1.058968297	0.866425943	0.103913512
ENSDART00000111948	sez6l2	-0.5142749	-0.140866125	0.323444655	0.419947542
ENSDART00000111966	arhgef10lb	0.250651161	0.391053857	0.470093443	0.290981355
ENSDART00000111982	sgce	-0.25805142	-0.148921708	-0.040980656	-0.017549451
ENSDART00000111993	si:dkey-19b23.15	-0.211537347	-0.357802737	-0.511679441	-0.364770311
ENSDART00000112003	adgrb1a	-0.32135813	-0.631154823	-0.317944877	0.097147786
ENSDART00000112028	si:ch211-180f4.1	2.108953042	1.952679055	1.909638244	1.352861352
ENSDART00000112032	ARHGAP22 (1 of many)	0.396989069	0.894459004	1.264783984	1.011214691
ENSDART00000112075		0.743523521	0.909798042	1.147105824	0.978802209
ENSDART00000112079	tlcd2	0.75377096	1.111252223	1.10627033	0.372244628
ENSDART00000112106	fam155a	-0.397020883	-0.419375873	-0.258307362	-0.051593808
ENSDART00000112116	ctss2.1	1.048314865	0.112315631	0.133571721	-0.538227279
ENSDART00000112127	dnase2b	0.959684518	0.954914684	0.226944152	-0.0439051
ENSDART00000112152	gas7a	-0.397395182	-0.5126218	-0.479089603	-0.475145138
ENSDART00000112155	rrm2	1.131329801	1.215867796	0.761186289	0.339828406
ENSDART00000112156	si:ch211-253b8.5	-0.087783514	-0.192500909	-0.49330541	-0.306875488
ENSDART00000112166	fam60al	-0.06751471	0.448215095	0.685621255	0.373624581
ENSDART00000112170	unm_hu7912	-0.404814593	-0.573062573	-0.192411138	0.071188401
ENSDART00000112183	cep170b	0.178654359	-0.17509023	-0.407988469	-0.254556637
ENSDART00000112201	mat2aa	0.26207582	0.205858664	0.104925379	0.050770536
ENSDART00000112211	si:ch211-264f5.2	0.548989898	0.369154347	-0.087668194	-1.075571179
ENSDART00000112227	BRINP3 (1 of many)	-0.375889079	-0.310381341	-0.391723747	-0.183098379
ENSDART00000112243	crlf1a	1.589627089	0.916105309	0.558277873	-0.020827681
ENSDART00000112270	tanc2a	-0.071755517	-0.057687988	0.274000197	0.309857736
ENSDART00000112287	gas2l1	-0.30204098	-0.119087794	0.361891535	0.246536224
ENSDART00000112296	si:ch211-152n14.4	-3.416151322	-1.061691573	-0.948841593	-2.14718796
ENSDART00000112299	gpr37a	-0.365323083	-0.2884934	-0.319868677	-0.150652035
ENSDART00000112301	trhde.1	-0.264986832	-0.332160295	-0.182975887	-0.056935825
ENSDART00000112312	lrch2	0.101952948	0.206054969	0.497048672	0.404162087
ENSDART00000112313	wdr43	2.967338106	2.621161906	3.405726008	2.58425126
ENSDART00000112330	rassf7a	-0.0890413	-0.097498745	-0.456633564	-0.220562537
ENSDART00000112333	cnnm2b	-0.413171039	-0.296279679	-0.539640998	-0.529524127
ENSDART00000112370	ano11	-1.080551268	-1.39069517	-0.608655336	0.046438031
ENSDART00000112407		-0.278472028	-0.07282662	0.029836447	-0.030627475

ENSDART00000112414	rapgef5a	-0.533059964	-0.605979704	-0.027243653	0.007460247
ENSDART00000112438	si:ch73-335m24.5	-0.12977304	-0.024649938	0.356284768	0.481799029
ENSDART00000112441	tlr7	-0.49393668	-0.654080017	-0.57399465	-0.158959649
ENSDART00000112445	tns2a	0.230856175	0.361046363	0.629539855	-0.041949691
ENSDART00000112460	dolk	0.234400054	0.274257795	0.504999751	0.504335897
ENSDART00000112470	ccdc88b	0.86030789	0.601824136	0.501988839	-0.28013627
ENSDART00000112484	nlgn2b	-0.707313853	-0.645741453	-0.193542068	0.120518294
ENSDART00000112493	syt6a	-0.290141292	-0.351590983	-0.39435722	-0.242348488
ENSDART00000112495	zgc:110340	0.095667849	-0.009447766	0.241257465	0.332404774
ENSDART00000112529	znf319a	-0.271600655	-0.228932764	-0.01869534	0.01981331
ENSDART00000112546	pkn3	-0.415654116	-0.225897422	-0.124759806	-0.079978695
ENSDART00000112550	mapk9	0.429625805	0.878911558	0.999507915	0.700660398
ENSDART00000112555	spc25	0.851542956	0.93394939	0.260782437	-0.118635043
ENSDART00000112567	ppfia4	-0.303784132	-0.279792224	0.037275964	0.113653516
ENSDART00000112579	scg2b	-0.360046965	-0.210825369	-0.351760362	-0.440018453
ENSDART00000112589	efna2b	-0.324619544	0.075961218	0.539972471	0.527283417
ENSDART00000112598	otud4	0.000834797	0.118841749	0.673957038	0.019221004
ENSDART00000112646	CNNM1	-0.428104026	-0.544400388	-0.592385966	-0.267977747
ENSDART00000112653	wdfy4	0.841684139	0.566530892	0.08064366	-0.41519717
ENSDART00000112655	nfasca	-0.501256457	-0.441261682	-0.289256026	-0.116172993
ENSDART00000112659	ipp	0.043576539	0.16998945	0.371873925	0.242338779
ENSDART00000112671	bicd1a	-0.391567366	-0.28876668	-0.040397452	0.195532163
ENSDART00000112694	fam171a2b	-0.001835186	0.12088862	0.541529951	0.657778888
ENSDART00000112698	RNF14 (1 of many)	0.142790619	0.542852416	0.218671557	0.088301354
ENSDART00000112711	kcnab1b	-0.291029319	-0.341790858	-0.379711216	-0.329063836
ENSDART00000112728	tmem175	-0.092618029	0.36107371	0.426939782	0.221686367
ENSDART00000112735	fam19a1a	-0.689638571	-0.889095001	-0.657230812	-0.483951597
ENSDART00000112743	nmbb	-0.255890888	-1.177861162	-0.767211345	-0.814299265
ENSDART00000112768	ubald1a	-0.163342676	-0.260286427	-0.366999537	-0.147535942
ENSDART00000112789	AKAP13 (1 of many)	0.092610458	-0.131036675	-0.325814147	-0.202546922
ENSDART00000112845	zgc:195173	-0.156651959	-0.182049019	-0.580426063	-0.462296521
ENSDART00000112856	TNC (1 of many)	-0.33689758	-0.285587425	-0.51132075	-1.127367477
ENSDART00000112883	fmnl2b	-0.095811604	0.162122972	0.557976718	0.595265469
ENSDART00000112895	FNDC10	-0.46103084	-0.039905964	0.379016706	0.57822448
ENSDART00000112900	si:ch211-150i13.1	-0.166104961	-0.246781433	-0.279012086	-0.114516059
ENSDART00000112909	tex2l	-0.590392514	-0.678584931	-0.756484503	-0.42899031
ENSDART00000112926	adora1b	0.387978003	0.609753862	0.576471225	0.261995134
ENSDART00000112956	slc9a6b	-0.347273739	-0.284992324	-0.155606241	-0.015875628
ENSDART00000112959	ankrd1a	2.226071129	3.68265785	3.832168007	2.790435602
ENSDART00000112967	rilp	-0.073950343	-0.174960248	-0.511756745	-0.145692497
ENSDART00000112987	stk32a	-0.326877709	-0.547605605	-0.241724362	0.046529539
ENSDART00000113004	CABZ01068356.1	-0.41749624	-0.503262627	-0.597219984	-0.132734415
ENSDART00000113058	gpsm2	-0.158548143	-0.583640034	-0.403831438	-0.106682634
ENSDART00000113077	FERMT3 (1 of many)	0.719070873	0.278758571	0.151005206	-0.238328508
ENSDART00000113081	gpr158b	-0.777838913	-0.859733549	-0.109236002	0.425659764
ENSDART00000113087	hmx1	-0.389387565	0.021377927	0.234018679	0.270888749
ENSDART00000113089	slc4a11	-0.230777933	-0.44932083	-0.596234565	-0.271629672
ENSDART00000113092	abhd10b	0.399222781	0.423215001	0.172340204	0.123654926
ENSDART00000113093	gba	0.473554928	0.246228233	-0.028007284	-0.118288896
ENSDART00000113097	hsd17b7	-0.488876774	0.314769091	0.517151777	0.148788215
ENSDART00000113098	hbaa1	0.802810656	0.919517173	1.438314563	0.102144076

ENSDART00000113101	smarcad1b	0.481835623	0.651517984	0.685097173	0.491069329
ENSDART00000113112	wscd1b	0.189205134	0.487630626	0.094129579	0.021242458
ENSDART00000113135	msl1a	-0.136399644	-0.187672093	-0.188075662	-0.302625769
ENSDART00000113142	phldb1b	0.143763621	0.070606141	0.367437708	0.295748576
ENSDART00000113162	pi4kaa	-0.145989753	-0.358429295	-0.235575632	-0.007193852
ENSDART00000113166	si:ch1073-159d7.7	2.749837608	3.675596621	2.897443721	1.850742921
ENSDART00000113171	nlgn1	-0.287475058	-0.532070389	-0.095767589	0.228264127
ENSDART00000113193	cbln2a	-0.405462517	-0.494893494	-0.545170143	-0.282281473
ENSDART00000113196	si:ch211-157b11.14	-0.299973945	-0.39773998	-0.291303207	-0.069868393
ENSDART00000113197	prok1	-0.431580361	-0.477066939	-0.390033973	-0.689815046
ENSDART00000113241	tmem163a	0.329551441	0.273820345	0.385569684	0.333416277
ENSDART00000113246	si:ch211-102c2.4	0.606357076	0.357270403	0.232028808	-0.115778826
ENSDART00000113248	b3galt1b	-0.287109392	-0.341327689	-0.370081074	-0.245129506
ENSDART00000113259	TARBP1	0.600840202	0.638996908	0.05884907	-0.019773553
ENSDART00000113280	FRMD5 (1 of many)	-0.439670364	-0.372637017	-0.247112316	-0.218940122
ENSDART00000113301	si:dkeyp-41f9.3	-0.138197975	-0.385879672	-0.585370915	-0.421818221
ENSDART00000113311	col9a1a	0.34651107	0.351090918	-0.438999012	-0.868499411
ENSDART00000113332	nav2a	0.038264794	-0.31474256	-0.162187636	0.073256894
ENSDART00000113342	tox3	-0.349601618	-0.245853476	-0.159173384	-0.016959002
ENSDART00000113347		-0.143628386	0.120543612	0.968351441	1.05709332
ENSDART00000113351	ncapd2	0.642337818	0.531864694	0.423422126	0.276399068
ENSDART00000113356	crtc1a	-0.294987163	-0.303249696	-0.252865857	-0.15461469
ENSDART00000113358	wdtc1	-0.236133684	-0.265438127	-0.168580048	-0.081863191
ENSDART00000113362	ip6k1	-0.059673843	-0.326658711	-0.456047384	-0.047258149
ENSDART00000113376	fam13b	-0.247096949	-0.341873515	-0.575011995	-0.179280023
ENSDART00000113384	lyrm4	-0.082514668	-0.167513155	-0.434528553	-0.231621665
ENSDART00000113405	inpp5d	0.955185108	0.470897855	0.050793817	-0.341647614
ENSDART00000113414	kirrel3a	-0.106976766	-0.45848865	-0.133882402	0.030163053
ENSDART00000113418	igsf3	0.097020516	0.19405944	0.607620623	0.491282072
ENSDART00000113441	mylk5	0.7890096	0.412195956	-0.130414718	-0.622741882
ENSDART00000113448	WDR31	-0.320489538	-0.191593939	-0.444041238	-0.334593236
ENSDART00000113449	LEPROT	0.490278812	0.508314674	0.201181129	0.107244197
ENSDART00000113454	chgb	-0.341137392	-0.331982228	-0.294276744	-0.206705528
ENSDART00000113486	mxra5a	0.309757309	0.386526989	0.398748931	0.219293393
ENSDART00000113493	tmem14ca	-0.005514111	-0.126364666	-0.303791848	-0.180226659
ENSDART00000113494	MB21D2 (1 of many)	-0.150231845	0.017615171	0.437179993	0.383202322
ENSDART00000113502	si:dkey-84j12.1	-0.363232597	-0.10430598	0.492332906	0.665079551
ENSDART00000113511	lzts1	-1.206756297	-0.713910989	-0.061680884	0.474255612
ENSDART00000113524	fam20ca	-0.141135887	-0.526654581	-0.212681972	0.069156499
ENSDART00000113532	CABZ01053588.1	0.66169386	1.399901635	1.305166578	0.721799042
ENSDART00000113550	si:dkey-16p21.7	-0.102214974	0.203772916	0.542107134	0.32053481
ENSDART00000113551	trim2b	-0.50417548	-0.207383112	-0.112448816	0.043074002
ENSDART00000113574	anln	1.428520356	0.903846206	0.598652735	0.284032371
ENSDART00000113589	gpx4b	0.633021831	-0.10742897	-0.312510701	-0.292370818
ENSDART00000113643	fbxo25	0.400029831	0.002394742	-0.303777301	0.069383699
ENSDART00000113649	impg1a	-0.10562067	-0.311339049	-0.59002435	-0.135488682
ENSDART00000113655	tmem26a	0.744638282	1.01684072	1.017300963	0.48692919
ENSDART00000113661	kif16bb	-0.079672799	-0.046364361	-0.755779075	-0.266178188
ENSDART00000113673	scpp5	1.321767426	0.530715467	0.931995129	0.748355856
ENSDART00000113699	hgfb	1.149006949	1.556415854	1.569424226	0.45979101
ENSDART00000113732	orc6	1.104098282	1.496929933	0.974585154	0.388395497

ENSDART00000113734	chrdl2	-0.47545279	-0.359333121	-0.57438139	-0.417914769
ENSDART00000113752	si:dkey-6i22.5	1.012202134	1.421252013	0.919204178	0.319242336
ENSDART00000113773	cdc42bpab	-0.428966416	-0.296406465	0.003528598	0.157857062
ENSDART00000113789	zgc:198419	1.388791656	0.463187117	0.161859067	-0.160692863
ENSDART00000113796	cacnb3b	-1.378695476	-1.717513295	-0.09050578	0.468842834
ENSDART00000113799	si:ch211-132b12.7	0.091573373	-0.269264305	-0.847675231	-0.371595689
ENSDART00000113805	nfatc2b	-0.301956989	-0.356397992	-0.30237662	-0.2051156
ENSDART00000113829	cdca7b	1.046232255	1.184963787	0.646928123	0.587090661
ENSDART00000113842	dpep2	-0.005800421	0.049668681	-0.202900206	-0.709132578
ENSDART00000113847	chpfa	-0.17880495	-0.243387936	-0.273328364	-0.205137329
ENSDART00000113853	prokr1a	-0.220461946	-0.109814734	-0.599222392	-0.17974305
ENSDART00000113859	crispld1a	0.006653123	-0.124334074	-0.47278833	-1.109435686
ENSDART00000113864	faxca	-0.531664025	-0.191322961	-0.005943785	0.107092327
ENSDART00000113890	rabep2	-0.293276158	-0.227772629	-0.308410671	-0.161791109
ENSDART00000113912	ntm	-0.178976918	-0.363265579	-0.513893339	-0.211182567
ENSDART00000113924	lrrc7	-0.537461037	-0.585108204	-0.303180727	-0.024642369
ENSDART00000113936	SAMD8	0.425621262	0.457128542	0.374121986	0.140204511
ENSDART00000113954	si:ch211-113e8.11	-0.149005557	-0.166754978	-0.25646894	-0.094366468
ENSDART00000113985	mctp1a	-0.392181593	-0.510456941	-0.185251742	0.106694132
ENSDART00000114000	zgc:101663	0.831482698	0.254442158	0.060051833	0.091105397
ENSDART00000114010	slitrk3a	-0.361859007	-0.532282315	-0.225491041	0.106517685
ENSDART00000114023	hspb9	2.157513115	1.55136025	0.742718373	1.461470885
ENSDART00000114024	fam107b	0.899175294	1.079533729	0.969523488	0.434748246
ENSDART00000114026	lingo2a	-0.52437969	-3.569137229	-2.642152541	-0.423360666
ENSDART00000114069	cnnm4b	-0.449604106	-0.276862048	-0.121356457	-0.173176154
ENSDART00000114078	si:dkey-193c22.2	0.250019316	-0.054913753	-0.32754917	-0.367609802
ENSDART00000114081	sms	-0.273814009	-0.150000435	0.170943482	0.265559933
ENSDART00000114083	mcf2la	-0.277595318	-0.392280599	-0.224516886	0.025969597
ENSDART00000114098	dkk2	-0.775548216	-0.461837556	-0.163285316	-0.107943795
ENSDART00000114099	BX545917.1	-0.259517692	-0.573524763	-0.511746601	-0.397812833
ENSDART00000114117	necab1	-0.468499392	-0.405674747	-0.46743135	-0.222378141
ENSDART00000114118	CU639468.1	-0.073130118	-0.36036163	-0.387238652	-0.163804242
ENSDART00000114134	wrb	-0.123951898	-0.095408141	-0.313002762	-0.1622087
ENSDART00000114162	ftr02	-0.105106717	-0.079876083	-0.28693813	-0.123365826
ENSDART00000114168	itga6a	1.998266436	2.016280588	1.463165074	0.79739173
ENSDART00000114169	bnip1a	-0.010894413	-0.404734866	-0.484887345	-0.670176287
ENSDART00000114172	pde6d	-0.201449776	-0.351770435	-0.511138243	-0.216254622
ENSDART00000114182	fyco1b	0.10137681	-0.156409007	-0.465781329	-0.01243848
ENSDART00000114226	grm6b	-0.535654703	-0.571067618	-0.480335418	-0.370568762
ENSDART00000114263	zgc:194242	-0.119842829	-0.109342365	-0.31411889	-0.410626816
ENSDART00000114267	map6b	-0.567896811	-0.403955591	0.492985119	0.85461741
ENSDART00000114272	nop2	0.471489342	0.531037709	0.420888693	0.177047326
ENSDART00000114282	rpsa	0.494276409	0.531800028	0.113194341	-0.004402994
ENSDART00000114293	areg	-0.232855169	-0.639973524	-1.102041863	-0.924035384
ENSDART00000114301	si:ch211-152p11.4	-0.510547269	-0.515838482	-0.89019375	-0.843865894
ENSDART00000114319	si:ch211-76l23.7	-0.274578898	-0.196596974	-0.473622321	-1.111584541
ENSDART00000114322	rusc1	-0.055969033	-0.296840251	-0.124744669	-0.048299568
ENSDART00000114336	FAM189B	-0.002256766	0.039346929	0.240983806	0.310705552
ENSDART00000114383	mesdc1	0.277986788	0.306020697	0.430972974	0.195133048
ENSDART00000114410	wdr62	0.584622073	0.50588924	0.329377091	0.089655627
ENSDART00000114432	ntng2a	-0.38146173	-0.278136767	-0.282818481	0.289761799

ENSDART00000114442	fndc5b	-0.498984664	-0.658119927	-0.45669978	-0.18817484
ENSDART00000114448	CHST8	-0.44164787	-0.464005415	0.047368929	0.426791372
ENSDART00000114497	si:dkey-204l11.1	0.312589581	0.828199841	0.99726607	0.068811015
ENSDART00000114533	mn1a	-0.109536494	-0.207833595	-0.685216226	-0.20481073
ENSDART00000114611	sipa1l1	-0.211106005	-0.264425665	-0.102896881	-0.110763007
ENSDART00000114659	maml3	-0.010023722	-0.260659863	-0.229491996	0.012935978
ENSDART00000114660	opn8b	-0.615308217	-0.695080703	-0.641485332	-0.112308149
ENSDART00000114663	b4galt2	-0.500896606	-0.53488094	-0.237386402	-0.192514996
ENSDART00000114676	gstm.2	0.102339801	-0.045272058	-0.466318631	-0.673317054
ENSDART00000114677	si:ch73-62l21.1	-0.323827666	-0.531978643	-0.239685061	0.043755156
ENSDART00000114678	nanos1	-0.186501948	-0.191589001	-0.393479142	-1.039695471
ENSDART00000114705	gprc5bb	-0.132340316	-0.338695197	-0.459834622	-0.091865364
ENSDART00000114711	sorcs1	-0.736062495	-0.837966489	-0.594713883	-0.208228471
ENSDART00000114719	frmpd1a	-0.29565145	-0.338039747	-0.498795038	-0.059125706
ENSDART00000114723	rapgefl1	-0.431713581	-0.430345279	-0.085299154	-0.113952199
ENSDART00000114746	lrrc58a	-0.133537399	0.014548786	-0.622798481	-0.259773873
ENSDART00000114748	vip	-0.375915262	-0.449108971	-0.514462327	-0.417228927
ENSDART00000114750	gabrb2	-0.56997023	-0.610789713	-0.475013901	-0.067415538
ENSDART00000114774	ptpn5	-0.403695841	-0.145979825	0.358989634	0.339107007
ENSDART00000114800	INAVA (1 of many)	0.074314999	0.343046405	0.508626667	0.501928464
ENSDART00000114888	picalmb	-0.141762949	-0.21097576	-0.321137175	-0.075858488
ENSDART00000114909	cuedc1a	-0.291201492	-0.290842243	-0.025622448	0.12561667
ENSDART00000114919	si:dkey-85k7.12	0.382806755	-0.226494664	2.101494432	-0.132340955
ENSDART00000114954	rapgef5b	-0.637101425	-0.566396383	-0.284127257	0.00714868
ENSDART00000114959	cdh24a	-0.51153845	-0.93145045	-0.229616461	0.228723625
ENSDART00000114964	zgc:165573	0.691787708	0.943542464	0.634593348	-0.039695615
ENSDART00000114975	slc5a7a	-0.574231808	-0.735930483	-0.690375373	-0.752661657
ENSDART00000115023	PARG	0.013352577	0.067569536	0.312910159	0.18617429
ENSDART00000115027	tmem151bb	-0.856524713	-0.708298022	0.291447963	0.495918048
ENSDART00000115030	dip2a	-0.094281248	0.006396805	0.484131414	0.408709873
ENSDART00000115049	mych	2.666296724	2.42380403	1.76502541	0.819436082
ENSDART00000115058	pbxip1a	-0.476509078	-0.442420659	-0.523364891	-0.310557938
ENSDART00000115088	birc5a	0.588066272	0.489725837	0.133434985	0.421614791
ENSDART00000115089	zgc:171482	-0.437080228	-0.507022457	-0.285662093	0.021798354
ENSDART00000115118	tns1a	-0.409165389	-0.800200161	-0.457588882	-0.074958883
ENSDART00000115128	usp2b	-0.251717928	-0.321635103	-0.314073339	-0.302633188
ENSDART00000115130	apbb1	-0.260426658	-0.249126495	-0.138007572	-0.113696464
ENSDART00000115138	rapgef4	-0.335306767	-0.551146979	-0.593390846	-0.144257686
ENSDART00000115141	cacnb3b	-1.10918155	-1.536403603	-0.203181521	0.418821499
ENSDART00000115157	tuba4l	0.217024219	0.564563401	0.525644709	0.157922416
ENSDART00000115161	reps2	-0.084468471	-0.327140094	-0.166340372	-0.127803804
ENSDART00000115221	adat2	0.066031062	-1.495132242	-2.125701316	-0.144340549
ENSDART00000115224	pvrl1a	-0.001527	-0.239159739	-0.512986688	-0.121351808
ENSDART00000115244	MEX3A	1.222333965	1.709642669	1.900449694	1.354844663
ENSDART00000115255	mcf2l2	0.795464349	0.848343644	0.628078884	0.002922432
ENSDART00000115260	si:ch211-113g11.6	-0.558167113	-1.193520332	-0.037233796	0.533309069
ENSDART00000115278	cx47.1	-0.445735222	-0.554690047	-0.399294096	-0.241214414
ENSDART00000115330	ppp1r14c	-0.176056212	-0.351217013	-0.000962023	-0.249969624
ENSDART00000115343	atp8b4	-0.126263785	-0.363733117	-0.385610912	-0.00148052
ENSDART00000115354	si:dkey-188i13.10	0.511265672	0.230301163	1.128651443	-0.074861923
ENSDART00000115356	rabif	-0.104235333	-0.157538528	-0.394672329	-0.327606577

ENSDART00000115365	rassf10a	-0.525931221	-0.509933384	-0.256813636	-0.202027084
ENSDART00000115370	mettl22	-0.250044414	-0.320129908	-0.572576042	-0.437533988
ENSDART00000115398	arid5a	0.65326128	0.28174461	0.163458034	-0.140420456
ENSDART00000115403	NAV1 (1 of many)	0.648363377	0.712988904	0.734499876	0.497918559
ENSDART00000115417	si:ch211-197l9.2	0.463829162	0.238718738	0.779021857	0.564789152
ENSDART00000115550	RNase_MRP	-0.088007656	-0.32868786	-0.789978682	-0.744862923
ENSDART00000115759		2.750406791	2.822768519	2.52417923	3.741762635
ENSDART00000115901	SNORA71	0.179219354	0.724792108	0.150624912	0.373922197
ENSDART00000115914	SNORA71	0.179219354	0.724792108	0.150624912	0.373922197
ENSDART00000116021		-0.209375753	0.199058885	-0.850598268	-0.180561216
ENSDART00000116415		-0.367308157	-0.297245306	-0.576085374	-0.866023911
ENSDART00000117636	dre-mir-21-1	1.553092638	1.563315667	0.906878174	0.77260601
ENSDART00000117680	dre-mir-21-2	3.366495632	3.222859121	2.199417673	1.472943682
ENSDART00000118106	SNORA57	0.429083089	0.942452098	0.631445196	0.725057114
ENSDART00000118384	SNORA16	0.565934898	0.9072012	0.499756229	0.494901316
ENSDART00000118961	5S_rRNA	-0.375589637	-0.40645143	-0.614838385	-0.339165991
ENSDART00000119160	SCARNA6	0.120700111	-0.063996796	-0.81140797	-1.067895541
ENSDART00000119311	SNORA53	-0.615548348	-0.821029313	-0.724467957	-1.224866543
ENSDART00000121226	FO704882.1	-0.227266262	-0.409349874	-0.661498137	-0.983304677
ENSDART00000121457	lbh	-0.556077202	-0.419617758	-0.340252817	-0.354555634
ENSDART00000121460	prdm8b	-0.383342673	-0.361617984	-0.117709411	0.067402978
ENSDART00000121476	asns	0.135112284	0.218356853	0.383559102	0.26633883
ENSDART00000121489	mybl2b	0.480424926	0.360866643	0.136903159	0.039795348
ENSDART00000121496	gpr153	-0.22286471	-0.331603234	-0.24476212	-0.082544732
ENSDART00000121503	cplx3b	-0.427974445	-0.393867199	-0.470572672	-0.340193244
ENSDART00000121531	mat2aa	0.194654747	0.17321442	0.334936873	0.070313151
ENSDART00000121545	brms1	-0.198338884	-0.120854433	-0.325603911	-0.103470514
ENSDART00000121598	phf10	-0.237213765	-0.309795055	-0.326418269	-0.213703564
ENSDART00000121647	PRMT8 (1 of many)	0.693543627	0.209410281	0.412670468	0.652513913
ENSDART00000121675	angptl1a	-0.785726694	-0.739578659	-0.711456	-1.277545302
ENSDART00000121684	nat8l	-0.712971152	-0.758652511	-0.423007329	-0.36098028
ENSDART00000121708	pcsk1nl	-0.536353064	-0.421583117	-0.086670577	-0.06684806
ENSDART00000121714	gnptab	0.326534887	0.246690992	0.1944971	0.101206309
ENSDART00000121716	FAM107A	-0.353665396	-0.22740498	-0.416694229	-0.314761483
ENSDART00000121722	si:dkey-274m17.3	-0.27733184	-0.305292621	-0.626644892	-0.318061843
ENSDART00000121731		-0.190786213	-0.244913354	-0.339998812	-0.082783766
ENSDART00000121756	sybu	-0.180478431	-0.141687617	-0.791670055	-0.226701204
ENSDART00000121817	fbln7	0.143978228	0.156275351	-0.420940219	-1.064416584
ENSDART00000121822		0.766367421	1.508595661	1.280597751	1.015709274
ENSDART00000121823	syngr3b	-0.148548395	-0.252198032	-0.105567779	0.074385745
ENSDART00000121826	bean1	-0.425046714	-0.600778843	-0.413029577	-0.180932047
ENSDART00000121837	efs	0.29165502	0.378275961	0.750399498	0.505660243
ENSDART00000121861	prph	1.238160677	2.697856064	2.616616233	2.213149799
ENSDART00000121864	slc27a6	-0.486568844	-0.183000547	-0.356047466	-0.257017832
ENSDART00000121866	desi1b	0.626366318	0.579237123	0.318409588	-0.099655493
ENSDART00000121867	eif3c	0.151751793	0.171944002	0.307946358	0.118389215
ENSDART00000121872	mast3b	-0.277342703	-0.556828614	-0.261994844	-0.168334337
ENSDART00000121874	nfasca	-0.536181619	-0.366142839	-0.19557692	-0.058427118
ENSDART00000121886	hdr	3.187835864	3.220765325	3.322516101	2.611876044
ENSDART00000121913	kctd12b	-0.424186634	-0.205906213	-0.149727383	-0.132895958
ENSDART00000121952	h2afy2	-0.217094069	-0.401231455	-0.429907398	-0.128476741

ENSDART00000121981	smarce1	0.060968412	0.140256121	0.341840216	0.044607056
ENSDART00000121984	ssbp3b	-0.092095029	0.005321722	0.831022729	0.629434393
ENSDART00000121989	cald1b	-0.204161416	-0.080240225	-0.306263705	-1.232197212
ENSDART00000121998	grwd1	0.591308312	0.087252775	-0.045976929	-0.167650618
ENSDART00000122015	fam169ab	-0.221722765	-0.165161236	-0.4562862	-0.136486601
ENSDART00000122037	rbfox1	-0.419101241	-0.392215043	0.102043249	0.360818016
ENSDART00000122041	nrcama	-0.49531831	-0.707479994	-0.094699246	0.009754423
ENSDART00000122059	scoca	-0.009226259	-0.04540809	-0.349436614	-0.115738429
ENSDART00000122074	slc1a7b	-0.35845108	-0.359766371	-0.277402725	-0.101652869
ENSDART00000122081	sybu	-0.707212841	-0.524661917	-0.600466578	-0.221891711
ENSDART00000122099	dynll2a	-0.463212519	-0.169502826	-0.013591394	0.001709702
ENSDART00000122101	tbx2b	-0.056468629	0.017248499	0.19422425	0.382938949
ENSDART00000122102	wee1	1.082199278	1.291191801	0.972164457	0.241130696
ENSDART00000122115	si:ch211-153j24.3	-1.030489347	-0.968888956	-1.068398619	-1.338359032
ENSDART00000122133	mkrn2os.1	0.117189294	0.106487037	0.442579902	0.198380082
ENSDART00000122159	si:dkey-118k5.3	0.43932377	0.244452571	0.265050848	-0.163246789
ENSDART00000122170	smc5	0.002327719	-0.01045929	0.55091638	0.063311494
ENSDART00000122238	tyr	1.0676401	0.617097515	0.165901971	-0.685666733
ENSDART00000122288	ctsla	0.76727009	0.372627978	0.369896487	-0.200637952
ENSDART00000122305	Metazoa_SRP	1.452964427	1.874874084	1.874716847	1.224958179
ENSDART00000122307	gcsha	-0.027923504	-0.123933497	-0.256124703	-0.163875624
ENSDART00000122321	slc33a1	0.868257035	0.839034235	0.725290511	0.201428784
ENSDART00000122348	gad1b	-0.426563629	-0.260162283	-0.435212228	-0.267475156
ENSDART00000122359	si:dkey-164f24.2	-0.316246733	-0.171154186	-0.508268616	-0.361228089
ENSDART00000122389	elmod1	-0.62893484	-0.673357812	-0.159560027	0.32261476
ENSDART00000122407	cdk1	1.677931921	1.534655447	0.824563588	0.384011667
ENSDART00000122429	klf15	-0.341190152	-0.616050077	-0.578895446	-0.337948098
ENSDART00000122433	trim33l	0.38510745	0.766516023	0.671400212	0.185347257
ENSDART00000122439	si:dkey-238d18.4	0.670270269	0.479235347	0.13889457	-0.356907703
ENSDART00000122454	citb	-0.619522503	-1.363556906	-0.83905374	-0.294671991
ENSDART00000122519	st8sia6	-0.258839848	-0.191246071	-0.329756561	-0.383108642
ENSDART00000122564	trmt10a	0.324659997	0.317173694	0.413928399	0.18195893
ENSDART00000122566	mxa	-0.032262098	-0.382486532	3.249286652	-1.622639363
ENSDART00000122574	ppp1r3aa	-0.153287429	-0.684598529	0.022675976	0.569399664
ENSDART00000122601	pgam1b	-0.261329131	-0.223010936	-0.252995896	-0.069406148
ENSDART00000122605	atxn7l1	-0.290852975	-0.415130691	-0.145082777	-0.005809908
ENSDART00000122617	si:ch211-24o10.6	-0.23677423	-0.709531228	3.09570127	-0.222670409
ENSDART00000122628	junba	0.280813702	0.583374691	0.41181471	0.073998168
ENSDART00000122632	si:ch211-173d10.4	-0.251574462	-0.294229692	-0.256969485	-0.062618481
ENSDART00000122654	rnd1a	-0.834540827	-0.662552714	-0.915729348	-0.640521788
ENSDART00000122665	mta3	-0.481842523	-0.542700403	0.031188286	0.042011127
ENSDART00000122681	CR848841.1	0.726082565	1.593466942	1.807590182	1.450515621
ENSDART00000122682	cabp1b	-0.294760944	-0.547217913	-0.530251845	-0.289128709
ENSDART00000122692	CABZ01112317.1	0.655801165	0.399271741	0.354973485	-0.117162708
ENSDART00000122696	hnrnpub	0.216889898	0.1794005	0.293328342	0.140752076
ENSDART00000122700	tenm3	0.157516404	0.267363723	0.981020214	1.113087141
ENSDART00000122716	PDE1C	-0.224024171	-0.483764938	-0.500061121	-0.40054702
ENSDART00000122742	rorcb	-0.773981248	0.298893511	0.803866913	0.643975269
ENSDART00000122747	tmem30aa	-0.229416393	-0.299526834	-0.201671762	-0.059032826
ENSDART00000122768	CU984579.1	0.668419525	0.524624014	0.112892825	-0.531459653
ENSDART00000122796	rgl3a	-0.364392925	-0.529093083	-0.585369163	-0.370045273

ENSDART00000122803	usp9	0.139967851	0.094990222	0.369664537	0.232490119
ENSDART00000122829	pik3r1	-0.369164024	-0.336790822	-0.454949598	-0.226248156
ENSDART00000122863	ptgs1	-0.31160045	-0.175184364	-0.407394512	-0.554967944
ENSDART00000122889	myhz1.3	0.065159767	3.686062072	4.139837605	2.765333679
ENSDART00000122891	tmem97	0.209697033	0.237651064	0.43725776	0.414256644
ENSDART00000122898	phf21b	0.630758244	-0.049111481	0.173874589	0.290793166
ENSDART00000122905	rac3b	-0.501948401	-0.363291367	-0.120049924	0.166932504
ENSDART00000122924	CABZ01089151.1	0.66835416	0.574150269	1.297855118	0.40982894
ENSDART00000122929	bbs4	-0.14534671	-0.308002271	-0.496501405	-0.252523436
ENSDART00000122930	scg3	-0.159257317	-0.25593578	-0.408715103	-0.119340458
ENSDART00000122945	noc2l	0.680401135	0.443128484	0.389864365	0.196289408
ENSDART00000122953	gnl3	0.534095037	0.261409226	0.267291075	0.063642911
ENSDART00000122966	hapln1a	-5.328261829	-0.041096146	-1.913311737	-3.425165696
ENSDART00000123002	nme4	3.640965481	3.577167408	3.045307609	2.652336979
ENSDART00000123040	pfkpb	-0.26169445	-0.241526904	-0.415842652	-0.206785479
ENSDART00000123063	fgf18a	0.196370642	0.632761225	0.668396752	0.413800422
ENSDART00000123081	zgc:173552	0.884992881	1.080371934	1.302685665	0.777637365
ENSDART00000123096	marcksa	1.85087225	2.605359702	2.620602183	2.013819083
ENSDART00000123117	ggh	0.485870455	0.424307993	0.067357919	-0.14640404
ENSDART00000123136	FP236812.4	0.346817394	0.374427758	0.928731714	0.426600457
ENSDART00000123152	cpeb4a	-0.291020391	-0.445618867	-0.40169983	-0.306227449
ENSDART00000123203	vbp1	0.24089733	0.440645383	0.41864886	0.239214051
ENSDART00000123254	dpp9	0.199548032	0.283576523	0.332661933	0.196698648
ENSDART00000123263	phf20a	3.204242437	4.684552705	5.028378766	3.758919167
ENSDART00000123278	pikfyve	0.216525391	0.503566219	-0.050873981	0.098099496
ENSDART00000123282	si:dkey-7i4.1	-0.131885839	-0.288104676	-0.328002164	-0.299371464
ENSDART00000123338	kif3a	0.128582487	0.488909245	0.490973182	0.361344362
ENSDART00000123350	sptlc2a	0.577620333	0.063030792	-0.107114764	-0.718762785
ENSDART00000123359	nlgn1	-0.188766802	-0.577714477	-0.350080443	0.032932651
ENSDART00000123360	susd5	-0.191753051	-0.73208098	-0.853142314	-0.526763596
ENSDART00000123364	vegfba	0.24629132	0.079711009	-0.059768872	-0.046839233
ENSDART00000123380	KCNIP4	-0.549899676	-0.507571644	-0.455586059	-0.106289449
ENSDART00000123381	si:ch211-117k10.3	-0.057115592	-0.184208007	-0.285329447	-0.136081942
ENSDART00000123392	rab11bb	-0.044482336	0.125801068	0.511028514	0.477170112
ENSDART00000123407	pdzd7b	-0.257572727	-0.396050586	-0.283495799	-0.108489618
ENSDART00000123409	si:ch1073-296d18.1	0.174260872	0.155958113	0.452524339	-0.02334577
ENSDART00000123450	KCNB2 (1 of many)	-0.271043423	-0.632402343	-0.828557597	-0.168958818
ENSDART00000123473	Metazoa_SRP	0.899034068	1.683872421	0.662894374	-0.48789514
ENSDART00000123479		-0.276038029	-0.380729607	-0.780200096	-0.278407445
ENSDART00000123505	rab11fip4a	-0.154164113	-0.404988879	-0.479622862	-0.179766383
ENSDART00000123506	si:dkeyp-115e12.6	0.611372618	1.198993764	1.526686816	0.680010003
ENSDART00000123518	tuba1b	0.343846477	0.74266829	1.086564506	0.883480954
ENSDART00000123519	ube2e1	0.178513765	0.355426845	0.339753979	0.0793807
ENSDART00000123534	cacna1g	-0.255311686	-0.53660486	-0.469326401	-0.279013734
ENSDART00000123544	tex2	0.532623113	0.735572961	0.212010938	-0.135781649
ENSDART00000123559	znf521	1.710422257	2.255410725	1.850379402	0.814044199
ENSDART00000123568	pmela	0.634272858	0.294790377	-0.243906899	-1.030971827
ENSDART00000123590	si:ch211-91p5.3	0.174508046	-0.021173312	0.906803099	-0.35666968
ENSDART00000123607	cyfip2	0.234079405	0.294840867	0.917405124	0.783474958
ENSDART00000123634	CABZ01076616.1	-0.026473104	-0.096132504	-0.342132107	-0.122009843
ENSDART00000123648	pcdh1a3	-0.182322903	0.28663259	1.138716215	1.048655391

ENSDART00000123713	gria1b	-0.296227004	-0.446226017	-0.161357303	0.142828716
ENSDART00000123797	pcp4b	-0.598256523	-0.489822031	-0.568163011	-0.081816834
ENSDART00000123820	tmem135	-0.104475369	-0.143585965	-0.370543783	-0.122253976
ENSDART00000123834	exoc7	0.170713952	0.17934497	0.358341658	0.328688768
ENSDART00000123839	arrb1	-0.44263739	-0.489445552	-0.281663489	0.083496974
ENSDART00000123844	si:dkey-77f5.3	-4.277427291	-0.495308507	-0.670171681	-0.698447042
ENSDART00000123851	fut8b	-0.379186004	-0.673089848	-0.195485297	0.141395943
ENSDART00000123868	dthd1	-0.700427011	-0.013611963	-0.073941373	-0.012612052
ENSDART00000123878	ppiaa	0.40248836	0.711363911	0.687992513	0.279031014
ENSDART00000123887	MAPK8IP1 (1 of many)	-0.420573616	-0.373470966	-0.247368538	-0.089797668
ENSDART00000123917	tmem47	-0.036122446	0.257386953	0.324417161	0.235522978
ENSDART00000123950	PRIMA1	-0.578840887	-0.634067967	-0.550475282	-0.159964437
ENSDART00000123966	chrng	1.138454485	1.750205793	1.604781052	0.687837172
ENSDART00000123970	mntb	-0.115861458	-0.101461538	-0.360694649	-0.01567692
ENSDART00000123972	smarca5	0.189313952	0.223635359	0.34449924	0.204975788
ENSDART00000123999	SLC27A2 (1 of many)	-1.114337124	-0.48467376	-0.495436914	-0.504796953
ENSDART00000124040	insm1a	0.50962443	1.551047216	1.738068624	1.104992333
ENSDART00000124075	mtss1lb	-0.362597648	-0.323844879	0.066492818	0.131710049
ENSDART00000124085	usp18	0.058285571	0.001224867	0.967078356	-0.263223461
ENSDART00000124112	pou4f2	-1.368026557	-1.308261738	0.344262724	0.908586045
ENSDART00000124140	zgc:101566	-0.242404826	-0.290163127	-0.636219456	-0.416307006
ENSDART00000124176	gfpt1	0.432149516	0.480766327	0.474022237	0.242457652
ENSDART00000124179	hist1h4l	0.531035238	0.617056656	0.928084772	0.625350282
ENSDART00000124217	map1sb	-0.319538816	-0.066329928	0.070606275	0.217460247
ENSDART00000124220	nptxrb	-0.324541852	-0.000122151	0.687818829	0.894343302
ENSDART00000124242	lamp2	0.063109036	0.101381463	-0.092849158	-0.275072982
ENSDART00000124244	rtn3	-0.261781297	-0.269593684	-0.376187628	-0.121031259
ENSDART00000124290	MFN1	-0.108610875	-0.185612855	-0.471855683	-0.083062873
ENSDART00000124298	klhl29	-0.069964723	-0.409007114	-0.117673983	0.023652831
ENSDART00000124300	rhoab	0.400265647	0.097339191	-0.031332977	-0.120716407
ENSDART00000124306		0.869134686	1.333529821	1.120901364	0.868931063
ENSDART00000124327	havcr2	0.988496388	0.35234238	0.213092908	0.061810802
ENSDART00000124329	cbx8a	-0.13552578	-0.23364206	-0.31740894	-0.19855692
ENSDART00000124331	si:ch1073-174d20.1	-0.948877018	-0.570444361	0.133772468	0.53293925
ENSDART00000124333	adcy5	-0.349730091	-0.163331233	-0.158561989	-0.605303683
ENSDART00000124335	smim4	-0.115055175	-0.371982424	-0.338416379	-0.248500803
ENSDART00000124346	fn1a	1.458758212	2.264590948	2.662817487	2.142590168
ENSDART00000124367	si:cabz01074946.1	1.457343255	1.182560392	0.713827232	0.020183118
ENSDART00000124439	cpn1	0.590321112	0.454790314	-0.048032667	-0.505920429
ENSDART00000124440	CABZ01029822.1	0.125194338	0.049659662	0.670452016	0.712162088
ENSDART00000124455	fbxl15	-0.435034118	-0.27455581	-0.042060966	0.121857574
ENSDART00000124480	adgrl1a	-0.174945207	-0.479872966	-0.185768365	0.06038548
ENSDART00000124485	pcdh2ab8	0.093884444	0.182265582	0.466276394	0.290720698
ENSDART00000124499	hprt1	0.192517236	0.178234353	0.277835111	0.149971609
ENSDART00000124534	mbnl2	-0.407254222	0.078580914	-0.044925118	0.120266311
ENSDART00000124562	zgc:152977	-0.424781995	-0.464146326	-0.707134675	-0.367623104
ENSDART00000124563		3.317882457	3.087315331	3.467692067	2.846513731
ENSDART00000124582	napbb	-0.403034353	-0.312472409	-0.341096083	-0.127811116
ENSDART00000124653	dhrs4	0.898977368	0.567421347	0.389421075	0.098869507
ENSDART00000124660	ascc3	0.285037378	0.269547515	0.334772964	0.241795126
ENSDART00000124662	rtn4a	-0.503868889	-0.627866078	-0.609450822	-0.190620657

ENSDART00000124663	npc2	1.578129482	0.684715762	0.389649636	0.031719264
ENSDART00000124676	sv2ba	-0.120324523	-0.401787287	-0.561843381	-0.191682717
ENSDART00000124708	gabra6b	-0.347504052	-0.445856563	-0.426704654	-0.236544239
ENSDART00000124710	dlg5a	-0.356924394	-0.265850728	-0.130233283	0.096340819
ENSDART00000124716	si:dkeyp-121d4.3	0.246129368	0.104493361	0.091668358	0.120636876
ENSDART00000124748	b2ml	-0.109461573	-0.036064969	-0.083861942	-0.342769695
ENSDART00000124751	kcnip3b	-1.29606503	-1.383048631	-0.490749215	-0.039127508
ENSDART00000124762	hsp70.1	-0.061931986	-0.652822267	-0.746588848	-0.837604985
ENSDART00000124773	ppid	0.236087087	0.322094513	0.445941037	0.285911702
ENSDART00000124800	fam212aa	-0.670414346	-0.372526126	-0.229531445	-0.379904435
ENSDART00000124809	acsbg2	0.494562304	1.915512609	1.979085784	0.982887956
ENSDART00000124827	lgi2a	-0.389885576	-0.462014195	-0.106597692	0.145509175
ENSDART00000124833	pdcd11	0.337926893	0.21951318	0.267537583	0.067942864
ENSDART00000124843	mtss1la	-0.820086229	0.028014444	0.433717014	0.003181165
ENSDART00000124868	lpl	0.342987533	-0.032282276	-0.168396519	-1.276175937
ENSDART00000124876	VSTM2B	-0.453041699	-0.513162057	-0.416504873	-0.137923272
ENSDART00000124925	si:ch211-235e9.8	-0.615429137	-1.514463249	-0.238786067	0.039474733
ENSDART00000124945	ubap1lb	0.21207026	-0.004652141	-0.773302877	-0.344881822
ENSDART00000124963	pkig	0.251389387	0.584439023	0.334726577	0.10933368
ENSDART00000124968	rpn2	0.26137974	0.104649464	0.042914045	-0.061671941
ENSDART00000124991	lmcd1	2.103120162	2.716646733	2.223480288	1.685964001
ENSDART00000124998	rtn2a	0.520408534	1.345034307	1.147677305	0.715797789
ENSDART00000125019	dhcr24	-0.273134055	-0.003097208	0.45273437	0.079225052
ENSDART00000125039	six6b	-0.391804698	-0.26201342	-0.503562642	-0.356304334
ENSDART00000125045	dscama	-0.36310557	-0.486996676	-0.080151729	0.074065642
ENSDART00000125058	nipsnap3a	0.560274837	0.34716726	0.187463486	0.300128027
ENSDART00000125074	kcnab2b	-0.745923366	-1.091983107	-0.420367552	0.000775062
ENSDART00000125097	si:dkey-126g1.7	0.825737488	0.388125468	-0.092297047	-0.324389582
ENSDART00000125116	tnfaip8l3	-0.29259694	-0.291950956	-0.382018775	-0.337072666
ENSDART00000125174	nr1i2	-0.056601149	-0.085618729	-0.264588441	-0.326132843
ENSDART00000125178	eif5a	0.322397945	0.394023524	0.161340886	-0.011483865
ENSDART00000125203	hopx	0.434700645	0.383648473	0.145485294	-0.121364202
ENSDART00000125281	ngfra	-0.140591626	0.234351775	0.618758909	0.552257695
ENSDART00000125284	nlgn2a	-0.210046545	-0.366872132	0.090424981	0.163948503
ENSDART00000125299	plk2a	-0.143584568	-0.170521478	-0.297471862	-0.183865098
ENSDART00000125302	fbn2b	0.974537401	0.789475875	1.035854452	0.178462064
ENSDART00000125344	skilb	-0.241964121	-0.627384358	-0.493621211	-0.368838752
ENSDART00000125348	id2b	-0.501394328	0.114793842	0.840457629	0.257023029
ENSDART00000125349	bada	0.528966306	1.266997252	1.043776037	0.694034948
ENSDART00000125371	mknk1	0.126961076	0.659607681	0.524109906	-0.03336174
ENSDART00000125381	gig2o	0.334408908	0.020553966	0.877629524	-0.325957124
ENSDART00000125397	kri1	-0.318299605	-0.278223015	-0.439932558	-0.332258967
ENSDART00000125430	pprc1	1.538620876	1.109491459	0.874206496	0.220876028
ENSDART00000125432	esrrd	-0.825323711	-0.251243419	-0.11920169	-0.154136212
ENSDART00000125440	hist1h4l	0.76925705	0.664322574	0.48261672	0.298833148
ENSDART00000125450	gpc1a	-0.40101998	-0.317779496	0.568583174	0.472068591
ENSDART00000125466	alpi.2	-0.05537717	-0.356245119	-0.658920603	-0.289506352
ENSDART00000125468	apodb	0.705398759	0.818661135	1.046059167	0.463604749
ENSDART00000125472	dnmt3bb.3	2.081331813	1.555687378	1.386584326	1.824843519
ENSDART00000125531	plppr5a	-0.485992962	-0.468686144	-0.487868254	-0.311270154
ENSDART00000125536	appb	-0.473539223	-0.363304525	0.075744005	0.226987466

ENSDART00000125561	atp6ap2	-0.203601896	-0.343976801	-0.371684174	-0.265876436
ENSDART00000125563	homer3b	-0.366344126	-0.72350131	-0.2232749	0.112901448
ENSDART00000125590	adam22	-0.13203003	0.438612977	0.776065364	0.829224171
ENSDART00000125594	zgc:194312	0.282121707	0.314684279	0.206211766	0.077189618
ENSDART00000125617	smoc1	-0.728822769	-0.318137722	-0.435938883	-0.283225846
ENSDART00000125619	thop1	-0.701072314	0.174567303	-0.277028493	0.035731797
ENSDART00000125638	tbx5b	-0.961612456	-0.494056529	-0.317150736	-0.11065371
ENSDART00000125674	pcdh2ab9	0.154453003	0.05176964	0.233264506	0.354320756
ENSDART00000125691	sntb1	-0.296403963	-0.433378655	-0.538134366	-0.484556917
ENSDART00000125733	chst1	-1.779723555	-0.640531075	-0.420086911	-0.327548455
ENSDART00000125753	rpl22	0.364344172	0.362576681	0.16140506	-0.121749389
ENSDART00000125759	si:dkey-22o12.2	-0.433356276	-0.386370833	-0.195481295	-0.118191078
ENSDART00000125766	camkvb	-0.518737667	-0.560273381	-0.364168693	-0.104855004
ENSDART00000125767	metap2a	-0.473789427	-0.340839673	-0.148249289	-0.006647252
ENSDART00000125824	tmem198a	-0.201432593	-0.312524143	-0.133551129	-0.103080035
ENSDART00000125835	si:dkey-16p6.1	0.273905491	0.038923635	0.125549673	0.126712455
ENSDART00000125864	camkk1b	-0.169636507	-0.512096721	-0.591294824	-0.14296099
ENSDART00000125900	hs3st3b1b	-0.2288214	-0.294028167	-0.23141286	0.023426889
ENSDART00000125923	cxcl14	-0.090480689	-0.043715373	-0.409912244	-0.49269465
ENSDART00000125925	tead1a	0.393334189	0.562637179	0.380089957	0.085328359
ENSDART00000125971	gpc1b	0.246742539	0.417837609	0.415208578	0.061504531
ENSDART00000125979	tshz3a	-0.321412605	-0.323547538	-0.394821529	-0.291421679
ENSDART00000126005	efna5a	-0.170916	0.110640262	0.306287547	0.169125563
ENSDART00000126022	rarab	-0.000236754	0.039022735	0.1518943	0.316882235
ENSDART00000126029	dhx58	0.173247162	-0.097510604	2.186112068	0.050096401
ENSDART00000126038	tcp1	0.327351769	0.30721299	0.45795341	0.229539742
ENSDART00000126041	ano10a	0.295415156	0.394412522	0.244334431	0.10037004
ENSDART00000126044	FO904898.3	0.246202383	0.067501502	-0.306505539	-0.628858511
ENSDART00000126076		-0.016912473	-0.350302068	-0.401962349	-0.001679825
ENSDART00000126084	ctdspl3	-0.310396194	-0.278185547	-0.408791973	-0.234117517
ENSDART00000126092	irs4a	-0.078713301	-0.053650022	0.329231719	0.37127028
ENSDART00000126148	zmp:0000000912	0.164571908	0.167838799	1.993878391	0.463546785
ENSDART00000126172	fn1b	1.985622531	1.85759146	1.646183965	1.167188621
ENSDART00000126196	gucy2f	-0.393875584	-0.283587572	-0.315272826	-0.060630266
ENSDART00000126198	lrrfip1a	0.470310797	0.07807124	-0.268319723	-0.389271494
ENSDART00000126205	st6gal1	-0.371204022	-0.447413159	-0.30616801	0.05897999
ENSDART00000126229	rps7	0.396514179	0.373862291	0.136780142	-0.080022773
ENSDART00000126253	fam124b	-0.802465606	0.105938283	0.208441414	0.082147827
ENSDART00000126259	zgc:110434	0.950260296	1.108697637	1.201811754	0.695021733
ENSDART00000126260	anxa6	0.678382414	0.731151807	0.839317026	0.481712468
ENSDART00000126282	nr1d1	-0.1014969	-0.191656676	-0.494430716	-0.13667305
ENSDART00000126299	gdi1	-0.113565456	0.226089497	0.711067572	0.636383115
ENSDART00000126300	shisa8b	-0.373408846	-0.464508355	-0.412634146	-0.258826308
ENSDART00000126301	prkab1b	0.061924737	-0.091377696	-0.203476199	-0.507302968
ENSDART00000126314	pcdh8	-0.25826413	-0.454008614	-0.231067015	-0.062706758
ENSDART00000126397	tspan4a	-0.347274376	-0.217924211	-0.29861217	-0.115670743
ENSDART00000126414	eml1	0.475872706	0.625227433	0.675164018	0.217118522
ENSDART00000126417	bri3	0.500014173	0.414528928	0.11265957	-0.17961569
ENSDART00000126444	mmp13a	0.046684026	3.040517608	1.846123489	-1.625378496
ENSDART00000126460	il34	0.203388167	0.052375422	-0.136564829	-0.587275531
ENSDART00000126470	zgc:85777	0.313383122	0.223125376	-0.098199946	-0.16516057

ENSDART00000126474	si:ch211-265o23.1	1.117262816	1.407297939	1.180818753	0.875131455
ENSDART00000126542	ppp1r14ba	0.329398236	1.23193061	1.655895613	1.416335991
ENSDART00000126559	rap1gap2a	-0.313212246	-0.347504992	-0.446138134	-0.231468434
ENSDART00000126565	si:ch211-150g13.3	-0.477986184	-0.41948879	0.219835784	0.507153945
ENSDART00000126578	si:ch211-200e2.1	-0.112922065	-0.175836986	-0.289153296	-0.021149659
ENSDART00000126588	rac3a	-0.258496234	0.207987177	0.575325222	0.581016326
ENSDART00000126621	pfdn1	0.222765458	0.317529573	0.180735937	0.092770718
ENSDART00000126634	si:dkey-237m9.2	2.393787497	2.476689815	2.575358161	0.828914545
ENSDART00000126640	RHOBTB3	-0.934122492	-0.733675156	-0.213973967	-0.180952113
ENSDART00000126661	rhobtb1	-0.165202139	-0.331565823	-0.522361525	-0.299461153
ENSDART00000126681	tardbpl	0.019310972	0.344433444	0.212149787	0.045304759
ENSDART00000126705	eif3ea	0.116700572	0.376861812	0.239268123	0.089153507
ENSDART00000126739	tspan5a	-0.28156215	-0.24229244	-0.35515584	-0.059588327
ENSDART00000126744	nrg3b	-0.484773132	-0.477903604	-0.212248622	0.011007147
ENSDART00000126766	erbb4b	-0.378985194	-0.566156911	-0.016423806	0.214915468
ENSDART00000126768	cc2d2a	0.207920961	0.256447903	0.267293619	0.323639423
ENSDART00000126780	rnaset2	0.561676509	0.243610345	-0.050562112	-0.093659569
ENSDART00000126826	tmem59	0.0843517	-0.210535259	-0.365503975	-0.184419203
ENSDART00000126842	flj11011l	-0.120546184	-0.161649138	-0.295902143	-0.182318736
ENSDART00000126845	ldb1a	-0.250500011	-0.460439315	-0.246081535	-0.230460175
ENSDART00000126866	ccser2b	0.139738782	0.390945754	0.269322283	0.164198289
ENSDART00000126870	ndufa4l2b	-0.364506533	-0.363911454	-0.576856062	-0.429052776
ENSDART00000126873	myhz2	-2.902978535	4.975016398	5.62978424	4.2721239
ENSDART00000126891	sept2	0.631165801	0.418693285	0.081387841	-0.156566045
ENSDART00000126897	lamtor5	0.078015812	-0.030543248	-0.309612502	-0.278777082
ENSDART00000126916	mob2b	0.26836075	-0.004670217	-0.398977994	-0.072830738
ENSDART00000126928	si:dkey-73n8.3	-0.354837143	0.244926012	-3.843926683	-0.176933271
ENSDART00000126945	adgrb3	-0.310129925	-0.752937386	-0.198981749	-0.020103794
ENSDART00000126963	ucp3	-1.126828953	2.695519216	3.149953006	2.002864913
ENSDART00000126966	esrrga	-0.248566713	-0.265310961	-0.321308408	-0.070523791
ENSDART00000126978	acadl	0.315070362	0.266918423	-0.034640916	-0.111553388
ENSDART00000126979	ubxn1	0.239657791	0.430777506	0.361799544	0.043577807
ENSDART00000126991	ascl1b	0.538037435	0.289776062	1.627554047	-0.154081652
ENSDART00000127009		0.669374866	0.368365497	0.110306036	-0.47074427
ENSDART00000127015	srrm4	-0.1898883	-0.282207317	-0.262672915	-0.106216594
ENSDART00000127047	tusc5a	-1.04192916	-0.556828029	0.377559818	0.604205052
ENSDART00000127050	nrxn3b	-0.323579219	-0.438342846	-0.422724255	0.054279865
ENSDART00000127062	egr3	-1.907539966	-1.048685041	-1.405658043	-1.087307214
ENSDART00000127073	lrrc75bb	-0.380397254	-0.264539972	-0.318155891	-0.090026751
ENSDART00000127093	CR749162.1	-1.06520168	-0.24719149	-0.825954556	-0.565925266
ENSDART00000127099	nr2e3	0.281603482	0.002911001	-0.560613973	-0.033774044
ENSDART00000127131	mtmr10	0.05904867	-0.240178	-0.607174209	-0.120957306
ENSDART00000127136	slco5a1b	-0.145489914	-0.411558687	0.007460079	-0.021875568
ENSDART00000127144	cacna2d2a	-0.206435603	-0.394190367	-0.167540436	0.000520565
ENSDART00000127157	hlfa	-0.089881453	-0.212609643	-0.346930267	-0.147102401
ENSDART00000127162	si:dkey-165a24.9	-0.154532095	-0.18408156	-0.261561109	-0.381673579
ENSDART00000127167	fam217b	-0.253255723	-0.39768374	-0.389011868	-0.208058743
ENSDART00000127173	clu	0.051800584	0.063760613	-0.14536086	-0.386244567
ENSDART00000127214	tmtc4	-0.125359905	0.4024138	0.47318852	0.32908352
ENSDART00000127216	fam161a	-0.082566753	-0.243407902	-0.44855597	-0.155296925
ENSDART00000127219	FAM155A (1 of many)	-0.247003441	-0.575960705	-0.390291652	-0.269667553

ENSDART00000127236	syt13	-0.555582206	-0.558950845	-0.738809627	-0.549910893
ENSDART00000127239	si:ch211-237i5.4	-0.435620687	-0.33667742	0.058425883	-0.022982403
ENSDART00000127274	sema7a	-0.198678021	-0.353988759	-0.478218296	-0.427230313
ENSDART00000127277	pum3	0.522067564	0.375606403	0.43721492	0.160017235
ENSDART00000127286	si:ch73-140j24.4	-0.467040467	-0.569237917	-0.172853738	0.202142114
ENSDART00000127318	frmpd1b	-0.135556639	-0.245654095	-0.299228813	-0.069164239
ENSDART00000127331	cdc37l1	-0.115815783	-0.102908381	-0.388723888	-0.245749713
ENSDART00000127350	enpp1	1.121109181	0.817391101	0.578924138	0.065420517
ENSDART00000127353	nr1d2b	-0.29103008	-0.243688493	-0.346507775	-0.100908835
ENSDART00000127355	CR855274.1	-1.80396628	-1.678561981	-0.998732969	-1.157402562
ENSDART00000127396	ppm1lb	-0.618109529	-0.371965944	0.29932303	0.461142505
ENSDART00000127397	utp15	0.476513751	0.260847638	0.257137466	0.080743511
ENSDART00000127398	CR753886.1	-0.159141993	-0.332294072	-0.331878243	-0.244595408
ENSDART00000127420	MDP1	1.927039626	2.737884833	2.522148059	1.176014094
ENSDART00000127426	ERBB4 (1 of many)	-0.100134914	-0.701677799	-0.360350675	-0.397963746
ENSDART00000127428	peli3	-0.233448958	-0.50115077	-0.383840384	-0.13792159
ENSDART00000127467	pygmb	-0.413201582	-0.432147367	-0.560055907	-0.134010249
ENSDART00000127496	loxl1	0.412088952	0.133411325	-0.193765804	-0.987510306
ENSDART00000127502	cdkl5	-0.227663486	-0.408317592	-0.318577534	0.048062851
ENSDART00000127504	trabd2a	-0.34035671	-0.379312956	-0.491238318	-0.073447564
ENSDART00000127506	si:ch73-380n15.2	-0.654968439	-0.466259045	-0.336236181	-0.320196074
ENSDART00000127508	thnsl2	0.534241071	0.753527133	0.557550473	0.458352807
ENSDART00000127513	znf385d	-0.506377127	-0.828614957	-0.387596815	-0.346943547
ENSDART00000127536	cbx7b	-0.401352835	-0.168978702	-0.391659396	-0.597817617
ENSDART00000127568	ankha	-0.068226585	-0.359480026	-0.686453521	-0.222767571
ENSDART00000127581	trim23	-0.305887304	-0.304885837	-0.114854094	-0.109504112
ENSDART00000127654	tagapb	-0.160683989	-0.122717735	-0.284237949	-0.235548963
ENSDART00000127656	cln6b	-0.363769389	-0.208045101	-0.481848131	-0.19203643
ENSDART00000127673	si:ch73-362m14.2	-0.314971012	-0.45829899	-0.590197547	-0.155316982
ENSDART00000127693	vps4b	0.2667443	0.328628912	0.300618305	0.02818525
ENSDART00000127695	rpl5b	0.469645013	0.524119827	0.418266352	0.096930554
ENSDART00000127699	si:ch73-335l21.4	1.682684641	2.104012275	1.803809975	0.730936452
ENSDART00000127717	FO704622.1	0.358399682	0.167598174	1.49594356	-0.017716595
ENSDART00000127719	kdm6ba	0.187905295	-0.040637976	0.338835017	0.306420124
ENSDART00000127753	adam15	0.405103987	0.059018909	-0.196195339	-0.053874543
ENSDART00000127769	plekhg7	-0.272021337	-0.179975164	-0.292604049	-0.294852391
ENSDART00000127795	tsku	0.321336842	0.111241615	-0.27306537	-0.619712406
ENSDART00000127834	zeb2a	-0.12592194	-0.244794968	-0.205587566	-0.079770723
ENSDART00000127854	ccni	3.365173646	3.708027214	3.915718651	4.117994882
ENSDART00000127859	trim63b	0.3316873	0.607011237	0.873528277	0.725631694
ENSDART00000127936	prdm2a	-0.397627804	-0.350024919	-0.182979057	0.028300134
ENSDART00000127971	TMEM184B (1 of many)	0.084681099	0.512647767	0.678333061	0.362195553
ENSDART00000127974	lrrk1	0.237699588	0.043061851	0.529534709	0.581549893
ENSDART00000127977	CABZ01016547.1	2.8163175	2.999575494	2.826245437	2.227407522
ENSDART00000127981	rgs9b	-0.268928405	-0.181867875	-0.388691411	-0.111378496
ENSDART00000127982	suox	0.48894768	0.565232546	0.460591806	0.13079859
ENSDART00000128011	tnfsf10l3	-0.207145482	-0.351713028	-0.904110327	-0.837055548
ENSDART00000128014	dnah2	-0.407226928	-0.498892771	-0.604204962	-0.453086208
ENSDART00000128024	wdsub1	-2.995723015	0.554879586	0.200121371	0.371936521
ENSDART00000128049	hunk	0.851411376	0.241049411	0.130293149	0.977016396
ENSDART00000128050	UTP14C	0.741190051	0.667056074	0.681031837	0.432356782

ENSDART00000128053	tmem121b	-0.393762063	-0.457536323	-0.455856107	-0.077381852
ENSDART00000128127	slc38a3b	-0.302545777	-0.299963192	-0.361680425	-0.612800784
ENSDART00000128149	fam32a	-0.07314666	-0.18816053	-0.368263865	-0.361807904
ENSDART00000128174	foxp1b	0.060807795	0.283454627	0.694455914	0.748409918
ENSDART00000128187	mbnl2	-0.194930621	-0.139118187	-0.302093023	-0.22924185
ENSDART00000128193	rnf152	0.402113699	0.480129081	0.225745135	0.017035201
ENSDART00000128198	scn12aa	-0.298887564	-0.309751416	-0.23551982	-0.15404656
ENSDART00000128223	CHCHD5	-0.405489857	-0.5400056	-0.416697607	-0.210022955
ENSDART00000128224	FO904873.1	-0.267293261	-0.580808884	-0.319669262	-0.131136583
ENSDART00000128226	slc6a11b	-0.388946235	-0.51506191	-0.246391039	0.364300065
ENSDART00000128246	polr1c	0.52692078	0.422008081	0.272814737	0.11358433
ENSDART00000128247	lsm3	-0.046938555	-0.089126233	-0.195168175	-0.315381754
ENSDART00000128271	si:dkey-114c15.7	-0.608254547	-0.511435956	-0.075108717	0.021034155
ENSDART00000128281	cntn1a	0.147578203	0.564199709	0.636356286	0.425015924
ENSDART00000128284	nudt9	0.256265084	0.958235099	0.875663921	0.386996856
ENSDART00000128302	stk35	-0.274652044	-0.340334252	-0.095489621	0.00314331
ENSDART00000128346	si:ch211-108c6.2	1.285191812	1.464423055	1.491945601	0.337219819
ENSDART00000128350	rad51d	0.854501715	0.522915635	0.508614026	0.238331303
ENSDART00000128457	ephb1	0.374973875	0.620484905	1.032855939	0.805207117
ENSDART00000128488	e2f8	3.852661131	-0.929008264	3.252279997	2.01178299
ENSDART00000128497	nat14	-0.333855923	-0.175587715	-0.007501498	0.001822083
ENSDART00000128535	dmbx1a	-3.533682655	-2.208339716	-0.232250672	-0.456601031
ENSDART00000128550	palb2	0.912196863	1.171643148	0.786370898	0.077481484
ENSDART00000128613	rps15a	0.315521947	0.355667698	0.260468714	-0.049823228
ENSDART00000128624	sgk494a	0.606573335	0.92915728	0.662849036	0.240608511
ENSDART00000128659	pcdh2ab10	0.122813964	0.17598472	0.479887931	0.226887021
ENSDART00000128673	CABZ01078944.1	0.788095742	0.425650182	0.277053304	-0.16966963
ENSDART00000128681	DYRK3	0.346870394	0.024160089	0.051669126	0.414421342
ENSDART00000128690	agap3	-0.218144691	-0.339443752	0.012538674	0.008782978
ENSDART00000128696	CABZ01035108.1	-0.30766878	-0.382099096	-0.769557831	-0.298631085
ENSDART00000128698	rnf34b	-0.122706526	-0.390373676	-0.658840165	-0.257572429
ENSDART00000128705	ndrg3a	-0.684036937	-0.148509308	0.416765356	0.500717941
ENSDART00000128715	kif3cb	0.538226215	1.727920879	1.708926367	1.162753729
ENSDART00000128721	rcvrn3	-0.045382046	-0.143627209	-0.579607896	-0.393091134
ENSDART00000128722	RGS9BP	-0.328845343	-0.481472863	-0.560905879	-0.19840165
ENSDART00000128742	CABZ01118678.1	0.234931395	0.748418816	0.975879993	0.726138214
ENSDART00000128743	tmem214	0.301583498	0.153317199	0.10691428	0.053655055
ENSDART00000128760	pacs2	-0.00152054	0.063293894	0.531537781	0.626023304
ENSDART00000128762	dedd1	-0.117364287	-0.167908679	-0.255046257	-0.080222107
ENSDART00000128763	si:ch211-204c21.1	2.511635154	2.527602305	2.787303506	2.348876075
ENSDART00000128784	vmhcl	0.489881537	1.799102022	2.075276497	1.004457616
ENSDART00000128786	pyya	0.897714064	0.120108152	-0.15783225	-0.181132487
ENSDART00000128794	gpm6ab	-0.753205656	-0.206704843	0.388986514	0.547688547
ENSDART00000128807	gpr22a	-0.50401992	-0.32421881	-0.337086094	-0.059033497
ENSDART00000128839	fez1	-0.463224643	-0.323499809	-0.160691051	0.039189596
ENSDART00000128859	prlra	-0.153277376	-0.232075961	-0.477332275	-0.11182176
ENSDART00000128894	dchs1b	0.298598944	0.508598812	0.623831745	0.178131833
ENSDART00000128917	rps4x	0.379768803	0.358465257	0.299276991	0.035010431
ENSDART00000128931	igf2bp2a	1.122085018	1.168437537	1.475101165	0.642256664
ENSDART00000128959	zgc:113223	-1.993126278	-0.750526857	-1.059101824	-0.610323514
ENSDART00000128965	bcar1	0.218283788	0.254312241	0.324642744	0.284302999

ENSDART00000128969	slit1a	-1.459038277	-0.917332072	-0.293864838	0.107599131
ENSDART00000128971	gm2a	0.278528878	0.08350244	-0.093671452	-0.160146628
ENSDART00000128975	slc25a32a	0.048183005	-0.130483176	-0.493409396	-0.216291506
ENSDART00000128978	CR391998.1	1.262775633	1.767194773	1.40829971	2.265912659
ENSDART00000128981		-2.281844224	0.709538363	0.831386018	0.078800359
ENSDART00000129032	CR847543.1	0.382490935	0.466481864	0.639639363	0.274235342
ENSDART00000129033	si:ch211-232b12.5	0.004752407	0.255447225	0.704679929	0.201922932
ENSDART00000129035	prdx5	-0.081081909	-0.174815089	-0.303582143	-0.169217832
ENSDART00000129055	nrg2b	-0.525587506	-0.415280837	-0.153878493	0.206094594
ENSDART00000129058	rheb	-0.430188972	-0.312082399	-0.301953935	-0.04976624
ENSDART00000129062	ikbke	0.350594041	0.34624224	0.094974339	-0.516125523
ENSDART00000129151	strbp	-0.586891224	-0.661538944	-0.132603114	0.173676112
ENSDART00000129155	vmp1	3.054036254	2.972129495	2.202758828	1.232955319
ENSDART00000129156	adcy7	-0.081832737	-0.232314587	-0.506962474	-0.282923342
ENSDART00000129167	mapre1b	-0.039417049	0.272931214	0.675389418	0.445435787
ENSDART00000129189	slc25a38b	-0.383549453	-0.49018808	-0.499376221	-0.30515595
ENSDART00000129202	DDX17	-0.231356945	-0.275266303	-0.31249155	-0.24318473
ENSDART00000129210	cry1ba	-0.306284135	0.315253029	0.635836839	0.449599168
ENSDART00000129214	COX7A2 (1 of many)	-0.085157083	-0.364093354	-0.62455009	-0.475756885
ENSDART00000129248	clstn1	-0.435055335	-0.404808779	-0.072836605	0.145009995
ENSDART00000129254	zgc:173552	0.679613651	0.919867905	0.987455267	0.613846632
ENSDART00000129268	fads2	-0.454418186	0.562318002	0.686613427	0.310070087
ENSDART00000129273	rps6ka1	-0.302406528	-0.323918919	-0.248745254	-0.049749754
ENSDART00000129308	ACVR1C	-0.083617591	-0.230914728	-0.630759756	-0.255735924
ENSDART00000129362	eef1a1l2	2.651521704	2.343527866	2.861864827	2.05813724
ENSDART00000129365	kcnb2	-0.416222923	-0.447467239	-0.257551256	-0.183749417
ENSDART00000129380	ppp5c	0.718884884	0.082309464	0.464958134	0.512508903
ENSDART00000129407	plin2	0.385951592	0.445429516	0.411868628	-0.015563455
ENSDART00000129423	slc37a1	0.300742512	0.561138843	0.611837666	0.153755959
ENSDART00000129427	vkorc1	-0.08593935	-0.003065091	-0.36339301	-0.313479955
ENSDART00000129458	fasn	-0.115300867	0.324414338	0.375920468	0.257409388
ENSDART00000129459	spata5l1	0.084981287	0.159429104	0.443203325	0.272680023
ENSDART00000129471	gucy1b3	-0.32002288	-0.161099136	-0.090567082	0.034491801
ENSDART00000129474	ogmb	-0.463469684	-0.477885606	-0.227590281	-0.024188229
ENSDART00000129490	gb:bc139872	-0.183429225	-0.314918476	-0.330428185	-0.307368048
ENSDART00000129498	mef2d	-0.188451231	-0.381445449	-0.263610552	-0.197973462
ENSDART00000129501	tmem235	-0.584898252	-0.649073781	-0.768355336	-0.056926947
ENSDART00000129511	robo2	-0.431501059	-0.512946091	-0.075617078	0.250070953
ENSDART00000129521	cpsf6	-0.125055538	-0.280935092	-0.125914075	-0.005682478
ENSDART00000129530	rps27a	0.327713738	0.311901912	0.216145511	-0.053626939
ENSDART00000129559	btbd3b	-0.741839183	-0.720572279	-0.364214548	0.15940566
ENSDART00000129569	gle1	2.578232986	2.574994626	4.293223748	4.445278491
ENSDART00000129593	znf217	0.260126556	0.54491492	0.40143457	0.121556273
ENSDART00000129597	sorbs3	0.016863628	0.072993125	-0.314882072	-0.546339357
ENSDART00000129617	atp6ap1b	0.602272356	0.200096368	-0.075510461	-0.466117163
ENSDART00000129643	aplnrb	-0.038448881	0.28030901	0.821411842	0.34582741
ENSDART00000129668	fam57bb	-0.325481107	-0.111394328	0.301848072	0.107888881
ENSDART00000129674	slc44a2	0.253178883	0.658899779	0.831363022	0.428394942
ENSDART00000129679	abcd4	-0.064830814	-0.217311868	-0.258015712	-0.43583137
ENSDART00000129704	abhd17aa	-0.089448965	-0.057797962	0.474531723	0.44635249
ENSDART00000129710	crtac1a	-0.277382094	0.025987329	-0.337990061	-0.608225673

ENSDART00000129744	grin1a	-0.756444509	-0.702815529	-0.204482345	0.004357228
ENSDART00000129749	psmb7	0.370284184	0.518350984	0.484691258	0.174219127
ENSDART00000129751	si:ch211-105f12.2	-0.375204291	-0.254543259	-0.612224773	-0.289815804
ENSDART00000129772	gck	2.162290839	3.39609934	3.505078852	1.699355805
ENSDART00000129777	lrp11	-0.275801225	-0.119366993	-0.12158768	0.035259399
ENSDART00000129819	si:ch211-151p13.8	-0.747854488	-0.665734365	-0.328239319	-0.007548317
ENSDART00000129834	nat16	-0.654571013	-0.560511117	-0.238004357	-0.212203603
ENSDART00000129866	pcsk2	-0.608807617	-0.601188495	-0.114458443	0.092104737
ENSDART00000129895	palm1b	0.197850318	0.594118975	0.532496272	0.226704229
ENSDART00000129898	ctsf	-3.184979516	-6.647256647	-2.226327525	0.123813808
ENSDART00000129903	mfsd10	0.819604016	0.72451413	0.409438046	0.004032617
ENSDART00000129910	tmem240a	-0.628384792	-0.334786051	0.214415995	0.163191044
ENSDART00000129916	tmem69	-0.236861508	-0.141159535	-0.401818194	-0.186294868
ENSDART00000129919	TIFA	0.580552682	0.759337474	0.369383589	-0.101109572
ENSDART00000129924	hmgb3a	-0.441161001	-0.289637174	0.049371785	0.069315625
ENSDART00000129963	rbp1	1.30707657	0.844804596	-0.023243783	-0.809227723
ENSDART00000129975	scamp4	0.678060482	0.570045099	0.569688168	0.357822663
ENSDART00000130007	GABRA2 (1 of many)	-0.583670012	-0.533882123	-0.170964807	0.218833255
ENSDART00000130093	c10h21orf59	-0.163395921	-0.484168269	-0.87971069	-0.649928123
ENSDART00000130103	kctd16a	-0.864638484	-0.862435538	0.056246994	0.316558654
ENSDART00000130105	psma3	0.177380698	0.356390892	0.223933012	0.001457342
ENSDART00000130125	gnao1a	-0.213616659	-0.159180808	0.167107834	0.384555308
ENSDART00000130131	sult6b1	0.679202914	0.436031956	0.32900324	-0.176606639
ENSDART00000130142	ube2w	0.018587872	-0.041621436	-0.37919808	-0.07627037
ENSDART00000130171	ncl	-0.573496849	-3.495808243	0.185293047	0.359494588
ENSDART00000130215	TMEM179 (1 of many)	-0.219747105	-0.315705451	-0.194562459	-0.061125473
ENSDART00000130264	rpz	-0.422979934	-0.28718615	-0.432905099	-0.242626605
ENSDART00000130273	traf3ip2b	0.459447337	1.01424616	0.933831804	0.205410709
ENSDART00000130277	rpl9	0.669628298	0.786807801	0.580702605	0.163431583
ENSDART00000130297	atg16l2	0.527142084	0.480414213	0.279348799	0.207971893
ENSDART00000130310	epb41l3a	-0.373985055	-0.254132145	0.194766582	0.26124696
ENSDART00000130317	arhgap20	-0.299393415	-0.261693015	-0.196202201	-0.493979065
ENSDART00000130343	parp12a	0.340672377	0.108363497	1.07497332	-0.29758817
ENSDART00000130353	fdps	0.408185548	0.643895342	0.766681528	0.202680939
ENSDART00000130366	si:dkey-21c1.4	-0.116466944	-0.239339578	-0.392905857	-0.311667635
ENSDART00000130375	rap1gap	-0.163733322	-0.471249398	-0.158019615	-0.014828203
ENSDART00000130388	gfra3	-0.144665825	-0.360556971	-0.224595855	-0.144682167
ENSDART00000130393	tlr7	-0.687566787	-0.954352742	-0.823581986	-0.270839916
ENSDART00000130397	akt2	0.652260614	-3.897305009	-1.351826378	-1.626684296
ENSDART00000130413	smc4	1.589552868	1.367681745	1.270952692	0.499256923
ENSDART00000130453	ahcyl2	-0.228112194	-0.250899226	-0.220486764	-0.265846948
ENSDART00000130472	pkhd1l1	0.896390148	0.750907757	1.185805195	0.60737198
ENSDART00000130494	moxd1	0.536918295	0.137560293	-0.344113602	-0.82692019
ENSDART00000130537	spag1a	-0.063362594	-0.300759627	-0.420912866	-0.152669564
ENSDART00000130546	maptb	0.390340472	1.128769701	1.393133544	1.028418073
ENSDART00000130554	isg15	0.469884875	0.09681696	3.101380991	0.025074196
ENSDART00000130569	st8sia5	-0.025137728	0.545403948	0.761405173	0.616910757
ENSDART00000130573	atp6ap1la	-0.419066331	-0.227150493	-0.139216735	0.064701891
ENSDART00000130594	afap1	0.283339001	0.479700622	0.53264953	0.107820725
ENSDART00000130601	CABZ01020840.1	1.211268037	0.754343565	0.351699193	-0.297623866
ENSDART00000130604	ssuh2.4	0.995807077	0.517001744	0.13232339	-0.007643053

ENSDART00000130623	dus4l	0.445376751	0.468587727	0.304661728	0.017366201
ENSDART00000130626	rars	0.486608727	0.578942242	0.46060205	0.253410521
ENSDART00000130632	hif1al2	0.724834586	1.00924352	1.339085575	0.665259415
ENSDART00000130648	scarb2c	0.530119641	0.364624401	0.255215167	-0.022903408
ENSDART00000130655	csnk1a1	-0.038868211	-0.151998306	-0.302247752	-0.150662513
ENSDART00000130675	pi4k2a	0.32642219	0.46314623	0.446740681	0.229275132
ENSDART00000130692	cry1aa	-0.303120324	-0.112884884	-0.294756028	-0.171760667
ENSDART00000130697	runx1	0.52419199	0.991442797	1.34044624	0.449640925
ENSDART00000130712	pcdh2aa15	-0.110623458	-0.061292827	0.28564543	0.306014091
ENSDART00000130722	cacnb2b	-0.144539412	-0.25934202	-0.052732887	0.085080318
ENSDART00000130741	fam20cb	-0.286292768	-0.244788679	-0.108258226	-0.149995213
ENSDART00000130780	clstn1	-0.461252674	-0.35014	-0.010361021	0.286350898
ENSDART00000130781	eif4a2	-0.090677214	-0.087646154	-0.280941023	-0.172536563
ENSDART00000130815	e2f3	0.222958128	0.350714695	0.469146338	0.300980509
ENSDART00000130818	ptprz1b	-0.275892935	-0.352115142	-0.541325565	-0.202924453
ENSDART00000130820	hbegfb	1.26577708	2.42087461	1.934889729	0.952474054
ENSDART00000130828	cnrip1a	-0.043463049	0.089234147	0.284898375	0.266071808
ENSDART00000130880	si:ch73-269m14.2	-0.24405591	-0.090252599	-0.043271301	-0.190219309
ENSDART00000130881	gabrr2a	-0.366410023	-0.492281459	-0.409834	-0.272101123
ENSDART00000130891	camk4	-0.554565795	-0.502410128	-0.607057653	-0.157488548
ENSDART00000130911	cmklr1	1.293061027	1.016453103	0.21085461	0.074156746
ENSDART00000130977	mtcl1	-0.152016911	-0.472594538	-0.24239267	-0.004366794
ENSDART00000131010	zgc:114175	-0.532140281	-0.400405891	0.129904669	0.109706695
ENSDART00000131014	sept7a	0.353546424	0.539836173	0.262138094	0.154511745
ENSDART00000131019	acadvl	0.465985818	0.491501242	0.281421395	0.167335906
ENSDART00000131027	TMEM216	-0.171407049	-0.12306379	-0.494331831	-0.331123076
ENSDART00000131030	gorasp1a	-0.03820825	-0.156177406	-0.398312611	-0.044697109
ENSDART00000131041	doc2b	-0.197976996	-0.14606408	-0.273923734	-0.068935148
ENSDART00000131072	rpz2	0.192971155	0.376625546	0.204804322	0.050600303
ENSDART00000131075	dusp23b	-0.349967426	-0.590231643	0.026826234	-0.132825162
ENSDART00000131101	alpl	-0.065559311	-0.084665618	-0.114820296	-0.355758107
ENSDART00000131110	pigx	-0.041181166	-0.247067458	-0.407100449	-0.233879595
ENSDART00000131126	bcl6a	-0.096649799	-0.044696983	0.295011601	0.436349263
ENSDART00000131134	si:ch211-206a7.2	-0.226522125	-0.472315068	-0.371394997	-0.101330709
ENSDART00000131137	arhgef16	0.581874028	0.364359378	0.072001305	-0.274180473
ENSDART00000131143	sept5b	-0.480326507	-0.332562488	0.283118689	0.526750102
ENSDART00000131161	sema3c	-0.186853445	0.134603767	0.50487559	0.558561927
ENSDART00000131177	adcy1b	-0.090441849	-0.360182616	-0.355106619	0.00963355
ENSDART00000131204	asap1b	-0.546891821	-0.455253903	-0.26913051	-0.092257648
ENSDART00000131206	march1	-0.087431315	-0.336892252	-0.48450384	-0.18585378
ENSDART00000131217	ippk	0.31853123	0.426685761	0.198265799	0.012388457
ENSDART00000131241	CD53	1.613639378	1.480348694	1.150180939	0.900907437
ENSDART00000131286	erlin2	-0.000462383	-0.183644452	-0.434826094	-0.105981059
ENSDART00000131288	adap2	0.075508361	0.577403234	0.52972951	0.102379235
ENSDART00000131319	ctps1a	0.387589066	0.225454863	0.161786406	-0.016402726
ENSDART00000131323	si:dkey-193c22.1	-0.375872197	-0.187390024	-0.399004983	-0.194977152
ENSDART00000131339	capns1a	0.087047749	0.358949459	0.212677567	0.044194443
ENSDART00000131349	mob2b	0.337219743	-0.092777728	-0.774689204	-0.397735432
ENSDART00000131354	si:ch211-235f12.2	-0.417898946	0.007918255	-0.299865948	-0.220993476
ENSDART00000131355	cntn3a.1	-0.54935923	-0.424970108	-0.640814852	-0.044155876
ENSDART00000131361	kcnip3b	-0.353450144	-1.035812483	-0.692091767	-0.071599751

ENSDART00000131400	CR792453.3	2.47650448	0.829837833	-0.069994818	-0.405416724
ENSDART00000131431	sulf2a	0.379800676	0.895838573	1.99747824	2.182238849
ENSDART00000131443	dpm2	-0.116130222	-0.034113196	-0.2501536	-0.189683686
ENSDART00000131477	si:dkeyp-72g9.4	-1.377600786	-1.267958825	-0.607564413	-0.337098864
ENSDART00000131478	kcnab1a	-0.495628945	-0.276495434	-0.300290472	-0.132892856
ENSDART00000131506	cdh6	-0.336043184	-0.313603925	-0.315311457	-0.136816058
ENSDART00000131508	col9a1b	-0.262030114	0.179891299	-0.305486246	-1.812812408
ENSDART00000131517	si:ch211-232m10.6	-0.306074293	-0.295884968	-0.441790198	-0.281067097
ENSDART00000131557	mvdb	0.053284375	1.694163577	1.512604373	0.78016058
ENSDART00000131558	zgc:103438	0.587891191	0.925990315	0.240143957	-0.080675365
ENSDART00000131579	ptprdb	-0.516674931	-0.642051878	0.273217974	0.528486925
ENSDART00000131582	cbx3b	-0.219219	-0.174464705	-0.354832647	-0.341719477
ENSDART00000131610	serbp1a	0.184686975	0.28885198	0.237091318	0.011396501
ENSDART00000131616	atp5f1	-0.440716151	-0.423675229	-0.427802237	-0.218465008
ENSDART00000131627	kcnn3	-0.085165392	-0.23467025	-0.387311243	-0.108636046
ENSDART00000131632	ptk6a	-0.239390363	0.61942439	1.358814463	0.292212059
ENSDART00000131649	sulf2b	-0.232056185	-0.450328943	-0.252313155	0.036414893
ENSDART00000131661	rps9	0.491211514	0.443239408	0.342502062	-0.078353052
ENSDART00000131694	selenbp1	0.440307718	0.23934875	0.219921774	-0.162883702
ENSDART00000131714	mbnl2	-0.406601788	-0.032543807	-0.091713267	0.092369986
ENSDART00000131721	fam65b	-0.06007994	0.061772028	0.37892142	0.335909594
ENSDART00000131724	pld3	0.801498613	0.654598157	0.377873753	0.229417497
ENSDART00000131731	mef2ca	0.234535922	-0.250149626	-0.381098175	-0.047609857
ENSDART00000131736	kcnq2a	-0.749729315	-0.34933354	0.575885203	0.723193716
ENSDART00000131768	abi1a	0.352423076	0.342648792	0.429769283	0.194498692
ENSDART00000131802	si:dkey-204f11.64	0.501807484	0.364460777	0.066146426	-0.103454568
ENSDART00000131846	osbpl11	0.131220058	0.159712436	0.340514183	0.184103052
ENSDART00000131848	BX004774.2	0.065629354	-0.435237015	-1.040450944	-0.818745042
ENSDART00000131860	rpl10	0.591996417	0.137912232	0.041524333	-0.247459563
ENSDART00000131861	tmsb2	0.228394728	0.766531981	0.776266217	0.553413207
ENSDART00000131866	trhra	-0.77311942	-0.482590954	-0.518111078	-0.348001173
ENSDART00000131880	ftr34	0.638978186	0.318226461	1.201576119	0.041863892
ENSDART00000131922	ucp3	0.966573752	1.664449524	1.416927563	1.012896527
ENSDART00000131940	si:dkey-30c15.10	1.656397572	1.745533662	1.503033451	0.676482739
ENSDART00000131944	tagln3b	0.184610888	0.818287372	1.023242059	0.555937355
ENSDART00000131954	irf2a	-0.009751778	-0.124292592	-0.35526394	-0.165453436
ENSDART00000131956	si:dkey-33c12.14	2.548697019	3.072973071	2.718694487	2.992696235
ENSDART00000131966	AL929520.1	1.864305398	1.102319083	0.530092783	0.456970098
ENSDART00000131973	lrmp	-0.070199137	-0.469517154	-1.098854311	-0.136597848
ENSDART00000131976	adipor1a	-0.018834429	-0.17416639	-0.271837866	-0.17995065
ENSDART00000131983	myhz1.2	-0.851007348	2.593036141	3.254881434	2.63776379
ENSDART00000131994	mthfd1l	2.796349329	3.017388421	3.058988321	0.924891143
ENSDART00000132027	spock2	-0.61060119	-0.435727551	-0.163124474	0.204372495
ENSDART00000132119	max	0.339991432	0.197083488	0.14057043	0.318686745
ENSDART00000132123	thsd7ba	-0.490674171	-0.551076996	-0.028899921	-0.007032812
ENSDART00000132146	hsd17b7	2.880332402	3.535090877	2.510243514	2.078786507
ENSDART00000132156	cirbpb	0.302166347	0.411233461	0.214244406	0.367929369
ENSDART00000132158	ptpn18	0.848975716	0.700592324	0.349323957	-0.024370763
ENSDART00000132170	dlg1	-0.324614076	0.23335851	0.829812481	1.031575976
ENSDART00000132171	CALHM1 (1 of many)	-0.125733999	-0.221900772	-0.83821175	-0.699866376
ENSDART00000132175	nadl1.1	0.709405923	0.881560036	1.238414697	1.046443747

ENSDART00000132189	mogs	0.278316469	0.095835936	0.119934101	0.001358604
ENSDART00000132206	si:dkey-242g16.2	0.572333551	0.964289734	1.608250495	0.992663233
ENSDART00000132216	paox1	0.706860126	0.311306251	-0.007602467	-0.279227462
ENSDART00000132235	klhl24b	0.278493921	0.515923362	0.500638596	0.185998384
ENSDART00000132278	rnf144ab	-0.480742338	-0.392838261	-0.157627213	-0.112649454
ENSDART00000132294	fut8a	-0.131220853	-0.116575506	0.125377151	0.342318079
ENSDART00000132296	klhl17	-0.721040801	-0.498955013	0.192513289	0.489858233
ENSDART00000132297	adam9	0.312303854	0.492550355	0.565368245	0.224580764
ENSDART00000132342	kiaa1549lb	-0.130776123	-0.485143208	-0.118054479	0.086040071
ENSDART00000132365	b3gnt5a	0.764122726	0.855145882	0.650888001	0.125992099
ENSDART00000132366	emp1	-1.811345528	-0.544421651	-0.327114312	0.270404939
ENSDART00000132367	si:ch211-235f12.2	0.051407266	-0.037706651	-0.368292836	-0.27129379
ENSDART00000132384	si:dkey-222b8.4	-0.294618002	-0.481620656	-0.505454645	-0.268964873
ENSDART00000132386	si:dkey-21e2.15	2.037905679	4.749110822	3.364621679	1.867440085
ENSDART00000132387	pvalb6	-0.612280143	-0.883743713	-0.424014361	-0.028285642
ENSDART00000132390	ankrd33ba	-0.590428215	-0.156095959	0.097976154	0.018978411
ENSDART00000132411	si:ch1073-184j22.2	-0.765110378	-1.0433591	-0.590491476	-0.153408732
ENSDART00000132426	stmn4	0.67103814	1.650930846	1.637640616	1.211182541
ENSDART00000132437	mknk2a	1.021696261	1.216138852	0.746540318	1.080690265
ENSDART00000132471	csmd2	-0.459001483	-0.911607393	-0.371722747	0.062349627
ENSDART00000132491	wdr83os	-0.018771998	-0.11821754	-0.249438444	-0.184919889
ENSDART00000132522	syt13	-0.475976654	-0.458152818	-0.514134367	-0.475986362
ENSDART00000132542	bdh2	0.716106023	0.289356437	-0.133918448	-0.268013639
ENSDART00000132543	klhdc8b	-0.423366405	-0.574887213	-0.139148992	-0.034877401
ENSDART00000132545	slc25a24	-0.397809038	-0.303980258	-0.481236788	-0.168683615
ENSDART00000132582	map4l	-0.700979622	-0.493129551	-0.385069171	-0.268022699
ENSDART00000132583	nrxn2a	-0.296541609	-0.578930697	-0.085512446	0.138469202
ENSDART00000132591	si:ch211-191i18.4	0.099974449	-0.068457558	-0.479286849	-0.192940747
ENSDART00000132605	shc2	0.131499648	0.254257685	0.501707163	0.375914832
ENSDART00000132637	rab34a	0.251888735	0.316357959	0.030371007	-1.217256649
ENSDART00000132658	si:ch211-194p6.12	0.494361641	0.293067303	0.277540839	0.243465173
ENSDART00000132659	INAVA (1 of many)	-0.598820951	-0.566244118	-0.753512572	-0.519207877
ENSDART00000132660	polr3gla	0.823813849	0.325249077	0.007723106	-0.29164484
ENSDART00000132663	si:ch211-163b2.4	0.347936584	0.995221531	0.769857122	0.372703166
ENSDART00000132664	hmbsa	1.719683433	3.421951592	1.469413717	1.652766494
ENSDART00000132667	si:ch211-213a13.2	1.006426879	1.056394216	0.902670878	0.63305431
ENSDART00000132685	kcnn1b	-1.625205585	-1.495755896	-0.13719571	0.28532188
ENSDART00000132691	adgrb3	-0.413275118	-0.450913383	-0.19602447	0.067293319
ENSDART00000132696	nckap1	0.516973126	0.759090684	0.845846182	0.627500278
ENSDART00000132710	pimr127	-0.279310009	-0.946546987	-0.201892971	-0.041573331
ENSDART00000132732	tmem86b	0.107129941	0.17748496	0.221749507	0.409811812
ENSDART00000132740	ppp3cca	-0.073909682	-0.313026697	-0.310470542	-0.074974696
ENSDART00000132742	apoc1	0.900470905	0.457797087	-0.066389631	0.150028097
ENSDART00000132744	ppcdc	0.019198695	-0.177358944	-0.471740583	-0.31681582
ENSDART00000132761	arhgdig	-3.192123378	0.713824666	1.228568907	0.922752193
ENSDART00000132767	pxdc1a	-0.518773739	-0.198850875	-0.436310873	-0.094674576
ENSDART00000132781	si:dkey-78l4.1	3.429067431	4.682405989	3.946041907	2.798505508
ENSDART00000132788	uap1	1.271261725	1.549537095	1.061485612	0.525207108
ENSDART00000132827	slc29a4	-0.39428721	-0.406514798	-0.162001645	-0.399843403
ENSDART00000132829	inadl	-0.343759067	-0.387749769	-0.321781319	-0.246918999
ENSDART00000132830	stoml3b	2.594772138	1.896323147	1.645470476	1.013599137

ENSDART00000132838	ank1a	-1.15020902	-1.58246422	-0.789622662	-0.12782961
ENSDART00000132884	cxxc5a	-0.136575965	0.119118699	0.415415664	0.166435977
ENSDART00000132911	vwc2l	-0.573360674	-0.839884971	-0.564408735	0.020047377
ENSDART00000132915	serpine3	-0.093377161	-0.220423221	-0.800809367	-1.077921671
ENSDART00000132930	pcbp3	-0.89548835	-0.955377646	-0.326630607	0.075971616
ENSDART00000132941	si:ch1073-287p18.1	-0.223138733	-0.188273	-0.453602691	-0.273864822
ENSDART00000132953	gsto2	2.493368999	2.571702083	1.494039414	1.444027796
ENSDART00000132981	si:dkey-126g1.9	0.817837135	2.40599851	2.663801596	3.155063025
ENSDART00000132982	leng9	3.031473639	2.780356944	2.241298703	1.634482288
ENSDART00000132995	ak3	0.087221034	0.050666468	-0.189471235	-0.418943401
ENSDART00000133028	mus81	-0.28108498	-0.562830421	-0.307741383	-0.072240912
ENSDART00000133030	psmd1	3.699340083	3.88833786	2.358920441	3.148062929
ENSDART00000133035	syt5a	-0.188232763	-0.271065673	-0.434073976	-0.186984299
ENSDART00000133036	elavl3	0.009017018	0.132987196	0.394658714	0.30975228
ENSDART00000133039	tac3a	-0.2410584	-0.489343566	-0.63037654	-0.413025711
ENSDART00000133042	lrriq1	1.127502736	0.596946573	0.146972775	0.204949423
ENSDART00000133059	cited4a	-0.002602779	0.055649701	-0.293136781	-0.030683128
ENSDART00000133079	c3a.5	1.938352782	2.924341422	2.291243352	0.500346416
ENSDART00000133092	st6galnac5a	-0.798487612	-0.408716424	-0.156113795	0.220161008
ENSDART00000133099	zgc:123105	-0.457591599	-0.29947281	-0.397070368	-0.177964487
ENSDART00000133109	rps28	0.456862734	0.459372965	0.307002895	0.056264372
ENSDART00000133114	bend5	-0.067900889	-0.281239203	0.001023992	-0.054776456
ENSDART00000133121	bub1	1.470451257	1.232808742	1.262010591	0.843166609
ENSDART00000133131	uba1	0.147030579	0.239356262	0.529136799	0.293288779
ENSDART00000133134	efemp2b	-0.009444426	0.019713651	-0.293460329	-0.603842237
ENSDART00000133143	gpd1b	-0.326643231	-0.124586783	-0.280591098	-0.372586222
ENSDART00000133167	si:dkey-98f17.5	0.605842267	0.54718235	0.319480313	0.041690788
ENSDART00000133172	palm1a	-0.485644015	-0.226508351	0.746051517	0.735100737
ENSDART00000133176	si:dkey-171o17.8	-0.335115287	-0.40576097	-0.392381982	-0.176105305
ENSDART00000133183	parp8	0.666223987	0.351288157	0.707539986	0.438871753
ENSDART00000133186	ntng1a	-0.121155505	-0.544453536	-0.029519061	0.307964269
ENSDART00000133194	tbc1d1	2.782559818	3.186152585	2.750502559	1.318506947
ENSDART00000133220	CR788254.2	-0.013907422	-0.231082485	-0.739605077	-0.487191429
ENSDART00000133223	COQ10A	-0.381890626	-0.12435669	0.152677483	0.108453147
ENSDART00000133242	syne2b	0.14436825	-0.456480635	-0.844135801	-0.206154738
ENSDART00000133267	grip2b	-0.249633664	-0.394997267	-0.432273427	-0.409837225
ENSDART00000133302	pfkfb4a	0.027105161	-0.087436901	-0.335656317	-0.089798414
ENSDART00000133304	npm1a	1.159038348	0.761096985	0.663684243	0.261874194
ENSDART00000133325	nell2b	-0.450880659	-0.507896343	-0.516937003	-0.181021434
ENSDART00000133330	fam214b	-0.307417712	-0.32820559	-0.047756819	0.003374146
ENSDART00000133348	si:ch211-240g9.1	-0.327991939	-0.379613386	-0.287166398	-0.033274341
ENSDART00000133354	irx4b	-1.675681332	-1.90655223	0.291488221	1.156903659
ENSDART00000133375	si:dkey-30c15.17	0.981163755	0.773615181	0.451221931	-0.42308326
ENSDART00000133384	nr2f1a	-0.318698493	-0.194424161	-0.079355488	-0.084390635
ENSDART00000133385	lrp1ba	-0.614935587	-0.929322267	-0.360685611	0.193952764
ENSDART00000133404	rps24	0.584478639	0.636503255	0.16891132	-0.309462265
ENSDART00000133419	CR847953.1	0.226198142	-0.018883626	0.274295718	0.480234099
ENSDART00000133440	prdm2a	-0.400160361	-0.315344703	-0.135326537	0.100769097
ENSDART00000133449	FO818659.1	-0.258028224	-0.214758594	-0.076374423	0.03227851
ENSDART00000133466	CR847503.1	-0.023677254	0.459363169	0.719903439	0.430626169
ENSDART00000133473	CU207343.1	0.284146912	1.104685019	1.557001573	0.994606199

ENSDART00000133485	BX004774.1	0.155994197	-0.204649087	-0.333501142	-0.428721476
ENSDART00000133487	fosb	-0.882572207	-1.273781011	-0.772506712	-0.914864674
ENSDART00000133496	CU467110.1	-0.265746647	-0.189978673	-0.362219245	-0.151144337
ENSDART00000133497	si:dkey-250k15.4	0.495799772	1.080770342	1.149092422	0.612727116
ENSDART00000133504	dab2ipb	-0.309610082	-0.535236809	-0.062134515	0.075905091
ENSDART00000133512	fosl1b	2.958627889	2.652355797	1.322528527	0.313643003
ENSDART00000133517	si:dkey-228b2.5	0.6930874	0.410660128	0.386710557	0.222079502
ENSDART00000133550	fkbpl	0.247078783	0.644847847	0.380481334	0.08075265
ENSDART00000133583	si:ch211-117m20.4	-0.073043862	-0.13648785	-0.437013406	-0.146443055
ENSDART00000133587	wsb2	-0.218297067	-0.234838526	-0.321740633	-0.105339309
ENSDART00000133613	AL954694.1	-0.189304139	-0.336191594	-0.52116431	-0.341767633
ENSDART00000133628	ankib1a	0.478883806	0.425404685	0.598914996	0.306729273
ENSDART00000133644	magi2a	-0.426844627	-0.586214992	-0.344780913	0.065236587
ENSDART00000133659	ncapg	0.759144886	0.927669912	0.449671959	0.278225274
ENSDART00000133661	tasp1	-0.129454637	-0.177592146	-0.299309238	-0.129141605
ENSDART00000133699	drd4b	-0.558425031	0.210961997	-0.355610364	-0.126077055
ENSDART00000133701	slc6a1a	-0.417718543	-0.444992936	-0.335539494	-0.221449117
ENSDART00000133707	st3gal3a	-0.191118701	-0.289597986	-0.435587316	-0.218434667
ENSDART00000133708	arhgef4	0.196005293	0.61534617	0.621912538	0.36146972
ENSDART00000133718	ddx5	0.680973492	0.739800314	0.555100275	0.545762532
ENSDART00000133721	si:ch211-156j22.4	0.531762631	0.492062657	0.618453608	0.322450987
ENSDART00000133735	cacng7b	-0.52911361	-0.351880161	-0.311790544	-0.261905914
ENSDART00000133743	camkk1a	-0.520213698	-0.784879077	-0.322164805	-0.513359014
ENSDART00000133775	pdlim3b	2.202802348	2.787120555	2.295385553	1.784176005
ENSDART00000133786	si:ch211-196l7.4	-0.265117484	-0.278594168	-0.287454952	-0.249634822
ENSDART00000133802	inpp5jb	-0.26851081	-0.522364408	-0.244805361	-0.012462884
ENSDART00000133823	arvcfb	0.059276527	-0.645309269	-0.357027269	-0.368113028
ENSDART00000133851	nyap2b	-0.344969094	0.36816036	0.857787268	0.825724613
ENSDART00000133867	arfip2a	-0.237325336	-0.320204428	-0.424850477	-0.153128941
ENSDART00000133869	kcnk1a	-0.389144165	-0.38978007	-0.667699511	-0.286776021
ENSDART00000133891	FP015789.1	-0.349747936	-0.468679484	-0.676764109	-0.710268862
ENSDART00000133902	clstn1	-0.274744137	-0.313184588	-0.391319406	-0.194558837
ENSDART00000133917	lrrtm1	-0.267345102	-0.23914052	-0.165843211	-0.135383045
ENSDART00000133922	atp2b3a	-0.330787062	-0.407621044	-0.300317219	-0.080866095
ENSDART00000133934	rps9	0.75844303	0.709617891	0.420926612	0.393006212
ENSDART00000133946	si:dkey-147f3.8	1.477973714	1.681506394	1.170700784	-0.068341199
ENSDART00000133954	mhc1uka	4.819339023	5.453978456	5.536236268	3.849327613
ENSDART00000133956	phactr3a	-0.466148377	-0.040906797	0.400008422	0.572153181
ENSDART00000133968	camkmt	0.353110068	0.455754701	0.544139309	0.380701512
ENSDART00000133981	bco2a	-0.093189558	-0.108753759	-0.2707511	-0.08847471
ENSDART00000133985	kcnd1	-1.187342293	-1.392518344	-0.747443213	-0.002522665
ENSDART00000134035	cntnap5b	-1.01200416	-1.154440105	-0.403471674	0.139198923
ENSDART00000134044	vdac3	-0.155270833	0.146773494	0.378912201	0.402649801
ENSDART00000134047	cyp2p10	-0.641341489	-0.436063229	-0.777629241	-0.630838584
ENSDART00000134052	si:dkey-17e16.8	0.112444287	0.152961651	0.336755528	0.189687147
ENSDART00000134053	usp2a	-0.375529791	-0.273036858	-0.320691469	-0.159380591
ENSDART00000134054	si:dkey-13i19.8	0.097967988	-0.010643615	-0.259041425	-0.27695026
ENSDART00000134064	mxd1	-0.125104323	-0.265030232	-0.047451672	0.092856449
ENSDART00000134083	arhgap21b	-0.613534268	-0.758748481	-1.557344733	-0.550383994
ENSDART00000134087	col14a1a	-0.156423731	-0.247987955	-0.214368462	-0.368307818
ENSDART00000134094	zgc:162193	0.951212203	1.108680472	1.240038838	0.640044887

ENSDART00000134128	impg1a	-0.245363296	-0.207318324	-0.487433197	-0.127440305
ENSDART00000134146	mtss1	0.331132861	0.244842152	0.229292017	-0.102446838
ENSDART00000134186	cplx4b	-0.392479495	-0.543085477	-0.51593407	-0.267696813
ENSDART00000134189	npm1a	1.523433306	1.012610127	1.305980875	0.439289484
ENSDART00000134190	sort1b	-0.233086943	-0.116629656	-0.586941063	-0.191255985
ENSDART00000134202	ccka	-0.373763887	-0.443030746	-0.635815918	-0.444990754
ENSDART00000134206	hspb8	0.868767381	0.59923642	0.380053431	0.101288698
ENSDART00000134241	olfm1a	-1.215716601	-1.195013518	0.048027409	0.32030838
ENSDART00000134242	zfand5a	0.011715993	-0.114565058	-0.599260538	-0.310476512
ENSDART00000134257	znf1027	2.316081338	1.234819597	2.431130405	1.907826667
ENSDART00000134261	dnm1b	-0.433650537	-0.451510325	0.138870574	0.465815695
ENSDART00000134269	fkbp1aa	0.162085973	0.545601006	0.44355879	0.374542557
ENSDART00000134307	dclk1a	-1.448196855	-0.41548572	-0.010990365	-0.586401585
ENSDART00000134393	arhgef9a	-0.866173468	-1.122671917	-0.27973916	-0.154746662
ENSDART00000134399	slkb	-0.338857379	-0.632619838	-0.770465733	-0.089077643
ENSDART00000134404	si:ch211-158d24.4	0.479480538	0.856462378	0.502566478	-0.11398241
ENSDART00000134431	pcbp3	-0.497499314	-0.525712825	-0.323624866	-0.140513929
ENSDART00000134455	zgc:100906	-0.319125396	0.101796228	0.299790493	0.225508495
ENSDART00000134470	fhdc1	0.136576688	0.100413162	-0.280286506	-0.662790087
ENSDART00000134472	taok3b	-0.264981976	-0.491201835	-0.021652579	0.079259902
ENSDART00000134475	gdap1	-0.459369959	-0.408869462	-0.283212336	-0.008346648
ENSDART00000134489	htr2cl1	-0.503209957	-0.409297224	-0.43743207	-0.200430987
ENSDART00000134506	si:dkey-196j8.2	-0.894031142	-1.351407426	-0.577940716	-0.262796642
ENSDART00000134514	smarca4a	0.020398578	0.110031025	0.339984244	0.255390677
ENSDART00000134518	cpa6	-0.382405278	-0.418058887	-0.443147645	-0.210306107
ENSDART00000134542	csde1	0.186004618	0.310572638	0.257821474	0.010065768
ENSDART00000134560	slc45a1	-0.495253985	-0.301829281	0.42188175	0.485995526
ENSDART00000134564	srsf5b	-0.27445076	-0.31745813	-0.113899297	-0.080138136
ENSDART00000134571	rpl13a	0.370507733	0.272503899	0.102928018	-0.095604932
ENSDART00000134602	gria2b	-0.15956996	-0.75241939	-0.264702392	0.253919663
ENSDART00000134608	phyhiplb	-0.442372787	-0.312061243	-0.158725579	-0.176785812
ENSDART00000134635	si:dkey-9i23.15	0.362598675	0.484654675	0.418405681	-0.053498182
ENSDART00000134649	CNGA1 (1 of many)	-0.31835112	-0.262022337	-0.573519962	-0.315754252
ENSDART00000134658	slc44a1a	0.707497042	0.591443803	0.629657706	0.651062281
ENSDART00000134661	gstcd	-0.989748846	-0.61234101	-0.452964111	-0.446444607
ENSDART00000134697	pik3ip1	-0.252912627	-0.195771257	-0.573002676	-0.212029641
ENSDART00000134715	elmod1	-1.115600356	-1.115324879	-0.105456948	0.485174818
ENSDART00000134717	tspan18a	0.042661269	0.285439514	0.614193732	0.731823089
ENSDART00000134722	bcl2l13	-0.594192147	-0.544774096	-0.184876076	-0.124896832
ENSDART00000134727	TNNC2 (1 of many)	-0.059692967	2.582271594	3.518368083	2.376076569
ENSDART00000134729	BX936308.1	-0.246065702	0.716060471	1.529870623	1.562652796
ENSDART00000134763	zgc:112056	0.091717396	-0.08997535	-0.362094913	-0.168303392
ENSDART00000134773	zfand5a	-0.170709233	-0.221425066	-0.980962271	-0.517481995
ENSDART00000134787	elavl4	1.446773831	1.526430349	1.657041106	1.070021064
ENSDART00000134801	ccdc92	0.127931369	0.677319918	0.797788944	0.697201329
ENSDART00000134813	rx1	-1.006947632	-0.71282108	-0.035273805	-0.106549447
ENSDART00000134816	slc2a3a	-0.406786727	-0.444029943	-0.052958142	0.129218418
ENSDART00000134819	slc5a6a	0.44313334	-0.037657655	-0.159985236	-0.577627715
ENSDART00000134832	rbpms2b	-1.576691045	-2.099790887	-0.701820765	0.187979586
ENSDART00000134843	sst6	1.638903894	2.735989277	2.458183626	2.301951228
ENSDART00000134855	efr3bb	-0.263384976	-0.381748192	-0.1453091	0.002074194

ENSDART00000134864	gigyf1b	-0.032906085	-0.141419247	-0.36943011	-0.170431533
ENSDART00000134868	zgc:113314	1.966628384	1.545280953	2.058380505	1.451511877
ENSDART00000134870	si:ch1073-170o4.1	0.916491768	1.072144968	0.377684395	0.341437061
ENSDART00000134890	dusp11	0.272279441	0.221032047	0.049148172	0.030930206
ENSDART00000134922	kctd7	-0.261930929	-0.25302271	-0.255500491	-0.12435787
ENSDART00000134924	actb2	0.443688406	0.512853066	0.501918371	0.442049165
ENSDART00000134934	pag1	-0.161885621	-0.157851391	-0.203479947	-0.293661261
ENSDART00000134941	prc1b	1.557351387	1.026365943	0.57100162	0.47167284
ENSDART00000134969	rnd2	-0.341329865	-0.337414256	-0.483308665	-0.257319117
ENSDART00000134973	pcnxl2	-0.772294262	-0.54715302	-0.00593982	0.313205165
ENSDART00000135029	BX927398.3	0.541935951	0.347132897	0.430074452	0.28825753
ENSDART00000135032	si:dkey-57a22.11	1.01281435	1.486077838	0.644934957	0.560278535
ENSDART00000135052	slc8a4a	-0.449869312	-0.676457859	-0.284767037	0.064060964
ENSDART00000135076	scrn3	0.148271997	-2.654959907	1.093269557	0.033366542
ENSDART00000135093	ola1	0.415909116	0.691871408	0.432580712	-0.033470782
ENSDART00000135118	efr3ba	-0.168363836	-0.303586815	-0.356914459	-0.264543553
ENSDART00000135125	serp2	4.40213E-05	0.384051284	0.40863701	0.667539121
ENSDART00000135133	FRMD5 (1 of many)	-0.238198919	-0.442975354	-0.623723948	-0.410224766
ENSDART00000135147	si:ch211-218d20.15	-0.111515958	0.398515484	0.749278715	0.887643411
ENSDART00000135164	pdcd6ip	0.194711079	0.272768586	0.208605784	0.054641806
ENSDART00000135184	pafah1b3	0.785314803	0.490699437	0.476757912	0.524529338
ENSDART00000135206	ppp6r2b	-0.281142732	-0.364406934	-0.331381396	-0.106942863
ENSDART00000135230	chd7	0.169349437	0.346809261	0.458651128	0.318074781
ENSDART00000135238	dok2	1.480525707	0.841647962	0.697333529	0.138703296
ENSDART00000135248	anxa13l	2.875521493	3.590436866	3.752911284	3.4448321
ENSDART00000135256	ubtd2	0.043462174	0.213810574	0.405778219	0.255726061
ENSDART00000135284	vsnl1a	-0.802778535	-0.782046001	-0.520311896	-0.04073792
ENSDART00000135285	clk2a	0.251961136	0.113407524	0.334958884	0.51104172
ENSDART00000135337	zgc:64189	0.108656283	-0.42063095	-0.437081395	-0.295412247
ENSDART00000135356	nabp1a	0.371235634	0.392313666	0.26759026	0.229305948
ENSDART00000135357	si:ch211-220f16.2	3.87916704	3.441079612	2.990059648	3.744387905
ENSDART00000135374	ptpn21	-0.060719081	-0.163907746	-0.321158981	-0.096688483
ENSDART00000135381	six4a	2.521510715	2.3671408	2.587075649	1.691172374
ENSDART00000135399	phf11	-0.105908519	0.331215825	2.01559672	0.351931859
ENSDART00000135403	nuak2	-0.203159013	-0.012266437	-0.34684763	-0.520334493
ENSDART00000135415	dysf	3.146286589	3.204629318	3.124686608	3.126558821
ENSDART00000135431	rabgap1l	1.914692926	2.028305841	2.310641205	1.631726628
ENSDART00000135436	jtb	-0.083914092	-0.13827795	-0.410566521	-0.208466854
ENSDART00000135472	zgc:86764	1.170468948	0.97326651	0.792803713	0.492808604
ENSDART00000135475	cadm2a	-0.589549243	-0.825400299	-0.33568035	0.065563209
ENSDART00000135479	fstl5	-0.187703631	-0.312045142	-0.413295205	-0.11029682
ENSDART00000135481	si:ch211-67n3.9	-0.264301646	-0.482759797	-0.356089399	-0.148916978
ENSDART00000135510	syne2b	-0.011968335	-0.47360345	-0.918119469	-0.289310189
ENSDART00000135513	rgs6	-0.368608418	-0.140264684	-0.010741291	0.084818744
ENSDART00000135517	cenpf	1.006357007	0.930294184	0.598168756	0.318387864
ENSDART00000135556	lonrf1	-0.330548829	0.12250287	-0.445965292	-0.332349033
ENSDART00000135569	gale	0.396996119	0.386168327	0.425741481	0.058303999
ENSDART00000135583	mlpha	2.247424425	4.049714869	4.15888721	1.709523081
ENSDART00000135587	cd9a	-0.312163633	-0.264335113	0.189621918	0.343999271
ENSDART00000135595	mhc1zaa	0.19156418	1.036766333	0.760670357	0.331702215
ENSDART00000135599	lypla2	-0.359594871	-0.446096937	-0.182980597	-0.086611879

ENSDART00000135602	si:ch73-290k24.6	-0.456455854	-0.524968049	-0.128888416	-0.002715321
ENSDART00000135606	TMEM269	-0.06366918	-0.165749005	-0.266297774	-0.18037018
ENSDART00000135608	gabrp	-2.860562828	-1.443836066	-0.572564003	-0.537301716
ENSDART00000135624	FAM163A	-0.714036121	-0.427639458	-0.142310427	0.163241634
ENSDART00000135631	myl6	0.462877699	0.806124657	0.938068157	0.529055927
ENSDART00000135661	slc20a1a	1.716116201	1.644628019	1.747440585	0.991048186
ENSDART00000135690	lactbl1a	-0.207283355	-0.397072585	-0.513557165	-0.348206521
ENSDART00000135692	UNC13A	-0.209420413	-0.513653337	-0.217224189	0.093101705
ENSDART00000135702	rgs11	-0.576481143	-0.793334647	-0.383725367	0.048951277
ENSDART00000135715	fam184a	-0.344643213	-0.481218539	-0.282456424	0.057932244
ENSDART00000135719	si:ch211-171h4.6	1.187005549	0.373648016	0.271137881	-0.125827965
ENSDART00000135730	lrmp	-0.028204227	-0.338623204	-0.590263694	-0.109841967
ENSDART00000135738	zgc:194990	0.067005172	-0.387370648	-0.731552205	-0.192679748
ENSDART00000135756	kirrel3a	-0.197416496	-0.816919597	-0.348887933	-0.004164348
ENSDART00000135764	si:dkey-70p6.1	-0.54777588	-0.724047584	0.062614493	0.292769909
ENSDART00000135768	lrp1ba	-0.685782746	-0.945707299	-0.285013804	0.195853428
ENSDART00000135781	plcg1	0.270370473	0.251405726	0.297242892	0.117629016
ENSDART00000135794	sepn1	0.371752242	0.285638022	0.016731978	-0.094169263
ENSDART00000135811	atp2b3a	-0.361290503	-0.436904447	-0.166639435	0.060424957
ENSDART00000135821	mfsd6b	0.610408738	1.551908888	1.729395905	1.289342978
ENSDART00000135860	rnf41l	-0.353446179	-0.473576771	-0.616364395	-0.289592085
ENSDART00000135888	rrp9	0.649352382	0.396806051	0.419459571	0.067410678
ENSDART00000135897	tmeff2a	-0.507501095	-0.570530323	-0.389897465	-0.071849112
ENSDART00000135919	amd1	-0.131714213	-0.276160556	-0.537901531	-0.163925689
ENSDART00000135965	plch1	-0.574356968	-0.567600235	-0.421755163	-0.154028312
ENSDART00000135973	nfyal	0.202128253	0.401502491	0.256237772	0.346886156
ENSDART00000135989	cep170aa	0.062401976	0.336049788	0.4713762	0.353973795
ENSDART00000136028	CR753844.1	0.308802389	0.230251216	0.146560512	0.144442899
ENSDART00000136049	syt10	-0.382437751	-0.322146795	-0.465356993	-0.383104059
ENSDART00000136084	mfhas1	-0.229093837	-0.123648609	-0.428888075	-0.346098599
ENSDART00000136091	c1ql2	-0.439731401	-0.550972938	-0.293500773	-0.243598849
ENSDART00000136103	ubap2a	0.173955465	0.276885256	0.421817829	0.278699527
ENSDART00000136123	kdm6bb	-0.32427763	-0.244056677	-0.170049978	-0.028150527
ENSDART00000136128	FP016056.1	-0.005351848	-0.270043208	-0.447280202	-0.270195958
ENSDART00000136141	kcnq3	-0.157652998	0.519381195	1.385651499	1.123132968
ENSDART00000136152	calua	0.347598327	0.562237059	0.505429013	0.302262295
ENSDART00000136154	gpr158a	-0.522434846	-0.680761714	-0.064607745	-0.081447185
ENSDART00000136161	faxcb	-0.355434657	-0.26288196	0.155305377	0.179364353
ENSDART00000136182	si:ch211-222n4.2	2.334818577	2.498262776	2.056155354	0.938824321
ENSDART00000136187	bloc1s4	0.27836522	0.479128543	0.397988078	0.159673351
ENSDART00000136190	si:ch211-133l5.7	0.046455816	-0.284643729	-0.455348095	-0.512893839
ENSDART00000136200	cbln2b	-0.48916567	-0.352468034	-0.442788192	-0.14816736
ENSDART00000136208	ncam1b	-0.292790209	-0.308372802	-0.421218735	-0.19466435
ENSDART00000136213	bzw2	0.202381787	-0.263816931	-0.545867534	-0.324585711
ENSDART00000136233	TENM3	-0.571502608	-0.593065001	0.242250375	0.436842415
ENSDART00000136239	kcnv2a	-0.144268598	-0.378520976	-0.127025179	0.043873086
ENSDART00000136255	otofa	-0.67545742	-0.815938232	-0.602392654	-0.103624276
ENSDART00000136267	slc35f3b	-0.474511888	-0.87524613	0.142496146	0.506913479
ENSDART00000136269	rimbp2	-0.196271668	-0.410552217	-0.160743106	0.064316711
ENSDART00000136296	CR847543.2	0.7189738	0.800339311	1.432214548	0.183639146
ENSDART00000136314	cthl	0.026768092	0.498239754	-0.095502024	-0.417996724

ENSDART00000136377	si:dkey-239h2.3	-0.060598158	-0.178560319	-0.404445305	-0.188298872
ENSDART00000136378	mb	0.060967695	-0.006783267	-0.887462271	-1.204052153
ENSDART00000136382	gadd45ba	1.037707985	1.186135766	0.645465362	0.508629
ENSDART00000136383	cd99	0.67248236	1.035021667	0.666974643	-0.164650894
ENSDART00000136386	si:ch211-210c8.7	0.436864275	0.595310791	0.386350533	0.024375719
ENSDART00000136390	hcn2b	-0.288020893	-0.351077892	-0.312560003	-0.049583355
ENSDART00000136418	sf3a1	0.245658224	0.479306215	0.291480298	0.207121133
ENSDART00000136439	bcl7a	0.000990732	0.374886928	0.452965996	0.230358497
ENSDART00000136447	si:dkey-161j23.7	-0.523562694	-0.013224352	-0.081189145	0.161013961
ENSDART00000136472	trim3b	-0.264051102	-0.238034524	-0.31432798	-0.179272082
ENSDART00000136488	zmat4a	-0.690962776	-0.637104526	-0.28999313	-0.088561559
ENSDART00000136492	si:ch211-158d24.4	0.620322497	0.916930916	0.610223614	0.170743477
ENSDART00000136497	CR318624.1	-0.290569983	-0.906819744	-0.441990313	0.029701559
ENSDART00000136523	CU467861.1	1.460403168	1.124457561	1.294733731	1.217332143
ENSDART00000136538	cish	-0.292880454	-0.45612089	-0.971812918	-1.16494411
ENSDART00000136543	tnni4b.1	1.504503498	0.982879899	0.857598266	-0.145526765
ENSDART00000136547	rpl10a	0.572905103	0.588248568	0.30592141	0.14058962
ENSDART00000136559	ano8a	-0.477178876	-0.572871399	-0.340924619	-0.093243478
ENSDART00000136578	syn1	-0.141221338	-0.191909782	0.363564902	0.481540125
ENSDART00000136592	ckbb	-0.985379774	-1.268435214	-0.989267529	-0.749991465
ENSDART00000136647	ext1c	-0.283149695	-0.275883116	-0.465218527	-0.113484828
ENSDART00000136649	si:dkey-92i17.2	0.27365425	1.100188551	1.105138404	0.404606112
ENSDART00000136653	ccdc106b	0.176566138	-0.096095881	-1.050174868	-0.31850308
ENSDART00000136654	grapb	-0.34841027	-0.721289328	-0.501261917	-0.353877694
ENSDART00000136655	ndufa3	-0.198867993	-0.196758365	-0.355261116	-0.136756127
ENSDART00000136692	ccnl1a	0.306138381	0.080789654	-0.032909692	0.080389121
ENSDART00000136695	yrk	1.103276689	0.393392175	0.380351314	-0.354474005
ENSDART00000136700	hunk	1.085007384	0.795931304	0.468549793	1.046395882
ENSDART00000136707	mical1	-0.443299702	-0.458390885	0.146469401	0.374257303
ENSDART00000136708	rbpms2b	-1.024689498	-0.80090545	-0.103078191	0.159293341
ENSDART00000136722	cdkn1a	1.083504495	0.97819224	0.258587523	0.060123909
ENSDART00000136726	magi3b	-0.381646177	-0.461208385	-0.226303301	-0.080158554
ENSDART00000136729	ebf1b	-2.79991744	-3.068779235	-0.720206417	0.313112936
ENSDART00000136733	glra1	-0.259893278	-0.432961012	-0.409765299	-0.248913141
ENSDART00000136744	foxq2	-0.278235678	-0.492834499	-0.366505779	-0.190958275
ENSDART00000136754	cdh13	-0.587587145	-0.627840519	-0.172128457	0.142910694
ENSDART00000136759	prdm13	-0.225066145	-0.413438688	-0.129520305	0.036146962
ENSDART00000136761	prkx	0.698491961	1.03323314	0.943847516	0.577218336
ENSDART00000136770	si:dkey-246e1.3	-0.264481758	-0.308595158	-0.554881445	-0.406535962
ENSDART00000136771	dnajc5ga	-0.246041931	-0.267357646	-0.352933326	-0.221144382
ENSDART00000136779	entpd5b	0.819562392	0.580844217	0.174817773	-0.04896424
ENSDART00000136782	si:dkey-24c2.7	4.902793471	4.654641885	4.451812012	3.610899339
ENSDART00000136789	tjp2a	-0.247782376	-0.511487885	-0.433268095	-0.195886379
ENSDART00000136797	btbd10a	-0.201172343	-0.244946837	0.724827561	0.837222426
ENSDART00000136805	rbm10	-4.539752918	-3.15582054	-1.19980751	-2.046490722
ENSDART00000136830	stmn4l	1.323269884	1.682347243	1.639725976	1.096832109
ENSDART00000136835	gpr84	0.70622544	0.183343809	-0.153052934	-0.427967202
ENSDART00000136836	rab11fip3	-0.285287519	-0.130906101	0.115401989	0.056439488
ENSDART00000136853	fam163b	-0.426911899	-0.715978676	-0.456698496	-0.087825906
ENSDART00000136865	cfp	0.329536618	0.225136408	-0.193120136	-0.891420593
ENSDART00000136873	clasp2	0.301439566	0.245236713	0.309865242	0.356365378

ENSDART00000136910	armc1l	0.626427947	0.775572599	0.634581641	0.292392529
ENSDART00000136914	tpd52l2a	-0.012405325	-0.254912754	-0.378255822	-0.151435535
ENSDART00000136943	si:dkey-21e2.13	2.273396315	3.247336732	1.860650754	0.467276759
ENSDART00000136977	wdr11	-0.244389043	-0.296442513	-0.19757682	-0.033240657
ENSDART00000137019	cmklr1	1.293423342	0.826870113	0.435422926	0.179038361
ENSDART00000137021	cabp4	-0.36160439	-0.600623701	-0.526609212	-0.179412794
ENSDART00000137026	fam208ab	-0.664193623	-0.40965157	-0.455043185	-0.262800574
ENSDART00000137036	ppiaa	0.323927062	0.685997539	0.775560135	0.372712842
ENSDART00000137037	iqsec3b	-0.405090433	-0.576572578	-0.30180595	0.030413937
ENSDART00000137038	asic2	-0.373156822	-0.72567768	-0.446804249	-0.152680828
ENSDART00000137047	scoca	-0.178388629	-0.216665672	-0.49140437	-0.218641566
ENSDART00000137061	klc1a	-0.086604178	0.023703263	0.276488629	0.211488076
ENSDART00000137083	impg2a	-0.438459919	-0.214037195	-0.28206161	-0.025553958
ENSDART00000137098	impdh1a	-0.236861779	-0.534821929	-0.182618892	0.088190256
ENSDART00000137110	sepw1	0.165066135	0.642417047	0.502149684	0.242826446
ENSDART00000137131	dnah7	-0.559601026	-0.233046623	-0.366131677	-0.080127176
ENSDART00000137135	mei4	-0.382006481	-0.256759987	-1.043399236	-0.457749266
ENSDART00000137185	cnga3b	-0.145832564	-0.301563517	-1.136000666	-0.708190865
ENSDART00000137203	ponzr6	2.803770034	2.268583293	2.342420518	1.869529587
ENSDART00000137212	dip2ca	0.131800934	-0.026475424	0.308770191	0.394512532
ENSDART00000137214	gpm6bb	-0.410929898	-0.351705205	-0.352631557	-0.244368267
ENSDART00000137228	si:rp71-80o10.4	-0.005305454	1.094390245	1.175846782	0.577915575
ENSDART00000137236	hpcal1	-0.065446201	-0.487196869	-0.639863396	-0.431847778
ENSDART00000137262	vdac3	-0.257205324	-0.141410703	-0.061503392	0.052713477
ENSDART00000137287	pebp1	-0.410574808	-0.266170824	-0.343257245	-0.128567003
ENSDART00000137291	METTL18	-0.209734106	0.269511362	-2.440335414	-0.139098945
ENSDART00000137292	tmem106bb	-0.265825137	-0.29210537	-0.411370487	-0.25270276
ENSDART00000137293	dia1b	-0.377972932	-0.365130624	-0.231479469	-0.228625621
ENSDART00000137308	plxnb1a	0.257511262	0.267128747	0.167145068	0.075399376
ENSDART00000137309	gemin5	0.29381973	0.279160353	0.398962684	0.210137956
ENSDART00000137315	rdh12	-0.392438907	-0.330126959	0.208728813	0.139463991
ENSDART00000137325	mvp	1.415505945	1.485945809	1.461661333	0.448751577
ENSDART00000137332	si:ch211-132g1.3	-2.704437973	-0.017410436	-0.395377626	-0.276512676
ENSDART00000137353	klhl17	-0.368352542	-0.314849207	-0.039056928	0.155772163
ENSDART00000137355	tet3	-0.245273707	-0.407219331	-0.050664281	-0.06569376
ENSDART00000137364	si:dkey-33c12.3	0.174067673	-0.534549963	-0.496919129	0.189169246
ENSDART00000137371	atp8b3	-0.113445648	-0.348083251	-0.436781891	-0.110584084
ENSDART00000137373	si:ch211-266i6.3	1.973293451	1.253222026	0.772689981	0.242956342
ENSDART00000137381	khdrbs1b	-0.12744256	-0.621948205	-0.491970892	0.090347508
ENSDART00000137391	celsr3	0.73044715	0.817745044	0.845582062	0.571451225
ENSDART00000137418	BX004774.2	-0.041779011	-0.239619277	-0.711634051	-0.543815741
ENSDART00000137424	c1qtnf4	-0.387372103	-0.312978581	-0.242907328	-0.243606392
ENSDART00000137426	gcgb	-0.226806487	-0.599969225	-0.659481129	-0.843993634
ENSDART00000137443	ANK1 (1 of many)	-0.657062014	-0.810024547	-0.456200062	-0.254232075
ENSDART00000137471	MFAP4 (1 of many)	1.677720699	2.807166131	2.128226179	1.146528193
ENSDART00000137485	tpd52l1	0.045987753	-0.292323377	-0.626535278	-0.266196407
ENSDART00000137486	klhl21	0.151379043	0.263264305	0.384374563	0.339611926
ENSDART00000137505	vamp1	-0.431791589	-0.393372103	-0.433651273	-0.142134375
ENSDART00000137561	ucp3	2.475646127	3.272328187	3.19387131	0.905806223
ENSDART00000137572	mpc1	-0.077665143	-0.162227554	-0.29007527	-0.035755645
ENSDART00000137620	si:ch73-46j18.5	-0.071928647	0.074859157	0.409828499	0.45946174

ENSDART00000137624	eef2a.2	2.619194541	2.67605773	3.551783602	3.00375243
ENSDART00000137628	mthfd1l	3.69201835	0.840641874	3.245529647	1.343967089
ENSDART00000137633	si:ch211-194e15.5	-0.00297372	-0.215384681	-0.930894546	-0.593142383
ENSDART00000137638	si:ch73-25f10.6	-0.304397619	-0.080258192	-0.006488078	0.003923632
ENSDART00000137648	si:dkeyp-123h10.2	-0.55097741	-0.596976893	-0.447594093	-0.275058949
ENSDART00000137659	cmpk2	0.149738862	-0.572975919	2.540871599	-0.412773402
ENSDART00000137679	sash1a	0.328343986	-0.118753926	-0.077897758	-0.030798
ENSDART00000137680	BX663503.1	-0.232606929	-0.407355209	-0.255389687	-0.079162056
ENSDART00000137728	st6gal1	-0.677667407	-0.699420985	-0.736040173	-0.083670402
ENSDART00000137756	hecw2a	-0.728856656	-1.003560302	-0.22088311	0.383324077
ENSDART00000137757	irf9	0.327252488	0.410367721	0.769408328	0.032820811
ENSDART00000137782	yif1a	0.379643142	0.667454267	0.241343175	0.280143891
ENSDART00000137795	ssrp1a	0.416335187	0.470997147	0.449691903	0.161896374
ENSDART00000137799	trim66	-0.46849178	-0.260779632	0.306613928	0.711500351
ENSDART00000137817	rap1gap2a	-0.351211604	-0.330279371	-0.569882036	-0.258477103
ENSDART00000137848	palmda	-0.364561265	-0.248835039	-0.231952958	-0.127331926
ENSDART00000137851	tfr1b	0.020013224	-0.000851268	0.44833745	0.43610072
ENSDART00000137858	alpk2	0.667374473	0.189664169	0.047615444	-0.048884335
ENSDART00000137889	ank1b	-0.447220804	-0.737203628	0.205089912	0.418795119
ENSDART00000137896	fibpb	-0.042394017	-0.144008268	-0.458312875	-0.18906893
ENSDART00000137899	tmem9	-0.365732748	-0.460492017	-0.592427521	-0.282793654
ENSDART00000137900	grin2ab	-0.452993272	-0.616161489	0.576592075	1.050321835
ENSDART00000137903	BX640512.2	-0.158278671	-0.01949183	1.915495669	-0.120027797
ENSDART00000137918	bbc3	0.101268325	0.767629492	0.853650102	0.865966102
ENSDART00000137920	si:ch211-169p10.1	-0.466773792	-0.281022003	0.027119426	-0.105594514
ENSDART00000137967	diras1b	-0.821870921	-0.072822932	0.659174512	0.085565822
ENSDART00000137973	grna	1.322360375	0.965948715	0.567189356	-0.062803655
ENSDART00000137984	SBNO2 (1 of many)	0.66540874	0.705510404	0.683617676	-0.079102756
ENSDART00000138026	gabra5	-0.35782795	-0.794620157	0.43219928	0.956240287
ENSDART00000138045	whsc1l1	-0.152220762	0.158744769	0.494234043	0.50708853
ENSDART00000138048	pbx3b	-0.599655595	-0.602159282	-0.228775798	-0.019503135
ENSDART00000138070	mb	-0.127824671	-0.447974107	-0.919218534	-1.274065689
ENSDART00000138081	bcl6ab	0.948060654	0.690527167	0.988530239	1.012261919
ENSDART00000138094	eef1b2	0.3943229	0.586163757	0.433468435	0.083803882
ENSDART00000138116	surf6	0.641305655	0.652953357	0.565146387	0.291720746
ENSDART00000138120	pbdc1	0.312703559	0.29678566	0.127166498	0.014012212
ENSDART00000138139	zgc:153157	-0.590921601	-0.369202687	-0.339560998	-0.377004198
ENSDART00000138140	SLC4A5 (1 of many)	-0.322967614	-0.430634585	-0.36463151	-0.15311364
ENSDART00000138143	si:ch211-233m11.1	0.782546411	0.773895723	0.974137465	0.285762089
ENSDART00000138174	slc16a12b	-0.100392011	0.567507799	1.765497005	0.866245296
ENSDART00000138185	gad1b	-0.304744145	-0.418178899	-0.358818296	-0.231950915
ENSDART00000138192	tnnt3b	-0.439749688	3.945748378	4.736531723	2.568867245
ENSDART00000138210	ubac1	-0.339032913	-0.702323176	-0.152684805	0.046765242
ENSDART00000138216	CR855311.1	1.84161592	1.501667665	0.707831526	-0.102637826
ENSDART00000138223	si:ch211-71m22.1	0.850777626	0.641874022	1.554600154	-0.004827675
ENSDART00000138232	arl13a	-0.123428499	-0.297389057	-0.827109894	-0.393133456
ENSDART00000138240	tpt1	2.855871394	2.728090369	3.227118826	0.916859453
ENSDART00000138246	EMB	-0.957103253	-1.089886746	-0.283796575	0.121320475
ENSDART00000138270	copz2	-0.133761169	-0.127273242	-0.35316272	-0.169760508
ENSDART00000138289	prex1	-0.517788451	-0.174645698	-0.909102985	-0.603337454
ENSDART00000138294	rab41	-0.246606124	-0.179576466	0.274617961	0.438688085

ENSDART00000138303	si:dkey-23c22.9	0.616317614	0.5640973	0.527774625	0.331889659
ENSDART00000138308	gpr186	-1.473279373	-0.953004046	-1.169373422	-1.314238919
ENSDART00000138312	zbtb18	-0.233659879	-0.456610703	-0.155318743	0.01760237
ENSDART00000138321	parp12b	0.255975647	0.107710029	0.742615255	0.386793554
ENSDART00000138328	xpo6	0.06358993	0.090933498	0.285770037	0.126601033
ENSDART00000138334	ppp2r2ab	-0.498478384	-0.353284175	-0.096797126	-0.036101553
ENSDART00000138350	rps12	0.592717981	0.744811164	0.572850481	0.279364485
ENSDART00000138351	cth	1.012814265	1.438428325	1.066275824	0.495805428
ENSDART00000138404	si:ch211-199g17.2	1.114605772	0.829720741	1.042136455	0.129449804
ENSDART00000138412	acbd5a	0.434518843	0.610473848	0.985396736	0.824271669
ENSDART00000138415	rnf207b	0.16648554	0.461805711	0.480213875	0.411411872
ENSDART00000138432	cul2	0.103145244	0.093005828	0.308783998	0.205589181
ENSDART00000138475	sorbs2a	-0.319831096	-0.27540368	-0.20838001	-0.137765232
ENSDART00000138477	BX571945.1	-0.597559877	-0.845904193	-0.62766334	-0.501592231
ENSDART00000138527	rab20	0.632157851	0.410164386	0.278506263	0.009483031
ENSDART00000138540	ttyh1	-0.563796304	-0.437755604	-0.092270149	0.165178407
ENSDART00000138541	rpgrip1	-0.096214383	-0.271667846	-0.617065899	-0.286110235
ENSDART00000138606	las1l	0.170918093	0.302723725	0.361920168	0.174466647
ENSDART00000138621	zgc:153867	3.188427096	3.682050782	3.111570491	2.809604178
ENSDART00000138633	cd99	1.201301736	1.818711588	1.295470825	0.581149232
ENSDART00000138658	si:ch211-117m20.4	-0.301116828	-0.255854166	-0.65213458	-0.329202684
ENSDART00000138661	tspan11	-0.312645804	-0.301551652	-0.855364566	-0.684847545
ENSDART00000138687	ppp1r9a	-0.410087357	-0.337226328	-0.257566135	-0.006942521
ENSDART00000138695	ank1b	-0.498943842	-0.553539638	-0.03799849	0.33423854
ENSDART00000138703	trpc7a	-0.333459575	-0.723289936	-0.168085186	-0.375723791
ENSDART00000138714	sult5a1	2.445310663	3.836816007	1.972331729	2.072167418
ENSDART00000138719	cyp3c4	-2.572466969	-4.228419984	0.354827699	-0.36271005
ENSDART00000138733	cdh12a	-0.402719414	-0.467471363	-0.247597318	-0.111519505
ENSDART00000138747	urb2	0.51682098	0.426109616	0.379169845	0.148916896
ENSDART00000138755	dclk1a	-0.464513032	-0.326162873	-0.241704881	-0.01162875
ENSDART00000138759	clta	-0.330987711	-0.247185854	1.110067577	0.98649153
ENSDART00000138769	cdh24b	-0.290981343	-0.436100905	-0.352222227	-0.098902248
ENSDART00000138793	si:ch211-203k16.3	-0.114803625	-0.341143223	-0.894722004	-0.064858026
ENSDART00000138820	lrtm2a	-0.205456304	-0.473378158	-0.285055681	-0.252340958
ENSDART00000138821	nfil3	1.376309909	2.121391468	1.99718084	1.39511595
ENSDART00000138822	SSBP4 (1 of many)	-0.268549166	-0.417362634	-0.271649078	-0.060566364
ENSDART00000138834	mab21l3	-0.591171056	-0.506133859	-0.165242719	-0.104286764
ENSDART00000138845	BX571724.1	-0.626541209	-0.809565722	-0.893218772	-2.725413714
ENSDART00000138850	cacna2d2b	-0.226630579	-0.433395437	-0.06209639	0.11961013
ENSDART00000138866	pbxip1a	-0.45481847	-0.358362517	-0.587330976	-0.331438505
ENSDART00000138878	ndufaf5	-0.068766975	-0.169285384	-0.3114844	-0.186576816
ENSDART00000138890	kcnd2	-0.399063976	-0.347742148	-0.33423415	-0.044907554
ENSDART00000138895	igsf9ba	-0.084504285	-0.116086258	0.293952477	0.264801901
ENSDART00000138915	cirbpb	0.297824374	0.314736859	0.24846932	0.269446209
ENSDART00000138922	elovl8a	-0.299886352	-0.255106336	-0.59316691	-0.339691401
ENSDART00000138954	plekha1b	-0.288171631	-0.266395551	0.185168153	0.476938782
ENSDART00000138963	rcan1a	0.723709877	0.557125789	0.817932454	0.892580049
ENSDART00000138995	vps9d1	0.170379692	-2.63011314	-0.343991992	-0.469718482
ENSDART00000138996	si:ch211-214p13.9	1.396990228	1.323446174	0.865221946	-0.306555509
ENSDART00000139002	dus2	0.373595906	0.357863942	0.47349873	0.230284081
ENSDART00000139033	lrp1bb	-0.300129828	-0.380241873	-0.239929923	-0.108887683

ENSDART00000139035	si:ch211-215c18.3	-1.205532305	-0.512285433	0.437440349	0.984798646
ENSDART00000139038	CU467861.1	1.207959792	0.605422627	0.760051253	0.598045855
ENSDART00000139042	si:ch211-132b12.7	0.072730857	-0.322841214	-0.765244522	-0.309126323
ENSDART00000139048	glsb	-0.011350798	-0.181323189	-0.40931902	-0.142239159
ENSDART00000139069	decr1	0.368869625	0.410230162	-0.003538468	-0.070408167
ENSDART00000139087	lgmn	0.782730519	0.338051823	0.153128345	-0.09953272
ENSDART00000139102	dbpb	0.113164671	-0.364559303	-0.423196921	-0.137052116
ENSDART00000139124	ckba	1.776728636	1.213212461	1.201789834	0.178980804
ENSDART00000139132	steap3	0.144648286	0.301641021	0.828461526	-0.036698505
ENSDART00000139151	si:ch211-193k19.1	1.470035909	0.356445905	0.995091982	1.049578611
ENSDART00000139174	fam46c	-0.16066992	-0.125475027	-0.118453643	-0.616919987
ENSDART00000139176	eif2s3	0.381558608	0.49753559	0.407153714	0.243011405
ENSDART00000139178	slc25a14	-0.405048408	-0.151867564	-0.020130795	0.082212943
ENSDART00000139191	trpc1	-0.495478264	-0.440776327	-0.289497882	-0.094213915
ENSDART00000139196	kif1ab	-0.338072705	-0.653527626	-0.314444638	-0.193117382
ENSDART00000139237	ret	-0.448934402	-0.273383642	-0.43725639	-0.287562193
ENSDART00000139241	rnf38	0.363207367	0.385948281	0.537494883	0.39766179
ENSDART00000139259	zfand5b	0.677389122	0.588881516	0.462655957	0.625250498
ENSDART00000139265	si:ch211-12e13.12	-0.4797238	-0.27645528	0.604278273	0.83384752
ENSDART00000139279	rbpms2b	-0.774960547	-0.830410704	0.12323811	0.508122144
ENSDART00000139299	zgc:162928	0.559235647	0.580335587	1.258831581	1.081287075
ENSDART00000139310	bcat2	-0.180902608	-0.445596686	-0.579108461	-0.343900677
ENSDART00000139324	si:dkeyp-41f9.3	-0.154282935	-0.395797654	-0.452635792	-0.310260961
ENSDART00000139329	cry-dash	-0.104880347	0.380813844	-0.32837694	-0.194368371
ENSDART00000139339	mrpl34	-0.322878188	-0.414873817	-0.404846888	-0.143871621
ENSDART00000139368	atp1b1a	1.877087153	1.808227818	1.283859651	0.966677715
ENSDART00000139387	atp1b1b	-0.121937317	0.340318468	0.40085255	0.510906972
ENSDART00000139392	rras	2.928045636	3.065800127	2.84735064	1.624630606
ENSDART00000139412	SMIM18	0.730335352	0.956017708	0.99931328	0.671209714
ENSDART00000139448	GALNTL6	-0.494238764	-0.383984121	-0.129586789	0.079582323
ENSDART00000139449	si:dkey-147f3.4	1.865073313	2.098559635	1.808022762	0.571602171
ENSDART00000139452	pde4d	0.066848851	-0.561165148	-0.294908352	0.123175575
ENSDART00000139454	pde4cb	0.616688899	0.67005432	0.986689732	0.473877974
ENSDART00000139461	spock3	-0.122253969	-0.220033244	-0.389207603	-0.090203867
ENSDART00000139464	CU467861.1	0.786254771	0.561892643	0.40560384	0.357004849
ENSDART00000139475	samd10b	-0.569533963	-0.524261904	-0.125238267	-0.046566236
ENSDART00000139477	trim110	-0.21821102	-0.289321848	-0.373728133	-0.159590708
ENSDART00000139479	slc12a9	0.435405363	0.483188456	0.53485188	0.300178427
ENSDART00000139493	olfm1b	-0.400757951	-0.50188464	-0.148663949	0.07973127
ENSDART00000139514	ano11	-1.063704055	-1.17363318	-0.628817004	-0.055546726
ENSDART00000139517	bbs4	-0.163398089	-0.230989705	-0.424869507	-0.049448344
ENSDART00000139560	ppp3r1a	-0.205912354	-0.312380575	-0.194151002	0.010760735
ENSDART00000139565	L3MBTL4	-0.137141942	-0.164863308	-0.259223674	-0.097063711
ENSDART00000139568	nsmfa	-0.224590353	-0.433156538	-0.157442583	-0.141070436
ENSDART00000139569	zgc:92658	-0.993075003	-0.842810673	-0.747118722	-0.361693255
ENSDART00000139596	adarb2	-0.297400744	-0.411863485	-0.163774062	-0.140762569
ENSDART00000139608	upf3a	-0.436308546	-0.475063889	-0.33694709	-0.215591369
ENSDART00000139623	si:dkey-85k15.6	1.245336652	2.138651628	1.967890086	1.386101724
ENSDART00000139633	pip4k2ab	-0.336773688	-0.358059228	-0.306910553	-0.048531465
ENSDART00000139644	lin7b	-0.266884847	0.193113139	-0.021741296	0.002335697
ENSDART00000139659	si:dkey-222f2.7	1.108120911	0.818052369	0.396214124	-0.100365974

ENSDART00000139668	sept8a	1.681949125	2.926818092	4.206471175	2.971436039
ENSDART00000139677	si:ch211-274p24.4	-0.523882432	-0.343944487	-0.011557753	0.069892959
ENSDART00000139682	eps8l3b	1.489223017	0.930088019	0.068279636	-0.057094698
ENSDART00000139684	lypla2	-0.275250904	-0.278688664	-0.279438301	-0.171299302
ENSDART00000139701	zfand5a	-0.357581428	-0.278083252	-0.905903537	-0.782247372
ENSDART00000139702	casp3a	0.559260909	1.157211853	1.445414898	1.128298777
ENSDART00000139715	dpm3	-0.11194386	-0.125527265	-0.41579896	-0.188378465
ENSDART00000139722	dmtn	1.105433633	1.577455563	1.872515003	1.217364091
ENSDART00000139727	si:dkey-14o18.2	-0.196190231	-0.516815682	-0.462096519	-0.093362239
ENSDART00000139763	stat5a	3.442434617	3.964844196	3.417693165	3.322184683
ENSDART00000139793	BX324164.1	-0.743832764	-0.340035488	-0.551790628	-0.137670464
ENSDART00000139795	syt2a	-0.704240682	-0.905957314	-0.438675098	0.054000022
ENSDART00000139796	BX276103.2	0.569427954	0.391382554	0.350830467	0.475593369
ENSDART00000139834	mybpha	-0.819804571	2.682359397	3.592112028	2.697092568
ENSDART00000139839	rbmx	0.438924108	0.178102249	0.089667681	0.22023097
ENSDART00000139841	gabrb1	-0.439523493	-0.431067027	-0.18481289	0.263500104
ENSDART00000139863	dcbld2	0.210663919	0.284613411	0.515685864	0.299926066
ENSDART00000139872	oip5-as1	-0.165940131	-0.248925583	-0.345111453	-0.229952519
ENSDART00000139892	elfn2a	0.520234542	0.439147218	0.642970325	0.553931258
ENSDART00000139893	kcnma1a	-0.580697526	-0.766998113	-0.101431021	-0.110885596
ENSDART00000139900	diaph3	0.052388836	-0.258521684	-0.529199016	-0.186043701
ENSDART00000139917	myha	0.315818594	4.091377824	5.948901319	4.44901869
ENSDART00000139937	frmpd1a	-0.391315372	-0.461157908	-0.442405648	-0.041073617
ENSDART00000139947	rapgef1a	0.201307435	0.3482683	0.17911742	0.096155984
ENSDART00000139954	BX005364.1	-0.374788931	-0.616534875	-0.121001872	0.068005074
ENSDART00000139964	rps27.2	1.536999183	2.160860082	1.306857449	1.796586172
ENSDART00000139994	bbx	-0.232049516	-0.386470684	-0.08432371	-0.161403736
ENSDART00000140026	smarcd3a	-0.62517615	-0.573651045	0.020278487	0.08791531
ENSDART00000140028	lrrc4.1	-0.491138395	-0.422849217	-0.374271959	-0.216352385
ENSDART00000140032	si:dkeyp-69c1.9	1.662700986	3.288206015	1.780235269	1.584891446
ENSDART00000140058	zgc:153867	-0.784474297	-0.55629273	0.467767396	0.765384352
ENSDART00000140086	SUN2	0.64389802	0.258658486	-0.305248612	-0.209793481
ENSDART00000140089	cngb1a	-0.120444883	-0.269286081	-0.242009625	-0.11168715
ENSDART00000140133	ldhbb	-1.001178594	-1.13585969	-1.022737795	-0.597954004
ENSDART00000140136	soga3b	-0.191550416	-0.105037683	0.304311303	0.351545117
ENSDART00000140145	ajap1	-0.237572949	-0.464416488	-0.62912768	-0.262821389
ENSDART00000140153	mri1	1.105039291	1.085223643	1.409079861	0.961058569
ENSDART00000140161	necab2	-0.820451686	-0.597146212	0.232199679	0.59216621
ENSDART00000140201	cct8	-0.644125552	-3.075796442	0.532907513	0.591603801
ENSDART00000140213	npas1	-0.414273238	-0.495063512	-0.247065017	-0.227188437
ENSDART00000140220	si:ch211-168k14.2	-0.180662482	-0.408562234	-0.217555172	-0.18329178
ENSDART00000140226	CYTH2	0.074864847	0.258157093	0.345085089	0.148251292
ENSDART00000140230	zgc:123068	0.653257046	0.043755773	2.027002482	0.024521261
ENSDART00000140253	arhgef10lb	0.447026855	0.470705895	0.502550702	0.280062014
ENSDART00000140255	si:dkey-12h9.6	0.01002873	0.192183301	0.434559619	0.43853246
ENSDART00000140284	zbtb16a	2.407975491	2.022869932	2.341949009	2.679474288
ENSDART00000140301	TTC9	0.099722728	0.582192427	0.813799221	0.513650171
ENSDART00000140302	naca	0.278198217	-2.435431446	-4.196452356	-2.112057753
ENSDART00000140365	slc12a5b	-0.506920714	-0.535232712	-0.246533716	0.015331251
ENSDART00000140375	zfyve27	-0.214217108	-0.191210439	-0.353015645	-0.21311838
ENSDART00000140422	slmapb	-0.858203956	-0.622061933	-0.375625924	0.17086689

ENSDART00000140430	ssx2ipb	-4.013140014	0.070172399	-2.728976212	-1.746786541
ENSDART00000140436	map2	-0.405015536	-0.195771527	-0.230781013	0.041022881
ENSDART00000140448	c1ql4a	-0.329031661	-0.450164426	-0.498485743	-0.359733933
ENSDART00000140469	deptor	-0.370280506	-0.334232129	-0.271445752	-0.25182283
ENSDART00000140476	atp6v0a2a	-0.389741082	0.011917076	0.174652763	0.265809184
ENSDART00000140481	nfu1	-0.046571941	-0.14924591	-0.367699445	-0.24132739
ENSDART00000140490	MFAP4 (1 of many)	1.29105804	2.228445007	1.795046858	1.318796638
ENSDART00000140523	mrc1b	0.693910087	0.60918831	0.270261416	-0.09789336
ENSDART00000140537	dhrs11a	0.251388963	0.647205288	0.714640117	0.4437028
ENSDART00000140540	si:ch211-241e1.3	0.461435917	0.712590644	1.616566418	0.894218174
ENSDART00000140553	frmpd1a	-0.279082186	-0.31970424	-0.46726564	-0.019879501
ENSDART00000140575	map4l	-0.647123698	-0.438384786	-0.184748643	-0.024632978
ENSDART00000140586	col1a1a	0.136049595	0.737681947	1.130025782	0.5982928
ENSDART00000140615	cry1ba	-0.093526328	0.597915555	0.380272469	0.502019284
ENSDART00000140632	bcl2l13	-0.49849375	-0.354616708	-0.335413941	-0.223271321
ENSDART00000140645	si:ch211-69e5.1	0.001617087	-0.376948151	-0.916309926	-0.180025883
ENSDART00000140650	ankrd6b	-0.31619699	-0.471630097	-0.512160686	-0.029088129
ENSDART00000140667	spna2	0.558956985	0.663728247	0.276626138	0.347556663
ENSDART00000140694	eif4a1a	0.811760097	0.762478852	0.622630963	0.570594418
ENSDART00000140695	cdca7a	1.06377599	0.897895687	0.374788915	0.044135144
ENSDART00000140711	tbc1d5	0.254578098	0.244282662	0.119026019	-0.013937387
ENSDART00000140718	sdhc	-0.135426633	-0.207907336	-0.390001522	-0.128998947
ENSDART00000140736	si:dkey-42i9.6	0.573524661	0.712949488	0.985257189	0.193370375
ENSDART00000140739	lrrc4bb	-0.565179268	-0.464872302	-0.152447222	-0.079782637
ENSDART00000140764	si:ch211-262h13.3	0.50717215	0.501857896	0.472590221	0.360293055
ENSDART00000140776	CR391940.1	0.57109507	0.234979352	0.004616304	-0.046528801
ENSDART00000140779	fundc1	-1.365145935	-0.380101108	-0.950161557	-1.079798871
ENSDART00000140782	proza	-0.315906897	-0.22277663	-0.304920909	-0.22580936
ENSDART00000140786	qtrtd1	0.520156295	0.318813771	0.348267514	0.13612068
ENSDART00000140788	gbp1	3.129821614	2.838243862	2.757243294	2.490411238
ENSDART00000140819	rgmd	-0.726396179	-0.581866824	-0.277540481	-0.271416627
ENSDART00000140827	bcl11ab	-0.293093875	-0.330078462	0.796150714	1.216507448
ENSDART00000140828	CU179758.1	-0.432947507	-0.14249918	-0.435392677	-0.381420161
ENSDART00000140852	rgcc	1.458728484	1.527927815	1.077034908	0.967514776
ENSDART00000140874	seph	0.619876107	0.651122471	0.195858296	-0.082542729
ENSDART00000140875	zgc:123010	0.555608218	0.491589354	0.22352835	-0.077508349
ENSDART00000140897	larp6	0.136488111	0.20186899	0.305132975	0.028670036
ENSDART00000140908	gse1	0.266945251	0.781532641	1.123032019	0.70398488
ENSDART00000140920	prom1b	0.88825196	0.404698565	0.393910982	0.372363663
ENSDART00000140940	cluhb	-1.005907144	-1.058846619	1.005581701	-0.029667711
ENSDART00000140970	si:ch73-52p7.1	1.046367332	0.798731839	0.265928239	-0.108652338
ENSDART00000140995	mfsd2b	-0.430905256	-0.255202968	-0.49728314	-0.134050367
ENSDART00000141030	pde4ca	-0.308097694	-0.281644648	-0.391359015	-0.416436171
ENSDART00000141031	BX004774.2	-0.096197487	-0.455215086	-0.947274727	-0.721728098
ENSDART00000141062	man2b1	2.731854161	3.054858723	3.461869821	2.8427063
ENSDART00000141068	sox11b	1.611838536	2.177304965	2.010624009	1.194765222
ENSDART00000141086	robo2	-0.237929535	-0.432116468	-0.02855362	0.131332743
ENSDART00000141087	crema	0.064696008	-0.100227063	-0.477732232	-0.233875873
ENSDART00000141103	gfra4a	-0.446789167	-0.472299982	-0.170582645	0.04427624
ENSDART00000141107	cd99	2.277092862	3.207492883	2.696592348	2.108764519
ENSDART00000141110	rpl10a	0.638320568	0.343299632	0.070055535	0.288835787

ENSDART00000141132	ppp6r2b	-0.175401955	-0.328399574	-0.337567841	-0.076173198
ENSDART00000141142	mvp	1.187603651	1.369710798	0.919014152	0.045597494
ENSDART00000141157	ablim3	-0.090063728	-0.09247473	-0.384522771	0.048083962
ENSDART00000141173	elavl4	2.4638469	2.569363943	2.090923257	1.273250901
ENSDART00000141177	slc24a4a	-0.524899894	-0.691002096	-0.394086808	-0.038902918
ENSDART00000141188	st3gal3b	0.577420785	0.203550656	-0.28232291	-0.213031688
ENSDART00000141193	clu	-0.012066925	0.064405046	-0.155611703	-0.357238215
ENSDART00000141194	mpp4a	-0.333593633	-0.165171491	-0.10082691	0.099007973
ENSDART00000141224	rpl4	0.608116905	0.375484686	0.195287178	0.211394215
ENSDART00000141227	anxa13l	-1.29005108	2.955855077	2.234819741	2.935945321
ENSDART00000141231	si:dkey-18a10.3	-0.291486428	-0.180620296	-0.412941733	-0.186503605
ENSDART00000141237	si:dkeyp-113d7.10	-0.138953924	0.055420663	0.3781204	0.217616879
ENSDART00000141238	sdk1b	-0.289169472	-0.436527814	-0.423787237	-0.252263885
ENSDART00000141263	ENKD1	-0.178779434	-0.320320795	-0.370791775	-0.057231507
ENSDART00000141265	znf106b	0.649691237	0.737898777	0.38852404	0.011375685
ENSDART00000141278	si:ch211-14c7.2	0.247729936	0.220022891	0.550240269	0.375370237
ENSDART00000141328	pip5kl1	0.719167688	0.681103792	0.759572826	0.613914474
ENSDART00000141338	enc1	0.132252305	0.206249243	0.415692262	0.451357063
ENSDART00000141340	si:dkey-183n20.15	-0.434296772	-0.549401302	-1.33032419	-0.748989345
ENSDART00000141345	si:ch211-133l5.7	0.067098833	-0.266407921	-0.458017208	-0.557765253
ENSDART00000141367	si:ch211-238n5.4	-0.027130317	0.13157615	0.368504617	0.417326096
ENSDART00000141373	si:rp71-36a1.1	0.482287147	0.869957532	0.833507441	0.171228281
ENSDART00000141382	rab34b	1.020539104	1.398669915	1.148932644	0.356641036
ENSDART00000141385	si:dkey-27p18.3	0.644114707	0.569712036	1.095314507	0.257528776
ENSDART00000141387	mtus2a	-0.19505707	-0.297253066	-0.281123654	-0.028898398
ENSDART00000141397	ube2e1	0.445794988	0.509089566	0.636760169	0.02502865
ENSDART00000141401	arhgap12b	-0.092120969	-0.196587501	-0.494285673	-0.272796913
ENSDART00000141421	flna	2.437429319	2.513695725	2.398098837	2.160679529
ENSDART00000141437	sdc2	0.001568967	0.007072686	-0.196613876	-0.36025161
ENSDART00000141444	abcg4a	-0.060112999	0.269266665	0.721083006	0.688157505
ENSDART00000141446	coro1cb	-0.332235831	0.115994662	0.453423489	0.231540088
ENSDART00000141487	pvrl3b	-0.333456043	-0.273917158	-0.488003853	-0.734425178
ENSDART00000141493	ANO2 (1 of many)	0.056182997	-0.29662332	-0.7863422	-0.307277237
ENSDART00000141553	pfkfb1	-0.184104594	-0.132769648	-0.647913533	-0.249279861
ENSDART00000141569	tpt1	0.218299383	0.390841103	0.22789951	-0.0744535
ENSDART00000141578	shmt1	2.367581981	4.511004025	4.354684857	-7.25195E-16
ENSDART00000141579	fbxo10	0.546896982	0.414684656	0.492652565	0.452194062
ENSDART00000141596	gpat3	0.765600139	0.644414234	0.654748666	0.141764441
ENSDART00000141597	lrrfip1b	-0.336053735	0.307524713	0.270509711	0.2738567
ENSDART00000141606	commd8	0.496320125	0.646713153	0.422272129	0.302789387
ENSDART00000141625	si:ch211-156p11.1	0.900660828	1.143855847	0.822477469	0.791150698
ENSDART00000141626	gtf2h5	0.582335915	0.792355335	0.589249078	0.547281799
ENSDART00000141629	nrxn2a	0.086318651	-0.075949898	0.321968103	0.300507297
ENSDART00000141634	grid2	-0.260805209	-0.581973695	-0.28493372	-0.055775802
ENSDART00000141657	anxa4	0.870599992	0.703638401	0.563849009	-0.254549515
ENSDART00000141671	socs2	-0.321775893	-0.091083829	-1.001751456	-1.186845404
ENSDART00000141676	rpl12	0.410901536	0.227715625	0.271640237	0.155083239
ENSDART00000141691	opn7d	-0.282482677	-0.654185182	-0.202456799	-0.407261262
ENSDART00000141697	mthfd1a	0.286969677	0.500882111	0.266896386	-0.002369285
ENSDART00000141714	nupr1	1.321058122	1.103287011	0.703763985	0.083130869
ENSDART00000141716	inpp4b	-0.094098482	-0.269093973	-0.37484544	0.075565559

ENSDART00000141734	hivep2a	-0.218148846	-0.337091836	-0.481607769	-0.327040294
ENSDART00000141750	tnnt3b	-1.89549374	3.616832877	4.325157956	3.265344737
ENSDART00000141752	asphd1	-0.390703626	-0.359372328	-0.291984856	0.011586147
ENSDART00000141779	fndc5a	-1.597130885	-1.939288998	-0.836516829	0.004414498
ENSDART00000141791	si:ch211-284e13.6	-0.25009242	-0.144926208	0.53594874	0.219211266
ENSDART00000141831	WDR31	-0.216001124	-0.160387595	-0.391138756	-0.241605138
ENSDART00000141835	tspan18b	-0.306678314	-0.201727051	0.109445214	0.302434848
ENSDART00000141837	esd	0.782720008	0.801148933	0.794217077	0.347191174
ENSDART00000141841	psmb7	2.547438262	1.548911002	3.335199455	2.703257309
ENSDART00000141868	si:dkey-184a18.5	0.011848086	0.007989242	-0.315131621	-0.067508448
ENSDART00000141876	ftr30	0.380222613	0.277973219	1.408194744	0.300573986
ENSDART00000141877	kcnk10a	1.57307368	1.979146889	1.661354893	0.924431526
ENSDART00000141917	ank1a	-1.167872922	-1.059135645	-0.600851313	-0.041118915
ENSDART00000141920	si:ch211-224l10.4	-1.330425532	-0.617989075	-0.986788428	-0.796603249
ENSDART00000141941	zfpm2a	3.791965294	3.402723828	3.788725401	3.249505128
ENSDART00000141955	mcf2b	-0.173106859	-0.26422462	-0.398065805	0.016363494
ENSDART00000141958	cdh24a	-0.328998631	-0.596758779	-0.216164592	0.127161048
ENSDART00000141974	acsl3b	-0.154671373	0.25230778	0.484219802	0.119435773
ENSDART00000141977	herc3	-0.166490009	-0.257660784	-0.135253579	-0.013930049
ENSDART00000141981	loxhd1a	0.329259022	0.257582221	0.952686722	1.148017375
ENSDART00000142010	ncam1b	-0.299560612	-0.317330716	-0.349579067	-0.156694047
ENSDART00000142013	penka	-0.474299096	-0.531588241	-0.656476493	-0.472875583
ENSDART00000142026	si:dkey-167i21.2	-0.765729811	-0.21936881	-0.933028715	-0.2803887
ENSDART00000142061	si:dkey-32e23.4	-0.369490073	-0.293305321	0.04108125	0.132687244
ENSDART00000142087	foxp2	-0.522019738	-0.4194261	0.065656049	0.27497631
ENSDART00000142088	si:ch211-218c6.8	1.058750612	0.593589729	0.534639469	0.071525268
ENSDART00000142104	cep162	0.409390298	0.286359079	0.288581786	0.16808044
ENSDART00000142112	vma21	-0.082665202	-0.082534524	-0.180302218	-0.34518071
ENSDART00000142122	cplx2l	-1.429903189	-0.89072761	0.133226492	0.114783163
ENSDART00000142129	tacr2	-0.694450519	-0.993924228	-0.826737427	-0.21380073
ENSDART00000142140	ppfia2	-0.37587085	-0.48411577	0.218087957	0.20796301
ENSDART00000142141	grin2aa	-0.499492643	-0.789969038	-0.312539264	0.08079802
ENSDART00000142146	dmtn	0.637268571	0.800533621	0.832554824	0.713529971
ENSDART00000142155	myh14	0.116679043	0.189831513	0.30854982	0.263648695
ENSDART00000142157	arl15a	-0.037401347	-0.185890543	-0.49114445	-0.348659966
ENSDART00000142158	hars	0.313357085	0.416680865	0.429639073	0.153346223
ENSDART00000142164	ccl38.1	0.183884553	0.311556871	-0.574993202	-1.074418737
ENSDART00000142171	syt6a	-0.424476082	-0.649670345	-0.435293945	-0.206511692
ENSDART00000142196	fam167ab	-0.920556961	-0.832834787	-1.167437341	-1.267796279
ENSDART00000142223	hivep3b	-0.312228644	-0.441058379	-0.334712198	-0.18438466
ENSDART00000142239	CR450716.1	-0.604404157	-0.186424277	-0.082867047	-0.199369979
ENSDART00000142336	hipk3b	-0.202639972	-0.175487886	-0.276049793	-0.081101671
ENSDART00000142356	smyd2a	2.425657932	2.779397529	2.178998127	1.672270997
ENSDART00000142377	MEGF9 (1 of many)	-0.489828408	-0.163053003	0.463102805	0.22210349
ENSDART00000142389	cracr2ab	1.038814867	1.254276576	0.507748466	-0.396339594
ENSDART00000142397	samd12	-0.674160268	-0.821942052	-0.657095298	-0.360701575
ENSDART00000142413	BX897741.1	-0.396693848	-0.590528792	-0.411359985	-0.422415067
ENSDART00000142422	tgm2l	1.901086414	0.665689502	0.275506536	0.14002471
ENSDART00000142428	josd2	0.235661569	0.659033561	0.722639048	0.359420457
ENSDART00000142450	zgc:153219	0.356379183	0.700229042	0.242962571	0.037010709
ENSDART00000142454	si:dkey-121a11.3	-0.218109071	-0.329830559	-0.334040533	-0.225452629

ENSDART00000142462	tshz3a	-1.120926636	-3.365499709	-0.84112851	-0.654584115
ENSDART00000142467	si:ch211-199g17.2	2.796012298	2.177853709	2.464842477	1.654064057
ENSDART00000142489	slco5a1b	-0.38890353	-0.329924623	-0.120653024	-0.00920305
ENSDART00000142492	eno1b	-0.238975644	-0.29896654	-0.354383484	-0.060042567
ENSDART00000142524	chrnb2a	-0.869143617	-0.99350722	-0.040693199	0.426386933
ENSDART00000142529	si:dkey-286j15.1	0.703675816	0.544043539	0.896839213	0.54754086
ENSDART00000142543	si:dkey-206f10.1	-0.205833854	-0.070068511	-0.826534181	-0.287245824
ENSDART00000142546	cdc42l2	0.502232145	0.904679926	0.447404351	0.132174294
ENSDART00000142560	pdxkb	0.184442367	0.163391714	0.33929775	0.221566535
ENSDART00000142569	specc1lb	0.378697075	0.547073638	0.617855803	0.275260653
ENSDART00000142572	sparc	-0.204187388	-0.23572234	-0.326346891	-0.277892827
ENSDART00000142584	alx1	1.361674144	1.279023188	1.723053191	-0.115335088
ENSDART00000142605	sorbs2b	-0.090898219	-0.353328393	-0.576838002	-0.278787769
ENSDART00000142634	FP067425.1	1.971749592	2.170812908	1.958026148	1.009935297
ENSDART00000142648	gramd1a	-0.23770878	-0.291013375	-0.237165881	-0.156534014
ENSDART00000142653	grm1a	-0.469462455	-0.467735907	-0.088400916	0.178280203
ENSDART00000142654	BX324123.2	-0.377689742	-0.458973085	-0.444383379	-0.275709525
ENSDART00000142661	osbpl2a	-0.307460163	-0.305018775	-0.245046053	-0.17275397
ENSDART00000142665	kctd15a	-0.244770563	-0.321970355	-0.179500858	-0.083583768
ENSDART00000142678	slc10a1	-0.780280747	-0.374232851	-0.51558614	-0.22594401
ENSDART00000142687	cnn2	0.635956839	-0.518048624	0.612837411	-3.662416494
ENSDART00000142691	trim35-10	1.464216907	1.327065997	0.977518865	0.007627538
ENSDART00000142692	eef1b2	0.349947783	0.50162657	0.39446346	0.093426844
ENSDART00000142726	creg2	-0.073456763	-0.046645374	-0.204358474	-0.271881041
ENSDART00000142731	rce1b	-0.103631801	-0.326325127	-0.391135117	-0.061782364
ENSDART00000142735	si:ch211-13f8.1	-0.324319655	-0.241821619	-0.588481479	-0.554389586
ENSDART00000142748	UNC13A	-0.222825276	-0.520698317	-0.335972829	0.134560543
ENSDART00000142772	eif4a1a	0.755438851	0.594665203	0.351260825	0.661749697
ENSDART00000142778	BX569784.1	-0.298918438	-0.277434239	-0.189295135	-0.216741666
ENSDART00000142806	stxbp5a	-0.250986358	-0.382479123	-0.333968332	-0.143471828
ENSDART00000142810	gabra3	-0.405359169	-0.863749916	-0.692169745	-0.647424845
ENSDART00000142815	fdps	0.434008031	0.982096797	0.955228312	0.608495312
ENSDART00000142837	enpp5	-0.408425134	-0.376571863	-0.363824939	-0.214479077
ENSDART00000142851	itih6	-0.217828278	-0.246312318	-4.361209214	-4.055941297
ENSDART00000142873	BX901918.1	0.845880305	0.530715844	1.008998061	0.591786557
ENSDART00000142880	BX294379.1	0.237830465	0.561801634	0.415821035	0.460224197
ENSDART00000142884	gngt2a	-0.004065507	-0.183032589	-0.673226204	-0.483998962
ENSDART00000142919	ssh1b	0.196097873	0.173857746	0.465900153	0.503515831
ENSDART00000142920	zgc:162150	1.709110448	2.426589338	1.780018708	1.501264432
ENSDART00000142922	slc17a5	0.2420628	0.323011659	0.210848621	0.174231112
ENSDART00000142975	si:ch1073-70f20.1	-0.443514404	-0.337920446	-0.430300513	-0.297428401
ENSDART00000142976	kcnip3a	-0.515340349	-0.554488278	-0.50826115	-0.165828333
ENSDART00000143000	rnf220b	-0.004804151	-0.254916502	-0.367501204	-0.153255414
ENSDART00000143048	lmo7b	-0.147694355	-0.241574624	-0.520231272	-0.160787429
ENSDART00000143056	zyx	0.416244119	0.475600395	0.593066786	0.15882784
ENSDART00000143066	ube2e1	0.330371306	0.357877825	0.149635317	0.181174607
ENSDART00000143083	CT573139.1	-0.081020454	-0.307026797	-0.341094511	-0.086434359
ENSDART00000143120	si:dkey-147f3.4	1.419591527	3.82528139	2.470866243	3.546981594
ENSDART00000143152	cpne5a	-0.664046359	-0.825602355	-0.316663455	0.050239809
ENSDART00000143165	tsc22d1	-0.098364585	-0.21923408	-0.39849137	0.017570634
ENSDART00000143199	wasf3a	-0.216537889	-0.280788628	-0.398676762	-0.269991874

ENSDART00000143200	kcnma1a	-0.226894558	-0.334282966	-0.480968248	-0.397036214
ENSDART00000143203	parp2	0.638789429	0.581199479	0.559257592	0.245299499
ENSDART00000143208	etnk1	0.29866149	0.19770258	0.103751026	0.382131244
ENSDART00000143245	nptnb	-0.0813348	-0.106325128	-0.477835951	-0.112902967
ENSDART00000143286	lnx1	-0.74422542	-0.772813048	-0.069056217	0.13158415
ENSDART00000143291	RPL41	0.451636798	0.464258672	0.193261311	0.034762109
ENSDART00000143376	rab3b	-0.460716785	-0.436622412	0.05069835	0.173014647
ENSDART00000143382	il1rapl2	-0.355933029	-0.71045348	-0.38678508	-0.068375826
ENSDART00000143410	EMB	-1.826210099	-1.592080235	-0.486327336	0.178400158
ENSDART00000143413	si:ch211-170d8.2	2.110280665	1.653225126	1.38414074	0.820720018
ENSDART00000143434	ubash3ba	-0.070569577	0.008399701	0.263717646	0.11493286
ENSDART00000143457	cndp2	0.393197537	0.458029638	0.473219597	0.244423454
ENSDART00000143503	usp21	-0.183997919	-0.332937745	-0.642449016	-0.356635014
ENSDART00000143519	impdh1a	-0.362024165	-0.511295198	-0.258726495	-0.069176235
ENSDART00000143554	ampd2b	-0.328956412	-0.382404642	-0.421715131	-0.209511328
ENSDART00000143562	gbp2	0.680404258	0.927169543	1.353668195	0.527828388
ENSDART00000143573	ncor1	0.450093687	0.292142618	0.666194454	0.343803309
ENSDART00000143582	si:ch211-236p22.1	-0.593497691	-1.183006832	-0.39659144	-0.180756629
ENSDART00000143587	ern2	0.504450294	0.214096186	0.104332211	0.050090305
ENSDART00000143618	birc7	0.867487648	0.469428911	0.548081783	0.007263478
ENSDART00000143625	map7d2b	0.025364857	-1.606530561	-4.429840485	-0.88152256
ENSDART00000143662	ankrd33aa	-0.118189532	-0.449119978	-0.599045615	0.004982045
ENSDART00000143697	si:dkeyp-69e1.8	0.277815064	0.526114648	0.670410394	0.498924627
ENSDART00000143703	flrt3	-0.315208699	-0.338016477	-0.216002726	-0.160149445
ENSDART00000143723	idi1	0.080932725	0.304370095	0.372374682	0.127047939
ENSDART00000143741	fkbp5	1.764298053	1.865156046	1.631976013	1.871443577
ENSDART00000143743	si:ch211-14a17.10	0.56355315	0.97468243	0.596767311	0.343957389
ENSDART00000143761	cadm4	-0.396807548	-0.426573725	-0.041424036	-0.105152637
ENSDART00000143766	tbc1d4	0.551251852	0.35059591	0.424872318	0.224396247
ENSDART00000143784	doc2d	-0.282456786	-0.449882775	-0.341326778	0.036564808
ENSDART00000143787	tnr	-0.299938525	-0.646994379	-0.19048806	-0.113023917
ENSDART00000143793	C3 (1 of many)	3.237329668	4.023510735	3.503827488	2.024562274
ENSDART00000143819	VASH1	0.033504925	0.324442057	0.595830734	0.534870749
ENSDART00000143829	nxnl1	-0.184554354	-0.325799787	-0.486032926	-0.185522737
ENSDART00000143840	gstk1	0.057517576	-0.140839441	-0.201267582	-0.834065155
ENSDART00000143846	si:ch211-198c19.3	0.524345776	0.185185856	-0.297806816	-0.062260629
ENSDART00000143860	si:ch211-154e10.1	-0.109941804	-0.36125539	-0.451271764	-0.06288628
ENSDART00000143867	syngap1b	-0.339959547	-0.514759454	-0.488418852	-0.182310837
ENSDART00000143874	akna	-0.124615674	-0.323808831	-0.482322372	-0.23639171
ENSDART00000143875	BX323074.2	0.32505808	-0.029313176	-0.099305279	-0.178101813
ENSDART00000143878	arhgef9a	-0.465269949	-0.707003765	-0.492066921	-0.306386806
ENSDART00000143887	taar12g	2.133495532	1.949189055	1.339108526	1.854618081
ENSDART00000143909	tbc1d9	-0.237612035	-0.281483225	-0.04707848	0.049665559
ENSDART00000143911	si:dkey-105e17.1	-0.529795865	-0.492017338	-0.300236892	-0.145540976
ENSDART00000143919	gabbr1b	-0.121288245	-0.415278076	-0.279488234	-0.175446101
ENSDART00000143928	rwdd1	0.331616231	0.265135658	0.236169655	0.064492205
ENSDART00000143938	sema3ga	0.034230149	-0.448431565	0.102011548	0.447499442
ENSDART00000143963	pcloa	-0.078913048	-0.544095469	-0.414092766	-0.012445983
ENSDART00000143969	BX247868.1	-1.179119979	-1.430780681	-0.233116942	0.342359274
ENSDART00000143999	tnnt3b	0.546685869	4.375596584	5.084902064	3.992962258
ENSDART00000144046	rapgef4	-0.382423404	-0.346599548	-0.357063132	-0.146328348

ENSDART00000144048	cecr1b	1.174703015	1.015529557	0.446419815	-0.23634795
ENSDART00000144050		-0.572877113	-0.701246998	-0.196460214	-0.040487474
ENSDART00000144053	si:dkey-21e2.10	0.342164757	1.862494203	1.068542572	-0.153388265
ENSDART00000144067	cabp5a	-0.477283062	-0.255392436	-0.467866686	-0.403996112
ENSDART00000144068	zgc:101851	0.417625916	0.309307199	0.017931645	-0.07898028
ENSDART00000144139	elavl4	1.756586356	1.578077782	0.938796608	0.66956976
ENSDART00000144175	ccdc136b	-0.408583735	-0.440451575	-0.345784282	-0.125541648
ENSDART00000144186	pou6f2	-0.474463335	-0.465571046	0.092941552	0.436081081
ENSDART00000144215	pcnxl2	-0.697249288	-0.448197444	0.040412938	0.382373048
ENSDART00000144216	gal3st1b	-0.491885196	-0.717325904	-0.58217274	-0.190076988
ENSDART00000144237	gig2p	0.374493634	0.174814468	1.411754829	-0.127638364
ENSDART00000144238	cry1bb	-2.336873942	0.347073563	-0.04859154	-0.311120285
ENSDART00000144246	tbxas1	-0.011108915	0.001179893	-0.320953776	-0.371393575
ENSDART00000144255	magixb	-0.339150091	-0.441125122	-0.366876369	-0.172380242
ENSDART00000144261	fryl	-0.221060382	-0.265751165	-0.423351874	-0.131445521
ENSDART00000144285	si:ch211-196g2.4	-0.287314781	-0.275198401	-0.33300774	-0.198378078
ENSDART00000144297	ubac2	-0.266999312	-0.798888734	-0.707434326	-0.564642121
ENSDART00000144303	AK6	0.166979081	-0.045967241	-0.30997345	-0.290616599
ENSDART00000144308	si:dkey-11o15.5	1.026391135	2.84679961	3.809819934	2.718686222
ENSDART00000144335	cyp27c1	1.539730469	0.927858614	0.30747539	-0.356140532
ENSDART00000144342	ppp1r14ba	0.017178827	0.781919568	1.244415225	1.189050173
ENSDART00000144346	cdkn1bb	0.579581082	0.718938042	0.058805886	-0.088669944
ENSDART00000144351	malt1	-0.740504685	-0.746576787	-0.600575844	-0.384172626
ENSDART00000144353	calcrla	-0.265245102	-0.29868229	-0.249979568	-0.195320595
ENSDART00000144361	ank1b	-0.731673078	-0.825040806	0.086374477	0.310723345
ENSDART00000144448	pcbp3	-0.386917442	-0.448839957	-0.257435258	-0.084182982
ENSDART00000144469	adora2b	-0.015759069	-0.127966141	-0.39792302	-0.033165164
ENSDART00000144500	si:ch211-235f1.3	2.218077738	0.39200338	2.195588647	1.510701941
ENSDART00000144503	ran	-0.075551405	0.448221073	0.249018928	0.118064827
ENSDART00000144516	lonrf1l	0.003516441	0.629397595	-0.101368349	0.117101343
ENSDART00000144544	col1a2	0.443294043	1.698639717	2.716305218	1.282115721
ENSDART00000144545	rc3h1a	0.1127689	0.086123881	0.323320533	0.192355814
ENSDART00000144555	si:dkey-84o3.3	0.84569976	1.063530072	1.082186309	0.816110648
ENSDART00000144592	sik2a	-0.05071398	-0.135507163	-0.424760133	0.101311014
ENSDART00000144600	bin1a	-0.647409952	-0.602565457	-0.5226216	-0.498459302
ENSDART00000144608	plcl1	-0.399605613	-0.241387332	-0.124693789	0.02448912
ENSDART00000144647	bcat1	-0.053055163	-0.144301718	-0.580079153	-0.061442763
ENSDART00000144681	anxa13l	0.688342481	1.628944564	2.065542903	1.40538163
ENSDART00000144702	clint1a	0.287820278	0.14751069	0.132691317	-0.0310018
ENSDART00000144711	tmem154	0.48356725	0.317274745	0.352966647	0.032904309
ENSDART00000144714	HTRA2	0.502271196	0.745024882	0.567017564	0.566812931
ENSDART00000144737	srsf3b	0.361810279	0.317106803	0.304841622	0.541582711
ENSDART00000144750	tnni2b.1	0.190106792	2.993607369	3.517771751	2.049682842
ENSDART00000144766	nars	0.166803353	0.38562143	0.598983821	0.308867409
ENSDART00000144767	gabra1	-0.494710008	-0.525246082	-0.558533509	-0.303902197
ENSDART00000144770	cast	1.895732545	2.316221594	1.433447785	1.629878019
ENSDART00000144802	dfnb31b	-0.206535765	-0.346317212	-0.484783097	-0.018383317
ENSDART00000144804	mxra7	-0.254910086	-0.321688262	-0.500299705	-0.323281006
ENSDART00000144813	march8	-0.52367029	-0.417394306	-0.174509938	-0.172830735
ENSDART00000144814	si:dkey-31f5.8	0.429768416	1.027387879	0.82502917	0.369456203
ENSDART00000144817	cfl1	0.341949415	0.252829572	0.49073898	0.258667203

ENSDART00000144841	si:dkey-242g16.2	0.827348141	1.743182823	1.970186443	1.411913803
ENSDART00000144842	dctn2	-0.498329292	-3.353631529	0.161761115	0.105336326
ENSDART00000144862	ptpmt1	3.64529742	2.311630713	2.226765633	3.455463537
ENSDART00000144886	dicp1.1	1.748045166	0.589930553	0.172712572	0.178536852
ENSDART00000144890	bokb	-0.312629523	-0.278040732	-0.730243904	-0.348692502
ENSDART00000144891	gpnmb	2.526942309	2.443053131	2.053909603	-0.779431544
ENSDART00000144894	bcl11ba	-0.656989822	-0.679320488	0.181338486	0.623847694
ENSDART00000144925	snx27a	-0.201299797	-0.367903189	-0.251100705	-0.13350684
ENSDART00000144946	adcyap1b	2.385755846	2.421463563	2.507590258	1.873754889
ENSDART00000144966	nt5c2l1	0.621464058	1.214449784	1.836289861	0.635815132
ENSDART00000144970	meis2b	-0.299148131	0.020280124	0.107132127	0.297996513
ENSDART00000144986	otud5a	0.068487051	0.299156316	0.307343363	0.221359831
ENSDART00000144993	tbc1d14	0.120746825	0.349052884	0.289914122	0.097064778
ENSDART00000144995	MFSD3	-0.419691404	-0.388696945	-0.281399212	-0.247251894
ENSDART00000145018	gprc5bb	-0.280944825	-0.565589689	-0.320436299	-0.038626462
ENSDART00000145019	akr1a1b	0.370317087	0.266617428	0.13791003	-0.009346531
ENSDART00000145022	CU459186.8	2.820516441	4.510118733	2.721368195	2.385722915
ENSDART00000145035	saga	-0.094875139	-0.251791894	-0.561974573	-0.363496236
ENSDART00000145055	pnrc2	-0.148748622	-0.240065036	-0.476375745	-0.443408468
ENSDART00000145065		4.897906418	2.757476262	4.60015098	5.019792283
ENSDART00000145095	celsr3	0.648145926	0.648607509	0.829081729	0.619308918
ENSDART00000145096	fam219ab	-0.366547695	-0.337752539	-0.191344563	-0.015845187
ENSDART00000145103	cntfr	0.307964555	0.391102809	0.626651223	0.264745691
ENSDART00000145107	agfg1b	-0.053414539	0.037458177	0.272453618	0.201504327
ENSDART00000145108	MFF (1 of many)	-0.387524941	-0.341112286	-0.251149344	-0.013627184
ENSDART00000145111	parp4	1.113854381	0.990369473	0.690636927	0.186507426
ENSDART00000145114	ptprsa	-0.133739633	-0.353332073	-0.057919239	0.041724305
ENSDART00000145124	dact3a	0.112856383	0.770301052	1.625107526	0.355230326
ENSDART00000145170	smox	0.719497557	0.099508547	-0.032495938	0.130866421
ENSDART00000145184	srrd	-3.467209407	0.181528862	-0.326150144	-0.189288253
ENSDART00000145198	steap4	0.551058744	0.178525002	-0.419104103	-0.970957834
ENSDART00000145210	ankle2	0.203906028	0.433259544	0.357710257	0.326696668
ENSDART00000145215	grin2ca	-0.197290629	-0.356995819	-0.467710351	-0.053225483
ENSDART00000145220	arvcfa	-0.706915106	-0.825791875	-0.448746575	-0.046016635
ENSDART00000145226	si:ch211-233h19.2	-0.372287835	-0.175787817	-0.260726103	-0.131614613
ENSDART00000145230	eef1db	0.569560585	0.627404265	1.018116547	0.789247315
ENSDART00000145258	glsb	-0.010345949	-0.147193332	-0.750348982	-0.227873062
ENSDART00000145269	ostm1	0.549197851	0.4592474	0.036533403	-0.163457465
ENSDART00000145275	si:dkey-147f3.8	1.443349889	1.631277345	1.233548809	-0.107779207
ENSDART00000145282	sepp1a	-0.462981641	-0.396376841	-0.662591735	-0.92136404
ENSDART00000145294	FEZ2 (1 of many)	0.025283475	-0.037737708	-0.286085795	-0.169439894
ENSDART00000145309	exoc3	2.170059758	1.622886137	1.185093243	1.97278521
ENSDART00000145330	apoc1	1.228570608	0.722853476	0.218571174	0.363892889
ENSDART00000145331	zgc:92066	3.539715592	2.15686966	1.135379743	1.013519624
ENSDART00000145342	cbln1	-0.285689309	-0.291683977	-0.350650667	-0.126158592
ENSDART00000145349	bcat1	-0.398195711	-0.070627262	-0.574724119	-0.164567732
ENSDART00000145364	sybu	-0.525383816	-0.345930455	-0.451355227	-0.158271762
ENSDART00000145377	rpl38	0.331388676	0.460944412	0.086514771	-0.0487643
ENSDART00000145379	fblim1	0.305913455	0.545649791	0.383397127	0.135782412
ENSDART00000145387	shank2	-0.271198688	-0.514736215	-0.289482704	-0.011228429
ENSDART00000145413	si:ch211-253p2.2	-0.612329709	-0.583952587	-0.545467055	-0.273784221

ENSDART00000145428	si:ch211-51e12.7	-0.292178538	-0.197575677	-0.034168177	0.070057863
ENSDART00000145434	gpr52	-0.413465096	-0.575440094	-0.276023467	-0.017738845
ENSDART00000145436	atp2a2a	-0.880456774	-0.552067998	-0.067905189	-0.332284195
ENSDART00000145449	rpl10	0.64701527	0.814914535	0.479616309	0.351438297
ENSDART00000145486	si:ch211-243a20.3	1.596806687	1.581489321	1.736665736	-0.106318155
ENSDART00000145489	ano11	-1.065264341	-1.355279668	-0.34443399	0.075205433
ENSDART00000145494	kcnq3	-0.150620266	0.575528836	1.458464727	1.126016522
ENSDART00000145545	dclk1a	-0.21717792	-0.314020567	-0.407893866	-0.042242501
ENSDART00000145558	si:ch211-237l4.6	-0.183280044	-0.231325216	-0.614649778	-0.510830341
ENSDART00000145562	robo4	1.739515239	1.001047952	0.857159879	-0.094789579
ENSDART00000145605	camk2d1	0.045519482	0.325117172	0.151136219	0.629830606
ENSDART00000145615	cacna1i	-0.38207214	-0.804209146	-0.404812647	-0.085276411
ENSDART00000145616	adarb1b	-0.549552658	-0.593167659	-0.056719481	0.082749855
ENSDART00000145668	si:ch211-236p5.2	-0.579324415	-0.253977811	-0.665119908	-0.600847942
ENSDART00000145673	si:ch211-126i22.5	0.167835794	0.336202454	0.573573931	0.33326684
ENSDART00000145681	atp6v1h	0.62737841	0.200274483	0.109012982	-0.483227692
ENSDART00000145691	PTP4A3 (1 of many)	0.061273139	-0.037655485	-0.389148491	-0.598282045
ENSDART00000145705	ablim1a	-0.310864127	0.043751002	0.292255753	0.332622511
ENSDART00000145728	sdhc	-0.035025769	-0.113602993	-0.325388824	-0.192371035
ENSDART00000145743	si:dkey-222h21.3	2.082143417	2.044714298	1.752169227	1.309410865
ENSDART00000145762	dnaaf1	-0.407466904	-0.372899622	-0.590562671	-0.565268948
ENSDART00000145775	mpp4a	-0.462462268	-0.206618846	-0.102159961	0.056169031
ENSDART00000145777	adgrb3	-0.131394962	-0.455026919	-0.13584478	0.067074927
ENSDART00000145778	eif4g1a	0.287822409	0.058653961	0.329719916	0.260115281
ENSDART00000145782	ccl34a.3	1.066630706	1.262530626	1.391273396	1.026476376
ENSDART00000145789	fbxo41	-0.304859	-0.365689262	-0.001299255	0.043794304
ENSDART00000145809	prkag3a	-4.153185554	-1.353616268	-0.923701192	-0.043754788
ENSDART00000145834	si:ch211-233a24.2	-0.054480296	-0.065552868	-0.373189	-0.049333125
ENSDART00000145835	tmx3	-0.913440216	0.029499449	0.308787653	0.34181057
ENSDART00000145852	arpc5b	3.848588728	2.885756027	4.201851599	3.883565062
ENSDART00000145859	slc25a23b	-0.271106084	-0.480607261	-0.132697812	0.164389769
ENSDART00000145862	gramd1ba	-0.165891466	-0.265579133	-0.22242289	-0.116668883
ENSDART00000145875	ANKRD34C	-0.466770544	-0.643453831	-0.660279878	0.01171991
ENSDART00000145886	osbpl3b	0.13475307	0.290875362	0.450588606	0.199350453
ENSDART00000145894	zgc:195245	-0.325743953	-0.471221888	-0.661613866	-0.246097731
ENSDART00000145907	gc3	-0.385628346	-0.300281403	-0.1220973	0.029888955
ENSDART00000145916	lingo4a	-0.858684072	-0.829176543	-0.734684966	-0.796722528
ENSDART00000145929	slc24a2	-0.262244646	-0.388750168	-0.249062022	0.013032324
ENSDART00000145933	si:rp71-68n21.9	0.834432396	0.464946482	0.375058394	-0.112544064
ENSDART00000145956	ptpn23a	0.252886396	0.192353563	0.35477406	0.187678789
ENSDART00000145959	sik2a	-0.340525942	-0.474784074	-0.456194757	0.011390214
ENSDART00000145970	si:dkey-83k24.5	0.413467032	0.037399564	-0.039197609	-0.146263052
ENSDART00000145979	col19a1	-0.158274269	-0.337487778	-0.291137211	-0.179712512
ENSDART00000145981	mmachc	-0.180799553	-0.336507302	-0.603391198	-0.368222803
ENSDART00000145983	cdkn1a	1.243824395	0.764194636	0.498342294	0.064303597
ENSDART00000145997	uap1	1.529889024	1.202439925	1.069222792	0.723659511
ENSDART00000145999	prx	1.586883428	3.204392125	2.633047079	1.399238803
ENSDART00000146005	gtpbp1	0.461505187	0.53679144	0.551879705	0.330029037
ENSDART00000146007	usf2	-0.027075797	-0.165338495	-0.358464486	-0.103064043
ENSDART00000146008	camkvl	-0.251523364	-0.299024254	-0.462517021	-0.14005057
ENSDART00000146055	ptchd4	-0.534340079	-0.556417786	0.177414073	0.199962898

ENSDART00000146070	arvcfb	-0.070894502	-0.208057535	-0.298756488	-0.110396791
ENSDART00000146101	mcoln1a	0.490011182	0.372261102	0.240356077	0.080050098
ENSDART00000146106	nfil3-5	0.032256315	1.067374086	1.300867348	1.052274713
ENSDART00000146107	ywhae1	-0.007595908	0.375656099	0.665999115	0.632685234
ENSDART00000146113	arhgef9b	-0.6095368	-0.587211831	-0.441711189	-0.048741645
ENSDART00000146114	si:ch211-139g16.8	1.21401658	0.728240858	0.247635929	-0.180282803
ENSDART00000146120	luzp2	-0.491780393	-0.439244253	0.071649151	0.105029542
ENSDART00000146132	si:ch211-254n4.3	-0.407187074	-0.522964952	-0.215771966	0.042955183
ENSDART00000146180	csrnp2	0.237147162	0.374829692	0.697707124	0.402573932
ENSDART00000146210	si:ch211-214p13.9	1.80985471	2.473449699	1.675655513	-0.45037085
ENSDART00000146222	BX548073.2	0.396874686	0.19924291	0.197044037	0.232842702
ENSDART00000146227	slc24a3	-0.627503774	-0.581196675	-0.447342274	-0.279205796
ENSDART00000146247	zc2hc1a	0.181680565	0.536623189	0.750568991	0.668481158
ENSDART00000146284	kcnh3	-0.880077839	-1.089282019	-0.771605014	-0.433474862
ENSDART00000146315	stx5al	-0.219102203	-0.145337646	-0.303930137	-0.044513676
ENSDART00000146321	fibcd1	-0.340172452	-0.594838596	-0.369380512	-0.071609744
ENSDART00000146327	sgk1	-0.407553517	-0.497547741	-0.442772363	-0.391494952
ENSDART00000146348	shisa7a	-0.257432452	-0.343813852	-0.064533631	0.047373088
ENSDART00000146371	olfm1b	-0.300182439	-0.072903502	0.253479105	0.553473031
ENSDART00000146373	nasp	3.16018099	3.265920313	2.837245417	1.580807285
ENSDART00000146380	map6d1	-0.622612519	-0.482084178	-0.312516702	-0.212427733
ENSDART00000146394	macf1a	0.292220827	0.266325023	0.282692477	0.251543817
ENSDART00000146400	st8sia5	0.352731191	0.917371468	1.067350167	0.852063315
ENSDART00000146411	sst6	0.736642137	1.700467827	1.549366173	1.261691667
ENSDART00000146422	samd12	-0.651610173	-1.082672704	-0.694390946	-0.535477884
ENSDART00000146424	bin1a	-0.478145779	-0.444599263	-0.2729609	-0.137965745
ENSDART00000146434	zgc:153115	0.499570303	0.817313835	0.902072926	0.441195032
ENSDART00000146455	si:dkey-245p14.7	1.534786293	2.054169347	1.459641722	1.163785428
ENSDART00000146463	samd11	-0.429238253	0.041148803	0.036400645	0.142792336
ENSDART00000146472	kcng4a	-0.174962319	-0.077658154	-0.410630934	-0.442662471
ENSDART00000146508	ldlrad4b	-0.645866164	-0.626215777	-0.091198282	-0.040482632
ENSDART00000146517	dpysl2b	0.345677828	1.022911519	1.287775094	0.946528154
ENSDART00000146525	chtopb	3.459860751	4.15390826	1.273403713	1.821621278
ENSDART00000146530	epha6	-0.320688922	-0.528894361	-0.185025461	0.089827402
ENSDART00000146553	pfkpb	-0.397983333	-0.578737682	-0.358519605	-0.195354249
ENSDART00000146563	magixa	0.075133988	0.355594726	0.57567448	0.363486006
ENSDART00000146564	si:ch211-198c19.3	1.407263699	0.462763747	-0.033079511	-0.600140762
ENSDART00000146567	cenpk	2.135920979	1.866039444	1.584297235	1.144121982
ENSDART00000146611	cxcr3.3	0.415816929	0.611232638	0.663522207	-0.008061564
ENSDART00000146632	rassf2a	0.029735353	-0.579515238	-0.449210112	-0.253532753
ENSDART00000146641	adgrb2	-0.339356043	-0.358957699	-0.00479776	0.052341655
ENSDART00000146648	cd99	0.568325087	1.118199036	0.745908705	-0.085222667
ENSDART00000146671	lgals3a	0.211860458	0.266423347	0.163466771	0.057807983
ENSDART00000146704	pax6b	-0.61310857	-0.673866221	-0.260691596	-0.192950711
ENSDART00000146708	adar	-0.042581428	-0.004444765	0.40535861	0.024953366
ENSDART00000146712	ccdc85a	-0.396306457	-0.805958759	-0.536293039	-0.356615708
ENSDART00000146715	st6galnac3	-0.320119064	-0.207968819	0.143950361	0.08219826
ENSDART00000146742	ewsr1b	0.415208463	0.221707988	0.300205339	0.348462361
ENSDART00000146744	nfkbiab	0.787570548	0.783257317	0.468593447	0.633863732
ENSDART00000146767	fosl1a	0.841007094	1.285901651	0.733422298	0.173089455
ENSDART00000146775	kank3	2.938783957	2.713493484	2.4846528	0.885288567

ENSDART00000146776	tspan2b	0.412627689	1.44398444	1.603529521	0.88938646
ENSDART00000146785	tmem163b	-0.68540579	-0.770110571	-0.115396875	0.356833449
ENSDART00000146808	si:ch73-184c24.1	1.14465672	1.950542793	2.864785673	1.868117236
ENSDART00000146872	emid1	0.014325328	0.393704295	0.644359098	0.692252594
ENSDART00000146873	tmem183a	2.267285842	2.498042527	2.600977396	2.807645399
ENSDART00000146892	abcg2d	0.141142908	-0.029073312	-0.499348256	-1.743973024
ENSDART00000146914	mpp6a	-0.247758591	-0.21742389	-0.181144549	-0.11922972
ENSDART00000146930	pdk3a	-0.237508559	-0.287205966	-0.210198002	-0.212117205
ENSDART00000146956	fibpb	0.004335049	-0.126807206	-0.29094593	-0.318585256
ENSDART00000146962	STX3 (1 of many)	-0.081644241	-0.246049482	-0.328837917	-0.149895857
ENSDART00000146991	si:ch211-127d4.3	-0.09259617	-0.379853865	-0.53001099	-0.593912213
ENSDART00000147001	si:dkeyp-72h1.1	-0.667988087	-0.713655804	-0.172938308	0.232582151
ENSDART00000147004	oxsr1b	-0.068072204	-0.071587779	-0.30197654	-0.095099951
ENSDART00000147009	si:dkey-251i10.2	2.257544115	2.36261429	1.933731915	0.997576718
ENSDART00000147019	si:ch211-235o23.1	0.394717538	0.700152429	0.448882118	0.273599764
ENSDART00000147068	olfml2ba	-0.192176717	0.036009408	-0.274084705	-0.587185898
ENSDART00000147105	tdh	-0.645059373	-0.498416454	-0.202501341	-0.068176647
ENSDART00000147110	tubb6	1.236908798	0.772404142	0.732906531	-0.307326846
ENSDART00000147137	si:dkey-161j23.6	-1.245759967	-0.352718506	0.050703213	0.033267232
ENSDART00000147175	si:dkeyp-69c1.7	-0.274009215	-0.118199859	0.157998072	0.359473526
ENSDART00000147188	trpm3	-0.625119804	-0.737554924	-0.108574358	0.285413653
ENSDART00000147201	slc5a7a	-0.334790717	-0.440092664	-0.539424926	-0.607892581
ENSDART00000147217	atrnl1a	-0.191475884	-0.379227232	-0.255587915	-0.309192132
ENSDART00000147218	efna3a	-0.679676128	-0.850928012	-0.177104508	-0.025688419
ENSDART00000147246	MPP4 (1 of many)	-0.296462508	-0.373112158	-0.170989023	-0.099561343
ENSDART00000147264	fam213b	0.418860252	0.552271711	0.440362956	0.212029386
ENSDART00000147265	rpl8	1.326651746	0.800643986	0.962132043	0.631592879
ENSDART00000147275	plat	0.870075522	0.84761039	0.802236758	0.134643658
ENSDART00000147285	tomm70a	-0.111704054	-0.295896007	-0.277787909	-0.179585993
ENSDART00000147308	amigo1	-0.366406631	-0.580638611	-0.462226604	-0.20619589
ENSDART00000147318	zgc:123105	-0.365732482	-0.633059491	-0.048801121	0.05490191
ENSDART00000147341	tubb4b	0.566193096	0.992023395	-0.136369873	0.130190834
ENSDART00000147343	dnase2	0.744692712	0.455061161	0.199303414	-0.152365099
ENSDART00000147368	osbp2	-0.137018463	-0.22987814	-0.404444797	-0.125629393
ENSDART00000147377	si:ch211-218c6.8	0.580687503	0.496233306	0.169999239	-0.049284637
ENSDART00000147394	rcvrna	-0.601511252	-0.165338563	-0.351313293	-0.435988789
ENSDART00000147422	arhgap23b	-0.106264079	-0.33507693	-0.670558797	-0.201207875
ENSDART00000147439	atp6v0b	-0.143289001	-0.224190834	-0.266752197	-0.126455259
ENSDART00000147461	pih1d1	2.042300587	3.359045894	1.936071133	3.025420547
ENSDART00000147464	ryr3	-0.823352364	-0.716491782	0.05941278	0.608108075
ENSDART00000147473	pcsk1	-0.408472737	-0.345735504	-0.351470668	-0.372482226
ENSDART00000147474	stmn2b	0.410261377	1.543598694	2.044768785	1.667647819
ENSDART00000147481	phf11	2.519105146	2.056646778	4.198159431	2.169035406
ENSDART00000147483	ephb6	-0.346946617	-0.195722816	0.204471928	0.396713027
ENSDART00000147496	wsb1	0.50240599	0.657726247	0.306985059	0.144649209
ENSDART00000147502	grhprb	0.278026338	-0.217638113	-0.512645749	-0.092734666
ENSDART00000147504	bzw2	0.031120244	-0.513458671	-0.643056049	-0.325765553
ENSDART00000147516	si:ch73-343l4.8	0.629933021	0.124466643	-0.147547456	-2.286690979
ENSDART00000147518	fam89b	0.288965136	0.576511697	0.598868415	0.448283091
ENSDART00000147527	si:dkey-9i23.14	2.675666068	2.038792867	2.055161538	1.544748993
ENSDART00000147581	celf5b	-0.452125975	-0.583204528	-0.153939994	-0.143297487

ENSDART00000147582	agmo	-0.172065266	-0.060434285	-0.409289881	-0.408628944
ENSDART00000147609	shc1	-0.131375988	0.113051171	0.56388659	0.681815315
ENSDART00000147617	bcl2l1	0.692333613	0.690280735	0.392732217	0.224132994
ENSDART00000147625	pik3r3b	-0.257748291	0.021181096	0.371087748	0.412468691
ENSDART00000147635	gpd1c	0.454472166	0.231690448	0.167995006	-0.010446218
ENSDART00000147658	bhlhe22	-0.302235414	-0.31899187	-0.305987775	-0.102192149
ENSDART00000147682	atp1a1a.2	1.135451283	0.290531702	0.256841542	-0.140895848
ENSDART00000147683	inpp4b	-0.101056224	-0.522680445	-0.598179821	-0.221205188
ENSDART00000147686	si:dkey-222p3.1	-0.111715584	-0.482884964	-0.578818209	-0.433340731
ENSDART00000147694	tm4sf4	-0.310160873	0.021226995	-0.077203568	-0.031986481
ENSDART00000147699	mical3b	0.112525626	0.108456465	0.226887131	0.765699718
ENSDART00000147729	rps24	0.371025561	0.416364277	0.295416172	0.004577514
ENSDART00000147731	BX927329.1	0.853591712	1.100390597	0.929772221	0.285035676
ENSDART00000147742	fgfr1a	-0.118664773	-0.073758578	-0.167833486	-0.603740653
ENSDART00000147779	sh3glb2b	-0.245646809	-0.296043128	-0.126401752	-0.042735049
ENSDART00000147787	znf512	1.743634906	1.038845919	2.753652323	1.346161863
ENSDART00000147789	slco2a1	-0.167945299	-0.15402624	-0.306762825	-0.440433916
ENSDART00000147793	gcdha	-0.253694486	-0.123690535	-0.220775945	-0.080919569
ENSDART00000147794	nt5c2l1	2.1295515	2.541950381	3.263642166	1.818207515
ENSDART00000147799	ndrg3b	-0.751369578	-0.184479497	0.170135881	-0.060187322
ENSDART00000147820	carkd	-0.239894355	-0.307342094	-0.0807657	-0.048288884
ENSDART00000147821	spsb4a	0.371275002	0.33311003	0.599280348	0.314031744
ENSDART00000147826	jade3	0.261388203	0.282176502	0.335957333	0.193451186
ENSDART00000147831	ACAP2 (1 of many)	-0.265239588	-0.086744823	0.171263201	0.342225898
ENSDART00000147849	klf6a	1.130982604	1.870693821	1.403514717	0.445060502
ENSDART00000147851	eef1b2	0.252066551	0.849118612	0.755935234	0.422914839
ENSDART00000147854	mfap5	-0.362752481	-0.424642186	-0.795916329	-0.74190928
ENSDART00000147868	vwc2	-0.776498099	-0.767193994	-0.55435848	-0.286994867
ENSDART00000147884	syne2b	0.046598878	-0.495566729	-1.074400394	-0.335549303
ENSDART00000147889	kat5a	0.445126528	0.474310239	0.435051995	0.434365446
ENSDART00000147902	megf11	-0.496471177	-0.600303312	-0.359789074	-0.208412389
ENSDART00000147903	lrit1a	-0.179060864	-0.15522738	-0.479781004	-0.346169989
ENSDART00000147905	fam213aa	-0.253972183	-0.158419429	-0.284170369	-0.196877631
ENSDART00000147938	heg1	-1.97027775	-0.730181455	-5.193199021	-2.593528186
ENSDART00000147963	bco2a	-0.106052135	-0.070354665	-0.433217487	-0.165630718
ENSDART00000147968	rbpms2b	-0.96188315	-0.47610893	0.419978504	0.426103029
ENSDART00000147991	g3bp1	3.532775357	2.102820417	2.620663227	2.000522751
ENSDART00000147998	plxna2	-0.136413264	-0.347150332	-0.077394761	0.05783618
ENSDART00000148011	cby1	0.256002046	0.39067073	0.214338805	0.125714094
ENSDART00000148038	lrfn4b	-0.35705614	-0.30916456	0.014604429	0.153474614
ENSDART00000148039	snx19a	-0.086070054	-0.238060141	-0.431834798	-0.166151211
ENSDART00000148055	si:ch211-67e16.11	-0.532227459	-0.535880084	-0.161015502	0.246252593
ENSDART00000148066	znf395b	-0.110739145	-0.366906743	-0.460042969	-0.183105049
ENSDART00000148073	shisa7b	-0.212233425	-0.392255987	-0.298336106	-0.168085028
ENSDART00000148076	fbxw7	-0.313125013	-0.183670552	-0.007156474	-0.059168192
ENSDART00000148080	rnf19b	0.061412453	-0.676245271	-0.33437989	-0.030752067
ENSDART00000148106	mef2aa	-0.081330082	-0.55524128	-0.609035118	-0.185252531
ENSDART00000148107	SRCIN1	-0.358492187	-0.594267639	-0.130298738	0.102935385
ENSDART00000148120	ephb2a	-0.204590684	-0.196760275	0.359222548	0.385663441
ENSDART00000148125	fam21c	0.420817307	0.137516364	0.034339244	-0.014273742
ENSDART00000148138	lrrc8aa	0.370743632	0.558987897	0.552524282	0.096775057

ENSDART00000148154	stx2a	0.73746977	1.316523952	0.880682553	0.300142462
ENSDART00000148163	frmd4bb	0.943172358	1.400649402	1.507648518	0.548522517
ENSDART00000148175	si:ch1073-440b2.1	-0.235967394	-0.243649374	-0.449710659	-0.316169237
ENSDART00000148181	inpp4aa	-0.260407808	-0.050773424	0.267315477	0.333876837
ENSDART00000148199	stxbp1a	-0.541285454	-0.573795052	-0.292066803	-0.020658402
ENSDART00000148213	yipf6	-0.115338541	-0.114709124	-0.248877885	-0.220567506
ENSDART00000148236	tmem179b	0.928914935	1.103132699	0.9233627	0.146290121
ENSDART00000148246	pole4	-0.080211224	-0.333184545	-0.567162517	-0.350297708
ENSDART00000148249	plppr4b	-0.24256353	-0.068311603	1.098973668	1.360337893
ENSDART00000148278	klf11a	-0.030586225	0.176099641	0.85687578	0.762070868
ENSDART00000148280	prr18	0.103510486	0.905956065	1.016662607	0.752186459
ENSDART00000148296	tnni1d	0.552273501	5.279876604	5.419924358	3.332159231
ENSDART00000148304	myo1f	0.706981048	0.452539664	0.150931707	-0.296211435
ENSDART00000148306	map2	-0.504312708	-0.348446647	-0.171538441	0.068561288
ENSDART00000148327	adamtsl7	2.218956465	1.395778664	0.846103004	0.026559812
ENSDART00000148353	usf2	-0.107987138	-0.265954127	-0.405201274	-0.200197809
ENSDART00000148388	smox	1.14663E-15	4.65884204	4.212605499	2.256263175
ENSDART00000148398	MRAS (1 of many)	1.275583164	1.112018386	0.557077896	0.217843634
ENSDART00000148405	si:ch211-113g11.6	-0.63339569	-0.861477951	-0.083811842	0.259008105
ENSDART00000148422	BX649485.3	1.236550789	0.98767472	0.358686087	-0.350942213
ENSDART00000148432	ptp4a3	0.27125556	0.468426427	0.608500351	-0.013500628
ENSDART00000148447	hnrnpaba	0.222094219	0.226002223	0.267110054	0.234088848
ENSDART00000148456	abcb4	-0.551564057	-0.079040965	-0.313501093	-1.124934266
ENSDART00000148459	cbx1b	-2.930250472	-0.688512699	0.434984356	0.566379272
ENSDART00000148465	slc1a1	-0.751249091	-0.22594806	0.456533505	0.753777978
ENSDART00000148475	cspg5a	0.090085539	0.726711302	1.793409667	1.632985142
ENSDART00000148531	arhgef25b	-0.052778378	-0.311697766	-0.416645212	-0.115394554
ENSDART00000148536	GK3P	-0.308304163	-0.269790809	-0.586409039	-0.39358576
ENSDART00000148537	roraa	-0.160828233	-0.221872095	-0.45696721	-0.083100906
ENSDART00000148540	fech	0.007306354	0.381217581	-0.12547162	-0.024425403
ENSDART00000148564	si:ch211-236p22.1	-0.699364761	-0.998683731	-0.566423622	-0.06995253
ENSDART00000148570	atp2b1b	-0.232205009	-0.177768331	-0.430678176	0.039634464
ENSDART00000148576	pygmb	-0.380093922	-0.425025096	-0.447938233	-0.092571282
ENSDART00000148580	BX005108.2	-0.474486107	-0.575204555	-0.246024325	-0.012613897
ENSDART00000148582	smpd1	0.404585024	0.130204031	0.022544887	-0.113079466
ENSDART00000148590	coro2ba	-0.3608902	-0.079968694	-0.141782791	0.048751465
ENSDART00000148601	insig1	-0.03765091	0.839125818	0.622258944	0.165134159
ENSDART00000148639	ubxn2a	-0.292754646	-0.267419804	-0.368360861	-0.228619688
ENSDART00000148643	phkb	-0.170217144	-0.13328883	-0.2898938	-0.026363851
ENSDART00000148648	rps25	0.412929704	0.39629643	0.197445263	0.016589956
ENSDART00000148653	tmem145	-0.454635973	-0.21216688	-0.078241248	0.035128268
ENSDART00000148659	clip2	0.500760868	0.904821279	1.156870185	0.693646301
ENSDART00000148661	stat2	0.11967996	-0.016653911	1.354000143	0.023510931
ENSDART00000148662	kcnc3a	-0.800710128	-1.106624714	-0.368118634	0.013489304
ENSDART00000148737	btk	1.027482823	0.714086499	0.58431037	0.237640489
ENSDART00000148741	met	0.874910977	0.435904294	-0.125027531	-0.62235308
ENSDART00000148764	ednrbb	1.564011968	2.847115487	1.8915508	0.999631285
ENSDART00000148766	dpf2	1.058462293	0.640887505	0.623810049	0.633616815
ENSDART00000148786	exoc5	0.05617118	0.166256406	0.294936832	0.214091952
ENSDART00000148789	wfs1b	0.104249443	0.302506651	0.278674006	0.047812088
ENSDART00000148794	jupb	-0.404857846	-0.638395215	-0.40436567	-0.262479222

ENSDART00000148795	dpf2	0.12341877	-0.325727004	-0.660147455	-0.013247322
ENSDART00000148802	nexn	-0.381565638	-0.122933428	-0.397541694	-0.196186804
ENSDART00000148822	adgrg1	0.746405238	1.031170771	0.764826924	0.143644812
ENSDART00000148831	fgf11b	-0.269342716	-0.341549392	-0.474527478	-0.280873475
ENSDART00000148834	si:ch1073-155h21.2	-0.200452217	-0.231957247	-0.32585922	-0.162282019
ENSDART00000148845	BX649485.4	2.24411597	1.39694109	1.224587369	-0.358910932
ENSDART00000148894	serpinb14	0.554435408	1.134239409	1.417283925	0.684075923
ENSDART00000148900	kcnc2	-0.819802341	-0.972756522	-0.275729768	0.337871594
ENSDART00000148927	tgfb2	0.452467242	0.589301589	0.923363405	0.590115295
ENSDART00000148930	lingo4b	-0.229730557	-0.308881186	-0.199125951	-0.339156928
ENSDART00000148958	fam150a	-0.081185595	-0.392189222	-0.650641125	-0.329645149
ENSDART00000148963	BX649485.1	1.148936794	0.907522814	0.409280883	-0.023295659
ENSDART00000148971	ctbp2a	-0.618298464	-0.594645977	-0.414478241	-0.261581238
ENSDART00000148975	CU657977.1	0.934819878	0.853574256	0.944102682	0.804503627
ENSDART00000148982	bin1b	0.009431427	-0.214307092	-0.49686004	-0.050411548
ENSDART00000148984	rpl31	0.518485066	0.61867131	0.274282849	0.246502602
ENSDART00000148993	hdac6	0.150136767	0.327518494	0.405976253	0.20477563
ENSDART00000148997	nos1apa	-0.375839925	-0.17136424	-0.288037974	-0.136490158
ENSDART00000149019	itpr1a	0.453945089	0.40663332	0.191730219	0.048560775
ENSDART00000149024	btbd7	-0.242016986	-0.307624283	-0.194506419	-0.161017473
ENSDART00000149029	sagb	-0.046274749	-0.449576755	-0.950069775	-0.043925247
ENSDART00000149032	oat	-0.368235431	-0.40939103	-0.17630927	-0.009811502
ENSDART00000149041	reep1	-0.249037096	0.218537351	0.449912583	0.370752516
ENSDART00000149043	CR855300.1	0.458598352	0.519077258	0.374073731	0.109291075
ENSDART00000149074	sall1b	1.974229239	2.883794957	2.506155499	1.735121972
ENSDART00000149079	sagb	-0.468101765	-0.230897218	-0.419750319	-0.182809331
ENSDART00000149089	anxa11b	0.040867541	0.398676842	0.148460283	-0.067955816
ENSDART00000149115	zgc:158659	1.901261478	2.561373529	3.048076346	2.366410956
ENSDART00000149121	clcf1	1.771744054	1.594454703	1.360720614	0.466339861
ENSDART00000149151	sptbn2	-0.256411956	-0.316484744	-0.145496042	0.017178704
ENSDART00000149158	dmxl2	-0.239475556	-0.38571718	-0.12120231	0.150747303
ENSDART00000149169	ssr2	0.474946343	0.309006192	0.206925318	-0.029893306
ENSDART00000149171	srp9	-0.011162136	-0.135869324	-0.296581397	-0.280358452
ENSDART00000149175	pou4f3	-1.520089487	-1.231379123	0.357488642	0.743891028
ENSDART00000149198	epas1a	0.235051634	0.343796351	0.502704685	-0.080436042
ENSDART00000149200	anxa11b	1.743372734	3.631513258	2.108721032	3.508584927
ENSDART00000149204	si:dkey-85n7.7	2.603000794	2.822199237	2.219318015	0.958882963
ENSDART00000149218	elk3	0.538927451	0.404579774	0.375543991	0.048753146
ENSDART00000149222	pglyrp2	-0.019258678	-0.204426336	-0.652532049	-2.674820692
ENSDART00000149237	xirp2a	0.190889632	0.253883647	0.37076536	0.19112506
ENSDART00000149260	nt5c3a	0.06053373	-0.254595189	-0.368052769	-0.218176136
ENSDART00000149267	kel	0.933657877	1.291347217	1.534062377	0.13083457
ENSDART00000149276	lzts3b	-0.642406039	-0.470964243	-0.143114993	-0.054979203
ENSDART00000149283	zgc:112001	-0.707617666	-0.492468931	0.006563399	0.231921747
ENSDART00000149297	slc20a2	-0.247675699	-0.319353289	-0.309560982	-0.222732695
ENSDART00000149299	cers2b	0.489876545	0.297388144	0.375525704	0.021430111
ENSDART00000149311	gpr63	-0.396864855	-0.311816928	-0.030936535	0.061966221
ENSDART00000149316	rasa1a	-0.4434975	-0.604862985	0.040513048	0.244834334
ENSDART00000149320	arl8a	0.200543842	0.321313695	0.387648659	0.1981557
ENSDART00000149335	kcna1a	-0.92291358	-0.966385056	-0.49989391	-0.001249229
ENSDART00000149339	si:ch211-175f12.2	-0.297380973	-0.332932663	-0.389357991	-0.204952824

ENSDART00000149350	fkbp4	-0.198226552	-0.122860425	-0.266664215	-0.086727437
ENSDART00000149351	scd	-0.095125933	1.291914984	1.511394876	0.539585936
ENSDART00000149352	si:ch211-163l21.11	-0.266781177	-0.351609906	-0.560319486	-0.211979525
ENSDART00000149367	si:ch73-290k24.5	-0.213839599	-0.466705261	-0.018322662	0.130528894
ENSDART00000149411	atxn1b	-0.356760798	-0.298446699	-0.334747495	-0.251003673
ENSDART00000149425	SLC25A22 (1 of many)	-0.428786809	-0.626535356	-0.125118952	0.12464047
ENSDART00000149429	boka	-0.220682727	-0.305502243	-0.692638969	-0.393597854
ENSDART00000149431	sema4aa	1.058121853	0.676658919	0.239884836	-0.1730827
ENSDART00000149443	pcdh19	-0.547618951	-0.594468642	-0.345755383	0.11053464
ENSDART00000149468	rps10	2.202930165	1.872426465	0.292067044	0.38759709
ENSDART00000149478	galns	1.09483472	0.726913129	0.227424057	0.052897193
ENSDART00000149486	def6b	4.188632994	4.544630212	4.117698226	0.985190768
ENSDART00000149510	f13a1b	0.823651803	0.467086429	0.602200924	0.078453199
ENSDART00000149512	CU467655.1	-0.320227651	-0.180971804	-0.440965145	-0.207148298
ENSDART00000149518	SLC25A22 (1 of many)	-1.000880918	-1.544420441	-0.824653844	-0.438490591
ENSDART00000149546	myo15aa	-0.410128868	-0.287434444	-0.355050844	-0.094306045
ENSDART00000149552	FP325120.1	-0.456523138	-0.25356238	-0.125718739	0.024031302
ENSDART00000149553	hmgcra	-0.282814218	-0.006298507	0.4014174	0.446278745
ENSDART00000149564	CU467655.1	-0.291119215	-0.167904608	-0.380163625	-0.141898613
ENSDART00000149569	CR848047.1	-0.094108393	-0.299800905	-0.352835774	-0.193405328
ENSDART00000149574	ca14	-0.121794187	-0.18625006	-0.561537732	-0.325393218
ENSDART00000149584	gng12a	0.603189677	0.302272198	-0.104854769	-0.377654322
ENSDART00000149594	ank3b	-0.174131144	-0.266770414	0.69684285	0.78513869
ENSDART00000149600	tpp1	0.822885392	0.356076016	0.047488091	-0.289304028
ENSDART00000149608	nexn	-0.437154987	-0.289194529	-0.346094746	-0.307861675
ENSDART00000149618	mid1	-0.406831126	-0.160364287	0.131354752	0.136351442
ENSDART00000149634	atp6ap2	-0.108949021	-0.143807338	-0.457436101	-0.199272375
ENSDART00000149652	AL928685.4	-0.221690272	-0.265792933	-0.407449839	-0.293257945
ENSDART00000149655	adam8b	1.224022341	1.219775985	1.033392521	0.588329024
ENSDART00000149659	ezh1	0.045526141	0.005041587	-0.251417621	-0.327846848
ENSDART00000149666	dock3	-0.065088232	-0.425598201	-0.009057049	0.161364952
ENSDART00000149684	si:dkeyp-52c3.2	2.125770421	2.315774699	2.351460593	2.938761267
ENSDART00000149685	gnao1a	-0.290173142	-0.321971232	0.118032615	0.301423091
ENSDART00000149689	mob1bb	-0.2705299	-0.18213785	-0.463988827	-0.361129298
ENSDART00000149711	slc4a2b	0.638486491	0.276788243	-0.283420926	-0.322786037
ENSDART00000149730	stox2a	0.026475093	0.234017219	0.506304198	0.292160739
ENSDART00000149740	si:ch211-149b19.2	-0.851560484	-0.688459713	0.123433485	0.442881876
ENSDART00000149760	triobpa	0.630702727	0.141628128	0.231366625	0.068551496
ENSDART00000149768	kif21a	-0.021643242	0.361851849	0.686688714	0.546979573
ENSDART00000149777	setx	0.020956021	0.009824369	0.394668282	0.077312111
ENSDART00000149784	scn3b	0.168579716	0.346498596	0.172197933	0.640507294
ENSDART00000149786	dhrsx	4.71086866	4.438400236	3.335716316	3.374642973
ENSDART00000149787	col4a3bpa	0.193295875	0.430904088	0.63143914	0.354005578
ENSDART00000149816	hspb1	-0.566005876	-0.156876752	-0.219080206	-0.313669761
ENSDART00000149821	ecsit	4.865284679	4.410737197	4.242321149	4.574026911
ENSDART00000149833	cnga3a	-0.072993636	-0.371949035	-0.58833358	0.07281763
ENSDART00000149859	gldn	-0.670802099	-0.540937826	-0.034323087	-0.157128438
ENSDART00000149871	cx40.8	0.501427104	0.583813885	0.280966778	-0.058317019
ENSDART00000149878	otud4	0.242358714	0.196542793	0.87579795	0.138730183
ENSDART00000149882	si:ch211-117c9.1	1.22256875	0.270055012	0.422501128	0.414018825
ENSDART00000149910	abl1	0.087815124	0.252042838	0.385819934	0.051793038

ENSDART00000149913	b4galt1	0.757613392	0.563165908	0.39069258	0.030742735
ENSDART00000149914	aatka	-0.270082025	-0.541170375	-0.440872523	-0.175726567
ENSDART00000149935	nt5c3a	0.05115392	-0.273408486	-0.497911915	-0.317644766
ENSDART00000149947	bcl2b	-0.663523207	-0.706570637	-0.112082166	-0.007869569
ENSDART00000149951	BX666064.1	1.493495344	0.978793976	0.565382246	-0.392061768
ENSDART00000149956	apc	0.195183663	0.26527048	0.385323133	0.453680151
ENSDART00000149960	fasn	2.288734609	2.375010827	1.541557565	1.919894603
ENSDART00000149966	b3gat3	-0.190835062	-0.286049968	-0.133094624	-0.134465202
ENSDART00000149978	fabp7a	0.33770709	0.764013846	0.558825405	0.488886506
ENSDART00000149992	si:dkey-231j24.3	-0.792591394	-0.543368341	0.065178142	0.204406436
ENSDART00000149993	pygmb	-0.443639651	-0.459929444	-0.472379886	-0.083989126
ENSDART00000150013	cd276	1.481977913	1.919249755	1.718653495	0.833090259
ENSDART00000150023	slc16a1a	-2.911491812	0.586509109	-0.148102592	-0.093926934
ENSDART00000150028	onecutl	-0.126745973	-0.253663984	-0.285061154	-0.103777564
ENSDART00000150033	bin1b	0.041335049	-0.185764451	-0.523044089	-0.09192537
ENSDART00000150036	adam23a	-0.62166293	-0.515362417	0.016147076	0.303932596
ENSDART00000150043	dhrsx	-0.370311342	-3.592572721	-0.142322612	-0.462525038
ENSDART00000150045	adamts18	-0.187059316	-0.292648437	-0.628764119	0.030985495
ENSDART00000150050	hnrnpaba	0.395350897	0.441973379	0.591388417	0.200313414
ENSDART00000150064	cdc14b	-0.488634498	-0.055933719	-0.080964497	0.027342931
ENSDART00000150068	sox6	-0.111707858	-0.047191704	0.355284385	0.700337568
ENSDART00000150072	si:dkey-85n7.8	0.988462904	1.496111016	1.983172292	0.611120113
ENSDART00000150088	BX649485.2	1.751466958	1.112873174	0.6867673	-0.722119651
ENSDART00000150116	alk	-0.197441993	-0.291262237	-0.113299878	-0.034468735
ENSDART00000150128	foxp1b	0.347545443	0.556370195	1.342974258	1.269825931
ENSDART00000150146	smarcd3b	-0.318612181	-0.278781268	0.723080439	0.640333876
ENSDART00000150148	slc7a6	0.22923155	0.720543979	1.05061766	1.171565717
ENSDART00000150149	tdp1	0.245251359	0.536780821	0.276916415	0.174119987
ENSDART00000150184	gpatch4	0.624444079	0.471783034	0.550844363	0.152733798
ENSDART00000150190	ggctb	-0.381240597	-0.099058914	-0.562063682	-1.136274784
ENSDART00000150201	MRAS (1 of many)	1.00558208	0.977546999	0.676765678	0.026394977
ENSDART00000150228	chata	-0.431975216	-0.532525907	-0.558690348	-0.371717362
ENSDART00000150353	mdh1aa	-0.527537579	-0.6816935	-0.262947	-0.041325795
ENSDART00000150362	si:ch211-197k17.3	-0.145582952	0.205377076	0.711396584	0.681320306
ENSDART00000150376	ldb1a	-0.16522649	-0.276396303	-0.2812216	-0.239874431
ENSDART00000150386	si:ch73-6k14.2	-0.397499259	-0.321996107	-1.565555621	-1.132350175
ENSDART00000150389	tmem168a	0.525344796	0.286469995	0.221730459	0.080011152
ENSDART00000150391	si:ch211-214j24.14	2.502222784	2.698584631	2.367333489	0.935046128
ENSDART00000150400	tbc1d2b	-4.044163911	-1.453975239	-0.803224358	-1.31483616
ENSDART00000150430	pane1	0.044208151	-3.417207412	-0.655487552	-0.439296687
ENSDART00000150484	BX465862.4	1.071580051	0.886976814	0.477743025	0.134467836
ENSDART00000150553	fam19a5a	-0.538194047	-0.928835726	-0.2762266	0.015308786
ENSDART00000150580	BX649307.1	0.257402348	0.657324095	0.496054556	0.285668011
ENSDART00000150651	BX465862.4	0.896323656	0.956165673	0.709439713	0.378868912
ENSDART00000150692		0.448027135	0.604850915	0.68748119	0.482541087
ENSDART00000150751	CR293532.1	1.967646607	1.446997774	2.118831868	1.197447208
ENSDART00000150789	USMG5	-0.285153636	-0.258859464	-0.457141251	-0.093210696
ENSDART00000150839	fam57bb	-0.627521697	-0.387137362	0.25664183	0.060693872
ENSDART00000150863	nrp2a	0.543372402	0.197731133	0.582446066	0.760458596
ENSDART00000150919	si:ch211-191i18.2	1.045780222	1.362401557	0.586788909	0.260330898
ENSDART00000150927	ubl7b	0.617142901	0.651681168	0.305601173	0.171994365

ENSDART00000150932	lpcat1	1.824153615	2.726791026	2.775021546	2.58536861
ENSDART00000150934	alg6	0.502789869	0.534691837	0.441461289	0.13055149
ENSDART00000150940	si:ch211-193k19.2	1.599024227	0.310661889	0.753508784	0.054490277
ENSDART00000150949	nfyc	-0.535274529	-0.301463031	-0.212807295	0.074596031
ENSDART00000150955	bmpr2a	-0.667543016	-0.436786519	-0.136898455	0.053141678
ENSDART00000150958	lrrc4ba	-0.155064877	-0.413501981	-0.171991106	-0.022578699
ENSDART00000150961	wdr91	4.358324282	4.633846856	4.480446443	4.649897846
ENSDART00000150975	smad7	-0.404377575	-0.51744405	-0.473359841	-0.49979754
ENSDART00000151013	ensaa	-0.338295629	-0.414678183	-0.638369138	-0.341312384
ENSDART00000151022	si:ch211-191i18.4	-0.318627462	-0.467051418	-0.723047751	-0.473341501
ENSDART00000151044	baz2ba	2.371956879	3.808025242	3.802702579	3.968575995
ENSDART00000151084	uqcrb	-0.019553677	-0.080328791	-0.732409977	-0.191112562
ENSDART00000151099	cacng5a	-0.404305845	-0.269968357	-0.142737848	0.07277718
ENSDART00000151127	thraa	-0.798583389	-0.802364861	-0.351487338	-0.589241182
ENSDART00000151143	rbfox1	-0.676737106	-0.648326228	0.275380809	0.524086709
ENSDART00000151158	ncaldb	0.047289673	-1.437087464	-0.332259167	0.054087527
ENSDART00000151189	sept12	-0.01197373	-0.348568497	-0.258432997	-0.257716236
ENSDART00000151195	march7	-0.18277274	0.240052097	0.74148711	0.799803253
ENSDART00000151200	mmp17a	-0.370431866	-1.068890772	-0.706081815	-0.179291377
ENSDART00000151203	rgs9a	-0.190499153	-0.256482975	-0.462262315	-0.086082277
ENSDART00000151205	aldh1l1	0.651327821	0.835468293	0.448149629	0.158439741
ENSDART00000151229	kcnj5	-0.403592435	-0.680204447	-0.664541848	-0.399506515
ENSDART00000151235	rcvrn3	-0.033671108	-0.144752489	-0.498884135	-0.315369716
ENSDART00000151237	oxnad1	0.332718432	0.237746325	0.070395116	0.076154746
ENSDART00000151246	gnb3b	-0.446677088	-0.413500571	-0.183093758	-0.072200588
ENSDART00000151247	scn1lab	0.293159534	0.684635422	0.628869811	0.299982725
ENSDART00000151252	myo10l1	-0.09485067	-0.323915115	0.494165668	0.855040148
ENSDART00000151282	si:ch73-248e21.5	0.830287534	0.642657587	0.149520253	-0.453672012
ENSDART00000151299	scn8ab	-0.339946061	-0.372438378	0.28752079	0.526264643
ENSDART00000151305	slc16a6b	-0.420275567	-0.158970487	-0.073625291	-0.053092638
ENSDART00000151311	GPR161 (1 of many)	0.101386436	0.588870162	0.204324291	-0.302118942
ENSDART00000151322	KRT18 (1 of many)	3.074943635	2.466392918	2.650579676	1.467715456
ENSDART00000151342	arhgap42a	0.69255684	0.716882566	0.356486042	0.299890281
ENSDART00000151358	rgl3a	-0.536133566	-0.674550597	-0.735844208	-0.535493428
ENSDART00000151383	eif1b	0.629753576	0.386164956	0.580421795	0.441741802
ENSDART00000151386	CU659670.1	-0.061309281	-0.197320939	-0.510180812	-0.129307388
ENSDART00000151390	si:ch211-195b13.1	0.447846827	0.275025282	0.031728831	0.543393224
ENSDART00000151393	zak	0.559981256	0.606971645	0.291742344	0.078029866
ENSDART00000151399	pak7	-0.095169867	-0.142909818	-0.239638425	0.065715442
ENSDART00000151404	abat	-0.288767469	-0.303619722	-0.197451372	-0.147078748
ENSDART00000151419	tgfa	-0.147139604	-0.1316737	-0.368157435	-0.244071655
ENSDART00000151423	c1ql3b	-0.428342943	-0.592751887	-0.458841393	-0.097254864
ENSDART00000151448	vamp4	0.36274105	0.703182259	0.708668654	0.04883985
ENSDART00000151467	slc44a2	1.227786397	2.405127088	2.406151956	1.882399418
ENSDART00000151489	LRRC2	-0.220896871	-0.332799828	-0.436191804	-0.38527929
ENSDART00000151514	wipi1	-0.487231741	-0.283410935	-0.040753743	-0.07881031
ENSDART00000151547	pnpla7a	0.300123054	0.241347051	0.10241403	-0.203931018
ENSDART00000151550	usp8	0.146844734	0.224801555	0.32026745	0.237867483
ENSDART00000151567	sgk494b	-0.371209064	-0.282108451	-0.446431854	-0.117181288
ENSDART00000151571	ahdc1	-0.753523402	-0.774930454	-0.265860844	-0.025283973
ENSDART00000151586	ftr56	2.21645788	0.879044173	3.24618964	0.72203566

ENSDART00000151611	BX537277.4	-0.440906854	-0.477467469	-0.367060961	-0.356985579
ENSDART00000151613	lhfpl2a	2.211015019	2.8976425	2.404140419	1.864383361
ENSDART00000151627	si:ch73-362m14.2	-1.029772234	-1.504293907	-1.467613461	-0.562650292
ENSDART00000151634	tnrc6c1	-0.074330004	-0.222991449	-0.321878312	-0.006293329
ENSDART00000151648	ntng2b	-0.44606387	-0.406664301	-0.175135105	0.111487092
ENSDART00000151661	wipf1b	0.584481191	0.31541115	0.189596046	-0.173471027
ENSDART00000151670	fbxl16	-0.100117774	-0.155204519	-0.627708744	-0.129502503
ENSDART00000151672	si:ch211-276a17.5	-0.716987346	-0.427751363	-0.201345778	-0.037618143
ENSDART00000151674	pcdh9	-0.204552278	-0.440082629	-0.022873178	0.221939437
ENSDART00000151679	si:ch73-233k15.2	0.305634484	0.260016753	1.072404438	-0.272816623
ENSDART00000151685	lhfpl4a	-0.450299252	-0.419390296	-0.093062661	0.17106537
ENSDART00000151698	syt3	-0.446756628	-0.536044497	-0.128625894	0.106551311
ENSDART00000151717	march7	0.010098896	0.251138543	0.521642437	0.401235935
ENSDART00000151718	tns1a	-0.41721923	-0.880763022	-0.123281901	0.108356531
ENSDART00000151739	pax10	-0.427677153	-0.286784205	-0.176549765	-0.047893912
ENSDART00000151743	tmem237b	0.052429896	-0.216668621	-0.455661391	-0.118191943
ENSDART00000151785	klc4	0.265672724	0.701188452	0.757625768	0.469102543
ENSDART00000151805	vamp4	0.722926228	0.32988105	0.08556674	0.068052265
ENSDART00000151808	rcvrn3	-0.028816037	-0.156203513	-0.580402071	-0.415774036
ENSDART00000151822	tuba8l4	0.798817947	0.734886282	0.324378588	0.272246902
ENSDART00000151842	lrrn3a	-0.579197629	-0.548770022	-0.143313453	0.249550285
ENSDART00000151853	btbd10a	-0.220795221	-0.048228521	0.169228662	0.338815973
ENSDART00000151874	gtf2h5	-0.186830286	-0.721090531	-0.840818906	-0.885273426
ENSDART00000151878	rab3gap2	0.026444427	0.181039642	0.242597677	0.247371128
ENSDART00000151881	rp1l1b	-0.468794546	-0.617233135	-0.603918539	-0.270450371
ENSDART00000151892	BX248324.2	1.389473856	2.137590244	1.979774015	1.113747327
ENSDART00000151899	ldlrb	-0.013482381	0.739364147	1.30137615	0.56832803
ENSDART00000151904	foxn3	-0.402174688	-0.533042351	-0.141780798	0.022724604
ENSDART00000151915	ptchd1	-0.713611001	-0.66822522	0.042208909	0.328774722
ENSDART00000151920	si:ch211-140m22.7	-0.371308279	-0.473110833	-0.103450206	0.052557108
ENSDART00000151921	dnmt3ab	-0.131014471	-0.104325638	0.10563751	0.340310238
ENSDART00000151970	si:ch211-248l17.3	-0.372087768	-0.337523565	0.171433041	0.720267147
ENSDART00000151999	ttc27	0.537657382	0.63628465	0.751878141	0.471568333
ENSDART00000152000	hectd1	2.43305833	3.547853034	-1.454489893	-0.017509384
ENSDART00000152005	ppp1r13ba	-0.032736325	-0.117362615	-0.454836475	-0.009028338
ENSDART00000152006	si:ch211-236p5.3	-1.010175109	-0.375171908	-0.903663548	-0.553145976
ENSDART00000152011	DOCK4 (1 of many)	-0.107148853	-0.150156526	0.321338768	0.540658105
ENSDART00000152019	si:ch211-234p6.5	-0.203738294	-0.079554548	-0.244044589	-0.027716123
ENSDART00000152068	AL590149.1	-0.322191556	-0.083241136	-0.42500156	-0.283301232
ENSDART00000152069	chst2b	-0.329630849	-0.401663244	-0.360860216	-0.363893389
ENSDART00000152122	si:ch211-176g6.2	1.234754318	1.008584835	0.458934208	-0.407921233
ENSDART00000152128	MAZ (1 of many)	-0.18246323	-0.447481895	-0.170629979	-0.120255425
ENSDART00000152145	si:ch211-207i1.2	-1.105453902	-1.700204988	-2.013950338	-1.361589232
ENSDART00000152152	si:ch73-24k9.2	1.048518423	0.665615029	1.113264445	-0.254081843
ENSDART00000152172	BX248418.1	-0.267252802	-0.476860817	-0.659952516	-0.030823789
ENSDART00000152181	mapta	-0.246945515	0.298203412	0.945051693	0.777575562
ENSDART00000152195	si:ch211-113a14.18	0.940862394	0.989824089	1.044244017	0.604507986
ENSDART00000152240	si:ch1073-174d20.2	0.11047675	0.254355415	0.357239942	0.147146143
ENSDART00000152253	kcnj13	-0.175334575	-0.22735068	-0.506366898	-0.552648836
ENSDART00000152269	syncrip	0.263965684	0.255402715	0.319958135	0.269096167
ENSDART00000152275	BX908780.3	-0.123347867	-0.42152381	-0.357247701	-0.183442147

ENSDART00000152277	si:ch73-168d20.1	0.909762162	0.836471756	1.203009656	0.352187699
ENSDART00000152284	si:ch73-301j1.1	-0.68804704	-0.624114046	1.993753311	-0.48476616
ENSDART00000152295	FAM83G	1.150380037	1.206829236	1.079415548	0.591155843
ENSDART00000152299	si:dkey-25o16.4	1.175283487	1.143366275	0.842863117	0.368204805
ENSDART00000152318	si:ch211-286b5.2	1.976393579	3.213645724	2.557166791	1.560923939
ENSDART00000152371	usp2a	-0.286860533	-0.506577502	-0.337595735	0.001938619
ENSDART00000152376	CLSTN2 (1 of many)	-0.314646809	-0.323760148	-0.422303692	-0.021838531
ENSDART00000152378	tgif1	0.194631245	0.419803811	0.14379228	-0.205607529
ENSDART00000152388	CR354435.3	1.276581803	1.114721557	1.265047914	0.436655827
ENSDART00000152400	dbf4b	0.645863235	0.809166025	0.868170031	0.806915507
ENSDART00000152409	mpp3b	-0.399859226	-0.270912664	-0.49906829	-0.114613724
ENSDART00000152428	arhgap32a	-0.870901224	-0.197122132	-0.387452332	-0.355800105
ENSDART00000152429	si:ch211-22i13.2	-0.050442481	-0.183983476	-0.310965899	-0.270852228
ENSDART00000152431	grid1b	-0.386738283	-0.602313736	-0.348261702	0.022251996
ENSDART00000152432	lratb.2	0.819163408	1.604350772	-0.35089707	-0.825333131
ENSDART00000152478	stambpl1	0.081881747	0.120249323	0.496702134	0.282826765
ENSDART00000152479	si:dkey-58j15.11	0.30322392	0.88586109	0.224237301	-0.316952944
ENSDART00000152489	irf3	-0.152502705	0.050924712	1.421424509	-0.375867622
ENSDART00000152510	ccser2b	0.726161978	0.836833026	1.118702985	0.610749876
ENSDART00000152513	arfip2a	-0.209731462	-0.427433763	-0.319460479	-0.125960154
ENSDART00000152520	sh3bgr	-0.276595165	-0.180114366	-0.559026228	-0.573707548
ENSDART00000152531	chrna10a	-0.39848157	-0.348304481	-0.425468269	-0.274269467
ENSDART00000152535	CR846082.2	-0.517845666	-0.356698278	-0.420655711	-0.212748903
ENSDART00000152556	FAM13C	-0.458278075	-0.375744323	-0.341944162	-0.232139437
ENSDART00000152562	eif3f	0.257721359	0.386526337	0.235538886	0.038968621
ENSDART00000152567	frmpd2	-0.319245931	-0.373961894	-0.203551264	-0.037181194
ENSDART00000152580	nlgn4a	-0.383612645	-0.391621152	-0.368243921	-0.24740854
ENSDART00000152603	egln2	-0.344914112	-0.184325703	-0.029073268	0.070142585
ENSDART00000152636	clocka	0.008631834	0.196030905	0.593132542	0.426491394
ENSDART00000152641	dnm3a	1.808565272	2.497351665	2.011092537	0.57584064
ENSDART00000152666	slc25a23a	-0.288940584	-0.374696227	0.31705403	0.139123262
ENSDART00000152672	zgc:152977	-0.296261868	-0.305183243	-0.595435728	-0.424752212
ENSDART00000152678	ifit10	0.50194473	0.092674262	4.442632616	1.169305685
ENSDART00000152680	pyyb	2.62463081	4.023644413	3.815932945	2.663962921
ENSDART00000152689	oplah	-0.018001137	-0.164313071	-0.461321001	-0.221934453
ENSDART00000152699	si:ch211-286b5.9	1.056007406	0.325301277	0.188178466	-0.341462888
ENSDART00000152703	BX901920.1	-0.098796288	0.57736287	0.482412674	0.325371495
ENSDART00000152720	MARK4 (1 of many)	-0.106851349	-0.112526868	-0.273643684	-0.009380005
ENSDART00000152766	CR354435.4	0.814174745	0.989990518	0.663039704	0.339445782
ENSDART00000152768	si:ch211-207i1.2	-0.201357893	-0.51029626	-0.665018804	-0.121583536
ENSDART00000152780	tex2l	-0.378255597	-0.430378354	-0.394491857	-0.23990429
ENSDART00000152788	mki67	1.022403776	0.934711217	0.634468641	0.246495311
ENSDART00000152789	etaa1	1.060104568	1.006586924	0.636303316	0.452429723
ENSDART00000152844	cdc42ep1a	-0.176432004	-0.234011448	-0.309447838	-0.303047875
ENSDART00000152855	prdx1	2.014399342	2.912277881	2.532639813	2.043057891
ENSDART00000152858	rcan2	-0.344037465	-0.451039286	-0.208970338	-0.321499331
ENSDART00000152879	ablim2	-0.320970088	-0.240571783	-0.134413966	0.04563774
ENSDART00000152892	slc16a8	2.182950408	0.789162193	-0.768133939	-1.779969995
ENSDART00000152907	si:ch1073-184j22.2	0.289101439	0.219661264	0.052983655	0.072807111
ENSDART00000152924	stxbp4	0.009805274	-0.210366163	0.385226099	0.296155125
ENSDART00000152936	gabrz	-0.611503657	-0.756211778	-0.185809647	0.356886347

ENSDART00000152942	jcada	-0.053928395	-0.076278472	-0.332447962	-0.296311136
ENSDART00000152954	rrm2	3.254915605	2.753529904	1.974728566	1.135298044
ENSDART00000152965	si:dkeyp-104f11.9	-0.448853405	-0.428655614	-0.583739797	-0.149977842
ENSDART00000152968	si:dkey-21c1.1	0.134479312	-0.180316529	-0.580606373	-0.202289334
ENSDART00000152973	RNF157	-0.237155894	-0.35583425	-0.110722293	0.097030733
ENSDART00000153013	eef1db	0.150654853	0.305331563	0.633860114	0.458033815
ENSDART00000153022	AL935194.1	-0.839883385	-0.952071296	-0.323659944	-0.119202215
ENSDART00000153086	kctd17	-0.109500243	-0.169719356	-0.312398256	-0.16098927
ENSDART00000153087	bmf2	0.285623957	0.323465948	0.348774195	0.627801645
ENSDART00000153124	CU570682.1	-0.274154453	-0.462025745	-0.49154679	-0.26836126
ENSDART00000153134	mark3b	-0.033481129	-0.166637764	0.542361776	0.482050901
ENSDART00000153146	rbfox3a	-0.546449898	-0.43963695	0.174623158	0.484406984
ENSDART00000153167	hlfb	-0.470839666	-0.326611575	-0.235752692	-0.264047019
ENSDART00000153177	mkl2a	-0.042089487	-0.340254508	-0.226976022	-0.084520833
ENSDART00000153187	thrab	-0.362356661	-0.551240408	-0.403932333	-0.192068079
ENSDART00000153190	vash2	-0.078136066	-0.226923996	-0.395295378	-0.160874636
ENSDART00000153194	si:dkey-38p12.3	0.25264745	0.485169484	0.572803819	0.07332987
ENSDART00000153199	afap1	0.840574953	1.106233667	1.438882155	1.005659187
ENSDART00000153200	si:ch211-194k22.8	0.210290351	0.451170205	0.37501933	0.225583201
ENSDART00000153212	adgra1a	0.27394507	0.382231602	0.735064793	0.705504548
ENSDART00000153217	eef1db	0.666551397	1.483640099	2.024242417	1.323561336
ENSDART00000153225	GUCY2C (1 of many)	3.255008425	3.866902647	2.201523204	2.781639432
ENSDART00000153227	nfe2l1b	0.688085235	1.301360444	1.138008541	0.729999096
ENSDART00000153245	stxbp4	-0.144159334	-0.445073444	0.187773192	0.216239919
ENSDART00000153247	si:ch211-120g10.1	-0.520249419	-0.414424265	-0.454570577	-0.107523144
ENSDART00000153248	chadlb	1.217222776	0.847984599	0.639882396	-0.038384888
ENSDART00000153256	cacna1g	-0.53708851	-0.717133052	-0.415346006	-0.243050395
ENSDART00000153284	tbkbp1	-0.504212532	-0.489626186	-0.240468452	0.072471561
ENSDART00000153289	nos1apb	-0.207119032	0.371227589	0.722590517	0.685079944
ENSDART00000153296	nsfb	0.330739865	0.349896221	-0.030123095	-0.208569864
ENSDART00000153306	hcar1-4	0.883818254	1.413387657	1.160612678	0.305432292
ENSDART00000153307	si:ch73-344o19.1	0.586530995	0.605191095	0.75799445	0.479323327
ENSDART00000153308	dcun1d4	-0.403892315	-0.460237446	-0.667559321	-0.876458812
ENSDART00000153311	syndig1l	-0.402504141	-0.415168554	-0.17721556	0.113761296
ENSDART00000153317	abracl	3.781753376	3.851435778	3.391861813	0.497752701
ENSDART00000153334	serinc1	0.574524524	0.605164909	0.10821359	0.438731193
ENSDART00000153339	adam17b	-0.104549731	-0.262233446	-0.335652873	-0.112647107
ENSDART00000153355	grin2cb	-0.524459194	-0.47344108	-0.357891852	0.017542632
ENSDART00000153375	CU571074.1	-0.192846631	-0.331910339	-0.069344033	0.142583214
ENSDART00000153377	eef1db	0.533867077	0.950085432	1.385417409	1.0336151
ENSDART00000153390	gpam	-0.267998677	-0.071527709	0.102371515	0.045213236
ENSDART00000153391	otofa	-0.790883428	-1.357026899	-0.581227909	-0.190433713
ENSDART00000153442	rxfp2a	-0.191591927	-0.398184153	-0.707111514	-0.312025768
ENSDART00000153445	exoc7	3.557809232	3.85646058	3.295092173	3.102150432
ENSDART00000153452	hsp90ab1	0.284157203	0.639252574	0.548907136	0.495783326
ENSDART00000153491	si:dkey-238d18.5	0.983293911	0.529719125	0.777356871	-0.049086963
ENSDART00000153510	si:ch73-160l8.2	0.056068509	0.128148598	1.161251796	0.352706117
ENSDART00000153514	si:ch211-121j5.4	-0.438676742	-0.189457468	-0.187818697	-0.117536785
ENSDART00000153562	ppfia3	-0.022654827	0.232304266	0.485406891	0.444415736
ENSDART00000153570	AL808129.1	-0.250097653	-0.308065347	-0.243803188	0.04714878
ENSDART00000153591	si:dkey-56f14.7	-0.35474859	-0.1685547	-0.219665386	-0.050831688

ENSDART00000153619	thsd7ab	-0.196544647	-0.395072863	-0.388102838	-0.013196959
ENSDART00000153654	piezo2a.2	0.718362747	0.545453706	0.495090248	0.182038228
ENSDART00000153657	trim3a	0.068235605	0.356875561	0.585605002	0.467600874
ENSDART00000153666	AL732567.1	0.845331402	1.050170733	0.86587331	0.828206988
ENSDART00000153673	kcnb1	-0.632196562	-0.836186932	-0.481100002	-0.109364665
ENSDART00000153700	BX088707.1	0.029406694	0.3559423	0.787563577	0.447534651
ENSDART00000153704	anks1ab	-0.440221806	-0.75898091	-0.133742531	0.137005152
ENSDART00000153730	CU856222.1	-0.553584022	-0.481855955	-0.007954528	0.343506731
ENSDART00000153731	si:dkey-79d12.5	1.619937595	1.685958359	0.880018827	0.052586274
ENSDART00000153743	rp1l1a	-0.216164399	-0.344666036	-0.324542341	-0.024409204
ENSDART00000153751	rnf146	2.626965217	2.377858038	3.541283251	3.121825847
ENSDART00000153773	cipca	-0.044231438	-0.31334692	-0.44667945	-0.17971329
ENSDART00000153824	napba	-0.634929885	-0.324273151	-0.096308603	0.10005339
ENSDART00000153828	atp2b3b	-0.474522045	-0.66566516	-0.285016921	0.075707306
ENSDART00000153834	sypl1	0.749942367	0.493446597	0.028135514	-0.603815485
ENSDART00000153841	lmcd1	1.804357101	2.873087613	2.523453656	2.002290777
ENSDART00000153847	si:dkey-7i4.24	4.195698891	4.052966176	3.712547417	1.148430087
ENSDART00000153897	ndnfl	-0.781136652	-1.071097643	-0.053351937	0.577095222
ENSDART00000153904	si:dkey-108k21.7	-0.206298886	-0.326824637	-0.265415499	-0.00099298
ENSDART00000153925	fut9a	-0.379466906	-0.312742341	-0.333889875	-0.027131911
ENSDART00000153937	prkd1	-0.431650217	-0.382419593	-0.495097396	-0.310742086
ENSDART00000153944	grm8a	-0.44814853	-0.63071211	-0.403185415	0.043396209
ENSDART00000153950	si:dkey-106n21.1	1.634202521	0.388901976	0.409369194	0.514423363
ENSDART00000153951	myo18ab	0.15536019	0.13591756	0.249484283	0.108775298
ENSDART00000153959	pdcl	-0.115849049	-0.178115822	-0.269080801	-0.124407546
ENSDART00000153968	apba2a	-0.094717367	0.193224107	0.542818077	0.184287162
ENSDART00000153990	CR848791.2	3.062868799	2.217074852	2.048805323	1.916392428
ENSDART00000153992	AL844141.1	0.512134791	0.207788846	-0.514504406	-0.130928693
ENSDART00000154039	cntnap2a	-0.318660467	-0.524836596	-0.23230439	0.054653452
ENSDART00000154045	glg1a	0.211468857	0.293330382	0.40088818	0.351496464
ENSDART00000154066	isg20	0.340483696	0.28200181	0.116846361	-0.121466791
ENSDART00000154077	arhgap29a	-0.13604217	-0.133932896	-0.339961978	-0.305768852
ENSDART00000154085	gipr	-0.077819916	-0.236822644	-0.445528895	-0.377678023
ENSDART00000154109	tp53inp2	0.254956915	0.876540848	0.694070175	0.684041374
ENSDART00000154132	nptx2a	-0.534082272	-0.549346998	-0.399917367	-0.21269489
ENSDART00000154141	BX649398.2	0.278828233	0.21540553	-0.861481785	-0.785329833
ENSDART00000154148	camk1a	-0.513931344	-0.665527159	-0.499415187	-0.020106333
ENSDART00000154187	fat3a	-0.16861177	-0.487078475	-0.285722941	-0.100061338
ENSDART00000154212	BX323824.1	0.386508588	0.636151192	0.398585293	0.423381311
ENSDART00000154217	tapbpl	-0.790387635	-1.110046917	-0.234167162	0.243016032
ENSDART00000154228	sgip1a	-0.210460002	-0.370824353	-0.364544583	-0.177769156
ENSDART00000154238	AL928908.3	-0.284715118	-0.465774511	-0.246230328	-0.209644064
ENSDART00000154253	VAMP1 (1 of many)	-0.590038106	-0.991557836	-0.153916319	0.292977881
ENSDART00000154256	gabbr1a	-0.701390177	-1.072254559	-0.049084095	0.380533583
ENSDART00000154282	CT737162.2	2.40869789	3.049681827	2.839672212	2.555397461
ENSDART00000154295	RPS17 (1 of many)	0.397556507	0.446477566	0.193480989	0.136840294
ENSDART00000154307	hsp90aa1.2	-0.273233427	-0.388821318	-0.732231951	-0.738083556
ENSDART00000154314	prph	1.502464936	3.204214666	3.264208069	2.453842104
ENSDART00000154316	birc6-as2	-0.437572334	-0.469057905	-0.275420253	-0.034926922
ENSDART00000154321	BX908750.1	0.337703917	0.710327992	0.54373662	0.188876538
ENSDART00000154324	CR848791.3	3.13610848	2.257927159	1.961382093	1.531269093

ENSDART00000154329	plppr3b	-0.697577837	-0.505300644	-0.575363423	-0.078498654
ENSDART00000154333	camkva	-0.731914874	-0.620456244	-0.460647205	-0.026116956
ENSDART00000154359	ppil1	0.446724137	0.384000004	0.201188176	-0.044104474
ENSDART00000154363	chst7	-0.08877204	-0.106063572	-0.492144158	-0.182223467
ENSDART00000154375	CR812464.1	-0.471069407	-0.288977412	-0.485957331	-0.299311124
ENSDART00000154377	HIST1H2BM (1 of many)	0.871205458	1.144896653	0.89956001	0.544514919
ENSDART00000154385	nhsl1a	-0.136848286	-0.0387183	-0.42116689	-0.266423729
ENSDART00000154393	helz2	-0.348999688	-0.710690348	2.383802665	-0.188541475
ENSDART00000154400	tmem121a	-0.218470186	-0.361690646	-0.304266051	-0.077189842
ENSDART00000154405	rac1b	0.253264939	0.144566871	0.063714363	0.127687084
ENSDART00000154418	relb	-0.043359819	0.083083005	0.26390054	-0.071618881
ENSDART00000154429	gnai2b	-0.171950228	-0.218087414	0.308490155	0.376283024
ENSDART00000154434	frmd4ba	0.373950657	0.549595304	0.324851185	-0.019839546
ENSDART00000154437	dyrk1ab	-0.405599591	-0.293606249	-0.153631672	-0.200586701
ENSDART00000154438	ltb4r	0.406290343	0.081638761	0.101793584	-0.225121243
ENSDART00000154460	FP243382.1	2.715231463	2.420271029	2.173535574	1.735635064
ENSDART00000154495	si:ch73-195i19.3	0.285619034	0.153921873	0.078118908	0.066673852
ENSDART00000154501	cntnap1	-0.715173489	-0.84270145	-0.359147123	0.122603648
ENSDART00000154503	syncripl	0.163572635	0.201195284	0.244411469	0.118655043
ENSDART00000154519	nrxn3a	-0.161675514	-0.445079711	-0.329497924	-0.050382407
ENSDART00000154539	slc17a7b	-0.194590302	-0.417792811	-0.270877884	0.095165091
ENSDART00000154543	prelid3a	0.578261204	0.829307037	0.712329147	0.291766611
ENSDART00000154573	prob1	1.935993422	2.091368151	1.752154811	1.442205958
ENSDART00000154627	eogt	-0.136366673	-0.382886458	-0.560103122	-0.203176718
ENSDART00000154628	fam101b	0.450957894	0.982590486	0.845377619	-0.490650185
ENSDART00000154633	chst15	-0.234696766	-0.27064598	-0.214752082	-0.155581795
ENSDART00000154638	kif26ab	2.382609513	1.668373417	1.704066313	1.906262158
ENSDART00000154652	irgq1	0.050480218	0.069496236	1.163101864	0.075649547
ENSDART00000154667	SORCS3	-0.864119649	-0.84034251	-0.370097899	0.056888282
ENSDART00000154679	ypel2b	-0.575722509	-0.671947843	-0.397167717	-0.03983803
ENSDART00000154682	CR847531.1	-0.541034447	-0.676553431	-0.554824044	-0.149530967
ENSDART00000154719	hip1	0.155954348	0.357624902	0.536540352	0.421858349
ENSDART00000154726	MYADM (1 of many)	1.393641215	1.121324137	1.000193747	0.38217935
ENSDART00000154730	tsc22d1	0.125146937	0.071380598	0.346511952	0.250641998
ENSDART00000154732	ptprt	-0.128635692	-0.441586772	-0.224155866	-0.045463061
ENSDART00000154748	sv2bb	-0.593359268	-0.537099772	-0.285465428	0.087161334
ENSDART00000154754	grik1b	-0.541985174	-0.220416154	-0.114773141	-0.128231092
ENSDART00000154760	CU302436.3	-0.931829111	-0.859047685	-0.631190961	-0.184130931
ENSDART00000154778	map1ab	-0.607759134	-0.711103734	-0.339973576	-0.303457691
ENSDART00000154783	BX511311.5	-0.420893047	-0.406058694	-0.342057398	-0.24848029
ENSDART00000154801	itgae.2	0.922252153	0.655667188	0.379341087	-0.144710616
ENSDART00000154809	CU467861.1	0.653813027	0.40784225	0.192026805	0.151191997
ENSDART00000154811	shank1	-0.390024258	-0.720748027	-0.113172669	0.256359072
ENSDART00000154846	CR381618.1	0.115176903	0.900181702	1.102625706	0.253393379
ENSDART00000154851	zgc:153405	0.317950752	1.076463864	0.674378682	0.394781222
ENSDART00000154860	CU682780.1	-0.134139035	-0.255412373	-0.351732915	-0.064350576
ENSDART00000154869	cacna1ha	-0.561980799	-0.554587419	-0.200655166	0.115415453
ENSDART00000154880	arhgef33	0.636994605	0.687196577	0.646579762	0.300121216
ENSDART00000154885	si:ch211-167j9.4	1.022951831	0.721088624	0.864353518	0.647190994
ENSDART00000154897	srsf10a	-0.118782385	-0.22214156	-0.484269556	-0.079154163
ENSDART00000154916	p4htm	-0.322717782	-0.242652877	-0.246255125	-0.141036056

ENSDART00000154917	ciartb	-0.07665894	-0.17887759	-0.516654864	-0.219843882
ENSDART00000154933	si:dkey-13p1.4	-2.565488296	-3.472379023	-0.405040918	0.505631615
ENSDART00000154945	si:dkey-7j14.6	0.563979024	0.172625977	0.010511104	-0.696238166
ENSDART00000154961	btbd10b	-0.035347687	0.05017631	0.315377826	0.177817418
ENSDART00000154978	si:ch211-284f22.3	0.111486547	0.17394927	0.442535968	0.352663532
ENSDART00000154985	ddx3b	-0.099315614	-0.086363684	0.465639279	0.388612698
ENSDART00000154991	b4galnt1b	-0.249570746	-0.126095261	0.190575038	0.316262718
ENSDART00000155033	srpk2	-0.213188431	0.160547857	0.269227711	0.471530406
ENSDART00000155038	si:dkeyp-100a5.4	-0.19105809	-0.704501455	-0.315530312	-0.360932845
ENSDART00000155051	mych	1.81452306	1.847457308	1.423042212	0.692601562
ENSDART00000155057	capns1b	0.743122931	1.233401578	0.848245138	0.67484109
ENSDART00000155066	atf5b	0.303223783	0.449779577	0.245269838	-0.118029433
ENSDART00000155078	CU466278.1	-0.496260032	-0.275828398	-0.937323619	-0.494833793
ENSDART00000155088	ankrd33ab	-0.091123111	-0.280595773	-2.122583018	-0.908625438
ENSDART00000155094	si:ch211-286c4.6	0.931010056	1.14507062	1.165043802	1.805632584
ENSDART00000155108	si:ch211-153l6.6	-0.396073461	-0.324628235	-0.330118035	-0.333690214
ENSDART00000155116	im:7152348	-0.17038115	-0.180140684	-0.567414199	-0.647722466
ENSDART00000155128	meis3	1.719560674	1.869294835	2.789896441	2.118152659
ENSDART00000155178	man1b1b	-0.197923135	-0.298790741	-0.488552512	-0.408314648
ENSDART00000155184	lcorl	-0.199231763	-0.444480785	-0.032192793	-0.024389475
ENSDART00000155188	ccnjl	0.284030133	0.391162848	0.492680219	0.242842213
ENSDART00000155190	maptb	1.096239137	1.804532446	2.232737802	1.717568221
ENSDART00000155203	ttbk1b	-0.886183357	-0.409260354	0.093887166	0.234308172
ENSDART00000155212	msi2b	-0.193481816	-0.280091063	-0.40298969	-0.141186615
ENSDART00000155227	si:dkey-248g15.2	-0.512231666	-0.490597761	-0.50523481	-0.410257249
ENSDART00000155229	rxfp3.3b	-0.899836922	-0.961252446	-0.631060044	-0.38629797
ENSDART00000155243	BX571762.2	-0.58394173	-0.66558831	-0.494788274	-0.139509269
ENSDART00000155256	VWA5A (1 of many)	0.207030506	0.487713239	0.641785484	0.122484219
ENSDART00000155262	nek1	-0.289218155	-0.162294169	0.032473831	0.21974529
ENSDART00000155267	si:ch211-149e23.4	0.305598533	0.378888393	0.64366915	0.422691793
ENSDART00000155268	pik3cb	0.672881324	0.55380807	0.246761254	0.040522215
ENSDART00000155273	FP102016.1	0.672121642	0.238521805	0.194526989	0.041260629
ENSDART00000155280	GDPGP1	0.576074599	0.656458452	0.674683139	0.299654628
ENSDART00000155306	CR925728.1	1.81197689	3.280738703	3.024711765	2.003163607
ENSDART00000155313	hspa4l	-0.318813042	-0.280574999	-0.269018374	0.033454147
ENSDART00000155320	atxn7l1	-0.301256075	-0.435278991	-0.214418032	-0.09758885
ENSDART00000155324	pdpk1a	-0.082713691	-0.135052421	-0.317218269	-0.180842854
ENSDART00000155327	ip6k2b	0.635904907	0.734729077	0.476402387	0.497162385
ENSDART00000155344	VWA5A (1 of many)	0.009320645	0.148549206	0.649252702	0.190646512
ENSDART00000155346	tbc1d24	-0.444989153	-0.328605075	-0.340554332	-0.035933185
ENSDART00000155368	si:ch73-95l15.3	1.516126674	2.634881995	2.849448463	2.215584039
ENSDART00000155397	soat2	-0.618559497	-0.31486995	-0.194806138	-0.125519338
ENSDART00000155427	reep6	0.890805905	0.284631883	0.369468446	0.541597773
ENSDART00000155440	eif3ba	0.265595321	0.439061322	0.461693724	0.229141991
ENSDART00000155445	si:dkey-7j14.6	4.555762034	3.357060696	4.122496376	1.366436667
ENSDART00000155456	gria4a	-0.214950555	-0.367205609	-0.037830298	0.237179158
ENSDART00000155458	ubap1la	-0.102679722	-0.709773263	-0.512828077	-0.333708251
ENSDART00000155464	ftr73	0.436406361	0.364310996	2.049125852	0.40110848
ENSDART00000155472	nudt8	-0.430833003	-0.309991274	-0.295400218	-0.125942696
ENSDART00000155480	ptprnb	-0.501074747	-0.734898939	-0.55867421	-0.198202123
ENSDART00000155494	CR384099.1	-0.702495416	-0.755760921	-0.229440848	0.245420937

ENSDART00000155496	gal3st2	-0.347287801	-0.358994611	-0.502209455	0.011977936
ENSDART00000155499	CU571319.1	0.914674175	1.459254054	0.740447631	0.310619615
ENSDART00000155502	sez6b	-0.382545228	-0.653854879	-0.149982953	0.168281376
ENSDART00000155519	si:ch211-139d20.3	0.778333948	0.572606648	0.403486279	0.486914049
ENSDART00000155525	si:rp71-45k5.4	0.233215264	0.362150294	0.25819678	0.006995576
ENSDART00000155537	VWA5A (1 of many)	1.206080865	0.987004598	1.27402251	0.722325665
ENSDART00000155539	CR786577.1	-0.296712547	-0.710798753	-0.341045728	0.032686751
ENSDART00000155553	CR931780.1	1.845884645	1.39773807	0.761518659	0.588325484
ENSDART00000155554	frs3	-0.288569277	-0.348098053	0.092392931	0.139305366
ENSDART00000155563	abca5	-0.240041791	-0.35443471	-0.098648169	0.068480985
ENSDART00000155580	ccdc32	-0.137851793	-0.182759184	-0.331717766	-0.143672128
ENSDART00000155592	pcdh17	-0.283490769	-0.422406754	-0.268768577	0.186259856
ENSDART00000155595	zgc:174263	-0.246339018	-0.142647436	-0.250696029	-0.144411513
ENSDART00000155610	tdg.2	0.198987795	0.279928785	0.39077816	0.364649362
ENSDART00000155658	rapgefl1	-0.418362292	-0.397338899	0.000660896	-0.001869225
ENSDART00000155666	znf576.1	-0.372721752	-0.23410815	-0.134923201	-0.013577883
ENSDART00000155683	CU855758.2	-0.197771803	-0.264936874	-0.616643588	-0.19367265
ENSDART00000155723	si:ch211-24o10.6	-0.255651916	0.580162906	3.993132482	0.654326546
ENSDART00000155751	nuak1b	0.785416985	1.722603254	1.683891996	1.302083807
ENSDART00000155755	CU469362.1	0.467928162	0.778645014	0.342312822	-0.038725012
ENSDART00000155757	si:dkey-16p21.8	-0.383053569	-0.315569666	-0.222717784	0.028079211
ENSDART00000155764	slc25a18	-0.081957182	-0.293577862	-0.279616954	-0.236786045
ENSDART00000155786	adgrl1a	-0.190147203	-0.479741899	-0.292479007	0.056973675
ENSDART00000155808	SRMS	-0.518504687	-0.21831974	0.182924623	0.347888554
ENSDART00000155817	spsb3b	0.397854128	-0.261249457	0.795238945	1.076488289
ENSDART00000155821	kcnt2	-1.064051569	-1.353278896	-1.209879321	-0.444718597
ENSDART00000155838	luzp1	-0.38633981	-0.211709262	-0.146984907	-0.314685818
ENSDART00000155839	CR759918.1	-0.263859971	-0.262088445	-0.379170984	-0.118091172
ENSDART00000155840	nyap2a	-0.526663005	-0.654892136	0.27374848	0.170536216
ENSDART00000155843	cntnap2a	-0.263075507	-0.343897302	0.676718804	0.99202798
ENSDART00000155844	nr1d4b	0.850133899	0.016728696	-0.171313869	0.165802614
ENSDART00000155848	CU929046.2	-0.462340311	-0.419607593	-0.082173547	-0.018364502
ENSDART00000155853	gprc5ba	-0.568480744	-0.680176078	-0.476445621	-0.089942946
ENSDART00000155859	larp4ab	-0.193577441	-0.330790279	-0.534105504	-0.201385609
ENSDART00000155864	CU929070.1	1.274116455	1.096698631	0.870265354	0.298589514
ENSDART00000155865	tmtops2a	-0.317973372	-0.37404155	-0.204729003	-0.178609254
ENSDART00000155866	zbtb16b	-0.434457287	-0.595844526	0.044569606	0.015262284
ENSDART00000155902	frya	-0.208619052	-0.429125005	-0.269458135	-0.004719121
ENSDART00000155935	clstn3	-0.324952015	-0.256429293	0.203826088	0.321978764
ENSDART00000155947		-0.00158363	0.212586024	0.278540905	0.169021899
ENSDART00000155949	grm8b	-0.426657348	-0.505637682	-0.163630603	0.069819734
ENSDART00000155956	ssx2ipa	-0.036908945	-0.252732501	-0.388773092	-0.156731785
ENSDART00000155973	si:ch211-282b22.1	0.150167669	-0.093228424	-0.31353999	-0.332567216
ENSDART00000155992	tjp1b	-0.305505503	-0.430213518	-0.153805225	0.023043416
ENSDART00000156002	tmem206	0.793441462	0.286165316	0.167291274	-0.130249565
ENSDART00000156008	adcy1b	-0.126277791	-0.148680925	-0.963041725	-0.495906031
ENSDART00000156012	si:dkey-35i13.1	-1.182557557	-0.949918392	-0.338171836	-0.056213778
ENSDART00000156019	phlpp1	-0.378155747	-0.257656641	-0.150573304	-0.126584813
ENSDART00000156034	VWA5A (1 of many)	1.04711946	1.090403168	1.260861512	0.694741705
ENSDART00000156038	epn2	0.165862475	0.434471463	0.333526121	0.113707323
ENSDART00000156051	dhx32b	-0.034036848	-0.386771049	-0.600165189	-0.131769991

ENSDART00000156062	calml4b	-0.12658352	-0.200610116	-0.571249563	-0.361192591
ENSDART00000156167	si:ch73-182a11.2	0.438099394	0.613388756	0.677790276	0.474745988
ENSDART00000156169	ube2o	-0.089939623	-0.17956897	-0.346977584	-0.218736801
ENSDART00000156179	sema7a	-0.273960356	-0.559537035	-0.479585038	-0.478067901
ENSDART00000156211	adcyap1r1b	-0.120918148	-0.186017	-0.540439336	-0.277725276
ENSDART00000156224	dhx33	0.472474316	0.556602191	0.639276654	0.492917502
ENSDART00000156247	CU468915.1	-0.147323171	-0.162953088	-0.662599642	-0.420048082
ENSDART00000156251	ehbp1	0.342339175	0.353753636	0.38894242	0.04618254
ENSDART00000156255	ago1	0.402935641	0.576022371	0.656640241	0.478601065
ENSDART00000156256	angpt4	-0.447666401	-0.531623063	-0.584960614	-0.899124282
ENSDART00000156257	wnk1a	-0.012583832	0.164942132	0.328941429	0.287930853
ENSDART00000156258	pfkpb	-0.354296164	-0.444812712	-0.357490287	-0.175370256
ENSDART00000156280	si:ch211-195b15.7	-0.343611395	-0.333351498	-0.20091334	-0.160599804
ENSDART00000156295	myhb	-2.03199835	4.211906798	4.339398917	2.940685436
ENSDART00000156306	ptgir	0.787352004	0.130407388	0.033259328	-0.478389399
ENSDART00000156340	si:ch211-167b20.8	-0.291865267	-0.289158159	-0.304691219	-0.111151074
ENSDART00000156341	fam117ba	-0.500099563	-0.265235629	0.202099115	0.368226992
ENSDART00000156351	zfat	-0.386268069	-0.362611932	-0.387180541	-0.301395116
ENSDART00000156365	BX248121.1	-0.069755444	-0.2828198	-0.35442143	-0.173325265
ENSDART00000156375	kcnq2b	-0.284091354	-0.197722227	0.27148365	0.512045099
ENSDART00000156380	CR848757.1	-0.431298815	-0.377014166	0.404962464	0.865655337
ENSDART00000156393	AL772314.1	-0.272422966	-0.157258449	0.218420805	0.368190657
ENSDART00000156403	gfpt1	0.402370916	0.732684838	0.696897001	0.375214811
ENSDART00000156411	fam98a	0.369288002	0.399859986	0.477248543	0.233728964
ENSDART00000156415	dock9b	0.187525252	0.556466903	0.665071238	0.341730571
ENSDART00000156418	itpk1b	-0.567310725	-0.6808899	-0.20043499	-0.215676742
ENSDART00000156428	hephl1a	0.427057323	0.215282086	0.277986668	-0.033310258
ENSDART00000156437	BX248501.1	-0.044614101	-0.492506227	-0.236687215	-0.012438349
ENSDART00000156438	tmem138	-0.131418974	-0.291816206	-0.603920899	-0.471900931
ENSDART00000156447	ap2a1	-0.168026562	-0.400633541	-0.149036642	-0.01932092
ENSDART00000156450	wnk4b	0.377998578	0.587708612	1.132797895	0.554283794
ENSDART00000156473	CR627483.1	-0.446874417	-0.944979531	-0.551426346	-0.288797786
ENSDART00000156492	tiam2a	-0.024981814	-0.236489206	-0.397107093	-0.120407246
ENSDART00000156499	prkcdb	0.617876805	0.366145269	0.057590279	-0.230105501
ENSDART00000156509	fam150bb	-0.200699582	-0.03489316	-0.310837396	-0.503245808
ENSDART00000156516	tmem74b	-0.490393884	-0.38109679	-0.101020414	0.194211555
ENSDART00000156517	GSE1	-0.323310165	-0.383945273	-0.036135566	-0.046797605
ENSDART00000156527	crhr1	-0.121975919	-0.246139891	-0.376184792	-0.120567191
ENSDART00000156535	vav3b	-0.155549402	0.298871549	0.871724785	0.959064908
ENSDART00000156546	plcxd1	0.33889602	0.161133476	0.572020307	0.368311625
ENSDART00000156568	CR387996.1	-0.565799723	-0.534585499	-0.855400153	-0.72349809
ENSDART00000156570	slc6a17	-0.448593137	-0.394227085	-0.032159276	0.074756396
ENSDART00000156574	si:ch211-270g19.5	-0.160521866	-0.305818991	-0.352315858	-0.223463705
ENSDART00000156575	dicp3.3	1.874714519	0.882148407	0.706359774	-0.079481712
ENSDART00000156577	si:ch211-130m23.5	-0.007378589	0.400588111	0.725075783	0.606240178
ENSDART00000156580	hspa12a	-0.664613601	-0.584311718	0.126225265	0.380065995
ENSDART00000156584	msi2b	-0.183156983	-0.402159154	-0.705145609	-0.389308076
ENSDART00000156607	CT737162.1	0.778225783	0.597499699	0.53495134	0.647915122
ENSDART00000156608	ap2a1	-0.16265507	-0.313644183	-0.332880329	-0.023511806
ENSDART00000156613	CR848683.1	-0.419259508	-0.300093589	-0.534359803	-0.243066507
ENSDART00000156615	amigo3	-0.174881747	-0.348495554	-0.328942663	0.002458748

ENSDART00000156668	BX569793.1	-0.298302127	-0.566890135	-0.349641739	-0.271668844
ENSDART00000156690	ftr88	0.478641753	0.356185618	1.950902073	0.248066147
ENSDART00000156705	si:dkeyp-72g9.4	-0.743143803	-0.697928004	-0.454613917	-0.077239927
ENSDART00000156741	BX663503.2	-0.41351136	-0.477289266	-0.358517546	-0.050787842
ENSDART00000156757	CU633486.1	-0.302968456	-0.206059	-0.531920994	-0.165463059
ENSDART00000156760	grik4	-0.347451056	-0.634709172	-0.373537369	-0.211041818
ENSDART00000156765	soga3a	0.515035881	0.806381678	1.137120698	0.812578505
ENSDART00000156774	dph6	0.485952337	0.860504803	0.608054536	0.313680056
ENSDART00000156792	kif1aa	-0.016449727	0.304055121	0.524314954	0.462526752
ENSDART00000156795	zcchc8	0.52372258	0.428501893	0.558201403	0.582679631
ENSDART00000156806	rps6kl1	0.03495508	0.325476415	0.37135553	0.249571068
ENSDART00000156820	si:dkey-7j14.5	-0.971869146	-0.757334904	0.08249331	0.800607437
ENSDART00000156822	cdip1	-0.168452851	-0.279910749	-0.292336463	-0.062208262
ENSDART00000156831	si:dkeyp-47f9.4	-0.215799622	-0.290204625	-0.428110244	-0.249285093
ENSDART00000156832	kif13ba	0.259733256	0.195728022	0.255457371	0.044129384
ENSDART00000156853	si:ch211-284e13.14	0.249769449	0.06508988	0.403860054	0.279589235
ENSDART00000156864	CR376745.2	-0.465037351	-0.777375633	-0.748299595	-0.606506499
ENSDART00000156877	prss56	-1.154037343	-0.806544973	-0.688753663	-0.465249106
ENSDART00000156885	EML5	-0.115526296	-0.315705485	-0.106202597	0.094534493
ENSDART00000156913	BX927184.1	1.282107066	1.77927465	1.802027966	1.376990986
ENSDART00000156918	si:dkey-16p21.8	-0.278111877	-0.271868386	-0.501824009	-0.172182623
ENSDART00000156923	plekhd1	-0.640317774	-0.396125765	-0.428766409	-0.43244988
ENSDART00000156928	nrxn3a	0.020925857	-0.341808434	-0.239366202	0.052782357
ENSDART00000156935	pbx4	0.960146191	0.964981474	3.716226941	3.606625876
ENSDART00000156965	osbpl5	-0.452639629	-0.228065482	0.296781507	0.346469129
ENSDART00000156967	itcha	-0.048958922	-0.233534262	-0.334119802	-0.057332431
ENSDART00000156970	si:ch73-265h17.5	1.250341969	1.481314496	1.76830523	0.8438116
ENSDART00000156972	slc12a7a	0.533547881	0.750475895	0.543271513	0.05077876
ENSDART00000156982	si:ch211-195b15.8	-0.203622983	-0.158924419	-0.050797883	0.351311882
ENSDART00000156995	sorl1	-0.129978292	-0.268802979	-0.097227509	0.102143372
ENSDART00000157018	stmn3	-1.008205118	-1.406964646	-0.116902659	0.621837811
ENSDART00000157043	rtn4r	-0.358605066	-0.3593825	-0.41712234	-0.136427457
ENSDART00000157045	CU861453.1	-0.184297698	-0.238197074	-0.240198755	-0.095792528
ENSDART00000157050	thoc5	-3.378273157	-0.016679798	0.227931101	0.074505779
ENSDART00000157058	si:dkey-276j7.1	-0.114061291	0.35744712	0.794710779	0.605587625
ENSDART00000157066	ppp1r16b	-0.047627869	-0.115324195	-0.408403631	-0.014573035
ENSDART00000157067	BX510324.1	-0.110028536	-3.040813753	-0.681076994	-0.348601244
ENSDART00000157116	cyp8b3	-4.272126467	-1.470280769	0.08019487	-1.373424813
ENSDART00000157122	elmsan1b	0.072375655	0.138875939	-0.375191445	-0.292342976
ENSDART00000157124	CT033825.1	-0.560424235	-0.809708864	-0.680777647	-0.144372819
ENSDART00000157125	vsnl1b	-0.832964446	-0.803942924	-0.29707501	0.109485311
ENSDART00000157127	lmcd1	1.160892936	2.271204052	2.063825472	1.282095985
ENSDART00000157129	slc12a5a	-0.482400538	-0.74419089	-0.401518291	-0.07447909
ENSDART00000157137	HIST1H2BM (1 of many)	0.980872833	1.453339934	1.015669985	0.930663607
ENSDART00000157139	tmbim1a	0.963253296	1.499596205	1.306783479	0.549513539
ENSDART00000157171	si:ch211-168d23.3	-0.276296251	-0.552181073	-0.115547916	-0.061680907
ENSDART00000157190	ostn	0.371505716	0.628806712	0.229636498	0.167623814
ENSDART00000157195	ncam2	-0.590792178	-0.719154845	-0.40764277	0.002619526
ENSDART00000157199	mkl2b	0.238133435	0.28120222	0.324920076	0.149313819
ENSDART00000157237	si:dkey-56f14.7	-0.377561364	-0.203140811	-0.202521092	-0.065412084
ENSDART00000157258	BX004991.1	-0.288834986	-0.59132286	-0.471054454	-0.432666578

ENSDART00000157264	RAP1GAP (1 of many)	-0.397821605	-0.344805586	-0.195900343	-0.114570879
ENSDART00000157265	trim13	-0.831738752	-0.32341984	-0.102064486	-0.071474505
ENSDART00000157302	si:dkey-7j14.5	-0.795010515	-0.583416344	0.327377123	0.951280694
ENSDART00000157330	necab3	-0.311593638	-0.396628677	-0.274835563	-0.023399638
ENSDART00000157338	gstz1	-0.391223456	-0.14748942	-0.590184545	-0.319150237
ENSDART00000157350	CT573344.1	0.592860846	1.013860007	1.275401288	0.850112465
ENSDART00000157378	hdac5	-0.360172826	-0.414916304	-0.488703112	-0.152605137
ENSDART00000157407	nacad	-0.349281215	0.532726398	0.824860187	0.457523633
ENSDART00000157414	plxna4	-0.256858488	-0.391015088	-0.285048615	-0.106219383
ENSDART00000157415	acvr2ab	-0.152487638	-0.319222405	-0.146638904	-0.027512421
ENSDART00000157416	si:dkey-23n7.10	0.164308792	0.543803953	1.528490128	1.278412625
ENSDART00000157428	pik3r3b	-0.522784766	-0.44851499	-0.152608244	0.028231585
ENSDART00000157437	park2	-0.135617767	-0.306295495	-0.445890787	-0.195890902
ENSDART00000157441	filip1l	1.025025125	2.361056221	1.959563135	1.214723721
ENSDART00000157449	clstn2	-0.339354151	-0.60478304	0.06475081	0.303731917
ENSDART00000157454	ncam1a	-0.363879899	-0.114400367	0.050687773	0.064981174
ENSDART00000157464	adkb	0.004906369	0.081747651	0.332869381	0.17633498
ENSDART00000157475	coro7	0.174942149	0.053392758	0.483358888	0.70362148
ENSDART00000157479	nr4a1	-1.499367308	-4.285014891	-2.179617587	-2.01791928
ENSDART00000157487	tfec	0.534318298	0.423012313	0.205933849	-0.313187686
ENSDART00000157515	FO704641.1	-0.135830256	-0.41280018	-0.451790561	-0.015210245
ENSDART00000157518	hspa12a	-0.767789618	-0.701014156	0.093464144	0.364648116
ENSDART00000157535	unc13c	-0.230182256	-0.566319347	-0.291866986	-0.102048139
ENSDART00000157538	npm3	0.706470742	0.616423608	0.411598819	-0.103962898
ENSDART00000157546		-4.297830881	-2.011048515	-0.106888147	-0.169461052
ENSDART00000157559	si:dkey-183n20.15	2.324560868	2.818869593	3.029863009	3.039600704
ENSDART00000157563	noctb	-0.197344301	-0.486083216	-0.268092319	0.128030661
ENSDART00000157570	asmt	-0.467907647	-0.512776687	-0.801096178	-0.257600065
ENSDART00000157581	dctn2	0.246810516	0.408963324	0.394068831	0.222953154
ENSDART00000157591	CT583642.1	0.946563077	0.733732925	0.580155557	-0.143517637
ENSDART00000157597	Metazoa_SRP	3.784983496	4.068975573	4.489534416	3.689887905
ENSDART00000157604	si:dkey-19c16.12	0.518457933	0.406892634	0.690003002	0.445775315
ENSDART00000157625	mapkapk3	0.008165693	-0.129188005	-0.419061818	-0.281468347
ENSDART00000157636	Metazoa_SRP	2.626880551	2.386640401	1.529298522	0.72995056
ENSDART00000157643	si:dkey-88e18.8	0.426205845	0.069942358	0.168991341	0.195596151
ENSDART00000157659	camta1b	-0.574265168	-0.690891494	-0.316064045	-0.339000781
ENSDART00000157661	pdlim5a	-0.256112858	-0.374018749	-0.32171501	-0.127659508
ENSDART00000157663	reln	-0.012850615	-0.065342924	0.75286937	0.74278543
ENSDART00000157678	nop53	0.221649254	0.357738319	0.319808875	0.087032288
ENSDART00000157682	kat2a	0.094563424	0.264392397	0.483214757	0.33498107
ENSDART00000157697	sec22bb	-0.209339329	-0.24617122	-0.297558263	-0.212223069
ENSDART00000157711	ap2m1b	-0.212778909	-0.504780227	-0.48236783	-0.089136332
ENSDART00000157726	fam20a	0.506263561	0.217850689	-0.118035628	-0.139373167
ENSDART00000157728	gdap1l1	-0.078507744	0.185505427	0.335339207	0.32984222
ENSDART00000157735	cdc42ep4a	0.06354285	0.460607642	0.576300047	0.396817817
ENSDART00000157755	cds1	-0.167833858	-0.281185983	-0.514312915	-0.176231692
ENSDART00000157769	pcdh1gc5	-0.403524411	-0.226721593	0.040583925	0.029815654
ENSDART00000157771	sumo2b	0.072691072	0.240484774	0.205807834	0.084424554
ENSDART00000157775	CR381700.1	0.888966397	0.292933465	0.058270274	0.51311949
ENSDART00000157788	CABZ01031894.1	-0.75935498	-0.746140313	-0.275189537	-0.221209558
ENSDART00000157815	elmo1	-0.27246831	-0.132899842	-0.049572106	0.087486375

ENSDART00000157817	lrrfip1b	-0.412751624	0.086992217	0.168101768	0.241775802
ENSDART00000157820	mfsd6a	0.403998194	0.775291086	0.690391907	-0.020489453
ENSDART00000157824	slc6a15	0.370043272	0.932121448	1.025205824	0.470213629
ENSDART00000157826	mpp1	0.283246867	0.228442011	0.159131554	0.043281733
ENSDART00000157852	crtac1b	-0.401370563	-0.59197436	-0.358347223	-0.176621049
ENSDART00000157855	ebf3a	-0.765871102	-0.436598509	0.580406091	0.942511549
ENSDART00000157871	mgst3a	0.496607086	0.574379254	0.622130807	0.504216816
ENSDART00000157873	hacd1	0.841608261	0.46729923	0.326975211	0.16703571
ENSDART00000157877	CABZ01092282.1	-0.281901821	-0.460522749	0.165109465	0.263636132
ENSDART00000157890	tcf7l1b	-4.555123239	-4.673271453	-1.829433189	-0.732249154
ENSDART00000157894	apaf1	0.69901776	0.613857472	0.804406673	0.575412751
ENSDART00000157899	dad1	0.443099375	0.390242441	0.192203328	-0.014224626
ENSDART00000157900	chmp1b	0.92760321	0.512046614	0.591242771	0.272231533
ENSDART00000157907	rpl34	0.216382556	0.50724745	0.251806906	0.167915072
ENSDART00000157919	med24	0.290079054	0.266724064	0.247847379	0.11623123
ENSDART00000157920	BX664622.3	0.309964523	0.2387983	0.211520656	0.43648414
ENSDART00000157937	supt3h	0.425159053	0.217206475	0.313741902	-0.075091294
ENSDART00000157943	TENM2	-0.301363214	-0.846881931	-0.344978545	0.012452
ENSDART00000157944	CR848788.1	0.572199002	0.285271614	0.143233659	0.192859067
ENSDART00000157958	nav3	0.289857115	0.341415382	0.725222646	0.845169813
ENSDART00000157976	hif1al2	0.549135047	0.585055165	1.624530088	0.686809008
ENSDART00000157979	plppr5b	-0.141986763	-0.474240998	-0.437275413	-0.217953336
ENSDART00000157998	cngb3.1	0.075294731	-0.249736043	-0.588641013	-0.198039383
ENSDART00000158016	flnca	5.435651542	4.853809922	4.737861308	3.940836376
ENSDART00000158017	GUCA1A (1 of many)	-1.245659292	-0.578230856	-1.064205686	-1.242533296
ENSDART00000158021		-0.273860764	-0.2373593	-0.233720455	-0.186840477
ENSDART00000158036	cript	-0.080102736	-0.07683769	-0.280131139	-0.196435314
ENSDART00000158042	mdga2a	-0.261819905	-0.306879785	-0.151540127	0.022043669
ENSDART00000158052	rnf130	-0.205769239	0.102328211	0.30768262	0.182366646
ENSDART00000158055	kcnh6a	-0.288242124	-0.323669014	-0.559331748	-0.259833446
ENSDART00000158072	gpc2	0.147140386	0.761040446	1.023252339	0.612693915
ENSDART00000158077	arr3b	-0.13602706	-0.148118881	-0.527427286	-0.215489656
ENSDART00000158098	gpr132b	-0.335128962	-0.097182083	0.890467313	0.848088338
ENSDART00000158123	magi1b	-0.699965885	-0.960349882	-0.3166938	0.663491004
ENSDART00000158147	calr3b	0.659493637	0.343153236	0.419640601	0.12486596
ENSDART00000158154	dlg2	-0.258379843	-0.32848175	-0.404306744	-0.064632311
ENSDART00000158169	cacna2d2a	-0.273970943	-0.601526504	-0.288906256	-0.138856816
ENSDART00000158183		-0.077181373	-0.541229915	-0.5215371	-0.066571696
ENSDART00000158202	CABZ01078449.1	-0.388078647	-0.185889564	-2.923902226	0.109460724
ENSDART00000158204	ntrk3a	-0.448230073	-0.669954401	-0.450935682	-0.114414424
ENSDART00000158212	zgc:92161	0.66498591	0.968065208	0.600651955	-0.278084019
ENSDART00000158213	akap12b	-1.00724688	-1.146388756	-0.599808188	0.009805796
ENSDART00000158245	si:ch73-1a9.3	-0.234948586	-0.211979293	-0.351222857	-0.141776696
ENSDART00000158259	lrtm1	-0.264679565	-0.523836662	-0.478235847	-0.361630227
ENSDART00000158260	c1galt1a	0.184684125	0.138397385	-0.286164045	-0.509177074
ENSDART00000158272	dclk1b	0.134331666	0.528656936	0.571389243	0.299537528
ENSDART00000158289	taok1b	-0.136932712	-0.168799291	-0.390684145	-0.122927658
ENSDART00000158290	rpl24	0.677207663	0.41367136	0.306049933	0.275882655
ENSDART00000158294	ppp2r2cb	-0.427479199	-0.698226267	-0.466339082	-0.260646218
ENSDART00000158301	CR749168.4	-0.284512048	-0.514337485	-0.519239658	-0.283197555
ENSDART00000158310	sat2b	-0.409697091	-0.182271077	-0.610869777	-0.543847015

ENSDART00000158311	klhl7	0.128707755	0.106332897	0.351073118	0.278667069
ENSDART00000158338	CNNM1	-0.362878072	-0.490740729	-0.646651234	-0.29213891
ENSDART00000158344	apbb2b	-0.312721901	-0.306004033	0.116860588	0.307202209
ENSDART00000158348	zmiz1b	0.167782576	0.103403196	0.408056208	0.722421278
ENSDART00000158354	adarb2	-0.457769313	-0.257870403	-0.020766954	-0.013045223
ENSDART00000158355	bsk146	-0.973617487	-0.7163723	-0.116416923	0.102394026
ENSDART00000158358	stk40	-0.305579471	-0.394234001	-0.179882029	-0.355392322
ENSDART00000158364	si:dkey-112e17.1	-0.140789793	-0.187774176	-0.188421319	-0.472049113
ENSDART00000158373	epc1b	0.106120055	0.059882061	0.624967071	0.299679159
ENSDART00000158380	si:dkey-35m8.1	-0.462314219	-0.595244504	0.018350172	0.199682571
ENSDART00000158395	dclk2a	-0.152411223	-0.291700953	-0.710292221	-0.218291454
ENSDART00000158429	tmem42b	-0.433937172	-0.428108528	-0.048957595	0.127616146
ENSDART00000158436	lypla1	0.03667991	-0.201169357	-0.480610097	-0.044568257
ENSDART00000158439	ece2b	-0.235035459	-0.256980434	-0.233206993	-0.167380402
ENSDART00000158440	arhgap12b	-0.039244537	-0.138433678	-0.355110347	-0.091493676
ENSDART00000158466	creb3l2	0.073267533	0.091531466	-0.07087733	-0.32121815
ENSDART00000158489	zgc:77058	-0.495737975	-0.228595493	0.141443601	0.440592752
ENSDART00000158500	atp8b2	-0.533001796	-0.553651375	-0.17260282	0.179842571
ENSDART00000158515		-0.601694587	-0.246037227	-0.439227399	-0.290329932
ENSDART00000158532	gnb2	-0.185937539	0.268112673	0.644184639	0.701859814
ENSDART00000158533	nup50	0.24455835	0.550142856	0.78129311	0.523300254
ENSDART00000158540	PRKCA (1 of many)	-0.338994629	-0.305121271	-0.237610526	-0.160203254
ENSDART00000158555	camsap2a	-0.296024961	-0.235576155	0.04836376	0.162312505
ENSDART00000158563	wu:fj29h11	0.059907274	-0.166860874	1.78519307	0.017403627
ENSDART00000158564	CU681842.1	-0.1327123	-0.276414972	-0.289623815	-0.090007903
ENSDART00000158581	spna2	0.480311119	0.377153274	0.267336877	-0.085977531
ENSDART00000158587	casd1	-0.21622109	-0.133405356	-0.572868553	-0.255662771
ENSDART00000158598	si:ch211-232b12.5	-0.304573383	0.212713124	0.452361247	0.038090441
ENSDART00000158618	cpne4b	-1.041271467	-0.75854166	0.438341768	1.045239509
ENSDART00000158646	dpp6a	-0.408023938	-0.433201282	-0.219888215	-0.008677852
ENSDART00000158671	sult4a1	-0.462609111	-0.368663113	-0.083169387	0.19862288
ENSDART00000158686	si:dkey-14k9.2	-0.49897987	-0.263068549	0.75542148	1.074830065
ENSDART00000158692	CABZ01112401.1	0.113542035	-0.344191992	-0.461207823	-0.256682679
ENSDART00000158696	mhc1zda	-0.851268531	-0.246667696	-0.324999925	-0.385360573
ENSDART00000158721		1.164230248	1.13549021	1.093110731	1.576844966
ENSDART00000158723	sncb	-0.257429657	-0.430720868	-0.069889176	0.08182323
ENSDART00000158733	si:dkey-33i11.1	1.813729642	1.289849367	0.876112899	0.279282769
ENSDART00000158734	CABZ01088933.1	-0.385251814	-0.457284358	0.104668245	0.198079674
ENSDART00000158739	Metazoa_SRP	1.431390172	1.797550169	1.401344471	0.830250287
ENSDART00000158743	zgc:113348	0.011068258	0.221603341	0.278365427	0.216955383
ENSDART00000158744	BX927336.3	4.021248005	3.307131569	3.405842268	3.528289574
ENSDART00000158766	CR848025.1	-0.596230295	-0.433122151	-0.989806185	-0.729213035
ENSDART00000158777	cirbpa	0.225987764	0.314210398	0.241597292	0.01082888
ENSDART00000158780	mapk8ip1a	0.350641915	0.585959364	0.286490226	-0.139620155
ENSDART00000158808	cnn3a	0.586268263	0.320248819	-0.106889335	-0.483977637
ENSDART00000158810	fkbp3	-0.588686525	-0.386876086	-0.522309858	-0.719247198
ENSDART00000158817	letm1	-0.280849194	-0.370052113	-0.172062949	-0.075482878
ENSDART00000158820	golga7bb	-0.441549718	-0.650879189	-0.491036037	-0.164497612
ENSDART00000158825	actc1b	0.015935591	2.858633574	3.729694641	2.583924646
ENSDART00000158826	CABZ01058650.1	0.277651195	0.407733859	0.060586967	-0.150969128
ENSDART00000158837	dync1h1	0.179298196	0.363338197	0.829945857	0.598133568

ENSDART00000158854	tpd52l1	-0.063123021	-0.257733521	-0.65813611	-0.260747428
ENSDART00000158917	abhd10a	-0.199951388	-0.281143798	-0.472212357	-0.246558895
ENSDART00000158919	lpar1	-0.789836287	-0.638879508	-0.710773837	-0.465534721
ENSDART00000158936	grin1a	-0.976292061	-1.003442352	-0.317903979	0.15163936
ENSDART00000158962	dpf2	0.023344289	-0.160855631	-0.651177712	-0.029134577
ENSDART00000158977	si:ch73-266f23.1	0.491629493	0.41114477	0.471295394	0.297543095
ENSDART00000159004	si:ch73-233f7.8	0.057778797	0.28613349	0.517419704	0.568556694
ENSDART00000159016	psmd1	0.220320535	0.390334714	0.382386975	0.213567973
ENSDART00000159024	btr29	2.93548853	3.916483422	3.440636421	1.361130093
ENSDART00000159034	rsu1	0.959727483	0.959427671	0.950095712	0.424675541
ENSDART00000159061	si:ch211-185a18.2	-0.270017947	-0.418794122	-0.535787315	-0.21407671
ENSDART00000159062	paqr7a	-0.415028281	-0.250472931	-0.476345463	-0.551526055
ENSDART00000159087	drd2b	-0.654775306	-0.766659414	-0.766987138	-0.309487495
ENSDART00000159103	nkd3	3.293495606	1.842810424	2.875712797	0.7112483
ENSDART00000159104	atp1b2a	-0.488650552	-0.417675156	-0.129746035	0.084213141
ENSDART00000159112	si:ch211-285j22.3	-0.41628689	-0.545704947	-0.599123468	-0.263727805
ENSDART00000159123	btbd17b	-0.172708316	-0.362184438	-0.675325658	-0.245697841
ENSDART00000159142	si:dkey-56m15.2	0.312405009	0.342224899	0.497281083	0.253644828
ENSDART00000159160	impdh1b	0.016028158	-0.054860403	0.596376969	0.34908306
ENSDART00000159163	c2cd2l	-0.400916824	-0.28913261	-0.285175907	-0.160469096
ENSDART00000159165	b3galt2	-0.421223634	-0.395995108	-0.436113716	-0.390524412
ENSDART00000159177	si:dkey-61n16.5	-0.004555446	0.268605493	-0.72825811	-0.687838681
ENSDART00000159178	hspa8	-1.810725395	-0.905134344	-6.819365873	-1.947204217
ENSDART00000159184	erc2	0.268311207	-0.09915171	0.379637611	0.748745363
ENSDART00000159246	ctsa	0.554249571	0.498653237	0.219459997	0.055975716
ENSDART00000159249	oxr1a	-0.748854688	-0.518408458	-0.301236362	-0.153948362
ENSDART00000159252	abca3b	0.181429492	0.428459166	0.640212151	0.464148107
ENSDART00000159256	kif5c	0.534952361	0.603334839	0.411520272	0.273079865
ENSDART00000159259	capn1b	0.6323058	1.402210713	1.384902028	1.097440242
ENSDART00000159274	sf3b3	0.246778314	0.098942964	0.083691016	-0.027346389
ENSDART00000159291	podxl2	-0.45367951	-0.207714244	0.333702352	0.497299671
ENSDART00000159296	si:ch73-138e16.4	-0.371496737	-0.627266426	-0.52708555	-0.509641971
ENSDART00000159300	mcm7	0.714704002	0.831682391	0.874291792	0.339164006
ENSDART00000159312	pim1	0.541604371	0.710042231	0.310719343	-0.194529837
ENSDART00000159316	mvda	0.177821409	1.524901044	1.830059782	0.927790622
ENSDART00000159328	FO704782.1	0.142074859	0.061082305	0.804157236	0.053378695
ENSDART00000159330	pcyox1	0.406833689	0.21789945	0.044242143	-0.158070725
ENSDART00000159340	ctnnd1	0.771872624	0.982959313	0.944642837	0.226155974
ENSDART00000159368	myo19	-0.061571264	-0.562765185	-0.263758975	-0.061266965
ENSDART00000159372	eif3ha	0.499618724	0.599836819	0.638052337	0.263915251
ENSDART00000159390	FO704846.1	-0.36021367	-0.403087033	0.444915596	0.998151126
ENSDART00000159409	unc45a	0.063679601	0.06008134	0.330631234	0.129605203
ENSDART00000159433	BX571691.1	2.919338685	1.742268402	2.797274384	2.742997875
ENSDART00000159434	tmem254	0.529951039	0.26685766	0.074245289	-0.042910999
ENSDART00000159435	pcdh2g28	0.000816386	0.116171	0.335030381	0.350715644
ENSDART00000159438	zgc:162730	0.775020432	0.781916155	0.788293058	0.232021834
ENSDART00000159450	ctnnd1	0.19135693	0.714895836	-0.026058241	0.026164146
ENSDART00000159454	CABZ01044277.1	0.109016927	0.066686129	0.335359626	0.279394938
ENSDART00000159466	FUT9 (1 of many)	-0.32488192	-0.508163292	-0.746293394	-0.756603469
ENSDART00000159486	anapc11	-0.082411238	-0.111278818	-0.383958603	-0.347049397
ENSDART00000159491	ddx46	0.204487031	0.180157737	0.416223492	0.263114106

ENSDART00000159493	baiap2a	-0.408759075	-0.584635172	-0.205977447	-0.074404323
ENSDART00000159519	SIKE1	-0.137869817	-0.17784406	-0.297014412	-0.12625892
ENSDART00000159520	dusp1	1.00607749	1.105172193	0.739133795	1.131411014
ENSDART00000159523	BX248501.1	-0.802081931	-1.281225474	-1.178391388	-0.841643515
ENSDART00000159534	leng9	0.34368633	0.401471128	0.132926162	0.109702136
ENSDART00000159543	si:ch211-284e13.6	-2.69508904	-0.200744398	-0.117258809	-0.389858773
ENSDART00000159581	CT583723.1	-0.203564175	-0.159816156	-0.458224986	-0.16588667
ENSDART00000159588	rnf24	-0.199572807	-0.336306858	-0.290748993	-0.299485771
ENSDART00000159589	rtn4rl1b	-0.618605611	-0.334861623	-0.03219779	0.419833076
ENSDART00000159601	serpini1	-0.44709139	-0.357709199	-0.198408132	0.076823185
ENSDART00000159604	mbd3b	-0.404570692	-0.312781624	-0.318828454	-0.248466452
ENSDART00000159608	fstl5	-0.296329015	-0.522282128	-0.291629941	-0.144161607
ENSDART00000159614	r3hdm1	-0.174685122	-0.341121434	0.099641284	0.042466817
ENSDART00000159620		0.106577262	0.152035698	0.604289635	0.634717201
ENSDART00000159627	scn4ba	-1.651719386	-1.747876787	-0.730948262	-0.207716251
ENSDART00000159629	mcm2	0.995796878	0.693727511	0.639304212	0.157223466
ENSDART00000159634	b3glcta	-0.092602693	-0.194400961	-0.44155461	-0.530900199
ENSDART00000159642	DPP10	-0.369468467	-0.43082527	-0.361060788	-0.270120898
ENSDART00000159652	sox4b	0.767109202	1.080655119	1.389017231	0.976257694
ENSDART00000159654	CR847844.2	0.025394661	-0.340096605	-0.548288987	-0.346027376
ENSDART00000159670	abtb1	-0.10462458	-0.155472174	-0.372376774	-0.233981977
ENSDART00000159673	dync1i2b	0.025252757	0.176262587	0.413723725	0.300516643
ENSDART00000159690	gpr75	-0.483974147	-0.377125661	0.037666989	0.123067934
ENSDART00000159693	NYAP1	-0.548984567	-0.516611795	-0.021366781	0.000605996
ENSDART00000159697	si:rp71-5o12.3	0.531788358	0.397969004	0.570994446	0.299640856
ENSDART00000159716	si:ch73-236c18.6	0.962463693	0.259951761	0.066014607	0.162933451
ENSDART00000159725	mtm1	-0.155968464	-0.279489824	-0.277642503	-0.186816665
ENSDART00000159727	pcdh11	-0.294947297	-0.365638864	-0.089140913	0.17419873
ENSDART00000159732	si:dkey-203a12.8	3.015691266	3.637172924	3.104587293	1.713663998
ENSDART00000159733	CABZ01063757.1	0.129910694	-0.203491407	-0.855433405	-0.227320323
ENSDART00000159745	PCP4L1 (1 of many)	-0.632453626	-0.700162017	-0.354659005	-0.133179733
ENSDART00000159747	rab11fip4b	-0.416792935	-0.701212044	-0.335764895	-0.067364991
ENSDART00000159752	sort1a	-0.095510148	-0.060577332	-0.362899297	-0.357946523
ENSDART00000159769	pam	-0.468861179	-0.403502349	-0.282541701	-0.241769832
ENSDART00000159782	agtpbp1	-0.044706055	0.173624177	0.473061984	0.407831162
ENSDART00000159805	CABZ01085924.1	-0.392684277	-0.456709342	0.004929566	0.18758642
ENSDART00000159845	epb41a	-0.285946023	-0.193154687	-0.17771991	-0.091337383
ENSDART00000159853	FQ311890.1	-0.276177481	-0.432407927	-0.032078728	0.27665545
ENSDART00000159859	tmem132e	-0.259691326	-0.228995571	-0.113717547	-0.037626683
ENSDART00000159871	ptk2aa	0.224270244	0.259268875	0.303476245	0.275768955
ENSDART00000159899	mapk10	-0.350531159	-0.285511604	-0.104624207	0.054397304
ENSDART00000159908	camk1db	-0.548571048	-0.478436516	-0.873630088	-0.641909911
ENSDART00000159916	tns1a	-0.270694335	-0.694531751	-0.372872148	-0.014128978
ENSDART00000159928	si:dkey-274m17.3	-0.105974275	-0.260400887	-0.454854053	-0.384734441
ENSDART00000159942	kcnc3b	-0.794025304	-1.361623092	-0.389946342	0.009706478
ENSDART00000159949	wdr1	0.362540326	0.308247591	0.564916756	0.170183845
ENSDART00000159950	si:ch1073-450f2.1	-0.285454261	-0.252056938	-0.418320337	-0.16839092
ENSDART00000159952	sema5bb	0.4270075	0.43121825	0.280410439	0.178648412
ENSDART00000159956	ndufa4l2a	0.563891792	0.528048098	0.326797956	0.131990505
ENSDART00000159958	hcn1	-0.047230014	-0.465327973	-0.263546106	-0.045423137
ENSDART00000159965	si:ch211-255i3.4	0.121150704	0.407179882	0.241107117	0.125992518

ENSDART00000159987	si:dkey-65b13.13	-0.209652039	-0.259703439	-0.637719895	-0.234430743
ENSDART00000159989	cpne7	-0.2792921	-0.302878059	-0.342117017	-0.194332814
ENSDART00000159997	cacna1ab	-0.118996824	-0.488974274	-0.025554874	0.331301032
ENSDART00000160000	spdl1	1.145158953	1.158182543	1.166185329	0.390123322
ENSDART00000160006	Metazoa_SRP	1.639017988	2.129447988	1.830106321	0.946987666
ENSDART00000160017	CABZ01058261.1	0.903038959	0.866986019	0.595792077	0.158138964
ENSDART00000160018	DUSP26	-0.660306321	-0.709450564	-0.129656337	0.007184288
ENSDART00000160048		1.164230248	1.13549021	1.093110731	1.576844966
ENSDART00000160055	CABZ01048399.1	2.04373243	1.562490919	0.966755902	1.237958862
ENSDART00000160073	sntg2	-0.084902345	-0.387083384	-0.20960703	-0.013813106
ENSDART00000160107	CU639413.1	0.54252914	0.409259037	1.419043143	0.130740048
ENSDART00000160115	per1a	0.041503641	-0.272737679	-0.476359218	-0.15653808
ENSDART00000160116	racgap1	0.647984241	0.386764067	0.354192772	0.100327502
ENSDART00000160134	hibadha	-0.191259291	-0.221221329	-0.647202373	-0.244609156
ENSDART00000160138	sobpa	-0.321225255	-0.895188795	-0.069068022	0.312612038
ENSDART00000160154	si:dkey-238i5.2	-0.200329967	0.657530632	1.151706484	1.107326351
ENSDART00000160155	PDCL3	1.150151141	1.512943629	1.437587964	2.391657524
ENSDART00000160175	SYNDIG1	-0.028604943	-0.23293463	-0.464850771	-0.153309965
ENSDART00000160177	kcnb2	4.745895577	4.144383899	5.457366505	5.666212289
ENSDART00000160189	hip1rb	-0.298878525	-0.25639736	0.130568312	0.08288489
ENSDART00000160201	tnfrsf11b	2.52634883	1.22900254	0.873090756	-0.039240801
ENSDART00000160206	rap2b	0.536023906	0.628551975	0.691733134	0.267820563
ENSDART00000160209	si:ch211-204a13.2	-0.408114948	-0.445711734	-0.270878027	0.146816503
ENSDART00000160220	kif5aa	-0.17024292	0.126849007	0.479028265	0.345007215
ENSDART00000160236	fras1	-0.444181856	-0.638830186	-0.533612869	-0.242512624
ENSDART00000160242	TYMP	-0.668893964	-0.374517624	-0.757876583	-0.434642219
ENSDART00000160245	si:ch73-236c18.7	2.124440879	1.318007353	1.287829039	0.437471879
ENSDART00000160284	pclob	-0.164621492	-0.643566861	-0.569524507	-0.247739035
ENSDART00000160288	FP236812.6	0.238382616	0.543365019	0.866982901	0.48841948
ENSDART00000160297	vcana	1.601498468	0.665214133	0.454293537	0.486706931
ENSDART00000160305	si:zfos-943e10.1	-0.732433247	-0.018482138	-0.2347001	-0.520119378
ENSDART00000160308	pde3a	-0.225066293	-0.312371173	0.037986462	0.082811204
ENSDART00000160324	CABZ01102047.1	-0.357465059	-0.236339235	0.433910345	0.432628055
ENSDART00000160328	zgc:109949	-0.362552375	-0.659300829	-0.471802707	-0.430381288
ENSDART00000160331	atp6v0e1	0.536560194	0.109862681	-0.220128717	-0.336777163
ENSDART00000160337	si:ch73-299h12.8	-0.912707831	-0.414120081	-0.53567861	-0.405510308
ENSDART00000160350	IQSEC1 (1 of many)	-0.73025949	-0.724161984	-0.227945297	-0.158150827
ENSDART00000160362	EGFLAM	-0.160131743	-0.324587558	-0.48719254	-0.200185739
ENSDART00000160400	gchfr	-0.373470826	-0.390793782	-0.620398999	-0.352985133
ENSDART00000160401	tmem63ba	-0.025633015	0.10439182	0.273009198	0.198949087
ENSDART00000160409	arf6a	0.10280988	-0.030227439	0.312347712	-0.095328656
ENSDART00000160424	ftr36	-0.057998525	-0.261050684	0.030750272	-0.246695716
ENSDART00000160425	LAMP1	-0.291224845	-0.711008477	-0.768820578	-0.726430297
ENSDART00000160431	dhrs13l1	-0.306310555	-0.206975564	-0.316329966	-0.293060295
ENSDART00000160439	CABZ01037298.1	-0.12483018	-0.201319697	-0.520978425	-0.240731645
ENSDART00000160444	si:dkey-95j14.1	-0.172108125	0.025439256	0.48271623	0.286285124
ENSDART00000160450	CU571069.1	-0.391785076	-0.855515025	-0.904047805	-0.612021184
ENSDART00000160464	pcdh1gb2	-0.570850707	-0.31567681	-0.173184848	-0.165810233
ENSDART00000160465	opn6a	-0.331059122	-0.451170972	-0.507697352	-0.105376089
ENSDART00000160466	ndrg4	-0.797536516	-0.447807808	-0.265830716	-0.169347229
ENSDART00000160468	ubl3a	-0.157200321	-0.291255908	-0.684230474	-0.436379037

ENSDART00000160469	si:ch211-271b14.1	-0.816113506	-0.956386746	-0.419124903	-0.031051269
ENSDART00000160492	alad	0.30171604	0.443307609	0.248348623	0.158762163
ENSDART00000160495	si:dkey-184p18.2	0.380936601	-0.937356475	-0.364392869	-0.267264411
ENSDART00000160503	slc15a4	0.534058917	0.262741482	0.05558204	-0.165911292
ENSDART00000160509	trip10a	5.462743185	3.19366481	2.422825884	1.530105247
ENSDART00000160527	prokr1a	0.020123926	-0.217659905	-0.314012019	-0.217346505
ENSDART00000160536	gnsb	1.380175579	0.91424754	0.488823056	0.00723105
ENSDART00000160538	arhgap21a	-0.416942655	-0.374699279	-1.045101409	-0.721453587
ENSDART00000160542	cabp2a	-0.310654407	-0.386778791	-0.172317372	-0.340396034
ENSDART00000160555	sema6e	-0.284106568	-0.324928323	-0.250029521	-0.168860793
ENSDART00000160562	impg2b	0.024461351	-0.26080833	-0.706384589	-0.340668028
ENSDART00000160564	cyyr1	-0.193986492	-0.219795811	-0.518063496	-0.536169671
ENSDART00000160566	PPEF2	0.06510909	0.104690545	-0.262166795	0.034536141
ENSDART00000160571	mxe	0.057431509	-0.237146132	2.037062599	-0.095948656
ENSDART00000160575	CU459186.1	0.73831154	1.064084865	0.743119809	0.540430362
ENSDART00000160582	creb5a	2.028867303	1.691967909	1.27021295	0.836790625
ENSDART00000160588	si:dkeyp-86d6.2	-0.052465168	-0.307686486	-0.441266657	-0.083441391
ENSDART00000160600	cecr5	-0.047380691	0.036242917	0.854821049	0.34511846
ENSDART00000160604	ftsj3	0.856590556	0.695492085	0.825371198	0.428239045
ENSDART00000160606	mat2aa	0.169445123	0.256103943	0.254062393	0.177493443
ENSDART00000160612	bin3	0.276069755	0.185522197	-0.128294303	-0.167255765
ENSDART00000160614	tmem106ba	-0.216998722	-0.275060394	-0.388680799	-0.295077411
ENSDART00000160652	iqsec3a	-0.564945044	-0.609250827	-0.334399494	-0.124185292
ENSDART00000160662	CABZ01056578.1	-0.678580777	-0.38089804	-0.298190661	-0.130306976
ENSDART00000160663	tnk2b	0.180874765	0.228576581	0.365115453	0.173216656
ENSDART00000160672	MYH7 (1 of many)	0.452458943	2.61830504	2.948579403	1.474866684
ENSDART00000160700	dnm1l	-0.370704077	-0.348232697	-0.495814147	-0.141373029
ENSDART00000160707	smx5	-0.006041688	-0.141079427	-0.427667941	-0.488718653
ENSDART00000160712	dusp1	0.749410253	0.773951106	0.584904324	1.278120339
ENSDART00000160715	pik3r3a	-0.538136397	-0.043164195	-0.249012844	-0.109162985
ENSDART00000160738	arhgap11a	2.137805097	2.112857321	2.062351639	2.227173194
ENSDART00000160742	clmn	-0.341601235	-0.332225059	-0.489517534	-0.288919784
ENSDART00000160753	GANAB (1 of many)	0.004666803	-0.095016348	-0.32432452	-0.161685037
ENSDART00000160763	Metazoa_SRP	1.465611255	3.00506243	2.307455794	0.729030238
ENSDART00000160766	pim1	0.465870139	0.649909313	0.413306503	0.082988542
ENSDART00000160767	zgc:153845	-0.366926311	-0.362052716	-0.223963795	-0.098406698
ENSDART00000160783	rorcb	-0.657257878	0.184912673	0.679992874	0.484278813
ENSDART00000160789	SDK2 (1 of many)	-0.531586731	-0.76443238	-0.126364502	0.194536592
ENSDART00000160809	agap1	-0.12094537	-0.11171461	0.245949776	0.322867446
ENSDART00000160811	b3gat1a	-0.509506437	-0.522178413	-0.230803048	0.152925891
ENSDART00000160829	mhc1zda	-0.410179214	-0.276613879	-0.418038837	-0.322538004
ENSDART00000160840	prkar1b	-0.31036091	-0.161481279	-0.113380497	-0.010356954
ENSDART00000160841	si:ch211-248g20.5	-0.153486145	-0.342330243	-0.525669956	-0.178412263
ENSDART00000160847	hgh1	0.505719048	0.435629442	0.388567431	0.068708352
ENSDART00000160850	inpp5e	-0.436095235	-0.00283574	0.156525888	0.020455167
ENSDART00000160866	cry2	-0.114688269	-0.279009524	-0.604836383	-0.332518261
ENSDART00000160881	pcdh1g30	-0.53895262	-0.083348601	0.159825551	0.241016465
ENSDART00000160885	stbd1	-0.425801769	-0.465748469	-0.195945725	0.034690374
ENSDART00000160901	FMNL1 (1 of many)	1.814810207	1.982271858	2.471309982	2.117776194
ENSDART00000160902	naga	0.641123507	0.233762511	0.291444137	-0.017668678
ENSDART00000160927	cers1	-0.519571193	-0.339170129	-0.296459142	-0.066758323

ENSDART00000160933	si:ch211-153l6.6	-0.192271617	-0.190332633	-0.289802511	-0.209468422
ENSDART00000160950	dyrk1b	-0.419283008	-0.231908493	0.058730046	0.190507005
ENSDART00000160955	slc38a3b	-0.475545538	-0.367921084	-0.471714594	-0.850494481
ENSDART00000160956	oaz1b	-0.133855172	-0.165932835	-0.380106363	-0.375655267
ENSDART00000160979	jpt1b	0.682460724	1.366219793	1.515562314	0.876953288
ENSDART00000161002	kbtbd11	-0.286857265	-0.122332373	-0.245379055	-0.228313034
ENSDART00000161003	cpne9	-1.056006542	-1.193405404	-0.117014879	0.290018934
ENSDART00000161007	ets2	-0.669649632	-0.198034695	-0.011258584	-0.233709301
ENSDART00000161008	filip1a	1.277613198	1.48763933	0.952639764	1.12798471
ENSDART00000161017	eif3s6ip	0.313830903	0.293421495	0.276651786	0.120270233
ENSDART00000161040	U1	0.811168663	0.450901632	0.418592021	0.580227266
ENSDART00000161048	psmf1	-0.002898908	-0.064926331	0.496331974	-0.135580746
ENSDART00000161056	zgc:63587	0.468520933	0.333532949	0.066863333	-0.028483781
ENSDART00000161059	tusc2a	0.068801861	-0.121312821	-0.423616648	-0.242714442
ENSDART00000161063	heatr1	0.383217268	0.256645773	0.173900631	0.113179939
ENSDART00000161081	satb1a	-0.172286726	0.059256047	0.306569639	0.367466245
ENSDART00000161087	efna3b	-0.321733474	-0.009174863	0.104028903	0.249289152
ENSDART00000161100	FAT3 (1 of many)	-0.16621971	-0.375379053	-0.163291758	-0.002633554
ENSDART00000161101	lyrm5a	-0.096487275	-0.208413777	-0.302906059	-0.144622611
ENSDART00000161107	pcdh1g9	0.063645152	0.386867409	0.439855094	0.563716536
ENSDART00000161115	CR382281.1	-1.148281272	-0.70950654	-0.315399276	0.210191426
ENSDART00000161117		0.194355392	0.331000906	0.496659366	0.20108058
ENSDART00000161121	oxct1a	-0.18384202	-0.521566935	-0.230939432	-0.260776637
ENSDART00000161137	scn1bb	-0.455645009	-0.546610376	0.183837534	0.407926687
ENSDART00000161153	GAN	-0.076211127	0.283155249	0.536837618	0.420868128
ENSDART00000161156	kcnj9	-0.647175508	-0.597438502	-0.398278727	-0.23455974
ENSDART00000161168	si:ch211-232d10.1	-0.094530829	-0.188391079	-0.421762057	-0.370881661
ENSDART00000161184	si:dkey-238f9.1	-0.217284848	-0.396117732	-0.370650729	0.049289618
ENSDART00000161216	dync1i2a	1.005522301	0.520541958	0.468549497	-0.022454144
ENSDART00000161230	arhgef12b	0.167990134	0.325221884	0.334885818	0.114051067
ENSDART00000161240	zgc:92161	0.584076871	0.663364233	0.486030672	0.10810748
ENSDART00000161244	CR293509.1	0.407469749	1.465267423	1.274347742	0.975210426
ENSDART00000161250	grin1b	-0.242810387	-0.349766254	-0.246157691	0.067747546
ENSDART00000161261	lmbrd2a	-0.185598793	-0.153033552	-0.360733538	-0.113007589
ENSDART00000161264	ntrk3b	-0.768053413	-0.724776652	-0.30616772	0.183034577
ENSDART00000161266	CU928046.1	-0.122276569	-0.195535625	-0.336584046	-0.188368371
ENSDART00000161272	dclk1a	-0.780154894	-0.444639927	-0.375433616	-0.486681237
ENSDART00000161297	lrrn3b	-0.369968613	-0.470809526	-0.36045804	-0.046069156
ENSDART00000161322	slc29a1a	0.535023852	0.636573744	0.365586045	-0.115892928
ENSDART00000161330	col27a1a	0.210670367	0.547410859	0.646435326	0.255665234
ENSDART00000161347	CT583728.4	1.170122423	1.14685482	1.328877257	0.769737347
ENSDART00000161377	cdk2ap2	0.241317874	0.341753789	0.416424679	0.144968334
ENSDART00000161389	CR388042.1	0.279433291	0.140072251	0.112517442	0.232646376
ENSDART00000161397	atg13	-0.137451879	-0.18705676	-0.405447689	-0.269252338
ENSDART00000161407	tnika	-0.172678134	0.105967748	0.465016741	0.501980476
ENSDART00000161414	meis2a	-0.352024462	-0.259692709	-0.258698057	-0.07620107
ENSDART00000161421	zgc:194627	1.998498644	1.298544665	0.773438314	0.249109157
ENSDART00000161436	pcdh7b	-0.687665671	-0.773740554	-0.245502692	0.12030382
ENSDART00000161440	limch1b	-0.008873535	-0.193340096	-0.430561872	-0.157445684
ENSDART00000161445	fkrp	-0.298106015	-0.219330579	-0.124679519	-0.039794435
ENSDART00000161456	zgc:153924	-0.044959851	0.322360252	0.292539556	-0.042756925

ENSDART00000161460	pmt	2.538801298	3.7270331	2.780124527	1.870914111
ENSDART00000161505	mgrn1a	-0.282029057	-0.374993034	-0.219589284	-0.246471373
ENSDART00000161507	zgc:153426	-1.880708803	-0.117657621	0.865737326	1.072245785
ENSDART00000161514	CABZ01079986.1	-0.184749182	1.474076268	0.73277167	-0.443845367
ENSDART00000161525	zgc:171704	0.267822787	-0.368878081	-1.0813007	-1.211102226
ENSDART00000161532	zfyve9b	-0.607365928	-0.871459147	-0.090833114	0.138377783
ENSDART00000161533	gfra1b	-0.396684985	-0.507239408	-0.32341327	-0.285757426
ENSDART00000161561	ANK1 (1 of many)	-0.90876402	-0.980007711	-0.299110873	-0.111310605
ENSDART00000161566	b4galt6	0.155163411	0.348743299	0.367166825	0.307075635
ENSDART00000161567	KCNT1 (1 of many)	-0.564631347	-0.618941642	-0.212804629	0.112863315
ENSDART00000161582	ubfd1	0.213892452	0.378867256	0.328612523	0.082100895
ENSDART00000161586	flot2a	0.149914225	0.375737842	0.414499477	0.21470497
ENSDART00000161597	si:ch211-262i1.3	0.726810222	0.918966234	1.284521547	0.120004192
ENSDART00000161613	SLC7A1 (1 of many)	0.777752401	1.137604367	0.924750387	0.450737498
ENSDART00000161646	sema6bb	-0.22063759	-0.362707192	-0.160882628	0.032299092
ENSDART00000161647	fnbp1b	0.397270086	0.395681626	0.254962279	-0.147831954
ENSDART00000161650	atp2a2b	0.018261356	0.090439762	0.265163492	0.020190481
ENSDART00000161652	si:dkeyp-72e1.9	-0.109528412	-0.231594686	-0.432617543	-0.203491956
ENSDART00000161662	ttbk1a	-0.363444816	0.177390115	0.591497043	0.421229884
ENSDART00000161673	CR450729.3	-0.466290317	-0.130444226	-0.527062063	-0.184859299
ENSDART00000161693	cetn2	-0.42442122	0.157217987	0.592113306	0.77779027
ENSDART00000161698	spock1	-0.400537237	-0.522818259	-0.36383883	-0.3494177
ENSDART00000161700	st3gal2	-0.330022994	-0.187434223	0.066957229	0.392836726
ENSDART00000161704	CNDP1	-0.617638491	-0.321066179	-0.670749127	-2.338544776
ENSDART00000161708	si:ch73-29l19.1	-0.183985257	-0.352166832	-0.310452263	-0.095145467
ENSDART00000161725	map7d1a	-0.696007823	-0.432281688	0.042931769	0.13512701
ENSDART00000161735	flrt1a	-0.150942569	-0.355176624	-0.261636355	-0.097731299
ENSDART00000161755	CU681842.1	-0.157328162	-0.245310878	-0.348302227	-0.161138988
ENSDART00000161770	cpne9	-0.832265602	-0.611063661	-0.201518154	0.170760147
ENSDART00000161773	KCNJ11 (1 of many)	-0.422252277	-0.360338989	-0.607728772	-0.332578335
ENSDART00000161798	si:ch211-188f17.1	-0.190236489	-0.379885925	-0.177844783	-0.0015826
ENSDART00000161807	gnl2	0.286549103	0.322764935	0.293655806	0.087475664
ENSDART00000161818	prr33	0.089254338	1.155971891	0.95184285	0.668590241
ENSDART00000161823	ubl3a	-0.089124518	-0.239047703	-0.471864878	-0.290785384
ENSDART00000161828	si:rp71-1h20.9	-1.646379844	-1.190834107	-1.032421214	-1.471047696
ENSDART00000161840	CT574575.1	-0.29915223	-0.372107241	-0.764083264	-0.340151107
ENSDART00000161854	pls3	0.235895362	0.649174918	0.495504879	0.31420242
ENSDART00000161869	dysf	1.077492273	1.30463907	1.440346007	1.017975966
ENSDART00000161882	bzw2	0.083435949	-0.31200966	-0.5490146	-0.324311149
ENSDART00000161892	vdra	-0.26830787	-0.422747599	-0.12129137	-0.077310915
ENSDART00000161906	vwa11	1.529478256	0.755786632	0.896680083	0.546980022
ENSDART00000161908	nsun2	4.199768382	2.926785137	4.095496425	2.988717061
ENSDART00000161925	rundc3aa	-0.101671945	0.461428265	0.452450402	0.467812596
ENSDART00000161930	strn4	-0.158687938	-0.097490818	0.286103327	0.194201646
ENSDART00000161932	npr3	0.255355781	0.183557252	-0.357151542	-1.063769364
ENSDART00000161938	si:dkeyp-9d4.3	-0.230392301	-0.461066854	-0.432261332	-0.276495229
ENSDART00000161967	ctsc	1.279775059	0.583635254	0.329059668	-0.157215155
ENSDART00000161978	BX649556.1	0.438096957	0.234757098	0.251734491	0.204407619
ENSDART00000161982	SCARNA6	0.10205479	-0.170611192	-0.840119974	-1.131956342
ENSDART00000161992	PAQR9	-0.193753966	-0.524789375	-0.118624882	0.042415903
ENSDART00000162002	grm1b	-0.568603213	-0.750999293	-0.21404532	-0.094504234

ENSDART00000162007	Metazoa_SRP	0.903949119	2.303055473	1.702580761	0.513671528
ENSDART00000162010	zgc:92140	-0.256831407	-0.245054913	-0.345912399	-0.346929099
ENSDART00000162018	CABZ01002768.2	0.265184254	0.128771486	0.086197325	0.254354536
ENSDART00000162023	plppr2a	0.019417247	-0.576626688	0.082766084	0.317388761
ENSDART00000162026	ninl	-0.053184172	-0.137296281	-0.336798097	-0.200288689
ENSDART00000162046	sez6a	-0.383252568	-0.442703172	-0.383098336	-0.145401001
ENSDART00000162055	mark1	-0.053725407	0.099849875	0.364172927	0.499618468
ENSDART00000162063	ppifb	-0.192515925	-0.861096392	-0.286677305	-0.512048401
ENSDART00000162070	tmem9	-0.208225931	-0.361514875	-0.511868133	-0.393713678
ENSDART00000162097	CABZ01044157.1	1.031550352	0.670727071	0.212982548	-0.249292553
ENSDART00000162119	ewsr1a	-3.071838226	0.182908872	-0.16348899	0.256173861
ENSDART00000162133	tenm4	-0.287462387	-0.412164757	-0.184969779	0.049525758
ENSDART00000162139	si:ch211-244c8.4	-0.176471944	0.210261822	0.365856922	0.312900704
ENSDART00000162145	FQ377629.1	-0.12898611	-0.128745299	0.191405016	0.357155255
ENSDART00000162200	STX3 (1 of many)	-0.155323726	-0.36938348	-0.41294698	-0.132327196
ENSDART00000162218	col10a1b	0.45003474	2.713575183	3.448261467	1.87125716
ENSDART00000162222	SHC3	-0.255397657	-0.359792108	-0.178043549	-0.323533675
ENSDART00000162228	grip2a	-0.209580983	-0.468643062	-0.36247099	-0.147880595
ENSDART00000162277	grip1	-0.456620328	-0.2169797	0.146006741	0.157130316
ENSDART00000162278	CABZ01053748.1	-0.203505904	-0.497916501	-0.162237791	0.002359168
ENSDART00000162282	dicp3.1	0.839763954	0.379787637	0.004488715	-0.034973807
ENSDART00000162290	GARNL3	0.087177538	0.188445106	0.266999217	0.231469459
ENSDART00000162294	utrn	-0.256734894	-0.284908669	-0.969336134	-0.369539596
ENSDART00000162296	cfp	0.329536618	0.225136408	-0.193120136	-0.891420593
ENSDART00000162302	bnip3	-0.278521269	-0.244198471	-0.204837653	-0.079960627
ENSDART00000162324	kalrnb	-0.363230198	-0.20882241	0.367737091	0.490925401
ENSDART00000162331	si:dkey-22o22.2	-0.434953721	-0.633123757	-0.287732073	-0.108064019
ENSDART00000162359	sorbs1	-0.104497249	-0.20837462	-0.418151878	-0.317718588
ENSDART00000162377	FP085414.1	0.809712454	1.275862162	1.389373351	0.791945088
ENSDART00000162381	dkk3a	-0.551126502	-0.361561955	-0.324385943	0.074151181
ENSDART00000162387	zbtb38	-0.362042869	-0.285747988	-0.199364681	-0.27814593
ENSDART00000162399	tacc3	1.052644038	0.862999188	0.469743238	-0.177095944
ENSDART00000162403	napgb	-0.42647327	-0.557407706	-0.383093342	-0.343712014
ENSDART00000162421	si:ch73-233f7.7	0.22834555	0.078820055	0.428518248	0.477794315
ENSDART00000162459	scarb2a	0.204566318	0.711810949	0.821282796	0.534817262
ENSDART00000162472	VWA5A (1 of many)	0.543940251	0.262980075	0.410699646	-0.136546959
ENSDART00000162474	irx1a	-1.132797992	-0.349993381	0.581252213	0.55366573
ENSDART00000162482	zgc:152863	0.539805357	0.660295595	0.432850415	0.095615261
ENSDART00000162485	pax6a	-0.383059238	-0.321515523	-0.107334897	0.077147684
ENSDART00000162493	kif5ab	-0.358862317	-0.407894033	-0.585721315	-0.34361757
ENSDART00000162501	abca4a	-0.194244085	-0.198091298	-0.350966144	-0.228681741
ENSDART00000162520	SHC3	-0.325188551	-0.499304719	0.001359259	-0.194280026
ENSDART00000162527	si:ch211-160f23.7	-0.331311011	-0.409364601	-0.344819459	-0.114250304
ENSDART00000162529	zgc:172139	0.398363371	0.414482162	0.745277151	0.505205904
ENSDART00000162539	VWA5A (1 of many)	1.505543925	2.134948516	2.442684472	2.675816303
ENSDART00000162540	Metazoa_SRP	1.640394936	1.730362994	1.214251464	0.854214156
ENSDART00000162541	thbs4a	1.94439423	3.279546426	3.089397424	2.286354886
ENSDART00000162555	dpyda.1	0.585872258	0.381337517	0.027895003	-0.679828257
ENSDART00000162564	CT027980.1	-0.351165538	-0.178285414	-0.533647461	-0.500417084
ENSDART00000162566	h2afvb	0.462673668	0.293160887	0.459514152	0.229847552
ENSDART00000162587	chd5	-0.534129477	-1.024077887	-0.70517204	-0.958580386

ENSDART00000162595	camk2g1	-0.045798893	-0.36296086	-0.180481912	0.032536235
ENSDART00000162601	scn2b	-0.529498392	-0.64570335	-0.359290818	0.039638437
ENSDART00000162607	CR361561.2	0.410555114	0.246904987	0.207590099	0.517188645
ENSDART00000162617	ppp2cb	0.050413288	0.287682373	0.42325509	0.191699941
ENSDART00000162622	FP103009.1	-0.352224047	-0.048475545	0.149997583	0.073530546
ENSDART00000162637	CABZ01054392.2	-0.811315315	0.030869752	-0.070916412	-0.071296331
ENSDART00000162664	5S_rRNA	-0.438943374	-1.186726175	-0.920371372	-0.548334869
ENSDART00000162668	cremb	4.310209888	4.052771	3.333577791	2.240090056
ENSDART00000162669	slc4a5	-0.13383932	-0.22542524	-0.430682825	-0.287279235
ENSDART00000162670	slc8a1b	-0.269852688	-0.529256481	-0.096682296	0.048355338
ENSDART00000162675	trim2b	-0.409757052	-0.631223683	-0.07653124	0.093922127
ENSDART00000162683	trappc9	-0.370182614	-0.24071592	-0.273499446	-0.133452933
ENSDART00000162696	cacnb4b	-0.474781259	-0.004559691	0.081636688	0.060425945
ENSDART00000162710	fgf13b	0.123584158	0.587591002	0.690906211	0.488537905
ENSDART00000162711	cnksr2b	-0.20949159	-0.28214605	-0.418907028	-0.082150791
ENSDART00000162714	pcdh10b	-0.340908137	-0.430434424	-0.069086695	0.178421467
ENSDART00000162722	zgc:153867	0.467799327	0.468299246	0.579069745	0.022677056
ENSDART00000162732		-0.497798966	-0.517625334	-0.441909965	-0.132483146
ENSDART00000162761	CU984600.2	0.225517614	-0.442281881	2.62985535	0.023562363
ENSDART00000162799	crb3a	0.362169262	0.040586113	-0.086039525	-0.014272311
ENSDART00000162804	KCNT1 (1 of many)	-0.406408577	-0.48233323	-0.378259831	0.110845983
ENSDART00000162827	si:dkey-92j12.5	-0.013228451	-0.265522895	-0.385437174	-0.080127062
ENSDART00000162838	agrn	0.183430669	0.084636207	0.451853257	0.572854924
ENSDART00000162850	irx3a	-0.694816328	-0.301932125	0.187764734	0.208405543
ENSDART00000162855	pcdh1g13	0.288424517	0.288939089	0.455966507	0.614698967
ENSDART00000162857	nr4a3	-1.482016444	-1.152680943	-0.606031454	-1.193862677
ENSDART00000162858	lmbrd1	-0.243655467	-0.23291492	-0.332336473	-0.114530821
ENSDART00000162868	PCMTD2 (1 of many)	-0.184710465	-0.224010966	-0.277853434	-0.160151685
ENSDART00000162875	rogdi	-0.224891588	-0.27706391	-0.36191309	-0.159630193
ENSDART00000162886	il1rapl1b	-0.535131005	-0.60836138	-0.536159373	-0.086785386
ENSDART00000162897	Metazoa_SRP	1.178659737	1.5924133	1.384383775	1.197556527
ENSDART00000162915	dclk2b	0.022821815	0.633802822	1.144009661	0.962163073
ENSDART00000162916	si:ch211-177d9.1	-0.561077771	-0.777629972	-0.458457407	-0.043076344
ENSDART00000162924	SCARNA2	-0.051299469	-0.033570311	-0.355924265	-0.225455165
ENSDART00000162940	mob2b	0.020755037	-0.2829937	-0.541864256	-0.204837959
ENSDART00000162958	si:ch211-260e23.9	0.345171245	0.431307401	0.079575678	0.236719343
ENSDART00000162970	znf1001	2.835681681	3.195296273	3.608291375	4.001715823
ENSDART00000162984	cacnb2a	-0.567328475	-0.858575333	0.120461387	0.550245867
ENSDART00000163006	magi1b	-0.558734199	-0.633598853	0.045195354	0.572492467
ENSDART00000163018	bet1l	0.723011674	0.444641169	0.411413339	0.530095318
ENSDART00000163023	sypb	-0.076679322	-0.067070783	-0.475180992	-0.188875227
ENSDART00000163025	slc37a1	0.146947624	0.663221495	0.752146696	0.093340063
ENSDART00000163026	igf2bp3	1.596757122	2.364080903	3.304264113	3.343316319
ENSDART00000163032	Metazoa_SRP	1.221075219	2.596056056	2.04902232	1.755997125
ENSDART00000163039	fgfrl1b	-0.263701101	-0.339844216	-0.261709078	-0.264179912
ENSDART00000163042	si:dkey-16p6.2	0.770521119	0.592959463	0.681550899	0.554585181
ENSDART00000163057	FP101882.1	-0.031169748	-0.097998763	-0.346688618	-0.474056029
ENSDART00000163075	zgc:173552	0.365012264	0.677919016	1.138040868	0.74357363
ENSDART00000163077	pcdh1g22	-0.379602696	-0.085601773	0.347287318	0.30809701
ENSDART00000163089	BX649411.2	0.422281392	0.001934371	-0.524390062	-0.335409314
ENSDART00000163093	lrp12	-0.07747456	-0.233717712	-0.371900586	-0.129203418

ENSDART00000163096	gpr22b	-1.106969479	-1.366483735	-0.599957035	0.051341663
ENSDART00000163106	cntnap5l	-0.230959418	-0.661142608	-0.469168415	-0.076363144
ENSDART00000163132	rab11fip5a	-0.667316443	-0.571914956	-0.021672662	0.188839381
ENSDART00000163137	elovl1a	1.359201374	2.32562135	1.99000125	0.940099891
ENSDART00000163149	slco1e1	0.176388599	0.147707318	-0.389938785	-1.215116068
ENSDART00000163174	si:ch73-42p12.2	0.635401366	0.972834844	0.669544171	0.150769788
ENSDART00000163182	tpm2	-0.110471418	-0.35380494	-0.587423123	-0.759315105
ENSDART00000163216	prune2	-0.414992243	-0.329741076	-0.094156508	0.003976732
ENSDART00000163238	stom	-0.421228902	-0.189947439	-0.023059486	-0.114725703
ENSDART00000163243	dpysl4	0.526851432	0.969802663	1.139409914	0.808513895
ENSDART00000163250	mef2cb	0.361224616	-0.300957895	-0.57227951	-0.080214844
ENSDART00000163305	fxyd6l	-0.209100518	-0.188713869	-0.620315584	-0.44198331
ENSDART00000163310	exosc4	-1.250232551	-1.071141483	-0.30340673	-0.290327378
ENSDART00000163327	rpl36	0.36660589	0.401460063	0.099733249	-0.024083137
ENSDART00000163337	wu:fj30f06	0.562927267	0.339612631	0.922878619	0.481097928
ENSDART00000163352	impg2b	-0.050961847	-0.309849144	-0.745225088	-0.302394708
ENSDART00000163353	lrp2a	-0.727729459	-1.488145344	-2.965914229	-0.433516037
ENSDART00000163355	gria2a	-0.37007276	-0.57694131	-0.293854127	0.240567837
ENSDART00000163367	TFR2	0.892525381	1.842645951	2.060867557	1.063290775
ENSDART00000163370	si:rp71-36a1.2	0.777729217	1.232972386	1.065047677	0.579071639
ENSDART00000163380	si:dkey-22o22.2	-0.500713087	-0.482673389	-0.426086075	-0.212897339
ENSDART00000163391	si:ch211-149k12.3	0.507081525	0.32718269	0.316220639	0.200966933
ENSDART00000163394	dgke	-0.154264184	-0.257555682	-0.346312178	-0.3002364
ENSDART00000163398	pla2g15	0.617752366	0.443695085	0.159097428	0.08811481
ENSDART00000163407	slc20a1a	0.970510209	0.576996952	0.406775549	-0.419444699
ENSDART00000163441	limk1a	1.134897491	0.940120128	1.036684953	0.715200814
ENSDART00000163445	zgc:109982	0.178949347	-0.334063834	-0.837985378	-0.307869575
ENSDART00000163463		0.439033688	0.673311945	0.275086265	0.11827711
ENSDART00000163491	reep6	-1.146193953	-0.500723418	-0.964434532	-0.007386228
ENSDART00000163516	BX927374.1	-0.474896067	-0.084073015	-0.358098021	-0.301541481
ENSDART00000163519	CABZ01050166.1	-0.484053035	-0.532447227	-0.143157501	0.191505756
ENSDART00000163521	si:ch1073-83n3.2	-0.135028227	-0.352956778	-0.389044273	-0.105210001
ENSDART00000163523	sez6l	-0.314616095	-0.257996779	-0.399166894	-0.159678617
ENSDART00000163526	mansc1	0.060022776	0.316271416	0.600176682	0.64715281
ENSDART00000163529	FAM126B	-0.519426512	-0.370293712	0.022402011	0.032038169
ENSDART00000163531	CU929294.1	-0.283562448	-0.225727022	-0.174388489	-0.430860144
ENSDART00000163539	scrt1a	-0.576475687	-0.187048661	0.063865515	0.398969662
ENSDART00000163565	plcl2	-0.007453137	-0.119106713	-0.251934099	-0.322673112
ENSDART00000163568	kcnc1a	-0.672590813	-0.97944076	-0.78875743	-0.1747006
ENSDART00000163577	fnbp1b	0.973038753	0.902992088	0.678793665	0.275094435
ENSDART00000163582	desi1b	0.334416001	0.338516182	0.11270838	-0.034533924
ENSDART00000163585	csf1ra	0.499534778	0.258389647	0.175653195	-0.286108065
ENSDART00000163597	CABZ01029366.1	-0.380235128	-0.253799187	-0.219703335	-0.156720283
ENSDART00000163609	mrps16	-0.312251198	-0.078513731	-0.202471055	-0.117952357
ENSDART00000163612	gabrb4	-0.445185281	-0.610684009	-0.211332691	0.127493005
ENSDART00000163616	homer1b	-0.487597805	-0.239525084	-0.239441952	-0.083482271
ENSDART00000163622	kirrel3l	-0.615947415	-0.493751402	-0.207879997	0.127527609
ENSDART00000163635	erbb4b	-0.423431984	-0.772483288	-0.24962409	0.07826798
ENSDART00000163656	CAMK2N1	-0.350066285	-0.376343666	-0.297112769	0.007693675
ENSDART00000163668	si:ch1073-392o20.2	0.543875349	0.345011244	0.082952674	-0.090250505
ENSDART00000163669	cplx2	-0.402497387	-0.424820216	-0.158797592	0.046498934

ENSDART00000163677		-0.639905731	-0.179601491	0.106036319	0.34272584
ENSDART00000163680	hmgcs1	0.250779539	0.717953967	2.106714123	1.525845318
ENSDART00000163724	SLA (1 of many)	1.207342807	0.416124608	0.383103847	0.193096581
ENSDART00000163728	dlg1l	-0.038220111	-0.173015233	-0.306246457	-0.06107777
ENSDART00000163741	pwwp2b	0.088274726	0.335331052	0.428666425	0.318380002
ENSDART00000163753	kcnc2	-0.67860628	-0.849972581	-0.373023023	-0.287955655
ENSDART00000163791	acss2l	-0.694063426	-0.500876422	-0.115488475	-0.045115535
ENSDART00000163793	slitrk6	-0.404346501	-0.384982132	-0.24261489	-0.144542921
ENSDART00000163794	wt1b	0.454982734	0.330694147	0.317167947	0.300420298
ENSDART00000163822	glula	0.714593131	0.414537751	0.09156337	0.359301323
ENSDART00000163867	gnb1b	-0.511340157	-0.236747213	0.117718298	0.111335872
ENSDART00000163870	si:dkey-51d8.3	0.628993426	0.565032034	0.497692882	0.102165836
ENSDART00000163882	si:zfos-932h1.2	0.498808647	0.465594286	0.502820055	0.062918681
ENSDART00000163892	ldha	-0.444310122	-0.296598203	-0.299673411	-0.138453042
ENSDART00000163897	lgi1b	-0.358188713	-0.274223364	-0.009680681	0.361584537
ENSDART00000163903	kcna2b	-1.041609083	-1.150732339	-0.278604653	0.240702332
ENSDART00000163908	rnasekb	-0.236636314	-0.265732137	-0.418465587	-0.246757364
ENSDART00000163909	sepw1	1.561335076	4.635912393	4.23829642	2.739511684
ENSDART00000163930	znfx1	0.194042843	0.097168789	1.523223234	0.201252376
ENSDART00000163935	med30	-0.198525489	-0.227576976	-0.505162548	-0.348410732
ENSDART00000163951	plppr1	-0.393500272	-0.316896098	-0.148613826	0.078198855
ENSDART00000163952	zgc:110045	-0.36736834	-0.55642828	-0.201308362	-0.286026212
ENSDART00000163965	brdt	-0.081678017	-0.160102967	-0.300823041	-0.066034281
ENSDART00000163976	CABZ01069287.1	0.247964945	-0.272161916	-0.664208233	-0.362164102
ENSDART00000163998	rps6ka3a	0.01131102	-0.11493059	-0.372925255	-0.200625555
ENSDART00000164015	zgc:66483	-0.233929761	-0.339147918	-0.342271085	-0.145997916
ENSDART00000164016	kif16ba	0.136253596	0.020087235	0.515201001	0.413972136
ENSDART00000164038	SAMD14	-0.096477793	0.217514922	0.565397199	0.769428937
ENSDART00000164044	CR339041.2	1.41667761	2.386461772	3.081137029	2.622909743
ENSDART00000164055	cap2	0.292123593	0.450008213	0.705800498	0.580755871
ENSDART00000164067	coq4	-0.217054082	-0.203935963	-0.344175456	-0.073829043
ENSDART00000164086	slc25a42	-0.002109364	-0.020496378	-0.265228332	-0.112125599
ENSDART00000164095	scpp8	1.980889265	1.287952222	0.276966611	-1.560257402
ENSDART00000164097	DRAP1 (1 of many)	-0.372264093	-0.35663103	-0.955802153	-1.03240984
ENSDART00000164102	cirbpa	0.225987764	0.314210398	0.241597292	0.01082888
ENSDART00000164107	mex3b	2.977404608	3.048404514	3.190626187	2.69620274
ENSDART00000164112	si:dkey-191g9.7	-0.2514272	-0.278147864	-0.225636717	-0.211416033
ENSDART00000164113	cpeb1a	1.336706075	1.190402311	1.006023313	0.300918459
ENSDART00000164114	grb2a	-0.50918281	-0.254702947	0.293231191	0.639895729
ENSDART00000164121	mboat2b	-0.365653488	-0.229675996	-0.118902893	-0.108744854
ENSDART00000164129	asic4b	-0.92148029	-1.144921239	-0.577321683	-0.304835302
ENSDART00000164139	nhsb	-0.370661862	-0.378323584	-0.044398497	-0.049874132
ENSDART00000164141	dmd	-0.285273333	-3.746442585	-0.310755288	-0.137191678
ENSDART00000164149	si:ch211-272n13.3	-0.267513209	-0.389121708	-0.597494594	-0.154083189
ENSDART00000164160	acss1	0.721950379	0.415946373	-0.236554542	-0.337079704
ENSDART00000164161	osbpl1a	0.351337908	0.311134875	-0.016374257	-0.257205353
ENSDART00000164163	si:dkey-202l22.3	-0.676171429	-1.127391316	0.216542828	0.638796234
ENSDART00000164175	si:dkey-19c16.12	0.421774029	0.293774627	0.297672563	0.254989754
ENSDART00000164178	prrt2	-0.547052118	-0.283537507	0.297793332	0.499767197
ENSDART00000164190	ksr2	-0.449890716	-0.587945856	-0.131638865	0.158651331
ENSDART00000164198	si:dkey-102m7.3	0.949739085	1.411170301	0.641678924	0.378323778

ENSDART00000164204	ubac2	3.978074982	4.357970342	4.255380594	4.157698498
ENSDART00000164207	impdh2	0.582365155	0.675083436	1.006708284	0.417204588
ENSDART00000164210	SI	1.002064064	1.522108486	0.992417222	0.446482404
ENSDART00000164218	AKAP13 (1 of many)	0.310415201	0.00930034	-0.071769787	0.052155489
ENSDART00000164282	si:zfos-1011f11.1	1.204368625	1.598509532	2.613199623	1.667018794
ENSDART00000164298	RNF122	0.037145597	0.153249015	0.432199178	0.405886045
ENSDART00000164305	dicp1.1	0.970381914	0.35730766	0.127672979	0.007978514
ENSDART00000164311	mfsd2aa	0.188250738	0.92622451	1.277563791	0.357720797
ENSDART00000164326	si:ch73-119p20.1	-0.36329758	-0.388299747	0.050179382	0.305653209
ENSDART00000164328	mical3b	0.181716224	-0.808395946	-0.697708655	-4.388355255
ENSDART00000164353	lsg1	0.428728295	-3.620309582	-3.671353201	-0.354626047
ENSDART00000164359	rpl24	0.373088629	0.328659722	0.253068013	0.037812576
ENSDART00000164361	gcgra	-0.404304946	-0.520606663	-0.179928762	-0.009148827
ENSDART00000164390	chmp1a	0.019722961	0.342209851	0.202368709	0.087357775
ENSDART00000164392	lrrc20	0.934799406	1.852088866	1.534338919	1.07983234
ENSDART00000164440	si:ch211-195b11.3	0.887240489	0.373309939	0.100891486	-0.191092546
ENSDART00000164454	plbd1	0.523653463	0.489772378	0.050282092	-0.147404491
ENSDART00000164456	ftr50	0.637566129	0.360052404	0.566672785	0.193605767
ENSDART00000164471	CABZ01084230.2	0.442580477	0.587031258	0.401618345	0.289449509
ENSDART00000164473	MAP7	-0.064645672	-0.140135551	-0.331730838	-0.206670905
ENSDART00000164484	dtnba	0.785988532	0.671033087	0.445356623	0.140878127
ENSDART00000164506	dlg1l	-0.401430092	-0.615035177	-0.528577315	-0.282785588
ENSDART00000164543	kalrna	0.833553496	1.079150473	0.963779963	0.661385395
ENSDART00000164563	elmo1	-0.350954029	-0.242629157	-0.047540163	0.212201861
ENSDART00000164566	akt3a	0.045255643	-0.432188612	-0.498284714	-0.321504061
ENSDART00000164581	galr2b	-0.842297369	-0.875318855	-0.561173438	-0.495794792
ENSDART00000164585	mvda	-0.034972011	1.767545823	1.673143467	0.929274175
ENSDART00000164597	si:ch73-127m5.1	-0.712165198	-0.239448184	0.59846121	0.690834997
ENSDART00000164609	si:ch211-126j24.1	-0.283466546	-0.277532239	-0.146779223	-0.04114819
ENSDART00000164612	MYH7 (1 of many)	0.128311883	3.589180748	4.208970977	3.051349831
ENSDART00000164621	ndrg4	-0.368994826	-0.243279912	-0.209623349	-0.144971204
ENSDART00000164623	ptn	-0.285740219	-0.301123559	-0.395585979	-0.169574914
ENSDART00000164647	slc12a2	-0.231168401	0.356367716	0.84043135	0.654792579
ENSDART00000164650	pdk3b	-0.133813888	-0.219494437	-0.474289524	-0.225858079
ENSDART00000164653	chl1b	0.395134275	0.182602935	0.205655131	0.15670367
ENSDART00000164658	si:ch211-225h24.2	0.868137568	0.716622061	0.306228611	-0.52736976
ENSDART00000164663	aclya	-0.268277102	0.066826112	0.215179278	0.06328981
ENSDART00000164670	frem2b	-0.279819948	-0.158818402	-0.300273376	-0.936234223
ENSDART00000164692	dctn4	-0.108443255	0.034030376	0.494831818	0.293587926
ENSDART00000164693	CT583672.1	-0.40975231	-0.465896033	-0.295925202	-0.161007007
ENSDART00000164695	arl3l1	-0.208201897	-0.292220291	-0.610724074	-0.376046469
ENSDART00000164700	sptbn1	-0.093232375	0.007130054	0.336225009	0.189868537
ENSDART00000164711	NFATC2 (1 of many)	0.774520656	0.760515741	0.734404519	0.027133082
ENSDART00000164712	cobll1b	-0.06356256	-0.113578182	-0.259720894	-0.442027325
ENSDART00000164726	si:dkey-200c24.1	-0.33778778	-0.588766562	-0.504539854	-0.492073189
ENSDART00000164729	SBSPON	-0.213183167	-0.151008123	-0.380923154	-0.573582751
ENSDART00000164733	sept15	-0.353566412	-0.328984592	-0.353599969	-0.269459941
ENSDART00000164759	cntn4	-0.356168225	-0.879631419	-0.511604399	-0.608850913
ENSDART00000164771	ppfibp1a	0.181979482	0.06403475	0.045243081	-0.766863631
ENSDART00000164773	CABZ01072036.1	-0.106672866	-0.494009284	-0.613761036	-0.476277849
ENSDART00000164791	fkbp3	-0.300438451	-0.231059822	-0.287267966	-0.464270989

ENSDART00000164792	cfap74	-0.15899724	-0.307564837	-0.482816939	-0.375147114
ENSDART00000164805	camk2b2	-1.031859946	-0.873779607	-0.527330652	-0.617230087
ENSDART00000164809	si:ch73-233f7.5	0.260792098	0.492638431	0.828767158	0.768289306
ENSDART00000164810	ano2	0.191725223	-0.262322186	-0.177000117	-0.045593369
ENSDART00000164816	cnr1	0.971414159	0.97686046	0.907214473	-0.258209562
ENSDART00000164844	rictora	-0.308713132	-0.348726405	-0.242009414	-0.189747444
ENSDART00000164853	cnp	1.119904973	1.074260191	2.53120974	1.692544703
ENSDART00000164855	crebl2	-0.127345071	-0.2122205	-0.432997567	-0.264872881
ENSDART00000164879	slc8a2b	-0.298961891	-0.426517442	-0.175774312	0.049460205
ENSDART00000164890	pxdc1a	-0.553855303	-0.268569574	-0.515208528	-0.163588555
ENSDART00000164891	trim25	1.17805401	1.473587562	2.17710407	1.406205223
ENSDART00000164902	ca9	0.158296773	0.100302393	-0.407653731	-0.624825129
ENSDART00000164904	nrp1a	0.238338546	0.288203349	0.415887875	0.403021552
ENSDART00000164928	mab21l1	-0.236289538	-0.264159199	-0.014378888	0.140356353
ENSDART00000164979	ARHGAP44 (1 of many)	-0.298736282	-0.611440452	-0.190082373	-0.04950856
ENSDART00000164982	cdh4	-0.415355609	-0.489911794	0.041403227	0.334639192
ENSDART00000164983	anapc15	-0.036185378	-0.198619926	-0.408385072	-0.233599439
ENSDART00000164988	bod1l1	0.085003082	0.094605568	0.323418829	0.18061312
ENSDART00000164989	si:ch211-121j5.4	-2.541662275	-0.597024815	-0.157115726	-0.615748868
ENSDART00000165000	zgc:136767	1.074177299	1.362099519	1.806009349	1.052151095
ENSDART00000165002	aqp11	-0.052004039	-0.030290396	-0.282964979	-0.265886582
ENSDART00000165004	gria3b	-0.410652366	-0.638891942	-0.274205904	0.132979921
ENSDART00000165006	hpca	-0.709011759	-0.754814005	-0.136865422	-0.15729929
ENSDART00000165018	cdc42se2	-0.053295614	-0.137825766	-0.478383249	-0.349419762
ENSDART00000165021	ndrg2	-0.572041099	-0.272188031	0.107233459	0.07090542
ENSDART00000165030	si:dkeyp-9d4.3	-0.136094032	-0.353586884	-0.269174482	-0.139574898
ENSDART00000165031	nr2f6b	0.621146984	0.406827879	0.54917158	0.373267529
ENSDART00000165049	impg2b	-0.010489295	-0.310856751	-0.727501593	-0.334825186
ENSDART00000165058	rims2a	-0.759006031	-0.607036719	-0.257997898	-0.111766909
ENSDART00000165065	uqcr10	-0.222678585	-0.318669731	-0.412855116	-0.201529958
ENSDART00000165066	eif2ak2	0.145136508	0.290335878	1.008511718	0.199274727
ENSDART00000165082	ppp1r1b	-0.592550768	-0.433249399	0.132918309	0.038849346
ENSDART00000165097	unm_sa1614	-0.093711156	-0.459181727	-0.424406225	-0.126303047
ENSDART00000165108	jph3	-0.254573235	-0.426555215	0.040987467	0.08534723
ENSDART00000165115	adcy3a	-0.345722559	-0.440118918	-0.380340005	-0.11083624
ENSDART00000165120	purab	-0.246648902	-0.188751457	-0.157104845	-0.035763175
ENSDART00000165124	si:ch73-213k20.5	-0.366310901	-0.428402723	-0.397554725	-0.148030091
ENSDART00000165141	elavl4	1.008784658	0.753903544	1.037731482	0.21557706
ENSDART00000165147	MFSD3	-0.380785464	-0.635551022	-0.165628582	-0.053496107
ENSDART00000165156	sept15	-0.316165192	-0.356420975	-0.295749689	-0.213768729
ENSDART00000165158	iqsec3a	-0.287263936	-0.339420666	-0.160997893	0.073213089
ENSDART00000165159	dscaml1	-0.229621803	-0.384977161	-0.055090744	0.170582623
ENSDART00000165186	si:dkey-33i11.9	1.335261857	1.027980768	0.465372707	0.114592431
ENSDART00000165195	CU633762.1	-0.318810448	-0.187254152	-0.150226726	-0.009770113
ENSDART00000165199	mapre2	0.262487966	1.260089875	1.425017722	0.706527865
ENSDART00000165201	pacsin3	-0.207834592	-0.16294471	-0.3385586	-0.115392716
ENSDART00000165207	fam160a1b	-0.089233769	-0.189976289	-0.269606776	-0.116139265
ENSDART00000165213	caskb	-0.0150989	0.201636443	0.432584514	0.386918381
ENSDART00000165216	dph5	0.422816122	0.515360344	0.448987609	0.161864292
ENSDART00000165223	pbx1b	-0.243991197	-0.377373898	-0.18693501	0.141565589
ENSDART00000165225	prkar1ab	-0.220219796	-0.315739009	-0.190831632	-0.05456146

ENSDART00000165228	kif5aa	-0.119845449	0.354985416	0.468393446	0.466605496
ENSDART00000165230	map4k4	-0.058109942	0.098582575	0.424133469	0.341075529
ENSDART00000165290	cyb5a	0.19405994	0.064877953	-0.350823438	-0.576368917
ENSDART00000165292	nsmfb	-0.267190978	-0.447188182	-0.263868703	-0.115307671
ENSDART00000165308	me2	-0.16075229	-0.327815721	-0.451710296	-0.323851706
ENSDART00000165309	pmt	1.560743592	2.051643174	1.965781873	1.006007351
ENSDART00000165318	thsd7bb	-0.173014975	0.179455909	1.051340912	1.022924373
ENSDART00000165326	adgrl2a	-0.346797592	-1.122813298	-2.2772027	-0.600399475
ENSDART00000165333	si:ch211-207l14.1	-0.144575592	-0.337549275	-0.714109722	-0.444634079
ENSDART00000165342	si:ch211-93f2.1	-0.158550393	0.117683559	-0.256320731	-0.510696237
ENSDART00000165370	nxph2b	-0.350612818	-0.344729721	-0.406615862	-0.05363573
ENSDART00000165400	slc1a2b	-0.437547383	-0.373563248	-0.176045295	-0.026222695
ENSDART00000165411		-0.599585032	-0.699498667	-0.398460986	-0.09881633
ENSDART00000165420	si:dkey-161j23.5	-0.418752064	-0.09080604	0.112517016	0.202865451
ENSDART00000165423	abcc8b	-0.169557865	-0.54863705	-0.322687745	-0.280461618
ENSDART00000165425	aak1a	-0.009756971	-0.269537246	-0.43668389	-0.03607957
ENSDART00000165427	myt1b	0.19969921	0.396205375	0.347986667	0.198976782
ENSDART00000165433	rgs2	0.779425284	0.385417705	0.555491331	-0.186899017
ENSDART00000165437	si:dkey-26m3.3	0.220558358	0.221546162	0.282286139	0.194663631
ENSDART00000165443	zgc:153615	-0.585165411	-0.400257189	-0.265863973	-0.020005469
ENSDART00000165448	rims2b	-0.321612136	-0.490793078	-0.295423658	0.030315799
ENSDART00000165453	epb41l3a	-0.480811799	0.184033911	0.640683351	0.633983333
ENSDART00000165454	CU915762.1	0.350263787	0.108189136	-0.057165889	0.160983657
ENSDART00000165472	snx2	0.34033168	-0.040635813	0.394688984	0.105073801
ENSDART00000165479	has3	0.356722134	0.209377149	0.150402808	0.132473433
ENSDART00000165484	jpt1a	0.363755224	1.1796439	1.211655345	0.942198247
ENSDART00000165486	rps18	0.252319024	0.42379942	0.281645226	-0.028370317
ENSDART00000165491	BX294383.1	0.352047768	0.065820831	0.027639622	0.019046581
ENSDART00000165499	rps17	0.504096498	0.637791027	0.385574489	0.024446186
ENSDART00000165541	map7d1b	0.274410575	0.322558005	0.501220001	0.438496507
ENSDART00000165542	si:dkeyp-72e1.9	0.115590472	-0.201422352	-0.774050016	-0.351374892
ENSDART00000165547	CABZ01045062.1	-0.214219167	-0.28679968	-0.112596272	0.066374108
ENSDART00000165548	ap2m1a	-0.100883554	-0.260493779	-0.290981943	-0.160008883
ENSDART00000165557	BX088524.3	-0.275785171	-0.331787981	-0.469187171	-0.127928431
ENSDART00000165570	rgs3a	-0.252183908	-0.25277418	-0.390798302	-0.347427277
ENSDART00000165572	zgc:162193	0.654407013	0.606984165	0.583589232	0.369009896
ENSDART00000165594	CT030188.1	0.949647945	1.541943795	1.047931527	0.898450066
ENSDART00000165609	barhl2	-0.31272019	-0.374018735	0.126371762	0.224852149
ENSDART00000165628	il4r.1	2.521820035	2.797213431	2.312532052	1.674117298
ENSDART00000165638	pax10	-0.459647113	-0.417992165	-0.200180992	-0.120970793
ENSDART00000165654	atp1b2a	-0.572829136	-0.493634763	-0.065881564	0.075479325
ENSDART00000165656	mxd3	0.030444632	0.928898979	1.014066924	0.787179484
ENSDART00000165659	epb41l3a	-0.249067696	-0.637410719	-0.072556493	-0.093775549
ENSDART00000165680	ntn4	0.240096357	0.070794746	-0.41629651	-0.558332982
ENSDART00000165698	pbx1a	-0.205212048	-0.330154536	0.047550564	0.321002436
ENSDART00000165710	gpbp1l1	-0.164201122	-0.182753901	-0.290062625	-0.125147623
ENSDART00000165715	BX284638.1	0.544886732	0.81514457	1.035521411	0.49952411
ENSDART00000165735	mdm4	0.677008086	0.457427602	0.365768719	0.56900659
ENSDART00000165743	gabrg2	-0.320626657	-0.470320689	-0.258833602	0.01857369
ENSDART00000165744	kif1b	-0.018685925	-0.126032816	0.340319561	0.347744455
ENSDART00000165757	pax6b	-1.030621281	-0.736504391	-0.510180699	-0.112500923

ENSDART00000165774	pax6a	-0.382942423	-0.218376905	0.094924321	0.282463268
ENSDART00000165775	nlrc3	2.263794952	2.415644973	1.694313933	0.888951658
ENSDART00000165785	pcdh10a	-0.2607204	-0.385620436	-0.040787194	0.144409091
ENSDART00000165824	setdb1b	0.086359323	0.217956862	0.320141564	0.188338727
ENSDART00000165835	ddx54	0.300757648	0.157122409	0.421066488	0.21493331
ENSDART00000165864	acox3	-0.089913641	-0.178492527	-0.263049665	-0.100985253
ENSDART00000165875	csnk1g1	0.004513388	0.131360351	0.256793811	0.293233915
ENSDART00000165877	purg	-0.159238984	-0.087175961	1.048436006	0.831059287
ENSDART00000165883	opn9	-0.186957867	-0.12243997	-0.333236797	-0.10321317
ENSDART00000165887	flvcr1	-0.333518999	-0.053749594	0.204875088	0.015969559
ENSDART00000165898	gbe1b	-0.311269447	-0.395300505	-0.303280984	-0.17462698
ENSDART00000165903	slmapa	-5.974312545	-3.688098031	-4.583524263	-0.943141088
ENSDART00000165912	si:ch73-380n15.2	-0.521676191	-0.515528635	-0.335328887	-0.345110245
ENSDART00000165920	nucb1	0.353439048	0.296422234	0.314532166	-0.093049018
ENSDART00000165932	sik1	1.156253383	0.676084919	0.687880039	1.28219737
ENSDART00000165938	CU466240.1	0.428025361	0.165679287	0.139754855	0.298251046
ENSDART00000165943	fam102aa	0.068136883	0.160885629	-0.464613828	-0.136577362
ENSDART00000165949	fahd2a	0.346683888	0.20577135	-0.135781443	-0.151237654
ENSDART00000165955	zhx3	-0.011624436	-0.109014479	0.013312322	0.351264725
ENSDART00000165973	npnt	-0.201530974	-0.119421961	-0.271951683	-0.242671185
ENSDART00000165974	agla	-0.253110547	-0.164938699	-0.089751197	0.189815825
ENSDART00000165979	sncgb	-0.522103986	-0.516181722	-0.379782599	-0.140861461
ENSDART00000165987	DST	0.207553536	0.19383587	0.337156934	0.233508941
ENSDART00000165991	lect1	-3.06408831	-0.430314435	0.807667083	0.659541685
ENSDART00000165993	f3a	1.175718098	1.946056838	1.259834727	0.15077318
ENSDART00000166025	irs2a	-0.31567938	-0.330763757	-0.333387068	-0.269837586
ENSDART00000166027	trpc1	-0.303725684	-0.42864481	-0.334157939	-0.231516525
ENSDART00000166028	mcf2la	-0.298550829	-0.194248433	-0.224063256	-0.02772208
ENSDART00000166040	sh3bp5b	0.359354612	1.105816025	1.101214783	0.69319763
ENSDART00000166042	vipr2	-0.341756427	-0.277003119	-0.379732317	-0.038627099
ENSDART00000166058	cmah	1.523029746	4.3199312	4.414697946	2.901009315
ENSDART00000166086	CABZ01055522.1	-0.167914645	-0.1533494	-0.033990591	0.313482965
ENSDART00000166101	tlr22	0.744049575	0.528297207	0.210656038	-0.353063092
ENSDART00000166105	frem1a	-0.140882698	-0.093532592	-0.525495905	-1.018483252
ENSDART00000166110	itga4	0.891623018	0.709388891	0.523610434	-0.253853847
ENSDART00000166114	sema3ab	-0.726704016	-0.305486009	-0.172433457	-0.053903823
ENSDART00000166120	si:ch211-212d10.1	1.398436882	1.472487792	1.228480368	0.254562361
ENSDART00000166135	zbtb47b	-0.402709337	-0.409852132	-0.474957603	-0.394147628
ENSDART00000166148	gabra1	-0.578656795	-0.788703398	-0.59715214	-0.072809071
ENSDART00000166152	DAB2 (1 of many)	0.967097608	0.419929333	0.22593558	-0.403858057
ENSDART00000166174	PARP12	0.236086028	0.041618587	1.350827793	0.15615804
ENSDART00000166175	zgc:171534	0.978558952	0.553291836	0.353063819	-0.014246545
ENSDART00000166177	impg2b	0.006257085	-0.447154942	-0.735592553	-0.255871486
ENSDART00000166192	pik3r5	0.977110656	0.482953861	0.341342874	-0.116377345
ENSDART00000166209	wu:fb44b02	0.715207055	0.457109376	0.301906263	-0.147536436
ENSDART00000166213	lcorl	-0.114650844	-0.153895276	-0.40050268	-0.037010303
ENSDART00000166224	smyhc2	1.194842223	5.367086788	6.852910143	4.718916943
ENSDART00000166241	inpp5b	-0.146961161	-0.217731387	-0.46875304	-0.117265313
ENSDART00000166242		0.20194611	0.241162188	0.512911525	0.246398136
ENSDART00000166246	ssuh2rs1	0.375757195	0.261052668	0.200946491	-0.016238014
ENSDART00000166254	gpn2	0.085397213	0.349689217	0.008215986	-0.050534057

ENSDART00000166259	wars	0.30804143	0.479087994	0.526413933	0.245904587
ENSDART00000166268	YTHDC2	0.015267861	-0.127181664	0.395706164	0.317645369
ENSDART00000166274	phlda1	-0.962333929	-0.775569749	0.008452009	0.660576028
ENSDART00000166308	cib2	-0.434731659	-0.335262372	-0.470437684	-0.240772493
ENSDART00000166313	thrb	-0.193382381	-0.415286369	-0.400030228	-0.136855946
ENSDART00000166317	mtus1b	-0.441732874	-0.537161422	0.305687832	0.223661346
ENSDART00000166324	ctnnd1	0.460487451	0.641759187	1.004918197	0.346719852
ENSDART00000166341	AL954715.1	0.715525501	0.583886882	0.488572453	0.64332223
ENSDART00000166351	nkrf	-0.332043429	-0.213944917	-0.117297764	-0.03436019
ENSDART00000166372	si:ch211-222l21.1	0.890920299	0.959321743	0.886208086	0.413211736
ENSDART00000166374	si:dkey-31f5.11	-0.246069029	-0.257528931	-0.445065012	-0.224168767
ENSDART00000166387	prickle2a	-0.236957906	-0.307926948	-0.968400315	-0.433283323
ENSDART00000166388	il6st	0.326944598	0.248050944	0.233084281	-0.066517896
ENSDART00000166395	fcer1gl	0.672549379	0.370716598	-0.127334281	-0.409142814
ENSDART00000166432	slc2a8	0.33290564	0.367124099	0.471066456	0.250147846
ENSDART00000166435	si:ch73-28h20.1	-0.187153724	-0.333881358	-0.353210004	-0.031764093
ENSDART00000166449	pik3r3a	-0.698577911	-0.3007013	-0.248394691	-0.547713549
ENSDART00000166463	cnot6b	-0.099106983	-0.163429858	-0.300924183	-0.105283807
ENSDART00000166470	CU855947.1	-0.432778868	-0.336734093	-0.219445916	-0.079633712
ENSDART00000166496	cat	0.249947463	0.551180864	0.073389354	-0.18327225
ENSDART00000166502	satb2	-0.540440017	-0.158642442	1.216799418	1.496340928
ENSDART00000166504	sod3b	1.107778201	3.874418447	1.285382343	3.248105088
ENSDART00000166508	fdft1	-0.467410589	-0.45021858	0.057865823	-0.125768039
ENSDART00000166515	si:dkeyp-57d7.4	-0.099733783	-0.492859873	-1.003446185	-0.404973513
ENSDART00000166518	ptprfb	0.067979508	-0.359782965	0.005118486	0.040886074
ENSDART00000166527	ftr87	-0.180960216	-0.104077306	-0.471569991	-0.343688512
ENSDART00000166531	pcdh15b	-0.215971807	-0.468745045	-0.336826359	0.084344716
ENSDART00000166533	zgc:123181	-0.682536387	-0.078871471	0.212564109	0.407498801
ENSDART00000166540	kcnip3b	-0.988549414	-0.704807394	-0.278733631	0.119291619
ENSDART00000166560	hpdl	0.319918962	0.542121436	0.616518616	0.488982996
ENSDART00000166561	ttc38	0.445202505	0.288306634	-0.06639877	-0.156646921
ENSDART00000166566	dgkh	-1.438871843	-1.330275705	-0.819153793	-0.068934495
ENSDART00000166575	ppp3ca	-0.493387865	-0.574192875	0.281204144	0.517021392
ENSDART00000166580	pak1	0.254456198	0.505135694	0.67991388	0.523148639
ENSDART00000166587	rhbdl3	1.011972997	0.720471419	0.019687653	-0.322773745
ENSDART00000166591	utp20	0.378085419	0.246513796	0.276819854	0.15697444
ENSDART00000166607	RAPGEF4 (1 of many)	-0.233842979	0.163001528	0.421327636	0.212581301
ENSDART00000166615	dnm2b	0.605203252	0.413218412	0.436607775	-0.016254619
ENSDART00000166618	slc43a3a	0.880026964	0.262110574	0.534125771	0.347202731
ENSDART00000166622	si:dkey-77g12.1	1.256692313	2.663525112	1.501030031	-0.081810387
ENSDART00000166632	si:zfos-741a10.3	1.2129476	0.572994738	0.242126634	0.029899239
ENSDART00000166634	rnf213b	0.322643691	-0.072602668	2.242716781	-0.029575481
ENSDART00000166645	HTR7 (1 of many)	-0.91335189	-0.965307725	-0.027473732	0.473063601
ENSDART00000166648		0.095481351	-0.09266769	0.456407618	0.379239728
ENSDART00000166650	bsg	-0.333252581	-0.445374368	-0.502358398	-0.336861466
ENSDART00000166659	lrrn2	-0.321629641	-0.404637061	-0.652990901	-0.235544369
ENSDART00000166664	MIDN (1 of many)	0.609413165	0.740169365	0.964140975	0.212765964
ENSDART00000166681	frrs1l	-0.4975582	-0.505651734	-0.29386319	-0.136005256
ENSDART00000166690	gadd45ga	1.824520476	2.195526461	0.938941318	1.401631316
ENSDART00000166693		0.184413566	-0.110459993	1.581805418	0.290855458
ENSDART00000166698	lrsam1	0.035853538	0.184404018	0.265787714	0.064864391

ENSDART00000166714	impg2b	-0.072742859	-0.391473223	-0.740015258	-0.360324281
ENSDART00000166724	myom1b	0.206144619	0.433459222	0.498383662	0.501410774
ENSDART00000166725	si:dkey-27j5.9	1.657914875	0.40212862	0.542892045	0.141569929
ENSDART00000166730	slitrk4	-0.433556166	-0.537605748	-0.487948345	-0.301835783
ENSDART00000166739	si:zfos-2326c3.2	0.356689661	0.198077273	0.030512595	-0.103105863
ENSDART00000166755	tril	1.481556618	0.917097925	0.535942919	0.363878102
ENSDART00000166756	mfsd11	0.314593735	0.230184492	0.795700169	0.565917277
ENSDART00000166829		-0.799969413	-0.359199835	-0.712167572	-2.039500454
ENSDART00000166843	tom1	-0.282793851	-0.146845245	-0.347923357	-0.109554429
ENSDART00000166889	nptna	-0.194507678	-0.523176341	-0.288117341	0.166715743
ENSDART00000166894	si:dkey-16p6.1	-3.041565726	-0.566863861	-1.581438015	-0.461000528
ENSDART00000166904	AL840638.1	-0.172150655	-0.246843461	-0.35641848	-0.195598477
ENSDART00000166957	purba	-0.28509761	-0.466147452	-0.146018067	-0.191067595
ENSDART00000166981	snx8b	-0.518943385	-0.495275193	-0.13677988	-0.169307474
ENSDART00000166992	rab6a	0.863374482	0.610837796	0.548836905	0.594870039
ENSDART00000167001	cited4b	-0.183655613	-0.263694381	-0.243829292	-0.142076267
ENSDART00000167025	si:dkey-203a12.7	1.830928705	3.142170624	2.521496169	0.613627975
ENSDART00000167032	tp53rk	0.299937927	0.221076682	0.117069539	0.10102477
ENSDART00000167035	mibp2	0.52185156	0.897496666	1.068299106	0.608631566
ENSDART00000167040	pop1	0.818813588	0.594298576	0.588084483	0.531688582
ENSDART00000167052	etv1	-0.327639473	-0.469002174	-0.32895701	-0.22464313
ENSDART00000167062	SNORD49	0.825973685	1.057295844	0.696610061	0.75374548
ENSDART00000167068	kifap3b	0.204750718	0.408132617	0.569098043	0.272936415
ENSDART00000167074	irf2	0.133065149	0.222164622	0.608761767	-0.104617341
ENSDART00000167099	CU459186.5	0.808307931	1.173553726	0.835552808	0.510196285
ENSDART00000167117	si:ch1073-469d17.2	-0.226355895	-0.436402154	-0.568359696	-0.320345187
ENSDART00000167128	pargl	0.159346244	0.133314978	0.619065571	-0.065913899
ENSDART00000167132	isg20l2	0.391451776	0.35980411	0.120415307	0.040629954
ENSDART00000167143	glyr1	-0.150233074	-0.208555766	-0.274679248	-0.191034867
ENSDART00000167145	ap1ar	0.285895558	0.368048827	0.350688731	0.119518374
ENSDART00000167154	trim9	-0.216985401	-0.363840661	-0.237534447	-0.020888972
ENSDART00000167164	MPV17L	-0.408335699	-0.461800821	-0.169486828	-0.082955483
ENSDART00000167177	ccnf	1.106246818	1.046935028	0.737028811	0.1071592
ENSDART00000167179	asf1ba	-0.325814646	-0.52479315	-0.773177729	-0.605632897
ENSDART00000167197	spata18	-0.430548228	-0.388964562	-0.077452756	-0.001886549
ENSDART00000167219	pcdh1g26	-0.056136761	0.084950376	0.428942573	0.447913813
ENSDART00000167226	pycr1b	0.1083633	0.252664475	0.730734657	0.419591074
ENSDART00000167279	klhl6	0.921934193	0.563560813	0.364277897	0.100202901
ENSDART00000167306	ldb2b	-0.838228207	-0.415457403	-0.325734782	0.110687477
ENSDART00000167322	si:ch73-233f7.3	-0.088252023	0.25632847	0.562157447	0.392412002
ENSDART00000167324	ebf3a	-0.585714789	-0.243731213	0.563349846	0.843370215
ENSDART00000167330	CU179758.1	-0.385159517	-0.273959547	-0.55992419	-0.56237529
ENSDART00000167355	nsfa	-0.252585329	-0.369691062	-0.484691854	-0.137375398
ENSDART00000167359	dusp27	1.665789827	1.31173846	1.624125812	1.13571557
ENSDART00000167388	vps33a	0.155328285	0.245633599	0.343008348	0.290252576
ENSDART00000167391	arhgap21a	-0.385966805	-0.235029359	-0.527723306	-0.308128762
ENSDART00000167423	si:dkey-242k1.6	-0.388268724	-0.337313048	-0.259026873	-0.237349722
ENSDART00000167430	lrrc24	-0.125265238	-0.400040145	-0.093389228	0.002539389
ENSDART00000167444		-0.313613371	0.27829227	0.501895099	0.307445871
ENSDART00000167449	sept15	-0.329936507	-0.127540958	-0.290094259	-0.186050001
ENSDART00000167451		-0.147201966	-0.622186255	-0.321157937	-0.196934609

ENSDART00000167461	BX897691.1	1.652413387	2.158428192	1.996842668	1.949150718
ENSDART00000167464	GNAZ	-0.341892906	-0.155068478	0.102581405	0.079027974
ENSDART00000167468	prpsap1	0.532862733	0.121505744	0.13944499	-0.034080691
ENSDART00000167502	CR855860.1	-0.377799337	-0.542912736	-0.423594114	-0.115674527
ENSDART00000167506	scg2a	-0.457333464	-0.29124415	-0.144155502	-0.510044484
ENSDART00000167514	abca1a	0.214033988	0.130340273	0.268396538	-0.115766571
ENSDART00000167523	dixdc1b	-0.34575392	-0.259524065	-0.191239322	-0.198083451
ENSDART00000167570	actr3b	-0.361119463	-0.449811578	-0.822009016	-0.639517901
ENSDART00000167584	aldh2.1	-0.136059736	-0.055428642	-0.441396328	-0.687740935
ENSDART00000167612	rnf34a	-0.125673437	-0.210904428	-0.512991238	-0.24247481
ENSDART00000167613	hmgcs1	-0.061825835	0.154559892	0.893983325	0.609306628
ENSDART00000167649	pik3r3a	-0.582543495	-0.143058653	-0.251972796	-0.459052361
ENSDART00000167660	pja2	-0.078344887	-0.326712885	-0.382150009	-0.239022195
ENSDART00000167664	atxn1a	-0.182914727	-0.487043114	-0.17911118	-0.100136551
ENSDART00000167666	dnajc21	0.569585132	0.384796247	0.537599141	0.160116436
ENSDART00000167667	FKBP15 (1 of many)	0.292301754	0.132288424	0.122963959	-0.01266455
ENSDART00000167696	si:ch73-233f7.4	0.264747026	0.489460093	0.625111029	0.721718733
ENSDART00000167726	RYR2	-0.554726037	-0.413134766	0.099586067	0.289722828
ENSDART00000167748	fbn2b	1.02333483	0.65734038	0.922883374	0.19244827
ENSDART00000167786		0.801350724	0.147008366	0.081187044	0.322292312
ENSDART00000167818	atp2b2	-0.603266889	-0.926553396	0.035397356	0.064456748
ENSDART00000167823	CU651662.1	0.332182102	0.108414441	-0.278380094	-0.999717307
ENSDART00000167824	timp4.3	-0.389539203	-0.013931581	-0.399789503	-0.590224284
ENSDART00000167847	eef1a1l2	-0.649781637	-0.501730111	-0.557800044	-0.257462283
ENSDART00000167861	cox4i1l	-0.902856097	-1.26820767	-1.375291282	-0.702334306
ENSDART00000167869		-3.349211506	-1.484996272	-1.5614357	-0.542819931
ENSDART00000167873	baiap2b	-0.183691734	-0.21009419	-0.266222434	-0.177287211
ENSDART00000167879	Metazoa_SRP	3.268404605	5.790076	5.563915837	5.137733652
ENSDART00000167881		0.345756532	0.215047296	0.187007292	0.450693648
ENSDART00000167937	p4hb	0.35105827	0.247669444	-0.015299318	-0.184059227
ENSDART00000167947	CABZ01079480.1	0.418778383	0.234022686	1.236946888	0.318728833
ENSDART00000167948	hcn1	-0.135796002	-0.504326543	-0.514845616	-0.152463147
ENSDART00000167956	tspan18a	-0.226187052	-0.301324705	-0.40566671	-0.255395447
ENSDART00000167963	nrg1	-0.278608256	-0.388339035	-0.420420553	-0.389329729
ENSDART00000167971	rps18	0.367747716	0.388045404	0.209564172	-0.058761163
ENSDART00000167977	kcnh4b	-0.815041676	-0.830195363	-1.200187818	-0.588368652
ENSDART00000167986	hadhb	0.310832473	0.238522601	0.004510639	-0.025410475
ENSDART00000167995	napba	-0.341681302	-0.334450461	-0.176497327	-0.027366162
ENSDART00000168002	laptm5	1.090713638	0.609708871	0.327355694	-0.215403696
ENSDART00000168004	rps18	0.800703549	0.805799198	0.576669388	0.538808303
ENSDART00000168028	si:dkey-225f23.5	-0.209864951	-0.328595081	-0.21349874	-0.05336952
ENSDART00000168036	rdh8b	-0.188576333	-0.341040166	-0.496499443	-0.09323063
ENSDART00000168038	edil3a	-0.585018977	-0.697545399	-0.17495912	0.33520597
ENSDART00000168083	Metazoa_SRP	1.497191597	1.645034783	1.435853831	0.615781715
ENSDART00000168084	scp2b	-0.093323025	-0.125270421	-0.400440129	-0.264110493
ENSDART00000168089	cyp27a7	-0.742372496	-0.705345887	-0.793489907	-0.543448957
ENSDART00000168107	crb2b	-0.111814011	-0.354478217	-0.285757255	0.093257061
ENSDART00000168121	lzts3a	-0.348828928	-0.543883063	-0.164874094	0.093345065
ENSDART00000168132	si:ch1073-82l19.1	-0.151537059	-0.297036072	-0.500517261	-2.428975421
ENSDART00000168155	si:ch211-194m7.4	2.689408284	1.434584454	0.930290361	-0.08573457
ENSDART00000168157	mkrn2os.2	0.50354684	0.675600001	1.137753613	0.994948824

ENSDART00000168160	pip5k1cb	-0.047296457	-0.437985038	-0.478356963	-0.284131261
ENSDART00000168162	FQ323156.1	-0.536894775	-1.163960845	-0.494433057	-0.466334383
ENSDART00000168163	hnrnpabb	0.035490772	0.022906035	0.318157336	0.096428682
ENSDART00000168167	rtcb	0.060457492	0.250042841	0.449158496	0.303788278
ENSDART00000168170	ttc39c	-0.270510539	-0.264194482	-0.052910688	-0.079707834
ENSDART00000168188	mettl1	0.565360225	0.37588106	0.377233069	0.251655436
ENSDART00000168201	golim4a	-0.134763119	-0.005515007	-0.245821925	-1.172515705
ENSDART00000168218	CABZ01111953.1	-0.582414072	-0.415065608	0.00894593	0.020084563
ENSDART00000168228	tmem184a	-0.3188994	-0.465602708	-0.713259634	-0.580015552
ENSDART00000168241	tubb2b	-0.194997441	0.285540167	0.888424822	0.706942534
ENSDART00000168246	spryd3	-0.145299627	-0.212114286	-0.257834151	-0.152966783
ENSDART00000168270	CABZ01048053.1	-0.515099411	-0.533680852	0.225344787	0.362581791
ENSDART00000168276	scarb1	-3.646800721	-2.308559886	0.370802474	0.243200954
ENSDART00000168277	boka	-0.236090037	-0.162724249	-0.56155479	-0.509356169
ENSDART00000168278	si:ch211-276i12.4	-0.631335043	-0.270754342	-0.641142033	-0.623314164
ENSDART00000168309	trim25	1.908271479	1.980136553	2.767316449	1.926127968
ENSDART00000168310	acap3b	-0.431660294	-0.622927148	-0.474200982	-0.250587028
ENSDART00000168317	apba1a	-0.329639415	-0.090034986	0.20399901	0.390106315
ENSDART00000168371	mibp	1.064831288	1.384792727	1.720455753	0.84273718
ENSDART00000168376	Metazoa_SRP	0.77672172	1.175457852	1.304833035	1.047872554
ENSDART00000168377	thrap3b	0.204741923	-0.103859097	0.348419918	0.136670023
ENSDART00000168396	FO904869.1	-0.490668054	-0.607434677	-0.194530972	0.12309886
ENSDART00000168419	rsu1	0.608707901	0.588635147	0.668370486	0.283061431
ENSDART00000168453	slc43a2a	-0.36850619	-0.532753158	0.198651765	0.248433931
ENSDART00000168472	creb5a	2.212193939	1.443174599	0.809493268	0.247644913
ENSDART00000168483	si:dkey-16p21.7	0.12768447	0.414452748	0.453712173	0.342486315
ENSDART00000168497	eef2b	0.448731314	0.547292163	0.7539331	0.364287935
ENSDART00000168518	asic4b	-0.92148029	-1.144921239	-0.577321683	-0.304835302
ENSDART00000168531	irf2bp2b	-0.128403316	-0.188582728	-0.404745176	-0.138034767
ENSDART00000168534	NPFFR2 (1 of many)	-0.514269049	-0.40572501	-0.533037042	-0.336902595
ENSDART00000168542	arf3a	0.034708072	0.783187174	0.81852048	0.521503928
ENSDART00000168556	ostf1	1.22625882	1.436442197	0.76765034	0.887655813
ENSDART00000168559	ttf2	-3.902752419	0.990904721	1.020977359	0.830250106
ENSDART00000168565	txnbb	0.43423077	0.785550255	1.235069922	1.025671009
ENSDART00000168568	CU861477.1	-0.399340079	-0.694343635	-0.183948068	0.054030168
ENSDART00000168608	rnaset2	1.085971834	0.685727013	0.397141355	0.382009137
ENSDART00000168610	si:ch211-283g2.2	1.147251625	1.274539863	1.168500693	0.162307024
ENSDART00000168616	ppa1a	0.095923853	-0.258115212	-0.556184684	-0.293684673
ENSDART00000168627	zfyve9a	-0.287315941	-0.130733792	-0.157388884	-0.011640067
ENSDART00000168631	cacna1hb	-0.565978595	-0.722362792	-0.435741841	-0.029665329
ENSDART00000168633	CABZ01085700.1	-0.53401794	-0.080184443	0.667052745	0.803861054
ENSDART00000168639	cry1ab	-0.18716056	-0.280815639	-0.387838008	-0.275846695
ENSDART00000168641	CT030188.1	0.86349998	1.431982341	1.038679795	-0.017052584
ENSDART00000168653	fam110b	-0.266298706	-0.306955651	-0.224541228	-0.085175606
ENSDART00000168683	lrrc8c	0.403272924	0.771214032	0.877257507	0.389191376
ENSDART00000168698	ostf1	0.435343641	0.254095599	-0.055556308	-0.273490651
ENSDART00000168705	si:ch73-103b11.2	-0.165771907	-0.231080784	-0.260448742	-0.241729977
ENSDART00000168718	chrm5a	-0.32077931	-0.748869105	-0.317792694	-0.07500967
ENSDART00000168727	zgc:64065	0.439077495	0.173893761	0.133684815	0.136149811
ENSDART00000168729	fam196ab	-0.54578901	-0.555866325	-0.405243034	0.063979844
ENSDART00000168749	nlgn3a	-0.417078437	-0.490224096	-0.240516544	-0.086053557

ENSDART00000168750	smarca1	-0.162085202	-0.116464254	-0.382463649	-0.380530416
ENSDART00000168754	cacnb2a	-0.312716955	-0.298709564	-0.211334034	-0.110938146
ENSDART00000168778	stt3a	0.3508706	0.104695298	-0.028500788	-0.125020463
ENSDART00000168799	ggps1	0.829174536	0.521929251	0.538342651	0.450861168
ENSDART00000168805	kcnk9	-0.553605245	-0.952161118	-0.308244106	0.289642563
ENSDART00000168821	CDH22	-0.242258311	-0.624670054	-0.303879761	-0.070304381
ENSDART00000168822	pabpc4	1.120879979	1.597763229	2.131974577	1.975353129
ENSDART00000168831	si:ch73-158p21.3	1.161771941	0.892729958	0.502592433	0.147950065
ENSDART00000168840	rpl35a	0.357031004	0.320334573	0.163051637	-0.036511823
ENSDART00000168849	si:ch211-147m6.2	1.772352788	0.845008987	-0.374275663	-0.738910487
ENSDART00000168850	taok1b	-0.044686095	-0.116318692	-0.429811181	-0.132610503
ENSDART00000168851	rab11fip2	0.212709727	0.193153756	0.330809666	0.288597218
ENSDART00000168876	si:dkey-81h8.1	-0.18803818	0.217329319	0.889109393	1.046963425
ENSDART00000168885	nrg1	-0.78621851	-0.719452399	0.167065606	0.289901187
ENSDART00000168898	hspa8	1.373917849	0.861861784	0.886683158	0.865443603
ENSDART00000168899	pcdh1g33	-0.406478127	-0.012838877	0.30409891	0.306864798
ENSDART00000168902	Metazoa_SRP	0.908254207	1.60749636	1.079432418	0.706761496
ENSDART00000168910	ablim3	-0.457548186	-0.818398336	-0.469699072	-0.283516024
ENSDART00000168913	CABZ01113883.1	0.283917325	-0.023921963	-0.385162746	-1.507337386
ENSDART00000168918	pcdh1gc6	0.501599317	0.826475212	1.380988505	1.066751642
ENSDART00000168920	nbeab	-0.251742312	-0.363594185	-0.174304201	-0.037829821
ENSDART00000168922	Metazoa_SRP	1.221075219	2.596056056	2.04902232	1.755997125
ENSDART00000168935	mfsd8	0.060271678	-0.029632781	-0.242261851	-0.178722202
ENSDART00000168957	cdh18a	-0.488858345	-0.440968125	-0.25726489	-0.177287767
ENSDART00000168969	htr7c	-0.617759768	-0.791263551	-0.091828465	0.038777953
ENSDART00000168996	plrdgb	-0.579612528	-0.647606525	-0.269512976	0.036793619
ENSDART00000169006	ak2	0.70404039	0.489848323	0.563968118	0.137147044
ENSDART00000169023	scn1lab	0.230826198	0.448869227	0.411715364	0.200031153
ENSDART00000169026	jak2a	0.024039251	0.103369971	0.328189294	0.009690028
ENSDART00000169040	si:ch211-235e9.8	-0.436326332	-0.357257361	-0.23322888	-0.084450729
ENSDART00000169052	elovl1b	0.945814486	1.07228669	0.819418014	0.365575121
ENSDART00000169060	si:ch211-212k18.5	-0.245044911	-0.301564521	-0.568174767	-0.780659219
ENSDART00000169061	CU138533.1	0.641184794	1.068185172	1.17226183	0.773255864
ENSDART00000169081	mtmr7b	-0.302136082	-0.184242994	0.142322853	0.09943817
ENSDART00000169091	psd2	-0.329495857	-0.192167097	0.082881422	0.17989231
ENSDART00000169105	pcdh1g29	-0.285141513	0.143203693	0.405619816	0.452362051
ENSDART00000169108	kcnj12a	-0.480725794	-0.616905664	-0.509410819	-0.355199716
ENSDART00000169116	cpne4a	-0.476761925	-0.590434794	-0.280550227	0.444813194
ENSDART00000169119	ndrg4	-0.4692852	-0.086247828	0.285804524	0.242386508
ENSDART00000169127	adgrb3	-0.338634159	-0.316792657	-0.154400064	0.089681207
ENSDART00000169129	ndrg4	-0.326143464	-0.61306806	-0.078381969	-0.216584442
ENSDART00000169130	ftr64	0.270335706	0.025822717	1.271602536	0.119170332
ENSDART00000169136	CABZ01007222.1	-0.363042867	-0.477045243	-0.379599521	-0.116458215
ENSDART00000169165	CABZ01021450.1	-0.130594664	-0.107324732	-0.381541063	-0.095206853
ENSDART00000169187	ptpro	0.079888282	0.61716785	1.250280006	1.189522062
ENSDART00000169201	pcdh2g16	0.673146228	0.480832534	0.40403692	0.542228011
ENSDART00000169202	si:ch211-153b23.5	4.343063064	5.139014825	3.462179286	1.548865865
ENSDART00000169209	ptrh1	0.68284848	0.772674127	0.446966304	0.22472723
ENSDART00000169210	CABZ01072550.1	-0.141044522	-0.137807967	-0.329360205	-0.030428593
ENSDART00000169228	vat1l	0.089392965	-0.09841303	-0.250056266	-0.442720544
ENSDART00000169243	znf384l	-0.366831524	-0.600767675	-0.247311028	0.205515141

ENSDART00000169248	drd4-rs	-0.178692855	-0.390296441	-0.526046415	-0.318223636
ENSDART00000169275	sdad1	0.31633119	0.22409068	0.190752906	0.014420983
ENSDART00000169309	si:dkey-203a12.2	1.554830383	2.508546052	1.961959042	0.546471184
ENSDART00000169322	zbtb21	-0.157953464	-0.169354872	-0.299279576	-0.291807058
ENSDART00000169341	nrp1a	0.186448987	0.415165621	0.563193133	0.501195382
ENSDART00000169343	kdf1b	-0.449286765	-0.274976378	-0.544184682	-0.396728296
ENSDART00000169363	CR678435.1	-0.089321474	-0.319658973	-0.354063558	-0.259523127
ENSDART00000169365	Metazoa_SRP	2.055456458	2.688758632	2.943606625	2.098435714
ENSDART00000169367	CABZ01061884.1	-0.298329532	-0.377577791	-0.54544765	-0.245416552
ENSDART00000169375	znf1151	2.128272751	1.140055383	3.010647967	3.121008171
ENSDART00000169376	PLEKHG5 (1 of many)	-0.003560342	-0.365335228	-0.516938003	-0.057649582
ENSDART00000169380	slc25a43	-0.226077688	0.088930874	-0.355915914	-0.508574161
ENSDART00000169407	fynb	-0.281673654	-0.267434526	-0.200981958	-0.171531273
ENSDART00000169447	sorbs1	0.299961188	0.420748275	0.143707838	0.107473415
ENSDART00000169452	CR848841.2	1.41519844	0.623487161	0.225042545	-0.111771198
ENSDART00000169462	fam189a1	-0.165720729	-0.526215514	-0.059981823	-0.273837885
ENSDART00000169477	tmem176l.1	0.297546209	0.073954032	-0.516480584	-0.864341304
ENSDART00000169484	amph	-0.176945265	-0.219628366	-0.476094931	-0.135317446
ENSDART00000169511	ppp2r2bb	-0.347621729	-0.40572046	-0.501873649	-0.158073428
ENSDART00000169514	cacna1db	-0.154801093	-0.201285475	-0.375153121	-0.07189092
ENSDART00000169531	traf2b	-0.006517414	-0.077905282	-0.353292738	-0.278704886
ENSDART00000169548	rgs7b	-0.179117493	-0.337814246	-0.235695562	-0.091038288
ENSDART00000169552	cdh13	-0.778914955	-0.463881372	-0.06287293	0.145633136
ENSDART00000169600	abhd4	0.160082589	0.423680439	-0.10396044	-0.08495852
ENSDART00000169609	tefb	-0.156114581	-0.555141888	-0.301378906	-0.24132076
ENSDART00000169614	nadsyn1	0.473419312	0.288129879	-0.022685337	-0.020046915
ENSDART00000169626	kcnc1a	-0.804732723	-1.394467436	-0.226498418	0.073837621
ENSDART00000169633	col4a3bpb	0.489929413	0.127889926	-0.192097119	-0.259910666
ENSDART00000169644	CU659306.1	0.286956046	0.461515845	0.629089247	0.128650576
ENSDART00000169648		0.47490981	0.626028668	1.036581019	0.748938091
ENSDART00000169663	CT573860.1	0.353646224	0.562802664	0.454063437	0.329757181
ENSDART00000169667	ptpro	-0.163271096	0.493460364	0.820001756	0.696103761
ENSDART00000169682	hdac4	-0.093013902	-0.31519447	0.302479135	0.371645007
ENSDART00000169687	dlg1	-0.200796553	-0.39742906	-0.281934456	-0.007509175
ENSDART00000169698	zgc:158343	-0.309656325	-0.821697026	-0.621795526	-0.564704541
ENSDART00000169710	utp23	0.386723475	0.25777974	0.117505162	0.115827548
ENSDART00000169717	ca5a	0.91389398	0.899982871	0.348639788	0.672790664
ENSDART00000169723	HERC5	-0.128535223	-0.136551326	0.521223162	-0.158454388
ENSDART00000169733	klhl23	-0.117061422	-0.244154323	-0.447638501	-0.20478162
ENSDART00000169746	ampd2a	-0.446564291	-0.523779729	-0.426555345	-0.117905151
ENSDART00000169764	syt1a	-0.535633412	-0.482595993	-0.403145231	-0.029612735
ENSDART00000169768	slc8a3	-0.133596734	-0.60366341	-0.159093114	0.187240856
ENSDART00000169789	grap2b	0.941263606	0.635408975	0.416956975	0.146969065
ENSDART00000169810	stub1	-0.352758303	-0.24873956	-0.212157701	-0.100812458
ENSDART00000169823	pnpla3	0.41574021	0.387767111	0.775977498	0.378524498
ENSDART00000169828	rlbp1a	-0.278789297	-0.290042852	-0.461967142	-0.22894081
ENSDART00000169834	CABZ01052431.1	0.752252615	0.635370532	0.523133762	0.005708707
ENSDART00000169845	pargl	0.069840572	-0.083042796	0.787500652	-0.250417109
ENSDART00000169852	RPL37A	0.3117075	0.379509479	0.225285723	0.045531307
ENSDART00000169853	slmapa	-0.949896363	-1.088521821	-0.936929707	-0.657167359
ENSDART00000169887	wu:fu71h07	0.98545678	0.406691046	0.849706316	0.386892251

ENSDART00000169892	srp54	2.340238808	3.996874038	2.498816008	1.178878233
ENSDART00000169895	zbtb18	0.009752394	-0.192437538	-0.328439949	-0.081774144
ENSDART00000169915	nop58	0.425406041	0.33755518	0.254231429	0.051624158
ENSDART00000169916	dmxl2	-0.252318602	-0.45068029	0.053959432	0.232942265
ENSDART00000169923	ndel1b	-0.007054921	-0.080749821	-0.505244725	-0.275115017
ENSDART00000169932	ccdc142	-0.157730988	-0.223081294	-0.3402281	-0.055715693
ENSDART00000169936	sox8a	-0.26561801	-0.150998063	-0.108428913	-0.111175355
ENSDART00000169940	tmem176l.1	0.227275106	0.228370918	-0.147494824	-0.481831855
ENSDART00000169942	stx3a	-0.199600759	-0.344637551	-0.343244623	-0.200314073
ENSDART00000169943	CABZ01084447.1	-0.382296742	-0.397697491	-0.572266726	-0.473542202
ENSDART00000169945	si:ch73-236c18.7	1.628999904	1.036261941	0.5890347	0.798137712
ENSDART00000169946		-0.124122046	-0.322722589	-0.278305257	0.008390957
ENSDART00000169948	GRM7	-0.415342332	-0.376852993	-0.226859206	-0.023200649
ENSDART00000169951	Metazoa_SRP	1.195279937	1.735123002	1.057793715	0.943058282
ENSDART00000169953	dctn1b	-0.327369492	-0.021477792	0.065821587	0.193833161
ENSDART00000169963	ryr1b	-0.099451213	-0.21486392	0.409324638	0.697954707
ENSDART00000169972	ndst1b	-0.191299747	-0.313711004	-0.152975304	-0.236354037
ENSDART00000169976	CABZ01034529.1	0.902549952	0.806876908	0.306228824	0.021333022
ENSDART00000169989	BX649355.1	0.986946969	0.719472766	0.338456492	0.762216016
ENSDART00000169993	stx12	0.289245416	0.334302649	0.162033659	0.014864416
ENSDART00000169999		0.330954109	-0.605210778	-0.313930686	-0.099685871
ENSDART00000170012	mapre3b	-0.205130823	-0.301066074	-0.42167523	-0.136278286
ENSDART00000170021		2.405989626	2.77310135	2.003596445	2.210644738
ENSDART00000170060	mdh1b	3.761355509	3.246314783	3.336555843	1.787365183
ENSDART00000170089	epha4b	-0.586426265	-0.698463168	-0.228203192	-0.016930318
ENSDART00000170095	CR388166.2	0.588171845	0.299671021	0.452688856	0.506464888
ENSDART00000170107	ppp2r2ba	0.343342582	0.340534473	0.3871703	0.276562577
ENSDART00000170116	uap1	0.399345456	0.560025587	0.455223073	0.418274301
ENSDART00000170133	eci1	2.097463401	3.784028698	2.248505252	3.097351651
ENSDART00000170165	cep97	-4.277597338	-0.154895502	-0.337498335	-0.461056374
ENSDART00000170184	fgd1	0.020796397	0.102196495	0.582360556	0.487921156
ENSDART00000170191	FQ311879.1	-0.155204603	-0.24875676	-0.288299334	-0.086604212
ENSDART00000170204	si:zfos-364h11.2	0.504424795	0.456776646	0.313589859	-0.247114093
ENSDART00000170208	limch1b	-0.042803542	-0.148322889	-0.621537079	-0.273084471
ENSDART00000170222	mibp	1.202449519	1.249757535	1.712700336	0.61416841
ENSDART00000170228	sept9b	-0.22300654	-0.343163525	-0.367834845	-0.241839556
ENSDART00000170284	creb3l3l	0.444489468	0.957040221	0.49606022	0.414651857
ENSDART00000170285	def8	-0.050118345	-0.13948188	-0.345534099	-0.220243255
ENSDART00000170304	CABZ01039096.1	1.863106121	1.329668381	1.247074235	0.639142974
ENSDART00000170324	tom1	-0.260627206	-0.142873643	-0.174666961	-0.048298907
ENSDART00000170325	map7d1b	-0.159995641	0.033353383	0.380064991	0.315967697
ENSDART00000170326	gadd45ba	1.741159479	1.754335562	1.06756424	0.870946071
ENSDART00000170341	apba1a	-0.229826313	-0.069954525	0.548704484	0.456234995
ENSDART00000170351	nbeal1	-0.047098407	-0.274555108	-0.182658383	-0.033615077
ENSDART00000170362	CABZ01088490.1	-0.602203241	-0.75748097	-0.591873288	-0.034015136
ENSDART00000170374	si:dkey-38l22.2	1.50589782	1.851640662	1.322719067	0.692201779
ENSDART00000170376	mpp7a	-0.022565767	-0.302686812	-0.467837523	-0.093897739
ENSDART00000170381	CR855389.1	-0.538290002	-0.347216121	-0.566215377	-0.407287476
ENSDART00000170383	CABZ01089777.1	-0.22190804	-0.652997161	-0.608886761	-0.372475629
ENSDART00000170385	tgm1l1	0.820949739	0.875506718	1.293638142	0.305667169
ENSDART00000170396	apbb2b	-0.997027217	-0.745617105	0.597402109	1.180709512

ENSDART00000170405	CARTPT (1 of many)	1.790273718	2.206490975	2.680185278	2.041251968
ENSDART00000170422	si:dkey-19b23.8	0.497209105	1.26444289	1.061660184	0.502612026
ENSDART00000170423	jakmip3	-0.243674673	-0.387799515	-0.202768181	0.090619484
ENSDART00000170433	tert	0.747709966	0.573674682	0.803557653	0.065172489
ENSDART00000170441	CLDN23	-0.099162778	-0.365194678	-0.492897369	-0.25219875
ENSDART00000170453	slc20a1b	-0.460714141	-0.247481668	-0.017046699	-0.023964599
ENSDART00000170456	shroom3	0.242207867	0.41278364	0.171514229	-0.137113926
ENSDART00000170460	nrxn3b	-0.2276052	-0.615850363	-0.134586161	-0.003465474
ENSDART00000170466	gch2	0.005943316	1.153050374	0.394171756	-0.303921757
ENSDART00000170470	ghrhra	-0.141377937	-0.217703208	-0.411536652	-0.240637833
ENSDART00000170510	RIMBP2 (1 of many)	-0.653406892	-0.568163429	-0.174788334	-0.007324529
ENSDART00000170512	ctnnd1	0.874551431	1.060254696	0.863956713	0.322404291
ENSDART00000170518	anxa1c	-0.336866673	-0.270774527	-0.035149981	-0.070098806
ENSDART00000170526	CABZ01021220.1	-0.34731151	-0.318997758	-0.303107193	-0.013605358
ENSDART00000170546	wdr17	-0.20142732	-0.213426713	-0.433783757	-0.087288031
ENSDART00000170562	ece2b	-0.279383738	-0.308974349	-0.125983301	-0.132241867
ENSDART00000170569	syt12	-0.747896951	-0.906145295	-0.362441574	-0.047384497
ENSDART00000170571	dmtn	0.084482265	0.234365609	0.453911363	0.30033427
ENSDART00000170572	lepa	4.559547685	4.778234325	4.366752593	2.765663683
ENSDART00000170575	nfat5b	-0.001493923	-0.392122738	-0.566909919	-0.288747412
ENSDART00000170583	march8	-0.037993536	-0.377054474	-0.284528801	-0.103767971
ENSDART00000170589	mibp	1.067201928	1.476075399	1.501871782	0.758874349
ENSDART00000170601	si:ch1073-228h2.2	1.512427811	1.187632525	1.253650535	1.028624188
ENSDART00000170620	ctxn1	-1.120908397	-0.319127099	0.144625495	0.239695278
ENSDART00000170630	BX511034.7	0.940574459	1.49905624	1.717428689	0.604931976
ENSDART00000170631	ebf1a	-0.245696473	-0.326200552	-0.132641619	0.115308885
ENSDART00000170632	gngt2b	-0.25352879	-0.46015013	-0.596390898	-0.26583618
ENSDART00000170671	megf8	0.369502187	0.913401505	1.13324158	0.463058503
ENSDART00000170675	col11a1a	-3.564960509	-0.565137585	-0.451612456	-1.057274574
ENSDART00000170680	ptprdb	-0.822263801	-1.170454394	0.28663796	0.697055522
ENSDART00000170684	btf3	0.237794169	0.784907824	0.656975875	0.357635511
ENSDART00000170695	lrit1b	-0.349980517	-0.215317539	-0.688918418	-0.304938883
ENSDART00000170700	nacc1b	-0.295710389	-0.416714615	0.013950982	0.110349783
ENSDART00000170709	st8sia1	-0.358435626	-0.336878724	-0.21402261	-0.093884674
ENSDART00000170752	tox2	-0.491067711	-0.314026775	-0.014696402	0.264527682
ENSDART00000170755	slc30a8	0.607491231	0.345740737	-0.026161917	-0.397029232
ENSDART00000170757	kntc1	0.719300782	0.704073311	0.501967558	0.060438055
ENSDART00000170758	tcirg1b	0.779415513	0.215400071	0.036968665	-0.339490039
ENSDART00000170762	slc44a5b	-0.083921908	-0.392138039	-0.663427101	-0.045247688
ENSDART00000170768	lect1	3.805179034	4.274135568	2.696697623	2.532825072
ENSDART00000170790	thrb	-0.205328436	-0.495702017	-0.443190893	-0.211013858
ENSDART00000170810	si:dkey-10p5.7	1.115159817	1.313044185	0.773496009	0.334257673
ENSDART00000170827	ccpg1	0.08168444	-0.121915293	-0.302033768	-0.268515452
ENSDART00000170834	znf1179	0.46327857	0.626036671	0.707498793	0.811192005
ENSDART00000170839	pcdh1g26	-0.648364026	-0.34360777	0.683964001	0.607743239
ENSDART00000170854	gphnb	-0.471165414	-0.533169869	-0.144401896	0.220388206
ENSDART00000170856	smu1b	0.353209488	-0.339997639	-0.798035862	-0.487523616
ENSDART00000170865	nme2b.1	0.635993303	0.679448572	0.689008761	0.355584206
ENSDART00000170872	tpm2	-0.143399476	2.621381225	3.304047609	1.793877447
ENSDART00000170874	phldb1b	0.7459988	0.426405219	0.977480972	0.780660286
ENSDART00000170875	BX663610.1	-0.528067918	-0.63401091	-0.217086155	-0.025338796

ENSDART00000170886	dennd1b	-0.037953425	-0.315629797	-0.364933587	-0.202214668
ENSDART00000170888	pkma	-0.382873513	-0.359191464	-0.202639045	0.04775771
ENSDART00000170932	rims2a	-0.482409185	-0.599060629	-0.273352321	-0.056203652
ENSDART00000170951	PXDN	0.459355707	0.64772414	0.996368549	0.498632459
ENSDART00000170952	pvrl2l	-0.402141005	-0.731362921	-0.043297784	0.281757642
ENSDART00000170955	fn3krp	-0.183544623	-0.156282547	-0.395153052	-0.231090781
ENSDART00000170983	lmnb2	0.185291183	0.299339057	0.275121803	0.118770566
ENSDART00000170993	afmid	-0.391958822	-0.311185654	-0.48659874	-0.071120804
ENSDART00000170998	tnrc6c2	-0.257719977	-0.319066529	-0.317919253	-0.242876098
ENSDART00000171003	CU929447.2	0.254824338	0.430023304	0.328306359	0.219409854
ENSDART00000171006	hpcal4	-0.435402713	-0.198074705	-0.466665783	-0.338540363
ENSDART00000171013	tenm4	-0.557784731	-0.616815207	-0.086642376	-0.101260046
ENSDART00000171014	ptprfa	-0.231763582	-0.479834972	-0.30404021	-0.129420632
ENSDART00000171021	rab3ip	0.020022814	0.162794734	0.729696081	0.694579303
ENSDART00000171041	tceb3	-0.063048764	-0.305499385	-0.056078786	-0.065276347
ENSDART00000171058	pald1a	0.52269022	0.19158808	0.108215435	-0.186659053
ENSDART00000171072	SEC14L1	-0.099969512	-0.17692833	-0.270780357	-0.178232632
ENSDART00000171073	tox2	-0.713271279	-0.691854818	-0.08505138	-0.233730891
ENSDART00000171090	sowahd	0.56962557	0.415649109	0.184768671	-0.065367372
ENSDART00000171091	zeb2b	-0.344020449	-0.404143226	-0.222307379	0.140992606
ENSDART00000171113	mibp	1.815496184	1.851942075	2.237510566	0.882565569
ENSDART00000171114	si:dkey-18j18.3	-5.054012109	-5.054146995	-1.846245188	0.223162792
ENSDART00000171137	pdia3	0.328843285	0.182764859	0.141869644	-0.049588298
ENSDART00000171169	numbl	-0.199436908	-0.274498759	0.034564123	0.069477302
ENSDART00000171178	nlrc3l	0.061705987	0.093907561	0.374016309	-0.032278843
ENSDART00000171179	si:ch211-241b2.5	1.438237825	1.596241839	1.013176313	0.055616895
ENSDART00000171182	FO704914.1	-0.372200999	-0.770608409	-0.45129487	-0.014659406
ENSDART00000171188	si:ch211-165d12.4	3.380342846	2.16159833	1.938428841	0.89654506
ENSDART00000171202	clrn1	-0.342498627	-0.174544267	-0.411984605	-0.164186516
ENSDART00000171215	slc6a3	-0.667608441	-0.754783262	-0.446986408	-0.343120625
ENSDART00000171235	calua	0.528206357	0.591046444	0.528298198	0.210887316
ENSDART00000171237	kcnj2b	-0.406552715	-0.524353506	-0.661059283	-0.393570981
ENSDART00000171244	ssh2b	-0.244446722	-0.139215381	0.546963377	0.2356201
ENSDART00000171252	man2a1	0.336633303	0.148045387	-0.143242682	-0.284272741
ENSDART00000171270	ckap2l	0.621565842	0.655006082	0.304610509	0.072068559
ENSDART00000171288	magi2b	-0.2674353	-0.525609031	-0.078206648	-0.00532511
ENSDART00000171289	si:ch73-158p21.2	0.432663487	0.278579945	0.160816438	0.272943666
ENSDART00000171306	stxbp1a	-0.614795684	-0.539858263	-0.126841305	0.057169963
ENSDART00000171316	si:dkey-262j3.7	-0.307347885	-0.015370213	0.260259549	0.204989519
ENSDART00000171320	dcp2	-0.072540161	-0.060086595	-0.320231159	-0.153613562
ENSDART00000171335	map4k4	0.106316233	0.336083411	0.398966135	0.337674059
ENSDART00000171336	dyrk4	0.12887622	-0.629074584	-0.893898922	0.00387951
ENSDART00000171343	FP102192.1	1.130120264	0.697435719	1.760333984	0.139610385
ENSDART00000171345		2.015201895	1.694676698	1.017867776	0.711389392
ENSDART00000171354	nt5c2l1	0.533832785	0.693578932	1.620342483	0.507169588
ENSDART00000171359	asic1b	-0.583045866	-0.669491195	-0.186449925	0.212036898
ENSDART00000171380	top1mt	0.983277478	1.316957074	1.074308725	0.32139594
ENSDART00000171382	epb41l3a	-0.440465797	0.150336785	0.618974575	0.751984906
ENSDART00000171393	efr3a	0.028329226	-0.20834437	-0.54511994	-0.110508442
ENSDART00000171396	pts	0.27960755	0.015420592	-0.484687881	-0.407740441
ENSDART00000171417	si:ch73-299h12.3	-0.719381589	-0.760232748	-0.826233659	-0.863901262

ENSDART00000171426	pdzph1	-0.416069913	-0.411889039	-0.457121209	-0.221062124
ENSDART00000171430	BX908401.1	0.558597997	0.282396796	0.308082083	0.228227036
ENSDART00000171433	tnni1d	0.40952122	3.032354782	4.009528497	2.823338021
ENSDART00000171456	rasgrf2b	-0.231105558	-0.246992498	-0.342919918	-0.045608772
ENSDART00000171466		-1.418832616	-2.063774189	-0.38972739	-0.735471862
ENSDART00000171490	PCDH8	-0.518696681	-0.628285802	-0.168709969	0.408565003
ENSDART00000171494	ssbp2	-0.298958691	-0.315587827	-0.028952479	0.152638316
ENSDART00000171496	CDK18	-0.371495713	-0.374286185	-0.433391037	-0.282164654
ENSDART00000171497	mfap4	2.949855284	1.228743784	1.366529806	0.589539203
ENSDART00000171506		-0.792662919	-0.4993043	0.08528346	0.180572156
ENSDART00000171523	CABZ01089030.1	-0.303576632	-0.51118876	-0.528168137	-0.25789403
ENSDART00000171524	CABZ01062422.1	0.017851166	0.325269151	0.385529332	0.004388505
ENSDART00000171571	FAM184A (1 of many)	-0.84950254	-1.068290057	0.155587747	0.361779982
ENSDART00000171589	hrasa	0.543862541	0.913653251	0.982614467	0.767663706
ENSDART00000171594	mef2aa	-0.649844171	-0.685014218	-0.301258241	-0.003741614
ENSDART00000171624	Metazoa_SRP	1.040618727	2.218519951	2.250692356	1.867352594
ENSDART00000171634	galnt13	0.214607397	-0.183374464	-0.358548812	-0.056557988
ENSDART00000171639	prkacbb	-0.216859972	-0.333993863	-0.380485937	-0.232933535
ENSDART00000171674	abhd8a	-0.482604068	-0.462135418	0.077091696	0.23737745
ENSDART00000171678	ubb	0.267730806	0.139565922	0.302768617	0.039108167
ENSDART00000171683	GABRG1	-0.455034367	-0.392373746	-0.437520168	-0.270886274
ENSDART00000171686	si:ch211-225h24.2	0.00451198	-0.128666512	-0.443683785	-0.077894977
ENSDART00000171687	XPO5	0.143122457	0.173342903	0.297288837	0.170261463
ENSDART00000171691	psmc3	0.15503219	0.267774928	0.185121834	-0.000325126
ENSDART00000171696	sergef	-0.83692277	-0.920324583	-0.711315475	-0.040508735
ENSDART00000171701	osmr	1.90654589	2.08952232	1.976929325	1.246005697
ENSDART00000171704	soul4	-0.335441282	-0.335430337	-0.104813252	-0.062286587
ENSDART00000171711	gpsm1a	-0.376386863	-0.294884117	-0.277096957	-0.079488929
ENSDART00000171728	FQ323156.1	-0.3925045	-0.548422409	-0.578285827	-0.21374206
ENSDART00000171743	sypa	-0.458751426	-0.455626528	-0.334063629	-0.142465032
ENSDART00000171749	TMEM150A (1 of many)	-0.745537318	-0.560172793	-0.666392254	-0.39318407
ENSDART00000171762	arhgef11	-0.293734421	-0.490220654	-0.20280274	0.049910063
ENSDART00000171777	syt7b	-0.587310767	-0.899783938	-0.612333236	-0.170082745
ENSDART00000171789		1.544321358	1.350890859	1.388203792	0.786947546
ENSDART00000171806	ppt1	0.388846083	0.447401215	0.166979487	-0.03135359
ENSDART00000171811	grk1b	2.834746901	2.005283407	2.325249515	3.603901667
ENSDART00000171815	abcc8b	0.00284442	-0.044162256	-0.758748776	-0.514944979
ENSDART00000171823	cdc14ab	-0.17216976	-0.3147465	-0.385013143	-0.20236803
ENSDART00000171824	si:ch211-227e10.2	-0.663768919	-0.251157559	-0.496668883	-0.404241953
ENSDART00000171830	uchl1	0.751081517	1.035376191	0.855000132	0.800147781
ENSDART00000171854	si:ch1073-303d10.1	-0.079667744	-0.472299802	-0.615705243	-0.228401157
ENSDART00000171867	fnbp1l	0.402488238	0.925028089	0.937640279	0.578874881
ENSDART00000171868	sgut1	-0.438334002	-0.252211506	-0.468859348	-0.232780566
ENSDART00000171871	cbfb	0.41950513	0.366762581	0.672082991	0.461647583
ENSDART00000171882	CABZ01088567.1	0.407931	0.822027708	0.621405649	0.24977578
ENSDART00000171891	iqsec2a	-0.225046822	-0.287658971	-0.015287221	0.082198509
ENSDART00000171900	oit3	-0.401327729	-0.441180087	-0.161045426	-0.008695935
ENSDART00000171913	AL773558.1	-0.541466018	-0.593947031	-0.853404048	-0.564731615
ENSDART00000171915	FO834898.1	-0.059070074	-0.319253855	-0.196715718	-0.032975583
ENSDART00000171916	myom2a	1.227196118	3.513600791	3.806244226	2.767902474
ENSDART00000171935	brpf3a	-0.333420883	-0.26685281	-0.204490748	-0.091216638

ENSDART00000171941	si:dkeyp-53d3.5	2.939427931	3.522632269	3.599150168	3.695003344
ENSDART00000171948	zgc:172282	-0.447105187	-0.64749442	-0.36124568	-0.081452365
ENSDART00000171951	slit2	-0.546372344	-0.56571293	-0.270916548	-0.035288929
ENSDART00000171965	galnt18a	-0.106299358	-0.103906292	-0.35180024	-0.003683356
ENSDART00000171975	si:ch211-230g14.6	-0.465912508	-0.525802535	-0.259029045	-0.276627158
ENSDART00000171977	ddx52	0.350026309	0.214664704	0.228079892	-0.041434316
ENSDART00000171996	myl12.1	0.233861142	0.344330245	0.260508491	0.06338781
ENSDART00000172014	actr3	3.712429453	3.456018148	3.177836674	3.174953878
ENSDART00000172016	prox1a	-0.34053446	-0.257376891	-0.299242567	-0.073418723
ENSDART00000172019	CU694219.1	-0.358471879	-0.088786066	-0.423165049	-1.570028426
ENSDART00000172022	dpysl4	0.270457415	0.912051797	1.185681882	0.874375314
ENSDART00000172045	zgc:73340	-0.432558368	-0.571519732	-0.606453829	-0.389736033
ENSDART00000172069	snrpg	0.452166641	0.481061823	0.338564477	0.058821342
ENSDART00000172076	hook1	-0.040243255	-0.111929437	-0.520350092	-0.090216969
ENSDART00000172080	ttbk2a	1.110423963	2.886130551	3.128605088	3.572451241
ENSDART00000172084	si:ch73-236j9.2	0.095305984	0.48958329	0.505184662	0.37060971
ENSDART00000172095		-1.418832616	-2.063774189	-0.38972739	-0.735471862
ENSDART00000172102	elof1	0.045486466	-0.036149641	-0.262027739	-0.168895436
ENSDART00000172114	calm3a	-0.154349916	-0.209222212	-0.409530683	-0.13969668
ENSDART00000172128	sez6l	-0.457515145	-0.649880208	-0.297003963	-0.214224661
ENSDART00000172135	sh3pxd2b	0.68385955	0.832942161	0.517904326	0.090715158
ENSDART00000172149	sh3rf2	-0.359308533	-0.521296357	-0.501584275	-0.200324498
ENSDART00000172166	NDUFC1	-0.092745693	-0.325133407	-0.417118078	-0.256060161
ENSDART00000172190	ajap1	-0.155573908	-0.463634934	-0.533953643	-0.131876486
ENSDART00000172201	trpv1	0.412354182	0.082280105	0.061779394	-0.254715035
ENSDART00000172207	si:ch211-9d9.1	1.222547885	0.297262625	0.271454205	-2.80261928
ENSDART00000172215	si:ch211-39i2.2	1.017534105	1.848603057	1.465869245	0.571196451
ENSDART00000172218	nsmfb	-0.786748472	-1.480880504	-0.428989171	-0.523180434
ENSDART00000172232	sv2a	-0.325501969	-0.478402861	-0.246153159	0.031695064
ENSDART00000172233	si:ch73-55i23.1	0.636981625	0.696972269	1.179674288	0.207432569
ENSDART00000172241	nsdhl	1.591273733	3.017393364	3.100136405	2.442907405
ENSDART00000172251	creb3l1	-0.33540662	0.193573394	-4.078076569	-0.842213331
ENSDART00000172267	KCNV1	-0.730291266	-0.683938113	-0.423486953	-0.317970814
ENSDART00000172274	kifap3a	2.52903808	3.896963711	3.83143073	1.193936031
ENSDART00000172279	cavin4a	-0.061511418	1.266222675	1.14872392	0.602916509
ENSDART00000172285	arf3b	-0.358958449	-0.189620663	0.143810604	0.126356011
ENSDART00000172294	ctps1b	-0.150552652	-0.426760874	-0.523567714	-0.150809326
ENSDART00000172300	slc38a4	-0.096535094	-0.136897206	-0.74017911	-0.38118016
ENSDART00000172301	tk1	1.414413476	0.945772512	1.08664013	0.593080412
ENSDART00000172305	lsm10	0.431922906	0.307479396	0.650924826	0.415051794
ENSDART00000172307	UBE2M	-0.026970956	0.061666236	0.299772523	0.276955817
ENSDART00000172309	twsg1a	0.181135796	0.092573769	-0.368073769	-0.475343856
ENSDART00000172310	zbtb4	-0.365422353	-0.277101341	-0.237436935	-0.247791483
ENSDART00000172327	taok1b	-0.323110832	-0.519144421	-0.19684291	0.126510211
ENSDART00000172329	CABZ01084963.1	-0.076477014	-0.479017439	-0.153366972	-0.215592031
ENSDART00000172335	cpne3	0.169360614	0.301780948	0.100312932	-0.018165212
ENSDART00000172336	cabp2a	-0.40143904	-0.491969769	-0.23184904	-0.50001549
ENSDART00000172337	rhot1a	-3.878236672	-5.686158747	-0.665991717	-0.822715826
ENSDART00000172338	cav1	0.970717786	1.027469024	1.102211393	0.680419868
ENSDART00000172357	tmem132a	-0.246648072	-0.102389273	-0.224742481	-0.519178406
ENSDART00000172367	sgip1b	-0.158702965	-0.245738955	-0.177100693	0.051555171

ENSDART00000172372	Metazoa_SRP	1.431390172	1.797550169	1.401344471	0.830250287
ENSDART00000172373	ompa	-0.333379791	-0.33389964	-0.383107757	-0.229559723
ENSDART00000172408	arhgap11a	0.695056063	0.093403628	0.066933123	0.263951638
ENSDART00000172409	il6st	2.752703405	3.785571298	4.464867139	2.64892811
ENSDART00000172428	FO704797.2	0.17851052	-0.259187206	-0.513103189	-0.127941479
ENSDART00000172430	si:dkey-203a12.3	2.592857339	1.584928476	2.433004816	1.57133529
ENSDART00000172433	BX323031.2	0.315248431	0.209012025	-0.000555571	0.219130901
ENSDART00000172437	dgkab	-0.119408916	-0.169975589	-0.468807215	-0.381241335
ENSDART00000172441	lima1a	0.350368785	0.588496789	0.46589541	0.178939257
ENSDART00000172444	ATG2A	0.113672015	0.132095473	0.291990145	0.186645397
ENSDART00000172460	FO904965.1	-0.329411304	-0.282535563	-0.05279435	0.008365177
ENSDART00000172465	gnb1b	-0.429380032	-0.139749463	0.050389396	0.071415749
ENSDART00000172496	add3a	-0.173242509	-0.314301562	-0.183893521	-0.110146899
ENSDART00000172510	elavl4	1.054519465	1.174247457	1.078840874	0.691066079
ENSDART00000172515	bzw1b	0.541293913	0.477440982	0.388001444	-0.287525957
ENSDART00000172547	insb	-0.25337952	-0.120760297	0.28326011	0.898589828
ENSDART00000172552	dyrk2	-1.00865602	-4.097564958	-0.48472737	-0.263450257
ENSDART00000172555	rps15a	0.386196694	0.41469411	0.231657808	-0.026260131
ENSDART00000172565	Metazoa_SRP	1.391513514	1.064695711	1.082381989	0.312800391
ENSDART00000172566	ptpro	-0.439852276	0.14868548	0.772757137	0.551836031
ENSDART00000172624	chn1	-0.429491719	-0.379515323	-0.144891996	-0.16828013
ENSDART00000172634	ryr3	-1.057085419	-1.586723574	-0.163110923	0.331797887
ENSDART00000172638	gria3a	-0.308989617	-0.39028425	-0.157465183	0.147319123
ENSDART00000172645	unc5da	-0.375739047	-0.576943357	-0.183677467	0.03667029
ENSDART00000172661	nrgnb	-0.441646104	-0.659637875	-0.590118296	-0.403632798
ENSDART00000172662	fam96a	-0.029501914	-0.191990056	-0.34693921	-0.272376792
ENSDART00000172664	kirrel3l	-0.579597094	-0.334224423	-0.00269739	0.256102934
ENSDART00000172672	angptl2b	-0.321678324	-0.001662718	-0.330394771	-0.841768465
ENSDART00000172689	drd4a	-0.269065132	-0.433761644	-0.356034882	-0.090259185
ENSDART00000172703	trnau1apa	3.525550379	4.03135258	2.121100592	1.557105834
ENSDART00000172766	psd3l	-0.547513249	-0.802641524	-0.228472689	0.098980101
ENSDART00000172775	tegt	-0.040616632	0.038263838	-0.257883683	-0.248241903
ENSDART00000172811	ubap1lb	0.254294803	-0.127702895	-0.996786994	-0.222342304
ENSDART00000172821	PHLPP2	-0.058117589	-0.547864216	-0.458033727	-0.042436898
ENSDART00000172862	kcnj14	-0.439592228	-0.323667837	-0.482461949	-0.197270237
ENSDART00000172874	kcnj10a	-0.114134993	-0.28596151	-0.331658021	-0.188992319
ENSDART00000172892	CR769769.2	0.615702823	0.344273677	0.373739938	0.336791427
ENSDART00000172893	si:ch211-207g17.2	1.081136407	0.564546524	-0.069203481	-0.244746802
ENSDART00000172899	hp1bp3	-0.268836711	-0.197039259	-0.254153947	-0.074858409
ENSDART00000172910	map1aa	-0.286879932	0.224710371	0.48760533	0.689542868
ENSDART00000172913	si:ch73-368j24.11	0.642626776	0.575984051	0.848181147	0.478362255
ENSDART00000172939	ptp4a1	0.526587293	0.493567323	0.439467476	0.194682023
ENSDART00000172944	FO681390.1	-0.061230657	-0.175829366	-0.634551101	-0.148743958
ENSDART00000172948	TSC22D3	0.014403639	-0.423782958	-0.297835045	-0.190699463
ENSDART00000172953	rpl29	0.354363113	0.457581567	0.206775836	0.026974968
ENSDART00000172975	slc6a1b	0.350767184	0.274634418	0.412920252	0.546226395
ENSDART00000172988	si:zfos-80g12.1	-1.526714169	-3.752673892	-1.791649817	-0.447660424
ENSDART00000173000	zgc:113184	0.45806543	0.269621809	0.185268488	0.0480413
ENSDART00000173011	GRXCR1	0.401804428	0.903685752	1.561252834	1.150514684
ENSDART00000173017	atp2b2	-0.485284755	-0.507921276	-0.213758166	0.050306247
ENSDART00000173020	utrn	-0.19228303	-0.092812836	-0.536357201	-0.334471005

ENSDART00000173052	map7d2b	0.261433378	1.055987677	1.048743858	0.7752628
ENSDART00000173056	arap3	-0.20638853	-0.271205037	-0.427111789	-0.285607029
ENSDART00000173060	rph3ab	-0.917567753	-0.595167162	0.673778236	0.835535825
ENSDART00000173072	AKAP13 (1 of many)	-0.027726032	-0.260210303	-0.309219665	0.02079698
ENSDART00000173083	FO704610.1	-0.146610059	-0.222029359	-0.309017936	-0.101957892
ENSDART00000173095	spred3	-0.542534865	-0.320111081	-0.209054264	-0.626959008
ENSDART00000173098	gemin6	0.181263356	0.385271136	0.184325919	0.274013326
ENSDART00000173108	gpc3	-0.979170892	-0.757011653	0.064815285	0.413031736
ENSDART00000173109	nrtn	-0.527610722	-0.443827628	-0.46689713	-0.285016979
ENSDART00000173113	si:ch211-129p13.1	-0.464452233	-0.696136912	-0.478469763	-0.206239945
ENSDART00000173119	pcdh11	-0.634370446	-0.732192902	-0.531501202	-0.25060062
ENSDART00000173126	klhl4	-0.765151123	-0.487744119	-0.047554388	-0.018081928
ENSDART00000173133	utrn	-0.11206335	-0.324597481	-0.223274112	-0.197393841
ENSDART00000173134	BX571952.1	-0.551010684	-0.633352911	-0.460069257	-0.004126181
ENSDART00000173143	lrp1aa	0.006114382	-0.023577911	-0.261635578	-0.555062861
ENSDART00000173145	rpl29	0.32197024	0.280412475	0.068293144	-0.107729465
ENSDART00000173149	dgkab	-0.056470672	-0.094386691	-0.40550946	-0.281791004
ENSDART00000173169	pclob	-0.241926883	-0.695024268	-0.666850982	-0.322920156
ENSDART00000173192	pcdh1b	-0.448371004	-0.996454455	-0.461950735	0.179661768
ENSDART00000173195	zgc:153146	2.291177749	2.913913042	2.827467346	2.606942928
ENSDART00000173210	kcnab2a	-0.633080178	-0.760216594	-0.295624932	-0.02803528
ENSDART00000173222	gpr101	-0.442714055	-0.312795744	-0.164884489	-0.01182968
ENSDART00000173228	slc6a11b	-1.626653664	-2.205227269	-0.432079303	0.774903159
ENSDART00000173237	mid2	-0.115282743	-0.310001722	-0.367171215	-0.341782639
ENSDART00000173257	si:dkey-97l20.6	-0.30598121	-0.315922233	-0.586659381	-0.206936738
ENSDART00000173267	tmem255a	-0.148788206	0.632219165	0.395055581	0.430496671
ENSDART00000173301	sfxn5b	-0.333126218	-0.098925577	-0.061350927	0.22562903
ENSDART00000173305	si:ch1073-456m8.1	0.206580067	0.587987288	0.667691948	0.271613596
ENSDART00000173346	ptp4a1	0.667015175	0.565109215	0.920811931	0.492033169
ENSDART00000173386	cacna1da	-0.09786225	-0.344688682	-0.169985603	0.048564501
ENSDART00000173398	pcdh19	-0.247538527	-0.756226364	0.093604274	0.28627895
ENSDART00000173400	FP236327.1	-0.107319508	-0.233503644	-0.613884847	-0.314094176
ENSDART00000173421	lingo2a	-0.727375414	-0.584905703	-0.169839849	0.100856447
ENSDART00000173423	pcdh11	-0.206659469	-0.430916352	-0.19368235	0.107223081
ENSDART00000173430	pfklb	-0.150367408	-0.216221217	-0.597100494	-0.348773684
ENSDART00000173436	si:dkey-280e21.3	0.740189589	1.373516231	1.387529232	0.976737711
TRANSGENE		3.62113341	4.519443337	2.76591932	1.041497447

Table S2. Differentially expressed transcripts (1% FDR) clustered based on temporal expression pattern.

Ensembl ID	zebrafish gene symbol	padj	LFC: 2dpi-0dpi	LFC: 4dpi-0dpi	LFC: 7dpi-0dpi	LFC: 12dpi-0dpi	cluster name
ENSDART00000001678	adam8a	0.001434846	1.934717518	1.802449426	1.368408045	-0.21633234	growth toward the midline
ENSDART00000002453	acsl4b	0.004676707	0.682559347	0.484852434	0.056581739	-0.183902113	growth toward the midline
ENSDART00000002556	mrto4	0.019563666	0.589078781	0.447556222	0.419365915	0.058126759	growth toward the midline
ENSDART00000002595	rpl21	0.004824695	0.36985839	0.3662658	0.201825483	-0.004056179	growth toward the midline
ENSDART00000002741	itprip	0.000474192	0.724094373	0.694272578	0.397322653	-0.062298334	growth toward the midline
ENSDART00000002945	NPC2 (1 of many)	0.001006824	3.182180208	2.136779184	1.408480393	0.09501531	growth toward the midline
ENSDART00000003001	rpl23a	0.003882278	0.412203534	0.396855245	0.244635942	-0.052563059	growth toward the midline
ENSDART00000003314	nusap1	0.035951327	0.825744448	0.908244509	0.647266522	0.3629471	growth toward the midline
ENSDART00000004065	zgc:91909	0.00334615	0.594004329	0.47164071	0.350756766	0.105236702	growth toward the midline
ENSDART00000004392	fkbp9	0.004270117	0.758370209	0.583896419	0.427971915	-0.289954899	growth toward the midline
ENSDART00000004664	tram1	0.002818463	0.410276471	0.318086482	0.103470746	-0.163408872	growth toward the midline
ENSDART00000004780	man2b1	0.002528575	0.352578138	0.195228445	0.062298599	-0.189062834	growth toward the midline
ENSDART00000006381	psen2	0.01158873	0.338086739	0.204848015	0.067000942	-0.024014416	growth toward the midline
ENSDART00000007642	zgc:110239	2.33589E-05	0.831334701	0.383173307	0.0742642	-0.168536089	growth toward the midline
ENSDART00000007827	spra	0.013245259	0.429147243	0.5653912	0.349944557	-0.12347627	growth toward the midline
ENSDART00000008373	fosl1a	0.001576361	0.78271954	0.570182098	0.226810387	-0.332448721	growth toward the midline
ENSDART00000008807	rpl12	0.006094081	0.258765274	0.397825613	0.190712058	-0.053203791	growth toward the midline
ENSDART00000009164	esco2	0.01613952	1.573729566	0.878542321	0.358323204	0.160150267	growth toward the midline
ENSDART00000009241	rpl35	0.001788569	0.440738233	0.45418276	0.310964715	0.055658665	growth toward the midline
ENSDART00000009343	pyroxd2	0.025731308	0.778556587	0.730253969	0.295998209	0.188824159	growth toward the midline
ENSDART00000010104	crtap	0.001353924	0.225199403	0.115819527	-0.25953471	-0.588755148	growth toward the midline
ENSDART00000010246	ugt1ab	0.02087678	1.578212697	1.169819697	0.509084599	-0.046131609	growth toward the midline
ENSDART00000010261	pno1	0.000847723	0.396735226	0.379415162	0.20506239	-0.042887698	growth toward the midline
ENSDART00000011570	zgc:101716	0.028885428	0.810996291	0.904458728	0.54949556	0.44631829	growth toward the midline
ENSDART00000011863	hdlbpa	0.002972631	0.44689202	0.322223522	0.244841165	-0.050194137	growth toward the midline
ENSDART00000013409	prmt3	0.000336326	0.583250552	0.486757337	0.420985526	-0.028812313	growth toward the midline
ENSDART00000014871	akr7a3	0.017769648	0.445881137	0.133128629	0.063605049	0.058812872	growth toward the midline
ENSDART00000015034	blvrb	0.000281817	0.700108065	0.61517331	0.539502173	-0.031641171	growth toward the midline
ENSDART00000015841	gstt1b	0.000149381	0.844147349	0.559916631	0.300223231	-0.404935077	growth toward the midline
ENSDART00000016814	fmnl2a	0.0006946	0.496433192	0.213976057	-0.143298216	-0.634969289	growth toward the midline
ENSDART00000016864	slc35f6	0.023157709	0.392001078	0.280281029	0.187768856	0.16995993	growth toward the midline
ENSDART00000016890	eif6	0.049349672	0.391815405	0.223439355	0.155230229	0.056706473	growth toward the midline
ENSDART00000017153	hps4	0.00377485	0.574215829	0.524013089	0.377950963	0.050651634	growth toward the midline
ENSDART00000017176	dkc1	0.026351976	0.45463167	0.261033913	0.276931433	0.02149911	growth toward the midline
ENSDART00000018261	akr1b1	0.003041627	1.00051996	0.934286328	0.670184322	0.161867768	growth toward the midline
ENSDART00000018304	mcm3	0.010839093	0.755786584	0.39595981	0.332438259	0.197655199	growth toward the midline
ENSDART00000018461	vmp1	0.013233841	0.430900752	0.323245721	0.184562524	0.049764543	growth toward the midline
ENSDART00000018523	ahcy	0.020417188	0.45208938	0.606696476	0.316876579	-0.072835379	growth toward the midline
ENSDART00000018625	napab	0.000529346	0.495528529	0.395594582	0.072464634	-0.31666363	growth toward the midline
ENSDART00000019003	psmd10	0.013692958	0.510870261	0.455922769	0.143258659	0.040691699	growth toward the midline
ENSDART00000019149	rpl7	0.003098732	0.429749031	0.41708455	0.235093103	-0.015027688	growth toward the midline
ENSDART00000019937	gadd45ga	0.013812134	0.98351774	1.304995817	0.165178221	0.462881948	growth toward the midline
ENSDART00000020167	slc16a9a	0.000935288	0.895506163	0.735954298	0.591907512	-0.466816888	growth toward the midline
ENSDART00000020252	pdia6	0.043796537	0.374033372	0.009098395	0.091775115	0.023604955	growth toward the midline
ENSDART00000020311	rpl27	0.001676296	0.430086554	0.381494789	0.182914397	-0.034120883	growth toward the midline
ENSDART00000020541	lipf	0.000672564	0.528006329	0.723139512	0.390796686	-0.03933789	growth toward the midline
ENSDART00000020655	psma5	0.023717938	0.424960543	0.53028483	0.342202642	0.090736505	growth toward the midline
ENSDART00000020810	sdcbp2	0.003682945	0.429533724	0.242026939	0.181097661	-0.126536592	growth toward the midline
ENSDART00000021037	hspa4a	0.022587526	0.42074258	0.381012047	0.243421438	0.132816774	growth toward the midline
ENSDART00000021069	rpl38	0.001331938	0.561397297	0.414839383	0.25425723	0.049186317	growth toward the midline
ENSDART00000021299	nmd3	0.014686352	0.353895501	0.176055483	0.10015969	0.031379837	growth toward the midline
ENSDART00000021788	pbk	0.043154442	1.438031033	0.973567544	0.667760742	0.195406357	growth toward the midline
ENSDART00000021798	fabp11a	0.000424137	3.578286828	1.692122399	1.073895347	-0.266988673	growth toward the midline
ENSDART00000021950	mthfd1b	0.002507467	1.089156306	0.599589593	0.18588711	-0.096895618	growth toward the midline
ENSDART00000022307	atic	4.11188E-05	1.012801143	0.518350872	0.289570342	-0.11925608	growth toward the midline
ENSDART00000022562	rhogb	0.013807548	0.97771224	0.454579503	0.287206454	-0.277565993	growth toward the midline
ENSDART00000022586	lrrc40	0.005807456	0.466457292	0.391920358	0.288821604	-0.015697706	growth toward the midline
ENSDART00000022634	acp2	0.004900248	0.356430058	0.295051752	0.030540215	-0.026401831	growth toward the midline
ENSDART00000022765	riok1	0.014326582	0.429965951	0.477495547	0.301061387	0.021181676	growth toward the midline
ENSDART00000023463	uap1l1	0.015884799	0.706648736	0.512468414	0.225773419	-0.015907861	growth toward the midline
ENSDART00000024194	kif11	0.007786427	1.409119378	1.269759488	0.810392576	0.401186663	growth toward the midline
ENSDART00000024208	nutf2l	0.001633797	0.951686549	0.855710394	0.537014601	-0.220899011	growth toward the midline
ENSDART00000024316	mcm5	0.041407171	1.292009744	0.913737369	0.774968175	0.275487022	growth toward the midline
ENSDART00000025096	larp1b	0.002618468	0.593217694	0.576647108	0.472990072	0.196836578	growth toward the midline
ENSDART00000025229	adi1	0.000305093	0.582007509	0.274560712	-0.200617197	-0.21352575	growth toward the midline
ENSDART00000025912	si:dkey-32n7.4	0.055404088	0.676729111	0.251567535	0.290364244	0.056388056	growth toward the midline
ENSDART00000026145	AMOTL1	0.001213585	1.0586494	0.570711762	0.741896115	0.335943589	growth toward the midline
ENSDART00000026178	kif4	0.009637478	0.630775369	0.400037801	0.195646982	0.069714171	growth toward the midline
ENSDART00000026492	flncb	0.000598789	1.767414623	1.156524725	0.931280558	0.481368808	growth toward the midline
ENSDART00000027393	ckmt1	0.033377037	0.722688207	0.598583347	0.435615456	0.204934783	growth toward the midline
ENSDART00000029133	snu13b	0.001170697	0.760760317	0.56984098	0.429270049	-0.029914173	growth toward the midline
ENSDART00000029380	bnip4	0.000434317	0.50246171	0.517948631	-0.062532971	-0.337429982	growth toward the midline

ENSDART00000029387	ppan	0.025186512	0.365110957	0.289310564	0.234072786	0.008675203	growth toward the midline
ENSDART00000030125	znhit3	0.032553766	0.760210037	0.691966223	0.487369205	0.388320697	growth toward the midline
ENSDART00000030890	hmox1a	0.001147964	2.623035746	1.043671484	1.096486903	0.061370069	growth toward the midline
ENSDART00000031047	cd63	0.001899261	1.025048213	0.521327717	0.067995805	-0.605090068	growth toward the midline
ENSDART00000031498	ccna2	0.001588425	1.569664461	1.378683751	0.895010724	0.581055828	growth toward the midline
ENSDART00000032290	esyt1a	0.001565956	0.414133861	0.186544426	0.071532791	-0.03493067	growth toward the midline
ENSDART00000032498	tspan36	2.7529E-05	1.455763612	0.805559086	0.192569945	-0.715366149	growth toward the midline
ENSDART00000033386	ocstamp	0.013948629	2.954317372	2.548446344	2.458244526	1.328553982	growth toward the midline
ENSDART00000033574	slc24a5	0.00217038	0.460212086	0.054768672	-0.306746704	-1.141417252	growth toward the midline
ENSDART00000033663	rps21	0.005949363	0.311890725	0.366197651	0.105076559	-0.038309008	growth toward the midline
ENSDART00000033761	glb1	0.021559058	0.347409105	0.187375641	0.031649312	0.073918812	growth toward the midline
ENSDART00000033980	lims1	0.002007275	0.497849916	0.369061975	0.247283344	-0.174205881	growth toward the midline
ENSDART00000034248	rab32a	0.001863533	0.364170748	0.270180665	-0.161567701	-0.659765413	growth toward the midline
ENSDART00000034377	cpa5	0.000100528	2.043914839	0.12107472	0.19928898	0.161423339	growth toward the midline
ENSDART00000034829	rrp12	0.00217038	0.494057186	0.423927766	0.372142811	0.099318986	growth toward the midline
ENSDART00000034850	dbi	0.002568245	1.011739121	0.830016329	0.708552366	0.209278197	growth toward the midline
ENSDART00000035093	col9a2	0.002649033	0.360349924	0.265960604	-0.052354611	-0.707154609	growth toward the midline
ENSDART00000035670	polr2eb	0.0033491	0.35113115	0.294169174	0.063963778	-0.045691283	growth toward the midline
ENSDART00000035739	tmem134	0.001247858	0.246242014	0.369929781	0.082611565	-0.161334554	growth toward the midline
ENSDART00000035944	clic5a	0.068646243	2.554130845	1.311062136	1.150965478	0.646568675	growth toward the midline
ENSDART00000036729	spi1b	0.004358158	0.910663058	0.717004711	0.095052493	-0.117318513	growth toward the midline
ENSDART00000037224	cst14a.2	0.003240086	0.549538616	0.553010386	0.34149596	-0.057482802	growth toward the midline
ENSDART00000038202	cndp2	0.001490782	1.63370988	0.725493655	0.197899383	-0.10985912	growth toward the midline
ENSDART00000038301	gnpda2	0.008531138	0.314221876	0.240267665	0.128522583	-0.033807169	growth toward the midline
ENSDART00000038696	flvcr2b	0.014130752	1.475160998	0.41510575	0.393658072	-0.063620422	growth toward the midline
ENSDART00000038888	hsdl2	0.018248495	0.331360296	0.293802861	0.248668235	0.085382052	growth toward the midline
ENSDART00000038990	jak1	0.006256994	0.414591598	0.220201928	0.070657217	-0.102548961	growth toward the midline
ENSDART00000039206	rps23	0.004633654	0.388349294	0.322594165	0.070172062	-0.095059659	growth toward the midline
ENSDART00000039312	ipo4	0.002755913	0.472349209	0.340323729	0.32654819	0.07839591	growth toward the midline
ENSDART00000040434	asah1b	0.000524004	0.563509259	0.420868789	-0.056225927	-0.32971968	growth toward the midline
ENSDART00000040771	rpl34	0.005202212	0.291435523	0.406434571	0.222752448	-0.006604457	growth toward the midline
ENSDART00000040827	ncaph2	0.025606413	0.654906877	0.777407912	0.63027477	0.226543553	growth toward the midline
ENSDART00000040900	baxb	0.005486177	0.66837016	0.786823818	0.515591205	0.094501002	growth toward the midline
ENSDART00000041114	psmb2	0.010442784	0.343581501	0.272779677	0.256301247	-0.006269993	growth toward the midline
ENSDART00000041504	tescb	0.004106215	0.44458368	-0.282095189	0.067591772	-0.116025945	growth toward the midline
ENSDART00000041992	dhrs12	0.001102685	0.21282475	0.60945999	-0.222288782	-0.248821854	growth toward the midline
ENSDART00000042250	rap1b	0.003665031	0.339690926	0.041784403	0.019413921	-0.132436477	growth toward the midline
ENSDART00000043173	rpl18	0.005040201	0.389153851	0.347510342	0.235541148	0.00423952	growth toward the midline
ENSDART00000043666	hs1bp3	0.003138642	1.060777617	0.463082325	0.168627927	-0.515305865	growth toward the midline
ENSDART00000043678	apobec2b	0.024850978	1.815363093	0.931239114	0.794076282	0.812995775	growth toward the midline
ENSDART00000044238	zgc:92066	0.000787471	1.060411872	1.124461317	0.621339815	0.051820002	growth toward the midline
ENSDART00000044264	mmp14b	0.018216055	0.997152397	1.115840894	0.915921565	0.094130452	growth toward the midline
ENSDART00000044314	itgav	0.004681738	0.188708464	-0.009103257	-0.190172369	-0.475418475	growth toward the midline
ENSDART00000044328	acss1	0.000783264	0.948368639	0.660526303	0.176495325	-0.460639914	growth toward the midline
ENSDART00000045933	sh3glb1b	0.002439392	0.424660866	0.274153114	0.044458674	-0.153303197	growth toward the midline
ENSDART00000046218	flnca	0.000103226	6.980277858	6.302871335	6.08375751	5.531037957	growth toward the midline
ENSDART00000046253	prkcq	0.008130201	0.856641094	1.199706901	0.196829623	-0.396081861	growth toward the midline
ENSDART00000046268	pmelb	0.000596344	0.894230709	0.185058494	-0.384535315	-0.811085128	growth toward the midline
ENSDART00000047191	glb1l	0.017715642	1.329711044	0.552831622	0.213363411	-0.364178392	growth toward the midline
ENSDART00000047728	melk	0.061287033	0.808255278	0.366287521	0.23278244	0.524042022	growth toward the midline
ENSDART00000048107	FP102018.1	0.000888975	0.402516182	0.222629184	-0.14999501	-0.581967671	growth toward the midline
ENSDART00000049465	slc19a1	0.0055878	0.32288456	0.126425631	-0.165912503	-0.040050165	growth toward the midline
ENSDART00000050399	npc2	0.002939613	0.729567353	0.287798394	-0.063690935	-0.310097689	growth toward the midline
ENSDART00000050847	GLDC	0.017654181	0.384969982	0.265449522	0.198357794	0.108750757	growth toward the midline
ENSDART00000051392	spns3	0.001042039	1.320734766	0.750958136	0.798555508	-0.096372125	growth toward the midline
ENSDART00000051491	sfrp1a	0.001342771	0.324084954	0.211296795	-0.122014524	-1.0775933	growth toward the midline
ENSDART00000051556	abca1b	0.004394152	0.962705135	0.587027009	0.239926413	-0.279230387	growth toward the midline
ENSDART00000051763	rps3a	0.02114031	0.36528781	0.402890251	0.287932185	0.011460462	growth toward the midline
ENSDART00000051906	ube2c	0.012497601	1.239012745	1.032037835	0.553625781	0.164264884	growth toward the midline
ENSDART00000052082	rpl30	0.00417836	0.377785904	0.342931306	0.098750304	-0.086094728	growth toward the midline
ENSDART00000052090	fuca1.2	0.012864775	0.607737239	0.489644017	0.400376939	0.091450373	growth toward the midline
ENSDART00000052104	fuca1.1	0.006465135	0.706031916	0.625980807	0.554044121	0.174374129	growth toward the midline
ENSDART00000052331	rps20	0.004925327	0.422414342	0.403711723	0.215205833	-0.059084102	growth toward the midline
ENSDART00000052730	rps13	0.00538212	0.392793291	0.407259594	0.231403052	0.009148185	growth toward the midline
ENSDART00000052761	rpl39	0.005353377	0.335691979	0.34926929	0.1141905	-0.000371665	growth toward the midline
ENSDART00000052917	slc3a2a	0.001434846	0.509465627	0.333845604	-0.024881296	-0.285712584	growth toward the midline
ENSDART00000053761	bms1	0.015390643	0.415838377	0.296751152	0.241962107	0.100266391	growth toward the midline
ENSDART00000053932	cbsa	0.000261206	1.196219296	0.806157212	0.553945426	0.279319174	growth toward the midline
ENSDART00000055287	zgc:109934	0.00013154	0.762332695	0.487859158	-0.045189444	-0.423101752	growth toward the midline
ENSDART00000055328	nek6	0.003694199	0.491226657	0.428884947	0.11516767	-0.052210357	growth toward the midline
ENSDART00000055428	cbx7a	0.000405977	0.780269243	0.615351279	0.524687311	-0.270346219	growth toward the midline
ENSDART00000056005	ascl1a	0.032093457	1.787445127	1.283325532	0.749862779	0.792492648	growth toward the midline
ENSDART00000056457	mitfa	0.005353377	0.929754118	0.38849893	0.30836176	-0.311729197	growth toward the midline
ENSDART00000056460	gbp1	0.045564466	3.785035011	3.151116027	3.74676295	2.092352698	growth toward the midline
ENSDART00000056686	mrc1b	0.004463264	0.993000172	0.810204425	0.489266441	-0.068136928	growth toward the midline

ENSDART00000058324	rpz4	0.00855067	0.754354311	0.61115296	0.469864295	-0.17226809	growth toward the midline
ENSDART00000058605	scpep1	0.002139835	0.659557613	0.259300703	0.157418507	-0.140654612	growth toward the midline
ENSDART00000058665	kif20bb	0.029064716	0.867797834	0.730592963	0.371647572	0.126469391	growth toward the midline
ENSDART00000058774	havcr1	0.002175185	1.378230425	0.803624411	0.332422006	0.11017803	growth toward the midline
ENSDART00000059001	C5AR1	0.048093489	0.862718576	0.211750471	0.205995335	0.011542683	growth toward the midline
ENSDART00000059013	sec61b	0.022498542	0.564454151	0.292288284	0.352954607	0.073391744	growth toward the midline
ENSDART00000059667	wdr75	0.022389941	0.34735847	0.29669502	0.189255306	0.039773826	growth toward the midline
ENSDART00000060363	rpl4	0.004878958	0.373964359	0.396853604	0.212646351	-0.016195336	growth toward the midline
ENSDART00000060444	rps29	0.007150368	0.369322836	0.397792199	0.13443893	-0.022749507	growth toward the midline
ENSDART00000060745	uba52	0.01809863	0.32327466	0.351581625	0.155851402	-0.01111286	growth toward the midline
ENSDART00000060765	nppb	0.001237101	4.501656565	4.501350371	1.812509246	-0.191856821	growth toward the midline
ENSDART00000060773	taar12b	0.025216223	2.538886261	1.95415769	1.735717491	1.72259581	growth toward the midline
ENSDART00000060865	rasal1b	0.024284969	1.120636523	0.769242819	0.458217171	0.392656742	growth toward the midline
ENSDART00000061736	si:dkey-4e7.3	0.017999639	0.80946555	0.660586871	0.14634027	0.03058403	growth toward the midline
ENSDART00000061926	stx11b.2	0.006909332	3.447906519	2.671571656	2.785952697	1.680276674	growth toward the midline
ENSDART00000062257	slc39a1	0.003541697	0.740781667	0.522946282	0.350404642	0.245154327	growth toward the midline
ENSDART00000062576	thyn1	0.006623227	0.902996379	0.741810201	0.792519989	0.219218083	growth toward the midline
ENSDART00000062671	tuba8l	0.009931768	1.253108099	0.545680562	0.42270127	-0.336104805	growth toward the midline
ENSDART00000062845	mmp9	0.014615758	2.08287012	1.917473556	1.298502519	-0.06470518	growth toward the midline
ENSDART00000062931	abracl	0.011668571	0.878200021	0.73529721	0.710676136	0.289919909	growth toward the midline
ENSDART00000062983	rpl10a	0.003138642	0.388498934	0.402298329	0.17374506	-0.059011317	growth toward the midline
ENSDART00000063107	cyp2p7	0.024411518	0.910849098	1.165275545	0.616791402	-0.023701278	growth toward the midline
ENSDART00000063251	ctsz	3.54236E-05	1.117383701	0.530773826	0.448185866	0.142841944	growth toward the midline
ENSDART00000063357	ccnb1	0.012018385	1.564145903	1.350801576	0.91228963	0.49209964	growth toward the midline
ENSDART00000063764	si:dkey-5n18.1	0.015077117	1.420288455	0.932190706	0.716992196	-0.355341421	growth toward the midline
ENSDART00000063874	vamp4	0.003602728	0.706049434	0.688342777	0.412447235	0.06985511	growth toward the midline
ENSDART00000063953	zgc:65997	0.015420308	0.11562047	0.484476273	-0.11592626	-0.024456549	growth toward the midline
ENSDART00000064113	abt1	0.009820514	0.598506286	0.454995795	0.386684419	0.116646594	growth toward the midline
ENSDART00000064511	il17a/f1	0.023872337	1.319538931	1.072672417	0.769667562	1.083526716	growth toward the midline
ENSDART00000064657	stx11a	0.006345193	1.187636483	0.704469765	0.33181163	-0.135694961	growth toward the midline
ENSDART00000064700	fuca2	0.001008722	0.436466876	0.137195427	-0.005101419	-0.171105064	growth toward the midline
ENSDART00000065057	itgb7	0.000118474	1.200094562	0.885337917	0.497721229	0.006866552	growth toward the midline
ENSDART00000065183	cldn2	0.000739355	0.624880574	0.284479914	-0.382379938	-0.379228488	growth toward the midline
ENSDART00000065208	nop16	0.021355398	0.539941841	0.465464987	0.330311237	0.01578578	growth toward the midline
ENSDART00000065337	kif20a	0.001229585	1.518648505	1.334799108	0.992824147	0.572892998	growth toward the midline
ENSDART00000065674	fybb	0.008250091	0.68375018	0.334314613	0.079534024	-0.242430054	growth toward the midline
ENSDART00000065805	tspan10	0.003267854	0.90782785	0.406843364	-0.022479662	-0.576522773	growth toward the midline
ENSDART00000065817	pou5f3	0.012966692	1.443769101	1.494882152	0.928756649	0.660724256	growth toward the midline
ENSDART00000066411	dlgap5	0.003138642	1.041688171	0.895823709	0.540002804	0.33349173	growth toward the midline
ENSDART00000066778	acad11	0.000741396	0.796145744	0.566674022	0.216041695	-0.190350168	growth toward the midline
ENSDART00000067193	adm2a	0.004020103	0.4356689	0.184728608	-0.002686414	-1.106691874	growth toward the midline
ENSDART00000067312	si:dkey-106n21.1	0.003805801	0.584969753	0.102146183	-0.031661382	0.069325035	growth toward the midline
ENSDART00000067594	cst14b.1	0.003629485	1.003408293	1.132156972	0.601894798	0.102871452	growth toward the midline
ENSDART00000067599	angptl2a	0.000103226	1.671461404	1.166310219	0.762115684	0.407368061	growth toward the midline
ENSDART00000067678	zgc:110339	0.001560042	0.949255799	0.562708107	0.125395868	-0.585210787	growth toward the midline
ENSDART00000073846	si:ch211-122f10.4	0.006183684	0.620706192	0.26696454	0.219379439	-0.011733355	growth toward the midline
ENSDART00000074010	ubald1b	0.002498008	0.486188172	0.523878559	0.246068485	0.014830353	growth toward the midline
ENSDART00000074161	slc4a2b	0.000953608	0.481685785	0.057728735	-0.200942778	-0.247347753	growth toward the midline
ENSDART00000074384	stx4	0.006812131	0.365157378	0.335603422	0.150714004	-0.050227712	growth toward the midline
ENSDART00000074438	cenpi	0.023769243	0.547415003	0.708397465	0.401862169	0.054393549	growth toward the midline
ENSDART00000075028	rps11	0.001443362	0.42100484	0.441088802	0.128015907	-0.080449898	growth toward the midline
ENSDART00000075262	cad	0.058741505	0.41294438	0.156970425	0.074105835	0.04769287	growth toward the midline
ENSDART00000075286	slc2a15b	0.000993187	1.023992215	0.671159354	0.170031714	-0.73573625	growth toward the midline
ENSDART00000075495	rpl23	0.005969755	0.390673252	0.341184618	0.264108815	-0.005521244	growth toward the midline
ENSDART00000076030	fbl	0.017381552	0.33280558	0.192834191	0.116054515	0.005015438	growth toward the midline
ENSDART00000076636	fzd2	0.002013265	0.563199333	0.424474998	0.171148118	-0.301884624	growth toward the midline
ENSDART00000077185	dgat1b	0.002147221	0.79138422	0.60487817	0.419420549	-0.346047138	growth toward the midline
ENSDART00000077259	ebna1bp2	0.007621679	0.420936188	0.344892308	0.188849483	0.008127131	growth toward the midline
ENSDART00000077386	prss16	0.000185931	0.714972584	0.32483027	0.071612523	-0.240875958	growth toward the midline
ENSDART00000077418	ctsba	0.016904127	1.550723066	1.033464808	0.97579905	0.475659932	growth toward the midline
ENSDART00000077511	ccr9a	0.000592988	1.513300322	1.48326526	0.981085195	0.082724583	growth toward the midline
ENSDART00000077545	slc7a7	5.02856E-05	1.304066715	0.45830859	0.136139102	0.056436424	growth toward the midline
ENSDART00000077707	llph	0.006605202	0.612018484	0.617804437	0.382050706	0.042759152	growth toward the midline
ENSDART00000077715	si:dkey-25e12.3	0.000947019	1.07190149	0.953044464	0.701373655	0.117439727	growth toward the midline
ENSDART00000077834	rps27.2	0.003329747	0.365621497	0.39961171	0.113670294	-0.081169937	growth toward the midline
ENSDART00000078266	rsl1d1	0.000775831	0.351365949	0.392552672	0.175928217	-0.070353732	growth toward the midline
ENSDART00000078596	hspd1	0.004163372	0.512169965	0.358816938	0.201006014	0.20720259	growth toward the midline
ENSDART00000078634	eif5a2	0.011846009	0.493280465	0.485481598	0.418411017	0.051860815	growth toward the midline
ENSDART00000078647	si:ch211-201h21.5	0.024809879	1.148616031	0.689283012	0.40532037	0.049879214	growth toward the midline
ENSDART00000078866	ifi30	0.001277839	1.574791131	0.896478525	0.373397245	-0.020268978	growth toward the midline
ENSDART00000079222	chaf1b	0.034090908	1.242408116	0.820706616	0.642546621	0.111426229	growth toward the midline
ENSDART00000079310	manba	0.003137832	0.495609624	0.109254813	0.005925635	-0.12409296	growth toward the midline
ENSDART00000079549	tpte	0.002192481	0.809167451	0.638911968	0.260848622	-0.250321254	growth toward the midline
ENSDART00000079695	zwilch	0.007014909	1.159509442	0.995361025	0.861360163	0.105564769	growth toward the midline
ENSDART00000079984	rpl22l1	0.0003783	0.678692893	0.584241724	0.331994973	-0.011632328	growth toward the midline

ENSDART00000080339	galm	0.000273326	0.868912331	0.484945087	-0.111697277	-0.429352779	growth toward the midline
ENSDART00000080377	aldoca	0.036855016	2.758856293	1.548001605	2.09362575	1.794239524	growth toward the midline
ENSDART00000080465	hells	0.018490842	0.812911201	0.936234984	0.557478835	0.182957003	growth toward the midline
ENSDART00000080523	itgae.2	0.00855067	1.067269567	0.812178684	0.361968863	0.003662922	growth toward the midline
ENSDART00000080712	slc43a3b	0.00855067	2.109023631	1.075213126	0.724428131	-0.154263604	growth toward the midline
ENSDART00000080864	magt1	0.006145665	0.441285986	0.302582629	0.047526992	-0.172355248	growth toward the midline
ENSDART00000080919	rpl36a	0.000996442	0.405736348	0.485454355	0.225930563	-0.002722727	growth toward the midline
ENSDART00000081343	plk1	0.006812131	1.547304713	1.292689538	1.02080622	0.322979307	growth toward the midline
ENSDART00000081568	tcf19l	0.027985198	0.712871286	0.397385898	0.351815803	0.227291981	growth toward the midline
ENSDART00000082011	lim2.2	0.05615844	1.304610538	0.978295786	0.86763989	0.856876261	growth toward the midline
ENSDART00000082050	zgc:174904	0.01376164	1.977093547	1.430607855	1.439069833	0.228460651	growth toward the midline
ENSDART00000082471	mfap2	0.013128447	0.61383585	0.779035446	0.354750417	0.055843134	growth toward the midline
ENSDART00000082517	rab43	0.000993187	0.357940531	0.212689136	-0.148032038	-0.068856369	growth toward the midline
ENSDART00000083033	sik1	0.0147298	1.146093805	0.631897288	0.512350737	1.123188354	growth toward the midline
ENSDART00000083126	cidec	0.00067643	3.092638419	2.402632883	1.147285193	-0.231828568	growth toward the midline
ENSDART00000083294	nol6	0.046592019	0.354535252	0.232360208	0.196448331	0.109158776	growth toward the midline
ENSDART00000083830	sdc4	0.000487602	0.356242427	0.466156404	0.190102023	-0.370187936	growth toward the midline
ENSDART00000084069	iqgap2	0.000512675	1.059547107	0.55852141	0.220571876	-0.354231411	growth toward the midline
ENSDART00000085309	dpcd	0.00270716	0.675929667	0.665840567	0.458693669	0.16198721	growth toward the midline
ENSDART00000085764	PLOD3	0.005493417	0.414167142	0.202506542	0.107388816	-0.10168282	growth toward the midline
ENSDART00000087114	alg5	0.020108219	0.317519293	0.464842808	0.220188706	0.059168575	growth toward the midline
ENSDART00000087311	oca2	0.000313682	0.449332393	0.338475194	-0.09076694	-0.174077968	growth toward the midline
ENSDART00000088042	myo10l3	0.014447102	0.440398704	0.46516816	0.325219038	-0.023584336	growth toward the midline
ENSDART00000088973	sytl2a	0.000317136	0.735131572	0.479031428	-0.049007315	-0.597552798	growth toward the midline
ENSDART00000089158	hmha1a	0.027703711	1.15366088	0.773436896	0.705540127	-0.035689576	growth toward the midline
ENSDART00000090528	rhoca	7.15704E-05	0.570199757	0.738551724	0.41277501	-0.074138712	growth toward the midline
ENSDART00000090883	gpnmb	0.001083951	1.78024327	1.792109343	1.484400673	-0.264951498	growth toward the midline
ENSDART00000091662	noc2l	0.007264334	0.778877814	0.364993344	0.393512667	-0.105586454	growth toward the midline
ENSDART00000092050	stab1	0.023363274	0.830009942	0.662655195	0.610953846	0.245694157	growth toward the midline
ENSDART00000092389	nup210	0.027686308	0.743287563	0.315006844	0.505674911	0.088834448	growth toward the midline
ENSDART00000093279	spi1b	0.002640644	0.891535687	0.563855895	0.100587929	-0.531177734	growth toward the midline
ENSDART00000098545	tmem150aa	0.010056242	0.458575321	0.166363456	0.137813682	-0.033806431	growth toward the midline
ENSDART00000098970	lin28a	0.050549016	3.476899763	1.864207832	0.92280153	0.893269961	growth toward the midline
ENSDART00000099138	ncf2	0.012263789	0.590402106	0.352177279	0.071326562	-0.071022128	growth toward the midline
ENSDART00000099839	map2k2b	0.04077574	4.411409189	4.089988152	4.160217635	3.732217772	growth toward the midline
ENSDART00000099947	samsn1a	0.007966006	0.989297054	0.26224771	0.078735734	-0.489522484	growth toward the midline
ENSDART00000099978	pdlim4	0.008900663	0.618309518	0.542997573	0.115667653	-0.045450127	growth toward the midline
ENSDART00000100145	lgals9l1	0.008814877	1.730493466	0.73620688	0.17444521	-0.331961456	growth toward the midline
ENSDART00000100458	si:dkey-73n10.1	0.0021138	1.648347098	0.766263068	0.204715566	-0.361349683	growth toward the midline
ENSDART00000100473	PLIN3	0.014485141	1.275027857	0.887739595	0.535269477	-0.111777778	growth toward the midline
ENSDART00000100813	rps24	0.004853289	0.369574152	0.327820695	0.143165091	-0.049601165	growth toward the midline
ENSDART00000101037	nhp2	0.003430859	0.465910387	0.391566586	0.240162003	-0.008355829	growth toward the midline
ENSDART00000101124	rnaseka	0.001851882	1.077486687	0.59165486	0.038549521	-0.354475184	growth toward the midline
ENSDART00000101143	mhc1zea	0.00820374	0.57323499	0.526487775	0.424884066	0.07882824	growth toward the midline
ENSDART00000101319	zgc:162396	0.01098705	0.344606585	0.405244483	0.227818995	0.060602938	growth toward the midline
ENSDART00000102062	timp2b	0.000933245	0.538603085	0.084002119	-0.039686845	-0.313946909	growth toward the midline
ENSDART00000102434	ehhadh	0.000776925	0.70922182	0.453174195	0.063745289	-0.562849237	growth toward the midline
ENSDART00000102459	rbp2a	0.000345998	3.492314469	2.902874566	1.90821276	1.340524369	growth toward the midline
ENSDART00000102981	col8a1a	0.017381552	1.700212586	0.444548046	0.772515926	0.395334934	growth toward the midline
ENSDART00000103076	arl8bb	0.002876227	0.705280948	0.466975375	0.584557674	0.096505859	growth toward the midline
ENSDART00000103368	rpl22	0.006345193	0.33350855	0.471741899	0.142215466	-0.053336675	growth toward the midline
ENSDART00000103491	rbp7b	0.000751152	2.051254968	1.285649693	0.558375641	-0.074104197	growth toward the midline
ENSDART00000103602	lgals2a	0.013513343	1.886236295	1.628346862	1.325259297	0.487532368	growth toward the midline
ENSDART00000103660	clcn7	0.002459431	0.412276024	0.162279088	-0.074638152	-0.273644307	growth toward the midline
ENSDART00000104519	stat3	0.004480141	0.6458865	0.459098575	0.438501049	0.201812376	growth toward the midline
ENSDART00000104687	pfn2	0.00311878	0.330558178	0.38737784	0.240819292	-0.058450663	growth toward the midline
ENSDART00000104835	hps5	0.005167494	0.972059444	0.583168765	0.57443346	-0.161192354	growth toward the midline
ENSDART00000105174	stm	0.000739646	3.414215545	0.041843024	-0.026881567	-0.531574221	growth toward the midline
ENSDART00000108581	si:dkey-17m8.1	0.014069453	0.789679514	0.574820222	0.300394764	-0.045841382	growth toward the midline
ENSDART00000108995	tex2	0.041898142	0.515345319	0.756490823	0.434164856	0.106684566	growth toward the midline
ENSDART00000109065	ccng1	0.001754036	0.498527324	0.814436551	0.266438219	-0.054038693	growth toward the midline
ENSDART00000109416	smc2	0.00879202	1.321753383	1.108242465	0.857262832	0.308883411	growth toward the midline
ENSDART00000109432	cercam	0.000991767	0.726258015	0.540687546	0.072936326	-0.232601995	growth toward the midline
ENSDART00000109464	g0s2	8.33897E-05	2.993222235	1.499538045	0.588261277	0.527162957	growth toward the midline
ENSDART00000109752	serpinh1a	0.000595605	0.578805227	0.380412306	0.080680913	-0.569755212	growth toward the midline
ENSDART00000109831	BX088653.1	0.022613341	0.96716269	0.47589947	0.338658984	-0.013815104	growth toward the midline
ENSDART00000110270	pwp2h	0.011929276	0.562435944	0.598132459	0.373677258	0.062993981	growth toward the midline
ENSDART00000110529	bub1bb	0.018115022	1.321570387	1.337769105	1.007493385	0.59867765	growth toward the midline
ENSDART00000110866	cln5	0.001248486	0.673394988	0.327334357	0.152385216	-0.174612372	growth toward the midline
ENSDART00000110935	si:ch1073-59l16.1	0.005875165	1.356882908	0.676384004	0.249167454	0.069788375	growth toward the midline
ENSDART00000111002	si:ch211-74f19.2	0.000220872	0.463826256	0.294740419	-0.053551592	-0.452436201	growth toward the midline
ENSDART00000111140	rpl29	0.009681558	0.336664553	0.356246951	0.105865493	-0.061220703	growth toward the midline
ENSDART00000111321	zgc:152830	0.001021091	0.702895787	0.346388089	0.27465484	0.003141495	growth toward the midline
ENSDART00000111531	epdl1	0.000962755	1.357192284	1.365774088	0.753975689	0.235060778	growth toward the midline
ENSDART00000111688	zgc:109934	0.000655075	0.61674072	0.212807687	-0.164219088	-0.482167701	growth toward the midline

ENSDART00000111806	cenpe	0.002947105	1.006157891	0.692740621	0.695639634	0.175054418	growth toward the midline
ENSDART00000112127	dnase2b	0.000645779	0.959684518	0.954914684	0.226944152	-0.0439051	growth toward the midline
ENSDART00000112155	rrm2	0.028641638	1.131329801	1.215867796	0.761186289	0.339828406	growth toward the midline
ENSDART00000112243	crlf1a	0.00448838	1.589627089	0.916105309	0.558277873	-0.020827681	growth toward the midline
ENSDART00000112470	ccdc88b	0.005891084	0.86030789	0.601824136	0.501988839	-0.28013627	growth toward the midline
ENSDART00000112653	wdfy4	0.003815452	0.841684139	0.566530892	0.08064366	-0.41519717	growth toward the midline
ENSDART00000113093	gba	0.00060684	0.473554928	0.246228233	-0.028007284	-0.118288896	growth toward the midline
ENSDART00000113259	TARBP1	0.002686125	0.600840202	0.638996908	0.05884907	-0.019773553	growth toward the midline
ENSDART00000113405	inpp5d	0.005845428	0.955185108	0.470897855	0.050793817	-0.341647614	growth toward the midline
ENSDART00000113441	mylk5	2.33589E-05	0.7890096	0.412195956	-0.130414718	-0.622741882	growth toward the midline
ENSDART00000113574	anln	0.001248576	1.428520356	0.903846206	0.598652735	0.284032371	growth toward the midline
ENSDART00000113589	gpx4b	0.002390041	0.633021831	-0.10742897	-0.312510701	-0.292370818	growth toward the midline
ENSDART00000113673	scpp5	0.009220964	1.321767426	0.530715467	0.931995129	0.748355856	growth toward the midline
ENSDART00000113789	zgc:198419	0.012732777	1.388791656	0.463187117	0.161859067	-0.160692863	growth toward the midline
ENSDART00000114000	zgc:101663	0.003860077	0.831482698	0.254442158	0.060051833	0.091105397	growth toward the midline
ENSDART00000114023	hspb9	0.063359837	2.157513115	1.55136025	0.742718373	1.461470885	growth toward the midline
ENSDART00000114282	rpsa	0.000461528	0.494276409	0.531800028	0.113194341	-0.004402994	growth toward the midline
ENSDART00000114410	wdr62	0.02432669	0.584622073	0.50588924	0.329377091	0.089655627	growth toward the midline
ENSDART00000114964	zgc:165573	0.000117076	0.691787708	0.943542464	0.634593348	-0.039695615	growth toward the midline
ENSDART00000115049	mych	6.48692E-06	2.666296724	2.42380403	1.76502541	0.819436082	growth toward the midline
ENSDART00000115255	mcf2l2	0.002771387	0.795464349	0.848343644	0.628078884	0.002922432	growth toward the midline
ENSDART00000117636	dre-mir-21-1	0.001938223	1.553092638	1.563315667	0.906878174	0.77260601	growth toward the midline
ENSDART00000117680	dre-mir-21-2	0.000180784	3.366495632	3.222859121	2.199417673	1.472943682	growth toward the midline
ENSDART00000121998	grwd1	0.006122188	0.591308312	0.087252775	-0.045976929	-0.167650618	growth toward the midline
ENSDART00000122238	tyr	0.000536544	1.0676401	0.617097515	0.165901971	-0.685666733	growth toward the midline
ENSDART00000122288	ctsla	0.000770834	0.76727009	0.372627978	0.369896487	-0.200637952	growth toward the midline
ENSDART00000122321	slc33a1	0.014050993	0.868257035	0.839034235	0.725290511	0.201428784	growth toward the midline
ENSDART00000122407	cdk1	0.003977971	1.677931921	1.534655447	0.824563588	0.384011667	growth toward the midline
ENSDART00000122692	CABZ01112317.1	0.003971559	0.655801165	0.399271741	0.354973485	-0.117162708	growth toward the midline
ENSDART00000122768	CU984579.1	0.001277839	0.668419525	0.524624014	0.112892825	-0.531459653	growth toward the midline
ENSDART00000122898	phf21b	0.008936495	0.630758244	-0.049111481	0.173874589	0.290793166	growth toward the midline
ENSDART00000122945	noc2l	0.001509592	0.680401135	0.443128484	0.389864365	0.196289408	growth toward the midline
ENSDART00000122953	gnl3	0.004847659	0.534095037	0.261409226	0.267291075	0.063642911	growth toward the midline
ENSDART00000123002	nme4	0.004429413	3.640965481	3.577167408	3.045307609	2.652336979	growth toward the midline
ENSDART00000123117	ggh	0.000844564	0.485870455	0.424307993	0.067357919	-0.14640404	growth toward the midline
ENSDART00000123473	Metazoa_SRP	0.011135896	0.899034068	1.683872421	0.662894374	-0.48789514	growth toward the midline
ENSDART00000123544	tex2	0.010129858	0.532623113	0.735572961	0.212010938	-0.135781649	growth toward the midline
ENSDART00000124367	si:cabz01074946.1	0.002461121	1.457343255	1.182560392	0.713827232	0.020183118	growth toward the midline
ENSDART00000124663	npc2	0.000902446	1.578129482	0.684715762	0.389649636	0.031719264	growth toward the midline
ENSDART00000124833	pdcd11	0.01245587	0.337926893	0.21951318	0.267537583	0.067942864	growth toward the midline
ENSDART00000124868	lpl	0.000214183	0.342987533	-0.032282276	-0.168396519	-1.276175937	growth toward the midline
ENSDART00000125097	si:dkey-126g1.7	0.00111498	0.825737488	0.388125468	-0.092297047	-0.324389582	growth toward the midline
ENSDART00000125178	eif5a	0.003534272	0.322397945	0.394023524	0.161340886	-0.011483865	growth toward the midline
ENSDART00000125430	pprc1	8.24104E-05	1.538620876	1.109491459	0.874206496	0.220876028	growth toward the midline
ENSDART00000125753	rpl22	0.00115719	0.364344172	0.362576681	0.16140506	-0.121749389	growth toward the midline
ENSDART00000126229	rps7	0.004986552	0.396514179	0.373862291	0.136780142	-0.080022773	growth toward the midline
ENSDART00000126417	bri3	0.002005251	0.500014173	0.414528928	0.11265957	-0.17961569	growth toward the midline
ENSDART00000126780	rnaset2	0.000371877	0.561676509	0.243610345	-0.050562112	-0.093659569	growth toward the midline
ENSDART00000126978	acadl	0.002156175	0.315070362	0.266918423	-0.034640916	-0.111553388	growth toward the midline
ENSDART00000127277	pum3	0.018496255	0.522067564	0.375606403	0.43721492	0.160017235	growth toward the midline
ENSDART00000127350	enpp1	0.000451785	1.121109181	0.817391101	0.578924138	0.065420517	growth toward the midline
ENSDART00000127496	loxl1	0.000342667	0.412088952	0.133411325	-0.193765804	-0.987510306	growth toward the midline
ENSDART00000127753	adam15	0.005335064	0.405103987	0.059018909	-0.196195339	-0.053874543	growth toward the midline
ENSDART00000127982	suox	0.014022061	0.48894768	0.565232546	0.460591806	0.13079859	growth toward the midline
ENSDART00000128049	hunk	0.016504069	0.851411376	0.241049411	0.130293149	0.977016396	growth toward the midline
ENSDART00000128246	polr1c	0.013474932	0.52692078	0.422008081	0.272814737	0.11358433	growth toward the midline
ENSDART00000128488	e2f8	0.003382652	3.852661131	-0.929008264	3.252279997	2.01178299	growth toward the midline
ENSDART00000128550	palb2	0.009615324	0.912196863	1.171643148	0.786370898	0.077481484	growth toward the midline
ENSDART00000128613	rps15a	0.004826213	0.315521947	0.355667698	0.260468714	-0.049823228	growth toward the midline
ENSDART00000128743	tmem214	0.02086388	0.301583498	0.153317199	0.10691428	0.053655055	growth toward the midline
ENSDART00000128786	pyya	0.000403881	0.897714064	0.120108152	-0.15783225	-0.181132487	growth toward the midline
ENSDART00000128917	rps4x	0.011474173	0.379768803	0.358465257	0.299276991	0.035010431	growth toward the midline
ENSDART00000129155	vmp1	1.77791E-05	3.054036254	2.972129495	2.202758828	1.232955319	growth toward the midline
ENSDART00000129380	ppp5c	0.000402095	0.718884884	0.082309464	0.464958134	0.512508903	growth toward the midline
ENSDART00000129617	atp6ap1b	0.000657061	0.602272356	0.200096368	-0.075510461	-0.466117163	growth toward the midline
ENSDART00000129903	mfsd10	0.000409421	0.819604016	0.72451413	0.409438046	0.004032617	growth toward the midline
ENSDART00000129919	TIFA	0.001882493	0.580552682	0.759337474	0.369383589	-0.101109572	growth toward the midline
ENSDART00000129963	rbp1	0.001296944	1.30707657	0.844804596	-0.023243783	-0.809227723	growth toward the midline
ENSDART00000130131	sult6b1	0.013901532	0.679202914	0.436031956	0.32900324	-0.176606639	growth toward the midline
ENSDART00000130297	atg16l2	0.036864554	0.527142084	0.480414213	0.279348799	0.207971893	growth toward the midline
ENSDART00000130494	moxd1	0.001779714	0.536918295	0.137560293	-0.344113602	-0.82692019	growth toward the midline
ENSDART00000130601	CABZ01020840.1	0.002663982	1.211268037	0.754343565	0.351699193	-0.297623866	growth toward the midline
ENSDART00000130604	ssuh2.4	0.003590782	0.995807077	0.517001744	0.13232339	-0.007643053	growth toward the midline
ENSDART00000130648	scarb2c	0.030497275	0.530119641	0.364624401	0.255215167	-0.022903408	growth toward the midline
ENSDART00000130911	cmklr1	0.011725523	1.293061027	1.016453103	0.21085461	0.074156746	growth toward the midline

ENSDART00000131217	ippk	0.009000055	0.31853123	0.426685761	0.198265799	0.012388457	growth toward the midline
ENSDART00000131241	CD53	0.022148005	1.613639378	1.480348694	1.150180939	0.900907437	growth toward the midline
ENSDART00000131319	ctps1a	0.008283592	0.387589066	0.225454863	0.161786406	-0.016402726	growth toward the midline
ENSDART00000131400	CR792453.3	0.001585773	2.47650448	0.829837833	-0.069994818	-0.405416724	growth toward the midline
ENSDART00000131558	zgc:103438	0.001133915	0.587891191	0.925990315	0.240143957	-0.080675365	growth toward the midline
ENSDART00000131661	rps9	0.01002859	0.491211514	0.443239408	0.342502062	-0.078353052	growth toward the midline
ENSDART00000131694	selenbp1	0.000844564	0.440307718	0.23934875	0.219921774	-0.162883702	growth toward the midline
ENSDART00000131724	pld3	0.002957541	0.801498613	0.654598157	0.377873753	0.229417497	growth toward the midline
ENSDART00000131802	si:dkey-204f11.64	0.010173835	0.501807484	0.364460777	0.066146426	-0.103454568	growth toward the midline
ENSDART00000131966	AL929520.1	0.003243686	1.864305398	1.102319083	0.530092783	0.456970098	growth toward the midline
ENSDART00000132216	paox1	0.005824405	0.706860126	0.311306251	-0.007602467	-0.279227462	growth toward the midline
ENSDART00000132637	rab34a	0.004827977	0.251888735	0.316357959	0.030371007	-1.217256649	growth toward the midline
ENSDART00000132660	polr3gla	0.007082106	0.823813849	0.325249077	0.007723106	-0.29164484	growth toward the midline
ENSDART00000132742	apoc1	0.007655029	0.900470905	0.457797087	-0.066389631	0.150028097	growth toward the midline
ENSDART00000132830	stoml3b	0.039723869	2.594772138	1.896323147	1.645470476	1.013599137	growth toward the midline
ENSDART00000133109	rps28	0.000937145	0.456862734	0.459372965	0.307002895	0.056264372	growth toward the midline
ENSDART00000133167	si:dkey-98f17.5	0.012693562	0.605842267	0.54718235	0.319480313	0.041690788	growth toward the midline
ENSDART00000133304	npm1a	0.000878399	1.159038348	0.761096985	0.663684243	0.261874194	growth toward the midline
ENSDART00000133375	si:dkey-30c15.17	0.00181079	0.981163755	0.773615181	0.451221931	-0.42308326	growth toward the midline
ENSDART00000133404	rps24	0.000717268	0.584478639	0.636503255	0.16891132	-0.309462265	growth toward the midline
ENSDART00000133512	fosl1b	3.29362E-06	2.958627889	2.652355797	1.322528527	0.313643003	growth toward the midline
ENSDART00000133517	si:dkey-228b2.5	0.032645202	0.6930874	0.410660128	0.386710557	0.222079502	growth toward the midline
ENSDART00000133659	ncapg	0.042253127	0.759144886	0.927669912	0.449671959	0.278225274	growth toward the midline
ENSDART00000133934	rps9	0.033833708	0.75844303	0.709617891	0.420926612	0.393006212	growth toward the midline
ENSDART00000133946	si:dkey-147f3.8	0.003898333	1.477973714	1.681506394	1.170700784	-0.068341199	growth toward the midline
ENSDART00000134189	npm1a	0.015751923	1.523433306	1.012610127	1.305980875	0.439289484	growth toward the midline
ENSDART00000134206	hspb8	0.001438131	0.868767381	0.59923642	0.380053431	0.101288698	growth toward the midline
ENSDART00000134404	si:ch211-158d24.4	0.024003814	0.479480538	0.856462378	0.502566478	-0.11398241	growth toward the midline
ENSDART00000134819	slc5a6a	2.95145E-05	0.44313334	-0.037657655	-0.159985236	-0.577627715	growth toward the midline
ENSDART00000134941	prc1b	0.0242075	1.557351387	1.026365943	0.57100162	0.47167284	growth toward the midline
ENSDART00000135032	si:dkey-57a22.11	0.026714705	1.01281435	1.486077838	0.644934957	0.560278535	growth toward the midline
ENSDART00000135238	dok2	0.013959384	1.480525707	0.841647962	0.697333529	0.138703296	growth toward the midline
ENSDART00000135472	zgc:86764	0.036021217	1.170468948	0.97326651	0.792803713	0.492808604	growth toward the midline
ENSDART00000135517	cenpf	0.003777754	1.006357007	0.930294184	0.598168756	0.318387864	growth toward the midline
ENSDART00000135719	si:ch211-171h4.6	0.033435193	1.187005549	0.373648016	0.271137881	-0.125827965	growth toward the midline
ENSDART00000135888	rrp9	0.002433479	0.649352382	0.396806051	0.419459571	0.067410678	growth toward the midline
ENSDART00000136382	gadd45ba	0.000960205	1.037707985	1.186135766	0.645465362	0.508629	growth toward the midline
ENSDART00000136383	cd99	0.001367004	0.67248236	1.035021667	0.666974643	-0.164650894	growth toward the midline
ENSDART00000136543	tnni4b.1	0.007654107	1.504503498	0.982879899	0.857598266	-0.145526765	growth toward the midline
ENSDART00000136722	cdkn1a	0.000598084	1.083504495	0.97819224	0.258587523	0.060123909	growth toward the midline
ENSDART00000136779	entpd5b	0.002650976	0.819562392	0.580844217	0.174817773	-0.04896424	growth toward the midline
ENSDART00000136835	gpr84	0.003982134	0.70622544	0.183343809	-0.153052934	-0.427967202	growth toward the midline
ENSDART00000136865	cfp	0.000230274	0.329536618	0.225136408	-0.193120136	-0.891420593	growth toward the midline
ENSDART00000137019	cmklr1	7.26498E-05	1.293423342	0.826870113	0.435422926	0.179038361	growth toward the midline
ENSDART00000137373	si:ch211-266i6.3	0.000114847	1.973293451	1.253222026	0.772689981	0.242956342	growth toward the midline
ENSDART00000137858	alpk2	0.024455662	0.667374473	0.189664169	0.047615444	-0.048884335	growth toward the midline
ENSDART00000137973	grna	0.005201666	1.322360375	0.965948715	0.567189356	-0.062803655	growth toward the midline
ENSDART00000137984	SBNO2 (1 of many)	0.001847086	0.66540874	0.705510404	0.683617676	-0.079102756	growth toward the midline
ENSDART00000138527	rab20	0.009820514	0.632157851	0.410164386	0.278506263	0.009483031	growth toward the midline
ENSDART00000138747	urb2	0.019322869	0.51682098	0.426109616	0.379169845	0.148916896	growth toward the midline
ENSDART00000138996	si:ch211-214p13.9	0.003686476	1.396990228	1.323446174	0.865221946	-0.306555509	growth toward the midline
ENSDART00000139087	lgmn	2.59942E-05	0.782730519	0.338051823	0.153128345	-0.09953272	growth toward the midline
ENSDART00000139124	ckba	0.004373845	1.776728636	1.213212461	1.201789834	0.178980804	growth toward the midline
ENSDART00000139659	si:dkey-222f2.7	0.007791135	1.108120911	0.818052369	0.396214124	-0.100365974	growth toward the midline
ENSDART00000139839	rbmx	0.034811028	0.438924108	0.178102249	0.089667681	0.22023097	growth toward the midline
ENSDART00000140086	SUN2	0.001030626	0.64389802	0.258658486	-0.305248612	-0.209793481	growth toward the midline
ENSDART00000140523	mrc1b	0.000638552	0.693910087	0.60918831	0.270261416	-0.09789336	growth toward the midline
ENSDART00000140667	spna2	0.034218044	0.558956985	0.663728247	0.276626138	0.347556663	growth toward the midline
ENSDART00000140776	CR391940.1	0.013366808	0.57109507	0.234979352	0.004616304	-0.046528801	growth toward the midline
ENSDART00000140788	gbp1	0.014906425	3.129821614	2.838243862	2.757243294	2.490411238	growth toward the midline
ENSDART00000140874	seph	0.005866285	0.619876107	0.651122471	0.195858296	-0.082542729	growth toward the midline
ENSDART00000140920	prom1b	0.039913243	0.88825196	0.404698565	0.393910982	0.372363663	growth toward the midline
ENSDART00000140970	si:ch73-52p7.1	0.011521596	1.046367332	0.798731839	0.265928239	-0.108652338	growth toward the midline
ENSDART00000141142	mvp	0.001130894	1.187603651	1.369710798	0.919014152	0.045597494	growth toward the midline
ENSDART00000141188	st3gal3b	0.000470359	0.577420785	0.203550656	-0.28232291	-0.213031688	growth toward the midline
ENSDART00000141657	anxa4	0.003268346	0.870599992	0.703638401	0.563849009	-0.254549515	growth toward the midline
ENSDART00000141676	rpl12	0.0451272	0.410901536	0.227715625	0.271640237	0.155083239	growth toward the midline
ENSDART00000141714	nupr1	0.022150836	1.321058122	1.103287011	0.703763985	0.083130869	growth toward the midline
ENSDART00000142088	si:ch211-218c6.8	0.009300169	1.058750612	0.593589729	0.534639469	0.071525268	growth toward the midline
ENSDART00000142104	cep162	0.025259321	0.409390298	0.286359079	0.288581786	0.16808044	growth toward the midline
ENSDART00000142389	cracr2ab	0.002199978	1.038814867	1.254276576	0.507748466	-0.396339594	growth toward the midline
ENSDART00000142422	tgm2l	0.026925307	1.901086414	0.665689502	0.275506536	0.14002471	growth toward the midline
ENSDART00000142467	si:ch211-199g17.2	0.017327178	2.796012298	2.177853709	2.464842477	1.654064057	growth toward the midline
ENSDART00000143066	ube2e1	0.020455943	0.330371306	0.357877825	0.149635317	0.181174607	growth toward the midline
ENSDART00000143291	RPL41	0.002755913	0.451636798	0.464258672	0.193261311	0.034762109	growth toward the midline

ENSDART00000143587	ern2	0.02452124	0.504450294	0.214096186	0.104332211	0.050090305	growth toward the midline
ENSDART00000143618	birc7	0.03238504	0.867487648	0.469428911	0.548081783	0.007263478	growth toward the midline
ENSDART00000143766	tbc1d4	0.027561798	0.551251852	0.35059591	0.424872318	0.224396247	growth toward the midline
ENSDART00000143846	si:ch211-198c19.3	0.000489924	0.524345776	0.185185856	-0.297806816	-0.062260629	growth toward the midline
ENSDART00000143875	BX323074.2	0.003519185	0.32505808	-0.029313176	-0.099305279	-0.178101813	growth toward the midline
ENSDART00000143928	rwdd1	0.018316862	0.331616231	0.265135658	0.236169655	0.064492205	growth toward the midline
ENSDART00000144048	cecr1b	0.000723654	1.174703015	1.015529557	0.446419815	-0.23634795	growth toward the midline
ENSDART00000144139	elavl4	0.003742624	1.756586356	1.578077782	0.938796608	0.66956976	growth toward the midline
ENSDART00000144711	tmem154	0.034302368	0.48356725	0.317274745	0.352966647	0.032904309	growth toward the midline
ENSDART00000144886	dicp1.1	0.024485347	1.748045166	0.589930553	0.172712572	0.178536852	growth toward the midline
ENSDART00000144891	gpnmb	0.000996442	2.526942309	2.443053131	2.053909603	-0.779431544	growth toward the midline
ENSDART00000145111	parp4	0.00078525	1.113854381	0.990369473	0.690636927	0.186507426	growth toward the midline
ENSDART00000145198	steap4	0.002341904	0.551058744	0.178525002	-0.419104103	-0.970957834	growth toward the midline
ENSDART00000145275	si:dkey-147f3.8	0.000993187	1.443349889	1.631277345	1.233548809	-0.107779207	growth toward the midline
ENSDART00000145330	apoc1	0.011155618	1.228570608	0.722853476	0.218571174	0.363892889	growth toward the midline
ENSDART00000145331	zgc:92066	0.060501903	3.539715592	2.15686966	1.135379743	1.013519624	growth toward the midline
ENSDART00000145377	rpl38	0.001017556	0.331388676	0.460944412	0.086514771	-0.0487643	growth toward the midline
ENSDART00000145562	robo4	0.000595605	1.739515239	1.001047952	0.857159879	-0.094789579	growth toward the midline
ENSDART00000145681	atp6v1h	0.000322013	0.62737841	0.200274483	0.109012982	-0.483227692	growth toward the midline
ENSDART00000145933	si:rp71-68n21.9	0.016453138	0.834432396	0.464946482	0.375058394	-0.112544064	growth toward the midline
ENSDART00000145983	cdkn1a	0.003897339	1.243824395	0.764194636	0.498342294	0.064303597	growth toward the midline
ENSDART00000146101	mcoln1a	0.002433479	0.490011182	0.372261102	0.240356077	0.080050098	growth toward the midline
ENSDART00000146564	si:ch211-198c19.3	0.000658214	1.407263699	0.462763747	-0.033079511	-0.600140762	growth toward the midline
ENSDART00000147265	rpl8	0.005893002	1.326651746	0.800643986	0.962132043	0.631592879	growth toward the midline
ENSDART00000147275	plat	0.013807548	0.870075522	0.84761039	0.802236758	0.134643658	growth toward the midline
ENSDART00000147341	tubb4b	0.0213019	0.566193096	0.992023395	-0.136369873	0.130190834	growth toward the midline
ENSDART00000147343	dnase2	0.000313682	0.744692712	0.455061161	0.199303414	-0.152365099	growth toward the midline
ENSDART00000147516	si:ch73-343l4.8	0.002706926	0.629933021	0.124466643	-0.147547456	-2.286690979	growth toward the midline
ENSDART00000147527	si:dkey-9i23.14	0.001146813	2.675666068	2.038792867	2.055161538	1.544748993	growth toward the midline
ENSDART00000147617	bcl2l1	0.002976628	0.692333613	0.690280735	0.392732217	0.224132994	growth toward the midline
ENSDART00000148125	fam21c	0.012864775	0.420817307	0.137516364	0.034339244	-0.014273742	growth toward the midline
ENSDART00000148327	adamtsl7	0.000283184	2.218956465	1.395778664	0.846103004	0.026559812	growth toward the midline
ENSDART00000148398	MRAS (1 of many)	0.000781831	1.275583164	1.112018386	0.557077896	0.217843634	growth toward the midline
ENSDART00000148582	smpd1	0.002176351	0.404585024	0.130204031	0.022544887	-0.113079466	growth toward the midline
ENSDART00000148648	rps25	0.003347171	0.412929704	0.39629643	0.197445263	0.016589956	growth toward the midline
ENSDART00000148737	btk	0.046077182	1.027482823	0.714086499	0.58431037	0.237640489	growth toward the midline
ENSDART00000148766	dpf2	0.01541945	1.058462293	0.640887505	0.623810049	0.633616815	growth toward the midline
ENSDART00000148845	BX649485.4	0.007338257	2.24411597	1.39694109	1.224587369	-0.358910932	growth toward the midline
ENSDART00000149019	itpr1a	0.025939466	0.453945089	0.40663332	0.191730219	0.048560775	growth toward the midline
ENSDART00000149043	CR855300.1	0.019593044	0.458598352	0.519077258	0.374073731	0.109291075	growth toward the midline
ENSDART00000149169	ssr2	0.000562043	0.474946343	0.309006192	0.206925318	-0.029893306	growth toward the midline
ENSDART00000149431	sema4aa	0.000470359	1.058121853	0.676658919	0.239884836	-0.1730827	growth toward the midline
ENSDART00000149468	rps10	0.017275314	2.202930165	1.872426465	0.292067044	0.38759709	growth toward the midline
ENSDART00000149478	galns	0.01015025	1.09483472	0.726913129	0.227424057	0.052897193	growth toward the midline
ENSDART00000149486	def6b	0.012263789	4.188632994	4.544630212	4.117698226	0.985190768	growth toward the midline
ENSDART00000149600	tpp1	4.91848E-05	0.822885392	0.356076016	0.047488091	-0.289304028	growth toward the midline
ENSDART00000149711	slc4a2b	0.000605314	0.638486491	0.276788243	-0.283420926	-0.322786037	growth toward the midline
ENSDART00000149760	triobpa	0.028230031	0.630702727	0.141628128	0.231366625	0.068551496	growth toward the midline
ENSDART00000149882	si:ch211-117c9.1	0.076090072	1.22256875	0.270055012	0.422501128	0.414018825	growth toward the midline
ENSDART00000149951	BX666064.1	0.012467303	1.493495344	0.978793976	0.565382246	-0.392061768	growth toward the midline
ENSDART00000150088	BX649485.2	0.007435419	1.751466958	1.112873174	0.6867673	-0.722119651	growth toward the midline
ENSDART00000150184	gpatch4	0.006624621	0.624444079	0.471783034	0.550844363	0.152733798	growth toward the midline
ENSDART00000150201	MRAS (1 of many)	0.001127896	1.00558208	0.977546999	0.676765678	0.026394977	growth toward the midline
ENSDART00000150389	tmem168a	0.066196151	0.525344796	0.286469995	0.221730459	0.080011152	growth toward the midline
ENSDART00000150484	BX465862.4	0.000615224	1.071580051	0.886976814	0.477743025	0.134467836	growth toward the midline
ENSDART00000150919	si:ch211-191i18.2	0.004498556	1.045780222	1.362401557	0.586788909	0.260330898	growth toward the midline
ENSDART00000150934	alg6	0.010983086	0.502789869	0.534691837	0.441461289	0.13055149	growth toward the midline
ENSDART00000150940	si:ch211-193k19.2	0.024493238	1.599024227	0.310661889	0.753508784	0.054490277	growth toward the midline
ENSDART00000151390	si:ch211-195b13.1	0.006097198	0.447846827	0.275025282	0.031728831	0.543393224	growth toward the midline
ENSDART00000151393	zak	0.000392364	0.559981256	0.606971645	0.291742344	0.078029866	growth toward the midline
ENSDART00000151805	vamp4	0.008628374	0.722926228	0.32988105	0.08556674	0.068052265	growth toward the midline
ENSDART00000152122	si:ch211-176g6.2	0.006040855	1.234754318	1.008584835	0.458934208	-0.407921233	growth toward the midline
ENSDART00000152299	si:dkey-25o16.4	0.014178743	1.175283487	1.143366275	0.842863117	0.368204805	growth toward the midline
ENSDART00000152378	tgif1	0.003122803	0.194631245	0.419803811	0.14379228	-0.205607529	growth toward the midline
ENSDART00000152432	lratb.2	0.001938223	0.819163408	1.604350772	-0.35089707	-0.825333131	growth toward the midline
ENSDART00000152699	si:ch211-286b5.9	0.004853289	1.056007406	0.325301277	0.188178466	-0.341462888	growth toward the midline
ENSDART00000152766	CR354435.4	0.039457626	0.814174745	0.989990518	0.663039704	0.339445782	growth toward the midline
ENSDART00000152788	mki67	0.018439771	1.022403776	0.934711217	0.634468641	0.246495311	growth toward the midline
ENSDART00000152892	slc16a8	0.000894529	2.182950408	0.789162193	-0.768133939	-1.779969995	growth toward the midline
ENSDART00000153248	chadlb	0.026040756	1.217222776	0.847984599	0.639882396	-0.038384888	growth toward the midline
ENSDART00000153654	piezo2a.2	0.004108486	0.718362747	0.545453706	0.495090248	0.182038228	growth toward the midline
ENSDART00000153731	si:dkey-79d12.5	2.59942E-05	1.619937595	1.685958359	0.880018827	0.052586274	growth toward the midline
ENSDART00000153847	si:dkey-7i4.24	0.017660302	4.195698891	4.052966176	3.712547417	1.148430087	growth toward the midline
ENSDART00000153950	si:dkey-106n21.1	0.013634701	1.634202521	0.388901976	0.409369194	0.514423363	growth toward the midline
ENSDART00000153990	CR848791.2	0.022463379	3.062868799	2.217074852	2.048805323	1.916392428	growth toward the midline

ENSDART00000154066	isg20	0.006815804	0.340483696	0.28200181	0.116846361	-0.121466791	growth toward the midline
ENSDART00000154295	RPS17 (1 of many)	0.002786216	0.397556507	0.446477566	0.193480989	0.136840294	growth toward the midline
ENSDART00000154324	CR848791.3	0.00017041	3.13610848	2.257927159	1.961382093	1.531269093	growth toward the midline
ENSDART00000154460	FP243382.1	0.01179775	2.715231463	2.420271029	2.173535574	1.735635064	growth toward the midline
ENSDART00000154638	kif26ab	0.032809386	2.382609513	1.668373417	1.704066313	1.906262158	growth toward the midline
ENSDART00000154726	MYADM (1 of many)	0.004207729	1.393641215	1.121324137	1.000193747	0.38217935	growth toward the midline
ENSDART00000154801	itgae.2	0.002196508	0.922252153	0.655667188	0.379341087	-0.144710616	growth toward the midline
ENSDART00000154809	CU467861.1	0.013110232	0.653813027	0.40784225	0.192026805	0.151191997	growth toward the midline
ENSDART00000155268	pik3cb	0.012263789	0.672881324	0.55380807	0.246761254	0.040522215	growth toward the midline
ENSDART00000155273	FP102016.1	0.003003196	0.672121642	0.238521805	0.194526989	0.041260629	growth toward the midline
ENSDART00000155427	reep6	0.001233735	0.890805905	0.284631883	0.369468446	0.541597773	growth toward the midline
ENSDART00000155519	si:ch211-139d20.3	0.030271642	0.778333948	0.572606648	0.403486279	0.486914049	growth toward the midline
ENSDART00000155553	CR931780.1	0.003710792	1.845884645	1.39773807	0.761518659	0.588325484	growth toward the midline
ENSDART00000155755	CU469362.1	0.001684124	0.467928162	0.778645014	0.342312822	-0.038725012	growth toward the midline
ENSDART00000155844	nr1d4b	0.00277366	0.850133899	0.016728696	-0.171313869	0.165802614	growth toward the midline
ENSDART00000155864	CU929070.1	0.002360359	1.274116455	1.096698631	0.870265354	0.298589514	growth toward the midline
ENSDART00000156002	tmem206	0.007346626	0.793441462	0.286165316	0.167291274	-0.130249565	growth toward the midline
ENSDART00000156428	hephl1a	0.008866484	0.427057323	0.215282086	0.277986668	-0.033310258	growth toward the midline
ENSDART00000156499	prkcdb	0.006467002	0.617876805	0.366145269	0.057590279	-0.230105501	growth toward the midline
ENSDART00000156575	dicp3.3	0.011810205	1.874714519	0.882148407	0.706359774	-0.079481712	growth toward the midline
ENSDART00000157190	ostn	0.030844025	0.371505716	0.628806712	0.229636498	0.167623814	growth toward the midline
ENSDART00000157487	tfec	0.001790465	0.534318298	0.423012313	0.205933849	-0.313187686	growth toward the midline
ENSDART00000157636	Metazoa_SRP	4.91295E-05	2.626880551	2.386640401	1.529298522	0.72995056	growth toward the midline
ENSDART00000157899	dad1	0.009401721	0.443099375	0.390242441	0.192203328	-0.014224626	growth toward the midline
ENSDART00000158212	zgc:92161	0.004952764	0.66498591	0.968065208	0.600651955	-0.278084019	growth toward the midline
ENSDART00000158581	spna2	0.00601663	0.480311119	0.377153274	0.267336877	-0.085977531	growth toward the midline
ENSDART00000158733	si:dkey-33i11.1	0.004853289	1.813729642	1.289849367	0.876112899	0.279282769	growth toward the midline
ENSDART00000158808	cnn3a	6.00847E-05	0.586268263	0.320248819	-0.106889335	-0.483977637	growth toward the midline
ENSDART00000159246	ctsa	0.004928313	0.554249571	0.498653237	0.219459997	0.055975716	growth toward the midline
ENSDART00000159330	pcyox1	0.007509621	0.406833689	0.21789945	0.044242143	-0.158070725	growth toward the midline
ENSDART00000159433	BX571691.1	0.030614624	2.919338685	1.742268402	2.797274384	2.742997875	growth toward the midline
ENSDART00000159434	tmem254	0.019015879	0.529951039	0.26685766	0.074245289	-0.042910999	growth toward the midline
ENSDART00000159534	leng9	0.002069136	0.34368633	0.401471128	0.132926162	0.109702136	growth toward the midline
ENSDART00000159629	mcm2	0.044268959	0.995796878	0.693727511	0.639304212	0.157223466	growth toward the midline
ENSDART00000159716	si:ch73-236c18.6	0.022944365	0.962463693	0.259951761	0.066014607	0.162933451	growth toward the midline
ENSDART00000160000	spdl1	0.01662249	1.145158953	1.158182543	1.166185329	0.390123322	growth toward the midline
ENSDART00000160116	racgap1	0.01613952	0.647984241	0.386764067	0.354192772	0.100327502	growth toward the midline
ENSDART00000160201	tnfrsf11b	4.91848E-05	2.52634883	1.22900254	0.873090756	-0.039240801	growth toward the midline
ENSDART00000160245	si:ch73-236c18.7	0.03675533	2.124440879	1.318007353	1.287829039	0.437471879	growth toward the midline
ENSDART00000160297	vcana	0.04873911	1.601498468	0.665214133	0.454293537	0.486706931	growth toward the midline
ENSDART00000160331	atp6v0e1	0.000593122	0.536560194	0.109862681	-0.220128717	-0.336777163	growth toward the midline
ENSDART00000160503	slc15a4	0.005895019	0.534058917	0.262741482	0.05558204	-0.165911292	growth toward the midline
ENSDART00000160536	gnsb	6.00847E-05	1.380175579	0.91424754	0.488823056	0.00723105	growth toward the midline
ENSDART00000160582	creb5a	4.11188E-05	2.028867303	1.691967909	1.27021295	0.836790625	growth toward the midline
ENSDART00000160902	naga	0.00155515	0.641123507	0.233762511	0.291444137	-0.017668678	growth toward the midline
ENSDART00000161040	U1	0.063982053	0.811168663	0.450901632	0.418592021	0.580227266	growth toward the midline
ENSDART00000161063	heatr1	0.020490278	0.383217268	0.256645773	0.173900631	0.113179939	growth toward the midline
ENSDART00000161216	dync1i2a	0.016102005	1.005522301	0.520541958	0.468549497	-0.022454144	growth toward the midline
ENSDART00000161240	zgc:92161	0.014596701	0.584076871	0.663364233	0.486030672	0.10810748	growth toward the midline
ENSDART00000161322	slc29a1a	0.003016249	0.535023852	0.636573744	0.365586045	-0.115892928	growth toward the midline
ENSDART00000161421	zgc:194627	0.005106608	1.998498644	1.298544665	0.773438314	0.249109157	growth toward the midline
ENSDART00000161906	vwa11	0.001097466	1.529478256	0.755786632	0.896680083	0.546980022	growth toward the midline
ENSDART00000161967	ctsc	0.000657061	1.279775059	0.583635254	0.329059668	-0.157215155	growth toward the midline
ENSDART00000162097	CABZ01044157.1	0.011403904	1.031550352	0.670727071	0.212982548	-0.249292553	growth toward the midline
ENSDART00000162282	dicp3.1	0.000996442	0.839763954	0.379787637	0.004488715	-0.034973807	growth toward the midline
ENSDART00000162296	cfp	0.000230274	0.329536618	0.225136408	-0.193120136	-0.891420593	growth toward the midline
ENSDART00000162472	VWA5A (1 of many)	0.004190237	0.543940251	0.262980075	0.410699646	-0.136546959	growth toward the midline
ENSDART00000162482	zgc:152863	0.019421916	0.539805357	0.660295595	0.432850415	0.095615261	growth toward the midline
ENSDART00000162668	cremb	4.56695E-06	4.310209888	4.052771	3.333577791	2.240090056	growth toward the midline
ENSDART00000162799	crb3a	0.012813136	0.362169262	0.040586113	-0.086039525	-0.014272311	growth toward the midline
ENSDART00000162958	si:ch211-260e23.9	0.01863352	0.345171245	0.431307401	0.079575678	0.236719343	growth toward the midline
ENSDART00000163018	bet1l	0.049630983	0.723011674	0.444641169	0.411413339	0.530095318	growth toward the midline
ENSDART00000163391	si:ch211-149k12.3	0.022976426	0.507081525	0.32718269	0.316220639	0.200966933	growth toward the midline
ENSDART00000163398	pla2g15	0.007621679	0.617752366	0.443695085	0.159097428	0.08811481	growth toward the midline
ENSDART00000163407	slc20a1a	0.005041829	0.970510209	0.576996952	0.406775549	-0.419444699	growth toward the midline
ENSDART00000163668	si:ch1073-392o20.2	0.002227565	0.543875349	0.345011244	0.082952674	-0.090250505	growth toward the midline
ENSDART00000163724	SLA (1 of many)	0.041839159	1.207342807	0.416124608	0.383103847	0.193096581	growth toward the midline
ENSDART00000163794	wt1b	0.02934736	0.454982734	0.330694147	0.317167947	0.300420298	growth toward the midline
ENSDART00000164113	cpeb1a	0.0004696	1.336706075	1.190402311	1.006023313	0.300918459	growth toward the midline
ENSDART00000164161	osbpl1a	0.000360396	0.351337908	0.311134875	-0.016374257	-0.257205353	growth toward the midline
ENSDART00000164198	si:dkey-102m7.3	0.011741843	0.949739085	1.411170301	0.641678924	0.378323778	growth toward the midline
ENSDART00000164305	dicp1.1	0.001163813	0.970381914	0.35730766	0.127672979	0.007978514	growth toward the midline
ENSDART00000164440	si:ch211-195b11.3	0.017379805	0.887240489	0.373309939	0.100891486	-0.191092546	growth toward the midline
ENSDART00000164484	dtnba	0.000293873	0.785988532	0.671033087	0.445356623	0.140878127	growth toward the midline
ENSDART00000165186	si:dkey-33i11.9	0.01609527	1.335261857	1.027980768	0.465372707	0.114592431	growth toward the midline

ENSDART00000165491	BX294383.1	0.040285619	0.352047768	0.065820831	0.027639622	0.019046581	growth toward the midline
ENSDART00000165499	rps17	0.001024575	0.504096498	0.637791027	0.385574489	0.024446186	growth toward the midline
ENSDART00000165735	mdm4	0.009204947	0.677008086	0.457427602	0.365768719	0.56900659	growth toward the midline
ENSDART00000165932	sik1	0.023071688	1.156253383	0.676084919	0.687880039	1.28219737	growth toward the midline
ENSDART00000165938	CU466240.1	0.062849789	0.428025361	0.165679287	0.139754855	0.298251046	growth toward the midline
ENSDART00000166152	DAB2 (1 of many)	0.00878097	0.967097608	0.419929333	0.22593558	-0.403858057	growth toward the midline
ENSDART00000166209	wu:fb44b02	0.001191783	0.715207055	0.457109376	0.301906263	-0.147536436	growth toward the midline
ENSDART00000166388	il6st	0.008110834	0.326944598	0.248050944	0.233084281	-0.066517896	growth toward the midline
ENSDART00000166395	fcer1gl	0.000698893	0.672549379	0.370716598	-0.127334281	-0.409142814	growth toward the midline
ENSDART00000166496	cat	0.008672693	0.249947463	0.551180864	0.073389354	-0.18327225	growth toward the midline
ENSDART00000166561	ttc38	0.002390041	0.445202505	0.288306634	-0.06639877	-0.156646921	growth toward the midline
ENSDART00000166591	utp20	0.017280106	0.378085419	0.246513796	0.276819854	0.15697444	growth toward the midline
ENSDART00000166632	si:zfos-741a10.3	0.016381744	1.2129476	0.572994738	0.242126634	0.029899239	growth toward the midline
ENSDART00000166690	gadd45ga	0.000475692	1.824520476	2.195526461	0.938941318	1.401631316	growth toward the midline
ENSDART00000166725	si:dkey-27j5.9	0.024411518	1.657914875	0.40212862	0.542892045	0.141569929	growth toward the midline
ENSDART00000166755	tril	0.013035616	1.481556618	0.917097925	0.535942919	0.363878102	growth toward the midline
ENSDART00000167279	klhl6	0.010535127	0.921934193	0.563560813	0.364277897	0.100202901	growth toward the midline
ENSDART00000167468	prpsap1	0.0101684	0.532862733	0.121505744	0.13944499	-0.034080691	growth toward the midline
ENSDART00000167748	fbn2b	0.005787591	1.02333483	0.65734038	0.922883374	0.19244827	growth toward the midline
ENSDART00000167786	NA	0.032175544	0.801350724	0.147008366	0.081187044	0.322292312	growth toward the midline
ENSDART00000167937	p4hb	0.000670207	0.35105827	0.247669444	-0.015299318	-0.184059227	growth toward the midline
ENSDART00000167971	rps18	0.008672693	0.367747716	0.388045404	0.209564172	-0.058761163	growth toward the midline
ENSDART00000167986	hadhb	0.00721984	0.310832473	0.238522601	0.004510639	-0.025410475	growth toward the midline
ENSDART00000168002	laptm5	0.00340435	1.090713638	0.609708871	0.327355694	-0.215403696	growth toward the midline
ENSDART00000168472	creb5a	0.024581131	2.212193939	1.443174599	0.809493268	0.247644913	growth toward the midline
ENSDART00000168608	rnaset2	0.002456687	1.085971834	0.685727013	0.397141355	0.382009137	growth toward the midline
ENSDART00000168727	zgc:64065	0.021156666	0.439077495	0.173893761	0.133684815	0.136149811	growth toward the midline
ENSDART00000168831	si:ch73-158p21.3	0.004606351	1.161771941	0.892729958	0.502592433	0.147950065	growth toward the midline
ENSDART00000168840	rpl35a	0.004477335	0.357031004	0.320334573	0.163051637	-0.036511823	growth toward the midline
ENSDART00000169006	ak2	0.034654408	0.70404039	0.489848323	0.563968118	0.137147044	growth toward the midline
ENSDART00000169202	si:ch211-153b23.5	0.004687334	4.343063064	5.139014825	3.462179286	1.548865865	growth toward the midline
ENSDART00000169209	ptrh1	0.000931909	0.68284848	0.772674127	0.446966304	0.22472723	growth toward the midline
ENSDART00000169600	abhd4	0.004142854	0.160082589	0.423680439	-0.10396044	-0.08495852	growth toward the midline
ENSDART00000169633	col4a3bpb	0.002735847	0.489929413	0.127889926	-0.192097119	-0.259910666	growth toward the midline
ENSDART00000169789	grap2b	0.054938882	0.941263606	0.635408975	0.416956975	0.146969065	growth toward the midline
ENSDART00000169834	CABZ01052431.1	0.000470359	0.752252615	0.635370532	0.523133762	0.005708707	growth toward the midline
ENSDART00000169976	CABZ01034529.1	0.002936628	0.902549952	0.806876908	0.306228824	0.021333022	growth toward the midline
ENSDART00000169993	stx12	0.009181211	0.289245416	0.334302649	0.162033659	0.014864416	growth toward the midline
ENSDART00000170060	mdh1b	0.024987601	3.761355509	3.246314783	3.336555843	1.787365183	growth toward the midline
ENSDART00000170304	CABZ01039096.1	0.03168371	1.863106121	1.329668381	1.247074235	0.639142974	growth toward the midline
ENSDART00000170326	gadd45ba	0.013456921	1.741159479	1.754335562	1.06756424	0.870946071	growth toward the midline
ENSDART00000170601	si:ch1073-228h2.2	0.032809386	1.512427811	1.187632525	1.253650535	1.028624188	growth toward the midline
ENSDART00000170755	slc30a8	0.000398071	0.607491231	0.345740737	-0.026161917	-0.397029232	growth toward the midline
ENSDART00000170758	tcirg1b	0.007786985	0.779415513	0.215400071	0.036968665	-0.339490039	growth toward the midline
ENSDART00000170810	si:dkey-10p5.7	0.01179775	1.115159817	1.313044185	0.773496009	0.334257673	growth toward the midline
ENSDART00000171090	sowahd	0.01612441	0.56962557	0.415649109	0.184768671	-0.065367372	growth toward the midline
ENSDART00000171179	si:ch211-241b2.5	0.005678515	1.438237825	1.596241839	1.013176313	0.055616895	growth toward the midline
ENSDART00000171270	ckap2l	0.013513343	0.621565842	0.655006082	0.304610509	0.072068559	growth toward the midline
ENSDART00000171345	NA	0.060649685	2.015201895	1.694676698	1.017867776	0.711389392	growth toward the midline
ENSDART00000171430	BX908401.1	0.055863259	0.558597997	0.282396796	0.308082083	0.228227036	growth toward the midline
ENSDART00000171789	NA	0.00889295	1.544321358	1.350890859	1.388203792	0.786947546	growth toward the midline
ENSDART00000172069	snrpg	0.014753252	0.452166641	0.481061823	0.338564477	0.058821342	growth toward the midline
ENSDART00000172135	sh3pxd2b	0.005584581	0.68385955	0.832942161	0.517904326	0.090715158	growth toward the midline
ENSDART00000172201	trpv1	0.0044585	0.412354182	0.082280105	0.061779394	-0.254715035	growth toward the midline
ENSDART00000172207	si:ch211-9d9.1	0.000466856	1.222547885	0.297262625	0.271454205	-2.80261928	growth toward the midline
ENSDART00000172408	arhgap11a	0.030198325	0.695056063	0.093403628	0.066933123	0.263951638	growth toward the midline
ENSDART00000172555	rps15a	0.004379828	0.386196694	0.41469411	0.231657808	-0.026260131	growth toward the midline
ENSDART00000172892	CR769769.2	0.02363886	0.615702823	0.344273677	0.373739938	0.336791427	growth toward the midline
ENSDART00000172893	si:ch211-207g17.2	0.01138794	1.081136407	0.564546524	-0.069203481	-0.244746802	growth toward the midline
ENSDART00000172953	rpl29	0.008586878	0.354363113	0.457581567	0.206775836	0.026974968	growth toward the midline
ENSDART00000173145	rpl29	0.006013085	0.32197024	0.280412475	0.068293144	-0.107729465	growth toward the midline
TRANSGENE	NA	7.26665E-10	3.62113341	4.519443337	2.76591932	1.041497447	growth toward the midline
ENSDART00000002186	uck2a	0.001969989	0.69693934	0.733529046	0.658757388	0.439621207	midline crossing
ENSDART00000002250	hs6st2	0.009748008	0.156001692	0.361544481	0.514379395	0.208727247	midline crossing
ENSDART00000002641	kif26aa	0.003651057	0.255726279	0.47696992	0.58781469	0.342072456	midline crossing
ENSDART00000002932	marcksb	0.000213603	1.016829958	1.677411962	1.758104803	1.229873931	midline crossing
ENSDART00000003066	cyth1a	0.000219336	0.591135613	0.827117646	0.654961234	0.137500638	midline crossing
ENSDART00000003076	usp28	0.011417724	1.676441425	4.659547988	4.384020448	3.839785573	midline crossing
ENSDART00000003296	sars	0.039604681	0.182171112	0.390636242	0.505424837	0.283972917	midline crossing
ENSDART00000003752	cct3	0.006479841	0.348496547	0.407130663	0.410390239	0.168454536	midline crossing
ENSDART00000003790	pwp1	0.01633867	0.32756693	0.411455433	0.339342319	0.085530698	midline crossing
ENSDART00000003891	jupa	0.020463519	0.359241944	1.297843964	1.552619484	0.724987275	midline crossing
ENSDART00000004423	iars	0.002440466	0.363891152	0.394414274	0.599518689	0.155539422	midline crossing
ENSDART00000004474	mapre1b	0.001177059	0.531914947	0.849936108	0.970422656	0.515027849	midline crossing
ENSDART00000005119	eif3i	0.018217202	0.208433038	0.349717288	0.244101269	0.053458955	midline crossing

ENSDART00000005593	casp3a	0.000114847	0.498469533	1.198942327	1.332280554	0.912417093	midline crossing
ENSDART00000005616	rnpep	0.025134747	0.523525834	0.750055071	0.77634383	0.462027268	midline crossing
ENSDART00000005847	nav3	6.75287E-05	0.773382381	1.009593057	0.901548272	0.783565613	midline crossing
ENSDART00000005944	rpl5a	0.001179374	0.381988038	0.550810639	0.338076702	0.013320357	midline crossing
ENSDART00000006058	eif2s1a	0.03277385	0.414016542	0.357076684	0.28859634	0.112415797	midline crossing
ENSDART00000006908	itgb3b	2.33589E-05	1.52546911	1.915133846	1.613887964	0.668451164	midline crossing
ENSDART00000007231	psmb1	0.012813136	0.336843399	0.41477024	0.441532829	0.103495117	midline crossing
ENSDART00000007789	idh1	0.001120005	0.209391072	0.491026617	0.59658334	0.190896441	midline crossing
ENSDART00000007857	mettl2a	0.019840997	0.337109376	0.448868897	0.378403392	0.127678255	midline crossing
ENSDART00000009477	cct8	0.008761738	0.2739469	0.4225965	0.299361505	0.113502813	midline crossing
ENSDART00000009549	rhag	0.050085111	0.97898574	1.27206332	1.678759018	0.617102069	midline crossing
ENSDART00000009609	eif5a	0.00304733	0.382861639	0.513300938	0.466153608	0.171038659	midline crossing
ENSDART00000010256	eif3m	0.008283592	0.301540845	0.415293475	0.295465222	0.073592881	midline crossing
ENSDART00000010271	aida	0.002673241	0.207262438	0.531449642	0.462716258	0.148174666	midline crossing
ENSDART00000010647	rcc2	0.040200236	0.312248055	0.54892666	0.398800486	0.1537421	midline crossing
ENSDART00000010683	impa1	0.024809879	0.486043053	0.448191747	0.382482237	0.291162031	midline crossing
ENSDART00000010997	tpm3	0.039972057	0.446911874	0.508209148	0.705423057	0.171806011	midline crossing
ENSDART00000011258	npl	0.042570446	0.373272645	0.54061543	0.468726439	0.170404917	midline crossing
ENSDART00000011447	sae1	0.006887747	0.282597281	0.674186287	0.734155697	0.424524708	midline crossing
ENSDART00000011691	baxa	0.00197074	0.500319714	0.752407609	0.690306826	0.30333895	midline crossing
ENSDART00000011699	nono	0.019981901	0.031722902	0.329157035	0.240624596	0.057167441	midline crossing
ENSDART00000011878	eif4a1b	0.023987365	0.134279506	0.344064551	0.309281202	0.284267369	midline crossing
ENSDART00000012164	tmod2	0.019466285	0.222306993	0.411676025	0.560901639	0.297325519	midline crossing
ENSDART00000012686	dnase1l4.1	0.01793082	0.654985436	1.279054377	1.240763186	0.531962605	midline crossing
ENSDART00000012791	sp8a	0.049626195	1.57251014	2.331511764	2.00847442	1.039704847	midline crossing
ENSDART00000014049	wdr36	0.014675171	0.429321608	0.423447996	0.351992847	0.181701695	midline crossing
ENSDART00000014058	zgc:100829	0.002661298	0.836226257	1.366715788	1.414785917	0.344237354	midline crossing
ENSDART00000014983	zgc:153867	0.00612514	0.36977967	0.600215172	0.463408621	0.079627153	midline crossing
ENSDART00000015103	hps3	0.030383057	0.398719216	0.34180849	0.31802597	0.20234989	midline crossing
ENSDART00000015193	chmp4bb	0.001754036	0.349511966	0.500219693	0.425249872	0.076172611	midline crossing
ENSDART00000015374	cyb5r1	0.020123282	0.481284363	0.621601719	0.549959374	0.29311095	midline crossing
ENSDART00000015628	klhl24b	0.021895107	0.257620397	0.582600724	0.358856753	0.009507758	midline crossing
ENSDART00000016112	capns1b	0.000336326	0.258538261	0.719472725	0.61845775	0.248329876	midline crossing
ENSDART00000016135	nfe2l3	0.02142158	0.030439041	0.342226134	0.430712572	0.086440986	midline crossing
ENSDART00000016464	dcps	0.017642395	0.045608002	0.377363095	-0.076988765	0.017970252	midline crossing
ENSDART00000016591	fgf6a	0.000507398	0.502410209	1.375431955	1.297903392	1.026892284	midline crossing
ENSDART00000016628	fam129bb	0.003338328	0.861865647	1.020541987	0.89470832	0.182764632	midline crossing
ENSDART00000016946	glud1a	0.066889451	0.489439701	0.519599049	0.659106358	0.391317016	midline crossing
ENSDART00000017230	snrpc	0.012958044	0.173923367	0.335809723	0.464722714	0.188235196	midline crossing
ENSDART00000017422	tbc1d17	0.000844564	0.129347814	0.49736446	0.343125168	-0.01149421	midline crossing
ENSDART00000018408	anxa13l	5.81566E-05	0.767695981	1.876785961	1.969202275	1.362090979	midline crossing
ENSDART00000018685	syt9a	0.018500869	0.383050894	0.997731574	0.678563906	0.37205351	midline crossing
ENSDART00000018743	phf20a	0.027269932	0.312029532	0.419745206	0.325238046	0.318832686	midline crossing
ENSDART00000019521	dip2ba	0.005277161	0.352814519	0.501689217	0.539560646	0.272762208	midline crossing
ENSDART00000019698	anxa5b	0.006899426	1.162500386	1.67744918	1.46667303	0.432101083	midline crossing
ENSDART00000019750	wdr5	0.067593058	0.151689533	0.300718903	0.396766724	0.199844693	midline crossing
ENSDART00000019818	ric8b	0.011108721	0.219425238	0.304273846	0.411142498	0.146708414	midline crossing
ENSDART00000020048	gsna	0.018144005	0.451913636	0.79824491	0.782008443	0.357949724	midline crossing
ENSDART00000020084	hsp90ab1	0.015156186	0.266636397	0.434039186	0.537534154	0.384889667	midline crossing
ENSDART00000020256	lgsn	0.036969899	1.361702081	2.686172063	1.394569806	0.349584846	midline crossing
ENSDART00000020638	rcan1a	0.04859805	0.548382891	0.378475469	0.383543152	0.544764217	midline crossing
ENSDART00000021666	rtca	0.004731414	0.450183742	0.82121323	0.721903733	0.20677388	midline crossing
ENSDART00000022060	atf3	2.2034E-05	2.958540036	3.348918067	2.852908333	1.811501419	midline crossing
ENSDART00000023123	nup88	0.017159406	0.221684741	0.406336851	0.406160624	0.250704697	midline crossing
ENSDART00000023156	eef1a1l2	0.002558097	0.512554224	0.741741761	0.709552788	0.28897731	midline crossing
ENSDART00000023709	ptp4a2b	0.023468821	0.271575512	0.562448581	0.634906429	0.086969331	midline crossing
ENSDART00000023779	vcp	0.053123832	0.230561184	0.246570266	0.325389872	0.137878127	midline crossing
ENSDART00000023833	eif2s3	0.013547844	0.172990624	0.349255612	0.311895175	0.045890851	midline crossing
ENSDART00000024309	rb1	0.001317626	0.736743325	0.968558289	0.720111001	0.238176467	midline crossing
ENSDART00000024320	ybx1	0.075082889	0.257630768	0.252218726	0.414084055	0.18414263	midline crossing
ENSDART00000024662	plppr3a	0.000164013	1.050789931	1.738581828	1.609411361	1.082826483	midline crossing
ENSDART00000024872	creb3l3l	0.003959852	0.316205692	0.628018553	0.525420097	0.254183225	midline crossing
ENSDART00000025385	cers2a	0.00721984	0.59368354	0.657003513	0.583941604	0.192392368	midline crossing
ENSDART00000025496	rras	0.010693822	0.483455604	0.817924325	0.502791397	-0.193021858	midline crossing
ENSDART00000025535	sept5a	0.004815134	0.521581645	1.371610673	0.945407012	0.566204111	midline crossing
ENSDART00000025550	top1mt	0.003833624	0.816108961	1.133963099	0.883225094	0.407533529	midline crossing
ENSDART00000025620	ppiaa	0.002705637	0.369681961	0.563046067	0.555154111	0.174901331	midline crossing
ENSDART00000025782	nup93	0.062586781	0.17632244	0.241668195	0.293793434	0.152368608	midline crossing
ENSDART00000026180	fabp7a	0.006795032	0.745585804	2.003621538	1.857780253	1.020257385	midline crossing
ENSDART00000026339	gtpbp4	0.048096163	0.113641949	0.327640711	0.222548232	0.071359586	midline crossing
ENSDART00000026409	cct4	0.00602897	0.459151422	0.538338734	0.621039126	0.313903974	midline crossing
ENSDART00000026814	ptp4a1	0.004605292	0.419787699	0.591512581	0.636748721	0.300535035	midline crossing
ENSDART00000027616	eif4g2a	0.000406473	0.333990776	0.435258045	0.644700731	0.350291836	midline crossing
ENSDART00000028090	eif2ak1	0.011517304	0.218229206	0.400243646	0.398265404	0.179590234	midline crossing
ENSDART00000029843	vezf1a	0.001561003	0.660507751	0.776194217	0.884683465	0.428803142	midline crossing

ENSDART00000030205	bnip3lb	0.010960196	0.087436849	0.295010863	0.335325724	0.120618456	midline crossing
ENSDART00000030691	clic4	0.028282855	0.290171682	0.490608957	0.279069846	0.046035564	midline crossing
ENSDART00000032322	abcg2c	0.004813371	0.455571157	0.699504596	0.641510999	0.335341634	midline crossing
ENSDART00000032324	hddc3	0.020485853	0.491004501	0.803369277	0.623276564	0.474326204	midline crossing
ENSDART00000032331	gmppab	0.008531138	0.298836739	0.452114759	0.362408461	-0.028707454	midline crossing
ENSDART00000032540	usp14	0.04750637	0.236991779	0.380670741	0.291281165	0.061189525	midline crossing
ENSDART00000032603	tspo	0.008848885	0.47255795	0.736846252	0.52533392	0.11686825	midline crossing
ENSDART00000032857	mapk11	0.047290605	0.353170314	0.603154564	0.6388701	0.458511735	midline crossing
ENSDART00000033316	vangl2	0.00277587	0.207091244	0.447705982	0.579933447	0.26125294	midline crossing
ENSDART00000033479	si:ch211-129c21.1	0.00140081	1.539581327	2.000726167	1.978342783	1.084446492	midline crossing
ENSDART00000033494	klf6a	0.000170861	1.084550888	1.797692613	1.625562666	0.460444617	midline crossing
ENSDART00000033566	smad1	0.004676707	0.486585209	1.249927606	1.264727704	0.536891999	midline crossing
ENSDART00000034523	tars	0.044608572	0.184292328	0.408838041	0.43347383	0.219690833	midline crossing
ENSDART00000034784	adcyap1b	0.000144061	1.680397515	2.015053329	1.852069794	1.09666998	midline crossing
ENSDART00000034935	desi2	0.009532057	0.073850834	0.377607475	0.427284588	0.193399467	midline crossing
ENSDART00000035899	pkp2	0.002199978	0.428534184	1.016588803	1.016153499	0.312913104	midline crossing
ENSDART00000036513	trib3	0.00028941	0.704680301	0.933969628	0.780405513	0.230790744	midline crossing
ENSDART00000036668	psmc1a	0.017576487	0.311771761	0.423109618	0.307731242	0.107467578	midline crossing
ENSDART00000036703	pfdn2	0.011267245	0.369409429	0.460943577	0.431067405	0.197162016	midline crossing
ENSDART00000036797	uchl1	0.000254175	1.004812771	1.916965558	1.938952257	1.446591845	midline crossing
ENSDART00000036939	gadd45ba	2.18017E-05	1.15110753	1.253886794	1.06146692	0.82843487	midline crossing
ENSDART00000037195	kif26bb	6.9873E-05	1.206892082	1.55094827	1.874551064	1.519778039	midline crossing
ENSDART00000037698	uck2b	6.99374E-05	0.641974401	1.119646558	1.077673009	0.598617402	midline crossing
ENSDART00000037709	nol11	0.023203992	0.254424392	0.434300513	0.299474854	0.102573384	midline crossing
ENSDART00000037846	focad	0.014575529	0.435964232	0.512370823	0.456134064	0.112317494	midline crossing
ENSDART00000037850	dync1li2	0.002061124	0.268558729	0.496891146	0.585345447	0.309833954	midline crossing
ENSDART00000039443	tuba8l4	0.002876227	0.45016266	0.547239574	0.701695743	0.194541839	midline crossing
ENSDART00000039746	epb41b	0.061238247	0.556721113	0.962382373	1.378686301	0.45048608	midline crossing
ENSDART00000040066	adam9	0.002930263	0.358589887	0.583904956	0.549640677	0.256288209	midline crossing
ENSDART00000040804	praf2	0.017079861	0.166225557	0.427661756	0.402173801	0.274020381	midline crossing
ENSDART00000041191	gyg2	0.019039254	0.909324979	1.617533024	1.086480874	0.519765295	midline crossing
ENSDART00000041279	tubb4b	0.017496248	0.433747505	0.468816243	0.626770783	0.445102188	midline crossing
ENSDART00000041468	ap1ar	0.000902118	0.253231094	0.535778421	0.657902256	0.098067269	midline crossing
ENSDART00000041877	csrnp1a	0.000166716	1.299872398	1.369159722	1.350463811	0.717287101	midline crossing
ENSDART00000042189	pdk2b	0.007668959	0.734399082	1.569570791	1.007918384	0.211070696	midline crossing
ENSDART00000042297	kdelc1	0.002843439	0.328425656	0.736886183	0.437422887	0.039597286	midline crossing
ENSDART00000042963	chst11	0.007952083	0.296547318	0.476599287	0.638913095	0.263644905	midline crossing
ENSDART00000044154	tnnt2c	0.012339321	0.256126919	0.781011759	0.616305237	0.377618007	midline crossing
ENSDART00000045299	ola1	0.002876227	0.514191862	0.666266592	0.533008533	0.271611648	midline crossing
ENSDART00000045479	syt4	0.022209173	0.199773705	0.561264928	0.42766733	-0.021116223	midline crossing
ENSDART00000045682	rrp36	0.024581131	0.38981283	0.603671455	0.338055221	0.296271067	midline crossing
ENSDART00000046115	mfsd2aa	0.004106312	0.413173515	1.356876257	1.539393726	0.598141045	midline crossing
ENSDART00000046209	acbd7	0.004793697	0.172970527	0.805855312	0.630012733	0.123911744	midline crossing
ENSDART00000046587	ap2m1a	0.018091301	0.393397352	0.521859633	0.500225377	0.048547949	midline crossing
ENSDART00000046973	capza1a	0.016550715	0.117659515	0.282132124	0.303069026	0.119362421	midline crossing
ENSDART00000047020	casp9	0.000847723	0.406413734	0.845063479	0.618979742	0.291728295	midline crossing
ENSDART00000047073	oxsr1a	0.02511033	0.179223329	0.268680956	0.376160209	0.212719894	midline crossing
ENSDART00000048383	creld2	0.024987601	0.499343204	0.520824125	0.482480278	0.212076819	midline crossing
ENSDART00000048977	abcf1	0.002096961	0.295994115	0.413143633	0.435605861	0.158915281	midline crossing
ENSDART00000049264	sdr16c5b	0.002217387	0.496225849	0.925512272	0.840394736	0.492626723	midline crossing
ENSDART00000049373	cmtr1	0.067400884	3.529734054	4.85424569	5.483410402	3.408663479	midline crossing
ENSDART00000049434	scamp4	0.020544535	0.767657025	0.801831806	0.544244726	0.399234701	midline crossing
ENSDART00000049464	fermt2	0.02086388	0.28468341	0.302053037	0.328009634	0.040193822	midline crossing
ENSDART00000051621	pgam5	0.017997171	0.243607349	0.33552261	0.455304621	0.149179919	midline crossing
ENSDART00000052168	hrh3	0.001356985	0.375251408	0.91270689	1.213693931	0.380712944	midline crossing
ENSDART00000052421	txnipa	0.000860824	1.054359781	1.031010785	1.048664605	0.430054853	midline crossing
ENSDART00000052539	myo1ea	0.012462623	0.574953247	0.619444947	0.654028338	0.173056841	midline crossing
ENSDART00000052656	rras2	0.005262446	0.556656504	0.801505793	0.534247735	0.118931735	midline crossing
ENSDART00000052915	ash1l	0.056562408	0.173309311	0.167145004	0.317504355	0.12008578	midline crossing
ENSDART00000053240	cab39l	0.013803709	0.313790093	0.462769475	0.441611468	0.290026864	midline crossing
ENSDART00000053304	si:ch211-114n24.6	0.000776925	0.627425485	0.596987541	0.770158215	0.21603311	midline crossing
ENSDART00000053325	tomm40l	0.008715601	0.351677862	0.400415779	0.406067875	0.134025152	midline crossing
ENSDART00000053925	mtmr7a	0.018204569	0.252266702	0.492232945	0.532543286	0.441567176	midline crossing
ENSDART00000054062	nek12	0.002113455	0.979847079	2.121821413	1.72566276	1.104924836	midline crossing
ENSDART00000054243	dpf2l	0.013354221	0.196658591	0.403359284	0.517168503	0.26603871	midline crossing
ENSDART00000054849	pls3	0.01571154	0.365005671	0.852391802	1.00799598	0.660436059	midline crossing
ENSDART00000054987	actb1	0.002610721	0.719668079	0.845173501	0.884967639	0.398548729	midline crossing
ENSDART00000055171	grapa	0.005866285	0.991444674	1.026501701	0.907734856	0.689609359	midline crossing
ENSDART00000055253	filip1l	0.03479782	3.442073683	3.796340861	4.199635374	3.015929109	midline crossing
ENSDART00000055325	psmb7	0.009948051	3.620821341	3.668842367	3.694371073	3.321198364	midline crossing
ENSDART00000055609	atf4b	0.027440234	0.138344185	0.51758353	0.353617358	0.087935611	midline crossing
ENSDART00000056254	stap2a	0.001692745	0.934389037	1.208596726	1.287672321	0.743017194	midline crossing
ENSDART00000056294	pitrm1	0.005065079	0.368247543	0.389541704	0.328836098	0.178268037	midline crossing
ENSDART00000056712	etfdh	0.011891263	0.23563688	0.425716128	0.377386118	0.136575345	midline crossing
ENSDART00000056865	ctnnbip1	0.001123946	0.431885199	0.971175617	1.010655462	0.599546466	midline crossing

ENSDART00000056987	marcksl1a	0.024073872	0.199644536	0.480185443	0.561780108	0.295794028	midline crossing
ENSDART00000057159	cacnb1	0.000793313	1.304600132	2.011299334	2.307200837	1.559835487	midline crossing
ENSDART00000057318	dusp8b	0.001038693	0.80659762	0.962100249	0.680537568	0.386784732	midline crossing
ENSDART00000058258	gng5	0.005032085	0.433032497	0.476321934	0.436326472	0.078774872	midline crossing
ENSDART00000058485	rai14	0.003945154	0.377402754	0.592516996	0.371359301	0.056635681	midline crossing
ENSDART00000059955	ildr1b	0.057189105	0.550873694	0.893755801	0.988897575	0.559320969	midline crossing
ENSDART00000060181	zgc:114174	0.000530705	0.760260394	1.239485919	1.043412191	0.428827471	midline crossing
ENSDART00000060255	blmh	0.003785955	0.318055181	0.435968503	0.441694486	0.152219233	midline crossing
ENSDART00000060812	adcyap1b	0.000184957	1.704872196	2.272116909	1.908485885	0.966573225	midline crossing
ENSDART00000060910	pimr138	0.000775407	0.646921071	1.324373168	1.239639246	0.98486272	midline crossing
ENSDART00000060919	qars	0.019842178	0.348349072	0.542908344	0.574420452	0.306598142	midline crossing
ENSDART00000061196	NA	0.000874891	0.755433358	1.498415514	1.365853078	0.846252469	midline crossing
ENSDART00000062704	plaa	0.003777754	0.222653649	0.333324552	0.491597491	0.251933193	midline crossing
ENSDART00000063359	ucp3	0.038034192	0.449516929	0.816201403	1.070983817	0.155240874	midline crossing
ENSDART00000063418	nsun5	0.015044537	0.551107756	0.480901258	0.442024494	0.196344317	midline crossing
ENSDART00000063912	jun	0.002341904	1.166146477	1.429328618	1.29995447	0.480560375	midline crossing
ENSDART00000063950	psmc1b	0.032472485	0.214901771	0.338782243	0.27453656	0.103637979	midline crossing
ENSDART00000064032	eif4ebp1	0.004205562	0.426193835	0.395196044	0.352513449	0.114800609	midline crossing
ENSDART00000064067	ehbp1	0.008004874	0.439358276	0.535882532	0.636938582	0.35957066	midline crossing
ENSDART00000064311	arhgdia	0.018217202	0.49745023	0.520698759	0.529544756	0.127344369	midline crossing
ENSDART00000064462	psma6l	0.016647785	0.604857346	0.425092318	0.840213456	-0.035624292	midline crossing
ENSDART00000064509	stmn4l	8.18133E-06	2.57043941	3.576220537	3.295095816	2.31833518	midline crossing
ENSDART00000064666	prnpb	0.033001298	1.397742128	1.699664466	1.642053833	0.642245778	midline crossing
ENSDART00000064789	txn	0.000250511	1.421742139	1.973125	1.39538042	0.274434823	midline crossing
ENSDART00000064826	mov10a	0.000860824	0.329642907	0.141820564	0.774895549	-0.030140661	midline crossing
ENSDART00000064968	rasgef1bb	0.00655086	0.10103678	0.334291276	0.155368522	-0.094945106	midline crossing
ENSDART00000065563	ccdc90b	0.007961928	0.430098539	0.770932327	0.278586215	0.111905478	midline crossing
ENSDART00000065728	nrsn1	0.017738289	0.207141455	0.540557286	0.398847193	0.186515655	midline crossing
ENSDART00000065807	kctd13	0.056884231	0.261042411	0.352189581	0.50608049	0.336398415	midline crossing
ENSDART00000066385	hbz	0.013081285	0.819612283	1.064127771	1.5494854	0.440895582	midline crossing
ENSDART00000066471	adam8b	0.000414404	1.018895271	1.190253553	1.122506289	0.517972783	midline crossing
ENSDART00000066760	cct5	0.004847659	0.325276039	0.436851681	0.379245954	0.163513221	midline crossing
ENSDART00000067053	vta1	0.003953024	0.2430424	0.457630564	0.367763165	0.149685321	midline crossing
ENSDART00000067147	ANKRD50	0.000616782	0.122102324	0.471943767	0.546358337	0.270036712	midline crossing
ENSDART00000067168	pdzrn4	0.000390311	0.330865082	1.464493171	1.433744497	0.793719225	midline crossing
ENSDART00000067500	si:dkey-280e21.3	0.004246082	0.421024272	0.822626751	0.979586691	0.632877181	midline crossing
ENSDART00000067542	kcnk10b	6.48692E-06	2.685681832	3.478119742	3.471523284	2.49042133	midline crossing
ENSDART00000067776	rab10	0.010011285	0.291742266	0.421774457	0.459760968	0.189497347	midline crossing
ENSDART00000073452	si:ch211-113a14.12	0.043318153	0.801955974	0.847093718	0.855214481	0.503313711	midline crossing
ENSDART00000073564	tes	0.005380857	0.818084026	1.011033966	0.902633694	0.089277909	midline crossing
ENSDART00000073705	abcf1	0.050560604	0.284347738	0.522430424	0.46970431	0.327069748	midline crossing
ENSDART00000073735	rrad	0.003154242	0.475668516	0.908053908	0.772751604	0.279273357	midline crossing
ENSDART00000073970	uap1	0.000781013	0.430311379	0.939111864	0.868311508	0.206630788	midline crossing
ENSDART00000073981	eif2s1b	0.019981901	0.229006291	0.299741922	0.510349465	0.185458194	midline crossing
ENSDART00000074317	GSK3B (1 of many)	0.040539001	0.32408852	0.570538304	0.615748631	0.310967098	midline crossing
ENSDART00000074400	tia1	0.004813371	0.181056891	0.310121029	0.46787956	0.221267203	midline crossing
ENSDART00000075009	eif2s2	0.015666457	0.324008789	0.419532633	0.338652657	0.07614456	midline crossing
ENSDART00000075129	lrrc47	0.043968276	0.095574868	0.246973338	0.327055852	0.135309168	midline crossing
ENSDART00000075187	pdzd11	0.061577067	1.031277997	1.70516213	1.574957324	1.135180778	midline crossing
ENSDART00000075299	zgc:153911	1.2193E-05	1.082638325	2.093301655	1.816780233	1.137984438	midline crossing
ENSDART00000075351	zgc:112285	0.031816406	0.930923442	1.110746674	0.609701543	0.79082813	midline crossing
ENSDART00000075491	pop5	0.007140172	0.444559136	0.7451635	0.571282976	0.537107281	midline crossing
ENSDART00000076161	hoxb5b	0.013675681	2.652791903	3.351280664	2.618897772	0.864721852	midline crossing
ENSDART00000076571	rtn1a	0.001728686	0.31833938	1.022959786	0.981624341	0.706878795	midline crossing
ENSDART00000076600	rpe65c	0.030945673	1.983711129	2.589375601	1.902404689	1.435704768	midline crossing
ENSDART00000076815	NA	0.020105477	2.456176486	3.15180058	2.959048559	2.414794476	midline crossing
ENSDART00000077197	tmsb	2.92142E-06	1.972985822	3.598018383	3.544381625	2.894174576	midline crossing
ENSDART00000077445	pim3	0.007776904	-0.017745548	0.425023548	-0.018053873	0.016773558	midline crossing
ENSDART00000077484	zhx2a	0.005803283	0.117201305	0.356185793	0.299966576	0.024327323	midline crossing
ENSDART00000078156	srm	0.063878819	0.127220715	0.22303682	0.345313656	0.085476496	midline crossing
ENSDART00000078304	lzic	0.034279564	0.119250866	0.44378754	0.377529349	0.128075007	midline crossing
ENSDART00000078306	arhgef2	0.005059311	0.429610468	0.807274797	0.854804837	0.533632211	midline crossing
ENSDART00000078412	rps8a	0.006621714	0.416303691	0.456350868	0.419741526	0.122907452	midline crossing
ENSDART00000078438	NA	0.072487837	0.22834743	0.583517385	0.218267131	0.08549295	midline crossing
ENSDART00000078522	eef1g	0.00148037	0.462831941	0.651007925	0.567355432	0.209607784	midline crossing
ENSDART00000078543	syt11b	0.000114847	0.975892204	1.6420197	1.925407845	1.338618886	midline crossing
ENSDART00000078611	jac2	0.020202236	3.404844888	3.429750736	2.921387124	1.113891823	midline crossing
ENSDART00000078949	afap1l1b	6.16509E-05	1.061182692	1.814819842	1.643265844	0.877712611	midline crossing
ENSDART00000078953	afap1l1b	0.00092561	0.880715307	1.833091326	1.686561879	0.852421188	midline crossing
ENSDART00000079050	nutf2	0.041930419	0.337010875	0.380281227	0.325036311	0.156803484	midline crossing
ENSDART00000079173	lepr	0.000168321	1.173965688	1.129708824	0.920018098	0.6672678	midline crossing
ENSDART00000079528	ilk	0.003451532	0.338564211	0.350049719	0.346485507	0.032055843	midline crossing
ENSDART00000079563	fas	0.029614425	0.786193523	0.954741877	1.62170637	0.201092535	midline crossing
ENSDART00000079803	nmt1b	0.044608572	0.899927422	1.156945789	1.408075075	0.459974747	midline crossing
ENSDART00000080342	josd2	0.005001984	0.375878923	0.743043487	0.777511515	0.447917768	midline crossing

ENSDART00000080351	dhx57	0.034302368	0.296631931	0.310315824	0.343123119	0.20162921	midline crossing
ENSDART00000080664	zgc:86709	0.020123282	1.428698865	3.355720442	2.498933956	1.732893579	midline crossing
ENSDART00000080829	hspa14	0.003158234	0.672662829	0.694717457	0.8195912	0.418167344	midline crossing
ENSDART00000080900	cfap57	0.015156186	2.52486974	2.757508228	2.941219811	1.994632033	midline crossing
ENSDART00000081338	slc9a5	0.000216813	0.890559011	1.045395859	1.329125407	1.036960607	midline crossing
ENSDART00000081468	ccdc79	0.001875423	0.331555315	0.505423262	0.318137438	-0.103372006	midline crossing
ENSDART00000081646	glrx	0.001482107	0.838457918	1.280146822	1.066830025	0.395822945	midline crossing
ENSDART00000081832	ptpdc1b	0.000675011	0.49240902	0.580136174	0.651643292	0.340422756	midline crossing
ENSDART00000081966	rtn4a	0.011135713	0.42137623	0.811004738	0.55995797	0.082545275	midline crossing
ENSDART00000082082	gars	0.009491292	0.395042441	0.664200633	0.717176297	0.392291214	midline crossing
ENSDART00000082223	tax1bp3	0.002818463	0.85130321	1.256170012	1.075265217	0.22088107	midline crossing
ENSDART00000082264	pxdc1b	0.000212316	0.801924492	1.033443039	0.634881962	0.139425975	midline crossing
ENSDART00000082434	tgif1	1.75467E-05	0.822910382	1.229522702	1.441745222	0.912580325	midline crossing
ENSDART00000082715	camsap3	0.001948843	0.302702799	0.347512275	0.333666179	-0.022433363	midline crossing
ENSDART00000082830	KIAA0895L	0.000166256	0.216380627	0.852873359	0.835551488	0.401334264	midline crossing
ENSDART00000082983	clip2	0.000480106	0.69105479	0.899482735	1.166942455	0.710683744	midline crossing
ENSDART00000083467	parp8	0.025639797	0.336619688	0.399001121	0.399118225	0.307424215	midline crossing
ENSDART00000083890	usp24	0.033976169	0.219267617	0.201082864	0.325420731	0.216599954	midline crossing
ENSDART00000084184	aimp1	0.018217202	0.209084138	0.262090446	0.396284966	0.096096937	midline crossing
ENSDART00000084373	frmd4bb	0.000871881	0.582631667	0.828834738	0.835563839	0.412330382	midline crossing
ENSDART00000084771	pde9a	0.000212316	0.836076081	1.443202889	1.33955445	1.070879094	midline crossing
ENSDART00000085135	tbl1x	0.004493561	0.180884774	0.566948084	0.5040067	0.049402086	midline crossing
ENSDART00000085528	zgc:158659	0.002794034	0.601601674	0.738184978	0.82446023	0.805817257	midline crossing
ENSDART00000085894	pgm5	0.000309757	1.697021259	1.783911903	2.232651815	1.757015772	midline crossing
ENSDART00000087991	fndc3bb	0.000250511	0.509196457	0.583942244	0.486760061	0.158477497	midline crossing
ENSDART00000088141	ankrd34bb	7.10385E-05	1.379198636	2.420328938	2.155206475	1.17838067	midline crossing
ENSDART00000088290	raph1b	0.018433591	0.812305175	0.733830465	0.671238907	0.330964135	midline crossing
ENSDART00000090174	dock9b	0.004106215	0.356010204	0.810416379	0.943578275	0.58912554	midline crossing
ENSDART00000090406	dock11	0.003111385	0.704689621	0.535408523	0.615472635	0.363574103	midline crossing
ENSDART00000090771	cyth1a	0.00507534	1.102348748	1.012723243	0.839881021	0.442796833	midline crossing
ENSDART00000091140	snx21	0.036614379	0.278697916	0.637246869	0.128859029	0.534229621	midline crossing
ENSDART00000091409	smarcad1a	0.010885977	0.370815487	0.374702329	0.447117461	0.290143851	midline crossing
ENSDART00000092884	lrrc58b	0.002894799	0.353547699	0.596933578	0.36096561	0.06120599	midline crossing
ENSDART00000097176	CABZ01090749.1	0.000169264	1.277443607	2.06888261	1.727457254	0.676889483	midline crossing
ENSDART00000097194	serinc5	0.057511608	0.184853776	0.289193819	0.374387724	0.244648582	midline crossing
ENSDART00000098285	atf5a	0.018496153	0.558593719	0.754458044	0.445954377	0.160619888	midline crossing
ENSDART00000098750	pdlim5b	0.005948998	0.340621907	0.643165703	0.410131138	0.075261902	midline crossing
ENSDART00000099102	sept5a	0.003118176	0.488119217	0.804836681	0.729175054	0.142689277	midline crossing
ENSDART00000099248	rabggtb	0.029966068	0.075526469	0.438084725	0.330428907	0.205346366	midline crossing
ENSDART00000099566	si:ch211-244o22.2	0.032262181	0.576141582	0.625409237	0.697152634	0.270183175	midline crossing
ENSDART00000099690	fam129ab	0.000806652	1.024572276	1.489725431	1.470862577	0.737761428	midline crossing
ENSDART00000099764	zgc:153031	0.034733648	0.216426658	0.538240257	0.500334626	0.476962119	midline crossing
ENSDART00000099769	ccdc22	0.012893882	0.325453994	0.346355629	0.367424682	0.130188245	midline crossing
ENSDART00000099849	arntl2	9.24337E-05	0.982355145	1.78511579	1.609893892	1.255639962	midline crossing
ENSDART00000100156	agpat4	0.000784082	1.221504112	1.6039826	1.49974957	0.799345848	midline crossing
ENSDART00000100415	map3k7cl	0.000292971	1.690116609	2.517738065	2.168584787	1.392466275	midline crossing
ENSDART00000101204	alcamb	4.11188E-05	1.334306533	2.490758317	2.513428399	1.826617796	midline crossing
ENSDART00000101943	rragca	0.024838127	0.302260739	0.264945177	0.40523597	0.261316897	midline crossing
ENSDART00000102260	si:dkey-222f8.3	0.01219127	0.869989363	1.191058069	0.864781519	0.175715859	midline crossing
ENSDART00000102411	dctn1b	0.062774652	0.519457248	0.529061234	0.697567556	0.548863629	midline crossing
ENSDART00000102419	igf2bp2a	0.009024763	0.598022036	0.736011973	0.87621145	0.419702221	midline crossing
ENSDART00000102672	nck2a	0.019099714	0.205356684	0.423877669	0.371996615	0.147611944	midline crossing
ENSDART00000102843	src	0.04997602	0.333102086	0.263030754	0.362117443	0.2047457	midline crossing
ENSDART00000102952	suz12a	0.000445792	0.614499433	0.610664915	0.72051821	0.430761521	midline crossing
ENSDART00000103463	dnajc2	0.048389146	0.193851448	0.30912042	0.441518195	0.223806707	midline crossing
ENSDART00000103753	fn1a	0.044310242	2.705855593	3.095500526	2.8206804	2.414837328	midline crossing
ENSDART00000103755	fn1b	0.006568275	4.126396153	4.640072685	4.590004259	2.904538972	midline crossing
ENSDART00000103911	CABZ01077217.1	0.031270127	0.385992878	0.554652123	0.464993777	0.2375762	midline crossing
ENSDART00000104317	klf7b	0.000976528	0.389436838	1.156291561	1.140404424	0.652703479	midline crossing
ENSDART00000104616	lepr	0.000171958	0.762949533	0.720319043	0.625239528	0.526452567	midline crossing
ENSDART00000105399	cbarpb	0.001847721	0.427437854	0.525694666	0.646908744	0.015511077	midline crossing
ENSDART00000105597	si:ch211-129c21.1	0.007788648	3.291282195	4.994402871	3.669154828	0.685912998	midline crossing
ENSDART00000105767	fhl1a	0.018500869	0.562168934	1.496722819	1.339494009	0.93265064	midline crossing
ENSDART00000105898	TSTD1	0.006964515	0.614724746	0.98570488	0.724640457	0.337367855	midline crossing
ENSDART00000106048	ctnnd1	0.001938223	0.632970644	0.745690204	0.779606908	0.274224061	midline crossing
ENSDART00000106081	BX511034.2	0.013026885	0.124750693	0.347355362	0.433795514	0.117013159	midline crossing
ENSDART00000106172	rac1a	0.010965837	0.326396929	0.503439113	0.328932342	0.053159748	midline crossing
ENSDART00000108493	gareml	0.000703816	2.608537205	3.376838547	3.284344596	2.814340641	midline crossing
ENSDART00000108596	zgc:162150	0.006040855	0.993292495	1.420859127	1.257973066	0.026357222	midline crossing
ENSDART00000108831	espnla	0.018765627	0.888146475	0.74973908	0.792131149	0.719500985	midline crossing
ENSDART00000108963	CABZ01102109.1	0.0044585	0.394501993	0.771748701	0.296144389	0.075524582	midline crossing
ENSDART00000109138	hbegfa	6.40327E-05	0.956645779	1.991576979	1.27153829	0.330229075	midline crossing
ENSDART00000109252	nudt5	0.003219301	0.602324776	0.61383313	0.580277921	0.203918347	midline crossing
ENSDART00000109319	arf1	0.004134049	0.284053831	0.377774948	0.307132687	0.034662092	midline crossing
ENSDART00000109425	nkd3	0.010173969	3.023582344	2.734865433	2.70294964	2.317338999	midline crossing

ENSDART00000109535	gmps	0.012765568	0.181071124	0.257853381	0.376587367	0.112990813	midline crossing
ENSDART00000109552	baz2ba	0.004360669	0.627489244	0.525669422	0.712410774	0.393101067	midline crossing
ENSDART00000109573	akap6	0.000144061	0.927127599	1.241947706	1.04133516	0.789730231	midline crossing
ENSDART00000109759	tmx2a	4.58725E-05	2.647169883	3.21425121	2.659532867	1.569734674	midline crossing
ENSDART00000110040	sox11a	8.488E-05	1.573346229	1.723237215	1.23195588	0.781584101	midline crossing
ENSDART00000110064	plaua	0.00106989	1.293433748	1.730730518	1.0162544	0.507140212	midline crossing
ENSDART00000110478	zgc:174906	0.002541273	0.44828184	1.063594358	0.857934554	0.434989632	midline crossing
ENSDART00000110497	tmem63a	0.008300402	0.205919412	0.504153398	0.414725435	0.171827856	midline crossing
ENSDART00000110622	si:ch211-222l21.1	0.004840707	0.830701135	0.950120833	0.646058632	0.069324807	midline crossing
ENSDART00000110627	EPB41L1	0.000414404	0.536765101	1.012243023	0.739789984	0.259906496	midline crossing
ENSDART00000110777	eef1db	0.022054847	0.19406492	0.18930318	0.407362874	-0.014164442	midline crossing
ENSDART00000111324	zgc:193807	0.018752626	2.638791453	3.489955154	3.039693941	3.396234279	midline crossing
ENSDART00000111706	si:dkey-108k21.14	0.021895107	0.724790653	1.05338345	1.058888091	0.439899526	midline crossing
ENSDART00000111707	cacnb1	0.000480969	1.693120335	2.349827744	2.415610432	1.652036859	midline crossing
ENSDART00000111923	ajuba	0.004299513	0.639088613	1.058968297	0.866425943	0.103913512	midline crossing
ENSDART00000111966	arhgef10lb	0.003104562	0.250651161	0.391053857	0.470093443	0.290981355	midline crossing
ENSDART00000112028	si:ch211-180f4.1	1.23761E-05	2.108953042	1.952679055	1.909638244	1.352861352	midline crossing
ENSDART00000112550	mapk9	0.000730604	0.429625805	0.878911558	0.999507915	0.700660398	midline crossing
ENSDART00000112698	RNF14 (1 of many)	0.001654051	0.142790619	0.542852416	0.218671557	0.088301354	midline crossing
ENSDART00000112926	adora1b	0.026842776	0.387978003	0.609753862	0.576471225	0.261995134	midline crossing
ENSDART00000113101	smarcad1b	0.003021903	0.481835623	0.651517984	0.685097173	0.491069329	midline crossing
ENSDART00000113241	tmem163a	0.011023029	0.329551441	0.273820345	0.385569684	0.333416277	midline crossing
ENSDART00000113532	CABZ01053588.1	8.39504E-05	0.66169386	1.399901635	1.305166578	0.721799042	midline crossing
ENSDART00000113699	hgfb	0.002024233	1.149006949	1.556415854	1.569424226	0.45979101	midline crossing
ENSDART00000113732	orc6	0.018651417	1.104098282	1.496929933	0.974585154	0.388395497	midline crossing
ENSDART00000113752	si:dkey-6i22.5	0.014624499	1.012202134	1.421252013	0.919204178	0.319242336	midline crossing
ENSDART00000114024	fam107b	0.002708716	0.899175294	1.079533729	0.969523488	0.434748246	midline crossing
ENSDART00000114168	itga6a	0.000135341	1.998266436	2.016280588	1.463165074	0.79739173	midline crossing
ENSDART00000114272	nop2	0.022328328	0.471489342	0.531037709	0.420888693	0.177047326	midline crossing
ENSDART00000114497	si:dkey-204l11.1	0.008595135	0.312589581	0.828199841	0.99726607	0.068811015	midline crossing
ENSDART00000115244	MEX3A	8.28889E-05	1.222333965	1.709642669	1.900449694	1.354844663	midline crossing
ENSDART00000115403	NAV1 (1 of many)	8.51245E-05	0.648363377	0.712988904	0.734499876	0.497918559	midline crossing
ENSDART00000118384	SNORA16	0.046563471	0.565934898	0.9072012	0.499756229	0.494901316	midline crossing
ENSDART00000121861	prph	0.000935288	1.238160677	2.697856064	2.616616233	2.213149799	midline crossing
ENSDART00000121886	hdr	0.013310088	3.187835864	3.220765325	3.322516101	2.611876044	midline crossing
ENSDART00000122102	wee1	1.91914E-05	1.082199278	1.291191801	0.972164457	0.241130696	midline crossing
ENSDART00000122305	Metazoa_SRP	0.002202211	1.452964427	1.874874084	1.874716847	1.224958179	midline crossing
ENSDART00000122433	trim33l	0.029170799	0.38510745	0.766516023	0.671400212	0.185347257	midline crossing
ENSDART00000122628	junba	0.016136147	0.280813702	0.583374691	0.41181471	0.073998168	midline crossing
ENSDART00000123096	marcksa	1.28435E-05	1.85087225	2.605359702	2.620602183	2.013819083	midline crossing
ENSDART00000123203	vbp1	0.026626189	0.24089733	0.440645383	0.41864886	0.239214051	midline crossing
ENSDART00000123254	dpp9	0.014674041	0.199548032	0.283576523	0.332661933	0.196698648	midline crossing
ENSDART00000123263	phf20a	0.000701449	3.204242437	4.684552705	5.028378766	3.758919167	midline crossing
ENSDART00000123409	si:ch1073-296d18.1	0.016381291	0.174260872	0.155958113	0.452524339	-0.02334577	midline crossing
ENSDART00000123559	znf521	0.000648976	1.710422257	2.255410725	1.850379402	0.814044199	midline crossing
ENSDART00000123878	ppiaa	0.001021091	0.40248836	0.711363911	0.687992513	0.279031014	midline crossing
ENSDART00000123972	smarca5	0.039476911	0.189313952	0.223635359	0.34449924	0.204975788	midline crossing
ENSDART00000124660	ascc3	0.010970596	0.285037378	0.269547515	0.334772964	0.241795126	midline crossing
ENSDART00000124809	acsbg2	4.91848E-05	0.494562304	1.915512609	1.979085784	0.982887956	midline crossing
ENSDART00000124991	lmcd1	0.002680527	2.103120162	2.716646733	2.223480288	1.685964001	midline crossing
ENSDART00000124998	rtn2a	0.001225897	0.520408534	1.345034307	1.147677305	0.715797789	midline crossing
ENSDART00000125349	bada	0.001185711	0.528966306	1.266997252	1.043776037	0.694034948	midline crossing
ENSDART00000125371	mknk1	0.001093301	0.126961076	0.659607681	0.524109906	-0.03336174	midline crossing
ENSDART00000125971	gpc1b	0.020961781	0.246742539	0.417837609	0.415208578	0.061504531	midline crossing
ENSDART00000126038	tcp1	0.031160253	0.327351769	0.30721299	0.45795341	0.229539742	midline crossing
ENSDART00000126041	ano10a	0.016565752	0.295415156	0.394412522	0.244334431	0.10037004	midline crossing
ENSDART00000126260	anxa6	0.059606535	0.678382414	0.731151807	0.839317026	0.481712468	midline crossing
ENSDART00000126414	eml1	0.019102956	0.475872706	0.625227433	0.675164018	0.217118522	midline crossing
ENSDART00000126474	si:ch211-265o23.1	0.039039737	1.117262816	1.407297939	1.180818753	0.875131455	midline crossing
ENSDART00000126866	ccser2b	0.025259321	0.139738782	0.390945754	0.269322283	0.164198289	midline crossing
ENSDART00000127420	MDP1	3.63088E-06	1.927039626	2.737884833	2.522148059	1.176014094	midline crossing
ENSDART00000127695	rpl5b	0.003761148	0.469645013	0.524119827	0.418266352	0.096930554	midline crossing
ENSDART00000127699	si:ch73-335l21.4	0.002818463	1.682684641	2.104012275	1.803809975	0.730936452	midline crossing
ENSDART00000127977	CABZ01016547.1	0.016879359	2.8163175	2.999575494	2.826245437	2.227407522	midline crossing
ENSDART00000128050	UTP14C	0.001174125	0.741190051	0.667056074	0.681031837	0.432356782	midline crossing
ENSDART00000128284	nudt9	0.002466999	0.256265084	0.958235099	0.875663921	0.386996856	midline crossing
ENSDART00000128346	si:ch211-108c6.2	0.021500865	1.285191812	1.464423055	1.491945601	0.337219819	midline crossing
ENSDART00000128624	sgk494a	0.001650308	0.606573335	0.92915728	0.662849036	0.240608511	midline crossing
ENSDART00000128715	kif3cb	0.000188131	0.538226215	1.727920879	1.708926367	1.162753729	midline crossing
ENSDART00000128894	dchs1b	0.040504381	0.298598944	0.508598812	0.623831745	0.178131833	midline crossing
ENSDART00000128931	igf2bp2a	0.017671888	1.122085018	1.168437537	1.475101165	0.642256664	midline crossing
ENSDART00000129362	eef1a1l2	0.00039492	2.651521704	2.343527866	2.861864827	2.05813724	midline crossing
ENSDART00000129423	slc37a1	0.001367004	0.300742512	0.561138843	0.611837666	0.153755959	midline crossing
ENSDART00000129593	znf217	0.055515409	0.260126556	0.54491492	0.40143457	0.121556273	midline crossing
ENSDART00000129674	slc44a2	0.015120147	0.253178883	0.658899779	0.831363022	0.428394942	midline crossing

ENSDART00000129749	psmb7	0.002585852	0.370284184	0.518350984	0.484691258	0.174219127	midline crossing
ENSDART00000129772	gck	0.004578126	2.162290839	3.39609934	3.505078852	1.699355805	midline crossing
ENSDART00000129895	palm1b	0.013143068	0.197850318	0.594118975	0.532496272	0.226704229	midline crossing
ENSDART00000129975	scamp4	0.001296944	0.678060482	0.570045099	0.569688168	0.357822663	midline crossing
ENSDART00000130277	rpl9	0.000286897	0.669628298	0.786807801	0.580702605	0.163431583	midline crossing
ENSDART00000130343	parp12a	0.011523209	0.340672377	0.108363497	1.07497332	-0.29758817	midline crossing
ENSDART00000130353	fdps	0.049349672	0.408185548	0.643895342	0.766681528	0.202680939	midline crossing
ENSDART00000130472	pkhd1l1	0.064070454	0.896390148	0.750907757	1.185805195	0.60737198	midline crossing
ENSDART00000130626	rars	0.001161091	0.486608727	0.578942242	0.46060205	0.253410521	midline crossing
ENSDART00000130675	pi4k2a	0.002785677	0.32642219	0.46314623	0.446740681	0.229275132	midline crossing
ENSDART00000130815	e2f3	0.079057004	0.222958128	0.350714695	0.469146338	0.300980509	midline crossing
ENSDART00000130820	hbegfb	0.000370918	1.26577708	2.42087461	1.934889729	0.952474054	midline crossing
ENSDART00000131768	abi1a	0.011319743	0.352423076	0.342648792	0.429769283	0.194498692	midline crossing
ENSDART00000131861	tmsb2	0.01863352	0.228394728	0.766531981	0.776266217	0.553413207	midline crossing
ENSDART00000131922	ucp3	0.045293752	0.966573752	1.664449524	1.416927563	1.012896527	midline crossing
ENSDART00000131940	si:dkey-30c15.10	0.000543419	1.656397572	1.745533662	1.503033451	0.676482739	midline crossing
ENSDART00000131956	si:dkey-33c12.14	0.026803174	2.548697019	3.072973071	2.718694487	2.992696235	midline crossing
ENSDART00000132146	hsd17b7	0.043380755	2.880332402	3.535090877	2.510243514	2.078786507	midline crossing
ENSDART00000132156	cirbpb	0.03016157	0.302166347	0.411233461	0.214244406	0.367929369	midline crossing
ENSDART00000132297	adam9	0.003234084	0.312303854	0.492550355	0.565368245	0.224580764	midline crossing
ENSDART00000132386	si:dkey-21e2.15	0.014410292	2.037905679	4.749110822	3.364621679	1.867440085	midline crossing
ENSDART00000132426	stmn4	0.000135804	0.67103814	1.650930846	1.637640616	1.211182541	midline crossing
ENSDART00000132437	mknk2a	0.012665885	1.021696261	1.216138852	0.746540318	1.080690265	midline crossing
ENSDART00000132663	si:ch211-163b2.4	0.00520796	0.347936584	0.995221531	0.769857122	0.372703166	midline crossing
ENSDART00000132696	nckap1	0.003535619	0.516973126	0.759090684	0.845846182	0.627500278	midline crossing
ENSDART00000132781	si:dkey-78l4.1	0.00038013	3.429067431	4.682405989	3.946041907	2.798505508	midline crossing
ENSDART00000132788	uap1	0.011788375	1.271261725	1.549537095	1.061485612	0.525207108	midline crossing
ENSDART00000132982	leng9	0.002204563	3.031473639	2.780356944	2.241298703	1.634482288	midline crossing
ENSDART00000133030	psmd1	0.054998036	3.699340083	3.88833786	2.358920441	3.148062929	midline crossing
ENSDART00000133183	parp8	0.045069362	0.666223987	0.351288157	0.707539986	0.438871753	midline crossing
ENSDART00000133497	si:dkey-250k15.4	0.028695396	0.495799772	1.080770342	1.149092422	0.612727116	midline crossing
ENSDART00000133628	ankib1a	0.004447566	0.478883806	0.425404685	0.598914996	0.306729273	midline crossing
ENSDART00000133708	arhgef4	0.03479782	0.196005293	0.61534617	0.621912538	0.36146972	midline crossing
ENSDART00000133775	pdlim3b	0.031565534	2.202802348	2.787120555	2.295385553	1.784176005	midline crossing
ENSDART00000133954	mhc1uka	4.64299E-05	4.819339023	5.453978456	5.536236268	3.849327613	midline crossing
ENSDART00000133968	camkmt	0.014401489	0.353110068	0.455754701	0.544139309	0.380701512	midline crossing
ENSDART00000134094	zgc:162193	0.04742464	0.951212203	1.108680472	1.240038838	0.640044887	midline crossing
ENSDART00000134269	fkbp1aa	0.020289809	0.162085973	0.545601006	0.44355879	0.374542557	midline crossing
ENSDART00000134658	slc44a1a	0.029185454	0.707497042	0.591443803	0.629657706	0.651062281	midline crossing
ENSDART00000134787	elavl4	0.000177237	1.446773831	1.526430349	1.657041106	1.070021064	midline crossing
ENSDART00000134843	sst6	0.005534146	1.638903894	2.735989277	2.458183626	2.301951228	midline crossing
ENSDART00000135093	ola1	0.004885993	0.415909116	0.691871408	0.432580712	-0.033470782	midline crossing
ENSDART00000135356	nabp1a	0.057008548	0.371235634	0.392313666	0.26759026	0.229305948	midline crossing
ENSDART00000135415	dysf	0.021103936	3.146286589	3.204629318	3.124686608	3.126558821	midline crossing
ENSDART00000135631	myl6	0.006911648	0.462877699	0.806124657	0.938068157	0.529055927	midline crossing
ENSDART00000135661	slc20a1a	0.021002038	1.716116201	1.644628019	1.747440585	0.991048186	midline crossing
ENSDART00000135973	nfyal	0.007974988	0.202128253	0.401502491	0.256237772	0.346886156	midline crossing
ENSDART00000136152	calua	0.009163406	0.347598327	0.562237059	0.505429013	0.302262295	midline crossing
ENSDART00000136182	si:ch211-222n4.2	0.000461528	2.334818577	2.498262776	2.056155354	0.938824321	midline crossing
ENSDART00000136187	bloc1s4	0.017000306	0.27836522	0.479128543	0.397988078	0.159673351	midline crossing
ENSDART00000136296	CR847543.2	0.012261156	0.7189738	0.800339311	1.432214548	0.183639146	midline crossing
ENSDART00000136492	si:ch211-158d24.4	0.020892176	0.620322497	0.916930916	0.610223614	0.170743477	midline crossing
ENSDART00000136761	prkx	0.002147221	0.698491961	1.03323314	0.943847516	0.577218336	midline crossing
ENSDART00000136782	si:dkey-24c2.7	3.29362E-06	4.902793471	4.654641885	4.451812012	3.610899339	midline crossing
ENSDART00000136830	stmn4l	0.000324566	1.323269884	1.682347243	1.639725976	1.096832109	midline crossing
ENSDART00000136873	clasp2	0.012069725	0.301439566	0.245236713	0.309865242	0.356365378	midline crossing
ENSDART00000136910	armc1l	0.0517318	0.626427947	0.775572599	0.634581641	0.292392529	midline crossing
ENSDART00000136943	si:dkey-21e2.13	0.047530035	2.273396315	3.247336732	1.860650754	0.467276759	midline crossing
ENSDART00000137036	ppiaa	0.007708544	0.323927062	0.685997539	0.775560135	0.372712842	midline crossing
ENSDART00000137110	sepw1	0.028322957	0.165066135	0.642417047	0.502149684	0.242826446	midline crossing
ENSDART00000137309	gemin5	0.01891028	0.29381973	0.279160353	0.398962684	0.210137956	midline crossing
ENSDART00000137325	mvp	0.001650308	1.415505945	1.485945809	1.461661333	0.448751577	midline crossing
ENSDART00000137471	MFAP4 (1 of many)	0.067929911	1.677720699	2.807166131	2.128226179	1.146528193	midline crossing
ENSDART00000137782	yif1a	0.072487837	0.379643142	0.667454267	0.241343175	0.280143891	midline crossing
ENSDART00000137795	ssrp1a	0.014296946	0.416335187	0.470997147	0.449691903	0.161896374	midline crossing
ENSDART00000138094	eef1b2	0.002299086	0.3943229	0.586163757	0.433468435	0.083803882	midline crossing
ENSDART00000138143	si:ch211-233m11.1	0.012369008	0.782546411	0.773895723	0.974137465	0.285762089	midline crossing
ENSDART00000138350	rps12	0.000740604	0.592717981	0.744811164	0.572850481	0.279364485	midline crossing
ENSDART00000138351	cth	0.000144591	1.012814265	1.438428325	1.066275824	0.495805428	midline crossing
ENSDART00000138606	las1l	0.028216825	0.170918093	0.302723725	0.361920168	0.174466647	midline crossing
ENSDART00000138621	zgc:153867	0.004376172	3.188427096	3.682050782	3.111570491	2.809604178	midline crossing
ENSDART00000138633	cd99	0.001871569	1.201301736	1.818711588	1.295470825	0.581149232	midline crossing
ENSDART00000138821	nfil3	0.000101984	1.376309909	2.121391468	1.99718084	1.39511595	midline crossing
ENSDART00000138915	cirbpb	0.015289564	0.297824374	0.314736859	0.24846932	0.269446209	midline crossing
ENSDART00000139002	dus2	0.040333038	0.373595906	0.357863942	0.47349873	0.230284081	midline crossing

ENSDART00000139176	eif2s3	0.02245917	0.381558608	0.49753559	0.407153714	0.243011405	midline crossing
ENSDART00000139241	rnf38	0.0044585	0.363207367	0.385948281	0.537494883	0.39766179	midline crossing
ENSDART00000139259	zfand5b	0.022389941	0.677389122	0.588881516	0.462655957	0.625250498	midline crossing
ENSDART00000139392	rras	0.003337326	2.928045636	3.065800127	2.84735064	1.624630606	midline crossing
ENSDART00000139412	SMIM18	0.035424549	0.730335352	0.956017708	0.99931328	0.671209714	midline crossing
ENSDART00000139449	si:dkey-147f3.4	0.000116387	1.865073313	2.098559635	1.808022762	0.571602171	midline crossing
ENSDART00000139454	pde4cb	0.055423595	0.616688899	0.67005432	0.986689732	0.473877974	midline crossing
ENSDART00000139479	slc12a9	0.004483567	0.435405363	0.483188456	0.53485188	0.300178427	midline crossing
ENSDART00000139722	dmtn	2.54845E-05	1.105433633	1.577455563	1.872515003	1.217364091	midline crossing
ENSDART00000139763	stat5a	0.018355029	3.442434617	3.964844196	3.417693165	3.322184683	midline crossing
ENSDART00000139892	elfn2a	0.064943452	0.520234542	0.439147218	0.642970325	0.553931258	midline crossing
ENSDART00000139947	rapgef1a	0.043449081	0.201307435	0.3482683	0.17911742	0.096155984	midline crossing
ENSDART00000140032	si:dkeyp-69c1.9	0.086002228	1.662700986	3.288206015	1.780235269	1.584891446	midline crossing
ENSDART00000140153	mri1	0.000415146	1.105039291	1.085223643	1.409079861	0.961058569	midline crossing
ENSDART00000140253	arhgef10lb	0.006717057	0.447026855	0.470705895	0.502550702	0.280062014	midline crossing
ENSDART00000140490	MFAP4 (1 of many)	0.010636065	1.29105804	2.228445007	1.795046858	1.318796638	midline crossing
ENSDART00000140537	dhrs11a	0.010679495	0.251388963	0.647205288	0.714640117	0.4437028	midline crossing
ENSDART00000140736	si:dkey-42i9.6	0.00486688	0.573524661	0.712949488	0.985257189	0.193370375	midline crossing
ENSDART00000141068	sox11b	8.488E-05	1.611838536	2.177304965	2.010624009	1.194765222	midline crossing
ENSDART00000141107	cd99	0.005438298	2.277092862	3.207492883	2.696592348	2.108764519	midline crossing
ENSDART00000141173	elavl4	0.000104621	2.4638469	2.569363943	2.090923257	1.273250901	midline crossing
ENSDART00000141328	pip5kl1	0.027686308	0.719167688	0.681103792	0.759572826	0.613914474	midline crossing
ENSDART00000141382	rab34b	0.005807113	1.020539104	1.398669915	1.148932644	0.356641036	midline crossing
ENSDART00000141385	si:dkey-27p18.3	0.023714388	0.644114707	0.569712036	1.095314507	0.257528776	midline crossing
ENSDART00000141578	shmt1	0.002456687	2.367581981	4.511004025	4.354684857	-7.25195E-16	midline crossing
ENSDART00000141606	commd8	0.007621679	0.496320125	0.646713153	0.422272129	0.302789387	midline crossing
ENSDART00000141625	si:ch211-156p11.1	0.075460344	0.900660828	1.143855847	0.822477469	0.791150698	midline crossing
ENSDART00000141837	esd	0.031729112	0.782720008	0.801148933	0.794217077	0.347191174	midline crossing
ENSDART00000141877	kcnk10a	0.000157775	1.57307368	1.979146889	1.661354893	0.924431526	midline crossing
ENSDART00000142146	dmtn	0.027138478	0.637268571	0.800533621	0.832554824	0.713529971	midline crossing
ENSDART00000142356	smyd2a	0.012342358	2.425657932	2.779397529	2.178998127	1.672270997	midline crossing
ENSDART00000142428	josd2	0.004387455	0.235661569	0.659033561	0.722639048	0.359420457	midline crossing
ENSDART00000142569	specc1lb	0.004391006	0.378697075	0.547073638	0.617855803	0.275260653	midline crossing
ENSDART00000142692	eef1b2	0.008579097	0.349947783	0.50162657	0.39446346	0.093426844	midline crossing
ENSDART00000142873	BX901918.1	0.068563448	0.845880305	0.530715844	1.008998061	0.591786557	midline crossing
ENSDART00000143056	zyx	0.011047999	0.416244119	0.475600395	0.593066786	0.15882784	midline crossing
ENSDART00000143457	cndp2	0.016866534	0.393197537	0.458029638	0.473219597	0.244423454	midline crossing
ENSDART00000143562	gbp2	0.021682969	0.680404258	0.927169543	1.353668195	0.527828388	midline crossing
ENSDART00000143723	idi1	0.030676359	0.080932725	0.304370095	0.372374682	0.127047939	midline crossing
ENSDART00000143741	fkbp5	2.85018E-05	1.764298053	1.865156046	1.631976013	1.871443577	midline crossing
ENSDART00000143793	C3 (1 of many)	0.009758715	3.237329668	4.023510735	3.503827488	2.024562274	midline crossing
ENSDART00000144503	ran	0.011399481	-0.075551405	0.448221073	0.249018928	0.118064827	midline crossing
ENSDART00000144516	lonrf1l	0.001677265	0.003516441	0.629397595	-0.101368349	0.117101343	midline crossing
ENSDART00000144814	si:dkey-31f5.8	0.019331363	0.429768416	1.027387879	0.82502917	0.369456203	midline crossing
ENSDART00000144817	cfl1	0.008723451	0.341949415	0.252829572	0.49073898	0.258667203	midline crossing
ENSDART00000144946	adcyap1b	0.016974377	2.385755846	2.421463563	2.507590258	1.873754889	midline crossing
ENSDART00000145095	celsr3	0.003711093	0.648145926	0.648607509	0.829081729	0.619308918	midline crossing
ENSDART00000145103	cntfr	0.071227112	0.307964555	0.391102809	0.626651223	0.264745691	midline crossing
ENSDART00000145379	fblim1	0.003095428	0.305913455	0.545649791	0.383397127	0.135782412	midline crossing
ENSDART00000145956	ptpn23a	0.029718853	0.252886396	0.192353563	0.35477406	0.187678789	midline crossing
ENSDART00000145999	prx	0.022530244	1.586883428	3.204392125	2.633047079	1.399238803	midline crossing
ENSDART00000146005	gtpbp1	0.02305104	0.461505187	0.53679144	0.551879705	0.330029037	midline crossing
ENSDART00000146411	sst6	0.000370918	0.736642137	1.700467827	1.549366173	1.261691667	midline crossing
ENSDART00000146434	zgc:153115	0.001249772	0.499570303	0.817313835	0.902072926	0.441195032	midline crossing
ENSDART00000146611	cxcr3.3	0.009119552	0.415816929	0.611232638	0.663522207	-0.008061564	midline crossing
ENSDART00000146744	nfkbiab	0.003196539	0.787570548	0.783257317	0.468593447	0.633863732	midline crossing
ENSDART00000147019	si:ch211-235o23.1	0.001130346	0.394717538	0.700152429	0.448882118	0.273599764	midline crossing
ENSDART00000147264	fam213b	0.046818285	0.418860252	0.552271711	0.440362956	0.212029386	midline crossing
ENSDART00000147518	fam89b	0.007332698	0.288965136	0.576511697	0.598868415	0.448283091	midline crossing
ENSDART00000147821	spsb4a	0.001871569	0.371275002	0.33311003	0.599280348	0.314031744	midline crossing
ENSDART00000147849	klf6a	0.000870446	1.130982604	1.870693821	1.403514717	0.445060502	midline crossing
ENSDART00000147851	eef1b2	0.000349374	0.252066551	0.849118612	0.755935234	0.422914839	midline crossing
ENSDART00000148011	cby1	0.052184053	0.256002046	0.39067073	0.214338805	0.125714094	midline crossing
ENSDART00000148138	lrrc8aa	0.002977128	0.370743632	0.558987897	0.552524282	0.096775057	midline crossing
ENSDART00000148154	stx2a	0.012799526	0.73746977	1.316523952	0.880682553	0.300142462	midline crossing
ENSDART00000148163	frmd4bb	0.000286897	0.943172358	1.400649402	1.507648518	0.548522517	midline crossing
ENSDART00000148388	smox	0.001452556	1.14663E-15	4.65884204	4.212605499	2.256263175	midline crossing
ENSDART00000148432	ptp4a3	0.003196078	0.27125556	0.468426427	0.608500351	-0.013500628	midline crossing
ENSDART00000148601	insig1	0.008603534	-0.03765091	0.839125818	0.622258944	0.165134159	midline crossing
ENSDART00000148659	clip2	0.000391808	0.500760868	0.904821279	1.156870185	0.693646301	midline crossing
ENSDART00000148822	adgrg1	0.015695496	0.746405238	1.031170771	0.764826924	0.143644812	midline crossing
ENSDART00000148993	hdac6	0.033606114	0.150136767	0.327518494	0.405976253	0.20477563	midline crossing
ENSDART00000149074	sall1b	2.18017E-05	1.974229239	2.883794957	2.506155499	1.735121972	midline crossing
ENSDART00000149115	zgc:158659	4.64273E-06	1.901261478	2.561373529	3.048076346	2.366410956	midline crossing
ENSDART00000149320	arl8a	0.011240617	0.200543842	0.321313695	0.387648659	0.1981557	midline crossing

ENSDART00000149910	abl1	0.034395507	0.087815124	0.252042838	0.385819934	0.051793038	midline crossing
ENSDART00000149978	fabp7a	0.000717715	0.33770709	0.764013846	0.558825405	0.488886506	midline crossing
ENSDART00000150013	cd276	1.1419E-05	1.481977913	1.919249755	1.718653495	0.833090259	midline crossing
ENSDART00000150149	tdp1	0.042010311	0.245251359	0.536780821	0.276916415	0.174119987	midline crossing
ENSDART00000150391	si:ch211-214j24.14	0.000870998	2.502222784	2.698584631	2.367333489	0.935046128	midline crossing
ENSDART00000150651	BX465862.4	0.011605032	0.896323656	0.956165673	0.709439713	0.378868912	midline crossing
ENSDART00000150692	NA	0.001955129	0.448027135	0.604850915	0.68748119	0.482541087	midline crossing
ENSDART00000150961	wdr91	0.030491542	4.358324282	4.633846856	4.480446443	4.649897846	midline crossing
ENSDART00000151247	scn1lab	0.035957782	0.293159534	0.684635422	0.628869811	0.299982725	midline crossing
ENSDART00000151448	vamp4	0.002952724	0.36274105	0.703182259	0.708668654	0.04883985	midline crossing
ENSDART00000151613	lhfpl2a	0.025409101	2.211015019	2.8976425	2.404140419	1.864383361	midline crossing
ENSDART00000151679	si:ch73-233k15.2	0.008447718	0.305634484	0.260016753	1.072404438	-0.272816623	midline crossing
ENSDART00000151785	klc4	0.002952432	0.265672724	0.701188452	0.757625768	0.469102543	midline crossing
ENSDART00000151892	BX248324.2	9.23128E-05	1.389473856	2.137590244	1.979774015	1.113747327	midline crossing
ENSDART00000151999	ttc27	0.000370918	0.537657382	0.63628465	0.751878141	0.471568333	midline crossing
ENSDART00000152240	si:ch1073-174d20.2	0.045564466	0.11047675	0.254355415	0.357239942	0.147146143	midline crossing
ENSDART00000152295	FAM83G	0.016063659	1.150380037	1.206829236	1.079415548	0.591155843	midline crossing
ENSDART00000152318	si:ch211-286b5.2	0.002663982	1.976393579	3.213645724	2.557166791	1.560923939	midline crossing
ENSDART00000152510	ccser2b	0.001067718	0.726161978	0.836833026	1.118702985	0.610749876	midline crossing
ENSDART00000152562	eif3f	0.018854609	0.257721359	0.386526337	0.235538886	0.038968621	midline crossing
ENSDART00000152641	dnm3a	0.000272902	1.808565272	2.497351665	2.011092537	0.57584064	midline crossing
ENSDART00000152680	pyyb	0.000396404	2.62463081	4.023644413	3.815932945	2.663962921	midline crossing
ENSDART00000152855	prdx1	0.045741098	2.014399342	2.912277881	2.532639813	2.043057891	midline crossing
ENSDART00000153194	si:dkey-38p12.3	0.015825859	0.25264745	0.485169484	0.572803819	0.07332987	midline crossing
ENSDART00000153199	afap1	0.00834805	0.840574953	1.106233667	1.438882155	1.005659187	midline crossing
ENSDART00000153200	si:ch211-194k22.8	0.033503027	0.210290351	0.451170205	0.37501933	0.225583201	midline crossing
ENSDART00000153225	GUCY2C (1 of many)	0.032143116	3.255008425	3.866902647	2.201523204	2.781639432	midline crossing
ENSDART00000153227	nfe2l1b	0.013323448	0.688085235	1.301360444	1.138008541	0.729999096	midline crossing
ENSDART00000153306	hcar1-4	0.004505979	0.883818254	1.413387657	1.160612678	0.305432292	midline crossing
ENSDART00000153307	si:ch73-344o19.1	0.006344596	0.586530995	0.605191095	0.75799445	0.479323327	midline crossing
ENSDART00000153452	hsp90ab1	0.030379124	0.284157203	0.639252574	0.548907136	0.495783326	midline crossing
ENSDART00000153666	AL732567.1	0.002559269	0.845331402	1.050170733	0.86587331	0.828206988	midline crossing
ENSDART00000153841	lmcd1	0.012070853	1.804357101	2.873087613	2.523453656	2.002290777	midline crossing
ENSDART00000154109	tp53inp2	0.001954005	0.254956915	0.876540848	0.694070175	0.684041374	midline crossing
ENSDART00000154212	BX323824.1	0.024957938	0.386508588	0.636151192	0.398585293	0.423381311	midline crossing
ENSDART00000154314	prph	6.48692E-06	1.502464936	3.204214666	3.264208069	2.453842104	midline crossing
ENSDART00000154321	BX908750.1	0.050473914	0.337703917	0.710327992	0.54373662	0.188876538	midline crossing
ENSDART00000154377	HIST1H2BM (1 of many)	0.051183892	0.871205458	1.144896653	0.89956001	0.544514919	midline crossing
ENSDART00000154543	prelid3a	0.006027331	0.578261204	0.829307037	0.712329147	0.291766611	midline crossing
ENSDART00000154573	prob1	0.001652227	1.935993422	2.091368151	1.752154811	1.442205958	midline crossing
ENSDART00000154628	fam101b	0.000888975	0.450957894	0.982590486	0.845377619	-0.490650185	midline crossing
ENSDART00000154846	CR381618.1	0.002605308	0.115176903	0.900181702	1.102625706	0.253393379	midline crossing
ENSDART00000154851	zgc:153405	0.027461561	0.317950752	1.076463864	0.674378682	0.394781222	midline crossing
ENSDART00000154880	arhgef33	0.01779887	0.636994605	0.687196577	0.646579762	0.300121216	midline crossing
ENSDART00000155051	mych	0.002016009	1.81452306	1.847457308	1.423042212	0.692601562	midline crossing
ENSDART00000155188	ccnjl	0.031062271	0.284030133	0.391162848	0.492680219	0.242842213	midline crossing
ENSDART00000155256	VWA5A (1 of many)	0.021065097	0.207030506	0.487713239	0.641785484	0.122484219	midline crossing
ENSDART00000155280	GDPGP1	0.018409732	0.576074599	0.656458452	0.674683139	0.299654628	midline crossing
ENSDART00000155440	eif3ba	0.00350062	0.265595321	0.439061322	0.461693724	0.229141991	midline crossing
ENSDART00000155537	VWA5A (1 of many)	0.000220872	1.206080865	0.987004598	1.27402251	0.722325665	midline crossing
ENSDART00000155751	nuak1b	1.33565E-05	0.785416985	1.722603254	1.683891996	1.302083807	midline crossing
ENSDART00000156034	VWA5A (1 of many)	8.51245E-05	1.04711946	1.090403168	1.260861512	0.694741705	midline crossing
ENSDART00000156038	epn2	0.034236293	0.165862475	0.434471463	0.333526121	0.113707323	midline crossing
ENSDART00000156167	si:ch73-182a11.2	0.007880813	0.438099394	0.613388756	0.677790276	0.474745988	midline crossing
ENSDART00000156224	dhx33	0.005024347	0.472474316	0.556602191	0.639276654	0.492917502	midline crossing
ENSDART00000156251	ehbp1	0.005375255	0.342339175	0.353753636	0.38894242	0.04618254	midline crossing
ENSDART00000156255	ago1	0.004702472	0.402935641	0.576022371	0.656640241	0.478601065	midline crossing
ENSDART00000156411	fam98a	0.014485141	0.369288002	0.399859986	0.477248543	0.233728964	midline crossing
ENSDART00000156415	dock9b	0.006352299	0.187525252	0.556466903	0.665071238	0.341730571	midline crossing
ENSDART00000156774	dph6	0.010921357	0.485952337	0.860504803	0.608054536	0.313680056	midline crossing
ENSDART00000156913	BX927184.1	0.000109677	1.282107066	1.77927465	1.802027966	1.376990986	midline crossing
ENSDART00000156970	si:ch73-265h17.5	0.019558454	1.250341969	1.481314496	1.76830523	0.8438116	midline crossing
ENSDART00000156972	slc12a7a	0.016311478	0.533547881	0.750475895	0.543271513	0.05077876	midline crossing
ENSDART00000157127	lmcd1	0.008776907	1.160892936	2.271204052	2.063825472	1.282095985	midline crossing
ENSDART00000157139	tmbim1a	0.004528919	0.963253296	1.499596205	1.306783479	0.549513539	midline crossing
ENSDART00000157350	CT573344.1	0.023257205	0.592860846	1.013860007	1.275401288	0.850112465	midline crossing
ENSDART00000157441	filip1l	0.004552273	1.025025125	2.361056221	1.959563135	1.214723721	midline crossing
ENSDART00000157597	Metazoa_SRP	0.000305619	3.784983496	4.068975573	4.489534416	3.689887905	midline crossing
ENSDART00000157604	si:dkey-19c16.12	0.067756277	0.518457933	0.406892634	0.690003002	0.445775315	midline crossing
ENSDART00000157678	nop53	0.020688276	0.221649254	0.357738319	0.319808875	0.087032288	midline crossing
ENSDART00000157824	slc6a15	0.001558204	0.370043272	0.932121448	1.025205824	0.470213629	midline crossing
ENSDART00000157894	apaf1	0.033808649	0.69901776	0.613857472	0.804406673	0.575412751	midline crossing
ENSDART00000157907	rpl34	0.021862757	0.216382556	0.50724745	0.251806906	0.167915072	midline crossing
ENSDART00000158016	flnca	4.50375E-07	5.435651542	4.853809922	4.737861308	3.940836376	midline crossing
ENSDART00000158272	dclk1b	0.007797716	0.134331666	0.528656936	0.571389243	0.299537528	midline crossing

ENSDART00000158739	Metazoa_SRP	0.027491007	1.431390172	1.797550169	1.401344471	0.830250287	midline crossing
ENSDART00000158744	BX927336.3	0.000371354	4.021248005	3.307131569	3.405842268	3.528289574	midline crossing
ENSDART00000159024	btr29	0.003508363	2.93548853	3.916483422	3.440636421	1.361130093	midline crossing
ENSDART00000159034	rsu1	0.003279742	0.959727483	0.959427671	0.950095712	0.424675541	midline crossing
ENSDART00000159256	kif5c	0.031850333	0.534952361	0.603334839	0.411520272	0.273079865	midline crossing
ENSDART00000159259	capn1b	4.91601E-05	0.6323058	1.402210713	1.384902028	1.097440242	midline crossing
ENSDART00000159300	mcm7	0.002876227	0.714704002	0.831682391	0.874291792	0.339164006	midline crossing
ENSDART00000159340	ctnnd1	0.000135332	0.771872624	0.982959313	0.944642837	0.226155974	midline crossing
ENSDART00000159372	eif3ha	0.001128218	0.499618724	0.599836819	0.638052337	0.263915251	midline crossing
ENSDART00000159652	sox4b	0.008897727	0.767109202	1.080655119	1.389017231	0.976257694	midline crossing
ENSDART00000159732	si:dkey-203a12.8	0.050696552	3.015691266	3.637172924	3.104587293	1.713663998	midline crossing
ENSDART00000159949	wdr1	0.082310414	0.362540326	0.308247591	0.564916756	0.170183845	midline crossing
ENSDART00000159965	si:ch211-255i3.4	0.025487547	0.121150704	0.407179882	0.241107117	0.125992518	midline crossing
ENSDART00000160006	Metazoa_SRP	0.001666219	1.639017988	2.129447988	1.830106321	0.946987666	midline crossing
ENSDART00000160206	rap2b	0.014591443	0.536023906	0.628551975	0.691733134	0.267820563	midline crossing
ENSDART00000160492	alad	0.036567119	0.30171604	0.443307609	0.248348623	0.158762163	midline crossing
ENSDART00000160575	CU459186.1	0.043586255	0.73831154	1.064084865	0.743119809	0.540430362	midline crossing
ENSDART00000160604	ftsj3	0.00161581	0.856590556	0.695492085	0.825371198	0.428239045	midline crossing
ENSDART00000160663	tnk2b	0.032721257	0.180874765	0.228576581	0.365115453	0.173216656	midline crossing
ENSDART00000160766	pim1	0.002635727	0.465870139	0.649909313	0.413306503	0.082988542	midline crossing
ENSDART00000160979	jpt1b	0.000229447	0.682460724	1.366219793	1.515562314	0.876953288	midline crossing
ENSDART00000161017	eif3s6ip	0.012456084	0.313830903	0.293421495	0.276651786	0.120270233	midline crossing
ENSDART00000161117	NA	0.052467991	0.194355392	0.331000906	0.496659366	0.20108058	midline crossing
ENSDART00000161244	CR293509.1	5.17084E-05	0.407469749	1.465267423	1.274347742	0.975210426	midline crossing
ENSDART00000161330	col27a1a	0.012339321	0.210670367	0.547410859	0.646435326	0.255665234	midline crossing
ENSDART00000161377	cdk2ap2	0.030614624	0.241317874	0.341753789	0.416424679	0.144968334	midline crossing
ENSDART00000161460	pmt	0.047763341	2.538801298	3.7270331	2.780124527	1.870914111	midline crossing
ENSDART00000161514	CABZ01079986.1	0.000250511	-0.184749182	1.474076268	0.73277167	-0.443845367	midline crossing
ENSDART00000161597	si:ch211-262i1.3	0.034395507	0.726810222	0.918966234	1.284521547	0.120004192	midline crossing
ENSDART00000161613	SLC7A1 (1 of many)	0.002341037	0.777752401	1.137604367	0.924750387	0.450737498	midline crossing
ENSDART00000161869	dysf	0.010359104	1.077492273	1.30463907	1.440346007	1.017975966	midline crossing
ENSDART00000162007	Metazoa_SRP	0.037519793	0.903949119	2.303055473	1.702580761	0.513671528	midline crossing
ENSDART00000162377	FP085414.1	0.001772851	0.809712454	1.275862162	1.389373351	0.791945088	midline crossing
ENSDART00000162722	zgc:153867	0.001130434	0.467799327	0.468299246	0.579069745	0.022677056	midline crossing
ENSDART00000162897	Metazoa_SRP	0.0215019	1.178659737	1.5924133	1.384383775	1.197556527	midline crossing
ENSDART00000163042	si:dkey-16p6.2	0.048041075	0.770521119	0.592959463	0.681550899	0.554585181	midline crossing
ENSDART00000163137	elovl1a	0.000445792	1.359201374	2.32562135	1.99000125	0.940099891	midline crossing
ENSDART00000163174	si:ch73-42p12.2	6.34429E-05	0.635401366	0.972834844	0.669544171	0.150769788	midline crossing
ENSDART00000163243	dpysl4	0.001058776	0.526851432	0.969802663	1.139409914	0.808513895	midline crossing
ENSDART00000163367	TFR2	4.96098E-05	0.892525381	1.842645951	2.060867557	1.063290775	midline crossing
ENSDART00000163370	si:rp71-36a1.2	0.007804676	0.777729217	1.232972386	1.065047677	0.579071639	midline crossing
ENSDART00000163441	limk1a	6.80867E-05	1.134897491	0.940120128	1.036684953	0.715200814	midline crossing
ENSDART00000164207	impdh2	0.000279675	0.582365155	0.675083436	1.006708284	0.417204588	midline crossing
ENSDART00000164210	SI	0.000224957	1.002064064	1.522108486	0.992417222	0.446482404	midline crossing
ENSDART00000164311	mfsd2aa	0.007723957	0.188250738	0.92622451	1.277563791	0.357720797	midline crossing
ENSDART00000164392	lrrc20	0.000250511	0.934799406	1.852088866	1.534338919	1.07983234	midline crossing
ENSDART00000164471	CABZ01084230.2	0.024883139	0.442580477	0.587031258	0.401618345	0.289449509	midline crossing
ENSDART00000164543	kalrna	0.002635555	0.833553496	1.079150473	0.963779963	0.661385395	midline crossing
ENSDART00000165141	elavl4	0.004826213	1.008784658	0.753903544	1.037731482	0.21557706	midline crossing
ENSDART00000165309	pmt	0.000144061	1.560743592	2.051643174	1.965781873	1.006007351	midline crossing
ENSDART00000165572	zgc:162193	0.029001753	0.654407013	0.606984165	0.583589232	0.369009896	midline crossing
ENSDART00000165594	CT030188.1	0.030766044	0.949647945	1.541943795	1.047931527	0.898450066	midline crossing
ENSDART00000165628	il4r.1	0.026868362	2.521820035	2.797213431	2.312532052	1.674117298	midline crossing
ENSDART00000165715	BX284638.1	0.000261206	0.544886732	0.81514457	1.035521411	0.49952411	midline crossing
ENSDART00000165993	f3a	0.000100528	1.175718098	1.946056838	1.259834727	0.15077318	midline crossing
ENSDART00000166040	sh3bp5b	5.24617E-05	0.359354612	1.105816025	1.101214783	0.69319763	midline crossing
ENSDART00000166120	si:ch211-212d10.1	0.005272363	1.398436882	1.472487792	1.228480368	0.254562361	midline crossing
ENSDART00000166259	wars	0.007387753	0.30804143	0.479087994	0.526413933	0.245904587	midline crossing
ENSDART00000166324	ctnnd1	0.02267327	0.460487451	0.641759187	1.004918197	0.346719852	midline crossing
ENSDART00000166432	slc2a8	0.044776229	0.33290564	0.367124099	0.471066456	0.250147846	midline crossing
ENSDART00000166560	hpdl	0.029524772	0.319918962	0.542121436	0.616518616	0.488982996	midline crossing
ENSDART00000166622	si:dkey-77g12.1	0.030464157	1.256692313	2.663525112	1.501030031	-0.081810387	midline crossing
ENSDART00000166992	rab6a	0.003458178	0.863374482	0.610837796	0.548836905	0.594870039	midline crossing
ENSDART00000167035	mibp2	0.000190025	0.52185156	0.897496666	1.068299106	0.608631566	midline crossing
ENSDART00000167062	SNORD49	0.030270278	0.825973685	1.057295844	0.696610061	0.75374548	midline crossing
ENSDART00000167145	ap1ar	0.00427909	0.285895558	0.368048827	0.350688731	0.119518374	midline crossing
ENSDART00000167359	dusp27	0.000892328	1.665789827	1.31173846	1.624125812	1.13571557	midline crossing
ENSDART00000167461	BX897691.1	0.046057741	1.652413387	2.158428192	1.996682668	1.949150718	midline crossing
ENSDART00000167666	dnajc21	0.014542918	0.569585132	0.384796247	0.537599141	0.160116436	midline crossing
ENSDART00000167879	Metazoa_SRP	0.015884799	3.268404605	5.790076	5.563915837	5.137733652	midline crossing
ENSDART00000168083	Metazoa_SRP	0.018792355	1.497191597	1.645034783	1.435853831	0.615781715	midline crossing
ENSDART00000168371	mibp	0.004889638	1.064831288	1.384792727	1.720455753	0.84273718	midline crossing
ENSDART00000168376	Metazoa_SRP	0.031983637	0.77672172	1.175457852	1.304833035	1.047872554	midline crossing
ENSDART00000168419	rsu1	0.004815134	0.608707901	0.588635147	0.668370486	0.283061431	midline crossing
ENSDART00000168497	eef2b	0.002633576	0.448731314	0.547292163	0.7539331	0.364287935	midline crossing

ENSDART00000168610	si:ch211-283g2.2	0.000871179	1.147251625	1.274539863	1.168500693	0.162307024	midline crossing
ENSDART00000168683	lrrc8c	0.039756837	0.403272924	0.771214032	0.877257507	0.389191376	midline crossing
ENSDART00000168902	Metazoa_SRP	0.06078884	0.908254207	1.60749636	1.079432418	0.706761496	midline crossing
ENSDART00000169023	scn1lab	0.043676774	0.230826198	0.448869227	0.411715364	0.200031153	midline crossing
ENSDART00000169052	elovl1b	0.016102005	0.945814486	1.07228669	0.819418014	0.365575121	midline crossing
ENSDART00000169061	CU138533.1	0.004085991	0.641184794	1.068185172	1.17226183	0.773255864	midline crossing
ENSDART00000169663	CT573860.1	0.009406102	0.353646224	0.562802664	0.454063437	0.329757181	midline crossing
ENSDART00000170095	CR388166.2	0.047568271	0.588171845	0.299671021	0.452688856	0.506464888	midline crossing
ENSDART00000170116	uap1	0.041037763	0.399345456	0.560025587	0.455223073	0.418274301	midline crossing
ENSDART00000170222	mibp	0.046085697	1.202449519	1.249757535	1.712700336	0.61416841	midline crossing
ENSDART00000170284	creb3l3l	0.064177665	0.444489468	0.957040221	0.49606022	0.414651857	midline crossing
ENSDART00000170374	si:dkey-38l22.2	5.17084E-05	1.50589782	1.851640662	1.322719067	0.692201779	midline crossing
ENSDART00000170422	si:dkey-19b23.8	0.000292971	0.497209105	1.26444289	1.061660184	0.502612026	midline crossing
ENSDART00000170572	lepa	0.00013528	4.559547685	4.778234325	4.366752593	2.765663683	midline crossing
ENSDART00000170589	mibp	0.028456497	1.067201928	1.476075399	1.501871782	0.758874349	midline crossing
ENSDART00000170671	megf8	0.003012872	0.369502187	0.913401505	1.13324158	0.463058503	midline crossing
ENSDART00000170684	btf3	0.027344176	0.237794169	0.784907824	0.656975875	0.357635511	midline crossing
ENSDART00000170768	lect1	0.055604098	3.805179034	4.274135568	2.696697623	2.532825072	midline crossing
ENSDART00000170865	nme2b.1	0.007383951	0.635993303	0.679448572	0.689008761	0.355584206	midline crossing
ENSDART00000170983	lmnb2	0.03397627	0.185291183	0.299339057	0.275121803	0.118770566	midline crossing
ENSDART00000171113	mibp	0.0245375	1.815496184	1.851942075	2.237510566	0.882565569	midline crossing
ENSDART00000171235	calua	0.003825103	0.528206357	0.591046444	0.528298198	0.210887316	midline crossing
ENSDART00000171343	FP102192.1	0.009511897	1.130120264	0.697435719	1.760333984	0.139610385	midline crossing
ENSDART00000171380	top1mt	0.004985516	0.983277478	1.316957074	1.074308725	0.32139594	midline crossing
ENSDART00000171589	hrasa	0.008728551	0.543862541	0.913653251	0.982614467	0.767663706	midline crossing
ENSDART00000171867	fnbp1l	0.000996442	0.402488238	0.925028089	0.937640279	0.578874881	midline crossing
ENSDART00000171882	CABZ01088567.1	0.0066372	0.407931	0.822027708	0.621405649	0.24977578	midline crossing
ENSDART00000171996	myl12.1	0.017201684	0.233861142	0.344330245	0.260508491	0.06338781	midline crossing
ENSDART00000172215	si:ch211-39i2.2	1.36144E-05	1.017534105	1.848603057	1.465869245	0.571196451	midline crossing
ENSDART00000172372	Metazoa_SRP	0.027491007	1.431390172	1.797550169	1.401344471	0.830250287	midline crossing
ENSDART00000172441	lima1a	0.014437179	0.350368785	0.588496789	0.46589541	0.178939257	midline crossing
ENSDART00000172510	elavl4	0.002554999	1.054519465	1.174247457	1.078840874	0.691066079	midline crossing
ENSDART00000173098	gemin6	0.046727574	0.181263356	0.385271136	0.184325919	0.274013326	midline crossing
ENSDART00000173195	zgc:153146	0.033291702	2.291177749	2.913913042	2.827467346	2.606942928	midline crossing
ENSDART00000173305	si:ch1073-456m8.1	0.012253248	0.206580067	0.587987288	0.667691948	0.271613596	midline crossing
ENSDART00000173436	si:dkey-280e21.3	8.39504E-05	0.740189589	1.373516231	1.387529232	0.976737711	midline crossing
ENSDART00000000486	cntn2	6.48692E-06	0.015280519	0.733067848	1.819345027	1.713832915	target selection
ENSDART00000002393	rundc3aa	0.000184957	-0.355283185	0.971542583	1.209230568	0.944629469	target selection
ENSDART00000002961	rcor2	0.001353924	-0.353333584	0.726682254	0.762799057	0.664353552	target selection
ENSDART00000003745	vim	0.015014385	0.110317427	0.81438402	0.975636783	0.646819018	target selection
ENSDART00000003998	ewsr1b	0.006990173	0.136063726	0.084077199	0.439371598	0.241694854	target selection
ENSDART00000004622	sf3b4	0.02954888	0.222493415	0.286225027	0.527062646	0.243398323	target selection
ENSDART00000005337	rimkla	0.001648434	-0.133054455	0.30447461	0.568720788	0.413212633	target selection
ENSDART00000005366	tpd52l2b	0.046265136	0.128420316	0.410964244	0.59220548	0.186138167	target selection
ENSDART00000005453	chd4a	0.006932921	0.299643374	0.423708585	0.655765013	0.391160008	target selection
ENSDART00000005496	kctd9b	0.010839093	0.161429839	0.406047159	0.541753399	0.319048316	target selection
ENSDART00000005609	kifap3a	0.003425348	0.203573738	0.628570592	0.743430197	0.605809471	target selection
ENSDART00000005720	stat1a	0.010250585	0.035880276	0.045195542	0.615137639	0.020671748	target selection
ENSDART00000006085	cbl	0.034881046	0.165119386	0.1775588	0.379174787	0.206196339	target selection
ENSDART00000006489	acsl4a	0.000400742	0.116872844	1.158097412	1.477099844	0.59458295	target selection
ENSDART00000006724	smarcd3b	0.00033575	-0.214336649	0.06424201	0.499562006	0.504466248	target selection
ENSDART00000006778	acat2	0.003807479	-0.122422954	0.489045334	0.688039864	0.280940334	target selection
ENSDART00000008038	sulf2a	1.1419E-05	0.420520728	1.274767381	2.360438199	2.491815243	target selection
ENSDART00000009194	aimp2	0.028420674	0.035867833	0.204745928	0.516008081	0.307277143	target selection
ENSDART00000009277	tuba1a	0.000180202	0.466164103	1.394077049	1.773814961	1.31379942	target selection
ENSDART00000010140	igf2bp3	0.01196819	0.357904839	0.436869624	1.38352682	1.118120985	target selection
ENSDART00000010144	pvalb2	0.002147221	0.379417352	4.690875982	5.447468716	3.629116223	target selection
ENSDART00000010274	dpysl5a	0.000114847	0.119382655	1.052869734	1.414498731	1.230769727	target selection
ENSDART00000010495	znrf1	0.011135896	0.045008698	0.17288769	0.379569247	0.252907322	target selection
ENSDART00000011224	itga10	0.008920935	2.439207816	3.888845597	4.302533523	3.055176776	target selection
ENSDART00000011568	syngr3a	0.003678598	-0.064295047	0.234968681	0.63147157	0.633880469	target selection
ENSDART00000011865	sec23b	0.002343787	-0.409405473	0.102005828	0.534469651	0.138588714	target selection
ENSDART00000012247	dhcr24	0.030292962	2.392110544	2.697063662	3.119814675	2.653468904	target selection
ENSDART00000012256	tnni2a.3	0.004454925	0.628164776	3.762159614	4.672246791	3.241031276	target selection
ENSDART00000013605	zbtb20	0.00075553	0.021191929	0.070670156	0.462529396	0.345025042	target selection
ENSDART00000013785	insig1	0.003330818	-0.148803263	0.843708665	1.082868772	0.366540247	target selection
ENSDART00000013961	mycla	0.015752787	0.091841991	0.503579515	0.855574629	0.294011787	target selection
ENSDART00000014806	npas2	0.005305184	-0.341343772	0.009000027	0.555800516	0.156695025	target selection
ENSDART00000014922	arhgap22	0.004853289	0.337853511	0.496203573	0.771443869	0.664153347	target selection
ENSDART00000015040	hrasb	0.001609185	-0.035400047	0.468215746	0.71708775	0.509094476	target selection
ENSDART00000015286	ankrd13b	0.001076713	-0.294042347	0.149598935	0.484140903	0.605167909	target selection
ENSDART00000015632	nkain1	0.000894529	0.221220236	0.815235139	1.176280245	0.965286214	target selection
ENSDART00000015979	farsb	0.041085489	0.087018819	0.265294461	0.405710239	0.211888538	target selection
ENSDART00000016303	irx2a	4.96098E-05	-0.381386746	0.064410059	0.752093904	0.989751717	target selection
ENSDART00000016360	si:ch73-199e17.1	0.028708281	2.690029092	3.363503415	3.69470671	3.529091333	target selection

ENSDART00000016370	dirc2	0.000512152	0.134905484	0.442850685	0.565287267	0.464631841	target selection
ENSDART00000016597	nfkbiab	0.038559823	0.207474067	0.316720279	0.269395099	0.411969915	target selection
ENSDART00000016710	scrn3	0.001328724	-0.247629441	-0.171036354	0.44401532	-0.052873668	target selection
ENSDART00000016860	ppp2r1bb	0.015469128	0.018499492	0.284521267	0.321334616	0.193996267	target selection
ENSDART00000017299	tdg.1	0.043370331	0.102853523	0.194463285	0.348171907	0.196594799	target selection
ENSDART00000018150	neurod6b	0.012342358	0.484226112	1.695956927	1.935318885	1.555476315	target selection
ENSDART00000018498	helz2	0.00076674	-0.063748361	-0.192163226	1.151139617	0.013207133	target selection
ENSDART00000018735	dnaja2l	0.000177237	-0.20745831	0.012628814	0.350147509	0.311991137	target selection
ENSDART00000019045	ebp	0.003165422	-0.186359255	0.947675675	1.363611199	0.55228711	target selection
ENSDART00000019199	rab39ba	0.013286981	-0.125963686	0.3041991	0.44382958	0.206562916	target selection
ENSDART00000019617	rsad2	0.008246433	-0.056061565	-0.489318095	2.739834278	-0.378754222	target selection
ENSDART00000019658	nacad	8.67551E-05	-0.254606168	0.44010123	0.744298293	0.29398429	target selection
ENSDART00000019925	GNB4	0.000718633	0.24039241	0.43881347	0.966289057	1.074168682	target selection
ENSDART00000020122	ywhah	0.000166651	-0.243048068	-0.041853394	0.44002204	0.422069749	target selection
ENSDART00000020621	mapk4	0.001185711	-0.137443479	0.345820304	0.800085717	0.474998646	target selection
ENSDART00000020970	pgm2	0.021002038	0.182379442	0.224260514	0.427220163	0.369397058	target selection
ENSDART00000021341	kif3ca	0.001627738	0.127548246	0.488322423	0.730937873	0.492487971	target selection
ENSDART00000021693	ank2a	0.031838485	0.088061869	0.283890991	0.488712819	0.41975176	target selection
ENSDART00000022270	arhgap33	0.034698678	0.060975731	0.144058893	0.360327899	0.271763038	target selection
ENSDART00000022909	klhl18	0.00115719	-0.153412936	0.073357396	0.397749012	0.249762723	target selection
ENSDART00000023278	fads2	0.00304733	-0.280275959	0.609167416	1.126645574	0.470269511	target selection
ENSDART00000023944	lmnl3	0.001714837	-0.134674249	0.538751787	0.920098245	0.528719758	target selection
ENSDART00000023959	arntl1a	7.43514E-05	0.340904931	1.030627189	1.45255612	0.975945139	target selection
ENSDART00000024135	tubb2	7.10385E-05	-0.097026879	1.085064176	1.27895467	1.027950073	target selection
ENSDART00000025852	tnni2b.1	0.000911246	-0.577234038	4.833234821	5.215670785	3.899605393	target selection
ENSDART00000026800	kifap3b	0.001775429	0.203180282	0.588115089	0.640508934	0.450843691	target selection
ENSDART00000026992	sox4a	3.6604E-05	0.168735758	0.553498244	0.871364123	0.630648218	target selection
ENSDART00000027158	psmd3	0.034155477	0.079773681	0.202742016	0.46801517	0.212038089	target selection
ENSDART00000027379	bicral	0.012896166	0.180958951	0.201052129	0.429766407	0.252075745	target selection
ENSDART00000027598	tpm3	0.022054847	0.692886998	2.316316343	2.715886041	1.570432549	target selection
ENSDART00000027718	fxr2	0.020107844	0.070260526	0.251047986	0.370057736	0.114198992	target selection
ENSDART00000027758	rtn1b	0.000135341	-0.626628578	0.207141964	0.617559397	0.433726227	target selection
ENSDART00000028607	chd6	0.008909863	0.00488027	-0.079639497	0.533277243	0.378096362	target selection
ENSDART00000029121	usp5	0.005099354	0.186533139	0.264124698	0.696851044	0.548402162	target selection
ENSDART00000031470	pafah1b1b	0.001868725	-0.06625793	0.19164974	0.422888398	0.388967525	target selection
ENSDART00000033724	fabp3	0.001967837	0.043357927	0.829727862	0.980692721	0.446444515	target selection
ENSDART00000034216	dync1h1	0.038721816	0.15631552	0.099035755	0.755817679	0.554910929	target selection
ENSDART00000034914	pvalb3	0.006583055	-0.375270417	3.922610155	4.415489459	3.238850832	target selection
ENSDART00000035245	spire2	9.16648E-05	0.223570717	0.667119992	0.874236758	0.626613708	target selection
ENSDART00000035409	zc2hc1a	0.000588633	0.100393521	0.424633369	0.532716266	0.492034687	target selection
ENSDART00000036926	vangl1	0.000398071	-0.329590873	-0.033233324	0.383553313	0.3451937	target selection
ENSDART00000038391	pkz	0.008061109	0.411244814	0.129537507	1.595328762	0.021626224	target selection
ENSDART00000040557	CRIP2 (1 of many)	0.000349374	-0.132882678	0.562285777	0.768710088	0.441120913	target selection
ENSDART00000042134	dock7	0.014840867	0.063646619	0.209946461	0.48316367	0.274325565	target selection
ENSDART00000042162	tm7sf2	0.008621509	0.1050645	0.758038931	0.952561873	0.4137868	target selection
ENSDART00000042218	pafah1b1a	0.024122965	0.049322983	0.127134272	0.313096774	0.220746894	target selection
ENSDART00000042255	rab6bb	0.000105127	0.249992007	1.135776594	1.498200114	1.307756366	target selection
ENSDART00000043932	atp2a1	0.046659681	1.138744452	4.285725335	5.558096993	3.994336477	target selection
ENSDART00000044000	plxna3	0.000326965	0.522678231	0.518528602	0.862237135	0.748340859	target selection
ENSDART00000044057	43346	0.00053559	-0.17670354	0.196164293	0.478730018	0.43476862	target selection
ENSDART00000044453	ano5a	0.00350062	-0.027551778	0.223592536	0.548330121	0.427829524	target selection
ENSDART00000044949	syt16	4.96098E-05	-0.001457693	-0.053066568	0.72824965	0.634879457	target selection
ENSDART00000045391	srgap2	0.005041829	0.042169336	0.118632088	0.377794988	0.307955564	target selection
ENSDART00000045410	thy1	8.488E-05	0.906274294	2.922050483	2.917431913	2.440516966	target selection
ENSDART00000046678	pak2b	0.02432669	0.067537468	0.265866932	0.367938524	0.197303091	target selection
ENSDART00000046933	sult1st5	0.009503355	0.218426293	0.869949792	0.854237199	0.702293309	target selection
ENSDART00000047082	gdap1l1	0.00058454	-0.237605119	0.285676986	0.669438597	0.59731396	target selection
ENSDART00000047143	specc1	0.039415449	0.250947915	0.397558549	0.627196335	0.309975843	target selection
ENSDART00000047409	myh14	0.014779327	0.151853201	0.372826224	0.643058289	0.555183408	target selection
ENSDART00000048110	six4b	0.002795018	0.757934328	2.322346312	2.776043581	1.88012445	target selection
ENSDART00000049194	gpr37b	2.18017E-05	-0.562015213	0.012994785	0.671207906	0.757619967	target selection
ENSDART00000051231	gnb2	0.002814147	-0.235263329	0.124417594	0.575032632	0.495502612	target selection
ENSDART00000051234	tnika	0.001841908	0.106489865	0.189584243	0.309030435	0.511149463	target selection
ENSDART00000051697	evla	0.003779087	-0.055749697	0.354222037	0.437976697	0.322467243	target selection
ENSDART00000052124	fam49al	0.000100528	-0.409057809	-0.177161374	0.563238983	0.47605027	target selection
ENSDART00000052511	hnrnpa0l	0.02613431	0.098563881	0.214926285	0.391405743	0.211573162	target selection
ENSDART00000053284	bcl9	0.003126045	-0.057531558	0.216352129	0.432379429	0.260365529	target selection
ENSDART00000053869	slc44a2	0.000322013	0.03477078	0.96666703	1.3453814	0.899279007	target selection
ENSDART00000054664	tnnc1b	0.019014873	-0.083935925	3.176389417	3.430200861	1.884280525	target selection
ENSDART00000054691	uba1	0.014095383	0.10035699	0.287144805	0.420196482	0.237625478	target selection
ENSDART00000055340	fus	0.000159901	-0.011490396	0.383733399	0.662780299	0.416441518	target selection
ENSDART00000055380	tubb5	2.92142E-06	2.169554793	3.899556169	4.316772272	3.57490014	target selection
ENSDART00000055756	tbc1d12a	0.00448854	-0.033893992	0.095679716	0.342628218	0.232107662	target selection
ENSDART00000056286	h1f0	0.001739475	-0.326358805	0.273543898	0.626230484	0.593578182	target selection
ENSDART00000056544	tox4a	0.02114031	0.131870382	0.296259606	0.350040714	0.249645538	target selection

ENSDART00000056795	hectd3	0.004611386	0.187585997	0.34309988	0.435708832	0.360997812	target selection
ENSDART00000057584	slc1a4	0.00134801	0.291595567	1.233259249	1.405286425	1.089173123	target selection
ENSDART00000058574	NA	0.032955534	3.231975259	2.942133944	3.360676782	3.717330017	target selection
ENSDART00000059586	spegb	4.52852E-05	0.60633626	1.046592342	1.773590673	1.534849845	target selection
ENSDART00000059756	ralba	0.014582204	0.050810042	0.323679093	0.529596851	0.259207353	target selection
ENSDART00000060561	csdc2a	0.006063577	-0.084744858	0.609682067	0.574951056	0.285883083	target selection
ENSDART00000060766	rab11a	0.001380734	0.179968247	0.479437298	0.674000894	0.468733557	target selection
ENSDART00000061417	si:ch211-245h14.1	0.00918017	0.537591116	0.459433616	1.387404638	-0.014689392	target selection
ENSDART00000061470	mtss1la	0.002100653	-0.342228031	0.053961203	0.113929451	0.018495568	target selection
ENSDART00000061523	il17a/f3	0.017627165	0.228114616	0.421937628	0.621201213	0.769032572	target selection
ENSDART00000061955	myl13	0.006605056	0.400602979	3.991209963	4.290518336	2.830575983	target selection
ENSDART00000062551	cyp51	0.0002611	0.018772193	0.850105191	1.080881624	0.554137375	target selection
ENSDART00000062560	zgc:77784	0.054165556	0.130172136	0.171187774	0.614861949	0.368542156	target selection
ENSDART00000063938	mast1a	0.000314801	0.148358744	0.555318149	1.032634027	1.008672267	target selection
ENSDART00000065097	dpysl3	0.000311162	-0.247117182	0.32883927	0.696704704	0.442557487	target selection
ENSDART00000065600	sc5d	0.009204947	-0.12560715	0.619273704	0.802648489	0.437253012	target selection
ENSDART00000066177	tuba2	7.26748E-05	-0.341980556	0.290211936	0.695639518	0.704284087	target selection
ENSDART00000066391	csnk1e	0.000593122	0.01172114	0.595315231	0.792117229	0.433182971	target selection
ENSDART00000066975	impdh1b	0.000512556	0.195914466	0.339716801	0.56808711	0.477613078	target selection
ENSDART00000067066	parp6b	1.45694E-05	-0.443166413	0.101161009	0.402103535	0.516387314	target selection
ENSDART00000067327	abhd2b	0.0021138	0.159640353	0.698443956	0.611964225	0.750739323	target selection
ENSDART00000073583	islr2	0.000634488	0.313523936	1.12325114	1.332162091	1.179316934	target selection
ENSDART00000074924	mbnl1	0.094159425	0.32889023	0.277718787	0.642145523	0.211541009	target selection
ENSDART00000074979	rnft2	0.02083117	0.06664962	0.449659345	0.588779493	0.279626887	target selection
ENSDART00000075112	clvs2	0.003104562	0.165980729	0.346379383	0.75563827	0.535875063	target selection
ENSDART00000075260	inab	2.82217E-05	-0.558694355	0.275313434	1.165237079	1.111612471	target selection
ENSDART00000075331	insm1b	0.009480138	0.180818368	0.558805202	0.852549514	0.856981121	target selection
ENSDART00000075743	eprs	0.03427447	0.181617497	0.208060281	0.357036503	0.248273691	target selection
ENSDART00000075808	apbb3	0.002539213	-0.025817957	0.161567226	0.465828121	0.45985711	target selection
ENSDART00000075902	klhl43	0.004256148	1.602362948	3.319039146	3.627754288	3.174379945	target selection
ENSDART00000076506	wisp1a	0.025227573	0.075916017	0.908100257	1.435223818	0.542654062	target selection
ENSDART00000076574	rtn1a	0.00434437	-0.132166015	0.374408359	0.606191338	0.476970779	target selection
ENSDART00000077215	ppp2r5b	0.002524433	0.185911454	0.229029828	0.615811771	0.611674376	target selection
ENSDART00000077459	smyd2a	0.042433381	0.109333535	0.326579335	0.423001533	0.365696401	target selection
ENSDART00000077539	tuba1c	0.00109551	-0.211130957	0.180769664	0.840172721	0.834422327	target selection
ENSDART00000078137	ankrd54	0.034178169	0.051281727	0.23783078	0.382868701	0.32135773	target selection
ENSDART00000078277	msmo1	0.001153812	0.087631699	1.109674669	1.47165822	0.852850391	target selection
ENSDART00000078334	celsr3	0.002605308	0.469496099	0.512562938	0.74195357	0.599858827	target selection
ENSDART00000078771	SBK1	0.00198437	-0.390774004	0.13684565	0.923517231	0.784697628	target selection
ENSDART00000079235	cd99l2	0.000613723	-0.167082345	0.448795434	0.91020128	0.864265064	target selection
ENSDART00000079283	tmeff1b	0.001133915	0.060962109	0.679659218	0.971148806	0.712213835	target selection
ENSDART00000079559	ifit16	0.03326116	0.716638095	0.325468397	2.311988055	-0.283531995	target selection
ENSDART00000079711	slc25a1a	0.000122706	0.025053389	0.916416559	1.283110285	0.925187833	target selection
ENSDART00000079778	ifit8	0.035967167	1.432186723	0.097505141	4.166007884	0.96250098	target selection
ENSDART00000080033	SSBP2 (1 of many)	0.034092146	0.123939988	0.461173497	0.600467157	0.3572706	target selection
ENSDART00000080042	rab33a	0.001631005	-0.045588719	0.341497657	0.630733663	0.503197845	target selection
ENSDART00000080256	nefma	1.75467E-05	0.572074143	2.009260502	2.281701505	1.529767044	target selection
ENSDART00000080289	prrc2c	0.035944076	0.060093623	0.047472332	0.306363857	0.189281148	target selection
ENSDART00000080602	map7d2b	0.001304993	0.243598476	0.831190471	1.239754477	0.898189276	target selection
ENSDART00000081059	rps6kb1b	0.042381041	0.156747941	0.167680861	0.344534054	0.174989656	target selection
ENSDART00000081092	si:dkeyp-77h1.4	0.000199258	-0.383994825	0.24121529	0.967657356	0.94823494	target selection
ENSDART00000081223	krt5	0.004002952	2.400508858	4.729984226	4.862312842	5.813921541	target selection
ENSDART00000081272	gcn1	0.02181186	0.168820856	0.100284898	0.363305756	0.2232516	target selection
ENSDART00000081601	cept1a	0.020882958	0.150762086	0.296977372	0.445683893	0.275050142	target selection
ENSDART00000082944	dock6	0.004360669	0.129469289	0.283374744	0.379764071	0.290753034	target selection
ENSDART00000083002	map1aa	0.00881795	0.023824463	0.057494175	0.8302085	0.470304447	target selection
ENSDART00000083212	fscn1a	0.001033408	-0.172521555	0.403697769	0.865067524	0.635846942	target selection
ENSDART00000083605	tbc1d25	0.020097848	0.094218097	0.226970926	0.332123969	0.140604089	target selection
ENSDART00000083628	ddit3	0.002192622	-0.053032847	0.505000026	0.598711637	0.25299651	target selection
ENSDART00000083797	TBC1D9B	0.000642503	0.273448063	0.469124554	0.581828184	0.40676136	target selection
ENSDART00000084819	arhgap35b	0.001659006	0.101645005	0.291379647	0.535032879	0.462050203	target selection
ENSDART00000085230	atl1	0.003020568	-0.283512177	0.138626411	0.619353197	0.442930265	target selection
ENSDART00000085263	SELENOI	0.02776376	0.036305982	0.149651758	0.460720877	0.164646013	target selection
ENSDART00000085319	sos2	0.038217845	0.062155314	0.173737983	0.464042198	0.208637802	target selection
ENSDART00000085388	bmp3	0.004205562	-0.146774305	1.240822845	1.494378971	0.416428855	target selection
ENSDART00000085453	cluhb	5.72005E-05	-0.484145834	-0.430773206	0.678836708	-0.017745757	target selection
ENSDART00000085684	ttll11	0.000375747	-0.16810055	0.020727161	0.348458395	0.408691778	target selection
ENSDART00000085719	si:ch211-10a23.2	0.001067718	0.141906082	0.927202925	1.15606105	0.59243708	target selection
ENSDART00000086301	irge4	0.015651339	0.875091617	0.681146857	1.737500297	0.803175592	target selection
ENSDART00000086333	jarid2a	0.000531966	-0.109318405	0.11715985	0.373609719	0.35216542	target selection
ENSDART00000086409	dync1i1	0.002016009	-0.021724393	0.427961112	0.551451734	0.438467635	target selection
ENSDART00000086434	tmcc2	0.000793313	0.022092982	0.338134833	0.850580716	0.840264518	target selection
ENSDART00000086537	NA	0.022370255	0.422133332	0.379254853	0.651471983	0.472158305	target selection
ENSDART00000086946	mov10b.1	0.016426365	0.41644893	0.305142124	1.89370555	0.04709269	target selection
ENSDART00000087105	myom2a	0.029772573	2.177324505	3.608086766	3.587707417	2.779461648	target selection

ENSDART00000087107	eif4g1a	0.01673556	0.252655247	-0.051267066	0.413833734	0.330964133	target selection
ENSDART00000087115	rims1b	0.002016009	-0.155072254	0.041830315	0.378907446	0.287621284	target selection
ENSDART00000087204	dusp3a	0.000778506	-0.327203373	-0.053264752	0.592884224	0.622404395	target selection
ENSDART00000088364	kif1aa	0.000306344	-0.06544832	0.34113245	0.707853532	0.648494399	target selection
ENSDART00000088653	prss12	0.001247242	-0.641371677	0.068962263	0.215098805	0.069158882	target selection
ENSDART00000089076	dot1l	0.009173096	0.094529933	0.165735937	0.448240264	0.463978672	target selection
ENSDART00000089079	mpnd	0.001644836	0.049906779	0.784892334	0.951903848	0.691834891	target selection
ENSDART00000089141	fsd1	0.000907455	0.140911421	0.537063785	0.66216846	0.489473142	target selection
ENSDART00000089445	agap1	0.028468759	0.111728293	0.12652166	0.321279752	0.223152778	target selection
ENSDART00000090596	fgf12b	0.000227894	-0.346267349	-0.034488788	0.704892235	0.660670447	target selection
ENSDART00000090748	pcdh1g9	0.004897476	0.151679401	0.129768355	0.681696549	0.559518569	target selection
ENSDART00000090874	kcnh7	0.000589961	0.089634054	0.066677874	0.800119794	0.739010611	target selection
ENSDART00000091241	si:ch73-22o12.1	0.003818266	-0.053659471	0.086463787	0.414689493	0.463272551	target selection
ENSDART00000091644	abi1b	0.016742251	0.181595456	0.359192672	0.565695572	0.32038751	target selection
ENSDART00000092270	r3hdm4	0.003016249	0.109330042	0.341009284	0.430965659	0.322117566	target selection
ENSDART00000092690	srebf2	0.003411856	-0.007030288	0.441941306	0.773565631	0.436893785	target selection
ENSDART00000092948	peli1b	0.025378177	0.088983174	0.130189996	0.342697853	0.254903056	target selection
ENSDART00000098057	ftr19	0.021658197	0.135898929	0.299355243	0.876453981	-0.013001669	target selection
ENSDART00000098284	ftr14	0.009877168	0.428599203	0.304151818	1.741077228	-0.09285898	target selection
ENSDART00000099977	mex3c	0.051405603	0.161233198	0.169039948	0.509657606	0.094152947	target selection
ENSDART00000100310	dbn1	0.015825859	0.014767176	0.221674025	0.550020524	0.324902581	target selection
ENSDART00000100332	fgf12b	0.00038813	-0.220479512	0.029676005	0.518232207	0.657400596	target selection
ENSDART00000100619	zgc:158803	0.028599369	0.343521186	0.388812734	0.518620429	0.45576524	target selection
ENSDART00000101038	tmie	0.000195011	0.304653896	1.158765103	1.299122704	1.103412502	target selection
ENSDART00000101282	bcr	0.005519422	0.172989029	0.500540591	0.708792261	0.385177654	target selection
ENSDART00000101537	mex3b	0.000847723	-0.20648165	0.480244705	0.713185315	0.514833911	target selection
ENSDART00000101789	flot2b	0.000781013	0.047673595	0.464453756	0.590584487	0.461504068	target selection
ENSDART00000102011	G3BP2 (1 of many)	0.015925333	0.183053703	0.292940696	0.414353657	0.391197687	target selection
ENSDART00000102212	tdp2a	0.010054985	1.019634105	0.831370615	2.830207945	0.310574015	target selection
ENSDART00000102305	cspg5a	9.03292E-05	-0.238742988	0.181941504	0.751580581	0.677972326	target selection
ENSDART00000102384	sesn2	0.029981898	0.221656214	0.707312613	0.831938385	0.650662976	target selection
ENSDART00000102520	palm1a	0.000475692	0.447081887	0.96607967	1.214937327	0.924772733	target selection
ENSDART00000102539	st8sia5	0.002696406	0.153973405	0.592846857	1.061043515	0.777183018	target selection
ENSDART00000102665	aste1a	0.002793888	0.478587949	0.328068557	2.324198666	0.218780766	target selection
ENSDART00000102712	tgm2a	0.024073602	0.064390317	0.421175856	0.197637738	0.730671181	target selection
ENSDART00000102715	tuba8l3	0.000489924	1.032352614	1.505768874	1.918452626	1.697934809	target selection
ENSDART00000102868	etnk2	0.002660549	-0.119773861	0.158574082	0.41890552	0.249052751	target selection
ENSDART00000103588	mxa	0.022415732	0.174383297	-0.012993595	1.447594397	0.019311963	target selection
ENSDART00000103622	irf7	0.002828188	-0.049332142	-0.2878598	1.533925917	-0.275107455	target selection
ENSDART00000103815	stmn2a	4.96098E-05	0.140137036	1.176009379	1.538144395	1.355356024	target selection
ENSDART00000103922	atat1	0.015462868	0.145026536	0.665460259	0.937418253	0.683690675	target selection
ENSDART00000104299	cnp	1.98934E-05	0.814960421	1.713687269	2.20652149	1.77606709	target selection
ENSDART00000104336	tnnc2	0.0274914	0.703924131	2.455705453	2.232729877	1.561721699	target selection
ENSDART00000104478	fxr1	0.013415803	0.018053797	0.098113697	0.377704225	0.105513892	target selection
ENSDART00000104523	arntl1b	0.000255127	-0.158144123	0.59291526	1.113237486	0.52944604	target selection
ENSDART00000104720	si:ch1073-385f13.3	0.037756042	0.258133799	0.237616235	1.745458762	-0.119672944	target selection
ENSDART00000104730	ifit14	0.018324408	0.457651023	0.403391968	2.518119653	0.537556431	target selection
ENSDART00000105405	cirbpb	0.023596757	0.001082658	0.185977938	0.46521688	-0.018730312	target selection
ENSDART00000105694	adprm	0.001249299	0.302167589	0.521514455	0.908630272	0.271324554	target selection
ENSDART00000105873	cry4	0.001698814	-0.404336974	0.122755184	0.671823255	0.515326773	target selection
ENSDART00000106120	ywhag2	0.014767815	0.097311782	0.334968358	0.584303472	0.612363287	target selection
ENSDART00000108796	stox2b	0.00058454	0.143615187	0.758742406	1.120462456	0.794663921	target selection
ENSDART00000109288	myo16	0.003478587	0.115485168	0.089369719	0.360073499	0.534996054	target selection
ENSDART00000109615	tmem255a	0.000634043	0.198392818	0.721353886	0.92551731	0.71998369	target selection
ENSDART00000109750	rap2aa	0.015651953	0.293487605	0.482823125	0.662590656	0.396980886	target selection
ENSDART00000109973	tp53bp1	0.000154883	0.061139928	0.539512942	0.657824217	0.222950524	target selection
ENSDART00000110512	ybx1	0.014352201	0.187678935	0.306636132	0.543417204	0.157462635	target selection
ENSDART00000110696	mxb	0.001413354	-0.714840475	-2.62990329	3.629462021	-0.04990443	target selection
ENSDART00000110964	bag6	0.014908663	0.132774646	0.150367292	0.354477336	0.199439987	target selection
ENSDART00000110994	sqlea	0.000140513	0.510664466	1.875874264	2.415595993	1.866281513	target selection
ENSDART00000111278	sorcs2	0.000322759	-0.061568527	0.240045859	0.726875042	0.744539967	target selection
ENSDART00000111303	rfx7	0.00161581	0.075232989	0.085195342	0.459783838	0.727409902	target selection
ENSDART00000111454	UBA6	0.018326914	-0.095626975	0.120665542	0.496662318	0.328619297	target selection
ENSDART00000111475	pcdh7a	0.008009641	0.109274008	0.268985487	0.465377244	0.354118271	target selection
ENSDART00000111480	bcorl1	0.017081311	0.013917394	0.173170994	0.344992278	0.232054818	target selection
ENSDART00000111561	zmp:0000000735	0.000689533	0.220285812	0.110420777	1.549961519	0.06406158	target selection
ENSDART00000111641	morn4	0.00308528	0.104472779	0.38320597	0.615725038	0.556227667	target selection
ENSDART00000111671	mri1	0.001380734	1.003264298	0.870246845	1.443034807	1.04475049	target selection
ENSDART00000111748	pcdhb	0.000529346	0.251557806	0.39162054	0.85552916	0.748922663	target selection
ENSDART00000112032	ARHGAP22 (1 of many)	0.001967837	0.396989069	0.894459004	1.264783984	1.011214691	target selection
ENSDART00000112075	NA	0.034693937	0.743523521	0.909798042	1.147105824	0.978802209	target selection
ENSDART00000112312	lrch2	0.015854118	0.101952948	0.206054969	0.497048672	0.404162087	target selection
ENSDART00000112313	wdr43	0.033970998	2.967338106	2.621161906	3.405726008	2.58425126	target selection
ENSDART00000112438	si:ch73-335m24.5	0.002769129	-0.12977304	-0.024649938	0.356284768	0.481799029	target selection
ENSDART00000112460	dolk	0.02235203	0.234400054	0.274257795	0.504999751	0.504335897	target selection

ENSDART00000112598	otud4	0.006973136	0.000834797	0.118841749	0.673957038	0.019221004	target selection
ENSDART00000112694	fam171a2b	0.001676296	-0.001835186	0.12088862	0.541529951	0.657778888	target selection
ENSDART00000112728	tmem175	0.004215641	-0.092618029	0.36107371	0.426939782	0.221686367	target selection
ENSDART00000112883	fmnl2b	0.000169466	-0.095811604	0.162122972	0.557976718	0.595265469	target selection
ENSDART00000113087	hmx1	0.000694227	-0.389387565	0.021377927	0.234018679	0.270888749	target selection
ENSDART00000113097	hsd17b7	0.001028061	-0.488876774	0.314769091	0.517151777	0.148788215	target selection
ENSDART00000113418	igsf3	0.023363274	0.097020516	0.19405944	0.607620623	0.491282072	target selection
ENSDART00000114919	si:dkey-85k7.12	0.012263789	0.382806755	-0.226494664	2.101494432	-0.132340955	target selection
ENSDART00000115023	PARG	0.014732696	0.013352577	0.067569536	0.312910159	0.18617429	target selection
ENSDART00000121837	efs	0.000534872	0.29165502	0.378275961	0.750399498	0.505660243	target selection
ENSDART00000121981	smarce1	0.04263061	0.060968412	0.140256121	0.341840216	0.044607056	target selection
ENSDART00000121984	ssbp3b	0.013312578	-0.092095029	0.005321722	0.831022729	0.629434393	target selection
ENSDART00000122133	mkrn2os.1	0.028489994	0.117189294	0.106487037	0.442579902	0.198380082	target selection
ENSDART00000122170	smc5	0.003077552	0.002327719	-0.01045929	0.55091638	0.063311494	target selection
ENSDART00000122681	CR848841.1	0.000177237	0.726082565	1.593466942	1.807590182	1.450515621	target selection
ENSDART00000122700	tenm3	0.01323139	0.157516404	0.267363723	0.981020214	1.113087141	target selection
ENSDART00000122742	rorcb	0.000390105	-0.773981248	0.298893511	0.803866913	0.643975269	target selection
ENSDART00000122803	usp9	0.023717938	0.139967851	0.094990222	0.369664537	0.232490119	target selection
ENSDART00000122889	myhz1.3	0.011565935	0.065159767	3.686062072	4.139837605	2.765333679	target selection
ENSDART00000122891	tmem97	0.01179775	0.209697033	0.237651064	0.43725776	0.414256644	target selection
ENSDART00000122924	CABZ01089151.1	0.020552635	0.66835416	0.574150269	1.297855118	0.40982894	target selection
ENSDART00000123063	fgf18a	0.006439631	0.196370642	0.632761225	0.668396752	0.413800422	target selection
ENSDART00000123136	FP236812.4	0.090688964	0.346817394	0.374427758	0.928731714	0.426600457	target selection
ENSDART00000123338	kif3a	0.021156666	0.128582487	0.488909245	0.490973182	0.361344362	target selection
ENSDART00000123518	tuba1b	0.00416486	0.343846477	0.74266829	1.086564506	0.883480954	target selection
ENSDART00000123590	si:ch211-91p5.3	0.006291033	0.174508046	-0.021173312	0.906803099	-0.35666968	target selection
ENSDART00000123607	cyfip2	0.009681558	0.234079405	0.294840867	0.917405124	0.783474958	target selection
ENSDART00000123648	pcdh1a3	6.48692E-06	-0.182322903	0.28663259	1.138716215	1.048655391	target selection
ENSDART00000124040	insm1a	1.45694E-05	0.50962443	1.551047216	1.738068624	1.104992333	target selection
ENSDART00000124085	usp18	0.007028791	0.058285571	0.001224867	0.967078356	-0.263223461	target selection
ENSDART00000124220	nptxrb	2.18017E-05	-0.324541852	-0.000122151	0.687818829	0.894343302	target selection
ENSDART00000124346	fn1a	0.000390105	1.458758212	2.264590948	2.662817487	2.142590168	target selection
ENSDART00000124440	CABZ01029822.1	0.001076608	0.125194338	0.049659662	0.670452016	0.712162088	target selection
ENSDART00000124485	pcdh2ab8	0.036614379	0.093884444	0.182265582	0.466276394	0.290720698	target selection
ENSDART00000125281	ngfra	0.001456887	-0.140591626	0.234351775	0.618758909	0.552257695	target selection
ENSDART00000125590	adam22	0.000133356	-0.13203003	0.438612977	0.776065364	0.829224171	target selection
ENSDART00000126029	dhx58	0.002259461	0.173247162	-0.097510604	2.186112068	0.050096401	target selection
ENSDART00000126148	zmp:0000000912	0.027039876	0.164571908	0.167838799	1.993878391	0.463546785	target selection
ENSDART00000126253	fam124b	0.007297303	-0.802465606	0.105938283	0.208441414	0.082147827	target selection
ENSDART00000126299	gdi1	0.001249299	-0.113565456	0.226089497	0.711067572	0.636383115	target selection
ENSDART00000126542	ppp1r14ba	6.23756E-05	0.329398236	1.23193061	1.655895613	1.416335991	target selection
ENSDART00000126588	rac3a	0.001030626	-0.258496234	0.207987177	0.575325222	0.581016326	target selection
ENSDART00000126873	myhz2	0.000888975	-2.902978535	4.975016398	5.62978424	4.2721239	target selection
ENSDART00000126991	ascl1b	0.003805801	0.538037435	0.289776062	1.627554047	-0.154081652	target selection
ENSDART00000127214	tmtc4	0.003138731	-0.125359905	0.4024138	0.47318852	0.32908352	target selection
ENSDART00000127717	FO704622.1	0.044011406	0.358399682	0.167598174	1.49594356	-0.017716595	target selection
ENSDART00000127854	ccni	0.023810358	3.365173646	3.708027214	3.915718651	4.117994882	target selection
ENSDART00000127971	TMEM184B (1 of many)	0.008188761	0.084681099	0.512647767	0.678333061	0.362195553	target selection
ENSDART00000128174	foxp1b	0.015925333	0.060807795	0.283454627	0.694455914	0.748409918	target selection
ENSDART00000128457	ephb1	0.008075493	0.374973875	0.620484905	1.032855939	0.805207117	target selection
ENSDART00000128659	pcdh2ab10	0.033001298	0.122813964	0.17598472	0.479887931	0.226887021	target selection
ENSDART00000128742	CABZ01118678.1	0.000370918	0.234931395	0.748418816	0.975879993	0.726138214	target selection
ENSDART00000128760	pacs2	0.002951261	-0.00152054	0.063293894	0.531537781	0.626023304	target selection
ENSDART00000128965	bcar1	0.029418485	0.218283788	0.254312241	0.324642744	0.284302999	target selection
ENSDART00000128978	CR391998.1	0.012467303	1.262775633	1.767194773	1.40829971	2.265912659	target selection
ENSDART00000129210	cry1ba	0.000444051	-0.306284135	0.315253029	0.635836839	0.449599168	target selection
ENSDART00000129643	aplnrb	0.039820338	-0.038448881	0.28030901	0.821411842	0.34582741	target selection
ENSDART00000129704	abhd17aa	0.001225897	-0.089448965	-0.057797962	0.474531723	0.44635249	target selection
ENSDART00000130546	maptb	0.000182178	0.390340472	1.128769701	1.393133544	1.028418073	target selection
ENSDART00000130554	isg15	0.009167819	0.469884875	0.09681696	3.101380991	0.025074196	target selection
ENSDART00000130569	st8sia5	0.00179529	-0.025137728	0.545403948	0.761405173	0.616910757	target selection
ENSDART00000131431	sulf2a	0.000307309	0.379800676	0.895838573	1.99747824	2.182238849	target selection
ENSDART00000131721	fam65b	0.003648083	-0.06007994	0.061772028	0.37892142	0.335909594	target selection
ENSDART00000131944	tagln3b	0.009948051	0.184610888	0.818287372	1.023242059	0.555937355	target selection
ENSDART00000132175	nadl1.1	0.000119513	0.709405923	0.881560036	1.238414697	1.046443747	target selection
ENSDART00000132206	si:dkey-242g16.2	0.027067133	0.572333551	0.964289734	1.608250495	0.992663233	target selection
ENSDART00000132761	arhgdig	0.00124178	-3.192123378	0.713824666	1.228568907	0.922752193	target selection
ENSDART00000133131	uba1	0.002246206	0.147030579	0.239356262	0.529136799	0.293288779	target selection
ENSDART00000133466	CR847503.1	0.009263143	-0.023677254	0.459363169	0.719903439	0.430626169	target selection
ENSDART00000133473	CU207343.1	0.024547153	0.284146912	1.104685019	1.557001573	0.994606199	target selection
ENSDART00000133851	nyap2b	6.48692E-06	-0.344969094	0.36816036	0.857787268	0.825724613	target selection
ENSDART00000134044	vdac3	0.00042933	-0.155270833	0.146773494	0.378912201	0.402649801	target selection
ENSDART00000134052	si:dkey-17e16.8	0.048722402	0.112444287	0.152961651	0.336755528	0.189687147	target selection
ENSDART00000134514	smarca4a	0.00733086	0.020398578	0.110031025	0.339984244	0.255390677	target selection
ENSDART00000134729	BX936308.1	3.77986E-05	-0.246065702	0.716060471	1.529870623	1.562652796	target selection

ENSDART00000134801	ccdc92	0.00194329	0.127931369	0.677319918	0.797788944	0.697201329	target selection
ENSDART00000135125	serp2	0.018167343	4.40213E-05	0.384051284	0.40863701	0.667539121	target selection
ENSDART00000135147	si:ch211-218d20.15	0.001847721	-0.111515958	0.398515484	0.749278715	0.887643411	target selection
ENSDART00000135230	chd7	0.009588901	0.169349437	0.346809261	0.458651128	0.318074781	target selection
ENSDART00000135248	anxa13l	0.005796202	2.875521493	3.590436866	3.752911284	3.4448321	target selection
ENSDART00000135399	phf11	0.025606413	-0.105908519	0.331215825	2.01559672	0.351931859	target selection
ENSDART00000135821	mfsd6b	0.000245464	0.610408738	1.551908888	1.729395905	1.289342978	target selection
ENSDART00000135989	cep170aa	0.001998468	0.062401976	0.336049788	0.4713762	0.353973795	target selection
ENSDART00000136103	ubap2a	0.017101809	0.173955465	0.276885256	0.421817829	0.278699527	target selection
ENSDART00000136141	kcnq3	1.15646E-05	-0.157652998	0.519381195	1.385651499	1.123132968	target selection
ENSDART00000136439	bcl7a	0.000926509	0.000990732	0.374886928	0.452965996	0.230358497	target selection
ENSDART00000137228	si:rp71-80o10.4	0.016583954	-0.005305454	1.094390245	1.175846782	0.577915575	target selection
ENSDART00000137624	eef2a.2	0.029418485	2.619194541	2.67605773	3.551783602	3.00375243	target selection
ENSDART00000137903	BX640512.2	0.003143898	-0.158278671	-0.01949183	1.915495669	-0.120027797	target selection
ENSDART00000137918	bbc3	0.000309757	0.101268325	0.767629492	0.853650102	0.865966102	target selection
ENSDART00000138081	bcl6ab	0.000589104	0.948060654	0.690527167	0.988530239	1.012261919	target selection
ENSDART00000138321	parp12b	0.041240008	0.255975647	0.107710029	0.742615255	0.386793554	target selection
ENSDART00000138759	clta	0.003329747	-0.330987711	-0.247185854	1.110067577	0.98649153	target selection
ENSDART00000139299	zgc:162928	0.01695739	0.559235647	0.580335587	1.258831581	1.081287075	target selection
ENSDART00000139387	atp1b1b	0.003609059	-0.121937317	0.340318468	0.40085255	0.510906972	target selection
ENSDART00000139702	casp3a	0.000935288	0.559260909	1.157211853	1.445414898	1.128298777	target selection
ENSDART00000139863	dcbld2	0.015156186	0.210663919	0.284613411	0.515685864	0.299926066	target selection
ENSDART00000139917	myha	0.009294776	0.315818594	4.091377824	5.948901319	4.44901869	target selection
ENSDART00000140230	zgc:123068	0.02114031	0.653257046	0.043755773	2.027002482	0.024521261	target selection
ENSDART00000140255	si:dkey-12h9.6	0.007190928	0.01002873	0.192183301	0.434559619	0.43853246	target selection
ENSDART00000140284	zbtb16a	0.028958693	2.407975491	2.022869932	2.341949009	2.679474288	target selection
ENSDART00000140301	TTC9	0.001356985	0.099722728	0.582192427	0.813799221	0.513650171	target selection
ENSDART00000140586	col1a1a	0.05696964	0.136049595	0.737681947	1.130025782	0.5982928	target selection
ENSDART00000140908	gse1	0.021503672	0.266945251	0.781532641	1.123032019	0.70398488	target selection
ENSDART00000140940	cluhb	1.88889E-05	-1.005907144	-1.058846619	1.005581701	-0.029667711	target selection
ENSDART00000141237	si:dkeyp-113d7.10	0.010693512	-0.138953924	0.055420663	0.3781204	0.217616879	target selection
ENSDART00000141278	si:ch211-14c7.2	0.002167167	0.247729936	0.220022891	0.550240269	0.375370237	target selection
ENSDART00000141338	enc1	0.008595135	0.132252305	0.206249243	0.415692262	0.451357063	target selection
ENSDART00000141367	si:ch211-238n5.4	0.007639785	-0.027130317	0.13157615	0.368504617	0.417326096	target selection
ENSDART00000141444	abcg4a	0.000281821	-0.060112999	0.269266665	0.721083006	0.688157505	target selection
ENSDART00000141446	coro1cb	0.00025494	-0.332235831	0.115994662	0.453423489	0.231540088	target selection
ENSDART00000141750	tnnt3b	0.003035847	-1.89549374	3.616832877	4.325157956	3.265344737	target selection
ENSDART00000141876	ftr30	0.039458126	0.380222613	0.277973219	1.408194744	0.300573986	target selection
ENSDART00000141974	acsl3b	0.002176351	-0.154671373	0.25230778	0.484219802	0.119435773	target selection
ENSDART00000141981	loxhd1a	0.006545343	0.329259022	0.257582221	0.952686722	1.148017375	target selection
ENSDART00000142155	myh14	0.030748502	0.116679043	0.189831513	0.30854982	0.263648695	target selection
ENSDART00000142377	MEGF9 (1 of many)	0.000470359	-0.489828408	-0.163053003	0.463102805	0.22210349	target selection
ENSDART00000142919	ssh1b	0.007672783	0.196097873	0.173857746	0.465900153	0.503515831	target selection
ENSDART00000143697	si:dkeyp-69e1.8	0.006141198	0.277815064	0.526114648	0.670410394	0.498924627	target selection
ENSDART00000143819	VASH1	0.001175355	0.033504925	0.324442057	0.595830734	0.534870749	target selection
ENSDART00000143999	tnnt3b	0.007788648	0.546685869	4.375596584	5.084902064	3.992962258	target selection
ENSDART00000144342	ppp1r14ba	9.11161E-05	0.017178827	0.781919568	1.244415225	1.189050173	target selection
ENSDART00000144681	anxa13l	0.000360396	0.688342481	1.628944564	2.065542903	1.40538163	target selection
ENSDART00000144737	srsf3b	0.029418485	0.361810279	0.317106803	0.304841622	0.541582711	target selection
ENSDART00000144766	nars	0.003162373	0.166803353	0.38562143	0.598983821	0.308867409	target selection
ENSDART00000145065	NA	0.00387273	4.897906418	2.757476262	4.60015098	5.019792283	target selection
ENSDART00000145124	dact3a	0.021766754	0.112856383	0.770301052	1.625107526	0.355230326	target selection
ENSDART00000145230	eef1db	0.009491292	0.569560585	0.627404265	1.018116547	0.789247315	target selection
ENSDART00000145494	kcnq3	5.06657E-05	-0.150620266	0.575528836	1.458464727	1.126016522	target selection
ENSDART00000145605	camk2d1	0.020107844	0.045519482	0.325117172	0.151136219	0.629830606	target selection
ENSDART00000145886	osbpl3b	0.023840313	0.13475307	0.290875362	0.450588606	0.199350453	target selection
ENSDART00000146106	nfil3-5	5.17084E-05	0.032256315	1.067374086	1.300867348	1.052274713	target selection
ENSDART00000146107	ywhae1	0.007536007	-0.007595908	0.375656099	0.665999115	0.632685234	target selection
ENSDART00000146180	csrnp2	0.003710792	0.237147162	0.374829692	0.697707124	0.402573932	target selection
ENSDART00000146247	zc2hc1a	0.010853056	0.181680565	0.536623189	0.750568991	0.668481158	target selection
ENSDART00000146400	st8sia5	0.004793697	0.352731191	0.917371468	1.067350167	0.852063315	target selection
ENSDART00000146517	dpysl2b	0.000112531	0.345677828	1.022911519	1.287775094	0.946528154	target selection
ENSDART00000146563	magixa	0.011667422	0.075133988	0.355594726	0.57567448	0.363486006	target selection
ENSDART00000146708	adar	0.009288398	-0.042581428	-0.004444765	0.40535861	0.024953366	target selection
ENSDART00000146776	tspan2b	0.000107854	0.412627689	1.44398444	1.603529521	0.88938646	target selection
ENSDART00000146872	emid1	0.002495957	0.014325328	0.393704295	0.644359098	0.692252594	target selection
ENSDART00000147474	stmn2b	2.74697E-05	0.410261377	1.543598694	2.044768785	1.667647819	target selection
ENSDART00000147481	phf11	0.071106895	2.519105146	2.056646778	4.198159431	2.169035406	target selection
ENSDART00000147609	shc1	4.71668E-05	-0.131375988	0.113051171	0.56388659	0.681815315	target selection
ENSDART00000147625	pik3r3b	0.00053559	-0.257748291	0.021181096	0.371087748	0.412468691	target selection
ENSDART00000147794	nt5c2l1	0.034100602	2.1295515	2.541950381	3.263642166	1.818207515	target selection
ENSDART00000148249	plppr4b	1.75467E-05	-0.24256353	-0.068311603	1.098973668	1.360337893	target selection
ENSDART00000148280	prr18	0.000193263	0.103510486	0.905956065	1.016662607	0.752186459	target selection
ENSDART00000148296	tnni1d	0.009288398	0.552273501	5.279876604	5.419924358	3.332159231	target selection
ENSDART00000148475	cspg5a	2.33589E-05	0.090085539	0.726711302	1.793409667	1.632985142	target selection

ENSDART00000148661	stat2	0.006978095	0.11967996	-0.016653911	1.354000143	0.023510931	target selection
ENSDART00000148927	tgfb2	0.017007145	0.452467242	0.589301589	0.923363405	0.590115295	target selection
ENSDART00000149041	reep1	0.002149847	-0.249037096	0.218537351	0.449912583	0.370752516	target selection
ENSDART00000149237	xirp2a	0.062966527	0.190889632	0.253883647	0.37076536	0.19112506	target selection
ENSDART00000149351	scd	5.17084E-05	-0.095125933	1.291914984	1.511394876	0.539585936	target selection
ENSDART00000149684	si:dkeyp-52c3.2	0.035317591	2.125770421	2.315774699	2.351460593	2.938761267	target selection
ENSDART00000149730	stox2a	0.007236331	0.026475093	0.234017219	0.506304198	0.292160739	target selection
ENSDART00000149768	kif21a	0.000349997	-0.021643242	0.361851849	0.686688714	0.546979573	target selection
ENSDART00000149777	setx	0.006824859	0.020956021	0.009824369	0.394668282	0.077312111	target selection
ENSDART00000149878	otud4	0.000390311	0.242358714	0.196542793	0.87579795	0.138730183	target selection
ENSDART00000149956	apc	0.012706968	0.195183663	0.26527048	0.385323133	0.453680151	target selection
ENSDART00000150128	foxp1b	0.020184087	0.347545443	0.556370195	1.342974258	1.269825931	target selection
ENSDART00000150148	slc7a6	7.6604E-05	0.22923155	0.720543979	1.05061766	1.171565717	target selection
ENSDART00000150362	si:ch211-197k17.3	0.00308528	-0.145582952	0.205377076	0.711396584	0.681320306	target selection
ENSDART00000150863	nrp2a	0.0002611	0.543372402	0.197731133	0.582446066	0.760458596	target selection
ENSDART00000151044	baz2ba	0.006344596	2.371956879	3.808025242	3.802702579	3.968575995	target selection
ENSDART00000151195	43166	0.003646166	-0.18277274	0.240052097	0.74148711	0.799803253	target selection
ENSDART00000151717	43166	0.000507818	0.010098896	0.251138543	0.521642437	0.401235935	target selection
ENSDART00000151899	ldlrb	0.001252059	-0.013482381	0.739364147	1.30137615	0.56832803	target selection
ENSDART00000152181	mapta	0.000943511	-0.246945515	0.298203412	0.945051693	0.777575562	target selection
ENSDART00000152284	si:ch73-301j1.1	0.003849593	-0.68804704	-0.624114046	1.993753311	-0.48476616	target selection
ENSDART00000152489	irf3	0.006723268	-0.152502705	0.050924712	1.421424509	-0.375867622	target selection
ENSDART00000152636	clocka	0.010610046	0.008631834	0.196030905	0.593132542	0.426491394	target selection
ENSDART00000152678	ifit10	0.037152675	0.50194473	0.092674262	4.442632616	1.169305685	target selection
ENSDART00000153013	eef1db	0.018003813	0.150654853	0.305331563	0.633860114	0.458033815	target selection
ENSDART00000153087	bmf2	0.016249513	0.285623957	0.323465948	0.348774195	0.627801645	target selection
ENSDART00000153212	adgra1a	0.000596344	0.27394507	0.382231602	0.735064793	0.705504548	target selection
ENSDART00000153217	eef1db	9.24337E-05	0.666551397	1.483640099	2.024242417	1.323561336	target selection
ENSDART00000153289	nos1apb	0.003126045	-0.207119032	0.371227589	0.722590517	0.685079944	target selection
ENSDART00000153377	eef1db	1.1419E-05	0.533867077	0.950085432	1.385417409	1.0336151	target selection
ENSDART00000153510	si:ch73-160l8.2	0.032861605	0.056068509	0.128148598	1.161251796	0.352706117	target selection
ENSDART00000153562	ppfia3	0.004813371	-0.022654827	0.232304266	0.485406891	0.444415736	target selection
ENSDART00000153657	trim3a	0.002721779	0.068235605	0.356875561	0.585605002	0.467600874	target selection
ENSDART00000153700	BX088707.1	0.01037926	0.029406694	0.3559423	0.787563577	0.447534651	target selection
ENSDART00000154045	glg1a	0.024649569	0.211468857	0.293330382	0.40088818	0.351496464	target selection
ENSDART00000154393	helz2	0.000250511	-0.348999688	-0.710690348	2.383802665	-0.188541475	target selection
ENSDART00000154719	hip1	0.001124982	0.155954348	0.357624902	0.536540352	0.421858349	target selection
ENSDART00000154978	si:ch211-284f22.3	0.034457087	0.111486547	0.17394927	0.442535968	0.352663532	target selection
ENSDART00000155094	si:ch211-286c4.6	0.018611393	0.931010056	1.14507062	1.165043802	1.805632584	target selection
ENSDART00000155128	meis3	0.088742714	1.719560674	1.869294835	2.789896441	2.118152659	target selection
ENSDART00000155190	maptb	0.000235238	1.096239137	1.804532446	2.232737802	1.717568221	target selection
ENSDART00000155267	si:ch211-149e23.4	0.033633212	0.305598533	0.378888393	0.64366915	0.422691793	target selection
ENSDART00000155368	si:ch73-95l15.3	0.006605202	1.516126674	2.634881995	2.849448463	2.215584039	target selection
ENSDART00000155464	ftr73	0.005040201	0.436406361	0.364310996	2.049125852	0.40110848	target selection
ENSDART00000155610	tdg.2	0.007955678	0.198987795	0.279928785	0.39077816	0.364649362	target selection
ENSDART00000155723	si:ch211-24o10.6	0.041249708	-0.255651916	0.580162906	3.993132482	0.654326546	target selection
ENSDART00000156295	myhb	0.001660423	-2.03199835	4.211906798	4.339398917	2.940685436	target selection
ENSDART00000156535	vav3b	2.2034E-05	-0.155549402	0.298871549	0.871724785	0.959064908	target selection
ENSDART00000156546	plcxd1	0.074703162	0.33889602	0.161133476	0.572020307	0.368311625	target selection
ENSDART00000156577	si:ch211-130m23.5	0.001555418	-0.007378589	0.400588111	0.725075783	0.606240178	target selection
ENSDART00000156690	ftr88	0.038938505	0.478641753	0.356185618	1.950902073	0.248066147	target selection
ENSDART00000156765	soga3a	0.005830767	0.515035881	0.806381678	1.137120698	0.812578505	target selection
ENSDART00000156792	kif1aa	0.004591248	-0.016449727	0.304055121	0.524314954	0.462526752	target selection
ENSDART00000156806	rps6kl1	0.014022061	0.03495508	0.325476415	0.37135553	0.249571068	target selection
ENSDART00000157058	si:dkey-276j7.1	0.003257025	-0.114061291	0.35744712	0.794710779	0.605587625	target selection
ENSDART00000157407	nacad	0.00067643	-0.349281215	0.532726398	0.824860187	0.457523633	target selection
ENSDART00000157475	coro7	0.000780014	0.174942149	0.053392758	0.483358888	0.70362148	target selection
ENSDART00000157663	reln	0.0021138	-0.012850615	-0.065342924	0.75286937	0.74278543	target selection
ENSDART00000157682	kat2a	0.0058872	0.094563424	0.264392397	0.483214757	0.33498107	target selection
ENSDART00000157817	lrrfip1b	0.000829135	-0.412751624	0.086992217	0.168101768	0.241775802	target selection
ENSDART00000157920	BX664622.3	0.008776907	0.309964523	0.2387983	0.211520656	0.43648414	target selection
ENSDART00000157958	nav3	0.000514549	0.289857115	0.341415382	0.725222646	0.845169813	target selection
ENSDART00000158072	gpc2	0.000503033	0.147140386	0.761040446	1.023252339	0.612693915	target selection
ENSDART00000158098	gpr132b	0.000239262	-0.335128962	-0.097182083	0.890467313	0.848088338	target selection
ENSDART00000158532	gnb2	0.000292971	-0.185937539	0.268112673	0.644184639	0.701859814	target selection
ENSDART00000158533	nup50	0.010377142	0.24455835	0.550142856	0.78129311	0.523300254	target selection
ENSDART00000158563	wu:fj29h11	0.00145023	0.059907274	-0.166860874	1.78519307	0.017403627	target selection
ENSDART00000158825	actc1b	0.011976464	0.015935591	2.858633574	3.729694641	2.583924646	target selection
ENSDART00000158837	dync1h1	0.002663982	0.179298196	0.363338197	0.829945857	0.598133568	target selection
ENSDART00000159252	abca3b	0.000342238	0.181429492	0.428459166	0.640212151	0.464148107	target selection
ENSDART00000159316	mvda	0.005340573	0.177821409	1.524901044	1.830059782	0.927790622	target selection
ENSDART00000159454	CABZ01044277.1	0.01993115	0.109016927	0.066686129	0.335359626	0.279394938	target selection
ENSDART00000159673	dync1i2b	0.004377247	0.025252757	0.176262587	0.413723725	0.300516643	target selection
ENSDART00000159782	agtpbp1	0.000322759	-0.044706055	0.173624177	0.473061984	0.407831162	target selection
ENSDART00000160107	CU639413.1	0.012966692	0.54252914	0.409259037	1.419043143	0.130740048	target selection

ENSDART00000160154	si:dkey-238i5.2	0.000470962	-0.200329967	0.657530632	1.151706484	1.107326351	target selection
ENSDART00000160288	FP236812.6	0.067256516	0.238382616	0.543365019	0.866982901	0.48841948	target selection
ENSDART00000160444	si:dkey-95j14.1	0.010110237	-0.172108125	0.025439256	0.48271623	0.286285124	target selection
ENSDART00000160571	mxe	0.005032085	0.057431509	-0.237146132	2.037062599	-0.095948656	target selection
ENSDART00000160600	cecr5	0.002653527	-0.047380691	0.036242917	0.854821049	0.34511846	target selection
ENSDART00000160783	rorcb	0.000119513	-0.657257878	0.184912673	0.679992874	0.484278813	target selection
ENSDART00000160901	FMNL1 (1 of many)	0.003432504	1.814810207	1.982271858	2.471309982	2.117776194	target selection
ENSDART00000161048	psmf1	0.005446855	-0.002898908	-0.064926331	0.496331974	-0.135580746	target selection
ENSDART00000161081	satb1a	0.000922422	-0.172286726	0.059256047	0.306569639	0.367466245	target selection
ENSDART00000161153	GAN	0.001865237	-0.076211127	0.283155249	0.536837618	0.420868128	target selection
ENSDART00000161407	tnika	0.002828188	-0.172678134	0.105967748	0.465016741	0.501980476	target selection
ENSDART00000161566	b4galt6	0.026706504	0.155163411	0.348743299	0.367166825	0.307075635	target selection
ENSDART00000161693	cetn2	0.000119513	-0.42442122	0.157217987	0.592113306	0.77779027	target selection
ENSDART00000162055	mark1	0.005808489	-0.053725407	0.099849875	0.364172927	0.499618468	target selection
ENSDART00000162459	scarb2a	0.014234373	0.204566318	0.711810949	0.821282796	0.534817262	target selection
ENSDART00000162529	zgc:172139	0.021599545	0.398363371	0.414482162	0.745277151	0.505205904	target selection
ENSDART00000162617	ppp2cb	0.012327284	0.050413288	0.287682373	0.42325509	0.191699941	target selection
ENSDART00000162761	CU984600.2	0.008281487	0.225517614	-0.442281881	2.62985535	0.023562363	target selection
ENSDART00000162838	agrn	0.003446605	0.183430669	0.084636207	0.451853257	0.572854924	target selection
ENSDART00000162915	dclk2b	0.000530154	0.022821815	0.633802822	1.144009661	0.962163073	target selection
ENSDART00000163526	mansc1	0.000511051	0.060022776	0.316271416	0.600176682	0.64715281	target selection
ENSDART00000163680	hmgcs1	0.000409858	0.250779539	0.717953967	2.106714123	1.525845318	target selection
ENSDART00000163741	pwwp2b	0.008900663	0.088274726	0.335331052	0.428666425	0.318380002	target selection
ENSDART00000163930	znfx1	0.000555101	0.194042843	0.097168789	1.523223234	0.201252376	target selection
ENSDART00000164038	SAMD14	0.00059038	-0.096477793	0.217514922	0.565397199	0.769428937	target selection
ENSDART00000164055	cap2	0.01463785	0.292123593	0.450008213	0.705800498	0.580755871	target selection
ENSDART00000164298	RNF122	0.025378177	0.037145597	0.153249015	0.432199178	0.405886045	target selection
ENSDART00000164585	mvda	0.003470173	-0.034972011	1.767545823	1.673143467	0.929274175	target selection
ENSDART00000164612	MYH7 (1 of many)	0.021678375	0.128311883	3.589180748	4.208970977	3.051349831	target selection
ENSDART00000164647	slc12a2	0.001104902	-0.231168401	0.356367716	0.84043135	0.654792579	target selection
ENSDART00000164809	si:ch73-233f7.5	9.56612E-05	0.260792098	0.492638431	0.828767158	0.768289306	target selection
ENSDART00000164853	cnp	0.01179775	1.119904973	1.074260191	2.53120974	1.692544703	target selection
ENSDART00000165066	eif2ak2	0.000768304	0.145136508	0.290335878	1.008511718	0.199274727	target selection
ENSDART00000165199	mapre2	0.003404085	0.262487966	1.260089875	1.425017722	0.706527865	target selection
ENSDART00000165230	map4k4	0.006674249	-0.058109942	0.098582575	0.424133469	0.341075529	target selection
ENSDART00000165318	thsd7bb	0.000400742	-0.173014975	0.179455909	1.051340912	1.022924373	target selection
ENSDART00000165484	jpt1a	0.001390124	0.363755224	1.1796439	1.211655345	0.942198247	target selection
ENSDART00000165656	mxd3	6.23756E-05	0.030444632	0.928898979	1.014066924	0.787179484	target selection
ENSDART00000165824	setdb1b	0.057995641	0.086359323	0.217956862	0.320141564	0.188338727	target selection
ENSDART00000165877	purg	0.000570625	-0.159238984	-0.087175961	1.048436006	0.831059287	target selection
ENSDART00000165987	DST	0.053793435	0.207553536	0.19383587	0.337156934	0.233508941	target selection
ENSDART00000166174	PARP12	0.001662054	0.236086028	0.041618587	1.350827793	0.15615804	target selection
ENSDART00000166224	smyhc2	0.001834127	1.194842223	5.367086788	6.852910143	4.718916943	target selection
ENSDART00000166242	NA	0.084522001	0.20194611	0.241162188	0.512911525	0.246398136	target selection
ENSDART00000166268	YTHDC2	0.005459224	0.015267861	-0.127181664	0.395706164	0.317645369	target selection
ENSDART00000166580	pak1	0.001850572	0.254456198	0.505135694	0.67991388	0.523148639	target selection
ENSDART00000166607	RAPGEF4 (1 of many)	0.000589961	-0.233842979	0.163001528	0.421327636	0.212581301	target selection
ENSDART00000166634	rnf213b	0.005353377	0.322643691	-0.072602668	2.242716781	-0.029575481	target selection
ENSDART00000166693	NA	0.006591698	0.184413566	-0.110459993	1.581805418	0.290855458	target selection
ENSDART00000166756	mfsd11	0.024397386	0.314593735	0.230184492	0.795700169	0.565917277	target selection
ENSDART00000167128	pargl	0.012921442	0.159346244	0.133314978	0.619065571	-0.065913899	target selection
ENSDART00000167219	pcdh1g26	0.001884118	-0.056136761	0.084950376	0.428942573	0.447913813	target selection
ENSDART00000167226	pycr1b	0.004907334	0.1083633	0.252664475	0.730734657	0.419591074	target selection
ENSDART00000167322	si:ch73-233f7.3	0.009706658	-0.088252023	0.25632847	0.562157447	0.392412002	target selection
ENSDART00000167388	vps33a	0.033661318	0.155328285	0.245633599	0.343008348	0.290252576	target selection
ENSDART00000167613	hmgcs1	0.006978095	-0.061825835	0.154559892	0.893983325	0.609306628	target selection
ENSDART00000167696	si:ch73-233f7.4	0.012726801	0.264747026	0.489460093	0.625111029	0.721718733	target selection
ENSDART00000167947	CABZ01079480.1	0.056544045	0.418778383	0.234022686	1.236946888	0.318728833	target selection
ENSDART00000168157	mkrn2os.2	0.002390041	0.50354684	0.675600001	1.137753613	0.994948824	target selection
ENSDART00000168167	rtcb	0.011929276	0.060457492	0.250042841	0.449158496	0.303788278	target selection
ENSDART00000168241	tubb2b	0.000133028	-0.194997441	0.285540167	0.888424822	0.706942534	target selection
ENSDART00000168483	si:dkey-16p21.7	0.019918813	0.12768447	0.414452748	0.453712173	0.342486315	target selection
ENSDART00000168542	arf3a	0.004338147	0.034708072	0.783187174	0.81852048	0.521503928	target selection
ENSDART00000168559	ttf2	0.00064709	-3.902752419	0.990904721	1.020977359	0.830250106	target selection
ENSDART00000168822	pabpc4	0.000648976	1.120879979	1.597763229	2.131974577	1.975353129	target selection
ENSDART00000168876	si:dkey-81h8.1	0.000251123	-0.18803818	0.217329319	0.889109393	1.046963425	target selection
ENSDART00000168899	pcdh1g33	0.000227894	-0.406478127	-0.012838877	0.30409891	0.306864798	target selection
ENSDART00000168918	pcdh1gc6	0.013984224	0.501599317	0.826475212	1.380988505	1.066751642	target selection
ENSDART00000169105	pcdh1g29	1.36144E-05	-0.285141513	0.143203693	0.405619816	0.452362051	target selection
ENSDART00000169130	ftr64	0.001382674	0.270335706	0.025822717	1.271602536	0.119170332	target selection
ENSDART00000169187	ptpro	0.001151232	0.079888282	0.61716785	1.250280006	1.189522062	target selection
ENSDART00000169341	nrp1a	0.010684329	0.186448987	0.415165621	0.563193133	0.501195382	target selection
ENSDART00000169648	NA	0.003509558	0.47490981	0.626028668	1.036581019	0.748938091	target selection
ENSDART00000169667	ptpro	1.98934E-05	-0.163271096	0.493460364	0.820001756	0.696103761	target selection
ENSDART00000169823	pnpla3	0.017079861	0.41574021	0.387767111	0.775977498	0.378524498	target selection

ENSDART00000169845	pargl	0.003678598	0.069840572	-0.083042796	0.787500652	-0.250417109	target selection
ENSDART00000170184	fgd1	0.00148037	0.020796397	0.102196495	0.582360556	0.487921156	target selection
ENSDART00000170341	apba1a	0.002945703	-0.229826313	-0.069954525	0.548704484	0.456234995	target selection
ENSDART00000170405	CARTPT (1 of many)	0.010911523	1.790273718	2.206490975	2.680185278	2.041251968	target selection
ENSDART00000170571	dmtn	0.016814523	0.084482265	0.234365609	0.453911363	0.30033427	target selection
ENSDART00000170874	phldb1b	0.048701514	0.7459988	0.426405219	0.977480972	0.780660286	target selection
ENSDART00000170951	PXDN	0.08148621	0.459355707	0.64772414	0.996368549	0.498632459	target selection
ENSDART00000171021	rab3ip	0.002818463	0.020022814	0.162794734	0.729696081	0.694579303	target selection
ENSDART00000171178	nlrc3l	0.007264025	0.061705987	0.093907561	0.374016309	-0.032278843	target selection
ENSDART00000171244	ssh2b	0.005083479	-0.244446722	-0.139215381	0.546963377	0.2356201	target selection
ENSDART00000171335	map4k4	0.002682809	0.106316233	0.336083411	0.398966135	0.337674059	target selection
ENSDART00000171354	nt5c2l1	0.075016406	0.533832785	0.693578932	1.620342483	0.507169588	target selection
ENSDART00000171382	epb41l3a	0.001372006	-0.440465797	0.150336785	0.618974575	0.751984906	target selection
ENSDART00000171433	tnni1d	0.008010071	0.40952122	3.032354782	4.009528497	2.823338021	target selection
ENSDART00000171624	Metazoa_SRP	0.007467603	1.040618727	2.218519951	2.250692356	1.867352594	target selection
ENSDART00000171871	cbfb	0.02115372	0.41950513	0.366762581	0.672082991	0.461647583	target selection
ENSDART00000172022	dpysl4	0.001494008	0.270457415	0.912051797	1.185681882	0.874375314	target selection
ENSDART00000173011	GRXCR1	0.000822037	0.401804428	0.903685752	1.561252834	1.150514684	target selection
ENSDART00000173052	map7d2b	0.000808461	0.261433378	1.055987677	1.048743858	0.7752628	target selection
ENSDART00000001313	rimbp2	0.004394152	-0.29201819	-0.480722019	-0.187717263	0.029819479	brain innervation
ENSDART00000001805	csmd2	0.014563941	-0.646636229	-0.876947398	-0.300952212	-0.072675683	brain innervation
ENSDART00000002691	tspan7b	0.000775407	-0.507607245	-0.383440036	-0.192864148	0.037733122	brain innervation
ENSDART00000002908	olfm1a	0.000941975	-0.612571688	-0.410284408	-0.115653646	0.220963821	brain innervation
ENSDART00000003825	cplx2l	6.48692E-06	-1.121107993	-0.523528158	0.430342309	0.533514858	brain innervation
ENSDART00000004416	lrp1ba	1.45694E-05	-0.680071532	-0.933155051	-0.315048731	0.19015716	brain innervation
ENSDART00000004548	barhl1b	0.009605408	-2.472825095	-1.41656094	-0.268019579	0.230183047	brain innervation
ENSDART00000004717	igf1	0.001566135	-0.688444818	-0.421253017	-0.11318285	0.107184312	brain innervation
ENSDART00000005086	atp1a1b	0.029368278	-0.434454878	-0.290341007	-0.096388542	-0.096973501	brain innervation
ENSDART00000005929	ppp3ca	0.003189261	-0.536938929	-0.189476468	0.016656951	0.377870736	brain innervation
ENSDART00000006380	tbx3a	0.004106215	-0.244591708	-0.352646993	-0.144447405	0.06961498	brain innervation
ENSDART00000006435	gpr27	0.005338552	-0.291018325	-0.294873264	-0.134468137	0.025589784	brain innervation
ENSDART00000006474	glra4b	2.84805E-05	-0.56482245	-0.497893783	0.107334542	0.502166045	brain innervation
ENSDART00000006602	pde4a	0.001271118	-0.284660623	-0.353015908	-0.053068214	0.108985293	brain innervation
ENSDART00000006612	tbr1b	2.95145E-05	-1.166029945	-1.008207372	0.617040877	1.169470893	brain innervation
ENSDART00000006619	rbpms2b	3.29362E-06	-0.996572205	-0.617915271	-0.145310289	0.143289699	brain innervation
ENSDART00000007208	lrrc4bb	0.000199258	-0.502121898	-0.528156819	0.019633372	0.067706598	brain innervation
ENSDART00000007308	wnt10a	0.000994591	-1.246823676	-1.58888825	-0.322358389	0.209755977	brain innervation
ENSDART00000007531	slit2	0.008856124	-0.281096395	-0.311205466	-0.174560483	0.038385548	brain innervation
ENSDART00000007584	snap25a	0.002371664	-0.520469397	-0.457109198	-0.208714288	-0.117586032	brain innervation
ENSDART00000007806	zbtb16a	0.004364661	-0.634450415	-0.731920804	0.190781828	-0.364381887	brain innervation
ENSDART00000007972	dlgap4b	0.009649948	-0.346497047	-0.558852799	-0.093125576	-0.08715572	brain innervation
ENSDART00000008594	tmem178	0.000118072	-0.626672748	-0.469011917	-0.272377327	0.052520712	brain innervation
ENSDART00000008711	gys1	0.002746026	-0.668450567	-0.607630136	-0.28295897	-0.009086585	brain innervation
ENSDART00000008840	otofa	0.003654787	-0.788283647	-0.975123326	-0.33101193	-0.149384457	brain innervation
ENSDART00000009691	scml4	0.001274316	-0.348233268	-0.325522885	-0.113421585	0.115178638	brain innervation
ENSDART00000009892	gabbr1a	9.33581E-05	-0.618262887	-0.683782394	0.013008531	0.484024166	brain innervation
ENSDART00000010119	eef1a2	7.10385E-05	-1.586399577	-1.404358062	-0.455450934	0.174130308	brain innervation
ENSDART00000010199	fam219ab	0.022425099	-0.329176512	-0.152550548	-0.14317011	0.01066054	brain innervation
ENSDART00000012391	cabp1a	0.002341037	-0.440359676	-0.517676368	-0.117661538	0.040314239	brain innervation
ENSDART00000012546	ctbp2a	0.01793082	-0.410954451	-0.209595307	-0.078015176	-0.146761341	brain innervation
ENSDART00000012862	isl2a	3.28594E-05	-0.663813396	0.011664777	0.746201858	1.074694305	brain innervation
ENSDART00000013228	cacna1aa	8.82236E-05	-0.425294008	-0.48842278	0.10428355	0.42170802	brain innervation
ENSDART00000013311	grm6a	5.17084E-05	-0.718441456	-0.89272341	-0.019297194	0.441642652	brain innervation
ENSDART00000014897	srgap1b	0.002585408	-0.562468132	-0.53652608	-0.21984937	0.24230424	brain innervation
ENSDART00000015279	rtn4rl1a	0.001754036	-0.620130019	-0.765846328	-0.205912996	0.07424375	brain innervation
ENSDART00000015418	irf2bpl	0.001028061	-0.517127602	-0.432851187	-0.112506589	-0.180160421	brain innervation
ENSDART00000015629	stxbp1a	0.003413489	-0.407999477	-0.254916105	-0.074938098	0.048338374	brain innervation
ENSDART00000016535	kcns3a	0.000529346	-1.299949965	-1.227726419	-0.464780559	0.150117362	brain innervation
ENSDART00000016916	gria4b	0.002016009	-0.27171917	-0.41554348	-0.145968175	0.021630803	brain innervation
ENSDART00000017229	ncam1a	0.001161091	-0.359842421	-0.143522661	0.045009487	0.122759016	brain innervation
ENSDART00000017309	ca16b	0.003700234	-0.395585068	-0.492018229	-0.260071918	-0.046674115	brain innervation
ENSDART00000017679	ppp2r2ca	0.000199258	-0.437371106	-0.275733569	0.22042039	0.361372826	brain innervation
ENSDART00000018351	zgc:65851	8.05022E-05	-1.669295979	-1.346342078	-0.126748353	0.319422369	brain innervation
ENSDART00000019140	rorab	0.000701449	-0.499015892	-0.249117065	-0.271786747	0.004902877	brain innervation
ENSDART00000019294	si:dkeyp-75b4.9	7.89787E-05	-0.789027106	-0.841731007	-0.221050872	0.359721563	brain innervation
ENSDART00000019573	zgc:65894	0.000144061	-0.648921378	-0.373996254	0.357740851	0.502243837	brain innervation
ENSDART00000020174	dynll2b	0.013618441	-0.376289244	-0.003912141	-0.039237308	0.00732876	brain innervation
ENSDART00000020569	creld1b	0.022767452	-0.396401062	-0.06913614	0.015512001	0.011512871	brain innervation
ENSDART00000021231	slmapb	5.24617E-05	-0.557559725	-0.496297491	-0.195575587	0.049672527	brain innervation
ENSDART00000022010	hivep2b	0.004657196	-0.404683257	-0.428225913	-0.085969125	0.061919909	brain innervation
ENSDART00000022042	scn8aa	2.1371E-05	-0.64882134	-0.631929626	0.169338069	0.591046475	brain innervation
ENSDART00000022768	ak5	8.39504E-05	-0.529999471	-0.599207091	-0.317508372	0.131326788	brain innervation
ENSDART00000022866	pisd	0.0088045	-0.555411298	-0.214837183	-0.086260422	-0.186182517	brain innervation
ENSDART00000022976	kctd16b	1.37519E-05	-0.982447595	-1.281976718	-0.566394426	0.110166911	brain innervation
ENSDART00000023038	dacha	0.000322945	-0.511894205	-0.418991667	-0.004522839	0.112876213	brain innervation

ENSDART00000023210	trim13	0.005927492	-0.37704188	-0.363410115	-0.204332616	0.075535016	brain innervation
ENSDART00000024778	robo3	0.006011043	-0.327155095	-0.251732012	-0.124129181	0.058627004	brain innervation
ENSDART00000025031	pou4f1	1.45694E-05	-0.37458842	-0.169318799	0.774588614	1.160187758	brain innervation
ENSDART00000025256	igfbp2b	7.89787E-05	-0.742463234	-0.107964006	0.616733017	0.74050304	brain innervation
ENSDART00000025494	hprt1l	0.007216648	-0.364550689	-0.385937863	-0.083973479	-0.129534044	brain innervation
ENSDART00000025583	fgf8a	0.004531744	-0.058331217	-0.819626176	0.05604093	0.237151994	brain innervation
ENSDART00000025997	dip2cb	0.000892442	-0.352423277	-0.192868324	0.083423296	0.161945092	brain innervation
ENSDART00000026152	asap2a	0.002746972	-0.416411014	-0.084267369	-0.025927141	0.001189582	brain innervation
ENSDART00000026378	slc25a6	8.89795E-05	-0.475274891	-0.471356749	-0.229323143	0.038491163	brain innervation
ENSDART00000026401	TMEM178B (1 of many)	0.010150208	-0.387053719	-0.214500403	-0.049460199	0.011667684	brain innervation
ENSDART00000027345	tmem59l	4.21243E-05	-0.559867163	-0.384910477	-0.005343137	0.202377241	brain innervation
ENSDART00000027398	kcna2a	2.54845E-05	-1.558634612	-1.413069345	-0.244650937	0.296687437	brain innervation
ENSDART00000027463	hmx4	0.005683277	-0.340009377	-0.236809718	-0.106768059	0.00156649	brain innervation
ENSDART00000028285	pgbd5	0.001885682	-0.471148265	-0.169774685	0.263561856	0.22277577	brain innervation
ENSDART00000028338	scamp5a	0.008022596	-0.358452184	-0.354788931	-0.092970627	0.058203665	brain innervation
ENSDART00000028390	fgf12a	6.48692E-06	-0.792701948	-0.713373555	-0.153423419	0.185286633	brain innervation
ENSDART00000028895	negr1	0.018126285	-0.362135851	-0.256191375	-0.15121136	-0.084875523	brain innervation
ENSDART00000029457	sh2d3ca	0.000340312	-0.605491696	-0.301886794	0.051143961	0.055429229	brain innervation
ENSDART00000030409	asap1b	0.006111083	-0.387126717	-0.32480613	-0.179905202	-0.016544261	brain innervation
ENSDART00000030773	foxo3a	0.000701449	-0.522674186	-0.346284342	-0.025883071	0.086783781	brain innervation
ENSDART00000031091	vsnl1a	1.75467E-05	-0.845008079	-0.77079299	-0.390375819	0.072718281	brain innervation
ENSDART00000031167	tfap2d	0.000251123	-0.967562253	-0.629795841	0.638492839	1.047009841	brain innervation
ENSDART00000031546	chrna6	2.82487E-05	-0.487671995	-0.420962215	0.210734418	0.46609085	brain innervation
ENSDART00000031937	diras1a	0.00058454	-0.474540918	-0.418703997	-0.179232074	-0.012563291	brain innervation
ENSDART00000032161	galnt14	0.009427844	-0.529482639	-0.362873656	-0.264119496	-0.01842007	brain innervation
ENSDART00000033361	ttyh3b	0.000150409	-0.3186879	-0.037617104	0.269211477	0.394612295	brain innervation
ENSDART00000033657	grm6b	0.000670763	-0.379593806	-0.511743403	-0.159835887	0.100793439	brain innervation
ENSDART00000034790	pcp4l1	0.000799851	-0.408039344	-0.390896541	-0.213514281	-0.035791454	brain innervation
ENSDART00000034849	grin1b	0.002361523	-0.325252553	-0.609188282	-0.185270574	0.122354295	brain innervation
ENSDART00000036015	ryr1b	0.000999216	-0.116788998	-0.165312146	0.372637833	0.693396813	brain innervation
ENSDART00000037126	eno2	4.91848E-05	-0.315372643	-0.196767149	0.300750061	0.433471501	brain innervation
ENSDART00000037265	olfm1b	0.001442301	-0.595000689	-1.108211527	-0.18099665	0.18250463	brain innervation
ENSDART00000037848	dpp6b	0.004843495	-0.469010511	-0.191315433	-0.026922326	-0.081540125	brain innervation
ENSDART00000038294	tp53inp1	0.025035616	-0.610161281	0.029061275	-0.033185195	-0.028504863	brain innervation
ENSDART00000038740	galnt9	1.45694E-05	-0.705450847	-0.542663541	-0.098391024	0.177029625	brain innervation
ENSDART00000040049	camk2d2	0.002828188	-0.441100303	-0.359209869	-0.184359733	0.062709499	brain innervation
ENSDART00000040184	tenm1	0.015961295	-0.25843006	-0.314319075	-0.175496746	-0.005818614	brain innervation
ENSDART00000040278	efna2a	0.000117841	-0.820985512	-0.748851824	-0.238813858	0.346525856	brain innervation
ENSDART00000040456	cdc42bpab	0.001587567	-0.484798472	-0.258449967	-0.177779388	0.139329121	brain innervation
ENSDART00000040500	tspan9a	0.025495825	-0.374305977	-0.544164723	-0.123124308	-0.153915406	brain innervation
ENSDART00000040537	gjd1a	0.000656024	-0.421902855	-0.214222628	0.04546907	0.177131603	brain innervation
ENSDART00000040669	sphkap	0.000260159	-0.388395859	-0.454597188	0.021563144	0.051812339	brain innervation
ENSDART00000041714	atp6v0a1b	0.001487934	-0.327889206	-0.302694525	-0.070700098	0.131627227	brain innervation
ENSDART00000041740	ubl7a	0.000296518	-0.21096997	-0.119372658	0.298633763	0.574535573	brain innervation
ENSDART00000041800	epha8	0.002472108	-0.01929649	-0.346405332	0.164805877	0.748357168	brain innervation
ENSDART00000041820	lingo1a	0.017075627	-0.3685918	-0.267056385	-0.206204269	-0.004686852	brain innervation
ENSDART00000041869	grin1a	0.006472926	-0.314741638	-0.554584424	-0.238417502	0.088185899	brain innervation
ENSDART00000042083	gria4a	0.0035639	-0.246573777	-0.413439839	-0.038291419	0.265641743	brain innervation
ENSDART00000042683	cadpsb	2.18017E-05	-0.417914059	-0.394489719	0.149869704	0.381243736	brain innervation
ENSDART00000042972	srpk1b	0.001312564	-0.590887937	-0.595753709	-0.259675811	-0.090086557	brain innervation
ENSDART00000042984	epha6	0.019981901	-4.335346398	-2.736601299	-1.023821359	0.479123476	brain innervation
ENSDART00000043312	srsf5a	0.000184045	-0.406457659	-0.349179029	0.276259838	-0.003669678	brain innervation
ENSDART00000043823	osbpl10b	0.014034602	-0.615302858	-0.415836047	-0.293972471	0.154234814	brain innervation
ENSDART00000043857	irx5a	8.26035E-05	-0.492824084	-0.561776224	0.072886283	0.234832625	brain innervation
ENSDART00000043924	mpp6b	0.002539705	-0.45376092	-0.273823891	-0.178237916	-0.157850955	brain innervation
ENSDART00000044241	KCNQ2 (1 of many)	1.2193E-05	-0.793205862	-0.43808803	0.589879296	0.84351704	brain innervation
ENSDART00000044276	dip2bb	0.000804407	-0.182549034	-0.085935994	0.236910154	0.379645319	brain innervation
ENSDART00000044371	tox	0.000891702	-0.382184105	-0.377592563	-0.085511133	0.05287602	brain innervation
ENSDART00000044423	magi1b	0.000170813	-0.521004582	-0.785194432	0.042021364	0.52355612	brain innervation
ENSDART00000044678	GABRA2 (1 of many)	0.020491546	-0.18505593	-0.452041699	-0.058625322	0.015850219	brain innervation
ENSDART00000045086	prkceb	0.014103468	-0.397671937	-0.107653926	0.08012307	0.031299867	brain innervation
ENSDART00000045628	irx6a	0.000781013	-0.424070635	-0.239579413	0.17038084	0.276181325	brain innervation
ENSDART00000045861	slc43a2a	0.000311245	-0.304475968	-0.378976343	0.111239312	0.188992557	brain innervation
ENSDART00000045888	tkta	0.001494578	-0.540178562	-0.404491163	-0.145350137	0.055411831	brain innervation
ENSDART00000046211	lnx2a	0.039476911	-0.592157981	-0.322543056	-0.148137638	-0.128059395	brain innervation
ENSDART00000046438	kcnk2b	0.000775831	-1.164610191	-1.614380279	-0.460863171	0.258871322	brain innervation
ENSDART00000046530	rab42a	0.000219336	-0.542354056	-0.349553972	-0.104901238	0.132258211	brain innervation
ENSDART00000047399	mmp24	4.91848E-05	-0.337136574	-0.281370104	0.467116638	0.594580786	brain innervation
ENSDART00000047569	igf2b	5.24617E-05	-0.538912754	-0.46572679	-0.194582206	0.102619195	brain innervation
ENSDART00000048855	mtus1b	0.002977128	-0.449558058	-0.427741832	0.166302333	0.000701193	brain innervation
ENSDART00000048994	pbx3b	0.000910662	-0.545498331	-0.428509616	0.129533791	0.149586816	brain innervation
ENSDART00000049177	rab6ba	2.09905E-05	-0.532131764	-0.115244957	0.417564602	0.557714845	brain innervation
ENSDART00000049462	rab15	0.000457208	-0.425089968	-0.185335259	0.21479615	0.289096284	brain innervation
ENSDART00000049992	syt9b	2.01848E-05	-0.672988403	-0.530192885	0.095421952	0.328394957	brain innervation
ENSDART00000050037	chrnb3b	6.48692E-06	-0.414911692	-0.004263798	0.444800143	0.950014126	brain innervation

ENSDART00000050077	sdcbp	0.000304005	-0.585127298	-0.412438306	-0.175403243	-0.033038502	brain innervation
ENSDART00000050303	b3gat2	0.001365289	-0.439065182	-0.488507748	-0.270928467	0.095807034	brain innervation
ENSDART00000050308	calm1b	0.011264952	-0.359535363	-0.241046962	-0.074464366	0.015414816	brain innervation
ENSDART00000050311	rltpr	0.000161203	-0.433814029	-0.24205503	0.339832334	0.433192877	brain innervation
ENSDART00000050445	trim2a	0.004134049	-0.427473932	-0.288810529	-0.127175492	0.041370255	brain innervation
ENSDART00000050762	phactr3b	0.00849815	-0.629592387	-0.363049766	-0.266715209	-0.083768273	brain innervation
ENSDART00000051182	arhgap4b	0.003900528	-0.41225839	-0.320516515	-0.225939723	-0.055826953	brain innervation
ENSDART00000051693	irx4a	0.000591404	-1.596922819	-2.014349322	0.164610846	0.924604512	brain innervation
ENSDART00000052113	lingo1b	0.029426879	-0.337763041	-0.148396867	-0.080123582	-0.033848116	brain innervation
ENSDART00000052346	gnao1b	0.000112893	-0.534691372	-0.39418636	-0.143300564	0.218777626	brain innervation
ENSDART00000052802	calb2b	3.76305E-05	-1.334486374	-1.407708939	-0.475837952	0.218368357	brain innervation
ENSDART00000054137	igfbp5b	0.000573039	-0.49960111	-0.321727345	0.242454388	0.365417817	brain innervation
ENSDART00000054452	dlgap1b	0.000993187	-0.55212867	-0.830902632	-0.186012386	0.281181047	brain innervation
ENSDART00000054552	cdh8	0.000956736	-0.482196874	-0.526834841	-0.273874965	0.0505098	brain innervation
ENSDART00000054833	rgs11	5.42724E-05	-0.582556718	-0.789821251	-0.427532995	0.127688375	brain innervation
ENSDART00000054989	fscn1b	0.000261639	-0.529299226	-0.417661269	0.066759573	0.494675778	brain innervation
ENSDART00000055038	rybpa	0.00042906	-0.189184893	-0.138456944	0.268105644	0.373370341	brain innervation
ENSDART00000055269	gng13b	1.11583E-05	-0.70656822	-0.547882014	0.352208471	0.531882213	brain innervation
ENSDART00000055936	isl2b	4.91848E-05	-1.527471977	-1.312125436	0.470803449	0.853726063	brain innervation
ENSDART00000056138	igsf8	0.001004207	-0.41438699	-0.276734989	-0.097715498	0.052342087	brain innervation
ENSDART00000056328	elovl4b	0.005164205	-0.472600721	-0.194626997	-0.10087467	0.110456628	brain innervation
ENSDART00000056369	cadm2a	0.04906434	-0.177754586	-0.327047473	-0.068598077	-0.03429359	brain innervation
ENSDART00000056639	faim2a	0.008797915	-0.345615546	-0.285647372	-0.169343793	0.000937504	brain innervation
ENSDART00000056671	brinp2	8.25087E-05	-0.564206507	-0.55260615	-0.353392227	0.031594069	brain innervation
ENSDART00000056885	CU929046.1	2.74697E-05	-0.462145697	-0.331087748	0.285634599	0.389113052	brain innervation
ENSDART00000057369	igfbp5a	0.010190417	-0.503493701	-0.12557648	0.011432007	-0.035083178	brain innervation
ENSDART00000058736	grm4	8.05022E-05	-0.475368296	-0.757934708	-0.299132614	0.099413724	brain innervation
ENSDART00000058829	scrt1b	0.000688325	-0.466509454	-0.564612343	-0.054278048	0.250358448	brain innervation
ENSDART00000058877	rap2ab	8.2583E-05	-0.786170126	-0.835369537	-0.021239814	0.42494962	brain innervation
ENSDART00000059179	nptxra	0.000962944	-0.572157626	-0.495039684	-0.356374901	-0.015079326	brain innervation
ENSDART00000059228	vil1	0.006605202	-0.324461409	-0.136026611	-0.08718702	0.103993243	brain innervation
ENSDART00000059631	BX936415.1	0.005303358	-0.988751505	-1.218737364	-0.343900834	0.242173034	brain innervation
ENSDART00000059841	si:ch211-257p13.3	1.18742E-05	-0.713606094	-0.675183925	0.07538087	0.401418787	brain innervation
ENSDART00000059869	adra2a	0.000142155	-1.042718178	-0.710015995	-0.232479367	0.220270649	brain innervation
ENSDART00000060051	fgf14	0.028223243	-0.493332703	-0.385375543	-0.154886482	-0.098910242	brain innervation
ENSDART00000060160	calb2a	1.97197E-05	-1.240040436	-1.237007367	-0.232768326	0.397108945	brain innervation
ENSDART00000060625	lgi3	6.84125E-05	-0.698992604	-0.869112792	-0.10986795	0.409134057	brain innervation
ENSDART00000062697	gfra2a	0.003719587	-0.450482933	-0.554254218	-0.069449306	-0.130685043	brain innervation
ENSDART00000062874	atp1b3b	0.000182199	-0.415562805	-0.319287363	-0.078895256	0.137689897	brain innervation
ENSDART00000062887	disp2	2.923E-05	-0.441631902	-0.296469901	0.444242177	0.781646932	brain innervation
ENSDART00000063783	itm2ca	0.000867732	-0.362486731	-0.282712517	-0.07258841	0.153576522	brain innervation
ENSDART00000063825	sprn	0.013488739	-0.451586162	-0.272184034	-0.06406637	0.092302795	brain innervation
ENSDART00000064672	NA	5.42724E-05	-1.010331454	-0.756315191	-0.126164495	0.278123254	brain innervation
ENSDART00000064833	mafaa	0.002047801	-0.957052225	-1.143383392	-0.379735148	0.067354966	brain innervation
ENSDART00000064902	ssbp4	0.000263251	-0.349245142	-0.241492171	0.147485085	0.214650497	brain innervation
ENSDART00000065159	zgc:158291	3.4928E-05	-1.165646756	-0.784542684	0.443310678	0.751149417	brain innervation
ENSDART00000065372	kcnj3b	9.03292E-05	-0.605628976	-0.778191304	-0.104344748	0.360074992	brain innervation
ENSDART00000065599	cadm1a	0.004110692	-0.54681119	-0.778847768	-0.187580903	0.116530415	brain innervation
ENSDART00000065929	hs6st3b	0.000935288	-0.878348455	-0.942691234	-0.255367675	0.253670478	brain innervation
ENSDART00000066192	glra2	0.00133046	-0.5075286	-0.700662657	-0.376893165	0.421840665	brain innervation
ENSDART00000066294	cdk5r1b	7.6604E-05	-0.511562763	-0.495240798	0.085364893	0.188592346	brain innervation
ENSDART00000066372	id4	4.11188E-05	-0.719733282	-0.518590484	0.441847928	0.655214602	brain innervation
ENSDART00000066839	slc35g2b	7.55931E-05	-0.671846523	-0.750415444	-0.309110396	0.04424778	brain innervation
ENSDART00000067514	rbpms2a	4.11188E-05	-1.175610868	-1.156235961	0.045281157	0.593799851	brain innervation
ENSDART00000067531	syn2a	8.82236E-05	-0.50370548	-0.414636803	-0.227904446	0.132370635	brain innervation
ENSDART00000073919	kcnc1b	2.71726E-05	-0.758440974	-0.784989596	-0.33092052	0.322314215	brain innervation
ENSDART00000073950	olfm1a	7.10385E-05	-0.852827208	-0.587563633	0.013637392	0.435090193	brain innervation
ENSDART00000074036	rcvrna	0.035967167	-0.553376367	-0.06531313	-0.11758335	-0.07626787	brain innervation
ENSDART00000074362	pcdh18b	0.00033039	-0.440881831	-0.447646282	-0.227758863	0.176890552	brain innervation
ENSDART00000074543	hs3st4	0.007620644	-0.453389419	-0.572006435	-0.310659668	0.002760951	brain innervation
ENSDART00000074678	chrnb3a	4.11188E-05	-0.683036397	-0.585209438	-0.069659384	0.207417172	brain innervation
ENSDART00000074698	opn3	0.003138642	-0.589287895	-0.235981184	0.224725485	-0.032745726	brain innervation
ENSDART00000074718	spire1b	0.006352299	-0.446651583	-0.478750814	-0.155393912	-0.07838085	brain innervation
ENSDART00000074997	CU302436.3	0.001679054	-0.857583363	-0.990723788	-0.187854306	0.199234775	brain innervation
ENSDART00000075123	pcp4a	4.91848E-05	-0.598752351	-0.632528063	-0.40265776	-0.022235336	brain innervation
ENSDART00000075278	atp1b4	0.008495106	-0.758712842	-0.43098592	-0.240547748	-0.077197984	brain innervation
ENSDART00000075340	eef1a1b	2.23813E-05	-0.66677551	-0.473919347	-0.344351157	0.106841151	brain innervation
ENSDART00000075749	ppp2r2ca	0.000117007	-0.492014929	-0.268923214	0.328067214	0.425314748	brain innervation
ENSDART00000075918	pcmtd2	0.00393919	-0.427264647	-0.257111837	-0.149394946	0.062644737	brain innervation
ENSDART00000076946	PDE4DIP	0.000299141	-0.575491599	-0.478988093	-0.210265098	-0.157443712	brain innervation
ENSDART00000077216	astn1	0.002342395	-0.280184115	-0.378831195	-0.109389357	0.054556021	brain innervation
ENSDART00000077222	ldlrad4b	0.002766629	-0.327819788	-0.475734059	-0.025614815	0.028486052	brain innervation
ENSDART00000078079	pcnxl2	0.000511051	-0.518593139	-0.325199094	0.020646332	0.403616211	brain innervation
ENSDART00000078115	sdhda	0.002274284	-0.602799581	-0.148637611	0.027660422	0.175615096	brain innervation
ENSDART00000078232	cdh10a	0.005048068	-0.495858439	-0.491434243	-0.331011431	0.033804718	brain innervation

ENSDART00000078561	SPTBN4	0.000403881	-0.239897718	-0.33102434	0.293671093	0.445086753	brain innervation
ENSDART00000078856	dlg3	0.001948843	-0.31041276	-0.361138693	-0.191257864	0.041004354	brain innervation
ENSDART00000079092	si:dkey-261i16.5	0.004123458	-0.526813533	-0.563690076	-0.369398724	0.061616577	brain innervation
ENSDART00000079138	ptenb	0.013692958	-0.337433521	-0.277401842	-0.145004066	-0.034401548	brain innervation
ENSDART00000079144	ptenb	0.00889295	-0.303152595	-0.238217531	-0.001915727	-0.031063744	brain innervation
ENSDART00000079443	GABRA2 (1 of many)	0.000263077	-0.623319426	-0.542833879	-0.157704672	0.138036393	brain innervation
ENSDART00000079840	rorca	0.006111083	-0.65562057	-0.064052995	0.161319682	0.103804992	brain innervation
ENSDART00000080064	CR847953.1	0.004084205	0.050009503	-0.040451977	0.259802571	0.438904414	brain innervation
ENSDART00000080486	ywhag1	1.75467E-05	-1.02392497	-0.686234935	-0.010129168	0.2177797	brain innervation
ENSDART00000080808	six3a	0.000687981	-0.437705937	-0.430575558	-0.140593988	-0.07114268	brain innervation
ENSDART00000080927	snap25b	0.001998235	-0.418114286	-0.385475041	-0.102955296	0.076752829	brain innervation
ENSDART00000081140	CT990561.1	0.000401616	-0.348057632	-0.380510005	-0.109063338	0.180807023	brain innervation
ENSDART00000082301	myrip	0.000993187	-0.137038516	-0.238384017	0.084718693	0.41131665	brain innervation
ENSDART00000082745	EMB	0.000245083	-0.651102521	-0.703010006	-0.074268357	0.192993872	brain innervation
ENSDART00000082842	JPH3 (1 of many)	0.000165745	-0.540363483	-0.227235849	0.34555956	0.43863208	brain innervation
ENSDART00000083040	hs3st2	0.000442216	-1.059711916	-1.469328404	-0.190665179	0.30791737	brain innervation
ENSDART00000083066	asphd2	0.000463544	-0.487940941	-0.316881347	-0.126036033	0.003992278	brain innervation
ENSDART00000083569	oaz2b	2.90186E-05	-0.447559352	-0.669337328	-0.007273423	0.348858251	brain innervation
ENSDART00000083670	CABZ01041604.1	0.025197766	-0.549505735	-0.427354636	-0.238335089	-0.140824262	brain innervation
ENSDART00000084014	cplx1	2.54845E-05	-0.741807214	-0.862001833	-0.211893904	0.449532765	brain innervation
ENSDART00000084119	si:ch1073-44g3.1	0.001946729	-0.384776638	-0.202587046	0.01646479	0.134112994	brain innervation
ENSDART00000084264	adcy2a	0.000976529	-0.574108823	-0.494423944	0.049230433	0.113035845	brain innervation
ENSDART00000084354	cpeb3	0.004463264	-0.1650884	-0.603027317	0.172363697	0.2398868	brain innervation
ENSDART00000084355	zgc:165481	0.000683159	-0.665978496	-0.883055142	-0.199024252	-0.082238068	brain innervation
ENSDART00000084730	zgc:162160	1.57453E-05	-0.758204424	-0.82447566	-0.164736838	0.207922006	brain innervation
ENSDART00000084890	si:ch211-284e13.4	0.000766042	-0.124688162	-0.262617592	0.237393976	0.369141192	brain innervation
ENSDART00000085210	cacna1ha	0.001618544	-0.413195046	-0.405391107	-0.236177845	0.006546496	brain innervation
ENSDART00000085253	mid1	0.001365289	-0.357661223	-0.330080838	-0.002902011	0.078651096	brain innervation
ENSDART00000085472	grm2a	0.000638552	-0.506007863	-0.654883229	-0.268518199	0.087304206	brain innervation
ENSDART00000085565	capn15	0.002791482	-0.382220369	-0.426351921	-0.021041552	0.033591442	brain innervation
ENSDART00000085573	rgs7bpa	0.003138642	-0.750985181	-0.701592527	-0.219110071	-0.146877341	brain innervation
ENSDART00000085743	AL935194.1	0.002127025	-0.774745358	-0.68914542	-0.32626184	0.039334462	brain innervation
ENSDART00000086617	gabbr2	1.88318E-05	-0.577324776	-0.926125922	-0.433004274	0.052999603	brain innervation
ENSDART00000086797	adgrl3.1	0.00088836	-0.114053477	-0.068166121	0.465121947	0.57391191	brain innervation
ENSDART00000087118	xylt1	0.004031897	-0.272537601	-0.39007956	-0.067871098	0.053209242	brain innervation
ENSDART00000087148	cbln4	0.000138972	-0.802680954	-0.769511703	-0.146403482	0.304907793	brain innervation
ENSDART00000087191	mark4a	0.000877772	-0.415170271	-0.372344968	0.042679549	0.108705644	brain innervation
ENSDART00000087280	cacnb3a	0.000177237	-0.323388935	-0.465191463	0.268224123	0.533679905	brain innervation
ENSDART00000087426	bcl11aa	3.5708E-05	-0.367520296	-0.334252656	0.232372171	0.511638417	brain innervation
ENSDART00000087441	GFOD1	0.020470078	-0.421601041	-0.219799435	-0.160457067	0.058971995	brain innervation
ENSDART00000087586	c2cd4a	0.000100528	-0.882018106	-1.482044104	-0.368555759	0.13043866	brain innervation
ENSDART00000087857	unc5db	0.000103226	-0.464548804	-0.771046574	-0.267052184	0.0901494	brain innervation
ENSDART00000088159	nrxn1a	0.00486688	-0.55423697	-0.307440551	0.007663974	-0.036725507	brain innervation
ENSDART00000088178	nrxn1a	0.000255127	-0.424942944	-0.357890595	-0.01594532	0.150075885	brain innervation
ENSDART00000088199	zgc:162707	0.004813371	-0.481388546	-0.51007365	-0.179950315	-0.039369231	brain innervation
ENSDART00000088249	hcn4l	0.001402519	-0.487910369	-0.340719397	0.02727434	0.039415999	brain innervation
ENSDART00000088833	si:ch73-233f7.1	0.00031728	-1.214678988	-0.552820604	-0.229157577	0.532052231	brain innervation
ENSDART00000089574	tub	0.003777754	-0.338880988	-0.401453479	-0.101730157	-0.038037712	brain innervation
ENSDART00000089699	prrt1	0.000415146	-0.603201784	-0.702306626	-0.280611263	-0.047540659	brain innervation
ENSDART00000089867	ppp2r2cb	0.018790846	-0.452836704	-0.44451904	-0.189867818	-0.082146078	brain innervation
ENSDART00000090019	zeb2b	0.016845819	-0.694941899	-0.428168304	-0.296660745	0.024845807	brain innervation
ENSDART00000090306	xpr1a	0.001093301	-0.158299732	-0.301120383	0.179396195	0.424625304	brain innervation
ENSDART00000090397	kiaa1549la	4.91848E-05	-0.446832614	-0.575803117	-0.232865535	0.10265337	brain innervation
ENSDART00000090844	zgc:153018	0.006825939	-0.414733046	-0.080390611	-0.214556251	-0.032786964	brain innervation
ENSDART00000091004	pcdh1a	0.000227894	-0.630879138	-0.627674521	-0.227167517	0.17418403	brain innervation
ENSDART00000091489	ppp1r9bb	0.000117869	-0.563706295	-0.346664158	-0.080794638	0.037246954	brain innervation
ENSDART00000091664	apc2	0.011436776	-0.58155429	-0.22615315	0.136652181	0.006999355	brain innervation
ENSDART00000091726	fam78ba	4.91848E-05	-0.542782218	-0.34207093	0.322203074	0.384590985	brain innervation
ENSDART00000091818	tulp4b	4.52852E-05	-0.531343855	-0.277019809	0.128892269	0.551121944	brain innervation
ENSDART00000091955	nrxn2b	0.004348675	-0.363238969	-0.38605701	-0.167716958	0.046918181	brain innervation
ENSDART00000092182	ppm1la	0.000340312	-0.411282562	-0.137536548	0.156346356	0.193635075	brain innervation
ENSDART00000092250	btbd11a	0.005509984	-0.250819977	-0.42148278	-0.144893414	0.093892491	brain innervation
ENSDART00000092257	PLD5	5.26677E-05	-0.97635635	-0.828911686	0.040708181	0.36058098	brain innervation
ENSDART00000092665	srebf1	0.004619636	-0.417658309	-0.274360919	0.114636817	0.022214824	brain innervation
ENSDART00000093310	celf5a	0.002632159	-0.268154664	-0.455004312	0.014815598	0.070405312	brain innervation
ENSDART00000097330	dnm1b	4.69744E-05	-0.51283412	-0.480365631	0.101619237	0.531582232	brain innervation
ENSDART00000097695	cntnap3	0.004847659	-0.160493697	-0.529313782	-0.088115665	0.248028839	brain innervation
ENSDART00000097935	si:dkey-226m8.10	0.000254175	-0.692305988	-0.817395111	-0.44855289	0.050175879	brain innervation
ENSDART00000098082	GJD2 (1 of many)	7.52983E-05	-0.795483498	-0.746707283	0.031300804	0.456345915	brain innervation
ENSDART00000098311	KCNJ4	0.008555913	-0.486217707	-0.434414335	-0.027097558	0.056787682	brain innervation
ENSDART00000098667	camk2b1	0.000104487	-0.575252799	-0.701444666	-0.414382214	0.077165391	brain innervation
ENSDART00000098727	svopa	0.0001406	-0.499297278	-0.394465259	0.04106692	0.031976449	brain innervation
ENSDART00000098840	ralgps1	0.000405977	-0.525377749	-0.528705894	-0.140236147	0.156796103	brain innervation
ENSDART00000098859	neurod6a	0.000108364	-0.523659161	-0.308368621	0.583701544	0.979244768	brain innervation
ENSDART00000099019	tmem91	2.12106E-05	-0.661722152	-0.699132453	-0.1920109	-0.057277853	brain innervation

ENSDART00000099049	NA	0.000503033	-0.472159773	-0.420532241	-0.250637325	-0.02437969	brain innervation
ENSDART00000099283	dalrd3	0.001372006	-0.272196688	-0.367831508	-0.108158836	0.07067938	brain innervation
ENSDART00000099325	si:dkey-27p18.5	3.38347E-05	-0.821017867	-0.681399645	-0.140207831	0.113491694	brain innervation
ENSDART00000099607	slc6a17	0.006359324	-0.493487825	-0.358976735	-0.156955324	0.010739948	brain innervation
ENSDART00000099872	slc17a6b	0.000184957	-0.645728405	-0.242319052	0.108083805	0.273480994	brain innervation
ENSDART00000099934	kcnc1a	0.023562909	-0.987934197	-1.510396524	-0.387995788	0.170469595	brain innervation
ENSDART00000100022	H2AFX (1 of many)	0.004391006	-0.512580891	-0.39296931	-0.220510282	-0.111022185	brain innervation
ENSDART00000100103	acss2l	0.006451154	-0.342716857	-0.308956489	-0.115247207	0.030743328	brain innervation
ENSDART00000100322	kcnh5b	4.91848E-05	-0.532935362	-0.444322241	-0.174846578	0.219995697	brain innervation
ENSDART00000100444	fam19a5a	8.2583E-05	-0.63242802	-0.536601669	-0.265992198	0.12320759	brain innervation
ENSDART00000100596	pcdh1a6	0.018324408	-0.483816366	-0.257584823	-0.06041051	0.00168094	brain innervation
ENSDART00000100658	esrra	8.74195E-05	-0.571475474	-0.395296451	0.169003635	0.212368184	brain innervation
ENSDART00000100743	cttnbp2	0.001176693	-0.40355339	-0.391763552	0.009167842	0.081098	brain innervation
ENSDART00000101070	dachd	0.00434437	-0.296270747	-0.351582482	-0.130791564	-0.009393292	brain innervation
ENSDART00000101134	khdrbs2	0.003070422	-0.661640499	-0.469301828	-0.265343985	-0.029039929	brain innervation
ENSDART00000101477	emp1	0.0002611	-0.241905322	-0.037268798	0.203715295	0.377786296	brain innervation
ENSDART00000101603	kidins220b	0.004633928	-0.01985848	-0.087358266	0.173253678	0.374974923	brain innervation
ENSDART00000101627	IGLON5	0.000503033	-0.423175903	-0.742308046	-0.313681293	0.102626627	brain innervation
ENSDART00000101631	satb1b	0.000172812	-0.360112936	-0.384116906	0.134197196	0.481946288	brain innervation
ENSDART00000101653	CU639469.1	2.18017E-05	-0.529716959	-0.505372118	-0.050085906	0.195515782	brain innervation
ENSDART00000101658	ppp1r1b	0.001434846	-0.639757199	-0.267805539	-0.098160681	-0.228099263	brain innervation
ENSDART00000102368	grin1a	4.69744E-05	-0.57807298	-0.694484674	-0.374619925	0.025284051	brain innervation
ENSDART00000102455	gucy1a3	0.001644836	-0.52073984	-0.506169014	-0.27571082	0.101070807	brain innervation
ENSDART00000102562	ankrd10b	0.000614271	-0.585231519	-0.124121182	0.139197554	0.179030577	brain innervation
ENSDART00000102782	gria2a	0.000180202	-0.472482927	-0.595061919	-0.203521695	0.050111807	brain innervation
ENSDART00000103043	nsfa	0.000872721	-0.472055845	-0.418381067	-0.313792962	-0.037512478	brain innervation
ENSDART00000103151	dlgap3	0.004847659	-0.304185948	-0.430889474	-0.103996234	0.188728565	brain innervation
ENSDART00000103407	tmem245	0.001938223	-0.353429601	-0.331655767	-0.089201446	0.066435984	brain innervation
ENSDART00000103750	fam131bb	0.000282529	-0.555314971	-0.519589863	-0.143114917	0.156539518	brain innervation
ENSDART00000104135	gabra5	1.15646E-05	-0.398056951	-0.510180941	0.262181183	0.751061332	brain innervation
ENSDART00000104188	igsf21b	0.000907455	-0.476667576	-0.274511883	-0.073843861	0.013583331	brain innervation
ENSDART00000104289	rab3ab	0.00027551	-0.744433679	-0.710299004	-0.400971594	-0.021261155	brain innervation
ENSDART00000104327	vsx1	0.00488592	-0.248525466	-0.619641572	-0.034766195	-0.028538746	brain innervation
ENSDART00000104751	mxi1	0.002350825	-0.384587031	-0.210909301	0.22179917	0.104111801	brain innervation
ENSDART00000104895	rgs7a	1.96612E-05	-0.76773786	-0.54211529	-0.053731925	0.298916153	brain innervation
ENSDART00000104933	eepd1	0.033087133	-0.181346781	-0.362247495	-0.01782264	0.011720383	brain innervation
ENSDART00000104950	atp1a3a	5.18727E-05	-0.469887861	-0.514207298	-0.064097938	0.181102676	brain innervation
ENSDART00000105484	si:ch211-216b21.2	0.000149497	-0.960990997	-0.619204359	-0.135410073	0.236742995	brain innervation
ENSDART00000105588	pcdh1b	0.006658285	-0.246217756	-0.446101289	-0.039720059	0.09005245	brain innervation
ENSDART00000105743	ntng1a	0.000844413	-0.262343031	-0.343435025	-0.06227485	0.248931143	brain innervation
ENSDART00000105753	olfm3a	0.008447718	-0.365975807	-0.465135675	-0.139223772	0.043379292	brain innervation
ENSDART00000105774	ek1	0.000784035	-0.422572147	-0.621215995	-0.219935537	-0.023014864	brain innervation
ENSDART00000105932	si:dkeyp-110e4.11	6.00847E-05	-0.775118699	-0.662012678	-0.111191342	0.348802036	brain innervation
ENSDART00000106166	rx1	0.002317622	-1.001905061	-0.358282402	0.191592029	-0.031411117	brain innervation
ENSDART00000106619	nrn1a	0.000319291	-1.225032386	-1.272422646	-0.361032349	0.032061464	brain innervation
ENSDART00000108535	gabrb1	0.000145808	-1.10767094	-1.249599593	-0.238929019	0.417524871	brain innervation
ENSDART00000108729	adamtsl2	0.000110744	-0.842317462	-0.70962753	0.040474933	0.841307736	brain innervation
ENSDART00000108736	adam12	0.000825994	-0.319460786	-0.316431452	0.445307645	0.87790832	brain innervation
ENSDART00000108808	frem2a	6.41137E-06	-0.381822233	-0.539670066	0.662155423	0.974116851	brain innervation
ENSDART00000108818	arhgap32a	0.00053265	-0.576365369	-0.434660146	-0.258610983	0.095307278	brain innervation
ENSDART00000109029	map6a	0.002736161	-0.330392405	-0.354970167	-0.034172445	0.05340091	brain innervation
ENSDART00000109040	gpr158a	0.001331938	-0.331785981	-0.632662732	-0.236983197	0.105308258	brain innervation
ENSDART00000109243	sema4bb	0.000494919	-0.591195487	-0.421350422	-0.25044186	-0.118505649	brain innervation
ENSDART00000109356	klf7a	0.000177237	-0.7063233	-0.659659661	-0.044315321	0.157900055	brain innervation
ENSDART00000109507	RAP1GDS1	0.00047864	-0.399835735	-0.351308804	0.076766568	0.119897865	brain innervation
ENSDART00000109511	si:ch211-186j3.6	0.020586406	-0.164289524	-0.357536961	-0.081167433	0.02827477	brain innervation
ENSDART00000109568	pip4k2ab	0.008492808	-0.31785183	-0.37156543	-0.209120418	0.064881056	brain innervation
ENSDART00000109833	zmat3	0.001153184	-0.462380241	-0.737159777	-0.151865097	0.090633241	brain innervation
ENSDART00000110004	frmpd3	0.000503033	-0.530320903	-0.511652794	-0.387982186	0.071931622	brain innervation
ENSDART00000110061	spock2	0.000177237	-0.746309396	-0.624301954	-0.158480844	0.116789254	brain innervation
ENSDART00000110126	cacng3b	0.000230274	-0.394325343	-0.354117689	-0.012321564	0.185829622	brain innervation
ENSDART00000110136	cntnap5b	0.000354446	-0.584561492	-0.553235513	-0.330365654	0.070638148	brain innervation
ENSDART00000110279	si:dkey-183c6.8	0.000114847	-0.455316373	-0.174866477	0.555526708	0.790514442	brain innervation
ENSDART00000110409	frmd5	0.001654051	-0.554751162	-0.59840736	-0.384150671	0.071106799	brain innervation
ENSDART00000110503	adam11	1.36144E-05	-0.835498408	-0.874358127	0.055154913	0.615136144	brain innervation
ENSDART00000110590	VSTM2B	0.004543629	-0.57346103	-0.627600936	-0.34222959	-0.040178972	brain innervation
ENSDART00000110679	lrfn5b	2.98725E-05	-0.958299909	-0.447121499	0.131848444	0.317540452	brain innervation
ENSDART00000110734	FAM163A	0.001644836	-0.371556039	-0.423320211	-0.272260914	0.021575603	brain innervation
ENSDART00000110751	tiam1a	3.21969E-05	-0.473694473	-0.373415926	0.157725394	0.265421677	brain innervation
ENSDART00000111131	elfn1b	0.001021091	-1.012125284	-0.755320746	-0.301892543	0.27076153	brain innervation
ENSDART00000111146	si:ch211-26b3.4	0.000598789	-0.414256231	-0.420417531	-0.178854692	0.040407002	brain innervation
ENSDART00000111156	pdp1	0.000503033	-0.641430349	-0.338633085	-0.014499951	0.034332362	brain innervation
ENSDART00000111203	tagln3a	0.000536544	-0.530904757	-0.489741378	-0.221881281	-0.037658487	brain innervation
ENSDART00000111301	luzp2	0.000592988	-0.629816567	-0.548346899	0.040106065	0.088332731	brain innervation
ENSDART00000111438	mgea5	0.002109252	-0.307275123	-0.024811963	0.160874404	0.159343333	brain innervation

ENSDART00000111506	lrrc75ba	4.11188E-05	-0.649545281	-0.408918781	-0.064365089	0.060080923	brain innervation
ENSDART00000111535	elfn1a	0.001460323	-0.273088167	-0.336173663	-0.097567542	0.139029668	brain innervation
ENSDART00000111639	rereb	0.003012303	-0.168137061	-0.390988508	-0.080335114	0.086629083	brain innervation
ENSDART00000111642	brinp3a.1	7.55931E-05	-0.710663483	-0.69660402	-0.30207639	-0.147819903	brain innervation
ENSDART00000111948	sez6l2	0.000199502	-0.5142749	-0.140866125	0.323444655	0.419947542	brain innervation
ENSDART00000112003	adgrb1a	0.00037001	-0.32135813	-0.631154823	-0.317944877	0.097147786	brain innervation
ENSDART00000112170	unm_hu7912	0.000340312	-0.404814593	-0.573062573	-0.192411138	0.071188401	brain innervation
ENSDART00000112370	ano11	1.45694E-05	-1.080551268	-1.39069517	-0.608655336	0.046438031	brain innervation
ENSDART00000112414	rapgef5a	0.002098034	-0.533059964	-0.605979704	-0.027243653	0.007460247	brain innervation
ENSDART00000112484	nlgn2b	2.18017E-05	-0.707313853	-0.645741453	-0.193542068	0.120518294	brain innervation
ENSDART00000112546	pkn3	0.017988416	-0.415654116	-0.225897422	-0.124759806	-0.079978695	brain innervation
ENSDART00000112671	bicd1a	0.000246292	-0.391567366	-0.28876668	-0.040397452	0.195532163	brain innervation
ENSDART00000112895	FNDC10	3.90156E-05	-0.46103084	-0.039905964	0.379016706	0.57822448	brain innervation
ENSDART00000113081	gpr158b	4.77268E-06	-0.777838913	-0.859733549	-0.109236002	0.425659764	brain innervation
ENSDART00000113171	nlgn1	0.000370918	-0.287475058	-0.532070389	-0.095767589	0.228264127	brain innervation
ENSDART00000113502	si:dkey-84j12.1	4.60467E-05	-0.363232597	-0.10430598	0.492332906	0.665079551	brain innervation
ENSDART00000113511	lzts1	0.000109579	-1.206756297	-0.713910989	-0.061680884	0.474255612	brain innervation
ENSDART00000113551	trim2b	0.0245375	-0.50417548	-0.207383112	-0.112448816	0.043074002	brain innervation
ENSDART00000113773	cdc42bpab	0.002362892	-0.428966416	-0.296406465	0.003528598	0.157857062	brain innervation
ENSDART00000113796	cacnb3b	1.63409E-06	-1.378695476	-1.717513295	-0.09050578	0.468842834	brain innervation
ENSDART00000113864	faxca	0.000400485	-0.531664025	-0.191322961	-0.005943785	0.107092327	brain innervation
ENSDART00000113924	lrrc7	0.000228698	-0.537461037	-0.585108204	-0.303180727	-0.024642369	brain innervation
ENSDART00000113985	mctp1a	0.000375747	-0.392181593	-0.510456941	-0.185251742	0.106694132	brain innervation
ENSDART00000114010	slitrk3a	0.006522424	-0.361859007	-0.532282315	-0.225491041	0.106517685	brain innervation
ENSDART00000114267	map6b	2.98725E-05	-0.567896811	-0.403955591	0.492985119	0.85461741	brain innervation
ENSDART00000114432	ntng2a	0.000596344	-0.38146173	-0.278136767	-0.282818481	0.289761799	brain innervation
ENSDART00000114448	CHST8	0.000248091	-0.44164787	-0.464005415	0.047368929	0.426791372	brain innervation
ENSDART00000114677	si:ch73-62l21.1	0.007349533	-0.323827666	-0.531978643	-0.239685061	0.043755156	brain innervation
ENSDART00000114723	rapgefl1	0.020836881	-0.431713581	-0.430345279	-0.085299154	-0.113952199	brain innervation
ENSDART00000114954	rapgef5b	0.002739168	-0.637101425	-0.566396383	-0.284127257	0.00714868	brain innervation
ENSDART00000114959	cdh24a	0.004579864	-0.51153845	-0.93145045	-0.229616461	0.228723625	brain innervation
ENSDART00000115027	tmem151bb	0.000172675	-0.856524713	-0.708298022	0.291447963	0.495918048	brain innervation
ENSDART00000115089	zgc:171482	0.003451532	-0.437080228	-0.507022457	-0.285662093	0.021798354	brain innervation
ENSDART00000115141	cacnb3b	0.000257119	-1.10918155	-1.536403603	-0.203181521	0.418821499	brain innervation
ENSDART00000115260	si:ch211-113g11.6	0.005572285	-0.558167113	-1.193520332	-0.037233796	0.533309069	brain innervation
ENSDART00000121460	prdm8b	0.00076978	-0.383342673	-0.361617984	-0.117709411	0.067402978	brain innervation
ENSDART00000121708	pcsk1nl	0.000169466	-0.536353064	-0.421583117	-0.086670577	-0.06684806	brain innervation
ENSDART00000121874	nfasca	0.002178187	-0.536181619	-0.366142839	-0.19557692	-0.058427118	brain innervation
ENSDART00000121913	kctd12b	0.015495815	-0.424186634	-0.205906213	-0.149727383	-0.132895958	brain innervation
ENSDART00000122037	rbfox1	0.000487998	-0.419101241	-0.392215043	0.102043249	0.360818016	brain innervation
ENSDART00000122041	nrcama	0.012857389	-0.49531831	-0.707479994	-0.094699246	0.009754423	brain innervation
ENSDART00000122099	dynll2a	0.004627449	-0.463212519	-0.169502826	-0.013591394	0.001709702	brain innervation
ENSDART00000122101	tbx2b	0.001190205	-0.056468629	0.017248499	0.19422425	0.382938949	brain innervation
ENSDART00000122389	elmod1	3.77986E-05	-0.62893484	-0.673357812	-0.159560027	0.32261476	brain innervation
ENSDART00000122574	ppp1r3aa	0.000793313	-0.153287429	-0.684598529	0.022675976	0.569399664	brain innervation
ENSDART00000122905	rac3b	0.000254175	-0.501948401	-0.363291367	-0.120049924	0.166932504	brain innervation
ENSDART00000123839	arrb1	0.000512152	-0.44263739	-0.489445552	-0.281663489	0.083496974	brain innervation
ENSDART00000123851	fut8b	0.013533882	-0.379186004	-0.673089848	-0.195485297	0.141395943	brain innervation
ENSDART00000123868	dthd1	0.020324061	-0.700427011	-0.013611963	-0.073941373	-0.012612052	brain innervation
ENSDART00000124112	pou4f2	2.95145E-05	-1.368026557	-1.308261738	0.344262724	0.908586045	brain innervation
ENSDART00000124331	si:ch1073-174d20.1	0.000107168	-0.948877018	-0.570444361	0.133772468	0.53293925	brain innervation
ENSDART00000124534	mbnl2	0.001000183	-0.407254222	0.078580914	-0.044925118	0.120266311	brain innervation
ENSDART00000124710	dlg5a	0.003770761	-0.356924394	-0.265850728	-0.130233283	0.096340819	brain innervation
ENSDART00000124751	kcnip3b	2.63402E-05	-1.29606503	-1.383048631	-0.490749215	-0.039127508	brain innervation
ENSDART00000124827	lgi2a	0.00013528	-0.389885576	-0.462014195	-0.106597692	0.145509175	brain innervation
ENSDART00000125045	dscama	0.001138119	-0.36310557	-0.486996676	-0.080151729	0.074065642	brain innervation
ENSDART00000125074	kcnab2b	0.001012163	-0.745923366	-1.091983107	-0.420367552	0.000775062	brain innervation
ENSDART00000125284	nlgn2a	0.000673063	-0.210046545	-0.366872132	0.090424981	0.163948503	brain innervation
ENSDART00000125432	esrrd	0.007748762	-0.825323711	-0.251243419	-0.11920169	-0.154136212	brain innervation
ENSDART00000125450	gpc1a	1.08487E-05	-0.40101998	-0.317779496	0.568583174	0.472068591	brain innervation
ENSDART00000125536	appb	0.000152384	-0.473539223	-0.363304525	0.075744005	0.226987466	brain innervation
ENSDART00000125563	homer3b	0.030075636	-0.366344126	-0.72350131	-0.2232749	0.112901448	brain innervation
ENSDART00000125638	tbx5b	0.032861605	-0.961612456	-0.494056529	-0.317150736	-0.11065371	brain innervation
ENSDART00000125733	chst1	0.032601457	-1.779723555	-0.640531075	-0.420086911	-0.327548455	brain innervation
ENSDART00000125759	si:dkey-22o12.2	0.004657196	-0.433356276	-0.386370833	-0.195481295	-0.118191078	brain innervation
ENSDART00000126640	RHOBTB3	0.006456063	-0.934122492	-0.733675156	-0.213973967	-0.180952113	brain innervation
ENSDART00000126744	nrg3b	0.000370918	-0.484773132	-0.477903604	-0.212248622	0.011007147	brain innervation
ENSDART00000126766	erbb4b	0.001365263	-0.378985194	-0.566156911	-0.016423806	0.214915468	brain innervation
ENSDART00000126945	adgrb3	0.006975472	-0.310129925	-0.752937386	-0.198981749	-0.020103794	brain innervation
ENSDART00000127047	tusc5a	2.01848E-05	-1.04192916	-0.556828029	0.377559818	0.604205052	brain innervation
ENSDART00000127136	slco5a1b	0.039811839	-0.145489914	-0.411558687	0.007460079	-0.021875568	brain innervation
ENSDART00000127286	si:ch73-140j24.4	6.73259E-05	-0.467040467	-0.569237917	-0.172853738	0.202142114	brain innervation
ENSDART00000128226	slc6a11b	6.9873E-05	-0.388946235	-0.51506191	-0.246391039	0.364300065	brain innervation
ENSDART00000128271	si:dkey-114c15.7	0.003693464	-0.608254547	-0.511435956	-0.075108717	0.021034155	brain innervation
ENSDART00000128302	stk35	0.014339104	-0.274652044	-0.340334252	-0.095489621	0.00314331	brain innervation

ENSDART00000128690	agap3	0.009899954	-0.218144691	-0.339443752	0.012538674	0.008782978	brain innervation
ENSDART00000128705	ndrg3a	0.000135332	-0.684036937	-0.148509308	0.416765356	0.500717941	brain innervation
ENSDART00000128794	gpm6ab	0.000948605	-0.753205656	-0.206704843	0.388986514	0.547688547	brain innervation
ENSDART00000128839	fez1	0.000920284	-0.463224643	-0.323499809	-0.160691051	0.039189596	brain innervation
ENSDART00000129055	nrg2b	0.00309942	-0.525587506	-0.415280837	-0.153878493	0.206094594	brain innervation
ENSDART00000129248	clstn1	0.000984416	-0.435055335	-0.404808779	-0.072836605	0.145009995	brain innervation
ENSDART00000129511	robo2	6.11958E-05	-0.431501059	-0.512946091	-0.075617078	0.250070953	brain innervation
ENSDART00000129559	btbd3b	0.000261206	-0.741839183	-0.720572279	-0.364214548	0.15940566	brain innervation
ENSDART00000129819	si:ch211-151p13.8	0.000362487	-0.747854488	-0.665734365	-0.328239319	-0.007548317	brain innervation
ENSDART00000129834	nat16	0.000882713	-0.654571013	-0.560511117	-0.238004357	-0.212203603	brain innervation
ENSDART00000129866	pcsk2	0.000314849	-0.608807617	-0.601188495	-0.114458443	0.092104737	brain innervation
ENSDART00000129910	tmem240a	0.000995127	-0.628384792	-0.334786051	0.214415995	0.163191044	brain innervation
ENSDART00000129924	hmgb3a	0.001276923	-0.441161001	-0.289637174	0.049371785	0.069315625	brain innervation
ENSDART00000130007	GABRA2 (1 of many)	7.10385E-05	-0.583670012	-0.533882123	-0.170964807	0.218833255	brain innervation
ENSDART00000130103	kctd16a	1.73574E-05	-0.864638484	-0.862435538	0.056246994	0.316558654	brain innervation
ENSDART00000130125	gnao1a	0.000604807	-0.213616659	-0.159180808	0.167107834	0.384555308	brain innervation
ENSDART00000130310	epb41l3a	0.00059038	-0.373985055	-0.254132145	0.194766582	0.26124696	brain innervation
ENSDART00000130573	atp6ap1la	0.001946729	-0.419066331	-0.227150493	-0.139216735	0.064701891	brain innervation
ENSDART00000131075	dusp23b	0.033109676	-0.349967426	-0.590231643	0.026826234	-0.132825162	brain innervation
ENSDART00000131126	bcl6a	0.005688069	-0.096649799	-0.044696983	0.295011601	0.436349263	brain innervation
ENSDART00000131143	sept5b	0.000106562	-0.480326507	-0.332562488	0.283118689	0.526750102	brain innervation
ENSDART00000131579	ptprdb	7.68811E-06	-0.516674931	-0.642051878	0.273217974	0.528486925	brain innervation
ENSDART00000131714	mbnl2	0.008848885	-0.406601788	-0.032543807	-0.091713267	0.092369986	brain innervation
ENSDART00000131736	kcnq2a	2.08005E-05	-0.749729315	-0.34933354	0.575885203	0.723193716	brain innervation
ENSDART00000132027	spock2	0.000219336	-0.61060119	-0.435727551	-0.163124474	0.204372495	brain innervation
ENSDART00000132123	thsd7ba	0.001248576	-0.490674171	-0.551076996	-0.028899921	-0.007032812	brain innervation
ENSDART00000132278	rnf144ab	0.012297973	-0.480742338	-0.392838261	-0.157627213	-0.112649454	brain innervation
ENSDART00000132294	fut8a	0.003138642	-0.131220853	-0.116575506	0.125377151	0.342318079	brain innervation
ENSDART00000132342	kiaa1549lb	0.011357239	-0.130776123	-0.485143208	-0.118054479	0.086040071	brain innervation
ENSDART00000132471	csmd2	6.2134E-05	-0.459001483	-0.911607393	-0.371722747	0.062349627	brain innervation
ENSDART00000132543	klhdc8b	0.004230106	-0.423366405	-0.574887213	-0.139148992	-0.034877401	brain innervation
ENSDART00000132583	nrxn2a	0.001331938	-0.296541609	-0.578930697	-0.085512446	0.138469202	brain innervation
ENSDART00000132685	kcnn1b	0.00155515	-1.625205585	-1.495755896	-0.13719571	0.28532188	brain innervation
ENSDART00000132691	adgrb3	0.007781888	-0.413275118	-0.450913383	-0.19602447	0.067293319	brain innervation
ENSDART00000132930	pcbp3	0.000849348	-0.89548835	-0.955377646	-0.326630607	0.075971616	brain innervation
ENSDART00000133092	st6galnac5a	0.000503033	-0.798487612	-0.408716424	-0.156113795	0.220161008	brain innervation
ENSDART00000133223	COQ10A	0.001579476	-0.381890626	-0.12435669	0.152677483	0.108453147	brain innervation
ENSDART00000133330	fam214b	0.006799542	-0.307417712	-0.32820559	-0.047756819	0.003374146	brain innervation
ENSDART00000133385	lrp1ba	0.000155554	-0.614935587	-0.929322267	-0.360685611	0.193952764	brain innervation
ENSDART00000133504	dab2ipb	0.019374625	-0.309610082	-0.535236809	-0.062134515	0.075905091	brain innervation
ENSDART00000133956	phactr3a	0.000149865	-0.466148377	-0.040906797	0.400008422	0.572153181	brain innervation
ENSDART00000133985	kcnd1	0.000248125	-1.187342293	-1.392518344	-0.747443213	-0.002522665	brain innervation
ENSDART00000134035	cntnap5b	0.005466942	-1.01200416	-1.154440105	-0.403471674	0.139198923	brain innervation
ENSDART00000134261	dnm1b	1.07133E-05	-0.433650537	-0.451510325	0.138870574	0.465815695	brain innervation
ENSDART00000134307	dclk1a	0.001654051	-1.448196855	-0.41548572	-0.010990365	-0.586401585	brain innervation
ENSDART00000134475	gdap1	0.004885039	-0.459369959	-0.408869462	-0.283212336	-0.008346648	brain innervation
ENSDART00000134564	srsf5b	0.01711161	-0.27445076	-0.31745813	-0.113899297	-0.080138136	brain innervation
ENSDART00000134715	elmod1	5.02856E-05	-1.115600356	-1.115324879	-0.105456948	0.485174818	brain innervation
ENSDART00000134722	bcl2l13	0.00372102	-0.594192147	-0.544774096	-0.184876076	-0.124896832	brain innervation
ENSDART00000134816	slc2a3a	0.00067643	-0.406786727	-0.444029943	-0.052958142	0.129218418	brain innervation
ENSDART00000134832	rbpms2b	0.003328957	-1.576691045	-2.099790887	-0.701820765	0.187979586	brain innervation
ENSDART00000134855	efr3bb	0.003310987	-0.263384976	-0.381748192	-0.1453091	0.002074194	brain innervation
ENSDART00000134973	pcnxl2	7.10385E-05	-0.772294262	-0.54715302	-0.00593982	0.313205165	brain innervation
ENSDART00000135052	slc8a4a	0.003870205	-0.449869312	-0.676457859	-0.284767037	0.064060964	brain innervation
ENSDART00000135284	vsnl1a	3.95966E-05	-0.802778535	-0.782046001	-0.520311896	-0.04073792	brain innervation
ENSDART00000135475	cadm2a	0.000283607	-0.589549243	-0.825400299	-0.33568035	0.065563209	brain innervation
ENSDART00000135513	rgs6	0.001782169	-0.368608418	-0.140264684	-0.010741291	0.084818744	brain innervation
ENSDART00000135602	si:ch73-290k24.6	0.008047992	-0.456455854	-0.524968049	-0.128888416	-0.002715321	brain innervation
ENSDART00000135624	FAM163A	0.011945865	-0.714036121	-0.427639458	-0.142310427	0.163241634	brain innervation
ENSDART00000135692	UNC13A	0.006141198	-0.209420413	-0.513653337	-0.217224189	0.093101705	brain innervation
ENSDART00000135702	rgs11	0.000709831	-0.576481143	-0.793334647	-0.383725367	0.048951277	brain innervation
ENSDART00000135715	fam184a	0.000322759	-0.344643213	-0.481218539	-0.282456424	0.057932244	brain innervation
ENSDART00000135764	si:dkey-70p6.1	0.000167073	-0.54777588	-0.724047584	0.062614493	0.292769909	brain innervation
ENSDART00000135768	lrp1ba	4.91848E-05	-0.685782746	-0.945707299	-0.285013804	0.195853428	brain innervation
ENSDART00000135811	atp2b3a	0.000535509	-0.361290503	-0.436904447	-0.166639435	0.060424957	brain innervation
ENSDART00000136123	kdm6bb	0.024284969	-0.32427763	-0.244056677	-0.170049978	-0.028150527	brain innervation
ENSDART00000136154	gpr158a	0.014511767	-0.522434846	-0.680761714	-0.064607745	-0.081447185	brain innervation
ENSDART00000136233	TENM3	2.33589E-05	-0.571502608	-0.593065001	0.242250375	0.436842415	brain innervation
ENSDART00000136269	rimbp2	0.016247803	-0.196271668	-0.410552217	-0.160743106	0.064316711	brain innervation
ENSDART00000136488	zmat4a	0.008573382	-0.690962776	-0.637104526	-0.28999313	-0.088561559	brain innervation
ENSDART00000136578	syn1	4.91848E-05	-0.141221338	-0.191909782	0.363564902	0.481540125	brain innervation
ENSDART00000136708	rbpms2b	1.12792E-05	-1.024689498	-0.80090545	-0.103078191	0.159293341	brain innervation
ENSDART00000136754	cdh13	0.000634488	-0.587587145	-0.627840519	-0.172128457	0.142910694	brain innervation
ENSDART00000136759	prdm13	0.00326136	-0.225066145	-0.413438688	-0.129520305	0.036146962	brain innervation
ENSDART00000137037	iqsec3b	0.006778044	-0.405090433	-0.576572578	-0.30180595	0.030413937	brain innervation

ENSDART00000137353	klhl17	0.00053559	-0.368352542	-0.314849207	-0.039056928	0.155772163	brain innervation
ENSDART00000137355	tet3	0.033062691	-0.245273707	-0.407219331	-0.050664281	-0.06569376	brain innervation
ENSDART00000137756	hecw2a	3.29362E-06	-0.728856656	-1.003560302	-0.22088311	0.383324077	brain innervation
ENSDART00000137889	ank1b	0.002868986	-0.447220804	-0.737203628	0.205089912	0.418795119	brain innervation
ENSDART00000137900	grin2ab	2.59942E-05	-0.452993272	-0.616161489	0.576592075	1.050321835	brain innervation
ENSDART00000138048	pbx3b	0.007841242	-0.599655595	-0.602159282	-0.228775798	-0.019503135	brain innervation
ENSDART00000138294	rab41	0.000446102	-0.246606124	-0.179576466	0.274617961	0.438688085	brain innervation
ENSDART00000138540	ttyh1	5.26677E-05	-0.563796304	-0.437755604	-0.092270149	0.165178407	brain innervation
ENSDART00000138695	ank1b	0.000591348	-0.498943842	-0.553539638	-0.03799849	0.33423854	brain innervation
ENSDART00000138834	mab21l3	0.00420635	-0.591171056	-0.506133859	-0.165242719	-0.104286764	brain innervation
ENSDART00000138850	cacna2d2b	0.008428026	-0.226630579	-0.433395437	-0.06209639	0.11961013	brain innervation
ENSDART00000139035	si:ch211-215c18.3	1.2512E-05	-1.205532305	-0.512285433	0.437440349	0.984798646	brain innervation
ENSDART00000139178	slc25a14	0.001193104	-0.405048408	-0.151867564	-0.020130795	0.082212943	brain innervation
ENSDART00000139265	si:ch211-12e13.12	0.00026634	-0.4797238	-0.27645528	0.604278273	0.83384752	brain innervation
ENSDART00000139279	rbpms2b	0.001175355	-0.774960547	-0.830410704	0.12323811	0.508122144	brain innervation
ENSDART00000139448	GALNTL6	0.000882263	-0.494238764	-0.383984121	-0.129586789	0.079582323	brain innervation
ENSDART00000139475	samd10b	0.001021091	-0.569533963	-0.524261904	-0.125238267	-0.046566236	brain innervation
ENSDART00000139493	olfm1b	0.002701643	-0.400757951	-0.50188464	-0.148663949	0.07973127	brain innervation
ENSDART00000139514	ano11	0.001023352	-1.063704055	-1.17363318	-0.628817004	-0.055546726	brain innervation
ENSDART00000139795	syt2a	7.6604E-05	-0.704240682	-0.905957314	-0.438675098	0.054000022	brain innervation
ENSDART00000139841	gabrb1	0.000278329	-0.439523493	-0.431067027	-0.18481289	0.263500104	brain innervation
ENSDART00000139954	BX005364.1	0.014034602	-0.374788931	-0.616534875	-0.121001872	0.068005074	brain innervation
ENSDART00000140161	necab2	2.33589E-05	-0.820451686	-0.597146212	0.232199679	0.59216621	brain innervation
ENSDART00000140365	slc12a5b	0.002585408	-0.506920714	-0.535232712	-0.246533716	0.015331251	brain innervation
ENSDART00000140436	map2	0.004423084	-0.405015536	-0.195771527	-0.230781013	0.041022881	brain innervation
ENSDART00000140476	atp6v0a2a	0.000363306	-0.389741082	0.011917076	0.174652763	0.265809184	brain innervation
ENSDART00000140575	map4l	0.032473859	-0.647123698	-0.438384786	-0.184748643	-0.024632978	brain innervation
ENSDART00000140739	lrrc4bb	0.029601456	-0.565179268	-0.464872302	-0.152447222	-0.079782637	brain innervation
ENSDART00000140827	bcl11ab	0.000602987	-0.293093875	-0.330078462	0.796150714	1.216507448	brain innervation
ENSDART00000141086	robo2	0.010303026	-0.237929535	-0.432116468	-0.02855362	0.131332743	brain innervation
ENSDART00000141103	gfra4a	0.0021138	-0.446789167	-0.472299982	-0.170582645	0.04427624	brain innervation
ENSDART00000141752	asphd1	0.007785924	-0.390703626	-0.359372328	-0.291984856	0.011586147	brain innervation
ENSDART00000141779	fndc5a	0.000119544	-1.597130885	-1.939288998	-0.836516829	0.004414498	brain innervation
ENSDART00000141917	ank1a	3.03667E-05	-1.167872922	-1.059135645	-0.600851313	-0.041118915	brain innervation
ENSDART00000141958	cdh24a	0.005763869	-0.328998631	-0.596758779	-0.216164592	0.127161048	brain innervation
ENSDART00000142061	si:dkey-32e23.4	0.001867076	-0.369490073	-0.293305321	0.04108125	0.132687244	brain innervation
ENSDART00000142087	foxp2	0.000565675	-0.522019738	-0.4194261	0.065656049	0.27497631	brain innervation
ENSDART00000142122	cplx2l	0.000340549	-1.429903189	-0.89072761	0.133226492	0.114783163	brain innervation
ENSDART00000142140	ppfia2	0.001332606	-0.37587085	-0.48411577	0.218087957	0.20796301	brain innervation
ENSDART00000142141	grin2aa	0.000227894	-0.499492643	-0.789969038	-0.312539264	0.08079802	brain innervation
ENSDART00000142489	slco5a1b	0.001879617	-0.38890353	-0.329924623	-0.120653024	-0.00920305	brain innervation
ENSDART00000142524	chrnb2a	9.33064E-06	-0.869143617	-0.99350722	-0.040693199	0.426386933	brain innervation
ENSDART00000142653	grm1a	0.003262292	-0.469462455	-0.467735907	-0.088400916	0.178280203	brain innervation
ENSDART00000143152	cpne5a	0.000387877	-0.664046359	-0.825602355	-0.316663455	0.050239809	brain innervation
ENSDART00000143286	lnx1	0.005464372	-0.74422542	-0.772813048	-0.069056217	0.13158415	brain innervation
ENSDART00000143410	EMB	2.1041E-05	-1.826210099	-1.592080235	-0.486327336	0.178400158	brain innervation
ENSDART00000143938	sema3ga	0.000230274	0.034230149	-0.448431565	0.102011548	0.447499442	brain innervation
ENSDART00000143969	BX247868.1	6.23756E-05	-1.179119979	-1.430780681	-0.233116942	0.342359274	brain innervation
ENSDART00000144050	NA	0.025646401	-0.572877113	-0.701246998	-0.196460214	-0.040487474	brain innervation
ENSDART00000144186	pou6f2	7.05055E-05	-0.474463335	-0.465571046	0.092941552	0.436081081	brain innervation
ENSDART00000144215	pcnxl2	1.45694E-05	-0.697249288	-0.448197444	0.040412938	0.382373048	brain innervation
ENSDART00000144361	ank1b	2.46332E-05	-0.731673078	-0.825040806	0.086374477	0.310723345	brain innervation
ENSDART00000144894	bcl11ba	3.52427E-05	-0.656989822	-0.679320488	0.181338486	0.623847694	brain innervation
ENSDART00000145096	fam219ab	0.007936937	-0.366547695	-0.337752539	-0.191344563	-0.015845187	brain innervation
ENSDART00000145108	MFF (1 of many)	0.004863057	-0.387524941	-0.341112286	-0.251149344	-0.013627184	brain innervation
ENSDART00000145114	ptprsa	0.011196176	-0.133739633	-0.353332073	-0.057919239	0.041724305	brain innervation
ENSDART00000145434	gpr52	0.010169943	-0.413465096	-0.575440094	-0.276023467	-0.017738845	brain innervation
ENSDART00000145489	ano11	0.000184045	-1.065264341	-1.355279668	-0.34443399	0.075205433	brain innervation
ENSDART00000145616	adarb1b	8.45506E-05	-0.549552658	-0.593167659	-0.056719481	0.082749855	brain innervation
ENSDART00000145775	mpp4a	0.007561534	-0.462462268	-0.206618846	-0.102159961	0.056169031	brain innervation
ENSDART00000145777	adgrb3	0.018611393	-0.131394962	-0.455026919	-0.13584478	0.067074927	brain innervation
ENSDART00000145809	prkag3a	0.029695868	-4.153185554	-1.353616268	-0.923701192	-0.043754788	brain innervation
ENSDART00000145835	tmx3	0.000403881	-0.913440216	0.029499449	0.308787653	0.34181057	brain innervation
ENSDART00000146055	ptchd4	6.99374E-05	-0.534340079	-0.556417786	0.177414073	0.199962898	brain innervation
ENSDART00000146120	luzp2	0.000370918	-0.491780393	-0.439244253	0.071649151	0.105029542	brain innervation
ENSDART00000146132	si:ch211-254n4.3	0.000799784	-0.407187074	-0.522964952	-0.215771966	0.042955183	brain innervation
ENSDART00000146530	epha6	0.000442465	-0.320688922	-0.528894361	-0.185025461	0.089827402	brain innervation
ENSDART00000146785	tmem163b	2.46332E-05	-0.68540579	-0.770110571	-0.115396875	0.356833449	brain innervation
ENSDART00000147001	si:dkeyp-72h1.1	0.00059038	-0.667988087	-0.713655804	-0.172938308	0.232582151	brain innervation
ENSDART00000147105	tdh	0.003138642	-0.645059373	-0.498416454	-0.202501341	-0.068176647	brain innervation
ENSDART00000147188	trpm3	0.001151232	-0.625119804	-0.737554924	-0.108574358	0.285413653	brain innervation
ENSDART00000147218	efna3a	0.00377485	-0.679676128	-0.850928012	-0.177104508	-0.025688419	brain innervation
ENSDART00000147464	ryr3	4.24705E-05	-0.823352364	-0.716491782	0.05941278	0.608108075	brain innervation
ENSDART00000147483	ephb6	0.000402225	-0.346946617	-0.195722816	0.204471928	0.396713027	brain innervation
ENSDART00000147699	mical3b	0.021599545	0.112525626	0.108456465	0.226887131	0.765699718	brain innervation

ENSDART00000147799	ndrg3b	0.015070633	-0.751369578	-0.184479497	0.170135881	-0.060187322	brain innervation
ENSDART00000147831	ACAP2 (1 of many)	0.000728778	-0.265239588	-0.086744823	0.171263201	0.342225898	brain innervation
ENSDART00000147998	plxna2	0.007335615	-0.136413264	-0.347150332	-0.077394761	0.05783618	brain innervation
ENSDART00000148055	si:ch211-67e16.11	0.000248091	-0.532227459	-0.535880084	-0.161015502	0.246252593	brain innervation
ENSDART00000148107	SRCIN1	5.24617E-05	-0.358492187	-0.594267639	-0.130298738	0.102935385	brain innervation
ENSDART00000148120	ephb2a	5.7178E-05	-0.204590684	-0.196760275	0.359222548	0.385663441	brain innervation
ENSDART00000148199	stxbp1a	0.002936628	-0.541285454	-0.573795052	-0.292066803	-0.020658402	brain innervation
ENSDART00000148405	si:ch211-113g11.6	0.005927492	-0.63339569	-0.861477951	-0.083811842	0.259008105	brain innervation
ENSDART00000148465	slc1a1	3.38347E-05	-0.751249091	-0.22594806	0.456533505	0.753777978	brain innervation
ENSDART00000148580	BX005108.2	0.002349998	-0.474486107	-0.575204555	-0.246024325	-0.012613897	brain innervation
ENSDART00000148590	coro2ba	0.007621679	-0.3608902	-0.079968694	-0.141782791	0.048751465	brain innervation
ENSDART00000148653	tmem145	0.019023689	-0.454635973	-0.21216688	-0.078241248	0.035128268	brain innervation
ENSDART00000148662	kcnc3a	0.003848394	-0.800710128	-1.106624714	-0.368118634	0.013489304	brain innervation
ENSDART00000148900	kcnc2	0.002497064	-0.819802341	-0.972756522	-0.275729768	0.337871594	brain innervation
ENSDART00000149032	oat	0.006013085	-0.368235431	-0.40939103	-0.17630927	-0.009811502	brain innervation
ENSDART00000149158	dmxl2	0.001023352	-0.239475556	-0.38571718	-0.12120231	0.150747303	brain innervation
ENSDART00000149175	pou4f3	3.33896E-05	-1.520089487	-1.231379123	0.357488642	0.743891028	brain innervation
ENSDART00000149276	lzts3b	0.009414182	-0.642406039	-0.470964243	-0.143114993	-0.054979203	brain innervation
ENSDART00000149283	zgc:112001	0.000242559	-0.707617666	-0.492468931	0.006563399	0.231921747	brain innervation
ENSDART00000149335	kcna1a	7.55931E-05	-0.92291358	-0.966385056	-0.49989391	-0.001249229	brain innervation
ENSDART00000149367	si:ch73-290k24.5	0.009217803	-0.213839599	-0.466705261	-0.018322662	0.130528894	brain innervation
ENSDART00000149425	SLC25A22 (1 of many)	0.000236845	-0.428786809	-0.626535356	-0.125118952	0.12464047	brain innervation
ENSDART00000149443	pcdh19	0.000181327	-0.547618951	-0.594468642	-0.345755383	0.11053464	brain innervation
ENSDART00000149552	FP325120.1	0.00320961	-0.456523138	-0.25356238	-0.125718739	0.024031302	brain innervation
ENSDART00000149618	mid1	0.002670678	-0.406831126	-0.160364287	0.131354752	0.136351442	brain innervation
ENSDART00000149666	dock3	0.004782655	-0.065088232	-0.425598201	-0.009057049	0.161364952	brain innervation
ENSDART00000149685	gnao1a	0.000111916	-0.290173142	-0.321971232	0.118032615	0.301423091	brain innervation
ENSDART00000149740	si:ch211-149b19.2	0.000439231	-0.851560484	-0.688459713	0.123433485	0.442881876	brain innervation
ENSDART00000149859	gldn	0.020986271	-0.670802099	-0.540937826	-0.034323087	-0.157128438	brain innervation
ENSDART00000149947	bcl2b	0.000305619	-0.663523207	-0.706570637	-0.112082166	-0.007869569	brain innervation
ENSDART00000149992	si:dkey-231j24.3	0.002102775	-0.792591394	-0.543368341	0.065178142	0.204406436	brain innervation
ENSDART00000150036	adam23a	3.77986E-05	-0.62166293	-0.515362417	0.016147076	0.303932596	brain innervation
ENSDART00000150068	sox6	0.001331938	-0.111707858	-0.047191704	0.355284385	0.700337568	brain innervation
ENSDART00000150146	smarcd3b	0.000677616	-0.318612181	-0.278781268	0.723080439	0.640333876	brain innervation
ENSDART00000150839	fam57bb	0.005203275	-0.627521697	-0.387137362	0.25664183	0.060693872	brain innervation
ENSDART00000150949	nfyc	0.013743075	-0.535274529	-0.301463031	-0.212807295	0.074596031	brain innervation
ENSDART00000150955	bmpr2a	0.012462623	-0.667543016	-0.436786519	-0.136898455	0.053141678	brain innervation
ENSDART00000151252	myo10l1	3.38347E-05	-0.09485067	-0.323915115	0.494165668	0.855040148	brain innervation
ENSDART00000151299	scn8ab	1.02827E-05	-0.339946061	-0.372438378	0.28752079	0.526264643	brain innervation
ENSDART00000151571	ahdc1	0.000666959	-0.753523402	-0.774930454	-0.265860844	-0.025283973	brain innervation
ENSDART00000151648	ntng2b	0.000275413	-0.44606387	-0.406664301	-0.175135105	0.111487092	brain innervation
ENSDART00000151672	si:ch211-276a17.5	0.026710645	-0.716987346	-0.427751363	-0.201345778	-0.037618143	brain innervation
ENSDART00000151674	pcdh9	0.000894529	-0.204552278	-0.440082629	-0.022873178	0.221939437	brain innervation
ENSDART00000151685	lhfpl4a	0.000326965	-0.450299252	-0.419390296	-0.093062661	0.17106537	brain innervation
ENSDART00000151698	syt3	0.000157185	-0.446756628	-0.536044497	-0.128625894	0.106551311	brain innervation
ENSDART00000151842	lrrn3a	2.01848E-05	-0.579197629	-0.548770022	-0.143313453	0.249550285	brain innervation
ENSDART00000151853	btbd10a	0.000362175	-0.220795221	-0.048228521	0.169228662	0.338815973	brain innervation
ENSDART00000151904	foxn3	0.000466769	-0.402174688	-0.533042351	-0.141780798	0.022724604	brain innervation
ENSDART00000151915	ptchd1	6.75287E-05	-0.713611001	-0.66822522	0.042208909	0.328774722	brain innervation
ENSDART00000151920	si:ch211-140m22.7	0.011455954	-0.371308279	-0.473110833	-0.103450206	0.052557108	brain innervation
ENSDART00000151921	dnmt3ab	0.001890284	-0.131014471	-0.104325638	0.10563751	0.340310238	brain innervation
ENSDART00000151970	si:ch211-248l17.3	0.000139914	-0.372087768	-0.337523565	0.171433041	0.720267147	brain innervation
ENSDART00000152011	DOCK4 (1 of many)	0.000227894	-0.107148853	-0.150156526	0.321338768	0.540658105	brain innervation
ENSDART00000152603	egln2	0.018920791	-0.344914112	-0.184325703	-0.029073268	0.070142585	brain innervation
ENSDART00000152924	stxbp4	0.002497064	0.009805274	-0.210366163	0.385226099	0.296155125	brain innervation
ENSDART00000152973	RNF157	0.002439392	-0.237155894	-0.35583425	-0.110722293	0.097030733	brain innervation
ENSDART00000153022	AL935194.1	0.000106562	-0.839883385	-0.952071296	-0.323659944	-0.119202215	brain innervation
ENSDART00000153146	rbfox3a	0.000155554	-0.546449898	-0.43963695	0.174623158	0.484406984	brain innervation
ENSDART00000153284	tbkbp1	0.000630366	-0.504212532	-0.489626186	-0.240468452	0.072471561	brain innervation
ENSDART00000153514	si:ch211-121j5.4	0.006293989	-0.438676742	-0.189457468	-0.187818697	-0.117536785	brain innervation
ENSDART00000153704	anks1ab	3.38532E-05	-0.440221806	-0.75898091	-0.133742531	0.137005152	brain innervation
ENSDART00000153730	CU856222.1	0.000116387	-0.553584022	-0.481855955	-0.007954528	0.343506731	brain innervation
ENSDART00000153824	napba	0.002994106	-0.634929885	-0.324273151	-0.096308603	0.10005339	brain innervation
ENSDART00000153828	atp2b3b	8.36884E-05	-0.474522045	-0.66566516	-0.285016921	0.075707306	brain innervation
ENSDART00000153897	ndnfl	8.26035E-05	-0.781136652	-1.071097643	-0.053351937	0.577095222	brain innervation
ENSDART00000154039	cntnap2a	0.000510711	-0.318660467	-0.524836596	-0.23230439	0.054653452	brain innervation
ENSDART00000154217	tapbpl	6.43148E-05	-0.790387635	-1.110046917	-0.234167162	0.243016032	brain innervation
ENSDART00000154253	VAMP1 (1 of many)	4.24705E-05	-0.590038106	-0.991557836	-0.153916319	0.292977881	brain innervation
ENSDART00000154256	gabbr1a	0.000825994	-0.701390177	-1.072254559	-0.049084095	0.380533583	brain innervation
ENSDART00000154333	camkva	0.000142247	-0.731914874	-0.620456244	-0.460647205	-0.026116956	brain innervation
ENSDART00000154501	cntnap1	0.000112893	-0.715173489	-0.84270145	-0.359147123	0.122603648	brain innervation
ENSDART00000154667	SORCS3	0.000413975	-0.864119649	-0.84034251	-0.370097899	0.056888282	brain innervation
ENSDART00000154748	sv2bb	0.000171543	-0.593359268	-0.537099772	-0.285465428	0.087161334	brain innervation
ENSDART00000154754	grik1b	0.004364661	-0.541985174	-0.220416154	-0.114773141	-0.128231092	brain innervation
ENSDART00000154811	shank1	0.000776925	-0.390024258	-0.720748027	-0.113172669	0.256359072	brain innervation

ENSDART00000154869	cacna1ha	7.43198E-05	-0.561980799	-0.554587419	-0.200655166	0.115415453	brain innervation
ENSDART00000155184	lcorl	0.021296835	-0.199231763	-0.444480785	-0.032192793	-0.024389475	brain innervation
ENSDART00000155397	soat2	0.016354858	-0.618559497	-0.31486995	-0.194806138	-0.125519338	brain innervation
ENSDART00000155494	CR384099.1	0.00148037	-0.702495416	-0.755760921	-0.229440848	0.245420937	brain innervation
ENSDART00000155502	sez6b	0.000937142	-0.382545228	-0.653854879	-0.149982953	0.168281376	brain innervation
ENSDART00000155563	abca5	0.003355787	-0.240041791	-0.35443471	-0.098648169	0.068480985	brain innervation
ENSDART00000155592	pcdh17	0.000461528	-0.283490769	-0.422406754	-0.268768577	0.186259856	brain innervation
ENSDART00000155658	rapgefl1	0.004578126	-0.418362292	-0.397338899	0.000660896	-0.001869225	brain innervation
ENSDART00000155666	znf576.1	0.015256508	-0.372721752	-0.23410815	-0.134923201	-0.013577883	brain innervation
ENSDART00000155757	si:dkey-16p21.8	0.008752524	-0.383053569	-0.315569666	-0.222717784	0.028079211	brain innervation
ENSDART00000155840	nyap2a	0.000605791	-0.526663005	-0.654892136	0.27374848	0.170536216	brain innervation
ENSDART00000155843	cntnap2a	8.92064E-05	-0.263075507	-0.343897302	0.676718804	0.99202798	brain innervation
ENSDART00000155848	CU929046.2	0.004951436	-0.462340311	-0.419607593	-0.082173547	-0.018364502	brain innervation
ENSDART00000155866	zbtb16b	8.488E-05	-0.434457287	-0.595844526	0.044569606	0.015262284	brain innervation
ENSDART00000155935	clstn3	0.000159813	-0.324952015	-0.256429293	0.203826088	0.321978764	brain innervation
ENSDART00000155949	grm8b	0.001007682	-0.426657348	-0.505637682	-0.163630603	0.069819734	brain innervation
ENSDART00000156012	si:dkey-35i13.1	1.41905E-06	-1.182557557	-0.949918392	-0.338171836	-0.056213778	brain innervation
ENSDART00000156341	fam117ba	7.05055E-05	-0.500099563	-0.265235629	0.202099115	0.368226992	brain innervation
ENSDART00000156375	kcnq2b	0.000178718	-0.284091354	-0.197722227	0.27148365	0.512045099	brain innervation
ENSDART00000156380	CR848757.1	2.98725E-05	-0.431298815	-0.377014166	0.404962464	0.865655337	brain innervation
ENSDART00000156393	AL772314.1	6.26753E-05	-0.272422966	-0.157258449	0.218420805	0.368190657	brain innervation
ENSDART00000156570	slc6a17	0.002913561	-0.448593137	-0.394227085	-0.032159276	0.074756396	brain innervation
ENSDART00000156705	si:dkeyp-72g9.4	0.000811547	-0.743143803	-0.697928004	-0.454613917	-0.077239927	brain innervation
ENSDART00000156820	si:dkey-7j14.5	4.77268E-06	-0.971869146	-0.757334904	0.08249331	0.800607437	brain innervation
ENSDART00000156965	osbpl5	5.8648E-05	-0.452639629	-0.228065482	0.296781507	0.346469129	brain innervation
ENSDART00000156982	si:ch211-195b15.8	0.000596344	-0.203622983	-0.158924419	-0.050797883	0.351311882	brain innervation
ENSDART00000157018	stmn3	4.91848E-05	-1.008205118	-1.406964646	-0.116902659	0.621837811	brain innervation
ENSDART00000157125	vsnl1b	2.15557E-05	-0.832964446	-0.803942924	-0.29707501	0.109485311	brain innervation
ENSDART00000157171	si:ch211-168d23.3	0.027638894	-0.276296251	-0.552181073	-0.115547916	-0.061680907	brain innervation
ENSDART00000157237	si:dkey-56f14.7	0.013233841	-0.377561364	-0.203140811	-0.202521092	-0.065412084	brain innervation
ENSDART00000157265	trim13	0.022539179	-0.831738752	-0.32341984	-0.102064486	-0.071474505	brain innervation
ENSDART00000157302	si:dkey-7j14.5	1.31948E-06	-0.795010515	-0.583416344	0.327377123	0.951280694	brain innervation
ENSDART00000157428	pik3r3b	0.001317626	-0.522784766	-0.44851499	-0.152608244	0.028231585	brain innervation
ENSDART00000157449	clstn2	0.000466769	-0.339354151	-0.60478304	0.06475081	0.303731917	brain innervation
ENSDART00000157454	ncam1a	0.005875165	-0.363879899	-0.114400367	0.050687773	0.064981174	brain innervation
ENSDART00000157518	hspa12a	5.66718E-05	-0.767789618	-0.701014156	0.093464144	0.364648116	brain innervation
ENSDART00000157769	pcdh1gc5	0.001135528	-0.403524411	-0.226721593	0.040583925	0.029815654	brain innervation
ENSDART00000157788	CABZ01031894.1	0.004627449	-0.75935498	-0.746140313	-0.275189537	-0.221209558	brain innervation
ENSDART00000158213	akap12b	0.000529346	-1.00724688	-1.146388756	-0.599808188	0.009805796	brain innervation
ENSDART00000158355	bsk146	0.000219336	-0.973617487	-0.7163723	-0.116416923	0.102394026	brain innervation
ENSDART00000158429	tmem42b	0.001002974	-0.433937172	-0.428108528	-0.048957595	0.127616146	brain innervation
ENSDART00000158489	zgc:77058	1.34007E-05	-0.495737975	-0.228595493	0.141443601	0.440592752	brain innervation
ENSDART00000158500	atp8b2	0.000419956	-0.533001796	-0.553651375	-0.17260282	0.179842571	brain innervation
ENSDART00000158618	cpne4b	4.49433E-05	-1.041271467	-0.75854166	0.438341768	1.045239509	brain innervation
ENSDART00000158671	sult4a1	8.51245E-05	-0.462609111	-0.368663113	-0.083169387	0.19862288	brain innervation
ENSDART00000158723	sncb	0.008362897	-0.257429657	-0.430720868	-0.069889176	0.08182323	brain innervation
ENSDART00000158936	grin1a	0.000250511	-0.976292061	-1.003442352	-0.317903979	0.15163936	brain innervation
ENSDART00000159104	atp1b2a	0.000804628	-0.488650552	-0.417675156	-0.129746035	0.084213141	brain innervation
ENSDART00000159249	oxr1a	0.000445792	-0.748854688	-0.518408458	-0.301236362	-0.153948362	brain innervation
ENSDART00000159291	podxl2	0.000184045	-0.45367951	-0.207714244	0.333702352	0.497299671	brain innervation
ENSDART00000159589	rtn4rl1b	0.00013806	-0.618605611	-0.334861623	-0.03219779	0.419833076	brain innervation
ENSDART00000159601	serpini1	0.000352454	-0.44709139	-0.357709199	-0.198408132	0.076823185	brain innervation
ENSDART00000159627	scn4ba	0.000451042	-1.651719386	-1.747876787	-0.730948262	-0.207716251	brain innervation
ENSDART00000159690	gpr75	0.002568245	-0.483974147	-0.377125661	0.037666989	0.123067934	brain innervation
ENSDART00000159693	NYAP1	0.001144712	-0.548984567	-0.516611795	-0.021366781	0.000605996	brain innervation
ENSDART00000159727	pcdh11	0.000494919	-0.294947297	-0.365638864	-0.089140913	0.17419873	brain innervation
ENSDART00000159805	CABZ01085924.1	0.000283607	-0.392684277	-0.456709342	0.004929566	0.18758642	brain innervation
ENSDART00000159853	FQ311890.1	0.000463544	-0.276177481	-0.432407927	-0.032078728	0.27665545	brain innervation
ENSDART00000159942	kcnc3b	0.000847723	-0.794025304	-1.361623092	-0.389946342	0.009706478	brain innervation
ENSDART00000160018	DUSP26	0.00184704	-0.660306321	-0.709450564	-0.129656337	0.007184288	brain innervation
ENSDART00000160138	sobpa	0.003891676	-0.321225255	-0.895188795	-0.069068022	0.312612038	brain innervation
ENSDART00000160350	IQSEC1 (1 of many)	0.012665885	-0.73025949	-0.724161984	-0.227945297	-0.158150827	brain innervation
ENSDART00000160464	pcdh1gb2	0.037611012	-0.570850707	-0.31567681	-0.173184848	-0.165810233	brain innervation
ENSDART00000160466	ndrg4	0.001418732	-0.797536516	-0.447807808	-0.265830716	-0.169347229	brain innervation
ENSDART00000160469	si:ch211-271b14.1	0.005629236	-0.816113506	-0.956386746	-0.419124903	-0.031051269	brain innervation
ENSDART00000160662	CABZ01056578.1	0.001955985	-0.678580777	-0.38089804	-0.298190661	-0.130306976	brain innervation
ENSDART00000160789	SDK2 (1 of many)	0.006197253	-0.531586731	-0.76443238	-0.126364502	0.194536592	brain innervation
ENSDART00000160809	agap1	0.000470359	-0.12094537	-0.11171461	0.245949776	0.322867446	brain innervation
ENSDART00000160811	b3gat1a	0.000188707	-0.509506437	-0.522178413	-0.230803048	0.152925891	brain innervation
ENSDART00000160881	pcdh1g30	0.001490782	-0.53895262	-0.083348601	0.159825551	0.241016465	brain innervation
ENSDART00000160885	stbd1	0.007301761	-0.425801769	-0.465748469	-0.195945725	0.034690374	brain innervation
ENSDART00000161003	cpne9	0.001482057	-1.056006542	-1.193405404	-0.117014879	0.290018934	brain innervation
ENSDART00000161115	CR382281.1	0.004084205	-1.148281272	-0.70950654	-0.315399276	0.210191426	brain innervation
ENSDART00000161137	scn1bb	2.29102E-05	-0.455645009	-0.546610376	0.183837534	0.407926687	brain innervation
ENSDART00000161264	ntrk3b	0.000108364	-0.768053413	-0.724776652	-0.30616772	0.183034577	brain innervation

ENSDART00000161436	pcdh7b	5.5105E-05	-0.687665671	-0.773740554	-0.245502692	0.12030382	brain innervation
ENSDART00000161507	zgc:153426	1.98934E-05	-1.880708803	-0.117657621	0.865737326	1.072245785	brain innervation
ENSDART00000161532	zfyve9b	0.000512152	-0.607365928	-0.871459147	-0.090833114	0.138377783	brain innervation
ENSDART00000161561	ANK1 (1 of many)	0.001021091	-0.90876402	-0.980007711	-0.299110873	-0.111310605	brain innervation
ENSDART00000161567	KCNT1 (1 of many)	0.000451042	-0.564631347	-0.618941642	-0.212804629	0.112863315	brain innervation
ENSDART00000161646	sema6bb	0.009956893	-0.22063759	-0.362707192	-0.160882628	0.032299092	brain innervation
ENSDART00000161700	st3gal2	0.000191101	-0.330022994	-0.187434223	0.066957229	0.392836726	brain innervation
ENSDART00000161725	map7d1a	0.007973168	-0.696007823	-0.432281688	0.042931769	0.13512701	brain innervation
ENSDART00000161992	PAQR9	0.016559516	-0.193753966	-0.524789375	-0.118624882	0.042415903	brain innervation
ENSDART00000162002	grm1b	0.001159496	-0.568603213	-0.750999293	-0.21404532	-0.094504234	brain innervation
ENSDART00000162023	plppr2a	0.001528476	0.019417247	-0.576626688	0.082766084	0.317388761	brain innervation
ENSDART00000162133	tenm4	0.007324183	-0.287462387	-0.412164757	-0.184969779	0.049525758	brain innervation
ENSDART00000162277	grip1	5.26677E-05	-0.456620328	-0.2169797	0.146006741	0.157130316	brain innervation
ENSDART00000162381	dkk3a	0.000417493	-0.551126502	-0.361561955	-0.324385943	0.074151181	brain innervation
ENSDART00000162601	scn2b	0.000115668	-0.529498392	-0.64570335	-0.359290818	0.039638437	brain innervation
ENSDART00000162670	slc8a1b	0.001547111	-0.269852688	-0.529256481	-0.096682296	0.048355338	brain innervation
ENSDART00000162675	trim2b	0.004332449	-0.409757052	-0.631223683	-0.07653124	0.093922127	brain innervation
ENSDART00000162714	pcdh10b	0.00041526	-0.340908137	-0.430434424	-0.069086695	0.178421467	brain innervation
ENSDART00000162850	irx3a	0.000148815	-0.694816328	-0.301932125	0.187764734	0.208405543	brain innervation
ENSDART00000162984	cacnb2a	0.000644738	-0.567328475	-0.858575333	0.120461387	0.550245867	brain innervation
ENSDART00000163006	magi1b	1.36917E-05	-0.558734199	-0.633598853	0.045195354	0.572492467	brain innervation
ENSDART00000163096	gpr22b	4.91848E-05	-1.106969479	-1.366483735	-0.599957035	0.051341663	brain innervation
ENSDART00000163216	prune2	0.005893002	-0.414992243	-0.329741076	-0.094156508	0.003976732	brain innervation
ENSDART00000163355	gria2a	0.001252059	-0.37007276	-0.57694131	-0.293854127	0.240567837	brain innervation
ENSDART00000163519	CABZ01050166.1	0.000257591	-0.484053035	-0.532447227	-0.143157501	0.191505756	brain innervation
ENSDART00000163529	FAM126B	0.002016009	-0.519426512	-0.370293712	0.022402011	0.032038169	brain innervation
ENSDART00000163539	scrt1a	7.10385E-05	-0.576475687	-0.187048661	0.063865515	0.398969662	brain innervation
ENSDART00000163612	gabrb4	0.000645779	-0.445185281	-0.610684009	-0.211332691	0.127493005	brain innervation
ENSDART00000163616	homer1b	0.002838981	-0.487597805	-0.239525084	-0.239441952	-0.083482271	brain innervation
ENSDART00000163622	kirrel3l	0.013286981	-0.615947415	-0.493751402	-0.207879997	0.127527609	brain innervation
ENSDART00000163635	erbb4b	0.011458427	-0.423431984	-0.772483288	-0.24962409	0.07826798	brain innervation
ENSDART00000163669	cplx2	0.011375495	-0.402497387	-0.424820216	-0.158797592	0.046498934	brain innervation
ENSDART00000163677	NA	0.000160913	-0.639905731	-0.179601491	0.106036319	0.34272584	brain innervation
ENSDART00000163791	acss2l	0.000960572	-0.694063426	-0.500876422	-0.115488475	-0.045115535	brain innervation
ENSDART00000163867	gnb1b	0.000919184	-0.511340157	-0.236747213	0.117718298	0.111335872	brain innervation
ENSDART00000163897	lgi1b	0.000144591	-0.358188713	-0.274223364	-0.009680681	0.361584537	brain innervation
ENSDART00000163903	kcna2b	5.02856E-05	-1.041609083	-1.150732339	-0.278604653	0.240702332	brain innervation
ENSDART00000163951	plppr1	0.00335169	-0.393500272	-0.316896098	-0.148613826	0.078198855	brain innervation
ENSDART00000164114	grb2a	0.000322945	-0.50918281	-0.254702947	0.293231191	0.639895729	brain innervation
ENSDART00000164121	mboat2b	0.01376164	-0.365653488	-0.229675996	-0.118902893	-0.108744854	brain innervation
ENSDART00000164163	si:dkey-202l22.3	3.90156E-05	-0.676171429	-1.127391316	0.216542828	0.638796234	brain innervation
ENSDART00000164178	prrt2	7.10385E-05	-0.547052118	-0.283537507	0.297793332	0.499767197	brain innervation
ENSDART00000164190	ksr2	0.000228698	-0.449890716	-0.587945856	-0.131638865	0.158651331	brain innervation
ENSDART00000164326	si:ch73-119p20.1	8.83909E-05	-0.36329758	-0.388299747	0.050179382	0.305653209	brain innervation
ENSDART00000164361	gcgra	0.001631005	-0.404304946	-0.520606663	-0.179928762	-0.009148827	brain innervation
ENSDART00000164597	si:ch73-127m5.1	0.000190025	-0.712165198	-0.239448184	0.59846121	0.690834997	brain innervation
ENSDART00000164879	slc8a2b	0.004027736	-0.298961891	-0.426517442	-0.175774312	0.049460205	brain innervation
ENSDART00000164982	cdh4	5.24617E-05	-0.415355609	-0.489911794	0.041403227	0.334639192	brain innervation
ENSDART00000164989	si:ch211-121j5.4	0.005899482	-2.541662275	-0.597024815	-0.157115726	-0.615748868	brain innervation
ENSDART00000165004	gria3b	0.001296944	-0.410652366	-0.638891942	-0.274205904	0.132979921	brain innervation
ENSDART00000165058	rims2a	0.004379828	-0.759006031	-0.607036719	-0.257997898	-0.111766909	brain innervation
ENSDART00000165082	ppp1r1b	0.000811535	-0.592550768	-0.433249399	0.132918309	0.038849346	brain innervation
ENSDART00000165108	jph3	0.008210772	-0.254573235	-0.426555215	0.040987467	0.08534723	brain innervation
ENSDART00000165158	iqsec3a	0.002139835	-0.287263936	-0.339420666	-0.160997893	0.073213089	brain innervation
ENSDART00000165400	slc1a2b	0.01293062	-0.437547383	-0.373563248	-0.176045295	-0.026222695	brain innervation
ENSDART00000165420	si:dkey-161j23.5	0.000212569	-0.418752064	-0.09080604	0.112517016	0.202865451	brain innervation
ENSDART00000165443	zgc:153615	4.64299E-05	-0.585165411	-0.400257189	-0.265863973	-0.020005469	brain innervation
ENSDART00000165609	barhl2	0.000401616	-0.31272019	-0.374018735	0.126371762	0.224852149	brain innervation
ENSDART00000165638	pax10	0.001292159	-0.459647113	-0.417992165	-0.200180992	-0.120970793	brain innervation
ENSDART00000165654	atp1b2a	5.13907E-05	-0.572829136	-0.493634763	-0.065881564	0.075479325	brain innervation
ENSDART00000165659	epb41l3a	0.027884112	-0.249067696	-0.637410719	-0.072556493	-0.093775549	brain innervation
ENSDART00000165698	pbx1a	0.000272902	-0.205212048	-0.330154536	0.047550564	0.321002436	brain innervation
ENSDART00000165744	kif1b	0.001276988	-0.018685925	-0.126032816	0.340319561	0.347744455	brain innervation
ENSDART00000165757	pax6b	0.019981901	-1.030621281	-0.736504391	-0.510180699	-0.112500923	brain innervation
ENSDART00000165785	pcdh10a	0.001354936	-0.2607204	-0.385620436	-0.040787194	0.144409091	brain innervation
ENSDART00000166114	sema3ab	0.01892315	-0.726704016	-0.305486009	-0.172433457	-0.053903823	brain innervation
ENSDART00000166351	nkrf	0.010292856	-0.332043429	-0.213944917	-0.117297764	-0.03436019	brain innervation
ENSDART00000166470	CU855947.1	0.009503355	-0.432778868	-0.336734093	-0.219445916	-0.079633712	brain innervation
ENSDART00000166502	satb2	1.1419E-05	-0.540440017	-0.158642442	1.216799418	1.496340928	brain innervation
ENSDART00000166508	fdft1	0.007609288	-0.467410589	-0.45021858	0.057865823	-0.125768039	brain innervation
ENSDART00000166533	zgc:123181	2.15557E-05	-0.682536387	-0.078871471	0.212564109	0.407498801	brain innervation
ENSDART00000166575	ppp3ca	0.000363739	-0.493387865	-0.574192875	0.281204144	0.517021392	brain innervation
ENSDART00000167306	ldb2b	0.003805801	-0.838228207	-0.415457403	-0.325734782	0.110687477	brain innervation
ENSDART00000167324	ebf3a	4.91848E-05	-0.585714789	-0.243731213	0.563349846	0.843370215	brain innervation
ENSDART00000167464	GNAZ	0.003016249	-0.341892906	-0.155068478	0.102581405	0.079027974	brain innervation

ENSDART00000167726	RYR2	5.17084E-05	-0.554726037	-0.413134766	0.099586067	0.289722828	brain innervation
ENSDART00000167995	napba	0.006669949	-0.341681302	-0.334450461	-0.176497327	-0.027366162	brain innervation
ENSDART00000168038	edil3a	7.05055E-05	-0.585018977	-0.697545399	-0.17495912	0.33520597	brain innervation
ENSDART00000168121	lzts3a	0.020009895	-0.348828928	-0.543883063	-0.164874094	0.093345065	brain innervation
ENSDART00000168270	CABZ01048053.1	0.000157185	-0.515099411	-0.533680852	0.225344787	0.362581791	brain innervation
ENSDART00000168396	FO904869.1	0.008573911	-0.490668054	-0.607434677	-0.194530972	0.12309886	brain innervation
ENSDART00000168453	slc43a2a	0.000169466	-0.36850619	-0.532753158	0.198651765	0.248433931	brain innervation
ENSDART00000168568	CU861477.1	0.002569884	-0.399340079	-0.694343635	-0.183948068	0.054030168	brain innervation
ENSDART00000168633	CABZ01085700.1	5.17084E-05	-0.53401794	-0.080184443	0.667052745	0.803861054	brain innervation
ENSDART00000168729	fam196ab	0.003860077	-0.54578901	-0.555866325	-0.405243034	0.063979844	brain innervation
ENSDART00000168749	nlgn3a	0.001942462	-0.417078437	-0.490224096	-0.240516544	-0.086053557	brain innervation
ENSDART00000168805	kcnk9	0.002102587	-0.553605245	-0.952161118	-0.308244106	0.289642563	brain innervation
ENSDART00000168885	nrg1	0.000322759	-0.78621851	-0.719452399	0.167065606	0.289901187	brain innervation
ENSDART00000168969	htr7c	0.004472999	-0.617759768	-0.791263551	-0.091828465	0.038777953	brain innervation
ENSDART00000169081	mtmr7b	0.000669667	-0.302136082	-0.184242994	0.142322853	0.09943817	brain innervation
ENSDART00000169127	adgrb3	0.004610119	-0.338634159	-0.316792657	-0.154400064	0.089681207	brain innervation
ENSDART00000169243	znf384l	0.012912717	-0.366831524	-0.600767675	-0.247311028	0.205515141	brain innervation
ENSDART00000169552	cdh13	0.000225609	-0.778914955	-0.463881372	-0.06287293	0.145633136	brain innervation
ENSDART00000169626	kcnc1a	0.003703617	-0.804732723	-1.394467436	-0.226498418	0.073837621	brain innervation
ENSDART00000169768	slc8a3	0.004351059	-0.133596734	-0.60366341	-0.159093114	0.187240856	brain innervation
ENSDART00000169948	GRM7	0.005631068	-0.415342332	-0.376852993	-0.226859206	-0.023200649	brain innervation
ENSDART00000170089	epha4b	0.011264004	-0.586426265	-0.698463168	-0.228203192	-0.016930318	brain innervation
ENSDART00000170165	cep97	0.016618446	-4.277597338	-0.154895502	-0.337498335	-0.461056374	brain innervation
ENSDART00000170396	apbb2b	2.95145E-05	-0.997027217	-0.745617105	0.597402109	1.180709512	brain innervation
ENSDART00000170423	jakmip3	0.002756705	-0.243674673	-0.387799515	-0.202768181	0.090619484	brain innervation
ENSDART00000170453	slc20a1b	0.003686476	-0.460714141	-0.247481668	-0.017046699	-0.023964599	brain innervation
ENSDART00000170460	nrxn3b	0.003312965	-0.2276052	-0.615850363	-0.134586161	-0.003465474	brain innervation
ENSDART00000170510	RIMBP2 (1 of many)	0.000415146	-0.653406892	-0.568163429	-0.174788334	-0.007324529	brain innervation
ENSDART00000170569	syt12	1.45694E-05	-0.747896951	-0.906145295	-0.362441574	-0.047384497	brain innervation
ENSDART00000170620	ctxn1	0.006828168	-1.120908397	-0.319127099	0.144625495	0.239695278	brain innervation
ENSDART00000170680	ptprdb	1.22823E-05	-0.822263801	-1.170454394	0.28663796	0.697055522	brain innervation
ENSDART00000170700	nacc1b	0.000772038	-0.295710389	-0.416714615	0.013950982	0.110349783	brain innervation
ENSDART00000170839	pcdh1g26	7.10385E-05	-0.648364026	-0.34360777	0.683964001	0.607743239	brain innervation
ENSDART00000170854	gphnb	2.98725E-05	-0.471165414	-0.533169869	-0.144401896	0.220388206	brain innervation
ENSDART00000170875	BX663610.1	0.008814649	-0.528067918	-0.63401091	-0.217086155	-0.025338796	brain innervation
ENSDART00000170888	pkma	0.009639463	-0.382873513	-0.359191464	-0.202639045	0.04775771	brain innervation
ENSDART00000170932	rims2a	0.000444888	-0.482409185	-0.599060629	-0.273352321	-0.056203652	brain innervation
ENSDART00000170952	pvrl2l	0.000405848	-0.402141005	-0.731362921	-0.043297784	0.281757642	brain innervation
ENSDART00000171013	tenm4	0.009203641	-0.557784731	-0.616815207	-0.086642376	-0.101260046	brain innervation
ENSDART00000171073	tox2	0.012896891	-0.713271279	-0.691854818	-0.08505138	-0.233730891	brain innervation
ENSDART00000171114	si:dkey-18j18.3	0.00335169	-5.054012109	-5.054146995	-1.846245188	0.223162792	brain innervation
ENSDART00000171288	magi2b	0.003659486	-0.2674353	-0.525609031	-0.078206648	-0.00532511	brain innervation
ENSDART00000171306	stxbp1a	0.001277839	-0.614795684	-0.539858263	-0.126841305	0.057169963	brain innervation
ENSDART00000171359	asic1b	0.000948363	-0.583045866	-0.669491195	-0.186449925	0.212036898	brain innervation
ENSDART00000171490	PCDH8	2.78074E-05	-0.518696681	-0.628285802	-0.168709969	0.408565003	brain innervation
ENSDART00000171494	ssbp2	0.000598084	-0.298958691	-0.315587827	-0.028952479	0.152638316	brain innervation
ENSDART00000171506	NA	6.00847E-05	-0.792662919	-0.4993043	0.08528346	0.180572156	brain innervation
ENSDART00000171571	FAM184A (1 of many)	0.001237101	-0.84950254	-1.068290057	0.155587747	0.361779982	brain innervation
ENSDART00000171594	mef2aa	0.010231208	-0.649844171	-0.685014218	-0.301258241	-0.003741614	brain innervation
ENSDART00000171674	abhd8a	0.000204711	-0.482604068	-0.462135418	0.077091696	0.23737745	brain innervation
ENSDART00000171704	soul4	0.004085991	-0.335441282	-0.335430337	-0.104813252	-0.062286587	brain innervation
ENSDART00000171951	slit2	0.003148896	-0.546372344	-0.56571293	-0.270916548	-0.035288929	brain innervation
ENSDART00000172232	sv2a	0.00145694	-0.325501969	-0.478402861	-0.246153159	0.031695064	brain innervation
ENSDART00000172465	gnb1b	0.003905267	-0.429380032	-0.139749463	0.050389396	0.071415749	brain innervation
ENSDART00000172552	dyrk2	0.007164707	-1.00865602	-4.097564958	-0.48472737	-0.263450257	brain innervation
ENSDART00000172624	chn1	0.001420911	-0.429491719	-0.379515323	-0.144891996	-0.16828013	brain innervation
ENSDART00000172634	ryr3	0.003072278	-1.057085419	-1.586723574	-0.163110923	0.331797887	brain innervation
ENSDART00000172638	gria3a	0.000581789	-0.308989617	-0.39028425	-0.157465183	0.147319123	brain innervation
ENSDART00000172645	unc5da	0.000935288	-0.375739047	-0.576943357	-0.183677467	0.03667029	brain innervation
ENSDART00000172664	kirrel3l	0.000274768	-0.579597094	-0.334224423	-0.00269739	0.256102934	brain innervation
ENSDART00000173017	atp2b2	0.002838732	-0.485284755	-0.507921276	-0.213758166	0.050306247	brain innervation
ENSDART00000173060	rph3ab	4.91848E-05	-0.917567753	-0.595167162	0.673778236	0.835535825	brain innervation
ENSDART00000173108	gpc3	2.23813E-05	-0.979170892	-0.757011653	0.064815285	0.413031736	brain innervation
ENSDART00000173126	klhl4	0.012397761	-0.765151123	-0.487744119	-0.047554388	-0.018081928	brain innervation
ENSDART00000173192	pcdh1b	0.001151232	-0.448371004	-0.996454455	-0.461950735	0.179661768	brain innervation
ENSDART00000173210	kcnab2a	6.7807E-05	-0.633080178	-0.760216594	-0.295624932	-0.02803528	brain innervation
ENSDART00000173228	slc6a11b	0.000233744	-1.626653664	-2.205227269	-0.432079303	0.774903159	brain innervation
ENSDART00000173301	sfxn5b	0.004367955	-0.333126218	-0.098925577	-0.061350927	0.22562903	brain innervation
ENSDART00000173421	lingo2a	0.000589104	-0.727375414	-0.584905703	-0.169839849	0.100856447	brain innervation
ENSDART00000000804	slc8a1b	0.0088045	-0.216070414	-0.730017061	-0.564883211	-0.309532287	downregulated during regeneration - early
ENSDART00000000876	nr4a1	0.000894056	-2.262372442	-2.399082592	-2.145415905	-1.543723254	downregulated during regeneration - early
ENSDART00000001907	slc16a3	0.026714705	-0.228617593	-0.852511558	-0.452127784	-0.215137636	downregulated during regeneration - early
ENSDART00000003008	gad1b	0.009594186	-0.375298425	-0.380219055	-0.456528647	-0.347996362	downregulated during regeneration - early
ENSDART00000003548	znf385a	0.001069696	-0.544762633	-0.635515273	-0.316063497	-0.128464507	downregulated during regeneration - early
ENSDART00000003736	anos1b	0.02243441	-0.470963792	-0.442368787	-0.444507654	-0.318716779	downregulated during regeneration - early

ENSDART00000004034	hpca	0.000804628	-0.538662149	-0.391929378	-0.242660916	-0.195867715	downregulated during regeneration - early
ENSDART00000004626	sec62	0.03450478	-0.335700502	-0.25907023	-0.14783183	-0.155829549	downregulated during regeneration - early
ENSDART00000005299	hsd17b12a	0.040876822	-0.345584522	-0.261321345	-0.169519073	-0.263954396	downregulated during regeneration - early
ENSDART00000005724	ncanb	0.007781565	-0.308688969	-0.586976696	-0.410319756	-0.132925594	downregulated during regeneration - early
ENSDART00000005738	slitrk2	0.002539213	-0.40615183	-0.499760308	-0.343598039	-0.061449053	downregulated during regeneration - early
ENSDART00000005842	fgf1a	0.001013659	-0.526483511	-0.462706743	-0.482028176	-0.1224057	downregulated during regeneration - early
ENSDART00000006417	pgm1	0.001451795	-0.367653769	-0.345273975	-0.348210636	-0.086795004	downregulated during regeneration - early
ENSDART00000007401	MAP3K13	0.019500385	-0.231843501	-0.363439198	-0.331818795	-0.269324908	downregulated during regeneration - early
ENSDART00000007522	anos1a	0.001275399	-0.500742525	-0.407287946	-0.526812004	-0.211300468	downregulated during regeneration - early
ENSDART00000007630	nhlh2	0.010056242	-0.28794611	-0.324673306	-0.385652145	-0.261092419	downregulated during regeneration - early
ENSDART00000007778	grik1a	0.008380474	-0.423674769	-0.190804556	-0.271114709	-0.0631136	downregulated during regeneration - early
ENSDART00000008287	pgam1a	0.011948793	-0.270559913	-0.416576315	-0.324017463	-0.168254728	downregulated during regeneration - early
ENSDART00000009337	eno1a	0.014647445	-0.396364992	-0.428345122	-0.288646494	-0.130833414	downregulated during regeneration - early
ENSDART00000009569	slc12a5b	0.001340966	-0.543274129	-0.639831062	-0.420973005	-0.072437192	downregulated during regeneration - early
ENSDART00000009653	kcna1b	0.000806652	-0.51649998	-0.917921415	-0.602149268	-0.420615578	downregulated during regeneration - early
ENSDART00000009777	glra3	0.000683221	-0.385455571	-0.554614528	-0.516083394	-0.22262256	downregulated during regeneration - early
ENSDART00000009938	tcf12	0.025246927	-0.385864267	-0.351608927	-0.283149928	-0.323464659	downregulated during regeneration - early
ENSDART00000010257	fam73a	0.046446554	-0.203941778	-0.403469011	-0.199714259	-0.045216291	downregulated during regeneration - early
ENSDART00000010452	zgc:91860	0.008679209	-0.346186809	-0.437676548	-0.463741509	-0.203120402	downregulated during regeneration - early
ENSDART00000011287	aqp4	0.010984291	-0.584037248	-0.688442177	-0.670108654	-0.488547149	downregulated during regeneration - early
ENSDART00000011627	irx7	0.004741095	-0.27830639	-0.509115666	-0.233145912	-0.150983114	downregulated during regeneration - early
ENSDART00000011936	ccdc106a	0.067939329	-0.452156269	-0.26003137	-0.330808493	-0.261299438	downregulated during regeneration - early
ENSDART00000012673	gnb3a	0.000657061	-0.611271729	-0.360057677	-0.265096652	-0.439594606	downregulated during regeneration - early
ENSDART00000013003	tfap2b	0.008192536	-0.299930992	-0.578681033	-0.25971747	-0.279510711	downregulated during regeneration - early
ENSDART00000013117	syt5b	0.003648083	-0.473395784	-0.439637374	-0.372750952	-0.393185839	downregulated during regeneration - early
ENSDART00000013360	ppp1r3cb	0.004590888	-0.357586146	-0.346668382	-0.405594995	-0.235559662	downregulated during regeneration - early
ENSDART00000014668	pcsk1	0.025485875	-0.444651661	-0.49391188	-0.327964124	-0.406864512	downregulated during regeneration - early
ENSDART00000014726	tp53i11b	0.03271715	-0.378677803	-0.267454179	-0.193372925	-0.148965425	downregulated during regeneration - early
ENSDART00000015333	gbx2	0.065341577	-0.202064704	-0.378779754	-0.261598975	-0.176372699	downregulated during regeneration - early
ENSDART00000015956	efna1b	0.054157717	-0.412330789	-0.211680079	-0.214205067	-0.214663513	downregulated during regeneration - early
ENSDART00000016181	ndrg3a	0.015357816	-0.359622862	-0.274997666	-0.226177976	-0.143111599	downregulated during regeneration - early
ENSDART00000016983	spon1a	0.001261708	-0.566333419	-0.54864414	-0.425205045	-0.214533594	downregulated during regeneration - early
ENSDART00000017259	fgf13a	0.058775394	-0.403933655	-0.684393894	-0.638256176	-0.336496098	downregulated during regeneration - early
ENSDART00000017551	slc6a1b	0.011705967	-0.387372781	-0.441576322	-0.34448646	-0.23699282	downregulated during regeneration - early
ENSDART00000017599	rem1	0.048973345	-0.151492945	-0.383680013	-0.100323724	-0.098680467	downregulated during regeneration - early
ENSDART00000017763	CABZ01071180.1	0.028986846	-0.429470778	-0.866508186	-0.658991937	-0.375481088	downregulated during regeneration - early
ENSDART00000017774	cacng5a	0.002016009	-0.685385238	-0.470575642	-0.348446742	-0.159039188	downregulated during regeneration - early
ENSDART00000018501	opn4.1	0.0088045	-0.494086134	-0.180456491	-0.369290026	-0.100666897	downregulated during regeneration - early
ENSDART00000018654	rnd1b	0.003262292	-0.711104866	-0.668463449	-0.311306501	-0.458881777	downregulated during regeneration - early
ENSDART00000019748	lin7a	0.004883865	-0.334365216	-0.254636287	-0.400398823	-0.216397551	downregulated during regeneration - early
ENSDART00000019905	fndc4b	0.000292971	-0.870561937	-0.746602431	-0.973680622	-0.402474596	downregulated during regeneration - early
ENSDART00000019949	ndrg2	0.015961295	-0.366275522	-0.294111898	-0.252300979	-0.169375948	downregulated during regeneration - early
ENSDART00000020054	opcml	0.001389805	-0.412711216	-0.540582472	-0.347997034	-0.057254512	downregulated during regeneration - early
ENSDART00000020249	dusp5	0.033661318	-0.842223428	-0.88819591	-1.001072274	-0.721604289	downregulated during regeneration - early
ENSDART00000020741	aldoaa	0.004379828	-0.416493866	-0.444493146	-0.351916406	-0.154405211	downregulated during regeneration - early
ENSDART00000021605	LRRC4C	0.002327436	-0.456880748	-0.587108705	-0.299621227	-0.131466817	downregulated during regeneration - early
ENSDART00000021609	gad2	0.02923464	-0.376538118	-0.375877531	-0.287892544	-0.231277899	downregulated during regeneration - early
ENSDART00000022290	mdh1aa	0.00652818	-0.386096556	-0.387245145	-0.361974171	-0.162628891	downregulated during regeneration - early
ENSDART00000022549	atp1b3a	0.005736694	-0.438514495	-0.341784188	-0.389598734	-0.186751495	downregulated during regeneration - early
ENSDART00000022579	GABRG3	0.002100653	-0.524427233	-0.895243712	-0.602147004	-0.152848624	downregulated during regeneration - early
ENSDART00000022625	nrarpb	0.033808649	-0.295040721	-0.303522559	-0.284578601	-0.228026779	downregulated during regeneration - early
ENSDART00000023562	CABZ01041610.1	0.000144061	-1.012987922	-1.132303657	-0.526387248	-0.210938966	downregulated during regeneration - early
ENSDART00000024304	per3	0.008088213	-0.478794008	-0.23341732	-0.352073188	-0.024889246	downregulated during regeneration - early
ENSDART00000024832	stat5a	0.004077075	-0.212236095	-0.408877789	-0.375060924	-0.185569558	downregulated during regeneration - early
ENSDART00000025466	slc18a2	0.042155205	-0.576881609	-0.281211576	-0.258781682	-0.379077041	downregulated during regeneration - early
ENSDART00000026174	dgkh	0.000322759	-0.6307495	-1.007774241	-0.588261808	-0.107017931	downregulated during regeneration - early
ENSDART00000026765	slc18a3a	0.00377171	-0.342520426	-0.454539851	-0.359489571	-0.310297658	downregulated during regeneration - early
ENSDART00000026766	aldocb	0.007676315	-0.483629021	-0.540701781	-0.406696036	-0.135568984	downregulated during regeneration - early
ENSDART00000027465	cacna2d4b	0.014868435	-0.370016882	-0.141565087	-0.247577188	0.068079685	downregulated during regeneration - early
ENSDART00000028500	nxn	0.008517342	-0.356492001	-0.304319321	-0.337794036	-0.002781587	downregulated during regeneration - early
ENSDART00000029703	kcnh1a	1.36144E-05	-0.531746722	-1.366001544	-0.565478925	0.09193536	downregulated during regeneration - early
ENSDART00000029981	ppp3cb	0.001261708	-0.453839737	-0.543662557	-0.385030051	0.007551117	downregulated during regeneration - early
ENSDART00000030811	cables2b	0.004679084	-0.198257533	-0.480718843	-0.449759807	-0.060172476	downregulated during regeneration - early
ENSDART00000031139	slc24a4b	0.00106989	-0.756345109	-1.188291659	-0.474432561	-0.06484409	downregulated during regeneration - early
ENSDART00000031265	rtn4r	0.006344596	-0.753039937	-0.836146377	-0.430012914	-0.160533624	downregulated during regeneration - early
ENSDART00000032695	asic4a	0.021143724	-0.405928595	-0.689262684	-0.527033258	-0.378352566	downregulated during regeneration - early
ENSDART00000033248	fam107b	0.004517445	-0.588148397	-0.439287754	-0.362004691	-0.311865235	downregulated during regeneration - early
ENSDART00000033362	gatad2b	0.00857344	-0.40244942	-0.286045422	-0.214483951	-0.084688348	downregulated during regeneration - early
ENSDART00000034421	cdk14	0.046595201	-0.37810724	-0.738234108	-0.33600573	-0.064327168	downregulated during regeneration - early
ENSDART00000034737	cpne8	0.000212569	-0.307817154	-0.810818352	-0.660194536	0.005050706	downregulated during regeneration - early
ENSDART00000034883	mcf2a	0.007383608	-0.149015362	-0.337699782	-0.173487291	0.057006752	downregulated during regeneration - early
ENSDART00000036050	rs1a	0.003706014	-0.391328986	-0.392177441	-0.469736828	-0.271292581	downregulated during regeneration - early
ENSDART00000036472	zgc:110852	0.003559691	-0.482143709	-0.509026678	-0.613205563	-0.094495965	downregulated during regeneration - early
ENSDART00000036997	camk2n1a	0.004142036	-0.377223565	-0.40737699	-0.242898936	-0.23919335	downregulated during regeneration - early
ENSDART00000037007	tpi1a	0.002762116	-0.595344218	-0.412573356	-0.319655345	-0.136192131	downregulated during regeneration - early

ENSDART00000037922	slc6a8	0.004497123	-0.298142259	-0.526146924	-0.491663812	-0.167137822	downregulated during regeneration - early
ENSDART00000038290	crhb	0.005277161	-0.533237668	-0.584543834	-0.601107267	-0.51443145	downregulated during regeneration - early
ENSDART00000039277	lhfpl3	0.000949477	-0.493635642	-0.41740998	-0.318633213	-0.189800628	downregulated during regeneration - early
ENSDART00000039571	camk2a	0.001363478	-0.838498146	-0.905374301	-0.593078838	-0.446882891	downregulated during regeneration - early
ENSDART00000040086	pacsin1a	0.019909357	-0.311462824	-0.286213879	-0.425986821	-0.031983095	downregulated during regeneration - early
ENSDART00000040275	kcnj11l	0.033377037	-0.790164361	-0.507671216	-0.85342057	-0.51870362	downregulated during regeneration - early
ENSDART00000040502	trpc5a	0.004129329	-0.568729425	-1.014519921	-0.522696272	-0.18638518	downregulated during regeneration - early
ENSDART00000040672	mecp2	0.047533941	-0.242382035	-0.413395534	-0.149398689	-0.148226048	downregulated during regeneration - early
ENSDART00000041388	cacng2a	0.015156186	-0.088843708	-0.391878721	-0.181623249	-0.021159404	downregulated during regeneration - early
ENSDART00000041503	slc4a4a	0.010983086	-0.308542931	-0.316588825	-0.297229141	-0.158105481	downregulated during regeneration - early
ENSDART00000041861	syt1a	0.033833708	-0.326937152	-0.373717465	-0.346529457	-0.191055943	downregulated during regeneration - early
ENSDART00000042386	unm_sa1261	0.008933267	-0.368338169	-0.259611498	-0.383565927	-0.183992374	downregulated during regeneration - early
ENSDART00000043058	TENM2	0.005410022	-0.559723778	-0.553182426	-0.524601572	-0.11140967	downregulated during regeneration - early
ENSDART00000043180	gria3b	0.000788653	-0.628974157	-0.919920549	-0.567970281	0.039022237	downregulated during regeneration - early
ENSDART00000043945	NA	0.013000886	-0.527606114	-0.345502165	-0.38556129	-0.085040536	downregulated during regeneration - early
ENSDART00000044294	fryb	0.002449444	-0.196859444	-0.540115494	-0.186847147	-0.085192364	downregulated during regeneration - early
ENSDART00000044733	NPBWR2	0.007828891	-0.533926323	-0.49881296	-0.385102988	-0.276547602	downregulated during regeneration - early
ENSDART00000044735	gria1b	0.029927553	-0.281408167	-0.37749512	-0.41239512	-0.179683873	downregulated during regeneration - early
ENSDART00000044896	camk2d2	0.00145694	-0.471410347	-0.378784897	-0.323522916	-0.120871605	downregulated during regeneration - early
ENSDART00000044986	rnd1a	0.003670956	-0.894483212	-0.275620607	-0.461458	-0.242681728	downregulated during regeneration - early
ENSDART00000045616	gabbr1b	0.008210772	-0.290531763	-0.52893984	-0.416723119	-0.192345482	downregulated during regeneration - early
ENSDART00000045842	rcan3	0.002351134	-0.531033223	-0.615136042	-0.34287969	-0.068102336	downregulated during regeneration - early
ENSDART00000046498	sema3fa	0.004560464	-0.424113863	-0.318396861	-0.293026202	-0.101692261	downregulated during regeneration - early
ENSDART00000046542	igf1rb	0.011264004	-0.265329623	-0.432225627	-0.135218314	-0.147354602	downregulated during regeneration - early
ENSDART00000046663	camta1b	0.022220725	-0.175908238	-0.543108898	-0.289499765	-0.001152787	downregulated during regeneration - early
ENSDART00000047416	slc4a8	0.004215641	-0.393460236	-0.539636776	-0.542467324	-0.330988764	downregulated during regeneration - early
ENSDART00000047541	bach1b	0.039802589	-0.343772395	-0.510091534	-0.468889885	-0.333515696	downregulated during regeneration - early
ENSDART00000048036	gem	0.018018574	-0.746612275	-0.695957849	-0.479539177	-0.330372498	downregulated during regeneration - early
ENSDART00000048365	syt6b	0.014002312	-0.461087231	-0.40037241	-0.458496339	-0.153902769	downregulated during regeneration - early
ENSDART00000048432	dlg4a	0.00231226	-0.380839229	-0.483618741	-0.31591813	-0.034480778	downregulated during regeneration - early
ENSDART00000048775	mbd3b	0.027491007	-0.291847963	-0.361950456	-0.358913558	-0.222238531	downregulated during regeneration - early
ENSDART00000048819	rassf2a	0.006222767	-0.204366055	-0.399787028	-0.377471637	-0.118487631	downregulated during regeneration - early
ENSDART00000048893	pcbp3	0.004826213	-0.37198127	-0.476664366	-0.345261768	-0.190932807	downregulated during regeneration - early
ENSDART00000049036	zgc:92275	0.008658551	-0.447578375	-0.84153571	-0.52710535	-0.386325614	downregulated during regeneration - early
ENSDART00000049240	tob1a	0.008289796	-0.553621268	-0.528794995	-0.629368997	-0.384085742	downregulated during regeneration - early
ENSDART00000050018	cnksr1	0.010245667	0.043033394	-0.349786212	-0.053406139	-0.094004348	downregulated during regeneration - early
ENSDART00000050559	sh3rf1	0.018714496	-0.402839399	-0.313691549	-0.2479685	-0.17125224	downregulated during regeneration - early
ENSDART00000051723	si:ch211-193k19.1	0.001416864	-0.524075663	-0.333228258	-0.259244771	-0.088981638	downregulated during regeneration - early
ENSDART00000052083	fjx1	0.030276337	-0.061315226	-0.667932816	-0.149604151	0.07387804	downregulated during regeneration - early
ENSDART00000052620	npy	0.000988999	-0.456788991	-0.449905237	-0.529362885	-0.310086201	downregulated during regeneration - early
ENSDART00000052989	ache	0.010699261	-0.369275035	-0.306496087	-0.342496448	-0.099646921	downregulated during regeneration - early
ENSDART00000053750	acsl2	7.10385E-05	-0.784570064	-0.626588121	-0.673780261	-0.449722468	downregulated during regeneration - early
ENSDART00000054674	mtnr1aa	0.014726917	-0.46530953	-0.508091587	-0.367618326	-0.477932737	downregulated during regeneration - early
ENSDART00000054736	bhlhe23	0.004928313	-0.369485488	-0.514619457	-0.390416516	-0.190127988	downregulated during regeneration - early
ENSDART00000054790	zmp:0000001069	0.000507818	-0.970537582	-1.254323928	-0.841864806	-0.072822598	downregulated during regeneration - early
ENSDART00000055019	ndufa4	0.000829971	-0.243803126	-0.403243102	-0.444723985	-0.066106721	downregulated during regeneration - early
ENSDART00000055134	ogfr	0.011591036	-0.383562939	-0.286066309	-0.21229445	-0.172842524	downregulated during regeneration - early
ENSDART00000055262	cdk5r1a	0.023933397	-0.345552765	-0.416237328	-0.217352163	-0.195826689	downregulated during regeneration - early
ENSDART00000055264	CA10 (1 of many)	0.032319936	-0.387267589	-0.307777286	-0.264643025	-0.127280824	downregulated during regeneration - early
ENSDART00000055465	si:ch211-149k23.9	0.051826156	-0.23663044	-0.394558494	-0.331258304	-0.323817823	downregulated during regeneration - early
ENSDART00000055890	znf385c	0.011543549	-0.397413591	-0.444425008	-0.419820845	-0.297765515	downregulated during regeneration - early
ENSDART00000056035	PMM1	0.019075692	-0.318342084	-0.457172695	-0.421149458	-0.159828821	downregulated during regeneration - early
ENSDART00000056721	ldhd	0.006978095	-0.445248015	-0.387879357	-0.435006793	-0.179899207	downregulated during regeneration - early
ENSDART00000056810	drd1b	0.002481856	-0.453660686	-0.522513213	-0.435699921	-0.247093083	downregulated during regeneration - early
ENSDART00000057258	slc12a5a	0.009167819	-0.776929597	-2.750292921	-0.618148385	-0.058229169	downregulated during regeneration - early
ENSDART00000057422	pacsin1a	0.016789533	-0.590114235	-0.673330755	-0.518895915	-0.206589128	downregulated during regeneration - early
ENSDART00000058415	zmp:0000001075	0.017815971	-0.428240629	-0.582010832	-0.289943093	-0.116590577	downregulated during regeneration - early
ENSDART00000058706	fosaa	0.004423084	-1.574082598	-1.225695241	-1.240461281	-1.154909276	downregulated during regeneration - early
ENSDART00000059446	znf385b	0.027304375	-0.260906147	-0.315001382	-0.161772411	-0.110285684	downregulated during regeneration - early
ENSDART00000059489	prmt8b	0.00072061	-0.385132635	-0.375733065	-0.525036565	0.065426538	downregulated during regeneration - early
ENSDART00000059984	deptor	0.033009621	-0.356805464	-0.334367455	-0.324987791	-0.320690594	downregulated during regeneration - early
ENSDART00000060056	tpi1b	0.004694	-0.330637537	-0.371028005	-0.31545087	-0.122811105	downregulated during regeneration - early
ENSDART00000060321	RAMP1	0.003256496	-0.401526014	-0.370225897	-0.385056936	-0.044799362	downregulated during regeneration - early
ENSDART00000060356	dgkh	0.001359425	-0.649006381	-0.810232451	-0.629585196	-0.212393687	downregulated during regeneration - early
ENSDART00000060946	sgsm1b	0.010421156	-0.173061984	-0.355540157	-0.215401885	-0.063998625	downregulated during regeneration - early
ENSDART00000062150	zgc:77752	0.038990469	-0.401367403	-0.150322453	-0.162634546	-0.096457711	downregulated during regeneration - early
ENSDART00000062603	cadm1b	0.040704846	-0.182938767	-0.538377364	-0.267052947	-0.164493851	downregulated during regeneration - early
ENSDART00000062736	coasy	0.038825302	-0.31852882	-0.407402551	-0.404610538	-0.370618301	downregulated during regeneration - early
ENSDART00000063551	ppm1e	0.011668571	-0.4375512	-0.67599947	-0.28655858	-0.160579724	downregulated during regeneration - early
ENSDART00000063703	si:dkey-71h2.2	0.004477335	-0.547517928	-0.696070326	-0.446988451	-0.222080286	downregulated during regeneration - early
ENSDART00000063704	crip3	0.023735552	-0.295432006	-0.264072284	-0.393391872	-0.087537117	downregulated during regeneration - early
ENSDART00000063706	fndc4a	1.36144E-05	-0.898991439	-1.194204845	-0.829294946	-0.104966589	downregulated during regeneration - early
ENSDART00000063779	efhd1	0.000461528	-0.527832136	-0.620416333	-0.244685479	-0.230757866	downregulated during regeneration - early
ENSDART00000063816	kcnk3a	0.00834001	-0.460164643	-0.659361375	-0.48206205	-0.175491486	downregulated during regeneration - early
ENSDART00000064241	nrxn3a	0.014683084	-0.593329637	-0.86602502	-0.45366796	-0.193651725	downregulated during regeneration - early

ENSDART00000064403	nptnb	0.050632772	-0.282191976	-0.43563203	-0.351497687	-0.147485108	downregulated during regeneration - early
ENSDART00000064866	prkab1a	0.004813371	-0.370484412	-0.353103565	-0.349388814	-0.328698623	downregulated during regeneration - early
ENSDART00000065361	etv5b	0.019698661	-0.645686026	-0.347502384	-0.293939032	-0.317696675	downregulated during regeneration - early
ENSDART00000065366	st6gal1	0.005057779	-0.520593433	-0.711298232	-0.60540202	-0.072634403	downregulated during regeneration - early
ENSDART00000065507	plppr2b	0.00275056	-0.22717931	-0.353326082	-0.20008884	0.028481334	downregulated during regeneration - early
ENSDART00000066269	arl4d	0.002710576	-0.431926082	-0.429953919	-0.294676093	-0.224713874	downregulated during regeneration - early
ENSDART00000066290	UTS2R	0.02114031	-0.720663714	-0.982454784	-0.512077429	-0.545080013	downregulated during regeneration - early
ENSDART00000066386	shisa9a	0.017384242	-0.462438855	-0.347473048	-0.310604934	-0.078066291	downregulated during regeneration - early
ENSDART00000066896	syt1a	0.019811481	-0.255336633	-0.349305824	-0.330024338	-0.178637525	downregulated during regeneration - early
ENSDART00000067190	tspan9b	0.000694227	-0.605960388	-0.476716761	-0.251844268	-0.266924971	downregulated during regeneration - early
ENSDART00000067211	gpr37l1b	0.009682631	-0.390917116	-0.36533905	-0.206436073	-0.113142613	downregulated during regeneration - early
ENSDART00000067733	zgc:77838	0.041639821	-0.192172111	-0.379046905	-0.170230593	-0.131538824	downregulated during regeneration - early
ENSDART00000067741	cacng6b	0.039457626	-2.498204907	-1.377375045	-1.107680653	-1.191335449	downregulated during regeneration - early
ENSDART00000067762	MYO1D	0.006648495	-0.207118869	-0.403579734	-0.286614596	-0.314212256	downregulated during regeneration - early
ENSDART00000073588	kcnj11	0.051199639	-0.465815764	-0.671487298	-0.425150531	-0.293255153	downregulated during regeneration - early
ENSDART00000073617	opn4xa	0.013394015	-0.377358091	-0.474333233	-0.655013246	-0.269116892	downregulated during regeneration - early
ENSDART00000074685	glrbb	0.001927288	-0.387255204	-0.421576892	-0.370807969	-0.094075713	downregulated during regeneration - early
ENSDART00000074838	kcnk3b	0.00778428	-0.808453975	-0.995140212	-0.30053552	-0.689829672	downregulated during regeneration - early
ENSDART00000074936	gabrr2a	0.040026502	-0.375623148	-0.534637844	-0.431940406	-0.359950744	downregulated during regeneration - early
ENSDART00000075510	ngb	0.004883865	-0.534714702	-0.310443771	-0.511019459	-0.235764625	downregulated during regeneration - early
ENSDART00000075935	vtnb	0.011844748	-0.377853158	-0.359520721	-0.399371834	-0.233041975	downregulated during regeneration - early
ENSDART00000075940	mtnr1ba	0.03016157	-0.423445838	-1.139676296	-0.754844062	-0.657118397	downregulated during regeneration - early
ENSDART00000075974	ism2b	0.049434429	-0.752954546	-0.582959852	-0.548810475	-0.377147389	downregulated during regeneration - early
ENSDART00000075993	crtc1b	0.069455159	-0.280354395	-0.577500657	-0.194615785	-0.207111517	downregulated during regeneration - early
ENSDART00000076215	CR735102.1	0.0256797	-0.360410997	-0.514821634	-0.137026978	-0.257442941	downregulated during regeneration - early
ENSDART00000077664	atp2b1a	0.015884799	-0.283300071	-0.323367416	-0.218529441	-0.07251725	downregulated during regeneration - early
ENSDART00000077805	gria2a	0.012308945	-0.63140941	-0.839709321	-0.362303249	-0.053629448	downregulated during regeneration - early
ENSDART00000077809	cyp26c1	0.003805801	-0.796873586	-0.763455549	-0.70044496	-0.582141171	downregulated during regeneration - early
ENSDART00000077823	lrit3a	0.035313407	-0.481897882	-0.48516269	-0.481656788	-0.455604814	downregulated during regeneration - early
ENSDART00000078249	kcnc3a	0.004480141	-0.908848026	-1.288714191	-0.644595688	0.010477706	downregulated during regeneration - early
ENSDART00000078795	ahcyl1	0.001402519	-0.360916144	-0.460734164	-0.414619806	-0.201451999	downregulated during regeneration - early
ENSDART00000079035	rap1gap	0.009758715	-0.57187807	-0.701331328	-0.299987324	-0.289210087	downregulated during regeneration - early
ENSDART00000079341	plch1	0.009734608	-0.457888719	-0.43639001	-0.345543016	-0.155469708	downregulated during regeneration - early
ENSDART00000079454	vamp2	0.027826876	-0.197978353	-0.366012835	-0.326357547	-0.1060827	downregulated during regeneration - early
ENSDART00000079597	vps36	0.031565534	-0.205754956	-0.373190829	-0.317459289	-0.134792851	downregulated during regeneration - early
ENSDART00000080430	gfra2b	9.16648E-05	-0.62088032	-0.576945607	-0.392707521	-0.144958503	downregulated during regeneration - early
ENSDART00000081129	cdk15	4.91848E-05	-0.731126626	-0.682923022	-0.544281357	-0.253398129	downregulated during regeneration - early
ENSDART00000081797	sash1b	0.031235096	-0.368433045	-0.365106826	-0.357216383	-0.178826864	downregulated during regeneration - early
ENSDART00000081926	CU856539.1	0.01098705	-0.250902601	-0.402416425	-0.31567263	-0.091643419	downregulated during regeneration - early
ENSDART00000081978	KCNJ6	0.003763847	-0.364130454	-0.389723131	-0.355694518	-0.126078935	downregulated during regeneration - early
ENSDART00000081990	strip2	0.005385838	-0.530994895	-0.582233551	-0.263527962	-0.22210065	downregulated during regeneration - early
ENSDART00000082012	gsk3aa	0.056003609	-0.41333003	-0.370003942	-0.288947953	-0.203102626	downregulated during regeneration - early
ENSDART00000082142	EFEMP1 (1 of many)	0.081611118	-1.76159347	-0.849874708	-0.584181957	-1.080912576	downregulated during regeneration - early
ENSDART00000082438	dlgap2a	0.01013158	-0.366533513	-0.540400426	-0.257659814	-0.037294062	downregulated during regeneration - early
ENSDART00000082604	galnt18b	0.073840455	-0.214631898	-0.351577935	-0.246678211	-0.218706176	downregulated during regeneration - early
ENSDART00000083100	NA	0.001483339	-0.514905306	-0.64333228	-0.306814422	-0.178642671	downregulated during regeneration - early
ENSDART00000083407	b4galnt4a	0.056841831	-0.141610106	-0.312267637	-0.129787335	-0.08625756	downregulated during regeneration - early
ENSDART00000083416	gabrd	0.001981601	-0.516993342	-0.41812722	-0.415766575	-0.292897654	downregulated during regeneration - early
ENSDART00000083427	slc25a29	0.004497123	-0.338331576	-0.636979798	-0.361185623	-0.191915619	downregulated during regeneration - early
ENSDART00000083453	slc32a1	0.006674249	-0.393731273	-0.378330473	-0.304655077	-0.16825712	downregulated during regeneration - early
ENSDART00000083788	CU633832.1	0.021355398	-0.745373175	-0.699527481	-0.49975457	-0.170614876	downregulated during regeneration - early
ENSDART00000084024	sv2c	0.003671994	-0.354928985	-0.65582646	-0.477522685	-0.220826118	downregulated during regeneration - early
ENSDART00000084381	sybu	0.003015186	-0.763177411	-0.608942869	-0.49552138	-0.188366829	downregulated during regeneration - early
ENSDART00000084530	coro2ba	0.034451982	-0.558160234	-0.686474796	-0.264666518	-0.204619183	downregulated during regeneration - early
ENSDART00000084803	asic2	0.006230689	-0.588195088	-0.477145292	-0.317477641	-0.106481463	downregulated during regeneration - early
ENSDART00000085121	sdk2b	0.030596624	-0.222566721	-0.365719278	-0.147657392	-0.012041014	downregulated during regeneration - early
ENSDART00000085438	rps6ka5	0.005012104	-0.245466951	-0.387017016	-0.309352273	-0.119154274	downregulated during regeneration - early
ENSDART00000085522	hspb6	0.048389146	-0.63697673	-0.382982575	-0.524822831	-0.392317705	downregulated during regeneration - early
ENSDART00000086117	kcnab2b	0.011382754	-0.362026581	-0.574025944	-0.45188395	-0.145196155	downregulated during regeneration - early
ENSDART00000086176	nckap1	0.036431263	-0.438983658	-0.533344195	-0.439721489	-0.241138066	downregulated during regeneration - early
ENSDART00000086181	cabp7b	0.018426524	-0.513778995	-0.579329622	-0.46209057	-0.104843884	downregulated during regeneration - early
ENSDART00000087196	zgc:153240	0.004497123	-0.381380177	-0.392146036	-0.277166885	-0.130863015	downregulated during regeneration - early
ENSDART00000087565	eva1a	0.005375255	-0.378976356	-0.384921525	-0.250389955	-0.168669364	downregulated during regeneration - early
ENSDART00000087654	adcy6a	0.006256994	-0.242190723	-0.49624198	-0.145481294	-0.053244608	downregulated during regeneration - early
ENSDART00000088146	ensab	0.029418485	-0.270112525	-0.281607362	-0.344659286	-0.084313447	downregulated during regeneration - early
ENSDART00000088240	sypb	0.022549623	-0.232689126	-0.445901517	-0.375392134	-0.149058325	downregulated during regeneration - early
ENSDART00000088270	yjefn3	0.006522424	-0.490178998	-0.350279664	-0.399921095	-0.213170988	downregulated during regeneration - early
ENSDART00000088881	git2a	0.019698661	-0.368814104	-0.568589271	-0.278482365	-0.109167728	downregulated during regeneration - early
ENSDART00000088908	srgap1a	0.001679054	-0.173831935	-0.499543132	-0.183148374	0.021193005	downregulated during regeneration - early
ENSDART00000089033	lingo3a	0.002706926	-0.421566075	-0.428623374	-0.267183911	-0.077777224	downregulated during regeneration - early
ENSDART00000089126	trhde.2	0.031143163	-0.409075032	-0.405058445	-0.383617798	-0.218104015	downregulated during regeneration - early
ENSDART00000089748	rorb	0.030623213	-0.330969943	-0.539847879	-0.425468683	-0.138099942	downregulated during regeneration - early
ENSDART00000089967	cacna1bb	0.093941409	-0.202836628	-0.60611593	-0.329318476	-0.282649478	downregulated during regeneration - early
ENSDART00000090252	atp2b3a	0.003519185	-0.293678426	-0.382019678	-0.276234519	-0.034568861	downregulated during regeneration - early
ENSDART00000090335	hipk2	0.016509232	-0.356266924	-0.261702878	-0.185362868	-0.057591831	downregulated during regeneration - early

ENSDART00000090580	si:dkey-215k6.1	0.007297303	-0.353341621	-0.406611115	-0.330167398	-0.116132596	downregulated during regeneration - early
ENSDART00000090669	pleca	0.078237713	-0.254130273	-0.426440212	-0.236918819	-0.12154508	downregulated during regeneration - early
ENSDART00000091151	nell2b	0.000442465	-0.408104365	-0.533629775	-0.525750199	-0.236120482	downregulated during regeneration - early
ENSDART00000091271	prkg2l	0.030415718	-0.404320096	-0.412762377	-0.544649422	-0.030408858	downregulated during regeneration - early
ENSDART00000091416	cntn3a.1	0.007676315	-0.478191534	-0.24621663	-0.434975173	-0.073764559	downregulated during regeneration - early
ENSDART00000091472	kcnv2b	0.007620644	-0.549604515	-0.416623158	-0.289225353	-0.099467789	downregulated during regeneration - early
ENSDART00000091532	ndnf	0.009217803	-0.379105533	-0.357674977	-0.332342823	-0.25490154	downregulated during regeneration - early
ENSDART00000091612	dab2ipa	0.015157115	-0.32378487	-0.312766229	-0.160534626	-0.215968275	downregulated during regeneration - early
ENSDART00000091683	alkbh5	0.020455943	-0.400075016	-0.448742364	-0.161106699	-0.178285375	downregulated during regeneration - early
ENSDART00000092013	tmtc1	0.011155618	-0.379958165	-0.299353758	-0.293525457	-0.123093715	downregulated during regeneration - early
ENSDART00000092051	CABZ01081780.1	0.001500572	-0.497076796	-0.683547714	-0.447049069	-0.155316145	downregulated during regeneration - early
ENSDART00000092183	lrrc3b	0.025950704	-0.469734111	-0.850621377	-0.496353665	-0.22903801	downregulated during regeneration - early
ENSDART00000092356	neto1	0.016069216	-0.404606374	-0.302098716	-0.383296194	-0.272667962	downregulated during regeneration - early
ENSDART00000092493	ptprt	0.007621679	-0.175495642	-0.444255461	-0.308001112	-0.058909837	downregulated during regeneration - early
ENSDART00000092647	cers1	0.035286858	-0.35221666	-0.292832737	-0.390981877	-0.135220693	downregulated during regeneration - early
ENSDART00000092691	pea15	0.000272535	-0.578361557	-0.742039167	-0.342285902	-0.180997926	downregulated during regeneration - early
ENSDART00000093236	TULP3	0.019409502	-0.257622072	-0.196815312	-0.318097587	-0.153320475	downregulated during regeneration - early
ENSDART00000097685	LINGO3 (1 of many)	0.000592646	-0.608919806	-0.847171007	-0.317394062	-0.126526617	downregulated during regeneration - early
ENSDART00000097770	gc3	0.022797575	-0.462816548	-0.433977737	-0.284956472	-0.124147363	downregulated during regeneration - early
ENSDART00000098424	trib2	0.000168321	-0.755876906	-0.434835466	-0.414057266	-0.366344874	downregulated during regeneration - early
ENSDART00000098639	cntn5	0.018537916	-0.259764888	-0.493723855	-0.530432667	-0.172558087	downregulated during regeneration - early
ENSDART00000098668	abcc8b	0.045086702	-0.217525237	-0.529877646	-0.409188595	-0.288785069	downregulated during regeneration - early
ENSDART00000099202	igsf11	0.016255169	-0.183727476	-0.31314564	-0.202768681	-0.036422441	downregulated during regeneration - early
ENSDART00000099235	rnf44	0.003130236	-0.234703995	-0.37643632	-0.256248531	0.012942846	downregulated during regeneration - early
ENSDART00000099869	slc17a7b	0.012556905	-0.359089552	-0.531552161	-0.360552754	0.022409885	downregulated during regeneration - early
ENSDART00000100074	pbx3a	0.034362521	-0.314908702	-0.751877287	-0.359257757	-0.077459645	downregulated during regeneration - early
ENSDART00000100223	zgc:91860	0.02057139	-0.412727201	-0.311203152	-0.280203316	-0.243367869	downregulated during regeneration - early
ENSDART00000100290	napbb	0.006047456	-0.403034353	-0.312472409	-0.341096083	-0.127811116	downregulated during regeneration - early
ENSDART00000100327	nptx1l	0.010245667	-0.348659958	-0.433920996	-0.393251042	-0.286427391	downregulated during regeneration - early
ENSDART00000100639	chrm4a	0.001996892	-0.541979173	-0.464947812	-0.239039654	-0.216630506	downregulated during regeneration - early
ENSDART00000100681	ncam2	0.01695739	-0.42007768	-0.748629002	-0.327699832	-0.08364547	downregulated during regeneration - early
ENSDART00000101231	syt7b	0.02142158	-0.85705254	-2.266705376	-0.529986528	0.010282974	downregulated during regeneration - early
ENSDART00000101948	GJA9 (1 of many)	0.063141225	-0.638779519	-0.432316335	-0.423080448	-0.46097286	downregulated during regeneration - early
ENSDART00000102445	clasp1a	0.056020682	-0.444419251	-0.511465397	-0.218813787	-0.372606163	downregulated during regeneration - early
ENSDART00000102461	rgs8	0.000273326	-0.536939066	-0.575929959	-0.463779418	-0.057720257	downregulated during regeneration - early
ENSDART00000102790	glrba	0.002147221	-0.477875363	-0.33299196	-0.36632817	-0.172063586	downregulated during regeneration - early
ENSDART00000102881	fam43b	0.029887325	-0.413239873	-0.283572592	-0.358392622	-0.273570929	downregulated during regeneration - early
ENSDART00000103532	kcnh5a	0.002023943	-0.481841968	-0.667651405	-0.403869416	-0.116032422	downregulated during regeneration - early
ENSDART00000103549	skib	0.029718853	-0.170611371	-0.408422527	-0.193614247	-0.134954723	downregulated during regeneration - early
ENSDART00000103640	hey1	0.023203992	-0.385659584	-0.747117397	-0.392306381	-0.416692063	downregulated during regeneration - early
ENSDART00000103646	kcng2	0.000233744	-0.538883844	-0.646084515	-0.39646557	0.008271529	downregulated during regeneration - early
ENSDART00000103894	cacng8b	3.32082E-05	-0.589374624	-0.680390231	-0.379441318	-0.105621743	downregulated during regeneration - early
ENSDART00000103980	crip2	0.004627449	-0.517107368	-0.364195263	-0.546778441	-0.267901424	downregulated during regeneration - early
ENSDART00000104008	dtnbp1b	0.006824859	-0.424284075	-0.457257716	-0.424822836	-0.199214695	downregulated during regeneration - early
ENSDART00000104027	rims3	0.000442465	-0.345163808	-0.70584123	-0.314103014	-0.01029762	downregulated during regeneration - early
ENSDART00000104293	pex5la	8.79987E-05	-0.678333589	-0.710207201	-0.577955484	-0.480904778	downregulated during regeneration - early
ENSDART00000104481	slc17a7a	0.009884056	-0.380361502	-0.377656856	-0.259723773	-0.186490981	downregulated during regeneration - early
ENSDART00000104722	cdk5r2a	0.016942898	-0.371112934	-0.317321269	-0.137896092	-0.229143186	downregulated during regeneration - early
ENSDART00000104828	gtpbp2b	0.002166891	-0.421398738	-0.385176207	-0.273058228	-0.083654585	downregulated during regeneration - early
ENSDART00000105477	lrrtm2	0.001866651	-0.540265042	-0.543880814	-0.470126158	-0.070951006	downregulated during regeneration - early
ENSDART00000105866	si:ch73-213k20.5	0.001847721	-0.515754761	-0.874847543	-0.892891327	-0.392973916	downregulated during regeneration - early
ENSDART00000106096	drd1a	0.032267182	-0.279465728	-0.508847304	-0.2349577	-0.295766526	downregulated during regeneration - early
ENSDART00000106186	prex2	0.010817469	-0.375401587	-0.276106515	-0.274390577	-0.295066887	downregulated during regeneration - early
ENSDART00000108507	CSMD3	0.008588572	-0.201722796	-0.640032982	-0.257154173	0.075603806	downregulated during regeneration - early
ENSDART00000108574	gramd1bb	0.016102005	-0.232214076	-0.405839915	-0.354417408	-0.144413075	downregulated during regeneration - early
ENSDART00000108814	nrip2	0.024870229	-0.213998541	-0.302896779	-0.276048404	-0.173620142	downregulated during regeneration - early
ENSDART00000108959	RNF208	0.003573129	-0.615021133	-0.757500788	-0.372077015	-0.309696014	downregulated during regeneration - early
ENSDART00000108990	pex5lb	0.015010849	-0.323313621	-0.539021494	-0.34249137	-0.198103063	downregulated during regeneration - early
ENSDART00000109014	GJC1	0.052919095	-0.38880893	-0.552238053	-0.31502822	-0.300328618	downregulated during regeneration - early
ENSDART00000109044	grm8a	0.000415801	-0.470031444	-0.746629562	-0.459544685	-0.031467357	downregulated during regeneration - early
ENSDART00000109224	CCKBR (1 of many)	0.012371829	-0.56783642	-0.51463677	-0.498556224	-0.286398879	downregulated during regeneration - early
ENSDART00000109257	RASGRF1	7.10385E-05	-0.570969407	-0.754386918	-0.426488142	-0.098297531	downregulated during regeneration - early
ENSDART00000109308	si:ch73-60h1.1	0.05270735	-0.222101504	-0.315217201	-0.340339223	-0.206722759	downregulated during regeneration - early
ENSDART00000109420	kcnip1b	2.98725E-05	-0.712749849	-0.701262469	-0.816924162	-0.561177799	downregulated during regeneration - early
ENSDART00000109486	gprc5ba	0.001396814	-0.628238673	-0.569388482	-0.508289237	-0.226450363	downregulated during regeneration - early
ENSDART00000109537	snphb	0.000529346	-0.466057723	-0.636087511	-0.318006337	-0.040021342	downregulated during regeneration - early
ENSDART00000109732	amigo1	0.034874709	-0.336374535	-0.536025942	-0.442802906	-0.174075192	downregulated during regeneration - early
ENSDART00000109990	abhd15a	0.035729768	-0.466192052	-0.318128979	-0.316342009	-0.301397533	downregulated during regeneration - early
ENSDART00000110016	ube2ql1	0.022726575	-0.274241146	-0.513839036	-0.485900432	-0.131799131	downregulated during regeneration - early
ENSDART00000110080	aatka	0.025606413	-0.15444172	-0.531359629	-0.399426039	-0.066680489	downregulated during regeneration - early
ENSDART00000110092	mdga1	0.024397386	-0.307586742	-0.496736277	-0.237358341	-0.048338544	downregulated during regeneration - early
ENSDART00000110383	map7a	0.000639961	-0.540958501	-0.669475763	-0.490700501	-0.102714716	downregulated during regeneration - early
ENSDART00000110432	kazna	0.080551232	-0.433352738	-0.292283864	-0.217890679	-0.318143053	downregulated during regeneration - early
ENSDART00000110447	slitrk3b	0.024397386	-0.707318961	-0.905108657	-0.279729387	-0.217159944	downregulated during regeneration - early
ENSDART00000110547	ANKRD34A	0.001359425	-0.459666228	-0.579840169	-0.409587984	-0.250229823	downregulated during regeneration - early

ENSDART00000110789	esyt2b	0.006322758	-0.389945342	-0.213760019	-0.33183342	-0.035634516	downregulated during regeneration - early
ENSDART00000110821	ttc19	0.010842	-0.356526006	-0.209564592	-0.274076967	-0.065932254	downregulated during regeneration - early
ENSDART00000110854	elk1	0.059586248	-0.317422178	-0.365267467	-0.144808984	-0.160403171	downregulated during regeneration - early
ENSDART00000110879	magi2b	0.020773351	-0.318025437	-0.557603079	-0.245924365	-0.15150652	downregulated during regeneration - early
ENSDART00000110974	zdhhc12b	0.011929276	-0.321463982	-0.336778233	-0.359206693	-0.209775575	downregulated during regeneration - early
ENSDART00000110976	col19a1	0.036381892	-0.113310455	-0.554338462	-0.336015886	-0.135833904	downregulated during regeneration - early
ENSDART00000111055	kcna4	0.004463264	-0.237309303	-0.448087799	-0.436530246	-0.275701844	downregulated during regeneration - early
ENSDART00000111080	adgrb3	0.02511369	-0.692483265	-0.604853517	-0.34586076	-0.181234915	downregulated during regeneration - early
ENSDART00000111111	nlgn4b	0.015044537	-0.558022607	-0.771924705	-0.300759795	-0.167214796	downregulated during regeneration - early
ENSDART00000111234	gdpd5a	0.025523567	-0.267734676	-0.323349922	-0.30731905	-0.184332435	downregulated during regeneration - early
ENSDART00000111261	cdh24b	0.001978324	-0.501530291	-0.712397312	-0.362295258	-0.168930493	downregulated during regeneration - early
ENSDART00000111323	AMIGO3 (1 of many)	0.023492759	-0.274114526	-0.487248865	-0.174614423	-0.21591611	downregulated during regeneration - early
ENSDART00000111456	rbm10	0.014371639	-0.2435311	-0.244375112	-0.319750026	-0.111931812	downregulated during regeneration - early
ENSDART00000111509	CABZ01072096.1	0.079186307	-0.19271783	-0.37213696	-0.269302922	-0.183488656	downregulated during regeneration - early
ENSDART00000111571	rap1gap2b	0.001659006	-0.543266541	-0.656699315	-0.515603594	-0.222139298	downregulated during regeneration - early
ENSDART00000111636	DTX4 (1 of many)	2.01848E-05	-1.011696111	-1.148570348	-0.809918586	-0.692193186	downregulated during regeneration - early
ENSDART00000111656	gpr78a	0.000190025	-0.727925057	-0.963113505	-0.708360358	-0.204121228	downregulated during regeneration - early
ENSDART00000111759	zmp:0000000794	0.02020282	-0.428447438	-0.466968786	-0.39807432	-0.23111401	downregulated during regeneration - early
ENSDART00000111823	GRIK3	0.003171283	-0.298812703	-0.633195231	-0.313411843	-0.167732562	downregulated during regeneration - early
ENSDART00000111841	BX248501.1	0.00748053	-0.587056195	-1.220156186	-0.870300177	-0.776464497	downregulated during regeneration - early
ENSDART00000112106	fam155a	0.010271025	-0.397020883	-0.419375873	-0.258307362	-0.051593808	downregulated during regeneration - early
ENSDART00000112296	si:ch211-152n14.4	0.042906616	-3.416151322	-1.061691573	-0.948841593	-2.14718796	downregulated during regeneration - early
ENSDART00000112299	gpr37a	0.027970823	-0.365323083	-0.2884934	-0.319868677	-0.150652035	downregulated during regeneration - early
ENSDART00000112301	trhde.1	0.014638227	-0.264986832	-0.332160295	-0.182975887	-0.056935825	downregulated during regeneration - early
ENSDART00000112441	tlr7	0.00039492	-0.49393668	-0.654080017	-0.57399465	-0.158959649	downregulated during regeneration - early
ENSDART00000112646	CNNM1	0.000705938	-0.428104026	-0.544400388	-0.592385966	-0.267977747	downregulated during regeneration - early
ENSDART00000112655	nfasca	0.000530673	-0.501256457	-0.441261682	-0.289256026	-0.116172993	downregulated during regeneration - early
ENSDART00000112735	fam19a1a	0.000783264	-0.689638571	-0.889095001	-0.657230812	-0.483951597	downregulated during regeneration - early
ENSDART00000112743	nmbb	0.023560515	-0.255890888	-1.177861162	-0.767211345	-0.814299265	downregulated during regeneration - early
ENSDART00000113004	CABZ01068356.1	0.025858283	-0.41749624	-0.503262627	-0.597219984	-0.132734415	downregulated during regeneration - early
ENSDART00000113058	gpsm2	0.017032668	-0.158548143	-0.583640034	-0.403831438	-0.106682634	downregulated during regeneration - early
ENSDART00000113162	pi4kaa	0.016474673	-0.145989753	-0.358429295	-0.235575632	-0.007193852	downregulated during regeneration - early
ENSDART00000113196	si:ch211-157b11.14	0.011627674	-0.299973945	-0.39773998	-0.291303207	-0.069868393	downregulated during regeneration - early
ENSDART00000113280	FRMD5 (1 of many)	0.004407873	-0.439670364	-0.372637017	-0.247112316	-0.218940122	downregulated during regeneration - early
ENSDART00000113454	chgb	0.034869075	-0.341137392	-0.331982228	-0.294276744	-0.206705528	downregulated during regeneration - early
ENSDART00000114083	mcf2la	0.003137832	-0.277595318	-0.392280599	-0.224516886	0.025969597	downregulated during regeneration - early
ENSDART00000114117	necab1	0.017561897	-0.468499392	-0.405674747	-0.46743135	-0.222378141	downregulated during regeneration - early
ENSDART00000114322	rusc1	0.045517543	-0.055969033	-0.296840251	-0.124744669	-0.048299568	downregulated during regeneration - early
ENSDART00000114442	fndc5b	0.001847721	-0.498984664	-0.658119927	-0.45669978	-0.18817484	downregulated during regeneration - early
ENSDART00000114660	opn8b	0.000645779	-0.615308217	-0.695080703	-0.641485332	-0.112308149	downregulated during regeneration - early
ENSDART00000114711	sorcs1	0.000116387	-0.736062495	-0.837966489	-0.594713883	-0.208228471	downregulated during regeneration - early
ENSDART00000114748	vip	0.00097129	-0.375915262	-0.449108971	-0.514462327	-0.417228927	downregulated during regeneration - early
ENSDART00000114750	gabrb2	0.00023578	-0.56997023	-0.610789713	-0.475013901	-0.067415538	downregulated during regeneration - early
ENSDART00000115058	pbxip1a	0.00153043	-0.476509078	-0.442420659	-0.523364891	-0.310557938	downregulated during regeneration - early
ENSDART00000115118	tns1a	5.24617E-05	-0.409165389	-0.800200161	-0.457588882	-0.074958883	downregulated during regeneration - early
ENSDART00000115138	rapgef4	0.007642045	-0.335306767	-0.551146979	-0.593390846	-0.144257686	downregulated during regeneration - early
ENSDART00000115161	reps2	0.075212446	-0.084468471	-0.327140094	-0.166340372	-0.127803804	downregulated during regeneration - early
ENSDART00000115278	cx47.1	0.000962944	-0.445735222	-0.554690047	-0.399294096	-0.241214414	downregulated during regeneration - early
ENSDART00000115365	rassf10a	0.008044263	-0.525931221	-0.509933384	-0.256813636	-0.202027084	downregulated during regeneration - early
ENSDART00000121457	lbh	0.006197253	-0.556077202	-0.419617758	-0.340252817	-0.354555634	downregulated during regeneration - early
ENSDART00000121496	gpr153	0.022523327	-0.22286471	-0.331603234	-0.24476212	-0.082544732	downregulated during regeneration - early
ENSDART00000121503	cplx3b	0.010056242	-0.427974445	-0.393867199	-0.470572672	-0.340193244	downregulated during regeneration - early
ENSDART00000121684	nat8l	0.000470359	-0.712971152	-0.758652511	-0.423007329	-0.36098028	downregulated during regeneration - early
ENSDART00000121826	bean1	0.000596344	-0.425046714	-0.600778843	-0.413029577	-0.180932047	downregulated during regeneration - early
ENSDART00000121872	mast3b	0.013889645	-0.277342703	-0.556828614	-0.261994844	-0.168334337	downregulated during regeneration - early
ENSDART00000122081	sybu	0.000261206	-0.707212841	-0.524661917	-0.600466578	-0.221891711	downregulated during regeneration - early
ENSDART00000122429	klf15	0.006222767	-0.341190152	-0.616050077	-0.578895446	-0.337948098	downregulated during regeneration - early
ENSDART00000122454	citb	0.001304993	-0.619522503	-1.363556906	-0.83905374	-0.294671991	downregulated during regeneration - early
ENSDART00000122654	rnd1a	0.045949377	-0.834540827	-0.662552714	-0.915729348	-0.640521788	downregulated during regeneration - early
ENSDART00000122682	cabp1b	0.009667225	-0.294760944	-0.547217913	-0.530251845	-0.289128709	downregulated during regeneration - early
ENSDART00000122747	tmem30aa	0.01889397	-0.229416393	-0.299526834	-0.201671762	-0.059032826	downregulated during regeneration - early
ENSDART00000123359	nlgn1	0.001676296	-0.188766802	-0.577714477	-0.350080443	0.032932651	downregulated during regeneration - early
ENSDART00000123534	cacna1g	0.007400402	-0.255311686	-0.53660486	-0.469326401	-0.279013734	downregulated during regeneration - early
ENSDART00000123797	pcp4b	0.000180202	-0.598256523	-0.489822031	-0.568163011	-0.081816834	downregulated during regeneration - early
ENSDART00000123844	si:dkey-77f5.3	0.019467351	-4.277427291	-0.495308507	-0.670171681	-0.698447042	downregulated during regeneration - early
ENSDART00000123950	PRIMA1	0.001128218	-0.578840887	-0.634067967	-0.550475282	-0.159964437	downregulated during regeneration - early
ENSDART00000124298	klhl29	0.013613126	-0.069964723	-0.409007114	-0.117673983	0.023652831	downregulated during regeneration - early
ENSDART00000124480	adgrl1a	0.006467002	-0.174945207	-0.479872966	-0.185768365	0.06038548	downregulated during regeneration - early
ENSDART00000124582	napbb	0.006047456	-0.403034353	-0.312472409	-0.341096083	-0.127811116	downregulated during regeneration - early
ENSDART00000124662	rtn4a	0.027810244	-0.503868889	-0.627866078	-0.609450822	-0.190620657	downregulated during regeneration - early
ENSDART00000124708	gabra6b	0.028541039	-0.347504052	-0.445856563	-0.426704654	-0.236544239	downregulated during regeneration - early
ENSDART00000124876	VSTM2B	0.000962944	-0.453041699	-0.513162057	-0.416504873	-0.137923272	downregulated during regeneration - early
ENSDART00000125344	skilb	0.010360832	-0.241964121	-0.627384358	-0.493621211	-0.368838752	downregulated during regeneration - early
ENSDART00000125531	plppr5a	0.001540499	-0.485992962	-0.468686144	-0.487868254	-0.311270154	downregulated during regeneration - early
ENSDART00000125766	camkvb	0.000261206	-0.518737667	-0.560273381	-0.364168693	-0.104855004	downregulated during regeneration - early

ENSDART00000125900	hs3st3b1b	0.005535018	-0.2288214	-0.294028167	-0.23141286	0.023426889	downregulated during regeneration - early
ENSDART00000126300	shisa8b	0.024559038	-0.373408846	-0.464508355	-0.412634146	-0.258826308	downregulated during regeneration - early
ENSDART00000126314	pcdh8	0.024963085	-0.25826413	-0.454008614	-0.231067015	-0.062706758	downregulated during regeneration - early
ENSDART00000126559	rap1gap2a	0.025646401	-0.313212246	-0.347504992	-0.446138134	-0.231468434	downregulated during regeneration - early
ENSDART00000126739	tspan5a	0.010049723	-0.28156215	-0.24229244	-0.35515584	-0.059588327	downregulated during regeneration - early
ENSDART00000126845	ldb1a	0.021507957	-0.250500011	-0.460439315	-0.246081535	-0.230460175	downregulated during regeneration - early
ENSDART00000126966	esrrga	0.017679749	-0.248566713	-0.265310961	-0.321308408	-0.070523791	downregulated during regeneration - early
ENSDART00000127062	egr3	0.052026135	-1.907539966	-1.048685041	-1.405658043	-1.087307214	downregulated during regeneration - early
ENSDART00000127144	cacna2d2a	0.013891126	-0.206435603	-0.394190367	-0.167540436	0.000520565	downregulated during regeneration - early
ENSDART00000127167	fam217b	0.004391006	-0.253255723	-0.39768374	-0.389011868	-0.208058743	downregulated during regeneration - early
ENSDART00000127219	FAM155A (1 of many)	0.037495039	-0.247003441	-0.575960705	-0.390291652	-0.269667553	downregulated during regeneration - early
ENSDART00000127236	syt13	7.44856E-05	-0.555582206	-0.558950845	-0.738809627	-0.549910893	downregulated during regeneration - early
ENSDART00000127426	ERBB4 (1 of many)	0.006128742	-0.100134914	-0.701677799	-0.360350675	-0.397963746	downregulated during regeneration - early
ENSDART00000127428	peli3	0.049273759	-0.233448958	-0.50115077	-0.383840384	-0.13792159	downregulated during regeneration - early
ENSDART00000127467	pygmb	0.000803251	-0.413201582	-0.432147367	-0.560055907	-0.134010249	downregulated during regeneration - early
ENSDART00000127506	si:ch73-380n15.2	0.004844187	-0.654968439	-0.466259045	-0.336236181	-0.320196074	downregulated during regeneration - early
ENSDART00000127513	znf385d	0.079618695	-0.506377127	-0.828614957	-0.387596815	-0.346943547	downregulated during regeneration - early
ENSDART00000128198	scn12aa	0.032530799	-0.298887564	-0.309751416	-0.23551982	-0.15404656	downregulated during regeneration - early
ENSDART00000128223	CHCHD5	0.048304569	-0.405489857	-0.5400056	-0.416697607	-0.210022955	downregulated during regeneration - early
ENSDART00000128224	FO904873.1	0.031307521	-0.267293261	-0.580808884	-0.319669262	-0.131136583	downregulated during regeneration - early
ENSDART00000128807	gpr22a	0.000870998	-0.50401992	-0.32421881	-0.337086094	-0.059033497	downregulated during regeneration - early
ENSDART00000128959	zgc:113223	0.022614665	-1.993126278	-0.750526857	-1.059101824	-0.610323514	downregulated during regeneration - early
ENSDART00000129058	rheb	0.002834268	-0.430188972	-0.312082399	-0.301953935	-0.04976624	downregulated during regeneration - early
ENSDART00000129202	DDX17	0.027985198	-0.231356945	-0.275266303	-0.31249155	-0.24318473	downregulated during regeneration - early
ENSDART00000129498	mef2d	0.03679078	-0.188451231	-0.381445449	-0.263610552	-0.197973462	downregulated during regeneration - early
ENSDART00000130215	TMEM179 (1 of many)	0.028527553	-0.219747105	-0.315705451	-0.194562459	-0.061125473	downregulated during regeneration - early
ENSDART00000130393	tlr7	0.010379183	-0.687566787	-0.954352742	-0.823581986	-0.270839916	downregulated during regeneration - early
ENSDART00000130881	gabrr2a	0.025544321	-0.366410023	-0.492281459	-0.409834	-0.272101123	downregulated during regeneration - early
ENSDART00000130891	camk4	0.000560128	-0.554565795	-0.502410128	-0.607057653	-0.157488548	downregulated during regeneration - early
ENSDART00000130977	mtcl1	0.038066989	-0.152016911	-0.472594538	-0.24239267	-0.004366794	downregulated during regeneration - early
ENSDART00000131134	si:ch211-206a7.2	0.008848885	-0.226522125	-0.472315068	-0.371394997	-0.101330709	downregulated during regeneration - early
ENSDART00000131204	asap1b	0.001304993	-0.546891821	-0.455253903	-0.26913051	-0.092257648	downregulated during regeneration - early
ENSDART00000131323	si:dkey-193c22.1	0.006648495	-0.375872197	-0.187390024	-0.399004983	-0.194977152	downregulated during regeneration - early
ENSDART00000131361	kcnip3b	0.008595135	-0.353450144	-1.035812483	-0.692091767	-0.071599751	downregulated during regeneration - early
ENSDART00000131478	kcnab1a	0.005028852	-0.495628945	-0.276495434	-0.300290472	-0.132892856	downregulated during regeneration - early
ENSDART00000131506	cdh6	0.022814815	-0.336043184	-0.313603925	-0.315311457	-0.136816058	downregulated during regeneration - early
ENSDART00000131866	trhra	0.014686352	-0.77311942	-0.482590954	-0.518111078	-0.348001173	downregulated during regeneration - early
ENSDART00000132387	pvalb6	2.98725E-05	-0.612280143	-0.883743713	-0.424014361	-0.028285642	downregulated during regeneration - early
ENSDART00000132411	si:ch1073-184j22.2	0.001677265	-0.765110378	-1.0433591	-0.590491476	-0.153408732	downregulated during regeneration - early
ENSDART00000132522	syt13	0.015571917	-0.475976654	-0.458152818	-0.514134367	-0.475986362	downregulated during regeneration - early
ENSDART00000132582	map4l	0.000829135	-0.700979622	-0.493129551	-0.385069171	-0.268022699	downregulated during regeneration - early
ENSDART00000132767	pxdc1a	0.019460106	-0.518773739	-0.198850875	-0.436310873	-0.094674576	downregulated during regeneration - early
ENSDART00000132829	inadl	0.020169482	-0.343759067	-0.387749769	-0.321781319	-0.246918999	downregulated during regeneration - early
ENSDART00000132838	ank1a	7.52983E-05	-1.15020902	-1.58246422	-0.789622662	-0.12782961	downregulated during regeneration - early
ENSDART00000132911	vwc2l	0.004885993	-0.573360674	-0.839884971	-0.564408735	0.020047377	downregulated during regeneration - early
ENSDART00000133028	mus81	0.049533299	-0.28108498	-0.562830421	-0.307741383	-0.072240912	downregulated during regeneration - early
ENSDART00000133099	zgc:123105	0.003047993	-0.457591599	-0.29947281	-0.397070368	-0.177964487	downregulated during regeneration - early
ENSDART00000133325	nell2b	0.000401619	-0.450880659	-0.507896343	-0.516937003	-0.181021434	downregulated during regeneration - early
ENSDART00000133487	fosb	0.034512759	-0.882572207	-1.273781011	-0.772506712	-0.914864674	downregulated during regeneration - early
ENSDART00000133644	magi2a	0.001489209	-0.426844627	-0.586214992	-0.344780913	0.065236587	downregulated during regeneration - early
ENSDART00000133701	slc6a1a	0.017494941	-0.417718543	-0.444992936	-0.335539494	-0.221449117	downregulated during regeneration - early
ENSDART00000133735	cacng7b	0.004879285	-0.52911361	-0.351880161	-0.311790544	-0.261905914	downregulated during regeneration - early
ENSDART00000133743	camkk1a	0.001676296	-0.520213698	-0.784879077	-0.322164805	-0.513359014	downregulated during regeneration - early
ENSDART00000133802	inpp5jb	0.006314264	-0.26851081	-0.522364408	-0.244805361	-0.012462884	downregulated during regeneration - early
ENSDART00000134053	usp2a	0.031101324	-0.375529791	-0.273036858	-0.320691469	-0.159380591	downregulated during regeneration - early
ENSDART00000134186	cplx4b	0.027048639	-0.392479495	-0.543085477	-0.51593407	-0.267696813	downregulated during regeneration - early
ENSDART00000134431	pcbp3	0.001578304	-0.497499314	-0.525712825	-0.323624866	-0.140513929	downregulated during regeneration - early
ENSDART00000134489	htr2cl1	0.010885977	-0.503209957	-0.409297224	-0.43743207	-0.200430987	downregulated during regeneration - early
ENSDART00000134518	cpa6	0.014368587	-0.382405278	-0.418058887	-0.443147645	-0.210306107	downregulated during regeneration - early
ENSDART00000134661	gstcd	0.067079	-0.989748846	-0.61234101	-0.452964111	-0.446444607	downregulated during regeneration - early
ENSDART00000135206	ppp6r2b	0.014582204	-0.281142732	-0.364406934	-0.331381396	-0.106942863	downregulated during regeneration - early
ENSDART00000135481	si:ch211-67n3.9	0.025747062	-0.264301646	-0.482759797	-0.356089399	-0.148916978	downregulated during regeneration - early
ENSDART00000135897	tmeff2a	0.000235661	-0.507501095	-0.570530323	-0.389897465	-0.071849112	downregulated during regeneration - early
ENSDART00000135965	plch1	0.00810207	-0.574356968	-0.567600235	-0.421755163	-0.154028312	downregulated during regeneration - early
ENSDART00000136200	cbln2b	0.007124918	-0.48916567	-0.352468034	-0.442788192	-0.14816736	downregulated during regeneration - early
ENSDART00000136255	otofa	0.000894529	-0.67545742	-0.815938232	-0.602392654	-0.103624276	downregulated during regeneration - early
ENSDART00000136390	hcn2b	0.012583499	-0.288020893	-0.351077892	-0.312560003	-0.049583355	downregulated during regeneration - early
ENSDART00000136497	CR318624.1	0.022425099	-0.290569983	-0.906819744	-0.441990313	0.029701559	downregulated during regeneration - early
ENSDART00000136559	ano8a	0.000185329	-0.477178876	-0.572871399	-0.340924619	-0.093243478	downregulated during regeneration - early
ENSDART00000136592	ckbb	0.021710741	-0.985379774	-1.268435214	-0.989267529	-0.749991465	downregulated during regeneration - early
ENSDART00000136654	grapb	0.038386468	-0.34841027	-0.721289328	-0.501261917	-0.353877694	downregulated during regeneration - early
ENSDART00000136726	magi3b	0.006204545	-0.381646177	-0.461208385	-0.226303301	-0.080158554	downregulated during regeneration - early
ENSDART00000136733	glra1	0.010376336	-0.259893278	-0.432961012	-0.409765299	-0.248913141	downregulated during regeneration - early
ENSDART00000136789	tjp2a	0.004813371	-0.247782376	-0.511487885	-0.433268095	-0.195886379	downregulated during regeneration - early
ENSDART00000136805	rbm10	0.044578922	-4.539752918	-3.15582054	-1.19980751	-2.046490722	downregulated during regeneration - early

ENSDART00000136853	fam163b	0.012342358	-0.426911899	-0.715978676	-0.456698496	-0.087825906	downregulated during regeneration - early
ENSDART00000137021	cabp4	0.005059311	-0.36160439	-0.600623701	-0.526609212	-0.179412794	downregulated during regeneration - early
ENSDART00000137038	asic2	0.002292785	-0.373156822	-0.72567768	-0.446804249	-0.152680828	downregulated during regeneration - early
ENSDART00000137214	gpm6bb	0.003631812	-0.410929898	-0.351705205	-0.352631557	-0.244368267	downregulated during regeneration - early
ENSDART00000137287	pebp1	0.030268752	-0.410574808	-0.266170824	-0.343257245	-0.128567003	downregulated during regeneration - early
ENSDART00000137293	dla1b	0.011346243	-0.377972932	-0.365130624	-0.231479469	-0.228625621	downregulated during regeneration - early
ENSDART00000137424	c1qtnf4	0.013968714	-0.387372103	-0.312978581	-0.242907328	-0.243606392	downregulated during regeneration - early
ENSDART00000137443	ANK1 (1 of many)	0.009480138	-0.657062014	-0.810024547	-0.456200062	-0.254232075	downregulated during regeneration - early
ENSDART00000137505	vamp1	0.002192481	-0.431791589	-0.393372103	-0.433651273	-0.142134375	downregulated during regeneration - early
ENSDART00000137648	si:dkeyp-123h10.2	0.001203555	-0.55097741	-0.596976893	-0.447594093	-0.275058949	downregulated during regeneration - early
ENSDART00000137680	BX663503.1	0.042893923	-0.232606929	-0.407355209	-0.255389687	-0.079162056	downregulated during regeneration - early
ENSDART00000137728	st6gal1	0.00818074	-0.677667407	-0.699420985	-0.736040173	-0.083670402	downregulated during regeneration - early
ENSDART00000137848	palmda	0.050492107	-0.364561265	-0.248835039	-0.231952958	-0.127331926	downregulated during regeneration - early
ENSDART00000138139	zgc:153157	0.006145665	-0.590921601	-0.369202687	-0.339560998	-0.377004198	downregulated during regeneration - early
ENSDART00000138140	SLC4A5 (1 of many)	0.02741598	-0.322967614	-0.430634585	-0.36463151	-0.15311364	downregulated during regeneration - early
ENSDART00000138185	gad1b	0.050170102	-0.304744145	-0.418178899	-0.358818296	-0.231950915	downregulated during regeneration - early
ENSDART00000138477	BX571945.1	0.007291871	-0.597559877	-0.845904193	-0.62766334	-0.501592231	downregulated during regeneration - early
ENSDART00000138687	ppp1r9a	0.010965837	-0.410087357	-0.337226328	-0.257566135	-0.006942521	downregulated during regeneration - early
ENSDART00000138733	cdh12a	0.010404332	-0.402719414	-0.467471363	-0.247597318	-0.111519505	downregulated during regeneration - early
ENSDART00000138755	dclk1a	0.013281778	-0.464513032	-0.326162873	-0.241704881	-0.01162875	downregulated during regeneration - early
ENSDART00000138820	lrtm2a	0.053649719	-0.205456304	-0.473378158	-0.285055681	-0.252340958	downregulated during regeneration - early
ENSDART00000138822	SSBP4 (1 of many)	0.003854547	-0.268549166	-0.417362634	-0.271649078	-0.060566364	downregulated during regeneration - early
ENSDART00000138890	kcnd2	0.006198931	-0.399063976	-0.347742148	-0.33423415	-0.044907554	downregulated during regeneration - early
ENSDART00000139033	lrp1bb	0.016464653	-0.300129828	-0.380241873	-0.239929923	-0.108887683	downregulated during regeneration - early
ENSDART00000139191	trpc1	0.008989451	-0.495478264	-0.440776327	-0.289497882	-0.094213915	downregulated during regeneration - early
ENSDART00000139196	kif1ab	0.024103105	-0.338072705	-0.653527626	-0.314444638	-0.193117382	downregulated during regeneration - early
ENSDART00000139560	ppp3r1a	0.023218149	-0.205912354	-0.312380575	-0.194151002	0.010760735	downregulated during regeneration - early
ENSDART00000139568	nsmfa	0.033226863	-0.224590353	-0.433156538	-0.157442583	-0.141070436	downregulated during regeneration - early
ENSDART00000139569	zgc:92658	5.24617E-05	-0.993075003	-0.842810673	-0.747118722	-0.361693255	downregulated during regeneration - early
ENSDART00000139608	upf3a	0.003570588	-0.436308546	-0.475063889	-0.33694709	-0.215591369	downregulated during regeneration - early
ENSDART00000139633	pip4k2ab	0.018433591	-0.336773688	-0.358059228	-0.306910553	-0.048531465	downregulated during regeneration - early
ENSDART00000139937	frmpd1a	0.008579097	-0.391315372	-0.461157908	-0.442405648	-0.041073617	downregulated during regeneration - early
ENSDART00000140028	lrrc4.1	0.00194329	-0.491138395	-0.422849217	-0.374271959	-0.216352385	downregulated during regeneration - early
ENSDART00000140220	si:ch211-168k14.2	0.057257329	-0.180662482	-0.408562234	-0.217555172	-0.18329178	downregulated during regeneration - early
ENSDART00000140632	bcl2l13	0.00107971	-0.49849375	-0.354616708	-0.335413941	-0.223271321	downregulated during regeneration - early
ENSDART00000140650	ankrd6b	0.004329931	-0.31619699	-0.471630097	-0.512160686	-0.029088129	downregulated during regeneration - early
ENSDART00000140995	mfsd2b	0.018126285	-0.430905256	-0.255202968	-0.49728314	-0.134050367	downregulated during regeneration - early
ENSDART00000141177	slc24a4a	8.45072E-05	-0.524899894	-0.691002096	-0.394086808	-0.038902918	downregulated during regeneration - early
ENSDART00000141634	grid2	0.003104562	-0.260805209	-0.581973695	-0.28493372	-0.055775802	downregulated during regeneration - early
ENSDART00000142010	ncam1b	0.027293911	-0.299560612	-0.317330716	-0.349579067	-0.156694047	downregulated during regeneration - early
ENSDART00000142013	penka	0.001690789	-0.474299096	-0.531588241	-0.656476493	-0.472875583	downregulated during regeneration - early
ENSDART00000142129	tacr2	0.0004696	-0.694450519	-0.993924228	-0.826737427	-0.21380073	downregulated during regeneration - early
ENSDART00000142171	syt6a	0.011695536	-0.424476082	-0.649670345	-0.435293945	-0.206511692	downregulated during regeneration - early
ENSDART00000142223	hivep3b	0.013948629	-0.312228644	-0.441058379	-0.334712198	-0.18438466	downregulated during regeneration - early
ENSDART00000142397	samd12	0.034654408	-0.674160268	-0.821942052	-0.657095298	-0.360701575	downregulated during regeneration - early
ENSDART00000142413	BX897741.1	0.02739836	-0.396693848	-0.590528792	-0.411359985	-0.422415067	downregulated during regeneration - early
ENSDART00000142654	BX324123.2	0.001948843	-0.377689742	-0.458973085	-0.444383379	-0.275709525	downregulated during regeneration - early
ENSDART00000142748	UNC13A	0.007676315	-0.222825276	-0.520698317	-0.335972829	0.134560543	downregulated during regeneration - early
ENSDART00000142806	stxbp5a	0.013774152	-0.250986358	-0.382479123	-0.333968332	-0.143471828	downregulated during regeneration - early
ENSDART00000142810	gabra3	0.005380857	-0.405359169	-0.863749916	-0.692169745	-0.647424845	downregulated during regeneration - early
ENSDART00000142837	enpp5	0.001384619	-0.408425134	-0.376571863	-0.363824939	-0.214479077	downregulated during regeneration - early
ENSDART00000142975	si:ch1073-70f20.1	0.018752626	-0.443514404	-0.337920446	-0.430300513	-0.297428401	downregulated during regeneration - early
ENSDART00000142976	kcnip3a	0.00059038	-0.515340349	-0.554488278	-0.50826115	-0.165828333	downregulated during regeneration - early
ENSDART00000143382	il1rapl2	0.002636211	-0.355933029	-0.71045348	-0.38678508	-0.068375826	downregulated during regeneration - early
ENSDART00000143554	ampd2b	0.018018574	-0.328956412	-0.382404642	-0.421715131	-0.209511328	downregulated during regeneration - early
ENSDART00000143582	si:ch211-236p22.1	0.000480969	-0.593497691	-1.183006832	-0.39659144	-0.180756629	downregulated during regeneration - early
ENSDART00000143703	flrt3	0.013070438	-0.315208699	-0.338016477	-0.216002726	-0.160149445	downregulated during regeneration - early
ENSDART00000143784	doc2d	0.00429824	-0.282456786	-0.449882775	-0.341326778	0.036564808	downregulated during regeneration - early
ENSDART00000143787	tnr	0.054368123	-0.299938525	-0.646994379	-0.19048806	-0.113023917	downregulated during regeneration - early
ENSDART00000143867	syngap1b	0.009560651	-0.339959547	-0.514759454	-0.488418852	-0.182310837	downregulated during regeneration - early
ENSDART00000143878	arhgef9a	0.001216477	-0.465269949	-0.707003765	-0.492066921	-0.306386806	downregulated during regeneration - early
ENSDART00000143911	si:dkey-105e17.1	0.001995188	-0.529795865	-0.492017338	-0.300236892	-0.145540976	downregulated during regeneration - early
ENSDART00000143919	gabbr1b	0.013735417	-0.121288245	-0.415278076	-0.279488234	-0.175446101	downregulated during regeneration - early
ENSDART00000144175	ccdc136b	0.006621714	-0.408583735	-0.440451575	-0.345784282	-0.125541648	downregulated during regeneration - early
ENSDART00000144255	magixb	0.023143967	-0.339150091	-0.441125122	-0.366876369	-0.172380242	downregulated during regeneration - early
ENSDART00000144297	ubac2	0.017079861	-0.266999312	-0.798888734	-0.707434326	-0.564642121	downregulated during regeneration - early
ENSDART00000144600	bin1a	0.005414718	-0.647409952	-0.602565457	-0.5226216	-0.498459302	downregulated during regeneration - early
ENSDART00000144995	MFSD3	0.030278232	-0.419691404	-0.388696945	-0.281399212	-0.247251894	downregulated during regeneration - early
ENSDART00000145220	arvcfa	0.000248952	-0.706915106	-0.825791875	-0.448746575	-0.046016635	downregulated during regeneration - early
ENSDART00000145226	si:ch211-233h19.2	0.050170102	-0.372287835	-0.175787817	-0.260726103	-0.131614613	downregulated during regeneration - early
ENSDART00000145342	cbln1	0.014767146	-0.285689309	-0.291683977	-0.350650667	-0.126158592	downregulated during regeneration - early
ENSDART00000145364	sybu	0.039389579	-0.525383816	-0.345930455	-0.451355227	-0.158271762	downregulated during regeneration - early
ENSDART00000145387	shank2	0.001007682	-0.271198688	-0.514736215	-0.289482704	-0.011228429	downregulated during regeneration - early
ENSDART00000145413	si:ch211-253p2.2	0.01662249	-0.612329709	-0.583952587	-0.545467055	-0.273784221	downregulated during regeneration - early
ENSDART00000145615	cacna1i	0.003514334	-0.38207214	-0.804209146	-0.404812647	-0.085276411	downregulated during regeneration - early

ENSDART00000145916	lingo4a	0.003535619	-0.858684072	-0.829176543	-0.734684966	-0.796722528	downregulated during regeneration - early
ENSDART00000145979	col19a1	0.019480943	-0.158274269	-0.337487778	-0.291137211	-0.179712512	downregulated during regeneration - early
ENSDART00000146113	arhgef9b	0.000103226	-0.6095368	-0.587211831	-0.441711189	-0.048741645	downregulated during regeneration - early
ENSDART00000146227	slc24a3	0.000387884	-0.627503774	-0.581196675	-0.447342274	-0.279205796	downregulated during regeneration - early
ENSDART00000146284	kcnh3	0.00027551	-0.880077839	-1.089282019	-0.771605014	-0.433474862	downregulated during regeneration - early
ENSDART00000146321	fibcd1	0.012665885	-0.340172452	-0.594838596	-0.369380512	-0.071609744	downregulated during regeneration - early
ENSDART00000146327	sgk1	0.004289396	-0.407553517	-0.497547741	-0.442772363	-0.391494952	downregulated during regeneration - early
ENSDART00000146380	map6d1	0.003805801	-0.622612519	-0.482084178	-0.312516702	-0.212427733	downregulated during regeneration - early
ENSDART00000146422	samd12	0.010712161	-0.651610173	-1.082672704	-0.694390946	-0.535477884	downregulated during regeneration - early
ENSDART00000146424	bin1a	0.007279599	-0.478145779	-0.444599263	-0.2729609	-0.137965745	downregulated during regeneration - early
ENSDART00000146712	ccdc85a	0.014638227	-0.396306457	-0.805958759	-0.536293039	-0.356615708	downregulated during regeneration - early
ENSDART00000147308	amigo1	0.02740918	-0.366406631	-0.580638611	-0.462226604	-0.20619589	downregulated during regeneration - early
ENSDART00000147473	pcsk1	0.010143774	-0.408472737	-0.345735504	-0.351470668	-0.372482226	downregulated during regeneration - early
ENSDART00000147658	bhlhe22	0.01205445	-0.302235414	-0.31899187	-0.305987775	-0.102192149	downregulated during regeneration - early
ENSDART00000147868	vwc2	0.003299709	-0.776498099	-0.767193994	-0.55435848	-0.286994867	downregulated during regeneration - early
ENSDART00000148073	shisa7b	0.034090908	-0.212233425	-0.392255987	-0.298336106	-0.168085028	downregulated during regeneration - early
ENSDART00000148564	si:ch211-236p22.1	0.00081324	-0.699364761	-0.998683731	-0.566423622	-0.06995253	downregulated during regeneration - early
ENSDART00000148576	pygmb	0.002081425	-0.380093922	-0.425025096	-0.447938233	-0.092571282	downregulated during regeneration - early
ENSDART00000148930	lingo4b	0.038693778	-0.229730557	-0.308881186	-0.199125951	-0.339156928	downregulated during regeneration - early
ENSDART00000148971	ctbp2a	0.015781384	-0.618298464	-0.594645977	-0.414478241	-0.261581238	downregulated during regeneration - early
ENSDART00000149079	sagb	0.045816167	-0.468101765	-0.230897218	-0.419750319	-0.182809331	downregulated during regeneration - early
ENSDART00000149297	slc20a2	0.01613952	-0.247675699	-0.319353289	-0.309560982	-0.222732695	downregulated during regeneration - early
ENSDART00000149411	atxn1b	0.052229213	-0.356760798	-0.298446699	-0.334747495	-0.251003673	downregulated during regeneration - early
ENSDART00000149608	nexn	0.041923827	-0.437154987	-0.289194529	-0.346094746	-0.307861675	downregulated during regeneration - early
ENSDART00000149914	aatka	0.000313682	-0.270082025	-0.541170375	-0.440872523	-0.175726567	downregulated during regeneration - early
ENSDART00000149993	pygmb	0.000820449	-0.443639651	-0.459929444	-0.472379886	-0.083989126	downregulated during regeneration - early
ENSDART00000150228	chata	0.006385907	-0.431975216	-0.532525907	-0.558690348	-0.371717362	downregulated during regeneration - early
ENSDART00000150353	mdh1aa	0.015618075	-0.527537579	-0.6816935	-0.262947	-0.041325795	downregulated during regeneration - early
ENSDART00000151127	thraa	0.039890399	-0.798583389	-0.802364861	-0.351487338	-0.589241182	downregulated during regeneration - early
ENSDART00000151200	mmp17a	0.001404149	-0.370431866	-1.068890772	-0.706081815	-0.179291377	downregulated during regeneration - early
ENSDART00000151229	kcnj5	0.032556649	-0.403592435	-0.680204447	-0.664541848	-0.399506515	downregulated during regeneration - early
ENSDART00000151358	rgl3a	0.000209364	-0.536133566	-0.674550597	-0.735844208	-0.535493428	downregulated during regeneration - early
ENSDART00000151423	c1ql3b	0.004627449	-0.428342943	-0.592751887	-0.458841393	-0.097254864	downregulated during regeneration - early
ENSDART00000151567	sgk494b	0.013519377	-0.371209064	-0.282108451	-0.446431854	-0.117181288	downregulated during regeneration - early
ENSDART00000151627	si:ch73-362m14.2	0.002433479	-1.029772234	-1.504293907	-1.467613461	-0.562650292	downregulated during regeneration - early
ENSDART00000152069	chst2b	0.01938191	-0.329630849	-0.401663244	-0.360860216	-0.363893389	downregulated during regeneration - early
ENSDART00000152371	usp2a	0.034504752	-0.286860533	-0.506577502	-0.337595735	0.001938619	downregulated during regeneration - early
ENSDART00000152376	CLSTN2 (1 of many)	0.008176596	-0.314646809	-0.323760148	-0.422303692	-0.021838531	downregulated during regeneration - early
ENSDART00000152409	mpp3b	0.050836368	-0.399859226	-0.270912664	-0.49906829	-0.114613724	downregulated during regeneration - early
ENSDART00000152531	chrna10a	0.027468632	-0.39848157	-0.348304481	-0.425468269	-0.274269467	downregulated during regeneration - early
ENSDART00000152556	FAM13C	0.014686352	-0.458278075	-0.375744323	-0.341944162	-0.232139437	downregulated during regeneration - early
ENSDART00000152580	nlgn4a	0.013502603	-0.383612645	-0.391621152	-0.368243921	-0.24740854	downregulated during regeneration - early
ENSDART00000152858	rcan2	0.017079861	-0.344037465	-0.451039286	-0.208970338	-0.321499331	downregulated during regeneration - early
ENSDART00000153167	hlfb	0.010966965	-0.470839666	-0.326611575	-0.235752692	-0.264047019	downregulated during regeneration - early
ENSDART00000153187	thrab	0.001402519	-0.362356661	-0.551240408	-0.403932333	-0.192068079	downregulated during regeneration - early
ENSDART00000153247	si:ch211-120g10.1	0.002147221	-0.520249419	-0.414424265	-0.454570577	-0.107523144	downregulated during regeneration - early
ENSDART00000153256	cacna1g	0.001006824	-0.53708851	-0.717133052	-0.415346006	-0.243050395	downregulated during regeneration - early
ENSDART00000153391	otofa	0.002551958	-0.790883428	-1.357026899	-0.581227909	-0.190433713	downregulated during regeneration - early
ENSDART00000153591	si:dkey-56f14.7	0.023379519	-0.35474859	-0.1685547	-0.219665386	-0.050831688	downregulated during regeneration - early
ENSDART00000153673	kcnb1	0.00060684	-0.632196562	-0.836186932	-0.481100002	-0.109364665	downregulated during regeneration - early
ENSDART00000153925	fut9a	0.002016009	-0.379466906	-0.312742341	-0.333889875	-0.027131911	downregulated during regeneration - early
ENSDART00000153937	prkd1	0.021615739	-0.431650217	-0.382419593	-0.495097396	-0.310742086	downregulated during regeneration - early
ENSDART00000154132	nptx2a	0.03060143	-0.534082272	-0.549346998	-0.399917367	-0.21269489	downregulated during regeneration - early
ENSDART00000154148	camk1a	9.45963E-05	-0.513931344	-0.665527159	-0.499415187	-0.020106333	downregulated during regeneration - early
ENSDART00000154187	fat3a	0.00879177	-0.16861177	-0.487078475	-0.285722941	-0.100061338	downregulated during regeneration - early
ENSDART00000154228	sgip1a	0.019808116	-0.210460002	-0.370824353	-0.364544583	-0.177769156	downregulated during regeneration - early
ENSDART00000154238	AL928908.3	0.098059417	-0.284715118	-0.465774511	-0.246230328	-0.209644064	downregulated during regeneration - early
ENSDART00000154437	dyrk1ab	0.025500103	-0.405599591	-0.293606249	-0.153631672	-0.200586701	downregulated during regeneration - early
ENSDART00000154539	slc17a7b	0.013310088	-0.194590302	-0.417792811	-0.270877884	0.095165091	downregulated during regeneration - early
ENSDART00000154679	ypel2b	0.000167073	-0.575722509	-0.671947843	-0.397167717	-0.03983803	downregulated during regeneration - early
ENSDART00000154682	CR847531.1	0.002649033	-0.541034447	-0.676553431	-0.554824044	-0.149530967	downregulated during regeneration - early
ENSDART00000154732	ptprt	0.007520653	-0.128635692	-0.441586772	-0.224155866	-0.045463061	downregulated during regeneration - early
ENSDART00000154760	CU302436.3	0.000164423	-0.931829111	-0.859047685	-0.631190961	-0.184130931	downregulated during regeneration - early
ENSDART00000154778	map1ab	0.016443396	-0.607759134	-0.711103734	-0.339973576	-0.303457691	downregulated during regeneration - early
ENSDART00000154783	BX511311.5	0.001907584	-0.420893047	-0.406058694	-0.342057398	-0.24848029	downregulated during regeneration - early
ENSDART00000154916	p4htm	0.025523567	-0.322717782	-0.242652877	-0.246255125	-0.141036056	downregulated during regeneration - early
ENSDART00000155108	si:ch211-153l6.6	0.024999915	-0.396073461	-0.324628235	-0.330118035	-0.333690214	downregulated during regeneration - early
ENSDART00000155229	rxfp3.3b	0.002524433	-0.899836922	-0.961252446	-0.631060044	-0.38629797	downregulated during regeneration - early
ENSDART00000155243	BX571762.2	0.000109677	-0.58394173	-0.66558831	-0.494788274	-0.139509269	downregulated during regeneration - early
ENSDART00000155346	tbc1d24	0.000529346	-0.444989153	-0.328605075	-0.340554332	-0.035933185	downregulated during regeneration - early
ENSDART00000155480	ptprnb	0.001147964	-0.501074747	-0.734898939	-0.55867421	-0.198202123	downregulated during regeneration - early
ENSDART00000155496	gal3st2	0.005444477	-0.347287801	-0.358994611	-0.502209455	0.011977936	downregulated during regeneration - early
ENSDART00000155539	CR786577.1	0.00335169	-0.296712547	-0.710798753	-0.341045728	0.032686751	downregulated during regeneration - early
ENSDART00000155786	adgrl1a	0.019467351	-0.190147203	-0.479741899	-0.292479007	0.056973675	downregulated during regeneration - early
ENSDART00000155821	kcnt2	0.00366084	-1.064051569	-1.353278896	-1.209879321	-0.444718597	downregulated during regeneration - early

ENSDART00000155853	gprc5ba	0.000668535	-0.568480744	-0.680176078	-0.476445621	-0.089942946	downregulated during regeneration - early
ENSDART00000155865	tmtops2a	0.015325737	-0.317973372	-0.37404155	-0.204729003	-0.178609254	downregulated during regeneration - early
ENSDART00000156019	phlpp1	0.010699261	-0.378155747	-0.257656641	-0.150573304	-0.126584813	downregulated during regeneration - early
ENSDART00000156280	si:ch211-195b15.7	0.04283071	-0.343611395	-0.333351498	-0.20091334	-0.160599804	downregulated during regeneration - early
ENSDART00000156340	si:ch211-167b20.8	0.018211877	-0.291865267	-0.289158159	-0.304691219	-0.111151074	downregulated during regeneration - early
ENSDART00000156351	zfat	0.027293911	-0.386268069	-0.362611932	-0.387180541	-0.301395116	downregulated during regeneration - early
ENSDART00000156418	itpk1b	0.001814914	-0.567310725	-0.6808899	-0.20043499	-0.215676742	downregulated during regeneration - early
ENSDART00000156447	ap2a1	0.048872862	-0.168026562	-0.400633541	-0.149036642	-0.01932092	downregulated during regeneration - early
ENSDART00000156473	CR627483.1	0.003693464	-0.446874417	-0.944979531	-0.551426346	-0.288797786	downregulated during regeneration - early
ENSDART00000156574	si:ch211-270g19.5	0.033836445	-0.160521866	-0.305818991	-0.352315858	-0.223463705	downregulated during regeneration - early
ENSDART00000156615	amigo3	0.011421096	-0.174881747	-0.348495554	-0.328942663	0.002458748	downregulated during regeneration - early
ENSDART00000156741	BX663503.2	0.000389264	-0.41351136	-0.477289266	-0.358517546	-0.050787842	downregulated during regeneration - early
ENSDART00000156760	grik4	0.048815993	-0.347451056	-0.634709172	-0.373537369	-0.211041818	downregulated during regeneration - early
ENSDART00000156864	CR376745.2	0.003637926	-0.465037351	-0.777375633	-0.748299595	-0.606506499	downregulated during regeneration - early
ENSDART00000156877	prss56	0.031769506	-1.154037343	-0.806544973	-0.688753663	-0.465249106	downregulated during regeneration - early
ENSDART00000156923	plekhd1	0.012263789	-0.640317774	-0.396125765	-0.428766409	-0.43244988	downregulated during regeneration - early
ENSDART00000156928	nrxn3a	0.007397439	0.020925857	-0.341808434	-0.239366202	0.052782357	downregulated during regeneration - early
ENSDART00000157043	rtn4r	0.022328328	-0.358605066	-0.3593825	-0.41712234	-0.136427457	downregulated during regeneration - early
ENSDART00000157124	CT033825.1	0.001443362	-0.560424235	-0.809708864	-0.680777647	-0.144372819	downregulated during regeneration - early
ENSDART00000157129	slc12a5a	0.000109677	-0.482400538	-0.74419089	-0.401518291	-0.07447909	downregulated during regeneration - early
ENSDART00000157195	ncam2	0.001019802	-0.590792178	-0.719154845	-0.40764277	0.002619526	downregulated during regeneration - early
ENSDART00000157330	necab3	0.001497317	-0.311593638	-0.396628677	-0.274835563	-0.023399638	downregulated during regeneration - early
ENSDART00000157414	plxna4	0.033833708	-0.256858488	-0.391015088	-0.285048615	-0.106219383	downregulated during regeneration - early
ENSDART00000157535	unc13c	0.004469286	-0.230182256	-0.566319347	-0.291866986	-0.102048139	downregulated during regeneration - early
ENSDART00000157659	camta1b	0.01608096	-0.574265168	-0.690891494	-0.316064045	-0.339000781	downregulated during regeneration - early
ENSDART00000157852	crtac1b	0.042224659	-0.401370563	-0.59197436	-0.358347223	-0.176621049	downregulated during regeneration - early
ENSDART00000157943	TENM2	0.003686025	-0.301363214	-0.846881931	-0.344978545	0.012452	downregulated during regeneration - early
ENSDART00000158169	cacna2d2a	0.001267982	-0.273970943	-0.601526504	-0.288906256	-0.138856816	downregulated during regeneration - early
ENSDART00000158204	ntrk3a	0.003654787	-0.448230073	-0.669954401	-0.450935682	-0.114414424	downregulated during regeneration - early
ENSDART00000158259	lrtm1	0.053954169	-0.264679565	-0.523836662	-0.478235847	-0.361630227	downregulated during regeneration - early
ENSDART00000158294	ppp2r2cb	0.007536007	-0.427479199	-0.698226267	-0.466339082	-0.260646218	downregulated during regeneration - early
ENSDART00000158358	stk40	0.042731689	-0.305579471	-0.394234001	-0.179882029	-0.355392322	downregulated during regeneration - early
ENSDART00000158515	NA	0.009800471	-0.601694587	-0.246037227	-0.439227399	-0.290329932	downregulated during regeneration - early
ENSDART00000158540	PRKCA (1 of many)	0.021044962	-0.338994629	-0.305121271	-0.237610526	-0.160203254	downregulated during regeneration - early
ENSDART00000158820	golga7bb	0.029287312	-0.441549718	-0.650879189	-0.491036037	-0.164497612	downregulated during regeneration - early
ENSDART00000158919	lpar1	0.053989613	-0.789836287	-0.638879508	-0.710773837	-0.465534721	downregulated during regeneration - early
ENSDART00000159087	drd2b	0.000503033	-0.654775306	-0.766659414	-0.766987138	-0.309487495	downregulated during regeneration - early
ENSDART00000159163	c2cd2l	0.008021107	-0.400916824	-0.28913261	-0.285175907	-0.160469096	downregulated during regeneration - early
ENSDART00000159165	b3galt2	0.001631005	-0.421223634	-0.395995108	-0.436113716	-0.390524412	downregulated during regeneration - early
ENSDART00000159368	myo19	0.038545701	-0.061571264	-0.562765185	-0.263758975	-0.061266965	downregulated during regeneration - early
ENSDART00000159493	baiap2a	0.031365399	-0.408759075	-0.584635172	-0.205977447	-0.074404323	downregulated during regeneration - early
ENSDART00000159523	BX248501.1	0.045817107	-0.802081931	-1.281225474	-1.178391388	-0.841643515	downregulated during regeneration - early
ENSDART00000159604	mbd3b	0.065285449	-0.404570692	-0.312781624	-0.318828454	-0.248466452	downregulated during regeneration - early
ENSDART00000159608	fstl5	0.043959869	-0.296329015	-0.522282128	-0.291629941	-0.144161607	downregulated during regeneration - early
ENSDART00000159745	PCP4L1 (1 of many)	7.10385E-05	-0.632453626	-0.700162017	-0.354659005	-0.133179733	downregulated during regeneration - early
ENSDART00000159769	pam	0.003805801	-0.468861179	-0.403502349	-0.282541701	-0.241769832	downregulated during regeneration - early
ENSDART00000159916	tns1a	0.00347249	-0.270694335	-0.694531751	-0.372872148	-0.014128978	downregulated during regeneration - early
ENSDART00000160236	fras1	0.023812224	-0.444181856	-0.638830186	-0.533612869	-0.242512624	downregulated during regeneration - early
ENSDART00000160328	zgc:109949	0.017825781	-0.362552375	-0.659300829	-0.471802707	-0.430381288	downregulated during regeneration - early
ENSDART00000160337	si:ch73-299h12.8	0.056707482	-0.912707831	-0.414120081	-0.53567861	-0.405510308	downregulated during regeneration - early
ENSDART00000160542	cabp2a	0.013233841	-0.310654407	-0.386778791	-0.172317372	-0.340396034	downregulated during regeneration - early
ENSDART00000160555	sema6e	0.029524772	-0.284106568	-0.324928323	-0.250029521	-0.168860793	downregulated during regeneration - early
ENSDART00000160652	iqsec3a	0.002605308	-0.564945044	-0.609250827	-0.334399494	-0.124185292	downregulated during regeneration - early
ENSDART00000160927	cers1	0.004232418	-0.519571193	-0.339170129	-0.296459142	-0.066758323	downregulated during regeneration - early
ENSDART00000161121	oxct1a	0.011687043	-0.18384202	-0.521566935	-0.230939432	-0.260776637	downregulated during regeneration - early
ENSDART00000161156	kcnj9	0.000230274	-0.647175508	-0.597438502	-0.398278727	-0.23455974	downregulated during regeneration - early
ENSDART00000161250	grin1b	0.009000055	-0.242810387	-0.349766254	-0.246157691	0.067747546	downregulated during regeneration - early
ENSDART00000161272	dclk1a	0.011155635	-0.780154894	-0.444639927	-0.375433616	-0.486681237	downregulated during regeneration - early
ENSDART00000161297	lrrn3b	0.008563	-0.369968613	-0.470809526	-0.36045804	-0.046069156	downregulated during regeneration - early
ENSDART00000161414	meis2a	0.012130485	-0.352024462	-0.259692709	-0.258698057	-0.07620107	downregulated during regeneration - early
ENSDART00000161533	gfra1b	0.017199091	-0.396684985	-0.507239408	-0.32341327	-0.285757426	downregulated during regeneration - early
ENSDART00000161698	spock1	0.008495106	-0.400537237	-0.522818259	-0.36383883	-0.3494177	downregulated during regeneration - early
ENSDART00000161708	si:ch73-29l19.1	0.048892164	-0.183985257	-0.352166832	-0.310452263	-0.095145467	downregulated during regeneration - early
ENSDART00000161735	flrt1a	0.026767073	-0.150942569	-0.355176624	-0.261636355	-0.097731299	downregulated during regeneration - early
ENSDART00000161798	si:ch211-188f17.1	0.022416821	-0.190236489	-0.379885925	-0.177844783	-0.0015826	downregulated during regeneration - early
ENSDART00000161828	si:rp71-1h20.9	0.000291847	-1.646379844	-1.190834107	-1.032421214	-1.471047696	downregulated during regeneration - early
ENSDART00000161938	si:dkeyp-9d4.3	0.040982425	-0.230392301	-0.461066854	-0.432261332	-0.276495229	downregulated during regeneration - early
ENSDART00000162063	ppifb	0.003061973	-0.192515925	-0.861096392	-0.286677305	-0.512048401	downregulated during regeneration - early
ENSDART00000162222	SHC3	0.03131236	-0.255397657	-0.359792108	-0.178043549	-0.323533675	downregulated during regeneration - early
ENSDART00000162228	grip2a	0.010667361	-0.209580983	-0.468643062	-0.36247099	-0.147880595	downregulated during regeneration - early
ENSDART00000162331	si:dkey-22o22.2	0.015731546	-0.434953721	-0.633123757	-0.287732073	-0.108064019	downregulated during regeneration - early
ENSDART00000162387	zbtb38	0.015925333	-0.362042869	-0.285747988	-0.199364681	-0.27814593	downregulated during regeneration - early
ENSDART00000162403	napgb	0.030278232	-0.42647327	-0.557407706	-0.383093342	-0.343712014	downregulated during regeneration - early
ENSDART00000162595	camk2g1	0.032809386	-0.045798893	-0.36296086	-0.180481912	0.032536235	downregulated during regeneration - early
ENSDART00000162683	trappc9	0.067505731	-0.370182614	-0.24071592	-0.273499446	-0.133452933	downregulated during regeneration - early

ENSDART00000162711	cnksr2b	0.026706504	-0.20949159	-0.28214605	-0.418907028	-0.082150791	downregulated during regeneration - early
ENSDART00000162732	NA	0.000536544	-0.497798966	-0.517625334	-0.441909965	-0.132483146	downregulated during regeneration - early
ENSDART00000162804	KCNT1 (1 of many)	0.004678129	-0.406408577	-0.48233323	-0.378259831	0.110845983	downregulated during regeneration - early
ENSDART00000162857	nr4a3	0.003361003	-1.482016444	-1.152680943	-0.606031454	-1.193862677	downregulated during regeneration - early
ENSDART00000162886	il1rapl1b	0.006723268	-0.535131005	-0.60836138	-0.536159373	-0.086785386	downregulated during regeneration - early
ENSDART00000162916	si:ch211-177d9.1	0.017970224	-0.561077771	-0.777629972	-0.458457407	-0.043076344	downregulated during regeneration - early
ENSDART00000163106	cntnap5l	0.001234581	-0.230959418	-0.661142608	-0.469168415	-0.076363144	downregulated during regeneration - early
ENSDART00000163380	si:dkey-22o22.2	0.002605308	-0.500713087	-0.482673389	-0.426086075	-0.212897339	downregulated during regeneration - early
ENSDART00000163491	reep6	0.023753852	-1.146193953	-0.500723418	-0.964434532	-0.007386228	downregulated during regeneration - early
ENSDART00000163523	sez6l	0.009922627	-0.314616095	-0.257996779	-0.399166894	-0.159678617	downregulated during regeneration - early
ENSDART00000163568	kcnc1a	0.003649921	-0.672590813	-0.97944076	-0.78875743	-0.1747006	downregulated during regeneration - early
ENSDART00000163597	CABZ01029366.1	0.009204947	-0.380235128	-0.253799187	-0.219703335	-0.156720283	downregulated during regeneration - early
ENSDART00000163793	slitrk6	0.012145748	-0.404346501	-0.384982132	-0.24261489	-0.144542921	downregulated during regeneration - early
ENSDART00000163892	ldha	0.068646243	-0.444310122	-0.296598203	-0.299673411	-0.138453042	downregulated during regeneration - early
ENSDART00000163952	zgc:110045	0.056216274	-0.36736834	-0.55642828	-0.201308362	-0.286026212	downregulated during regeneration - early
ENSDART00000164015	zgc:66483	0.030464157	-0.233929761	-0.339147918	-0.342271085	-0.145997916	downregulated during regeneration - early
ENSDART00000164129	asic4b	2.97728E-05	-0.92148029	-1.144921239	-0.577321683	-0.304835302	downregulated during regeneration - early
ENSDART00000164506	dlg1l	0.011264952	-0.401430092	-0.615035177	-0.528577315	-0.282785588	downregulated during regeneration - early
ENSDART00000164581	galr2b	0.012570369	-0.842297369	-0.875318855	-0.561173438	-0.495794792	downregulated during regeneration - early
ENSDART00000164621	ndrg4	0.023203992	-0.368994826	-0.243279912	-0.209623349	-0.144971204	downregulated during regeneration - early
ENSDART00000164623	ptn	0.025195087	-0.285740219	-0.301123559	-0.395585979	-0.169574914	downregulated during regeneration - early
ENSDART00000164693	CT583672.1	0.003678598	-0.40975231	-0.465896033	-0.295925202	-0.161007007	downregulated during regeneration - early
ENSDART00000164733	43358	0.015015416	-0.353566412	-0.328984592	-0.353599969	-0.269459941	downregulated during regeneration - early
ENSDART00000164844	rictora	0.015981762	-0.308713132	-0.348726405	-0.242009414	-0.189747444	downregulated during regeneration - early
ENSDART00000164890	pxdc1a	0.02057139	-0.553855303	-0.268569574	-0.515208528	-0.163588555	downregulated during regeneration - early
ENSDART00000164979	ARHGAP44 (1 of many)	0.017944789	-0.298736282	-0.611440452	-0.190082373	-0.04950856	downregulated during regeneration - early
ENSDART00000165115	adcy3a	0.026979418	-0.345722559	-0.440118918	-0.380340005	-0.11083624	downregulated during regeneration - early
ENSDART00000165124	si:ch73-213k20.5	0.021047643	-0.366310901	-0.428402723	-0.397554725	-0.148030091	downregulated during regeneration - early
ENSDART00000165292	nsmfb	0.006359324	-0.267190978	-0.447188182	-0.263868703	-0.115307671	downregulated during regeneration - early
ENSDART00000165370	nxph2b	0.001996892	-0.350612818	-0.344729721	-0.406615862	-0.05363573	downregulated during regeneration - early
ENSDART00000165411	NA	0.000228491	-0.599585032	-0.699498667	-0.398460986	-0.09881633	downregulated during regeneration - early
ENSDART00000165423	abcc8b	0.052957916	-0.169557865	-0.54863705	-0.322687745	-0.280461618	downregulated during regeneration - early
ENSDART00000165448	rims2b	0.01901087	-0.321612136	-0.490793078	-0.295423658	0.030315799	downregulated during regeneration - early
ENSDART00000165743	gabrg2	0.002854235	-0.320626657	-0.470320689	-0.258833602	0.01857369	downregulated during regeneration - early
ENSDART00000165898	gbe1b	0.008897545	-0.311269447	-0.395300505	-0.303280984	-0.17462698	downregulated during regeneration - early
ENSDART00000165903	slmapa	0.006879176	-5.974312545	-3.688098031	-4.583524263	-0.943141088	downregulated during regeneration - early
ENSDART00000165912	si:ch73-380n15.2	0.000461528	-0.521676191	-0.515528635	-0.335328887	-0.345110245	downregulated during regeneration - early
ENSDART00000165979	sncgb	0.004112749	-0.522103986	-0.516181722	-0.379782599	-0.140861461	downregulated during regeneration - early
ENSDART00000166025	irs2a	0.03450478	-0.31567938	-0.330763757	-0.333387068	-0.269837586	downregulated during regeneration - early
ENSDART00000166027	trpc1	0.019215781	-0.303725684	-0.42864481	-0.334157939	-0.231516525	downregulated during regeneration - early
ENSDART00000166042	vipr2	0.00847573	-0.341756427	-0.277003119	-0.379732317	-0.038627099	downregulated during regeneration - early
ENSDART00000166135	zbtb47b	0.004311817	-0.402709337	-0.409852132	-0.474957603	-0.394147628	downregulated during regeneration - early
ENSDART00000166148	gabra1	3.38347E-05	-0.578656795	-0.788703398	-0.59715214	-0.072809071	downregulated during regeneration - early
ENSDART00000166308	cib2	0.004624842	-0.434731659	-0.335262372	-0.470437684	-0.240772493	downregulated during regeneration - early
ENSDART00000166531	pcdh15b	0.014638227	-0.215971807	-0.468745045	-0.336826359	0.084344716	downregulated during regeneration - early
ENSDART00000166681	frrs1l	0.005203275	-0.4975582	-0.505651734	-0.29386319	-0.136005256	downregulated during regeneration - early
ENSDART00000166730	slitrk4	0.026033323	-0.433556166	-0.537605748	-0.487948345	-0.301835783	downregulated during regeneration - early
ENSDART00000166889	nptna	0.001225897	-0.194507678	-0.523176341	-0.288117341	0.166715743	downregulated during regeneration - early
ENSDART00000166957	purba	0.055560475	-0.28509761	-0.466147452	-0.146018067	-0.191067595	downregulated during regeneration - early
ENSDART00000167052	etv1	0.01072353	-0.327639473	-0.469002174	-0.32895701	-0.22464313	downregulated during regeneration - early
ENSDART00000167164	MPV17L	0.002297766	-0.408335699	-0.461800821	-0.169486828	-0.082955483	downregulated during regeneration - early
ENSDART00000167451	NA	0.03168371	-0.147201966	-0.622186255	-0.321157937	-0.196934609	downregulated during regeneration - early
ENSDART00000167502	CR855860.1	0.030485193	-0.377799337	-0.542912736	-0.423594114	-0.115674527	downregulated during regeneration - early
ENSDART00000167664	atxn1a	0.004580336	-0.182914727	-0.487043114	-0.17911118	-0.100136551	downregulated during regeneration - early
ENSDART00000167847	eef1a1l2	0.028902271	-0.649781637	-0.501730111	-0.557800044	-0.257462283	downregulated during regeneration - early
ENSDART00000168089	cyp27a7	0.044240808	-0.742372496	-0.705345887	-0.793489907	-0.543448957	downregulated during regeneration - early
ENSDART00000168310	acap3b	0.015781384	-0.431660294	-0.622927148	-0.474200982	-0.250587028	downregulated during regeneration - early
ENSDART00000168518	asic4b	2.97728E-05	-0.92148029	-1.144921239	-0.577321683	-0.304835302	downregulated during regeneration - early
ENSDART00000168534	NPFFR2 (1 of many)	0.012462623	-0.514269049	-0.40572501	-0.533037042	-0.336902595	downregulated during regeneration - early
ENSDART00000168631	cacna1hb	0.008579097	-0.565978595	-0.722362792	-0.435741841	-0.029665329	downregulated during regeneration - early
ENSDART00000168718	chrm5a	0.033210482	-0.32077931	-0.748869105	-0.317792694	-0.07500967	downregulated during regeneration - early
ENSDART00000168754	cacnb2a	0.028142339	-0.312716955	-0.298709564	-0.211334034	-0.110938146	downregulated during regeneration - early
ENSDART00000168821	CDH22	0.019500385	-0.242258311	-0.624670054	-0.303879761	-0.070304381	downregulated during regeneration - early
ENSDART00000168920	nbeab	0.028147746	-0.251742312	-0.363594185	-0.174304201	-0.037829821	downregulated during regeneration - early
ENSDART00000169040	si:ch211-235e9.8	0.001532808	-0.436326332	-0.357257361	-0.23322888	-0.084450729	downregulated during regeneration - early
ENSDART00000169108	kcnj12a	0.013360877	-0.480725794	-0.616905664	-0.509410819	-0.355199716	downregulated during regeneration - early
ENSDART00000169136	CABZ01007222.1	0.002957541	-0.363042867	-0.477045243	-0.379599521	-0.116458215	downregulated during regeneration - early
ENSDART00000169548	rgs7b	0.024602158	-0.179117493	-0.337814246	-0.235695562	-0.091038288	downregulated during regeneration - early
ENSDART00000169609	tefb	0.032368681	-0.156114581	-0.555141888	-0.301378906	-0.24132076	downregulated during regeneration - early
ENSDART00000169687	dlg1	0.023714388	-0.200796553	-0.39742906	-0.281934456	-0.007509175	downregulated during regeneration - early
ENSDART00000169746	ampd2a	0.000935288	-0.446564291	-0.523779729	-0.426555345	-0.117905151	downregulated during regeneration - early
ENSDART00000169764	syt1a	0.009024763	-0.535633412	-0.482595993	-0.403145231	-0.029612735	downregulated during regeneration - early
ENSDART00000169810	stub1	0.029463211	-0.352758303	-0.24873956	-0.212157701	-0.100812458	downregulated during regeneration - early
ENSDART00000170362	CABZ01088490.1	0.001365289	-0.602203241	-0.75748097	-0.591873288	-0.034015136	downregulated during regeneration - early
ENSDART00000170709	st8sia1	0.007584916	-0.358435626	-0.336878724	-0.21402261	-0.093884674	downregulated during regeneration - early

ENSDART00000170993	afmid	0.002016009	-0.391958822	-0.311185654	-0.48659874	-0.071120804	downregulated during regeneration - early
ENSDART00000170998	tnrc6c2	0.033092405	-0.257719977	-0.319066529	-0.317919253	-0.242876098	downregulated during regeneration - early
ENSDART00000171014	ptprfa	0.004256148	-0.231763582	-0.479834972	-0.30404021	-0.129420632	downregulated during regeneration - early
ENSDART00000171041	tceb3	0.028527553	-0.063048764	-0.305499385	-0.056078786	-0.065276347	downregulated during regeneration - early
ENSDART00000171182	FO704914.1	0.000532548	-0.372200999	-0.770608409	-0.45129487	-0.014659406	downregulated during regeneration - early
ENSDART00000171215	slc6a3	0.001490782	-0.667608441	-0.754783262	-0.446986408	-0.343120625	downregulated during regeneration - early
ENSDART00000171426	pdzph1	0.00721984	-0.416069913	-0.411889039	-0.457121209	-0.221062124	downregulated during regeneration - early
ENSDART00000171496	CDK18	0.00320961	-0.371495713	-0.374286185	-0.433391037	-0.282164654	downregulated during regeneration - early
ENSDART00000171523	CABZ01089030.1	0.001040281	-0.303576632	-0.51118876	-0.528168137	-0.25789403	downregulated during regeneration - early
ENSDART00000171683	GABRG1	0.028772568	-0.455034367	-0.392373746	-0.437520168	-0.270886274	downregulated during regeneration - early
ENSDART00000171696	sergef	0.00024376	-0.83692277	-0.920324583	-0.711315475	-0.040508735	downregulated during regeneration - early
ENSDART00000171711	gpsm1a	0.009294776	-0.376386863	-0.294884117	-0.277096957	-0.079488929	downregulated during regeneration - early
ENSDART00000171728	FQ323156.1	0.000261206	-0.3925045	-0.548422409	-0.578285827	-0.21374206	downregulated during regeneration - early
ENSDART00000171743	sypa	0.000648054	-0.458751426	-0.455626528	-0.334063629	-0.142465032	downregulated during regeneration - early
ENSDART00000171749	TMEM150A (1 of many)	0.00159523	-0.745537318	-0.560172793	-0.666392254	-0.39318407	downregulated during regeneration - early
ENSDART00000171762	arhgef11	0.023596757	-0.293734421	-0.490220654	-0.20280274	0.049910063	downregulated during regeneration - early
ENSDART00000171777	syt7b	0.000155554	-0.587310767	-0.899783938	-0.612333236	-0.170082745	downregulated during regeneration - early
ENSDART00000171868	sgut1	0.013366886	-0.438334002	-0.252211506	-0.468859348	-0.232780566	downregulated during regeneration - early
ENSDART00000171935	brpf3a	0.010653756	-0.333420883	-0.26685281	-0.204490748	-0.091216638	downregulated during regeneration - early
ENSDART00000171948	zgc:172282	0.000409858	-0.447105187	-0.64749442	-0.36124568	-0.081452365	downregulated during regeneration - early
ENSDART00000172045	zgc:73340	0.004702472	-0.432558368	-0.571519732	-0.606453829	-0.389736033	downregulated during regeneration - early
ENSDART00000172128	sez6l	0.001036975	-0.457515145	-0.649880208	-0.297003963	-0.214224661	downregulated during regeneration - early
ENSDART00000172149	sh3rf2	0.000935288	-0.359308533	-0.521296357	-0.501584275	-0.200324498	downregulated during regeneration - early
ENSDART00000172218	nsmfb	0.001185822	-0.786748472	-1.480880504	-0.428989171	-0.523180434	downregulated during regeneration - early
ENSDART00000172267	KCNV1	0.026772289	-0.730291266	-0.683938113	-0.423486953	-0.317970814	downregulated during regeneration - early
ENSDART00000172310	zbtb4	0.013310088	-0.365422353	-0.277101341	-0.237436935	-0.247791483	downregulated during regeneration - early
ENSDART00000172329	CABZ01084963.1	0.005531203	-0.076477014	-0.479017439	-0.153366972	-0.215592031	downregulated during regeneration - early
ENSDART00000172336	cabp2a	0.007145684	-0.40143904	-0.491969769	-0.23184904	-0.50001549	downregulated during regeneration - early
ENSDART00000172337	rhot1a	0.009491292	-3.878236672	-5.686158747	-0.665991717	-0.822715826	downregulated during regeneration - early
ENSDART00000172373	ompa	0.040539001	-0.333379791	-0.33389964	-0.383107757	-0.229559723	downregulated during regeneration - early
ENSDART00000172496	add3a	0.056985155	-0.173242509	-0.314301562	-0.183893521	-0.110146899	downregulated during regeneration - early
ENSDART00000172661	nrgnb	0.0006387	-0.441646104	-0.659637875	-0.590118296	-0.403632798	downregulated during regeneration - early
ENSDART00000172689	drd4a	0.00510387	-0.269065132	-0.433761644	-0.356034882	-0.090259185	downregulated during regeneration - early
ENSDART00000172862	kcnj14	0.044172739	-0.439592228	-0.323667837	-0.482461949	-0.197270237	downregulated during regeneration - early
ENSDART00000173109	nrtn	0.012342358	-0.527610722	-0.443827628	-0.46689713	-0.285016979	downregulated during regeneration - early
ENSDART00000173113	si:ch211-129p13.1	0.001328724	-0.464452233	-0.696136912	-0.478469763	-0.206239945	downregulated during regeneration - early
ENSDART00000173119	pcdh11	0.000993187	-0.634370446	-0.732192902	-0.531501202	-0.25060062	downregulated during regeneration - early
ENSDART00000173133	utrn	0.064203588	-0.11206335	-0.324597481	-0.223274112	-0.197393841	downregulated during regeneration - early
ENSDART00000173134	BX571952.1	0.000520606	-0.551010684	-0.633352911	-0.460069257	-0.004126181	downregulated during regeneration - early
ENSDART00000173386	cacna1da	0.028175613	-0.09786225	-0.344688682	-0.169985603	0.048564501	downregulated during regeneration - early
ENSDART00000000069	slc9a3r1a	0.024869861	-0.019828621	-0.165096913	-0.579587833	-0.318451026	downregulated during regeneration - mid
ENSDART00000000192	ptpn4b	0.005203881	-0.253123991	-0.355280061	-0.416236309	-0.192007	downregulated during regeneration - mid
ENSDART00000002029	fkbp8	0.030637152	-0.081020923	-0.107047698	-0.329759128	-0.094897987	downregulated during regeneration - mid
ENSDART00000003170	mid1ip1l	0.009183287	-0.359873821	-0.329776238	-0.564188118	-0.321447922	downregulated during regeneration - mid
ENSDART00000003335	snx12	0.02794941	-0.102084433	-0.181980514	-0.47779818	-0.16493026	downregulated during regeneration - mid
ENSDART00000003465	gipc2	0.02229367	-0.142551263	-0.216969239	-0.344609382	-0.269869981	downregulated during regeneration - mid
ENSDART00000003939	syngr1a	0.024987601	0.053450312	-0.149997984	-0.371497084	-0.066149387	downregulated during regeneration - mid
ENSDART00000004075	uqcc2	0.023840313	-0.167833305	-0.222493804	-0.340331706	-0.224913678	downregulated during regeneration - mid
ENSDART00000004241	inhbaa	0.001317626	0.208890493	-0.32024378	-0.370155553	-0.220482209	downregulated during regeneration - mid
ENSDART00000004420	rab4a	0.034580091	-0.104588348	-0.223085036	-0.330744686	-0.154422823	downregulated during regeneration - mid
ENSDART00000005573	tmem237b	0.009950411	0.093826402	-0.187405072	-0.456194249	-0.210768209	downregulated during regeneration - mid
ENSDART00000005590	churc1	0.018990086	-0.030881068	-0.24423848	-0.319974325	-0.284115834	downregulated during regeneration - mid
ENSDART00000005733	tma16	0.047982796	-0.013223994	-0.144098168	-0.425909208	-0.168461973	downregulated during regeneration - mid
ENSDART00000005957	lrit1a	0.025420775	-0.250152343	-0.282879742	-0.392873609	-0.351289311	downregulated during regeneration - mid
ENSDART00000006927	use1	0.036974502	-0.314698173	-0.230168127	-0.342013543	-0.20782604	downregulated during regeneration - mid
ENSDART00000007021	atp6v1ba	0.031629016	-0.168038972	-0.268033099	-0.367112615	-0.152327119	downregulated during regeneration - mid
ENSDART00000008302	insra	0.048141934	-0.066772744	-0.178139649	-0.319689592	-0.098012672	downregulated during regeneration - mid
ENSDART00000009740	smad7	0.030913639	-0.082405078	-0.352637537	-0.492507075	-0.066549718	downregulated during regeneration - mid
ENSDART00000010378	myo3b	0.013233841	0.121341422	-0.396947151	-0.677290933	-0.232688387	downregulated during regeneration - mid
ENSDART00000011149	fam185a	0.051285619	-0.225870361	-0.305876809	-0.396323932	-0.356813487	downregulated during regeneration - mid
ENSDART00000011229	sub1b	0.026680132	-0.082257398	-0.179031151	-0.405374566	-0.271111897	downregulated during regeneration - mid
ENSDART00000011317	ntm	0.027578317	-0.235410973	-0.452806913	-0.580240928	-0.252707536	downregulated during regeneration - mid
ENSDART00000012229	fkbp1b	0.049049155	-0.199888531	-0.218085861	-0.490853212	-0.25173597	downregulated during regeneration - mid
ENSDART00000012376	gabrr1	0.006630005	-0.444264456	-0.314539223	-0.677749333	-0.339326443	downregulated during regeneration - mid
ENSDART00000012478	mmadhc	0.028172785	-0.097599671	-0.214733382	-0.311326383	-0.186284094	downregulated during regeneration - mid
ENSDART00000012938	phgdh	0.077650287	-0.315221747	-0.241109028	-0.448879252	-0.249449553	downregulated during regeneration - mid
ENSDART00000013411	cahz	0.037241402	-0.282657507	-0.146895201	-0.382063619	-0.133810291	downregulated during regeneration - mid
ENSDART00000013839	tmbim4	0.056276304	-0.093382601	-0.138429676	-0.303111951	-0.191824583	downregulated during regeneration - mid
ENSDART00000014021	slc25a39	0.007324183	-0.175830036	-0.233948732	-0.630252381	-0.299226836	downregulated during regeneration - mid
ENSDART00000014031	dpf2	0.020424071	0.062076591	-0.192206303	-0.356876024	-0.045146421	downregulated during regeneration - mid
ENSDART00000014632	katnb1	0.009776962	-0.1660519	-0.366507385	-0.386560357	-0.02476169	downregulated during regeneration - mid
ENSDART00000015710	snrkb	0.033001298	-0.234771599	-0.444259361	-0.383002363	-0.24868656	downregulated during regeneration - mid
ENSDART00000015755	rasl11b	0.004084205	-0.21929532	-0.630137444	-0.720127948	-0.404206999	downregulated during regeneration - mid
ENSDART00000015951	bsg	0.037560436	-0.039046362	-0.213267965	-0.489263219	-0.308938953	downregulated during regeneration - mid
ENSDART00000016099	CASKIN2	0.026969746	-0.196661177	-0.318487444	-0.498724654	-0.242993173	downregulated during regeneration - mid

ENSDART00000016803	grpel1	0.047568271	-0.106418203	-0.12256904	-0.467582595	-0.298130125	downregulated during regeneration - mid
ENSDART00000018047	zgc:112294	0.033754546	-0.129085452	-0.359907528	-0.863079854	-0.32905376	downregulated during regeneration - mid
ENSDART00000018117	ppp1r14aa	0.009559586	-0.10674343	-0.179038105	-0.425069279	-0.232436913	downregulated during regeneration - mid
ENSDART00000018972	zgc:92818	0.010173835	0.012004445	-0.040419062	-0.784852086	-0.38349701	downregulated during regeneration - mid
ENSDART00000019053	faima	0.060563605	-0.147906168	-0.161120397	-0.359996788	-0.164587924	downregulated during regeneration - mid
ENSDART00000021260	sept8b	0.002390041	-0.246039495	-0.120094514	-0.518439508	-0.250786208	downregulated during regeneration - mid
ENSDART00000021346	arl3l2	0.060063095	-0.339022452	-0.304754125	-0.490852638	-0.247921956	downregulated during regeneration - mid
ENSDART00000022393	si:dkeyp-57f11.2	0.005040201	0.163453067	-0.122007101	-0.459561011	0.032532197	downregulated during regeneration - mid
ENSDART00000022688	tob1b	0.00387273	-0.393522131	-0.302261171	-0.612204875	-0.372656833	downregulated during regeneration - mid
ENSDART00000022998	ANO2 (1 of many)	0.026969746	0.005158406	-0.146342409	-0.548931715	-0.196350546	downregulated during regeneration - mid
ENSDART00000023588	guca1a	0.012263789	0.527717677	-0.52699324	-1.299472888	-0.244316084	downregulated during regeneration - mid
ENSDART00000023686	ankrd33ab	0.007529247	-0.23872945	-0.400254403	-1.062972805	-0.551724897	downregulated during regeneration - mid
ENSDART00000023806	zgc:110319	0.067739819	-0.195130618	-0.122813174	-0.337219496	-0.098833156	downregulated during regeneration - mid
ENSDART00000024136	gngt2a	0.005454674	0.175894019	0.078473268	-0.788482627	-0.299650763	downregulated during regeneration - mid
ENSDART00000024331	glsb	0.018752626	0.029280722	-0.254060441	-0.66092512	-0.487127171	downregulated during regeneration - mid
ENSDART00000024619	gorasp1a	0.010796842	-0.145027203	-0.174354089	-0.499227113	-0.186036756	downregulated during regeneration - mid
ENSDART00000025046	ppp1caa	0.031235096	0.04938914	-0.175273742	-0.396680839	-0.049267892	downregulated during regeneration - mid
ENSDART00000025414	slc2a1a	0.029003347	0.059681786	-0.155207235	-0.469818878	-0.137194664	downregulated during regeneration - mid
ENSDART00000025962	gyg1a	0.016443396	-0.132586486	-0.231554909	-0.320101499	-0.261622046	downregulated during regeneration - mid
ENSDART00000026692	ubtd1a	0.001139558	-0.10869502	-0.342281946	-0.985605367	-0.704345293	downregulated during regeneration - mid
ENSDART00000027050	cnga3b	0.004666996	-0.139313288	-0.277577971	-1.175814494	-0.697938349	downregulated during regeneration - mid
ENSDART00000027454	si:ch211-207i1.2	0.016936477	-0.470697042	-0.923472632	-1.21436549	-0.514660002	downregulated during regeneration - mid
ENSDART00000028048	necap1	0.01084285	-0.117195999	-0.341344035	-0.478671947	-0.252467934	downregulated during regeneration - mid
ENSDART00000029459	gipr	0.007928614	-0.120368109	-0.334116608	-0.559985722	-0.423861096	downregulated during regeneration - mid
ENSDART00000029774	tmem55bb	0.04854903	-0.03621571	-0.196356812	-0.337053328	-0.094153869	downregulated during regeneration - mid
ENSDART00000030885	uckl1a	0.010869905	0.183203205	-0.212616494	-0.705475026	-0.013040484	downregulated during regeneration - mid
ENSDART00000030920	gid8a	0.037565208	-0.116648875	-0.277182294	-0.355798783	-0.233597587	downregulated during regeneration - mid
ENSDART00000031426	skilb	0.000177771	-0.234186907	-0.553407151	-0.565936684	-0.420869627	downregulated during regeneration - mid
ENSDART00000031638	slc48a1a	0.029570651	-0.121591299	-0.156663294	-0.340136476	-0.182759405	downregulated during regeneration - mid
ENSDART00000031650	hsp70l	0.005097739	-0.846526969	-1.057845455	-1.357523269	-1.215078404	downregulated during regeneration - mid
ENSDART00000032459	aqp1a.1	0.010333906	-0.275584647	-0.402261221	-0.531838868	-0.410567004	downregulated during regeneration - mid
ENSDART00000033325	slc25a24	0.027461561	-0.429859875	-0.309149544	-0.52291133	-0.286046915	downregulated during regeneration - mid
ENSDART00000034441	tcp11l2	0.019467351	0.020775808	-0.208761169	-0.796222831	-0.248557177	downregulated during regeneration - mid
ENSDART00000034705	ntmt1	0.034977529	-0.020729663	-0.187405157	-0.44452011	-0.329716724	downregulated during regeneration - mid
ENSDART00000036373	cfap206	0.011264004	0.026049802	0.014543106	-0.421468514	-0.063922477	downregulated during regeneration - mid
ENSDART00000037065	sccpdhb	0.030676359	-0.135500672	-0.263394229	-0.35527789	-0.147169147	downregulated during regeneration - mid
ENSDART00000038924	sult1st1	0.028350112	0.0423301	-0.129133641	-0.497646461	-0.249352869	downregulated during regeneration - mid
ENSDART00000039295	lrrfip1a	0.001138119	0.201172846	-0.052744603	-0.60255637	-0.234396047	downregulated during regeneration - mid
ENSDART00000039485	gabarapl2	0.031009754	-0.111002032	-0.162035427	-0.331707696	-0.213225245	downregulated during regeneration - mid
ENSDART00000040116	tnrc5	0.037247868	-0.132560784	-0.181210054	-0.368693498	-0.22290886	downregulated during regeneration - mid
ENSDART00000040346	efr3ba	0.045439062	-0.15754636	-0.295932904	-0.334562517	-0.212090743	downregulated during regeneration - mid
ENSDART00000041417	camk1b	0.063189142	-0.259356662	-0.222277099	-0.408590245	-0.120593834	downregulated during regeneration - mid
ENSDART00000041443	igsf21a	0.078639351	-0.224595347	-0.278632007	-0.419145006	-0.220026937	downregulated during regeneration - mid
ENSDART00000041707	unc119a	0.031766969	-0.21008147	-0.190962771	-0.5453967	-0.153347487	downregulated during regeneration - mid
ENSDART00000042276	nxph1	0.036299878	-0.320960824	-0.220089718	-0.50268354	-0.230788559	downregulated during regeneration - mid
ENSDART00000042572	ablim1b	0.036021217	0.023130185	-0.084172657	-0.818233794	-0.360713717	downregulated during regeneration - mid
ENSDART00000042599	dennd6aa	0.041657173	-0.0532753	-0.029202145	-0.371955611	-0.136277272	downregulated during regeneration - mid
ENSDART00000043492	trappc6bl	0.010042504	-0.12751818	-0.21743347	-0.476970052	-0.302538631	downregulated during regeneration - mid
ENSDART00000043507	ciarta	0.063631477	-0.034891069	-0.156171585	-0.423543489	-0.111653268	downregulated during regeneration - mid
ENSDART00000043801	cabp5b	0.010073304	-0.44296443	-0.237302409	-0.884658288	-0.372660272	downregulated during regeneration - mid
ENSDART00000043855	dclk2a	0.001528476	-0.104441255	-0.395008642	-0.62766162	-0.184810585	downregulated during regeneration - mid
ENSDART00000043953	mfsd2b	0.011605032	-0.226277331	-0.20295362	-0.451440771	0.020277702	downregulated during regeneration - mid
ENSDART00000045303	tmprss9	0.052101742	-0.116725282	-0.194770382	-0.365140883	-0.064254559	downregulated during regeneration - mid
ENSDART00000045374	smad3a	0.043076195	-0.063329624	-0.157712113	-0.303211349	-0.108940629	downregulated during regeneration - mid
ENSDART00000046066	capn1a	0.045050212	-0.174433704	-0.141559786	-0.304523077	-0.134358035	downregulated during regeneration - mid
ENSDART00000046951	ptpn11b	0.011788375	-0.12533711	-0.212829251	-0.391615008	-0.186327808	downregulated during regeneration - mid
ENSDART00000047362	msra	0.006658285	-0.031803809	-0.196438308	-0.422002476	-0.295991402	downregulated during regeneration - mid
ENSDART00000049885	si:dkey-172j4.3	0.036535058	-0.146868316	-0.089398841	-0.317501099	-0.03734398	downregulated during regeneration - mid
ENSDART00000050202	rcan3	0.007786427	-0.240590006	-0.307380747	-0.423427653	-0.130410568	downregulated during regeneration - mid
ENSDART00000050271	hexb	0.004813371	0.237342774	-0.182676311	-0.489143261	-0.233593029	downregulated during regeneration - mid
ENSDART00000050750	rrm2b	0.073861488	-0.324758311	-0.221575036	-0.394470152	-0.266012642	downregulated during regeneration - mid
ENSDART00000051566	zgc:101016	0.024284969	0.072400267	-0.278957969	-0.822407258	-0.192676389	downregulated during regeneration - mid
ENSDART00000052065	si:rp71-39b20.4	0.019747468	-0.313076871	-0.443642109	-0.50831344	-0.2481846	downregulated during regeneration - mid
ENSDART00000052318	mdka	0.007797716	-0.324471926	-0.37363799	-0.54050807	-0.370260207	downregulated during regeneration - mid
ENSDART00000052322	zgc:110699	0.012719677	-0.266905411	-0.248249923	-0.482205646	-0.223841905	downregulated during regeneration - mid
ENSDART00000053367	hmgn3	0.044180927	-0.174648563	-0.165335262	-0.310027237	-0.181225286	downregulated during regeneration - mid
ENSDART00000053494	anks4b	0.048286815	-0.562445249	-0.270911364	-0.580434775	-0.289114333	downregulated during regeneration - mid
ENSDART00000054007	slc8a4b	0.027048639	-0.011734485	-0.299949751	-0.451416872	-0.315264472	downregulated during regeneration - mid
ENSDART00000054408	gsg1l	0.001512737	-0.338795387	-0.334881942	-0.642215658	-0.428166696	downregulated during regeneration - mid
ENSDART00000054472	tll1	0.030023854	-0.241128673	-0.208136	-0.494254448	-0.202017577	downregulated during regeneration - mid
ENSDART00000054760	zgc:162144	0.018655612	-0.154581168	-0.586579914	-0.580706302	-0.278802593	downregulated during regeneration - mid
ENSDART00000055186	atp5j2	0.01462956	-0.168904246	-0.309514947	-0.449394402	-0.201772036	downregulated during regeneration - mid
ENSDART00000055611	isca2	0.041099023	-0.127743819	-0.187288303	-0.524298231	-0.307747827	downregulated during regeneration - mid
ENSDART00000055817	PIGG	0.023931815	-0.041747749	-0.262998575	-0.390921965	-0.198616638	downregulated during regeneration - mid
ENSDART00000056213	pik3r1	0.029029749	-0.34639661	-0.306212461	-0.436349985	-0.247937167	downregulated during regeneration - mid

ENSDART00000056734	setd7	0.028411307	-0.044426299	-0.050437779	-0.332150365	-0.191170985	downregulated during regeneration - mid
ENSDART00000056735	rgs20	0.035861975	-0.263112929	-0.317514258	-0.646869905	-0.425880318	downregulated during regeneration - mid
ENSDART00000056927	egln1a	0.069201585	-0.373304639	-0.331810821	-0.548972472	-0.202283187	downregulated during regeneration - mid
ENSDART00000057918	si:ch211-147h1.4	0.006788162	0.058919084	-0.340722361	-0.680330981	-0.211102009	downregulated during regeneration - mid
ENSDART00000057957	itm2cb	0.004277486	-0.410626815	-0.396999775	-0.657592832	-0.426965619	downregulated during regeneration - mid
ENSDART00000058255	bbs5	0.033037209	-0.153890395	-0.351139568	-0.380364475	-0.12338798	downregulated during regeneration - mid
ENSDART00000058339	ap3s2	0.051751157	-0.205243617	-0.191498501	-0.306412801	-0.176311273	downregulated during regeneration - mid
ENSDART00000058470	pik3r1	0.020653282	-0.272621258	-0.391826992	-0.565271638	-0.177820971	downregulated during regeneration - mid
ENSDART00000058628	ccsapb	0.038389384	-0.109779777	-0.249497929	-0.321372328	-0.06107705	downregulated during regeneration - mid
ENSDART00000058785	fam210ab	0.009653548	-0.074078069	-0.284264661	-0.891770658	-0.393378695	downregulated during regeneration - mid
ENSDART00000058936	scamp5b	0.046680552	-0.278094691	-0.317619737	-0.403892079	-0.134676622	downregulated during regeneration - mid
ENSDART00000060001	pnp6	0.007828891	-0.139557872	-0.321502493	-0.386186078	-0.030509602	downregulated during regeneration - mid
ENSDART00000060015	chka	0.008595135	-0.144459192	-0.274568124	-0.456317122	-0.171918553	downregulated during regeneration - mid
ENSDART00000060184	chka	0.004120762	-0.101617548	-0.321325171	-0.420229119	-0.136541498	downregulated during regeneration - mid
ENSDART00000060532	zgc:110796	0.029635773	-0.139659209	-0.284517331	-0.33969202	-0.16468656	downregulated during regeneration - mid
ENSDART00000061106	bhlhe41	0.000553147	0.043056173	-0.423617996	-0.754959095	-0.246095493	downregulated during regeneration - mid
ENSDART00000061265	rnf141	0.032782977	-0.12379903	-0.215585747	-0.342006011	-0.212187704	downregulated during regeneration - mid
ENSDART00000061745	inpp4ab	0.000385909	-0.337306716	-0.94773917	-0.831022148	-0.394471236	downregulated during regeneration - mid
ENSDART00000062402	tpd52l1	0.005726323	-0.180007689	-0.341056198	-0.772074089	-0.368132956	downregulated during regeneration - mid
ENSDART00000062556	sod2	0.00889295	-0.183508685	-0.287491323	-0.424543766	-0.237833299	downregulated during regeneration - mid
ENSDART00000062935	heca	0.065650579	-0.371152131	-0.569989768	-0.691744059	-0.51577268	downregulated during regeneration - mid
ENSDART00000063835	otx5	0.082497078	-0.207695123	-0.226833273	-0.417236259	-0.126675877	downregulated during regeneration - mid
ENSDART00000063944	tmem30ab	0.018132587	-0.052321591	-0.186777496	-0.351757869	-0.167369044	downregulated during regeneration - mid
ENSDART00000064738	atpif1b	0.005335064	-0.12884804	-0.286001583	-0.530012586	-0.266598201	downregulated during regeneration - mid
ENSDART00000064842	padi2	0.046738515	-0.110475283	-0.200123181	-0.394420172	-0.195593685	downregulated during regeneration - mid
ENSDART00000064913	fto	0.054038459	-0.098234146	-0.428383355	-0.257568512	-0.239748235	downregulated during regeneration - mid
ENSDART00000065143	unc119b	0.020870612	-0.086346577	-0.282642438	-0.651558775	-0.265879719	downregulated during regeneration - mid
ENSDART00000065467	dedd1	0.0321404	-0.00326722	-0.100306912	-0.35251663	-0.041277003	downregulated during regeneration - mid
ENSDART00000065755	gpn3	0.095709295	-0.172157475	-0.155442383	-0.381057864	-0.19097226	downregulated during regeneration - mid
ENSDART00000066256	vti1a	0.042541047	-0.147124368	-0.216073091	-0.331785573	-0.195609352	downregulated during regeneration - mid
ENSDART00000066259	kcnk1a	0.055503316	-0.289802216	-0.227947241	-0.67177	-0.392395516	downregulated during regeneration - mid
ENSDART00000066380	ca7	0.023602236	-0.236035328	-0.017625204	-0.765902599	-0.026455411	downregulated during regeneration - mid
ENSDART00000066382	aqp8a.1	0.025161694	-0.164652843	-0.325293072	-0.493634593	-0.109118528	downregulated during regeneration - mid
ENSDART00000066655	mybl1	0.005666275	-0.255810306	-0.487313899	-0.72749049	-0.668391364	downregulated during regeneration - mid
ENSDART00000066963	atp6v1f	0.047327566	-0.089791813	-0.169571399	-0.304106913	-0.234245815	downregulated during regeneration - mid
ENSDART00000067005	bcat1	0.046674561	-0.064708558	-0.22197643	-0.301840187	-0.097404482	downregulated during regeneration - mid
ENSDART00000067078	plekhg5a	0.081075709	-0.147306721	-0.274518319	-0.555343847	-0.255866929	downregulated during regeneration - mid
ENSDART00000067448	acat1	0.021507957	-0.217922513	-0.251881532	-0.396031362	-0.209577052	downregulated during regeneration - mid
ENSDART00000067510	crabp1a	0.029243935	-0.186487239	-0.41656224	-0.606281006	-0.420918895	downregulated during regeneration - mid
ENSDART00000067764	stk17a	0.02229764	-0.185636748	-0.198053553	-0.515379898	-0.253038095	downregulated during regeneration - mid
ENSDART00000073694	smu1b	0.006932921	-0.023572641	-0.696487436	-0.934479138	-0.393819626	downregulated during regeneration - mid
ENSDART00000073932	NA	0.003072278	0.121194935	-0.376988949	-0.788016576	-0.265683315	downregulated during regeneration - mid
ENSDART00000074117	aspa	0.007828891	-0.032383232	-0.304124913	-0.397629458	-0.358898864	downregulated during regeneration - mid
ENSDART00000075070	hsf2	0.014034602	-0.066635095	-0.276496416	-0.474097609	-0.186498896	downregulated during regeneration - mid
ENSDART00000075421	sord	0.017496481	-0.008165085	-0.251228216	-0.437538687	-0.226988953	downregulated during regeneration - mid
ENSDART00000075513	aqp9b	0.023071688	-0.181865878	-0.358464178	-0.54013493	-0.322928595	downregulated during regeneration - mid
ENSDART00000076929	prkg2	0.055091796	-0.322610383	-0.221985796	-0.523406711	-0.304941411	downregulated during regeneration - mid
ENSDART00000077619	b3gat1b	0.017905694	-0.266663197	-0.223974588	-0.393683459	-0.125347427	downregulated during regeneration - mid
ENSDART00000077724	gnb5b	0.032645202	-0.216593155	-0.279473354	-0.485943487	-0.092161444	downregulated during regeneration - mid
ENSDART00000077839	atf7b	0.014656899	-0.232485852	-0.196508033	-0.391624214	-0.206346003	downregulated during regeneration - mid
ENSDART00000078014	poldip2	0.028918785	0.012504595	-0.191194187	-0.45353852	-0.221096745	downregulated during regeneration - mid
ENSDART00000078226	mtnr1bb	0.021380755	-0.285812097	-0.289316068	-0.599481549	-0.276891324	downregulated during regeneration - mid
ENSDART00000078311	zgc:154093	0.050880744	-0.170397489	-0.310689773	-0.421136702	-0.191616401	downregulated during regeneration - mid
ENSDART00000078336	klc3	0.011790336	-0.15725421	-0.267140148	-0.672772286	-0.331888789	downregulated during regeneration - mid
ENSDART00000078838	rab3aa	0.031838485	-0.225658115	-0.303506805	-0.374028659	-0.138563861	downregulated during regeneration - mid
ENSDART00000078908	usp1	0.022814815	-0.163356677	-0.229514103	-0.329514373	-0.251997637	downregulated during regeneration - mid
ENSDART00000079104	ndufs6	0.003180081	-0.146008815	-0.377028896	-0.443194118	-0.327081615	downregulated during regeneration - mid
ENSDART00000079629	ppm1nb	0.004971122	-0.279926594	-0.345062767	-0.462173038	-0.171806172	downregulated during regeneration - mid
ENSDART00000079686	zmp:0000001103	0.006141198	0.143973249	-0.889323937	-0.759747318	-0.487193938	downregulated during regeneration - mid
ENSDART00000079810	si:dkey-222b8.4	0.001538399	-0.076664011	-0.231174428	-0.530241167	-0.088965105	downregulated during regeneration - mid
ENSDART00000079866	slc30a9	0.045584509	-0.061810697	-0.211371943	-0.307919743	-0.172076689	downregulated during regeneration - mid
ENSDART00000080389	fam13a	0.004095858	0.342651645	-0.065971229	-0.119440295	0.116639155	downregulated during regeneration - mid
ENSDART00000081214	selt1a	0.041966249	-0.31898529	-0.284714031	-0.513594635	-0.412445291	downregulated during regeneration - mid
ENSDART00000081325	dynll1	0.0066372	-0.057380796	-0.1118203	-0.516198015	-0.112248637	downregulated during regeneration - mid
ENSDART00000081611	cgnb	0.001086517	-0.200219306	-0.438466186	-1.067503957	-0.17391972	downregulated during regeneration - mid
ENSDART00000081761	bin1b	0.028778753	-0.023096846	-0.153451242	-0.459181763	-0.132136814	downregulated during regeneration - mid
ENSDART00000081781	PLEKHG3	0.034279564	-0.076813883	-0.173391418	-0.382548311	-0.034437681	downregulated during regeneration - mid
ENSDART00000082066	atpv0e2	0.002085662	-0.268294846	-0.377237748	-0.604078331	-0.39998077	downregulated during regeneration - mid
ENSDART00000082523	impa2	0.00795396	-0.208830066	-0.398914852	-0.498863304	-0.267214166	downregulated during regeneration - mid
ENSDART00000082622	fsd1l	0.03087552	-0.046506283	-0.340407639	-0.41095417	-0.201162325	downregulated during regeneration - mid
ENSDART00000082937	fscn2a	0.054349324	-0.244556215	-0.436229092	-0.571250568	-0.199107259	downregulated during regeneration - mid
ENSDART00000084007	TULP2	0.035633594	-0.184892554	-0.234742374	-0.492430755	-0.167640019	downregulated during regeneration - mid
ENSDART00000084011	cplx4a	0.024776472	-0.220118176	-0.343953082	-0.589002173	-0.338126511	downregulated during regeneration - mid
ENSDART00000084135	ubtd1a	0.002016009	-0.22864625	-0.383233305	-0.947657938	-0.688823144	downregulated during regeneration - mid
ENSDART00000084353	tbc1d10ab	0.051425296	-0.174770874	-0.349891521	-0.619429045	-0.244339697	downregulated during regeneration - mid

ENSDART00000084417	timm17b	0.014386448	-0.134748392	-0.15578708	-0.404232794	-0.18557959	downregulated during regeneration - mid
ENSDART00000084792	prosc	0.057325828	-0.109541047	-0.121094609	-0.31852643	-0.202300262	downregulated during regeneration - mid
ENSDART00000084965	cep104	0.022501885	-0.196545786	-0.225873142	-0.456327943	-0.208454995	downregulated during regeneration - mid
ENSDART00000085252	pqlc3	0.004793697	-0.117366098	-0.27675705	-0.43476147	-0.253093536	downregulated during regeneration - mid
ENSDART00000085277	pfkmb	0.010085498	0.010653815	-0.414332737	-0.69351419	-0.144457554	downregulated during regeneration - mid
ENSDART00000085693	gpm6bb	0.009450076	-0.236628216	-0.325741908	-0.400571921	-0.218025638	downregulated during regeneration - mid
ENSDART00000085716	mtmr10	0.000364305	0.168573884	-0.120117023	-0.772748173	-0.357648365	downregulated during regeneration - mid
ENSDART00000086263	mettl7a	0.05857751	-0.420009286	-0.158826062	-0.791334734	-0.334080113	downregulated during regeneration - mid
ENSDART00000086619	prkca	0.027625402	-0.184045571	-0.418285198	-0.5357326	-0.310736719	downregulated during regeneration - mid
ENSDART00000086753	dapk2a	0.010335438	0.129419785	-0.339935803	-1.219284243	-0.369104907	downregulated during regeneration - mid
ENSDART00000086994	nat15	0.031838485	-0.144704703	-0.149678764	-0.337458722	-0.152039739	downregulated during regeneration - mid
ENSDART00000087070	abcc5	0.0132651	0.063506972	-0.209331316	-0.331966374	-0.070006315	downregulated during regeneration - mid
ENSDART00000087300	gabrb3	0.027218185	-0.106002759	-0.319333142	-0.574890474	-0.11105969	downregulated during regeneration - mid
ENSDART00000087570	BRSK2 (1 of many)	0.039389579	-0.035673495	-0.289130617	-0.495870531	-0.115059951	downregulated during regeneration - mid
ENSDART00000087643	tesk2	0.017205213	-0.250604524	-0.077411225	-0.349635246	-0.097357509	downregulated during regeneration - mid
ENSDART00000088027	ssx2ipb	0.061068477	-0.254273665	-0.300537366	-0.659689526	-0.365610708	downregulated during regeneration - mid
ENSDART00000089042	kcnh4b	0.028142339	-0.293363138	-0.305961812	-0.46205676	-0.207459851	downregulated during regeneration - mid
ENSDART00000089161	CCDC181	0.032175544	-0.238708752	-0.276570098	-0.364394143	-0.212879757	downregulated during regeneration - mid
ENSDART00000089526	otc	0.028981739	-0.228950714	-0.50691522	-0.941930968	-0.587900491	downregulated during regeneration - mid
ENSDART00000090079	synm	0.001644836	-0.113622285	-0.399027729	-0.70355851	-0.251171145	downregulated during regeneration - mid
ENSDART00000090484	tecpr1a	0.027062614	-0.079583722	-0.244227266	-0.30265493	-0.135390116	downregulated during regeneration - mid
ENSDART00000090611	sh3gl2a	0.003738261	-0.3620314	-0.426828792	-0.627861098	-0.256916064	downregulated during regeneration - mid
ENSDART00000091183	erfl3	0.034693937	-0.064022984	-0.222171088	-0.380622737	-0.084018803	downregulated during regeneration - mid
ENSDART00000091452	TULP2	0.003694199	-0.183522253	-0.233522919	-0.588056807	-0.14814607	downregulated during regeneration - mid
ENSDART00000091615	iffo1a	0.045036938	-0.255558776	-0.319129649	-0.402938235	-0.1114809	downregulated during regeneration - mid
ENSDART00000091620	atp8a1	0.025084869	-0.02813322	-0.262306637	-0.342910163	-0.163047911	downregulated during regeneration - mid
ENSDART00000091707	dbpa	0.017575469	-0.158262702	-0.327851995	-0.357551076	-0.129469939	downregulated during regeneration - mid
ENSDART00000091729	mlc1	0.000239262	-0.413445193	-0.441446959	-0.597560289	-0.400243567	downregulated during regeneration - mid
ENSDART00000092114	ERBB4 (1 of many)	0.013394015	-0.124937285	-0.553537291	-0.521885412	-0.338309306	downregulated during regeneration - mid
ENSDART00000092416	rabl2	0.018433591	-0.237808365	-0.250658742	-0.360133928	-0.144079351	downregulated during regeneration - mid
ENSDART00000093093	coro2bb	0.051450895	-0.315281988	-0.251087256	-0.471013192	-0.151819548	downregulated during regeneration - mid
ENSDART00000093331	rreb1a	0.018126285	-0.024901133	-0.155149005	-0.517234878	-0.050769944	downregulated during regeneration - mid
ENSDART00000097338	napaa	0.005374512	-0.21867082	-0.198889828	-0.441263528	-0.268359175	downregulated during regeneration - mid
ENSDART00000097466	fam169aa	0.080339729	-0.25560307	-0.389662586	-0.539552997	-0.303655	downregulated during regeneration - mid
ENSDART00000098263	kctd9a	0.018790846	-0.029411295	-0.237424393	-0.359070767	-0.190010537	downregulated during regeneration - mid
ENSDART00000098648	gc2	0.024271936	-0.195652835	-0.378422178	-0.402398473	-0.153861921	downregulated during regeneration - mid
ENSDART00000099180	elovl8a	0.019558454	-0.351208319	-0.254455279	-0.702580214	-0.196165774	downregulated during regeneration - mid
ENSDART00000099244	CDHR2	0.017384242	-0.127835174	-0.277679177	-0.519160965	-0.069385603	downregulated during regeneration - mid
ENSDART00000099392	irgq2	0.055536456	-0.080759981	-0.21229475	-0.382096269	-0.042741954	downregulated during regeneration - mid
ENSDART00000100131	si:ch211-242e8.1	0.00099671	0.043404066	-0.450710823	-0.466087638	0.073034701	downregulated during regeneration - mid
ENSDART00000100194	msi2b	0.036147745	-0.174290948	-0.286012891	-0.448717518	-0.171818835	downregulated during regeneration - mid
ENSDART00000100287	grk7a	0.01613952	-0.29429259	-0.34179807	-0.766271921	-0.26689268	downregulated during regeneration - mid
ENSDART00000100667	skia	0.021156666	-0.180939747	-0.108825609	-0.328725059	-0.076853639	downregulated during regeneration - mid
ENSDART00000100762	inpp4ab	0.000398071	-0.155677368	-0.41981961	-0.539565797	-0.358079101	downregulated during regeneration - mid
ENSDART00000100869	ppp3r1b	0.04032679	-0.102347685	-0.214447853	-0.31663745	-0.211814655	downregulated during regeneration - mid
ENSDART00000100885	nrn1la	0.001326538	-0.520024314	-0.30927413	-0.690569972	-0.461401545	downregulated during regeneration - mid
ENSDART00000101576	tmem230b	0.009263143	0.001436175	-0.176978383	-0.445323711	-0.253116022	downregulated during regeneration - mid
ENSDART00000102898	zgc:158258	0.013286981	-0.205000336	-0.263168834	-0.54350319	-0.257007642	downregulated during regeneration - mid
ENSDART00000102969	spock3	0.026589151	-0.0552616	-0.214726057	-0.592493725	-0.285702337	downregulated during regeneration - mid
ENSDART00000103070	cdk17	0.006385907	0.018864851	-0.23502559	-0.366641807	-0.137520544	downregulated during regeneration - mid
ENSDART00000103267	fam212ab	0.059492509	-0.276107898	-0.473876533	-0.614056911	-0.421775209	downregulated during regeneration - mid
ENSDART00000103293	ndufa5	0.014686352	-0.136898371	-0.38263765	-0.527827076	-0.351287298	downregulated during regeneration - mid
ENSDART00000103450	lactbl1b	0.002609113	-0.310148031	-0.346227281	-0.611014657	-0.245438949	downregulated during regeneration - mid
ENSDART00000103487	zgc:195001	0.006385907	-0.335765581	-0.344851478	-0.707965666	-0.31393888	downregulated during regeneration - mid
ENSDART00000103628	btbd6a	0.003515537	-0.278201553	-0.439382236	-0.547351526	-0.157769223	downregulated during regeneration - mid
ENSDART00000104234	gjd2b	0.009000055	-0.226334837	-0.451484904	-0.506629051	-0.204050045	downregulated during regeneration - mid
ENSDART00000104279	znf516	0.018737197	-0.151066862	-0.30868422	-0.587761132	-0.246031248	downregulated during regeneration - mid
ENSDART00000104298	ndufb5	0.062748344	-0.141095269	-0.212773777	-0.353488244	-0.122828248	downregulated during regeneration - mid
ENSDART00000104307	eml1	0.005712911	0.215532654	-0.343903678	-0.604837308	-0.271121591	downregulated during regeneration - mid
ENSDART00000104487	cox4i2	0.004567663	0.027176247	-0.218042175	-0.688187338	-0.440124172	downregulated during regeneration - mid
ENSDART00000104536	chsy1	0.002085662	0.021777196	-0.175630111	-0.644756219	-0.079001152	downregulated during regeneration - mid
ENSDART00000104674	camk1db	0.011089782	-0.318172003	-0.339895825	-0.49572727	-0.229789777	downregulated during regeneration - mid
ENSDART00000104708	abhd12	0.030797346	-0.134054463	-0.304895231	-0.501902497	-0.262631127	downregulated during regeneration - mid
ENSDART00000105485	si:dkey-78l4.14	0.009178848	-0.103336903	-0.24562657	-0.381336593	-0.194197896	downregulated during regeneration - mid
ENSDART00000105545	arl3	0.035698906	-0.028403306	-0.158533169	-0.38830015	-0.193573844	downregulated during regeneration - mid
ENSDART00000105561	si:dkey-42p8.3	0.005648279	-0.126414242	-0.359710173	-0.388006213	-0.105772793	downregulated during regeneration - mid
ENSDART00000105602	elovl6	0.064667433	-0.283618771	-0.171237628	-0.388002017	-0.174233768	downregulated during regeneration - mid
ENSDART00000105667	si:dkey-121a11.3	0.07695913	-0.369671493	-0.58967553	-0.842073302	-0.52980672	downregulated during regeneration - mid
ENSDART00000105681	cdc14ab	0.008133889	-0.104501589	-0.239167071	-0.39502699	-0.259112662	downregulated during regeneration - mid
ENSDART00000105974	pvalb9	0.005388807	-0.398960737	-0.428084025	-0.618672138	-0.278532192	downregulated during regeneration - mid
ENSDART00000108943	shroom2a	0.0263983	0.03299057	-0.127271939	-0.499446332	-0.229050052	downregulated during regeneration - mid
ENSDART00000108989	adamts14	0.018280387	-0.185318243	-0.40325002	-0.417840329	-0.182643804	downregulated during regeneration - mid
ENSDART00000109023	fmnl3	0.0083599	-0.186687955	-0.312847961	-0.533074314	-0.280720309	downregulated during regeneration - mid
ENSDART00000109037	cdk5r2b	0.061835261	-0.48847507	-0.44246865	-0.592226601	-0.418268347	downregulated during regeneration - mid
ENSDART00000109314	arhgap10	0.008289796	-0.214061464	-0.171864796	-0.398074347	-0.037252057	downregulated during regeneration - mid

ENSDART00000109440	adamts9	0.042577687	-0.162680626	-0.21784206	-0.326391297	-0.145144869	downregulated during regeneration - mid
ENSDART00000109485	gal3st3	0.008289796	-0.139388785	-0.282505485	-0.388230748	-0.163229068	downregulated during regeneration - mid
ENSDART00000109567	nhsa	0.005353377	-0.05015222	-0.331046598	-0.348641258	-0.190382203	downregulated during regeneration - mid
ENSDART00000109570	NA	0.009680262	-0.315520739	-0.355339215	-1.137398742	-0.355379016	downregulated during regeneration - mid
ENSDART00000109604	palmdb	0.002896578	-0.228743892	-0.477529612	-0.779896236	-0.307691368	downregulated during regeneration - mid
ENSDART00000109876	sun1	0.020586406	-0.255190881	-0.339472925	-0.466317308	-0.169389668	downregulated during regeneration - mid
ENSDART00000110033	abhd8b	0.063929589	-0.069420963	-0.144372686	-0.35601389	-0.098305508	downregulated during regeneration - mid
ENSDART00000110041	lrrc38a	0.006744186	-0.167375954	-0.314913504	-0.608219505	-0.293673506	downregulated during regeneration - mid
ENSDART00000110431	si:ch73-280o22.2	0.030699866	-0.128270202	-0.267395445	-0.366965125	-0.13375727	downregulated during regeneration - mid
ENSDART00000110571	fam150a	0.015571917	-0.089437558	-0.463371816	-0.643350488	-0.328581938	downregulated during regeneration - mid
ENSDART00000110606	abhd16a	0.001679054	0.034068276	-0.290276411	-0.385494504	-0.249441823	downregulated during regeneration - mid
ENSDART00000111021	si:dkeyp-14d3.1	0.005053949	-0.147889699	-0.589075251	-0.655456899	-0.257575305	downregulated during regeneration - mid
ENSDART00000111271	CABZ01080074.2	0.007536007	0.091182245	-0.411344825	-0.551440399	-0.297376381	downregulated during regeneration - mid
ENSDART00000111343	lmf2a	0.022218278	-0.264313993	-0.256081583	-0.425329386	-0.228346273	downregulated during regeneration - mid
ENSDART00000111536	rmdn2	0.042891	-0.153020266	-0.402861584	-0.346146607	-0.222410217	downregulated during regeneration - mid
ENSDART00000111625	ninl	0.004129329	-0.236310585	-0.326810382	-0.437556672	-0.154099243	downregulated during regeneration - mid
ENSDART00000111666	npdc1a	0.037722848	-0.090316422	-0.266587539	-0.30135563	-0.100212297	downregulated during regeneration - mid
ENSDART00000111717	fscn2b	0.015420308	-0.120313044	-0.274854028	-0.483029563	-0.127989104	downregulated during regeneration - mid
ENSDART00000111993	si:dkey-19b23.15	0.001555418	-0.211537347	-0.357802737	-0.511679441	-0.364770311	downregulated during regeneration - mid
ENSDART00000112156	si:ch211-253b8.5	0.014985328	-0.087783514	-0.192500909	-0.49330541	-0.306875488	downregulated during regeneration - mid
ENSDART00000112768	ubald1a	0.0312149	-0.163342676	-0.260286427	-0.366999537	-0.147535942	downregulated during regeneration - mid
ENSDART00000112967	rilp	0.001611738	-0.073950343	-0.174960248	-0.511756745	-0.145692497	downregulated during regeneration - mid
ENSDART00000113089	slc4a11	0.060667717	-0.230777933	-0.44932083	-0.596234565	-0.271629672	downregulated during regeneration - mid
ENSDART00000113362	ip6k1	0.017686321	-0.059673843	-0.326658711	-0.456047384	-0.047258149	downregulated during regeneration - mid
ENSDART00000113376	fam13b	0.00601663	-0.247096949	-0.341873515	-0.575011995	-0.179280023	downregulated during regeneration - mid
ENSDART00000113384	lyrm4	0.026927975	-0.082514668	-0.167513155	-0.434528553	-0.231621665	downregulated during regeneration - mid
ENSDART00000113649	impg1a	0.03238504	-0.10562067	-0.311339049	-0.59002435	-0.135488682	downregulated during regeneration - mid
ENSDART00000113799	si:ch211-132b12.7	0.001099064	0.091573373	-0.269264305	-0.847675231	-0.371595689	downregulated during regeneration - mid
ENSDART00000113853	prokr1a	0.062959243	-0.220461946	-0.109814734	-0.599222392	-0.17974305	downregulated during regeneration - mid
ENSDART00000113912	ntm	0.012936971	-0.178976918	-0.363265579	-0.513893339	-0.211182567	downregulated during regeneration - mid
ENSDART00000114118	CU639468.1	0.004971122	-0.073130118	-0.36036163	-0.387238652	-0.163804242	downregulated during regeneration - mid
ENSDART00000114134	wrb	0.049748908	-0.123951898	-0.095408141	-0.313002762	-0.1622087	downregulated during regeneration - mid
ENSDART00000114172	pde6d	0.006969648	-0.201449776	-0.351770435	-0.511138243	-0.216254622	downregulated during regeneration - mid
ENSDART00000114182	fyco1b	0.017131781	0.10137681	-0.156409007	-0.465781329	-0.01243848	downregulated during regeneration - mid
ENSDART00000114533	mn1a	0.064969544	-0.109536494	-0.207833595	-0.685216226	-0.20481073	downregulated during regeneration - mid
ENSDART00000114705	gprc5bb	0.016311478	-0.132340316	-0.338695197	-0.459834622	-0.091865364	downregulated during regeneration - mid
ENSDART00000114719	frmpd1a	0.0134351	-0.29565145	-0.338039747	-0.498795038	-0.059125706	downregulated during regeneration - mid
ENSDART00000114746	lrrc58a	0.018924903	-0.133537399	0.014548786	-0.622798481	-0.259773873	downregulated during regeneration - mid
ENSDART00000115221	adat2	0.007676315	0.066031062	-1.495132242	-2.125701316	-0.144340549	downregulated during regeneration - mid
ENSDART00000115224	pvrl1a	0.012357429	-0.001527	-0.239159739	-0.512986688	-0.121351808	downregulated during regeneration - mid
ENSDART00000115343	atp8b4	0.007152296	-0.126263785	-0.363733117	-0.385610912	-0.00148052	downregulated during regeneration - mid
ENSDART00000115370	mettl22	0.044172739	-0.250044414	-0.320129908	-0.572576042	-0.437533988	downregulated during regeneration - mid
ENSDART00000121545	brms1	0.048520479	-0.198338884	-0.120854433	-0.325603911	-0.103470514	downregulated during regeneration - mid
ENSDART00000121598	phf10	0.052326315	-0.237213765	-0.309795055	-0.326418269	-0.213703564	downregulated during regeneration - mid
ENSDART00000121952	h2afy2	0.039334382	-0.217094069	-0.401231455	-0.429907398	-0.128476741	downregulated during regeneration - mid
ENSDART00000122059	scoca	0.051450895	-0.009226259	-0.04540809	-0.349436614	-0.115738429	downregulated during regeneration - mid
ENSDART00000122716	PDE1C	0.014686352	-0.224024171	-0.483764938	-0.500061121	-0.40054702	downregulated during regeneration - mid
ENSDART00000122929	bbs4	0.023936488	-0.14534671	-0.308002271	-0.496501405	-0.252523436	downregulated during regeneration - mid
ENSDART00000123040	pfkpb	0.018655612	-0.26169445	-0.241526904	-0.415842652	-0.206785479	downregulated during regeneration - mid
ENSDART00000123360	susd5	0.005867979	-0.191753051	-0.73208098	-0.853142314	-0.526763596	downregulated during regeneration - mid
ENSDART00000123450	KCNB2 (1 of many)	0.014634638	-0.271043423	-0.632402343	-0.828557597	-0.168958818	downregulated during regeneration - mid
ENSDART00000123505	rab11fip4a	0.004897476	-0.154164113	-0.404988879	-0.479622862	-0.179766383	downregulated during regeneration - mid
ENSDART00000123820	tmem135	0.049596963	-0.104475369	-0.143585965	-0.370543783	-0.122253976	downregulated during regeneration - mid
ENSDART00000123970	mntb	0.011725523	-0.115861458	-0.101461538	-0.360694649	-0.01567692	downregulated during regeneration - mid
ENSDART00000124140	zgc:101566	0.000727221	-0.242404826	-0.290163127	-0.636219456	-0.416307006	downregulated during regeneration - mid
ENSDART00000124290	MFN1	0.012467303	-0.108610875	-0.185612855	-0.471855683	-0.083062873	downregulated during regeneration - mid
ENSDART00000124329	cbx8a	0.01903005	-0.13552578	-0.23364206	-0.31740894	-0.19855692	downregulated during regeneration - mid
ENSDART00000124335	smim4	0.017820373	-0.115055175	-0.371982424	-0.338416379	-0.248500803	downregulated during regeneration - mid
ENSDART00000124562	zgc:152977	0.011844097	-0.424781995	-0.464146326	-0.707134675	-0.367623104	downregulated during regeneration - mid
ENSDART00000124676	sv2ba	0.018790846	-0.120324523	-0.401787287	-0.561843381	-0.191682717	downregulated during regeneration - mid
ENSDART00000124945	ubap1lb	0.007333969	0.21207026	-0.004652141	-0.773302877	-0.344881822	downregulated during regeneration - mid
ENSDART00000125299	plk2a	0.086996216	-0.143584568	-0.170521478	-0.297471862	-0.183865098	downregulated during regeneration - mid
ENSDART00000125397	kri1	0.069160004	-0.318299605	-0.278223015	-0.439932558	-0.332258967	downregulated during regeneration - mid
ENSDART00000125466	alpi.2	0.052273162	-0.05537717	-0.356245119	-0.658920603	-0.289506352	downregulated during regeneration - mid
ENSDART00000125561	atp6ap2	0.008210772	-0.203601896	-0.343976801	-0.371684174	-0.265876436	downregulated during regeneration - mid
ENSDART00000125864	camkk1b	0.039389579	-0.169636507	-0.512096721	-0.591294824	-0.14296099	downregulated during regeneration - mid
ENSDART00000125979	tshz3a	0.02052819	-0.321412605	-0.323547538	-0.394821529	-0.291421679	downregulated during regeneration - mid
ENSDART00000126076	NA	0.012675019	-0.016912473	-0.350302068	-0.401962349	-0.001679825	downregulated during regeneration - mid
ENSDART00000126084	ctdspl3	0.009272964	-0.310396194	-0.278185547	-0.408791973	-0.234117517	downregulated during regeneration - mid
ENSDART00000126282	nr1d1	0.008088213	-0.1014969	-0.191656676	-0.494430716	-0.13667305	downregulated during regeneration - mid
ENSDART00000126661	rhobtb1	0.007785924	-0.165202139	-0.331565823	-0.522361525	-0.299461153	downregulated during regeneration - mid
ENSDART00000126842	flj11011l	0.051190638	-0.120546184	-0.161649138	-0.295902143	-0.182318736	downregulated during regeneration - mid
ENSDART00000126916	mob2b	0.004538993	0.26836075	-0.004670217	-0.398977994	-0.072830738	downregulated during regeneration - mid
ENSDART00000126928	si:dkey-73n8.3	0.012465298	-0.354837143	0.244926012	-3.843926683	-0.176933271	downregulated during regeneration - mid
ENSDART00000127131	mtmr10	0.016195506	0.05904867	-0.240178	-0.607174209	-0.120957306	downregulated during regeneration - mid

ENSDART00000127216	fam161a	0.056105466	-0.082566753	-0.243407902	-0.44855597	-0.155296925	downregulated during regeneration - mid
ENSDART00000127274	sema7a	0.056867894	-0.198678021	-0.353988759	-0.478218296	-0.427230313	downregulated during regeneration - mid
ENSDART00000127331	cdc37l1	0.022102317	-0.115815783	-0.102908381	-0.388723888	-0.245749713	downregulated during regeneration - mid
ENSDART00000127398	CR753886.1	0.031973637	-0.159141993	-0.332294072	-0.331878243	-0.244595408	downregulated during regeneration - mid
ENSDART00000127568	ankha	0.014386448	-0.068226585	-0.359480026	-0.686453521	-0.222767571	downregulated during regeneration - mid
ENSDART00000127656	cln6b	0.003337702	-0.363769389	-0.208045101	-0.481848131	-0.19203643	downregulated during regeneration - mid
ENSDART00000128696	CABZ01035108.1	0.038217845	-0.30766878	-0.382099096	-0.769557831	-0.298631085	downregulated during regeneration - mid
ENSDART00000128698	rnf34b	0.019215781	-0.122706526	-0.390373676	-0.658840165	-0.257572429	downregulated during regeneration - mid
ENSDART00000128721	rcvrn3	0.023143967	-0.045382046	-0.143627209	-0.579607896	-0.393091134	downregulated during regeneration - mid
ENSDART00000128722	RGS9BP	0.039271588	-0.328845343	-0.481472863	-0.560905879	-0.19840165	downregulated during regeneration - mid
ENSDART00000128859	prlra	0.029917459	-0.153277376	-0.232075961	-0.477332275	-0.11182176	downregulated during regeneration - mid
ENSDART00000128975	slc25a32a	0.003209414	0.048183005	-0.130483176	-0.493409396	-0.216291506	downregulated during regeneration - mid
ENSDART00000129156	adcy7	0.012672965	-0.081832737	-0.232314587	-0.506962474	-0.282923342	downregulated during regeneration - mid
ENSDART00000129308	ACVR1C	0.02188897	-0.083617591	-0.230914728	-0.630759756	-0.255735924	downregulated during regeneration - mid
ENSDART00000129751	si:ch211-105f12.2	0.063999698	-0.375204291	-0.254543259	-0.612224773	-0.289815804	downregulated during regeneration - mid
ENSDART00000130093	c10h21orf59	0.010893084	-0.163395921	-0.484168269	-0.87971069	-0.649928123	downregulated during regeneration - mid
ENSDART00000130142	ube2w	0.018144005	0.018587872	-0.041621436	-0.37919808	-0.07627037	downregulated during regeneration - mid
ENSDART00000130366	si:dkey-21c1.4	0.040026502	-0.116466944	-0.239339578	-0.392905857	-0.311667635	downregulated during regeneration - mid
ENSDART00000130537	spag1a	0.016180105	-0.063362594	-0.300759627	-0.420912866	-0.152669564	downregulated during regeneration - mid
ENSDART00000130818	ptprz1b	0.007396212	-0.275892935	-0.352115142	-0.541325565	-0.202924453	downregulated during regeneration - mid
ENSDART00000131206	43160	0.02794941	-0.087431315	-0.336892252	-0.48450384	-0.18585378	downregulated during regeneration - mid
ENSDART00000131286	erlin2	0.045086702	-0.000462383	-0.183644452	-0.434826094	-0.105981059	downregulated during regeneration - mid
ENSDART00000131349	mob2b	0.003887497	0.337219743	-0.092777728	-0.774689204	-0.397735432	downregulated during regeneration - mid
ENSDART00000131517	si:ch211-232m10.6	0.039816981	-0.306074293	-0.295884968	-0.441790198	-0.281067097	downregulated during regeneration - mid
ENSDART00000131627	kcnn3	0.030198325	-0.085165392	-0.23467025	-0.387311243	-0.108636046	downregulated during regeneration - mid
ENSDART00000131848	BX004774.2	0.004739684	0.065629354	-0.435237015	-1.040450944	-0.818745042	downregulated during regeneration - mid
ENSDART00000131973	lrmp	0.011245854	-0.070199137	-0.469517154	-1.098854311	-0.136597848	downregulated during regeneration - mid
ENSDART00000132591	si:ch211-191i18.4	0.016122246	0.099974449	-0.068457558	-0.479286849	-0.192940747	downregulated during regeneration - mid
ENSDART00000132744	ppcdc	0.004289396	0.019198695	-0.177358944	-0.471740583	-0.31681582	downregulated during regeneration - mid
ENSDART00000133035	syt5a	0.045873551	-0.188232763	-0.271065673	-0.434073976	-0.186984299	downregulated during regeneration - mid
ENSDART00000133039	tac3a	0.010923556	-0.2410584	-0.489343566	-0.63037654	-0.413025711	downregulated during regeneration - mid
ENSDART00000133220	CR788254.2	0.033633212	-0.013907422	-0.231082485	-0.739605077	-0.487191429	downregulated during regeneration - mid
ENSDART00000133242	syne2b	0.0092524	0.14436825	-0.456480635	-0.844135801	-0.206154738	downregulated during regeneration - mid
ENSDART00000133583	si:ch211-117m20.4	0.037722848	-0.073043862	-0.13648785	-0.437013406	-0.146443055	downregulated during regeneration - mid
ENSDART00000133613	AL954694.1	0.044914837	-0.189304139	-0.336191594	-0.52116431	-0.341767633	downregulated during regeneration - mid
ENSDART00000133661	tasp1	0.035967167	-0.129454637	-0.177592146	-0.299309238	-0.129141605	downregulated during regeneration - mid
ENSDART00000133867	arfip2a	0.037668797	-0.237325336	-0.320204428	-0.424850477	-0.153128941	downregulated during regeneration - mid
ENSDART00000133869	kcnk1a	0.066233336	-0.389144165	-0.38978007	-0.667699511	-0.286776021	downregulated during regeneration - mid
ENSDART00000134190	sort1b	0.011533823	-0.233086943	-0.116629656	-0.586941063	-0.191255985	downregulated during regeneration - mid
ENSDART00000134399	slkb	0.002060335	-0.338857379	-0.632619838	-0.770465733	-0.089077643	downregulated during regeneration - mid
ENSDART00000134697	pik3ip1	0.001209453	-0.252912627	-0.195771257	-0.573002676	-0.212029641	downregulated during regeneration - mid
ENSDART00000134773	zfand5a	0.01494686	-0.170709233	-0.221425066	-0.980962271	-0.517481995	downregulated during regeneration - mid
ENSDART00000134864	gigyf1b	0.0256797	-0.032906085	-0.141419247	-0.36943011	-0.170431533	downregulated during regeneration - mid
ENSDART00000135118	efr3ba	0.060997845	-0.168363836	-0.303586815	-0.356914459	-0.264543553	downregulated during regeneration - mid
ENSDART00000135374	ptpn21	0.062431525	-0.060719081	-0.163907746	-0.321158981	-0.096688483	downregulated during regeneration - mid
ENSDART00000135436	jtb	0.036479303	-0.083914092	-0.13827795	-0.410566521	-0.208466854	downregulated during regeneration - mid
ENSDART00000135479	fstl5	0.046057741	-0.187703631	-0.312045142	-0.413295205	-0.11029682	downregulated during regeneration - mid
ENSDART00000135510	syne2b	0.002913561	-0.011968335	-0.47360345	-0.918119469	-0.289310189	downregulated during regeneration - mid
ENSDART00000135690	lactbl1a	0.016724084	-0.207283355	-0.397072585	-0.513557165	-0.348206521	downregulated during regeneration - mid
ENSDART00000135738	zgc:194990	0.007620644	0.067005172	-0.387370648	-0.731552205	-0.192679748	downregulated during regeneration - mid
ENSDART00000135860	rnf41l	0.023081255	-0.353446179	-0.473576771	-0.616364395	-0.289592085	downregulated during regeneration - mid
ENSDART00000135919	amd1	0.013040781	-0.131714213	-0.276160556	-0.537901531	-0.163925689	downregulated during regeneration - mid
ENSDART00000136208	ncam1b	0.026842776	-0.292790209	-0.308372802	-0.421218735	-0.19466435	downregulated during regeneration - mid
ENSDART00000136647	ext1c	0.014963248	-0.283149695	-0.275883116	-0.465218527	-0.113484828	downregulated during regeneration - mid
ENSDART00000136770	si:dkey-246e1.3	0.017642395	-0.264481758	-0.308595158	-0.554881445	-0.406535962	downregulated during regeneration - mid
ENSDART00000136771	dnajc5ga	0.026478876	-0.246041931	-0.267357646	-0.352933326	-0.221144382	downregulated during regeneration - mid
ENSDART00000137047	scoca	0.043980855	-0.178388629	-0.216665672	-0.49140437	-0.218641566	downregulated during regeneration - mid
ENSDART00000137185	cnga3b	0.013354221	-0.145832564	-0.301563517	-1.136000666	-0.708190865	downregulated during regeneration - mid
ENSDART00000137236	hpcal1	0.014842483	-0.065446201	-0.487196869	-0.639863396	-0.431847778	downregulated during regeneration - mid
ENSDART00000137292	tmem106bb	0.004741095	-0.265825137	-0.29210537	-0.411370487	-0.25270276	downregulated during regeneration - mid
ENSDART00000137364	si:dkey-33c12.3	0.0001406	0.174067673	-0.534549963	-0.496919129	0.189169246	downregulated during regeneration - mid
ENSDART00000137633	si:ch211-194e15.5	0.009667225	-0.00297372	-0.215384681	-0.930894546	-0.593142383	downregulated during regeneration - mid
ENSDART00000137817	rap1gap2a	0.029185454	-0.351211604	-0.330279371	-0.569882036	-0.258477103	downregulated during regeneration - mid
ENSDART00000137896	fibpb	0.035286858	-0.042394017	-0.144008268	-0.458312875	-0.18906893	downregulated during regeneration - mid
ENSDART00000137899	tmem9	0.056822582	-0.365732748	-0.460492017	-0.592427521	-0.282793654	downregulated during regeneration - mid
ENSDART00000138232	arl13a	0.018790846	-0.123428499	-0.297389057	-0.827109894	-0.393133456	downregulated during regeneration - mid
ENSDART00000138270	copz2	0.022463985	-0.133761169	-0.127273242	-0.35316272	-0.169760508	downregulated during regeneration - mid
ENSDART00000138541	rpgrip1	0.043421442	-0.096214383	-0.271667846	-0.617065899	-0.286110235	downregulated during regeneration - mid
ENSDART00000138658	si:ch211-117m20.4	0.082561098	-0.301116828	-0.255854166	-0.65213458	-0.329202684	downregulated during regeneration - mid
ENSDART00000138793	si:ch211-203k16.3	0.033480582	-0.114803625	-0.341143223	-0.894722004	-0.064858026	downregulated during regeneration - mid
ENSDART00000138866	pbxip1a	0.000760227	-0.45481847	-0.358362517	-0.587330976	-0.331438505	downregulated during regeneration - mid
ENSDART00000138922	elovl8a	0.097148471	-0.299886352	-0.255106336	-0.59316691	-0.339691401	downregulated during regeneration - mid
ENSDART00000139042	si:ch211-132b12.7	0.002274284	0.072730857	-0.322841214	-0.765244522	-0.309126323	downregulated during regeneration - mid
ENSDART00000139102	dbpb	0.001993937	0.113164671	-0.364559303	-0.423196921	-0.137052116	downregulated during regeneration - mid
ENSDART00000139310	bcat2	0.032825581	-0.180902608	-0.445596686	-0.579108461	-0.343900677	downregulated during regeneration - mid

ENSDART00000139461	spock3	0.043085704	-0.122253969	-0.220033244	-0.389207603	-0.090203867	downregulated during regeneration - mid
ENSDART00000139477	trim110	0.030645263	-0.21821102	-0.289321848	-0.373728133	-0.159590708	downregulated during regeneration - mid
ENSDART00000139715	dpm3	0.028357685	-0.11194386	-0.125527265	-0.41579896	-0.188378465	downregulated during regeneration - mid
ENSDART00000139900	diaph3	0.028408144	0.052388836	-0.258521684	-0.529199016	-0.186043701	downregulated during regeneration - mid
ENSDART00000140145	ajap1	0.019585067	-0.237572949	-0.464416488	-0.62912768	-0.262821389	downregulated during regeneration - mid
ENSDART00000140375	zfyve27	0.02776376	-0.214217108	-0.191210439	-0.353015645	-0.21311838	downregulated during regeneration - mid
ENSDART00000140553	frmpd1a	0.012556905	-0.279082186	-0.31970424	-0.46726564	-0.019879501	downregulated during regeneration - mid
ENSDART00000141031	BX004774.2	0.016675166	-0.096197487	-0.455215086	-0.947274727	-0.721728098	downregulated during regeneration - mid
ENSDART00000141157	ablim3	0.029525765	-0.090063728	-0.09247473	-0.384522771	0.048083962	downregulated during regeneration - mid
ENSDART00000141340	si:dkey-183n20.15	0.02850112	-0.434296772	-0.549401302	-1.33032419	-0.748989345	downregulated during regeneration - mid
ENSDART00000141493	ANO2 (1 of many)	0.006900346	0.056182997	-0.29662332	-0.7863422	-0.307277237	downregulated during regeneration - mid
ENSDART00000141734	hivep2a	0.017814842	-0.218148846	-0.337091836	-0.481607769	-0.327040294	downregulated during regeneration - mid
ENSDART00000142157	arl15a	0.011135896	-0.037401347	-0.185890543	-0.49114445	-0.348659966	downregulated during regeneration - mid
ENSDART00000142454	si:dkey-121a11.3	0.046134939	-0.218109071	-0.329830559	-0.334040533	-0.225452629	downregulated during regeneration - mid
ENSDART00000142543	si:dkey-206f10.1	0.009178848	-0.205833854	-0.070068511	-0.826534181	-0.287245824	downregulated during regeneration - mid
ENSDART00000142605	sorbs2b	0.003262292	-0.090898219	-0.353328393	-0.576838002	-0.278787769	downregulated during regeneration - mid
ENSDART00000142731	rce1b	0.030361713	-0.103631801	-0.326325127	-0.391135117	-0.061782364	downregulated during regeneration - mid
ENSDART00000143165	tsc22d1	0.012984014	-0.098364585	-0.21923408	-0.39849137	0.017570634	downregulated during regeneration - mid
ENSDART00000143199	wasf3a	0.033862985	-0.216537889	-0.280788628	-0.398676762	-0.269991874	downregulated during regeneration - mid
ENSDART00000143245	nptnb	0.00231226	-0.0813348	-0.106325128	-0.477835951	-0.112902967	downregulated during regeneration - mid
ENSDART00000143503	usp21	0.010231208	-0.183997919	-0.332937745	-0.642449016	-0.356635014	downregulated during regeneration - mid
ENSDART00000143625	map7d2b	0.042433381	0.025364857	-1.606530561	-4.429840485	-0.88152256	downregulated during regeneration - mid
ENSDART00000143662	ankrd33aa	0.01899428	-0.118189532	-0.449119978	-0.599045615	0.004982045	downregulated during regeneration - mid
ENSDART00000143829	nxnl1	0.034874709	-0.184554354	-0.325799787	-0.486032926	-0.185522737	downregulated during regeneration - mid
ENSDART00000143874	akna	0.021937256	-0.124615674	-0.323808831	-0.482322372	-0.23639171	downregulated during regeneration - mid
ENSDART00000144592	sik2a	0.005427451	-0.05071398	-0.135507163	-0.424760133	0.101311014	downregulated during regeneration - mid
ENSDART00000144802	dfnb31b	0.012605558	-0.206535765	-0.346317212	-0.484783097	-0.018383317	downregulated during regeneration - mid
ENSDART00000144804	mxra7	0.020724346	-0.254910086	-0.321688262	-0.500299705	-0.323281006	downregulated during regeneration - mid
ENSDART00000144890	bokb	0.052287778	-0.312629523	-0.278040732	-0.730243904	-0.348692502	downregulated during regeneration - mid
ENSDART00000145035	saga	0.025257904	-0.094875139	-0.251791894	-0.561974573	-0.363496236	downregulated during regeneration - mid
ENSDART00000145258	glsb	0.012086831	-0.010345949	-0.147193332	-0.750348982	-0.227873062	downregulated during regeneration - mid
ENSDART00000145545	dclk1a	0.028108682	-0.21717792	-0.314020567	-0.407893866	-0.042242501	downregulated during regeneration - mid
ENSDART00000145728	sdhc	0.056867894	-0.035025769	-0.113602993	-0.325388824	-0.192371035	downregulated during regeneration - mid
ENSDART00000145834	si:ch211-233a24.2	0.017986602	-0.054480296	-0.065552868	-0.373189	-0.049333125	downregulated during regeneration - mid
ENSDART00000145894	zgc:195245	0.033020938	-0.325743953	-0.471221888	-0.661613866	-0.246097731	downregulated during regeneration - mid
ENSDART00000146008	camkvl	0.050814856	-0.251523364	-0.299024254	-0.462517021	-0.14005057	downregulated during regeneration - mid
ENSDART00000146962	STX3 (1 of many)	0.041930419	-0.081644241	-0.246049482	-0.328837917	-0.149895857	downregulated during regeneration - mid
ENSDART00000147368	osbp2	0.027388792	-0.137018463	-0.22987814	-0.404444797	-0.125629393	downregulated during regeneration - mid
ENSDART00000147422	arhgap23b	0.009929644	-0.106264079	-0.33507693	-0.670558797	-0.201207875	downregulated during regeneration - mid
ENSDART00000147502	grhprb	0.004106215	0.278026338	-0.217638113	-0.512645749	-0.092734666	downregulated during regeneration - mid
ENSDART00000147504	bzw2	0.002177213	0.031120244	-0.513458671	-0.643056049	-0.325765553	downregulated during regeneration - mid
ENSDART00000147884	syne2b	0.0066372	0.046598878	-0.495566729	-1.074400394	-0.335549303	downregulated during regeneration - mid
ENSDART00000147903	lrit1a	0.028192085	-0.179060864	-0.15522738	-0.479781004	-0.346169989	downregulated during regeneration - mid
ENSDART00000148039	snx19a	0.037712225	-0.086070054	-0.238060141	-0.431834798	-0.166151211	downregulated during regeneration - mid
ENSDART00000148066	znf395b	0.020342933	-0.110739145	-0.366906743	-0.460042969	-0.183105049	downregulated during regeneration - mid
ENSDART00000148106	mef2aa	0.026582721	-0.081330082	-0.55524128	-0.609035118	-0.185252531	downregulated during regeneration - mid
ENSDART00000148175	si:ch1073-440b2.1	0.004197991	-0.235967394	-0.243649374	-0.449710659	-0.316169237	downregulated during regeneration - mid
ENSDART00000148246	pole4	0.028322957	-0.080211224	-0.333184545	-0.567162517	-0.350297708	downregulated during regeneration - mid
ENSDART00000148353	usf2	0.006964046	-0.107987138	-0.265954127	-0.405201274	-0.200197809	downregulated during regeneration - mid
ENSDART00000148536	GK3P	0.017775184	-0.308304163	-0.269790809	-0.586409039	-0.39358576	downregulated during regeneration - mid
ENSDART00000148537	roraa	0.01612441	-0.160828233	-0.221872095	-0.45696721	-0.083100906	downregulated during regeneration - mid
ENSDART00000148639	ubxn2a	0.015578607	-0.292754646	-0.267419804	-0.368360861	-0.228619688	downregulated during regeneration - mid
ENSDART00000148831	fgf11b	0.048355827	-0.269342716	-0.341549392	-0.474527478	-0.280873475	downregulated during regeneration - mid
ENSDART00000148958	fam150a	0.006385907	-0.081185595	-0.392189222	-0.650641125	-0.329645149	downregulated during regeneration - mid
ENSDART00000148982	bin1b	0.017473198	0.009431427	-0.214307092	-0.49686004	-0.050411548	downregulated during regeneration - mid
ENSDART00000149029	sagb	0.030697622	-0.046274749	-0.449576755	-0.950069775	-0.043925247	downregulated during regeneration - mid
ENSDART00000149339	si:ch211-175f12.2	0.046869499	-0.297380973	-0.332932663	-0.389357991	-0.204952824	downregulated during regeneration - mid
ENSDART00000149352	si:ch211-163l21.11	0.045582714	-0.266781177	-0.351609906	-0.560319486	-0.211979525	downregulated during regeneration - mid
ENSDART00000149429	boka	0.007908312	-0.220682727	-0.305502243	-0.692638969	-0.393597854	downregulated during regeneration - mid
ENSDART00000149512	CU467655.1	0.058548676	-0.320227651	-0.180971804	-0.440965145	-0.207148298	downregulated during regeneration - mid
ENSDART00000149634	atp6ap2	0.075143936	-0.108949021	-0.143807338	-0.457436101	-0.199272375	downregulated during regeneration - mid
ENSDART00000149935	nt5c3a	0.022408352	0.05115392	-0.273408486	-0.497911915	-0.317644766	downregulated during regeneration - mid
ENSDART00000150045	adamts18	0.038201053	-0.187059316	-0.292648437	-0.628764119	0.030985495	downregulated during regeneration - mid
ENSDART00000150789	USMG5	0.058128769	-0.285153636	-0.258859464	-0.457141251	-0.093210696	downregulated during regeneration - mid
ENSDART00000151013	ensaa	0.001153184	-0.338295629	-0.414678183	-0.638369138	-0.341312384	downregulated during regeneration - mid
ENSDART00000151022	si:ch211-191i18.4	0.046753078	-0.318627462	-0.467051418	-0.723047751	-0.473341501	downregulated during regeneration - mid
ENSDART00000151203	rgs9a	0.049667416	-0.190499153	-0.256482975	-0.462262315	-0.086082277	downregulated during regeneration - mid
ENSDART00000151386	CU659670.1	0.044578949	-0.061309281	-0.197320939	-0.510180812	-0.129307388	downregulated during regeneration - mid
ENSDART00000151419	tgfa	0.049309412	-0.147139604	-0.1316737	-0.368157435	-0.244071655	downregulated during regeneration - mid
ENSDART00000151670	fbxl16	0.055597767	-0.100117774	-0.155204519	-0.627708744	-0.129502503	downregulated during regeneration - mid
ENSDART00000151808	rcvrn3	0.013770716	-0.028816037	-0.156203513	-0.580402071	-0.415774036	downregulated during regeneration - mid
ENSDART00000152005	ppp1r13ba	0.024276272	-0.032736325	-0.117362615	-0.454836475	-0.009028338	downregulated during regeneration - mid
ENSDART00000152172	BX248418.1	0.005026826	-0.267252802	-0.476860817	-0.659952516	-0.030823789	downregulated during regeneration - mid
ENSDART00000152689	oplah	0.01992766	-0.018001137	-0.164313071	-0.461321001	-0.221934453	downregulated during regeneration - mid
ENSDART00000152768	si:ch211-207i1.2	0.036445549	-0.201357893	-0.51029626	-0.665018804	-0.121583536	downregulated during regeneration - mid

ENSDART00000152968	si:dkey-21c1.1	0.005983812	0.134479312	-0.180316529	-0.580606373	-0.202289334	downregulated during regeneration - mid
ENSDART00000153086	kctd17	0.063822766	-0.109500243	-0.169719356	-0.312398256	-0.16098927	downregulated during regeneration - mid
ENSDART00000153124	CU570682.1	0.019075692	-0.274154453	-0.462025745	-0.49154679	-0.26836126	downregulated during regeneration - mid
ENSDART00000153190	vash2	0.019554708	-0.078136066	-0.226923996	-0.395295378	-0.160874636	downregulated during regeneration - mid
ENSDART00000153442	rxfp2a	0.01711161	-0.191591927	-0.398184153	-0.707111514	-0.312025768	downregulated during regeneration - mid
ENSDART00000153773	cipca	0.004341144	-0.044231438	-0.31334692	-0.44667945	-0.17971329	downregulated during regeneration - mid
ENSDART00000154085	gipr	0.032143116	-0.077819916	-0.236822644	-0.445528895	-0.377678023	downregulated during regeneration - mid
ENSDART00000154627	eogt	0.014130752	-0.136366673	-0.382886458	-0.560103122	-0.203176718	downregulated during regeneration - mid
ENSDART00000154917	ciartb	0.004147933	-0.07665894	-0.17887759	-0.516654864	-0.219843882	downregulated during regeneration - mid
ENSDART00000155078	CU466278.1	0.067207866	-0.496260032	-0.275828398	-0.937323619	-0.494833793	downregulated during regeneration - mid
ENSDART00000155088	ankrd33ab	0.019678793	-0.091123111	-0.280595773	-2.122583018	-0.908625438	downregulated during regeneration - mid
ENSDART00000155324	pdpk1a	0.051252746	-0.082713691	-0.135052421	-0.317218269	-0.180842854	downregulated during regeneration - mid
ENSDART00000155458	ubap1la	0.036933769	-0.102679722	-0.709773263	-0.512828077	-0.333708251	downregulated during regeneration - mid
ENSDART00000155580	ccdc32	0.028277545	-0.137851793	-0.182759184	-0.331717766	-0.143672128	downregulated during regeneration - mid
ENSDART00000155839	CR759918.1	0.036715052	-0.263859971	-0.262088445	-0.379170984	-0.118091172	downregulated during regeneration - mid
ENSDART00000155859	larp4ab	0.007335615	-0.193577441	-0.330790279	-0.534105504	-0.201385609	downregulated during regeneration - mid
ENSDART00000155956	ssx2ipa	0.040205407	-0.036908945	-0.252732501	-0.388773092	-0.156731785	downregulated during regeneration - mid
ENSDART00000156008	adcy1b	0.021755167	-0.126277791	-0.148680925	-0.963041725	-0.495906031	downregulated during regeneration - mid
ENSDART00000156051	dhx32b	0.010222513	-0.034036848	-0.386771049	-0.600165189	-0.131769991	downregulated during regeneration - mid
ENSDART00000156169	ube2o	0.055683104	-0.089939623	-0.17956897	-0.346977584	-0.218736801	downregulated during regeneration - mid
ENSDART00000156179	sema7a	0.031092135	-0.273960356	-0.559537035	-0.479585038	-0.478067901	downregulated during regeneration - mid
ENSDART00000156211	adcyap1r1b	0.029377262	-0.120918148	-0.186017	-0.540439336	-0.277725276	downregulated during regeneration - mid
ENSDART00000156247	CU468915.1	0.04446722	-0.147323171	-0.162953088	-0.662599642	-0.420048082	downregulated during regeneration - mid
ENSDART00000156365	BX248121.1	0.044459756	-0.069755444	-0.2828198	-0.35442143	-0.173325265	downregulated during regeneration - mid
ENSDART00000156438	tmem138	0.003538125	-0.131418974	-0.291816206	-0.603920899	-0.471900931	downregulated during regeneration - mid
ENSDART00000156492	tiam2a	0.033593902	-0.024981814	-0.236489206	-0.397107093	-0.120407246	downregulated during regeneration - mid
ENSDART00000156527	crhr1	0.045012134	-0.121975919	-0.246139891	-0.376184792	-0.120567191	downregulated during regeneration - mid
ENSDART00000156568	CR387996.1	0.023717938	-0.565799723	-0.534585499	-0.855400153	-0.72349809	downregulated during regeneration - mid
ENSDART00000156757	CU633486.1	0.075604115	-0.302968456	-0.206059	-0.531920994	-0.165463059	downregulated during regeneration - mid
ENSDART00000156831	si:dkeyp-47f9.4	0.018916133	-0.215799622	-0.290204625	-0.428110244	-0.249285093	downregulated during regeneration - mid
ENSDART00000156918	si:dkey-16p21.8	0.016886751	-0.278111877	-0.271868386	-0.501824009	-0.172182623	downregulated during regeneration - mid
ENSDART00000156967	itcha	0.025939466	-0.048958922	-0.233534262	-0.334119802	-0.057332431	downregulated during regeneration - mid
ENSDART00000157066	ppp1r16b	0.028214992	-0.047627869	-0.115324195	-0.408403631	-0.014573035	downregulated during regeneration - mid
ENSDART00000157437	park2	0.017079861	-0.135617767	-0.306295495	-0.445890787	-0.195890902	downregulated during regeneration - mid
ENSDART00000157515	FO704641.1	0.005096692	-0.135830256	-0.41280018	-0.451790561	-0.015210245	downregulated during regeneration - mid
ENSDART00000157570	asmt	0.002360359	-0.467907647	-0.512776687	-0.801096178	-0.257600065	downregulated during regeneration - mid
ENSDART00000157755	cds1	0.00817514	-0.167833858	-0.281185983	-0.514312915	-0.176231692	downregulated during regeneration - mid
ENSDART00000157979	plppr5b	0.003299709	-0.141986763	-0.474240998	-0.437275413	-0.217953336	downregulated during regeneration - mid
ENSDART00000158055	kcnh6a	0.064248443	-0.288242124	-0.323669014	-0.559331748	-0.259833446	downregulated during regeneration - mid
ENSDART00000158077	arr3b	0.063631477	-0.13602706	-0.148118881	-0.527427286	-0.215489656	downregulated during regeneration - mid
ENSDART00000158202	CABZ01078449.1	0.039683894	-0.388078647	-0.185889564	-2.923902226	0.109460724	downregulated during regeneration - mid
ENSDART00000158289	taok1b	0.039389579	-0.136932712	-0.168799291	-0.390684145	-0.122927658	downregulated during regeneration - mid
ENSDART00000158301	CR749168.4	0.010360832	-0.284512048	-0.514337485	-0.519239658	-0.283197555	downregulated during regeneration - mid
ENSDART00000158338	CNNM1	0.001487078	-0.362878072	-0.490740729	-0.646651234	-0.29213891	downregulated during regeneration - mid
ENSDART00000158395	dclk2a	0.01163651	-0.152411223	-0.291700953	-0.710292221	-0.218291454	downregulated during regeneration - mid
ENSDART00000158440	arhgap12b	0.041157695	-0.039244537	-0.138433678	-0.355110347	-0.091493676	downregulated during regeneration - mid
ENSDART00000158587	casd1	0.047492859	-0.21622109	-0.133405356	-0.572868553	-0.255662771	downregulated during regeneration - mid
ENSDART00000158692	CABZ01112401.1	0.000824931	0.113542035	-0.344191992	-0.461207823	-0.256682679	downregulated during regeneration - mid
ENSDART00000158854	tpd52l1	0.028449452	-0.063123021	-0.257733521	-0.65813611	-0.260747428	downregulated during regeneration - mid
ENSDART00000158917	abhd10a	0.008288008	-0.199951388	-0.281143798	-0.472212357	-0.246558895	downregulated during regeneration - mid
ENSDART00000158962	dpf2	0.022330586	0.023344289	-0.160855631	-0.651177712	-0.029134577	downregulated during regeneration - mid
ENSDART00000159061	si:ch211-185a18.2	0.068645885	-0.270017947	-0.418794122	-0.535787315	-0.21407671	downregulated during regeneration - mid
ENSDART00000159123	btbd17b	0.02583682	-0.172708316	-0.362184438	-0.675325658	-0.245697841	downregulated during regeneration - mid
ENSDART00000159178	hspa8	0.00434437	-1.810725395	-0.905134344	-6.819365873	-1.947204217	downregulated during regeneration - mid
ENSDART00000159581	CT583723.1	0.081780045	-0.203564175	-0.159816156	-0.458224986	-0.16588667	downregulated during regeneration - mid
ENSDART00000159654	CR847844.2	0.019450344	0.025394661	-0.340096605	-0.548288987	-0.346027376	downregulated during regeneration - mid
ENSDART00000159670	abtb1	0.013224152	-0.10462458	-0.155472174	-0.372376774	-0.233981977	downregulated during regeneration - mid
ENSDART00000159733	CABZ01063757.1	0.004296618	0.129910694	-0.203491407	-0.855433405	-0.227320323	downregulated during regeneration - mid
ENSDART00000159950	si:ch1073-450f2.1	0.040770952	-0.285454261	-0.252056938	-0.418320337	-0.16839092	downregulated during regeneration - mid
ENSDART00000159987	si:dkey-65b13.13	0.035889612	-0.209652039	-0.259703439	-0.637719895	-0.234430743	downregulated during regeneration - mid
ENSDART00000160115	per1a	0.005681638	0.041503641	-0.272737679	-0.476359218	-0.15653808	downregulated during regeneration - mid
ENSDART00000160134	hibadha	0.01949596	-0.191259291	-0.221221329	-0.647202373	-0.244609156	downregulated during regeneration - mid
ENSDART00000160175	SYNDIG1	0.040346146	-0.028604943	-0.23293463	-0.464850771	-0.153309965	downregulated during regeneration - mid
ENSDART00000160242	TYMP	0.002200182	-0.668893964	-0.374517624	-0.757876583	-0.434642219	downregulated during regeneration - mid
ENSDART00000160284	pclob	0.022160325	-0.164621492	-0.643566861	-0.569524507	-0.247739035	downregulated during regeneration - mid
ENSDART00000160400	gchfr	0.016130084	-0.373470826	-0.390793782	-0.620398999	-0.352985133	downregulated during regeneration - mid
ENSDART00000160439	CABZ01037298.1	0.009820514	-0.12483018	-0.201319697	-0.520978425	-0.240731645	downregulated during regeneration - mid
ENSDART00000160450	CU571069.1	0.000313682	-0.391785076	-0.855515025	-0.904047805	-0.612021184	downregulated during regeneration - mid
ENSDART00000160468	ubl3a	0.003138642	-0.157200321	-0.291255908	-0.684230474	-0.436379037	downregulated during regeneration - mid
ENSDART00000160495	si:dkey-184p18.2	0.011672204	0.380936601	-0.937356475	-0.364392869	-0.267264411	downregulated during regeneration - mid
ENSDART00000160538	arhgap21a	0.006013085	-0.416942655	-0.374699279	-1.045101409	-0.721453587	downregulated during regeneration - mid
ENSDART00000160562	impg2b	0.011993283	0.024461351	-0.26080833	-0.706384589	-0.340668028	downregulated during regeneration - mid
ENSDART00000160700	dnm1l	0.023931815	-0.370704077	-0.348232697	-0.495814147	-0.141373029	downregulated during regeneration - mid
ENSDART00000160742	clmn	0.002103359	-0.341601235	-0.332225059	-0.489517534	-0.288919784	downregulated during regeneration - mid
ENSDART00000160753	GANAB (1 of many)	0.023696289	0.004666803	-0.095016348	-0.32432452	-0.161685037	downregulated during regeneration - mid

ENSDART00000160841	si:ch211-248g20.5	0.022501885	-0.153486145	-0.342330243	-0.525669956	-0.178412263	downregulated during regeneration - mid
ENSDART00000160866	cry2	0.002361523	-0.114688269	-0.279009524	-0.604836383	-0.332518261	downregulated during regeneration - mid
ENSDART00000161059	tusc2a	0.013390976	0.068801861	-0.121312821	-0.423616648	-0.242714442	downregulated during regeneration - mid
ENSDART00000161261	lmbrd2a	0.083564138	-0.185598793	-0.153033552	-0.360733538	-0.113007589	downregulated during regeneration - mid
ENSDART00000161266	CU928046.1	0.025523567	-0.122276569	-0.195535625	-0.336584046	-0.188368371	downregulated during regeneration - mid
ENSDART00000161397	atg13	0.021797231	-0.137451879	-0.18705676	-0.405447689	-0.269252338	downregulated during regeneration - mid
ENSDART00000161440	limch1b	0.039972057	-0.008873535	-0.193340096	-0.430561872	-0.157445684	downregulated during regeneration - mid
ENSDART00000161652	si:dkeyp-72e1.9	0.040887007	-0.109528412	-0.231594686	-0.432617543	-0.203491956	downregulated during regeneration - mid
ENSDART00000161755	CU681842.1	0.03135437	-0.157328162	-0.245310878	-0.348302227	-0.161138988	downregulated during regeneration - mid
ENSDART00000161773	KCNJ11 (1 of many)	0.040962132	-0.422252277	-0.360338989	-0.607728772	-0.332578335	downregulated during regeneration - mid
ENSDART00000161823	ubl3a	0.020595702	-0.089124518	-0.239047703	-0.471864878	-0.290785384	downregulated during regeneration - mid
ENSDART00000161840	CT574575.1	0.006228736	-0.29915223	-0.372107241	-0.764083264	-0.340151107	downregulated during regeneration - mid
ENSDART00000161882	bzw2	0.010880494	0.083435949	-0.31200966	-0.5490146	-0.324311149	downregulated during regeneration - mid
ENSDART00000162070	tmem9	0.005875165	-0.208225931	-0.361514875	-0.511868133	-0.393713678	downregulated during regeneration - mid
ENSDART00000162493	kif5ab	0.000645779	-0.358862317	-0.407894033	-0.585721315	-0.34361757	downregulated during regeneration - mid
ENSDART00000162664	5S_rRNA	0.014771508	-0.438943374	-1.186726175	-0.920371372	-0.548334869	downregulated during regeneration - mid
ENSDART00000162669	slc4a5	0.010442784	-0.13383932	-0.22542524	-0.430682825	-0.287279235	downregulated during regeneration - mid
ENSDART00000162827	si:dkey-92j12.5	0.030149141	-0.013228451	-0.265522895	-0.385437174	-0.080127062	downregulated during regeneration - mid
ENSDART00000162858	lmbrd1	0.053770748	-0.243655467	-0.23291492	-0.332336473	-0.114530821	downregulated during regeneration - mid
ENSDART00000162875	rogdi	0.014149547	-0.224891588	-0.27706391	-0.36191309	-0.159630193	downregulated during regeneration - mid
ENSDART00000162940	mob2b	0.021735616	0.020755037	-0.2829937	-0.541864256	-0.204837959	downregulated during regeneration - mid
ENSDART00000163093	lrp12	0.015282229	-0.07747456	-0.233717712	-0.371900586	-0.129203418	downregulated during regeneration - mid
ENSDART00000163250	mef2cb	0.003671994	0.361224616	-0.300957895	-0.57227951	-0.080214844	downregulated during regeneration - mid
ENSDART00000163352	impg2b	0.015981762	-0.050961847	-0.309849144	-0.745225088	-0.302394708	downregulated during regeneration - mid
ENSDART00000163445	zgc:109982	0.01612441	0.178949347	-0.334063834	-0.837985378	-0.307869575	downregulated during regeneration - mid
ENSDART00000163521	si:ch1073-83n3.2	0.019433758	-0.135028227	-0.352956778	-0.389044273	-0.105210001	downregulated during regeneration - mid
ENSDART00000163908	rnasekb	0.014026672	-0.236636314	-0.265732137	-0.418465587	-0.246757364	downregulated during regeneration - mid
ENSDART00000163935	med30	0.026243123	-0.198525489	-0.227576976	-0.505162548	-0.348410732	downregulated during regeneration - mid
ENSDART00000163976	CABZ01069287.1	0.008492808	0.247964945	-0.272161916	-0.664208233	-0.362164102	downregulated during regeneration - mid
ENSDART00000163998	rps6ka3a	0.02187407	0.01131102	-0.11493059	-0.372925255	-0.200625555	downregulated during regeneration - mid
ENSDART00000164566	akt3a	0.001867076	0.045255643	-0.432188612	-0.498284714	-0.321504061	downregulated during regeneration - mid
ENSDART00000164650	pdk3b	0.093881144	-0.133813888	-0.219494437	-0.474289524	-0.225858079	downregulated during regeneration - mid
ENSDART00000164695	arl3l1	0.005956006	-0.208201897	-0.292220291	-0.610724074	-0.376046469	downregulated during regeneration - mid
ENSDART00000164773	CABZ01072036.1	0.021489826	-0.106672866	-0.494009284	-0.613761036	-0.476277849	downregulated during regeneration - mid
ENSDART00000164792	cfap74	0.02603196	-0.15899724	-0.307564837	-0.482816939	-0.375147114	downregulated during regeneration - mid
ENSDART00000164855	crebl2	0.013882038	-0.127345071	-0.2122205	-0.432997567	-0.264872881	downregulated during regeneration - mid
ENSDART00000164983	anapc15	0.022231819	-0.036185378	-0.198619926	-0.408385072	-0.233599439	downregulated during regeneration - mid
ENSDART00000165018	cdc42se2	0.019981901	-0.053295614	-0.137825766	-0.478383249	-0.349419762	downregulated during regeneration - mid
ENSDART00000165049	impg2b	0.011108721	-0.010489295	-0.310856751	-0.727501593	-0.334825186	downregulated during regeneration - mid
ENSDART00000165065	uqcr10	0.046716104	-0.222678585	-0.318669731	-0.412855116	-0.201529958	downregulated during regeneration - mid
ENSDART00000165308	me2	0.026211285	-0.16075229	-0.327815721	-0.451710296	-0.323851706	downregulated during regeneration - mid
ENSDART00000165333	si:ch211-207l14.1	0.024829185	-0.144575592	-0.337549275	-0.714109722	-0.444634079	downregulated during regeneration - mid
ENSDART00000165425	aak1a	0.033202686	-0.009756971	-0.269537246	-0.43668389	-0.03607957	downregulated during regeneration - mid
ENSDART00000165542	si:dkeyp-72e1.9	0.014624499	0.115590472	-0.201422352	-0.774050016	-0.351374892	downregulated during regeneration - mid
ENSDART00000165557	BX088524.3	0.018703026	-0.275785171	-0.331787981	-0.469187171	-0.127928431	downregulated during regeneration - mid
ENSDART00000166177	impg2b	0.000397106	0.006257085	-0.447154942	-0.735592553	-0.255871486	downregulated during regeneration - mid
ENSDART00000166241	inpp5b	0.040796459	-0.146961161	-0.217731387	-0.46875304	-0.117265313	downregulated during regeneration - mid
ENSDART00000166313	thrb	0.0223553	-0.193382381	-0.415286369	-0.400030228	-0.136855946	downregulated during regeneration - mid
ENSDART00000166374	si:dkey-31f5.11	0.05364057	-0.246069029	-0.257528931	-0.445065012	-0.224168767	downregulated during regeneration - mid
ENSDART00000166463	cnot6b	0.033934614	-0.099106983	-0.163429858	-0.300924183	-0.105283807	downregulated during regeneration - mid
ENSDART00000166515	si:dkeyp-57d7.4	0.006203091	-0.099733783	-0.492859873	-1.003446185	-0.404973513	downregulated during regeneration - mid
ENSDART00000166650	bsg	0.017988416	-0.333252581	-0.445374368	-0.502358398	-0.336861466	downregulated during regeneration - mid
ENSDART00000166659	lrrn2	0.002931511	-0.321629641	-0.404637061	-0.652990901	-0.235544369	downregulated during regeneration - mid
ENSDART00000166714	impg2b	0.033226863	-0.072742859	-0.391473223	-0.740015258	-0.360324281	downregulated during regeneration - mid
ENSDART00000166843	tom1	0.02794941	-0.282793851	-0.146845245	-0.347923357	-0.109554429	downregulated during regeneration - mid
ENSDART00000167117	si:ch1073-469d17.2	0.075212446	-0.226355895	-0.436402154	-0.568359696	-0.320345187	downregulated during regeneration - mid
ENSDART00000167179	asf1ba	0.000512281	-0.325814646	-0.52479315	-0.773177729	-0.605632897	downregulated during regeneration - mid
ENSDART00000167391	arhgap21a	0.004364661	-0.385966805	-0.235029359	-0.527723306	-0.308128762	downregulated during regeneration - mid
ENSDART00000167570	actr3b	0.000292971	-0.361119463	-0.449811578	-0.822009016	-0.639517901	downregulated during regeneration - mid
ENSDART00000167612	rnf34a	0.030702335	-0.125673437	-0.210904428	-0.512991238	-0.24247481	downregulated during regeneration - mid
ENSDART00000167660	pja2	0.003557275	-0.078344887	-0.326712885	-0.382150009	-0.239022195	downregulated during regeneration - mid
ENSDART00000167948	hcn1	0.022898139	-0.135796002	-0.504326543	-0.514845616	-0.152463147	downregulated during regeneration - mid
ENSDART00000167977	kcnh4b	0.021960856	-0.815041676	-0.830195363	-1.200187818	-0.588368652	downregulated during regeneration - mid
ENSDART00000168036	rdh8b	0.047560325	-0.188576333	-0.341040166	-0.496499443	-0.09323063	downregulated during regeneration - mid
ENSDART00000168160	pip5k1cb	0.007934853	-0.047296457	-0.437985038	-0.478356963	-0.284131261	downregulated during regeneration - mid
ENSDART00000168531	irf2bp2b	0.089399502	-0.128403316	-0.188582728	-0.404745176	-0.138034767	downregulated during regeneration - mid
ENSDART00000168616	ppa1a	0.022223646	0.095923853	-0.258115212	-0.556184684	-0.293684673	downregulated during regeneration - mid
ENSDART00000168639	cry1ab	0.023297649	-0.18716056	-0.280815639	-0.387838008	-0.275846695	downregulated during regeneration - mid
ENSDART00000168850	taok1b	0.052592075	-0.044686095	-0.116318692	-0.429811181	-0.132610503	downregulated during regeneration - mid
ENSDART00000169363	CR678435.1	0.029303695	-0.089321474	-0.319658973	-0.354063558	-0.259523127	downregulated during regeneration - mid
ENSDART00000169367	CABZ01061884.1	0.037612568	-0.298329532	-0.377577791	-0.54544765	-0.245416552	downregulated during regeneration - mid
ENSDART00000169376	PLEKHG5 (1 of many)	0.004338147	-0.003560342	-0.365335228	-0.516938003	-0.057649582	downregulated during regeneration - mid
ENSDART00000169484	amph	0.012342358	-0.176945265	-0.219628366	-0.476094931	-0.135317446	downregulated during regeneration - mid
ENSDART00000169511	ppp2r2bb	0.005388807	-0.347621729	-0.40572046	-0.501873649	-0.158073428	downregulated during regeneration - mid
ENSDART00000169733	klhl23	0.013248249	-0.117061422	-0.244154323	-0.447638501	-0.20478162	downregulated during regeneration - mid

ENSDART00000169828	rlbp1a	0.024411518	-0.278789297	-0.290042852	-0.461967142	-0.22894081	downregulated during regeneration - mid
ENSDART00000169932	ccdc142	0.032850794	-0.157730988	-0.223081294	-0.3402281	-0.055715693	downregulated during regeneration - mid
ENSDART00000169943	CABZ01084447.1	0.000529346	-0.382296742	-0.397697491	-0.572266726	-0.473542202	downregulated during regeneration - mid
ENSDART00000170012	mapre3b	0.020911664	-0.205130823	-0.301066074	-0.42167523	-0.136278286	downregulated during regeneration - mid
ENSDART00000170208	limch1b	0.017620686	-0.042803542	-0.148322889	-0.621537079	-0.273084471	downregulated during regeneration - mid
ENSDART00000170285	def8	0.009606339	-0.050118345	-0.13948188	-0.345534099	-0.220243255	downregulated during regeneration - mid
ENSDART00000170376	mpp7a	0.034023228	-0.022565767	-0.302686812	-0.467837523	-0.093897739	downregulated during regeneration - mid
ENSDART00000170546	wdr17	0.062857343	-0.20142732	-0.213426713	-0.433783757	-0.087288031	downregulated during regeneration - mid
ENSDART00000170575	nfat5b	0.018611393	-0.001493923	-0.392122738	-0.566909919	-0.288747412	downregulated during regeneration - mid
ENSDART00000170583	43167	0.022767452	-0.037993536	-0.377054474	-0.284528801	-0.103767971	downregulated during regeneration - mid
ENSDART00000170695	lrit1b	0.041328295	-0.349980517	-0.215317539	-0.688918418	-0.304938883	downregulated during regeneration - mid
ENSDART00000170762	slc44a5b	0.022987263	-0.083921908	-0.392138039	-0.663427101	-0.045247688	downregulated during regeneration - mid
ENSDART00000170856	smu1b	0.004570574	0.353209488	-0.339997639	-0.798035862	-0.487523616	downregulated during regeneration - mid
ENSDART00000170955	fn3krp	0.064000341	-0.183544623	-0.156282547	-0.395153052	-0.231090781	downregulated during regeneration - mid
ENSDART00000171202	clrn1	0.023596757	-0.342498627	-0.174544267	-0.411984605	-0.164186516	downregulated during regeneration - mid
ENSDART00000171237	kcnj2b	0.002963545	-0.406552715	-0.524353506	-0.661059283	-0.393570981	downregulated during regeneration - mid
ENSDART00000171320	dcp2	0.018930059	-0.072540161	-0.060086595	-0.320231159	-0.153613562	downregulated during regeneration - mid
ENSDART00000171393	efr3a	0.008676069	0.028329226	-0.20834437	-0.54511994	-0.110508442	downregulated during regeneration - mid
ENSDART00000171639	prkacbb	0.016570302	-0.216859972	-0.333993863	-0.380485937	-0.232933535	downregulated during regeneration - mid
ENSDART00000171823	cdc14ab	0.07124152	-0.17216976	-0.3147465	-0.385013143	-0.20236803	downregulated during regeneration - mid
ENSDART00000172076	hook1	0.057207579	-0.040243255	-0.111929437	-0.520350092	-0.090216969	downregulated during regeneration - mid
ENSDART00000172114	calm3a	0.016086191	-0.154349916	-0.209222212	-0.409530683	-0.13969668	downregulated during regeneration - mid
ENSDART00000172166	NDUFC1	0.016665414	-0.092745693	-0.325133407	-0.417118078	-0.256060161	downregulated during regeneration - mid
ENSDART00000172190	ajap1	0.020568703	-0.155573908	-0.463634934	-0.533953643	-0.131876486	downregulated during regeneration - mid
ENSDART00000172294	ctps1b	0.001175355	-0.150552652	-0.426760874	-0.523567714	-0.150809326	downregulated during regeneration - mid
ENSDART00000172662	fam96a	0.013435197	-0.029501914	-0.191990056	-0.34693921	-0.272376792	downregulated during regeneration - mid
ENSDART00000172811	ubap1lb	0.017531533	0.254294803	-0.127702895	-0.996786994	-0.222342304	downregulated during regeneration - mid
ENSDART00000172944	FO681390.1	0.035889612	-0.061230657	-0.175829366	-0.634551101	-0.148743958	downregulated during regeneration - mid
ENSDART00000173056	arap3	0.010245667	-0.20638853	-0.271205037	-0.427111789	-0.285607029	downregulated during regeneration - mid
ENSDART00000173169	pclob	0.015925333	-0.241926883	-0.695024268	-0.666850982	-0.322920156	downregulated during regeneration - mid
ENSDART00000173400	FP236327.1	0.017516593	-0.107319508	-0.233503644	-0.613884847	-0.314094176	downregulated during regeneration - mid
ENSDART00000173430	pfklb	0.002058842	-0.150367408	-0.216221217	-0.597100494	-0.348773684	downregulated during regeneration - mid
ENSDART00000003042	mdkb	0.000235661	-0.457982371	-0.488604373	-0.629508021	-0.485178616	downregulated during regeneration - late
ENSDART00000003612	NA	0.0080829	-0.145765054	-0.499207076	-0.35686816	-0.607333409	downregulated during regeneration - late
ENSDART00000004903	rdh10b	0.01613952	-0.385872948	-0.19159484	-0.565090954	-0.486336464	downregulated during regeneration - late
ENSDART00000005053	slc12a4	0.020066139	-0.135764977	-0.039802706	-0.399029267	-0.999098872	downregulated during regeneration - late
ENSDART00000007122	guca1b	0.03198451	-0.373174817	-0.243090423	-0.518795688	-0.641476933	downregulated during regeneration - late
ENSDART00000008010	pdk2a	0.008401701	-0.070635847	0.148907691	-0.31523766	-0.171233724	downregulated during regeneration - late
ENSDART00000008326	pon2	0.003003196	0.005358778	-0.010211923	-0.591626082	-0.490299784	downregulated during regeneration - late
ENSDART00000008607	ttyh2l	0.001115539	0.127438148	-0.071060271	-0.346686037	-0.327410338	downregulated during regeneration - late
ENSDART00000009952	zfand5a	0.001528476	-0.376361025	-0.47427013	-1.180526792	-1.063292908	downregulated during regeneration - late
ENSDART00000010046	rhpn2	0.005024347	-0.531285649	-0.280012714	-0.367908087	-0.481376216	downregulated during regeneration - late
ENSDART00000011135	kita	0.005077923	-0.1368269	-0.266279657	-0.502022945	-0.592221276	downregulated during regeneration - late
ENSDART00000011519	slc6a1l	0.002718393	0.105782424	-0.162202998	-0.466558128	-0.662799909	downregulated during regeneration - late
ENSDART00000014843	bdnf	0.000108354	-0.572929745	-0.434797241	-0.517838864	-0.969054184	downregulated during regeneration - late
ENSDART00000015095	uts1	0.00114562	-0.304915972	-0.432455801	-0.651436618	-0.693973757	downregulated during regeneration - late
ENSDART00000017485	sf3b6	0.015609101	-0.19155437	-0.273140548	-0.376775786	-0.432460535	downregulated during regeneration - late
ENSDART00000018054	trh	0.001423412	-0.369432982	-0.400024601	-0.784012545	-0.865751572	downregulated during regeneration - late
ENSDART00000018347	cab39l1	0.011046859	-0.259625217	-0.162823278	-0.312896931	-0.294075261	downregulated during regeneration - late
ENSDART00000018886	ghra	0.006658285	-0.12746236	-0.080311259	-0.403757478	-0.551820215	downregulated during regeneration - late
ENSDART00000019766	tgfb3	0.020798309	-0.14127581	-0.182290633	-0.363811528	-0.271524635	downregulated during regeneration - late
ENSDART00000020999	angptl1a	0.068605693	-0.447558553	-0.389827093	-0.516507166	-1.101603289	downregulated during regeneration - late
ENSDART00000021121	stx5al	0.020719498	-0.079551416	-0.239231712	-0.554538789	-0.564757113	downregulated during regeneration - late
ENSDART00000021168	rxrga	0.00269825	0.040179684	-0.194377683	-0.381512289	-0.377392107	downregulated during regeneration - late
ENSDART00000022051	gins1	0.011046859	-0.049060202	-0.07395231	-0.409999594	-0.285702931	downregulated during regeneration - late
ENSDART00000022581	rab22a	0.018963501	-0.072513798	-0.08434934	-0.299709625	-0.230388715	downregulated during regeneration - late
ENSDART00000023613	her6	0.030622517	-0.335042933	-0.234567638	-0.250120727	-0.59712361	downregulated during regeneration - late
ENSDART00000024328	slc34a2a	0.032189514	-1.294382652	-0.588426598	-0.664087867	-1.114341851	downregulated during regeneration - late
ENSDART00000026085	ptges	0.014800178	-0.133597623	-0.186036004	-0.449634161	-0.455296325	downregulated during regeneration - late
ENSDART00000026316	sema3gb	0.023200703	-0.148485986	-0.183297493	-0.3621349	-0.465943338	downregulated during regeneration - late
ENSDART00000027417	zgc:171704	0.00039492	0.227240571	-0.665719796	-1.161293822	-1.438167938	downregulated during regeneration - late
ENSDART00000029946	ube2b	0.013774152	-0.262228145	-0.117966704	-0.377845713	-0.176574464	downregulated during regeneration - late
ENSDART00000030579	crhbp	0.001133915	0.109353477	-0.053663997	-0.382440888	-0.742296052	downregulated during regeneration - late
ENSDART00000031727	vamp8	0.000787471	0.086516161	-0.020853386	-0.34297459	-0.531441391	downregulated during regeneration - late
ENSDART00000032393	ginm1	0.012470284	-0.054454012	-0.092963972	-0.327748186	-0.224389181	downregulated during regeneration - late
ENSDART00000032502	nebl	0.026706504	-0.405575453	-0.348273521	-0.638746976	-0.69843843	downregulated during regeneration - late
ENSDART00000034638	ccdc28a	0.018433591	-0.059055282	-0.067802136	-0.368600818	-0.274995246	downregulated during regeneration - late
ENSDART00000035031	sgk1	0.002559781	0.036456511	-0.228295468	-0.323414575	-0.393803665	downregulated during regeneration - late
ENSDART00000035067	abhd2a	0.008989451	-0.280282686	-0.122469223	-0.369410687	-0.218167862	downregulated during regeneration - late
ENSDART00000038310	ormdl3	0.010195593	-0.300841314	-0.124106092	-0.535837175	-0.413488994	downregulated during regeneration - late
ENSDART00000038674	tmem230a	0.005028852	-0.312877262	-0.360088684	-0.444507782	-0.485519275	downregulated during regeneration - late
ENSDART00000039399	cavin2a	0.000442465	-0.454722967	-0.463350309	-0.727916614	-0.482344794	downregulated during regeneration - late
ENSDART00000042123	cx52.6	0.003425348	-0.622226461	-0.079417665	-0.692157173	-0.611068819	downregulated during regeneration - late
ENSDART00000042307	fam60a	0.000248952	-0.454119179	-0.522927402	-0.624149133	-1.020018948	downregulated during regeneration - late
ENSDART00000042481	phf23a	0.019414194	-0.371442433	-0.230558793	-0.302265692	-0.28776115	downregulated during regeneration - late

ENSDART00000043226	guca1c	0.014712033	-0.531402053	-0.370893475	-0.804093243	-0.754648782	downregulated during regeneration - late
ENSDART00000044426	si:dkey-240h12.4	0.016178224	0.077516947	-0.039107616	-0.22879164	-0.507759534	downregulated during regeneration - late
ENSDART00000044860	maff	0.002310171	0.057535561	0.039208376	-0.366680053	-0.441007369	downregulated during regeneration - late
ENSDART00000045126	lama5	0.007419702	-0.044158589	0.007727115	-0.324426294	-0.496259172	downregulated during regeneration - late
ENSDART00000046360	rhoua	0.001489209	-0.234114921	-0.11992892	-0.459682602	-0.396381281	downregulated during regeneration - late
ENSDART00000046716	cited2	0.010493088	-0.252125812	-0.225219779	-0.427941506	-0.423861644	downregulated during regeneration - late
ENSDART00000047378	sst3	0.024602158	-0.489863502	-0.562162931	-0.649979789	-0.586308909	downregulated during regeneration - late
ENSDART00000049075	add3a	0.023840313	-0.25124279	-0.167771329	-0.330721354	-0.242087591	downregulated during regeneration - late
ENSDART00000050230	tspan3a	0.014404044	-0.260999172	-0.146975695	-0.364155673	-0.280149491	downregulated during regeneration - late
ENSDART00000051197	c10h21orf59	0.043628495	-0.175790498	-0.1331203	-0.516409592	-0.442586367	downregulated during regeneration - late
ENSDART00000051357	zmat5	0.04367746	-0.126081267	-0.164432805	-0.376616148	-0.245219161	downregulated during regeneration - late
ENSDART00000051516	tacr1a	0.03254446	-0.234436772	-0.252170036	-0.371966451	-0.377528767	downregulated during regeneration - late
ENSDART00000051546	rps6ka3a	0.004239143	-0.063638799	0.031915539	-0.376231824	-0.261540289	downregulated during regeneration - late
ENSDART00000051655	snrnp27	0.019324618	-0.149284802	-0.196914421	-0.340354698	-0.296345743	downregulated during regeneration - late
ENSDART00000051948	si:dkey-17m8.2	0.029473595	-2.249269734	-0.678959768	-1.034434829	-1.318620097	downregulated during regeneration - late
ENSDART00000052029	cart3	0.000305619	-0.527262177	-0.518524777	-0.63363087	-0.567396556	downregulated during regeneration - late
ENSDART00000052423	spry2	0.008879163	-0.373633249	-0.274093568	-0.317163264	-0.534996547	downregulated during regeneration - late
ENSDART00000052703	nucb2b	0.005154611	-0.072514611	-0.317049949	-0.490530665	-0.459714357	downregulated during regeneration - late
ENSDART00000052912	pcdh20	0.016905836	-0.38541457	-0.110151808	-0.462368809	-0.238274084	downregulated during regeneration - late
ENSDART00000053310	tmem18	0.023272159	-0.091641733	-0.057752607	-0.353980434	-0.264448619	downregulated during regeneration - late
ENSDART00000053463	mgll	0.063259686	-0.18197879	-0.150318561	-0.320930933	-0.180307684	downregulated during regeneration - late
ENSDART00000053773	lsm6	0.013538515	-0.104076864	-0.094751595	-0.350900229	-0.413521857	downregulated during regeneration - late
ENSDART00000053916	mtnr1ab	0.032098855	-0.333808774	-0.363646472	-0.291230509	-0.411800208	downregulated during regeneration - late
ENSDART00000054386	qdprb1	0.066779801	-0.130677564	-0.146959168	-0.522581238	-0.268035824	downregulated during regeneration - late
ENSDART00000054462	smim19	0.016203475	-0.090105146	-0.174385509	-0.37090077	-0.289350438	downregulated during regeneration - late
ENSDART00000054867	aup1	0.017245517	-0.021467306	-0.053295505	-0.309019838	-0.217937602	downregulated during regeneration - late
ENSDART00000055139	col9a3	0.000444888	0.340683061	0.20859259	-0.192921312	-1.195377277	downregulated during regeneration - late
ENSDART00000055706	her15.1	0.021144178	-1.483608056	-1.154441068	-0.698110478	-1.436427969	downregulated during regeneration - late
ENSDART00000055709	her2	0.030146589	-0.984524994	-0.833253569	-0.717745512	-1.233522642	downregulated during regeneration - late
ENSDART00000055779	ggact.2	0.030279926	-0.390632373	-0.197002813	-0.565987089	-0.546417063	downregulated during regeneration - late
ENSDART00000056939	zgc:85858	0.037793991	-0.254903494	-0.357469282	-0.615277126	-0.499477333	downregulated during regeneration - late
ENSDART00000056996	sfrp5	0.011389892	0.077859934	0.01622227	-0.244583159	-1.042810978	downregulated during regeneration - late
ENSDART00000057124	tefa	0.007560757	-0.17959692	0.074135039	-0.330628318	-0.262330789	downregulated during regeneration - late
ENSDART00000057125	tefa	0.002939873	-0.190705988	0.094171259	-0.306461854	-0.301060608	downregulated during regeneration - late
ENSDART00000057439	parla	0.056801891	-0.763945698	-0.13237506	-0.4980372	-0.373141189	downregulated during regeneration - late
ENSDART00000057519	zgc:194209	0.001633797	0.014343488	-0.12072846	-0.449908342	-0.453116382	downregulated during regeneration - late
ENSDART00000057689	bag3	0.030198325	-0.537315577	-0.216588912	-0.658528557	-0.795247472	downregulated during regeneration - late
ENSDART00000057865	ier3ip1	0.003879543	-0.032004312	-0.136273649	-0.345681209	-0.364367077	downregulated during regeneration - late
ENSDART00000058667	RDH13 (1 of many)	0.0088045	-0.138024992	-0.086121321	-0.690305733	-0.540517763	downregulated during regeneration - late
ENSDART00000059478	lrrc32	0.00518047	-0.083635057	-0.196945688	-0.286292563	-0.79945825	downregulated during regeneration - late
ENSDART00000059550	lrrc51	0.005486754	-1.204791639	-0.640574252	-1.049736423	-1.0433072	downregulated during regeneration - late
ENSDART00000060174	jagn1a	0.011982948	-0.416430339	-0.038483688	-0.018273384	-0.314381472	downregulated during regeneration - late
ENSDART00000060304	dhrs13a.3	0.001013659	-0.00917183	0.036729289	-0.280144118	-0.407439325	downregulated during regeneration - late
ENSDART00000061007	mt2	0.000122194	-0.024087588	-0.437804286	-0.890429121	-0.686083783	downregulated during regeneration - late
ENSDART00000061117	rrbp1b	0.019354723	-0.094086695	0.04423759	-0.324207933	-0.60750175	downregulated during regeneration - late
ENSDART00000061261	cx43	0.004463264	-0.064274202	-0.132171143	-0.392515504	-0.502400185	downregulated during regeneration - late
ENSDART00000061435	hsbp1b	0.014624499	-0.02310974	-0.083751559	-0.317854229	-0.256774332	downregulated during regeneration - late
ENSDART00000062066	si:dkey-177p2.6	0.005438298	-0.250914133	-0.251912097	-0.422547135	-0.291399654	downregulated during regeneration - late
ENSDART00000062143	zgc:77650	0.004824695	-0.109887255	-0.133378465	-0.376117658	-0.331935654	downregulated during regeneration - late
ENSDART00000062181	RALGDS	0.019466285	-0.386705371	-0.180287867	-0.473841885	-0.479084169	downregulated during regeneration - late
ENSDART00000062518	gstr	0.027807337	-0.173225499	-0.241367808	-0.232576695	-0.339957813	downregulated during regeneration - late
ENSDART00000063625	gpx3	0.000679804	0.093413749	0.050792899	-0.431389541	-0.288399492	downregulated during regeneration - late
ENSDART00000063804	wu:fj39g12	0.000278198	-1.205265642	-1.354971991	-1.517691105	-1.318916155	downregulated during regeneration - late
ENSDART00000064111	faub	0.021419678	0.024926044	-0.061766338	-0.402186419	-0.29870419	downregulated during regeneration - late
ENSDART00000064376	sod1	0.030292962	-0.248477917	-0.212122084	-0.427881094	-0.390078569	downregulated during regeneration - late
ENSDART00000064878	gxylt2	0.010154159	-0.088947943	-0.180342387	-0.380560197	-0.388684923	downregulated during regeneration - late
ENSDART00000066703	rdh10a	0.019015879	-0.287660525	-0.158056129	-0.356819377	-0.262167994	downregulated during regeneration - late
ENSDART00000067239	guca1g	0.003508363	-0.979413995	-1.042352228	-1.597708164	-1.275542792	downregulated during regeneration - late
ENSDART00000067362	cart2	0.014638227	-0.749355761	-0.384853037	-0.719287733	-0.625188951	downregulated during regeneration - late
ENSDART00000067446	slc38a4	0.000237691	-0.251589327	-0.331078479	-0.606665517	-0.538910759	downregulated during regeneration - late
ENSDART00000074100	osgn1	0.000510711	0.255332714	0.013078065	-0.613186841	-0.487873824	downregulated during regeneration - late
ENSDART00000074380	tsga10	0.026816553	-0.358572407	-0.32335115	-0.582555062	-0.41305108	downregulated during regeneration - late
ENSDART00000074458	ptpmt1	0.006314435	-0.138560892	-0.279060294	-0.535667209	-0.518833125	downregulated during regeneration - late
ENSDART00000075150	bmp4	0.0114419	-0.321654174	-0.192558055	-0.354985644	-0.520317573	downregulated during regeneration - late
ENSDART00000075499	si:dkey-283b1.7	0.001565956	-0.753135255	-0.256638623	-0.866666345	-0.691414461	downregulated during regeneration - late
ENSDART00000075519	aldh1a2	0.047619389	-0.136704985	-0.161016939	-0.528203419	-0.420522124	downregulated during regeneration - late
ENSDART00000075663	cracr2b	0.004668635	0.080855847	0.057965052	-0.927246946	-0.842015409	downregulated during regeneration - late
ENSDART00000076066	lin37	0.005026727	-0.271630989	-0.17922011	-0.496068109	-0.578879729	downregulated during regeneration - late
ENSDART00000077008	alox5ap	0.0014833	0.122223757	-0.082901221	-0.550940123	-0.70030614	downregulated during regeneration - late
ENSDART00000077080	PTP4A3 (1 of many)	0.006896678	0.077833133	0.004217905	-0.463336754	-0.666773375	downregulated during regeneration - late
ENSDART00000077411	cxcl12b	0.001960123	-0.236947042	-0.2488008	-0.593903488	-0.559818867	downregulated during regeneration - late
ENSDART00000077951	pcolce2b	0.013245259	-0.119540012	-0.232226227	-0.447813206	-1.286575617	downregulated during regeneration - late
ENSDART00000078148	smc1a	0.041808931	-3.399991944	-0.069804776	-1.248223526	-0.569589215	downregulated during regeneration - late
ENSDART00000078181	SLC3A2 (1 of many)	0.020491546	-0.23816401	-0.122270906	-0.56307324	-1.283472558	downregulated during regeneration - late
ENSDART00000078449	itih6	0.002591094	-0.23325998	-0.04381884	-0.494745518	-1.555031348	downregulated during regeneration - late

ENSDART00000078858	si:ch73-86n18.1	0.003028992	0.106229924	-0.155735751	-0.58770094	-0.854457693	downregulated during regeneration - late
ENSDART00000078916	smim7	0.014686352	-0.217464923	-0.109941617	-0.350272436	-0.25934653	downregulated during regeneration - late
ENSDART00000079112	calca	0.031235096	-0.439449486	-0.32190484	-0.601830596	-0.728154291	downregulated during regeneration - late
ENSDART00000079879	si:dkey-91i10.3	0.060601627	-0.580141681	-0.553279281	-0.678150475	-0.52463511	downregulated during regeneration - late
ENSDART00000080014	rps8b	0.012554187	-0.261943359	-0.309301007	-0.481979191	-0.409711249	downregulated during regeneration - late
ENSDART00000081946	zgc:112332	0.003060275	-0.051059621	0.027246103	-0.289131429	-0.652053172	downregulated during regeneration - late
ENSDART00000082151	uqcrh	0.037614097	-0.212671975	-0.358890471	-0.337939592	-0.281374001	downregulated during regeneration - late
ENSDART00000083367	prcp	0.014634638	-0.058674879	-0.090308681	-0.37460188	-0.414832363	downregulated during regeneration - late
ENSDART00000084055	fzd7a	0.04742464	-0.350921417	-0.318886271	-0.299513467	-0.86880486	downregulated during regeneration - late
ENSDART00000084131	fam160b2	0.009592472	0.036570039	-0.082247362	-0.429951116	-0.30533058	downregulated during regeneration - late
ENSDART00000084598	vimp	0.005671256	-0.003252442	-0.076004385	-0.324006724	-0.304272734	downregulated during regeneration - late
ENSDART00000084861	cish	0.004022079	-0.446878588	-0.18032932	-1.508647694	-1.506136587	downregulated during regeneration - late
ENSDART00000085294	tnfrsf9a	0.001754036	-0.468498688	-0.352709139	-0.665957795	-1.403982945	downregulated during regeneration - late
ENSDART00000085442	mut	0.009834259	-0.094284216	-0.181855717	-0.336054518	-0.342951233	downregulated during regeneration - late
ENSDART00000086051	mecom	0.04049764	-0.550304416	-0.298852875	-0.293768303	-1.334615988	downregulated during regeneration - late
ENSDART00000086905	nrn1lb	0.000133356	-0.455174799	-0.417502528	-0.690990621	-0.656036806	downregulated during regeneration - late
ENSDART00000087339	cdon	0.026243123	-0.358409006	-0.160970657	-0.495687672	-0.865355598	downregulated during regeneration - late
ENSDART00000088342	cytip	0.00140081	0.001918005	-0.070156162	-0.471206178	-0.369058853	downregulated during regeneration - late
ENSDART00000089015	zbtb7a	0.00191624	-0.478666207	-0.203919761	-0.738823143	-0.713706626	downregulated during regeneration - late
ENSDART00000089339	dph7	0.018858047	-0.317220664	-0.267955931	-0.402220665	-0.245139947	downregulated during regeneration - late
ENSDART00000089342	cfap126	0.014365121	-0.196227391	-0.233968621	-0.449699376	-0.526724945	downregulated during regeneration - late
ENSDART00000091124	aifm3	0.010447806	-0.238811768	-0.279253276	-0.598306352	-0.861256088	downregulated during regeneration - late
ENSDART00000091156	tacc1	0.012895641	-0.166984032	-0.162668339	-0.396514006	-0.330157066	downregulated during regeneration - late
ENSDART00000091252	spata13	0.001728321	0.108055641	-0.023595371	-0.309799561	-0.91906317	downregulated during regeneration - late
ENSDART00000093000	PLEKHB1	0.010802023	-0.366311468	-0.143931789	-0.403311135	-0.393296015	downregulated during regeneration - late
ENSDART00000098045	gas1b	0.005181375	-0.225161666	-0.17230943	-0.331649886	-0.52065088	downregulated during regeneration - late
ENSDART00000098575	trim110	0.020470078	-0.538539785	-0.074004136	-0.613819609	-0.257802554	downregulated during regeneration - late
ENSDART00000099089	ggcx	0.031769506	-0.059935848	-0.149142859	-0.333945915	-0.254577798	downregulated during regeneration - late
ENSDART00000099208	asph	0.026872157	-0.288641006	-0.271409423	-0.460024196	-0.387402543	downregulated during regeneration - late
ENSDART00000099476	fam174b	0.003432504	-0.249603276	-0.126773683	-0.376574062	-0.35637543	downregulated during regeneration - late
ENSDART00000099528	spry4	0.000910711	-1.005535524	-0.789062042	-0.760146662	-1.480463024	downregulated during regeneration - late
ENSDART00000100286	fgfr4	0.004958321	-0.349487842	-0.279142199	-0.557520873	-0.83628708	downregulated during regeneration - late
ENSDART00000100386	mstnb	0.032308002	-0.232479836	-0.19741782	-0.546065599	-0.487217319	downregulated during regeneration - late
ENSDART00000100605	ttc32	0.004111621	-0.362267617	-0.196641209	-0.477779827	-0.443477008	downregulated during regeneration - late
ENSDART00000102125	schip1	0.014880314	-0.065634739	-0.008431238	-0.356995513	-0.237294266	downregulated during regeneration - late
ENSDART00000103586	hdac9b	0.003730132	-0.63802499	-0.480501706	-0.6152094	-0.590506452	downregulated during regeneration - late
ENSDART00000103785	ggact.3	0.044966319	-1.209900002	-0.367470534	-1.050388105	-0.531293993	downregulated during regeneration - late
ENSDART00000103795	ggact.1	0.008574428	0.007439004	-0.050660849	-0.406911538	-0.573615848	downregulated during regeneration - late
ENSDART00000104043	dkk3b	0.003678598	0.124905707	-0.440538945	-0.566305744	-1.06504734	downregulated during regeneration - late
ENSDART00000104257	lpin1	0.011195192	-0.263973453	-0.162876624	-0.357103753	-0.3626544	downregulated during regeneration - late
ENSDART00000104545	avpr2ab	0.033298538	-0.127228111	-0.325246207	-0.456539735	-1.288721053	downregulated during regeneration - late
ENSDART00000104592	pm20d1.2	0.003337853	-0.022497234	-0.114097556	-0.391118153	-0.365853437	downregulated during regeneration - late
ENSDART00000105323	zgc:162255	0.003678598	-0.250927131	-0.470845634	-0.44137647	-0.557741796	downregulated during regeneration - late
ENSDART00000105813	krt222	0.028543949	-0.522679169	-0.470823148	-0.650543043	-0.842913393	downregulated during regeneration - late
ENSDART00000105903	lsm5	0.003228981	0.135971292	-0.078983757	-0.333257253	-0.331997667	downregulated during regeneration - late
ENSDART00000109124	rspo2	0.005414718	0.077521406	0.271943255	-0.228972258	-0.854369599	downregulated during regeneration - late
ENSDART00000109546	unc5b	0.002533609	-0.076752071	-0.101810869	-0.423560106	-0.292578205	downregulated during regeneration - late
ENSDART00000109853	zmp:0000000801	0.002881312	-0.4485216	-0.462342097	-0.503236916	-0.960013508	downregulated during regeneration - late
ENSDART00000110544	znf219	0.031344075	-0.234403747	-0.210413301	-0.211690001	-0.32688667	downregulated during regeneration - late
ENSDART00000111165	best2	0.019487475	-0.15773889	-0.249582303	-0.643700083	-1.151697377	downregulated during regeneration - late
ENSDART00000111767	si:ch211-39k3.2	0.001551573	-0.094085509	-0.095643794	-0.600534706	-0.396103136	downregulated during regeneration - late
ENSDART00000111799	HEPACAM (1 of many)	0.022218395	-0.300776869	-0.301240305	-0.377807505	-0.329578876	downregulated during regeneration - late
ENSDART00000111905	BX649498.1	0.031814592	-0.175767299	-0.206924623	-0.494153858	-0.425985895	downregulated during regeneration - late
ENSDART00000112333	cnnm2b	0.00144644	-0.413171039	-0.296279679	-0.539640998	-0.529524127	downregulated during regeneration - late
ENSDART00000112579	scg2b	0.00638925	-0.360046965	-0.210825369	-0.351760362	-0.440018453	downregulated during regeneration - late
ENSDART00000112845	zgc:195173	0.001259259	-0.156651959	-0.182049019	-0.580426063	-0.462296521	downregulated during regeneration - late
ENSDART00000112856	TNC (1 of many)	0.024409241	-0.33689758	-0.285587425	-0.51132075	-1.127367477	downregulated during regeneration - late
ENSDART00000113197	prok1	0.007586659	-0.431580361	-0.477066939	-0.390033973	-0.689815046	downregulated during regeneration - late
ENSDART00000113734	chrdl2	0.03875014	-0.47545279	-0.359333121	-0.57438139	-0.417914769	downregulated during regeneration - late
ENSDART00000113859	crispld1a	0.000346997	0.006653123	-0.124334074	-0.47278833	-1.109435686	downregulated during regeneration - late
ENSDART00000114319	si:ch211-76l23.7	0.037291854	-0.274578898	-0.196596974	-0.473622321	-1.111584541	downregulated during regeneration - late
ENSDART00000114676	gstm.2	0.00889295	0.102339801	-0.045272058	-0.466318631	-0.673317054	downregulated during regeneration - late
ENSDART00000115356	rabif	0.025960292	-0.104235333	-0.157538528	-0.394672329	-0.327606577	downregulated during regeneration - late
ENSDART00000116415	NA	0.01907307	-0.367308157	-0.297245306	-0.576085374	-0.866023911	downregulated during regeneration - late
ENSDART00000121226	FO704882.1	0.007797716	-0.227266262	-0.409349874	-0.661498137	-0.983304677	downregulated during regeneration - late
ENSDART00000121675	angptl1a	0.033012979	-0.785726694	-0.739578659	-0.711456	-1.277545302	downregulated during regeneration - late
ENSDART00000121716	FAM107A	0.022475824	-0.353665396	-0.22740498	-0.416694229	-0.314761483	downregulated during regeneration - late
ENSDART00000121817	fbln7	0.002545259	0.143978228	0.156275351	-0.420940219	-1.064416584	downregulated during regeneration - late
ENSDART00000122359	si:dkey-164f24.2	0.005230737	-0.316246733	-0.171154186	-0.508268616	-0.361228089	downregulated during regeneration - late
ENSDART00000122519	st8sia6	0.009263888	-0.258839848	-0.191246071	-0.329756561	-0.383108642	downregulated during regeneration - late
ENSDART00000122966	hapln1a	0.031344075	-5.328261829	-0.041096146	-1.913311737	-3.425165696	downregulated during regeneration - late
ENSDART00000123282	si:dkey-7i4.1	0.03479782	-0.131885839	-0.288104676	-0.328002164	-0.299371464	downregulated during regeneration - late
ENSDART00000124762	hsp70.1	0.013341759	-0.061931986	-0.652822267	-0.746588848	-0.837604985	downregulated during regeneration - late
ENSDART00000125116	tnfaip8l3	0.008910267	-0.29259694	-0.291950956	-0.382018775	-0.337072666	downregulated during regeneration - late
ENSDART00000125691	sntb1	0.003107952	-0.296403963	-0.433378655	-0.538134366	-0.484556917	downregulated during regeneration - late

ENSDART00000125923	cxcl14	0.013886694	-0.090480689	-0.043715373	-0.409912244	-0.49269465	downregulated during regeneration - late
ENSDART00000126870	ndufa4l2b	0.005353377	-0.364506533	-0.363911454	-0.576856062	-0.429052776	downregulated during regeneration - late
ENSDART00000127093	CR749162.1	0.045674994	-1.06520168	-0.24719149	-0.825954556	-0.565925266	downregulated during regeneration - late
ENSDART00000127536	cbx7b	0.007396728	-0.401352835	-0.168978702	-0.391659396	-0.597817617	downregulated during regeneration - late
ENSDART00000128011	tnfsf10l3	0.014034602	-0.207145482	-0.351713028	-0.904110327	-0.837055548	downregulated during regeneration - late
ENSDART00000128014	dnah2	0.046252381	-0.407226928	-0.498892771	-0.604204962	-0.453086208	downregulated during regeneration - late
ENSDART00000128127	slc38a3b	0.005596862	-0.302545777	-0.299963192	-0.361680425	-0.612800784	downregulated during regeneration - late
ENSDART00000128149	fam32a	0.003797938	-0.07314666	-0.18816053	-0.368263865	-0.361807904	downregulated during regeneration - late
ENSDART00000129214	COX7A2 (1 of many)	0.01962183	-0.085157083	-0.364093354	-0.62455009	-0.475756885	downregulated during regeneration - late
ENSDART00000129597	sorbs3	0.003535619	0.016863628	0.072993125	-0.314882072	-0.546339357	downregulated during regeneration - late
ENSDART00000129710	crtac1a	0.003137832	-0.277382094	0.025987329	-0.337990061	-0.608225673	downregulated during regeneration - late
ENSDART00000130264	rpz	0.050182269	-0.422979934	-0.28718615	-0.432905099	-0.242626605	downregulated during regeneration - late
ENSDART00000131027	TMEM216	0.033109676	-0.171407049	-0.12306379	-0.494331831	-0.331123076	downregulated during regeneration - late
ENSDART00000131582	cbx3b	0.014192021	-0.219219	-0.174464705	-0.354832647	-0.341719477	downregulated during regeneration - late
ENSDART00000132915	serpine3	0.007368463	-0.093377161	-0.220423221	-0.800809367	-1.077921671	downregulated during regeneration - late
ENSDART00000132995	ak3	0.000854936	0.087221034	0.050666468	-0.189471235	-0.418943401	downregulated during regeneration - late
ENSDART00000133496	CU467110.1	0.0321077	-0.265746647	-0.189978673	-0.362219245	-0.151144337	downregulated during regeneration - late
ENSDART00000133891	FP015789.1	0.00918017	-0.349747936	-0.468679484	-0.676764109	-0.710268862	downregulated during regeneration - late
ENSDART00000134202	ccka	0.012864775	-0.373763887	-0.443030746	-0.635815918	-0.444990754	downregulated during regeneration - late
ENSDART00000135403	nuak2	0.022463379	-0.203159013	-0.012266437	-0.34684763	-0.520334493	downregulated during regeneration - late
ENSDART00000135556	lonrf1	0.00148037	-0.330548829	0.12250287	-0.445965292	-0.332349033	downregulated during regeneration - late
ENSDART00000136049	syt10	0.00992455	-0.382437751	-0.322146795	-0.465356993	-0.383104059	downregulated during regeneration - late
ENSDART00000136084	mfhas1	0.005371229	-0.229093837	-0.123648609	-0.428888075	-0.346098599	downregulated during regeneration - late
ENSDART00000136190	si:ch211-133l5.7	0.008776907	0.046455816	-0.284643729	-0.455348095	-0.512893839	downregulated during regeneration - late
ENSDART00000136538	cish	0.003012303	-0.292880454	-0.45612089	-0.971812918	-1.16494411	downregulated during regeneration - late
ENSDART00000138308	gpr186	0.002651594	-1.473279373	-0.953004046	-1.169373422	-1.314238919	downregulated during regeneration - late
ENSDART00000138661	tspan11	0.00016317	-0.312645804	-0.301551652	-0.855364566	-0.684847545	downregulated during regeneration - late
ENSDART00000139174	fam46c	0.014064789	-0.16066992	-0.125475027	-0.118453643	-0.616919987	downregulated during regeneration - late
ENSDART00000139701	zfand5a	0.001239881	-0.357581428	-0.278083252	-0.905903537	-0.782247372	downregulated during regeneration - late
ENSDART00000140779	fundc1	0.026565285	-1.365145935	-0.380101108	-0.950161557	-1.079798871	downregulated during regeneration - late
ENSDART00000140828	CU179758.1	0.006872094	-0.432947507	-0.14249918	-0.435392677	-0.381420161	downregulated during regeneration - late
ENSDART00000141030	pde4ca	0.026489121	-0.308097694	-0.281644648	-0.391359015	-0.416436171	downregulated during regeneration - late
ENSDART00000141345	si:ch211-133l5.7	0.002874771	0.067098833	-0.266407921	-0.458017208	-0.557765253	downregulated during regeneration - late
ENSDART00000141437	sdc2	0.011417724	0.001568967	0.007072686	-0.196613876	-0.36025161	downregulated during regeneration - late
ENSDART00000141487	pvrl3b	0.003733102	-0.333456043	-0.273917158	-0.488003853	-0.734425178	downregulated during regeneration - late
ENSDART00000141671	socs2	0.000634801	-0.321775893	-0.091083829	-1.001751456	-1.186845404	downregulated during regeneration - late
ENSDART00000141920	si:ch211-224l10.4	0.059256345	-1.330425532	-0.617989075	-0.986788428	-0.796603249	downregulated during regeneration - late
ENSDART00000142112	vma21	0.030307206	-0.082665202	-0.082534524	-0.180302218	-0.34518071	downregulated during regeneration - late
ENSDART00000142196	fam167ab	0.003623005	-0.920556961	-0.832834787	-1.167437341	-1.267796279	downregulated during regeneration - late
ENSDART00000142735	si:ch211-13f8.1	0.003979354	-0.324319655	-0.241821619	-0.588481479	-0.554389586	downregulated during regeneration - late
ENSDART00000142851	itih6	0.001733341	-0.217828278	-0.246312318	-4.361209214	-4.055941297	downregulated during regeneration - late
ENSDART00000143200	kcnma1a	0.037170694	-0.226894558	-0.334282966	-0.480968248	-0.397036214	downregulated during regeneration - late
ENSDART00000143840	gstk1	0.026706504	0.057517576	-0.140839441	-0.201267582	-0.834065155	downregulated during regeneration - late
ENSDART00000144067	cabp5a	0.012049052	-0.477283062	-0.255392436	-0.467866686	-0.403996112	downregulated during regeneration - late
ENSDART00000145282	sepp1a	0.000461528	-0.462981641	-0.396376841	-0.662591735	-0.92136404	downregulated during regeneration - late
ENSDART00000145558	si:ch211-237l4.6	0.040053922	-0.183280044	-0.231325216	-0.614649778	-0.510830341	downregulated during regeneration - late
ENSDART00000145762	dnaaf1	0.000461528	-0.407466904	-0.372899622	-0.590562671	-0.565268948	downregulated during regeneration - late
ENSDART00000146472	kcng4a	0.010687727	-0.174962319	-0.077658154	-0.410630934	-0.442662471	downregulated during regeneration - late
ENSDART00000146892	abcg2d	0.014710738	0.141142908	-0.029073312	-0.499348256	-1.743973024	downregulated during regeneration - late
ENSDART00000147068	olfml2ba	0.013360877	-0.192176717	0.036009408	-0.274084705	-0.587185898	downregulated during regeneration - late
ENSDART00000147201	slc5a7a	0.014511734	-0.334790717	-0.440092664	-0.539424926	-0.607892581	downregulated during regeneration - late
ENSDART00000147582	agmo	0.00862856	-0.172065266	-0.060434285	-0.409289881	-0.408628944	downregulated during regeneration - late
ENSDART00000147854	mfap5	2.23813E-05	-0.362752481	-0.424642186	-0.795916329	-0.74190928	downregulated during regeneration - late
ENSDART00000148456	abcb4	0.020586406	-0.551564057	-0.079040965	-0.313501093	-1.124934266	downregulated during regeneration - late
ENSDART00000149222	pglyrp2	0.005895019	-0.019258678	-0.204426336	-0.652532049	-2.674820692	downregulated during regeneration - late
ENSDART00000149574	ca14	0.025606413	-0.121794187	-0.18625006	-0.561537732	-0.325393218	downregulated during regeneration - late
ENSDART00000149652	AL928685.4	0.043205592	-0.221690272	-0.265792933	-0.407449839	-0.293257945	downregulated during regeneration - late
ENSDART00000149659	ezh1	0.003513906	0.045526141	0.005041587	-0.251417621	-0.327846848	downregulated during regeneration - late
ENSDART00000149689	mob1bb	0.002365371	-0.2705299	-0.18213785	-0.463988827	-0.361129298	downregulated during regeneration - late
ENSDART00000149816	hspb1	0.017271665	-0.566005876	-0.156876752	-0.219080206	-0.313669761	downregulated during regeneration - late
ENSDART00000150386	si:ch73-6k14.2	0.000164423	-0.397499259	-0.321996107	-1.565555621	-1.132350175	downregulated during regeneration - late
ENSDART00000152068	AL590149.1	0.016178224	-0.322191556	-0.083241136	-0.42500156	-0.283301232	downregulated during regeneration - late
ENSDART00000152145	si:ch211-207i1.2	0.024547153	-1.105453902	-1.700204988	-2.013950338	-1.361589232	downregulated during regeneration - late
ENSDART00000152253	kcnj13	0.002332929	-0.175334575	-0.22735068	-0.506366898	-0.552648836	downregulated during regeneration - late
ENSDART00000152942	jcada	0.004739684	-0.053928395	-0.076278472	-0.332447962	-0.296311136	downregulated during regeneration - late
ENSDART00000154141	BX649398.2	0.000216813	0.278828233	0.21540553	-0.861481785	-0.785329833	downregulated during regeneration - late
ENSDART00000155838	luzp1	0.061460285	-0.38633981	-0.211709262	-0.146984907	-0.314685818	downregulated during regeneration - late
ENSDART00000156256	angpt4	0.024383427	-0.447666401	-0.531623063	-0.584960614	-0.899124282	downregulated during regeneration - late
ENSDART00000156509	fam150bb	0.026883176	-0.200699582	-0.03489316	-0.310837396	-0.503245808	downregulated during regeneration - late
ENSDART00000157338	gstz1	0.062825179	-0.391223456	-0.14748942	-0.590184545	-0.319150237	downregulated during regeneration - late
ENSDART00000158260	c1galt1a	0.000459643	0.184684125	0.138397385	-0.286164045	-0.509177074	downregulated during regeneration - late
ENSDART00000159588	rnf24	0.016665414	-0.199572807	-0.336306858	-0.290748993	-0.299485771	downregulated during regeneration - late
ENSDART00000159752	sort1a	0.002661298	-0.095510148	-0.060577332	-0.362899297	-0.357946523	downregulated during regeneration - late
ENSDART00000159908	camk1db	0.001174125	-0.548571048	-0.478436516	-0.873630088	-0.641909911	downregulated during regeneration - late
ENSDART00000160305	si:zfos-943e10.1	4.9299E-05	-0.732433247	-0.018482138	-0.2347001	-0.520119378	downregulated during regeneration - late

ENSDART00000160425	LAMP1	0.002170917	-0.291224845	-0.711008477	-0.768820578	-0.726430297	downregulated during regeneration - late
ENSDART00000160431	dhrs13l1	0.026954719	-0.306310555	-0.206975564	-0.316329966	-0.293060295	downregulated during regeneration - late
ENSDART00000160707	smx5	0.001677265	-0.006041688	-0.141079427	-0.427667941	-0.488718653	downregulated during regeneration - late
ENSDART00000160829	mhc1zda	0.014447102	-0.410179214	-0.276613879	-0.418038837	-0.322538004	downregulated during regeneration - late
ENSDART00000160955	slc38a3b	0.012986721	-0.475545538	-0.367921084	-0.471714594	-0.850494481	downregulated during regeneration - late
ENSDART00000160956	oaz1b	0.012598032	-0.133855172	-0.165932835	-0.380106363	-0.375655267	downregulated during regeneration - late
ENSDART00000161525	zgc:171704	0.000191101	0.267822787	-0.368878081	-1.0813007	-1.211102226	downregulated during regeneration - late
ENSDART00000161932	npr3	0.00058454	0.255355781	0.183557252	-0.357151542	-1.063769364	downregulated during regeneration - late
ENSDART00000162010	zgc:92140	0.011458427	-0.256831407	-0.245054913	-0.345912399	-0.346929099	downregulated during regeneration - late
ENSDART00000162359	sorbs1	0.007620644	-0.104497249	-0.20837462	-0.418151878	-0.317718588	downregulated during regeneration - late
ENSDART00000162564	CT027980.1	0.000781831	-0.351165538	-0.178285414	-0.533647461	-0.500417084	downregulated during regeneration - late
ENSDART00000162587	chd5	0.003143898	-0.534129477	-1.024077887	-0.70517204	-0.958580386	downregulated during regeneration - late
ENSDART00000163305	fxyd6l	0.013876441	-0.209100518	-0.188713869	-0.620315584	-0.44198331	downregulated during regeneration - late
ENSDART00000163394	dgke	0.019521843	-0.154264184	-0.257555682	-0.346312178	-0.3002364	downregulated during regeneration - late
ENSDART00000163565	plcl2	0.005353377	-0.007453137	-0.119106713	-0.251934099	-0.322673112	downregulated during regeneration - late
ENSDART00000164729	SBSPON	0.014479917	-0.213183167	-0.151008123	-0.380923154	-0.573582751	downregulated during regeneration - late
ENSDART00000164791	fkbp3	0.030292962	-0.300438451	-0.231059822	-0.287267966	-0.464270989	downregulated during regeneration - late
ENSDART00000165290	cyb5a	0.002005251	0.19405994	0.064877953	-0.350823438	-0.576368917	downregulated during regeneration - late
ENSDART00000165570	rgs3a	0.01962183	-0.252183908	-0.25277418	-0.390798302	-0.347427277	downregulated during regeneration - late
ENSDART00000166105	frem1a	0.02305984	-0.140882698	-0.093532592	-0.525495905	-1.018483252	downregulated during regeneration - late
ENSDART00000166449	pik3r3a	0.049250284	-0.698577911	-0.3007013	-0.248394691	-0.547713549	downregulated during regeneration - late
ENSDART00000166527	ftr87	0.023542744	-0.180960216	-0.104077306	-0.471569991	-0.343688512	downregulated during regeneration - late
ENSDART00000166829	NA	0.030198325	-0.799969413	-0.359199835	-0.712167572	-2.039500454	downregulated during regeneration - late
ENSDART00000167330	CU179758.1	0.003900528	-0.385159517	-0.273959547	-0.55992419	-0.56237529	downregulated during regeneration - late
ENSDART00000167506	scg2a	0.003405768	-0.457333464	-0.29124415	-0.144155502	-0.510044484	downregulated during regeneration - late
ENSDART00000167824	timp4.3	0.01037926	-0.389539203	-0.013931581	-0.399789503	-0.590224284	downregulated during regeneration - late
ENSDART00000167963	nrg1	0.037106045	-0.278608256	-0.388339035	-0.420420553	-0.389329729	downregulated during regeneration - late
ENSDART00000168201	golim4a	0.009167687	-0.134763119	-0.005515007	-0.245821925	-1.172515705	downregulated during regeneration - late
ENSDART00000168228	tmem184a	0.016284951	-0.3188994	-0.465602708	-0.713259634	-0.580015552	downregulated during regeneration - late
ENSDART00000168278	si:ch211-276i12.4	0.002327436	-0.631335043	-0.270754342	-0.641142033	-0.623314164	downregulated during regeneration - late
ENSDART00000168750	smarca1	0.005353377	-0.162085202	-0.116464254	-0.382463649	-0.380530416	downregulated during regeneration - late
ENSDART00000169228	vat1l	0.00721984	0.089392965	-0.09841303	-0.250056266	-0.442720544	downregulated during regeneration - late
ENSDART00000169380	slc25a43	0.005026727	-0.226077688	0.088930874	-0.355915914	-0.508574161	downregulated during regeneration - late
ENSDART00000170381	CR855389.1	0.046578416	-0.538290002	-0.347216121	-0.566215377	-0.407287476	downregulated during regeneration - late
ENSDART00000171006	hpcal4	0.014069453	-0.435402713	-0.198074705	-0.466665783	-0.338540363	downregulated during regeneration - late
ENSDART00000171417	si:ch73-299h12.3	0.004952764	-0.719381589	-0.760232748	-0.826233659	-0.863901262	downregulated during regeneration - late
ENSDART00000171815	abcc8b	0.014959825	0.00284442	-0.044162256	-0.758748776	-0.514944979	downregulated during regeneration - late
ENSDART00000171824	si:ch211-227e10.2	0.024734469	-0.663768919	-0.251157559	-0.496668883	-0.404241953	downregulated during regeneration - late
ENSDART00000172019	CU694219.1	0.010271025	-0.358471879	-0.088786066	-0.423165049	-1.570028426	downregulated during regeneration - late
ENSDART00000172357	tmem132a	0.051805764	-0.246648072	-0.102389273	-0.224742481	-0.519178406	downregulated during regeneration - late
ENSDART00000172672	angptl2b	0.010046682	-0.321678324	-0.001662718	-0.330394771	-0.841768465	downregulated during regeneration - late
ENSDART00000173237	mid2	0.032645202	-0.115282743	-0.310001722	-0.367171215	-0.341782639	downregulated during regeneration - late

Table S3. Ingenuity Pathway Analysis of temporally clustered, differentially expressed genes.

Ingenuity Canonical Pathways	-log(p-value	Ratio	cluster	genes	collapsed pathways
EIF2 Signaling	23.8	0.163	growth toward the midline	RPL36A,RPS27,RPS23,RPS18,RPL39,Rpl22l1,RPL35A,RPS4X,RPL7,RPS11,RPS28,RPS7,RPL35,MAP2K2,RPS13,UBA52,RPS9,MRAS,RPL21,RPS17,RPL18,RPS24,RPL4,RPL30,RPL12,RPL23,RPS21,RPL10A,RPL27,RPL15,RPL8,PIK3CB,RPS15A,RPS25,RPL38,RPSA	EIF2 Signaling
mTOR Signaling	12	0.114	growth toward the midline	PRKCQ,PLD3,RPS27,RHOC,RPS23,RPS18,RPS21,RPS4X,RPS11,RPS28,RPS7,HMOX1,RHOG,RPS13,PRKCD,RPS9,MRAS,RPS25,RPS15A,PIK3CB,RPS17,RPS24,RPSA	mTOR Signaling
Regulation of eIF4 and p70S6K Signaling	9.52	0.115	growth toward the midline	RPS27,RPS23,RPS18,RPS21,RPS4X,RPS11,RPS28,RPS7,MAP2K2,RPS13,RPS9,MRAS,RPS25,PIK3CB,RPS15A,RPS17,RPS24,RPSA	mTOR Signaling
Phagosome Maturation	6.48	0.0946	growth toward the midline	TUBB4B,TCIRG1,ATP6AP1,CTSZ,CTSV,CTSA,ATP6V1H,TUBA8,NCF2,CTSB,CTSC,NAPA,BET1L,ATP6V0E1	Phagosome Maturation
IL-8 Signaling	5.69	0.0761	growth toward the midline	PRKCQ,PLD3,RHOC,CSTB,BCL2L1,HMOX1,ITGAM,RHOG,MAP2K2,PRKCD,NCF2,ITGAV,MRAS,PIK3CB,MMP9	IL-8 Signaling
IL-3 Signaling	4	0.0964	growth toward the midline	JAK1,PRKCQ,MAP2K2,PRKCD,MRAS,PIK3CB,STAT3,INPP5D	IL-3 Signaling
EIF2 Signaling	9.84	0.0995	midline crossing	EIF2AK1,ATF3,EIF3H,RRAS,HRAS,TRIB3,EIF2S3,EIF2S1,EIF2S2,RPL9,EIF3M,EIF3F,RRAS2,EIF3B,EIF4A1,EIF3I,ATF4,RPL5,AGO1,RPS12,ACTC1,EIF3L	EIF2 Signaling
Regulation of eIF4 and p70S6K Signaling	9.15	0.115	midline crossing	EIF3H,RRAS,HRAS,EIF2S3,EIF2S1,MAPK11,EIF2S2,EIF4EBP1,EIF3M,EIF3F,RRAS2,EIF3B,EIF4A1,MKNK1,EIF3I,RPS12,AGO1,EIF3L	mTOR Signaling
Germ Cell-Sertoli Cell Junction Signaling	7.65	0.0983	midline crossing	SRC,RRAS,TUBB4B,RAC1,ITGA6,ILK,TUBA4A,MAPK9,HRAS,GSN,LIMK1,RRAS2,TUBA3E,JUP,ACTC1,ACTG1,CTNND1	RAS/MAPK signaling
Agrin Interactions at Neuromuscular Junction	7.31	0.159	midline crossing	SRC,RRAS2,JUN,RRAS,RAC1,ITGA6,MAPK9,HRAS,ACTC1,ACTG1,ITGB3	agrin interactions
Virus Entry via Endocytic Pathways	6.93	0.118	midline crossing	SRC,AP2M1,RRAS,HLA-B,ITGA6,ABL1,RAC1,HRAS,ITGB3,RRAS2,FLNC,ACTC1,ACTG1	endocytic pathways
Integrin Signaling	6.87	0.0822	midline crossing	RAP2B,RAPGEF1,SRC,RRAS,ARF1,RAC1,ITGA6,ILK,ABL1,HRAS,TNK2,GSN,ITGB3,CAPNS1,RRAS2,ACTC1,ACTG1,MYL12A	integrin signaling
Epithelial Adherens Junction Signaling	6.28	0.0959	midline crossing	SRC,RAPGEF1,MYL6,RRAS,TUBB4B,RAC1,TUBA4A,HRAS,RRAS2,JUP,TUBA3E,ACTC1,ACTG1,CTNND1	adhesive junctions
Sertoli Cell-Sertoli Cell Junction Signaling	5.96	0.0843	midline crossing	SRC,RRAS,TUBB4B,RAC1,ILK,TUBA4A,MAPK9,HRAS,MAPK11,JUN,RRAS2,JUP,TUBA3E,ACTC1,ACTG1	adhesive junctions
Paxillin Signaling	5.93	0.106	midline crossing	SRC,RRAS2,RRAS,ARF1,RAC1,ITGA6,MAPK9,HRAS,MAPK11,ACTC1,ACTG1,ITGB3	adhesive junctions
Estrogen-Dependent Breast Cancer Signaling	5.69	0.125	midline crossing	SRC,RRAS2,JUN,RRAS,CREB3,DHRS11,HSD17B7,HRAS,ATF4,STAT5B	RAS/MAPK signaling
Molecular Mechanisms of Cancer	5.36	0.0558	midline crossing	RAP2B,RAPGEF1,SRC,ARHGEF4,RRAS,RAC1,APAF1,ABL1,MAPK9,HRAS,BAX,E2F3,MAPK11,FAS,RB1,JUN,RRAS2,CASP9,NFKBIA,ARHGEF2,SMAD1,CTNND1	RAS/MAPK signaling
Remodeling of Epithelial Adherens Junctions	5.33	0.13	midline crossing	SRC,NME1,TUBB4B,MAPRE1,TUBA4A,TUBA3E,ACTC1,ACTG1,CTNND1	adhesive junctions
Prostate Cancer Signaling	4.93	0.103	midline crossing	RB1,NFKBIA,CASP9,RRAS2,HSP90AB1,RRAS,CREB3,ABL1,HRAS,ATF4	RAS/MAPK signaling
UVC-Induced MAPK Signaling	4.91	0.163	midline crossing	SRC,RRAS2,JUN,RRAS,MAPK9,HRAS,MAPK11	RAS/MAPK signaling
NRF2-mediated Oxidative Stress Response	4.85	0.0725	midline crossing	USP14,RRAS,PRDX1,MAPK9,HRAS,DNAJC21,JUN,RRAS2,VCP,ATF4,TXN,FKBP5,ACTC1,ACTG1	NRF2-mediated Oxidative Stress Response
Role of Tissue Factor in Cancer	4.73	0.0887	midline crossing	SRC,RRAS2,RRAS,RAC1,ITGA6,HRAS,STAT5B,MAPK11,F3,ITGB3,LIMK1	RAS/MAPK signaling
mTOR Signaling	4.65	0.0697	midline crossing	EIF3H,RRAS,RAC1,FKBP1A,HRAS,EIF4EBP1,EIF3M,EIF3F,RRAS2,EIF3B,EIF4A1,EIF3I,RPS12,EIF3L	mTOR Signaling
Apoptosis Signaling	4.38	0.1	midline crossing	CAPNS1,NFKBIA,CASP9,RRAS2,RRAS,APAF1,HRAS,BAX,FAS	apoptosis signaling
Myc Mediated Apoptosis Signaling	4.38	0.114	midline crossing	CASP9,RRAS2,RRAS,APAF1,MAPK9,HRAS,BAX,FAS	apoptosis signaling
Caveolar-mediated Endocytosis Signaling	4.33	0.113	midline crossing	SRC,FLNC,HLA-B,ITGA6,ABL1,ACTC1,ACTG1,ITGB3	endocytic pathways
Death Receptor Signaling	4.27	0.0968	midline crossing	NFKBIA,CASP9,APAF1,PARP8,PARP12,ACTC1,ACTG1,FAS,LIMK1	apoptosis signaling
tRNA Charging	4.14	0.154	midline crossing	RARS,GARS,TARS,SARS,IARS,QARS	tRNA Charging
ILK Signaling	4.12	0.066	midline crossing	FN1,MYL6,FERMT2,CREB3,ILK,MAPK9,ITGB3,JUN,FLNC,ATF4,RSU1,ACTC1,ACTG1	integrin signaling
ATM Signaling	4.09	0.0918	midline crossing	NFKBIA,GADD45B,JUN,CREB3,ABL1,MAPK9,ATF4,TDP1,MAPK11	ATM Signaling
Superpathway of Cholesterol Biosynthesis	13.1	0.393	target selection	MVD,SQLE,EBP,ACAT2,DHCR24,HSD17B7,MSMO1,TM7SF2,HMGCS1,SC5D,CYP51A1	cholesterol biosynthesis
Cholesterol Biosynthesis I	11.7	0.615	target selection	SQLE,EBP,DHCR24,HSD17B7,MSMO1,TM7SF2,SC5D,CYP51A1	cholesterol biosynthesis
Cholesterol Biosynthesis II (via 24,25-dihydrolanosterol)	11.7	0.615	target selection	SQLE,EBP,DHCR24,HSD17B7,MSMO1,TM7SF2,SC5D,CYP51A1	cholesterol biosynthesis
Cholesterol Biosynthesis III (via Desmosterol)	11.7	0.615	target selection	SQLE,EBP,DHCR24,HSD17B7,MSMO1,TM7SF2,SC5D,CYP51A1	cholesterol biosynthesis
Actin Cytoskeleton Signaling	9.06	0.0837	target selection	MYH4,FN1,SOS2,HRAS,FGD1,MYH7,BCAR1,RAC3,SSH1,APC,PIK3R3,SHC1,PAK1,CYFIP2,FGF18,PAK2,ARHGAP35,ACTC1,MYL3	actin cytoskeleton signaling
Breast Cancer Regulation by Stathmin1	8.94	0.0878	target selection	TUBA1B,PPP2R5B,SOS2,TUBB2A,TUBA4A,HRAS,TUBB,PPP1R14B,TUBB2B,PIK3R3,SHC1,PPP2CB,PAK1,CAMK2D,TUBA1A,GNB2,TUBA1C,PPP2R1B	axon guidance
Axonal Guidance Signaling	8.59	0.0574	target selection	TUBA1B,PLXNA3,NRP2,BMP3,SOS2,TUBB2A,TUBA4A,ADAM22,HRAS,L1CAM,DPYSL5,TUBB,BCAR1,RAC3,TUBB2B,PIK3R3,SHC1,PAK1,EPHB1,TUBA1A,PAK2,GNB2,SRGAP2,TUBA1C,MYL3,NRP1	axon guidance
Epithelial Adherens Junction Signaling	7.57	0.0959	target selection	MYH4,TUBA1B,TUBB2A,TUBA4A,HRAS,MYH7,TUBB,APC,TUBB2B,TUBA1A,TGFB2,TUBA1C,ACTC1,MYL3	adhesive junctions
Germ Cell-Sertoli Cell Junction Signaling	7.47	0.0867	target selection	TUBA1B,TUBB2A,TUBA4A,HRAS,TUBB,RAC3,BCAR1,TUBB2B,PIK3R3,PAK1,TUBA1A,PAK2,TGFB2,TUBA1C,ACTC1	adhesive junctions
Remodeling of Epithelial Adherens Junctions	7.27	0.145	target selection	TUBA1B,TUBA1A,TUBB2A,TUBA4A,TUBA1C,TUBB,ACTC1,APC,TUBB2B,Mapre2	adhesive junctions
14-3-3-mediated Signaling	6.35	0.0916	target selection	PIK3R3,TUBA1B,CBL,TUBA1A,YWHAH,TUBB2A,TUBA4A,HRAS,VIM,TUBA1C,TUBB,TUBB2B	14-3-3-mediated signaling
Zymosterol Biosynthesis	6.21	0.667	target selection	HSD17B7,MSMO1,TM7SF2,CYP51A1	cholesterol biosynthesis
ERK/MAPK Signaling	5.88	0.07	target selection	YWHAH,PPP2R5B,SOS2,HRAS,PPP1R14B,RAC3,BCAR1,PIK3R3,SHC1,PPP2CB,PAK1,PAK2,STAT1,PPP2R1B	ERK/MAPK signaling
Gap Junction Signaling	5.27	0.0667	target selection	PIK3R3,CSNK1E,TUBA1B,DBN1,TUBA1A,TUBB2A,SOS2,TUBA4A,HRAS,TUBA1C,TUBB,ACTC1,TUBB2B	gap junction signaling
Phagosome Maturation	4.99	0.0743	target selection	DYNC1H1,TUBA1B,Dync1i2,TUBA1A,VPS33A,DYNC1I1,TUBB2A,TUBA4A,TUBA1C,TUBB,TUBB2B	phagasome maturation
PI3K/AKT Signaling	4.86	0.08	target selection	PIK3R3,SHC1,RPS6KB1,PPP2CB,NFKBIA,YWHAH,SOS2,PPP2R5B,HRAS,PPP2R1B	PI3/AKT signaling
PTEN Signaling	4.24	0.0756	target selection	PIK3R3,SHC1,RPS6KB1,CBL,YWHAH,SOS2,HRAS,BCAR1,RAC3	PTEN signaling
Integrin Signaling	4.07	0.0548	target selection	PIK3R3,SHC1,PAK1,ARF3,PAK2,SOS2,RALB,HRAS,ITGA10,BCAR1,ACTC1,RAC3	integrin signaling
Ephrin B Signaling	4.06	0.0959	target selection	EPHB1,PAK1,CBL,ABI1,GNB2,HRAS,RAC3	ephrin B signaling
Glutamate Receptor Signaling	12.2	0.263	brain innervation	GRIN1,GRM2,GRIN2A,GRM8,GRM1,SLC1A1,GRIP1,HOMER3,GRM4,GNB1,GRM6,SLC1A2,HOMER1,GRIA3,GRIK1	glutamate receptor signaling
Synaptic Long Term Depression	8.98	0.115	brain innervation	GRM2,CACNA2D2,GUCY1A1,GRM8,GRM1,RYR2,CACNA1H,CACNB3,GRM4,GNAZ,CACNG3,IGF1,GNAO1,PPM1L,RYR3,GRM6,PRKCE,PPP2R2C,CACNB2,GRIA3	synaptic LTD
Calcium Signaling	8.44	0.102	brain innervation	RAP2A,GRIN1,GRIN2A,CACNA2D2,CHRNA6,RYR2,MEF2A,SLC8A3,CACNA1H,SLC8A2,CACNB3,ATP2B2,CACNG3,ATP2B3,RYR3,CACNB2,CHRNB3,SLC8A1,PPP3CA,GRIA3,GRIK1	calcium signaling
CREB Signaling in Neurons	8.22	0.0991	brain innervation	GRIN1,ADCY2,GRIN2A,GRM2,CACNA2D2,GRM8,GRM1,CACNA1H,GNG13,CACNB3,GRM4,GNAZ,PIK3R3,GNB1,CACNG3,GNAO1,GRM6,PRKCE,CACNB2,GRIK1,GRIA3	CREB signaling
GABA Receptor Signaling	7.85	0.147	brain innervation	GABRA5,ADCY2,CACNA2D2,CACNA1H,GABBR1,GPHN,CACNB3,CACNG3,DNM1,NSF,GABBR2,GABRB1,CACNB2,GABRA2	GABA receptor signaling
Opioid Signaling Pathway	6.52	0.0823	brain innervation	GRIN1,ADCY2,GRIN2A,CACNA2D2,RYR2,RGS7,CACNA1H,CACNB3,RGS6,RAC3,SLC12A5,GNB1,CACNG3,ARRB1,GNAO1,RYR3,PRKCE,RGS11,CACNB2,PPP3CA	opiod signaling
Axonal Guidance Signaling	6.25	0.0618	brain innervation	EPHB2,GNG13,SEMA6B,ADAM11,PLXNA2,GNB1,EPHB6,NTNG1,IGF1,NTNG2,UNC5D,PRKCE,ADAM23,TUBB4A,ROBO2,PPP3CA,EFNA2,SLIT2,GNAZ,ROBO3,RAC3,PIK3R3,SEMA3A,EPHA6,ADAM12,WNT10A,GNAO1,LINGO1	axon guidance
Netrin Signaling	5.96	0.154	brain innervation	CACNG3,CACNA2D2,RYR2,RYR3,CACNA1H,UNC5D,CACNB2,CACNB3,RAC3,PPP3CA	netrin signaling
Role of NFAT in Cardiac Hypertrophy	4.69	0.0731	brain innervation	ADCY2,CACNA2D2,MEF2A,CACNA1H,SLC8A3,GNG13,SLC8A2,CACNB3,PIK3R3,GNB1,CACNG3,IGF1,PRKCE,CACNB2,SLC8A1,PPP3CA	NFAT signaling
nNOS Signaling in Skeletal Muscle Cells	4.64	0.171	brain innervation	CACNG3,CACNA2D2,RYR2,RYR3,CACNA1H,CACNB2,CACNB3	nNOS signaling
Chondroitin Sulfate Biosynthesis	4.57	0.14	brain innervation	CHST1,SULT4A1,XYLT1,HS3ST2,B3GAT1,HS6ST3,HS3ST4,B3GAT2	glycosaminoglycan biosynthesis
G Beta Gamma Signaling	4.48	0.0965	brain innervation	CACNG3,GNB1,ADCY2,CACNA2D2,GNAO1,CACNA1H,GNG13,PRKCE,CACNB2,CACNB3,GNAZ	GPCR signaling
Dermatan Sulfate Biosynthesis	4.46	0.136	brain innervation	CHST1,SULT4A1,XYLT1,HS3ST2,B3GAT1,HS6ST3,HS3ST4,B3GAT2	glycosaminoglycan biosynthesis
Gαi Signaling	4.28	0.0917	brain innervation	GNB1,GABBR2,ADCY2,GRM2,ADRA2A,GRM8,RGS7,GRM6,GNG13,GABBR1,GRM4	GPCR signaling
α-Adrenergic Signaling	4	0.103	brain innervation	GNB1,ADCY2,GYS1,ADRA2A,SLC8A3,GNG13,PRKCE,SLC8A2,SLC8A1	adrenergic signaling

GABA Receptor Signaling	17.8	0.242	downregulated during regeneration - early	CACNA1G,CACNA1I,CACNG6,SLC32A1,AP2A1,CACNA1D,ADCY3,GABBR1,GABRB2,CACNG2,GAD2,GABRG2,GPR37,GABRG1,CACNA1B,GABRA6,SLC6A1,CACNG7,CACNB2,GABRA1,CACNG8,GABRD,CACNA2D4	GABA receptor signaling
Opioid Signaling Pathway	13.6	0.119	downregulated during regeneration - early	CACNA1I,AP2A1,CAMK4,SLC12A5,CACNG2,GNB3,CAMK2A,PPP3R1,CACNG7,CACNB2,CACNG8,CACNA2D4,PRKD1,PPP3CA,CACNA1G,GRIN1,CACNG6,CACNA1D,ADCY3,RPS6KA5,NPBWR2,FOSB,CACNA1B,KCNJ5,KCNJ9,PENK,RGS8,KCNJ6,CAMK2G	opiod signaling
Calcium Signaling	12.8	0.126	downregulated during regeneration - early	CACNA1I,CAMK4,ATP2B1,GRIA1,CACNG2,CAMK2A,PPP3R1,CACNG7,CACNB2,CACNG8,CACNA2D4,PPP3CA,CACNA1G,CACNG6,GRIN1,CACNA1D,TRPC1,CHRNA10,CACNA1B,ATP2B3,MEF2D,CAMKK1,RCAN3,SLC8A1,GRIA3,CAMK2G	calcium signaling
Role of NFAT in Cardiac Hypertrophy	10.6	0.11	downregulated during regeneration - early	CACNA1G,CACNA1I,CACNG6,CACNA1D,CAMK4,ADCY3,PLCH1,CACNG2,CAMK2A,GNB3,CACNA1B,MEF2D,PPP3R1,IGF1R,CACNG7,RCAN3,IRS2,CACNB2,CACNG8,SLC8A1,CACNA2D4,PRKD1,PPP3CA,CAMK2G	NFAT signaling
CREB Signaling in Neurons	10.1	0.108	downregulated during regeneration - early	CACNA1G,CACNA1I,CACNG6,GRIN1,CACNA1D,CAMK4,GRID2,GRIA1,ADCY3,PLCH1,CACNG2,GNB3,CAMK2A,GRIK4,CACNA1B,CACNG7,IRS2,CACNB2,CACNG8,CACNA2D4,PRKD1,CAMK2G,GRIA3	CREB signaling
nNOS Signaling in Skeletal Muscle Cells	9.36	0.268	downregulated during regeneration - early	CACNA1I,CACNA1G,CACNG2,CACNG6,CACNA1D,CAMK4,CACNA1B,CACNG7,CACNB2,CACNG8,CACNA2D4	nNOS signaling
Dopamine-DARPP32 Feedback in cAMP Signaling	8.9	0.116	downregulated during regeneration - early	KCNJ12,GRIN1,CACNA1D,CAMK4,PPP1R3C,ADCY3,DRD2,PLCH1,KCNJ11,DRD1,KCNJ14,KCNJ5,PPP3R1,CAMKK1,KCNJ9,DRD4,KCNJ6,PRKD1,PPP3CA	dopamine signaling
Corticotropin Releasing Hormone Signaling	8.39	0.122	downregulated during regeneration - early	CACNA1G,CACNA1I,CACNG6,CACNA1D,CAMK4,GUCY2D,ADCY3,CRH,CACNG2,CACNA1B,MEF2D,NR4A1,CACNG7,CACNB2,CACNG8,CACNA2D4,PRKD1	corticotropin-releasing hormone signaling
Netrin Signaling	8.14	0.185	downregulated during regeneration - early	CACNA1I,CACNA1G,CACNG2,CACNG6,CACNA1D,CACNA1B,PPP3R1,CACNG7,CACNB2,CACNG8,CACNA2D4,PPP3CA	netrin signaling
G Beta Gamma Signaling	7.91	0.132	downregulated during regeneration - early	CACNA1I,CACNA1G,CACNG6,CACNA1D,CACNG2,GNB3,CACNA1B,KCNJ5,KCNJ9,CACNG7,CACNB2,KCNJ6,CACNG8,CACNA2D4,PRKD1	GPCR signaling
Synaptic Long Term Depression	7.68	0.103	downregulated during regeneration - early	CACNA1G,CACNA1I,CACNG6,CACNA1D,GUCY2D,GRID2,GRIA1,CRH,PLCH1,CACNG2,CACNA1B,IGF1R,CACNG7,CACNB2,CACNG8,CACNA2D4,PRKD1,GRIA3	synaptic LTD
GNRH Signaling	7.26	0.103	downregulated during regeneration - early	CACNA1G,CACNA1I,CACNG6,CACNA1D,CAMK4,MAP3K13,ADCY3,CACNG2,GNB3,CAMK2A,CACNA1B,CACNG7,CACNB2,CACNG8,CACNA2D4,PRKD1,CAMK2G	GNRH signaling
CCR5 Signaling in Macrophages	7.14	0.137	downregulated during regeneration - early	CACNA1I,CACNA1G,CACNG6,CACNA1D,CAMK4,CACNG2,GNB3,CACNA1B,CACNG7,CACNB2,CACNG8,CACNA2D4,PRKD1	CCRG signaling
GPCR-Mediated Nutrient Sensing in Enteroendocrine Cells	7.14	0.125	downregulated during regeneration - early	CACNA1I,CACNA1G,CACNG6,CACNA1D,ADCY3,RAPGEF4,PLCH1,CACNG2,CACNA1B,CACNG7,CACNB2,CACNG8,CACNA2D4,PRKD1	GPCR signaling
PKCθ Signaling in T Lymphocytes	6.73	0.101	downregulated during regeneration - early	CACNA1G,CACNA1I,CACNG6,CACNA1D,MAP3K13,CACNG2,CAMK2A,CACNA1B,PPP3R1,CACNG7,IRS2,CACNB2,CACNG8,CACNA2D4,PPP3CA,CAMK2G	
FcγRIIB Signaling in B Lymphocytes	6.21	0.139	downregulated during regeneration - early	CACNA1I,CACNA1G,CACNG2,CACNG6,CACNA1D,CACNA1B,CACNG7,CACNB2,IRS2,CACNG8,CACNA2D4	
Type II Diabetes Mellitus Signaling	5.44	0.0909	downregulated during regeneration - early	CACNA1G,CACNG2,CACNA1I,CACNG6,CACNA1D,PRKAB1,CACNA1B,CACNG7,CACNB2,IRS2,CACNG8,CACNA2D4,PRKD1,KCNJ11	
Androgen Signaling	5.29	0.0949	downregulated during regeneration - early	CACNA1I,CACNA1G,CACNG6,CACNA1D,CAMK4,CACNG2,GNB3,CACNA1B,CACNG7,CACNB2,CACNG8,CACNA2D4,PRKD1	
Gap Junction Signaling	4.89	0.0769	downregulated during regeneration - early	TJP2,GUCY2D,GRIA1,ADCY3,GJA9,DRD2,GJC1,PLCH1,LPAR1,DRD1,PPP3R1,IRS2,PRKD1,PPP3CA,GRIA3	gap junction signaling
Glutamate Receptor Signaling	4.7	0.14	downregulated during regeneration - early	GRIN1,CAMK4,GNB3,GRIK4,SLC17A7,GRID2,GRIA1,GRIA3	glutamate receptor signaling
Synaptic Long Term Potentiation	4.36	0.0902	downregulated during regeneration - early	GRIN1,CAMK2A,CAMK4,GRIA1,PPP1R3C,PPP3R1,PLCH1,PPP3CA,PRKD1,CAMK2G,GRIA3	synaptic LTD
cAMP-mediated signaling	4.1	0.0658	downregulated during regeneration - early	CAMK4,VIPR2,CHRM4,ADCY3,GABBR1,RAPGEF4,DRD2,CHRM5,CAMK2A,LPAR1,DRD1,PPP3R1,DRD4,PPP3CA,CAMK2G	cAMP-mediated signaling
Gustation Pathway	4.08	0.0779	downregulated during regeneration - early	CACNA1I,CACNA1G,CACNG2,CACNG6,CACNA1D,GNB3,CACNA1B,ADCY3,CACNG7,CACNB2,CACNG8,CACNA2D4	
Cellular Effects of Sildenafil (Viagra)	4.08	0.084	downregulated during regeneration - early	SLC4A5,CACNG2,CACNG6,CACNA1D,CAMK4,GPR37,GUCY2D,ADCY3,CACNG7,CACNG8,PLCH1	
Neuroinflammation Signaling Pathway	4.08	0.0579	downregulated during regeneration - early	GRIN1,GRIA1,GABBR1,GABRB2,GAD2,GABRG2,GABRG1,KCNJ5,PPP3R1,GABRA6,KCNJ9,SLC6A1,TLR7,IRS2,KCNJ6,GABRA1,GABRD,PPP3CA	neuroinflammation signaling
Phototransduction Pathway	4.74	0.132	downregulated during regeneration - late	PRKACB,GNB5,GUCY2F,SAG,RGS9BP,GNGT2,PDE6D	phototransduction

Table S4. Differentially accesible sequences with distance to nearest annotated genes and nearest differentially-expressed genes.

new_seqnames	chr	start	end	width	overall_distance	overall_gene	overall_DE_distance	overall_DE_gene	timepoint
**chr1:27509-28008**	chr1	27509	28008	500	70	eed	15791	cep97	differentially open at 2dpi
**chr1:185007-185506**	chr1	185007	185506	500	11536	grtp1a	-79577	cenpe	differentially open at 2dpi
**chr1:58446298-58446797**	chr1	58446298	58446797	500	-7231	lpar2b	133113	mfap4	differentially open at 2dpi
**chr1:58479677-58480176**	chr1	58479677	58480176	500	-8	thumpd1	166491	mfap4	differentially open at 2dpi
**chr1:58542861-58543360**	chr1	58542861	58543360	500	1938	olfm2b	229675	mfap4	differentially open at 2dpi
**chr1:58608084-58608583**	chr1	58608084	58608583	500	104	parn	294899	mfap4	differentially open at 2dpi
**chr2:51352621-51353120**	chr2	51352621	51353120	500	2	ftr67	698402	dad1	differentially open at 2dpi
**chr2:52763439-52764389**	chr2	52763439	52764389	951	28200	tle2c	-336627	scn12aa	differentially open at 2dpi
**chr2:54965275-54965774**	chr2	54965275	54965774	500	-970	ankrd12	-56216	acss2l	differentially open at 2dpi
**chr2:56491466-56491965**	chr2	56491466	56491965	500	40208	gpx4b	40208	gpx4b	differentially open at 2dpi
**chr3:52202-52701**	chr3	52202	52701	500	-30528	mhc1zfa	-3902825	plbd1	differentially open at 2dpi
**chr3:1454919-1455418**	chr3	1454919	1455418	500	-7	wbp2nl	-2500109	plbd1	differentially open at 2dpi
**chr3:1463472-1463971**	chr3	1463472	1463971	500	8547	wbp2nl	-2491555	plbd1	differentially open at 2dpi
**chr3:4122604-4123103**	chr3	4122604	4123103	500	128600	art4	167577	plbd1	differentially open at 2dpi
**chr3:4123577-4124076**	chr3	4123577	4124076	500	129572	art4	168549	plbd1	differentially open at 2dpi
**chr3:5139540-5140039**	chr3	5139540	5140039	500	17995	tefb	227365	txn2	differentially open at 2dpi
**chr3:11625609-11626108**	chr3	11625609	11626108	500	32071	hlfa	-7.76E+05	ccnf	differentially open at 2dpi
**chr3:32328635-32329134**	chr3	32328635	32329134	500	-32035	trpm4a	49820	rras	differentially open at 2dpi
**chr3:36970639-36971138**	chr3	36970639	36971138	500	32	psmc3ip	673091	baxa	differentially open at 2dpi
**chr3:38598193-38598692**	chr3	38598193	38598692	500	175698	si:dkey-106c17.2	-8.28E+05	aldoaa	differentially open at 2dpi
**chr3:55222028-55222527**	chr3	55222028	55222527	500	-1291	si:dkey-188h10.3	-483963	rhbdf1a	differentially open at 2dpi
**chr3:55995907-55996406**	chr3	55995907	55996406	500	-40111	srl	-511125	rac1b	differentially open at 2dpi
**chr3:57474388-57474887**	chr3	57474388	57474887	500	-14839	cyth1a	-14839	cyth1a	differentially open at 2dpi
**chr3:59285313-59285812**	chr3	59285313	59285812	500	-106203	nptx1l	-106203	nptx1l	differentially open at 2dpi
**chr3:60369415-60369914**	chr3	60369415	60369914	500	-4502	anks4b	-4502	anks4b	differentially open at 2dpi
**chr3:61682115-61682614**	chr3	61682115	61682614	500	168342	srebf1	168342	srebf1	differentially open at 2dpi
**chr4:9945910-9946409**	chr4	9945910	9946409	500	35542	sbf1	35542	sbf1	differentially open at 2dpi
**chr4:68244163-68245059**	chr4	68244163	68245059	897	407814	znf1117	-40932543	fam19a5a	differentially open at 2dpi
**chr5:1090761-1091632**	chr5	1090761	1091632	872	11308	mbd2	-608799	dhx33	differentially open at 2dpi
**chr5:11919877-11920376**	chr5	11919877	11920376	500	-95059	zgc:171242	104502	ksr2	differentially open at 2dpi
**chr5:37564957-37565456**	chr5	37564957	37565456	500	-10354	gucy2d	79835	slc12a9	differentially open at 2dpi
**chr5:37904932-37905431**	chr5	37904932	37905431	500	1646	atp1b2b	-260141	slc12a9	differentially open at 2dpi
**chr5:50861109-50861608**	chr5	50861109	50861608	500	64942	rasgrf2b	323059	homer1b	differentially open at 2dpi
**chr5:52934307-52934806**	chr5	52934307	52934806	500	-354	taf6	1157357	ccnb1	differentially open at 2dpi
**chr5:53882442-53882941**	chr5	53882442	53882941	500	25298	ssna1	209221	ccnb1	differentially open at 2dpi
**chr5:64184151-64184650**	chr5	64184151	64184650	500	-43	si:ch211-236k19.4	8691	zgc:101858	differentially open at 2dpi
**chr5:66928807-66929306**	chr5	66928807	66929306	500	53220	zbtb20	-136024	si:dkey-251i10.2	differentially open at 2dpi
**chr5:71139273-71139772**	chr5	71139273	71139772	500	71080	fam78ab	-2869087	ank1a	differentially open at 2dpi
**chr5:71403458-71404338**	chr5	71403458	71404338	881	-6085	ddx54	-3133463	ank1a	differentially open at 2dpi
**chr6:327105-327604**	chr6	327105	327604	500	5604	pde6ha	92121	grap2b	differentially open at 2dpi
**chr6:526191-526690**	chr6	526191	526690	500	-34639	zgc:92360	-106965	grap2b	differentially open at 2dpi
**chr6:11900072-11900571**	chr6	11900072	11900571	500	129106	wdsub1	129106	wdsub1	differentially open at 2dpi
**chr6:25271538-25272037**	chr6	25271538	25272037	500	-1	kyat3	553745	barhl2	differentially open at 2dpi
**chr6:58980501-58981000**	chr6	58980501	58981000	500	26	mars	-210349	soat2	differentially open at 2dpi
**chr6:59759972-59760471**	chr6	59759972	59760471	500	-2546	zgc:65895	392387	slc32a1	differentially open at 2dpi
**chr6:59865579-59866078**	chr6	59865579	59866078	500	-34759	ddx3b	286781	slc32a1	differentially open at 2dpi
**chr7:5304709-5305208**	chr7	5304709	5305208	500	-51551	vangl2	-413506	ltb4r	differentially open at 2dpi
**chr7:6944553-6945429**	chr7	6944553	6945429	877	130125	actn3b	315381	pfdn2	differentially open at 2dpi
**chr7:7351882-7352381**	chr7	7351882	7352381	500	90394	nit1	-91760	pfdn2	differentially open at 2dpi
**chr7:10318592-10319091**	chr7	10318592	10319091	500	-1169	zfand6	1493865	mex3b	differentially open at 2dpi
**chr7:24647925-24648424**	chr7	24647925	24648424	500	37590	krcp	37590	krcp	differentially open at 2dpi
**chr7:40885503-40886002**	chr7	40885503	40886002	500	5600	scrib	173085	ncf2	differentially open at 2dpi
**chr7:69217869-69218368**	chr7	69217869	69218368	500	6310	ctdnep1b	65170	slc1a1	differentially open at 2dpi
**chr7:73160434-73160933**	chr7	73160434	73160933	500	-64881	cbln6	-1522829	cacnb2a	differentially open at 2dpi
**chr7:73948002-73948501**	chr7	73948002	73948501	500	-50472	cldnd1a	-2310397	cacnb2a	differentially open at 2dpi
**chr7:73951811-73952310**	chr7	73951811	73952310	500	-54280	cldnd1a	-2314205	cacnb2a	differentially open at 2dpi
**chr7:74078097-74078596**	chr7	74078097	74078596	500	8340	ppp1cbl	-2440491	cacnb2a	differentially open at 2dpi
**chr9:5470172-5470671**	chr9	5470172	5470671	500	19075	tnfsf13b	-1623689	si:dkey-4e7.3	differentially open at 2dpi
**chr9:44213691-44214190**	chr9	44213691	44214190	500	217825	ube2e3	-477399	znf385b	differentially open at 2dpi
**chr10:153604-154103**	chr10	153604	154103	500	14006	erg	-33955	ets2	differentially open at 2dpi
**chr10:384344-384843**	chr10	384344	384843	500	39623	zgc:171929	-173891	psmg1	differentially open at 2dpi
**chr10:443696-444195**	chr10	443696	444195	500	-72955	npffr1l3	-233243	psmg1	differentially open at 2dpi
**chr10:1799621-1800120**	chr10	1799621	1800120	500	2	epb41l4a	-1589167	psmg1	differentially open at 2dpi
**chr10:5135268-5135767**	chr10	5135268	5135767	500	-20	zgc:113274	-278421	nfil3	differentially open at 2dpi
**chr10:5211717-5212216**	chr10	5211717	5212216	500	-22074	sptlc1	-201973	nfil3	differentially open at 2dpi
**chr10:6381773-6382272**	chr10	6381773	6382272	500	-55	zgc:114200	-569140	il6st	differentially open at 2dpi
**chr10:13701874-13702373**	chr10	13701874	13702373	500	112604	cntfr	659549	lpar1	differentially open at 2dpi
**chr11:165810-166309**	chr11	165810	166309	500	-12993	zgc:172352	103162	slc11a2	differentially open at 2dpi
**chr11:12802692-12803447**	chr11	12802692	12803447	756	230	zgpat	291573	elovl1b	differentially open at 2dpi
**chr11:20623277-20623776**	chr11	20623277	20623776	500	230478	slc6a14	353656	cdh4	differentially open at 2dpi
**chr11:45076061-45076560**	chr11	45076061	45076560	500	17205	sos1	337578	tk1	differentially open at 2dpi
**chr12:3830775-3831274**	chr12	3830775	3831274	500	-3675	nif3l1	19675	cabp5a	differentially open at 2dpi
**chr12:16832439-16832938**	chr12	16832439	16832938	500	904	pank1b	246466	lipf	differentially open at 2dpi
**chr12:34189941-34190440**	chr12	34189941	34190440	500	17907	socs3b	144084	birc5a	differentially open at 2dpi
**chr12:38575744-38576243**	chr12	38575744	38576243	500	-57150	abca5	193924	rpl38	differentially open at 2dpi
**chr12:48551037-48551536**	chr12	48551037	48551536	500	11658	tex47	244390	lrrc20	differentially open at 2dpi
**chr13:1001301-1001800**	chr13	1001301	1001800	500	25	wdr92	-1782	pno1	differentially open at 2dpi

**chr13:1484577-1485076**	chr13	1484577	1485076	500	-206298	bag2	-206298	bag2	differentially open at 2dpi
**chr13:6989718-6990217**	chr13	6989718	6990217	500	30213	LOC795051	-933230	hells	differentially open at 2dpi
**chr13:51544130-51544629**	chr13	51544130	51544629	500	25604	klc1b	73078	lbh	differentially open at 2dpi
**chr13:51623489-51623988**	chr13	51623489	51623988	500	-6280	lbh	-6280	lbh	differentially open at 2dpi
**chr14:181543-182042**	chr14	181543	182042	500	21534	gpc2	31590	mcm7	differentially open at 2dpi
**chr14:24633380-24633879**	chr14	24633380	24633879	500	103294	pde6a	646233	cplx2	differentially open at 2dpi
**chr14:51841744-51842243**	chr14	51841744	51842243	500	-6172	rpl26	-13465	atp6v0e1	differentially open at 2dpi
**chr15:22879-23378**	chr15	22879	23378	500	-266	bace1	320244	limk1a	differentially open at 2dpi
**chr15:116690-117189**	chr15	116690	117189	500	-12598	cyp2y3	226432	limk1a	differentially open at 2dpi
**chr15:201627-202126**	chr15	201627	202126	500	-14330	LOC100002960	141496	limk1a	differentially open at 2dpi
**chr15:1569009-1569954**	chr15	1569009	1569954	946	-159	ift80	-234	smc4	differentially open at 2dpi
**chr15:20593110-20593609**	chr15	20593110	20593609	500	-220	tnfaip1	387086	usp2a	differentially open at 2dpi
**chr15:44093163-44093662**	chr15	44093163	44093662	500	23567	zgc:112998	63611	lrrc51	differentially open at 2dpi
**chr15:45801381-45801880**	chr15	45801381	45801880	500	22858	lrfn1	176107	sagb	differentially open at 2dpi
**chr15:47041964-47042463**	chr15	47041964	47042463	500	-16574	sik3	1416691	sagb	differentially open at 2dpi
**chr15:47515380-47515879**	chr15	47515380	47515879	500	-118293	cep57	1890107	sagb	differentially open at 2dpi
**chr16:1384691-1385190**	chr16	1384691	1385190	500	94195	sim1a	647278	irx1a	differentially open at 2dpi
**chr16:2582464-2582963**	chr16	2582464	2582963	500	-2223	acot22	-261727	adcy3a	differentially open at 2dpi
**chr16:20249721-20250220**	chr16	20249721	20250220	500	-17526	ahr1a	-165515	hdac9b	differentially open at 2dpi
**chr16:48595152-48595651**	chr16	48595152	48595651	500	76547	ext1a	-136770	eif3ha	differentially open at 2dpi
**chr16:54619065-54619564**	chr16	54619065	54619564	500	15369	pklr	1540191	trip13	differentially open at 2dpi
**chr17:487548-488047**	chr17	487548	488047	500	-29921	chrm5a	-372235	gchfr	differentially open at 2dpi
**chr17:1395288-1395787**	chr17	1395288	1395787	500	-6587	dio3a	113774	wars	differentially open at 2dpi
**chr17:8489676-8490175**	chr17	8489676	8490175	500	45616	ctbp2a	45616	ctbp2a	differentially open at 2dpi
**chr17:28689744-28690243**	chr17	28689744	28690243	500	-211	strn3	104643	g2e3	differentially open at 2dpi
**chr17:43711273-43711772**	chr17	43711273	43711772	500	38413	ahsa1b	-670253	npc2	differentially open at 2dpi
**chr17:51613348-51613847**	chr17	51613348	51613847	500	20204	nol10	478212	adi1	differentially open at 2dpi
**chr18:272145-272644**	chr18	272145	272644	500	-12073	larp6a	-746281	pkma	differentially open at 2dpi
**chr18:5548260-5548759**	chr18	5548260	5548759	500	16453	slc24a5	-89086	mibp2	differentially open at 2dpi
**chr18:6627792-6628291**	chr18	6627792	6628291	500	76073	fam168a	469717	utp15	differentially open at 2dpi
**chr18:7072291-7072790**	chr18	7072291	7072790	500	-110	calub	25219	utp15	differentially open at 2dpi
**chr18:8700770-8701269**	chr18	8700770	8701269	500	-79132	si:ch211-220f12.1	-712952	sema3ab	differentially open at 2dpi
**chr18:41520738-41521237**	chr18	41520738	41521237	500	-964	ccnl1a	-964	ccnl1a	differentially open at 2dpi
**chr18:50489493-50489992**	chr18	50489493	50489992	500	37722	cd276	37722	cd276	differentially open at 2dpi
**chr19:19931582-19932081**	chr19	19931582	19932081	500	-7732	jazf1a	590331	mpp6b	differentially open at 2dpi
**chr19:20512302-20512801**	chr19	20512302	20512801	500	9611	mpp6b	9611	mpp6b	differentially open at 2dpi
**chr19:25880479-25880978**	chr19	25880479	25880978	500	-112	rpa3	-193838	rims3	differentially open at 2dpi
**chr20:6486555-6487054**	chr20	6486555	6487054	500	-16897	mep1b	-1202574	ism2b	differentially open at 2dpi
**chr20:15652847-15653346**	chr20	15652847	15653346	500	-148	jun	-148	jun	differentially open at 2dpi
**chr20:15656516-15657393**	chr20	15656516	15657393	878	-3684	si:dkey-86e18.2	3710	jun	differentially open at 2dpi
**chr20:15687329-15687828**	chr20	15687329	15687828	500	-13254	faslg	34334	jun	differentially open at 2dpi
**chr20:17982452-17982951**	chr20	17982452	17982951	500	142181	cdh2	-281083	aqp4	differentially open at 2dpi
**chr20:20868444-20868943**	chr20	20868444	20868943	500	37340	ppp1r13bb	53391	ckbb	differentially open at 2dpi
**chr20:38079431-38079930**	chr20	38079431	38079930	500	-49304	angel2	-50019	galnt14	differentially open at 2dpi
**chr20:39316562-39317061**	chr20	39316562	39317061	500	-149	cyp39a1	-31215	rcan2	differentially open at 2dpi
**chr20:54830661-54831160**	chr20	54830661	54831160	500	-18186	ppp1r35	540969	rps29	differentially open at 2dpi
**chr21:697792-698291**	chr21	697792	698291	500	-838	oaz1b	395532	lhfpl2a	differentially open at 2dpi
**chr21:4375223-4375722**	chr21	4375223	4375722	500	-183	dolk	283687	lrrc8aa	differentially open at 2dpi
**chr21:11235062-11235561**	chr21	11235062	11235561	500	-110	rtkn2b	-128574	dnajc21	differentially open at 2dpi
**chr21:39416988-39417487**	chr21	39416988	39417487	500	-224	mntb	104537	dynll2b	differentially open at 2dpi
**chr22:20971838-20972337**	chr22	20971838	20972337	500	-46618	fkbp8	49628	ssbp4	differentially open at 2dpi
**chr22:37094885-37095384**	chr22	37094885	37095384	500	-109487	si:dkey-37m8.11	-601092	psmd1	differentially open at 2dpi
**chr23:1280885-1281384**	chr23	1280885	1281384	500	-145829	psmb11b	715726	ogfr	differentially open at 2dpi
**chr23:2174238-2174737**	chr23	2174238	2174737	500	-147271	prdm5	-307710	tcp1	differentially open at 2dpi
**chr23:14677630-14678129**	chr23	14677630	14678129	500	-30018	nkain4	216449	birc7	differentially open at 2dpi
**chr23:16765279-16766037**	chr23	16765279	16766037	759	296	fkbp1ab	-9815	sdcbp2	differentially open at 2dpi
**chr23:39321018-39321517**	chr23	39321018	39321517	500	26749	bmf1	-132751	src	differentially open at 2dpi
**chr23:45941247-45941746**	chr23	45941247	45941746	500	28838	ednrab	920679	atp1b2a	differentially open at 2dpi
**chr25:136191-136690**	chr25	136191	136690	500	-45643	zgc:114188	2772371	scamp2	differentially open at 2dpi
**chr25:222688-223187**	chr25	222688	223187	500	626	zgc:92481	2685873	scamp2	differentially open at 2dpi
**chr25:10696492-10696991**	chr25	10696492	10696991	500	37	ap3s2	246291	zgc:110339	differentially open at 2dpi
**chrUn_KN149790v1:100748-101247**	chrUn_KN149790v1	100748	101247	500	-6772	zgc:171422	NA	NA	differentially open at 2dpi
**chrUn_KN150361v1:39209-39708**	chrUn_KN150361v1	39209	39708	500	NA	NA	NA	NA	differentially open at 2dpi
**chrUn_KN150600v1:341-840**	chrUn_KN150600v1	341	840	500	NA	NA	NA	NA	differentially open at 2dpi
**chr2:15088215-15088714**	chr2	15088215	15088714	500	-100384	leprot	1076793	ebna1bp2	differentially closed at 2dpi
**chr5:61868726-61869225**	chr5	61868726	61869225	500	-57698	abr	-1.11E+06	ncf1	differentially closed at 2dpi
**chr7:5468715-5469214**	chr7	5468715	5469214	500	-103604	arhgef11	-577512	ltb4r	differentially closed at 2dpi
**chr20:15442099-15442598**	chr20	15442099	15442598	500	177367	prdx6	-210896	jun	differentially closed at 2dpi
**chr20:18383573-18384072**	chr20	18383573	18384072	500	30430	psma8	120037	aqp4	differentially closed at 2dpi
**chr21:24198964-24199463**	chr21	24198964	24199463	500	282640	cadm1a	282640	cadm1a	differentially closed at 2dpi
**chr22:17120757-17121529**	chr22	17120757	17121529	773	26763	frem1b	-460764	polr2eb	differentially closed at 2dpi
**chr22:30060403-30060902**	chr22	30060403	30060902	500	-79	si:dkey-286j15.1	-86975	dusp5	differentially closed at 2dpi
**chr24:16096606-16097105**	chr24	16096606	16097105	500	-305731	sema5a	-593269	cbln2b	differentially closed at 2dpi
**chr6:59759972-59760471**	chr6	59759972	59760471	500	-2546	zgc:65895	392387	slc32a1	differentially open at 12dpi
**chr6:59899089-59900027**	chr6	59899089	59900027	939	-1029	ddx3b	253051	slc32a1	differentially open at 12dpi
**chr7:6944553-6945429**	chr7	6944553	6945429	877	130125	actn3b	315381	pfdn2	differentially open at 12dpi
**chr9:17415700-17416199**	chr9	17415700	17416199	500	-5523	zgc:101559	-15126	rgcc	differentially open at 12dpi
**chr15:253918-254417**	chr15	253918	254417	500	115	sept4b	89204	limk1a	differentially open at 12dpi
**chr17:43296504-43297003**	chr17	43296504	43297003	500	141105	plk4	-255485	npc2	differentially open at 12dpi
**chr2:81394-81893**	chr2	81394	81893	500	31014	zgc:153913	-1514947	exosc4	differentially closed at 12dpi

**chr2:19780119-19781338**	chr2	19780119	19781338	1220	-66386	pimr141	265831	elovl1a	differentially closed at 12dpi
**chr2:19781474-19781973**	chr2	19781474	19781973	500	-65390	pimr141	266827	elovl1a	differentially closed at 12dpi
**chr2:19787237-19788192**	chr2	19787237	19788192	956	-59400	pimr141	272817	elovl1a	differentially closed at 12dpi
**chr2:19813598-19814097**	chr2	19813598	19814097	500	-33266	pimr141	298951	elovl1a	differentially closed at 12dpi
**chr2:19817067-19817998**	chr2	19817067	19817998	932	-29582	pimr141	302635	elovl1a	differentially closed at 12dpi
**chr2:19818257-19818756**	chr2	19818257	19818756	500	-28608	pimr141	303609	elovl1a	differentially closed at 12dpi
**chr2:19822778-19823647**	chr2	19822778	19823647	870	-23902	pimr141	308315	elovl1a	differentially closed at 12dpi
**chr2:19826215-19827613**	chr2	19826215	19827613	1399	-20200	pimr141	312017	elovl1a	differentially closed at 12dpi
**chr3:55093026-55093806**	chr3	55093026	55093806	781	52958	mafga	-355101	rhbdf1a	differentially closed at 12dpi
**chr3:55095105-55095604**	chr3	55095105	55095604	500	51020	mafga	-357039	rhbdf1a	differentially closed at 12dpi
**chr3:55100242-55101294**	chr3	55100242	55101294	1053	45606	mafga	-362453	rhbdf1a	differentially closed at 12dpi
**chr3:55105417-55106689**	chr3	55105417	55106689	1273	40321	mafga	-367738	rhbdf1a	differentially closed at 12dpi
**chr4:28604846-28605345**	chr4	28604846	28605345	500	-126528	mir430a-18	-1293028	fam19a5a	differentially closed at 12dpi
**chr5:47142933-47143815**	chr5	47142933	47143815	883	-36609	cox7c	-1237825	hapln1a	differentially closed at 12dpi
**chr7:52730398-52730897**	chr7	52730398	52730897	500	-38212	LOC557301	-447240	tcf12	differentially closed at 12dpi
**chr7:52740095-52740594**	chr7	52740095	52740594	500	-47908	LOC557301	-456936	tcf12	differentially closed at 12dpi
**chr7:52742377-52743183**	chr7	52742377	52743183	807	-50344	LOC557301	-459372	tcf12	differentially closed at 12dpi
**chr7:52748837-52749336**	chr7	52748837	52749336	500	-56650	LOC557301	-465678	tcf12	differentially closed at 12dpi
**chr7:54788736-54789235**	chr7	54788736	54789235	500	-22586	snai3	543078	galns	differentially closed at 12dpi
**chr7:54790698-54791197**	chr7	54790698	54791197	500	-20624	snai3	541116	galns	differentially closed at 12dpi
**chr8:34228765-34229586**	chr8	34228765	34229586	822	-152153	pbx3b	-152153	pbx3b	differentially closed at 12dpi
**chr8:34229943-34230784**	chr8	34229943	34230784	842	-153341	pbx3b	-153341	pbx3b	differentially closed at 12dpi
**chr8:34235505-34236004**	chr8	34235505	34236004	500	-158731	pbx3b	-158731	pbx3b	differentially closed at 12dpi
**chr8:34239947-34240446**	chr8	34239947	34240446	500	-163173	pbx3b	-163173	pbx3b	differentially closed at 12dpi
**chr8:34243228-34243727**	chr8	34243228	34243727	500	-166455	pbx3b	-166455	pbx3b	differentially closed at 12dpi
**chr8:34244143-34244642**	chr8	34244143	34244642	500	-167369	pbx3b	-167369	pbx3b	differentially closed at 12dpi
**chr8:34846434-34847288**	chr8	34846434	34847288	855	49679	hpdb	-769838	pbx3b	differentially closed at 12dpi
**chr8:34851982-34852936**	chr8	34851982	34852936	955	55277	hpdb	-775436	pbx3b	differentially closed at 12dpi
**chr8:34857277-34857776**	chr8	34857277	34857776	500	60344	hpdb	-780503	pbx3b	differentially closed at 12dpi
**chr8:34874225-34874724**	chr8	34874225	34874724	500	77292	hpdb	-797451	pbx3b	differentially closed at 12dpi
**chr10:16847552-16848051**	chr10	16847552	16848051	500	62555	stoml2	304222	slc27a6	differentially closed at 12dpi
**chr10:16848134-16848633**	chr10	16848134	16848633	500	61973	stoml2	304804	slc27a6	differentially closed at 12dpi
**chr10:16848644-16849143**	chr10	16848644	16849143	500	61463	stoml2	305314	slc27a6	differentially closed at 12dpi
**chr10:16851015-16852861**	chr10	16851015	16852861	1847	58419	stoml2	308358	slc27a6	differentially closed at 12dpi
**chr10:16858621-16859391**	chr10	16858621	16859391	771	51351	stoml2	315426	slc27a6	differentially closed at 12dpi
**chr10:16880577-16881974**	chr10	16880577	16881974	1398	29081	stoml2	337696	slc27a6	differentially closed at 12dpi
**chr11:17430700-17431898**	chr11	17430700	17431898	1199	148053	fam19a4b	-1279472	magi1b	differentially closed at 12dpi
**chr11:17432488-17432987**	chr11	17432488	17432987	500	146614	fam19a4b	-1278033	magi1b	differentially closed at 12dpi
**chr11:17432997-17433496**	chr11	17432997	17433496	500	146106	fam19a4b	-1277525	magi1b	differentially closed at 12dpi
**chr11:17433633-17434603**	chr11	17433633	17434603	971	145234	fam19a4b	-1276653	magi1b	differentially closed at 12dpi
**chr13:18988177-18988676**	chr13	18988177	18988676	500	-164595	eif3s10	807947	eif4e1c	differentially closed at 12dpi
**chr14:19788805-19789543**	chr14	19788805	19789543	739	-70165	fmr1	1928907	ssrp1a	differentially closed at 12dpi
**chr14:19792068-19792567**	chr14	19792068	19792567	500	-67021	fmr1	1925763	ssrp1a	differentially closed at 12dpi
**chr14:19792901-19794038**	chr14	19792901	19794038	1138	-65869	fmr1	1924611	ssrp1a	differentially closed at 12dpi
**chr14:19796029-19797437**	chr14	19796029	19797437	1409	-62606	fmr1	1921348	ssrp1a	differentially closed at 12dpi
**chr14:19810584-19811525**	chr14	19810584	19811525	942	-48285	fmr1	1907027	ssrp1a	differentially closed at 12dpi
**chr15:14830321-14830820**	chr15	14830321	14830820	500	13985	or129-1	-556208	zgc:162193	differentially closed at 12dpi
**chr16:26006060-26007021**	chr16	26006060	26007021	962	-91798	irge4	352429	arhgef1b	differentially closed at 12dpi
**chr16:26013553-26014547**	chr16	26013553	26014547	995	-99308	irge4	344919	arhgef1b	differentially closed at 12dpi
**chr16:26053213-26053712**	chr16	26053213	26053712	500	-85665	prss59.1	305507	arhgef1b	differentially closed at 12dpi
**chr16:26056249-26056748**	chr16	26056249	26056748	500	-82629	prss59.1	302471	arhgef1b	differentially closed at 12dpi
**chr16:26058671-26059170**	chr16	26058671	26059170	500	-80207	prss59.1	300049	arhgef1b	differentially closed at 12dpi
**chr16:26065441-26065940**	chr16	26065441	26065940	500	-73437	prss59.1	293279	arhgef1b	differentially closed at 12dpi
**chr16:26067798-26068703**	chr16	26067798	26068703	906	-70877	prss59.1	290719	arhgef1b	differentially closed at 12dpi
**chr17:45219176-45219675**	chr17	45219176	45219675	500	27546	capn3a	184145	tmem206	differentially closed at 12dpi
**chr17:45222066-45222565**	chr17	45222066	45222565	500	30436	capn3a	181255	tmem206	differentially closed at 12dpi
**chr17:46968319-46968818**	chr17	46968319	46968818	500	-38468	zgc:103755	1242885	smoc1	differentially closed at 12dpi
**chr17:46977515-46978014**	chr17	46977515	46978014	500	-29272	zgc:103755	1233689	smoc1	differentially closed at 12dpi
**chr17:46978496-46978995**	chr17	46978496	46978995	500	-28290	zgc:103755	1232707	smoc1	differentially closed at 12dpi
**chr17:46981453-46982699**	chr17	46981453	46982699	1247	-24960	zgc:103755	1229377	smoc1	differentially closed at 12dpi
**chr20:4437575-4438074**	chr20	4437575	4438074	500	-68077	ipcef1	846406	ism2b	differentially closed at 12dpi
**chr21:25809834-25810333**	chr21	25809834	25810333	500	-44733	mettl27	-225121	rpl23a	differentially closed at 12dpi
**chr21:25823531-25824030**	chr21	25823531	25824030	500	-58429	mettl27	-211425	rpl23a	differentially closed at 12dpi
**chr21:44162938-44163437**	chr21	44162938	44163437	500	45439	fgf1b	-205396	camk2a	differentially closed at 12dpi
**chr22:24497163-24497662**	chr22	24497163	24497662	500	-35194	wdr47b	-275554	rgs2	differentially closed at 12dpi
**chr22:24500122-24501449**	chr22	24500122	24501449	1328	-31820	wdr47b	-278928	rgs2	differentially closed at 12dpi
**chr22:24503566-24505214**	chr22	24503566	24505214	1649	-28216	wdr47b	-282532	rgs2	differentially closed at 12dpi
**chr23:12684435-12685935**	chr23	12684435	12685935	1501	-166455	snx21	-274391	phactr3a	differentially closed at 12dpi
**chr23:16244775-16246161**	chr23	16244775	16246161	1387	-85259	mrgbp	-122666	eef1a2	differentially closed at 12dpi
**chr24:16118773-16120428**	chr24	16118773	16120428	1656	-282987	sema5a	-616013	cbln2b	differentially closed at 12dpi
**chr24:16126674-16127173**	chr24	16126674	16127173	500	-275663	sema5a	-623337	cbln2b	differentially closed at 12dpi
**chrUn_KN149692v1:4089-5235**	chrUn_KN149692v1	4089	5235	1147	NA	NA	NA	NA	differentially closed at 12dpi
**chrUn_KN149692v1:5249-6134**	chrUn_KN149692v1	5249	6134	886	NA	NA	NA	NA	differentially closed at 12dpi
**chrUn_KN149692v1:10411-11564**	chrUn_KN149692v1	10411	11564	1154	NA	NA	NA	NA	differentially closed at 12dpi
**chrUn_KN149692v1:13822-14321**	chrUn_KN149692v1	13822	14321	500	NA	NA	NA	NA	differentially closed at 12dpi
**chrUn_KN149692v1:16713-17212**	chrUn_KN149692v1	16713	17212	500	NA	NA	NA	NA	differentially closed at 12dpi
**chrUn_KN149692v1:20397-21478**	chrUn_KN149692v1	20397	21478	1082	NA	NA	NA	NA	differentially closed at 12dpi

Table S5. Differentially expressed transcripts that encode transcription factors with and without known motifs.

Ensembl ID	zebrafish gene symbol	padj	LFC: 2dpi-0dpi	LFC: 4dpi-0dpi	LFC: 7dpi-0dpi	LFC: 12dpi-0dpi	cluster name
ENSDART00000140760	e2f7	0.044060134	0.798632306	0.496792942	-0.026056819	-0.454809271	growth toward the midline
ENSDART00000056005	ascl1a	0.032093457	1.787445127	1.283325532	0.749862779	0.792492648	growth toward the midline
ENSDART00000128488	e2f8	0.003382652	3.852661131	-0.929008264	3.252279997	2.01178299	growth toward the midline
ENSDART00000010248	mitfb	0.042577687	0.302243319	0.237275907	0.106756262	-0.337810921	growth toward the midline
ENSDART00000157487	tfec	0.001790465	0.534318298	0.423012313	0.205933849	-0.313187686	growth toward the midline
ENSDART00000114288	foxp4	0.028339664	0.17723361	0.325948274	0.017102596	-0.613519733	growth toward the midline
ENSDART00000158466	creb3l2	0.011352496	0.073267533	0.091531466	-0.07087733	-0.32121815	growth toward the midline
ENSDART00000164766	tfec	0.012357429	0.976052476	1.18639141	0.809154671	-1.643283392	growth toward the midline
ENSDART00000142584	alx1	0.047364517	1.361674144	1.279023188	1.723053191	-0.115335088	growth toward the midline
ENSDART00000081468	ccdc79	0.001875423	0.331555315	0.505423262	0.318137438	-0.103372006	growth toward the midline
ENSDART00000155066	atf5b	0.027985198	0.303223783	0.449779577	0.245269838	-0.118029433	growth toward the midline
ENSDART00000160644	rela	0.030637152	0.027046368	0.222531925	0.299056597	-0.13648552	growth toward the midline
ENSDART00000146767	fosl1a	0.046117855	0.841007094	1.285901651	0.733422298	0.173089455	growth toward the midline
ENSDART00000152378	tgif1	0.003122803	0.194631245	0.419803811	0.14379228	-0.205607529	growth toward the midline
ENSDART00000051549	tp53	0.029170799	0.110640197	0.281712522	0.180041895	-0.162598759	growth toward the midline
ENSDART00000013409	prmt3	0.000336326	0.583250552	0.486757337	0.420985526	-0.028812313	growth toward the midline
ENSDART00000104519	stat3	0.004480141	0.6458865	0.459098575	0.438501049	0.201812376	growth toward the midline
ENSDART00000058277	znf800b	0.018079865	0.363600272	0.378301813	0.156623376	-0.047842936	growth toward the midline
ENSDART00000103982	nfe2l1a	0.007828891	0.652859432	0.675562602	0.38648833	-0.18398816	growth toward the midline
ENSDART00000093279	spi1b	0.002640644	0.891535687	0.563855895	0.100587929	-0.531177734	growth toward the midline
ENSDART00000036729	spi1b	0.004358158	0.910663058	0.717004711	0.095052493	-0.117318513	growth toward the midline
ENSDART00000008373	fosl1a	0.001576361	0.78271954	0.570182098	0.226810387	-0.332448721	growth toward the midline
ENSDART00000093199	tead3b	0.016523707	1.081516431	0.86043279	0.478204597	-0.215799128	growth toward the midline
ENSDART00000062702	cebpb	0.018433591	0.973724375	0.549827472	0.384246832	-0.627629236	growth toward the midline
ENSDART00000056457	mitfa	0.005353377	0.929754118	0.38849893	0.30836176	-0.311729197	growth toward the midline
ENSDART00000080854	stat3	0.03033326	0.856010749	0.444560766	0.379796349	-0.326021696	growth toward the midline
ENSDART00000017185	tbx20	0.035967167	0.559146809	0.370265655	-0.151461394	-0.656442573	growth toward the midline
ENSDART00000086053	PRDM16	0.037061149	0.751033854	0.474975066	0.399761078	-0.264438466	growth toward the midline
ENSDART00000052366	cebpa	0.035286858	0.847474055	0.814939712	0.318232429	-0.173654256	growth toward the midline
ENSDART00000062854	nfe2l2a	0.008134819	0.155734951	0.146836561	-0.112595282	-0.207726174	growth toward the midline
ENSDART00000016135	nfe2l3	0.02142158	0.030439041	0.342226134	0.430712572	0.086440986	midline crossing
ENSDART00000141068	sox11b	8.49E-05	1.611838536	2.177304965	2.010624009	1.194765222	midline crossing
ENSDART00000149074	sall1b	2.18E-05	1.974229239	2.883794957	2.506155499	1.735121972	midline crossing
ENSDART00000138821	nfil3	0.000101984	1.376309909	2.121391468	1.99718084	1.39511595	midline crossing
ENSDART00000153227	nfe2l1b	0.013323448	0.688085235	1.301360444	1.138008541	0.729999096	midline crossing
ENSDART00000082434	tgif1	1.75E-05	0.822910382	1.229522702	1.441745222	0.912580325	midline crossing
ENSDART00000104317	klf7b	0.000976528	0.389436838	1.156291561	1.140404424	0.652703479	midline crossing
ENSDART00000099849	arntl2	9.24E-05	0.982355145	1.78511579	1.609893892	1.255639962	midline crossing
ENSDART00000123263	phf20a	0.000701449	3.204242437	4.684552705	5.028378766	3.758919167	midline crossing
ENSDART00000124040	insm1a	1.46E-05	0.50962443	1.551047216	1.738068624	1.104992333	midline crossing
ENSDART00000159652	sox4b	0.008897727	0.767109202	1.080655119	1.389017231	0.976257694	midline crossing
ENSDART00000062576	thyn1	0.006623227	0.902996379	0.741810201	0.792519989	0.219218083	midline crossing
ENSDART00000098285	atf5a	0.018496153	0.558593719	0.754458044	0.445954377	0.160619888	midline crossing
ENSDART00000063912	jun	0.002341904	1.166146477	1.429328618	1.29995447	0.480560375	midline crossing
ENSDART00000022060	atf3	2.20E-05	2.958540036	3.348918067	2.852908333	1.811501419	midline crossing
ENSDART00000110040	sox11a	8.49E-05	1.573346229	1.723237215	1.23195588	0.781584101	midline crossing
ENSDART00000141941	zfpm2a	0.046610115	3.791965294	3.402723828	3.788725401	3.249505128	midline crossing
ENSDART00000161610	tcf3b	0.04032679	0.066966853	0.197082202	0.107652202	-0.03222147	midline crossing
ENSDART00000012791	sp8a	0.049626195	1.57251014	2.331511764	2.00847442	1.039704847	midline crossing
ENSDART00000122628	junba	0.016136147	0.280813702	0.583374691	0.41181471	0.073998168	midline crossing
ENSDART00000024872	creb3l3l	0.003959852	0.316205692	0.628018553	0.525420097	0.254183225	midline crossing
ENSDART00000123559	znf521	0.000648976	1.710422257	2.255410725	1.850379402	0.814044199	midline crossing
ENSDART00000147849	klf6a	0.000870446	1.130982604	1.870693821	1.403514717	0.445060502	midline crossing
ENSDART00000076161	hoxb5b	0.013675681	2.652791903	3.351280664	2.618897772	0.864721852	midline crossing
ENSDART00000033494	klf6a	0.000170861	1.084550888	1.797692613	1.625562666	0.460444617	midline crossing
ENSDART00000077484	zhx2a	0.005803283	0.117201305	0.356185793	0.299966576	0.024327323	midline crossing
ENSDART00000055609	atf4b	0.027440234	0.138344185	0.51758353	0.353617358	0.087935611	midline crossing
ENSDART00000138081	bcl6ab	0.000589104	0.948060654	0.690527167	0.988530239	1.012261919	midline crossing
ENSDART00000140284	zbtb16a	0.028958693	2.407975491	2.022869932	2.341949009	2.679474288	midline crossing
ENSDART00000132119	max	0.028918785	0.339991432	0.197083488	0.14057043	0.318686745	midline crossing
ENSDART00000139763	stat5a	0.018355029	3.442434617	3.964844196	3.417693165	3.322184683	midline crossing
ENSDART00000109552	baz2ba	0.004360669	0.627489244	0.525669422	0.712410774	0.393101067	midline crossing
ENSDART00000029843	vezf1a	0.001561003	0.660507751	0.776194217	0.884683465	0.428803142	midline crossing
ENSDART00000041877	csrnp1a	0.000166716	1.299872398	1.369159722	1.350463811	0.717287101	midline crossing
ENSDART00000170865	nme2b.1	0.007383951	0.635993303	0.679448572	0.689008761	0.355584206	midline crossing
ENSDART00000135381	six4a	0.04980099	2.521510715	2.3671408	2.587075649	1.691172374	midline crossing
ENSDART00000018743	phf20a	0.027269932	0.312029532	0.419745206	0.325238046	0.318832686	midline crossing
ENSDART00000163794	wt1b	0.02934736	0.454982734	0.330694147	0.317167947	0.300420298	midline crossing
ENSDART00000066317	foxn2b	0.033486585	-0.194754205	-0.013664444	0.221561118	-0.080707177	target selection
ENSDART00000158598	si:ch211-232b12.5	0.004900248	-0.304573383	0.212713124	0.452361247	0.038090441	target selection

ENSDART00000154418	relb	0.010687727	-0.043359819	0.083083005	0.26390054	-0.071618881	target selection
ENSDART00000114473	trafd1	0.018765627	-0.080068432	0.005557339	0.252422771	-0.057760312	target selection
ENSDART00000123197	relb	0.019118248	-0.261655378	-0.003226565	0.486237661	-0.265984214	target selection
ENSDART00000152489	irf3	0.006723268	-0.152502705	0.050924712	1.421424509	-0.375867622	target selection
ENSDART00000141792	stat1b	0.033143681	-1.394223287	-0.705218411	2.324563925	-0.328164239	target selection
ENSDART00000103622	irf7	0.002828188	-0.049332142	-0.2878598	1.533925917	-0.275107455	target selection
ENSDART00000148661	stat2	0.006978095	0.11967996	-0.016653911	1.354000143	0.023510931	target selection
ENSDART00000005720	stat1a	0.010250585	0.035880276	0.045195542	0.615137639	0.020671748	target selection
ENSDART00000156935	pbx4	0.039411387	0.960146191	0.964981474	3.716226941	3.606625876	target selection
ENSDART00000151044	baz2ba	0.006344596	2.371956879	3.808025242	3.802702579	3.968575995	target selection
ENSDART00000023959	arntl1a	7.44E-05	0.340904931	1.030627189	1.45255612	0.975945139	target selection
ENSDART00000104523	arntl1b	0.000255127	-0.158144123	0.59291526	1.113237486	0.52944604	target selection
ENSDART00000083628	ddit3	0.002192622	-0.053032847	0.505000026	0.598711637	0.25299651	target selection
ENSDART00000110512	ybx1	0.014352201	0.187678935	0.306636132	0.543417204	0.157462635	target selection
ENSDART00000135647	si:ch211-113e8.10	0.041164305	-0.117833263	-0.003423122	0.202369294	0.16110756	target selection
ENSDART00000025575	clocka	0.041666185	-0.204691736	0.089686311	0.498135587	0.316118853	target selection
ENSDART00000152636	clocka	0.010610046	0.008631834	0.196030905	0.593132542	0.426491394	target selection
ENSDART00000089076	dot1l	0.009173096	0.094529933	0.165735937	0.448240264	0.463978672	target selection
ENSDART00000013605	zbtb20	0.00075553	0.021191929	0.070670156	0.462529396	0.345025042	target selection
ENSDART00000165877	purg	0.000570625	-0.159238984	-0.087175961	1.048436006	0.831059287	target selection
ENSDART00000000280	stat1b	0.037541166	-0.005066017	0.035352481	0.819259146	0.412054224	target selection
ENSDART00000087450	klf13	0.013792445	-0.22166629	-0.059571459	0.622443971	0.288396102	target selection
ENSDART00000146180	csrnp2	0.003710792	0.237147162	0.374829692	0.697707124	0.402573932	target selection
ENSDART00000026992	sox4a	3.66E-05	0.168735758	0.553498244	0.871364123	0.630648218	target selection
ENSDART00000048110	six4b	0.002795018	0.757934328	2.322346312	2.776043581	1.88012445	target selection
ENSDART00000013961	mycla	0.015752787	0.091841991	0.503579515	0.855574629	0.294011787	target selection
ENSDART00000165656	mxd3	6.24E-05	0.030444632	0.928898979	1.014066924	0.787179484	target selection
ENSDART00000018150	neurod6b	0.012342358	0.484226112	1.695956927	1.935318885	1.555476315	target selection
ENSDART00000084819	arhgap35b	0.001659006	0.101645005	0.291379647	0.535032879	0.462050203	target selection
ENSDART00000075331	insm1b	0.009480138	0.180818368	0.558805202	0.852549514	0.856981121	target selection
ENSDART00000123147	zbtb8a	0.026040305	-0.281752517	0.027773484	0.518421131	0.366031095	target selection
ENSDART00000018945	meis3	0.00431294	-0.263611366	0.389783427	1.01804147	0.898863275	target selection
ENSDART00000111014	myca	0.024986086	-0.198726525	0.073855205	0.316369866	0.312548093	target selection
ENSDART00000092690	srebf2	0.003411856	-0.007030288	0.441941306	0.773565631	0.436893785	target selection
ENSDART00000104712	hmg20a	0.038745219	0.003064442	0.120221197	0.366113134	0.275609671	target selection
ENSDART00000033755	homezb	0.043076195	-0.059847052	0.060728596	0.214721864	0.105513871	target selection
ENSDART00000047724	zeb1b	0.032175544	-0.128023017	-0.020882446	0.202453179	0.103960026	target selection
ENSDART00000085728	klf8	0.014069453	-0.229536876	-0.061888352	0.204863755	0.114771443	target selection
ENSDART00000037516	znf827	0.032136194	0.117251719	-0.001942433	0.235271462	0.223866066	target selection
ENSDART00000150128	foxp1b	0.020184087	0.347545443	0.556370195	1.342974258	1.269825931	target selection
ENSDART00000128174	foxp1b	0.015925333	0.060807795	0.283454627	0.694455914	0.748409918	target selection
ENSDART00000060193	thap3	0.039451639	0.219009088	0.189913345	0.546438828	0.599142566	target selection
ENSDART00000154730	tsc22d1	0.044459756	0.125146937	0.071380598	0.346511952	0.250641998	target selection
ENSDART00000114246	adnpb	0.022770625	-0.221340948	-0.183838281	-0.013868823	0.044049817	brain innervation
ENSDART00000109356	klf7a	0.000177237	-0.7063233	-0.659659661	-0.044315321	0.157900055	brain innervation
ENSDART00000144186	pou6f2	7.05E-05	-0.474463335	-0.465571046	0.092941552	0.436081081	brain innervation
ENSDART00000124112	pou4f2	2.95E-05	-1.368026557	-1.308261738	0.344262724	0.908586045	brain innervation
ENSDART00000051693	irx4a	0.000591404	-1.596922819	-2.014349322	0.164610846	0.924604512	brain innervation
ENSDART00000149175	pou4f3	3.34E-05	-1.520089487	-1.231379123	0.357488642	0.743891028	brain innervation
ENSDART00000055936	isl2b	4.92E-05	-1.527471977	-1.312125436	0.470803449	0.853726063	brain innervation
ENSDART00000023038	dacha	0.000322945	-0.511894205	-0.418991667	-0.004522839	0.112876213	brain innervation
ENSDART00000142087	foxp2	0.000565675	-0.522019738	-0.4194261	0.065656049	0.27497631	brain innervation
ENSDART00000100508	ebf1b	0.010170292	-0.252122108	-0.356560297	-0.077233007	0.218128715	brain innervation
ENSDART00000162485	pax6a	0.013416969	-0.383059238	-0.321515523	-0.107334897	0.077147684	brain innervation
ENSDART00000112529	znf319a	0.023015173	-0.271600655	-0.228932764	-0.01869534	0.01981331	brain innervation
ENSDART00000165609	barhl2	0.000401616	-0.31272019	-0.374018735	0.126371762	0.224852149	brain innervation
ENSDART00000010896	isl1	0.002539705	-0.281634356	-0.275090348	0.147086866	0.205392134	brain innervation
ENSDART00000145072	neurod4	0.04301836	-0.171174556	-0.165007281	0.082300141	0.101934405	brain innervation
ENSDART00000165774	pax6a	0.007156543	-0.382942423	-0.218376905	0.094924321	0.282463268	brain innervation
ENSDART00000101316	znf618	0.019075692	-0.33316659	-0.306975689	-0.091658088	0.079150867	brain innervation
ENSDART00000064833	mafaa	0.002047801	-0.957052225	-1.143383392	-0.379735148	0.067354966	brain innervation
ENSDART00000004548	barhl1b	0.009605408	-2.472825095	-1.41656094	-0.268019579	0.230183047	brain innervation
ENSDART00000006380	tbx3a	0.004106215	-0.244591708	-0.352646993	-0.144447405	0.06961498	brain innervation
ENSDART00000101070	dachd	0.00434437	-0.296270747	-0.351582482	-0.130791564	-0.009393292	brain innervation
ENSDART00000151904	foxn3	0.000466769	-0.402174688	-0.533042351	-0.141780798	0.022724604	brain innervation
ENSDART00000124751	kcnip3b	2.63E-05	-1.29606503	-1.383048631	-0.490749215	-0.039127508	brain innervation
ENSDART00000151571	ahdc1	0.000666959	-0.753523402	-0.774930454	-0.265860844	-0.025283973	brain innervation
ENSDART00000080808	six3a	0.000687981	-0.437705937	-0.430575558	-0.140593988	-0.07114268	brain innervation
ENSDART00000171594	mef2aa	0.010231208	-0.649844171	-0.685014218	-0.301258241	-0.003741614	brain innervation
ENSDART00000062982	foxo3b	0.005646833	-0.232285352	-0.219543276	-0.013673206	0.18814406	brain innervation
ENSDART00000009691	scml4	0.001274316	-0.348233268	-0.325522885	-0.113421585	0.115178638	brain innervation
ENSDART00000160784	satb1b	0.006456063	-0.404843174	-0.932859181	0.352272227	0.695966701	brain innervation
ENSDART00000101631	satb1b	0.000172812	-0.360112936	-0.384116906	0.134197196	0.481946288	brain innervation

ENSDART00000144894	bcl11ba	3.52E-05	-0.656989822	-0.679320488	0.181338486	0.623847694	brain innervation
ENSDART00000165698	pbx1a	0.000272902	-0.205212048	-0.330154536	0.047550564	0.321002436	brain innervation
ENSDART00000081170	cux1a	0.000900234	-0.092953946	-0.2283391	0.138990685	0.296890549	brain innervation
ENSDART00000160675	dpf1	0.00210146	-0.344267461	-0.462495684	0.239034332	0.46811192	brain innervation
ENSDART00000045628	irx6a	0.000781013	-0.424070635	-0.239579413	0.17038084	0.276181325	brain innervation
ENSDART00000048994	pbx3b	0.000910662	-0.545498331	-0.428509616	0.129533791	0.149586816	brain innervation
ENSDART00000077157	six3b	0.008086296	-0.273810969	-0.17356253	0.033591817	0.098241195	brain innervation
ENSDART00000136245	cux1a	0.013366886	-0.163227222	-0.237382603	-0.00302824	0.164419575	brain innervation
ENSDART00000154646	bcl11ba	0.001677265	-0.533670184	-0.516802708	0.152594691	0.570166001	brain innervation
ENSDART00000153150	myt1la	0.001328724	-0.378588425	-0.355041474	0.131237693	0.393706803	brain innervation
ENSDART00000166565	tshz3b	0.011919383	-0.202679362	-0.107608077	0.106987356	0.28761259	brain innervation
ENSDART00000013148	pou3f1	0.00849815	-0.360437586	-0.322601739	-0.060819622	0.211329038	brain innervation
ENSDART00000162945	sox5	0.023596757	-0.177385379	-0.174200373	-0.003535976	0.152149827	brain innervation
ENSDART00000171091	zeb2b	0.008482497	-0.344020449	-0.404143226	-0.222307379	0.140992606	brain innervation
ENSDART00000170631	ebf1a	0.016255169	-0.245696473	-0.326200552	-0.132641619	0.115308885	brain innervation
ENSDART00000156464	prr12b	0.021599545	-0.068684116	-0.215586059	0.031477455	0.130187301	brain innervation
ENSDART00000167440	zfhx3	0.018342429	-0.067144102	-0.226395351	0.21751863	0.266797189	brain innervation
ENSDART00000167538	lcor	0.031258003	-0.151917658	-0.14482463	0.130691961	0.223379898	brain innervation
ENSDART00000165223	pbx1b	0.015188807	-0.243991197	-0.377373898	-0.18693501	0.141565589	brain innervation
ENSDART00000172410	jazf1a	0.045787549	-0.354446023	-0.388940674	-0.011638795	-0.018894552	brain innervation
ENSDART00000022010	hivep2b	0.004657196	-0.404683257	-0.428225913	-0.085969125	0.061919909	brain innervation
ENSDART00000138048	pbx3b	0.007841242	-0.599655595	-0.602159282	-0.228775798	-0.019503135	brain innervation
ENSDART00000137355	tet3	0.033062691	-0.245273707	-0.407219331	-0.050664281	-0.06569376	brain innervation
ENSDART00000104790	znf292b	0.044988705	-0.062768954	-0.233087007	-0.040681021	0.023710275	brain innervation
ENSDART00000136729	ebf1b	0.021027344	-2.79991744	-3.068779235	-0.720206417	0.313112936	brain innervation
ENSDART00000132589	mef2d	0.028030725	-0.20910625	-0.209165039	-0.020444617	0.107291243	brain innervation
ENSDART00000003884	mynn	0.033340088	-0.108806257	-0.247003557	0.197551703	0.151198862	brain innervation
ENSDART00000104327	vsx1	0.00488592	-0.248525466	-0.619641572	-0.034766195	-0.028538746	brain innervation
ENSDART00000084035	znf532	0.047492859	-0.199573234	-0.524026601	-0.06012509	0.154210784	brain innervation
ENSDART00000134064	mxd1	0.015571917	-0.125104323	-0.265030232	-0.047451672	0.092856449	brain innervation
ENSDART00000136759	prdm13	0.00326136	-0.225066145	-0.413438688	-0.129520305	0.036146962	brain innervation
ENSDART00000166540	kcnip3b	0.047816671	-0.988549414	-0.704807394	-0.278733631	0.119291619	brain innervation
ENSDART00000030773	foxo3a	0.000701449	-0.522674186	-0.346284342	-0.025883071	0.086783781	brain innervation
ENSDART00000111842	sall1a	0.033435193	-0.3702182	-0.245271728	-0.043742586	0.007554245	brain innervation
ENSDART00000166976	esrrb	0.030820243	-0.481593528	-0.114040699	0.229959907	0.235353141	brain innervation
ENSDART00000151575	thraa	0.013354221	-0.36498113	-0.584870078	0.329302923	-0.150628947	brain innervation
ENSDART00000007806	zbtb16a	0.004364661	-0.634450415	-0.731920804	0.190781828	-0.364381887	brain innervation
ENSDART00000125158	znf574	0.029152083	-0.158249462	-0.223654122	0.188172427	0.054732788	brain innervation
ENSDART00000011890	zbtb22a	0.03843558	-0.42030539	-0.536466631	0.147549186	0.05725602	brain innervation
ENSDART00000155866	zbtb16b	8.49E-05	-0.434457287	-0.595844526	0.044569606	0.015262284	brain innervation
ENSDART00000001795	sp3a	0.0407578	-0.269877105	-0.305437708	-0.047306155	-0.033496274	brain innervation
ENSDART00000109493	tshz2	0.008648063	-0.247840671	0.10394662	0.365597315	0.467215515	brain innervation
ENSDART00000083296	ZNF423	0.00814358	-0.278315489	0.051691519	0.407184973	0.489807123	brain innervation
ENSDART00000058685	zfpm2a	0.011667422	-0.080421638	-0.032548615	0.512175645	0.757319343	brain innervation
ENSDART00000110069	CABZ01075131.1	0.008675958	-0.117445692	-0.062527677	0.307574487	0.429328576	brain innervation
ENSDART00000131126	bcl6a	0.005688069	-0.096649799	-0.044696983	0.295011601	0.436349263	brain innervation
ENSDART00000111303	rfx7	0.00161581	0.075232989	0.085195342	0.459783838	0.727409902	brain innervation
ENSDART00000122101	tbx2b	0.001190205	-0.056468629	0.017248499	0.19422425	0.382938949	brain innervation
ENSDART00000065139	gfi1ab	0.023335678	-0.252440054	-0.123023724	0.269699656	0.68808671	brain innervation
ENSDART00000150938	sox6	0.001331938	-0.111707858	-0.047191704	0.355284385	0.700337568	brain innervation
ENSDART00000012862	isl2a	3.29E-05	-0.663813396	0.011664777	0.746201858	1.074694305	brain innervation
ENSDART00000158634	ebf3a	0.006418819	-0.930015824	-0.218309076	0.836197649	1.139950385	brain innervation
ENSDART00000166502	satb2	1.14E-05	-0.540440017	-0.158642442	1.216799418	1.496340928	brain innervation
ENSDART00000025031	pou4f1	1.46E-05	-0.37458842	-0.169318799	0.774588614	1.160187758	brain innervation
ENSDART00000016303	irx2a	4.96E-05	-0.381386746	0.064410059	0.752093904	0.989751717	brain innervation
ENSDART00000098859	neurod6a	0.000108364	-0.523659161	-0.308368621	0.583701544	0.979244768	brain innervation
ENSDART00000031167	tfap2d	0.000251123	-0.967562253	-0.629795841	0.638492839	1.047009841	brain innervation
ENSDART00000006612	tbr1b	2.95E-05	-1.166029945	-1.008207372	0.617040877	1.169470893	brain innervation
ENSDART00000157855	ebf3a	0.000670771	-0.765871102	-0.436598509	0.580406091	0.942511549	brain innervation
ENSDART00000167324	ebf3a	4.92E-05	-0.585714789	-0.243731213	0.563349846	0.843370215	brain innervation
ENSDART00000128504	si:dkey-106g10.7	0.03328728	0.018091706	0.0404047	0.309180984	0.459844792	brain innervation
ENSDART00000165955	zhx3	0.013572162	-0.011624436	-0.109014479	0.013312322	0.351264725	brain innervation
ENSDART00000092357	sgsm2	0.018543094	-0.379055867	-0.375730796	-0.097265456	-0.053118339	brain innervation
ENSDART00000170400	ahdc1	0.034543013	-2.952674589	-2.088515533	0.893679477	1.203421878	brain innervation
ENSDART00000150949	nfyc	0.013743075	-0.535274529	-0.301463031	-0.212807295	0.074596031	brain innervation
ENSDART00000166351	nkrf	0.010292856	-0.332043429	-0.213944917	-0.117297764	-0.03436019	brain innervation
ENSDART00000090019	zeb2b	0.016845819	-0.694941899	-0.428168304	-0.296660745	0.024845807	brain innervation
ENSDART00000018603	tbx4	0.04873911	-0.302508569	-0.188111077	-0.131947864	0.000377602	brain innervation
ENSDART00000171642	dpf1	0.005353377	-0.380689139	0.060927985	0.320062308	0.358114425	brain innervation
ENSDART00000113087	hmx1	0.000694227	-0.389387565	0.021377927	0.234018679	0.270888749	brain innervation
ENSDART00000162474	irx1a	0.002931603	-1.132797992	-0.349993381	0.581252213	0.55366573	brain innervation
ENSDART00000162850	irx3a	0.000148815	-0.694816328	-0.301932125	0.187764734	0.208405543	brain innervation
ENSDART00000172990	znf628	0.031889344	-0.174846517	-0.081526828	0.094662541	0.071436879	brain innervation

ENSDART00000112756	ZNF608	0.004908881	-0.246715323	-0.122479962	0.171371612	0.114220238	brain innervation
ENSDART00000090226	znf319b	0.009551702	-0.155602419	-0.092883138	0.255379558	0.168359048	brain innervation
ENSDART00000053782	scrt2	0.003310987	-0.252422437	-0.133353661	0.196438899	0.160308382	brain innervation
ENSDART00000100658	esrra	8.74E-05	-0.571475474	-0.395296451	0.169003635	0.212368184	brain innervation
ENSDART00000008906	znf503	0.001473115	-0.367880648	-0.389528031	0.161538025	0.184463	brain innervation
ENSDART00000164488	mbnl2	0.0337199	-3.245293256	-3.314362259	0.902121491	1.413765252	brain innervation
ENSDART00000130163	nr2f2	0.012656704	-0.280168769	-0.057880311	0.350863039	0.468160546	brain innervation
ENSDART00000074833	rx3	0.010379183	-0.279918529	-0.301637969	0.143576872	0.172520697	brain innervation
ENSDART00000006737	sox5	0.038386468	-0.253791611	-0.278700286	0.184042389	0.275951824	brain innervation
ENSDART00000109347	nacc2	0.002373876	-0.303011569	-0.338345203	0.399887844	0.337648971	brain innervation
ENSDART00000133959	clockb	0.010583972	-0.261160651	-0.146340415	0.224249915	0.15359596	brain innervation
ENSDART00000108775	ubp1	0.021499635	-0.192682892	-0.115240547	0.10453418	0.097860664	brain innervation
ENSDART00000092665	srebf1	0.004619636	-0.417658309	-0.274360919	0.114636817	0.022214824	brain innervation
ENSDART00000104751	mxi1	0.002350825	-0.384587031	-0.210909301	0.22179917	0.104111801	brain innervation
ENSDART00000131714	mbnl2	0.008848885	-0.406601788	-0.032543807	-0.091713267	0.092369986	brain innervation
ENSDART00000124534	mbnl2	0.001000183	-0.407254222	0.078580914	-0.044925118	0.120266311	brain innervation
ENSDART00000155666	znf576.1	0.015256508	-0.372721752	-0.23410815	-0.134923201	-0.013577883	brain innervation
ENSDART00000047541	bach1b	0.039802589	-0.343772395	-0.510091534	-0.468889885	-0.333515696	downregulated during regeneration - early
ENSDART00000007630	nhlh2	0.010056242	-0.28794611	-0.324673306	-0.385652145	-0.261092419	downregulated during regeneration - early
ENSDART00000129498	mef2d	0.03679078	-0.188451231	-0.381445449	-0.263610552	-0.197973462	downregulated during regeneration - early
ENSDART00000055890	znf385c	0.011543549	-0.397413591	-0.444425008	-0.419820845	-0.297765515	downregulated during regeneration - early
ENSDART00000024832	stat5a	0.004077075	-0.212236095	-0.408877789	-0.375060924	-0.185569558	downregulated during regeneration - early
ENSDART00000082346	tfap2a	0.038054224	-0.238141907	-0.307957291	-0.242159487	-0.230339237	downregulated during regeneration - early
ENSDART00000013003	tfap2b	0.008192536	-0.299930992	-0.578681033	-0.25971747	-0.279510711	downregulated during regeneration - early
ENSDART00000167052	etv1	0.01072353	-0.327639473	-0.469002174	-0.32895701	-0.22464313	downregulated during regeneration - early
ENSDART00000157659	camta1b	0.01608096	-0.574265168	-0.690891494	-0.316064045	-0.339000781	downregulated during regeneration - early
ENSDART00000169609	tefb	0.032368681	-0.156114581	-0.555141888	-0.301378906	-0.24132076	downregulated during regeneration - early
ENSDART00000103640	hey1	0.023203992	-0.385659584	-0.747117397	-0.392306381	-0.416692063	downregulated during regeneration - early
ENSDART00000089748	rorb	0.030623213	-0.330969943	-0.539847879	-0.425468683	-0.138099942	downregulated during regeneration - early
ENSDART00000122429	klf15	0.006222767	-0.341190152	-0.616050077	-0.578895446	-0.337948098	downregulated during regeneration - early
ENSDART00000153187	thrab	0.001402519	-0.362356661	-0.551240408	-0.403932333	-0.192068079	downregulated during regeneration - early
ENSDART00000054736	bhlhe23	0.004928313	-0.369485488	-0.514619457	-0.390416516	-0.190127988	downregulated during regeneration - early
ENSDART00000125344	skilb	0.010360832	-0.241964121	-0.627384358	-0.493621211	-0.368838752	downregulated during regeneration - early
ENSDART00000131361	kcnip3b	0.008595135	-0.353450144	-1.035812483	-0.692091767	-0.071599751	downregulated during regeneration - early
ENSDART00000026017	bhlhe40	0.046057741	-0.186426622	-0.329757373	-0.286559283	-0.071751826	downregulated during regeneration - early
ENSDART00000131134	si:ch211-206a7.2	0.008848885	-0.226522125	-0.472315068	-0.371394997	-0.101330709	downregulated during regeneration - early
ENSDART00000046663	camta1b	0.022220725	-0.175908238	-0.543108898	-0.289499765	-0.001152787	downregulated during regeneration - early
ENSDART00000172199	zmat4a	0.029447477	-0.170025595	-0.220098279	-0.198411401	0.018178403	downregulated during regeneration - early
ENSDART00000142223	hivep3b	0.013948629	-0.312228644	-0.441058379	-0.334712198	-0.18438466	downregulated during regeneration - early
ENSDART00000147658	bhlhe22	0.01205445	-0.302235414	-0.31899187	-0.305987775	-0.102192149	downregulated during regeneration - early
ENSDART00000126966	esrrga	0.017679749	-0.248566713	-0.265310961	-0.321308408	-0.070523791	downregulated during regeneration - early
ENSDART00000127353	nr1d2b	0.027085654	-0.29103008	-0.243688493	-0.346507775	-0.100908835	downregulated during regeneration - early
ENSDART00000040672	mecp2	0.047533941	-0.242382035	-0.413395534	-0.149398689	-0.148226048	downregulated during regeneration - early
ENSDART00000057644	lhx4	0.048389146	-0.267668007	-0.268543602	-0.190125613	-0.119751289	downregulated during regeneration - early
ENSDART00000089923	znf652	0.026337068	-0.30560984	-0.322535846	-0.127141181	-0.092457617	downregulated during regeneration - early
ENSDART00000157890	tcf7l1b	0.035686017	-4.555123239	-4.673271453	-1.829433189	-0.732249154	downregulated during regeneration - early
ENSDART00000136488	zmat4a	0.008573382	-0.690962776	-0.637104526	-0.28999313	-0.088561559	downregulated during regeneration - early
ENSDART00000164349	e2f4	0.042377891	-0.246759183	-0.226331986	-0.10688884	-0.101679787	downregulated during regeneration - early
ENSDART00000003548	znf385a	0.001069696	-0.544762633	-0.635515273	-0.316063497	-0.128464507	downregulated during regeneration - early
ENSDART00000033362	gatad2b	0.00857344	-0.40244942	-0.286045422	-0.214483951	-0.084688348	downregulated during regeneration - early
ENSDART00000026865	l3mbtl1a	0.016106242	-0.339631378	-0.338161449	-0.153879513	-0.055902641	downregulated during regeneration - early
ENSDART00000037879	crx	0.018920791	-0.254014844	-0.310425293	-0.22793154	-0.020194538	downregulated during regeneration - early
ENSDART00000135443	kdm5ba	0.039457626	-0.212935098	-0.210173481	-0.205976005	-0.095029648	downregulated during regeneration - early
ENSDART00000165757	pax6b	0.019981901	-1.030621281	-0.736504391	-0.510180699	-0.112500923	downregulated during regeneration - early
ENSDART00000059446	znf385b	0.027304375	-0.260906147	-0.315001382	-0.161772411	-0.110285684	downregulated during regeneration - early
ENSDART00000165120	purab	0.041240008	-0.246648902	-0.188751457	-0.157104845	-0.035763175	downregulated during regeneration - early
ENSDART00000009938	tcf12	0.025246927	-0.385864267	-0.351608927	-0.283149928	-0.323464659	downregulated during regeneration - early
ENSDART00000048775	mbd3b	0.027491007	-0.291847963	-0.361950456	-0.358913558	-0.222238531	downregulated during regeneration - early
ENSDART00000156351	zfat	0.027293911	-0.386268069	-0.362611932	-0.387180541	-0.301395116	downregulated during regeneration - early
ENSDART00000166135	zbtb47b	0.004311817	-0.402709337	-0.409852132	-0.474957603	-0.394147628	downregulated during regeneration - early
ENSDART00000055706	her15.1	0.021144178	-1.483608056	-1.154441068	-0.698110478	-1.436427969	downregulated during regeneration - early
ENSDART00000133487	fosb	0.034512759	-0.882572207	-1.273781011	-0.772506712	-0.914864674	downregulated during regeneration - early
ENSDART00000000876	nr4a1	0.000894056	-2.262372442	-2.399082592	-2.145415905	-1.543723254	downregulated during regeneration - early
ENSDART00000151127	thraa	0.039890399	-0.798583389	-0.802364861	-0.351487338	-0.589241182	downregulated during regeneration - early
ENSDART00000162857	nr4a3	0.003361003	-1.482016444	-1.152680943	-0.606031454	-1.193862677	downregulated during regeneration - early
ENSDART00000153167	hlfb	0.010966965	-0.470839666	-0.326611575	-0.235752692	-0.264047019	downregulated during regeneration - early
ENSDART00000065361	etv5b	0.019698661	-0.645686026	-0.347502384	-0.293939032	-0.317696675	downregulated during regeneration - early
ENSDART00000162387	zbtb38	0.015925333	-0.362042869	-0.285747988	-0.199364681	-0.27814593	downregulated during regeneration - early
ENSDART00000172310	zbtb4	0.013310088	-0.365422353	-0.277101341	-0.237436935	-0.247791483	downregulated during regeneration - early
ENSDART00000054020	hivep3b	0.042576139	0.041000622	-0.364922266	-0.147208921	-0.149490959	downregulated during regeneration - mid
ENSDART00000169283	znf644b	0.046753078	0.013670999	-0.206539721	-0.202072284	-0.100289754	downregulated during regeneration - mid
ENSDART00000161387	tcf12	0.008531138	0.370378706	0.009395421	-0.374363269	-0.152945712	downregulated during regeneration - mid
ENSDART00000127099	nr2e3	0.030676359	0.281603482	0.002911001	-0.560613973	-0.033774044	downregulated during regeneration - mid

ENSDART00000163250	mef2cb	0.003671994	0.361224616	-0.300957895	-0.57227951	-0.080214844	downregulated during regeneration - mid
ENSDART00000131731	mef2ca	0.006008543	0.234535922	-0.250149626	-0.381098175	-0.047609857	downregulated during regeneration - mid
ENSDART00000113286	phf19	0.035367252	0.32344819	-0.020838317	-0.493273441	-0.244699917	downregulated during regeneration - mid
ENSDART00000093331	rreb1a	0.018126285	-0.024901133	-0.155149005	-0.517234878	-0.050769944	downregulated during regeneration - mid
ENSDART00000104279	znf516	0.018737197	-0.151066862	-0.30868422	-0.587761132	-0.246031248	downregulated during regeneration - mid
ENSDART00000143165	tsc22d1	0.012984014	-0.098364585	-0.21923408	-0.39849137	0.017570634	downregulated during regeneration - mid
ENSDART00000148537	roraa	0.01612441	-0.160828233	-0.221872095	-0.45696721	-0.083100906	downregulated during regeneration - mid
ENSDART00000100667	skia	0.021156666	-0.180939747	-0.108825609	-0.328725059	-0.076853639	downregulated during regeneration - mid
ENSDART00000123970	mntb	0.011725523	-0.115861458	-0.101461538	-0.360694649	-0.01567692	downregulated during regeneration - mid
ENSDART00000077839	atf7b	0.014656899	-0.232485852	-0.196508033	-0.391624214	-0.206346003	downregulated during regeneration - mid
ENSDART00000165710	gpbp1l1	0.036571732	-0.164201122	-0.182753901	-0.290062625	-0.125147623	downregulated during regeneration - mid
ENSDART00000124740	ncoa2	0.048038072	-0.028796998	-0.187850383	-0.230877254	0.045612327	downregulated during regeneration - mid
ENSDART00000164082	znf609a	0.041293307	0.094336402	-0.129509301	-0.187095832	0.037446717	downregulated during regeneration - mid
ENSDART00000045374	smad3a	0.043076195	-0.063329624	-0.157712113	-0.303211349	-0.108940629	downregulated during regeneration - mid
ENSDART00000166313	thrb	0.0223553	-0.193382381	-0.415286369	-0.400030228	-0.136855946	downregulated during regeneration - mid
ENSDART00000031426	skilb	0.000177771	-0.234186907	-0.553407151	-0.565936684	-0.420869627	downregulated during regeneration - mid
ENSDART00000148106	mef2aa	0.026582721	-0.081330082	-0.55524128	-0.609035118	-0.185252531	downregulated during regeneration - mid
ENSDART00000091707	dbpa	0.017575469	-0.158262702	-0.327851995	-0.357551076	-0.129469939	downregulated during regeneration - mid
ENSDART00000148066	znf395b	0.020342933	-0.110739145	-0.366906743	-0.460042969	-0.183105049	downregulated during regeneration - mid
ENSDART00000075070	hsf2	0.014034602	-0.066635095	-0.276496416	-0.474097609	-0.186498896	downregulated during regeneration - mid
ENSDART00000148353	usf2	0.006964046	-0.107987138	-0.265954127	-0.405201274	-0.200197809	downregulated during regeneration - mid
ENSDART00000061106	bhlhe41	0.000553147	0.043056173	-0.423617996	-0.754959095	-0.246095493	downregulated during regeneration - mid
ENSDART00000139102	dbpb	0.001993937	0.113164671	-0.364559303	-0.423196921	-0.137052116	downregulated during regeneration - mid
ENSDART00000100181	sall3b	0.034764006	-0.170454734	-0.209016072	-0.285639823	-0.232809645	downregulated during regeneration - mid
ENSDART00000141734	hivep2a	0.017814842	-0.218148846	-0.337091836	-0.481607769	-0.327040294	downregulated during regeneration - mid
ENSDART00000170575	nfat5b	0.018611393	-0.001493923	-0.392122738	-0.566909919	-0.288747412	downregulated during regeneration - mid
ENSDART00000143874	akna	0.021937256	-0.124615674	-0.323808831	-0.482322372	-0.23639171	downregulated during regeneration - mid
ENSDART00000127157	hlfa	0.048701514	-0.089881453	-0.212609643	-0.346930267	-0.147102401	downregulated during regeneration - mid
ENSDART00000126282	nr1d1	0.008088213	-0.1014969	-0.191656676	-0.494430716	-0.13667305	downregulated during regeneration - mid
ENSDART00000078529	kin	0.03060143	-1.437736057	-0.295663012	-0.493580296	-4.734617185	downregulated during regeneration - late
ENSDART00000055709	her2	0.030146589	-0.984524994	-0.833253569	-0.717745512	-1.233522642	downregulated during regeneration - late
ENSDART00000023613	her6	0.030622517	-0.335042933	-0.234567638	-0.250120727	-0.59712361	downregulated during regeneration - late
ENSDART00000086051	mecom	0.04049764	-0.550304416	-0.298852875	-0.293768303	-1.334615988	downregulated during regeneration - late
ENSDART00000110544	znf219	0.031344075	-0.234403747	-0.210413301	-0.211690001	-0.32688667	downregulated during regeneration - late
ENSDART00000053367	hmgn3	0.044180927	-0.174648563	-0.165335262	-0.310027237	-0.181225286	downregulated during regeneration - late
ENSDART00000089015	zbtb7a	0.00191624	-0.478666207	-0.203919761	-0.738823143	-0.713706626	downregulated during regeneration - late
ENSDART00000164855	crebl2	0.013882038	-0.127345071	-0.2122205	-0.432997567	-0.264872881	downregulated during regeneration - late
ENSDART00000066655	mybl1	0.005666275	-0.255810306	-0.487313899	-0.72749049	-0.668391364	downregulated during regeneration - late
ENSDART00000125174	nr1i2	0.028902271	-0.056601149	-0.085618729	-0.264588441	-0.326132843	downregulated during regeneration - late
ENSDART00000167844	mafk	0.014095383	-0.00863672	0.25450476	-0.276014619	-0.283559487	downregulated during regeneration - late
ENSDART00000057124	tefa	0.007560757	-0.17959692	0.074135039	-0.330628318	-0.262330789	downregulated during regeneration - late
ENSDART00000057125	tefa	0.002939873	-0.190705988	0.094171259	-0.306461854	-0.301060608	downregulated during regeneration - late
ENSDART00000172251	creb3l1	0.029283678	-0.33540662	0.193573394	-4.078076569	-0.842213331	downregulated during regeneration - late
ENSDART00000128815	stra13	0.002651628	0.38711598	0.144426216	-0.403589602	-0.227357691	downregulated during regeneration - late
ENSDART00000005263	usf1l	0.019856195	0.061508869	0.080355717	-0.137096705	-0.208096843	downregulated during regeneration - late
ENSDART00000044860	maff	0.002310171	0.057535561	0.039208376	-0.366680053	-0.441007369	downregulated during regeneration - late
ENSDART00000078781	znf706	0.034124552	0.015897963	-0.052514643	-0.230153549	-0.188792592	downregulated during regeneration - late

Table S6. Differentially expressed transcripts that encode transcription factors with known motifs.

Ensembl ID	zebrafish gene symbol	padj	LFC: 2dpi-0dpi	LFC: 4dpi-0dpi	LFC: 7dpi-0dpi	LFC: 12dpi-0dpi	cluster name
ENSDART00000140760	e2f7	0.044060134	0.798632306	0.496792942	-0.026056819	-0.454809271	growth toward the midline
ENSDART00000056005	ascl1a	0.032093457	1.787445127	1.283325532	0.749862779	0.792492648	growth toward the midline
ENSDART00000128488	e2f8	0.003382652	3.852661131	-0.929008264	3.252279997	2.01178299	growth toward the midline
ENSDART00000132119	max	0.028918785	0.339991432	0.197083488	0.14057043	0.318686745	growth toward the midline
ENSDART00000163794	wt1b	0.02934736	0.454982734	0.330694147	0.317167947	0.300420298	growth toward the midline
ENSDART00000033494	klf6a	0.000170861	1.084550888	1.797692613	1.625562666	0.460444617	growth toward the midline
ENSDART00000147849	klf6a	0.000870446	1.130982604	1.870693821	1.403514717	0.445060502	growth toward the midline
ENSDART00000076161	hoxb5b	0.013675681	2.652791903	3.351280664	2.618897772	0.864721852	growth toward the midline
ENSDART00000122628	junba	0.016136147	0.280813702	0.583374691	0.41181471	0.073998168	growth toward the midline
ENSDART00000146767	fosl1a	0.046117855	0.841007094	1.285901651	0.733422298	0.173089455	growth toward the midline
ENSDART00000161610	tcf3b	0.04032679	0.066966853	0.197082202	0.107652202	-0.03222147	growth toward the midline
ENSDART00000051549	tp53	0.029170799	0.110640197	0.281712522	0.180041895	-0.162598759	growth toward the midline
ENSDART00000164711	NFATC2 (1 of many)	0.017032668	0.774520656	0.760515741	0.734404519	0.027133082	growth toward the midline
ENSDART00000063912	jun	0.002341904	1.166146477	1.429328618	1.29995447	0.480560375	growth toward the midline
ENSDART00000022060	atf3	2.20E-05	2.958540036	3.348918067	2.852908333	1.811501419	growth toward the midline
ENSDART00000110040	sox11a	8.49E-05	1.573346229	1.723237215	1.23195588	0.781584101	growth toward the midline
ENSDART00000104519	stat3	0.004480141	0.6458865	0.459098575	0.438501049	0.201812376	growth toward the midline
ENSDART00000142584	alx1	0.047364517	1.361674144	1.279023188	1.723053191	-0.115335088	growth toward the midline
ENSDART00000135381	six4a	0.04980099	2.521510715	2.3671408	2.587075649	1.691172374	growth toward the midline
ENSDART00000012791	sp8a	0.049626195	1.57251014	2.331511764	2.00847442	1.039704847	growth toward the midline
ENSDART00000160644	rela	0.030637152	0.027046368	0.222531925	0.299056597	-0.13648552	growth toward the midline
ENSDART00000010248	mitfb	0.042577687	0.302243319	0.237275907	0.106756262	-0.337810921	growth toward the midline
ENSDART00000157487	tfec	0.001790465	0.534318298	0.423012313	0.205933849	-0.313187686	growth toward the midline
ENSDART00000164766	tfec	0.012357429	0.976052476	1.18639141	0.809154671	-1.643283392	growth toward the midline
ENSDART00000158466	creb3l2	0.011352496	0.073267533	0.091531466	-0.07087733	-0.32121815	growth toward the midline
ENSDART00000152378	tgif1	0.003122803	0.194631245	0.419803811	0.14379228	-0.205607529	growth toward the midline
ENSDART00000062702	cebpb	0.018433591	0.973724375	0.549827472	0.384246832	-0.627629236	growth toward the midline
ENSDART00000056457	mitfa	0.005353377	0.929754118	0.38849893	0.30836176	-0.311729197	growth toward the midline
ENSDART00000080854	stat3	0.03033326	0.856010749	0.444560766	0.379796349	-0.326021696	growth toward the midline
ENSDART00000093279	spi1b	0.002640644	0.891535687	0.563855895	0.100587929	-0.531177734	growth toward the midline
ENSDART00000036729	spi1b	0.004358158	0.910663058	0.717004711	0.095052493	-0.117318513	growth toward the midline
ENSDART00000008373	fosl1a	0.001576361	0.78271954	0.570182098	0.226810387	-0.332448721	growth toward the midline
ENSDART00000093199	tead3b	0.016523707	1.081516431	0.86043279	0.478204597	-0.215799128	growth toward the midline
ENSDART00000017185	tbx20	0.035967167	0.559146809	0.370265655	-0.151461394	-0.656442573	growth toward the midline
ENSDART00000052366	cebpa	0.035286858	0.847474055	0.814939712	0.318232429	-0.173654256	growth toward the midline

ENSDART00000005263	usf1l	0.019856195	0.061508869	0.080355717	-0.137096705	-0.208096843	growth toward the midline
ENSDART00000062854	nfe2l2a	0.008134819	0.155734951	0.146836561	-0.112595282	-0.207726174	growth toward the midline
ENSDART00000138081	bcl6ab	0.000589104	0.948060654	0.690527167	0.988530239	1.012261919	midline crossing
ENSDART00000104317	klf7b	0.000976528	0.389436838	1.156291561	1.140404424	0.652703479	midline crossing
ENSDART00000124040	insm1a	1.46E-05	0.50962443	1.551047216	1.738068624	1.104992333	midline crossing
ENSDART00000048110	six4b	0.002795018	0.757934328	2.322346312	2.776043581	1.88012445	midline crossing
ENSDART00000159652	sox4b	0.008897727	0.767109202	1.080655119	1.389017231	0.976257694	midline crossing
ENSDART00000092690	srebf2	0.003411856	-0.007030288	0.441941306	0.773565631	0.436893785	midline crossing
ENSDART00000033755	homezb	0.043076195	-0.059847052	0.060728596	0.214721864	0.105513871	midline crossing
ENSDART00000018945	meis3	0.00431294	-0.263611366	0.389783427	1.01804147	0.898863275	midline crossing
ENSDART00000075331	insm1b	0.009480138	0.180818368	0.558805202	0.852549514	0.856981121	midline crossing
ENSDART00000150128	foxp1b	0.020184087	0.347545443	0.556370195	1.342974258	1.269825931	midline crossing
ENSDART00000128174	foxp1b	0.015925333	0.060807795	0.283454627	0.694455914	0.748409918	midline crossing
ENSDART00000023959	arntl1a	7.44E-05	0.340904931	1.030627189	1.45255612	0.975945139	midline crossing
ENSDART00000026992	sox4a	3.66E-05	0.168735758	0.553498244	0.871364123	0.630648218	midline crossing
ENSDART00000083628	ddit3	0.002192622	-0.053032847	0.505000026	0.598711637	0.25299651	midline crossing
ENSDART00000110512	ybx1	0.014352201	0.187678935	0.306636132	0.543417204	0.157462635	midline crossing
ENSDART00000141068	sox11b	8.49E-05	1.611838536	2.177304965	2.010624009	1.194765222	midline crossing
ENSDART00000138821	nfil3	0.000101984	1.376309909	2.121391468	1.99718084	1.39511595	midline crossing
ENSDART00000082434	tgif1	1.75E-05	0.822910382	1.229522702	1.441745222	0.912580325	midline crossing
ENSDART00000139763	stat5a	0.018355029	3.442434617	3.964844196	3.417693165	3.322184683	midline crossing
ENSDART00000055609	atf4b	0.027440234	0.138344185	0.51758353	0.353617358	0.087935611	midline crossing
ENSDART00000152636	clocka	0.010610046	0.008631834	0.196030905	0.593132542	0.426491394	target selection
ENSDART00000166976	esrrb	0.030820243	-0.481593528	-0.114040699	0.229959907	0.235353141	target selection
ENSDART00000066317	foxn2b	0.033486585	-0.194754205	-0.013664444	0.221561118	-0.080707177	target selection
ENSDART00000104523	arntl1b	0.000255127	-0.158144123	0.59291526	1.113237486	0.52944604	target selection
ENSDART00000133959	clockb	0.010583972	-0.261160651	-0.146340415	0.224249915	0.15359596	target selection
ENSDART00000047724	zeb1b	0.032175544	-0.128023017	-0.020882446	0.202453179	0.103960026	target selection
ENSDART00000085728	klf8	0.014069453	-0.229536876	-0.061888352	0.204863755	0.114771443	target selection
ENSDART00000013605	zbtb20	0.00075553	0.021191929	0.070670156	0.462529396	0.345025042	target selection
ENSDART00000087450	klf13	0.013792445	-0.22166629	-0.059571459	0.622443971	0.288396102	target selection
ENSDART00000000280	stat1b	0.037541166	-0.005066017	0.035352481	0.819259146	0.412054224	target selection
ENSDART00000151575	thraa	0.013354221	-0.36498113	-0.584870078	0.329302923	-0.150628947	target selection
ENSDART00000154418	relb	0.010687727	-0.043359819	0.083083005	0.26390054	-0.071618881	target selection
ENSDART00000123197	relb	0.019118248	-0.261655378	-0.003226565	0.486237661	-0.265984214	target selection
ENSDART00000152489	irf3	0.006723268	-0.152502705	0.050924712	1.421424509	-0.375867622	target selection
ENSDART00000141792	stat1b	0.033143681	-1.394223287	-0.705218411	2.324563925	-0.328164239	target selection
ENSDART00000103622	irf7	0.002828188	-0.049332142	-0.2878598	1.533925917	-0.275107455	target selection

ENSDART00000148661	stat2	0.006978095	0.11967996	-0.016653911	1.354000143	0.023510931	target selection
ENSDART00000005720	stat1a	0.010250585	0.035880276	0.045195542	0.615137639	0.020671748	target selection
ENSDART00000156935	pbx4	0.039411387	0.960146191	0.964981474	3.716226941	3.606625876	target selection
ENSDART00000025575	clocka	0.041666185	-0.204691736	0.089686311	0.498135587	0.316118853	target selection
ENSDART00000125158	znf574	0.029152083	-0.158249462	-0.223654122	0.188172427	0.054732788	target selection
ENSDART00000092665	srebf1	0.004619636	-0.417658309	-0.274360919	0.114636817	0.022214824	target selection
ENSDART00000104751	mxi1	0.002350825	-0.384587031	-0.210909301	0.22179917	0.104111801	target selection
ENSDART00000111014	myca	0.024986086	-0.198726525	0.073855205	0.316369866	0.312548093	brain innervation
ENSDART00000083296	ZNF423	0.00814358	-0.278315489	0.051691519	0.407184973	0.489807123	brain innervation
ENSDART00000171642	dpf1	0.005353377	-0.380689139	0.060927985	0.320062308	0.358114425	brain innervation
ENSDART00000113087	hmx1	0.000694227	-0.389387565	0.021377927	0.234018679	0.270888749	brain innervation
ENSDART00000162474	irx1a	0.002931603	-1.132797992	-0.349993381	0.581252213	0.55366573	brain innervation
ENSDART00000162850	irx3a	0.000148815	-0.694816328	-0.301932125	0.187764734	0.208405543	brain innervation
ENSDART00000053782	scrt2	0.003310987	-0.252422437	-0.133353661	0.196438899	0.160308382	brain innervation
ENSDART00000166834	RFX7 (1 of many)	0.009820514	-0.017124727	-0.055450521	0.18457609	0.267891323	brain innervation
ENSDART00000111303	rfx7	0.00161581	0.075232989	0.085195342	0.459783838	0.727409902	brain innervation
ENSDART00000131126	bcl6a	0.005688069	-0.096649799	-0.044696983	0.295011601	0.436349263	brain innervation
ENSDART00000065139	gfi1ab	0.023335678	-0.252440054	-0.123023724	0.269699656	0.68808671	brain innervation
ENSDART00000012862	isl2a	3.29E-05	-0.663813396	0.011664777	0.746201858	1.074694305	brain innervation
ENSDART00000025031	pou4f1	1.46E-05	-0.37458842	-0.169318799	0.774588614	1.160187758	brain innervation
ENSDART00000016303	irx2a	4.96E-05	-0.381386746	0.064410059	0.752093904	0.989751717	brain innervation
ENSDART00000130163	nr2f2	0.012656704	-0.280168769	-0.057880311	0.350863039	0.468160546	brain innervation
ENSDART00000122101	tbx2b	0.001190205	-0.056468629	0.017248499	0.19422425	0.382938949	brain innervation
ENSDART00000150068	sox6	0.001331938	-0.111707858	-0.047191704	0.355284385	0.700337568	brain innervation
ENSDART00000062982	foxo3b	0.005646833	-0.232285352	-0.219543276	-0.013673206	0.18814406	brain innervation
ENSDART00000030773	foxo3a	0.000701449	-0.522674186	-0.346284342	-0.025883071	0.086783781	brain innervation
ENSDART00000100658	esrra	8.74E-05	-0.571475474	-0.395296451	0.169003635	0.212368184	brain innervation
ENSDART00000108775	ubp1	0.021499635	-0.192682892	-0.115240547	0.10453418	0.097860664	brain innervation
ENSDART00000074833	rx3	0.010379183	-0.279918529	-0.301637969	0.143576872	0.172520697	brain innervation
ENSDART00000006737	sox5	0.038386468	-0.253791611	-0.278700286	0.184042389	0.275951824	brain innervation
ENSDART00000011890	zbtb22a	0.03843558	-0.42030539	-0.536466631	0.147549186	0.05725602	brain innervation
ENSDART00000001795	sp3a	0.0407578	-0.269877105	-0.305437708	-0.047306155	-0.033496274	brain innervation
ENSDART00000018603	tbx4	0.04873911	-0.302508569	-0.188111077	-0.131947864	0.000377602	brain innervation
ENSDART00000003884	mynn	0.033340088	-0.108806257	-0.247003557	0.197551703	0.151198862	brain innervation
ENSDART00000104327	vsx1	0.00488592	-0.248525466	-0.619641572	-0.034766195	-0.028538746	brain innervation
ENSDART00000165223	pbx1b	0.015188807	-0.243991197	-0.377373898	-0.18693501	0.141565589	brain innervation
ENSDART00000167440	zfhx3	0.018342429	-0.067144102	-0.226395351	0.21751863	0.266797189	brain innervation
ENSDART00000167538	lcor	0.031258003	-0.151917658	-0.14482463	0.130691961	0.223379898	brain innervation

ENSDART00000013148	pou3f1	0.00849815	-0.360437586	-0.322601739	-0.060819622	0.211329038	brain innervation
ENSDART00000109356	klf7a	0.000177237	-0.7063233	-0.659659661	-0.044315321	0.157900055	brain innervation
ENSDART00000138048	pbx3b	0.007841242	-0.599655595	-0.602159282	-0.228775798	-0.019503135	brain innervation
ENSDART00000136245	cux1a	0.013366886	-0.163227222	-0.237382603	-0.00302824	0.164419575	brain innervation
ENSDART00000170631	ebf1a	0.016255169	-0.245696473	-0.326200552	-0.132641619	0.115308885	brain innervation
ENSDART00000064833	mafaa	0.002047801	-0.957052225	-1.143383392	-0.379735148	0.067354966	brain innervation
ENSDART00000004548	barhl1b	0.009605408	-2.472825095	-1.41656094	-0.268019579	0.230183047	brain innervation
ENSDART00000006380	tbx3a	0.004106215	-0.244591708	-0.352646993	-0.144447405	0.06961498	brain innervation
ENSDART00000151904	foxn3	0.000466769	-0.402174688	-0.533042351	-0.141780798	0.022724604	brain innervation
ENSDART00000080808	six3a	0.000687981	-0.437705937	-0.430575558	-0.140593988	-0.07114268	brain innervation
ENSDART00000171594	mef2aa	0.010231208	-0.649844171	-0.685014218	-0.301258241	-0.003741614	brain innervation
ENSDART00000136729	ebf1b	0.021027344	-2.79991744	-3.068779235	-0.720206417	0.313112936	brain innervation
ENSDART00000162945	sox5	0.023596757	-0.177385379	-0.174200373	-0.003535976	0.152149827	brain innervation
ENSDART00000132589	mef2d	0.028030725	-0.20910625	-0.209165039	-0.020444617	0.107291243	brain innervation
ENSDART00000165698	pbx1a	0.000272902	-0.205212048	-0.330154536	0.047550564	0.321002436	brain innervation
ENSDART00000144894	bcl11ba	3.52E-05	-0.656989822	-0.679320488	0.181338486	0.623847694	brain innervation
ENSDART00000006612	tbr1b	2.95E-05	-1.166029945	-1.008207372	0.617040877	1.169470893	brain innervation
ENSDART00000154646	bcl11ba	0.001677265	-0.533670184	-0.516802708	0.152594691	0.570166001	brain innervation
ENSDART00000160675	dpf1	0.00210146	-0.344267461	-0.462495684	0.239034332	0.46811192	brain innervation
ENSDART00000144186	pou6f2	7.05E-05	-0.474463335	-0.465571046	0.092941552	0.436081081	brain innervation
ENSDART00000124112	pou4f2	2.95E-05	-1.368026557	-1.308261738	0.344262724	0.908586045	brain innervation
ENSDART00000051693	irx4a	0.000591404	-1.596922819	-2.014349322	0.164610846	0.924604512	brain innervation
ENSDART00000149175	pou4f3	3.34E-05	-1.520089487	-1.231379123	0.357488642	0.743891028	brain innervation
ENSDART00000055936	isl2b	4.92E-05	-1.527471977	-1.312125436	0.470803449	0.853726063	brain innervation
ENSDART00000165609	barhl2	0.000401616	-0.31272019	-0.374018735	0.126371762	0.224852149	brain innervation
ENSDART00000010896	isl1	0.002539705	-0.281634356	-0.275090348	0.147086866	0.205392134	brain innervation
ENSDART00000100508	ebf1b	0.010170292	-0.252122108	-0.356560297	-0.077233007	0.218128715	brain innervation
ENSDART00000081170	cux1a	0.000900234	-0.092953946	-0.2283391	0.138990685	0.296890549	brain innervation
ENSDART00000162485	pax6a	0.013416969	-0.383059238	-0.321515523	-0.107334897	0.077147684	brain innervation
ENSDART00000165774	pax6a	0.007156543	-0.382942423	-0.218376905	0.094924321	0.282463268	brain innervation
ENSDART00000142087	foxp2	0.000565675	-0.522019738	-0.4194261	0.065656049	0.27497631	brain innervation
ENSDART00000048994	pbx3b	0.000910662	-0.545498331	-0.428509616	0.129533791	0.149586816	brain innervation
ENSDART00000077157	six3b	0.008086296	-0.273810969	-0.17356253	0.033591817	0.098241195	brain innervation
ENSDART00000040672	mecp2	0.047533941	-0.242382035	-0.413395534	-0.149398689	-0.148226048	downregulated during regeneration - early
ENSDART00000037879	crx	0.018920791	-0.254014844	-0.310425293	-0.22793154	-0.020194538	downregulated during regeneration - early
ENSDART00000122429	klf15	0.006222767	-0.341190152	-0.616050077	-0.578895446	-0.337948098	downregulated during regeneration - early
ENSDART00000166313	thrb	0.0223553	-0.193382381	-0.415286369	-0.400030228	-0.136855946	downregulated during regeneration - early
ENSDART00000089748	rorb	0.030623213	-0.330969943	-0.539847879	-0.425468683	-0.138099942	downregulated during regeneration - early

ENSDART00000000876	nr4a1	0.000894056	-2.262372442	-2.399082592	-2.145415905	-1.543723254	downregulated during regeneration - early
ENSDART00000153187	thrab	0.001402519	-0.362356661	-0.551240408	-0.403932333	-0.192068079	downregulated during regeneration - early
ENSDART00000054736	bhlhe23	0.004928313	-0.369485488	-0.514619457	-0.390416516	-0.190127988	downregulated during regeneration - early
ENSDART00000167052	etv1	0.01072353	-0.327639473	-0.469002174	-0.32895701	-0.22464313	downregulated during regeneration - early
ENSDART00000026017	bhlhe40	0.046057741	-0.186426622	-0.329757373	-0.286559283	-0.071751826	downregulated during regeneration - early
ENSDART00000131134	si:ch211-206a7.2	0.008848885	-0.226522125	-0.472315068	-0.371394997	-0.101330709	downregulated during regeneration - early
ENSDART00000024832	stat5a	0.004077075	-0.212236095	-0.408877789	-0.375060924	-0.185569558	downregulated during regeneration - early
ENSDART00000091707	dbpa	0.017575469	-0.158262702	-0.327851995	-0.357551076	-0.129469939	downregulated during regeneration - early
ENSDART00000126966	esrrga	0.017679749	-0.248566713	-0.265310961	-0.321308408	-0.070523791	downregulated during regeneration - early
ENSDART00000127353	nr1d2b	0.027085654	-0.29103008	-0.243688493	-0.346507775	-0.100908835	downregulated during regeneration - early
ENSDART00000047541	bach1b	0.039802589	-0.343772395	-0.510091534	-0.468889885	-0.333515696	downregulated during regeneration - early
ENSDART00000007630	nhlh2	0.010056242	-0.28794611	-0.324673306	-0.385652145	-0.261092419	downregulated during regeneration - early
ENSDART00000129498	mef2d	0.03679078	-0.188451231	-0.381445449	-0.263610552	-0.197973462	downregulated during regeneration - early
ENSDART00000164349	e2f4	0.042377891	-0.246759183	-0.226331986	-0.10688884	-0.101679787	downregulated during regeneration - early
ENSDART00000157890	tcf7l1b	0.035686017	-4.555123239	-4.673271453	-1.829433189	-0.732249154	downregulated during regeneration - early
ENSDART00000165757	pax6b	0.019981901	-1.030621281	-0.736504391	-0.510180699	-0.112500923	downregulated during regeneration - early
ENSDART00000169609	tefb	0.032368681	-0.156114581	-0.555141888	-0.301378906	-0.24132076	downregulated during regeneration - early
ENSDART00000103640	hey1	0.023203992	-0.385659584	-0.747117397	-0.392306381	-0.416692063	downregulated during regeneration - early
ENSDART00000013003	tfap2b	0.008192536	-0.299930992	-0.578681033	-0.25971747	-0.279510711	downregulated during regeneration - early
ENSDART00000082346	tfap2a	0.038054224	-0.238141907	-0.307957291	-0.242159487	-0.230339237	downregulated during regeneration - early
ENSDART00000147658	bhlhe22	0.01205445	-0.302235414	-0.31899187	-0.305987775	-0.102192149	downregulated during regeneration - early
ENSDART00000057644	lhx4	0.048389146	-0.267668007	-0.268543602	-0.190125613	-0.119751289	downregulated during regeneration - early
ENSDART00000009938	tcf12	0.025246927	-0.385864267	-0.351608927	-0.283149928	-0.323464659	downregulated during regeneration - early
ENSDART00000065361	etv5b	0.019698661	-0.645686026	-0.347502384	-0.293939032	-0.317696675	downregulated during regeneration - early
ENSDART00000055709	her2	0.030146589	-0.984524994	-0.833253569	-0.717745512	-1.233522642	downregulated during regeneration - early
ENSDART00000055706	her15.1	0.021144178	-1.483608056	-1.154441068	-0.698110478	-1.436427969	downregulated during regeneration - early
ENSDART00000133487	fosb	0.034512759	-0.882572207	-1.273781011	-0.772506712	-0.914864674	downregulated during regeneration - early
ENSDART00000151127	thraa	0.039890399	-0.798583389	-0.802364861	-0.351487338	-0.589241182	downregulated during regeneration - early
ENSDART00000153167	hlfb	0.010966965	-0.470839666	-0.326611575	-0.235752692	-0.264047019	downregulated during regeneration - early
ENSDART00000086051	mecom	0.04049764	-0.550304416	-0.298852875	-0.293768303	-1.334615988	downregulated during regeneration - early
ENSDART00000023613	her6	0.030622517	-0.335042933	-0.234567638	-0.250120727	-0.59712361	downregulated during regeneration - early
ENSDART00000125174	nr1i2	0.028902271	-0.056601149	-0.085618729	-0.264588441	-0.326132843	downregulated during regeneration - late
ENSDART00000089015	zbtb7a	0.00191624	-0.478666207	-0.203919761	-0.738823143	-0.713706626	downregulated during regeneration - late
ENSDART00000044860	maff	0.002310171	0.057535561	0.039208376	-0.366680053	-0.441007369	downregulated during regeneration - late
ENSDART00000167844	mafk	0.014095383	-0.00863672	0.25450476	-0.276014619	-0.283559487	downregulated during regeneration - late
ENSDART00000057124	tefa	0.007560757	-0.17959692	0.074135039	-0.330628318	-0.262330789	downregulated during regeneration - late
ENSDART00000057125	tefa	0.002939873	-0.190705988	0.094171259	-0.306461854	-0.301060608	downregulated during regeneration - late
ENSDART00000163250	mef2cb	0.003671994	0.361224616	-0.300957895	-0.57227951	-0.080214844	downregulated during regeneration - late

ENSDART00000131731	mef2ca	0.006008543	0.234535922	-0.250149626	-0.381098175	-0.047609857	downregulated during regeneration - late
ENSDART00000161387	tcf12	0.008531138	0.370378706	0.009395421	-0.374363269	-0.152945712	downregulated during regeneration - late
ENSDART00000127099	nr2e3	0.030676359	0.281603482	0.002911001	-0.560613973	-0.033774044	downregulated during regeneration - late
ENSDART00000066655	mybl1	0.005666275	-0.255810306	-0.487313899	-0.72749049	-0.668391364	downregulated during regeneration - late
ENSDART00000172251	creb3l1	0.029283678	-0.33540662	0.193573394	-4.078076569	-0.842213331	downregulated during regeneration - late
ENSDART00000170575	nfat5b	0.018611393	-0.001493923	-0.392122738	-0.566909919	-0.288747412	downregulated during regeneration - late
ENSDART00000148106	mef2aa	0.026582721	-0.081330082	-0.55524128	-0.609035118	-0.185252531	downregulated during regeneration - late
ENSDART00000075070	hsf2	0.014034602	-0.066635095	-0.276496416	-0.474097609	-0.186498896	downregulated during regeneration - late
ENSDART00000148353	usf2	0.006964046	-0.107987138	-0.265954127	-0.405201274	-0.200197809	downregulated during regeneration - late
ENSDART00000061106	bhlhe41	0.000553147	0.043056173	-0.423617996	-0.754959095	-0.246095493	downregulated during regeneration - late
ENSDART00000139102	dbpb	0.001993937	0.113164671	-0.364559303	-0.423196921	-0.137052116	downregulated during regeneration - late
ENSDART00000077839	atf7b	0.014656899	-0.232485852	-0.196508033	-0.391624214	-0.206346003	downregulated during regeneration - late
ENSDART00000045374	smad3a	0.043076195	-0.063329624	-0.157712113	-0.303211349	-0.108940629	downregulated during regeneration - late
ENSDART00000093331	rreb1a	0.018126285	-0.024901133	-0.155149005	-0.517234878	-0.050769944	downregulated during regeneration - late
ENSDART00000148537	roraa	0.01612441	-0.160828233	-0.221872095	-0.45696721	-0.083100906	downregulated during regeneration - late
ENSDART00000123970	mntb	0.011725523	-0.115861458	-0.101461538	-0.360694649	-0.01567692	downregulated during regeneration - late
ENSDART00000127157	hlfa	0.048701514	-0.089881453	-0.212609643	-0.346930267	-0.147102401	downregulated during regeneration - late
ENSDART00000126282	nr1d1	0.008088213	-0.1014969	-0.191656676	-0.494430716	-0.13667305	downregulated during regeneration - late

Table S7. Transcription factors that are differentially expressed during regeneration.

ENSEMBL ID	zebrafish gene symbol	cluster order	HGNC symbol	motif status	DNA binding domain	#motifs
ENSDART00000128815	stra13	1	CENPX	n	Unknown	0
ENSDART00000098285	atf5a	1	ATF5	n	bZIP	0
ENSDART00000062576	thyn1	1	THYN1	n	Unknown	0
ENSDART00000013409	prmt3	1	PRMT3	n	C2H2 ZF	0
ENSDART00000058277	znf800b	1	ZNF800	n	C2H2 ZF	0
ENSDART00000103982	nfe2l1a	1	NFE2L1	n	bZIP	0
ENSDART00000081468	ccdc79	1	TERB1	n	bZIP	0
ENSDART00000155066	atf5b	1	ATF5	n	bZIP	0
ENSDART00000114288	foxp4	1	FOXP4	n	Forkhead	0
ENSDART00000086053	PRDM16	1	PRDM16	n	C2H2 ZF	0
ENSDART00000140760	e2f7	1	E2F7	y	E2F	1
ENSDART00000056005	ascl1a	1	ASCL1	y	bHLH	1
ENSDART00000128488	e2f8	1	E2F8	y	E2F	1
ENSDART00000132119	max	1	MAX	y	bHLH	4
ENSDART00000163794	wt1b	1	WT1	y	C2H2 ZF	8
ENSDART00000033494	klf6a	1	KLF6	y	C2H2 ZF	39
ENSDART00000147849	klf6a	1	KLF6	y	C2H2 ZF	39
ENSDART00000076161	hoxb5b	1	HOXB5	y	Homeodomain	1
ENSDART00000122628	junba	1	JUNB	y	bZIP	10
ENSDART00000146767	fosl1a	1	FOSL1	y	bZIP	6
ENSDART00000161610	tcf3b	1	TCF3	y	bHLH	3
ENSDART00000051549	tp53	1	TP53	y	p53	3
ENSDART00000164711	NFATC2	1	NFATC2	y	Rel	1
ENSDART00000063912	jun	1	JUN	y	bZIP	12
ENSDART00000022060	atf3	1	ATF3	y	bZIP	1
ENSDART00000110040	sox11a	1	SOX11	y	HMG/Sox	1
ENSDART00000104519	stat3	1	STAT3	y	STAT	2
ENSDART00000142584	alx1	1	ALX1	y	Homeodomain	1
ENSDART00000135381	six4a	1	SIX4	y	Homeodomain	24
ENSDART00000012791	sp8a	1	SP8	y	C2H2 ZF	1
ENSDART00000160644	rela	1	RELA	y	Rel	1
ENSDART00000010248	mitfb	1	MITF	y	bHLH	2
ENSDART00000157487	tfec	1	TFEC	y	bHLH	1
ENSDART00000164766	tfec	1	TFEC	y	bHLH	1
ENSDART00000158466	creb3l2	1	CREB3L2	y	bZIP	1
ENSDART00000152378	tgif1	1	TGIF1	y	Homeodomain	1
ENSDART00000062702	cebpb	1	CEBPB	y	bZIP	2
ENSDART00000056457	mitfa	1	MITF	y	bHLH	2
ENSDART00000080854	stat3	1	STAT3	y	STAT	2
ENSDART00000093279	spi1b	1	SPIB	y	Ets	1
ENSDART00000036729	spi1b	1	SPIB	y	Ets	1
ENSDART00000008373	fosl1a	1	FOSL1	y	bZIP	6
ENSDART00000093199	tead3b	1	TEAD3	y	TEA	1
ENSDART00000017185	tbx20	1	TBX20	y	T-box	1
ENSDART00000052366	cebpa	1	CEBPA	y	bZIP	4
ENSDART00000005263	usf1l	1	USF1	y	bHLH	2
ENSDART00000062854	nfe2l2a	1	NFE2L2	y	bZIP	2
ENSDART00000016135	nfe2l3	2	NFE2L3	n	bZIP	0
ENSDART00000123263	phf20a	2	PHF20	n	AT hook	0
ENSDART00000146180	csrnp2	2	CSRNP2	n	Unknown	0
ENSDART00000013961	mycla	2	MYCL	n	bHLH	0
ENSDART00000018150	neurod6b	2	NEUROD6	n	bHLH	0
ENSDART00000165656	mxd3	2	MXD3	n	bHLH	0
ENSDART00000114473	trafd1	2	TRAFD1	n	C2H2 ZF	0
ENSDART00000158598	si:ch211-232b12.5	2	ZHX3	n	#N/A	0
ENSDART00000151044	baz2ba	2	BAZ2B	n	MBD	0
ENSDART00000141941	zfpm2a	2	ZFPM2	n	C2H2 ZF	0
ENSDART00000109552	baz2ba	2	BAZ2B	n	MBD	0
ENSDART00000029843	vezf1a	2	VEZF1	n	C2H2 ZF	0
ENSDART00000041877	csrnp1a	2	CSRNP1	n	Unknown	0
ENSDART00000170865	nme2b.1	2	NME2	n	Unknown	0
ENSDART00000018743	phf20a	2	PHF20	n	AT hook	0
ENSDART00000099849	arntl2	2	ARNTL2	n	bHLH	0
ENSDART00000149074	sall1b	2	SALL1	n	C2H2 ZF	0
ENSDART00000153227	nfe2l1b	2	NFE2L1	n	bZIP	0
ENSDART00000024872	creb3l3l	2	CREB3L3	n	bZIP	0
ENSDART00000123559	znf521	2	ZNF521	n	C2H2 ZF	0
ENSDART00000077484	zhx2a	2	ZHX2	n	Homeodomain	0
ENSDART00000138081	bcl6ab	2	BCL6	y	C2H2 ZF	1
ENSDART00000104317	klf7b	2	KLF7	y	C2H2 ZF	38
ENSDART00000124040	insm1a	2	INSM1	y	C2H2 ZF	1
ENSDART00000048110	six4b	2	SIX4	y	Homeodomain	24
ENSDART00000159652	sox4b	2	SOX4	y	HMG/Sox	1
ENSDART00000092690	srebf2	2	SREBF2	y	bHLH	1
ENSDART00000033755	homezb	2	HOMEZ	y	Homeodomain	1
ENSDART00000018945	meis3	2	MEIS3	y	Homeodomain	1
ENSDART00000075331	insm1b	2	INSM1	y	C2H2 ZF	1
ENSDART00000150128	foxp1b	2	FOXP1	y	Forkhead	2
ENSDART00000128174	foxp1b	2	FOXP1	y	Forkhead	2
ENSDART00000023959	arntl1a	2	ARNTL	y	bHLH	1
ENSDART00000026992	sox4a	2	SOX4	y	HMG/Sox	1
ENSDART00000083628	ddit3	2	DDIT3	y	bZIP	1
ENSDART00000110512	ybx1	2	YBX1	y	CSD	4
ENSDART00000141068	sox11b	2	SOX11	y	HMG/Sox	1
ENSDART00000138821	nfil3	2	NFIL3	y	bZIP	1
ENSDART00000082434	tgif1	2	TGIF1	y	Homeodomain	1
ENSDART00000139763	stat5a	2	STAT5A	y	STAT	1
ENSDART00000055609	atf4b	2	ATF4	y	bZIP	1
ENSDART00000172990	znf628	3	ZNF628	n	C2H2 ZF	0
ENSDART00000112756	ZNF608	3	ZNF608	n	C2H2 ZF	0

DE transcript cluster	DETF cluster order	total TFs	TFs with motifs
1clust5	1	40	31
2clust4	1,2	76	48
3clust2	3	41	18
4clust6	4	75	51
5clust7	5	62	34
6clust3	6	41	23
7clust1	6	41	23

ENSDART00000090226	znf319b	3	ZNF319	n	C2H2 ZF	0
ENSDART00000098859	neurod6a	3	NEUROD6	n	bHLH	0
ENSDART00000031167	tfap2d	3	TFAP2D	n	AP-2	0
ENSDART00000166502	satb2	3	SATB2	n	CUT; Homeodomain	0
ENSDART00000167324	ebf3a	3	EBF3	n	EBF1	0
ENSDART00000157855	ebf3a	3	EBF3	n	EBF1	0
ENSDART00000158634	ebf3a	3	EBF3	n	EBF1	0
ENSDART00000128504	si:dkey-106g10.7	3	C11orf95	n	#N/A	0
ENSDART00000109493	tshz2	3	TSHZ2	n	C2H2 ZF	0
ENSDART00000058685	zfpm2a	3	ZFPM2	n	C2H2 ZF	0
ENSDART00000110069	CABZ01075131.1	3	CSRNP3	n	Unknown	0
ENSDART00000166565	tshz3b	3	TSHZ3	n	C2H2 ZF	0
ENSDART00000123147	zbtb8a	3	ZBTB8A	n	C2H2 ZF	0
ENSDART00000084819	arhgap35b	3	ARHGAP35	n	Unknown	0
ENSDART00000104712	hmg20a	3	HMG20A	n	HMG/Sox	0
ENSDART00000135647	si:ch211-113e8.10	3	ZFP91	n	C2H2 ZF	0
ENSDART00000165877	purg	3	PURG	n	Unknown	0
ENSDART00000089076	dot1l	3	DOT1L	n	AT hook	0
ENSDART00000140284	zbtb16a	3	ZBTB16	n	C2H2 ZF	0
ENSDART00000165955	zhx3	3	ZHX3	n	#N/A	0
ENSDART00000037516	znf827	3	ZNF827	n	C2H2 ZF	0
ENSDART00000060193	thap3	3	THAP3	n	THAP finger	0
ENSDART00000154730	tsc22d1	3	TSC22D1	n	Unknown	0
ENSDART00000152636	clocka	3	CLOCK	y	bHLH	1
ENSDART00000166976	esrrb	3	ESRRB	y	Nuclear receptor	3
ENSDART00000066317	foxn2b	3	FOXN2	y	Forkhead	11
ENSDART00000104523	arntl1b	3	ARNTL	y	bHLH	1
ENSDART00000133959	clockb	3	CLOCK	y	bHLH	1
ENSDART00000047724	zeb1b	3	ZEB1	y	C2H2 ZF; Homeodomain	3
ENSDART00000085728	klf8	3	KLF8	y	C2H2 ZF	38
ENSDART00000013605	zbtb20	3	ZBTB20	y	C2H2 ZF	2
ENSDART00000087450	klf13	3	KLF13	y	C2H2 ZF	1
ENSDART00000000280	stat1b	3	STAT1	y	STAT	4
ENSDART00000151575	thraa	3	THRA	y	Nuclear receptor	15
ENSDART00000154418	relb	3	RELB	y	Rel	1
ENSDART00000123197	relb	3	RELB	y	Rel	1
ENSDART00000152489	irf3	3	IRF3	y	IRF	1
ENSDART00000141792	stat1b	3	STAT1	y	STAT	4
ENSDART00000103622	irf7	3	IRF7	y	IRF	1
ENSDART00000148661	stat2	3	STAT2	y	STAT	1
ENSDART00000005720	stat1a	3	STAT1	y	STAT	4
ENSDART00000156935	pbx4	3	PBX4	y	Homeodomain	15
ENSDART00000025575	clocka	3	CLOCK	y	bHLH	1
ENSDART00000125158	znf574	3	ZNF574	y	C2H2 ZF	2
ENSDART00000092665	srebf1	3	SREBF1	y	bHLH	1
ENSDART00000104751	mxi1	3	MXI1	y	bHLH	1
ENSDART00000101316	znf618	4	ZNF618	n	C2H2 ZF	0
ENSDART00000112529	znf319a	4	ZNF319	n	C2H2 ZF	0
ENSDART00000172410	jazf1a	4	JAZF1	n	C2H2 ZF	0
ENSDART00000089923	znf652	4	ZNF652	n	C2H2 ZF	0
ENSDART00000101070	dachd	4	DACH2	n	Unknown	0
ENSDART00000114246	adnpb	4	ADNP2	n	Homeodomain	0
ENSDART00000023038	dacha	4	DACH2	n	Unknown	0
ENSDART00000045628	irx6a	4	IRX6	n	Homeodomain	0
ENSDART00000145072	neurod4	4	NEUROD4	n	bHLH	0
ENSDART00000109347	nacc2	4	NACC2	n	Unknown	0
ENSDART00000008906	znf503	4	ZNF503	n	C2H2 ZF	0
ENSDART00000155866	zbtb16b	4	ZBTB16	n	C2H2 ZF	0
ENSDART00000164488	mbnl2	4	MBNL2	n	CCCH ZF	0
ENSDART00000160784	satb1b	4	SATB1	n	CUT; Homeodomain	0
ENSDART00000101631	satb1b	4	SATB1	n	CUT; Homeodomain	0
ENSDART00000137355	tet3	4	TET3	n	CxxC	0
ENSDART00000007806	zbtb16a	4	ZBTB16	n	C2H2 ZF	0
ENSDART00000046663	camta1b	4	CAMTA1	n	CG-1	0
ENSDART00000104790	znf292b	4	ZNF292	n	C2H2 ZF	0
ENSDART00000084035	znf532	4	ZNF532	n	C2H2 ZF	0
ENSDART00000134064	mxd1	4	MXD1	n	bHLH	0
ENSDART00000136759	prdm13	4	PRDM13	n	C2H2 ZF	0
ENSDART00000156464	prr12b	4	PRR12	n	AT hook	0
ENSDART00000153150	myt1la	4	MYT1L	n	C2H2 ZF	0
ENSDART00000171091	zeb2b	4	ZEB2	n	C2H2 ZF; Homeodomain	0
ENSDART00000131714	mbnl2	4	MBNL2	n	CCCH ZF	0
ENSDART00000124534	mbnl2	4	MBNL2	n	CCCH ZF	0
ENSDART00000164643	ZNF609 (1 of many)	4	ZNF609	n	C2H2 ZF	0
ENSDART00000155666	znf576.1	4	ZNF576	n	C2H2 ZF	0
ENSDART00000111014	myca	4	MYC	y	bHLH	4
ENSDART00000083296	ZNF423	4	ZNF423	y	C2H2 ZF	1
ENSDART00000171642	dpf1	4	DPF1	y	C2H2 ZF	2
ENSDART00000113087	hmx1	4	HMX1	y	Homeodomain	1
ENSDART00000162474	irx1a	4	IRX1	y	Homeodomain	17
ENSDART00000162850	irx3a	4	IRX3	y	Homeodomain	17
ENSDART00000053782	scrt2	4	SCRT2	y	C2H2 ZF	1
ENSDART00000166834	RFX7 (1 of many)	4	RFX7	y	RFX	14
ENSDART00000111303	rfx7	4	RFX7	y	RFX	14
ENSDART00000131126	bcl6a	4	BCL6	y	C2H2 ZF	1
ENSDART00000065139	gfi1ab	4	GFI1	y	C2H2 ZF	1
ENSDART00000012862	isl2a	4	ISL2	y	Homeodomain	1
ENSDART00000025031	pou4f1	4	POU4F1	y	Homeodomain; POU	1
ENSDART00000016303	irx2a	4	IRX2	y	Homeodomain	18
ENSDART00000130163	nr2f2	4	NR2F2	y	Nuclear receptor	1
ENSDART00000122101	tbx2b	4	TBX2	y	T-box	1
ENSDART00000150068	sox6	4	SOX6	y	HMG/Sox	1
ENSDART00000062982	foxo3b	4	FOXO3	y	Forkhead	2

ENSDART00000030773	foxo3a	4	FOXO3	y	Forkhead	2
ENSDART00000100658	esrra	4	ESRRA	y	Nuclear receptor	2
ENSDART00000108775	ubp1	4	UBP1	y	Grainyhead	8
ENSDART00000074833	rx3	4	RAX	y	Homeodomain	1
ENSDART00000006737	sox5	4	SOX5	y	HMG/Sox	1
ENSDART00000011890	zbtb22a	4	ZBTB22	y	C2H2 ZF	4
ENSDART00000001795	sp3a	4	SP3	y	C2H2 ZF	1
ENSDART00000018603	tbx4	4	TBX4	y	T-box	1
ENSDART00000003884	mynn	4	MYNN	y	C2H2 ZF	1
ENSDART00000104327	vsx1	4	VSX1	y	Homeodomain	1
ENSDART00000165223	pbx1b	4	PBX1	y	Homeodomain	1
ENSDART00000167440	zfhx3	4	ZFHX3	y	C2H2 ZF; Homeodomain	2
ENSDART00000167538	lcor	4	LCOR	y	Pipsqueak	4
ENSDART00000013148	pou3f1	4	POU3F1	y	Homeodomain; POU	1
ENSDART00000109356	klf7a	4	KLF7	y	C2H2 ZF	38
ENSDART00000138048	pbx3b	4	PBX3	y	Homeodomain	1
ENSDART00000136245	cux1a	4	CUX1	y	CUT; Homeodomain	1
ENSDART00000170631	ebf1a	4	EBF1	y	EBF1	3
ENSDART00000064833	mafaa	4	MAFA	y	bZIP	25
ENSDART00000004548	barhl1b	4	BARHL1	y	Homeodomain	1
ENSDART00000006380	tbx3a	4	TBX3	y	T-box	14
ENSDART00000151904	foxn3	4	FOXN3	y	Forkhead	11
ENSDART00000080808	six3a	4	SIX3	y	Homeodomain	1
ENSDART00000171594	mef2aa	4	MEF2A	y	MADS box	3
ENSDART00000136729	ebf1b	4	EBF1	y	EBF1	3
ENSDART00000162945	sox5	4	SOX5	y	HMG/Sox	1
ENSDART00000132589	mef2d	4	MEF2D	y	MADS box	1
ENSDART00000165698	pbx1a	4	PBX1	y	Homeodomain	1
ENSDART00000144894	bcl11ba	4	BCL11B	y	C2H2 ZF	3
ENSDART00000006612	tbr1b	4	TBR1	y	T-box	1
ENSDART00000154646	bcl11ba	4	BCL11B	y	C2H2 ZF	3
ENSDART00000160675	dpf1	4	DPF1	y	C2H2 ZF	2
ENSDART00000144186	pou6f2	4	POU6F2	y	Homeodomain; POU	1
ENSDART00000124112	pou4f2	4	POU4F2	y	Homeodomain; POU	1
ENSDART00000051693	irx4a	4	IRX4	y	Homeodomain	17
ENSDART00000149175	pou4f3	4	POU4F3	y	Homeodomain; POU	1
ENSDART00000055936	isl2b	4	ISL2	y	Homeodomain	1
ENSDART00000165609	barhl2	4	BARHL2	y	Homeodomain	1
ENSDART00000010896	isl1	4	ISL1	y	Homeodomain	10
ENSDART00000100508	ebf1b	4	EBF1	y	EBF1	3
ENSDART00000081170	cux1a	4	CUX1	y	CUT; Homeodomain	1
ENSDART00000162485	pax6a	4	PAX6	y	Homeodomain; Paired box	1
ENSDART00000165774	pax6a	4	PAX6	y	Homeodomain; Paired box	1
ENSDART00000142087	foxp2	4	FOXP2	y	Forkhead	1
ENSDART00000048994	pbx3b	4	PBX3	y	Homeodomain	1
ENSDART00000077157	six3b	4	SIX3	y	Homeodomain	1
ENSDART00000022010	hivep2b	5	HIVEP2	n	C2H2 ZF	0
ENSDART00000136488	zmat4a	5	ZMAT4	n	C2H2 ZF	0
ENSDART00000151571	ahdc1	5	AHDC1	n	AT hook	0
ENSDART00000059446	znf385b	5	ZNF385B	n	C2H2 ZF	0
ENSDART00000165120	purab	5	PURA	n	Unknown	0
ENSDART00000172199	zmat4a	5	ZMAT4	n	C2H2 ZF	0
ENSDART00000166540	kcnip3b	5	KCNIP3	n	Unknown	0
ENSDART00000111842	sall1a	5	SALL1	n	C2H2 ZF	0
ENSDART00000150949	nfyc	5	NFYC	n	Unknown	0
ENSDART00000166351	nkrf	5	NKRF	n	Unknown	0
ENSDART00000090019	zeb2b	5	ZEB2	n	C2H2 ZF; Homeodomain	0
ENSDART00000009691	scml4	5	SCML4	n	AT hook	0
ENSDART00000170400	ahdc1	5	AHDC1	n	AT hook	0
ENSDART00000124751	kcnip3b	5	KCNIP3	n	Unknown	0
ENSDART00000003548	znf385a	5	ZNF385A	n	C2H2 ZF	0
ENSDART00000092357	sgsm2	5	SGSM2	n	BED ZF	0
ENSDART00000026865	l3mbtl1a	5	L3MBTL1	n	C2H2 ZF	0
ENSDART00000033362	gatad2b	5	GATAD2B	n	GATA	0
ENSDART00000078529	kin	5	KIN	n	C2H2 ZF	0
ENSDART00000162857	nr4a3	5	NR4A3	n	Nuclear receptor	0
ENSDART00000110544	znf219	5	ZNF219	n	C2H2 ZF	0
ENSDART00000156351	zfat	5	ZFAT	n	C2H2 ZF	0
ENSDART00000166135	zbtb47b	5	ZBTB47	n	C2H2 ZF	0
ENSDART00000048775	mbd3b	5	MBD3	n	MBD	0
ENSDART00000157659	camta1b	5	CAMTA1	n	CG-1	0
ENSDART00000142223	hivep3b	5	HIVEP3	n	C2H2 ZF	0
ENSDART00000055890	znf385c	5	ZNF385C	n	C2H2 ZF	0
ENSDART00000135443	kdm5ba	5	KDM5B	n	ARID/BRIGHT	0
ENSDART00000162387	zbtb38	5	ZBTB38	n	C2H2 ZF	0
ENSDART00000172310	zbtb4	5	ZBTB4	n	C2H2 ZF	0
ENSDART00000040672	mecp2	5	MECP2	y	MBD; AT hook	2
ENSDART00000037879	crx	5	CRX	y	Homeodomain	1
ENSDART00000122429	klf15	5	KLF15	y	C2H2 ZF	6
ENSDART00000166313	thrb	5	THRB	y	Nuclear receptor	17
ENSDART00000089748	rorb	5	RORB	y	Nuclear receptor	1
ENSDART00000000876	nr4a1	5	NR4A1	y	Nuclear receptor	1
ENSDART00000153187	thrab	5	THRA	y	Nuclear receptor	15
ENSDART00000054736	bhlhe23	5	BHLHE23	y	bHLH	1
ENSDART00000167052	etv1	5	ETV1	y	Ets	1
ENSDART00000026017	bhlhe40	5	BHLHE40	y	bHLH	2
ENSDART00000131134	si:ch211-206a7.2	5	FOXO6	y	Forkhead	1
ENSDART00000024832	stat5a	5	STAT5A	y	STAT	1
ENSDART00000091707	dbpa	5	DBP	y	bZIP	1
ENSDART00000126966	esrrga	5	ESRRG	y	Nuclear receptor	1
ENSDART00000127353	nr1d2b	5	NR1D2	y	Nuclear receptor	7
ENSDART00000047541	bach1b	5	BACH1	y	bZIP	1
ENSDART00000007630	nhlh2	5	NHLH2	y	bHLH	21

ENSDART00000129498	mef2d	5	MEF2D	y	MADS box	1
ENSDART00000164349	e2f4	5	E2F4	y	E2F	1
ENSDART00000157890	tcf7l1b	5	TCF7L1	y	HMG/Sox	1
ENSDART00000165757	pax6b	5	PAX6	y	Homeodomain; Paired box	1
ENSDART00000169609	tefb	5	TEF	y	bZIP	1
ENSDART00000103640	hey1	5	HEY1	y	bHLH	1
ENSDART00000013003	tfap2b	5	TFAP2B	y	AP-2	1
ENSDART00000082346	tfap2a	5	TFAP2A	y	AP-2	5
ENSDART00000147658	bhlhe22	5	BHLHE22	y	bHLH	1
ENSDART00000057644	lhx4	5	LHX4	y	Homeodomain	1
ENSDART00000009938	tcf12	5	TCF12	y	bHLH	1
ENSDART00000065361	etv5b	5	ETV5	y	Ets	1
ENSDART00000055709	her2	5	HES5	y	bHLH	1
ENSDART00000055706	her15.1	5	HES5	y	bHLH	1
ENSDART00000133487	fosb	5	FOSB	y	bZIP	3
ENSDART00000151127	thraa	5	THRA	y	Nuclear receptor	15
ENSDART00000153167	hlfb	5	HLF	y	bZIP	2
ENSDART00000086051	mecom	5	MECOM	y	C2H2 ZF	1
ENSDART00000023613	her6	5	HES1	y	bHLH	1
ENSDART00000031426	skilb	6	SKIL	n	Unknown	0
ENSDART00000148066	znf395b	6	ZNF395	n	C2H2 ZF	0
ENSDART00000125344	skilb	6	SKIL	n	Unknown	0
ENSDART00000131361	kcnip3b	6	KCNIP3	n	Unknown	0
ENSDART00000054020	hivep3b	6	HIVEP3	n	C2H2 ZF	0
ENSDART00000169283	znf644b	6	ZNF644	n	C2H2 ZF	0
ENSDART00000100667	skia	6	SKI	n	Unknown	0
ENSDART00000100181	sall3b	6	SALL3	n	C2H2 ZF	0
ENSDART00000141734	hivep2a	6	HIVEP2	n	C2H2 ZF	0
ENSDART00000124740	ncoa2	6	NCOA2	n	bHLH	0
ENSDART00000164082	znf609a	6	ZNF609	n	C2H2 ZF	0
ENSDART00000143165	tsc22d1	6	TSC22D1	n	Unknown	0
ENSDART00000143874	akna	6	AKNA	n	AT hook	0
ENSDART00000104279	znf516	6	ZNF516	n	C2H2 ZF	0
ENSDART00000165710	gpbp1l1	6	GPBP1L1	n	Unknown	0
ENSDART00000053367	hmgn3	6	HMGN3	n	HMG/Sox	0
ENSDART00000164855	crebl2	6	CREBL2	n	bZIP	0
ENSDART00000113286	phf19	6	PHF1	n	Unknown	0
ENSDART00000078781	znf706	6	ZNF706	n	C2H2 ZF	0
ENSDART00000125174	nr1i2	6	NR1I2	y	Nuclear receptor	5
ENSDART00000089015	zbtb7a	6	ZBTB7A	y	C2H2 ZF	2
ENSDART00000044860	maff	6	MAFF	y	bZIP	2
ENSDART00000167844	mafk	6	MAFK	y	bZIP	2
ENSDART00000057124	tefa	6	TEF	y	bZIP	1
ENSDART00000057125	tefa	6	TEF	y	bZIP	1
ENSDART00000163250	mef2cb	6	MEF2C	y	MADS box	1
ENSDART00000131731	mef2ca	6	MEF2C	y	MADS box	1
ENSDART00000161387	tcf12	6	TCF12	y	bHLH	1
ENSDART00000127099	nr2e3	6	NR2E3	y	Nuclear receptor	1
ENSDART00000066655	mybl1	6	MYBL1	y	Myb/SANT	1
ENSDART00000172251	creb3l1	6	CREB3L1	y	bZIP	1
ENSDART00000170575	nfat5b	6	NFAT5	y	Rel	1
ENSDART00000148106	mef2aa	6	MEF2A	y	MADS box	3
ENSDART00000075070	hsf2	6	HSF2	y	HSF	1
ENSDART00000148353	usf2	6	USF2	y	bHLH	2
ENSDART00000061106	bhlhe41	6	BHLHE41	y	bHLH	1
ENSDART00000139102	dbpb	6	DBP	y	bZIP	1
ENSDART00000077839	atf7b	6	ATF7	y	bZIP	1
ENSDART00000045374	smad3a	6	SMAD3	y	SMAD	2
ENSDART00000093331	rreb1a	6	RREB1	y	C2H2 ZF	1
ENSDART00000148537	roraa	6	RORA	y	Nuclear receptor	1
ENSDART00000123970	mntb	6	MNT	y	bHLH	1
ENSDART00000127157	hlfa	6	HLF	y	bZIP	2
ENSDART00000126282	nr1d1	6	NR1D1	y	Nuclear receptor	7

Table S8. Gene ontology (GO) analysis of putative Jun transcriptional targets.

GO.ID	Term	Annotated	Significant	Expected	pvalue	Genes
GO:0003824	catalytic activity	5689	404	355.24	0.0414	abca1a;abcb4;abcf1;abcg2c;abhd14a;abhd4;abl1;acadl;acadvl;ache;acsl2;acsl4a;acsl4b;acss1;adam15;adam23a;adam8a;adam8b;adat2;adhfe1;adkb;adss;agpat4;ahcy;ahcyl1;ahcyl2;ak2;alad;aldh4a1;aldoaa;aldoca;alg6;alpk2;ampd2b;ankib1a;apobec2b;arih2;ash1l;asns;asph;atic;atp1a1b;atp2a1;atp2b2;atp6ap1b;atp6ap1la;bcat2;blmh;btk;bub1bb;cad;camk1ga;camk1gb;camk2b2;camkmt;casp9;cbsa;cdc14ab;cdc42bpab;cdc42l;cdk1;cdk2;cdkn1a;cenpe;cers2a;cers4b;ckba;clk2a;clpp;cmtr1;cndp2;colgalt2;cracr2ab;csnk1a1;csnk1e;csnk1g1;ctbp2a;cth;cthl;ctsba;ctsc;ctsz;cyb5r1;dad1;dalrd3;dclk1b;ddx54;dgat1b;dgkab;dhfr;dhx32b;dhx33;dhx57;dip2ba;dnase1l4.1;dpf2;dpf2l;dph6;dpysl2b;dpysl4;dus2;dusp27;dusp6;dync1h1;dyrk1ab;ebp;ech1;eef1a1l2;eef2b;ehhadh;eif2ak1;eif2s3;eif3ha;eif4a1b;elovl1b;entpd5b;ern2;esd;etfdh;fahd2a;fam213b;fdps;fkbp1aa;fkbp5;fkbp9;ftsj3;fuca1.1;fuca1.2;galm;galns;gars;gba;gbp1;GDPGP1;ggh;gig2o;glrx;glud1a;gna12a;gnpda2;gnsb;gpx4a;GSK3B (1 of many);gstm.1;gstm.2;gtpbp1;guk1a;hadhb;hectd3;hibadha;hmbsa;hmox1a;hpdl;hspa14;hunk;iars;icmt;idi1;ifi30;ifit14;ifit16;ilk;impa1;impdh1b;impdh2;inpp4aa;ip6k1;ippk;isg20;jak1;kat5a;kif1b;kif20bb;kif26ab;kif26bb;kif3ca;kif3cb;kif4;kif5c;klc1a;klc3;klc4;klhl21;ksr2;lgmn;lgsn;limk1a;lipf;loxl4;lrsam1;lypla1;lypla2;manba;mapk4;mapk9;marcksb;mcm2;mcm5;mcm7;megf8;melk;mettl1;METTL18;mettl2a;mical1;mknk1;moxd1;mri1;msra;mthfd1l;mtm1;mtmr7a;myo10l3;myo18aa;myo19;myo1ea;nars;nat16;nek12;nme2b.1;nme4;nme7;npl;nsdhl;nsun5;nt5c2l1;nudt4b;nudt5;nudt9;oat;otud5a;OTUD7A;oxnad1;pafah1b3;paox1;parp2;parp4;parp8;pbk;pcsk1;pde4ca;pde4cb;pde9a;pdia6;pdk2b;pfkmb;pgam1a;pgm1;pgm2;pgm5;pik3cb;pip5kl1;pitrm1;pkn1a;pla2g15;plcxd1;pld3;plk1;pmt;pnpla7a;pnpla8;polr1c;polr2eb;polr3gla;ppcdc;ppil1;ppp5c;ppt1;prkx;prmt3;prpsap1;prss16;psen2;psma5;psma6l;psmb1;psmb2;psmc1a;ptges;ptp4a2b;ptp4a3;ptpdc1b;ptpn21;ptpn23a;pygmb;pyroxd2;qars;rab10;rab11bb;rab13;rab20;rab32a;rab3ab;rab41;rab42a;rab43;rabggtb;rabl2;rac1a;ran;rap1b;rap2b;rars;rbbp9;rhbdl3;rhoca;rhot1a;rnaseka;RNF14 (1 of many);rnf19b;rnpep;rps6kb1b;rrad;rragca;rras;rras2;rrm2;rtca;sae1;sardh;sars;sccpdhb;scpep1;sec14l8;sept3;setdb1a;setdb1b;sgk494a;sh3pxd2b;shmt1;si:ch1073-296d18.1;si:ch211-117c9.1;si:ch211-188f17.1;si:ch211-195b13.1;si:ch211-195b15.8;si:ch211-282j22.3;si:ch211-63o20.7;si:ch73-127m5.1;si:dkey-16p21.7;si:dkey-204f11.64;si:dkey-222f8.3;si:dkey-226m8.10;si:dkey-30c15.17;si:dkey-32e23.4;si:dkey-9i23.14;sik1;SLA (1 of many);slc3a2a;smarca5;smarcad1b;smox;smpd1;sod3b;spast;spra;spsb4a;src;srpk1a;st3gal3b;stambpl1;steap4;sult5a1;sult6b1;suox;tars;tcirg1b;tgm1l1;tmprss9;top1mt;tpte;trmt61a;tuba2;tuba8l;tubb4b;txn;tyr;uap1l1;ube2c;ube2e1;uck2a;uck2b;ugdh;umps;uqcrq;usp18;vcp;vcpip1;wars;wdsub1;wee1;zak;zdhhc12b;zgc:101663;zgc:103438;zgc:110269;zgc:112285;zgc:152830;zgc:153031;zgc:158659;zgc:162396;zgc:171704;zgc:198419;zgc:77752;zgc:92066
GO:0016787	hydrolase activity	2346	180	146.49	0.03182	abca1a;abcb4;abcf1;abcg2c;abhd14a;abhd4;ache;adam15;adam23a;adam8a;adam8b;adat2;ahcy;ahcyl1;ahcyl2;ampd2b;apobec2b;atic;atp1a1b;atp2a1;atp2b2;atp6ap1b;atp6ap1la;blmh;cad;casp9;cdc14ab;cdc42l;cenpe;clpp;cndp2;cracr2ab;ctsba;ctsc;ctsz;ddx54;dhx32b;dhx33;dhx57;dnase1l4.1;dpysl2b;dpysl4;dusp27;dusp6;dync1h1;eef1a1l2;eef2b;eif2s3;eif3ha;eif4a1b;entpd5b;ern2;esd;fuca1.1;fuca1.2;galns;gba;gbp1;ggh;gna12a;gnpda2;gnsb;gtpbp1;hspa14;iars;idi1;impa1;inpp4aa;isg20;kif1b;kif20bb;kif26ab;kif26bb;kif3ca;kif3cb;kif4;kif5c;klc1a;klc3;klc4;lgmn;lipf;lypla1;lypla2;manba;mcm2;mcm5;mcm7;megf8;mthfd1l;mtm1;mtmr7a;myo10l3;myo18aa;myo19;myo1ea;nt5c2l1;nudt4b;nudt5;nudt9;otud5a;OTUD7A;pafah1b3;pcsk1;pde4ca;pde4cb;pde9a;plcxd1;pnpla7a;pnpla8;ppp5c;ppt1;prss16;psen2;psma5;psma6l;psmb1;psmb2;psmc1a;ptp4a2b;ptp4a3;ptpdc1b;ptpn21;ptpn23a;rab10;rab11bb;rab13;rab20;rab32a;rab3ab;rab41;rab42a;rab43;rabl2;rac1a;ran;rap1b;rap2b;rbbp9;rhbdl3;rhoca;rhot1a;rnaseka;rnpep;rrad;rragca;rras;rras2;scpep1;sec14l8;sept3;si:ch211-195b15.8;si:ch211-282j22.3;si:ch73-127m5.1;si:dkey-204f11.64;si:dkey-222f8.3;si:dkey-226m8.10;si:dkey-30c15.17;si:dkey-32e23.4;si:dkey-9i23.14;smarca5;smarcad1b;smpd1;spast;stambpl1;tcirg1b;tmprss9;tpte;tuba2;tuba8l;tubb4b;usp18;vcp;vcpip1;zgc:103438;zgc:110269;zgc:112285;zgc:152830;zgc:171704;zgc:77752
GO:0005525	GTP binding	518	47	32.35	0.0064	adss;anxa6;arf1;ARL3 (1 of many);arl8a;arl8bb;cdc42l;cracr2ab;eef1a1l2;eef2b;eif2s3;gbp1;gna12a;gnl3;gtpbp1;gtpbp4;megf8;rab10;rab11bb;rab13;rab20;rab32a;rab3ab;rab41;rab42a;rab43;rabl2;rac1a;ran;rap1b;rap2b;rhoca;rhot1a;rrad;rragca;rras;rras2;sec14l8;sept3;si:dkey-32e23.4;si:dkey-98f17.5;si:dkeyp-69e1.8;tuba2;tuba8l;tubb4b;zgc:171704;zgc:77650
GO:0008289	lipid binding	384	40	23.98	0.02919	acbd5a;adap2;aida;anxa6;apoeb;col4a3bpa;col4a3bpb;cpne3;cpne9;esyt1a;fabp11a;fabp7a;fermt2;hs1bp3;melk;osbpl1a;pacsin3;picalmb;prom1b;pxdc1a;rasal1b;rbp1;rbp2a;rbp7b;rlbp1a;sdc4;sdcbp2;sh3pxd2b;si:ch211-195b13.1;si:dkey-231j24.3;snx12;snx19a;sptbn1;sptbn2;syt4;syt5a;syt6a;tex2;vcp;vps36
GO:0003924	GTPase activity	347	37	21.67	0.00103	cdc42l;cracr2ab;eef1a1l2;eef2b;eif2s3;gbp1;gna12a;gtpbp1;megf8;rab10;rab11bb;rab13;rab20;rab32a;rab3ab;rab41;rab42a;rab43;rabl2;rac1a;ran;rap1b;rap2b;rhoca;rhot1a;rrad;rragca;rras;rras2;sec14l8;sept3;si:dkey-204f11.64;si:dkey-32e23.4;tuba2;tuba8l;tubb4b;zgc:171704

GO:0016887	ATPase activity	315	28	19.67	0.03947	abca1a;abcb4;abcf1;abcg2c;atp1a1b;atp2a1;atp2b2;atp6ap1b;atp6ap1la;cenpe;ddx54;dhx32b;dhx33;dhx57;eif4a1b;hspa14;kif1b;kif20bb;kif3ca;kif3cb;kif4;kif5c;psmc1a;si:dkey-226m8.10;smarca5;spast;tcirg1b;vcp
GO:0008017	microtubule binding	179	25	11.18	0.00647	camsap2a;camsap3;ccdc88b;ccsapb;cenpe;clasp2;dpysl2b;eml1;jakmip3;kif1b;kif20bb;kif26ab;kif26bb;kif3ca;kif3cb;kif4;kif5c;map6b;mapre1b;mapre3b;nusap1;pafah1b1a;prc1b;si:dkey-32e23.4;spast
GO:0005085	guanyl-nucleotide exchange factor activity	165	21	10.3	0.04798	arhgef10lb;arhgef2;arhgef33;arhgef4;cyth1a;dennd5b;dennd6aa;dock11;dock9b;kalrna;mcf2b;mcf2l2;mcf2la;rabif;rapgef1a;rapgef4;rasgef1bb;sec61b;sh2d3ca;si:dkey-38p12.3;tiam2a
GO:0004725	protein tyrosine phosphatase activity	112	12	6.99	0.04675	cdc14ab;dusp6;mtm1;mtmr7a;ptp4a2b;ptp4a3;ptpdc1b;ptpn21;ptpn23a;si:ch211-195b15.8;tpte;zgc:77752
GO:0019843	rRNA binding	32	11	2	0.00013	cirbpb;nop53;ppan;rpl12;rpl23;rpl5b;rpl8;rpl9;rps18;rps4x;rps9
GO:0003743	translation initiation factor activity	61	11	3.81	0.00125	eif2s1a;eif2s1b;eif2s2;eif2s3;eif3ba;eif3ha;eif3i;eif3m;eif4a1b;eif4g2a;eif6
GO:0000149	SNARE binding	90	11	5.62	0.01428	napga;snap25a;stx11a;stx11b.1;stx11b.2;stx12;stx3a;stx4;syt4;syt5a;syt6a
GO:0051082	unfolded protein binding	87	11	5.43	0.01927	cct4;cct8;hsp90aa1.2;hsp90ab1;hspa14;pfdn1;pfdn2;pfdn4;ssuh2.2;ssuh2.4;tcp1
GO:0005089	Rho guanyl-nucleotide exchange factor activity	86	10	5.37	0.0414	arhgef10lb;arhgef2;arhgef33;arhgef4;kalrna;mcf2b;mcf2l2;mcf2la;si:dkey-38p12.3;tiam2a
GO:0008138	protein tyrosine/serine/threonine phosphatase activity	57	9	3.56	0.01616	cdc14ab;dusp27;dusp6;ptp4a2b;ptp4a3;ptpdc1b;si:ch211-195b15.8;tpte;zgc:77752
GO:0005484	SNAP receptor activity	34	8	2.12	0.00095	sec22bb;snap25a;stx11a;stx11b.1;stx11b.2;stx12;stx3a;stx4
GO:0003746	translation elongation factor activity	27	7	1.69	0.00107	eef1a1l2;eef1b2;eef1db;eef1g;eef2b;eif5a2;gtpbp1
GO:0004715	non-membrane spanning protein tyrosine kinase activity	41	7	2.56	0.01262	abl1;btk;jak1;melk;SLA (1 of many);src;wee1
GO:0031492	nucleosomal DNA binding	44	7	2.75	0.01831	CU459186.1;hmgn3;si:ch211-113a14.12;si:dkey-108k21.14;si:dkey-261m9.12;zgc:153405;zgc:173552
GO:0017049	GTP-Rho binding	18	6	1.12	0.00057	anln;cdc42ep1a;cdc42ep2;cdc42ep5;pkn1a;zgc:154093
GO:0030170	pyridoxal phosphate binding	43	6	2.69	0.04941	cbsa;cth;cthl;oat;pygmb;shmt1
GO:0043022	ribosome binding	23	5	1.44	0.0448	c1qbp;eif2s1a;eif2s1b;eif5a2;eif6
GO:0051010	microtubule plus-end binding	9	4	0.56	0.00148	clasp2;mapre1b;mapre3b;pafah1b1a
GO:0044325	ion channel binding	21	4	1.31	0.03861	cbarpb;rims1b;scn1bb;si:ch211-126j24.1
GO:0031386	protein tag	10	3	0.62	0.02092	rps27a;sumo2b;uba52
GO:0070840	dynein complex binding	10	3	0.62	0.02092	cenpf;dctn1b;pafah1b1a
GO:0008474	palmitoyl-(protein) hydrolase activity	11	3	0.69	0.02745	lypla1;lypla2;ppt1
GO:0008199	ferric iron binding	12	3	0.75	0.03494	zgc:109934;zgc:198419;zgc:92066
GO:0003962	cystathionine gamma-synthase activity	2	2	0.12	0.0039	cth;cthl
GO:0004123	cystathionine gamma-lyase activity	2	2	0.12	0.0039	cth;cthl
GO:0004146	dihydrofolate reductase activity	2	2	0.12	0.0039	dhfr;zgc:153031
GO:0004614	phosphoglucomutase activity	2	2	0.12	0.0039	pgm1;pgm5
GO:0004814	arginine-tRNA ligase activity	3	2	0.19	0.0112	dalrd3;rars
GO:0004560	alpha-L-fucosidase activity	3	2	0.19	0.0112	fuca1.1;fuca1.2
GO:0003938	IMP dehydrogenase activity	3	2	0.19	0.0112	impdh1b;impdh2
GO:0046592	polyamine oxidase activity	3	2	0.19	0.0112	paox1;smox
GO:0043515	kinetochore binding	3	2	0.19	0.0112	clasp2;spdl1
GO:0046933	proton-transporting ATP synthase activity, rotational mechanism	4	2	0.25	0.02148	atp6ap1b;atp6ap1la
GO:0008097	5S rRNA binding	4	2	0.25	0.02148	nop53;rpl5b
GO:0005159	insulin-like growth factor receptor binding	5	2	0.31	0.03432	igf1;socs2
GO:0005528	FK506 binding	5	2	0.31	0.03432	fkbp1aa;fkbp5
GO:0004332	fructose-bisphosphate aldolase activity	5	2	0.31	0.03432	aldoaa;aldoca
GO:0008022	protein C-terminus binding	5	2	0.31	0.03432	pdzd11;sae1
GO:0015232	heme transporter activity	6	2	0.37	0.04938	flvcr1;flvcr2b
GO:0051011	microtubule minus-end binding	6	2	0.37	0.04938	camsap2a;camsap3
GO:0003708	retinoic acid receptor activity	6	2	0.37	0.04938	nr1d4b;rarab

Table S9. Gene ontology analysis of regeneration-associated genes with peak expression during initial axon growth toward the midline (Fig. 1C).

GO.ID	Term	Annotated	Significant	Expected	pvalue	Genes
GO:0023052	signaling	4223	838	754.01	0.03835	[illegible]1a;adgrl3.1;adm2a;adora1b;adra2a;afap1;agap1;agrn;aida;ajuba;akap12b;akt3a;aldh1a2;alg5;angptl1a;angptl2b;ank1a;ank1b;ank2a;ankrd6b;anxa6;ap2m1a;apaf1;apba1a;apc;apc2;aplnrb;appb;arap3;arhgap10;arhgap11a;arhgap12b;arhgap21a;arhgap22;arhgap23b;arhgap32a;arhgap33;arhgap35b;arhgap4b;arhgdia;arhgdig;arhgef10lb;arhgef11;arhgef2;arhgef33;arhgef4;arhgef9a;arhgef9b;arr3b;arrb1;atl1;avpr2ab;baxa;baxb;bbc3;bbs4;bcar1;bcl2b;bcl2l1;bcl6a;bcl9;bcr;bdnf;bhlhe41;bmp3;bmp4;bmpr2a;bnip3lb;bnip4;btk;BX897691.1;cab39l;cab39l1;CABZ01081780.1;cacna1aa;cacna1bb;cacna1g;cacna1ha;cacna1hb;cacna1i;cacnb1;cacnb2a;cacnb3a;cacnb3b;cacng2a;cacng3b;cacng5a;cacng7b;cacng8b;cad;cadpsb;calb2a;calb2b;calca;calm1b;calm3a;camk4;cart2;cart3;cbl;cby1;ccdc136b;ccdc28a;ccka;ccr9a;cd276;cd63;cdc42bpab;cdc42se2;cdkn1a;cdon;cds1;celsr3;cetn2;chata;chn1;chrm4a;chrm5a;chrna10a;chrna6;chrnb2a;chrnb3a;chrnb3b;chsy1;cib2;cish;citb;clstn1;clstn2;clstn3;cmklr1;cnksr2b;cntfr;col1a1a;cplx2;cplx2l;cplx3b;cplx4a;cplx4b;CR382281.1;creb3l3l;crhb;crhbp;crhr1;crlf1a;csnk1e;CT030188.1;CT990561.1;cx43;cxcr3.3;cyth1a;dab2ipa;dab2ipb;dact3a;dclk1a;dclk1b;dclk2a;dclk2b;ddit3;dedd1;def8;deptor;dgke;dgkh;dicp3.1;dicp3.3;diras1a;dkk3a;dkk3b;dlg1l;dlg3;dlg4a;dlgap1b;dlgap2a;dlgap3;dlgap4b;dlgap5;dmxl2;dock11;dock3;DOCK4 (1 of many);dock6;dock7;dock9b;dot1l;drd1a;drd1b;drd2b;drd4a;dusp5;efna1b;efna2a;efna3a;ek1;enpp1;epha4b;epha6;ephb1;ephb2a;ephb6;erbb4b;erlin2;esrra;esrrga;f3a;fam13b;fas;fermt2;fgd1;fgf11b;fgf12a;fgf12b;fgf13a;fgf14;fgf18a;fgf1a;fgf6a;fgf8a;fgfr4;fjx1;fndc5b;foxo3a;fscn1a;fto;fzd2;fzd7a;gabbr1a;gabbr1b;gabbr2;gabra1;gabra3;gabra5;gabra6b;gabrb1;gabrb2;gabrb3;gabrb4;gabrd;gabrg2;gabrr1;gabrr2a;gadd45ba;gadd45ga;galr2b;gatad2b;gc2;gcgra;gck;gdi1;gem;ghra;gid8a;gipr;gjd2b;glra1;glra2;glra3;glra4b;glrba;glrbb;gnao1a;gnao1b;GNAZ;gnb1b;gnb2;gnb3a;gnb5b;gng13b;gng5;gngt2a;gpc1a;gpc1b;gpc2;gpc3;gpr132b;gpr153;gpr158a;gpr158b;gpr186;gpr22a;gpr22b;gpr27;gpr37a;gpr37b;gpr37l1b;gpr52;gpr75;gpr78a;gpr84;gprc5ba;gprc5bb;gpx4b;grb2a;gria1b;gria2a;gria3a;gria3b;gria4a;gria4b;grid2;grik1a;grik1b;GRIK3;grik4;grin1a;grin1b;grin2aa;grin2ab;grk7a;grm1a;grm1b;grm2a;grm4;grm6a;grm6b;grm8a;grm8b;hbegfa;hbegfb;hcar1-4;hcn1;hdr;her15.1;her2;her6;hey1;hgfb;hmgn3;hmha1a;homer1b;homer3b;hrasa;hrasb;hrh3;hsp90ab1;htr2cl1;htr7c;hunk;id4;igf1;igf1rb;igf2b;il17a/f1;il17a/f3;il1rapl1b;il1rapl2;il4r.1;il6st;ilk;impa1;impa2;inhbaa;inpp5b;insra;iqgap2;iqsec3a;iqsec3b;irs2a;itga10;itga6a;itgae.2;itgav;itgb3b;itgb7;itpr1a;jagn1a;jak1;jun;jupa;kalrna;kat2a;kcnab1a;kcnab2b;kcnh1a;kcnh3;kcnh4b;kcnh5a;kcnh5b;kcnk3a;kcnk3b;kcnq3;kctd13;kctd9a;kita;klhl24b;klhl6;ksr2;lepa;lepr;lims1;lin7a;lpar1;magi2a;map2k2b;MAP3K13;map4k4;mapk11;mapk4;mapk9;mark4a;mast1a;mast3b;mcf2a;mcf2l2;mcf2la;mdka;mdkb;mecom;melk;mgll;mibp2;mknk1;mknk2a;mstnb;mtnr1aa;mtnr1ab;mtnr1ba;mtnr1bb;myo10l1;
GO:0034220	ion transmembrane transport	780	243	139.27	0.00029	ankha;anxa6;aplnrb;aqp1a.1;asic1b;asic2;asic4a;asic4b;atp1a1b;atp1a3a;atp1b1b;atp1b2a;atp1b3a;atp1b3b;atp2a1;atp2b1a;atp2b2;atp2b3a;atp2b3b;atp6ap1b;atp6ap1la;atp6v0a1b;atp6v0a2a;atp6v0e1;atp6v1ba;atp6v1f;atp6v1h;atpv0e2;best2;CABZ01081780.1;cacna1aa;cacna1bb;cacna1da;cacna1g;cacna1ha;cacna1hb;cacna1i;cacna2d2a;cacna2d2b;cacna2d4b;cacnb1;cacnb2a;cacnb3a;cacnb3b;cacng2a;cacng3b;cacng5a;cacng6b;cacng7b;cacng8b;cbarpb;chrna10a;chrna6;chrnb2a;chrnb3a;chrnb3b;clcn7;clic4;clic5a;cnga3b;cox4i2;COX7A2 (1 of many);fam155a;fxyd6l;gabra1;gabra3;gabra5;gabra6b;gabrb1;gabrb2;gabrb3;gabrb4;gabrd;gabrg2;gabrr1;gabrr2a;gem;gjd2b;glra1;glra2;glra3;glra4b;glrba;glrbb;gria2a;gria3a;gria3b;gria4a;gria4b;grid2;grik1a;grik1b;GRIK3;grik4;grin1a;grin1b;grin2aa;grin2ab;hcn1;hcn2b;hcn4l;hecw2a;htr2cl1;itpr1a;jph3;kcna1a;kcna1b;kcna2a;kcna2b;kcna4;kcnab1a;kcnab2a;kcnab2b;kcnb1;kcnc1a;kcnc1b;kcnc2;kcnc3a;kcnc3b;kcnd1;kcnd2;kcng2;kcng4a;kcnh1a;kcnh3;kcnh4b;kcnh5a;kcnh5b;kcnh6a;kcnh7;kcnj11;kcnj11l;kcnj12a;kcnj13;kcnj14;kcnj2b;kcnj3b;kcnj5;KCNJ6;kcnj9;kcnk10a;kcnk10b;kcnk1a;kcnk2b;kcnk3a;kcnk3b;kcnk9;kcnma1a;kcnn1b;kcnn3;kcnq2a;kcnq2b;kcnq3;kcns3a;kcnt2;KCNV1;kcnv2b;klhl24b;lrrc8aa;lrrc8c;mcoln1a;ndufa4;piezo2a.2;ppifb;rem1;rhag;rrad;ryr1b;RYR2;ryr3;scn12aa;scn1bb;scn1lab;scn8aa;scn8ab;sfxn5b;shank1;shank2;shisa7b;shisa8b;si:ch1073-450f2.1;si:ch211-140m22.7;si:ch73-335m24.5;si:ch73-380n15.2;si:rp71-39b20.4;slc12a2;slc12a4;slc12a5a;slc12a5b;slc12a7a;slc12a9;slc15a4;slc16a3;slc16a8;slc17a7a;slc17a7b;slc18a3a;slc20a2;slc24a3;slc24a4a;slc24a4b;slc24a5;slc25a14;slc25a29;slc25a32a;slc30a8;slc30a9;slc32a1;slc33a1;slc34a2a;slc38a3b;slc38a4;slc39a1;slc43a2a;slc4a2b;slc4a4a;slc4a8;slc5a6a;slc6a15;SLC7A1 (1 of many);slc7a7;slc8a1b;slc8a2b;slc8a3;slc8a4a;slc8a4b;slc9a5;snap25a;snap25b;steap4;tcirg1b;tmem163b;tmem175;tpte;trpc1;trpc5a;trpm3;trpv1;ttyh1;ttyh2l;ttyh3b;vdac3;zgc:162160

GO:0006468	protein phosphorylation	1204	213	214.97	0.04144	aak1a;aatka;abl1;acsl4a;ACVR1C;adcyap1b;adgrg1;aida;akt3a;alpk2;ankrd6b;aplnrb;atg13;bcr;bmp3;bmp4;bmpr2a;bsk146;btk;bub1bb;cab39l;cab39l1;camk1a;camk1b;camk1db;camk2a;camk2b1;camk2d1;camk2d2;camk2g1;camk2n1a;camk4;camkk1a;camkk1b;camkva;camkvb;camkvl;cart2;cart3;cbl;ccna2;ccnb1;ccng1;ccni;ccnjl;cdc42bpab;cdk1;cdk14;cdk15;cdk17;CDK18;cdk5r1a;cdk5r1b;cdk5r2a;cdk5r2b;cdkn1a;cds1;csnk1e;CT990561.1;dapk2a;dclk1a;dclk1b;dclk2a;deptor;dicp3.1;dicp3.3;dusp5;dyrk1ab;dyrk2;eif2ak1;eif2ak2;ek1;enpp1;epha4b;epha6;ephb1;ephb2a;ephb6;erbb4b;ern2;fgfr4;gadd45ba;gadd45ga;gc2;gcn1;ghra;gprc5ba;gprc5bb;grk7a;gsk3aa;GSK3B (1 of many);hipk2;hunk;igf1rb;il6st;ilk;inhbaa;inpp5jb;insra;jak1;kcnh1a;kcnh3;kcnh4b;kcnh5a;kcnh5b;kita;ksr2;lepr;limk1a;map2k2b;MAP3K13;map4k4;mapk11;mapk4;mapk9;mark1;mark4a;mast1a;mast3b;melk;mknk1;mknk2a;mstnb;mylk5;myo3b;nek12;nek6;ntrk3a;ntrk3b;nuak1b;nuak2;oxsr1a;pak1;pak2b;pbk;pdk2a;pdk2b;pdk3b;pdpk1a;pebp1;pik3cb;pim1;pim3;pimr138;pkn3;pkz;plk1;plk2a;ppp2r5b;prkacbb;prkca;prkcdb;prkceb;prkcq;prkd1;prkg2;prkx;prnpb;prok1;qars;rap1b;riok1;rps6ka3a;rps6ka5;rps6kb1b;rps6kl1;sgk1;sgk494a;sgk494b;sh2d3ca;sh3bp5b;sh3rf1;sh3rf2;si:ch1073-184j22.2;si:ch211-117c9.1;si:ch211-147h1.4;si:ch211-191i18.4;si:ch211-195b13.1;si:ch211-195b15.8;si:ch211-255i3.4;si:ch73-60h1.1;si:ch73-62l21.1;si:dkey-16p21.7;si:dkey-17m8.1;si:dkey-240h12.4;sik1;sik2a;SLA (1 of many);slkb;smarca4a;snrkb;spegb;spry2;spry4;src;srpk1b;stk17a;stk35;stk40;taok1b;tesk2;tgfb2;tgfb3;tnika;tnk2b;trib2;trib3;unc119a;unc119b;unm_sa1261;wee1;zak;zgc:158659
GO:0050790	regulation of catalytic activity	883	180	157.66	0.0051	acap3b;adcyap1b;agap1;agap3;aida;alox5ap;anos1a;anos1b;appb;arap3;arhgap10;arhgap12b;arhgap21a;arhgap23b;arhgap32a;arhgap33;arhgap4b;arhgdia;arhgdig;asap1b;asap2a;atg13;atp1b1b;atp1b2a;atp1b3a;atp1b3b;aup1;baxa;baxb;bbc3;bcl2l1;bcr;birc7;calm1b;calm3a;camk2n1a;cap2;cart2;cart3;cbl;ccna2;ccnb1;ccng1;ccni;ccnjl;cdk5r1a;cdk5r1b;cdk5r2a;cdk5r2b;cdkn1a;chn1;cish;cst14a.2;cst14b.1;ctsba;dab2ipa;dab2ipb;deptor;DOCK4 (1 of many);dusp5;dynll1;dynll2a;dynll2b;elmod1;ensaa;ensab;esco2;gadd45ba;gadd45ga;gchfr;gcn1;gdi1;git2a;gprc5ba;gprc5bb;gpsm1a;gpsm2;grpel1;guca1a;guca1b;guca1c;guca1g;hdr;hip1;hmha1a;igf1rb;iqgap2;map2k2b;MAP3K13;map4k4;mcm2;ncf2;nek12;oaz1b;oaz2b;oxsr1a;pafah1b1a;pak1;pak2b;pcsk1nl;phactr3a;phactr3b;pik3r1;pik3r3a;pik3r3b;plxna2;ppifb;ppp1r14aa;ppp1r14ba;ppp1r1b;ppp1r3cb;ppp2r2bb;ppp2r2ca;ppp2r2cb;ppp2r5b;prkab1a;prnpb;prok1;prpsap1;psmd1;psmd3;qars;rap1b;rap1gap;rap1gap2a;rap1gap2b;RAP1GDS1;rasgef1bb;rcan1a;rcan2;rcan3;rgl3a;rgs6;rgs8;rictora;rxfp2a;sec23b;serpine3;serpinh1a;serpini1;sgsm1b;sh3bp5b;sh3pxd2b;si:ch1073-184j22.2;si:ch211-191i18.4;si:ch211-195b11.3;si:ch211-195b15.7;si:ch211-195b15.8;si:ch211-255i3.4;si:ch211-270g19.5;si:dkey-17m8.1;si:dkey-203a12.8;si:dkey-242g16.2;si:dkey-77g12.1;slkb;smarca4a;socs2;spry2;spry4;srgap1a;srgap1b;srgap2;stxbp5a;syngap1b;taok1b;tbc1d10ab;tbc1d12a;tbc1d17;tbc1d25;tbc1d4;tiam1a;timp2b;timp4.3;tnika;trappc6bl;trib2;trib3;unc119a;unc119b;zak
GO:0007010	cytoskeleton organization	787	180	140.52	0.04594	abl1;ablim1b;ablim3;ache;actr3b;add3a;afap1;ajuba;anln;ap1ar;apc;apc2;arhgef10lb;arhgef11;atat1;baiap2a;bcar1;CABZ01044277.1;CABZ01118678.1;calm1b;calm3a;camsap3;cap2;capza1a;cbfb;ccdc88b;ccsapb;cct5;cetn2;cfap206;cfl1;clasp2;clstn1;cnn3a;coro1cb;coro2ba;coro2bb;CU639469.1;dbn1;dchs1b;diaph3;dlg1l;dmtn;dpysl2b;dpysl3;DST;dusp27;dync1li2;efs;eml1;epb41b;epb41l3a;espnla;evla;fermt2;flncb;fmnl2a;fmnl2b;fmnl3;frmd5;fscn1a;fscn1b;fscn2a;fscn2b;GAN;grip2a;gsna;havcr1;hip1;hook1;inpp5jb;iqsec3a;iqsec3b;katnb1;kif11;kif4;lima1a;lrmp;map1aa;map1ab;map2;map4l;map6a;map6b;map6d1;map7a;map7d1a;map7d2b;mapre1b;mapre2;mapre3b;maptb;mark1;mark4a;mast1a;mef2aa;mef2d;mical3b;myom2a;nckap1;nusap1;pacsin1a;pafah1b1a;pafah1b1b;pfn2;pgm5;pleca;pls3;ppp1r9a;ppp1r9bb;prc1b;rac3a;rac3b;racgap1;rhobtb1;rhoca;rhogb;rhoua;rictora;rnd1a;rnd1b;sdc4;sdcbp;sdcbp2;sept15;sept3;sept5a;sept5b;shroom2a;si:ch211-114n24.6;si:ch211-207i1.2;si:ch73-199e17.1;si:dkey-56f14.7;si:dkeyp-57f11.2;sorbs1;sorbs2b;sorbs3;spag1a;specc1;specc1lb;spire1b;spire2;ssh1b;ssh2b;stmn2a;stmn2b;stmn3;stmn4;stmn4l;sun1;syne2b;tacc1;tmod2;tmsb;tmsb2;tnnt2c;tnnt3b;tpm3;triobpa;tuba1a;tuba1b;tuba1c;tuba2;tuba8l;tuba8l3;tuba8l4;tubb2;tubb2b;tubb4b;tubb5;vangl2;vcp;vil1;wasf3a;xirp2a;zgc:152977;zgc:153426;zgc:154093;zgc:65894;zgc:86764

GO:0048666	neuron development	570	177	101.77	0.01571	ache;adcy1b;adcyap1b;agrn;alcamb;aldoaa;amigo1;anos1a;apc;appb;arl3l1;arl3l2;asah1b;ascl1a;atat1;atp2b1a;bbs4;bbs5;bdnf;bsg;CABZ01118678.1;cad;camsap3;capn1a;casp3a;casp9;ccsapb;cdh4;chd7;clstn1;cnp;cntfr;cntn2;cntn3a.1;col19a1;ctbp2a;cxcl12b;cyfip2;diras1a;dpysl2b;dpysl3;dpysl4;dpysl5a;dscama;e2f8;efna1b;efna2a;epha4b;etv1;etv5b;ewsr1b;fryb;fus;gldn;gpm6ab;gprc5ba;gprc5bb;grip1;grna;hcn1;hecw2a;hmx1;il6st;inab;inpp5jb;insm1a;irx4a;isl2a;islr2;kcnc3a;kctd13;kif1b;kif3a;kif5ab;kif5c;lgmn;lingo1a;lingo1b;llph;map1aa;map1ab;map2;map4k4;map4l;maptb;mcoln1a;mecp2;ncam1a;ncam1b;nckap1;ndrg4;nefma;nexn;nfasca;ninl;nptna;nptnb;nrg1;nrp1a;nrp2a;nrxn2a;nsfa;nyap2a;nyap2b;olfm1a;pacsin1a;pak1;pcdh18b;plppr4b;pls3;plxna2;ppp1r9a;ppp1r9bb;prmt8b;ptprfa;rab10;rac1a;rb1;rbpms2a;rbpms2b;robo2;robo3;robo4;rpgrip1;rtn4a;rtn4r;scn8aa;sema3fa;sema3ga;sema3gb;sema4aa;sema4bb;sema6bb;sema6e;sema7a;shank1;shank2;si:ch1073-450f2.1;si:ch211-113g11.6;si:ch211-129c21.1;si:dkey-16p21.7;skia;skib;skilb;slc33a1;slit2;slitrk2;slitrk3a;slitrk3b;slitrk4;slitrk6;snap25b;sox11a;sox11b;srgap2;ssh1b;ssh2b;stat3;stmn2a;stmn2b;stmn3;stmn4;stmn4l;tbx2b;tenm1;TENM2;tenm3;tenm4;thrb;tmem106bb;tmem59l;tnika;tnr;uba1;vangl2;zfyve27;zgc:77784
GO:0007268	chemical synaptic transmission	352	147	62.85	0.00147	adcyap1b;apba1a;bdnf;CABZ01081780.1;cacna1aa;cacna1bb;cacnb1;cacnb2a;cacnb3a;cacnb3b;cacng2a;cacng3b;cacng5a;cacng7b;cacng8b;cadpsb;calb2a;calb2b;chata;chrm4a;chrm5a;chrna10a;chrna6;chrnb2a;chrnb3a;chrnb3b;clstn1;clstn2;clstn3;cplx2;cplx2l;cplx3b;cplx4a;cplx4b;CR382281.1;dlg1l;dlg3;dlg4a;dlgap1b;dlgap2a;drd4a;gabra1;gabra3;gabra5;gabra6b;gabrb1;gabrb2;gabrb3;gabrb4;gabrd;gabrg2;gabrr1;gabrr2a;glra1;glra2;glra3;glra4b;glrba;glrbb;grik1a;grik1b;GRIK3;grik4;grin2aa;grin2ab;grm1a;grm1b;grm2a;grm4;grm6a;grm6b;grm8a;grm8b;hcn1;htr2cl1;htr7c;kat2a;kcnq3;lin7a;napaa;napab;napba;napbb;neto1;nlgn1;nlgn2a;nlgn2b;nlgn3a;nptx2a;nrxn2a;nsmfa;nsmfb;otofa;pdzd11;penka;plcl2;ppfia3;ppp1r9bb;rimbp2;rims1b;rims2a;rims2b;rims3;sdcbp;sdcbp2;shank1;shank2;shisa7b;shisa8b;si:ch1073-450f2.1;si:ch73-380n15.2;slc12a4;slc12a5a;slc12a5b;slc12a7a;slc17a6b;slc17a7a;slc17a7b;snap25a;snap25b;stx11a;stx11b.2;stx2a;stx4;sv2a;sv2ba;sv2bb;sv2c;syn1;syn2a;sypa;sypb;syt10;syt11b;syt12;syt13;syt1a;syt2a;syt3;syt4;syt5a;syt5b;syt6a;syt6b;syt7b;syt9a;syt9b
GO:0031175	neuron projection development	443	143	79.1	0.00597	adcy1b;adcyap1b;agrn;alcamb;aldoaa;amigo1;anos1a;apc;appb;arl3l1;arl3l2;asah1b;ascl1a;bdnf;bsg;CABZ01118678.1;cad;camsap3;capn1a;casp3a;casp9;clstn1;cnp;cntfr;cntn2;cntn3a.1;col19a1;cxcl12b;cyfip2;dpysl2b;dpysl3;dpysl4;dpysl5a;dscama;e2f8;efna1b;efna2a;epha4b;etv5b;fryb;fus;gpm6ab;gprc5ba;gprc5bb;grip1;grna;hecw2a;il6st;inab;inpp5jb;isl2a;islr2;kcnc3a;kctd13;kif1b;kif3a;kif5ab;kif5c;lgmn;lingo1a;llph;map1aa;map1ab;map2;map4k4;map4l;maptb;mecp2;ncam1a;ncam1b;nckap1;nefma;nexn;nfasca;nptna;nptnb;nrp1a;nrp2a;nrxn2a;nsfa;nyap2a;nyap2b;olfm1a;pacsin1a;pak1;pcdh18b;plppr4b;pls3;plxna2;ppp1r9a;ppp1r9bb;prmt8b;ptprfa;rab10;rac1a;rb1;rbpms2a;rbpms2b;robo2;robo3;robo4;rtn4a;rtn4r;scn8aa;sema3fa;sema3ga;sema3gb;sema4aa;sema4bb;sema6bb;sema6e;sema7a;shank1;shank2;si:ch1073-450f2.1;si:ch211-113g11.6;si:ch211-129c21.1;si:dkey-16p21.7;slc33a1;slit2;slitrk2;slitrk3a;slitrk3b;slitrk4;slitrk6;srgap2;ssh1b;ssh2b;stat3;stmn2a;stmn2b;stmn3;stmn4;stmn4l;tenm3;tmem106bb;tmem59l;tnika;tnr;uba1;vangl2;zfyve27;zgc:77784
GO:0007417	central nervous system development	519	127	92.67	0.00406	adcyap1b;adgrg1;agrn;agtpbp1;alcamb;aldh1a2;aldoaa;amigo1;anos1a;apc;appb;ascl1a;ascl1b;asphd1;atp1a1b;atp1a3a;bcl11aa;bcl11ab;bdnf;cad;capn1a;ccsapb;cenpf;chn1;cnnm2b;cntn2;CU639469.1;CU861477.1;cxcl12b;cyp26c1;dpysl2b;drd4a;dync1h1;eif3ha;enpp1;etv5b;ewsr1b;fgf8a;fn1a;gbx2;glra1;h2afy2;hapln1a;her15.1;her6;hgfb;hmgcs1;hsbp1b;igf1rb;igf2b;insm1a;irx7;josd2;jun;katnb1;kcnb1;kcnh1a;kctd13;kirrel3l;lgi1b;lzic;map2;marcksa;marcksb;marcksl1a;mdkb;meis3;msi2b;ncam1a;ncanb;nlrc3l;nono;nrg1;nsfa;ntrk3a;otud4;pafah1b1a;pax6b;pcdh10b;pcdh19;pebp1;pou4f2;pou4f3;prdm8b;prmt8b;prnpb;psen2;psmd3;ptpro;ptprsa;pycr1b;qars;rbfox1;robo2;rps15a;rps4x;rtn4a;sez6l2;sfrp5;shank1;shank2;SHC3;si:ch1073-450f2.1;si:ch73-119p20.1;six3a;slc33a1;slc7a7;slc9a3r1a;slit2;smarca4a;smc1a;sox11a;sox11b;spry4;syngap1b;tacc1;tbx2b;tgif1;thraa;tpp1;usp21;usp24;usp28;vangl2;vcana;vwc2;ywhag1

GO:0016477	cell migration	546	119	97.49	0.02508	akap12b;alcamb;ankrd6b;anos1a;anxa6;apc;apc2;aplnrb;apoc1;arhgap4b;arrb1;atp1a1b;bcar1;ccr9a;chd7;cmklr1;cntn2;ctnnd1;cxcl12b;cxcl14;cxcr3.3;dmxl2;dpysl3;dscama;efs;esrra;fermt2;fgf8a;fmnl3;fn1a;fscn1a;fscn1b;fscn2a;fscn2b;fzd2;fzd7a;git2a;gnb1b;gpc3;grip2a;hgfb;hsp90ab1;igf1rb;igsf11;ildr1b;inpp5d;insm1a;isl2a;itgav;itgb3b;itgb7;jagn1a;kcnj13;kita;klf6a;lama5;megf8;meis3;mmp14b;mmp9;mt2;nckap1;nrg1;nrp1a;nrp2a;nsmfa;nsmfb;nusap1;pak1;pik3cb;plekhg5a;plxna2;prcp;ptges;ptpn23a;pum3;rac1a;rac3a;rac3b;rap1b;rcc2;rhobtb1;rhoca;rnd1a;rnd1b;robo4;rtn4a;rxfp3.3b;sdc2;sdc4;sema3fa;sema3ga;sema3gb;sema4aa;sema4bb;sema6bb;sema6e;sema7a;si:ch211-113g11.6;si:ch211-129c21.1;si:ch211-215c18.3;si:dkey-56f14.7;slc24a5;slit2;smarca4a;srgap1a;srgap1b;srgap2;sst6;stat3;tbx2b;thy1;tmie;tmsb;tmsb2;vangl2;zbtb4;zeb2b;zgc:158659
GO:0048812	neuron projection morphogenesis	351	115	62.67	0.01301	adcy1b;agrn;alcamb;aldoaa;anos1a;apc;appb;arl3l1;arl3l2;asah1b;bdnf;bsg;cad;capn1a;casp3a;casp9;clstn1;cntn2;cntn3a.1;col19a1;cxcl12b;cyfip2;dpysl2b;dpysl3;dpysl4;dpysl5a;dscama;e2f8;efna1b;efna2a;epha4b;etv5b;fus;grna;hecw2a;inab;isl2a;islr2;kcnc3a;kctd13;kif1b;kif3a;kif5ab;kif5c;llph;map1aa;map1ab;map4k4;mecp2;ncam1a;ncam1b;nckap1;nexn;nfasca;nptna;nptnb;nrp1a;nrp2a;nrxn2a;nsfa;nyap2a;nyap2b;olfm1a;pacsin1a;pak1;pcdh18b;plppr4b;pls3;plxna2;prmt8b;ptprfa;rac1a;rb1;rbpms2a;rbpms2b;robo2;robo3;robo4;rtn4a;rtn4r;scn8aa;sema3fa;sema3ga;sema3gb;sema4aa;sema4bb;sema6bb;sema6e;sema7a;shank1;shank2;si:ch1073-450f2.1;si:ch211-113g11.6;si:ch211-129c21.1;si:dkey-16p21.7;slc33a1;slit2;slitrk2;slitrk3a;slitrk3b;slitrk4;slitrk6;srgap2;ssh1b;ssh2b;stat3;tenm3;tmem106bb;tmem59l;tnika;tnr;uba1;vangl2;zfyve27;zgc:77784
GO:0006412	translation	426	113	76.06	0.0002	aimp1;ajuba;atp6v0e1;cirbpb;cpeb1a;cpeb3;dalrd3;disp2;dph6;dre-mir-21-1;dre-mir-21-2;eef1a1b;eef1a1l2;eef1a2;eef1b2;eef1db;eef1g;eef2a.2;eef2b;eif2ak1;eif2ak2;eif2s1a;eif2s1b;eif2s2;eif2s3;eif3ba;eif3f;eif3ha;eif3i;eif3m;eif3s6ip;eif4a1b;eif4ebp1;eif4g1a;eif4g2a;eif5a;eif5a2;eif6;eprs;farsb;faub;fxr1;fxr2;gars;gcn1;gemin5;gtpbp1;gtpbp2b;hspa14;iars;igf2bp3;lin28a;mov10b.1;nars;nck2a;nhp2;pkz;qars;rars;rpl10a;rpl12;rpl18;rpl21;rpl22;rpl22l1;rpl23;rpl23a;rpl27;rpl29;rpl30;rpl34;rpl35;rpl35a;rpl36a;rpl38;rpl39;rpl4;RPL41;rpl5a;rpl5b;rpl8;rpl9;rps11;rps12;rps13;rps15a;rps17;rps18;rps21;rps23;rps24;rps27.2;rps28;rps29;rps3a;rps4x;rps6kb1b;rps7;rps8a;rps8b;rps9;rpsa;rwdd1;sars;shmt1;tars;tnrc6c2;uba52;ube2e1;upf3a;uqcc2;wars;ybx1
GO:0007017	microtubule-based process	400	98	71.42	0.01987	apc;apc2;arhgef11;atat1;CABZ01044277.1;CABZ01118678.1;calm1b;calm3a;camsap3;ccdc88b;ccsapb;cenpe;cetn2;cfap206;clasp2;clstn1;CU639469.1;dchs1b;dctn1b;dnah2;DST;dync1h1;dync1i1;dync1i2a;dync1i2b;dync1li2;dynll1;dynll2a;dynll2b;eml1;hook1;inpp5jb;ippk;katnb1;kif11;kif1aa;kif1ab;kif1b;kif20a;kif20bb;kif21a;kif3a;kif3ca;kif3cb;kif4;kif5ab;kif5c;lrmp;map1aa;map1ab;map2;map4l;map6a;map6b;map6d1;map7a;map7d1a;map7d2b;mapre1b;mapre2;mapre3b;maptb;mark1;mark4a;nusap1;pafah1b1a;pafah1b1b;prc1b;racgap1;rhot1a;si:ch211-114n24.6;si:ch211-235e9.8;si:ch211-257p13.3;si:ch73-199e17.1;spag1a;stmn2a;stmn2b;stmn3;stmn4;stmn4l;tacc1;tuba1a;tuba1b;tuba1c;tuba2;tuba8l;tuba8l3;tuba8l4;tubb2;tubb2b;tubb4b;tubb5;vangl2;vbp1;vcp;zgc:153426;zgc:65894;zgc:86764
GO:0007409	axonogenesis	295	95	52.67	0.04257	adcy1b;agrn;alcamb;aldoaa;anos1a;apc;appb;arl3l1;arl3l2;asah1b;bsg;capn1a;casp3a;casp9;clstn1;cntn2;cntn3a.1;col19a1;cxcl12b;cyfip2;dpysl2b;dpysl3;dpysl5a;dscama;e2f8;efna1b;efna2a;epha4b;etv5b;fus;grna;isl2a;islr2;kcnc3a;kctd13;kif1b;kif3a;kif5ab;kif5c;map1aa;map1ab;mecp2;ncam1a;ncam1b;nexn;nfasca;nptna;nptnb;nrp1a;nrp2a;nrxn2a;nsfa;olfm1a;pak1;plppr4b;pls3;plxna2;ptprfa;rb1;rbpms2a;rbpms2b;robo2;robo3;robo4;rtn4a;rtn4r;scn8aa;sema3fa;sema3ga;sema3gb;sema4aa;sema4bb;sema6bb;sema6e;sema7a;si:ch211-113g11.6;si:ch211-129c21.1;si:dkey-16p21.7;slc33a1;slit2;slitrk2;slitrk3a;slitrk3b;slitrk4;slitrk6;ssh1b;ssh2b;stat3;tenm3;tmem59l;tnr;uba1;vangl2;zfyve27;zgc:77784
GO:0007264	small GTPase mediated signal transduction	379	93	67.67	0.00016	akap12b;arhgap32a;arhgap33;arhgdia;arhgdig;arhgef10lb;arhgef11;arhgef2;arhgef33;arhgef4;arhgef9a;arhgef9b;bcr;ccdc28a;cdc42se2;chn1;cyth1a;dock11;dock3;DOCK4 (1 of many);dock6;dock7;dock9b;fgd1;gdi1;grb2a;iqsec3a;iqsec3b;kalrna;kctd13;ksr2;mcf2a;mcf2l2;mcf2la;plekhg5a;prex2;rab10;rab11a;rab15;rab20;rab22a;rab32a;rab33a;rab34a;rab34b;rab39ba;rab3aa;rab3ab;rab41;rab42a;rab43;rab4a;rab6ba;rab6bb;rabif;rabl2;rac1a;rac3a;rac3b;ralba;ralgps1;rap1b;rap1gap;rap1gap2a;rap1gap2b;rapgef1a;rapgef4;rapgef5a;rapgef5b;rapgefl1;rasal1b;rasgef1bb;RASGRF1;rgl3a;rhobtb1;rhoca;rhogb;rhot1a;rhoua;rnd1a;rnd1b;robo4;sh2d3ca;si:dkey-38p12.3;sos2;spata13;spry2;spry4;tiam1a;tiam2a;vav3b;zgc:154093;zgc:91909

GO:0007420	brain development	373	88	66.6	0.00193	adcyap1b;agtpbp1;alcamb;aldh1a2;aldoaa;amigo1;anos1a;apc;ascl1a;ascl1b;asphd1;atp1a1b;atp1a3a;bcl11aa;bcl11ab;bdnf;capn1a;ccsapb;cenpf;cnnm2b;CU639469.1;cxcl12b;cyp26c1;dpysl2b;eif3ha;enpp1;etv5b;fgf8a;fn1a;gbx2;h2afy2;her15.1;her6;hgfb;igf2b;irx7;jun;katnb1;kcnb1;kcnh1a;kctd13;kirrel3l;lgi1b;marcksa;marcksb;mdkb;meis3;msi2b;nrg1;nsfa;otud4;pafah1b1a;pax6b;pcdh10b;pcdh19;pebp1;pou4f2;pou4f3;prmt8b;psen2;psmd3;ptpro;ptprsa;pycr1b;qars;rbfox1;rps15a;rps4x;rtn4a;sez6l2;sfrp5;shank1;shank2;si:ch1073-450f2.1;si:ch73-119p20.1;six3a;slc9a3r1a;sox11a;sox11b;spry4;syngap1b;tacc1;tbx2b;tgif1;thraa;vangl2;vwc2;ywhag1
GO:0023014	signal transduction by protein phosphorylation	326	67	58.21	0.03029	acsl4a;adcyap1b;aida;ankrd6b;bmp3;bmp4;cab39l;cab39l1;cart2;cart3;cds1;CT990561.1;dicp3.1;dicp3.3;dusp5;epha4b;epha6;ephb1;ephb2a;ephb6;erbb4b;gadd45ba;gadd45ga;igf1rb;ilk;inhbaa;insra;kcnh1a;kcnh3;kcnh4b;kcnh5a;kcnh5b;kita;map2k2b;MAP3K13;map4k4;mapk11;mapk4;mapk9;mstnb;nek12;ntrk3a;ntrk3b;oxsr1a;pak1;pak2b;pebp1;prok1;qars;rap1b;sh3rf1;sh3rf2;si:ch1073-184j22.2;si:ch211-191i18.4;si:ch211-195b15.8;si:dkey-17m8.1;slkb;smarca4a;spry2;spry4;taok1b;tgfb2;tgfb3;tnika;trib2;trib3;zak
GO:0050804	modulation of chemical synaptic transmission	110	59	19.64	0.01314	adcyap1b;bdnf;CABZ01081780.1;cacng2a;cacng3b;cacng5a;cacng7b;cacng8b;calb2a;calb2b;clstn1;clstn2;clstn3;cplx2;cplx2l;cplx3b;cplx4a;cplx4b;grik1a;grik1b;GRIK3;grik4;grin2aa;grin2ab;grm1a;grm1b;grm2a;grm4;grm6a;grm6b;grm8a;grm8b;kat2a;napaa;napab;napba;napbb;neto1;nlgn1;nlgn2a;nlgn2b;nlgn3a;nptx2a;nsmfa;nsmfb;plcl2;ppp1r9bb;rims1b;rims2a;rims2b;rims3;shank1;shank2;shisa7b;shisa8b;si:ch1073-450f2.1;sypa;sypb;syt12
GO:0051726	regulation of cell cycle	519	58	92.67	0.00636	adcyap1b;anapc15;apc;brinp2;brinp3a.1;bub1bb;cables2b;ccna2;ccnb1;ccng1;ccni;ccnjl;ccsapb;cdk1;cdk15;cdkn1a;cetn2;chmp4bb;dlg1l;dot1l;dync1li2;e2f3;e2f8;etv5b;gadd45ba;gadd45ga;hsp90ab1;id4;insm1a;jun;junba;kif11;mdka;mdm4;pea15;pebp1;pim3;pimr138;ppp1r13ba;prc1b;rab11fip4a;rb1;rpl23a;rpl35;rpl36a;rpl7;rps15a;rps18;rps29;rps7;rps8a;si:ch211-147h1.4;si:dkey-25o16.4;si:dkey-79d12.5;spdl1;ssrp1a;tp53bp1;zwilch
GO:0006816	calcium ion transport	169	57	30.17	0.00343	anxa6;aplnrb;atp2a1;atp2b1a;atp2b2;atp2b3a;atp2b3b;CABZ01081780.1;cacna1aa;cacna1bb;cacna1da;cacna1g;cacna1ha;cacna1hb;cacna1i;cacna2d2a;cacna2d2b;cacna2d4b;cacnb1;cacnb2a;cacnb3a;cacnb3b;cacng2a;cacng3b;cacng5a;cacng6b;cacng7b;cacng8b;cbarpb;fam155a;gem;homer1b;homer3b;htr2cl1;itpr1a;jph3;mcoln1a;psen2;rem1;rrad;ryr1b;RYR2;ryr3;si:ch211-270g19.5;si:ch73-335m24.5;slc24a3;slc24a4a;slc24a4b;slc24a5;slc8a1b;slc8a2b;slc8a3;slc8a4a;slc8a4b;trpc1;trpc5a;trpv1
GO:0042391	regulation of membrane potential	157	56	28.03	0.00309	adcyap1b;cacna1g;cacna1ha;cacna1hb;cacna1i;chrna10a;chrna6;chrnb2a;chrnb3a;chrnb3b;gabra1;gabra3;gabra5;gabra6b;gabrb1;gabrb2;gabrb3;gabrb4;gabrd;gabrg2;gabrr1;gabrr2a;glra1;glra2;glra3;glra4b;glrba;glrbb;grin2aa;grin2ab;grm1a;grm1b;kcnab1a;kcnab2b;kcnh1a;kcnh3;kcnh4b;kcnh5a;kcnh5b;kcnh6a;kcnh7;kcnt2;piezo2a.2;rims1b;rims2a;rims2b;rims3;scn12aa;scn1bb;scn1lab;scn8aa;scn8ab;shank1;shank2;si:ch1073-450f2.1;si:ch73-380n15.2
GO:0007015	actin filament organization	253	55	45.17	0.03905	actr3b;add3a;ap1ar;arhgef10lb;arhgef11;baiap2a;bcar1;cap2;capza1a;cct5;cfl1;dbn1;dlg1l;dpysl3;efs;espnla;evla;fmnl3;fscn1a;fscn1b;fscn2a;fscn2b;gsna;hip1;lima1a;mical3b;myom2a;pfn2;pls3;ppp1r9a;ppp1r9bb;rac3a;rac3b;rhobtb1;rhoca;rhogb;rhoua;rnd1a;rnd1b;sdc4;shroom2a;sorbs1;sorbs2b;sorbs3;spire1b;spire2;ssh1b;ssh2b;tmod2;tmsb;tmsb2;tpm3;vangl2;vil1;zgc:154093
GO:0070588	calcium ion transmembrane transport	146	48	26.07	0.00014	anxa6;aplnrb;atp2a1;atp2b1a;atp2b2;atp2b3a;atp2b3b;CABZ01081780.1;cacna1aa;cacna1bb;cacna1da;cacna1g;cacna1ha;cacna1hb;cacna1i;cacna2d2a;cacna2d2b;cacna2d4b;cacnb1;cacnb2a;cacnb3a;cacnb3b;cacng2a;cacng3b;cacng5a;cacng6b;cacng7b;cacng8b;cbarpb;fam155a;gem;htr2cl1;itpr1a;jph3;mcoln1a;rem1;rrad;ryr1b;RYR2;ryr3;si:ch73-335m24.5;slc24a3;slc24a4a;slc24a4b;slc24a5;trpc1;trpc5a;trpv1
GO:0007269	neurotransmitter secretion	71	41	12.68	0.01159	cadpsb;cplx2;cplx2l;cplx3b;cplx4a;cplx4b;lin7a;napaa;napab;napba;napbb;otofa;pdzd11;ppfia3;rims1b;rims2a;rims2b;rims3;snap25a;snap25b;stx11a;stx11b.2;stx2a;stx4;syn1;syn2a;syt10;syt11b;syt12;syt13;syt1a;syt2a;syt3;syt4;syt5a;syt5b;syt6a;syt6b;syt7b;syt9a;syt9b
GO:0035335	peptidyl-tyrosine dephosphorylation	112	31	20	0.00656	ca16b;cdc14ab;dusp23b;DUSP26;dusp3a;dusp5;mtmr7a;mtmr7b;ptenb;ptp4a1;ptp4a2b;ptp4a3;ptpdc1b;ptpmt1;ptpn11b;ptpn21;ptpn23a;ptpn4b;ptprdb;ptprfa;ptprnb;ptpro;ptprsa;ptprt;ptprz1b;si:ch1073-184j22.2;si:ch211-195b15.8;ssh1b;ssh2b;tpte;zgc:77752
GO:0007018	microtubule-based movement	156	31	27.85	0.01777	arhgef11;ccsapb;cenpe;cfap206;clstn1;dctn1b;dnah2;dync1h1;dync1i1;dync1i2a;dync1i2b;dync1li2;ippk;kif11;kif1aa;kif1ab;kif1b;kif20a;kif20bb;kif21a;kif3a;kif3ca;kif3cb;kif4;kif5ab;kif5c;pafah1b1a;pafah1b1b;rhot1a;si:ch211-235e9.8;si:ch211-257p13.3
GO:0003143	embryonic heart tube morphogenesis	113	30	20.18	0.04242	apc;atp1a1b;atp6ap1b;atp6v1f;bbs4;bbs5;bmp4;bmpr2a;bnip3lb;cacnb2a;camk2a;camk2g1;cenpf;chd7;fabp3;fn1a;fzd2;gpr22a;mef2cb;ndrg4;pak1;pkp2;scn12aa;skia;skib;slc8a4a;smarca4a;tbx2b;tbx5b;trib3
GO:0035249	synaptic transmission, glutamatergic	50	29	8.93	0.0001	adcyap1b;cacng2a;cacng3b;cacng5a;cacng7b;cacng8b;grik1a;grik1b;GRIK3;grik4;grm1a;grm1b;grm2a;grm4;grm6a;grm6b;grm8a;grm8b;napaa;napab;napba;napbb;nptx2a;shank1;shank2;si:ch1073-450f2.1;slc17a6b;slc17a7a;slc17a7b

GO:0051480	regulation of cytosolic calcium ion concentration	166	28	29.64	0.00462	adcyap1b;aplnrb;atp2b1a;atp2b2;atp2b3a;atp2b3b;calb2a;calb2b;ccr9a;cmklr1;cxcr3.3;fam155a;grm1a;grm1b;htr2cl1;jph3;mcoln1a;pvalb2;pvalb3;pvalb6;pvalb9;rxfp3.3b;ryr1b;RYR2;ryr3;slc8a4a;trpc1;trpc5a
GO:0007626	locomotory behavior	85	28	15.18	0.01321	abhd12;adgrl3.1;atp1a3a;bag3;chata;fhl1a;flnca;flncb;gdpd5a;gprc5ba;gprc5bb;htr2cl1;mecp2;nr1d1;pleca;psmd3;ryr1b;scn1lab;scn8aa;slc18a2;slc1a2b;snap25a;snap25b;sncb;sncgb;syngap1b;tpp1;vcp
GO:0050770	regulation of axonogenesis	90	27	16.07	0.04238	arl3l1;arl3l2;casp3a;casp9;clstn1;cntn2;dpysl2b;epha4b;pak1;plxna2;rbpms2a;rbpms2b;robo2;sema3fa;sema3ga;sema3gb;sema4aa;sema4bb;sema6bb;sema6e;sema7a;si:ch211-113g11.6;si:ch211-129c21.1;slit2;ssh1b;ssh2b;zfyve27
GO:1902476	chloride transmembrane transport	69	24	12.32	0.00057	best2;clcn7;clic4;clic5a;gabra1;gabra3;gabra5;gabra6b;gabrg2;glra1;glra2;glra3;glra4b;glrba;glrbb;slc12a2;slc12a4;slc12a5a;slc12a5b;slc12a7a;slc12a9;ttyh1;ttyh2l;ttyh3b
GO:0050806	positive regulation of synaptic transmission	42	22	7.5	0.00014	adcyap1b;CABZ01081780.1;cacng2a;cacng3b;cacng5a;cacng7b;cacng8b;calb2a;calb2b;clstn1;clstn2;clstn3;grin2aa;grin2ab;rims1b;rims2a;rims2b;rims3;shank1;shank2;si:ch1073-450f2.1;syt12
GO:0060078	regulation of postsynaptic membrane potential	66	22	11.78	0.00055	adcyap1b;chrna10a;chrna6;chrnb2a;chrnb3a;chrnb3b;gabra1;gabra3;gabra5;gabra6b;gabrg2;glra1;glrba;glrbb;grin2aa;grin2ab;grm1a;grm1b;shank1;shank2;si:ch1073-450f2.1;si:ch73-380n15.2
GO:0007623	circadian rhythm	64	22	11.43	0.01789	arntl1a;arntl2;ciarta;ciartb;cipca;clocka;htr7c;id4;impdh2;mitfa;nfil3;nfil3-5;npas2;npy;nr1d1;nr1d4b;otx5;per1a;per3;prok1;scn1lab;si:ch211-132b12.7
GO:0048167	regulation of synaptic plasticity	38	21	6.78	0.00617	CABZ01081780.1;calb2a;calb2b;grin2aa;grin2ab;kat2a;neto1;nsmfa;nsmfb;rims1b;rims2a;rims2b;rims3;shank1;shank2;shisa7b;shisa8b;si:ch1073-450f2.1;sypa;sypb;syt12
GO:0042176	regulation of protein catabolic process	81	21	14.46	0.03176	ankib1a;apc2;csnk1e;gpc3;hecw2a;hsp90ab1;itcha;oaz2b;psmc1a;psmc1b;psmd1;psmd3;RNF14 (1 of many);stub1;timp2b;timp4.3;trib2;trib3;uqcc2;vcp;vip
GO:0045666	positive regulation of neuron differentiation	62	21	11.07	0.03898	adcyap1b;ascl1a;ascl1b;brinp2;brinp3a.1;cntfr;cntn2;gprc5ba;gprc5bb;il6st;llph;mdkb;pacsin1a;plxna2;shank1;shank2;si:ch1073-450f2.1;si:ch211-194k22.8;tcf12;tenm3;zfyve27
GO:0002181	cytoplasmic translation	59	20	10.53	0.00381	atp6v0e1;cpeb1a;cpeb3;dph6;eif2s3;eif3ba;eif3ha;eif3i;eif3m;eif3s6ip;hspa14;lin28a;rpl29;rpl35a;rpl8;rpl9;rps21;rps28;rwdd1;ube2e1
GO:0045765	regulation of angiogenesis	73	20	13.03	0.03705	adgrb1a;adgrb3;aplnrb;dab2ipa;dab2ipb;iars;jcada;mt2;nme2b.1;prkd1;prok1;ptenb;sars;sox11b;tars;tmem184a;unc5b;VASH1;vash2;yjefn3
GO:0042074	cell migration involved in gastrulation	70	19	12.5	0.02761	ankrd6b;apoc1;ctnnd1;cxcl12b;dscama;esrra;fzd7a;git2a;gpc3;itgav;megf8;mmp14b;ptges;rac1a;stat3;tbx2b;vangl2;zbtb4;zeb2b
GO:0031018	endocrine pancreas development	61	19	10.89	0.03783	adamts9;apc;ascl1b;c2cd4a;fscn1a;fzd2;gck;insm1a;insm1b;irx3a;kcnip3b;kcnj11;lepr;pax6b;sept15;slc30a8;sox4b;srebf1;zgc:153115
GO:0043065	positive regulation of apoptotic process	70	19	12.5	0.04987	apc;baxa;baxb;bbc3;bmf2;bnip3lb;bnip4;casp3a;casp9;dab2ipa;dab2ipb;esco2;fas;hdr;hip1;kctd13;melk;osgn1;ppifb
GO:0031101	fin regeneration	59	18	10.53	0.01234	aldh1a2;chd4a;col1a1a;ctsba;cx43;esco2;hapln1a;hspd1;igf1rb;igf2b;inhbaa;kdm6bb;krt5;mmp9;nrg1;smarca4a;tubb5;vdac3
GO:0051017	actin filament bundle assembly	55	17	9.82	0.00536	add3a;arhgef10lb;arhgef11;baiap2a;dpysl3;espnla;fscn1a;fscn1b;fscn2a;fscn2b;lima1a;pfn2;pls3;rhoca;rnd1a;rnd1b;sdc4
GO:0019226	transmission of nerve impulse	24	16	4.29	0.01099	cacna1g;cacna1ha;cacna1hb;cacna1i;cacng2a;cacng3b;cacng5a;cacng7b;cacng8b;kcnab1a;kcnab2b;nfasca;scn12aa;scn1lab;scn8aa;scn8ab
GO:0017158	regulation of calcium ion-dependent exocytosis	21	16	3.75	0.01951	cacna1g;cacna1ha;cacna1hb;cacna1i;napaa;napab;napba;napbb;rims1b;rims2a;rims2b;rims3;scamp5a;syt10;syt9a;syt9b
GO:0009636	response to toxic substance	63	16	11.25	0.02054	abcb4;alox5ap;blmh;cat;eif2ak2;esd;fam213b;gpx3;gpx4b;gstk1;loxhd1a;pebp1;pkz;prdx1;sod1;sod2
GO:0030705	cytoskeleton-dependent intracellular transport	56	15	10	0.00097	ccdc88b;clstn1;dctn1b;hook1;kif1aa;kif1ab;kif1b;kif3a;kif5ab;kif5c;map6a;map6b;pafah1b1a;rhot1a;si:ch211-235e9.8
GO:0071526	semaphorin-plexin signaling pathway	47	15	8.39	0.00323	nrp1a;nrp2a;plxna2;plxna3;plxna4;sema3fa;sema3ga;sema3gb;sema4aa;sema4bb;sema6bb;sema6e;sema7a;si:ch211-113g11.6;si:ch211-129c21.1
GO:0070121	Kupffer's vesicle development	52	15	9.28	0.03473	atp6ap1b;atp6v1f;bbs4;bbs5;cx43;fgf8a;gpr22a;inpp5b;itgav;nkd3;rab11a;rab3ip;sdc2;tcp1;unc119b
GO:0009266	response to temperature stimulus	52	15	9.28	0.04259	bhlhe41;cirbpb;clocka;cry1ba;cry2;dnaja2l;hsf2;hsp70.1;hsp70l;hspa14;hspb1;pebp1;trpm3;trpv1;ucp3
GO:0031110	regulation of microtubule polymerization or depolymerization	31	14	5.54	0.00032	apc;apc2;CABZ01118678.1;camsap3;clasp2;inpp5jb;mapre1b;mapre2;mapre3b;stmn2a;stmn2b;stmn3;stmn4;stmn4l
GO:0046928	regulation of neurotransmitter secretion	20	14	3.57	0.00346	cplx2;cplx2l;cplx3b;cplx4a;cplx4b;napaa;napab;napba;napbb;rims1b;rims2a;rims2b;rims3;syt12
GO:0006096	glycolytic process	35	14	6.25	0.01863	aldoaa;aldoca;aldocb;eno1a;eno2;gck;pfklb;pfkmb;pfkpb;pgam1a;pkma;scn1lab;tpi1a;tpi1b
GO:0060216	definitive hemopoiesis	48	14	8.57	0.03709	angptl1a;angptl2b;camk4;cbl;dkc1;hdac6;hsp70l;mbd3b;pbx1a;rpl22l1;rps24;sae1;scml4;tfec
GO:0046854	phosphatidylinositol phosphorylation	48	14	8.57	0.03709	cish;impa1;impa2;pi4k2a;pi4kaa;pik3cb;pik3r1;pik3r3a;pik3r3b;pip4k2ab;pip5k1cb;pip5kl1;socs2;zgc:158659
GO:0006414	translational elongation	42	13	7.5	0.03805	dph6;eef1a1b;eef1a1l2;eef1a2;eef1b2;eef1db;eef1g;eef2a.2;eef2b;eif5a;eif5a2;gtpbp1;gtpbp2b

GO:0015991	ATP hydrolysis coupled proton transport	35	12	6.25	0.01482	atp1a1b;atp1a3a;atp6ap1b;atp6ap1la;atp6v0a1b;atp6v0a2a;atp6v0e1;atp6v1ba;atp6v1f;atp6v1h;atpv0e2;tcirg1b
GO:0048048	embryonic eye morphogenesis	36	12	6.43	0.02238	cct3;hs6st2;mfap2;plxna2;sfrp1a;sfrp5;smarca4a;sox11a;sox11b;sox4a;sox4b;vangl2
GO:0055075	potassium ion homeostasis	28	12	5	0.03705	atp1a1b;atp1a3a;atp1b1b;atp1b2a;atp1b3a;atp1b3b;slc12a2;slc12a4;slc12a5a;slc12a5b;slc12a7a;slc12a9
GO:0033334	fin morphogenesis	51	12	9.11	0.03729	apc;bmp4;col1a1a;cx43;fras1;frem1a;frem2a;grip2a;lama5;mmp9;pi4kaa;tll1
GO:0048791	calcium ion-regulated exocytosis of neurotransmitter	19	11	3.39	0.00011	rims1b;rims2a;rims2b;rims3;syt11b;syt1a;syt2a;syt4;syt5a;syt5b;syt7b
GO:0048488	synaptic vesicle endocytosis	21	11	3.75	0.00032	dnm1b;dnm3a;nlgn1;nlgn2a;nlgn2b;nlgn3a;si:ch211-233a24.2;syt1a;syt2a;syt5a;syt5b
GO:0007019	microtubule depolymerization	23	11	4.11	0.01108	apc;apc2;CABZ01118678.1;camsap3;ccsapb;katnb1;stmn2a;stmn2b;stmn3;stmn4;stmn4l
GO:0048843	negative regulation of axon extension involved in axon guidance	31	11	5.54	0.01472	robo2;sema3fa;sema3ga;sema3gb;sema4aa;sema4bb;sema6bb;sema6e;sema7a;si:ch211-113g11.6;si:ch211-129c21.1
GO:0048013	ephrin receptor signaling pathway	33	11	5.89	0.03808	CT990561.1;efna1b;efna2a;efna3a;ek1;epha4b;epha6;ephb1;ephb2a;ephb6;si:dkeyp-9d4.3
GO:0008286	insulin receptor signaling pathway	36	11	6.43	0.03981	cish;enpp1;foxo3a;igf1rb;insra;irs2a;pik3r1;pik3r3a;pik3r3b;si:ch211-284e13.4;socs2
GO:0097120	receptor localization to synapse	22	11	3.93	0.04254	cacng2a;cacng3b;cacng5a;cacng7b;cacng8b;dlg1l;dlg3;dlg4a;gphnb;kif5ab;kif5c
GO:2000311	regulation of AMPA receptor activity	19	10	3.39	0.00062	cacng2a;cacng3b;cacng5a;cacng7b;cacng8b;shank1;shank2;shisa7b;shisa8b;si:ch1073-450f2.1
GO:0050919	negative chemotaxis	30	10	5.36	0.03058	sema3fa;sema3ga;sema3gb;sema4aa;sema4bb;sema6bb;sema6e;sema7a;si:ch211-113g11.6;si:ch211-129c21.1
GO:0051968	positive regulation of synaptic transmission, glutamatergic	18	9	3.21	0.00187	adcyap1b;cacng2a;cacng3b;cacng5a;cacng7b;cacng8b;shank1;shank2;si:ch1073-450f2.1
GO:0048813	dendrite morphogenesis	23	9	4.11	0.01117	hecw2a;prmt8b;rbpms2a;rbpms2b;shank1;shank2;si:ch1073-450f2.1;tenm3;tmem106bb
GO:0060291	long-term synaptic potentiation	20	9	3.57	0.01457	CABZ01081780.1;calb2a;calb2b;grin2aa;grin2ab;shank1;shank2;si:ch1073-450f2.1;syt12
GO:0044772	mitotic cell cycle phase transition	83	9	14.82	0.03204	anapc15;bub1bb;cdk1;chmp4bb;clasp2;ensaa;lzts1;rb1;spdl1
GO:0031103	axon regeneration	22	9	3.93	0.03219	ascl1a;cnp;cntfr;cntn2;dpysl2b;epha4b;il6st;lgmn;lingo1a
GO:0070593	dendrite self-avoidance	13	8	2.32	0.00056	bsg;cntn3a.1;dscama;nexn;nptna;nptnb;robo3;robo4
GO:0032922	circadian regulation of gene expression	13	8	2.32	0.00056	ciarta;ciartb;clocka;nfil3-5;nr1d1;otx5;per1a;per3
GO:0000470	maturation of LSU-rRNA	18	8	3.21	0.00626	ftsj3;las1l;nhp2;rpl10a;rpl35;rpl7;rsl1d1;snu13b
GO:2000300	regulation of synaptic vesicle exocytosis	10	8	1.79	0.01115	napaa;napab;napba;napbb;rims1b;rims2a;rims2b;rims3
GO:0043200	response to amino acid	24	8	4.29	0.01204	glra1;glra2;glra3;glra4b;glrba;glrbb;rragca;sesn2
GO:0006171	cAMP biosynthetic process	26	8	4.64	0.01761	adcy1b;adcy2a;adcy3a;adcy6a;adcy7;cap2;rxfp2a;si:dkey-206f10.1
GO:0006817	phosphate ion transport	17	8	3.04	0.0318	ankha;slc17a7a;slc17a7b;slc20a1a;slc20a1b;slc20a2;slc25a14;slc34a2a
GO:1901385	regulation of voltage-gated calcium channel activity	12	8	2.14	0.03849	cacnb1;cacnb2a;cacnb3a;cacnb3b;cbarpb;gem;rem1;rrad
GO:0006884	cell volume homeostasis	23	8	4.11	0.03986	lrrc8aa;lrrc8c;slc12a2;slc12a4;slc12a5a;slc12a5b;slc12a7a;slc12a9
GO:0046847	filopodium assembly	13	7	2.32	0.00092	arhgef9b;dpysl3;fmnl3;gpm6ab;robo4;srgap2;trpc1
GO:0007157	heterophilic cell-cell adhesion via plasma membrane cell adhesion molecules	12	7	2.14	0.00196	igsf21a;igsf21b;pvrl2l;si:ch73-22o12.1;tenm1;TENM2;tenm3
GO:0048168	regulation of neuronal synaptic plasticity	10	7	1.79	0.00275	neto1;nsmfa;nsmfb;shisa7b;shisa8b;sypa;sypb
GO:0045879	negative regulation of smoothened signaling pathway	14	7	2.5	0.0061	cdon;gatad2b;gpc3;sox11a;sox11b;sox4a;sox4b
GO:0009648	photoperiodism	15	7	2.68	0.00626	arntl1a;arntl1b;arntl2;clocka;npas2;per1a;per3
GO:0070654	sensory epithelium regeneration	15	7	2.68	0.00969	apobec2b;ascl1a;fzd2;hbegfa;insm1a;mdka;tgif1
GO:1903670	regulation of sprouting angiogenesis	22	7	3.93	0.01969	aplnrb;iars;jcada;mt2;sars;sox11b;tars
GO:0043113	receptor clustering	14	7	2.5	0.02054	agrn;dlg1l;dlg3;dlg4a;gphnb;grna;slc9a3r1a
GO:0055064	chloride ion homeostasis	17	7	3.04	0.02107	calca;slc12a2;slc12a4;slc12a5a;slc12a5b;slc12a7a;slc12a9
GO:0046686	response to cadmium ion	17	7	3.04	0.02107	baxa;gadd45ba;hsp70.1;hsp70l;jun;mt2;sod2
GO:0099590	neurotransmitter receptor internalization	17	7	3.04	0.03224	cacng2a;cacng3b;cacng5a;cacng7b;cacng8b;dnm1b;dnm3a
GO:0000027	ribosomal large subunit assembly	19	7	3.39	0.0394	mrto4;nop53;ppan;rpl12;rpl23a;rpl5a;rpl5b
GO:0051932	synaptic transmission, GABAergic	10	6	1.79	0.00092	gabra1;gabra3;gabra5;gabra6b;gabrg2;plcl2
GO:0060012	synaptic transmission, glycinergic	9	6	1.61	0.00165	glra1;glra2;glra3;glra4b;glrba;glrbb
GO:0051597	response to methylmercury	10	6	1.79	0.0035	baxa;gadd45ba;jun;mt2;sod1;sod2
GO:0030032	lamellipodium assembly	16	6	2.86	0.0112	ablim1b;ablim3;dmtn;robo4;ssh1b;ssh2b
GO:0061386	closure of optic fissure	13	6	2.32	0.0176	sfrp1a;sfrp5;sox11a;sox11b;sox4a;sox4b
GO:0008345	larval locomotory behavior	13	6	2.32	0.0176	psmd3;ryr1b;scn8aa;slc1a2b;sncb;sncgb

GO:0036376	sodium ion export across plasma membrane	13	6	2.32	0.0176	atp1a1b;atp1a3a;atp1b1b;atp1b2a;atp1b3a;atp1b3b
GO:0030007	cellular potassium ion homeostasis	13	6	2.32	0.0176	atp1a1b;atp1a3a;atp1b1b;atp1b2a;atp1b3a;atp1b3b
GO:0098970	postsynaptic neurotransmitter receptor diffusion trapping	13	6	2.32	0.0176	cacng2a;cacng3b;cacng5a;cacng7b;cacng8b;gphnb
GO:0043280	positive regulation of cysteine-type endopeptidase activity involved in apoptotic process	24	6	4.29	0.02063	baxa;baxb;bbc3;esco2;hdr;hip1
GO:0006883	cellular sodium ion homeostasis	14	6	2.5	0.02623	atp1a1b;atp1a3a;atp1b1b;atp1b2a;atp1b3a;atp1b3b
GO:0099054	presynapse assembly	9	6	1.61	0.03181	bdnf;mecp2;nlgn1;nlgn2a;nlgn2b;nlgn3a
GO:0007196	adenylate cyclase-inhibiting G-protein coupled glutamate receptor signaling pathway	5	5	0.89	0.00018	grm4;grm6a;grm6b;grm8a;grm8b
GO:0045956	positive regulation of calcium ion-dependent exocytosis	7	5	1.25	0.00276	cacna1g;cacna1ha;cacna1hb;cacna1i;scamp5a
GO:0098700	neurotransmitter loading into synaptic vesicle	7	5	1.25	0.00435	slc17a6b;slc17a7a;slc17a7b;slc18a2;slc32a1
GO:0042984	regulation of amyloid precursor protein biosynthetic process	7	5	1.25	0.00568	itm2ca;itm2cb;necab1;necab2;necab3
GO:0002138	retinoic acid biosynthetic process	8	5	1.43	0.00568	apc;hmx4;rbp1;rdh10a;rdh10b
GO:0000160	phosphorelay signal transduction system	8	5	1.43	0.00627	kcnh1a;kcnh3;kcnh4b;kcnh5a;kcnh5b
GO:0031023	microtubule organizing center organization	48	5	8.57	0.03199	calm1b;calm3a;cetn2;clasp2;pafah1b1a
GO:0030878	thyroid gland development	11	5	1.96	0.03228	bcl2l1;fgf8a;thraa;thrab;thrb
GO:0034625	fatty acid elongation, monounsaturated fatty acid	12	5	2.14	0.04738	elovl1a;elovl1b;elovl4b;elovl6;elovl8a
GO:0034626	fatty acid elongation, polyunsaturated fatty acid	12	5	2.14	0.04738	elovl1a;elovl1b;elovl4b;elovl6;elovl8a
GO:0046855	inositol phosphate dephosphorylation	12	5	2.14	0.04738	impa1;impa2;inpp5b;inpp5jb;ptenb
GO:0019367	fatty acid elongation, saturated fatty acid	12	5	2.14	0.04738	elovl1a;elovl1b;elovl4b;elovl6;elovl8a
GO:0098943	neurotransmitter receptor transport, postsynaptic endosome to lysosome	12	5	2.14	0.04738	cacng2a;cacng3b;cacng5a;cacng7b;cacng8b
GO:0010807	regulation of synaptic vesicle priming	4	4	0.71	0.00101	napaa;napab;napba;napbb
GO:0035494	SNARE complex disassembly	4	4	0.71	0.00101	napaa;napab;napba;napbb
GO:0045162	clustering of voltage-gated sodium channels	6	4	1.07	0.0112	gldn;ndrg4;nsfa;snap25b
GO:0021634	optic nerve formation	6	4	1.07	0.0112	klf6a;klf7b;smarca4a;tuba1a
GO:0006177	GMP biosynthetic process	8	4	1.43	0.0197	gmps;hprt1l;impdh1b;impdh2
GO:0007158	neuron cell-cell adhesion	7	4	1.25	0.02245	nlgn1;nlgn2a;nlgn2b;nlgn3a
GO:0097104	postsynaptic membrane assembly	7	4	1.25	0.02245	nlgn1;nlgn2a;nlgn2b;nlgn3a
GO:0097105	presynaptic membrane assembly	7	4	1.25	0.02245	nlgn1;nlgn2a;nlgn2b;nlgn3a
GO:0021772	olfactory bulb development	7	4	1.25	0.02245	anos1a;enpp1;msi2b;ptprsa
GO:0051965	positive regulation of synapse assembly	7	4	1.25	0.02245	clstn1;clstn2;clstn3;lrrc4bb
GO:0046548	retinal rod cell development	7	4	1.25	0.02245	rpgrip1;sox11a;sox11b;tbx2b
GO:0003208	cardiac ventricle morphogenesis	9	4	1.61	0.03185	fgf8a;fhl1a;ilk;nrg1
GO:0007026	negative regulation of microtubule depolymerization	8	4	1.43	0.03864	apc;apc2;CABZ01118678.1;camsap3
GO:0060999	positive regulation of dendritic spine development	8	4	1.43	0.03864	llph;shank1;shank2;si:ch1073-450f2.1
GO:0043652	engulfment of apoptotic cell	8	4	1.43	0.03864	havcr1;rac3a;rac3b;rhobtb1
GO:0006855	drug transmembrane transport	8	4	1.43	0.03864	abcg2c;abcg2d;slc18a2;slc18a3a
GO:0051591	response to cAMP	9	4	1.61	0.04273	enpp1;jun;junba;pebp1
GO:0070884	regulation of calcineurin-NFAT signaling cascade	3	3	0.54	0.00569	rcan1a;rcan2;rcan3
GO:0060509	Type I pneumocyte differentiation	3	3	0.54	0.00569	thraa;thrab;thrb
GO:1901842	negative regulation of high voltage-gated calcium channel activity	3	3	0.54	0.00569	gem;rem1;rrad
GO:0010996	response to auditory stimulus	4	3	0.71	0.0197	kcnma1a;otofa;wrb
GO:0036368	cone photoresponse recovery	4	3	0.71	0.0197	eml1;rcvrn3;rcvrna
GO:0035385	Roundabout signaling pathway	4	3	0.71	0.0197	robo2;robo3;zgc:77784
GO:0016199	axon midline choice point recognition	4	3	0.71	0.0197	adcy1b;robo2;robo3

GO:0000447	endonucleolytic cleavage in ITS1 to separate SSU-rRNA from 5.8S rRNA and LSU-rRNA from tricistronic rRNA transcript (SSU-rRNA, 5.8S rRNA, LSU-rRNA)	4	3	0.71	0.0197	abt1;rps21;rpsa
GO:0019388	galactose catabolic process	6	3	1.07	0.03186	galm;pgm1;pgm5
GO:0071376	cellular response to corticotropin-releasing hormone stimulus	5	3	0.89	0.04274	crhr1;nr4a1;nr4a3
GO:0042264	peptidyl-aspartic acid hydroxylation	5	3	0.89	0.04274	asph;asphd1;asphd2
GO:0010998	regulation of translational initiation by eIF2 alpha phosphorylation	5	3	0.89	0.04274	eif2ak1;eif2ak2;pkz
GO:2000463	positive regulation of excitatory postsynaptic potential	5	3	0.89	0.04274	shank1;shank2;si:ch1073-450f2.1
GO:0097107	postsynaptic density assembly	5	3	0.89	0.04274	shank1;shank2;si:ch1073-450f2.1
GO:0097264	self proteolysis	5	3	0.89	0.04274	TENM2;tenm3;tenm4
GO:1904825	protein localization to microtubule plus-end	5	3	0.89	0.04274	mapre1b;mapre2;mapre3b
GO:0045634	regulation of melanocyte differentiation	2	2	0.36	0.03187	ctbp2a;hipk2
GO:0051012	microtubule sliding	2	2	0.36	0.03187	pafah1b1a;pafah1b1b
GO:0045905	positive regulation of translational termination	2	2	0.36	0.03187	eif5a;eif5a2
GO:0048025	negative regulation of mRNA splicing, via spliceosome	2	2	0.36	0.03187	rbmx;ybx1
GO:0038026	reelin-mediated signaling pathway	2	2	0.36	0.03187	dab2ipa;dab2ipb
GO:1904059	regulation of locomotor rhythm	2	2	0.36	0.03187	nr1d1;scn1lab
GO:0044210	'de novo' CTP biosynthetic process	2	2	0.36	0.03187	ctps1a;ctps1b
GO:0036071	N-glycan fucosylation	2	2	0.36	0.03187	fut8a;fut8b
GO:0002154	thyroid hormone mediated signaling pathway	2	2	0.36	0.03187	thraa;thrb
GO:0035024	negative regulation of Rho protein signal transduction	2	2	0.36	0.03187	akap12b;kctd13
GO:0030948	negative regulation of vascular endothelial growth factor receptor signaling pathway	2	2	0.36	0.03187	dab2ipa;dab2ipb
GO:0008295	spermidine biosynthetic process	2	2	0.36	0.03187	amd1;srm
GO:0001778	plasma membrane repair	2	2	0.36	0.03187	anxa6;dysf
GO:0006452	translational frameshifting	2	2	0.36	0.03187	eif5a;eif5a2
GO:0033578	protein glycosylation in Golgi	2	2	0.36	0.03187	fut8a;fut8b
GO:0021766	hippocampus development	2	2	0.36	0.03187	enpp1;pebp1
GO:0048240	sperm capacitation	2	2	0.36	0.03187	abhd2a;pebp1
GO:0009098	leucine biosynthetic process	2	2	0.36	0.03187	bcat1;bcat2
GO:0009099	valine biosynthetic process	2	2	0.36	0.03187	bcat1;bcat2
GO:0051969	regulation of transmission of nerve impulse	2	2	0.36	0.03187	kcnab1a;kcnab2b
GO:0009449	gamma-aminobutyric acid biosynthetic process	2	2	0.36	0.03187	gad1b;gad2
GO:0006696	ergosterol biosynthetic process	2	2	0.36	0.03187	acat2;fdft1
GO:0015820	leucine transport	2	2	0.36	0.03187	slc6a15;slc6a17
GO:0000461	endonucleolytic cleavage to generate mature 3'-end of SSU-rRNA from (SSU-rRNA, 5.8S rRNA, LSU-rRNA)	2	2	0.36	0.03187	rps21;rpsa
GO:0035553	oxidative single-stranded RNA demethylation	2	2	0.36	0.03187	alkbh5;fto
GO:0060290	transdifferentiation	2	2	0.36	0.03187	insm1a;insm1b

Table S10. RNA sample quality control.

Sample Name	Nanodrop					Bioanalyzer						Qubit	
	260/280	260/230	Nanodrop Conc (ng/ul)	Dilution factor	Nano final (ng/ul)	RIN	From (bp)	To (bp)	Total average size	size distribution in CV (%)	Bionalyzer Conc. (ng/ul)	Qubit ng/ul	Total Qubit ug
0RNA_1	2.18	0.42	2.6	20	52	8.7	179	1328	382	35.6	14.952	52.2	1.044
0RNA_2	2.39	1.15	2.5	20	50	8.6	156	1351	372	33.7	38.755	61	1.22
0RNA_3	1.65	0.8	2.8	20	56	8.6	149	1358	360	29.5	23.6	53.2	1.064
2RNA_1	1.65	1.68	4	20	80	8.6	182	1345	373	33.6	56.448	96.6	1.932
2RNA_2	1.72	0.49	6.5	10	65	7.8	171	1354	388	37.9	18.571	48.6	0.972
2RNA_3	2.11	2.01	1.96	20	39.2	8.6	174	1328	363	30.9	62.109	49.6	0.992
4RNA_1	1.76	0.35	3.8	20	76	8.2	171	1321	380	38.5	13.475	79	1.58
4RNA_2	2.1	2.11	2.12	20	42.4	8.5	170	1353	385	38.9	82.079	60.2	1.204
4RNA_3	1.88	1.03	3.33	20	66.6	7.7	180	1323	410	40.3	41.509	45.6	0.912
7RNA_1	1.93	0.38	48.9	20	48.9	7.8	141	1331	404	42.2	16.036	62.2	1.244
7RNA_2	1.9	0.73	58.3	20	58.3	8.3	183	1366	413	40.5	29.565	57.8	1.156
7RNA_3	1.99	0.36	57.3	20	57.3	8.6	183	1341	398	39.2	14.112	53.2	1.064
12RNA_1	1.77	0.15	4.9	10	49	8.6	186	1340	382	37	5.55	45	0.99
12RNA_2	1.91	0.68	49.9	20	49.9	8	173	1337	387	38	25.84	51.4	1.028
12RNA_3	2.18	1.7	5.72	10	57.2	8.7	187	1335	400	37.6	63.92	67.2	1.344

Table S11. ATAC-seq library sample quality control.

Sample Name	Nanodrop			Bioanalyzer				Qubit	
	260/280	260/230	Nanodrop Conc (ng/ul)	Bio Avg fragment	Molarity (nmol/L)	Average size	Bionalyzer Conc. (ng/ul)	Qubit ng/ul	Total Qubit ug
0dpiATAC_1	2.05	1.86	30.3	384	48.4	338	4.327	16.8	252
0dpiATAC_2	2.08	0.8	23.7	503	20.2	536	6.111	10.5	157.5
0dpiATAC_3	1.89	1.75	61.2	396	49.4	301	32.282	27	405
2dpiATAC_1	1.86	1.45	75.6	359	69.5	351	5.156	25.4	381
2dpiATAC_2	1.93	1.38	45.7	513	10.2	587	3.37	10.4	156
2dpiATAC_3	2.11	2.01	39.2	352	56.4	339	9.158	18	270
4dpiATAC_1	1.81	0.61	50.71	403	18.6	361	7.845	11.3	169.5
4dpiATAC_2	2.12	0.56	48.12	397	27.3	363	61.829	6.62	99.3
4dpiATAC_3	1.86	1.74	37.9	365	53.7	286	5.698	23.2	348
7dpiATAC_2	1.9	0.73	58.3	492	17.3	573	6.33	11.3	169.5
7dpiATAC_3	1.91	2.06	39.6	367	48.1	307	4.975	16.9	253.5
12dpiATAC_1	2.02	1.82	21.5	467	33.7	485	30.625	17.2	258
12dpiATAC_2	1.92	0.7	56.44	363	63.9	350	6.925	28	420
12dpiATAC_3	2.12	0.66	56.5	364	85.4	326	6.165	26	390

Table S12. PCR primers for ATAC-seq libraries based on Nextera indices .

Sample Name	Primer Sequences	Index
0dpiATAC_1	**CAAGCAGAAGACGGCATACGAGATTCGCCTTAGTCTCGTGGGCTCGGAGATGT**	Ad2.1_TAAGGCGA
0dpiATAC_2	**CAAGCAGAAGACGGCATACGAGATTTCTGCCTGTCTCGTGGGCTCGGAGATGT**	Ad2.3_AGGCAGAA
0dpiATAC_3	**CAAGCAGAAGACGGCATACGAGATGTAGAGAGGTCTCGTGGGCTCGGAGATGT**	Ad2.7_CTCTCTAC
2dpiATAC_1	**CAAGCAGAAGACGGCATACGAGATCAGCCTCGGTCTCGTGGGCTCGGAGATGT**	Ad2.10_CGAGGCTG
2dpiATAC_2	**CAAGCAGAAGACGGCATACGAGATTGCCTCTTGTCTCGTGGGCTCGGAGATGT**	Ad2.11_AAGAGGCA
2dpiATAC_3	**CAAGCAGAAGACGGCATACGAGATTCCTCTACGTCTCGTGGGCTCGGAGATGT**	Ad2.12_GTAGAGGA
4dpiATAC_1	**CAAGCAGAAGACGGCATACGAGATGCTCAGGAGTCTCGTGGGCTCGGAGATGT**	Ad2.4_TCCTGAGC
4dpiATAC_2	**CAAGCAGAAGACGGCATACGAGATAGGAGTCCGTCTCGTGGGCTCGGAGATGT**	Ad2.5_GGACTCCT
4dpiATAC_3	**CAAGCAGAAGACGGCATACGAGATCATGCCTAGTCTCGTGGGCTCGGAGATGT**	Ad2.6_TAGGCATG
7dpiATAC_2	**CAAGCAGAAGACGGCATACGAGATCCTCTCTGGTCTCGTGGGCTCGGAGATGT**	Ad2.8_CAGAGAGG
7dpiATAC_3	**CAAGCAGAAGACGGCATACGAGATAGCGTAGCGTCTCGTGGGCTCGGAGATGT**	Ad2.9_GCTACGCT
12dpiATAC_1	**CAAGCAGAAGACGGCATACGAGATATCACGACGTCTCGTGGGCTCGGAGATGT**	Ad2.13_GTCGTGAT
12dpiATAC_2	**CAAGCAGAAGACGGCATACGAGATACAGTGGTGTCTCGTGGGCTCGGAGATGT**	Ad2.14_ACCACTGT
12dpiATAC_3	**CAAGCAGAAGACGGCATACGAGATCAGATCCAGTCTCGTGGGCTCGGAGATGT**	Ad2.15_TGGATCTG
